# Evolutionary and structural analysis of SARS-CoV-2 specific evasion of host immunity

**DOI:** 10.1038/s41435-020-00120-6

**Published:** 2020-12-03

**Authors:** Irfan Hussain, Nashaiman Pervaiz, Abbas Khan, Shoaib Saleem, Huma Shireen, Dong-Qing Wei, Viviane Labrie, Yiming Bao, Amir Ali Abbasi

**Affiliations:** 1 National Center for Bioinformatics, Program of Comparative and Evolutionary Genomics, Faculty of Biological Sciences, Quaid-i-Azam University, Islamabad, 45320 Pakistan; 2 State Key Lab of Microbial Metabolism, Department of Bioinformatics and Biological Statistics, School of Life Sciences and Biotechnology, Shanghai Jiao Tong University, 200240 Shanghai, PR China; 3 State Key Laboratory of Microbial Metabolism, Shanghai-Islamabad-Belgrade Joint Innovation Center on Antibacterial Resistances, Joint Laboratory of International Cooperation in Metabolic and Developmental Sciences, Ministry of Education and School of Life Sciences and Biotechnology, Shanghai Jiao Tong University, 200030 Shanghai, PR China; 4 Peng Cheng Laboratory, Vanke Cloud City, Phase I Building 8, Xili Street, Nashan District, Guangdong, 518055 Shenzhen PR China; 5 Center for Neurodegenerative Science, Van Andel Research Institute, Grand Rapids, MI 49503 USA; 6 Division of Psychiatry and Behavioral Medicine, College of Human Medicine, Michigan State University, Grand Rapids, MI 49503 USA; 7 National Genomics Data Center & CAS Key Laboratory of Genome Sciences and Information, Beijing Institute of Genomics, Chinese Academy of Sciences, China National Center for Bioinformation, 100101 Beijing, PR China; 8 University of Chinese Academy of Sciences, 100101 Beijing, PR China

**Keywords:** Immune evasion, Viral infection

## Abstract

The outbreak of coronavirus disease 2019 (COVID-19) caused by severe acute respiratory syndrome coronavirus 2 (SARS-CoV-2) is spreading fast worldwide. There is a pressing need to understand how the virus counteracts host innate immune responses. Deleterious clinical manifestations of coronaviruses have been associated with virus-induced direct dysregulation of innate immune responses occurring via viral macrodomains located within nonstructural protein-3 (Nsp3). However, no substantial information is available concerning the relationship of macrodomains to the unusually high pathogenicity of SARS-CoV-2. Here, we show that structural evolution of macrodomains may impart a critical role to the unique pathogenicity of SARS-CoV-2. Using sequence, structural, and phylogenetic analysis, we identify a specific set of historical substitutions that recapitulate the evolution of the macrodomains that counteract host immune response. These evolutionary substitutions may alter and reposition the secondary structural elements to create new intra-protein contacts and, thereby, may enhance the ability of SARS-CoV-2 to inhibit host immunity. Further, we find that the unusual virulence of this virus is potentially the consequence of Darwinian selection‐driven epistasis in protein evolution. Our findings warrant further characterization of macrodomain-specific evolutionary substitutions in in vitro and in vivo models to determine their inhibitory effects on the host immune system.

## Introduction

Since the first reports of patients with atypical pneumonia or coronavirus disease 2019 (COVID-19) in Wuhan, China in late December 2019, the outbreak has now become a pandemic with global socioeconomic impact. Presently, there is no vaccine or specific antiviral treatment for COVID-19. On 7 January 2020, through genome sequencing technology, a novel coronavirus (nCoV) was identified as the causative pathogen, named as 2019-nCoV (also referred as SARS-CoV-2) [1].

CoVs are commonly associated with respiratory and gastrointestinal tract infections and constitute a phylogenetically diverse viral group, comprising of four genera: *alphacoronavirus* (α-CoV), *betacoronavirus* (β-CoV), *gammacoronavirus* (γ-CoV), and *deltacoronavirus* (δ-CoVs) [2]. They are complex pathogens that are known to infect multiple host species, including humans [3, 4]. Before the emergence of COVID-19, six CoVs were known to infect humans. For instance, HCoV‐NL63 (β-CoV), HCoV‐229E (β-CoV), HCoV‐OC43 (α-CoV), and HKU1 (α-CoV) can cause mild upper respiratory infections, whereas SARS‐CoV (β-CoV) and MERS‐CoV (β-CoV) can infect the lower respiratory tract of humans and cause severe respiratory syndrome [5]. Severe acute respiratory syndrome coronavirus 2 (SARS-CoV-2) belonging to the group of β-CoVs, is the seventh CoV to infect humans and the third β-CoV to infect the lower respiratory tract. The mortality rate of SARS (9.6%) and MERS (34%) is reportedly higher than that of COVID-19 (3–6%) [6]. However, the spread of SARS-CoV-2 infection is remarkably wide and rapid [7].

Comparative genomics has revealed that SARS-CoV-2 possesses a genome architecture typical of CoVs, comprising of a ~29.8 kilobase (kb) single‐stranded positive‐sense RNA (+ssRNA) that contains 14 ORFs encoding for 27 proteins. The 5′-terminus of the genome encodes for two long polyproteins, pp1ab (7096 amino acids) and pp1a (4405 amino acids) [1]. These polyproteins are processed by virally encoded proteases to produce 10 nonstructural proteins (nsp1–nsp10), and, in addition, pp1ab uniquely produces nsp13 to nsp16 and pp1a uniquely produces nsp11. The 3′-terminus of the SARS-CoV-2 genome encodes for four main structural proteins: spike (S), membrane (M), envelope (E), nucleocapsid (N), and eight accessory proteins [8].

The availability of SARS-CoV-2 genome sequence data has initiated efforts to design diagnostic tests and potent therapies [9]. Furthermore, there is an urgent need to explore the evolutionary origin and phylogenetics of SARS-CoV-2 with the potential implication that it will further our understanding of disease pathogenesis and spread [1, 10, 11]. Traditionally, inference of evolutionary relationships among CoVs has relied heavily on comparisons of whole genome sequence data or of their critical structural genes, such as the *S* gene, which encodes the spike protein [1]. Nucleotide datasets comprised of multiple distinct genes exhibiting heterogeneity in their mode and rate of sequence evolution can adversely affect phylogenetic reconstruction [12]. Evading this problem is particularly challenging in CoVs. Comparative analysis has revealed a heterogeneous rate of evolution of portions of the genome of CoVs, with ~60% shared identity within nsp coding regions and only ~40% shared identity in the remaining one-third of the genome coding for structural proteins. This heterogeneity in substitution rates in coding regions of CoVs can potentially complicate attempts to reconstruct the evolutionary history of CoVs [13]. Specifically, the rapid rate of evolution of structural genes (such as the *S* gene) may cause a gene-based analysis to blur the history of the taxa [14]. In contrast, pp1ab is a large, slow-evolving domain in SARS-COV-2 capable of circumventing the potential pitfalls of using genomic-based approaches to reconstruct the history of SARS-CoV-2 [5]. Here, based on completely sequenced genomes of SARS-CoV-2, covering at least 39 distinct global territories, we conducted an in-depth comparative analysis of the 7096-aa replicase polyprotein pp1ab, comparing it to the corresponding homologous polyproteins of 83 related CoVs [15]. To best of our knowledge, this is the first attempt to use the full-length pp1ab polyprotein to predict SARS-CoV-2 relatedness to other members of the *Coronavirinae* subfamily. We next focused on macrodomains encoded within Nsp3 of pp1ab to investigate their structural evolution in light of previous associations between macrodomains and virulence, and the potential of these associations in designing a novel therapeutic strategy for the treatment of SARS-CoV-2 -induced severe infections.

## Materials and methods

### Sequence collection

The amino acid and coding sequences of ORF1ab gene from four genera of the subfamily Coronavirinae; Alphacoronavirus, Betacoronavirus, Gammacoronavirus, and Deltacoronavirus were retrieved from GenBank (http://www.ncbi.nlm.nih.gov) [16], 2019 Novel Coronavirus Resource of CNCB/NGDC [15, 17] and the GISAID databank (https://www.gisaid.org/) [18]. In total 247 sequences from four genera of subfamily Coronavirinae were used in this study (Supplementary Tables S1, S2; Supplementary Alignment File).

### Phylogenetic and sequence analysis

The sequence alignment of 121 Coronavirinae polyprotein pp1ab sequences was performed using CLUSTALW (default parameters) [19]. The phylogenetic tree of the subfamily Coronavirinae, including SARS-CoV-2 was reconstructed in MEGA 5.05 by the maximum likelihood (ML) method with the Whelan and Goldman (WAG) amino-acid substitution model [20, 21] The phylogenetic tree with the upmost log likelihood scores was selected. A neighbor-joining (NJ) tree was also reconstructed using uncorrected proportion (p) distance and the Jones, Taylor, and Thornton method (as amino-acid substitution model) to calculate evolutionary distances between coronaviruses [22, 23]. Topological reliability of the NJ and ML tree was tested by bootstrap analysis (1000 pseudoreplicates) [23].

All proteins encoded by the ORF1ab gene were aligned for SARS-CoV-2 (YP_009725299.1), bat-RaTG13 (QHR63299.1), bat-ZC45 (AVP78030.1), bat-ZXC21 (AVP78041.1), and SARS-CoVBJ01 (AAP30028.1) using MAFFT and Clustal Omega [24, 25]. Amino acids substitutions unique to SARS-CoV-2 were identified by manual inspection of the alignments (Supplementary Table S3). A sequence similarity plot was produced (sliding window with 5 amino acid-step) using the Plotcon software available in the EMBOSS software suite [26].

Thermodynamic state function, the ∆∆G (Gibbs free energy) of a substitution from ancestral protein to its altered version was predicted by employing the conformationally constrained environment-specific substitution tables [27]. The putative physicochemical impact of each substitution on protein structure and function was estimated using the BLOSUM-62 substitution matrix [28].

### Ancestral sequence reconstruction

The ML method implemented in MEGA was used to reconstruct ancestral sequences of SARS-CoV-2 and bat-RaTG13/ZC45/ZXC21 based on amino-acid substitutions identified in the WAG model [20, 21, 29]. Separately, we also inferred the ancestral sequence using the PRANK program that accepted insertions and deletions as distinct evolutionary events [30]. The consensus of PRANK and MEGA ancestral sequence of SARS-CoV-2 and bat-RaTG13/ZC45/ZXC was used in this current study. Sequence alignments for ancestral reconstruction were performed using MUSCLE and MAFFT (default parameters) [24, 31]

### Structural analysis

After the divergence from Bat-CoV-RaTG13, the SARS-CoV-2 nonstructural protein-3 (Nsp3) accumulated a greater number of substitutions compared to any other nonstructural protein encoded by the SARS-CoV-2 ORF1ab gene. To determine the functional effects of these Nsp3 substitutions in SARS-CoV-2, we performed a 3D structural analysis that examined all relevant protein structures using a homology modeling approach in Modeler [32, 33]. For the structural analysis, we based template selection on high sequence homology and amino-acid length/coverage and obtained the following templates from RCSB databank [34]: 2WCT, 2JZF, 2KQV, 2ACF, 6MEA, and 5DUS. Protein structures were predicted using the Discrete Optimized Protein Energy score, followed by implementation of the energy minimization protocol in PyMOL to maximally improve the quality of the modeled structures [35]. The quality of the predicted protein structures were further validated by Rampage Ramachandran plot analysis [36]. Superimposition of the modeled protein structures was performed with PyMOL, and root mean square deviation values were assessed [35, 37]. Furthermore, sequence-based secondary structure elements were determined using the PSIPRED server [38].

For comparative binding analysis of Mac-1 to ADPr, crystallographic structures of MERS-CoV (5DUS), SARS-CoV (2FAV), and SAR-CoV-2 (6W02) Mac-1 (within Nsp3) were obtained from the RCSB databank [34]. AutoDock [39] was used to perform an induce-fit docking (IFD) protocol with 30 conformers, while keeping the rest of the parameters default. IFD modeling offers mutual conformational adaptations of a protein receptor to a ligand, which enables for better accuracy than docking to a rigid target [40]. The best docking complexes were selected based on docking score. ADPr (ADP-ribose) and Mac-1 superimposition between CoVs were visualized in PyMOL. Interactions of the key residues of Mac-1 with ADPr protein was obtained and visualized in PyMOL. To confirm the differences in binding affinities of Mac-1 (for ADPr) of MERS-CoV, SARS-CoV, and SAR-CoV-2, DoGsitescorer (https://proteins.plus/) was used to calculate volume, surface area and druggability scores of binding cavities [41].

## Results and discussion

**Fig. 1:**
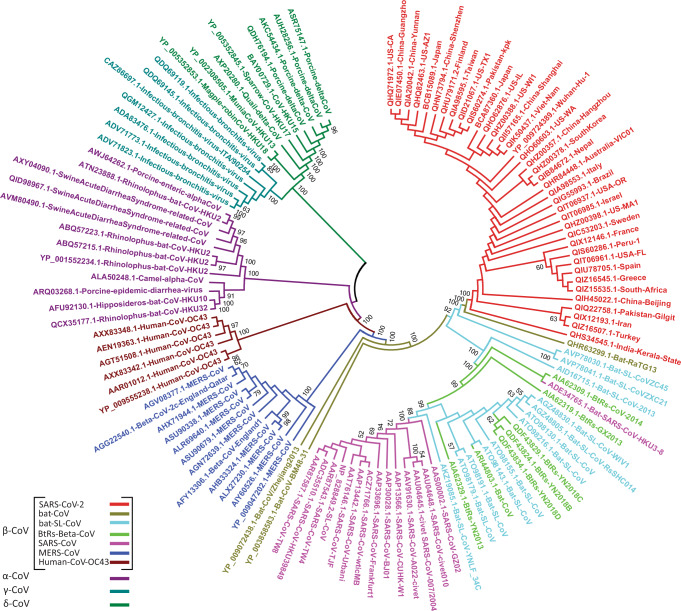
Phylogenetic characterization of SARS-CoV-2 based on the 7096-aa polyprotein pp1ab. Phylogenetic tree demonstrating the relationship of SARS-CoV-2 to other CoVs. Phylogenetic analysis involved 121 pp1ab sequences from the subfamily *Coronavirinae*, including representatives of four genera; α-CoV, β-CoV, γ-CoV, and δ-CoV. The color codes distinguish between various groups/types of coronaviruses. The phylogenetic tree was reconstructed using the maximum likelihood method with the WAG substitution model. Bootstrap values ≥50% are shown along the branches. Scaled phylogram of this tree with branch lengths reflecting the amount of genetic change is provided in Supplementary Fig. S1.

ML and NJ trees exhibited similar topologies, where the cluster of δ-CoVs and γ-CoVs was the first to diverge, followed by α-CoVs, β-OC43-CoVs, β-MERS-CoVs, and β-SARS-CoVs, respectively (Fig. 1; Supplementary Figs. S1, S2). Tree topology confirms the direct grouping of SARS-CoV-2 with batCoV-RaTG13, which diverged from a cluster of batSLCoVZC45 and batSLCoVZXC21.This pattern places the SARS-CoV-2 and the three batSL-CoVs in a distinct phylogenetic group compare to SARS-CoVs and other SARS-like coronaviruses (Fig. 1).

**Fig. 2:**
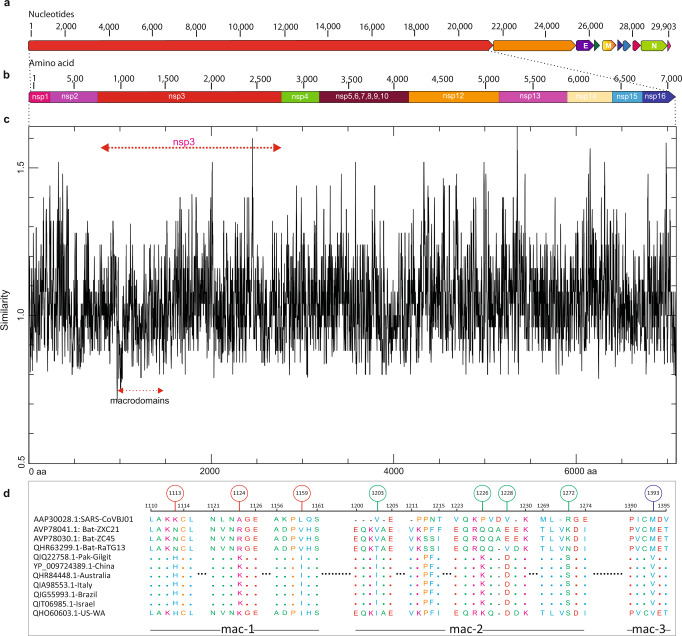
Identification of sequence divergence at the polyprotein pp1ab locus in SARS-CoV-2. **a** Schematic of SARS-CoV-2 genome. Genomic organization of SARS-CoV-2 with numbering above the block referring to nucleotide positions. Structural proteins, including spike (S), envelope (E), membrane (M), and nucleocapsid (N) proteins, as well as nonstructural proteins (nsps) translated from ORF1ab and accessory proteins are indicated. **b** Schematic of 7096-aa replicase polyprotein pp1ab with its 15 sub-proteins (nsp1–nsp10 and nsp12–nsp16). **c** Comparative sequence analysis of the pp1ab domain for completely sequenced genomes of SARS-CoV-2 from at least 39 distinct global territories. SARS-CoV-2 sequences were compared to the corresponding homologous sequence from batSL-CoVs and SARS-CoVBJ01. Macrodomains within Nsp3 are demarcated by red arrow. *Y* axis depicts similarity scores between CoVs and the *X* axis refers to the relative residue position. Lower scores signify low sequence conservation, with trough corresponding to the least conserved regions. **d** Macrodomains sequences encoding Mac-1, Mac-2, and Mac-3 proteins exhibiting exceptional divergence in SARS-CoV-2 relative to bat-RaTG13/ZC45/ZXC21 and SARS-CoVBJ01. Green, red, and blue circles, respectively, differentiate between Mac-1, Mac-2, and Mac-2 specific substitutions.

Phylogenetic separation of SARS-CoV-2 from SARS-CoVs suggests that after their origin from the Hp-βCoV/BM48-31-like common ancestor, these two distinct lineages of CoVs were subjected to different genetic selection pressures. This may have led to notable differences in their infectivity, transmissibility, pathogenesis, and host tropism. This speculation prompted us to elucidate the putative functional uniqueness of SARS-CoV-2 using a pp1ab-based analysis. In SARS-CoV-2 genomes sampled from multiple, distinct geographic locations, we searched for amino-acid substitutions in the 7096-aa long pp1ab sequence by comparison with the closely related batSL-CoVs (RaTG13/ZC45/ZXC21) and the distantly related representatives of the β-CoV lineage (Fig. 2a–c; Supplementary Tables S1, S2). Inspection of comparative data revealed a total of 90 replacements/insertions/deletions in pp1ab of SARS-CoV-2 (Supplementary Fig. 3; Supplementary Table S3). Among these, 53 amino-acid differences were found to be fixed in pp1ab of SARS-CoV-2, as compared with batSL-CoVs (Supplementary Table S3). The Nsp3 within pp1ab appeared to be crucial to the evolutionary diversification of SARS-CoV-2, harboring 31 of the 53 fixed substitutions (Supplementary Table S3). Fixation of 31 amino-acid replacements within Nsp3 of SARS-CoV-2 was further validated through analysis of representative sequence data from seven distinct clades of SARS-CoV-2 (G, GH, GR, L, O, S, and V) reported in GISAID (https://www.gisaid.org) [18] (Supplementary Table S4; Supplementary Alignment File). Nsp3 is the largest multi-domain protein produced by CoVs, playing many important roles in the viral life cycle. In particular, macrodomains encoded within Nsp3 of CoVs have been demonstrated as critical in counteracting the host innate immune response [42].

**Fig. 3:**
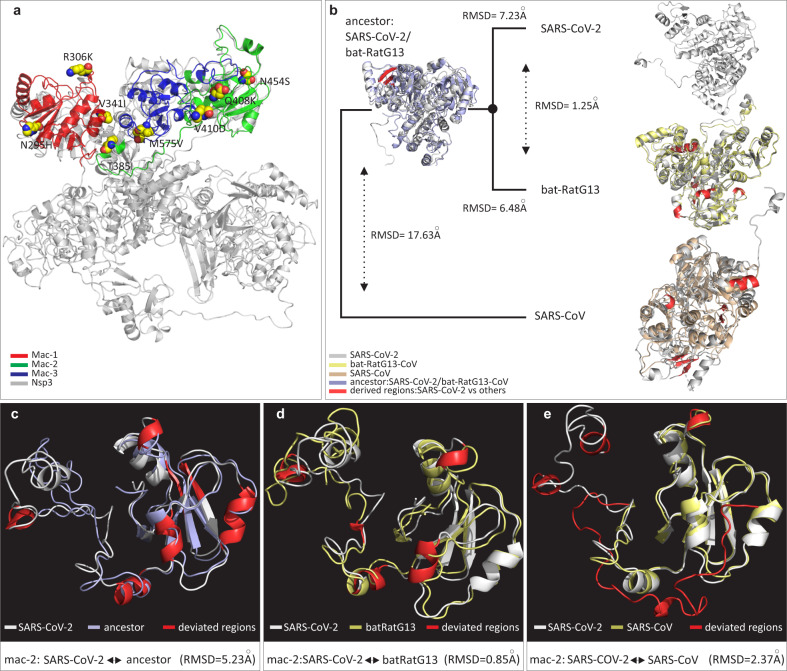
Protein structural analysis of macrodomains examining the effects of specific fixed substitutions within Nsp3 of SARS-CoV-2. **a** Protein structure of SARS-CoV-2 Nsp3 depicting types and locations of fixed amino-acid substitutions within distinct macrodomains. The fixed amino-acid replacements are shown as spheres and labeled with the amino-acid position in Nsp3 protein. **b** Comparison of the three-dimensional (3D) conformations of the macrodomains (Mac-1, Mac-2, and Mac-3) within Nsp3. SARS-CoV-2 (YP_009725299.1) macrodomains were also compared to the corresponding homologous protein regions of bat-RatG13 (QHR63299.1), SARS-CoVBJ01 (AAP30028.1), and the predicted ancestral macrodomain of SARS-CoV-2 and bat-RaTG13/ZC45/ZXC21. Structural deviations in terms of backbone torsion angles (Φ°, Ψ°) are represented in red color and were examined by RMSD (root mean square deviation) values. **c–e** Close-up of 3D conformations of SARS-CoV-2 Mac-2 with corresponding homologous domains from predicted ancestor (aforementioned; left panel), bat-RatG13 (middle panel), and SARS-CoV (AAP30028.1; right panel). Comparisons of 3D conformations for Mac-1 is provided in Fig. 4 and sequence secondary structural level details for comparisons in **b**–**e** are given in Supplementary Tables S6, S7.

Given their roles in virulence and pathogenesis, macrodomains of SARS-CoV-2 were subjected to further scrutiny through sequence, structural, and evolutionary analysis. Macrodomains located in Nsp3 were found to be particularly enriched with fixed amino-acid replacements specific to SARS-CoV-2 (Fig. 2d). In total, eight substitutions were fixed in the macrodomains of SARS-CoV-2 (Supplementary Table S5). These eight substitutions, though divergent from corresponding homologous positions in closely related bats (RaTG13/ZC45/ZXC21), are otherwise exceptionally constrained; not a single amino-acid difference was noted among the 39 SARS-CoV-2 genomes at these sites (Supplementary Fig. S4). Conceivably, these substitutions are the consequence of accelerated rates of sequence evolution, which may have been driven by positive Darwinian selection after the divergence of SARS-CoV-2 and batSL-CoVs and prior to its first reported outbreak. Among the identified set of macrodomain-specific fixed substitutions, three were found to reside within macrodomain 1 (Mac-1), four in macrodomain 2 (Mac-2), whereas only a single amino-acid substitution was found to be fixed in macrodomain 3 (Mac-3) of SARS-CoV-2 (Fig. 3a).

Next, we further determined the impact of the fixed amino-acid substitutions identified within the macrodomains of Nsp3 of SARS-CoV-2 by predicting the thermodynamic state function. For this we determined the ∆∆G (Gibbs free energy) of a substitution in SARS-CoV-2 relative to the ancestral protein (∆∆G_ances→CoV2_) [27]. All of the eight fixed replacements appeared to have significant destabilizing effects on protein structure (∆∆G < 0.0) (Supplementary Table S5). Since mutations that modulate enzymatic functions or ligand binding are often destabilizing [43], the thermodynamic effects of the substitutions unique to SARS-CoV-2 macrodomains support their adaptive significance. In addition, examination of physicochemical property changes resulting from amino-acid substitutions in the SARS-CoV-2 macrodomains predict that, with respect to their effects on protein structure and function, all fixed replacements in macrodomains of SARS-CoV-2 are of radical type, implying their biological significance (Supplementary Table S5).

The globular Mac-1 of Nsp3 contains a conserved cleft that binds ADP-ribose (ADPr). Mac-1 was found to possess hydrolase activity that removes ADPr from target proteins, a biochemical feature of SARS-CoV-2 that is considered essential in counteracting the host antiviral response of ADP-ribosylation [42]. Reducing the capacity for Mac-1 to remove ADPr, especially in CoVs, results in an attenuation of virulence and a greater sensitivity to host innate immune responses [44–46]. Furthermore, the Macrodomain 2 and Macrodomain 3 (Mac-2 and 3) of SARS-CoVs are known to be indispensable for its replication or transcription, as these macrodomains bind nucleic acids, with a preference for purine-rich RNA sequences, such as G-rich stretches [47]. mRNAs for host antiviral responses and apoptotic signaling harbor long poly(G) stretches at their 3′ untranslated regions, and thus are prime targets for Mac-2 and 3 mediated disruption of host immunity [48, 49]. Furthermore, it has been demonstrated that Mac-2 and 3, together with the papain-like protease domain (PL2pro) of Nsp3, interact with RCHY1(E3 ubiquitin ligase) and intensify RCHY1-mediated ubiquitination, which consequently induces p53 degradation. Hence, human SARS-CoVs via their Mac-2 and 3 domains downregulate p53, a major determinant of antiviral innate immunity, thus leading to delayed activation of p53-targeted immunity genes [49]. Intriguingly, Mac-2 and 3 are specifically present in Nsp3 of SARS-CoVs and highly related viruses (batSL-CoVs) known to cause high levels of pathogenicity in humans, but are not present in CoVs that cause mild infections [42].

**Fig. 4:**
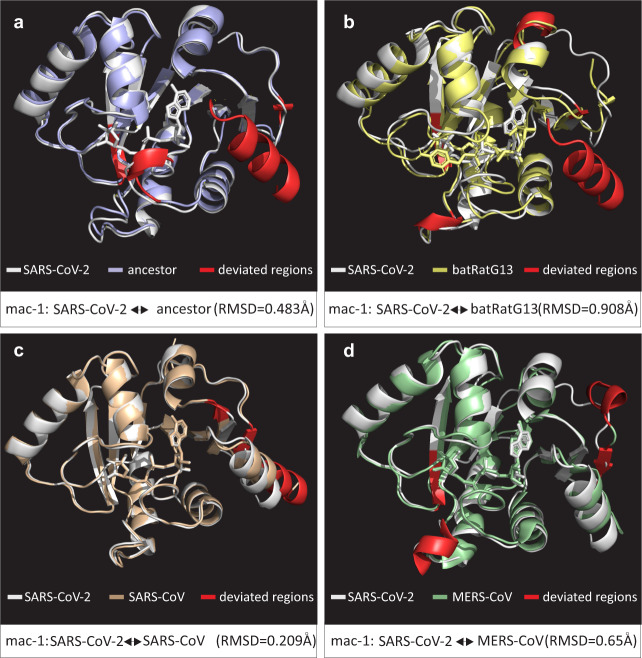
Comparisons of 3D conformations of SARS-CoV-2 macrodomain 1. Comparisons of 3D conformations of SARS-CoV-2 macrodomain 1 with corresponding homologous domains from (**a**) predicted ancestral macrodomain 1 (of SARS-CoV-2 and bat-RaTG13/ZC45/ZXC21), (**b**) bat-RatG13 (QHR63299.1), (**c**) SARS-CoV (PDB entry: 2FAV), and (**d**) MERS-CoV (PDB entry: 5HOL). Descriptions of color codes are given in each panel. Deviated residues in terms of backbone torsion angles (Φ°, Ψ°) are shown in red color. Structural deviations were examined by RMSD values. Note: Primary sequence and secondary structural level details for comparisons in **a**–**d** are given in Supplementary Tables S6, S7.

Herein, we sought to evaluate the functional significance of fixed amino-acid replacements specific to SARS-CoV-2 by modeling the macrodomains (Mac-1, 2, and 3) of SARS-CoV-2 (YP_009725299.1), bat-RaTG13 (QHR63299.1), and SARS-CoV-BJ01 (AAP30028.1). Furthermore, the ancestral macrodomain protein sequence of SARS-CoV-2 and bat-RaTG13/ZC45/ZXC21 was also predicted and modeled. The 3D superimposition of macrodomain structures revealed that during the course of evolution, SARS-CoV-2 macrodomains have had significant transitions in various secondary structural elements (SSEs) (Fig. 3b). A continuous transition from loops to core SSEs was observed (Supplementary Table S6). For instance, the macrodomains of SARS-CoV-2 contain about 62% of their residues in SSEs (Supplementary Table S6). In contrast, the macrodomains of SARS-CoV had about 41% of their total residues occurring in loops and the remaining 59% were in SSEs, such as α-helices and β-sheets (Supplementary Table S6). Furthermore, multiple substitutions scattered across the SARS-CoV-2 macrodomains were found to reposition specific protein regions within Mac-1 and Mac-2, in three-dimensional space (Figs. 3c–e, 4; Supplementary Table S7). In particular, there were drastic changes in the conformation of Mac-2 of SARS-CoV-2 (Fig. 3c–e). Structural evolution of Mac-2 involves considerable alterations in sequence, length, and conformation of core SSEs, implicating a functional relevance of these substitutions (Supplementary Table S7).

**Fig. 5:**
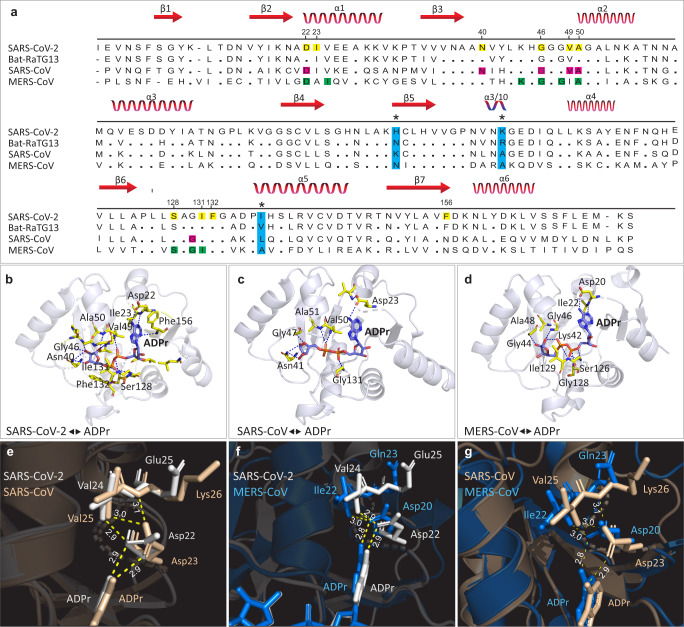
Sequence and structural comparison of Mac-1 protein in SARS-CoV-2 to other CoVs and Mac-1 capacity to bind ADPr. **a** Comparison of Mac-1 sequence of SARS-CoV-2 (YP_009725299.1) to that of MERS-CoV (PDB entry: 5HOL), SARS-CoV (PDB entry: 2FAV), and bat-RatG13 (QHR63299.1). Secondary structure elements are depicted in red at the top of the alignment. Alignment positions with fixed substitutions within Mac-1 that are specific to SARS-CoV-2 are highlighted in light blue columns with asterisk (_*_) symbol. Amino-acid residues that form a hydrogen bond with ADP-ribose (ADPr) are highlighted as yellow, pink, and green for SARS-CoV-2, SARS-CoV, and MERS-CoV, respectively. **b**–**d** Structures of the Mac-1 complex with ADPr. In each case, amino-acid residues that form a hydrogen bond with ADPr molecule are shown. **e**–**g** Comparison of interactions with ADPr in the Mac-1 adenine pocket of SARS-CoV-2, MERS-CoV, and SARS-CoV. Hydrogen bonds are shown as yellow dashed lines and bond lengths are given in Å units.

To further connect protein conformational changes with functions of macrodomains, we used a molecular docking approach to determine the binding affinity of ADPr to Mac-1. Structural comparisons revealed that there is considerable divergence in ADPr binding between SARS-CoV-2 and other CoVs (Fig. 5b–d). ADPr binding to the Mac-1 domain of SARS-CoV-2 (−9.46 kcal/mol) was found to be more efficient than in the human SARS-CoV (−8.59 kcal/mol) (Supplementary Table S8). Intriguingly, the binding affinity of SARS-CoV-2 Mac-1 for ADPr was comparable to that of MERS-CoV Mac-1 (−9.70 kcal/mol). This suggest that SARS-CoV-2 may evade host antiviral ADPr activity similar to that of the highly pathogenic MERS-CoV [50].

To gain insights into the molecular mechanisms behind the similar ADPr binding affinities of SARS-CoV-2 and MERS-CoV macrodomains, we investigated their binding clefts. In MERS-CoV, Asp-20 within the α1-helix of Mac-1 has been shown to be critical for binding specificity; its side chain forms a direct contact with ADPr through hydrogen bonding with the N-6 atom of the pyrimidine ring in the adenine moiety [51] (Fig. 5a, f, g). This residue is known to be conserved among macrodomains of CoVs. It determines the degrees of curvature at the adenine moieties within binding pocket, and hence the binding affinity of ADPr [51] (Fig. 5a). Previous reports that superposition the MERS-CoV macrodomains showed that the same oxygen atom on the Asp-20 side chain forms two hydrogen bonds; one with the N-6 atom in a pyrimidine ring of ADPr and the other with nitrogen in the Ile-22 backbone amide in the α1-helix. This results in the displacement of the Asp-20 side chain into the adenine cavity, which strengthens ADPr binding to effectively limit host antiviral ADPr activity [51]. In contrast, the equivalent residue of SARS-CoV, Asp-23 forms a only single hydrogen bond with ADPr via an oxygen atom in its side chain and binds to nitrogen atoms in Val-25 and Lys-26 of the α1-helix via another hydrogen bond [51]. Intriguingly, inspection of equivalent hydrogen bonding patterns in SARS-CoV-2 revealed a closer resemblance to MERS-CoV than to SARS-CoV (Fig. 5e, f). For example, in SARS-CoV-2, the oxygen atom on Asp22 side chain forms two hydrogen bonds, one with the N-6 atom in a pyrimidine ring of ADPr and the other with nitrogen in the Val-24 of the α1-helix, which is similar to MERS-CoV, and, accordingly, may displace the Asp22 side chain into the adenine cavity (Fig. 5f). Furthermore, studies of MERS-CoV provide evidence that the stabilization of ADPr in the binding pocket of Mac-1 is mainly determined by hydrogen-bond strength [51]. In the MERS-CoV the hydrogen-bond lengths formed by the Asp-20 side chain with ADPr and Ile-22 are 2.9 and 3.0 Å, respectively (Fig. 5f). This is comparable to the SARS-CoV-2 hydrogen-bond lengths formed by the Asp-23 side chain with ADPr and Val-24, which for both is 2.9 Å (Fig. 5e, f). Conceivably, the comparable affinities of MERS-CoV and SARS-CoV-2 macrodomains for ADPr may partly result from similarities in the their hydrogen bonding patterns, hydrogen-bond lengths/strength, and the positioning of the side chains of Asp-20/Asp-22 with respect to cleft that holds the adenine moiety. Thus, it appears that the biophysical and structural aspects of ADPr binding site evolution may provide SARS-CoV-2 and MERS-CoV with extraordinary adaptive abilities, which enable these viruses to evade host innate defense pathways. To further validate these findings, we analyzed the druggability scores of ADPr binding pockets of Mac-1 of SARS-CoV, MERS-CoV, and SARS-CoV-2 by using DoGSiteScorer web server [41]. Binding clefts of MERS-CoV and SARS-CoV-2 depict high druggability scores as compared to SARS-CoV (Supplementary Table S9). Higher druggability scores are considered to reflect a greater ability of protein pocket to bind to its target ligand [41]. Thus, druggability assessment suggests that macrodomains of MERS-CoV and SARS-CoV-2 are more druggable and hence provide better binding cavities for conformational optimization of ADPr as compared to SARS-CoV.

Previously, it was suggested that the differential binding affinity of Mac-1 for ADPr may result from different amino-acid compositions in the α1-helix [51]. Here, we showed that, though the corresponding residues within the α1-helices of SARS-CoV-2 and MERS-CoV are highly divergent, their binding affinities for ADPr are similar (Fig. 5a; Supplementary Table S8). Therefore, it is possible that the evolved increase in binding affinity of SARS-CoV-2 Mac-1 for ADPr may have been facilitated by epistatic effects of three fixed amino-acid substitutions located near the binding cleft, but that do not contact the ADPr via direct hydrogen bonding (Fig. 5a). Evolution by protein conformational epistasis may play a significant role in differential binding affinities of Mac-1 for ADPr [43]. Interestingly, differential binding affinities of Mac-1 for ADPr have been associated with the differences in the pathogenicity of coronaviruses [45, 51]. Thus, the comparable affinities of SARS-CoV-2 and MERS-CoV macrodomains for ADPr fits well with the notion that current mortality rates grossly underestimate the threat posed by COVID-19 [6].

This study has revealed functionally unique amino-acid replacements within the macrodomains of Nsp3, that are likely to maximize SARS-CoV-2 activity against human innate immune responses. The precise residue-level structural information in our study may benefit the design of anti-SARS-CoV-2 drug treatments. Macrodomains have previously been shown to be potent drug targets. For instance, an AlphaScreen based assay has recently identified a small molecule inhibitor, GeA-69 (a carbazole-based compound) that targets the macrodomain of human PARP14 (poly-ADP-ribose polymerase 14), a pro-survival protein associated with human inflammatory diseases and various types of cancers [52]. In addition, the combination of structure-based virtual screening and molecular dynamics simulation approaches have been successful in identifying potential inhibitors targeting viral macrodomains [53].

## Supplementary information

Supplementary Figures

Supplementary Tables

Supplementary Alignment File
