# Supplementary material for: Evolutionary and structural analysis of SARS-CoV-2 specific evasion of host immunity
Source: Genes Immun. 2020 Dec 3;21(6):409–19. doi: 10.1038/s41435-020-00120-6 (PMC7711619; doi:10.1038/s41435-020-00120-6)
Supplement: Supplementary file 1 — Supplementary Figures [file 41435_2020_120_MOESM1_ESM.pdf]

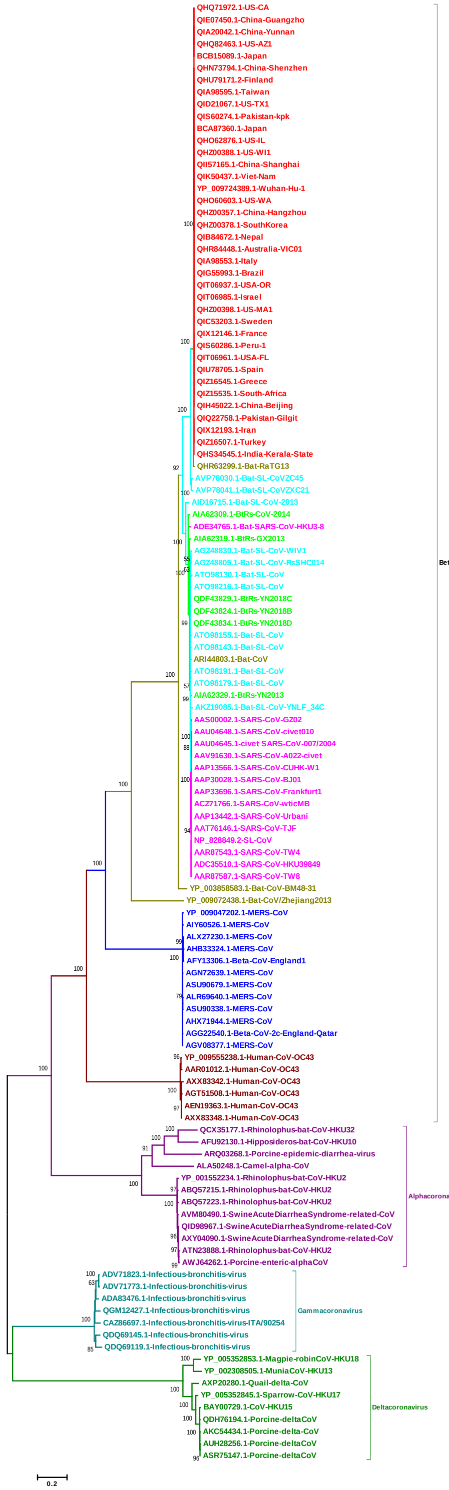

Supplementary Fig. S1. Maximum likelihood tree of SARS-CoV-2 based on the 7,096-aa polyprotein pp1ab. Phylogenetic analysis involved 121 pp1ab sequences from the subfamily Coronavirinae, including representatives of four genera; α -CoV, β-CoV ,γ-CoV, and δ-CoV. Phylogenetic tree was reconstructed using the maximum likelihood method with the WAG substitution model. Bootstrap values >50% are shown along the branches. Scale bar shows amino acid substitution per site. Tree branches representing novel coronavirus SARS-CoV-2 are shown in red color. The other Coronavirinae species are color coded as follows: Alphacoronavirus; purple, Betacoronavirus; olive Bat-CoV, aqua Bat-SL-CoV, lime BtRs-Beta-CoV, fuchsia SARS-CoV, blue MERS-CoV, maroon Human-CoV-OC43, Gammacoronavirus; teal and Deltacoronavirus; green.

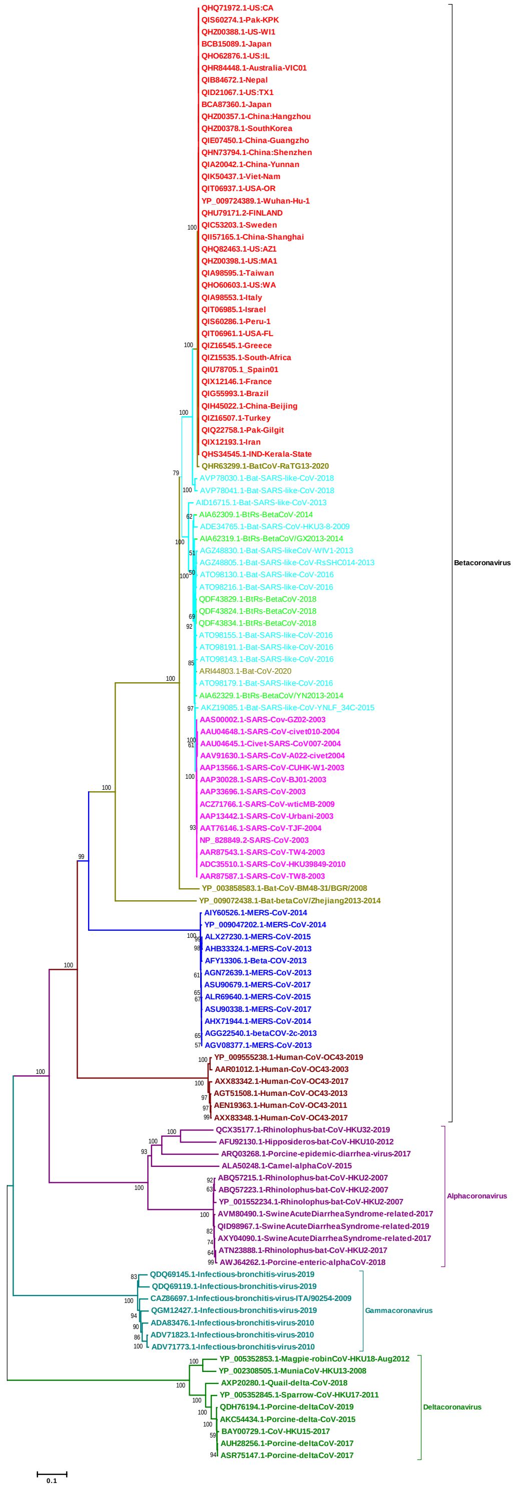

Supplementary Fig. S2. Neighbor Joining tree of SARS-CoV-2 based on the 7,096-aa polyprotein pp1ab. Phylogenetic analysis involved 121 pp1ab sequences from the subfamily Coronavirinae, including representatives of four genera; α -CoV, β-CoV ,γ-CoV, and δ-CoV.. The phylogenetic tree was reconstructed using the neighbor Joining (NJ) method with Jones-Taylor-Thornton (JTT) amino acid substitution model. Bootstrap values >50% are shown along the branches. Scale bar shows amino acid substitution per site. Tree branches representing novel coronavirus SARS-CoV-2 are shown in red color. The other Coronavirinae species were color coded as Alphacoronavirus; purple, Betacoronavirus; olive Bat-CoV, aqua Bat-SL-CoV, lime BtRs-Beta-CoV, fuchsia SARS-CoV, blue MERS-CoV, maroon Human-CoV-OC43, Gammacoronavirus ;teal and Deltacoronavirus; green.

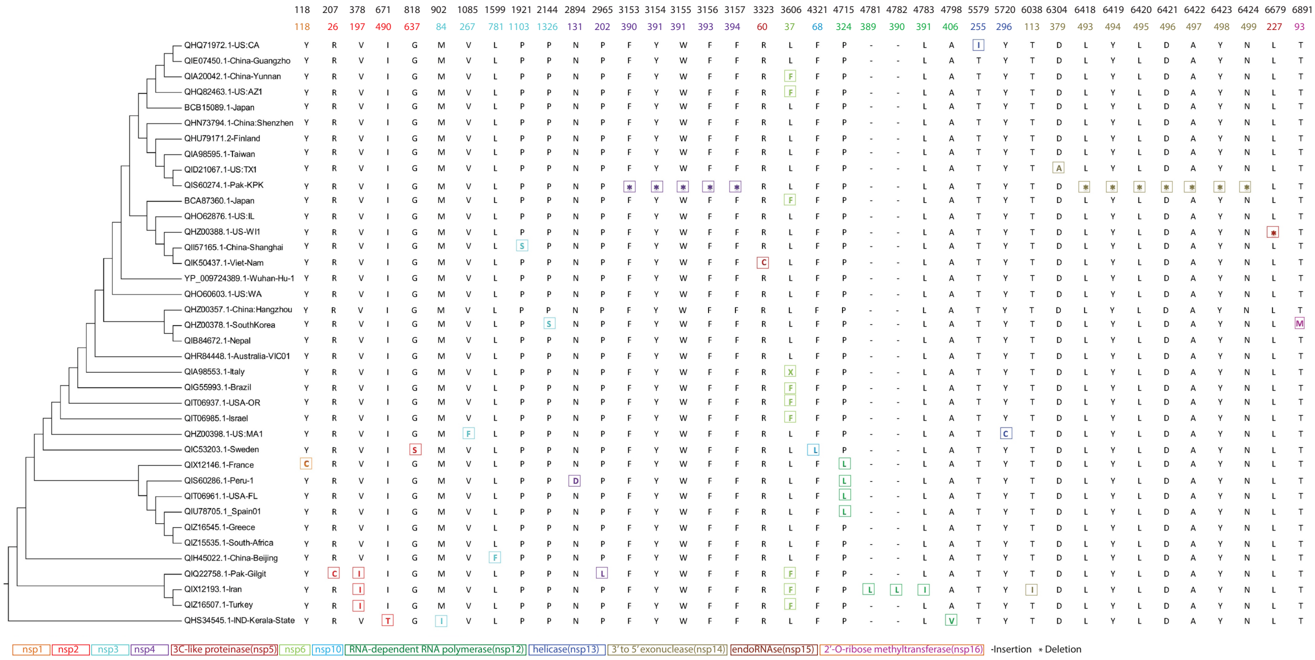

Supplementary Fig. S3. Individual or region specific substitutions/indel within poly-protein pp1ab of SARS-COV-2. Poly-protein pp1ab comparison among the currently available worldwide data of SARS-CoV-2 from 38 distinct global territories.Amino acids positions with respect to poly-protein pp1ab and non structural proteins (Nsps: color coded) are given. Individual or region specific substitutions and indels are color coded with respect to specific non-structural proteins. Pak-KPK pp1ab (QIS60274.1) appears to be the most divergent among the analyzed SARS-CoV-2 genomes harboring 12 deletions. Asterisk (*) symbols indicate deletion, while short-dash line represents insertions.

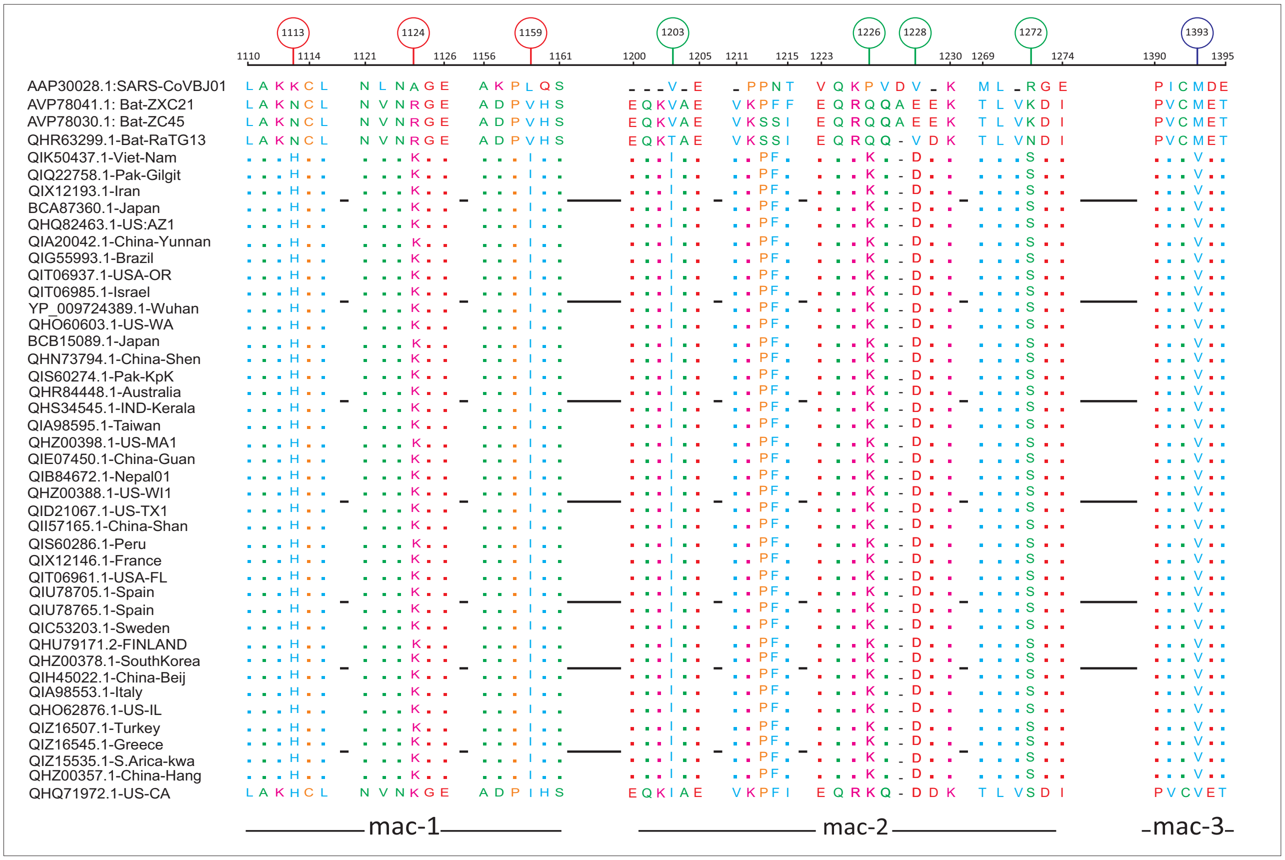

Supplementary Fig. S4. Fixed amino acid replacements within macrodomains of SARS-CoV-2 Multiple sequence alignments of macrodomains containing protein segments (within Nsp3) of pp1ab from SARS-CoV-2 genomes sampled from 39 distinct global territories. These alignments also include corresponding homologous sequences from bat-RaTG13 (QHR63299.1), bat-ZC45 (AVP78030.1), bat-ZXC21 (AVP78041.1) and SARS-CoVBJ01 (AAP30028.1). Green, red and blue circles, respectively, differentiate between Mac-1, Mac-2 and Mac-2 specific substitutions.
