# Supplementary material for: Evolutionary and structural analysis of SARS-CoV-2 specific evasion of host immunity
Source: Genes Immun. 2020 Dec 3;21(6):409–19. doi: 10.1038/s41435-020-00120-6 (PMC7711619; doi:10.1038/s41435-020-00120-6)
Supplement: Supplementary file 2 — Supplementary Tables [file 41435_2020_120_MOESM2_ESM.pdf]

Supplementary Table S1. The pp1ab polyprotein sequences derived from the genomes of SARS-CoV-2 sampled from multiple, distinct geographic locations.

S.No	Genome accession (NCBI)	Protein accession (NCBI)	Protein Length (aa)	Collection Date (NCBI)	Locality
1	NC_045512 (NCBI-RefSeq)	YP_009724389.1	7096	2019-12	China
2	LC528232	BCA87360.1	7096	2020-02-10	Japan
3	LC529905	BCB15089.1	7096	2020-01	Japan
4	MN938384	QHN73794.1	7096	2020-01-10	China: Shenzhen
5	MN985325	QHO60603.1	7096	2020-01-19	US:WA
6	MN988713	QHO62876.1	7096	2020-01-21	US:IL
7	MN994468	QHQ71972.1	7096	2020-01-22	US:CA
8	MN997409	QHQ82463.1	7096	2020-01-22	US:AZ1
9	MT007544	QHR84448.1	7096	2020-01-25	Australia-VIC01
10	MT012098	QHS34545.1	7096	2020-02-27	IND-Kerala-State
11	MT020781	QHU79171.2	7096	2020-01-29	FINLAND
12	MT039873	QHZ00357.1	7096	2020-01-20	China:Hangzhou
13	MT039887	QHZ00388.1	7096	2020-01-31	US-WI1
14	MT039888	QHZ00398.1	7096	2020-01-29	US-MA1
15	MT039890	QHZ00378.1	7096	2020-01	SouthKorea
16	MT049951	QIA20042.1	7096	2020-01-17	China-Yunnan
17	MT066156	QIA98553.1	7096	2020-01-30	Italy
18	MT066175	QIA98595.1	7096	2020-01-31	Taiwan
19	MT072688	QIB84672.1	7096	2020-01-13	Nepal
20	MT093571	QIC53203.1	7096	2020-02-07	Sweden
21	MT106054	QID21067.1	7096	2020-02-11	US-TX1
22	MT121215	QII57165.1	7096	2020-02-02	China-Shanghai
23	MT123290	QIE07450.1	7096	2020-02-05	China-Guangzho
24	MT126808	QIG55993.1	7096	2020-02-28	Brazil
25	MT135041	QIH45022.1	7096	2020-01-26	China-Beijing
26	MT192772	QIK50437.1	7096	2020-01-22	Viet-Nam
27	MT240479	QIQ22758.1	7096	2020-03-04	Pakistan-Gilgit
28	MT262993	QIS60274.1	7084	2020-03-12	Pakistan: KPK
29	MT263074	QIS60286.1	7096	2020-03-10	Peru
30	MT276597	QIT06985.1	7096	2020-02	Israel
31	MT276330	QIT06961.1	7096	2020-02-28	USA: FL
32	MT276328	QIT06937.1	7096	2020-02-27	USA: OR
33	MT292569	QIU78705.1	7096	2020-03-09	Spain01
34	MT292574	QIU78765.1	7096	2020-03-02	Spain02
35	MT320891	QIX12193.1	7098	2020-03-09	Iran
36	MT320538	QIX12146.1	7096	2020-03	France
37	MT327745	QIZ16507.1	7096	2020-03-17	Turkey
38	MT328033	QIZ16545.1	7096	2020-03-18	Greece
39	MT324062	QIZ15535.1	7096	2020-03-07	South Africa: KwaZulu-Natal

This table depicts NCBI derived accession numbers of pp1ab polyproteins, their respective amino acid (aa) length and submission date at NCBI. Accession numbers of concerned SARS-CoV-2 genomes and source locality is also given.

**Supplementary Table S2. The pp1ab polyprotein sequences from the subfamily *Coronavirinae*, including representatives of four genera: Alphacoronavirus, Betacoronavirus, Gammacoronavirus, and Deltacoronavirus**					
**S.No**	**Protein accession NCBI**	**Protein length (aa)**	**Name**	**Genera**	**Year**
1	NP_828849.2	7073	SARS-CoV	Betacoronavirus	2003
2	YP_009047202.1	7078	MERS-CoV	Betacoronavirus	2012
3	YP_009072438.1	7247	Bat Hp-beta-CoV/ Zhejiang2013	Betacoronavirus	2013
4	ATO98143.1	7073	Bat SARS-like-CoV	Betacoronavirus	2016
5	AAS00002.1	7073	SARS-Cov GZ02	Betacoronavirus	2003
6	QDF43824.1	7073	BtRs-BetaCoV/YN2018B	Betacoronavirus	2018
7	AIA62309.1	7070	BtRs-BetaCoV/HuB2013	Betacoronavirus	2014
8	AAV91630.1	7073	SARS-CoV A022	Betacoronavirus	2004
9	QHR63299.1	7095	BatCoV RaTG13	Betacoronavirus	2020
10	AVP78030.1	7092	Bat SARS-like CoV (bat-SL-CoVZC45)	Betacoronavirus	2018
11	AID16715.1	7063	Bat SARS-like CoV	Betacoronavirus	2013
12	AGZ48830.1	7073	Bat SARS-like CoV WIV1	Betacoronavirus	2013
13	AAP13566.1	7073	SARS CoV CUHK-W1	Betacoronavirus	2003
14	ACZ71766.1	7073	SARS CoV wtic-MB	Betacoronavirus	2009
15	AAP13442.1	7073	SARS CoV Urbani	Betacoronavirus	2003
16	AAR87543.1	7073	SARS CoV TW4	Betacoronavirus	2003
17	ADC35510.1	7073	SARS CoV HKU-39849	Betacoronavirus	2010
18	AAP33696.1	7073	SARS CoV	Betacoronavirus	2003
19	ATO98130.1	7073	Bat SARS-like CoV	Betacoronavirus	2016
20	AGZ48805.1	7073	Bat SARS-like CoV RsSHC014	Betacoronavirus	2013
21	AAP30028.1	7073	SARS CoV BJ01	Betacoronavirus	2003
22	AAR87587.1	7073	SARS CoV TW8	Betacoronavirus	2003
23	ATO98216.1	7073	Bat SARS-like CoV	Betacoronavirus	2016
24	AVP78041.1	7070	Bat SARS-like CoV (bat-SL-CoVZXC21)	Betacoronavirus	2018
25	ARI44803.1	7073	Bat CoV	Betacoronavirus	2017
26	ATO98191.1	7073	Bat SARS-like CoV	Betacoronavirus	2016
27	QDF43834.1	7073	CoV BtRs-BetaCoV/YN2018D	Betacoronavirus	2018
28	QDF43829.1	7073	CoV BtRs-BetaCoV/YN2018C	Betacoronavirus	2018
29	ATO98155.1	7073	Bat SARS-like CoV	Betacoronavirus	2016
30	ATO98179.1	7073	Bat SARS-like CoV	Betacoronavirus	2016
31	AFY13306.1	7078	BetaCOV England 1	Betacoronavirus	2013
32	AGG22540.1	7078	Human betaCOV 2c England-Qatar/2012	Betacoronavirus	2013
33	AGV08377.1	7078	MERS-CoV	Betacoronavirus	2013
34	ASU90679.1	7078	MERS-CoV	Betacoronavirus	2017
35	AHX71944.1	7078	MERS-CoV	Betacoronavirus	2014

36	AGN72639.1	7078	MERS-CoV	Betacoronavirus	2013
37	ALR69640.1	7078	MERS-CoV	Betacoronavirus	2015
38	AIY60526.1	7078	MERS-CoV	Betacoronavirus	2014
39	ASU90338.1	7078	MERS-CoV	Betacoronavirus	2017
40	ALX27230.1	7078	MERS-CoV	Betacoronavirus	2015
41	AHB33324.1	7078	MERS-CoV	Betacoronavirus	2013
42	YP_009555238.1	7095	Human CoV-OC43	Betacoronavirus	2019
43	AAR01012.1	7095	Human CoV-OC43	Betacoronavirus	2003
44	AGT51508.1	7095	Human CoV-OC43	Betacoronavirus	2013
45	AEN19363.1	7095	Human CoV-OC43	Betacoronavirus	2011
46	AXX83342.1	7095	Human CoV-OC43	Betacoronavirus	2017
47	AXX83348.1	7095	Human CoV-OC43	Betacoronavirus	2017
48	YP_001552234.1	6727	Rhinolophus bat CoV HKU2	Alphacoronavirus	2007
49	ABQ57215.1	6727	Rhinolophus bat CoV HKU2	Alphacoronavirus	2007
50	ABQ57223.1	6727	Rhinolophus bat CoV HKU2	Alphacoronavirus	2007
51	AVM80490.1	6728	Swine acute diarrhea syndrome related CoV	Coronavirinae	2017
52	AXY04090.1	6728	Swine acute diarrhea syndrome CoV	Coronavirinae	2017
53	QID98967.1	6728	Swine acute diarrhea syndrome CoV	Coronavirinae	2019
54	ATN23888.1	6728	Rhinolophus bat CoV HKU2	Alphacoronavirus	2017
55	AWJ64262.1	6728	Porcine enteric alphaCoV	Alphacoronavirus	2018
56	QCX35177.1	6712	Rhinolophus bat CoV HKU32	Alphacoronavirus	2019
57	AFU92130.1	6783	Hipposideros bat CoV HKU10	Alphacoronavirus	2012
58	ALA50248.1	6763	Camel alphaCoV	Alphacoronavirus	2015
59	ARQ03268.1	6781	Porcine epidemic diarrhea virus	Alphacoronavirus	2017
60	ADE34765.1	7071	Bat SARS CoV HKU3-8	Betacoronavirus	2009
61	AKZ19085.1	7073	Bat SARS-like CoV YNLF_34C	Betacoronavirus	2015
62	AIA62319.1	7071	BtRs-BetaCoV/GX2013	Betacoronavirus	2014
63	AAU04648.1	7073	SARS CoV civet010	Betacoronavirus	2004
64	AAU04645.1	7073	Civet SARS CoV 007/2004	Betacoronavirus	2004
65	AAT76146.1	7073	SARS CoV TJF	Betacoronavirus	2004
66	AIA62329.1	7073	BtRs-BetaCoV/YN2013	Betacoronavirus	2014
67	ADV71823.1	6630	Infectious bronchitis virus	Gammacoronavirus	2010
68	ADV71773.1	6630	Infectious bronchitis virus	Gammacoronavirus	2010
69	ADA83476.1	6630	Infectious bronchitis virus-Mass41	Gammacoronavirus	2009
70	CAZ86697.1	6631	Infectious bronchitis virus	Gammacoronavirus	2009

			ITA/90254/2005		
71	QGM12427.1	6639	Infectious bronchitis virus	Gammacoronavirus	2019
72	QDQ69145.1	6627	Infectious bronchitis virus	Gammacoronavirus	2019
73	QDQ69119.1	6623	Infectious bronchitis virus	Gammacoronavirus	2019
74	AUH28256.1	6265	Porcine delta CoV	Coronavirinae	2017
75	ASR75147.1	6265	Porcine delta CoV	Coronavirinae	2017
76	AKC54434.1	6267	Porcine delta CoV	Deltacoronavirus	2015
77	QDH76194.1	6265	Porcine delta CoV	Deltacoronavirus	2019
78	BAY00729.1	6262	Coronavirus HKU15	Coronavirinae	2017
79	YP_005352845.1	6277	Sparrow CoV HKU17	Deltacoronavirus	2011
80	AXP20280.1	6260	Quail delta CoV	Deltacoronavirus	2018
81	YP_005352853.1	6253	Magpie-robin CoV HKU18	Deltacoronavirus	2011
82	YP_002308505.1	6307	Munia CoV HKU13-3514	Deltacoronavirus	2008
83	YP_003858583.1	7064	Bat-CoV: BM48-31/BGR/2008	Unclassified	2009

This table depicts the NCBI (National Center for Bioinformatics) derived accession numbers of pp1ab polyproteins, their amino acid (aa) length, defined name in literature and taxonomic classification. Dates of submission in NCBI are also given in the last column. Note: The list of corresponding homologous sequences from SARS-CoV-2 are given in Table 1.

Supplementary Table S3. Fixed amino acid substitutions in polyprotein pp1ab of SARS-CoV-2.

S.No	Amino Acid Position	Bat-SL-CoV (AVP78030.1/ AVP78041. 1)	Bat-CoV-RaTG13 (QHR63299.1)	SARS-CoV-2 (YP_009725299.1)	Neutral/Radical	Localization
1	38	A	A	V	Neutral (0)	nsp1 (leader protein)
2	93	D	N	E	Neutral (0)	nsp1 (leader protein)
3	114	V	T	I	Radical (-1)	nsp1 (leader protein)
4	280	T	T	I	Radical (-1)	nsp2
5	376	P	P	S	Radical (-1)	nsp2
6	417	Y	Y	H	Radical (+2)	nsp2
7	498	T	T	A	Neutral (0)	nsp2
8	591	V	V	A	Neutral (0)	nsp2
9	859	S/N	T	A	Neutral (0)	nsp3
10	940	Q	D	E	Radical (+2)	nsp3
11	967	F	V	A	Neutral (0)	nsp3
12	968	S	T	A	Neutral (0)	nsp3
13	969	S	P	L	Radical (-3)	nsp3
14	991	-	E	Q	Radical (+2)	nsp3
15	993	A	D	G	Radical (-1)	nsp3
16	996	-	V	D	Radical (-3)	nsp3
17	1001	N	I	T	Radical (-1)	nsp3
18	1002	K	T	I	Radical (-1)	nsp3
19	1004	P	S	T	Radical (+1)	nsp3
20	1016	S	P	L	Radical (-3)	nsp3
21	1023	S	-	I	Radical (-2)	nsp3
22	1089	S/K	H	D	Radical (-1)	nsp3
23	1113	N	N	H	Radical (+1)	nsp3
24	1124	R	R	K	Radical (+2)	nsp3
25	1159	V	V	I	Radical (+3)	nsp3
26	1203	T	T	I	Radical (-1)	nsp3
27	1226	Q	Q	K	Radical (+1)	nsp3
28	1228	A	V	D	Radical (-3)	nsp3
29	1272	K	N	S	Radical (+1)	nsp3
30	1393	M	M	V	Radical (+1)	nsp3
31	1733	N	N	S	Radical (+1)	nsp3
32	1779	L	L	F	Neutral (0)	nsp3
33	1795	Q	Q	K	Radical (+1)	nsp3
34	1822	V	I	T	Radical (-1)	nsp3
35	2033	T	T	A	Neutral (0)	nsp3
36	2061	I	I	V	Radical (+3)	nsp3
37	2082	D	D	N	Radical (+1)	nsp3
38	2083	G	G	S	Neutral (0)	nsp3
39	2405	T	T	N	Neutral (0)	nsp3
40	3143	V	V	A	Neutral (0)	nsp4
41	3145	V	V	I	Radical (+3)	nsp4
42	3443	T	T	N	Neutral (0)	3C-like proteinase (nsp5)
43	3606	V	V	F/L	Radical (-1)/(+1)	nsp6
44	4590	D	D	N	Radical (+1)	RNA-dependent RNA polymerase (nsp12)
45	4625	I	I	V	Radical (+3)	RNA-dependent RNA polymerase (nsp12)
46	6137	K	K	R	Radical (+2)	3'-to-5' exonuclease (nsp14)
47	6184	Q	R	L	Radical (-2)	3'-to-5' exonuclease (nsp14)
48	6566	N/S	N	T	Neutral (0)	endoRNAse (nsp15)
49	6580	E	N	D	Neutral (0)	endoRNAse (nsp15)
50	6653	E	K	Q	Radical (+1)	endoRNAse (nsp15)
51	6695	G	R	S	Radical (-1)	endoRNAse (nsp15)
52	6710	S	S	F	Radical (-2)	endoRNAse (nsp15)
53	6715	L	L	F	Neutral (0)	endoRNAse (nsp15)

This table shows the SARS-CoV-2 specific replacements in polyprotein pp1ab since its divergence from bat coronaviruses . The 6th column illustrates the putative physicochemical impact of each substitution on protein structure and function. The numbers in the brackets are the log-odds score for each substitution that is prophesied according to BLOSUM 62 substitution matrix. Negative number indicates less likely substitution while positive number point toward preferred substitutions. The 7th column symbolizes the protein name where particular substitution resides. Amino acid positions in the second column are according to SARS-CoV-2 pp1ab .

Supplementary Table S4. Intra-clade comparison of fixed Amino acid substitutions in nsp3 of SARS-CoV-2

S.No	Amino Acid position-orf1ab	Amino Acid position-nsp3	YP_009725299.1	G	GH	GR	L	O	S	V
1	859	41	A	A	A	A	A	A	A	A
2	940	123	E	E	E	E	E	E	E	E
3	967	149	A	A	A	A	A	A	A	A
4	968	150	A	A	A	A	A	A	A	A
5	969	151	L	L	L	L	L	L	L	L
6	991	173	Q	Q	Q	Q	Q	Q	Q	Q
7	993	175	G	G	G	G	G	G	G	G
8	996	178	D	D	D	D	D	D	D	D
9	1001	183	T	T	T	T	T	T	T	T
10	1002	184	I	I	I	I	I	I	I	I
11	1004	186	T	T	T	T	T	T	T	T
12	1016	198	L	L	L	L	L	L	L	L
13	1023	205	I	I	I	I	I	I	I	I
14	1089	271	D	D	D	D	D	D	D	D
15	1113	295	H	H	H	H	H	H	H	H
16	1124	306	K	K	K	K	K	K	K	K
17	1159	341	I	I	I	I	I	I	I	I
18	1203	385	I	I	I	I	I	I	I	I
19	1226	408	K	K	K	K	K	K	K	K
20	1228	410	D	D	D	D	D	D	D	D
21	1272	454	S	S	S	S	S	S	S	S
22	1393	575	V	V	V	V	V	V	V	V
23	1733	905	S	S	S	S	S	S	S	S
24	1779	961	F	F	F	F	F	F	F	F
25	1795	977	K	K	K	K	K	K	K	K
26	1822	1004	T	T	T	T	T	T	T	T
27	2033	1205	A	A	A	A	A	A	A	A
28	2061	1243	V	V	V	V	V	V	V	V
29	2082	1264	N	N	N	N	N	N	N	N
30	2083	1265	S	S	S	S	S	S	S	S
31	2405	1587	N	N	N	N	N	N	N	N

This table shows the intra clade comparison of SARS-CoV-2 specific replacements in nsp3 since its divergence from bat coronaviruses. The putative physicochemical impact of amino acid substitution in column 4 on protein structure and function are given in supplementary table S3. Column 2 and 3 depicts the position of these residues with respect to orf1ab and nsp3 respectively. G, GH,GR, L, O ,S and V are seven distinct clades of SARS-CoV-2 reported in GISAID (https://www.gisaid.org). Green highlihted sections depict amino acid replacements in the macrodomains of SARS-CoV-2.

Supplementary Table S5. Fixed amino acid replacements in the macrodomains of SARS-CoV-2.

S.No	Substitution Positions	Domain type	Neutral/Radical	Impact on protein stabality (ΔΔG)
1	N295H	Macrodomain-1 (X domain)	Radical (+1)	Destabilizing (-0.396)
2	R306K	Macrodomain-1 (X domain)	Radical (+2)	Destabilizing (-0.586)
3	V341I	Macrodomain-1 (X domain)	Radical (+3)	Destabilizing (-0.035)
4	T385I	Macrodomain-2	Radical (-1)	Destabilizing (-1.803)
5	Q408K	Macrodomain-2	Radical (+1)	Destabilizing (-0.604)
6	V410D	Macrodomain-2	Radical (-3)	Destabilizing (-0.29)
7	N454S	Macrodomain-2	Radical (+1)	Destabilizing (-0.849)
8	M575V	Macrodomain-3	Radical (+1)	Destabilizing (-0.392)

After splitting from bat SARS-CoVs (RaTG13/ZC45/ZXC21), macrodomains of SARS-CoV-2 (YP_009725299.1) have experienced in total eight fixed amino acid replacements. This table depicts positions of these replacements (with respect to Nsp3 protein), their location within specific macrodomain sub-types. The 4th column depicts the putative physicochemical impact of each replacement on protein/structure function, the number within brackets are the log odds associated with changing the amino acids. Positive numbers imply a preferred change, zero implies a neutral change and negative numbers imply an un-preferred change. The 5th column depicts the putative impact of each replacement on the stability of protein structure. The number within brackets depicts the protein stability free energy change (ΔΔG) upon single amino acid substitution. ΔΔG (kcal/mole) is calculated by using structure information. Positive values imply an increase in the stability of protein structure. Conversely, negative numbers imply a decrease in the stability of protein structure.

**Supplementary Table S6. Comparsion of secondary structure elements (SSEs) in macrodomains.**			
**Coronavirus**	**SSEs Type**		
	**Alpha helix**	**Beta Sheets**	**Random coils**
SARS-CoV2 (YP_009725299.1)	38.56% (209 residues)	23.06% (125 residues)	38.38% (208 resides)
bat-RaTG13 (QHR63299.1)	40.85% (221 residues)	22.37% (121 residues)	36.79% (199 resides)
SARS-CoV (BJ01:AAP30028.1)	37.15% (198 residues)	19.14% (102 residues)	43.71% (237 resides)
Ancestor: SARS-CoV-2 and bat-RaTG13/ZC45/ZXC21	38.31% (208 residues)	21.36% (116 residues)	40.33% (219 resides)

This table summarizes the differences in % population of the SSEs in macrodomains of various coronaviruses (CoVs) listed in the first column. The GenBank accession number of the protein sequence is given within brackets; first column. The Number within brackets in the second, third and fourth column depicts the total number of amino acid residues occupied by each SSE type.

Supplementary Table S7. Structural deviations in the backbone torsion angles of the macrodomain-1 and macrodomain-2

3D-superimposition of protein structures between lineages	Major changes in the backbone torsion angles(Φ °,Ψ °)residue numbers)		Noticeable changes in secondary structural elements (SSEs)	
	Macrodomain-1	Macrodomain-2	Macrodomain-1	Macrodomain-2
Ancestor vs SARS-CoV-2	11-16, 100-106	199-236, 272-279,305-312,	10-11,78-80,130-133,156-168,	222-226, 246-252,268-273,279-285,291-293,301-304
bat-RaTG13 vs SARS-CoV-2	4-16,20-22,39-48	209-222,323-327,	10-11, 27-29,78-80,118-120, 156-168,	222-226, 237-238,246-252, 268-273,307-309
SARS-CoV vs SARS-CoV-2	11-16, 18-22, 161-168	199-245	11-16, 161-168	200-204,222-226,308-310,
MERS-CoV vs SARS-CoV-2	1-5, 25-32, 82-91,	-	1-9, 78-80, 97-101,	-

The 2nd and 3rd columns of this table shows the effect of lineage specific substitutions on the backbone torsion angles of SARS-CoV-2 (YP_009725299.1) macrodomains (Macrodomain 1 and Macrodomain 2) in comparison with corresponding macrodomains of bat-RaTG13 (QHR63299.1), SARS-CoV (BJ01:AAP30028.1), MERS-CoV (PDB entry: 5HOL) and predicted ancestor of SARS-CoV-2 and bat-RaTG13/ZC45/ZXC21. 5th and 6th columns show the residue positions for noticeable transitions in SSEs within Macrodomain 1 and Macrodomain 2 of SARS-CoV-2, in comparison with corresponding homologous macrodomains of bat-RaTG13 (QHR63299.1), SARS-CoV (BJ01:AAP30028.1), MERS-CoV (PDB entry: 5DUS) and predicted ancestor of SARS-CoV-2 and bat-RaTG13/ZC45/ZXC21. Note: MERS-CoV genome does not code for macrodomain-2 and hence not analyzed here.

Supplementary Table S8. ADPr-binding pattern of macrodomain-1			
**S. No**	**Virus type**	**Interacting residues with ADPr**	**Docking Score (kcal/mol)**
1	MERS-CoV (PDB entry: 5DUS)	Asp20, Ile21, Lys42, Gly44,Gly46, Ala48, Ser126, Gly128, Ile129	-9.70
2	SARS-CoV-2 (YP_009725299.1)	Asp22, Ile23, Asn40, Gly46, Val49, Ala50, Ser128, Phe132, Ile131, Phe156	-9.46
3	SARS-CoV (PDB entry: 2FAV)	Asp23, Asn41, Gly47, Val50, Ala51, Gly131	-8.59

This table shows the results of molecular docking of ADP-ribose molecule with the macrodomain-1 of different coronaviruses. Amino acid residues of macrodomain-1 which are actively involved in the hydrogen bonding with the ADP-ribose molecule are given in the 3rd column. The docking scores (kcal/mol) are given in the 4th column. The lowest the docking score the higher is the binding affinity. ADPr; adenosine diphosphate (ADP)-ribose.

**Supplementary Table S9: Predicted features (Volume, Surface area and Drug score) of the MERS-CoV, SARS-CoV and SARs-CoV-2 macrodomain-I.**			
**Virus type**	**Volume (Å^3)**	**Surface Area (Å^3)**	**Druggability score**
MERS-CoV (PDB entry: 5DUS)	733.31	873.7	0.82
SARs-CoV-2 (YP_009725299.1)	705.02	765.66	0.81
SARS-CoV (PDB entry: 2FAV)	697.6	888.71	0.79

This table shows comparison of volume and surface area and druggability scores of the binding cleft of Mac1 of three coronaviruses. The higher the druggability score the more druggable the binding pocket is.
