# Supplementary material for: Evolutionary and structural analysis of SARS-CoV-2 specific evasion of host immunity
Source: Genes Immun. 2020 Dec 3;21(6):409–19. doi: 10.1038/s41435-020-00120-6 (PMC7711619; doi:10.1038/s41435-020-00120-6)
Supplement: Supplementary file 3 — Supplementary Alignment File [file 41435_2020_120_MOESM3_ESM.pdf]

Supplementary Alignment File: Concatenated alignments of 126 sequences (polyprotein pp1ab/Orfab of SARS-CoV-2) from 7 distinct clades (G, GH,GR, L, O ,S and V) of SARS-CoV-2 reported in GISAID (https://www.gisaid.org). Alignment (rich text format) of each distinct clade is represented by 18 sequences. Black highlighted residues represent Nsp3 protein.

Clade G

```
ORF1ab            1 MESLVPGFNEKTHVQLSLPVLQVRDVLVRGFGDSVEEVLSEARQHLKDGTCGLVEVEKGV
EPI_ISL_413575    1 ------------------------------------------------------------
EPI_ISL_413588    1 ------------------------------------------------------------
EPI_ISL_413589    1 ------------------------------------------------------------
EPI_ISL_424221    1 ------------------------------------------------------------
EPI_ISL_424233    1 ------------------------------------------------------------
EPI_ISL_424236    1 ------------------------------------------------------------
EPI_ISL_424238    1 ------------------------------------------------------------
EPI_ISL_424248    1 ------------------------------------------------------------
EPI_ISL_424254    1 ------------------------------------------------------------
EPI_ISL_425548    1 ------------------------------------------------------------
EPI_ISL_425581    1 ------------------------------------------------------------
EPI_ISL_426881    1 ------------------------------------------------------------
EPI_ISL_426883    1 ------------------------------------------------------------
EPI_ISL_426888    1 ------------------------------------------------------------
EPI_ISL_426900    1 ------------------------------------------------------------
EPI_ISL_426901    1 ------------------------------------------------------------
EPI_ISL_426903    1 ------------------------------------------------------------
EPI_ISL_426904    1 ------------------------------------------------------------
EPI_ISL_426907    1 ------------------------------------------------------------
EPI_ISL_426910    1 ------------------------------------------------------------
```

```
ORF1ab            1 MESLVPGFNEKTHVQLSLPVLQVRDVLVRGFGDSVEEVLSEARQHLKDGTCGLVEVEKGV
EPI_ISL_413575    1 ------------------------------------------------------------
EPI_ISL_413588    1 ------------------------------------------------------------
EPI_ISL_413589    1 ------------------------------------------------------------
EPI_ISL_424221    1 ------------------------------------------------------------
EPI_ISL_424233    1 ------------------------------------------------------------
EPI_ISL_424236    1 ------------------------------------------------------------
EPI_ISL_424238    1 ------------------------------------------------------------
EPI_ISL_424248    1 ------------------------------------------------------------
EPI_ISL_424254    1 ------------------------------------------------------------
EPI_ISL_425548    1 ------------------------------------------------------------
EPI_ISL_425581    1 ------------------------------------------------------------
EPI_ISL_426881    1 ------------------------------------------------------------
EPI_ISL_426883    1 ------------------------------------------------------------
EPI_ISL_426888    1 ------------------------------------------------------------
EPI_ISL_426900    1 ------------------------------------------------------------
EPI_ISL_426901    1 ------------------------------------------------------------
EPI_ISL_426903    1 ------------------------------------------------------------
EPI_ISL_426904    1 ------------------------------------------------------------
EPI_ISL_426907    1 ------------------------------------------------------------
EPI_ISL_426910    1 ------------------------------------------------------------
```

```
ORF1ab           61 LPQLEQPYVFIKRSDARTAPHGHVMVELVAELEGIQYGRSGETLGVLVPHVGEIPVAYRK
EPI_ISL_413575    1 ------------------------------------------------------------
EPI_ISL_413588    1 ------------------------------------------------------------
EPI_ISL_413589    1 ------------------------------------------------------------
EPI_ISL_424221    1 ------------------------------------------------------------
EPI_ISL_424233    1 ------------------------------------------------------------
EPI_ISL_424236    1 ------------------------------------------------------------
EPI_ISL_424238    1 ------------------------------------------------------------
EPI_ISL_424248    1 ------------------------------------------------------------
EPI_ISL_424254    1 ------------------------------------------------------------
EPI_ISL_425548    1 ------------------------------------------------------------
EPI_ISL_425581    1 ------------------------------------------------------------
EPI_ISL_426881    1 ------------------------------------------------------------
EPI_ISL_426883    1 ------------------------------------------------------------
EPI_ISL_426888    1 ------------------------------------------------------------
EPI_ISL_426900    1 ------------------------------------------------------------
EPI_ISL_426901    1 ------------------------------------------------------------
EPI_ISL_426903    1 ------------------------------------------------------------
EPI_ISL_426904    1 ------------------------------------------------------------
EPI_ISL_426907    1 ------------------------------------------------------------
EPI_ISL_426910    1 ------------------------------------------------------------
ORF1ab           61 LPQLEQPYVFIKRSDARTAPHGHVMVELVAELEGIQYGRSGETLGVLVPHVGEIPVAYRK
EPI_ISL_413575    1 ------------------------------------------------------------
EPI_ISL_413588    1 ------------------------------------------------------------
EPI_ISL_413589    1 ------------------------------------------------------------
EPI_ISL_424221    1 ------------------------------------------------------------
EPI_ISL_424233    1 ------------------------------------------------------------
EPI_ISL_424236    1 ------------------------------------------------------------
EPI_ISL_424238    1 ------------------------------------------------------------
EPI_ISL_424248    1 ------------------------------------------------------------
EPI_ISL_424254    1 ------------------------------------------------------------
EPI_ISL_425548    1 ------------------------------------------------------------
EPI_ISL_425581    1 ------------------------------------------------------------
EPI_ISL_426881    1 ------------------------------------------------------------
EPI_ISL_426883    1 ------------------------------------------------------------
EPI_ISL_426888    1 ------------------------------------------------------------
EPI_ISL_426900    1 ------------------------------------------------------------
EPI_ISL_426901    1 ------------------------------------------------------------
EPI_ISL_426903    1 ------------------------------------------------------------
EPI_ISL_426904    1 ------------------------------------------------------------
EPI_ISL_426907    1 ------------------------------------------------------------
EPI_ISL_426910    1 ------------------------------------------------------------
```

```
ORF1ab          121 VLLRKNGNKGAGGHSYGADLKSFDLGDELGTDPYEDFQENWNTKHSSGVTRELMRELNGG
EPI_ISL_413575    1 ------------------------------------------------------------
EPI_ISL_413588    1 ------------------------------------------------------------
EPI_ISL_413589    1 ------------------------------------------------------------
EPI_ISL_424221    1 ------------------------------------------------------------
EPI_ISL_424233    1 ------------------------------------------------------------
EPI_ISL_424236    1 ------------------------------------------------------------
EPI_ISL_424238    1 ------------------------------------------------------------
EPI_ISL_424248    1 ------------------------------------------------------------
EPI_ISL_424254    1 ------------------------------------------------------------
EPI_ISL_425548    1 ------------------------------------------------------------
EPI_ISL_425581    1 ------------------------------------------------------------
EPI_ISL_426881    1 ------------------------------------------------------------
EPI_ISL_426883    1 ------------------------------------------------------------
EPI_ISL_426888    1 ------------------------------------------------------------
EPI_ISL_426900    1 ------------------------------------------------------------
EPI_ISL_426901    1 ------------------------------------------------------------
EPI_ISL_426903    1 ------------------------------------------------------------
EPI_ISL_426904    1 ------------------------------------------------------------
EPI_ISL_426907    1 ------------------------------------------------------------
EPI_ISL_426910    1 ------------------------------------------------------------
ORF1ab          121 VLLRKNGNKGAGGHSYGADLKSFDLGDELGTDPYEDFQENWNTKHSSGVTRELMRELNGG
EPI_ISL_413575    1 ------------------------------------------------------------
EPI_ISL_413588    1 ------------------------------------------------------------
EPI_ISL_413589    1 ------------------------------------------------------------
EPI_ISL_424221    1 ------------------------------------------------------------
EPI_ISL_424233    1 ------------------------------------------------------------
EPI_ISL_424236    1 ------------------------------------------------------------
EPI_ISL_424238    1 ------------------------------------------------------------
EPI_ISL_424248    1 ------------------------------------------------------------
EPI_ISL_424254    1 ------------------------------------------------------------
EPI_ISL_425548    1 ------------------------------------------------------------
EPI_ISL_425581    1 ------------------------------------------------------------
EPI_ISL_426881    1 ------------------------------------------------------------
EPI_ISL_426883    1 ------------------------------------------------------------
EPI_ISL_426888    1 ------------------------------------------------------------
EPI_ISL_426900    1 ------------------------------------------------------------
EPI_ISL_426901    1 ------------------------------------------------------------
EPI_ISL_426903    1 ------------------------------------------------------------
EPI_ISL_426904    1 ------------------------------------------------------------
EPI_ISL_426907    1 ------------------------------------------------------------
EPI_ISL_426910    1 ------------------------------------------------------------
```

```
ORF1ab          181 AYTRYVDNNFCGPDGYPLECIKDLLARAGKASCTLSEQLDFIDTKRGVYCCREHEHEIAW
EPI_ISL_413575    1 ------------------------------------------------------------
EPI_ISL_413588    1 ------------------------------------------------------------
EPI_ISL_413589    1 ------------------------------------------------------------
EPI_ISL_424221    1 ------------------------------------------------------------
EPI_ISL_424233    1 ------------------------------------------------------------
EPI_ISL_424236    1 ------------------------------------------------------------
EPI_ISL_424238    1 ------------------------------------------------------------
EPI_ISL_424248    1 ------------------------------------------------------------
EPI_ISL_424254    1 ------------------------------------------------------------
EPI_ISL_425548    1 ------------------------------------------------------------
EPI_ISL_425581    1 ------------------------------------------------------------
EPI_ISL_426881    1 ------------------------------------------------------------
EPI_ISL_426883    1 ------------------------------------------------------------
EPI_ISL_426888    1 ------------------------------------------------------------
EPI_ISL_426900    1 ------------------------------------------------------------
EPI_ISL_426901    1 ------------------------------------------------------------
EPI_ISL_426903    1 ------------------------------------------------------------
EPI_ISL_426904    1 ------------------------------------------------------------
EPI_ISL_426907    1 ------------------------------------------------------------
EPI_ISL_426910    1 ------------------------------------------------------------
ORF1ab          181 AYTRYVDNNFCGPDGYPLECIKDLLARAGKASCTLSEQLDFIDTKRGVYCCREHEHEIAW
EPI_ISL_413575    1 ------------------------------------------------------------
EPI_ISL_413588    1 ------------------------------------------------------------
EPI_ISL_413589    1 ------------------------------------------------------------
EPI_ISL_424221    1 ------------------------------------------------------------
EPI_ISL_424233    1 ------------------------------------------------------------
EPI_ISL_424236    1 ------------------------------------------------------------
EPI_ISL_424238    1 ------------------------------------------------------------
EPI_ISL_424248    1 ------------------------------------------------------------
EPI_ISL_424254    1 ------------------------------------------------------------
EPI_ISL_425548    1 ------------------------------------------------------------
EPI_ISL_425581    1 ------------------------------------------------------------
EPI_ISL_426881    1 ------------------------------------------------------------
EPI_ISL_426883    1 ------------------------------------------------------------
EPI_ISL_426888    1 ------------------------------------------------------------
EPI_ISL_426900    1 ------------------------------------------------------------
EPI_ISL_426901    1 ------------------------------------------------------------
EPI_ISL_426903    1 ------------------------------------------------------------
EPI_ISL_426904    1 ------------------------------------------------------------
EPI_ISL_426907    1 ------------------------------------------------------------
EPI_ISL_426910    1 ------------------------------------------------------------
```

```
ORF1ab          241 YTERSEKSYELQTPFEIKLAKKFDTFNGECPNFVFPLNSIIKTIQPRVEKKKLDGFMGRI
EPI_ISL_413575    1 ------------------------------------------------------------
EPI_ISL_413588    1 ------------------------------------------------------------
EPI_ISL_413589    1 ------------------------------------------------------------
EPI_ISL_424221    1 ------------------------------------------------------------
EPI_ISL_424233    1 ------------------------------------------------------------
EPI_ISL_424236    1 ------------------------------------------------------------
EPI_ISL_424238    1 ------------------------------------------------------------
EPI_ISL_424248    1 ------------------------------------------------------------
EPI_ISL_424254    1 ------------------------------------------------------------
EPI_ISL_425548    1 ------------------------------------------------------------
EPI_ISL_425581    1 ------------------------------------------------------------
EPI_ISL_426881    1 ------------------------------------------------------------
EPI_ISL_426883    1 ------------------------------------------------------------
EPI_ISL_426888    1 ------------------------------------------------------------
EPI_ISL_426900    1 ------------------------------------------------------------
EPI_ISL_426901    1 ------------------------------------------------------------
EPI_ISL_426903    1 ------------------------------------------------------------
EPI_ISL_426904    1 ------------------------------------------------------------
EPI_ISL_426907    1 ------------------------------------------------------------
EPI_ISL_426910    1 ------------------------------------------------------------
ORF1ab          241 YTERSEKSYELQTPFEIKLAKKFDTFNGECPNFVFPLNSIIKTIQPRVEKKKLDGFMGRI
EPI_ISL_413575    1 ------------------------------------------------------------
EPI_ISL_413588    1 ------------------------------------------------------------
EPI_ISL_413589    1 ------------------------------------------------------------
EPI_ISL_424221    1 ------------------------------------------------------------
EPI_ISL_424233    1 ------------------------------------------------------------
EPI_ISL_424236    1 ------------------------------------------------------------
EPI_ISL_424238    1 ------------------------------------------------------------
EPI_ISL_424248    1 ------------------------------------------------------------
EPI_ISL_424254    1 ------------------------------------------------------------
EPI_ISL_425548    1 ------------------------------------------------------------
EPI_ISL_425581    1 ------------------------------------------------------------
EPI_ISL_426881    1 ------------------------------------------------------------
EPI_ISL_426883    1 ------------------------------------------------------------
EPI_ISL_426888    1 ------------------------------------------------------------
EPI_ISL_426900    1 ------------------------------------------------------------
EPI_ISL_426901    1 ------------------------------------------------------------
EPI_ISL_426903    1 ------------------------------------------------------------
EPI_ISL_426904    1 ------------------------------------------------------------
EPI_ISL_426907    1 ------------------------------------------------------------
EPI_ISL_426910    1 ------------------------------------------------------------
```

```
ORF1ab          301 RSVYPVASPNECNQMCLSTLMKCDHCGETSWQTGDFVKATCEFCGTENLTKEGATTCGYL
EPI_ISL_413575    1 ------------------------------------------------------------
EPI_ISL_413588    1 ------------------------------------------------------------
EPI_ISL_413589    1 ------------------------------------------------------------
EPI_ISL_424221    1 ------------------------------------------------------------
EPI_ISL_424233    1 ------------------------------------------------------------
EPI_ISL_424236    1 ------------------------------------------------------------
EPI_ISL_424238    1 ------------------------------------------------------------
EPI_ISL_424248    1 ------------------------------------------------------------
EPI_ISL_424254    1 ------------------------------------------------------------
EPI_ISL_425548    1 ------------------------------------------------------------
EPI_ISL_425581    1 ------------------------------------------------------------
EPI_ISL_426881    1 ------------------------------------------------------------
EPI_ISL_426883    1 ------------------------------------------------------------
EPI_ISL_426888    1 ------------------------------------------------------------
EPI_ISL_426900    1 ------------------------------------------------------------
EPI_ISL_426901    1 ------------------------------------------------------------
EPI_ISL_426903    1 ------------------------------------------------------------
EPI_ISL_426904    1 ------------------------------------------------------------
EPI_ISL_426907    1 ------------------------------------------------------------
EPI_ISL_426910    1 ------------------------------------------------------------
```


```
ORF1ab          301 RSVYPVASPNECNQMCLSTLMKCDHCGETSWQTGDFVKATCEFCGTENLTKEGATTCGYL
EPI_ISL_413575    1 ------------------------------------------------------------
EPI_ISL_413588    1 ------------------------------------------------------------
EPI_ISL_413589    1 ------------------------------------------------------------
EPI_ISL_424221    1 ------------------------------------------------------------
EPI_ISL_424233    1 ------------------------------------------------------------
EPI_ISL_424236    1 ------------------------------------------------------------
EPI_ISL_424238    1 ------------------------------------------------------------
EPI_ISL_424248    1 ------------------------------------------------------------
EPI_ISL_424254    1 ------------------------------------------------------------
EPI_ISL_425548    1 ------------------------------------------------------------
EPI_ISL_425581    1 ------------------------------------------------------------
EPI_ISL_426881    1 ------------------------------------------------------------
EPI_ISL_426883    1 ------------------------------------------------------------
EPI_ISL_426888    1 ------------------------------------------------------------
EPI_ISL_426900    1 ------------------------------------------------------------
EPI_ISL_426901    1 ------------------------------------------------------------
EPI_ISL_426903    1 ------------------------------------------------------------
EPI_ISL_426904    1 ------------------------------------------------------------
EPI_ISL_426907    1 ------------------------------------------------------------
EPI_ISL_426910    1 ------------------------------------------------------------
```

```
ORF1ab          361 PQNAVVKIYCPACHNSEVGPEHSLAEYHNESGLKTILRKGGRTIAFGGCVFSYVGCHNKC
EPI_ISL_413575    1 ------------------------------------------------------------
EPI_ISL_413588    1 ------------------------------------------------------------
EPI_ISL_413589    1 ------------------------------------------------------------
EPI_ISL_424221    1 ------------------------------------------------------------
EPI_ISL_424233    1 ------------------------------------------------------------
EPI_ISL_424236    1 ------------------------------------------------------------
EPI_ISL_424238    1 ------------------------------------------------------------
EPI_ISL_424248    1 ------------------------------------------------------------
EPI_ISL_424254    1 ------------------------------------------------------------
EPI_ISL_425548    1 ------------------------------------------------------------
EPI_ISL_425581    1 ------------------------------------------------------------
EPI_ISL_426881    1 ------------------------------------------------------------
EPI_ISL_426883    1 ------------------------------------------------------------
EPI_ISL_426888    1 ------------------------------------------------------------
EPI_ISL_426900    1 ------------------------------------------------------------
EPI_ISL_426901    1 ------------------------------------------------------------
EPI_ISL_426903    1 ------------------------------------------------------------
EPI_ISL_426904    1 ------------------------------------------------------------
EPI_ISL_426907    1 ------------------------------------------------------------
EPI_ISL_426910    1 ------------------------------------------------------------
```

```
ORF1ab          361 PQNAVVKIYCPACHNSEVGPEHSLAEYHNESGLKTILRKGGRTIAFGGCVFSYVGCHNKC
EPI_ISL_413575    1 ------------------------------------------------------------
EPI_ISL_413588    1 ------------------------------------------------------------
EPI_ISL_413589    1 ------------------------------------------------------------
EPI_ISL_424221    1 ------------------------------------------------------------
EPI_ISL_424233    1 ------------------------------------------------------------
EPI_ISL_424236    1 ------------------------------------------------------------
EPI_ISL_424238    1 ------------------------------------------------------------
EPI_ISL_424248    1 ------------------------------------------------------------
EPI_ISL_424254    1 ------------------------------------------------------------
EPI_ISL_425548    1 ------------------------------------------------------------
EPI_ISL_425581    1 ------------------------------------------------------------
EPI_ISL_426881    1 ------------------------------------------------------------
EPI_ISL_426883    1 ------------------------------------------------------------
EPI_ISL_426888    1 ------------------------------------------------------------
EPI_ISL_426900    1 ------------------------------------------------------------
EPI_ISL_426901    1 ------------------------------------------------------------
EPI_ISL_426903    1 ------------------------------------------------------------
EPI_ISL_426904    1 ------------------------------------------------------------
EPI_ISL_426907    1 ------------------------------------------------------------
EPI_ISL_426910    1 ------------------------------------------------------------
```

```
ORF1ab          421 AYWVPRASANIGCNHTGVVGEGSEGLNDNLLEILQKEKVNINIVGDFKLNEEIAIILASF
EPI_ISL_413575    1 ------------------------------------------------------------
EPI_ISL_413588    1 ------------------------------------------------------------
EPI_ISL_413589    1 ------------------------------------------------------------
EPI_ISL_424221    1 ------------------------------------------------------------
EPI_ISL_424233    1 ------------------------------------------------------------
EPI_ISL_424236    1 ------------------------------------------------------------
EPI_ISL_424238    1 ------------------------------------------------------------
EPI_ISL_424248    1 ------------------------------------------------------------
EPI_ISL_424254    1 ------------------------------------------------------------
EPI_ISL_425548    1 ------------------------------------------------------------
EPI_ISL_425581    1 ------------------------------------------------------------
EPI_ISL_426881    1 ------------------------------------------------------------
EPI_ISL_426883    1 ------------------------------------------------------------
EPI_ISL_426888    1 ------------------------------------------------------------
EPI_ISL_426900    1 ------------------------------------------------------------
EPI_ISL_426901    1 ------------------------------------------------------------
EPI_ISL_426903    1 ------------------------------------------------------------
EPI_ISL_426904    1 ------------------------------------------------------------
EPI_ISL_426907    1 ------------------------------------------------------------
EPI_ISL_426910    1 ------------------------------------------------------------
ORF1ab          421 AYWVPRASANIGCNHTGVVGEGSEGLNDNLLEILQKEKVNINIVGDFKLNEEIAIILASF
EPI_ISL_413575    1 ------------------------------------------------------------
EPI_ISL_413588    1 ------------------------------------------------------------
EPI_ISL_413589    1 ------------------------------------------------------------
EPI_ISL_424221    1 ------------------------------------------------------------
EPI_ISL_424233    1 ------------------------------------------------------------
EPI_ISL_424236    1 ------------------------------------------------------------
EPI_ISL_424238    1 ------------------------------------------------------------
EPI_ISL_424248    1 ------------------------------------------------------------
EPI_ISL_424254    1 ------------------------------------------------------------
EPI_ISL_425548    1 ------------------------------------------------------------
EPI_ISL_425581    1 ------------------------------------------------------------
EPI_ISL_426881    1 ------------------------------------------------------------
EPI_ISL_426883    1 ------------------------------------------------------------
EPI_ISL_426888    1 ------------------------------------------------------------
EPI_ISL_426900    1 ------------------------------------------------------------
EPI_ISL_426901    1 ------------------------------------------------------------
EPI_ISL_426903    1 ------------------------------------------------------------
EPI_ISL_426904    1 ------------------------------------------------------------
EPI_ISL_426907    1 ------------------------------------------------------------
EPI_ISL_426910    1 ------------------------------------------------------------
```

```
ORF1ab          481 SASTSAFVETVKGLDYKAFKQIVESCGNFKVTKGKAKKGAWNIGEQKSILSPLYAFASEA
EPI_ISL_413575    1 ------------------------------------------------------------
EPI_ISL_413588    1 ------------------------------------------------------------
EPI_ISL_413589    1 ------------------------------------------------------------
EPI_ISL_424221    1 ------------------------------------------------------------
EPI_ISL_424233    1 ------------------------------------------------------------
EPI_ISL_424236    1 ------------------------------------------------------------
EPI_ISL_424238    1 ------------------------------------------------------------
EPI_ISL_424248    1 ------------------------------------------------------------
EPI_ISL_424254    1 ------------------------------------------------------------
EPI_ISL_425548    1 ------------------------------------------------------------
EPI_ISL_425581    1 ------------------------------------------------------------
EPI_ISL_426881    1 ------------------------------------------------------------
EPI_ISL_426883    1 ------------------------------------------------------------
EPI_ISL_426888    1 ------------------------------------------------------------
EPI_ISL_426900    1 ------------------------------------------------------------
EPI_ISL_426901    1 ------------------------------------------------------------
EPI_ISL_426903    1 ------------------------------------------------------------
EPI_ISL_426904    1 ------------------------------------------------------------
EPI_ISL_426907    1 ------------------------------------------------------------
EPI_ISL_426910    1 ------------------------------------------------------------
```

```
ORF1ab          481 SASTSAFVETVKGLDYKAFKQIVESCGNFKVTKGKAKKGAWNIGEQKSILSPLYAFASEA
EPI_ISL_413575    1 ------------------------------------------------------------
EPI_ISL_413588    1 ------------------------------------------------------------
EPI_ISL_413589    1 ------------------------------------------------------------
EPI_ISL_424221    1 ------------------------------------------------------------
EPI_ISL_424233    1 ------------------------------------------------------------
EPI_ISL_424236    1 ------------------------------------------------------------
EPI_ISL_424238    1 ------------------------------------------------------------
EPI_ISL_424248    1 ------------------------------------------------------------
EPI_ISL_424254    1 ------------------------------------------------------------
EPI_ISL_425548    1 ------------------------------------------------------------
EPI_ISL_425581    1 ------------------------------------------------------------
EPI_ISL_426881    1 ------------------------------------------------------------
EPI_ISL_426883    1 ------------------------------------------------------------
EPI_ISL_426888    1 ------------------------------------------------------------
EPI_ISL_426900    1 ------------------------------------------------------------
EPI_ISL_426901    1 ------------------------------------------------------------
EPI_ISL_426903    1 ------------------------------------------------------------
EPI_ISL_426904    1 ------------------------------------------------------------
EPI_ISL_426907    1 ------------------------------------------------------------
EPI_ISL_426910    1 ------------------------------------------------------------
```

```
ORF1ab           541 ARVVRSIFSRTLETAQNSVRVLQKAAITILDGISQYSLRLIDAMMFTSDLATNNLVVMAY
EPI_ISL_413575     1 ------------------------------------------------------------
EPI_ISL_413588     1 ------------------------------------------------------------
EPI_ISL_413589     1 ------------------------------------------------------------
EPI_ISL_424221     1 ------------------------------------------------------------
EPI_ISL_424233     1 ------------------------------------------------------------
EPI_ISL_424236     1 ------------------------------------------------------------
EPI_ISL_424238     1 ------------------------------------------------------------
EPI_ISL_424248     1 ------------------------------------------------------------
EPI_ISL_424254     1 ------------------------------------------------------------
EPI_ISL_425548     1 ------------------------------------------------------------
EPI_ISL_425581     1 ------------------------------------------------------------
EPI_ISL_426881     1 ------------------------------------------------------------
EPI_ISL_426883     1 ------------------------------------------------------------
EPI_ISL_426888     1 ------------------------------------------------------------
EPI_ISL_426900     1 ------------------------------------------------------------
EPI_ISL_426901     1 ------------------------------------------------------------
EPI_ISL_426903     1 ------------------------------------------------------------
EPI_ISL_426904     1 ------------------------------------------------------------
EPI_ISL_426907     1 ------------------------------------------------------------
EPI_ISL_426910     1 ------------------------------------------------------------
```

```
ORF1ab           541 ARVVRSIFSRTLETAQNSVRVLQKAAITILDGISQYSLRLIDAMMFTSDLATNNLVVMAY
EPI_ISL_413575     1 ------------------------------------------------------------
EPI_ISL_413588     1 ------------------------------------------------------------
EPI_ISL_413589     1 ------------------------------------------------------------
EPI_ISL_424221     1 ------------------------------------------------------------
EPI_ISL_424233     1 ------------------------------------------------------------
EPI_ISL_424236     1 ------------------------------------------------------------
EPI_ISL_424238     1 ------------------------------------------------------------
EPI_ISL_424248     1 ------------------------------------------------------------
EPI_ISL_424254     1 ------------------------------------------------------------
EPI_ISL_425548     1 ------------------------------------------------------------
EPI_ISL_425581     1 ------------------------------------------------------------
EPI_ISL_426881     1 ------------------------------------------------------------
EPI_ISL_426883     1 ------------------------------------------------------------
EPI_ISL_426888     1 ------------------------------------------------------------
EPI_ISL_426900     1 ------------------------------------------------------------
EPI_ISL_426901     1 ------------------------------------------------------------
EPI_ISL_426903     1 ------------------------------------------------------------
EPI_ISL_426904     1 ------------------------------------------------------------
EPI_ISL_426907     1 ------------------------------------------------------------
EPI_ISL_426910     1 ------------------------------------------------------------
```

```
ORF1ab          601 ITGGVVQLTSQWLTNIFGTVYEKLKPVLDWLEEKFKEGVEFLRDGWEIVKFISTCACEIV
EPI_ISL_413575    1 ------------------------------------------------------------
EPI_ISL_413588    1 ------------------------------------------------------------
EPI_ISL_413589    1 ------------------------------------------------------------
EPI_ISL_424221    1 ------------------------------------------------------------
EPI_ISL_424233    1 ------------------------------------------------------------
EPI_ISL_424236    1 ------------------------------------------------------------
EPI_ISL_424238    1 ------------------------------------------------------------
EPI_ISL_424248    1 ------------------------------------------------------------
EPI_ISL_424254    1 ------------------------------------------------------------
EPI_ISL_425548    1 ------------------------------------------------------------
EPI_ISL_425581    1 ------------------------------------------------------------
EPI_ISL_426881    1 ------------------------------------------------------------
EPI_ISL_426883    1 ------------------------------------------------------------
EPI_ISL_426888    1 ------------------------------------------------------------
EPI_ISL_426900    1 ------------------------------------------------------------
EPI_ISL_426901    1 ------------------------------------------------------------
EPI_ISL_426903    1 ------------------------------------------------------------
EPI_ISL_426904    1 ------------------------------------------------------------
EPI_ISL_426907    1 ------------------------------------------------------------
EPI_ISL_426910    1 ------------------------------------------------------------
ORF1ab          601 ITGGVVQLTSQWLTNIFGTVYEKLKPVLDWLEEKFKEGVEFLRDGWEIVKFISTCACEIV
EPI_ISL_413575    1 ------------------------------------------------------------
EPI_ISL_413588    1 ------------------------------------------------------------
EPI_ISL_413589    1 ------------------------------------------------------------
EPI_ISL_424221    1 ------------------------------------------------------------
EPI_ISL_424233    1 ------------------------------------------------------------
EPI_ISL_424236    1 ------------------------------------------------------------
EPI_ISL_424238    1 ------------------------------------------------------------
EPI_ISL_424248    1 ------------------------------------------------------------
EPI_ISL_424254    1 ------------------------------------------------------------
EPI_ISL_425548    1 ------------------------------------------------------------
EPI_ISL_425581    1 ------------------------------------------------------------
EPI_ISL_426881    1 ------------------------------------------------------------
EPI_ISL_426883    1 ------------------------------------------------------------
EPI_ISL_426888    1 ------------------------------------------------------------
EPI_ISL_426900    1 ------------------------------------------------------------
EPI_ISL_426901    1 ------------------------------------------------------------
EPI_ISL_426903    1 ------------------------------------------------------------
EPI_ISL_426904    1 ------------------------------------------------------------
EPI_ISL_426907    1 ------------------------------------------------------------
EPI_ISL_426910    1 ------------------------------------------------------------
```

```
ORF1ab           661 GGQIVTCAKEIKESVQTFFKLVNKFLALCADSIIIGGAKLKALNLGETFVTHSKGLYRKC
EPI_ISL_413575     1 ------------------------------------------------------------
EPI_ISL_413588     1 ------------------------------------------------------------
EPI_ISL_413589     1 ------------------------------------------------------------
EPI_ISL_424221     1 ------------------------------------------------------------
EPI_ISL_424233     1 ------------------------------------------------------------
EPI_ISL_424236     1 ------------------------------------------------------------
EPI_ISL_424238     1 ------------------------------------------------------------
EPI_ISL_424248     1 ------------------------------------------------------------
EPI_ISL_424254     1 ------------------------------------------------------------
EPI_ISL_425548     1 ------------------------------------------------------------
EPI_ISL_425581     1 ------------------------------------------------------------
EPI_ISL_426881     1 ------------------------------------------------------------
EPI_ISL_426883     1 ------------------------------------------------------------
EPI_ISL_426888     1 ------------------------------------------------------------
EPI_ISL_426900     1 ------------------------------------------------------------
EPI_ISL_426901     1 ------------------------------------------------------------
EPI_ISL_426903     1 ------------------------------------------------------------
EPI_ISL_426904     1 ------------------------------------------------------------
EPI_ISL_426907     1 ------------------------------------------------------------
EPI_ISL_426910     1 ------------------------------------------------------------
```

```
ORF1ab           661 GGQIVTCAKEIKESVQTFFKLVNKFLALCADSIIIGGAKLKALNLGETFVTHSKGLYRKC
EPI_ISL_413575     1 ------------------------------------------------------------
EPI_ISL_413588     1 ------------------------------------------------------------
EPI_ISL_413589     1 ------------------------------------------------------------
EPI_ISL_424221     1 ------------------------------------------------------------
EPI_ISL_424233     1 ------------------------------------------------------------
EPI_ISL_424236     1 ------------------------------------------------------------
EPI_ISL_424238     1 ------------------------------------------------------------
EPI_ISL_424248     1 ------------------------------------------------------------
EPI_ISL_424254     1 ------------------------------------------------------------
EPI_ISL_425548     1 ------------------------------------------------------------
EPI_ISL_425581     1 ------------------------------------------------------------
EPI_ISL_426881     1 ------------------------------------------------------------
EPI_ISL_426883     1 ------------------------------------------------------------
EPI_ISL_426888     1 ------------------------------------------------------------
EPI_ISL_426900     1 ------------------------------------------------------------
EPI_ISL_426901     1 ------------------------------------------------------------
EPI_ISL_426903     1 ------------------------------------------------------------
EPI_ISL_426904     1 ------------------------------------------------------------
EPI_ISL_426907     1 ------------------------------------------------------------
EPI_ISL_426910     1 ------------------------------------------------------------
```

```
ORF1ab           721 VKSREETGLLMPLKAPKEIIFLEGETLPTEVLTEEVVLKTGDLQPLEQPTSEAVEAPLVG
EPI_ISL_413575     1 ------------------------------------------------------------
EPI_ISL_413588     1 ------------------------------------------------------------
EPI_ISL_413589     1 ------------------------------------------------------------
EPI_ISL_424221     1 ------------------------------------------------------------
EPI_ISL_424233     1 ------------------------------------------------------------
EPI_ISL_424236     1 ------------------------------------------------------------
EPI_ISL_424238     1 ------------------------------------------------------------
EPI_ISL_424248     1 ------------------------------------------------------------
EPI_ISL_424254     1 ------------------------------------------------------------
EPI_ISL_425548     1 ------------------------------------------------------------
EPI_ISL_425581     1 ------------------------------------------------------------
EPI_ISL_426881     1 ------------------------------------------------------------
EPI_ISL_426883     1 ------------------------------------------------------------
EPI_ISL_426888     1 ------------------------------------------------------------
EPI_ISL_426900     1 ------------------------------------------------------------
EPI_ISL_426901     1 ------------------------------------------------------------
EPI_ISL_426903     1 ------------------------------------------------------------
EPI_ISL_426904     1 ------------------------------------------------------------
EPI_ISL_426907     1 ------------------------------------------------------------
EPI_ISL_426910     1 ------------------------------------------------------------
```


```
ORF1ab           721 VKSREETGLLMPLKAPKEIIFLEGETLPTEVLTEEVVLKTGDLQPLEQPTSEAVEAPLVG
EPI_ISL_413575     1 ------------------------------------------------------------
EPI_ISL_413588     1 ------------------------------------------------------------
EPI_ISL_413589     1 ------------------------------------------------------------
EPI_ISL_424221     1 ------------------------------------------------------------
EPI_ISL_424233     1 ------------------------------------------------------------
EPI_ISL_424236     1 ------------------------------------------------------------
EPI_ISL_424238     1 ------------------------------------------------------------
EPI_ISL_424248     1 ------------------------------------------------------------
EPI_ISL_424254     1 ------------------------------------------------------------
EPI_ISL_425548     1 ------------------------------------------------------------
EPI_ISL_425581     1 ------------------------------------------------------------
EPI_ISL_426881     1 ------------------------------------------------------------
EPI_ISL_426883     1 ------------------------------------------------------------
EPI_ISL_426888     1 ------------------------------------------------------------
EPI_ISL_426900     1 ------------------------------------------------------------
EPI_ISL_426901     1 ------------------------------------------------------------
EPI_ISL_426903     1 ------------------------------------------------------------
EPI_ISL_426904     1 ------------------------------------------------------------
EPI_ISL_426907     1 ------------------------------------------------------------
EPI_ISL_426910     1 ------------------------------------------------------------
```

```
ORF1ab          781 TPVCINGLMLLEIKDTEKYCALAPNMMVTNNTFTLKGGAPTKVTFGDDTVIEVQGYKSVN
EPI_ISL_413575    1 --------------------------------------APTKVTFGDDTVIEVQGYKSVN
EPI_ISL_413588    1 --------------------------------------APTKVTFGDDTVIEVQGYKSVN
EPI_ISL_413589    1 --------------------------------------APTKVTFGDDTVIEVQGYKSVN
EPI_ISL_424221    1 --------------------------------------APTKVTFGDDTVIEVQGYKSVN
EPI_ISL_424233    1 --------------------------------------APTKVTFGDDTVIEVQGYKSVN
EPI_ISL_424236    1 --------------------------------------APTKVTFGDDTVIEVQGYKSVN
EPI_ISL_424238    1 --------------------------------------APTKVTFGDDTVIEVQGYKSVN
EPI_ISL_424248    1 --------------------------------------APTKVTFGDDTVIEVQGYKSVN
EPI_ISL_424254    1 --------------------------------------APTKVTFGDDTVIEVQGYKSVN
EPI_ISL_425548    1 --------------------------------------APTKVTFGDDTVIEVQGYKSVN
EPI_ISL_425581    1 --------------------------------------APTKVTFGDDTVIEVQGYKSVN
EPI_ISL_426881    1 --------------------------------------APTKVTFGDDTVIEVQGYKSVN
EPI_ISL_426883    1 --------------------------------------APTKVTFGDDTVIEVQGYKSVN
EPI_ISL_426888    1 --------------------------------------APTKVTFGDDTVIEVQGYKSVN
EPI_ISL_426900    1 --------------------------------------APTKVTFGDDTVIEVQGYKSVN
EPI_ISL_426901    1 --------------------------------------APTKVTFGDDTVIEVQGYKSVN
EPI_ISL_426903    1 --------------------------------------APTKVTFGDDTVIEVQGYKSVN
EPI_ISL_426904    1 --------------------------------------APTKVTFGDDTVIEVQGYKSVN
EPI_ISL_426907    1 --------------------------------------APTKVTFGDDTVIEVQGYKSVN
EPI_ISL_426910    1 --------------------------------------APTKVTFGDDTVIEVQGYKSVN
ORF1ab          781 TPVCINGLMLLEIKDTEKYCALAPNMMVTNNTFTLKGGAPTKVTFGDDTVIEVQGYKSVN
EPI_ISL_413575    1 --------------------------------------APTKVTFGDDTVIEVQGYKSVN
EPI_ISL_413588    1 --------------------------------------APTKVTFGDDTVIEVQGYKSVN
EPI_ISL_413589    1 --------------------------------------APTKVTFGDDTVIEVQGYKSVN
EPI_ISL_424221    1 --------------------------------------APTKVTFGDDTVIEVQGYKSVN
EPI_ISL_424233    1 --------------------------------------APTKVTFGDDTVIEVQGYKSVN
EPI_ISL_424236    1 --------------------------------------APTKVTFGDDTVIEVQGYKSVN
EPI_ISL_424238    1 --------------------------------------APTKVTFGDDTVIEVQGYKSVN
EPI_ISL_424248    1 --------------------------------------APTKVTFGDDTVIEVQGYKSVN
EPI_ISL_424254    1 --------------------------------------APTKVTFGDDTVIEVQGYKSVN
EPI_ISL_425548    1 --------------------------------------APTKVTFGDDTVIEVQGYKSVN
EPI_ISL_425581    1 --------------------------------------APTKVTFGDDTVIEVQGYKSVN
EPI_ISL_426881    1 --------------------------------------APTKVTFGDDTVIEVQGYKSVN
EPI_ISL_426883    1 --------------------------------------APTKVTFGDDTVIEVQGYKSVN
EPI_ISL_426888    1 --------------------------------------APTKVTFGDDTVIEVQGYKSVN
EPI_ISL_426900    1 --------------------------------------APTKVTFGDDTVIEVQGYKSVN
EPI_ISL_426901    1 --------------------------------------APTKVTFGDDTVIEVQGYKSVN
EPI_ISL_426903    1 --------------------------------------APTKVTFGDDTVIEVQGYKSVN
EPI_ISL_426904    1 --------------------------------------APTKVTFGDDTVIEVQGYKSVN
EPI_ISL_426907    1 --------------------------------------APTKVTFGDDTVIEVQGYKSVN
EPI_ISL_426910    1 --------------------------------------APTKVTFGDDTVIEVQGYKSVN
```

ORF1ab	841	ITFELDERIDKVLNEKCSAYTVELGTEVNEFACVVADAVIKTLQPVSELLTPLGIDLDEW
EPI_ISL_413575	23	ITFELDERIDKVLNEKCSAYTVELGTEVNEFACVVADAVIKTLQPVSELLTPLGIDLDEW
EPI_ISL_413588	23	ITFELDERIDKVLNEKCSAYTVELGTEVNEFACVVADAVIKTLQPVSELLTPLGIDLDEW
EPI_ISL_413589	23	ITFELDERIDKVLNEKCSAYTVELGTEVNEFACVVADAVIKTLQPVSELLTPLGIDLDEW
EPI_ISL_424221	23	ITFELDERIDKVLNEKCSAYTVELGTEVNEFACVVADAVIKTLQPVSELLTPLGIDLDEW
EPI_ISL_424233	23	ITFELDERIDKVLNEKCSAYTVELGTEVNEFACVVADAVIKTLQPVSELLTPLGIDLDEW
EPI_ISL_424236	23	ITFELDERIDKVLNEKCSAYTVELGTEVNEFACVVADAVIKTLQPVSELLTPLGIDLDEW
EPI_ISL_424238	23	ITFELDERIDKVLNEKCSAYTVELGTEVNEFACVVADAVIKTLQPVSELLTPLGIDLDEW
EPI_ISL_424248	23	ITFELDERIDKVLNEKCSAYTVELGTEVNEFACVVADAVIKTLQPVSELLTPLGIDLDEW
EPI_ISL_424254	23	ITFELDERIDKVLNEKCSAYTVELGTEVNEFACVVADAVIKTLQPVSELLTPLGIDLDEW
EPI_ISL_425548	23	ITFELDERIDKVLNEKCSAYTVELGTEVNEFACVVADAVIKTLQPVSELLTPLGIDLDEW
EPI_ISL_425581	23	ITFELDERIDKVLNEKCSAYTVELGTEVNEFACVVADAVIKTLQPVSELLTPLGIDLDEW
EPI_ISL_426881	23	ITFELDERIDKVLNEKCSAYTVELGTEVNEFACVVADAVIKTLQPVSELLTPLGIDLDEW
EPI_ISL_426883	23	ITFELDERIDKVLNEKCSAYTVELGTEVNEFACVVADAVIKTLQPVSELLTPLGIDLDEW
EPI_ISL_426888	23	ITFELDERIDKVLNEKCSAYTVELGTEVNEFACVVADAVIKTLQPVSELLTPLGIDLDEW
EPI_ISL_426900	23	ITFELDERIDKVLNEKCSAYTVELGTEVNEFACVVADAVIKTLQPVSELLTPLGIDLDEW
EPI_ISL_426901	23	ITFELDERIDKVLNEKCSAYTVELGTEVNEFACVVADAVIKTLQPVSELLTPLGIDLDEW
EPI_ISL_426903	23	ITFELDERIDKVLNEKCSAYTVELGTEVNEFACVVADAVIKTLQPVSELLTPLGIDLDEW
EPI_ISL_426904	23	ITFELDERIDKVLNEKCSAYTVELGTEVNEFACVVADAVIKTLQPVSELLTPLGIDLDEW
EPI_ISL_426907	23	ITFELDERIDKVLNEKCSAYTVELGTEVNEFACVVADAVIKTLQPVSELLTPLGIDLDEW
EPI_ISL_426910	23	ITFELDERIDKVLNEKCSAYTVELGTEVNEFACVVADAVIKTLQPVSELLTPLGIDLDEW
ORF1ab	841	ITFELDERIDKVLNEKCSAYTVELGTEVNEFACVVADAVIKTLQPVSELLTPLGIDLDEW
EPI_ISL_413575	23	ITFELDERIDKVLNEKCSAYTVELGTEVNEFACVVADAVIKTLQPVSELLTPLGIDLDEW
EPI_ISL_413588	23	ITFELDERIDKVLNEKCSAYTVELGTEVNEFACVVADAVIKTLQPVSELLTPLGIDLDEW
EPI_ISL_413589	23	ITFELDERIDKVLNEKCSAYTVELGTEVNEFACVVADAVIKTLQPVSELLTPLGIDLDEW
EPI_ISL_424221	23	ITFELDERIDKVLNEKCSAYTVELGTEVNEFACVVADAVIKTLQPVSELLTPLGIDLDEW
EPI_ISL_424233	23	ITFELDERIDKVLNEKCSAYTVELGTEVNEFACVVADAVIKTLQPVSELLTPLGIDLDEW
EPI_ISL_424236	23	ITFELDERIDKVLNEKCSAYTVELGTEVNEFACVVADAVIKTLQPVSELLTPLGIDLDEW
EPI_ISL_424238	23	ITFELDERIDKVLNEKCSAYTVELGTEVNEFACVVADAVIKTLQPVSELLTPLGIDLDEW
EPI_ISL_424248	23	ITFELDERIDKVLNEKCSAYTVELGTEVNEFACVVADAVIKTLQPVSELLTPLGIDLDEW
EPI_ISL_424254	23	ITFELDERIDKVLNEKCSAYTVELGTEVNEFACVVADAVIKTLQPVSELLTPLGIDLDEW
EPI_ISL_425548	23	ITFELDERIDKVLNEKCSAYTVELGTEVNEFACVVADAVIKTLQPVSELLTPLGIDLDEW
EPI_ISL_425581	23	ITFELDERIDKVLNEKCSAYTVELGTEVNEFACVVADAVIKTLQPVSELLTPLGIDLDEW
EPI_ISL_426881	23	ITFELDERIDKVLNEKCSAYTVELGTEVNEFACVVADAVIKTLQPVSELLTPLGIDLDEW
EPI_ISL_426883	23	ITFELDERIDKVLNEKCSAYTVELGTEVNEFACVVADAVIKTLQPVSELLTPLGIDLDEW
EPI_ISL_426888	23	ITFELDERIDKVLNEKCSAYTVELGTEVNEFACVVADAVIKTLQPVSELLTPLGIDLDEW
EPI_ISL_426900	23	ITFELDERIDKVLNEKCSAYTVELGTEVNEFACVVADAVIKTLQPVSELLTPLGIDLDEW
EPI_ISL_426901	23	ITFELDERIDKVLNEKCSAYTVELGTEVNEFACVVADAVIKTLQPVSELLTPLGIDLDEW
EPI_ISL_426903	23	ITFELDERIDKVLNEKCSAYTVELGTEVNEFACVVADAVIKTLQPVSELLTPLGIDLDEW
EPI_ISL_426904	23	ITFELDERIDKVLNEKCSAYTVELGTEVNEFACVVADAVIKTLQPVSELLTPLGIDLDEW
EPI_ISL_426907	23	ITFELDERIDKVLNEKCSAYTVELGTEVNEFACVVADAVIKTLQPVSELLTPLGIDLDEW
EPI_ISL_426910	23	ITFELDERIDKVLNEKCSAYTVELGTEVNEFACVVADAVIKTLQPVSELLTPLGIDLDEW

```
ORF1ab           901 SMATYYLFDESGEFKLASHMYCSFYPPDEDEEEGDCEEEEFEPSTQYEYGTEDDYQGKPL
EPI_ISL_413575    83 SMATYYLFDESGEFKLASHMYCSFYPPDEDEEEGDCEEEEFEPSTQYEYGTEDDYQGKPL
EPI_ISL_413588    83 SMATYYLFDESGEFKLASHMYCSFYPPDEDEEEGDCEEEEFEPSTQYEYGTEDDYQGKPL
EPI_ISL_413589    83 SMATYYLFDESGEFKLASHMYCSFYPPDEDEEEGDCEEEEFEPSTQYEYGTEDDYQGKPL
EPI_ISL_424221    83 SMATYYLFDESGEFKLASHMYCSFYPPDEDEEEGDCEEEEFEPSTQYEYGTEDDYQGKPL
EPI_ISL_424233    83 SMATYYLFDESGEFKLASHMYCSFYPPDEDEEEGDCEEEEFEPSTQYEYGTEDDYQGKPL
EPI_ISL_424236    83 SMATYYLFDESGEFKLASHMYCSFYPPDEDEEEGDCEEEEFEPSTQYEYGTEDDYQGKPL
EPI_ISL_424238    83 SMATYYLFDESGEFKLASHMYCSFYPPDEDEEEGDCEEEEFEPSTQYEYGTEDDYQGKPL
EPI_ISL_424248    83 SMATYYLFDESGEFKLASHMYCSFYPPDEDEEEGDCEEEEFEPSTQYEYGTEDDYQGKPL
EPI_ISL_424254    83 SMATYYLFDESGEFKLASHMYCSFYPPDEDEEEGDCEEEEFEPSTQYEYGTEDDYQGKPL
EPI_ISL_425548    83 SMATYYLFDESGEFKLASHMYCSFYPPDEDEEEGDCEEEEFEPSTQYEYGTEDDYQGKPL
EPI_ISL_425581    83 SMATYYLFDESGEFKLASHMYCSFYPPDEDEEEGDCEEEEFEPSTQYEYGTEDDYQGKPL
EPI_ISL_426881    83 SMATYYLFDESGEFKLASHMYCSFYPPDEDEEEGDCEEEEFEPSTQYEYGTEDDYQGKPL
EPI_ISL_426883    83 SMATYYLFDESGEFKLASHMYCSFYPPDEDEEEGDCEEEEFEPSTQYEYGTEDDYQGKPL
EPI_ISL_426888    83 SMATYYLFDESGEFKLASHMYCSFYPPDEDEEEGDCEEEEFEPSTQYEYGTEDDYQGKPL
EPI_ISL_426900    83 SMATYYLFDESGEFKLASHMYCSFYPPDEDEEEGDCEEEEFEPSTQYEYGTEDDYQGKPL
EPI_ISL_426901    83 SMATYYLFDESGEFKLASHMYCSFYPPDEDEEEGDCEEEEFEPSTQYEYGTEDDYQGKPL
EPI_ISL_426903    83 SMATYYLFDESGEFKLASHMYCSFYPPDEDEEEGDCEEEEFEPSTQYEYGTEDDYQGKPL
EPI_ISL_426904    83 SMATYYLFDESGEFKLASHMYCSFYPPDEDEEEGDCEEEEFEPSTQYEYGTEDDYQGKPL
EPI_ISL_426907    83 SMATYYLFDESGEFKLASHMYCSFYPPDEDEEEGDCEEEEFEPSTQYEYGTEDDYQGKPL
EPI_ISL_426910    83 SMATYYLFDESGEFKLASHMYCSFYPPDEDEEEGDCEEEEFEPSTQYEYGTEDDYQGKPL
```


```
ORF1ab           901 SMATYYLFDESGEFKLASHMYCSFYPPDEDEEEGDCEEEEFEPSTQYEYGTEDDYQGKPL
EPI_ISL_413575    83 SMATYYLFDESGEFKLASHMYCSFYPPDEDEEEGDCEEEEFEPSTQYEYGTEDDYQGKPL
EPI_ISL_413588    83 SMATYYLFDESGEFKLASHMYCSFYPPDEDEEEGDCEEEEFEPSTQYEYGTEDDYQGKPL
EPI_ISL_413589    83 SMATYYLFDESGEFKLASHMYCSFYPPDEDEEEGDCEEEEFEPSTQYEYGTEDDYQGKPL
EPI_ISL_424221    83 SMATYYLFDESGEFKLASHMYCSFYPPDEDEEEGDCEEEEFEPSTQYEYGTEDDYQGKPL
EPI_ISL_424233    83 SMATYYLFDESGEFKLASHMYCSFYPPDEDEEEGDCEEEEFEPSTQYEYGTEDDYQGKPL
EPI_ISL_424236    83 SMATYYLFDESGEFKLASHMYCSFYPPDEDEEEGDCEEEEFEPSTQYEYGTEDDYQGKPL
EPI_ISL_424238    83 SMATYYLFDESGEFKLASHMYCSFYPPDEDEEEGDCEEEEFEPSTQYEYGTEDDYQGKPL
EPI_ISL_424248    83 SMATYYLFDESGEFKLASHMYCSFYPPDEDEEEGDCEEEEFEPSTQYEYGTEDDYQGKPL
EPI_ISL_424254    83 SMATYYLFDESGEFKLASHMYCSFYPPDEDEEEGDCEEEEFEPSTQYEYGTEDDYQGKPL
EPI_ISL_425548    83 SMATYYLFDESGEFKLASHMYCSFYPPDEDEEEGDCEEEEFEPSTQYEYGTEDDYQGKPL
EPI_ISL_425581    83 SMATYYLFDESGEFKLASHMYCSFYPPDEDEEEGDCEEEEFEPSTQYEYGTEDDYQGKPL
EPI_ISL_426881    83 SMATYYLFDESGEFKLASHMYCSFYPPDEDEEEGDCEEEEFEPSTQYEYGTEDDYQGKPL
EPI_ISL_426883    83 SMATYYLFDESGEFKLASHMYCSFYPPDEDEEEGDCEEEEFEPSTQYEYGTEDDYQGKPL
EPI_ISL_426888    83 SMATYYLFDESGEFKLASHMYCSFYPPDEDEEEGDCEEEEFEPSTQYEYGTEDDYQGKPL
EPI_ISL_426900    83 SMATYYLFDESGEFKLASHMYCSFYPPDEDEEEGDCEEEEFEPSTQYEYGTEDDYQGKPL
EPI_ISL_426901    83 SMATYYLFDESGEFKLASHMYCSFYPPDEDEEEGDCEEEEFEPSTQYEYGTEDDYQGKPL
EPI_ISL_426903    83 SMATYYLFDESGEFKLASHMYCSFYPPDEDEEEGDCEEEEFEPSTQYEYGTEDDYQGKPL
EPI_ISL_426904    83 SMATYYLFDESGEFKLASHMYCSFYPPDEDEEEGDCEEEEFEPSTQYEYGTEDDYQGKPL
EPI_ISL_426907    83 SMATYYLFDESGEFKLASHMYCSFYPPDEDEEEGDCEEEEFEPSTQYEYGTEDDYQGKPL
EPI_ISL_426910    83 SMATYYLFDESGEFKLASHMYCSFYPPDEDEEEGDCEEEEFEPSTQYEYGTEDDYQGKPL
```

```
ORF1ab          961 EFGATSAALQPEEEQEEDWLDDDSQQTVGQQDGSEDNQTTTIQTIVEVQPQLEMELTPVV
EPI_ISL_413575  143 EFGATSAALQHEEEQEEDWLDDDSQQTVGQQDGSEDNQTTTIQTIVEVQPQLEMELTPVV
EPI_ISL_413588  143 EFGATSAALQHEEEQEEDWLDDDSQQTVGQQDGSEDNQTTTIQTIVEVQPQLEMELTPVV
EPI_ISL_413589  143 EFGATSAALQPEEEQEEDWLDDDSQQTVGQQDGSEDNQTTTIQTIVEVQPQLEMELTPVV
EPI_ISL_424221  143 EFGATSAALQPEEEQEEDWLDDDSQQTVGQQDGSEDNQTTTIQTIVEVQPQLEMELTPVV
EPI_ISL_424233  143 EFGATSAALQPEEEQEEDWLDDDSQQTVGQQDGSEDNQTTTIQTIVEVQPQLEMELTPVV
EPI_ISL_424236  143 EFGATSAALQPEEEQEEDWLDDDSQQTVGQQDGSEDNQTTTIQTIVEVQPQLEMELTPVV
EPI_ISL_424238  143 EFGATSAALQPEEEQEEDWLDDDSQQTVGQQDGSEDNQTTTIQTIVEVQPQLEMELTPVV
EPI_ISL_424248  143 EFGATSAALQPEEEQEEDWLDDDSQQTVGQQDGSEDNQTTTIQTIVEVQPQLEMELTPVV
EPI_ISL_424254  143 EFGATSAALQPEEEQEEDWLDDDSQQTVGQQDGSEDNQTTTIQTIVEVQPQLEMELTPVV
EPI_ISL_425548  143 EFGATSAALQPEEEQEEDWLDDDSQQTVGQQDGSEDNQTTTIQTIVEVQPQLEMELTPVV
EPI_ISL_425581  143 EFGATSAALQPEEEQEEDWLDDDSQQTVGQQDGSEDNQTTTIQTIVEVQPQLEMELTPVV
EPI_ISL_426881  143 EFGATSAALQPEEEQEEDWLDDDSQQTVGQQDGSEDNQTTTIQTIVEVQPQLEMELTPVV
EPI_ISL_426883  143 EFGATSAALQPEEEQEEDWLDDDSQQTVGQQDGSEDNQTTTIQTIVEVQPQLEMELTPVV
EPI_ISL_426888  143 EFGATSAALQPEEEQEEDWLDDDSQQTVGQQDGSEDNQTTTIQTIVEVQPQLEMELTPVV
EPI_ISL_426900  143 EFGATSAALQPEEEQEEDWLDDDSQQTVGQQDGSEDNQTTTIQTIVEVQPQLEMELTPVV
EPI_ISL_426901  143 EFGATSAALQPEEEQEEDWLDDDSQQTVGQQDGSEDNQTTTIQTIVEVQPQLEMELTPVV
EPI_ISL_426903  143 EFGATSAALQPEEEQEEDWLDDDSQQTVGQQDGSEDNQTTTIQTIVEVQPQLEMELTPVV
EPI_ISL_426904  143 EFGATSAALQPEEEQEEDWLDDDSQQTVGQQDGSEDNQTTTIQTIVEVQPQLEMELTPVV
EPI_ISL_426907  143 EFGATSAALQPEEEQEEDWLDDDSQQTVGQQDGSEDNQTTTIQTIVEVQPQLEMELTPVV
EPI_ISL_426910  143 EFGATSAALQPEEEQEEDWLDDDSQQTVGQQDGSEDNQTTTIQTIVEVQPQLEMELTPVV
ORF1ab          961 EFGATSAALQPEEEQEEDWLDDDSQQTVGQQDGSEDNQTTTIQTIVEVQPQLEMELTPVV
EPI_ISL_413575  143 EFGATSAALQHEEEQEEDWLDDDSQQTVGQQDGSEDNQTTTIQTIVEVQPQLEMELTPVV
EPI_ISL_413588  143 EFGATSAALQHEEEQEEDWLDDDSQQTVGQQDGSEDNQTTTIQTIVEVQPQLEMELTPVV
EPI_ISL_413589  143 EFGATSAALQPEEEQEEDWLDDDSQQTVGQQDGSEDNQTTTIQTIVEVQPQLEMELTPVV
EPI_ISL_424221  143 EFGATSAALQPEEEQEEDWLDDDSQQTVGQQDGSEDNQTTTIQTIVEVQPQLEMELTPVV
EPI_ISL_424233  143 EFGATSAALQPEEEQEEDWLDDDSQQTVGQQDGSEDNQTTTIQTIVEVQPQLEMELTPVV
EPI_ISL_424236  143 EFGATSAALQPEEEQEEDWLDDDSQQTVGQQDGSEDNQTTTIQTIVEVQPQLEMELTPVV
EPI_ISL_424238  143 EFGATSAALQPEEEQEEDWLDDDSQQTVGQQDGSEDNQTTTIQTIVEVQPQLEMELTPVV
EPI_ISL_424248  143 EFGATSAALQPEEEQEEDWLDDDSQQTVGQQDGSEDNQTTTIQTIVEVQPQLEMELTPVV
EPI_ISL_424254  143 EFGATSAALQPEEEQEEDWLDDDSQQTVGQQDGSEDNQTTTIQTIVEVQPQLEMELTPVV
EPI_ISL_425548  143 EFGATSAALQPEEEQEEDWLDDDSQQTVGQQDGSEDNQTTTIQTIVEVQPQLEMELTPVV
EPI_ISL_425581  143 EFGATSAALQPEEEQEEDWLDDDSQQTVGQQDGSEDNQTTTIQTIVEVQPQLEMELTPVV
EPI_ISL_426881  143 EFGATSAALQPEEEQEEDWLDDDSQQTVGQQDGSEDNQTTTIQTIVEVQPQLEMELTPVV
EPI_ISL_426883  143 EFGATSAALQPEEEQEEDWLDDDSQQTVGQQDGSEDNQTTTIQTIVEVQPQLEMELTPVV
EPI_ISL_426888  143 EFGATSAALQPEEEQEEDWLDDDSQQTVGQQDGSEDNQTTTIQTIVEVQPQLEMELTPVV
EPI_ISL_426900  143 EFGATSAALQPEEEQEEDWLDDDSQQTVGQQDGSEDNQTTTIQTIVEVQPQLEMELTPVV
EPI_ISL_426901  143 EFGATSAALQPEEEQEEDWLDDDSQQTVGQQDGSEDNQTTTIQTIVEVQPQLEMELTPVV
EPI_ISL_426903  143 EFGATSAALQPEEEQEEDWLDDDSQQTVGQQDGSEDNQTTTIQTIVEVQPQLEMELTPVV
EPI_ISL_426904  143 EFGATSAALQPEEEQEEDWLDDDSQQTVGQQDGSEDNQTTTIQTIVEVQPQLEMELTPVV
EPI_ISL_426907  143 EFGATSAALQPEEEQEEDWLDDDSQQTVGQQDGSEDNQTTTIQTIVEVQPQLEMELTPVV
EPI_ISL_426910  143 EFGATSAALQPEEEQEEDWLDDDSQQTVGQQDGSEDNQTTTIQTIVEVQPQLEMELTPVV
```

```
ORF1ab         1021 QTIEVNSFSGYLKLTDNVYIKNADIVEEAKKVKPTVVVNAANVYLKHGGGVAGALNKATN
EPI_ISL_413575  203 QTIEVNSFSGYLKLTDNVYIKNADIVEEAKKVKPTVVVNAANVYLKHGGGVAGALNKATN
EPI_ISL_413588  203 QTIEVNSFSGYLKLTDNVYIKNADIVEEAKKVKPTVVVNAANVYLKHGGGVAGALNKATN
EPI_ISL_413589  203 QTIEVNSFSGYLKLTDNVYIKNADIVEEAKKVKPTVVVNAANVYLKHGGGVAGALNKATN
EPI_ISL_424221  203 QTIEVNSFSGYLKLTDNVYIKNADIVEEAKKVKPTVVVNAANVYLKHGGGVAGALNKATN
EPI_ISL_424233  203 QTIEVNSFSGYLKLTDNVYIKNADIVEEAKKVKPTVVVNAANVYLKHGGGVAGALNKATN
EPI_ISL_424236  203 QTIEVNSFSGYLKLTDNVYIKNADIVEEAKKVKPTVVVNAANVYLKHGGGVAGALNKATN
EPI_ISL_424238  203 QTIEVNSFSGYLKLTDNVYIKNADIVEEAKKVKPTVVVNAANVYLKHGGGVAGALNKATN
EPI_ISL_424248  203 QTIEVNSFSGYLKLTDNVYIKNADIVEEAKKVKPTVVVNAANVYLKHGGGVAGALNKATN
EPI_ISL_424254  203 QTIEVNSFSGYLKLTDNVYIKNADIVEEAKKVKPTVVVNAANVYLKHGGGVAGALNKATN
EPI_ISL_425548  203 QTIEVNSFSGYLKLTDNVYIKNADIVEEAKKVKPTVVVNAANVYLKHGGGVAGALNKATN
EPI_ISL_425581  203 QTIEVNSFSGYLKLTDNVYIKNADIVEEAKKVKPTVVVNAANVYLKHGGGVAGALNKATN
EPI_ISL_426881  203 QTIEVNSFSGYLKLTDNVYIKNADIVEEAKKVKPTVVVNAANVYLKHGGGVAGALNKATN
EPI_ISL_426883  203 QTIEVNSFSGYLKLTDNVYIKNADIVEEAKKVKPTVVVNAANVYLKHGGGVAGALNKATN
EPI_ISL_426888  203 QTIEVNSFSGYLKLTDNVYIKNADIVEEAKKVKPTVVVNAANVYLKHGGGVAGALNKATN
EPI_ISL_426900  203 QTIEVNSFSGYLKLTDNVYIKNADIVEEAKKVKPTVVVNAANVYLKHGGGVAGALNKATN
EPI_ISL_426901  203 QTIEVNSFSGYLKLTDNVYIKNADIVEEAKKVKPTVVVNAANVYLKHGGGVAGALNKATN
EPI_ISL_426903  203 QTIEVNSFSGYLKLTDNVYIKNADIVEEAKKVKPTVVVNAANVYLKHGGGVAGALNKATN
EPI_ISL_426904  203 QTIEVNSFSGYLKLTDNVYIKNADIVEEAKKVKPTVVVNAANVYLKHGGGVAGALNKATN
EPI_ISL_426907  203 QTIEVNSFSGYLKLTDNVYIKNADIVEEAKKVKPTVVVNAANVYLKHGGGVAGALNKATN
EPI_ISL_426910  203 QTIEVNSFSGYLKLTDNVYIKNADIVEEAKKVKPTVVVNAANVYLKHGGGVAGALNKATN
ORF1ab         1021 QTIEVNSFSGYLKLTDNVYIKNADIVEEAKKVKPTVVVNAANVYLKHGGGVAGALNKATN
EPI_ISL_413575  203 QTIEVNSFSGYLKLTDNVYIKNADIVEEAKKVKPTVVVNAANVYLKHGGGVAGALNKATN
EPI_ISL_413588  203 QTIEVNSFSGYLKLTDNVYIKNADIVEEAKKVKPTVVVNAANVYLKHGGGVAGALNKATN
EPI_ISL_413589  203 QTIEVNSFSGYLKLTDNVYIKNADIVEEAKKVKPTVVVNAANVYLKHGGGVAGALNKATN
EPI_ISL_424221  203 QTIEVNSFSGYLKLTDNVYIKNADIVEEAKKVKPTVVVNAANVYLKHGGGVAGALNKATN
EPI_ISL_424233  203 QTIEVNSFSGYLKLTDNVYIKNADIVEEAKKVKPTVVVNAANVYLKHGGGVAGALNKATN
EPI_ISL_424236  203 QTIEVNSFSGYLKLTDNVYIKNADIVEEAKKVKPTVVVNAANVYLKHGGGVAGALNKATN
EPI_ISL_424238  203 QTIEVNSFSGYLKLTDNVYIKNADIVEEAKKVKPTVVVNAANVYLKHGGGVAGALNKATN
EPI_ISL_424248  203 QTIEVNSFSGYLKLTDNVYIKNADIVEEAKKVKPTVVVNAANVYLKHGGGVAGALNKATN
EPI_ISL_424254  203 QTIEVNSFSGYLKLTDNVYIKNADIVEEAKKVKPTVVVNAANVYLKHGGGVAGALNKATN
EPI_ISL_425548  203 QTIEVNSFSGYLKLTDNVYIKNADIVEEAKKVKPTVVVNAANVYLKHGGGVAGALNKATN
EPI_ISL_425581  203 QTIEVNSFSGYLKLTDNVYIKNADIVEEAKKVKPTVVVNAANVYLKHGGGVAGALNKATN
EPI_ISL_426881  203 QTIEVNSFSGYLKLTDNVYIKNADIVEEAKKVKPTVVVNAANVYLKHGGGVAGALNKATN
EPI_ISL_426883  203 QTIEVNSFSGYLKLTDNVYIKNADIVEEAKKVKPTVVVNAANVYLKHGGGVAGALNKATN
EPI_ISL_426888  203 QTIEVNSFSGYLKLTDNVYIKNADIVEEAKKVKPTVVVNAANVYLKHGGGVAGALNKATN
EPI_ISL_426900  203 QTIEVNSFSGYLKLTDNVYIKNADIVEEAKKVKPTVVVNAANVYLKHGGGVAGALNKATN
EPI_ISL_426901  203 QTIEVNSFSGYLKLTDNVYIKNADIVEEAKKVKPTVVVNAANVYLKHGGGVAGALNKATN
EPI_ISL_426903  203 QTIEVNSFSGYLKLTDNVYIKNADIVEEAKKVKPTVVVNAANVYLKHGGGVAGALNKATN
EPI_ISL_426904  203 QTIEVNSFSGYLKLTDNVYIKNADIVEEAKKVKPTVVVNAANVYLKHGGGVAGALNKATN
EPI_ISL_426907  203 QTIEVNSFSGYLKLTDNVYIKNADIVEEAKKVKPTVVVNAANVYLKHGGGVAGALNKATN
EPI_ISL_426910  203 QTIEVNSFSGYLKLTDNVYIKNADIVEEAKKVKPTVVVNAANVYLKHGGGVAGALNKATN
```

ORF1ab	1081	NAMQVESDDYIATNGPLKVGGSCVLSGHNLAKHCLHVVGPNVNKGEDIQLLKSAYENFNQ
EPI_ISL_413575	263	NAMQVESDDYIATNGPLKVGGSCVLSGHNLAKHCLHVVGPNVNKGEDIQLLKSAYENFNQ
EPI_ISL_413588	263	NAMQVESDDYIATNGPLKVGGSCVLSGHNLAKHCLHVVGPNVNKGEDIQLLKSAYENFNQ
EPI_ISL_413589	263	NAMQVESDDYIATNGPLKVGGSCVLSGHNLAKHCLHVVGPNVNKGEDIQLLKSAYENFNQ
EPI_ISL_424221	263	NAMQVESDDYIATNGPLKVGGSCVLSGHNLAKHCLHVVGPNVNKGEDIQLLKSAYENFNQ
EPI_ISL_424233	263	NAMQVESDDYIATNGPLKVGGSCVLSGHNLAKHCLHVVGPNVNKGEDIQLLKSAYENFNQ
EPI_ISL_424236	263	NAMQVESDDYIATNGPLKVGGSCVLSGHNLAKHCLHVVGPNVNKGEDIQLLKSAYENFNQ
EPI_ISL_424238	263	NAMQVESDDYIATNGPLKVGGSCVLSGHNLAKHCLHVVGPNVNKGEDIQLLKSAYENFNQ
EPI_ISL_424248	263	NAMQVESDDYIATNGPLKVGGSCVLSGHNLAKHCLHVVGPNVNKGEDIQLLKSAYENFNQ
EPI_ISL_424254	263	NAMQVESDDYIATNGPLKVGGSCVLSGHNLAKHCLHVVGPNVNKGEDIQLLKSAYENFNQ
EPI_ISL_425548	263	NAMQVESDDYIATNGPLKVGGSCVLSGHNLAKHCLHVVGPNVNKGEDIQLLKSAYENFNQ
EPI_ISL_425581	263	NAMQVESDDYIATNGPLKVGGSCVLSGHNLAKHCLHVVGPNVNKGEDIQLLKSAYENFNQ
EPI_ISL_426881	263	NAMQVESDDYIATNGPLKVGGSCVLSGHNLAKHCLHVVGPNVNKGEDIQLLKSAYENFNQ
EPI_ISL_426883	263	NAMQVESDDYIATNGPLKVGGSCVLSGHNLAKHCLHVVGPNVNKGEDIQLLKSAYENFNQ
EPI_ISL_426888	263	NAMQVESDDYIATNGPLKVGGSCVLSGHNLAKHCLHVVGPNVNKGEDIQLLKSAYENFNQ
EPI_ISL_426900	263	NAMQVESDDYIATNGPLKVGGSCVLSGHNLAKHCLHVVGPNVNKGEDIQLLKSAYENFNQ
EPI_ISL_426901	263	NAMQVESDDYIATNGPLKVGGSCVLSGHNLAKHCLHVVGPNVNKGEDIQLLKSAYENFNQ
EPI_ISL_426903	263	NAMQVESDDYIATNGPLKVGGSCVLSGHNLAKHCLHVVGPNVNKGEDIQLLKSAYENFNQ
EPI_ISL_426904	263	NAMQVESDDYIATNGPLKVGGSCVLSGHNLAKHCLHVVGPNVNKGEDIQLLKSAYENFNQ
EPI_ISL_426907	263	NAMQVESDDYIATNGPLKVGGSCVLSGHNLAKHCLHVVGPNVNKGEDIQLLKSAYENFNQ
EPI_ISL_426910	263	NAMQVESDDYIATNGPLKVGGSCVLSGHNLAKHCLHVVGPNVNKGEDIQLLKSAYENFNQ
ORF1ab	1081	NAMQVESDDYIATNGPLKVGGSCVLSGHNLAKHCLHVVGPNVNKGEDIQLLKSAYENFNQ
EPI_ISL_413575	263	NAMQVESDDYIATNGPLKVGGSCVLSGHNLAKHCLHVVGPNVNKGEDIQLLKSAYENFNQ
EPI_ISL_413588	263	NAMQVESDDYIATNGPLKVGGSCVLSGHNLAKHCLHVVGPNVNKGEDIQLLKSAYENFNQ
EPI_ISL_413589	263	NAMQVESDDYIATNGPLKVGGSCVLSGHNLAKHCLHVVGPNVNKGEDIQLLKSAYENFNQ
EPI_ISL_424221	263	NAMQVESDDYIATNGPLKVGGSCVLSGHNLAKHCLHVVGPNVNKGEDIQLLKSAYENFNQ
EPI_ISL_424233	263	NAMQVESDDYIATNGPLKVGGSCVLSGHNLAKHCLHVVGPNVNKGEDIQLLKSAYENFNQ
EPI_ISL_424236	263	NAMQVESDDYIATNGPLKVGGSCVLSGHNLAKHCLHVVGPNVNKGEDIQLLKSAYENFNQ
EPI_ISL_424238	263	NAMQVESDDYIATNGPLKVGGSCVLSGHNLAKHCLHVVGPNVNKGEDIQLLKSAYENFNQ
EPI_ISL_424248	263	NAMQVESDDYIATNGPLKVGGSCVLSGHNLAKHCLHVVGPNVNKGEDIQLLKSAYENFNQ
EPI_ISL_424254	263	NAMQVESDDYIATNGPLKVGGSCVLSGHNLAKHCLHVVGPNVNKGEDIQLLKSAYENFNQ
EPI_ISL_425548	263	NAMQVESDDYIATNGPLKVGGSCVLSGHNLAKHCLHVVGPNVNKGEDIQLLKSAYENFNQ
EPI_ISL_425581	263	NAMQVESDDYIATNGPLKVGGSCVLSGHNLAKHCLHVVGPNVNKGEDIQLLKSAYENFNQ
EPI_ISL_426881	263	NAMQVESDDYIATNGPLKVGGSCVLSGHNLAKHCLHVVGPNVNKGEDIQLLKSAYENFNQ
EPI_ISL_426883	263	NAMQVESDDYIATNGPLKVGGSCVLSGHNLAKHCLHVVGPNVNKGEDIQLLKSAYENFNQ
EPI_ISL_426888	263	NAMQVESDDYIATNGPLKVGGSCVLSGHNLAKHCLHVVGPNVNKGEDIQLLKSAYENFNQ
EPI_ISL_426900	263	NAMQVESDDYIATNGPLKVGGSCVLSGHNLAKHCLHVVGPNVNKGEDIQLLKSAYENFNQ
EPI_ISL_426901	263	NAMQVESDDYIATNGPLKVGGSCVLSGHNLAKHCLHVVGPNVNKGEDIQLLKSAYENFNQ
EPI_ISL_426903	263	NAMQVESDDYIATNGPLKVGGSCVLSGHNLAKHCLHVVGPNVNKGEDIQLLKSAYENFNQ
EPI_ISL_426904	263	NAMQVESDDYIATNGPLKVGGSCVLSGHNLAKHCLHVVGPNVNKGEDIQLLKSAYENFNQ
EPI_ISL_426907	263	NAMQVESDDYIATNGPLKVGGSCVLSGHNLAKHCLHVVGPNVNKGEDIQLLKSAYENFNQ
EPI_ISL_426910	263	NAMQVESDDYIATNGPLKVGGSCVLSGHNLAKHCLHVVGPNVNKGEDIQLLKSAYENFNQ

ORF1ab	1141	HEVLLAPLLSAGIFGADPIHSLRVCVDTVRTNVYLAVFDKNLYDKLVSSFLEMKSEKQVE
EPI_ISL_413575	323	HEVLLAPLLSAGIFGADPIHSLRVCVDTVRTNVYLAVFDKNLYDKLVSSFLEMKSEKQVE
EPI_ISL_413588	323	HEVLLAPLLSAGIFGADPIHSLRVCVDTVRTNVYLAVFDKNLYDKLVSSFLEMKSEKQVE
EPI_ISL_413589	323	HEVLLAPLLSAGIFGADPIHSLRVCVDTVRTNVYLAVFDKNLYDKLVSSFLEMKSEKQVE
EPI_ISL_424221	323	HEVLLAPLLSAGIFGADPIHSLRVCVDTVRTNVYLAVFDKNLYDKLVSSFLEMKSEKQVE
EPI_ISL_424233	323	HEVLLAPLLSAGIFGADPIHSLRVCVDTVRTNVYLAVFDKNLYDKLVSSFLEMKSEKQVE
EPI_ISL_424236	323	HEVLLAPLLSAGIFGADPIHSLRVCVDTVRTNVYLAVFDKNLYDKLVSSFLEMKSEKQVE
EPI_ISL_424238	323	HEVLLAPLLSAGIFGADPIHSLRVCVDTVRTNVYLAVFDKNLYDKLVSSFLEMKSEKQVE
EPI_ISL_424248	323	HEVLLAPLLSAGIFGADPIHSLRVCVDTVRTNVYLAVFDKNLYDKLVSSFLEMKSEKQVE
EPI_ISL_424254	323	HEVLLAPLLSAGIFGADPIHSLRVCVDTVRTNVYLAVFDKNLYDKLVSSFLEMKSEKQVE
EPI_ISL_425548	323	HEVLLAPLLSAGIFGADPIHSLRVCVDTVRTNVYLAVFDKNLYDKLVSSFLEMKSEKQVE
EPI_ISL_425581	323	HEVLLAPLLSAGIFGADPIHSLRVCVDTVRTNVYLAVFDKNLYDKLVSSFLEMKSEKQVE
EPI_ISL_426881	323	HEVLLAPLLSAGIFGADPIHSLRVCVDTVRTNVYLAVFDKNLYDKLVSSFLEMKSEKQVE
EPI_ISL_426883	323	HEVLLAPLLSAGIFGADPIHSLRVCVDTVRTNVYLAVFDKNLYDKLVSSFLEMKSEKQVE
EPI_ISL_426888	323	HEVLLAPLLSAGIFGADPIHSLRVCVDTVRTNVYLAVFDKNLYDKLVSSFLEMKSEKQVE
EPI_ISL_426900	323	HEVLLAPLLSAGIFGADPIHSLRVCVDTVRTNVYLAVFDKNLYDKLVSSFLEMKSEKQVE
EPI_ISL_426901	323	HEVLLAPLLSAGIFGADPIHSLRVCVDTVRTNVYLAVFDKNLYDKLVSSFLEMKSEKQVE
EPI_ISL_426903	323	HEVLLAPLLSAGIFGADPIHSLRVCVDTVRTNVYLAVFDKNLYDKLVSSFLEMKSEKQVE
EPI_ISL_426904	323	HEVLLAPLLSAGIFGADPIHSLRVCVDTVRTNVYLAVFDKNLYDKLVSSFLEMKSEKQVE
EPI_ISL_426907	323	HEVLLAPLLSAGIFGADPIHSLRVCVDTVRTNVYLAVFDKNLYDKLVSSFLEMKSEKQVE
EPI_ISL_426910	323	HEVLLAPLLSAGIFGADPIHSLRVCVDTVRTNVYLAVFDKNLYDKLVSSFLEMKSEKQVE
ORF1ab	1141	HEVLLAPLLSAGIFGADPIHSLRVCVDTVRTNVYLAVFDKNLYDKLVSSFLEMKSEKQVE
EPI_ISL_413575	323	HEVLLAPLLSAGIFGADPIHSLRVCVDTVRTNVYLAVFDKNLYDKLVSSFLEMKSEKQVE
EPI_ISL_413588	323	HEVLLAPLLSAGIFGADPIHSLRVCVDTVRTNVYLAVFDKNLYDKLVSSFLEMKSEKQVE
EPI_ISL_413589	323	HEVLLAPLLSAGIFGADPIHSLRVCVDTVRTNVYLAVFDKNLYDKLVSSFLEMKSEKQVE
EPI_ISL_424221	323	HEVLLAPLLSAGIFGADPIHSLRVCVDTVRTNVYLAVFDKNLYDKLVSSFLEMKSEKQVE
EPI_ISL_424233	323	HEVLLAPLLSAGIFGADPIHSLRVCVDTVRTNVYLAVFDKNLYDKLVSSFLEMKSEKQVE
EPI_ISL_424236	323	HEVLLAPLLSAGIFGADPIHSLRVCVDTVRTNVYLAVFDKNLYDKLVSSFLEMKSEKQVE
EPI_ISL_424238	323	HEVLLAPLLSAGIFGADPIHSLRVCVDTVRTNVYLAVFDKNLYDKLVSSFLEMKSEKQVE
EPI_ISL_424248	323	HEVLLAPLLSAGIFGADPIHSLRVCVDTVRTNVYLAVFDKNLYDKLVSSFLEMKSEKQVE
EPI_ISL_424254	323	HEVLLAPLLSAGIFGADPIHSLRVCVDTVRTNVYLAVFDKNLYDKLVSSFLEMKSEKQVE
EPI_ISL_425548	323	HEVLLAPLLSAGIFGADPIHSLRVCVDTVRTNVYLAVFDKNLYDKLVSSFLEMKSEKQVE
EPI_ISL_425581	323	HEVLLAPLLSAGIFGADPIHSLRVCVDTVRTNVYLAVFDKNLYDKLVSSFLEMKSEKQVE
EPI_ISL_426881	323	HEVLLAPLLSAGIFGADPIHSLRVCVDTVRTNVYLAVFDKNLYDKLVSSFLEMKSEKQVE
EPI_ISL_426883	323	HEVLLAPLLSAGIFGADPIHSLRVCVDTVRTNVYLAVFDKNLYDKLVSSFLEMKSEKQVE
EPI_ISL_426888	323	HEVLLAPLLSAGIFGADPIHSLRVCVDTVRTNVYLAVFDKNLYDKLVSSFLEMKSEKQVE
EPI_ISL_426900	323	HEVLLAPLLSAGIFGADPIHSLRVCVDTVRTNVYLAVFDKNLYDKLVSSFLEMKSEKQVE
EPI_ISL_426901	323	HEVLLAPLLSAGIFGADPIHSLRVCVDTVRTNVYLAVFDKNLYDKLVSSFLEMKSEKQVE
EPI_ISL_426903	323	HEVLLAPLLSAGIFGADPIHSLRVCVDTVRTNVYLAVFDKNLYDKLVSSFLEMKSEKQVE
EPI_ISL_426904	323	HEVLLAPLLSAGIFGADPIHSLRVCVDTVRTNVYLAVFDKNLYDKLVSSFLEMKSEKQVE
EPI_ISL_426907	323	HEVLLAPLLSAGIFGADPIHSLRVCVDTVRTNVYLAVFDKNLYDKLVSSFLEMKSEKQVE
EPI_ISL_426910	323	HEVLLAPLLSAGIFGADPIHSLRVCVDTVRTNVYLAVFDKNLYDKLVSSFLEMKSEKQVE

```
ORF1ab          1201 QKIAEIPKEEVKPFITESKPSVEQRKQDDKKIKACVEEVTTTLEETKFLTENLLLYIDIN
EPI_ISL_413575   383 QKIAEIPKEEVKPFITESKPSVEQRKQDDKKIKACVEEVTTTLEETKFLTENLLLYIDIN
EPI_ISL_413588   383 QKIAEIPKEEVKPFITESKPSVEQRKQDDKKIKACVEEVTTTLEETKFLTENLLLYIDIN
EPI_ISL_413589   383 QKIAEIPKEEVKPFITESKPSVEQRKQDDKKIKACVEEVTTTLEETKFLTENLLLYIDIN
EPI_ISL_424221   383 QKIAEIPKEEVKPFITESKPSVEQRKQDDKKIKACVEEVTTTLEETKFLTENLLLYIDIN
EPI_ISL_424233   383 QKIAEIPKEEVKPFITESKPSVEQRKQDDKKIKACVEEVTTTLEETKFLTENLLLYIDIN
EPI_ISL_424236   383 QKIAEIPKEEVKPFITESKPSVEQRKQDDKKIKACVEEVTTTLEETKFLTENLLLYIDIN
EPI_ISL_424238   383 QKIAEIPKEEVKPFITESKPSVEQRKQDDKKIKACVEEVTTTLEETKFLTENLLLYIDIN
EPI_ISL_424248   383 QKIAEIPKEEVKPFITESKPSVEQRKQDDKKIKACVEEVTTTLEETKFLTENLLLYIDIN
EPI_ISL_424254   383 QKIAEIPKEEVKPFITESKPSVEQRKQDDKKIKACVEEVTTTLEETKFLTENLLLYIDIN
EPI_ISL_425548   383 QKIAEIPKEEVKPFITESKPSVEQRKQDDKKIKACVEEVTTTLEETKFLTENLLLYIDIN
EPI_ISL_425581   383 QKIAEIPKEEVKPFITESKPSVEQRKQDDKKIKACVEEVTTTLEETKFLTENLLLYIDIN
EPI_ISL_426881   383 QKIAEIPKEEVKPFITESKPSVEQRKQDDKKIKACVEEVTTTLEETKFLTENLLLYIDIN
EPI_ISL_426883   383 QKIAEIPKEEVKPFITESKPSVEQRKQDDKKIKACVEEVTTTLEETKFLTENLLLYIDIN
EPI_ISL_426888   383 QKIAEIPKEEVKPFITESKPSVEQRKQDDKKIKACVEEVTTTLEETKFLTENLLLYIDIN
EPI_ISL_426900   383 QKIAEIPKEEVKPFITESKPSVEQRKQDDKKIKACVEEVTTTLEETKFLTENLLLYIDIN
EPI_ISL_426901   383 QKIAEIPKEEVKPFITESKPSVEQRKQDDKKIKACVEEVTTTLEETKFLTENLLLYIDIN
EPI_ISL_426903   383 QKIAEIPKEEVKPFITESKPSVEQRKQDDKKIKACVEEVTTTLEETKFLTENLLLYIDIN
EPI_ISL_426904   383 QKIAEIPKEEVKPFITESKPSVEQRKQDDKKIKACVEEVTTTLEETKFLTENLLLYIDIN
EPI_ISL_426907   383 QKIAEIPKEEVKPFITESKPSVEQRKQDDKKIKACVEEVTTTLEETKFLTENLLLYIDIN
EPI_ISL_426910   383 QKIAEIPKEEVKPFITESKPSVEQRKQDDKKIKACVEEVTTTLEETKFLTENLLLYIDIN
```

```
ORF1ab          1201 QKIAEIPKEEVKPFITESKPSVEQRKQDDKKIKACVEEVTTTLEETKFLTENLLLYIDIN
EPI_ISL_413575   383 QKIAEIPKEEVKPFITESKPSVEQRKQDDKKIKACVEEVTTTLEETKFLTENLLLYIDIN
EPI_ISL_413588   383 QKIAEIPKEEVKPFITESKPSVEQRKQDDKKIKACVEEVTTTLEETKFLTENLLLYIDIN
EPI_ISL_413589   383 QKIAEIPKEEVKPFITESKPSVEQRKQDDKKIKACVEEVTTTLEETKFLTENLLLYIDIN
EPI_ISL_424221   383 QKIAEIPKEEVKPFITESKPSVEQRKQDDKKIKACVEEVTTTLEETKFLTENLLLYIDIN
EPI_ISL_424233   383 QKIAEIPKEEVKPFITESKPSVEQRKQDDKKIKACVEEVTTTLEETKFLTENLLLYIDIN
EPI_ISL_424236   383 QKIAEIPKEEVKPFITESKPSVEQRKQDDKKIKACVEEVTTTLEETKFLTENLLLYIDIN
EPI_ISL_424238   383 QKIAEIPKEEVKPFITESKPSVEQRKQDDKKIKACVEEVTTTLEETKFLTENLLLYIDIN
EPI_ISL_424248   383 QKIAEIPKEEVKPFITESKPSVEQRKQDDKKIKACVEEVTTTLEETKFLTENLLLYIDIN
EPI_ISL_424254   383 QKIAEIPKEEVKPFITESKPSVEQRKQDDKKIKACVEEVTTTLEETKFLTENLLLYIDIN
EPI_ISL_425548   383 QKIAEIPKEEVKPFITESKPSVEQRKQDDKKIKACVEEVTTTLEETKFLTENLLLYIDIN
EPI_ISL_425581   383 QKIAEIPKEEVKPFITESKPSVEQRKQDDKKIKACVEEVTTTLEETKFLTENLLLYIDIN
EPI_ISL_426881   383 QKIAEIPKEEVKPFITESKPSVEQRKQDDKKIKACVEEVTTTLEETKFLTENLLLYIDIN
EPI_ISL_426883   383 QKIAEIPKEEVKPFITESKPSVEQRKQDDKKIKACVEEVTTTLEETKFLTENLLLYIDIN
EPI_ISL_426888   383 QKIAEIPKEEVKPFITESKPSVEQRKQDDKKIKACVEEVTTTLEETKFLTENLLLYIDIN
EPI_ISL_426900   383 QKIAEIPKEEVKPFITESKPSVEQRKQDDKKIKACVEEVTTTLEETKFLTENLLLYIDIN
EPI_ISL_426901   383 QKIAEIPKEEVKPFITESKPSVEQRKQDDKKIKACVEEVTTTLEETKFLTENLLLYIDIN
EPI_ISL_426903   383 QKIAEIPKEEVKPFITESKPSVEQRKQDDKKIKACVEEVTTTLEETKFLTENLLLYIDIN
EPI_ISL_426904   383 QKIAEIPKEEVKPFITESKPSVEQRKQDDKKIKACVEEVTTTLEETKFLTENLLLYIDIN
EPI_ISL_426907   383 QKIAEIPKEEVKPFITESKPSVEQRKQDDKKIKACVEEVTTTLEETKFLTENLLLYIDIN
EPI_ISL_426910   383 QKIAEIPKEEVKPFITESKPSVEQRKQDDKKIKACVEEVTTTLEETKFLTENLLLYIDIN
```

```
ORF1ab           1261 GNLHPDSATLVSDIDITFLKKDAPYIVGDVVQEGVLTAVVIPTKKAGGTTEMLAKALRKV
EPI_ISL_413575    443 GNLHPDSATLVSDIDITFLKKDAPYIVGDVVQEGVLTAVVIPTKKAGGTTEMLAKALRKV
EPI_ISL_413588    443 GNLHPDSATLVSDIDITFLKKDAPYIVGDVVQEGVLTAVVIPTKKAGGTTEMLAKALRKV
EPI_ISL_413589    443 GNLHPDSATLVSDIDITFLKKDAPYIVGDVVQEGVLTAVVIPTKKAGGTTEMLAKALRKV
EPI_ISL_424221    443 GNLHPDSATLVSDIDITFLKKDAPYIVGDVVQEGVLTAVVIPTKKAGGTTEMLAKALRKV
EPI_ISL_424233    443 GNLHPDSATLVSDIDITFLKKDAPYIVGDVVQEGVLTAVVIPTKKAGGTTEMLAKALRKV
EPI_ISL_424236    443 GNLHPDSATLVSDIDITFLKKDAPYIVGDVVQEGVLTAVVIPTKKAGGTTEMLAKALRKV
EPI_ISL_424238    443 GNLHPDSATLVSDIDITFLKKDAPYIVGDVVQEGVLTAVVIPTKKAGGTTEMLAKALRKV
EPI_ISL_424248    443 GNLHPDSATLVSDIDITFLKKDAPYIVGDVVQEGVLTAVVIPTKKAGGTTEMLAKALRKV
EPI_ISL_424254    443 GNLHPDSATLVSDIDITFLKKDAPYIVGDVVQEGVLTAVVIPTKKAGGTTEMLAKALRKV
EPI_ISL_425548    443 GNLHPDSATLVSDIDITFLKKDAPYIVGDVVQEGVLTAVVIPTKKAGGTTEMLAKALRKV
EPI_ISL_425581    443 GNLHPDSATLVSDIDITFLKKDAPYIVGDVVQEGVLTAVVIPTKKAGGTTEMLAKALRKV
EPI_ISL_426881    443 GNLHPDSATLVSDIDITFLKKDAPYIVGDVVQEGVLTAVVIPTKKAGGTTEMLAKALRKV
EPI_ISL_426883    443 GNLHPDSATLVSDIDITFLKKDAPYIVGDVVQEGVLTAVVIPTKKAGGTTEMLAKALRKV
EPI_ISL_426888    443 GNLHPDSATLVSDIDITFLKKDAPYIVGDVVQEGVLTAVVIPTKKAGGTTEMLAKALRKV
EPI_ISL_426900    443 GNLHPDSATLVSDIDITFLKKDAPYIVGDVVQEGVLTAVVIPTKKAGGTTEMLAKALRKV
EPI_ISL_426901    443 GNLHPDSATLVSDIDITFLKKDAPYIVGDVVQEGVLTAVVIPTKKAGGTTEMLAKALRKV
EPI_ISL_426903    443 GNLHPDSATLVSDIDITFLKKDAPYIVGDVVQEGVLTAVVIPTKKAGGTTEMLAKALRKV
EPI_ISL_426904    443 GNLHPDSATLVSDIDITFLKKDAPYIVGDVVQEGVLTAVVIPTKKAGGTTEMLAKALRKV
EPI_ISL_426907    443 GNLHPDSATLVSDIDITFLKKDAPYIVGDVVQEGVLTAVVIPTKKAGGTTEMLAKALRKV
EPI_ISL_426910    443 GNLHPDSATLVSDIDITFLKKDAPYIVGDVVQEGVLTAVVIPTKKAGGTTEMLAKALRKV
ORF1ab           1261 GNLHPDSATLVSDIDITFLKKDAPYIVGDVVQEGVLTAVVIPTKKAGGTTEMLAKALRKV
EPI_ISL_413575    443 GNLHPDSATLVSDIDITFLKKDAPYIVGDVVQEGVLTAVVIPTKKAGGTTEMLAKALRKV
EPI_ISL_413588    443 GNLHPDSATLVSDIDITFLKKDAPYIVGDVVQEGVLTAVVIPTKKAGGTTEMLAKALRKV
EPI_ISL_413589    443 GNLHPDSATLVSDIDITFLKKDAPYIVGDVVQEGVLTAVVIPTKKAGGTTEMLAKALRKV
EPI_ISL_424221    443 GNLHPDSATLVSDIDITFLKKDAPYIVGDVVQEGVLTAVVIPTKKAGGTTEMLAKALRKV
EPI_ISL_424233    443 GNLHPDSATLVSDIDITFLKKDAPYIVGDVVQEGVLTAVVIPTKKAGGTTEMLAKALRKV
EPI_ISL_424236    443 GNLHPDSATLVSDIDITFLKKDAPYIVGDVVQEGVLTAVVIPTKKAGGTTEMLAKALRKV
EPI_ISL_424238    443 GNLHPDSATLVSDIDITFLKKDAPYIVGDVVQEGVLTAVVIPTKKAGGTTEMLAKALRKV
EPI_ISL_424248    443 GNLHPDSATLVSDIDITFLKKDAPYIVGDVVQEGVLTAVVIPTKKAGGTTEMLAKALRKV
EPI_ISL_424254    443 GNLHPDSATLVSDIDITFLKKDAPYIVGDVVQEGVLTAVVIPTKKAGGTTEMLAKALRKV
EPI_ISL_425548    443 GNLHPDSATLVSDIDITFLKKDAPYIVGDVVQEGVLTAVVIPTKKAGGTTEMLAKALRKV
EPI_ISL_425581    443 GNLHPDSATLVSDIDITFLKKDAPYIVGDVVQEGVLTAVVIPTKKAGGTTEMLAKALRKV
EPI_ISL_426881    443 GNLHPDSATLVSDIDITFLKKDAPYIVGDVVQEGVLTAVVIPTKKAGGTTEMLAKALRKV
EPI_ISL_426883    443 GNLHPDSATLVSDIDITFLKKDAPYIVGDVVQEGVLTAVVIPTKKAGGTTEMLAKALRKV
EPI_ISL_426888    443 GNLHPDSATLVSDIDITFLKKDAPYIVGDVVQEGVLTAVVIPTKKAGGTTEMLAKALRKV
EPI_ISL_426900    443 GNLHPDSATLVSDIDITFLKKDAPYIVGDVVQEGVLTAVVIPTKKAGGTTEMLAKALRKV
EPI_ISL_426901    443 GNLHPDSATLVSDIDITFLKKDAPYIVGDVVQEGVLTAVVIPTKKAGGTTEMLAKALRKV
EPI_ISL_426903    443 GNLHPDSATLVSDIDITFLKKDAPYIVGDVVQEGVLTAVVIPTKKAGGTTEMLAKALRKV
EPI_ISL_426904    443 GNLHPDSATLVSDIDITFLKKDAPYIVGDVVQEGVLTAVVIPTKKAGGTTEMLAKALRKV
EPI_ISL_426907    443 GNLHPDSATLVSDIDITFLKKDAPYIVGDVVQEGVLTAVVIPTKKAGGTTEMLAKALRKV
EPI_ISL_426910    443 GNLHPDSATLVSDIDITFLKKDAPYIVGDVVQEGVLTAVVIPTKKAGGTTEMLAKALRKV
```

```
ORF1ab          1321 PTDNYITTYPGQGLNGYTVEEAKTVLKKCKSAFYILPSIISNEKQEILGTVSWNLREMLA
EPI_ISL_413575   503 PTDNYITTYPGQGLNGYTVEEAKTVLKKCKSAFYILPSIISNEKQEILGTVSWNLREMLA
EPI_ISL_413588   503 PTDNYITTYPGQGLNGYTVEEAKTVLKKCKSAFYILPSIISNEKQEILGTVSWNLREMLA
EPI_ISL_413589   503 PTDNYITTYPGQGLNGYTVEEAKTVLKKCKSAFYILPSIISNEKQEILGTVSWNLREMLA
EPI_ISL_424221   503 PTDNYITTYPGQGLNGYTVEEAKTVLKKCKSAFYILPSIISNEKQEILGTVSWNLREMLA
EPI_ISL_424233   503 PTDNYITTYPGQGLNGYTVEEAKTVLKKCKSAFYILPSIISNEKQEILGTVSWNLREMLA
EPI_ISL_424236   503 PTDNYITTYPGQGLNGYTVEEAKTVLKKCKSAFYILPSIISNEKQEILGTVSWNLREMLA
EPI_ISL_424238   503 PTDNYITTYPGQGLNGYTVEEAKTVLKKCKSAFYILPSIISNEKQEILGTVSWNLREMLA
EPI_ISL_424248   503 PTDNYITTYPGQGLNGYTVEEAKTVLKKCKSAFYILPSIISNEKQEILGTVSWNLREMLA
EPI_ISL_424254   503 PTDNYITTYPGQGLNGYTVEEAKTVLKKCKSAFYILPSIISNEKQEILGTVSWNLREMLA
EPI_ISL_425548   503 PTDNYITTYPGQGLNGYTVEEAKTVLKKCKSAFYILPSIISNEKQEILGTVSWNLREMLA
EPI_ISL_425581   503 PTDNYITTYPGQGLNGYTVEEAKTVLKKCKSAFYILPSIISNEKQEILGTVSWNLREMLA
EPI_ISL_426881   503 PTDNYITTYPGQGLNGYTVEEAKTVLKKCKSAFYILPSIISNEKQEILGTVSWNLREMLA
EPI_ISL_426883   503 PTDNYITTYPGQGLNGYTVEEAKTVLKKCKSAFYILPSIISNEKQEILGTVSWNLREMLA
EPI_ISL_426888   503 PTDNYITTYPGQGLNGYTVEEAKTVLKKCKSAFYILPSIISNEKQEILGTVSWNLREMLA
EPI_ISL_426900   503 PTDNYITTYPGQGLNGYTVEEAKTVLKKCKSAFYILPSIISNEKQEILGTVSWNLREMLA
EPI_ISL_426901   503 PTDNYITTYPGQGLNGYTVEEAKTVLKKCKSAFYILPSIISNEKQEILGTVSWNLREMLA
EPI_ISL_426903   503 PTDNYITTYPGQGLNGYTVEEAKTVLKKCKSAFYILPSIISNEKQEILGTVSWNLREMLA
EPI_ISL_426904   503 PTDNYITTYPGQGLNGYTVEEAKTVLKKCKSAFYILPSIISNEKQEILGTVSWNLREMLA
EPI_ISL_426907   503 PTDNYITTYPGQGLNGYTVEEAKTVLKKCKSAFYILPSIISNEKQEILGTVSWNLREMLA
EPI_ISL_426910   503 PTDNYITTYPGQGLNGYTVEEAKTVLKKCKSAFYILPSIISNEKQEILGTVSWNLREMLA
```

```
ORF1ab          1321 PTDNYITTYPGQGLNGYTVEEAKTVLKKCKSAFYILPSIISNEKQEILGTVSWNLREMLA
EPI_ISL_413575   503 PTDNYITTYPGQGLNGYTVEEAKTVLKKCKSAFYILPSIISNEKQEILGTVSWNLREMLA
EPI_ISL_413588   503 PTDNYITTYPGQGLNGYTVEEAKTVLKKCKSAFYILPSIISNEKQEILGTVSWNLREMLA
EPI_ISL_413589   503 PTDNYITTYPGQGLNGYTVEEAKTVLKKCKSAFYILPSIISNEKQEILGTVSWNLREMLA
EPI_ISL_424221   503 PTDNYITTYPGQGLNGYTVEEAKTVLKKCKSAFYILPSIISNEKQEILGTVSWNLREMLA
EPI_ISL_424233   503 PTDNYITTYPGQGLNGYTVEEAKTVLKKCKSAFYILPSIISNEKQEILGTVSWNLREMLA
EPI_ISL_424236   503 PTDNYITTYPGQGLNGYTVEEAKTVLKKCKSAFYILPSIISNEKQEILGTVSWNLREMLA
EPI_ISL_424238   503 PTDNYITTYPGQGLNGYTVEEAKTVLKKCKSAFYILPSIISNEKQEILGTVSWNLREMLA
EPI_ISL_424248   503 PTDNYITTYPGQGLNGYTVEEAKTVLKKCKSAFYILPSIISNEKQEILGTVSWNLREMLA
EPI_ISL_424254   503 PTDNYITTYPGQGLNGYTVEEAKTVLKKCKSAFYILPSIISNEKQEILGTVSWNLREMLA
EPI_ISL_425548   503 PTDNYITTYPGQGLNGYTVEEAKTVLKKCKSAFYILPSIISNEKQEILGTVSWNLREMLA
EPI_ISL_425581   503 PTDNYITTYPGQGLNGYTVEEAKTVLKKCKSAFYILPSIISNEKQEILGTVSWNLREMLA
EPI_ISL_426881   503 PTDNYITTYPGQGLNGYTVEEAKTVLKKCKSAFYILPSIISNEKQEILGTVSWNLREMLA
EPI_ISL_426883   503 PTDNYITTYPGQGLNGYTVEEAKTVLKKCKSAFYILPSIISNEKQEILGTVSWNLREMLA
EPI_ISL_426888   503 PTDNYITTYPGQGLNGYTVEEAKTVLKKCKSAFYILPSIISNEKQEILGTVSWNLREMLA
EPI_ISL_426900   503 PTDNYITTYPGQGLNGYTVEEAKTVLKKCKSAFYILPSIISNEKQEILGTVSWNLREMLA
EPI_ISL_426901   503 PTDNYITTYPGQGLNGYTVEEAKTVLKKCKSAFYILPSIISNEKQEILGTVSWNLREMLA
EPI_ISL_426903   503 PTDNYITTYPGQGLNGYTVEEAKTVLKKCKSAFYILPSIISNEKQEILGTVSWNLREMLA
EPI_ISL_426904   503 PTDNYITTYPGQGLNGYTVEEAKTVLKKCKSAFYILPSIISNEKQEILGTVSWNLREMLA
EPI_ISL_426907   503 PTDNYITTYPGQGLNGYTVEEAKTVLKKCKSAFYILPSIISNEKQEILGTVSWNLREMLA
EPI_ISL_426910   503 PTDNYITTYPGQGLNGYTVEEAKTVLKKCKSAFYILPSIISNEKQEILGTVSWNLREMLA
```

```
ORF1ab          1381 HAEETRKLMPVCVETKAIVSTIQRKYKGIKIQEGVVDYGARFYFYTSKTTVASLINTLND
EPI_ISL_413575   563 HAEETRKLMPVCVETKAIVSTIQRKYKGIKIQEGVVDYGARFYFYTSKTTVASLINTLND
EPI_ISL_413588   563 HAEETRKLMPVCVETKAIVSTIQRKYKGIKIQEGVVDYGARFYFYTSKTTVASLINTLND
EPI_ISL_413589   563 HAEETRKLMPVCVETKAIVSTIQRKYKGIKIQEGVVDYGARFYFYTSKTTVASLINTLND
EPI_ISL_424221   563 HAEETRKLMPVCVETKAIVSTIQRKYKGIKIQEGVVDYGARFYFYTSKTTVASLINTLND
EPI_ISL_424233   563 HAEETRKLMPVCVETKAIVSTIQRKYKGIKIQEGVVDYGARFYFYTSKTTVASLINTLND
EPI_ISL_424236   563 HAEETRKLMPVCVETKAIVSTIQRKYKGIKIQEGVVDYGARFYFYTSKTTVASLINTLND
EPI_ISL_424238   563 HAEETRKLMPVCVETKAIVSTIQRKYKGIKIQEGVVDYGARFYFYTSKTTVASLINTLND
EPI_ISL_424248   563 HAEETRKLMPVCVETKAIVSTIQRKYKGIKIQEGVVDYGARFYFYTSKTTVASLINTLND
EPI_ISL_424254   563 HAEETRKLMPVCVETKAIVSTIQRKYKGIKIQEGVVDYGARFYFYTSKTTVASLINTLND
EPI_ISL_425548   563 HAEETRKLMPVCVETKAIVSTIQRKYKGIKIQEGVVDYGARFYFYTSKTTVASLINTLND
EPI_ISL_425581   563 HAEETRKLMPVCVETKAIVSTIQRKYKGIKIQEGVVDYGARFYFYTSKTTVASLINTLND
EPI_ISL_426881   563 HAEETRKLMPVCVETKAIVSTIQRKYKGIKIQEGVVDYGARFYFYTSKTTVASLINTLND
EPI_ISL_426883   563 HAEETRKLMPVCVETKAIVSTIQRKYKGIKIQEGVVDYGARFYFYTSKTTVASLINTLND
EPI_ISL_426888   563 HAEETRKLMPVCVETKAIVSTIQRKYKGIKIQEGVVDYGARFYFYTSKTTVASLINTLND
EPI_ISL_426900   563 HAEETRKLMPVCVETKAIVSTIQRKYKGIKIQEGVVDYGARFYFYTSKTTVASLINTLND
EPI_ISL_426901   563 HAEETRKLMPVCVETKAIVSTIQRKYKGIKIQEGVVDYGARFYFYTSKTTVASLINTLND
EPI_ISL_426903   563 HAEETRKLMPVCVETKAIVSTIQRKYKGIKIQEGVVDYGARFYFYTSKTTVASLINTLND
EPI_ISL_426904   563 HAEETRKLMPVCVETKAIVSTIQRKYKGIKIQEGVVDYGARFYFYTSKTTVASLINTLND
EPI_ISL_426907   563 HAEETRKLMPVCVETKAIVSTIQRKYKGIKIQEGVVDYGARFYFYTSKTTVASLINTLND
EPI_ISL_426910   563 HAEETRKLMPVCVETKAIVSTIQRKYKGIKIQEGVVDYGARFYFYTSKTTVASLINTLND
ORF1ab          1381 HAEETRKLMPVCVETKAIVSTIQRKYKGIKIQEGVVDYGARFYFYTSKTTVASLINTLND
EPI_ISL_413575   563 HAEETRKLMPVCVETKAIVSTIQRKYKGIKIQEGVVDYGARFYFYTSKTTVASLINTLND
EPI_ISL_413588   563 HAEETRKLMPVCVETKAIVSTIQRKYKGIKIQEGVVDYGARFYFYTSKTTVASLINTLND
EPI_ISL_413589   563 HAEETRKLMPVCVETKAIVSTIQRKYKGIKIQEGVVDYGARFYFYTSKTTVASLINTLND
EPI_ISL_424221   563 HAEETRKLMPVCVETKAIVSTIQRKYKGIKIQEGVVDYGARFYFYTSKTTVASLINTLND
EPI_ISL_424233   563 HAEETRKLMPVCVETKAIVSTIQRKYKGIKIQEGVVDYGARFYFYTSKTTVASLINTLND
EPI_ISL_424236   563 HAEETRKLMPVCVETKAIVSTIQRKYKGIKIQEGVVDYGARFYFYTSKTTVASLINTLND
EPI_ISL_424238   563 HAEETRKLMPVCVETKAIVSTIQRKYKGIKIQEGVVDYGARFYFYTSKTTVASLINTLND
EPI_ISL_424248   563 HAEETRKLMPVCVETKAIVSTIQRKYKGIKIQEGVVDYGARFYFYTSKTTVASLINTLND
EPI_ISL_424254   563 HAEETRKLMPVCVETKAIVSTIQRKYKGIKIQEGVVDYGARFYFYTSKTTVASLINTLND
EPI_ISL_425548   563 HAEETRKLMPVCVETKAIVSTIQRKYKGIKIQEGVVDYGARFYFYTSKTTVASLINTLND
EPI_ISL_425581   563 HAEETRKLMPVCVETKAIVSTIQRKYKGIKIQEGVVDYGARFYFYTSKTTVASLINTLND
EPI_ISL_426881   563 HAEETRKLMPVCVETKAIVSTIQRKYKGIKIQEGVVDYGARFYFYTSKTTVASLINTLND
EPI_ISL_426883   563 HAEETRKLMPVCVETKAIVSTIQRKYKGIKIQEGVVDYGARFYFYTSKTTVASLINTLND
EPI_ISL_426888   563 HAEETRKLMPVCVETKAIVSTIQRKYKGIKIQEGVVDYGARFYFYTSKTTVASLINTLND
EPI_ISL_426900   563 HAEETRKLMPVCVETKAIVSTIQRKYKGIKIQEGVVDYGARFYFYTSKTTVASLINTLND
EPI_ISL_426901   563 HAEETRKLMPVCVETKAIVSTIQRKYKGIKIQEGVVDYGARFYFYTSKTTVASLINTLND
EPI_ISL_426903   563 HAEETRKLMPVCVETKAIVSTIQRKYKGIKIQEGVVDYGARFYFYTSKTTVASLINTLND
EPI_ISL_426904   563 HAEETRKLMPVCVETKAIVSTIQRKYKGIKIQEGVVDYGARFYFYTSKTTVASLINTLND
EPI_ISL_426907   563 HAEETRKLMPVCVETKAIVSTIQRKYKGIKIQEGVVDYGARFYFYTSKTTVASLINTLND
EPI_ISL_426910   563 HAEETRKLMPVCVETKAIVSTIQRKYKGIKIQEGVVDYGARFYFYTSKTTVASLINTLND
```

ORF1ab	1441	LNETLVTMPLGYVTHGLNLEEAARYMRSLKVPATVSVSSPDAVTAYNGYLTSSSKTPEEH
EPI_ISL_413575	623	LNETLVTMPLGYVTHGLNLEEAARYMRSLKVPATVSVSSPDAVTAYNGYLTSSSKTPEEH
EPI_ISL_413588	623	LNETLVTMPLGYVTHGLNLEEAARYMRSLKVPATVSVSSPDAVTAYNGYLTSSSKTPEEH
EPI_ISL_413589	623	LNETLVTMPLGYVTHGLNLEEAARYMRSLKVPATVSVSSPDAVTAYNGYLTSSSKTPEEH
EPI_ISL_424221	623	LNETLVTMPLGYVTHGLNLEEAARYMRSLKVPATVSVSSPDAVTAYNGYLTSSSKTPEEH
EPI_ISL_424233	623	LNETLVTMPLGYVTHGLNLEEAARYMRSLKVPATVSVSSPDAVTAYNGYLTSSSKTPEEH
EPI_ISL_424236	623	LNETLVTMPLGYVTHGLNLEEAARYMRSLKVPATVSVSSPDAVTAYNGYLTSSSKTPEEH
EPI_ISL_424238	623	LNETLVTMPLGYVTHGLNLEEAARYMRSLKVPATVSVSSPDAVTAYNGYLTSSSKTPEEH
EPI_ISL_424248	623	LNETLVTMPLGYVTHGLNLEEAARYMRSLKVPATVSVSSPDAVTAYNGYLTSSSKTPEEH
EPI_ISL_424254	623	LNETLVTMPLGYVTHGLNLEEAARYMRSLKVPATVSVSSPDAVTAYNGYLTSSSKTPEEH
EPI_ISL_425548	623	LNETLVTMPLGYVTHGLNLEEAARYMRSLKVPATVSVSSPDAVTAYNGYLTSSSKTPEEH
EPI_ISL_425581	623	LNETLVTMPLGYVTHGLNLEEAARYMRSLKVPATVSVSSPDAVTAYNGYLTSSSKTPEEH
EPI_ISL_426881	623	LNETLVTMPLGYVTHGLNLEEAARYMRSLKVPATVSVSSPDAVTAYNGYLTSSSKTPEEH
EPI_ISL_426883	623	LNETLVTMPLGYVTHGLNLEEAARYMRSLKVPATVSVSSPDAVTAYNGYLTSSSKTPEEH
EPI_ISL_426888	623	LNETLVTMPLGYVTHGLNLEEAARYMRSLKVPATVSVSSPDAVTAYNGYLTSSSKTPEEH
EPI_ISL_426900	623	LNETLVTMPLGYVTHGLNLEEAARYMRSLKVPATVSVSSPDAVTAYNGYLTSSSKTPEEH
EPI_ISL_426901	623	LNETLVTMPLGYVTHGLNLEEAARYMRSLKVPATVSVSSPDAVTAYNGYLTSSSKTPEEH
EPI_ISL_426903	623	LNETLVTMPLGYVTHGLNLEEAARYMRSLKVPATVSVSSPDAVTAYNGYLTSSSKTPEEH
EPI_ISL_426904	623	LNETLVTMPLGYVTHGLNLEEAARYMRSLKVPATVSVSSPDAVTAYNGYLTSSSKTPEEH
EPI_ISL_426907	623	LNETLVTMPLGYVTHGLNLEEAARYMRSLKVPATVSVSSPDAVTAYNGYLTSSSKTPEEH
EPI_ISL_426910	623	LNETLVTMPLGYVTHGLNLEEAARYMRSLKVPATVSVSSPDAVTAYNGYLTSSSKTPEEH

ORF1ab	1441	LNETLVTMPLGYVTHGLNLEEAARYMRSLKVPATVSVSSPDAVTAYNGYLTSSSKTPEEH
EPI_ISL_413575	623	LNETLVTMPLGYVTHGLNLEEAARYMRSLKVPATVSVSSPDAVTAYNGYLTSSSKTPEEH
EPI_ISL_413588	623	LNETLVTMPLGYVTHGLNLEEAARYMRSLKVPATVSVSSPDAVTAYNGYLTSSSKTPEEH
EPI_ISL_413589	623	LNETLVTMPLGYVTHGLNLEEAARYMRSLKVPATVSVSSPDAVTAYNGYLTSSSKTPEEH
EPI_ISL_424221	623	LNETLVTMPLGYVTHGLNLEEAARYMRSLKVPATVSVSSPDAVTAYNGYLTSSSKTPEEH
EPI_ISL_424233	623	LNETLVTMPLGYVTHGLNLEEAARYMRSLKVPATVSVSSPDAVTAYNGYLTSSSKTPEEH
EPI_ISL_424236	623	LNETLVTMPLGYVTHGLNLEEAARYMRSLKVPATVSVSSPDAVTAYNGYLTSSSKTPEEH
EPI_ISL_424238	623	LNETLVTMPLGYVTHGLNLEEAARYMRSLKVPATVSVSSPDAVTAYNGYLTSSSKTPEEH
EPI_ISL_424248	623	LNETLVTMPLGYVTHGLNLEEAARYMRSLKVPATVSVSSPDAVTAYNGYLTSSSKTPEEH
EPI_ISL_424254	623	LNETLVTMPLGYVTHGLNLEEAARYMRSLKVPATVSVSSPDAVTAYNGYLTSSSKTPEEH
EPI_ISL_425548	623	LNETLVTMPLGYVTHGLNLEEAARYMRSLKVPATVSVSSPDAVTAYNGYLTSSSKTPEEH
EPI_ISL_425581	623	LNETLVTMPLGYVTHGLNLEEAARYMRSLKVPATVSVSSPDAVTAYNGYLTSSSKTPEEH
EPI_ISL_426881	623	LNETLVTMPLGYVTHGLNLEEAARYMRSLKVPATVSVSSPDAVTAYNGYLTSSSKTPEEH
EPI_ISL_426883	623	LNETLVTMPLGYVTHGLNLEEAARYMRSLKVPATVSVSSPDAVTAYNGYLTSSSKTPEEH
EPI_ISL_426888	623	LNETLVTMPLGYVTHGLNLEEAARYMRSLKVPATVSVSSPDAVTAYNGYLTSSSKTPEEH
EPI_ISL_426900	623	LNETLVTMPLGYVTHGLNLEEAARYMRSLKVPATVSVSSPDAVTAYNGYLTSSSKTPEEH
EPI_ISL_426901	623	LNETLVTMPLGYVTHGLNLEEAARYMRSLKVPATVSVSSPDAVTAYNGYLTSSSKTPEEH
EPI_ISL_426903	623	LNETLVTMPLGYVTHGLNLEEAARYMRSLKVPATVSVSSPDAVTAYNGYLTSSSKTPEEH
EPI_ISL_426904	623	LNETLVTMPLGYVTHGLNLEEAARYMRSLKVPATVSVSSPDAVTAYNGYLTSSSKTPEEH
EPI_ISL_426907	623	LNETLVTMPLGYVTHGLNLEEAARYMRSLKVPATVSVSSPDAVTAYNGYLTSSSKTPEEH
EPI_ISL_426910	623	LNETLVTMPLGYVTHGLNLEEAARYMRSLKVPATVSVSSPDAVTAYNGYLTSSSKTPEEH

ORF1ab	1501	FIETISLAGSYKDWSYSGQSTQLGIEFLKRGDKSVYYTSNPTTFHLDGEVITFDNLKTLL
EPI_ISL_413575	683	FIETISLAGSYKDWSYSGQSTQLGIEFLKRGDKSVYYTSNPTTFHLDGEVITFDNLKTLL
EPI_ISL_413588	683	FIETISLAGSYKDWSYSGQSTQLGIEFLKRGDKSVYYTSNPTTFHLDGEVITFDNLKTLL
EPI_ISL_413589	683	FIETISLAGSYKDWSYSGQSTQLGIEFLKRGDKSVYYTSNPTTFHLDGEVITFDNLKTLL
EPI_ISL_424221	683	FIETISLAGSYKDWSYSGQSTQLGIEFLKRGDKSVYYTSNPTTFHLDGEVITFDNLKTLL
EPI_ISL_424233	683	FIETISLAGSYKDWSYSGQSTQLGIEFLKRGDKSVYYTSNPTTFHLDGEVITFDNLKTLL
EPI_ISL_424236	683	FIETISLAGSYKDWSYSGQSTQLGIEFLKRGDKSVYYTSNPTTFHLDGEVITFDNLKTLL
EPI_ISL_424238	683	FIETISLAGSYKDWSYSGQSTQLGIEFLKRGDKSVYYTSNPTTFHLDGEVITFDNLKTLL
EPI_ISL_424248	683	FIETISLAGSYKDWSYSGQSTQLGIEFLKRGDKSVYYTSNPTTFHLDGEVITFDNLKTLL
EPI_ISL_424254	683	FIETISLAGSYKDWSYSGQSTQLGIEFLKRGDKSVYYTSNPTTFHLDGEVITFDNLKTLL
EPI_ISL_425548	683	FIETISLAGSYKDWSYSGQSTQLGIEFLKRGDKSVYYTSNPTTFHLDGEVITFDNLKTLL
EPI_ISL_425581	683	FIETISLAGSYKDWSYSGQSTQLGIEFLKRGDKSVYYTSNPTTFHLDGEVITFDNLKTLL
EPI_ISL_426881	683	FIETISLAGSYKDWSYSGQSTQLGIEFLKRGDKSVYYTSNPTTFHLDGEVITFDNLKTLL
EPI_ISL_426883	683	FIETISLAGSYKDWSYSGQSTQLGIEFLKRGDKSVYYTSNPTTFHLDGEVITFDNLKTLL
EPI_ISL_426888	683	FIETISLAGSYKDWSYSGQSTQLGIEFLKRGDKSVYYTSNPTTFHLDGEVITFDNLKTLL
EPI_ISL_426900	683	FIETISLAGSYKDWSYSGQSTQLGIEFLKRGDKSVYYTSNPTTFHLDGEVITFDNLKTLL
EPI_ISL_426901	683	FIETISLAGSYKDWSYSGQSTQLGIEFLKRGDKSVYYTSNPTTFHLDGEVITFDNLKTLL
EPI_ISL_426903	683	FIETISLAGSYKDWSYSGQSTQLGIEFLKRGDKSVYYTSNPTTFHLDGEVITFDNLKTLL
EPI_ISL_426904	683	FIETISLAGSYKDWSYSGQSTQLGIEFLKRGDKSVYYTSNPTTFHLDGEVITFDNLKTLL
EPI_ISL_426907	683	FIETISLAGSYKDWSYSGQSTQLGIEFLKRGDKSVYYTSNPTTFHLDGEVITFDNLKTLL
EPI_ISL_426910	683	FIETISLAGSYKDWSYSGQSTQLGIEFLKRGDKSVYYTSNPTTFHLDGEVITFDNLKTLL
ORF1ab	1501	FIETISLAGSYKDWSYSGQSTQLGIEFLKRGDKSVYYTSNPTTFHLDGEVITFDNLKTLL
EPI_ISL_413575	683	FIETISLAGSYKDWSYSGQSTQLGIEFLKRGDKSVYYTSNPTTFHLDGEVITFDNLKTLL
EPI_ISL_413588	683	FIETISLAGSYKDWSYSGQSTQLGIEFLKRGDKSVYYTSNPTTFHLDGEVITFDNLKTLL
EPI_ISL_413589	683	FIETISLAGSYKDWSYSGQSTQLGIEFLKRGDKSVYYTSNPTTFHLDGEVITFDNLKTLL
EPI_ISL_424221	683	FIETISLAGSYKDWSYSGQSTQLGIEFLKRGDKSVYYTSNPTTFHLDGEVITFDNLKTLL
EPI_ISL_424233	683	FIETISLAGSYKDWSYSGQSTQLGIEFLKRGDKSVYYTSNPTTFHLDGEVITFDNLKTLL
EPI_ISL_424236	683	FIETISLAGSYKDWSYSGQSTQLGIEFLKRGDKSVYYTSNPTTFHLDGEVITFDNLKTLL
EPI_ISL_424238	683	FIETISLAGSYKDWSYSGQSTQLGIEFLKRGDKSVYYTSNPTTFHLDGEVITFDNLKTLL
EPI_ISL_424248	683	FIETISLAGSYKDWSYSGQSTQLGIEFLKRGDKSVYYTSNPTTFHLDGEVITFDNLKTLL
EPI_ISL_424254	683	FIETISLAGSYKDWSYSGQSTQLGIEFLKRGDKSVYYTSNPTTFHLDGEVITFDNLKTLL
EPI_ISL_425548	683	FIETISLAGSYKDWSYSGQSTQLGIEFLKRGDKSVYYTSNPTTFHLDGEVITFDNLKTLL
EPI_ISL_425581	683	FIETISLAGSYKDWSYSGQSTQLGIEFLKRGDKSVYYTSNPTTFHLDGEVITFDNLKTLL
EPI_ISL_426881	683	FIETISLAGSYKDWSYSGQSTQLGIEFLKRGDKSVYYTSNPTTFHLDGEVITFDNLKTLL
EPI_ISL_426883	683	FIETISLAGSYKDWSYSGQSTQLGIEFLKRGDKSVYYTSNPTTFHLDGEVITFDNLKTLL
EPI_ISL_426888	683	FIETISLAGSYKDWSYSGQSTQLGIEFLKRGDKSVYYTSNPTTFHLDGEVITFDNLKTLL
EPI_ISL_426900	683	FIETISLAGSYKDWSYSGQSTQLGIEFLKRGDKSVYYTSNPTTFHLDGEVITFDNLKTLL
EPI_ISL_426901	683	FIETISLAGSYKDWSYSGQSTQLGIEFLKRGDKSVYYTSNPTTFHLDGEVITFDNLKTLL
EPI_ISL_426903	683	FIETISLAGSYKDWSYSGQSTQLGIEFLKRGDKSVYYTSNPTTFHLDGEVITFDNLKTLL
EPI_ISL_426904	683	FIETISLAGSYKDWSYSGQSTQLGIEFLKRGDKSVYYTSNPTTFHLDGEVITFDNLKTLL
EPI_ISL_426907	683	FIETISLAGSYKDWSYSGQSTQLGIEFLKRGDKSVYYTSNPTTFHLDGEVITFDNLKTLL
EPI_ISL_426910	683	FIETISLAGSYKDWSYSGQSTQLGIEFLKRGDKSVYYTSNPTTFHLDGEVITFDNLKTLL

ORF1ab	1561	SLREVRTIKVFTTVDNINLHTQVVDMSMTYGQQFGPTYLDGADVTKIKPHNSHEGKTFYV
EPI_ISL_413575	743	SLREVRTIKVFTTVDNINLHTQVVDMSMTYGQQFGPTYLDGADVTKIKPHNSHEGKTFYV
EPI_ISL_413588	743	SLREVRTIKVFTTVDNINLHTQVVDMSMTYGQQFGPTYLDGADVTKIKPHNSHEGKTFYV
EPI_ISL_413589	743	SLREVRTIKVFTTVDNINLHTQVVDMSMTYGQQFGPTYLDGADVTKIKPHNSHEGKTFYV
EPI_ISL_424221	743	SLREVRTIKVFTTVDNINLHTQVVDMSMTYGQQFGPTYLDGADVTKIKPHNSHEGKTFYV
EPI_ISL_424233	743	SLREVRTIKVFTTVDNINLHTQVVDMSMTYGQQFGPTYLDGADVTKIKPHNSHEGKTFYV
EPI_ISL_424236	743	SLREVRTIKVFTTVDNINLHTQVVDMSMTYGQQFGPTYLDGADVTKIKPHNSHEGKTFYV
EPI_ISL_424238	743	SLREVRTIKVFTTVDNINLHTQVVDMSMTYGQQFGPTYLDGADVTKIKPHNSHEGKTFYV
EPI_ISL_424248	743	SLREVRTIKVFTTVDNINLHTQVVDMSMTYGQQFGPTYLDGADVTKIKPHNSHEGKTFYV
EPI_ISL_424254	743	SLREVRTIKVFTTVDNINLHTQVVDMSMTYGQQFGPTYLDGADVTKIKPHNSHEGKTFYV
EPI_ISL_425548	743	SLREVRTIKVFTTVDNINLHTQVVDMSMTYGQQFGPTYLDGADVTKIKPHNSHEGKTFYV
EPI_ISL_425581	743	SLREVRTIKVFTTVDNINLHTQVVDMSMTYGQQFGPTYLDGADVTKIKPHNSHEGKTFYV
EPI_ISL_426881	743	SLREVRTIKVFTTVDNINLHTQVVDMSMTYGQQFGPTYLDGADVTKIKPHNSHEGKTFYV
EPI_ISL_426883	743	SLREVRTIKVFTTVDNINLHTQVVDMSMTYGQQFGPTYLDGADVTKIKPHNSHEGKTFYV
EPI_ISL_426888	743	SLREVRTIKVFTTVDNINLHTQVVDMSMTYGQQFGPTYLDGADVTKIKPHNSHEGKTFYV
EPI_ISL_426900	743	SLREVRTIKVFTTVDNINLHTQVVDMSMTYGQQFGPTYLDGADVTKIKPHNSHEGKTFYV
EPI_ISL_426901	743	SLREVRTIKVFTTVDNINLHTQVVDMSMTYGQQFGPTYLDGADVTKIKPHNSHEGKTFYV
EPI_ISL_426903	743	SLREVRTIKVFTTVDNINLHTQVVDMSMTYGQQFGPTYLDGADVTKIKPHNSHEGKTFYV
EPI_ISL_426904	743	SLREVRTIKVFTTVDNINLHTQVVDMSMTYGQQFGPTYLDGADVTKIKPHNSHEGKTFYV
EPI_ISL_426907	743	SLREVRTIKVFTTVDNINLHTQVVDMSMTYGQQFGPTYLDGADVTKIKPHNSHEGKTFYV
EPI_ISL_426910	743	SLREVRTIKVFTTVDNINLHTQVVDMSMTYGQQFGPTYLDGADVTKIKPHNSHEGKTFYV
ORF1ab	1561	SLREVRTIKVFTTVDNINLHTQVVDMSMTYGQQFGPTYLDGADVTKIKPHNSHEGKTFYV
EPI_ISL_413575	743	SLREVRTIKVFTTVDNINLHTQVVDMSMTYGQQFGPTYLDGADVTKIKPHNSHEGKTFYV
EPI_ISL_413588	743	SLREVRTIKVFTTVDNINLHTQVVDMSMTYGQQFGPTYLDGADVTKIKPHNSHEGKTFYV
EPI_ISL_413589	743	SLREVRTIKVFTTVDNINLHTQVVDMSMTYGQQFGPTYLDGADVTKIKPHNSHEGKTFYV
EPI_ISL_424221	743	SLREVRTIKVFTTVDNINLHTQVVDMSMTYGQQFGPTYLDGADVTKIKPHNSHEGKTFYV
EPI_ISL_424233	743	SLREVRTIKVFTTVDNINLHTQVVDMSMTYGQQFGPTYLDGADVTKIKPHNSHEGKTFYV
EPI_ISL_424236	743	SLREVRTIKVFTTVDNINLHTQVVDMSMTYGQQFGPTYLDGADVTKIKPHNSHEGKTFYV
EPI_ISL_424238	743	SLREVRTIKVFTTVDNINLHTQVVDMSMTYGQQFGPTYLDGADVTKIKPHNSHEGKTFYV
EPI_ISL_424248	743	SLREVRTIKVFTTVDNINLHTQVVDMSMTYGQQFGPTYLDGADVTKIKPHNSHEGKTFYV
EPI_ISL_424254	743	SLREVRTIKVFTTVDNINLHTQVVDMSMTYGQQFGPTYLDGADVTKIKPHNSHEGKTFYV
EPI_ISL_425548	743	SLREVRTIKVFTTVDNINLHTQVVDMSMTYGQQFGPTYLDGADVTKIKPHNSHEGKTFYV
EPI_ISL_425581	743	SLREVRTIKVFTTVDNINLHTQVVDMSMTYGQQFGPTYLDGADVTKIKPHNSHEGKTFYV
EPI_ISL_426881	743	SLREVRTIKVFTTVDNINLHTQVVDMSMTYGQQFGPTYLDGADVTKIKPHNSHEGKTFYV
EPI_ISL_426883	743	SLREVRTIKVFTTVDNINLHTQVVDMSMTYGQQFGPTYLDGADVTKIKPHNSHEGKTFYV
EPI_ISL_426888	743	SLREVRTIKVFTTVDNINLHTQVVDMSMTYGQQFGPTYLDGADVTKIKPHNSHEGKTFYV
EPI_ISL_426900	743	SLREVRTIKVFTTVDNINLHTQVVDMSMTYGQQFGPTYLDGADVTKIKPHNSHEGKTFYV
EPI_ISL_426901	743	SLREVRTIKVFTTVDNINLHTQVVDMSMTYGQQFGPTYLDGADVTKIKPHNSHEGKTFYV
EPI_ISL_426903	743	SLREVRTIKVFTTVDNINLHTQVVDMSMTYGQQFGPTYLDGADVTKIKPHNSHEGKTFYV
EPI_ISL_426904	743	SLREVRTIKVFTTVDNINLHTQVVDMSMTYGQQFGPTYLDGADVTKIKPHNSHEGKTFYV
EPI_ISL_426907	743	SLREVRTIKVFTTVDNINLHTQVVDMSMTYGQQFGPTYLDGADVTKIKPHNSHEGKTFYV
EPI_ISL_426910	743	SLREVRTIKVFTTVDNINLHTQVVDMSMTYGQQFGPTYLDGADVTKIKPHNSHEGKTFYV

```
ORF1ab          1621 LPNDDTLRVEAFEYYHTTDPSFLGRYMSALNHTKKWKYPQVNGLTSIKWADNNCYLATAL
EPI_ISL_413575   803 LPNDDTLRVEAFEYYHTTDPSFLGRYMSALNHTKKWKYPQVNGLTSIKWADNNCYLATAL
EPI_ISL_413588   803 LPNDDTLRVEAFEYYHTTDPSFLGRYMSALNHTKKWKYPQVNGLTSIKWADNNCYLATAL
EPI_ISL_413589   803 LPNDDTLRVEAFEYYHTTDPSFLGRYMSALNHTKKWKYPQVNGLTSIKWADNNCYLATAL
EPI_ISL_424221   803 LPNDDTLRVEAFEYYHTTDPSFLGRYMSALNHTKKWKYPQVNGLTSIKWADNNCYLATAL
EPI_ISL_424233   803 LPNDDTLRVEAFEYYHTTDPSFLGRYMSALNHTKKWKYPQVNGLTSIKWADNNCYLATAL
EPI_ISL_424236   803 LPNDDTLRVEAFEYYHTTDPSFLGRYMSALNHTKKWKYPQVNGLTSIKWADNNCYLATAL
EPI_ISL_424238   803 LPNDDTLRVEAFEYYHTTDPSFLGRYMSALNHTKKWKYPQVNGLTSIKWADNNCYLATAL
EPI_ISL_424248   803 LPNDDTLRVEAFEYYHTTDPSFLGRYMSALNHTKKWKYPQVNGLTSIKWADNNCYLATAL
EPI_ISL_424254   803 LPNDDTLRVEAFEYYHTTDPSFLGRYMSALNHTKKWKYPQVNGLTSIKWADNNCYLATAL
EPI_ISL_425548   803 LPNDDTLRVEAFEYYHTTDPSFLGRYMSALNHTKKWKYPQVNGLTSIKWADNNCYLATAL
EPI_ISL_425581   803 LPNDDTLRVEAFEYYHTTDPSFLGRYMSALNHTKKWKYPQVNGLTSIKWADNNCYLATAL
EPI_ISL_426881   803 LPNDDTLRVEAFEYYHTTDPSFLGRYMSALNHTKKWKYPQVNGLTSIKWADNNCYLATAL
EPI_ISL_426883   803 LPNDDTLRVEAFEYYHTTDPSFLGRYMSALNHTKKWKYPQVNGLTSIKWADNNCYLATAL
EPI_ISL_426888   803 LPNDDTLRVEAFEYYHTTDPSFLGRYMSALNHTKKWKYPQVNGLTSIKWADNNCYLATAL
EPI_ISL_426900   803 LPNDDTLRVEAFEYYHTTDPSFLGRYMSALNHTKKWKYPQVNGLTSIKWADNNCYLATAL
EPI_ISL_426901   803 LPNDDTLRVEAFEYYHTTDPSFLGRYMSALNHTKKWKYPQVNGLTSIKWADNNCYLATAL
EPI_ISL_426903   803 LPNDDTLRVEAFEYYHTTDPSFLGRYMSALNHTKKWKYPQVNGLTSIKWADNNCYLATAL
EPI_ISL_426904   803 LPNDDTLRVEAFEYYHTTDPSFLGRYMSALNHTKKWKYPQVNGLTSIKWADNNCYLATAL
EPI_ISL_426907   803 LPNDDTLRVEAFEYYHTTDPSFLGRYMSALNHTKKWKYPQVNGLTSIKWADNNCYLATAL
EPI_ISL_426910   803 LPNDDTLRVEAFEYYHTTDPSFLGRYMSALNHTKKWKYPQVNGLTSIKWADNNCYLATAL
```

```
ORF1ab          1621 LPNDDTLRVEAFEYYHTTDPSFLGRYMSALNHTKKWKYPQVNGLTSIKWADNNCYLATAL
EPI_ISL_413575   803 LPNDDTLRVEAFEYYHTTDPSFLGRYMSALNHTKKWKYPQVNGLTSIKWADNNCYLATAL
EPI_ISL_413588   803 LPNDDTLRVEAFEYYHTTDPSFLGRYMSALNHTKKWKYPQVNGLTSIKWADNNCYLATAL
EPI_ISL_413589   803 LPNDDTLRVEAFEYYHTTDPSFLGRYMSALNHTKKWKYPQVNGLTSIKWADNNCYLATAL
EPI_ISL_424221   803 LPNDDTLRVEAFEYYHTTDPSFLGRYMSALNHTKKWKYPQVNGLTSIKWADNNCYLATAL
EPI_ISL_424233   803 LPNDDTLRVEAFEYYHTTDPSFLGRYMSALNHTKKWKYPQVNGLTSIKWADNNCYLATAL
EPI_ISL_424236   803 LPNDDTLRVEAFEYYHTTDPSFLGRYMSALNHTKKWKYPQVNGLTSIKWADNNCYLATAL
EPI_ISL_424238   803 LPNDDTLRVEAFEYYHTTDPSFLGRYMSALNHTKKWKYPQVNGLTSIKWADNNCYLATAL
EPI_ISL_424248   803 LPNDDTLRVEAFEYYHTTDPSFLGRYMSALNHTKKWKYPQVNGLTSIKWADNNCYLATAL
EPI_ISL_424254   803 LPNDDTLRVEAFEYYHTTDPSFLGRYMSALNHTKKWKYPQVNGLTSIKWADNNCYLATAL
EPI_ISL_425548   803 LPNDDTLRVEAFEYYHTTDPSFLGRYMSALNHTKKWKYPQVNGLTSIKWADNNCYLATAL
EPI_ISL_425581   803 LPNDDTLRVEAFEYYHTTDPSFLGRYMSALNHTKKWKYPQVNGLTSIKWADNNCYLATAL
EPI_ISL_426881   803 LPNDDTLRVEAFEYYHTTDPSFLGRYMSALNHTKKWKYPQVNGLTSIKWADNNCYLATAL
EPI_ISL_426883   803 LPNDDTLRVEAFEYYHTTDPSFLGRYMSALNHTKKWKYPQVNGLTSIKWADNNCYLATAL
EPI_ISL_426888   803 LPNDDTLRVEAFEYYHTTDPSFLGRYMSALNHTKKWKYPQVNGLTSIKWADNNCYLATAL
EPI_ISL_426900   803 LPNDDTLRVEAFEYYHTTDPSFLGRYMSALNHTKKWKYPQVNGLTSIKWADNNCYLATAL
EPI_ISL_426901   803 LPNDDTLRVEAFEYYHTTDPSFLGRYMSALNHTKKWKYPQVNGLTSIKWADNNCYLATAL
EPI_ISL_426903   803 LPNDDTLRVEAFEYYHTTDPSFLGRYMSALNHTKKWKYPQVNGLTSIKWADNNCYLATAL
EPI_ISL_426904   803 LPNDDTLRVEAFEYYHTTDPSFLGRYMSALNHTKKWKYPQVNGLTSIKWADNNCYLATAL
EPI_ISL_426907   803 LPNDDTLRVEAFEYYHTTDPSFLGRYMSALNHTKKWKYPQVNGLTSIKWADNNCYLATAL
EPI_ISL_426910   803 LPNDDTLRVEAFEYYHTTDPSFLGRYMSALNHTKKWKYPQVNGLTSIKWADNNCYLATAL
```

ORF1ab	1681	LTLQQIELKFNPPALQDAYYRARAGEAANFCALILAYCNKTVGELGDVRETMSYLFQHAN
EPI_ISL_413575	863	LTLQQIELKFNPPALQDAYYRARAGEAANFCALILAYCNKTVGELGDVRETMSYLFQHAN
EPI_ISL_413588	863	LTLQQIELKFNPPALQDAYYRARAGEAANFCALILAYCNKTVGELGDVRETMSYLFQHAN
EPI_ISL_413589	863	LTLQQIELKFNPPALQDAYYRARAGEAANFCALILAYCNKTVGELGDVRETMSYLFQHAN
EPI_ISL_424221	863	LTLQQIELKFNPPALQDAYYRARAGEAANFCALILAYCNKTVGELGDVRETMSYLFQHAN
EPI_ISL_424233	863	LTLQQIELKFNPPALQDAYYRARAGEAANFCALILAYCNKTVGELGDVRETMSYLFQHAN
EPI_ISL_424236	863	LTLQQIELKFNPPALQDAYYRARAGEAANFCALILAYCNKTVGELGDVRETMSYLFQHAN
EPI_ISL_424238	863	LTLQQIELKFNPPALQDAYYRARAGEAANFCALILAYCNKTVGELGDVRETMSYLFQHAN
EPI_ISL_424248	863	LTLQQIELKFNPPALQDAYYRARAGEAANFCALILAYCNKTVGELGDVRETMSYLFQHAN
EPI_ISL_424254	863	LTLQQIELKFNPPALQDAYYRARAGEAANFCALILAYCNKTVGELGDVRETMSYLFQHAN
EPI_ISL_425548	863	LTLQQIELKFNPPALQDAYYRARAGEAANFCALILAYCNKTVGELGDVRETMSYLFQHAN
EPI_ISL_425581	863	LTLQQIELKFNPPALQDAYYRARAGEAANFCALILAYCNKTVGELGDVRETMSYLFQHAN
EPI_ISL_426881	863	LTLQQIELKFNPPALQDAYYRARAGEAANFCALILAYCNKTVGELGDVRETMSYLFQHAN
EPI_ISL_426883	863	LTLQQIELKFNPPALQDAYYRARAGEAANFCALILAYCNKTVGELGDVRETMSYLFQHAN
EPI_ISL_426888	863	LTLQQIELKFNPPALQDAYYRARAGEAANFCALILAYCNKTVGELGDVRETMSYLFQHAN
EPI_ISL_426900	863	LTLQQIELKFNPPALQDAYYRARAGEAANFCALILAYCNKTVGELGDVRETMSYLFQHAN
EPI_ISL_426901	863	LTLQQIELKFNPPALQDAYYRARAGEAANFCALILAYCNKTVGELGDVRETMSYLFQHAN
EPI_ISL_426903	863	LTLQQIELKFNPPALQDAYYRARAGEAANFCALILAYCNKTVGELGDVRETMSYLFQHAN
EPI_ISL_426904	863	LTLQQIELKFNPPALQDAYYRARAGEAANFCALILAYCNKTVGELGDVRETMSYLFQHAN
EPI_ISL_426907	863	LTLQQIELKFNPPALQDAYYRARAGEAANFCALILAYCNKTVGELGDVRETMSYLFQHAN
EPI_ISL_426910	863	LTLQQIELKFNPPALQDAYYRARAGEAANFCALILAYCNKTVGELGDVRETMSYLFQHAN
ORF1ab	1681	LTLQQIELKFNPPALQDAYYRARAGEAANFCALILAYCNKTVGELGDVRETMSYLFQHAN
EPI_ISL_413575	863	LTLQQIELKFNPPALQDAYYRARAGEAANFCALILAYCNKTVGELGDVRETMSYLFQHAN
EPI_ISL_413588	863	LTLQQIELKFNPPALQDAYYRARAGEAANFCALILAYCNKTVGELGDVRETMSYLFQHAN
EPI_ISL_413589	863	LTLQQIELKFNPPALQDAYYRARAGEAANFCALILAYCNKTVGELGDVRETMSYLFQHAN
EPI_ISL_424221	863	LTLQQIELKFNPPALQDAYYRARAGEAANFCALILAYCNKTVGELGDVRETMSYLFQHAN
EPI_ISL_424233	863	LTLQQIELKFNPPALQDAYYRARAGEAANFCALILAYCNKTVGELGDVRETMSYLFQHAN
EPI_ISL_424236	863	LTLQQIELKFNPPALQDAYYRARAGEAANFCALILAYCNKTVGELGDVRETMSYLFQHAN
EPI_ISL_424238	863	LTLQQIELKFNPPALQDAYYRARAGEAANFCALILAYCNKTVGELGDVRETMSYLFQHAN
EPI_ISL_424248	863	LTLQQIELKFNPPALQDAYYRARAGEAANFCALILAYCNKTVGELGDVRETMSYLFQHAN
EPI_ISL_424254	863	LTLQQIELKFNPPALQDAYYRARAGEAANFCALILAYCNKTVGELGDVRETMSYLFQHAN
EPI_ISL_425548	863	LTLQQIELKFNPPALQDAYYRARAGEAANFCALILAYCNKTVGELGDVRETMSYLFQHAN
EPI_ISL_425581	863	LTLQQIELKFNPPALQDAYYRARAGEAANFCALILAYCNKTVGELGDVRETMSYLFQHAN
EPI_ISL_426881	863	LTLQQIELKFNPPALQDAYYRARAGEAANFCALILAYCNKTVGELGDVRETMSYLFQHAN
EPI_ISL_426883	863	LTLQQIELKFNPPALQDAYYRARAGEAANFCALILAYCNKTVGELGDVRETMSYLFQHAN
EPI_ISL_426888	863	LTLQQIELKFNPPALQDAYYRARAGEAANFCALILAYCNKTVGELGDVRETMSYLFQHAN
EPI_ISL_426900	863	LTLQQIELKFNPPALQDAYYRARAGEAANFCALILAYCNKTVGELGDVRETMSYLFQHAN
EPI_ISL_426901	863	LTLQQIELKFNPPALQDAYYRARAGEAANFCALILAYCNKTVGELGDVRETMSYLFQHAN
EPI_ISL_426903	863	LTLQQIELKFNPPALQDAYYRARAGEAANFCALILAYCNKTVGELGDVRETMSYLFQHAN
EPI_ISL_426904	863	LTLQQIELKFNPPALQDAYYRARAGEAANFCALILAYCNKTVGELGDVRETMSYLFQHAN
EPI_ISL_426907	863	LTLQQIELKFNPPALQDAYYRARAGEAANFCALILAYCNKTVGELGDVRETMSYLFQHAN
EPI_ISL_426910	863	LTLQQIELKFNPPALQDAYYRARAGEAANFCALILAYCNKTVGELGDVRETMSYLFQHAN

```
ORF1ab          1741 LDSCKRVLNVVCKTCGQQQTTLKGVEAVMYMGTLSYEQFKKGVQIPCTCGKQATKYLVQQ
EPI_ISL_413575   923 LDSCKRVLNVVCKTCGQQQTTLKGVEAVMYMGTLSYEQFKKGVQIPCTCGKQATKYLVQQ
EPI_ISL_413588   923 LDSCKRVLNVVCKTCGQQQTTLKGVEAVMYMGTLSYEQFKKGVQIPCTCGKQATKYLVQQ
EPI_ISL_413589   923 LDSCKRVLNVVCKTCGQQQTTLKGVEAVMYMGTLSYEQFKKGVQIPCTCGKQATKYLVQQ
EPI_ISL_424221   923 LDSCKRVLNVVCKTCGQQQTTLKGVEAVMYMGTLSYEQFKKGVQIPCTCGKQATKYLVQQ
EPI_ISL_424233   923 LDSCKRVLNVVCKTCGQQQTTLKGVEAVMYMGTLSYEQFKKGVQIPCTCGKQATKYLVQQ
EPI_ISL_424236   923 LDSCKRVLNVVCKTCGQQQTTLKGVEAVMYMGTLSYEQFKKGVQIPCTCGKQATKYLVQQ
EPI_ISL_424238   923 LDSCKRVLNVVCKTCGQQQTTLKGVEAVMYMGTLSYEQFKKGVQIPCTCGKQATKYLVQQ
EPI_ISL_424248   923 LDSCKRVLNVVCKTCGQQQTTLKGVEAVMYMGTLSYEQFKKGVQIPCTCGKQATKYLVQQ
EPI_ISL_424254   923 LDSCKRVLNVVCKTCGQQQTTLKGVEAVMYMGTLSYEQFKKGVQIPCTCGKQATKYLVQQ
EPI_ISL_425548   923 LDSCKRVLNVVCKTCGQQQTTLKGVEAVMYMGTLSYEQFKKGVQIPCTCGKQATKYLVQQ
EPI_ISL_425581   923 LDSCKRVLNVVCKTCGQQQTTLKGVEAVMYMGTLSYEQFKKGVQIPCTCGKQATKYLVQQ
EPI_ISL_426881   923 LDSCKRVLNVVCKTCGQQQTTLKGVEAVMYMGTLSYEQFKKGVQIPCTCGKQATKYLVQQ
EPI_ISL_426883   923 LDSCKRVLNVVCKTCGQQQTTLKGVEAVMYMGTLSYEQFKKGVQIPCTCGKQATKYLVQQ
EPI_ISL_426888   923 LDSCKRVLNVVCKTCGQQQTTLKGVEAVMYMGTLSYEQFKKGVQIPCTCGKQATKYLVQQ
EPI_ISL_426900   923 LDSCKRVLNVVCKTCGQQQTTLKGVEAVMYMGTLSYEQFKKGVQIPCTCGKQATKYLVQQ
EPI_ISL_426901   923 LDSCKRVLNVVCKTCGQQQTTLKGVEAVMYMGTLSYEQFKKGVQIPCTCGKQATKYLVQQ
EPI_ISL_426903   923 LDSCKRVLNVVCKTCGQQQTTLKGVEAVMYMGTLSYEQFKKGVQIPCTCGKQATKYLVQQ
EPI_ISL_426904   923 LDSCKRVLNVVCKTCGQQQTTLKGVEAVMYMGTLSYEQFKKGVQIPCTCGKQATKYLVQQ
EPI_ISL_426907   923 LDSCKRVLNVVCKTCGQQQTTLKGVEAVMYMGTLSYEQFKKGVQIPCTCGKQATKYLVQQ
EPI_ISL_426910   923 LDSCKRVLNVVCKTCGQQQTTLKGVEAVMYMGTLSYEQFKKGVQIPCTCGKQATKYLVQQ
ORF1ab          1741 LDSCKRVLNVVCKTCGQQQTTLKGVEAVMYMGTLSYEQFKKGVQIPCTCGKQATKYLVQQ
EPI_ISL_413575   923 LDSCKRVLNVVCKTCGQQQTTLKGVEAVMYMGTLSYEQFKKGVQIPCTCGKQATKYLVQQ
EPI_ISL_413588   923 LDSCKRVLNVVCKTCGQQQTTLKGVEAVMYMGTLSYEQFKKGVQIPCTCGKQATKYLVQQ
EPI_ISL_413589   923 LDSCKRVLNVVCKTCGQQQTTLKGVEAVMYMGTLSYEQFKKGVQIPCTCGKQATKYLVQQ
EPI_ISL_424221   923 LDSCKRVLNVVCKTCGQQQTTLKGVEAVMYMGTLSYEQFKKGVQIPCTCGKQATKYLVQQ
EPI_ISL_424233   923 LDSCKRVLNVVCKTCGQQQTTLKGVEAVMYMGTLSYEQFKKGVQIPCTCGKQATKYLVQQ
EPI_ISL_424236   923 LDSCKRVLNVVCKTCGQQQTTLKGVEAVMYMGTLSYEQFKKGVQIPCTCGKQATKYLVQQ
EPI_ISL_424238   923 LDSCKRVLNVVCKTCGQQQTTLKGVEAVMYMGTLSYEQFKKGVQIPCTCGKQATKYLVQQ
EPI_ISL_424248   923 LDSCKRVLNVVCKTCGQQQTTLKGVEAVMYMGTLSYEQFKKGVQIPCTCGKQATKYLVQQ
EPI_ISL_424254   923 LDSCKRVLNVVCKTCGQQQTTLKGVEAVMYMGTLSYEQFKKGVQIPCTCGKQATKYLVQQ
EPI_ISL_425548   923 LDSCKRVLNVVCKTCGQQQTTLKGVEAVMYMGTLSYEQFKKGVQIPCTCGKQATKYLVQQ
EPI_ISL_425581   923 LDSCKRVLNVVCKTCGQQQTTLKGVEAVMYMGTLSYEQFKKGVQIPCTCGKQATKYLVQQ
EPI_ISL_426881   923 LDSCKRVLNVVCKTCGQQQTTLKGVEAVMYMGTLSYEQFKKGVQIPCTCGKQATKYLVQQ
EPI_ISL_426883   923 LDSCKRVLNVVCKTCGQQQTTLKGVEAVMYMGTLSYEQFKKGVQIPCTCGKQATKYLVQQ
EPI_ISL_426888   923 LDSCKRVLNVVCKTCGQQQTTLKGVEAVMYMGTLSYEQFKKGVQIPCTCGKQATKYLVQQ
EPI_ISL_426900   923 LDSCKRVLNVVCKTCGQQQTTLKGVEAVMYMGTLSYEQFKKGVQIPCTCGKQATKYLVQQ
EPI_ISL_426901   923 LDSCKRVLNVVCKTCGQQQTTLKGVEAVMYMGTLSYEQFKKGVQIPCTCGKQATKYLVQQ
EPI_ISL_426903   923 LDSCKRVLNVVCKTCGQQQTTLKGVEAVMYMGTLSYEQFKKGVQIPCTCGKQATKYLVQQ
EPI_ISL_426904   923 LDSCKRVLNVVCKTCGQQQTTLKGVEAVMYMGTLSYEQFKKGVQIPCTCGKQATKYLVQQ
EPI_ISL_426907   923 LDSCKRVLNVVCKTCGQQQTTLKGVEAVMYMGTLSYEQFKKGVQIPCTCGKQATKYLVQQ
EPI_ISL_426910   923 LDSCKRVLNVVCKTCGQQQTTLKGVEAVMYMGTLSYEQFKKGVQIPCTCGKQATKYLVQQ
```

ORF1ab	1801	ESPFVMMSAPPAQYELKHGTFTCASEYTGNYQCGHYKHITSKETLYCIDGALLTKSSEYK
EPI_ISL_413575	983	ESPFVMMSAPPAQYELKHGTFTCASEYTGNYQCGHYKHITSKETLYCIDGALLTKSSEYK
EPI_ISL_413588	983	ESPFVMMSAPPAQYELKHGTFTCASEYTGNYQCGHYKHITSKETLYCIDGALLTKSSEYK
EPI_ISL_413589	983	ESPFVMMSAPPAQYELKHGTFTCASEYTGNYQCGHYKHITSKETLYCIDGALLTKSSEYK
EPI_ISL_424221	983	ESPFVMMSAPPAQYELKHGTFTCASEYTGNYQCGHYKHITSKETLYCIDGALLTKSSEYK
EPI_ISL_424233	983	ESPFVMMSAPPAQYELKHGTFTCASEYTGNYQCGHYKHITSKETLYCIDGALLTKSSEYK
EPI_ISL_424236	983	ESPFVMMSAPPAQYELKHGTFTCASEYTGNYQCGHYKHITSKETLYCIDGALLTKSSEYK
EPI_ISL_424238	983	ESPFVMMSAPPAQYELKHGTFTCASEYTGNYQCGHYKHITSKETLYCIDGALLTKSSEYK
EPI_ISL_424248	983	ESPFVMMSAPPAQYELKHGTFTCASEYTGNYQCGHYKHITSKETLYCIDGALLTKSSEYK
EPI_ISL_424254	983	ESPFVMMSAPPAQYELKHGTFTCASEYTGNYQCGHYKHITSKETLYCIDGALLTKSSEYK
EPI_ISL_425548	983	ESPFVMMSAPPAQYELKHGTFTCASEYTGNYQCGHYKHITSKETLYCIDGALLTKSSEYK
EPI_ISL_425581	983	ESPFVMMSAPPAQYELKHGTFTCASEYTGNYQCGHYKHITSKETLYCIDGALLTKSSEYK
EPI_ISL_426881	983	ESPFVMMSAPPAQYELKHGTFTCASEYTGNYQCGHYKHITSKETLYCIDGALLTKSSEYK
EPI_ISL_426883	983	ESPFVMMSAPPAQYELKHGTFTCASEYTGNYQCGHYKHITSKETLYCIDGALLTKSSEYK
EPI_ISL_426888	983	ESPFVMMSAPPAQYELKHGTFTCASEYTGNYQCGHYKHITSKETLYCIDGALLTKSSEYK
EPI_ISL_426900	983	ESPFVMMSAPPAQYELKHGTFTCASEYTGNYQCGHYKHITSKETLYCIDGALLTKSSEYK
EPI_ISL_426901	983	ESPFVMMSAPPAQYELKHGTFTCASEYTGNYQCGHYKHITSKETLYCIDGALLTKSSEYK
EPI_ISL_426903	983	ESPFVMMSAPPAQYELKHGTFTCASEYTGNYQCGHYKHITSKETLYCIDGALLTKSSEYK
EPI_ISL_426904	983	ESPFVMMSAPPAQYELKHGTFTCASEYTGNYQCGHYKHITSKETLYCIDGALLTKSSEYK
EPI_ISL_426907	983	ESPFVMMSAPPAQYELKHGTFTCASEYTGNYQCGHYKHITSKETLYCIDGALLTKSSEYK
EPI_ISL_426910	983	ESPFVMMSAPPAQYELKHGTFTCASEYTGNYQCGHYKHITSKETLYCIDGALLTKSSEYK
ORF1ab	1801	ESPFVMMSAPPAQYELKHGTFTCASEYTGNYQCGHYKHITSKETLYCIDGALLTKSSEYK
EPI_ISL_413575	983	ESPFVMMSAPPAQYELKHGTFTCASEYTGNYQCGHYKHITSKETLYCIDGALLTKSSEYK
EPI_ISL_413588	983	ESPFVMMSAPPAQYELKHGTFTCASEYTGNYQCGHYKHITSKETLYCIDGALLTKSSEYK
EPI_ISL_413589	983	ESPFVMMSAPPAQYELKHGTFTCASEYTGNYQCGHYKHITSKETLYCIDGALLTKSSEYK
EPI_ISL_424221	983	ESPFVMMSAPPAQYELKHGTFTCASEYTGNYQCGHYKHITSKETLYCIDGALLTKSSEYK
EPI_ISL_424233	983	ESPFVMMSAPPAQYELKHGTFTCASEYTGNYQCGHYKHITSKETLYCIDGALLTKSSEYK
EPI_ISL_424236	983	ESPFVMMSAPPAQYELKHGTFTCASEYTGNYQCGHYKHITSKETLYCIDGALLTKSSEYK
EPI_ISL_424238	983	ESPFVMMSAPPAQYELKHGTFTCASEYTGNYQCGHYKHITSKETLYCIDGALLTKSSEYK
EPI_ISL_424248	983	ESPFVMMSAPPAQYELKHGTFTCASEYTGNYQCGHYKHITSKETLYCIDGALLTKSSEYK
EPI_ISL_424254	983	ESPFVMMSAPPAQYELKHGTFTCASEYTGNYQCGHYKHITSKETLYCIDGALLTKSSEYK
EPI_ISL_425548	983	ESPFVMMSAPPAQYELKHGTFTCASEYTGNYQCGHYKHITSKETLYCIDGALLTKSSEYK
EPI_ISL_425581	983	ESPFVMMSAPPAQYELKHGTFTCASEYTGNYQCGHYKHITSKETLYCIDGALLTKSSEYK
EPI_ISL_426881	983	ESPFVMMSAPPAQYELKHGTFTCASEYTGNYQCGHYKHITSKETLYCIDGALLTKSSEYK
EPI_ISL_426883	983	ESPFVMMSAPPAQYELKHGTFTCASEYTGNYQCGHYKHITSKETLYCIDGALLTKSSEYK
EPI_ISL_426888	983	ESPFVMMSAPPAQYELKHGTFTCASEYTGNYQCGHYKHITSKETLYCIDGALLTKSSEYK
EPI_ISL_426900	983	ESPFVMMSAPPAQYELKHGTFTCASEYTGNYQCGHYKHITSKETLYCIDGALLTKSSEYK
EPI_ISL_426901	983	ESPFVMMSAPPAQYELKHGTFTCASEYTGNYQCGHYKHITSKETLYCIDGALLTKSSEYK
EPI_ISL_426903	983	ESPFVMMSAPPAQYELKHGTFTCASEYTGNYQCGHYKHITSKETLYCIDGALLTKSSEYK
EPI_ISL_426904	983	ESPFVMMSAPPAQYELKHGTFTCASEYTGNYQCGHYKHITSKETLYCIDGALLTKSSEYK
EPI_ISL_426907	983	ESPFVMMSAPPAQYELKHGTFTCASEYTGNYQCGHYKHITSKETLYCIDGALLTKSSEYK
EPI_ISL_426910	983	ESPFVMMSAPPAQYELKHGTFTCASEYTGNYQCGHYKHITSKETLYCIDGALLTKSSEYK

ORF1ab	1861	GPITDVFYKENSYTTTIKPVTYKLDGVVCTEIDPKLDNYYKKDNSYFTEQPIDLVPNQPY
EPI_ISL_413575	1043	GPITDVFYKENSYTTTIKPVTYKLDGVVCTEIDPKLDNYYKKDNSYFTEQPIDLVPNQPY
EPI_ISL_413588	1043	GPITDVFYKENSYTTTIKPVTYKLDGVVCTEIDPKLDNYYKKDNSYFTEQPIDLVPNQPY
EPI_ISL_413589	1043	GPITDVFYKENSYTTTIKPVTYKLDGVVCTEIDPKLDNYYKKDNSYFTEQPIDLVPNQPY
EPI_ISL_424221	1043	GPITDVFYKENSYTTTIKPVTYKLDGVVCTEIDPKLDNYYKKDNSYFTEQPIDLVPNQPY
EPI_ISL_424233	1043	GPITDVFYKENSYTTTIKPVTYKLDGVVCTEIDPKLDNYYKKDNSYFTEQPIDLVPNQPY
EPI_ISL_424236	1043	GPITDVFYKENSYTTTIKPVTYKLDGVVCTEIDPKLDNYYKKDNSYFTEQPIDLVPNQPY
EPI_ISL_424238	1043	GPITDVFYKENSYTTTIKPVTYKLDGVVCTEIDPKLDNYYKKDNSYFTEQPIDLVPNQPY
EPI_ISL_424248	1043	GPITDVFYKENSYTTTIKPVTYKLDGVVCTEIDPKLDNYYKKDNSYFTEQPIDLVPNQPY
EPI_ISL_424254	1043	GPITDVFYKENSYTTTIKPVTYKLDGVVCTEIDPKLDNYYKKDNSYFTEQPIDLVPNQPY
EPI_ISL_425548	1043	GPITDVFYKENSYTTTIKPVTYKLDGVVCTEIDPKLDNYYKKDNSYFTEQPIDLVPNQPY
EPI_ISL_425581	1043	GPITDVFYKENSYTTTIKPVTYKLDGVVCTEIDPKLDNYYKKDNSYFTEQPIDLVPNQPY
EPI_ISL_426881	1043	GPITDVFYKENSYTTTIKPVTYKLDGVVCTEIDPKLDNYYKKDNSYFTEQPIDLVPNQPY
EPI_ISL_426883	1043	GPITDVFYKENSYTTTIKPVTYKLDGVVCTEIDPKLDNYYKKDNSYFTEQPIDLVPNQPY
EPI_ISL_426888	1043	GPITDVFYKENSYTTTIKPVTYKLDGVVCTEIDPKLDNYYKKDNSYFTEQPIDLVPNQPY
EPI_ISL_426900	1043	GPITDVFYKENSYTTTIKPVTYKLDGVVCTEIDPKLDNYYKKDNSYFTEQPIDLVPNQPY
EPI_ISL_426901	1043	GPITDVFYKENSYTTTIKPVTYKLDGVVCTEIDPKLDNYYKKDNSYFTEQPIDLVPNQPY
EPI_ISL_426903	1043	GPITDVFYKENSYTTTIKPVTYKLDGVVCTEIDPKLDNYYKKDNSYFTEQPIDLVPNQPY
EPI_ISL_426904	1043	GPITDVFYKENSYTTTIKPVTYKLDGVVCTEIDPKLDNYYKKDNSYFTEQPIDLVPNQPY
EPI_ISL_426907	1043	GPITDVFYKENSYTTTIKPVTYKLDGVVCTEIDPKLDNYYKKDNSYFTEQPIDLVPNQPY
EPI_ISL_426910	1043	GPITDVFYKENSYTTTIKPVTYKLDGVVCTEIDPKLDNYYKKDNSYFTEQPIDLVPNQPY
ORF1ab	1861	GPITDVFYKENSYTTTIKPVTYKLDGVVCTEIDPKLDNYYKKDNSYFTEQPIDLVPNQPY
EPI_ISL_413575	1043	GPITDVFYKENSYTTTIKPVTYKLDGVVCTEIDPKLDNYYKKDNSYFTEQPIDLVPNQPY
EPI_ISL_413588	1043	GPITDVFYKENSYTTTIKPVTYKLDGVVCTEIDPKLDNYYKKDNSYFTEQPIDLVPNQPY
EPI_ISL_413589	1043	GPITDVFYKENSYTTTIKPVTYKLDGVVCTEIDPKLDNYYKKDNSYFTEQPIDLVPNQPY
EPI_ISL_424221	1043	GPITDVFYKENSYTTTIKPVTYKLDGVVCTEIDPKLDNYYKKDNSYFTEQPIDLVPNQPY
EPI_ISL_424233	1043	GPITDVFYKENSYTTTIKPVTYKLDGVVCTEIDPKLDNYYKKDNSYFTEQPIDLVPNQPY
EPI_ISL_424236	1043	GPITDVFYKENSYTTTIKPVTYKLDGVVCTEIDPKLDNYYKKDNSYFTEQPIDLVPNQPY
EPI_ISL_424238	1043	GPITDVFYKENSYTTTIKPVTYKLDGVVCTEIDPKLDNYYKKDNSYFTEQPIDLVPNQPY
EPI_ISL_424248	1043	GPITDVFYKENSYTTTIKPVTYKLDGVVCTEIDPKLDNYYKKDNSYFTEQPIDLVPNQPY
EPI_ISL_424254	1043	GPITDVFYKENSYTTTIKPVTYKLDGVVCTEIDPKLDNYYKKDNSYFTEQPIDLVPNQPY
EPI_ISL_425548	1043	GPITDVFYKENSYTTTIKPVTYKLDGVVCTEIDPKLDNYYKKDNSYFTEQPIDLVPNQPY
EPI_ISL_425581	1043	GPITDVFYKENSYTTTIKPVTYKLDGVVCTEIDPKLDNYYKKDNSYFTEQPIDLVPNQPY
EPI_ISL_426881	1043	GPITDVFYKENSYTTTIKPVTYKLDGVVCTEIDPKLDNYYKKDNSYFTEQPIDLVPNQPY
EPI_ISL_426883	1043	GPITDVFYKENSYTTTIKPVTYKLDGVVCTEIDPKLDNYYKKDNSYFTEQPIDLVPNQPY
EPI_ISL_426888	1043	GPITDVFYKENSYTTTIKPVTYKLDGVVCTEIDPKLDNYYKKDNSYFTEQPIDLVPNQPY
EPI_ISL_426900	1043	GPITDVFYKENSYTTTIKPVTYKLDGVVCTEIDPKLDNYYKKDNSYFTEQPIDLVPNQPY
EPI_ISL_426901	1043	GPITDVFYKENSYTTTIKPVTYKLDGVVCTEIDPKLDNYYKKDNSYFTEQPIDLVPNQPY
EPI_ISL_426903	1043	GPITDVFYKENSYTTTIKPVTYKLDGVVCTEIDPKLDNYYKKDNSYFTEQPIDLVPNQPY
EPI_ISL_426904	1043	GPITDVFYKENSYTTTIKPVTYKLDGVVCTEIDPKLDNYYKKDNSYFTEQPIDLVPNQPY
EPI_ISL_426907	1043	GPITDVFYKENSYTTTIKPVTYKLDGVVCTEIDPKLDNYYKKDNSYFTEQPIDLVPNQPY
EPI_ISL_426910	1043	GPITDVFYKENSYTTTIKPVTYKLDGVVCTEIDPKLDNYYKKDNSYFTEQPIDLVPNQPY

```
ORF1ab         1921 PNASFDNFKFVCDNIKFADDLNQLTGYKKPASRELKVTFFPDLNGDVVAIDYKHYTPSFK
EPI_ISL_413575 1103 PNASFDNFKFVCDNIKFADDLNQLTGYKKPASRELKVTFFPDLNGDVVAIDYKHYTPSFK
EPI_ISL_413588 1103 PNASFDNFKFVCDNIKFADDLNQLTGYKKPASRELKVTFFPDLNGDVVAIDYKHYTPSFK
EPI_ISL_413589 1103 PNASFDNFKFVCDNIKFADDLNQLTGYKKPASRELKVTFFPDLNGDVVAIDYKHYTPSFK
EPI_ISL_424221 1103 PNASFDNFKFVCDNIKFADDLNQLTGYKKPASRELKVTFFPDLNGDVVAIDYKHYTPSFK
EPI_ISL_424233 1103 PNASFDNFKFVCDNIKFADDLNQLTGYKKPASRELKVTFFPDLNGDVVAIDYKHYTPSFK
EPI_ISL_424236 1103 PNASFDNFKFVCDNIKFADDLNQLTGYKKPASRELKVTFFPDLNGDVVAIDYKHYTPSFK
EPI_ISL_424238 1103 PNASFDNFKFVCDNIKFADDLNQLTGYKKPASRELKVTFFPDLNGDVVAIDYKHYTPSFK
EPI_ISL_424248 1103 PNASFDNFKFVCDNIKFADDLNQLTGYKKPASRELKVTFFPDLNGDVVAIDYKHYTPSFK
EPI_ISL_424254 1103 PNASFDNFKFVCDNIKFADDLNQLTGYKKPASRELKVTFFPDLNGDVVAIDYKHYTPSFK
EPI_ISL_425548 1103 PNASFDNFKFVCDNIKFADDLNQLTGYKKPASRELKVTFFPDLNGDVVAIDYKHYTPSFK
EPI_ISL_425581 1103 PNASFDNFKFVCDNIKFADDLNQLTGYKKPASRELKVTFFPDLNGDVVAIDYKHYTPSFK
EPI_ISL_426881 1103 PNASFDNFKFVCDNIKFADDLNQLTGYKKPASRELKVTFFPDLNGDVVAIDYKHYTPSFK
EPI_ISL_426883 1103 PNASFDNFKFVCDNIKFADDLNQLTGYKKPASRELKVTFFPDLNGDVVAIDYKHYTPSFK
EPI_ISL_426888 1103 PNASFDNFKFVCDNIKFADDLNQLTGYKKPASRELKVTFFPDLNGDVVAIDYKHYTPSFK
EPI_ISL_426900 1103 PNASFDNFKFVCDNIKFADDLNQLTGYKKPASRELKVTFFPDLNGDVVAIDYKHYTPSFK
EPI_ISL_426901 1103 PNASFDNFKFVCDNIKFADDLNQLTGYKKPASRELKVTFFPDLNGDVVAIDYKHYTPSFK
EPI_ISL_426903 1103 PNASFDNFKFVCDNIKFADDLNQLTGYKKPASRELKVTFFPDLNGDVVAIDYKHYTPSFK
EPI_ISL_426904 1103 PNASFDNFKFVCDNIKFADDLNQLTGYKKPASRELKVTFFPDLNGDVVAIDYKHYTPSFK
EPI_ISL_426907 1103 PNASFDNFKFVCDNIKFADDLNQLTGYKKPASRELKVTFFPDLNGDVVAIDYKHYTPSFK
EPI_ISL_426910 1103 PNASFDNFKFVCDNIKFADDLNQLTGYKKPASRELKVTFFPDLNGDVVAIDYKHYTPSFK
ORF1ab         1921 PNASFDNFKFVCDNIKFADDLNQLTGYKKPASRELKVTFFPDLNGDVVAIDYKHYTPSFK
EPI_ISL_413575 1103 PNASFDNFKFVCDNIKFADDLNQLTGYKKPASRELKVTFFPDLNGDVVAIDYKHYTPSFK
EPI_ISL_413588 1103 PNASFDNFKFVCDNIKFADDLNQLTGYKKPASRELKVTFFPDLNGDVVAIDYKHYTPSFK
EPI_ISL_413589 1103 PNASFDNFKFVCDNIKFADDLNQLTGYKKPASRELKVTFFPDLNGDVVAIDYKHYTPSFK
EPI_ISL_424221 1103 PNASFDNFKFVCDNIKFADDLNQLTGYKKPASRELKVTFFPDLNGDVVAIDYKHYTPSFK
EPI_ISL_424233 1103 PNASFDNFKFVCDNIKFADDLNQLTGYKKPASRELKVTFFPDLNGDVVAIDYKHYTPSFK
EPI_ISL_424236 1103 PNASFDNFKFVCDNIKFADDLNQLTGYKKPASRELKVTFFPDLNGDVVAIDYKHYTPSFK
EPI_ISL_424238 1103 PNASFDNFKFVCDNIKFADDLNQLTGYKKPASRELKVTFFPDLNGDVVAIDYKHYTPSFK
EPI_ISL_424248 1103 PNASFDNFKFVCDNIKFADDLNQLTGYKKPASRELKVTFFPDLNGDVVAIDYKHYTPSFK
EPI_ISL_424254 1103 PNASFDNFKFVCDNIKFADDLNQLTGYKKPASRELKVTFFPDLNGDVVAIDYKHYTPSFK
EPI_ISL_425548 1103 PNASFDNFKFVCDNIKFADDLNQLTGYKKPASRELKVTFFPDLNGDVVAIDYKHYTPSFK
EPI_ISL_425581 1103 PNASFDNFKFVCDNIKFADDLNQLTGYKKPASRELKVTFFPDLNGDVVAIDYKHYTPSFK
EPI_ISL_426881 1103 PNASFDNFKFVCDNIKFADDLNQLTGYKKPASRELKVTFFPDLNGDVVAIDYKHYTPSFK
EPI_ISL_426883 1103 PNASFDNFKFVCDNIKFADDLNQLTGYKKPASRELKVTFFPDLNGDVVAIDYKHYTPSFK
EPI_ISL_426888 1103 PNASFDNFKFVCDNIKFADDLNQLTGYKKPASRELKVTFFPDLNGDVVAIDYKHYTPSFK
EPI_ISL_426900 1103 PNASFDNFKFVCDNIKFADDLNQLTGYKKPASRELKVTFFPDLNGDVVAIDYKHYTPSFK
EPI_ISL_426901 1103 PNASFDNFKFVCDNIKFADDLNQLTGYKKPASRELKVTFFPDLNGDVVAIDYKHYTPSFK
EPI_ISL_426903 1103 PNASFDNFKFVCDNIKFADDLNQLTGYKKPASRELKVTFFPDLNGDVVAIDYKHYTPSFK
EPI_ISL_426904 1103 PNASFDNFKFVCDNIKFADDLNQLTGYKKPASRELKVTFFPDLNGDVVAIDYKHYTPSFK
EPI_ISL_426907 1103 PNASFDNFKFVCDNIKFADDLNQLTGYKKPASRELKVTFFPDLNGDVVAIDYKHYTPSFK
EPI_ISL_426910 1103 PNASFDNFKFVCDNIKFADDLNQLTGYKKPASRELKVTFFPDLNGDVVAIDYKHYTPSFK
```

```
ORF1ab         1981 KGAKLLHKPIVWHVNNATNKATYKPNTWCIRCLWSTKPVETSNSFDVLKSEDAQGMDNLA
EPI_ISL_413575 1163 KGAKLLHKPIVWHVNNATNKATYKPNTWCIRCLWSTKPVETSNSFDVLKSEDAQGMDNLA
EPI_ISL_413588 1163 KGAKLLHKPIVWHVNNATNKATYKPNTWCIRCLWSTKPVETSNSFDVLKSEDAQGMDNLA
EPI_ISL_413589 1163 KGAKLLHKPIVWHVNNATNKATYKPNTWCIRCLWSTKPVETSNSFDVLKSEDAQGMDNLA
EPI_ISL_424221 1163 KGAKLLHKPIVWHVNNATNKATYKPNTWCIRCLWSTKPVETSNSFDVLKSEDAQGMDNLA
EPI_ISL_424233 1163 KGAKLLHKPIVWHVNNATNKATYKPNTWCIRCLWSTKPVETSNSFDVLKSEDAQGMDNLA
EPI_ISL_424236 1163 KGAKLLHKPIVWHVNNATNKATYKPNTWCIRCLWSTKPVETSNSFDVLKSEDAQGMDNLA
EPI_ISL_424238 1163 KGAKLLHKPIVWHVNNATNKATYKPNTWCIRCLWSTKPVETSNSFDVLKSEDAQGMDNLA
EPI_ISL_424248 1163 KGAKLLHKPIVWHVNNATNKATYKPNTWCIRCLWSTKPVETSNSFDVLKSEDAQGMDNLA
EPI_ISL_424254 1163 KGAKLLHKPIVWHVNNATNKATYKPNTWCIRCLWSTKPVETSNSFDVLKSEDAQGMDNLA
EPI_ISL_425548 1163 KGAKLLHKPIVWHVNNATNKATYKPNTWCIRCLWSTKPVETSNSFDVLKSEDAQGMDNLA
EPI_ISL_425581 1163 KGAKLLHKPIVWHVNNATNKATYKPNTWCIRCLWSTKPVETSNSFDVLKSEDAQGMDNLA
EPI_ISL_426881 1163 KGAKLLHKPIVWHVNNATNKATYKPNTWCIRCLWSTKPVETSNSFDVLKSEDAQGMDNLA
EPI_ISL_426883 1163 KGAKLLHKPIVWHVNNATNKATYKPNTWCIRCLWSTKPVETSNSFDVLKSEDAQGMDNLA
EPI_ISL_426888 1163 KGAKLLHKPIVWHVNNATNKATYKPNTWCIRCLWSTKPVETSNSFDVLKSEDAQGMDNLA
EPI_ISL_426900 1163 KGAKLLHKPIVWHVNNATNKATYKPNTWCIRCLWSTKPVETSNSFDVLKSEDAQGMDNLA
EPI_ISL_426901 1163 KGAKLLHKPIVWHVNNATNKATYKPNTWCIRCLWSTKPVETSNSFDVLKSEDAQGMDNLA
EPI_ISL_426903 1163 KGAKLLHKPIVWHVNNATNKATYKPNTWCIRCLWSTKPVETSNSFDVLKSEDAQGMDNLA
EPI_ISL_426904 1163 KGAKLLHKPIVWHVNNATNKATYKPNTWCIRCLWSTKPVETSNSFDVLKSEDAQGMDNLA
EPI_ISL_426907 1163 KGAKLLHKPIVWHVNNATNKATYKPNTWCIRCLWSTKPVETSNSFDVLKSEDAQGMDNLA
EPI_ISL_426910 1163 KGAKLLHKPIVWHVNNATNKATYKPNTWCIRCLWSTKPVETSNSFDVLKSEDAQGMDNLA
ORF1ab         1981 KGAKLLHKPIVWHVNNATNKATYKPNTWCIRCLWSTKPVETSNSFDVLKSEDAQGMDNLA
EPI_ISL_413575 1163 KGAKLLHKPIVWHVNNATNKATYKPNTWCIRCLWSTKPVETSNSFDVLKSEDAQGMDNLA
EPI_ISL_413588 1163 KGAKLLHKPIVWHVNNATNKATYKPNTWCIRCLWSTKPVETSNSFDVLKSEDAQGMDNLA
EPI_ISL_413589 1163 KGAKLLHKPIVWHVNNATNKATYKPNTWCIRCLWSTKPVETSNSFDVLKSEDAQGMDNLA
EPI_ISL_424221 1163 KGAKLLHKPIVWHVNNATNKATYKPNTWCIRCLWSTKPVETSNSFDVLKSEDAQGMDNLA
EPI_ISL_424233 1163 KGAKLLHKPIVWHVNNATNKATYKPNTWCIRCLWSTKPVETSNSFDVLKSEDAQGMDNLA
EPI_ISL_424236 1163 KGAKLLHKPIVWHVNNATNKATYKPNTWCIRCLWSTKPVETSNSFDVLKSEDAQGMDNLA
EPI_ISL_424238 1163 KGAKLLHKPIVWHVNNATNKATYKPNTWCIRCLWSTKPVETSNSFDVLKSEDAQGMDNLA
EPI_ISL_424248 1163 KGAKLLHKPIVWHVNNATNKATYKPNTWCIRCLWSTKPVETSNSFDVLKSEDAQGMDNLA
EPI_ISL_424254 1163 KGAKLLHKPIVWHVNNATNKATYKPNTWCIRCLWSTKPVETSNSFDVLKSEDAQGMDNLA
EPI_ISL_425548 1163 KGAKLLHKPIVWHVNNATNKATYKPNTWCIRCLWSTKPVETSNSFDVLKSEDAQGMDNLA
EPI_ISL_425581 1163 KGAKLLHKPIVWHVNNATNKATYKPNTWCIRCLWSTKPVETSNSFDVLKSEDAQGMDNLA
EPI_ISL_426881 1163 KGAKLLHKPIVWHVNNATNKATYKPNTWCIRCLWSTKPVETSNSFDVLKSEDAQGMDNLA
EPI_ISL_426883 1163 KGAKLLHKPIVWHVNNATNKATYKPNTWCIRCLWSTKPVETSNSFDVLKSEDAQGMDNLA
EPI_ISL_426888 1163 KGAKLLHKPIVWHVNNATNKATYKPNTWCIRCLWSTKPVETSNSFDVLKSEDAQGMDNLA
EPI_ISL_426900 1163 KGAKLLHKPIVWHVNNATNKATYKPNTWCIRCLWSTKPVETSNSFDVLKSEDAQGMDNLA
EPI_ISL_426901 1163 KGAKLLHKPIVWHVNNATNKATYKPNTWCIRCLWSTKPVETSNSFDVLKSEDAQGMDNLA
EPI_ISL_426903 1163 KGAKLLHKPIVWHVNNATNKATYKPNTWCIRCLWSTKPVETSNSFDVLKSEDAQGMDNLA
EPI_ISL_426904 1163 KGAKLLHKPIVWHVNNATNKATYKPNTWCIRCLWSTKPVETSNSFDVLKSEDAQGMDNLA
EPI_ISL_426907 1163 KGAKLLHKPIVWHVNNATNKATYKPNTWCIRCLWSTKPVETSNSFDVLKSEDAQGMDNLA
EPI_ISL_426910 1163 KGAKLLHKPIVWHVNNATNKATYKPNTWCIRCLWSTKPVETSNSFDVLKSEDAQGMDNLA
```

```
ORF1ab         2041 CEDLKPVSEEVVENPTIQKDVLECNVKTTEVVGDIILKPANNSLKITEEVGHTDLMAAYV
EPI_ISL_413575 1223 CEDLKPVSEEVVENPTIQKDVLECNVKTTEVVGDIILKPANNSLKITEEVGHTDLMAAYV
EPI_ISL_413588 1223 CEDLKPVSEEVVENPTIQKDVLECNVKTTEVVGDIILKPANNSLKITEEVGHTDLMAAYV
EPI_ISL_413589 1223 CEDLKPVSEEVVENPTIQKDVLECNVKTTEVVGDIILKPANNSLKITEEVGHTDLMAAYV
EPI_ISL_424221 1223 CEDLKPVSEEVVENPTIQKDVLECNVKTTEVVGDIILKPANNSLKITEEVGHTDLMAAYV
EPI_ISL_424233 1223 CEDLKPVSEEVVENPTIQKDVLECNVKTTEVVGDIILKPANNSLKITEEVGHTDLMAAYV
EPI_ISL_424236 1223 CEDLKPVSEEVVENPTIQKDVLECNVKTTEVVGDIILKPANNSLKITEEVGHTDLMAAYV
EPI_ISL_424238 1223 CEDLKPVSEEVVENPTIQKDVLECNVKTTEVVGDIILKPANNSLKITEEVGHTDLMAAYV
EPI_ISL_424248 1223 CEDLKPVSEEVVENPTIQKDVLECNVKTTEVVGDIILKPANNSLKITEEVGHTDLMAAYV
EPI_ISL_424254 1223 CEDLKPVSEEVVENPTIQKDVLECNVKTTEVVGDIILKPANNSLKITEEVGHTDLMAAYV
EPI_ISL_425548 1223 CEDLKPVSEEVVENPTIQKDVLECNVKTTEVVGDIILKPANNSLKITEEVGHTDLMAAYV
EPI_ISL_425581 1223 CEDLKPVSEEVVENPTIQKDVLECNVKTTEVVGDIILKPANNSLKITEEVGHTDLMAAYV
EPI_ISL_426881 1223 CEDLKPVSEEVVENPTIQKDVLECNVKTTEVVGDIILKPANNSLKITEEVGHTDLMAAYV
EPI_ISL_426883 1223 CEDLKPVSEEVVENPTIQKDVLECNVKTTEVVGDIILKPANNSLKITEEVGHTDLMAAYV
EPI_ISL_426888 1223 CEDLKPVSEEVVENPTIQKDVLECNVKTTEVVGDIILKPANNSLKITEEVGHTDLMAAYV
EPI_ISL_426900 1223 CEDLKPVSEEVVENPTIQKDVLECNVKTTEVVGDIILKPANNSLKITEEVGHTDLMAAYV
EPI_ISL_426901 1223 CEDLKPVSEEVVENPTIQKDVLECNVKTTEVVGDIILKPANNSLKITEEVGHTDLMAAYV
EPI_ISL_426903 1223 CEDLKPVSEEVVENPTIQKDVLECNVKTTEVVGDIILKPANNSLKITEEVGHTDLMAAYV
EPI_ISL_426904 1223 CEDLKPVSEEVVENPTIQKDVLECNVKTTEVVGDIILKPANNSLKITEEVGHTDLMAAYV
EPI_ISL_426907 1223 CEDLKPVSEEVVENPTIQKDVLECNVKTTEVVGDIILKPANNSLKITEEVGHTDLMAAYV
EPI_ISL_426910 1223 CEDLKPVSEEVVENPTIQKDVLECNVKTTEVVGDIILKPANNSLKITEEVGHTDLMAAYV
ORF1ab         2041 CEDLKPVSEEVVENPTIQKDVLECNVKTTEVVGDIILKPANNSLKITEEVGHTDLMAAYV
EPI_ISL_413575 1223 CEDLKPVSEEVVENPTIQKDVLECNVKTTEVVGDIILKPANNSLKITEEVGHTDLMAAYV
EPI_ISL_413588 1223 CEDLKPVSEEVVENPTIQKDVLECNVKTTEVVGDIILKPANNSLKITEEVGHTDLMAAYV
EPI_ISL_413589 1223 CEDLKPVSEEVVENPTIQKDVLECNVKTTEVVGDIILKPANNSLKITEEVGHTDLMAAYV
EPI_ISL_424221 1223 CEDLKPVSEEVVENPTIQKDVLECNVKTTEVVGDIILKPANNSLKITEEVGHTDLMAAYV
EPI_ISL_424233 1223 CEDLKPVSEEVVENPTIQKDVLECNVKTTEVVGDIILKPANNSLKITEEVGHTDLMAAYV
EPI_ISL_424236 1223 CEDLKPVSEEVVENPTIQKDVLECNVKTTEVVGDIILKPANNSLKITEEVGHTDLMAAYV
EPI_ISL_424238 1223 CEDLKPVSEEVVENPTIQKDVLECNVKTTEVVGDIILKPANNSLKITEEVGHTDLMAAYV
EPI_ISL_424248 1223 CEDLKPVSEEVVENPTIQKDVLECNVKTTEVVGDIILKPANNSLKITEEVGHTDLMAAYV
EPI_ISL_424254 1223 CEDLKPVSEEVVENPTIQKDVLECNVKTTEVVGDIILKPANNSLKITEEVGHTDLMAAYV
EPI_ISL_425548 1223 CEDLKPVSEEVVENPTIQKDVLECNVKTTEVVGDIILKPANNSLKITEEVGHTDLMAAYV
EPI_ISL_425581 1223 CEDLKPVSEEVVENPTIQKDVLECNVKTTEVVGDIILKPANNSLKITEEVGHTDLMAAYV
EPI_ISL_426881 1223 CEDLKPVSEEVVENPTIQKDVLECNVKTTEVVGDIILKPANNSLKITEEVGHTDLMAAYV
EPI_ISL_426883 1223 CEDLKPVSEEVVENPTIQKDVLECNVKTTEVVGDIILKPANNSLKITEEVGHTDLMAAYV
EPI_ISL_426888 1223 CEDLKPVSEEVVENPTIQKDVLECNVKTTEVVGDIILKPANNSLKITEEVGHTDLMAAYV
EPI_ISL_426900 1223 CEDLKPVSEEVVENPTIQKDVLECNVKTTEVVGDIILKPANNSLKITEEVGHTDLMAAYV
EPI_ISL_426901 1223 CEDLKPVSEEVVENPTIQKDVLECNVKTTEVVGDIILKPANNSLKITEEVGHTDLMAAYV
EPI_ISL_426903 1223 CEDLKPVSEEVVENPTIQKDVLECNVKTTEVVGDIILKPANNSLKITEEVGHTDLMAAYV
EPI_ISL_426904 1223 CEDLKPVSEEVVENPTIQKDVLECNVKTTEVVGDIILKPANNSLKITEEVGHTDLMAAYV
EPI_ISL_426907 1223 CEDLKPVSEEVVENPTIQKDVLECNVKTTEVVGDIILKPANNSLKITEEVGHTDLMAAYV
EPI_ISL_426910 1223 CEDLKPVSEEVVENPTIQKDVLECNVKTTEVVGDIILKPANNSLKITEEVGHTDLMAAYV
```

```
ORF1ab         2101 DNSSLTIKKPNELSRVLGLKTLATHGLAAVNSVPWDTIANYAKPFLNKVVSTTTNIVTRC
EPI_ISL_413575 1283 DNSSLTIKKPNELSRVLGLKTLATHGLAAVNSVPWDTIANYAKPFLNKVVSTTTNIVTRC
EPI_ISL_413588 1283 DNSSLTIKKPNELSRVLGLKTLATHGLAAVNSVPWDTIANYAKPFLNKVVSTTTNIVTRC
EPI_ISL_413589 1283 DNSSLTIKKPNELSRVLGLKTLATHGLAAVNSVPWDTIANYAKPFLNKVVSTTTNIVTRC
EPI_ISL_424221 1283 DNSSLTIKKPNELSRVLGLKTLATHGLAAVNSVPWDTIANYAKPFLNKVVSTTTNIVTRC
EPI_ISL_424233 1283 DNSSLTIKKPNELSRVLGLKTLATHGLAAVNSVPWDTIANYAKPFLNKVVSTTTNIVTRC
EPI_ISL_424236 1283 DNSSLTIKKPNELSRVLGLKTLATHGLAAVNSVPWDTIANYAKPFLNKVVSTTTNIVTRC
EPI_ISL_424238 1283 DNSSLTIKKPNELSRVLGLKTLATHGLAAVNSVPWDTIANYAKPFLNKVVSTTTNIVTRC
EPI_ISL_424248 1283 DNSSLTIKKPNELSRVLGLKTLATHGLAAVNSVPWDTIANYAKPFLNKVVSTTTNIVTRC
EPI_ISL_424254 1283 DNSSLTIKKPNELSRVLGLKTLATHGLAAVNSVPWDTIANYAKPFLNKVVSTTTNIVTRC
EPI_ISL_425548 1283 DNSSLTIKKPNELSRVLGLKTLATHGLAAVNSVPWDTIANYAKPFLNKVVSTTTNIVTRC
EPI_ISL_425581 1283 DNSSLTIKKPNELSRVLGLKTLATHGLAAVNSVPWDTIANYAKPFLNKVVSTTTNIVTRC
EPI_ISL_426881 1283 DNSSLTIKKPNELSRVLGLKTLATHGLAAVNSVPWDTIANYAKPFLNKVVSTTTNIVTRC
EPI_ISL_426883 1283 DNSSLTIKKPNELSRVLGLKTLATHGLAAVNSVPWDTIANYAKPFLNKVVSTTTNIVTRC
EPI_ISL_426888 1283 DNSSLTIKKPNELSRVLGLKTLATHGLAAVNSVPWDTIANYAKPFLNKVVSTTTNIVTRC
EPI_ISL_426900 1283 DNSSLTIKKPNELSRVLGLKTLATHGLAAVNSVPWDTIANYAKPFLNKVVSTTTNIVTRC
EPI_ISL_426901 1283 DNSSLTIKKPNELSRVLGLKTLATHGLAAVNSVPWDTIANYAKPFLNKVVSTTTNIVTRC
EPI_ISL_426903 1283 DNSSLTIKKPNELSRVLGLKTLATHGLAAVNSVPWDTIANYAKPFLNKVVSTTTNIVTRC
EPI_ISL_426904 1283 DNSSLTIKKPNELSRVLGLKTLATHGLAAVNSVPWDTIANYAKPFLNKVVSTTTNIVTRC
EPI_ISL_426907 1283 DNSSLTIKKPNELSRVLGLKTLATHGLAAVNSVPWDTIANYAKPFLNKVVSTTTNIVTRC
EPI_ISL_426910 1283 DNSSLTIKKPNELSRVLGLKTLATHGLAAVNSVPWDTIANYAKPFLNKVVSTTTNIVTRC
ORF1ab         2101 DNSSLTIKKPNELSRVLGLKTLATHGLAAVNSVPWDTIANYAKPFLNKVVSTTTNIVTRC
EPI_ISL_413575 1283 DNSSLTIKKPNELSRVLGLKTLATHGLAAVNSVPWDTIANYAKPFLNKVVSTTTNIVTRC
EPI_ISL_413588 1283 DNSSLTIKKPNELSRVLGLKTLATHGLAAVNSVPWDTIANYAKPFLNKVVSTTTNIVTRC
EPI_ISL_413589 1283 DNSSLTIKKPNELSRVLGLKTLATHGLAAVNSVPWDTIANYAKPFLNKVVSTTTNIVTRC
EPI_ISL_424221 1283 DNSSLTIKKPNELSRVLGLKTLATHGLAAVNSVPWDTIANYAKPFLNKVVSTTTNIVTRC
EPI_ISL_424233 1283 DNSSLTIKKPNELSRVLGLKTLATHGLAAVNSVPWDTIANYAKPFLNKVVSTTTNIVTRC
EPI_ISL_424236 1283 DNSSLTIKKPNELSRVLGLKTLATHGLAAVNSVPWDTIANYAKPFLNKVVSTTTNIVTRC
EPI_ISL_424238 1283 DNSSLTIKKPNELSRVLGLKTLATHGLAAVNSVPWDTIANYAKPFLNKVVSTTTNIVTRC
EPI_ISL_424248 1283 DNSSLTIKKPNELSRVLGLKTLATHGLAAVNSVPWDTIANYAKPFLNKVVSTTTNIVTRC
EPI_ISL_424254 1283 DNSSLTIKKPNELSRVLGLKTLATHGLAAVNSVPWDTIANYAKPFLNKVVSTTTNIVTRC
EPI_ISL_425548 1283 DNSSLTIKKPNELSRVLGLKTLATHGLAAVNSVPWDTIANYAKPFLNKVVSTTTNIVTRC
EPI_ISL_425581 1283 DNSSLTIKKPNELSRVLGLKTLATHGLAAVNSVPWDTIANYAKPFLNKVVSTTTNIVTRC
EPI_ISL_426881 1283 DNSSLTIKKPNELSRVLGLKTLATHGLAAVNSVPWDTIANYAKPFLNKVVSTTTNIVTRC
EPI_ISL_426883 1283 DNSSLTIKKPNELSRVLGLKTLATHGLAAVNSVPWDTIANYAKPFLNKVVSTTTNIVTRC
EPI_ISL_426888 1283 DNSSLTIKKPNELSRVLGLKTLATHGLAAVNSVPWDTIANYAKPFLNKVVSTTTNIVTRC
EPI_ISL_426900 1283 DNSSLTIKKPNELSRVLGLKTLATHGLAAVNSVPWDTIANYAKPFLNKVVSTTTNIVTRC
EPI_ISL_426901 1283 DNSSLTIKKPNELSRVLGLKTLATHGLAAVNSVPWDTIANYAKPFLNKVVSTTTNIVTRC
EPI_ISL_426903 1283 DNSSLTIKKPNELSRVLGLKTLATHGLAAVNSVPWDTIANYAKPFLNKVVSTTTNIVTRC
EPI_ISL_426904 1283 DNSSLTIKKPNELSRVLGLKTLATHGLAAVNSVPWDTIANYAKPFLNKVVSTTTNIVTRC
EPI_ISL_426907 1283 DNSSLTIKKPNELSRVLGLKTLATHGLAAVNSVPWDTIANYAKPFLNKVVSTTTNIVTRC
EPI_ISL_426910 1283 DNSSLTIKKPNELSRVLGLKTLATHGLAAVNSVPWDTIANYAKPFLNKVVSTTTNIVTRC
```

ORF1ab	2161	LNRVCTNYMPYFFTLLLQLCTFTRSTNSRIKASMPTTIAKNTVKSVGKFCLEASFNYLKS
EPI_ISL_413575	1343	LNRVCTNYMPYFFTLLLQLCTFTRSTNSRIKAFMPTTIAKNTVKSVGKFCLEASFNYLKS
EPI_ISL_413588	1343	LNRVCTNYMPYFFTLLLQLCTFTRSTNSRIKAFMPTTIAKNTVKSVGKFCLEASFNYLKS
EPI_ISL_413589	1343	LNRVCTNYMPYFFTLLLQLCTFTRSTNSRIKASMPTTIAKNTVKSVGKFCLEASFNYLKS
EPI_ISL_424221	1343	LNRVCTNYMPYFFTLLLQLCTFTRSTNSRIKASMPTTIAKNTVKSVGKFCLEASFNYLKS
EPI_ISL_424233	1343	LNRVCTNYMPYFFTLLLQLCTFTRSTNSRIKASMPTTIAKNTVKSVGKFCLEASFNYLKS
EPI_ISL_424236	1343	LNRVCTNYMPYFFTLLLQLCTFTRSTNSRIKASMPTTIAKNTVKSVGKFCLEASFNYLKS
EPI_ISL_424238	1343	LNRVCTNYMPYFFTLLLQLCTFTRSTNSRIKASMPTTIAKNTVKSVGKFCLEASFNYLKS
EPI_ISL_424248	1343	LNRVCTNYMPYFFTLLLQLCTFTRSTNSRIKASMPTTIAKNTVKSVGKFCLEASFNYLKS
EPI_ISL_424254	1343	LNRVCTNYMPYFFTLLLQLCTFTRSTNSRIKASMPTTIAKNTVKSVGKFCLEASFNYLKS
EPI_ISL_425548	1343	LNRVCTNYMPYFFTLLLQLCTFTRSTNSRIKASMPTTIAKNTVKSVGKFCLEASFNYLKS
EPI_ISL_425581	1343	LNRVCTNYMPYFFTLLLQLCTFTRSTNSRIKASMPTTIAKNTVKSVGKFCLEASFNYLKS
EPI_ISL_426881	1343	LNRVCTNYMPYFFTLLLQLCTFTRSTNSRIKASMPTTIAKNTVKSVGKFCLEASFNYLKS
EPI_ISL_426883	1343	LNRVCTNYMPYFFTLLLQLCTFTRSTNSRIKASMPTTIAKNTVKSVGKFCLEASFNYLKS
EPI_ISL_426888	1343	LNRVCTNYMPYFFTLLLQLCTFTRSTNSRIKASMPTTIAKNTVKSVGKFCLEASFNYLKS
EPI_ISL_426900	1343	LNRVCTNYMPYFFTLLLQLCTFTRSTNSRIKASMPTTIAKNTVKSVGKFCLEASFNYLKS
EPI_ISL_426901	1343	LNRVCTNYMPYFFTLLLQLCTFTRSTNSRIKASMPTTIAKNTVKSVGKFCLEASFNYLKS
EPI_ISL_426903	1343	LNRVCTNYMPYFFTLLLQLCTFTRSTNSRIKASMPTTIAKNTVKSVGKFCLEASFNYLKS
EPI_ISL_426904	1343	LNRVCTNYMPYFFTLLLQLCTFTRSTNSRIKASMPTTIAKNTVKSVGKFCLEASFNYLKS
EPI_ISL_426907	1343	LNRVCTNYMPYFFTLLLQLCTFTRSTNSRIKASMPTTIAKNTVKSVGKFCLEASFNYLKS
EPI_ISL_426910	1343	LNRVCTNYMPYFFTLLLQLCTFTRSTNSRIKASMPTTIAKNTVKSVGKFCLEASFNYLKS

ORF1ab	2161	LNRVCTNYMPYFFTLLLQLCTFTRSTNSRIKASMPTTIAKNTVKSVGKFCLEASFNYLKS
EPI_ISL_413575	1343	LNRVCTNYMPYFFTLLLQLCTFTRSTNSRIKAFMPTTIAKNTVKSVGKFCLEASFNYLKS
EPI_ISL_413588	1343	LNRVCTNYMPYFFTLLLQLCTFTRSTNSRIKAFMPTTIAKNTVKSVGKFCLEASFNYLKS
EPI_ISL_413589	1343	LNRVCTNYMPYFFTLLLQLCTFTRSTNSRIKASMPTTIAKNTVKSVGKFCLEASFNYLKS
EPI_ISL_424221	1343	LNRVCTNYMPYFFTLLLQLCTFTRSTNSRIKASMPTTIAKNTVKSVGKFCLEASFNYLKS
EPI_ISL_424233	1343	LNRVCTNYMPYFFTLLLQLCTFTRSTNSRIKASMPTTIAKNTVKSVGKFCLEASFNYLKS
EPI_ISL_424236	1343	LNRVCTNYMPYFFTLLLQLCTFTRSTNSRIKASMPTTIAKNTVKSVGKFCLEASFNYLKS
EPI_ISL_424238	1343	LNRVCTNYMPYFFTLLLQLCTFTRSTNSRIKASMPTTIAKNTVKSVGKFCLEASFNYLKS
EPI_ISL_424248	1343	LNRVCTNYMPYFFTLLLQLCTFTRSTNSRIKASMPTTIAKNTVKSVGKFCLEASFNYLKS
EPI_ISL_424254	1343	LNRVCTNYMPYFFTLLLQLCTFTRSTNSRIKASMPTTIAKNTVKSVGKFCLEASFNYLKS
EPI_ISL_425548	1343	LNRVCTNYMPYFFTLLLQLCTFTRSTNSRIKASMPTTIAKNTVKSVGKFCLEASFNYLKS
EPI_ISL_425581	1343	LNRVCTNYMPYFFTLLLQLCTFTRSTNSRIKASMPTTIAKNTVKSVGKFCLEASFNYLKS
EPI_ISL_426881	1343	LNRVCTNYMPYFFTLLLQLCTFTRSTNSRIKASMPTTIAKNTVKSVGKFCLEASFNYLKS
EPI_ISL_426883	1343	LNRVCTNYMPYFFTLLLQLCTFTRSTNSRIKASMPTTIAKNTVKSVGKFCLEASFNYLKS
EPI_ISL_426888	1343	LNRVCTNYMPYFFTLLLQLCTFTRSTNSRIKASMPTTIAKNTVKSVGKFCLEASFNYLKS
EPI_ISL_426900	1343	LNRVCTNYMPYFFTLLLQLCTFTRSTNSRIKASMPTTIAKNTVKSVGKFCLEASFNYLKS
EPI_ISL_426901	1343	LNRVCTNYMPYFFTLLLQLCTFTRSTNSRIKASMPTTIAKNTVKSVGKFCLEASFNYLKS
EPI_ISL_426903	1343	LNRVCTNYMPYFFTLLLQLCTFTRSTNSRIKASMPTTIAKNTVKSVGKFCLEASFNYLKS
EPI_ISL_426904	1343	LNRVCTNYMPYFFTLLLQLCTFTRSTNSRIKASMPTTIAKNTVKSVGKFCLEASFNYLKS
EPI_ISL_426907	1343	LNRVCTNYMPYFFTLLLQLCTFTRSTNSRIKASMPTTIAKNTVKSVGKFCLEASFNYLKS
EPI_ISL_426910	1343	LNRVCTNYMPYFFTLLLQLCTFTRSTNSRIKASMPTTIAKNTVKSVGKFCLEASFNYLKS

ORF1ab	2221	PNFSKLINIIIWFLLLSVCLGSLIYSTAALGVLMSNLGMPSYCTGYREGYLNSTNVTIAT
EPI_ISL_413575	1403	PNFSKLINIIIWFLLLSVCLGSLIYSTAALGVLMSNLGMPSYCTGYREGYLNSTNVTIAT
EPI_ISL_413588	1403	PNFSKLINIIIWFLLLSVCLGSLIYSTAALGVLMSNLGMPSYCTGYREGYLNSTNVTIAT
EPI_ISL_413589	1403	PNFSKLINIIIWFLLLSVCLGSLIYSTAALGVLMSNLGMPSYCTGYREGYLNSTNVTIAT
EPI_ISL_424221	1403	PNFSKLINIIIWFLLLSVCLGSLIYSTAALGVLMSNLGMPSYCTGYREGYLNSTNVTIAT
EPI_ISL_424233	1403	PNFSKLINIIIWFLLLSVCLGSLIYSTAALGVLMSNLGMPSYCTGYREGYLNSTNVTIAT
EPI_ISL_424236	1403	PNFSKLINIIIWFLLLSVCLGSLIYSTAALGVLMSNLGMPSYCTGYREGYLNSTNVTIAT
EPI_ISL_424238	1403	PNFSKLINIIIWFLLLSVCLGSLIYSTAALGVLMSNLGMPSYCTGYREGYLNSTNVTIAT
EPI_ISL_424248	1403	PNFSKLINIIIWFLLLSVCLGSLIYSTAALGVLMSNLGMPSYCTGYREGYLNSTNVTIAT
EPI_ISL_424254	1403	PNFSKLINIIIWFLLLSVCLGSLIYSTAALGVLMSNLGMPSYCTGYREGYLNSTNVTIAT
EPI_ISL_425548	1403	PNFSKLINIIIWFLLLSVCLGSLIYSTAALGVLMSNLGMPSYCTGYREGYLNSTNVTIAT
EPI_ISL_425581	1403	PNFSKLINIIIWFLLLSVCLGSLIYSTAALGVLMSNLGMPSYCTGYREGYLNSTNVTIAT
EPI_ISL_426881	1403	PNFSKLINIIIWFLLLSVCLGSLIYSTAALGVLMSNLGMPSYCTGYREGYLNSTNVTIAT
EPI_ISL_426883	1403	PNFSKLINIIIWFLLLSVCLGSLIYSTAALGVLMSNLGMPSYCTGYREGYLNSTNVTIAT
EPI_ISL_426888	1403	PNFSKLINIIIWFLLLSVCLGSLIYSTAALGVLMSNLGMPSYCTGYREGYLNSTNVTIAT
EPI_ISL_426900	1403	PNFSKLINIIIWFLLLSVCLGSLIYSTAALGVLMSNLGMPSYCTGYREGYLNSTNVTIAT
EPI_ISL_426901	1403	PNFSKLINIIIWFLLLSVCLGSLIYSTAALGVLMSNLGMPSYCTGYREGYLNSTNVTIAT
EPI_ISL_426903	1403	PNFSKLINIIIWFLLLSVCLGSLIYSTAALGVLMSNLGMPSYCTGYREGYLNSTNVTIAT
EPI_ISL_426904	1403	PNFSKLINIIIWFLLLSVCLGSLIYSTAALGVLMSNLGMPSYCTGYREGYLNSTNVTIAT
EPI_ISL_426907	1403	PNFSKLINIIIWFLLLSVCLGSLIYSTAALGVLMSNLGMPSYCTGYREGYLNSTNVTIAT
EPI_ISL_426910	1403	PNFSKLINIIIWFLLLSVCLGSLIYSTAALGVLMSNLGMPSYCTGYREGYLNSTNVTIAT
ORF1ab	2221	PNFSKLINIIIWFLLLSVCLGSLIYSTAALGVLMSNLGMPSYCTGYREGYLNSTNVTIAT
EPI_ISL_413575	1403	PNFSKLINIIIWFLLLSVCLGSLIYSTAALGVLMSNLGMPSYCTGYREGYLNSTNVTIAT
EPI_ISL_413588	1403	PNFSKLINIIIWFLLLSVCLGSLIYSTAALGVLMSNLGMPSYCTGYREGYLNSTNVTIAT
EPI_ISL_413589	1403	PNFSKLINIIIWFLLLSVCLGSLIYSTAALGVLMSNLGMPSYCTGYREGYLNSTNVTIAT
EPI_ISL_424221	1403	PNFSKLINIIIWFLLLSVCLGSLIYSTAALGVLMSNLGMPSYCTGYREGYLNSTNVTIAT
EPI_ISL_424233	1403	PNFSKLINIIIWFLLLSVCLGSLIYSTAALGVLMSNLGMPSYCTGYREGYLNSTNVTIAT
EPI_ISL_424236	1403	PNFSKLINIIIWFLLLSVCLGSLIYSTAALGVLMSNLGMPSYCTGYREGYLNSTNVTIAT
EPI_ISL_424238	1403	PNFSKLINIIIWFLLLSVCLGSLIYSTAALGVLMSNLGMPSYCTGYREGYLNSTNVTIAT
EPI_ISL_424248	1403	PNFSKLINIIIWFLLLSVCLGSLIYSTAALGVLMSNLGMPSYCTGYREGYLNSTNVTIAT
EPI_ISL_424254	1403	PNFSKLINIIIWFLLLSVCLGSLIYSTAALGVLMSNLGMPSYCTGYREGYLNSTNVTIAT
EPI_ISL_425548	1403	PNFSKLINIIIWFLLLSVCLGSLIYSTAALGVLMSNLGMPSYCTGYREGYLNSTNVTIAT
EPI_ISL_425581	1403	PNFSKLINIIIWFLLLSVCLGSLIYSTAALGVLMSNLGMPSYCTGYREGYLNSTNVTIAT
EPI_ISL_426881	1403	PNFSKLINIIIWFLLLSVCLGSLIYSTAALGVLMSNLGMPSYCTGYREGYLNSTNVTIAT
EPI_ISL_426883	1403	PNFSKLINIIIWFLLLSVCLGSLIYSTAALGVLMSNLGMPSYCTGYREGYLNSTNVTIAT
EPI_ISL_426888	1403	PNFSKLINIIIWFLLLSVCLGSLIYSTAALGVLMSNLGMPSYCTGYREGYLNSTNVTIAT
EPI_ISL_426900	1403	PNFSKLINIIIWFLLLSVCLGSLIYSTAALGVLMSNLGMPSYCTGYREGYLNSTNVTIAT
EPI_ISL_426901	1403	PNFSKLINIIIWFLLLSVCLGSLIYSTAALGVLMSNLGMPSYCTGYREGYLNSTNVTIAT
EPI_ISL_426903	1403	PNFSKLINIIIWFLLLSVCLGSLIYSTAALGVLMSNLGMPSYCTGYREGYLNSTNVTIAT
EPI_ISL_426904	1403	PNFSKLINIIIWFLLLSVCLGSLIYSTAALGVLMSNLGMPSYCTGYREGYLNSTNVTIAT
EPI_ISL_426907	1403	PNFSKLINIIIWFLLLSVCLGSLIYSTAALGVLMSNLGMPSYCTGYREGYLNSTNVTIAT
EPI_ISL_426910	1403	PNFSKLINIIIWFLLLSVCLGSLIYSTAALGVLMSNLGMPSYCTGYREGYLNSTNVTIAT

```
ORF1ab         2281 YCTGSIPCSVCLSGLDSLDTYPSLETIQITISSFKWDLTAFGLVAEWFLAYILFTRFFYV
EPI_ISL_413575 1463 YCTGSIPCSVCLSGLDSLDTYPSLETIQITISSFKWDLTAFGLVAEWFLAYILFTRFFYV
EPI_ISL_413588 1463 YCTGSIPCSVCLSGLDSLDTYPSLETIQITISSFKWDLTAFGLVAEWFLAYILFTRFFYV
EPI_ISL_413589 1463 YCTGSIPCSVCLSGLDSLDTYPSLETIQITISSFKWDLTAFGLVAEWFLAYILFTRFFYV
EPI_ISL_424221 1463 YCTGSIPCSVCLSGLDSLDTYPSLETIQITISSFKWDLTAFGLVAEWFLAYILFTRFFYV
EPI_ISL_424233 1463 YCTGSIPCSVCLSGLDSLDTYPSLETIQITISSFKWDLTAFGLVAEWFLAYILFTRFFYV
EPI_ISL_424236 1463 YCTGSIPCSVCLSGLDSLDTYPSLETIQITISSFKWDLTAFGLVAEWFLAYILFTRFFYV
EPI_ISL_424238 1463 YCTGSIPCSVCLSGLDSLDTYPSLETIQITISSFKWDLTAFGLVAEWFLAYILFTRFFYV
EPI_ISL_424248 1463 YCTGSIPCSVCLSGLDSLDTYPSLETIQITISSFKWDLTAFGLVAEWFLAYILFTRFFYV
EPI_ISL_424254 1463 YCTGSIPCSVCLSGLDSLDTYPSLETIQITISSFKWDLTAFGLVAEWFLAYILFTRFFYV
EPI_ISL_425548 1463 YCTGSIPCSVCLSGLDSLDTYPSLETIQITISSFKWDLTAFGLVAEWFLAYILFTRFFYV
EPI_ISL_425581 1463 YCTGSIPCSVCLSGLDSLDTYPSLETIQITISSFKWDLTAFGLVAEWFLAYILFTRFFYV
EPI_ISL_426881 1463 YCTGSIPCSVCLSGLDSLDTYPSLETIQITISSFKWDLTAFGLVAEWFLAYILFTRFFYV
EPI_ISL_426883 1463 YCTGSIPCSVCLSGLDSLDTYPSLETIQITISSFKWDLTAFGLVAEWFLAYILFTRFFYV
EPI_ISL_426888 1463 YCTGSIPCSVCLSGLDSLDTYPSLETIQITISSFKWDLTAFGLVAEWFLAYILFTRFFYV
EPI_ISL_426900 1463 YCTGSIPCSVCLSGLDSLDTYPSLETIQITISSFKWDLTAFGLVAEWFLAYILFTRFFYV
EPI_ISL_426901 1463 YCTGSIPCSVCLSGLDSLDTYPSLETIQITISSFKWDLTAFGLVAEWFLAYILFTRFFYV
EPI_ISL_426903 1463 YCTGSIPCSVCLSGLDSLDTYPSLETIQITISSFKWDLTAFGLVAEWFLAYILFTRFFYV
EPI_ISL_426904 1463 YCTGSIPCSVCLSGLDSLDTYPSLETIQITISSFKWDLTAFGLVAEWFLAYILFTRFFYV
EPI_ISL_426907 1463 YCTGSIPCSVCLSGLDSLDTYPSLETIQITISSFKWDLTAFGLVAEWFLAYILFTRFFYV
EPI_ISL_426910 1463 YCTGSIPCSVCLSGLDSLDTYPSLETIQITISSFKWDLTAFGLVAEWFLAYILFTRFFYV
ORF1ab         2281 YCTGSIPCSVCLSGLDSLDTYPSLETIQITISSFKWDLTAFGLVAEWFLAYILFTRFFYV
EPI_ISL_413575 1463 YCTGSIPCSVCLSGLDSLDTYPSLETIQITISSFKWDLTAFGLVAEWFLAYILFTRFFYV
EPI_ISL_413588 1463 YCTGSIPCSVCLSGLDSLDTYPSLETIQITISSFKWDLTAFGLVAEWFLAYILFTRFFYV
EPI_ISL_413589 1463 YCTGSIPCSVCLSGLDSLDTYPSLETIQITISSFKWDLTAFGLVAEWFLAYILFTRFFYV
EPI_ISL_424221 1463 YCTGSIPCSVCLSGLDSLDTYPSLETIQITISSFKWDLTAFGLVAEWFLAYILFTRFFYV
EPI_ISL_424233 1463 YCTGSIPCSVCLSGLDSLDTYPSLETIQITISSFKWDLTAFGLVAEWFLAYILFTRFFYV
EPI_ISL_424236 1463 YCTGSIPCSVCLSGLDSLDTYPSLETIQITISSFKWDLTAFGLVAEWFLAYILFTRFFYV
EPI_ISL_424238 1463 YCTGSIPCSVCLSGLDSLDTYPSLETIQITISSFKWDLTAFGLVAEWFLAYILFTRFFYV
EPI_ISL_424248 1463 YCTGSIPCSVCLSGLDSLDTYPSLETIQITISSFKWDLTAFGLVAEWFLAYILFTRFFYV
EPI_ISL_424254 1463 YCTGSIPCSVCLSGLDSLDTYPSLETIQITISSFKWDLTAFGLVAEWFLAYILFTRFFYV
EPI_ISL_425548 1463 YCTGSIPCSVCLSGLDSLDTYPSLETIQITISSFKWDLTAFGLVAEWFLAYILFTRFFYV
EPI_ISL_425581 1463 YCTGSIPCSVCLSGLDSLDTYPSLETIQITISSFKWDLTAFGLVAEWFLAYILFTRFFYV
EPI_ISL_426881 1463 YCTGSIPCSVCLSGLDSLDTYPSLETIQITISSFKWDLTAFGLVAEWFLAYILFTRFFYV
EPI_ISL_426883 1463 YCTGSIPCSVCLSGLDSLDTYPSLETIQITISSFKWDLTAFGLVAEWFLAYILFTRFFYV
EPI_ISL_426888 1463 YCTGSIPCSVCLSGLDSLDTYPSLETIQITISSFKWDLTAFGLVAEWFLAYILFTRFFYV
EPI_ISL_426900 1463 YCTGSIPCSVCLSGLDSLDTYPSLETIQITISSFKWDLTAFGLVAEWFLAYILFTRFFYV
EPI_ISL_426901 1463 YCTGSIPCSVCLSGLDSLDTYPSLETIQITISSFKWDLTAFGLVAEWFLAYILFTRFFYV
EPI_ISL_426903 1463 YCTGSIPCSVCLSGLDSLDTYPSLETIQITISSFKWDLTAFGLVAEWFLAYILFTRFFYV
EPI_ISL_426904 1463 YCTGSIPCSVCLSGLDSLDTYPSLETIQITISSFKWDLTAFGLVAEWFLAYILFTRFFYV
EPI_ISL_426907 1463 YCTGSIPCSVCLSGLDSLDTYPSLETIQITISSFKWDLTAFGLVAEWFLAYILFTRFFYV
EPI_ISL_426910 1463 YCTGSIPCSVCLSGLDSLDTYPSLETIQITISSFKWDLTAFGLVAEWFLAYILFTRFFYV
```

```
ORF1ab         2341 LGLAAIMQLFFSYFAVHFISNSWLMWLIINLVQMAPISAMVRMYIFFASFYYVWKSYVHV
EPI_ISL_413575 1523 LGLAAIMQLFFSYFAVHFISNSWLMWLIINLVQMAPISAMVRMYIFFASFYYVWKSYVHV
EPI_ISL_413588 1523 LGLAAIMQLFFSYFAVHFISNSWLMWLIINLVQMAPISAMVRMYIFFASFYYVWKSYVHV
EPI_ISL_413589 1523 LGLAAIMQLFFSYFAVHFISNSWLMWLIINLVQMAPISAMVRMYIFFASFYYVWKSYVHV
EPI_ISL_424221 1523 LGLAAIMQLFFSYFAVHFISNSWLMWLIINLVQMAPISAMVRMYIFFASFYYVWKSYVHV
EPI_ISL_424233 1523 LGLAAIMQLFFSYFAVHFISNSWLMWLIINLVQMAPISAMVRMYIFFASFYYVWKSYVHV
EPI_ISL_424236 1523 LGLAAIMQLFFSYFAVHFISNSWLMWLIINLVQMAPISAMVRMYIFFASFYYVWKSYVHV
EPI_ISL_424238 1523 LGLAAIMQLFFSYFAVHFISNSWLMWLIINLVQMAPISAMVRMYIFFASFYYVWKSYVHV
EPI_ISL_424248 1523 LGLAAIMQLFFSYFAVHFISNSWLMWLIINLVQMAPISAMVRMYIFFASFYYVWKSYVHV
EPI_ISL_424254 1523 LGLAAIMQLFFSYFAVHFISNSWLMWLIINLVQMAPISAMVRMYIFFASFYYVWKSYVHV
EPI_ISL_425548 1523 LGLAAIMQLFFSYFAVHFISNSWLMWLIINLVQMAPISAMVRMYIFFASFYYVWKSYVHV
EPI_ISL_425581 1523 LGLAAIMQLFFSYFAVHFISNSWLMWLIINLVQMAPISAMVRMYIFFASFYYVWKSYVHV
EPI_ISL_426881 1523 LGLAAIMQLFFSYFAVHFISNSWLMWLIINLVQMAPISAMVRMYIFFASFYYVWKSYVHV
EPI_ISL_426883 1523 LGLAAIMQLFFSYFAVHFISNSWLMWLIINLVQMAPISAMVRMYIFFASFYYVWKSYVHV
EPI_ISL_426888 1523 LGLAAIMQLFFSYFAVHFISNSWLMWLIINLVQMAPISAMVRMYIFFASFYYVWKSYVHV
EPI_ISL_426900 1523 LGLAAIMQLFFSYFAVHFISNSWLMWLIINLVQMAPISAMVRMYIFFASFYYVWKSYVHV
EPI_ISL_426901 1523 LGLAAIMQLFFSYFAVHFISNSWLMWLIINLVQMAPISAMVRMYIFFASFYYVWKSYVHV
EPI_ISL_426903 1523 LGLAAIMQLFFSYFAVHFISNSWLMWLIINLVQMAPISAMVRMYIFFASFYYVWKSYVHV
EPI_ISL_426904 1523 LGLAAIMQLFFSYFAVHFISNSWLMWLIINLVQMAPISAMVRMYIFFASFYYVWKSYVHV
EPI_ISL_426907 1523 LGLAAIMQLFFSYFAVHFISNSWLMWLIINLVQMAPISAMVRMYIFFASFYYVWKSYVHV
EPI_ISL_426910 1523 LGLAAIMQLFFSYFAVHFISNSWLMWLIINLVQMAPISAMVRMYIFFASFYYVWKSYVHV
ORF1ab         2341 LGLAAIMQLFFSYFAVHFISNSWLMWLIINLVQMAPISAMVRMYIFFASFYYVWKSYVHV
EPI_ISL_413575 1523 LGLAAIMQLFFSYFAVHFISNSWLMWLIINLVQMAPISAMVRMYIFFASFYYVWKSYVHV
EPI_ISL_413588 1523 LGLAAIMQLFFSYFAVHFISNSWLMWLIINLVQMAPISAMVRMYIFFASFYYVWKSYVHV
EPI_ISL_413589 1523 LGLAAIMQLFFSYFAVHFISNSWLMWLIINLVQMAPISAMVRMYIFFASFYYVWKSYVHV
EPI_ISL_424221 1523 LGLAAIMQLFFSYFAVHFISNSWLMWLIINLVQMAPISAMVRMYIFFASFYYVWKSYVHV
EPI_ISL_424233 1523 LGLAAIMQLFFSYFAVHFISNSWLMWLIINLVQMAPISAMVRMYIFFASFYYVWKSYVHV
EPI_ISL_424236 1523 LGLAAIMQLFFSYFAVHFISNSWLMWLIINLVQMAPISAMVRMYIFFASFYYVWKSYVHV
EPI_ISL_424238 1523 LGLAAIMQLFFSYFAVHFISNSWLMWLIINLVQMAPISAMVRMYIFFASFYYVWKSYVHV
EPI_ISL_424248 1523 LGLAAIMQLFFSYFAVHFISNSWLMWLIINLVQMAPISAMVRMYIFFASFYYVWKSYVHV
EPI_ISL_424254 1523 LGLAAIMQLFFSYFAVHFISNSWLMWLIINLVQMAPISAMVRMYIFFASFYYVWKSYVHV
EPI_ISL_425548 1523 LGLAAIMQLFFSYFAVHFISNSWLMWLIINLVQMAPISAMVRMYIFFASFYYVWKSYVHV
EPI_ISL_425581 1523 LGLAAIMQLFFSYFAVHFISNSWLMWLIINLVQMAPISAMVRMYIFFASFYYVWKSYVHV
EPI_ISL_426881 1523 LGLAAIMQLFFSYFAVHFISNSWLMWLIINLVQMAPISAMVRMYIFFASFYYVWKSYVHV
EPI_ISL_426883 1523 LGLAAIMQLFFSYFAVHFISNSWLMWLIINLVQMAPISAMVRMYIFFASFYYVWKSYVHV
EPI_ISL_426888 1523 LGLAAIMQLFFSYFAVHFISNSWLMWLIINLVQMAPISAMVRMYIFFASFYYVWKSYVHV
EPI_ISL_426900 1523 LGLAAIMQLFFSYFAVHFISNSWLMWLIINLVQMAPISAMVRMYIFFASFYYVWKSYVHV
EPI_ISL_426901 1523 LGLAAIMQLFFSYFAVHFISNSWLMWLIINLVQMAPISAMVRMYIFFASFYYVWKSYVHV
EPI_ISL_426903 1523 LGLAAIMQLFFSYFAVHFISNSWLMWLIINLVQMAPISAMVRMYIFFASFYYVWKSYVHV
EPI_ISL_426904 1523 LGLAAIMQLFFSYFAVHFISNSWLMWLIINLVQMAPISAMVRMYIFFASFYYVWKSYVHV
EPI_ISL_426907 1523 LGLAAIMQLFFSYFAVHFISNSWLMWLIINLVQMAPISAMVRMYIFFASFYYVWKSYVHV
EPI_ISL_426910 1523 LGLAAIMQLFFSYFAVHFISNSWLMWLIINLVQMAPISAMVRMYIFFASFYYVWKSYVHV
```

ORF1ab	2401	VDGCNSSTCMMCYKRNRATRVECTTIVNGVRRSFYVYANGGKGFCKLHNWNCVNCDTFCA
EPI_ISL_413575	1583	VDGCNSSTCMMCYKRNRATRVECTTIVNGVRRSFYVYANGGKGFCKLHNWNCVNCDTFCA
EPI_ISL_413588	1583	VDGCNSSTCMMCYKRNRATRVECTTIVNGVRRSFYVYANGGKGFCKLHNWNCVNCDTFCA
EPI_ISL_413589	1583	VDGCNSSTCMMCYKRNRATRVECTTIVNGVRRSFYVYANGGKGFCKLHNWNCVNCDTFCA
EPI_ISL_424221	1583	VDGCNSSTCMMCYKRNRATRVECTTIVNGVRRSFYVYANGGKGFCKLHNWNCVNCDTFCA
EPI_ISL_424233	1583	VDGCNSSTCMMCYKRNRATRVECTTIVNGVRRSFYVYANGGKGFCKLHNWNCVNCDTFCA
EPI_ISL_424236	1583	VDGCNSSTCMMCYKRNRATRVECTTIVNGVRRSFYVYANGGKGFCKLHNWNCVNCDTFCA
EPI_ISL_424238	1583	VDGCNSSTCMMCYKRNRATRVECTTIVNGVRRSFYVYANGGKGFCKLHNWNCVNCDTFCA
EPI_ISL_424248	1583	VDGCNSSTCMMCYKRNRATRVECTTIVNGVRRSFYVYANGGKGFCKLHNWNCVNCDTFCA
EPI_ISL_424254	1583	VDGCNSSTCMMCYKRNRATRVECTTIVNGVRRSFYVYANGGKGFCKLHNWNCVNCDTFCA
EPI_ISL_425548	1583	VDGCNSSTCMMCYKRNRATRVECTTIVNGVRRSFYVYANGGKGFCKLHNWNCVNCDTFCA
EPI_ISL_425581	1583	VDGCNSSTCMMCYKRNRATRVECTTIVNGVRRSFYVYANGGKGFCKLHNWNCVNCDTFCA
EPI_ISL_426881	1583	VDGCNSSTCMMCYKRNRATRVECTTIVNGVRRSFYVYANGGKGFCKLHNWNCVNCDTFCA
EPI_ISL_426883	1583	VDGCNSSTCMMCYKRNRATRVECTTIVNGVRRSFYVYANGGKGFCKLHNWNCVNCDTFCA
EPI_ISL_426888	1583	VDGCNSSTCMMCYKRNRATRVECTTIVNGVRRSFYVYANGGKGFCKLHNWNCVNCDTFCA
EPI_ISL_426900	1583	VDGCNSSTCMMCYKRNRATRVECTTIVNGVRRSFYVYANGGKGFCKLHNWNCVNCDTFCA
EPI_ISL_426901	1583	VDGCNSSTCMMCYKRNRATRVECTTIVNGVRRSFYVYANGGKGFCKLHNWNCVNCDTFCA
EPI_ISL_426903	1583	VDGCNSSTCMMCYKRNRATRVECTTIVNGVRRSFYVYANGGKGFCKLHNWNCVNCDTFCA
EPI_ISL_426904	1583	VDGCNSSTCMMCYKRNRATRVECTTIVNGVRRSFYVYANGGKGFCKLHNWNCVNCDTFCA
EPI_ISL_426907	1583	VDGCNSSTCMMCYKRNRATRVECTTIVNGVRRSFYVYANGGKGFCKLHNWNCVNCDTFCA
EPI_ISL_426910	1583	VDGCNSSTCMMCYKRNRATRVECTTIVNGVRRSFYVYANGGKGFCKLHNWNCVNCDTFCA
ORF1ab	2401	VDGCNSSTCMMCYKRNRATRVECTTIVNGVRRSFYVYANGGKGFCKLHNWNCVNCDTFCA
EPI_ISL_413575	1583	VDGCNSSTCMMCYKRNRATRVECTTIVNGVRRSFYVYANGGKGFCKLHNWNCVNCDTFCA
EPI_ISL_413588	1583	VDGCNSSTCMMCYKRNRATRVECTTIVNGVRRSFYVYANGGKGFCKLHNWNCVNCDTFCA
EPI_ISL_413589	1583	VDGCNSSTCMMCYKRNRATRVECTTIVNGVRRSFYVYANGGKGFCKLHNWNCVNCDTFCA
EPI_ISL_424221	1583	VDGCNSSTCMMCYKRNRATRVECTTIVNGVRRSFYVYANGGKGFCKLHNWNCVNCDTFCA
EPI_ISL_424233	1583	VDGCNSSTCMMCYKRNRATRVECTTIVNGVRRSFYVYANGGKGFCKLHNWNCVNCDTFCA
EPI_ISL_424236	1583	VDGCNSSTCMMCYKRNRATRVECTTIVNGVRRSFYVYANGGKGFCKLHNWNCVNCDTFCA
EPI_ISL_424238	1583	VDGCNSSTCMMCYKRNRATRVECTTIVNGVRRSFYVYANGGKGFCKLHNWNCVNCDTFCA
EPI_ISL_424248	1583	VDGCNSSTCMMCYKRNRATRVECTTIVNGVRRSFYVYANGGKGFCKLHNWNCVNCDTFCA
EPI_ISL_424254	1583	VDGCNSSTCMMCYKRNRATRVECTTIVNGVRRSFYVYANGGKGFCKLHNWNCVNCDTFCA
EPI_ISL_425548	1583	VDGCNSSTCMMCYKRNRATRVECTTIVNGVRRSFYVYANGGKGFCKLHNWNCVNCDTFCA
EPI_ISL_425581	1583	VDGCNSSTCMMCYKRNRATRVECTTIVNGVRRSFYVYANGGKGFCKLHNWNCVNCDTFCA
EPI_ISL_426881	1583	VDGCNSSTCMMCYKRNRATRVECTTIVNGVRRSFYVYANGGKGFCKLHNWNCVNCDTFCA
EPI_ISL_426883	1583	VDGCNSSTCMMCYKRNRATRVECTTIVNGVRRSFYVYANGGKGFCKLHNWNCVNCDTFCA
EPI_ISL_426888	1583	VDGCNSSTCMMCYKRNRATRVECTTIVNGVRRSFYVYANGGKGFCKLHNWNCVNCDTFCA
EPI_ISL_426900	1583	VDGCNSSTCMMCYKRNRATRVECTTIVNGVRRSFYVYANGGKGFCKLHNWNCVNCDTFCA
EPI_ISL_426901	1583	VDGCNSSTCMMCYKRNRATRVECTTIVNGVRRSFYVYANGGKGFCKLHNWNCVNCDTFCA
EPI_ISL_426903	1583	VDGCNSSTCMMCYKRNRATRVECTTIVNGVRRSFYVYANGGKGFCKLHNWNCVNCDTFCA
EPI_ISL_426904	1583	VDGCNSSTCMMCYKRNRATRVECTTIVNGVRRSFYVYANGGKGFCKLHNWNCVNCDTFCA
EPI_ISL_426907	1583	VDGCNSSTCMMCYKRNRATRVECTTIVNGVRRSFYVYANGGKGFCKLHNWNCVNCDTFCA
EPI_ISL_426910	1583	VDGCNSSTCMMCYKRNRATRVECTTIVNGVRRSFYVYANGGKGFCKLHNWNCVNCDTFCA

```
ORF1ab         2461 GSTFISDEVARDLSLQFKRPINPTDQSSYIVDSVTVKNGSIHLYFDKAGQKTYERHSLSH
EPI_ISL_413575 1643 GSTFISDEVARDLSLQFKRPINPTDQSSYIVDSVTVKNGSIHLYFDKAGQKTYERHSLSH
EPI_ISL_413588 1643 GSTFISDEVARDLSLQFKRPINPTDQSSYIVDSVTVKNGSIHLYFDKAGQKTYERHSLSH
EPI_ISL_413589 1643 GSTFISDEVARDLSLQFKRPINPTDQSSYIVDSVTVKNGSIHLYFDKAGQKTYERHSLSH
EPI_ISL_424221 1643 GSTFISDEVARDLSLQFKRPINPTDQSSYIVDSVTVKNGSIHLYFDKAGQKTYERHSLSH
EPI_ISL_424233 1643 GSTFISDEVARDLSLQFKRPINPTDQSSYIVDSVTVKNGSIHLYFDKAGQKTYERHSLSH
EPI_ISL_424236 1643 GSTFISDEVARDLSLQFKRPINPTDQSSYIVDSVTVKNGSIHLYFDKAGQKTYERHSLSH
EPI_ISL_424238 1643 GSTFISDEVARDLSLQFKRPINPTDQSSYIVDSVTVKNGSIHLYFDKAGQKTYERHSLSH
EPI_ISL_424248 1643 GSTFISDEVARDLSLQFKRPINPTDQSSYIVDSVTVKNGSIHLYFDKAGQKTYERHSLSH
EPI_ISL_424254 1643 GSTFISDEVARDLSLQFKRPINPTDQSSYIVDSVTVKNGSIHLYFDKAGQKTYERHSLSH
EPI_ISL_425548 1643 GSTFISDEVARDLSLQFKRPINPTDQSSYIVDSVTVKNGSIHLYFDKAGQKTYERHSLSH
EPI_ISL_425581 1643 GSTFISDEVARDLSLQFKRPINPTDQSSYIVDSVTVKNGSIHLYFDKAGQKTYERHSLSH
EPI_ISL_426881 1643 GSTFISDEVARDLSLQFKRPINPTDQSSYIVDSVTVKNGSIHLYFDKAGQKTYERHSLSH
EPI_ISL_426883 1643 GSTFISDEVARDLSLQFKRPINPTDQSSYIVDSVTVKNGSIHLYFDKAGQKTYERHSLSH
EPI_ISL_426888 1643 GSTFISDEVARDLSLQFKRPINPTDQSSYIVDSVTVKNGSIHLYFDKAGQKTYERHSLSH
EPI_ISL_426900 1643 GSTFISDEVARDLSLQFKRPINPTDQSSYIVDSVTVKNGSIHLYFDKAGQKTYERHSLSH
EPI_ISL_426901 1643 GSTFISDEVARDLSLQFKRPINPTDQSSYIVDSVTVKNGSIHLYFDKAGQKTYERHSLSH
EPI_ISL_426903 1643 GSTFISDEVARDLSLQFKRPINPTDQSSYIVDSVTVKNGSIHLYFDKAGQKTYERHSLSH
EPI_ISL_426904 1643 GSTFISDEVARDLSLQFKRPINPTDQSSYIVDSVTVKNGSIHLYFDKAGQKTYERHSLSH
EPI_ISL_426907 1643 GSTFISDEVARDLSLQFKRPINPTDQSSYIVDSVTVKNGSIHLYFDKAGQKTYERHSLSH
EPI_ISL_426910 1643 GSTFISDEVARDLSLQFKRPINPTDQSSYIVDSVTVKNGSIHLYFDKAGQKTYERHSLSH
ORF1ab         2461 GSTFISDEVARDLSLQFKRPINPTDQSSYIVDSVTVKNGSIHLYFDKAGQKTYERHSLSH
EPI_ISL_413575 1643 GSTFISDEVARDLSLQFKRPINPTDQSSYIVDSVTVKNGSIHLYFDKAGQKTYERHSLSH
EPI_ISL_413588 1643 GSTFISDEVARDLSLQFKRPINPTDQSSYIVDSVTVKNGSIHLYFDKAGQKTYERHSLSH
EPI_ISL_413589 1643 GSTFISDEVARDLSLQFKRPINPTDQSSYIVDSVTVKNGSIHLYFDKAGQKTYERHSLSH
EPI_ISL_424221 1643 GSTFISDEVARDLSLQFKRPINPTDQSSYIVDSVTVKNGSIHLYFDKAGQKTYERHSLSH
EPI_ISL_424233 1643 GSTFISDEVARDLSLQFKRPINPTDQSSYIVDSVTVKNGSIHLYFDKAGQKTYERHSLSH
EPI_ISL_424236 1643 GSTFISDEVARDLSLQFKRPINPTDQSSYIVDSVTVKNGSIHLYFDKAGQKTYERHSLSH
EPI_ISL_424238 1643 GSTFISDEVARDLSLQFKRPINPTDQSSYIVDSVTVKNGSIHLYFDKAGQKTYERHSLSH
EPI_ISL_424248 1643 GSTFISDEVARDLSLQFKRPINPTDQSSYIVDSVTVKNGSIHLYFDKAGQKTYERHSLSH
EPI_ISL_424254 1643 GSTFISDEVARDLSLQFKRPINPTDQSSYIVDSVTVKNGSIHLYFDKAGQKTYERHSLSH
EPI_ISL_425548 1643 GSTFISDEVARDLSLQFKRPINPTDQSSYIVDSVTVKNGSIHLYFDKAGQKTYERHSLSH
EPI_ISL_425581 1643 GSTFISDEVARDLSLQFKRPINPTDQSSYIVDSVTVKNGSIHLYFDKAGQKTYERHSLSH
EPI_ISL_426881 1643 GSTFISDEVARDLSLQFKRPINPTDQSSYIVDSVTVKNGSIHLYFDKAGQKTYERHSLSH
EPI_ISL_426883 1643 GSTFISDEVARDLSLQFKRPINPTDQSSYIVDSVTVKNGSIHLYFDKAGQKTYERHSLSH
EPI_ISL_426888 1643 GSTFISDEVARDLSLQFKRPINPTDQSSYIVDSVTVKNGSIHLYFDKAGQKTYERHSLSH
EPI_ISL_426900 1643 GSTFISDEVARDLSLQFKRPINPTDQSSYIVDSVTVKNGSIHLYFDKAGQKTYERHSLSH
EPI_ISL_426901 1643 GSTFISDEVARDLSLQFKRPINPTDQSSYIVDSVTVKNGSIHLYFDKAGQKTYERHSLSH
EPI_ISL_426903 1643 GSTFISDEVARDLSLQFKRPINPTDQSSYIVDSVTVKNGSIHLYFDKAGQKTYERHSLSH
EPI_ISL_426904 1643 GSTFISDEVARDLSLQFKRPINPTDQSSYIVDSVTVKNGSIHLYFDKAGQKTYERHSLSH
EPI_ISL_426907 1643 GSTFISDEVARDLSLQFKRPINPTDQSSYIVDSVTVKNGSIHLYFDKAGQKTYERHSLSH
EPI_ISL_426910 1643 GSTFISDEVARDLSLQFKRPINPTDQSSYIVDSVTVKNGSIHLYFDKAGQKTYERHSLSH
```

```
ORF1ab         2521 FVNLDNLRANNTKGSLPINVIVFDGKSKCEESSAKSASVYYSQLMCQPILLLDQALVSDV
EPI_ISL_413575 1703 FVNLDNLRANNTKGSLPINVIVFDGKSKCEESSAKSASVYYSQLMCQPILLLDQALVSDV
EPI_ISL_413588 1703 FVNLDNLRANNTKGSLPINVIVFDGKSKCEESSAKSASVYYSQLMCQPILLLDQALVSDV
EPI_ISL_413589 1703 FVNLDNLRANNTKGSLPINVIVFDGKSKCEESSAKSASVYYSQLMCQPILLLDQALVSDV
EPI_ISL_424221 1703 FVNLDNLRANNTKGSLPINVIVFDGKSKCEESSAKSASVYYSQLMCQPILLLDQALVSDV
EPI_ISL_424233 1703 FVNLDNLRANNTKGSLPINVIVFDGKSKCEESSAKSASVYYSQLMCQPILLLDQALVSDV
EPI_ISL_424236 1703 FVNLDNLRANNTKGSLPINVIVFDGKSKCEESSAKSASVYYSQLMCQPILLLDQALVSDV
EPI_ISL_424238 1703 FVNLDNLRANNTKGSLPINVIVFDGKSKCEESSAKSASVYYSQLMCQPILLLDQALVSDV
EPI_ISL_424248 1703 FVNLDNLRANNTKGSLPINVIVFDGKSKCEESSAKSASVYYSQLMCQPILLLDQALVSDV
EPI_ISL_424254 1703 FVNLDNLRANNTKGSLPINVIVFDGKSKCEESSAKSASVYYSQLMCQPILLLDQALVSDV
EPI_ISL_425548 1703 FVNLDNLRANNTKGSLPINVIVFDGKSKCEESSAKSASVYYSQLMCQPILLLDQALVSDV
EPI_ISL_425581 1703 FVNLDNLRANNTKGSLPINVIVFDGKSKCEESSAKSASVYYSQLMCQPILLLDQALVSDV
EPI_ISL_426881 1703 FVNLDNLRANNTKGSLPINVIVFDGKSKCEESSAKSASVYYSQLMCQPILLLDQALVSDV
EPI_ISL_426883 1703 FVNLDNLRANNTKGSLPINVIVFDGKSKCEESSAKSASVYYSQLMCQPILLLDQALVSDV
EPI_ISL_426888 1703 FVNLDNLRANNTKGSLPINVIVFDGKSKCEESSAKSASVYYSQLMCQPILLLDQALVSDV
EPI_ISL_426900 1703 FVNLDNLRANNTKGSLPINVIVFDGKSKCEESSAKSASVYYSQLMCQPILLLDQALVSDV
EPI_ISL_426901 1703 FVNLDNLRANNTKGSLPINVIVFDGKSKCEESSAKSASVYYSQLMCQPILLLDQALVSDV
EPI_ISL_426903 1703 FVNLDNLRANNTKGSLPINVIVFDGKSKCEESSAKSASVYYSQLMCQPILLLDQALVSDV
EPI_ISL_426904 1703 FVNLDNLRANNTKGSLPINVIVFDGKSKCEESSAKSASVYYSQLMCQPILLLDQALVSDV
EPI_ISL_426907 1703 FVNLDNLRANNTKGSLPVNVIVFDGKSKCEESSAKSASVYYSQLMCQPILLLDQALVSDV
EPI_ISL_426910 1703 FVNLDNLRANNTKGSLPVNVIVFDGKSKCEESSAKSASVYYSQLMCQPILLLDQALVSDV
ORF1ab         2521 FVNLDNLRANNTKGSLPINVIVFDGKSKCEESSAKSASVYYSQLMCQPILLLDQALVSDV
EPI_ISL_413575 1703 FVNLDNLRANNTKGSLPINVIVFDGKSKCEESSAKSASVYYSQLMCQPILLLDQALVSDV
EPI_ISL_413588 1703 FVNLDNLRANNTKGSLPINVIVFDGKSKCEESSAKSASVYYSQLMCQPILLLDQALVSDV
EPI_ISL_413589 1703 FVNLDNLRANNTKGSLPINVIVFDGKSKCEESSAKSASVYYSQLMCQPILLLDQALVSDV
EPI_ISL_424221 1703 FVNLDNLRANNTKGSLPINVIVFDGKSKCEESSAKSASVYYSQLMCQPILLLDQALVSDV
EPI_ISL_424233 1703 FVNLDNLRANNTKGSLPINVIVFDGKSKCEESSAKSASVYYSQLMCQPILLLDQALVSDV
EPI_ISL_424236 1703 FVNLDNLRANNTKGSLPINVIVFDGKSKCEESSAKSASVYYSQLMCQPILLLDQALVSDV
EPI_ISL_424238 1703 FVNLDNLRANNTKGSLPINVIVFDGKSKCEESSAKSASVYYSQLMCQPILLLDQALVSDV
EPI_ISL_424248 1703 FVNLDNLRANNTKGSLPINVIVFDGKSKCEESSAKSASVYYSQLMCQPILLLDQALVSDV
EPI_ISL_424254 1703 FVNLDNLRANNTKGSLPINVIVFDGKSKCEESSAKSASVYYSQLMCQPILLLDQALVSDV
EPI_ISL_425548 1703 FVNLDNLRANNTKGSLPINVIVFDGKSKCEESSAKSASVYYSQLMCQPILLLDQALVSDV
EPI_ISL_425581 1703 FVNLDNLRANNTKGSLPINVIVFDGKSKCEESSAKSASVYYSQLMCQPILLLDQALVSDV
EPI_ISL_426881 1703 FVNLDNLRANNTKGSLPINVIVFDGKSKCEESSAKSASVYYSQLMCQPILLLDQALVSDV
EPI_ISL_426883 1703 FVNLDNLRANNTKGSLPINVIVFDGKSKCEESSAKSASVYYSQLMCQPILLLDQALVSDV
EPI_ISL_426888 1703 FVNLDNLRANNTKGSLPINVIVFDGKSKCEESSAKSASVYYSQLMCQPILLLDQALVSDV
EPI_ISL_426900 1703 FVNLDNLRANNTKGSLPINVIVFDGKSKCEESSAKSASVYYSQLMCQPILLLDQALVSDV
EPI_ISL_426901 1703 FVNLDNLRANNTKGSLPINVIVFDGKSKCEESSAKSASVYYSQLMCQPILLLDQALVSDV
EPI_ISL_426903 1703 FVNLDNLRANNTKGSLPINVIVFDGKSKCEESSAKSASVYYSQLMCQPILLLDQALVSDV
EPI_ISL_426904 1703 FVNLDNLRANNTKGSLPINVIVFDGKSKCEESSAKSASVYYSQLMCQPILLLDQALVSDV
EPI_ISL_426907 1703 FVNLDNLRANNTKGSLPVNVIVFDGKSKCEESSAKSASVYYSQLMCQPILLLDQALVSDV
EPI_ISL_426910 1703 FVNLDNLRANNTKGSLPVNVIVFDGKSKCEESSAKSASVYYSQLMCQPILLLDQALVSDV
```

```
ORF1ab         2581 GDSAEVAVKMFDAYVNTFSSTFNVPMEKLKTLVATAEAELAKNVSLDNVLSTFISAARQG
EPI_ISL_413575 1763 GDSAEVAVKMFDAYVNTFSSTFNVPMEKLKTLVATAEAELAKNVSLDNVLSTFISAARQG
EPI_ISL_413588 1763 GDSAEVAVKMFDAYVNTFSSTFNVPMEKLKTLVATAEAELAKNVSLDNVLSTFISAARQG
EPI_ISL_413589 1763 GDSAEVAVKMFDAYVNTFSSTFNVPMEKLKTLVATAEAELAKNVSLDNVLSTFISAARQG
EPI_ISL_424221 1763 GDSAEVAVKMFDAYVNTFSSTFNVPMEKLKTLVATAEAELAKNVSLDNVLSTFISAARQG
EPI_ISL_424233 1763 GDSAEVAVKMFDAYVNTFSSTFNVPMEKLKTLVATAEAELAKNVSLDNVLSTFISAARQG
EPI_ISL_424236 1763 GDSAEVAVKMFDAYVNTFSSTFNVPMEKLKTLVATAEAELAKNVSLDNVLSTFISAARQG
EPI_ISL_424238 1763 GDSAEVAVKMFDAYVNTFSSTFNVPMEKLKTLVATAEAELAKNVSLDNVLSTFISAARQG
EPI_ISL_424248 1763 GDSAEVAVKMFDAYVNTFSSTFNVPMEKLKTLVATAEAELAKNVSLDNVLSTFISAARQG
EPI_ISL_424254 1763 GDSAEVAVKMFDAYVNTFSSTFNVPMEKLKTLVATAEAELAKNVSLDNVLSTFISAARQG
EPI_ISL_425548 1763 GDSAEVAVKMFDAYVNTFSSTFNVPMEKLKTLVATAEAELAKNVSLDNVLSTFISAARQG
EPI_ISL_425581 1763 GDSAEVAVKMFDAYVNTFSSTFNVPMEKLKTLVATAEAELAKNVSLDNVLSTFISAARQG
EPI_ISL_426881 1763 GDSAEVAVKMFDAYVNTFSSTFNVPMEKLKTLVATAEAELAKNVSLDNVLSTFISAARQG
EPI_ISL_426883 1763 GDSAEVAVKMFDAYVNTFSSTFNVPMEKLKTLVATAEAELAKNVSLDNVLSTFISAARQG
EPI_ISL_426888 1763 GDSAEVAVKMFDAYVNTFSSTFNVPMEKLKTLVATAEAELAKNVSLDNVLSTFISAARQG
EPI_ISL_426900 1763 GDSAEVAVKMFDAYVNTFSSTFNVPMEKLKTLVATAEAELAKNVSLDNVLSTFISAARQG
EPI_ISL_426901 1763 GDSAEVAVKMFDAYVNTFSSTFNVPMEKLKTLVATAEAELAKNVSLDNVLSTFISAARQG
EPI_ISL_426903 1763 GDSAEVAVKMFDAYVNTFSSTFNVPMEKLKTLVATAEAELAKNVSLDNVLSTFISAARQG
EPI_ISL_426904 1763 GDSAEVAVKMFDAYVNTFSSTFNVPMEKLKTLVATAEAELAKNVSLDNVLSTFISAARQG
EPI_ISL_426907 1763 GDSAEVAVKMFDAYVNTFSSTFNVPMEKLKTLVATAEAELAKNVSLDNVLSTFISAARQG
EPI_ISL_426910 1763 GDSAEVAVKMFDAYVNTFSSTFNVPMEKLKTLVATAEAELAKNVSLDNVLSTFISAARQG
ORF1ab         2581 GDSAEVAVKMFDAYVNTFSSTFNVPMEKLKTLVATAEAELAKNVSLDNVLSTFISAARQG
EPI_ISL_413575 1763 GDSAEVAVKMFDAYVNTFSSTFNVPMEKLKTLVATAEAELAKNVSLDNVLSTFISAARQG
EPI_ISL_413588 1763 GDSAEVAVKMFDAYVNTFSSTFNVPMEKLKTLVATAEAELAKNVSLDNVLSTFISAARQG
EPI_ISL_413589 1763 GDSAEVAVKMFDAYVNTFSSTFNVPMEKLKTLVATAEAELAKNVSLDNVLSTFISAARQG
EPI_ISL_424221 1763 GDSAEVAVKMFDAYVNTFSSTFNVPMEKLKTLVATAEAELAKNVSLDNVLSTFISAARQG
EPI_ISL_424233 1763 GDSAEVAVKMFDAYVNTFSSTFNVPMEKLKTLVATAEAELAKNVSLDNVLSTFISAARQG
EPI_ISL_424236 1763 GDSAEVAVKMFDAYVNTFSSTFNVPMEKLKTLVATAEAELAKNVSLDNVLSTFISAARQG
EPI_ISL_424238 1763 GDSAEVAVKMFDAYVNTFSSTFNVPMEKLKTLVATAEAELAKNVSLDNVLSTFISAARQG
EPI_ISL_424248 1763 GDSAEVAVKMFDAYVNTFSSTFNVPMEKLKTLVATAEAELAKNVSLDNVLSTFISAARQG
EPI_ISL_424254 1763 GDSAEVAVKMFDAYVNTFSSTFNVPMEKLKTLVATAEAELAKNVSLDNVLSTFISAARQG
EPI_ISL_425548 1763 GDSAEVAVKMFDAYVNTFSSTFNVPMEKLKTLVATAEAELAKNVSLDNVLSTFISAARQG
EPI_ISL_425581 1763 GDSAEVAVKMFDAYVNTFSSTFNVPMEKLKTLVATAEAELAKNVSLDNVLSTFISAARQG
EPI_ISL_426881 1763 GDSAEVAVKMFDAYVNTFSSTFNVPMEKLKTLVATAEAELAKNVSLDNVLSTFISAARQG
EPI_ISL_426883 1763 GDSAEVAVKMFDAYVNTFSSTFNVPMEKLKTLVATAEAELAKNVSLDNVLSTFISAARQG
EPI_ISL_426888 1763 GDSAEVAVKMFDAYVNTFSSTFNVPMEKLKTLVATAEAELAKNVSLDNVLSTFISAARQG
EPI_ISL_426900 1763 GDSAEVAVKMFDAYVNTFSSTFNVPMEKLKTLVATAEAELAKNVSLDNVLSTFISAARQG
EPI_ISL_426901 1763 GDSAEVAVKMFDAYVNTFSSTFNVPMEKLKTLVATAEAELAKNVSLDNVLSTFISAARQG
EPI_ISL_426903 1763 GDSAEVAVKMFDAYVNTFSSTFNVPMEKLKTLVATAEAELAKNVSLDNVLSTFISAARQG
EPI_ISL_426904 1763 GDSAEVAVKMFDAYVNTFSSTFNVPMEKLKTLVATAEAELAKNVSLDNVLSTFISAARQG
EPI_ISL_426907 1763 GDSAEVAVKMFDAYVNTFSSTFNVPMEKLKTLVATAEAELAKNVSLDNVLSTFISAARQG
EPI_ISL_426910 1763 GDSAEVAVKMFDAYVNTFSSTFNVPMEKLKTLVATAEAELAKNVSLDNVLSTFISAARQG
```

```
ORF1ab         2641 FVDSDVETKDVVECLKLSHQSDIEVTGDSCNNYMLTYNKVENMTPRDLGACIDCSARHIN
EPI_ISL_413575 1823 FVDSDVETKDVVECLKLSHQSDIEVTGDSCNNYMLTYNKVENMTPRDLGACIDCSARHIN
EPI_ISL_413588 1823 FVDSDVETKDVVECLKLSHQSDIEVTGDSCNNYMLTYNKVENMTPRDLGACIDCSARHIN
EPI_ISL_413589 1823 FVDSDVETKDVVECLKLSHQSDIEVTGDSCNNYMLTYNKVENMTPRDLGACIDCSARHIN
EPI_ISL_424221 1823 FVDSDVETKDVVECLKLSHQSDIEVTGDSCNNYMLTYNKVENMTPRDLGACIDCSARHIN
EPI_ISL_424233 1823 FVDSDVETKDVVECLKLSHQSDIEVTGDSCNNYMLTYNKVENMTPRDLGACIDCSARHIN
EPI_ISL_424236 1823 FVDSDVETKDVVECLKLSHQSDIEVTGDSCNNYMLTYNKVENMTPRDLGACIDCSARHIN
EPI_ISL_424238 1823 FVDSDVETKDVVECLKLSHQSDIEVTGDSCNNYMLTYNKVENMTPRDLGACIDCSARHIN
EPI_ISL_424248 1823 FVDSDVETKDVVECLKLSHQSDIEVTGDSCNNYMLTYNKVENMTPRDLGACIDCSARHIN
EPI_ISL_424254 1823 FVDSDVETKDVVECLKLSHQSDIEVTGDSCNNYMLTYNKVENMTPRDLGACIDCSARHIN
EPI_ISL_425548 1823 FVDSDVETKDVVECLKLSHQSDIEVTGDSCNNYMLTYNKVENMTPRDLGACIDCSARHIN
EPI_ISL_425581 1823 FVDSDVETKDVVECLKLSHQSDIEVTGDSCNNYMLTYNKVENMTPRDLGACIDCSARHIN
EPI_ISL_426881 1823 FVDSDVETKDVVECLKLSHQSDIEVTGDSCNNYMLTYNKVENMTPRDLGACIDCSARHIN
EPI_ISL_426883 1823 FVDSDVETKDVVECLKLSHQSDIEVTGDSCNNYMLTYNKVENMTPRDLGACIDCSARHIN
EPI_ISL_426888 1823 FVDSDVETKDVVECLKLSHQSDIEVTGDSCNNYMLTYNKVENMTPRDLGACIDCSARHIN
EPI_ISL_426900 1823 FVDSDVETKDVVECLKLSHQSDIEVTGDSCNNYMLTYNKVENMTPRDLGACIDCSARHIN
EPI_ISL_426901 1823 FVDSDVETKDVVECLKLSHQSDIEVTGDSCNNYMLTYNKVENMTPRDLGACIDCSARHIN
EPI_ISL_426903 1823 FVDSDVETKDVVECLKLSHQSDIEVTGDSCNNYMLTYNKVENMTPRDLGACIDCSARHIN
EPI_ISL_426904 1823 FVDSDVETKDVVECLKLSHQSDIEVTGDSCNNYMLTYNKVENMTPRDLGACIDCSARHIN
EPI_ISL_426907 1823 FVDSDVETKDVVECLKLSHQSDIEVTGDSCNNYMLTYNKVENMTPRDLGACIDCSARHIN
EPI_ISL_426910 1823 FVDSDVETKDVVECLKLSHQSDIEVTGDSCNNYMLTYNKVENMTPRDLGACIDCSARHIN
ORF1ab         2641 FVDSDVETKDVVECLKLSHQSDIEVTGDSCNNYMLTYNKVENMTPRDLGACIDCSARHIN
EPI_ISL_413575 1823 FVDSDVETKDVVECLKLSHQSDIEVTGDSCNNYMLTYNKVENMTPRDLGACIDCSARHIN
EPI_ISL_413588 1823 FVDSDVETKDVVECLKLSHQSDIEVTGDSCNNYMLTYNKVENMTPRDLGACIDCSARHIN
EPI_ISL_413589 1823 FVDSDVETKDVVECLKLSHQSDIEVTGDSCNNYMLTYNKVENMTPRDLGACIDCSARHIN
EPI_ISL_424221 1823 FVDSDVETKDVVECLKLSHQSDIEVTGDSCNNYMLTYNKVENMTPRDLGACIDCSARHIN
EPI_ISL_424233 1823 FVDSDVETKDVVECLKLSHQSDIEVTGDSCNNYMLTYNKVENMTPRDLGACIDCSARHIN
EPI_ISL_424236 1823 FVDSDVETKDVVECLKLSHQSDIEVTGDSCNNYMLTYNKVENMTPRDLGACIDCSARHIN
EPI_ISL_424238 1823 FVDSDVETKDVVECLKLSHQSDIEVTGDSCNNYMLTYNKVENMTPRDLGACIDCSARHIN
EPI_ISL_424248 1823 FVDSDVETKDVVECLKLSHQSDIEVTGDSCNNYMLTYNKVENMTPRDLGACIDCSARHIN
EPI_ISL_424254 1823 FVDSDVETKDVVECLKLSHQSDIEVTGDSCNNYMLTYNKVENMTPRDLGACIDCSARHIN
EPI_ISL_425548 1823 FVDSDVETKDVVECLKLSHQSDIEVTGDSCNNYMLTYNKVENMTPRDLGACIDCSARHIN
EPI_ISL_425581 1823 FVDSDVETKDVVECLKLSHQSDIEVTGDSCNNYMLTYNKVENMTPRDLGACIDCSARHIN
EPI_ISL_426881 1823 FVDSDVETKDVVECLKLSHQSDIEVTGDSCNNYMLTYNKVENMTPRDLGACIDCSARHIN
EPI_ISL_426883 1823 FVDSDVETKDVVECLKLSHQSDIEVTGDSCNNYMLTYNKVENMTPRDLGACIDCSARHIN
EPI_ISL_426888 1823 FVDSDVETKDVVECLKLSHQSDIEVTGDSCNNYMLTYNKVENMTPRDLGACIDCSARHIN
EPI_ISL_426900 1823 FVDSDVETKDVVECLKLSHQSDIEVTGDSCNNYMLTYNKVENMTPRDLGACIDCSARHIN
EPI_ISL_426901 1823 FVDSDVETKDVVECLKLSHQSDIEVTGDSCNNYMLTYNKVENMTPRDLGACIDCSARHIN
EPI_ISL_426903 1823 FVDSDVETKDVVECLKLSHQSDIEVTGDSCNNYMLTYNKVENMTPRDLGACIDCSARHIN
EPI_ISL_426904 1823 FVDSDVETKDVVECLKLSHQSDIEVTGDSCNNYMLTYNKVENMTPRDLGACIDCSARHIN
EPI_ISL_426907 1823 FVDSDVETKDVVECLKLSHQSDIEVTGDSCNNYMLTYNKVENMTPRDLGACIDCSARHIN
EPI_ISL_426910 1823 FVDSDVETKDVVECLKLSHQSDIEVTGDSCNNYMLTYNKVENMTPRDLGACIDCSARHIN
```

```
ORF1ab         2701 AQVAKSHNIALIWNVKDFMSLSEQLRKQIRSAAKKNNLPFKLTCATTRQVVNVVTTKIAL
EPI_ISL_413575 1883 AQVAKSHNIALIWNVKDFMSLSEQLRKQIRSAAKKNNLPFKLTCATTRQVVNVVTTKIAL
EPI_ISL_413588 1883 AQVAKSHNIALIWNVKDFMSLSEQLRKQIRSAAKKNNLPFKLTCATTRQVVNVVTTKIAL
EPI_ISL_413589 1883 AQVAKSHNIALIWNVKDFMSLSEQLRKQIRSAAKKNNLPFKLTCATTRQVVNVVTTKIAL
EPI_ISL_424221 1883 AQVAKSHNIALIWNVKDFMSLSEQLRKQIRSAAKKNNLPFKLTCATTRQVVNVVTTKIAL
EPI_ISL_424233 1883 AQVAKSHNIALIWNVKDFMSLSEQLRKQIRSAAKKNNLPFKLTCATTRQVVNVVTTKIAL
EPI_ISL_424236 1883 AQVAKSHNIALIWNVKDFMSLSEQLRKQIRSAAKKNNLPFKLTCATTRQVVNVVTTKIAL
EPI_ISL_424238 1883 AQVAKSHNIALIWNVKDFMSLSEQLRKQIRSAAKKNNLPFKLTCATTRQVVNVVTTKIAL
EPI_ISL_424248 1883 AQVAKSHNIALIWNVKDFMSLSEQLRKQIRSAAKKNNLPFKLTCATTRQVVNVVTTKIAL
EPI_ISL_424254 1883 AQVAKSHNIALIWNVKDFMSLSEQLRKQIRSAAKKNNLPFKLTCATTRQVVNVVTTKIAL
EPI_ISL_425548 1883 AQVAKSHNIALIWNVKDFMSLSEQLRKQIRSAAKKNNLPFKLTCATTRQVVNVVTTKIAL
EPI_ISL_425581 1883 AQVAKSHNIALIWNVKDFMSLSEQLRKQIRSAAKKNNLPFKLTCATTRQVVNVVTTKIAL
EPI_ISL_426881 1883 AQVAKSHNIALIWNVKDFMSLSEQLRKQIRSAAKKNNLPFKLTCATTRQVVNVVTTKIAL
EPI_ISL_426883 1883 AQVAKSHNIALIWNVKDFMSLSEQLRKQIRSAAKKNNLPFKLTCATTRQVVNVVTTKIAL
EPI_ISL_426888 1883 AQVAKSHNIALIWNVKDFMSLSEQLRKQIRSAAKKNNLPFKLTCATTRQVVNVVTTKIAL
EPI_ISL_426900 1883 AQVAKSHNIALIWNVKDFMSLSEQLRKQIRSAAKKNNLPFKLTCATTRQVVNVVTTKIAL
EPI_ISL_426901 1883 AQVAKSHNIALIWNVKDFMSLSEQLRKQIRSAAKKNNLPFKLTCATTRQVVNVVTTKIAL
EPI_ISL_426903 1883 AQVAKSHNIALIWNVKDFMSLSEQLRKQIRSAAKKNNLPFKLTCATTRQVVNVVTTKIAL
EPI_ISL_426904 1883 AQVAKSHNIALIWNVKDFMSLSEQLRKQIRSAAKKNNLPFKLTCATTRQVVNVVTTKIAL
EPI_ISL_426907 1883 AQVAKSHNIALIWNVKDFMSLSEQLRKQIRSAAKKNNLPFKLTCATTRQVVNVVTTKIAL
EPI_ISL_426910 1883 AQVAKSHNIALIWNVKDFMSLSEQLRKQIRSAAKKNNLPFKLTCATTRQVVNVVTTKIAL
ORF1ab         2701 AQVAKSHNIALIWNVKDFMSLSEQLRKQIRSAAKKNNLPFKLTCATTRQVVNVVTTKIAL
EPI_ISL_413575 1883 AQVAKSHNIALIWNVKDFMSLSEQLRKQIRSAAKKNNLPFKLTCATTRQVVNVVTTKIAL
EPI_ISL_413588 1883 AQVAKSHNIALIWNVKDFMSLSEQLRKQIRSAAKKNNLPFKLTCATTRQVVNVVTTKIAL
EPI_ISL_413589 1883 AQVAKSHNIALIWNVKDFMSLSEQLRKQIRSAAKKNNLPFKLTCATTRQVVNVVTTKIAL
EPI_ISL_424221 1883 AQVAKSHNIALIWNVKDFMSLSEQLRKQIRSAAKKNNLPFKLTCATTRQVVNVVTTKIAL
EPI_ISL_424233 1883 AQVAKSHNIALIWNVKDFMSLSEQLRKQIRSAAKKNNLPFKLTCATTRQVVNVVTTKIAL
EPI_ISL_424236 1883 AQVAKSHNIALIWNVKDFMSLSEQLRKQIRSAAKKNNLPFKLTCATTRQVVNVVTTKIAL
EPI_ISL_424238 1883 AQVAKSHNIALIWNVKDFMSLSEQLRKQIRSAAKKNNLPFKLTCATTRQVVNVVTTKIAL
EPI_ISL_424248 1883 AQVAKSHNIALIWNVKDFMSLSEQLRKQIRSAAKKNNLPFKLTCATTRQVVNVVTTKIAL
EPI_ISL_424254 1883 AQVAKSHNIALIWNVKDFMSLSEQLRKQIRSAAKKNNLPFKLTCATTRQVVNVVTTKIAL
EPI_ISL_425548 1883 AQVAKSHNIALIWNVKDFMSLSEQLRKQIRSAAKKNNLPFKLTCATTRQVVNVVTTKIAL
EPI_ISL_425581 1883 AQVAKSHNIALIWNVKDFMSLSEQLRKQIRSAAKKNNLPFKLTCATTRQVVNVVTTKIAL
EPI_ISL_426881 1883 AQVAKSHNIALIWNVKDFMSLSEQLRKQIRSAAKKNNLPFKLTCATTRQVVNVVTTKIAL
EPI_ISL_426883 1883 AQVAKSHNIALIWNVKDFMSLSEQLRKQIRSAAKKNNLPFKLTCATTRQVVNVVTTKIAL
EPI_ISL_426888 1883 AQVAKSHNIALIWNVKDFMSLSEQLRKQIRSAAKKNNLPFKLTCATTRQVVNVVTTKIAL
EPI_ISL_426900 1883 AQVAKSHNIALIWNVKDFMSLSEQLRKQIRSAAKKNNLPFKLTCATTRQVVNVVTTKIAL
EPI_ISL_426901 1883 AQVAKSHNIALIWNVKDFMSLSEQLRKQIRSAAKKNNLPFKLTCATTRQVVNVVTTKIAL
EPI_ISL_426903 1883 AQVAKSHNIALIWNVKDFMSLSEQLRKQIRSAAKKNNLPFKLTCATTRQVVNVVTTKIAL
EPI_ISL_426904 1883 AQVAKSHNIALIWNVKDFMSLSEQLRKQIRSAAKKNNLPFKLTCATTRQVVNVVTTKIAL
EPI_ISL_426907 1883 AQVAKSHNIALIWNVKDFMSLSEQLRKQIRSAAKKNNLPFKLTCATTRQVVNVVTTKIAL
EPI_ISL_426910 1883 AQVAKSHNIALIWNVKDFMSLSEQLRKQIRSAAKKNNLPFKLTCATTRQVVNVVTTKIAL
```

```
ORF1ab         2761 KGGKIVNNWLKQLIKVTLVFLFVAAIFYLITPVHVMSKHTDFSSEIIGYKAIDGGVTRDI
EPI_ISL_413575 1943 KGG---------------------------------------------------------
EPI_ISL_413588 1943 KGG---------------------------------------------------------
EPI_ISL_413589 1943 KGG---------------------------------------------------------
EPI_ISL_424221 1943 KGG---------------------------------------------------------
EPI_ISL_424233 1943 KGG---------------------------------------------------------
EPI_ISL_424236 1943 KGG---------------------------------------------------------
EPI_ISL_424238 1943 KGG---------------------------------------------------------
EPI_ISL_424248 1943 KGG---------------------------------------------------------
EPI_ISL_424254 1943 KGG---------------------------------------------------------
EPI_ISL_425548 1943 KGG---------------------------------------------------------
EPI_ISL_425581 1943 KGG---------------------------------------------------------
EPI_ISL_426881 1943 KGG---------------------------------------------------------
EPI_ISL_426883 1943 KGG---------------------------------------------------------
EPI_ISL_426888 1943 KGG---------------------------------------------------------
EPI_ISL_426900 1943 KGG---------------------------------------------------------
EPI_ISL_426901 1943 KGG---------------------------------------------------------
EPI_ISL_426903 1943 KGG---------------------------------------------------------
EPI_ISL_426904 1943 KGG---------------------------------------------------------
EPI_ISL_426907 1943 KGG---------------------------------------------------------
EPI_ISL_426910 1943 KGG---------------------------------------------------------
ORF1ab         2761 KGGKIVNNWLKQLIKVTLVFLFVAAIFYLITPVHVMSKHTDFSSEIIGYKAIDGGVTRDI
EPI_ISL_413575 1943 KGG---------------------------------------------------------
EPI_ISL_413588 1943 KGG---------------------------------------------------------
EPI_ISL_413589 1943 KGG---------------------------------------------------------
EPI_ISL_424221 1943 KGG---------------------------------------------------------
EPI_ISL_424233 1943 KGG---------------------------------------------------------
EPI_ISL_424236 1943 KGG---------------------------------------------------------
EPI_ISL_424238 1943 KGG---------------------------------------------------------
EPI_ISL_424248 1943 KGG---------------------------------------------------------
EPI_ISL_424254 1943 KGG---------------------------------------------------------
EPI_ISL_425548 1943 KGG---------------------------------------------------------
EPI_ISL_425581 1943 KGG---------------------------------------------------------
EPI_ISL_426881 1943 KGG---------------------------------------------------------
EPI_ISL_426883 1943 KGG---------------------------------------------------------
EPI_ISL_426888 1943 KGG---------------------------------------------------------
EPI_ISL_426900 1943 KGG---------------------------------------------------------
EPI_ISL_426901 1943 KGG---------------------------------------------------------
EPI_ISL_426903 1943 KGG---------------------------------------------------------
EPI_ISL_426904 1943 KGG---------------------------------------------------------
EPI_ISL_426907 1943 KGG---------------------------------------------------------
EPI_ISL_426910 1943 KGG---------------------------------------------------------
```

```
ORF1ab          2821 ASTDTCFANKHADFDTWFSQRGGSYTNDKACPLIAAVITREVGFVVPGLPGTILRTTNGD
EPI_ISL_413575       ------------------------------------------------------------
EPI_ISL_413588       ------------------------------------------------------------
EPI_ISL_413589       ------------------------------------------------------------
EPI_ISL_424221       ------------------------------------------------------------
EPI_ISL_424233       ------------------------------------------------------------
EPI_ISL_424236       ------------------------------------------------------------
EPI_ISL_424238       ------------------------------------------------------------
EPI_ISL_424248       ------------------------------------------------------------
EPI_ISL_424254       ------------------------------------------------------------
EPI_ISL_425548       ------------------------------------------------------------
EPI_ISL_425581       ------------------------------------------------------------
EPI_ISL_426881       ------------------------------------------------------------
EPI_ISL_426883       ------------------------------------------------------------
EPI_ISL_426888       ------------------------------------------------------------
EPI_ISL_426900       ------------------------------------------------------------
EPI_ISL_426901       ------------------------------------------------------------
EPI_ISL_426903       ------------------------------------------------------------
EPI_ISL_426904       ------------------------------------------------------------
EPI_ISL_426907       ------------------------------------------------------------
EPI_ISL_426910       ------------------------------------------------------------
ORF1ab          2821 ASTDTCFANKHADFDTWFSQRGGSYTNDKACPLIAAVITREVGFVVPGLPGTILRTTNGD
EPI_ISL_413575       ------------------------------------------------------------
EPI_ISL_413588       ------------------------------------------------------------
EPI_ISL_413589       ------------------------------------------------------------
EPI_ISL_424221       ------------------------------------------------------------
EPI_ISL_424233       ------------------------------------------------------------
EPI_ISL_424236       ------------------------------------------------------------
EPI_ISL_424238       ------------------------------------------------------------
EPI_ISL_424248       ------------------------------------------------------------
EPI_ISL_424254       ------------------------------------------------------------
EPI_ISL_425548       ------------------------------------------------------------
EPI_ISL_425581       ------------------------------------------------------------
EPI_ISL_426881       ------------------------------------------------------------
EPI_ISL_426883       ------------------------------------------------------------
EPI_ISL_426888       ------------------------------------------------------------
EPI_ISL_426900       ------------------------------------------------------------
EPI_ISL_426901       ------------------------------------------------------------
EPI_ISL_426903       ------------------------------------------------------------
EPI_ISL_426904       ------------------------------------------------------------
EPI_ISL_426907       ------------------------------------------------------------
EPI_ISL_426910       ------------------------------------------------------------
```

```
ORF1ab              2881 FLHFLPRVFSAVGNICYTPSKLIEYTDFATSACVLAAECTIFKDASGKPVPYCYDTNVLE
EPI_ISL_413575           ------------------------------------------------------------
EPI_ISL_413588           ------------------------------------------------------------
EPI_ISL_413589           ------------------------------------------------------------
EPI_ISL_424221           ------------------------------------------------------------
EPI_ISL_424233           ------------------------------------------------------------
EPI_ISL_424236           ------------------------------------------------------------
EPI_ISL_424238           ------------------------------------------------------------
EPI_ISL_424248           ------------------------------------------------------------
EPI_ISL_424254           ------------------------------------------------------------
EPI_ISL_425548           ------------------------------------------------------------
EPI_ISL_425581           ------------------------------------------------------------
EPI_ISL_426881           ------------------------------------------------------------
EPI_ISL_426883           ------------------------------------------------------------
EPI_ISL_426888           ------------------------------------------------------------
EPI_ISL_426900           ------------------------------------------------------------
EPI_ISL_426901           ------------------------------------------------------------
EPI_ISL_426903           ------------------------------------------------------------
EPI_ISL_426904           ------------------------------------------------------------
EPI_ISL_426907           ------------------------------------------------------------
EPI_ISL_426910           ------------------------------------------------------------
```


```
ORF1ab              2881 FLHFLPRVFSAVGNICYTPSKLIEYTDFATSACVLAAECTIFKDASGKPVPYCYDTNVLE
EPI_ISL_413575           ------------------------------------------------------------
EPI_ISL_413588           ------------------------------------------------------------
EPI_ISL_413589           ------------------------------------------------------------
EPI_ISL_424221           ------------------------------------------------------------
EPI_ISL_424233           ------------------------------------------------------------
EPI_ISL_424236           ------------------------------------------------------------
EPI_ISL_424238           ------------------------------------------------------------
EPI_ISL_424248           ------------------------------------------------------------
EPI_ISL_424254           ------------------------------------------------------------
EPI_ISL_425548           ------------------------------------------------------------
EPI_ISL_425581           ------------------------------------------------------------
EPI_ISL_426881           ------------------------------------------------------------
EPI_ISL_426883           ------------------------------------------------------------
EPI_ISL_426888           ------------------------------------------------------------
EPI_ISL_426900           ------------------------------------------------------------
EPI_ISL_426901           ------------------------------------------------------------
EPI_ISL_426903           ------------------------------------------------------------
EPI_ISL_426904           ------------------------------------------------------------
EPI_ISL_426907           ------------------------------------------------------------
EPI_ISL_426910           ------------------------------------------------------------
```

```
ORF1ab         2941 GSVAYESLRPDTRYVLMDGSIIQFPNTYLEGSVRVVTTFDSEYCRHGTCERSEAGVCVST
EPI_ISL_413575      ------------------------------------------------------------
EPI_ISL_413588      ------------------------------------------------------------
EPI_ISL_413589      ------------------------------------------------------------
EPI_ISL_424221      ------------------------------------------------------------
EPI_ISL_424233      ------------------------------------------------------------
EPI_ISL_424236      ------------------------------------------------------------
EPI_ISL_424238      ------------------------------------------------------------
EPI_ISL_424248      ------------------------------------------------------------
EPI_ISL_424254      ------------------------------------------------------------
EPI_ISL_425548      ------------------------------------------------------------
EPI_ISL_425581      ------------------------------------------------------------
EPI_ISL_426881      ------------------------------------------------------------
EPI_ISL_426883      ------------------------------------------------------------
EPI_ISL_426888      ------------------------------------------------------------
EPI_ISL_426900      ------------------------------------------------------------
EPI_ISL_426901      ------------------------------------------------------------
EPI_ISL_426903      ------------------------------------------------------------
EPI_ISL_426904      ------------------------------------------------------------
EPI_ISL_426907      ------------------------------------------------------------
EPI_ISL_426910      ------------------------------------------------------------
```

```
ORF1ab         2941 GSVAYESLRPDTRYVLMDGSIIQFPNTYLEGSVRVVTTFDSEYCRHGTCERSEAGVCVST
EPI_ISL_413575      ------------------------------------------------------------
EPI_ISL_413588      ------------------------------------------------------------
EPI_ISL_413589      ------------------------------------------------------------
EPI_ISL_424221      ------------------------------------------------------------
EPI_ISL_424233      ------------------------------------------------------------
EPI_ISL_424236      ------------------------------------------------------------
EPI_ISL_424238      ------------------------------------------------------------
EPI_ISL_424248      ------------------------------------------------------------
EPI_ISL_424254      ------------------------------------------------------------
EPI_ISL_425548      ------------------------------------------------------------
EPI_ISL_425581      ------------------------------------------------------------
EPI_ISL_426881      ------------------------------------------------------------
EPI_ISL_426883      ------------------------------------------------------------
EPI_ISL_426888      ------------------------------------------------------------
EPI_ISL_426900      ------------------------------------------------------------
EPI_ISL_426901      ------------------------------------------------------------
EPI_ISL_426903      ------------------------------------------------------------
EPI_ISL_426904      ------------------------------------------------------------
EPI_ISL_426907      ------------------------------------------------------------
EPI_ISL_426910      ------------------------------------------------------------
```

```
ORF1ab          3001 SGRWVLNNDYYRSLPGVFCGVDAVNLLTNMFTPLIQPIGALDISASIVAGGIVAIVVTCL
EPI_ISL_413575       ------------------------------------------------------------
EPI_ISL_413588       ------------------------------------------------------------
EPI_ISL_413589       ------------------------------------------------------------
EPI_ISL_424221       ------------------------------------------------------------
EPI_ISL_424233       ------------------------------------------------------------
EPI_ISL_424236       ------------------------------------------------------------
EPI_ISL_424238       ------------------------------------------------------------
EPI_ISL_424248       ------------------------------------------------------------
EPI_ISL_424254       ------------------------------------------------------------
EPI_ISL_425548       ------------------------------------------------------------
EPI_ISL_425581       ------------------------------------------------------------
EPI_ISL_426881       ------------------------------------------------------------
EPI_ISL_426883       ------------------------------------------------------------
EPI_ISL_426888       ------------------------------------------------------------
EPI_ISL_426900       ------------------------------------------------------------
EPI_ISL_426901       ------------------------------------------------------------
EPI_ISL_426903       ------------------------------------------------------------
EPI_ISL_426904       ------------------------------------------------------------
EPI_ISL_426907       ------------------------------------------------------------
EPI_ISL_426910       ------------------------------------------------------------
```

```
ORF1ab          3001 SGRWVLNNDYYRSLPGVFCGVDAVNLLTNMFTPLIQPIGALDISASIVAGGIVAIVVTCL
EPI_ISL_413575       ------------------------------------------------------------
EPI_ISL_413588       ------------------------------------------------------------
EPI_ISL_413589       ------------------------------------------------------------
EPI_ISL_424221       ------------------------------------------------------------
EPI_ISL_424233       ------------------------------------------------------------
EPI_ISL_424236       ------------------------------------------------------------
EPI_ISL_424238       ------------------------------------------------------------
EPI_ISL_424248       ------------------------------------------------------------
EPI_ISL_424254       ------------------------------------------------------------
EPI_ISL_425548       ------------------------------------------------------------
EPI_ISL_425581       ------------------------------------------------------------
EPI_ISL_426881       ------------------------------------------------------------
EPI_ISL_426883       ------------------------------------------------------------
EPI_ISL_426888       ------------------------------------------------------------
EPI_ISL_426900       ------------------------------------------------------------
EPI_ISL_426901       ------------------------------------------------------------
EPI_ISL_426903       ------------------------------------------------------------
EPI_ISL_426904       ------------------------------------------------------------
EPI_ISL_426907       ------------------------------------------------------------
EPI_ISL_426910       ------------------------------------------------------------
```

```
ORF1ab         3061 AYYFMRFRRAFGEYSHVVAFNTLLFLMSFTVLCLTPVYSFLPGVYSVIYLYLTFYLTNDV
EPI_ISL_413575      ------------------------------------------------------------
EPI_ISL_413588      ------------------------------------------------------------
EPI_ISL_413589      ------------------------------------------------------------
EPI_ISL_424221      ------------------------------------------------------------
EPI_ISL_424233      ------------------------------------------------------------
EPI_ISL_424236      ------------------------------------------------------------
EPI_ISL_424238      ------------------------------------------------------------
EPI_ISL_424248      ------------------------------------------------------------
EPI_ISL_424254      ------------------------------------------------------------
EPI_ISL_425548      ------------------------------------------------------------
EPI_ISL_425581      ------------------------------------------------------------
EPI_ISL_426881      ------------------------------------------------------------
EPI_ISL_426883      ------------------------------------------------------------
EPI_ISL_426888      ------------------------------------------------------------
EPI_ISL_426900      ------------------------------------------------------------
EPI_ISL_426901      ------------------------------------------------------------
EPI_ISL_426903      ------------------------------------------------------------
EPI_ISL_426904      ------------------------------------------------------------
EPI_ISL_426907      ------------------------------------------------------------
EPI_ISL_426910      ------------------------------------------------------------
```


```
ORF1ab         3061 AYYFMRFRRAFGEYSHVVAFNTLLFLMSFTVLCLTPVYSFLPGVYSVIYLYLTFYLTNDV
EPI_ISL_413575      ------------------------------------------------------------
EPI_ISL_413588      ------------------------------------------------------------
EPI_ISL_413589      ------------------------------------------------------------
EPI_ISL_424221      ------------------------------------------------------------
EPI_ISL_424233      ------------------------------------------------------------
EPI_ISL_424236      ------------------------------------------------------------
EPI_ISL_424238      ------------------------------------------------------------
EPI_ISL_424248      ------------------------------------------------------------
EPI_ISL_424254      ------------------------------------------------------------
EPI_ISL_425548      ------------------------------------------------------------
EPI_ISL_425581      ------------------------------------------------------------
EPI_ISL_426881      ------------------------------------------------------------
EPI_ISL_426883      ------------------------------------------------------------
EPI_ISL_426888      ------------------------------------------------------------
EPI_ISL_426900      ------------------------------------------------------------
EPI_ISL_426901      ------------------------------------------------------------
EPI_ISL_426903      ------------------------------------------------------------
EPI_ISL_426904      ------------------------------------------------------------
EPI_ISL_426907      ------------------------------------------------------------
EPI_ISL_426910      ------------------------------------------------------------
```

```
ORF1ab          3121 SFLAHIQWMVMFTPLVPFWITIAYIICISTKHFYWFFSNYLKRRVVFNGVSFSTFEEAAL
EPI_ISL_413575       ------------------------------------------------------------
EPI_ISL_413588       ------------------------------------------------------------
EPI_ISL_413589       ------------------------------------------------------------
EPI_ISL_424221       ------------------------------------------------------------
EPI_ISL_424233       ------------------------------------------------------------
EPI_ISL_424236       ------------------------------------------------------------
EPI_ISL_424238       ------------------------------------------------------------
EPI_ISL_424248       ------------------------------------------------------------
EPI_ISL_424254       ------------------------------------------------------------
EPI_ISL_425548       ------------------------------------------------------------
EPI_ISL_425581       ------------------------------------------------------------
EPI_ISL_426881       ------------------------------------------------------------
EPI_ISL_426883       ------------------------------------------------------------
EPI_ISL_426888       ------------------------------------------------------------
EPI_ISL_426900       ------------------------------------------------------------
EPI_ISL_426901       ------------------------------------------------------------
EPI_ISL_426903       ------------------------------------------------------------
EPI_ISL_426904       ------------------------------------------------------------
EPI_ISL_426907       ------------------------------------------------------------
EPI_ISL_426910       ------------------------------------------------------------
```

```
ORF1ab          3121 SFLAHIQWMVMFTPLVPFWITIAYIICISTKHFYWFFSNYLKRRVVFNGVSFSTFEEAAL
EPI_ISL_413575       ------------------------------------------------------------
EPI_ISL_413588       ------------------------------------------------------------
EPI_ISL_413589       ------------------------------------------------------------
EPI_ISL_424221       ------------------------------------------------------------
EPI_ISL_424233       ------------------------------------------------------------
EPI_ISL_424236       ------------------------------------------------------------
EPI_ISL_424238       ------------------------------------------------------------
EPI_ISL_424248       ------------------------------------------------------------
EPI_ISL_424254       ------------------------------------------------------------
EPI_ISL_425548       ------------------------------------------------------------
EPI_ISL_425581       ------------------------------------------------------------
EPI_ISL_426881       ------------------------------------------------------------
EPI_ISL_426883       ------------------------------------------------------------
EPI_ISL_426888       ------------------------------------------------------------
EPI_ISL_426900       ------------------------------------------------------------
EPI_ISL_426901       ------------------------------------------------------------
EPI_ISL_426903       ------------------------------------------------------------
EPI_ISL_426904       ------------------------------------------------------------
EPI_ISL_426907       ------------------------------------------------------------
EPI_ISL_426910       ------------------------------------------------------------
```

```
ORF1ab         3181 CTFLLNKEMYLKLRSDVLLPLTQYNRYLALYNKYKYFSGAMDTTSYREAACCHLAKALND
EPI_ISL_413575      ------------------------------------------------------------
EPI_ISL_413588      ------------------------------------------------------------
EPI_ISL_413589      ------------------------------------------------------------
EPI_ISL_424221      ------------------------------------------------------------
EPI_ISL_424233      ------------------------------------------------------------
EPI_ISL_424236      ------------------------------------------------------------
EPI_ISL_424238      ------------------------------------------------------------
EPI_ISL_424248      ------------------------------------------------------------
EPI_ISL_424254      ------------------------------------------------------------
EPI_ISL_425548      ------------------------------------------------------------
EPI_ISL_425581      ------------------------------------------------------------
EPI_ISL_426881      ------------------------------------------------------------
EPI_ISL_426883      ------------------------------------------------------------
EPI_ISL_426888      ------------------------------------------------------------
EPI_ISL_426900      ------------------------------------------------------------
EPI_ISL_426901      ------------------------------------------------------------
EPI_ISL_426903      ------------------------------------------------------------
EPI_ISL_426904      ------------------------------------------------------------
EPI_ISL_426907      ------------------------------------------------------------
EPI_ISL_426910      ------------------------------------------------------------
ORF1ab         3181 CTFLLNKEMYLKLRSDVLLPLTQYNRYLALYNKYKYFSGAMDTTSYREAACCHLAKALND
EPI_ISL_413575      ------------------------------------------------------------
EPI_ISL_413588      ------------------------------------------------------------
EPI_ISL_413589      ------------------------------------------------------------
EPI_ISL_424221      ------------------------------------------------------------
EPI_ISL_424233      ------------------------------------------------------------
EPI_ISL_424236      ------------------------------------------------------------
EPI_ISL_424238      ------------------------------------------------------------
EPI_ISL_424248      ------------------------------------------------------------
EPI_ISL_424254      ------------------------------------------------------------
EPI_ISL_425548      ------------------------------------------------------------
EPI_ISL_425581      ------------------------------------------------------------
EPI_ISL_426881      ------------------------------------------------------------
EPI_ISL_426883      ------------------------------------------------------------
EPI_ISL_426888      ------------------------------------------------------------
EPI_ISL_426900      ------------------------------------------------------------
EPI_ISL_426901      ------------------------------------------------------------
EPI_ISL_426903      ------------------------------------------------------------
EPI_ISL_426904      ------------------------------------------------------------
EPI_ISL_426907      ------------------------------------------------------------
EPI_ISL_426910      ------------------------------------------------------------
```

```
ORF1ab         3241 FSNSGSDVLYQPPQTSITSAVLQSGFRKMAFPSGKVEGCMVQVTCGTTTLNGLWLDDVVY
EPI_ISL_413575      ------------------------------------------------------------
EPI_ISL_413588      ------------------------------------------------------------
EPI_ISL_413589      ------------------------------------------------------------
EPI_ISL_424221      ------------------------------------------------------------
EPI_ISL_424233      ------------------------------------------------------------
EPI_ISL_424236      ------------------------------------------------------------
EPI_ISL_424238      ------------------------------------------------------------
EPI_ISL_424248      ------------------------------------------------------------
EPI_ISL_424254      ------------------------------------------------------------
EPI_ISL_425548      ------------------------------------------------------------
EPI_ISL_425581      ------------------------------------------------------------
EPI_ISL_426881      ------------------------------------------------------------
EPI_ISL_426883      ------------------------------------------------------------
EPI_ISL_426888      ------------------------------------------------------------
EPI_ISL_426900      ------------------------------------------------------------
EPI_ISL_426901      ------------------------------------------------------------
EPI_ISL_426903      ------------------------------------------------------------
EPI_ISL_426904      ------------------------------------------------------------
EPI_ISL_426907      ------------------------------------------------------------
EPI_ISL_426910      ------------------------------------------------------------
```


```
ORF1ab         3241 FSNSGSDVLYQPPQTSITSAVLQSGFRKMAFPSGKVEGCMVQVTCGTTTLNGLWLDDVVY
EPI_ISL_413575      ------------------------------------------------------------
EPI_ISL_413588      ------------------------------------------------------------
EPI_ISL_413589      ------------------------------------------------------------
EPI_ISL_424221      ------------------------------------------------------------
EPI_ISL_424233      ------------------------------------------------------------
EPI_ISL_424236      ------------------------------------------------------------
EPI_ISL_424238      ------------------------------------------------------------
EPI_ISL_424248      ------------------------------------------------------------
EPI_ISL_424254      ------------------------------------------------------------
EPI_ISL_425548      ------------------------------------------------------------
EPI_ISL_425581      ------------------------------------------------------------
EPI_ISL_426881      ------------------------------------------------------------
EPI_ISL_426883      ------------------------------------------------------------
EPI_ISL_426888      ------------------------------------------------------------
EPI_ISL_426900      ------------------------------------------------------------
EPI_ISL_426901      ------------------------------------------------------------
EPI_ISL_426903      ------------------------------------------------------------
EPI_ISL_426904      ------------------------------------------------------------
EPI_ISL_426907      ------------------------------------------------------------
EPI_ISL_426910      ------------------------------------------------------------
```

```
ORF1ab         3301 CPRHVICTSEDMLNPNYEDLLIRKSNHNFLVQAGNVQLRVIGHSMQNCVLKLKVDTANPK
EPI_ISL_413575      ------------------------------------------------------------
EPI_ISL_413588      ------------------------------------------------------------
EPI_ISL_413589      ------------------------------------------------------------
EPI_ISL_424221      ------------------------------------------------------------
EPI_ISL_424233      ------------------------------------------------------------
EPI_ISL_424236      ------------------------------------------------------------
EPI_ISL_424238      ------------------------------------------------------------
EPI_ISL_424248      ------------------------------------------------------------
EPI_ISL_424254      ------------------------------------------------------------
EPI_ISL_425548      ------------------------------------------------------------
EPI_ISL_425581      ------------------------------------------------------------
EPI_ISL_426881      ------------------------------------------------------------
EPI_ISL_426883      ------------------------------------------------------------
EPI_ISL_426888      ------------------------------------------------------------
EPI_ISL_426900      ------------------------------------------------------------
EPI_ISL_426901      ------------------------------------------------------------
EPI_ISL_426903      ------------------------------------------------------------
EPI_ISL_426904      ------------------------------------------------------------
EPI_ISL_426907      ------------------------------------------------------------
EPI_ISL_426910      ------------------------------------------------------------
```

```
ORF1ab         3301 CPRHVICTSEDMLNPNYEDLLIRKSNHNFLVQAGNVQLRVIGHSMQNCVLKLKVDTANPK
EPI_ISL_413575      ------------------------------------------------------------
EPI_ISL_413588      ------------------------------------------------------------
EPI_ISL_413589      ------------------------------------------------------------
EPI_ISL_424221      ------------------------------------------------------------
EPI_ISL_424233      ------------------------------------------------------------
EPI_ISL_424236      ------------------------------------------------------------
EPI_ISL_424238      ------------------------------------------------------------
EPI_ISL_424248      ------------------------------------------------------------
EPI_ISL_424254      ------------------------------------------------------------
EPI_ISL_425548      ------------------------------------------------------------
EPI_ISL_425581      ------------------------------------------------------------
EPI_ISL_426881      ------------------------------------------------------------
EPI_ISL_426883      ------------------------------------------------------------
EPI_ISL_426888      ------------------------------------------------------------
EPI_ISL_426900      ------------------------------------------------------------
EPI_ISL_426901      ------------------------------------------------------------
EPI_ISL_426903      ------------------------------------------------------------
EPI_ISL_426904      ------------------------------------------------------------
EPI_ISL_426907      ------------------------------------------------------------
EPI_ISL_426910      ------------------------------------------------------------
```

```
ORF1ab         3361 TPKYKFVRIQPGQTFSVLACYNGSPSGVYQCAMRPNFTIKGSFLNGSCGSVGFNIDYDCV
EPI_ISL_413575      ------------------------------------------------------------
EPI_ISL_413588      ------------------------------------------------------------
EPI_ISL_413589      ------------------------------------------------------------
EPI_ISL_424221      ------------------------------------------------------------
EPI_ISL_424233      ------------------------------------------------------------
EPI_ISL_424236      ------------------------------------------------------------
EPI_ISL_424238      ------------------------------------------------------------
EPI_ISL_424248      ------------------------------------------------------------
EPI_ISL_424254      ------------------------------------------------------------
EPI_ISL_425548      ------------------------------------------------------------
EPI_ISL_425581      ------------------------------------------------------------
EPI_ISL_426881      ------------------------------------------------------------
EPI_ISL_426883      ------------------------------------------------------------
EPI_ISL_426888      ------------------------------------------------------------
EPI_ISL_426900      ------------------------------------------------------------
EPI_ISL_426901      ------------------------------------------------------------
EPI_ISL_426903      ------------------------------------------------------------
EPI_ISL_426904      ------------------------------------------------------------
EPI_ISL_426907      ------------------------------------------------------------
EPI_ISL_426910      ------------------------------------------------------------
ORF1ab         3361 TPKYKFVRIQPGQTFSVLACYNGSPSGVYQCAMRPNFTIKGSFLNGSCGSVGFNIDYDCV
EPI_ISL_413575      ------------------------------------------------------------
EPI_ISL_413588      ------------------------------------------------------------
EPI_ISL_413589      ------------------------------------------------------------
EPI_ISL_424221      ------------------------------------------------------------
EPI_ISL_424233      ------------------------------------------------------------
EPI_ISL_424236      ------------------------------------------------------------
EPI_ISL_424238      ------------------------------------------------------------
EPI_ISL_424248      ------------------------------------------------------------
EPI_ISL_424254      ------------------------------------------------------------
EPI_ISL_425548      ------------------------------------------------------------
EPI_ISL_425581      ------------------------------------------------------------
EPI_ISL_426881      ------------------------------------------------------------
EPI_ISL_426883      ------------------------------------------------------------
EPI_ISL_426888      ------------------------------------------------------------
EPI_ISL_426900      ------------------------------------------------------------
EPI_ISL_426901      ------------------------------------------------------------
EPI_ISL_426903      ------------------------------------------------------------
EPI_ISL_426904      ------------------------------------------------------------
EPI_ISL_426907      ------------------------------------------------------------
EPI_ISL_426910      ------------------------------------------------------------
```

```
ORF1ab         3421 SFCYMHHMELPTGVHAGTDLEGNFYGPFVDRQTAQAAGTDTTITVNVLAWLYAAVINGDR
EPI_ISL_413575      ------------------------------------------------------------
EPI_ISL_413588      ------------------------------------------------------------
EPI_ISL_413589      ------------------------------------------------------------
EPI_ISL_424221      ------------------------------------------------------------
EPI_ISL_424233      ------------------------------------------------------------
EPI_ISL_424236      ------------------------------------------------------------
EPI_ISL_424238      ------------------------------------------------------------
EPI_ISL_424248      ------------------------------------------------------------
EPI_ISL_424254      ------------------------------------------------------------
EPI_ISL_425548      ------------------------------------------------------------
EPI_ISL_425581      ------------------------------------------------------------
EPI_ISL_426881      ------------------------------------------------------------
EPI_ISL_426883      ------------------------------------------------------------
EPI_ISL_426888      ------------------------------------------------------------
EPI_ISL_426900      ------------------------------------------------------------
EPI_ISL_426901      ------------------------------------------------------------
EPI_ISL_426903      ------------------------------------------------------------
EPI_ISL_426904      ------------------------------------------------------------
EPI_ISL_426907      ------------------------------------------------------------
EPI_ISL_426910      ------------------------------------------------------------
ORF1ab         3421 SFCYMHHMELPTGVHAGTDLEGNFYGPFVDRQTAQAAGTDTTITVNVLAWLYAAVINGDR
EPI_ISL_413575      ------------------------------------------------------------
EPI_ISL_413588      ------------------------------------------------------------
EPI_ISL_413589      ------------------------------------------------------------
EPI_ISL_424221      ------------------------------------------------------------
EPI_ISL_424233      ------------------------------------------------------------
EPI_ISL_424236      ------------------------------------------------------------
EPI_ISL_424238      ------------------------------------------------------------
EPI_ISL_424248      ------------------------------------------------------------
EPI_ISL_424254      ------------------------------------------------------------
EPI_ISL_425548      ------------------------------------------------------------
EPI_ISL_425581      ------------------------------------------------------------
EPI_ISL_426881      ------------------------------------------------------------
EPI_ISL_426883      ------------------------------------------------------------
EPI_ISL_426888      ------------------------------------------------------------
EPI_ISL_426900      ------------------------------------------------------------
EPI_ISL_426901      ------------------------------------------------------------
EPI_ISL_426903      ------------------------------------------------------------
EPI_ISL_426904      ------------------------------------------------------------
EPI_ISL_426907      ------------------------------------------------------------
EPI_ISL_426910      ------------------------------------------------------------
```

```
ORF1ab         3481 WFLNRFTTTLNDFNLVAMKYNYEPLTQDHVDILGPLSAQTGIAVLDMCASLKELLQNGMN
EPI_ISL_413575      ------------------------------------------------------------
EPI_ISL_413588      ------------------------------------------------------------
EPI_ISL_413589      ------------------------------------------------------------
EPI_ISL_424221      ------------------------------------------------------------
EPI_ISL_424233      ------------------------------------------------------------
EPI_ISL_424236      ------------------------------------------------------------
EPI_ISL_424238      ------------------------------------------------------------
EPI_ISL_424248      ------------------------------------------------------------
EPI_ISL_424254      ------------------------------------------------------------
EPI_ISL_425548      ------------------------------------------------------------
EPI_ISL_425581      ------------------------------------------------------------
EPI_ISL_426881      ------------------------------------------------------------
EPI_ISL_426883      ------------------------------------------------------------
EPI_ISL_426888      ------------------------------------------------------------
EPI_ISL_426900      ------------------------------------------------------------
EPI_ISL_426901      ------------------------------------------------------------
EPI_ISL_426903      ------------------------------------------------------------
EPI_ISL_426904      ------------------------------------------------------------
EPI_ISL_426907      ------------------------------------------------------------
EPI_ISL_426910      ------------------------------------------------------------
```


```
ORF1ab         3481 WFLNRFTTTLNDFNLVAMKYNYEPLTQDHVDILGPLSAQTGIAVLDMCASLKELLQNGMN
EPI_ISL_413575      ------------------------------------------------------------
EPI_ISL_413588      ------------------------------------------------------------
EPI_ISL_413589      ------------------------------------------------------------
EPI_ISL_424221      ------------------------------------------------------------
EPI_ISL_424233      ------------------------------------------------------------
EPI_ISL_424236      ------------------------------------------------------------
EPI_ISL_424238      ------------------------------------------------------------
EPI_ISL_424248      ------------------------------------------------------------
EPI_ISL_424254      ------------------------------------------------------------
EPI_ISL_425548      ------------------------------------------------------------
EPI_ISL_425581      ------------------------------------------------------------
EPI_ISL_426881      ------------------------------------------------------------
EPI_ISL_426883      ------------------------------------------------------------
EPI_ISL_426888      ------------------------------------------------------------
EPI_ISL_426900      ------------------------------------------------------------
EPI_ISL_426901      ------------------------------------------------------------
EPI_ISL_426903      ------------------------------------------------------------
EPI_ISL_426904      ------------------------------------------------------------
EPI_ISL_426907      ------------------------------------------------------------
EPI_ISL_426910      ------------------------------------------------------------
```

```
ORF1ab          3541 GRTILGSALLEDEFTPFDVVRQCSGVTFQSAVKRTIKGTHHWLLLTILTSLLVLVQSTQW
EPI_ISL_413575       ------------------------------------------------------------
EPI_ISL_413588       ------------------------------------------------------------
EPI_ISL_413589       ------------------------------------------------------------
EPI_ISL_424221       ------------------------------------------------------------
EPI_ISL_424233       ------------------------------------------------------------
EPI_ISL_424236       ------------------------------------------------------------
EPI_ISL_424238       ------------------------------------------------------------
EPI_ISL_424248       ------------------------------------------------------------
EPI_ISL_424254       ------------------------------------------------------------
EPI_ISL_425548       ------------------------------------------------------------
EPI_ISL_425581       ------------------------------------------------------------
EPI_ISL_426881       ------------------------------------------------------------
EPI_ISL_426883       ------------------------------------------------------------
EPI_ISL_426888       ------------------------------------------------------------
EPI_ISL_426900       ------------------------------------------------------------
EPI_ISL_426901       ------------------------------------------------------------
EPI_ISL_426903       ------------------------------------------------------------
EPI_ISL_426904       ------------------------------------------------------------
EPI_ISL_426907       ------------------------------------------------------------
EPI_ISL_426910       ------------------------------------------------------------
```


```
ORF1ab          3541 GRTILGSALLEDEFTPFDVVRQCSGVTFQSAVKRTIKGTHHWLLLTILTSLLVLVQSTQW
EPI_ISL_413575       ------------------------------------------------------------
EPI_ISL_413588       ------------------------------------------------------------
EPI_ISL_413589       ------------------------------------------------------------
EPI_ISL_424221       ------------------------------------------------------------
EPI_ISL_424233       ------------------------------------------------------------
EPI_ISL_424236       ------------------------------------------------------------
EPI_ISL_424238       ------------------------------------------------------------
EPI_ISL_424248       ------------------------------------------------------------
EPI_ISL_424254       ------------------------------------------------------------
EPI_ISL_425548       ------------------------------------------------------------
EPI_ISL_425581       ------------------------------------------------------------
EPI_ISL_426881       ------------------------------------------------------------
EPI_ISL_426883       ------------------------------------------------------------
EPI_ISL_426888       ------------------------------------------------------------
EPI_ISL_426900       ------------------------------------------------------------
EPI_ISL_426901       ------------------------------------------------------------
EPI_ISL_426903       ------------------------------------------------------------
EPI_ISL_426904       ------------------------------------------------------------
EPI_ISL_426907       ------------------------------------------------------------
EPI_ISL_426910       ------------------------------------------------------------
```

```
ORF1ab         3601 SLFFFLYENAFLPFAMGIIAMSAFAMMFVKHKHAFLCLFLLPSLATVAYFNMVYMPASWV
EPI_ISL_413575      ------------------------------------------------------------
EPI_ISL_413588      ------------------------------------------------------------
EPI_ISL_413589      ------------------------------------------------------------
EPI_ISL_424221      ------------------------------------------------------------
EPI_ISL_424233      ------------------------------------------------------------
EPI_ISL_424236      ------------------------------------------------------------
EPI_ISL_424238      ------------------------------------------------------------
EPI_ISL_424248      ------------------------------------------------------------
EPI_ISL_424254      ------------------------------------------------------------
EPI_ISL_425548      ------------------------------------------------------------
EPI_ISL_425581      ------------------------------------------------------------
EPI_ISL_426881      ------------------------------------------------------------
EPI_ISL_426883      ------------------------------------------------------------
EPI_ISL_426888      ------------------------------------------------------------
EPI_ISL_426900      ------------------------------------------------------------
EPI_ISL_426901      ------------------------------------------------------------
EPI_ISL_426903      ------------------------------------------------------------
EPI_ISL_426904      ------------------------------------------------------------
EPI_ISL_426907      ------------------------------------------------------------
EPI_ISL_426910      ------------------------------------------------------------
```


```
ORF1ab         3601 SLFFFLYENAFLPFAMGIIAMSAFAMMFVKHKHAFLCLFLLPSLATVAYFNMVYMPASWV
EPI_ISL_413575      ------------------------------------------------------------
EPI_ISL_413588      ------------------------------------------------------------
EPI_ISL_413589      ------------------------------------------------------------
EPI_ISL_424221      ------------------------------------------------------------
EPI_ISL_424233      ------------------------------------------------------------
EPI_ISL_424236      ------------------------------------------------------------
EPI_ISL_424238      ------------------------------------------------------------
EPI_ISL_424248      ------------------------------------------------------------
EPI_ISL_424254      ------------------------------------------------------------
EPI_ISL_425548      ------------------------------------------------------------
EPI_ISL_425581      ------------------------------------------------------------
EPI_ISL_426881      ------------------------------------------------------------
EPI_ISL_426883      ------------------------------------------------------------
EPI_ISL_426888      ------------------------------------------------------------
EPI_ISL_426900      ------------------------------------------------------------
EPI_ISL_426901      ------------------------------------------------------------
EPI_ISL_426903      ------------------------------------------------------------
EPI_ISL_426904      ------------------------------------------------------------
EPI_ISL_426907      ------------------------------------------------------------
EPI_ISL_426910      ------------------------------------------------------------
```

```
ORF1ab          3661 MRIMTWLDMVDTSLSGFKLKDCVMYASAVVLLILMTARTVYDDGARRVWTLMNVLTLVYK
EPI_ISL_413575       ------------------------------------------------------------
EPI_ISL_413588       ------------------------------------------------------------
EPI_ISL_413589       ------------------------------------------------------------
EPI_ISL_424221       ------------------------------------------------------------
EPI_ISL_424233       ------------------------------------------------------------
EPI_ISL_424236       ------------------------------------------------------------
EPI_ISL_424238       ------------------------------------------------------------
EPI_ISL_424248       ------------------------------------------------------------
EPI_ISL_424254       ------------------------------------------------------------
EPI_ISL_425548       ------------------------------------------------------------
EPI_ISL_425581       ------------------------------------------------------------
EPI_ISL_426881       ------------------------------------------------------------
EPI_ISL_426883       ------------------------------------------------------------
EPI_ISL_426888       ------------------------------------------------------------
EPI_ISL_426900       ------------------------------------------------------------
EPI_ISL_426901       ------------------------------------------------------------
EPI_ISL_426903       ------------------------------------------------------------
EPI_ISL_426904       ------------------------------------------------------------
EPI_ISL_426907       ------------------------------------------------------------
EPI_ISL_426910       ------------------------------------------------------------
ORF1ab          3661 MRIMTWLDMVDTSLSGFKLKDCVMYASAVVLLILMTARTVYDDGARRVWTLMNVLTLVYK
EPI_ISL_413575       ------------------------------------------------------------
EPI_ISL_413588       ------------------------------------------------------------
EPI_ISL_413589       ------------------------------------------------------------
EPI_ISL_424221       ------------------------------------------------------------
EPI_ISL_424233       ------------------------------------------------------------
EPI_ISL_424236       ------------------------------------------------------------
EPI_ISL_424238       ------------------------------------------------------------
EPI_ISL_424248       ------------------------------------------------------------
EPI_ISL_424254       ------------------------------------------------------------
EPI_ISL_425548       ------------------------------------------------------------
EPI_ISL_425581       ------------------------------------------------------------
EPI_ISL_426881       ------------------------------------------------------------
EPI_ISL_426883       ------------------------------------------------------------
EPI_ISL_426888       ------------------------------------------------------------
EPI_ISL_426900       ------------------------------------------------------------
EPI_ISL_426901       ------------------------------------------------------------
EPI_ISL_426903       ------------------------------------------------------------
EPI_ISL_426904       ------------------------------------------------------------
EPI_ISL_426907       ------------------------------------------------------------
EPI_ISL_426910       ------------------------------------------------------------
```

```
ORF1ab         3721 VYYGNALDQAISMWALIISVTSNYSGVVTTVMFLARGIVFMCVEYCPIFFITGNTLQCIM
EPI_ISL_413575      ------------------------------------------------------------
EPI_ISL_413588      ------------------------------------------------------------
EPI_ISL_413589      ------------------------------------------------------------
EPI_ISL_424221      ------------------------------------------------------------
EPI_ISL_424233      ------------------------------------------------------------
EPI_ISL_424236      ------------------------------------------------------------
EPI_ISL_424238      ------------------------------------------------------------
EPI_ISL_424248      ------------------------------------------------------------
EPI_ISL_424254      ------------------------------------------------------------
EPI_ISL_425548      ------------------------------------------------------------
EPI_ISL_425581      ------------------------------------------------------------
EPI_ISL_426881      ------------------------------------------------------------
EPI_ISL_426883      ------------------------------------------------------------
EPI_ISL_426888      ------------------------------------------------------------
EPI_ISL_426900      ------------------------------------------------------------
EPI_ISL_426901      ------------------------------------------------------------
EPI_ISL_426903      ------------------------------------------------------------
EPI_ISL_426904      ------------------------------------------------------------
EPI_ISL_426907      ------------------------------------------------------------
EPI_ISL_426910      ------------------------------------------------------------
ORF1ab         3721 VYYGNALDQAISMWALIISVTSNYSGVVTTVMFLARGIVFMCVEYCPIFFITGNTLQCIM
EPI_ISL_413575      ------------------------------------------------------------
EPI_ISL_413588      ------------------------------------------------------------
EPI_ISL_413589      ------------------------------------------------------------
EPI_ISL_424221      ------------------------------------------------------------
EPI_ISL_424233      ------------------------------------------------------------
EPI_ISL_424236      ------------------------------------------------------------
EPI_ISL_424238      ------------------------------------------------------------
EPI_ISL_424248      ------------------------------------------------------------
EPI_ISL_424254      ------------------------------------------------------------
EPI_ISL_425548      ------------------------------------------------------------
EPI_ISL_425581      ------------------------------------------------------------
EPI_ISL_426881      ------------------------------------------------------------
EPI_ISL_426883      ------------------------------------------------------------
EPI_ISL_426888      ------------------------------------------------------------
EPI_ISL_426900      ------------------------------------------------------------
EPI_ISL_426901      ------------------------------------------------------------
EPI_ISL_426903      ------------------------------------------------------------
EPI_ISL_426904      ------------------------------------------------------------
EPI_ISL_426907      ------------------------------------------------------------
EPI_ISL_426910      ------------------------------------------------------------
```

```
ORF1ab          3781 LVYCFLGYFCTCYFGLFCLLNRYFRLTLGVYDYLVSTQEFRYMNSQGLLPPKNSIDAFKL
EPI_ISL_413575       ------------------------------------------------------------
EPI_ISL_413588       ------------------------------------------------------------
EPI_ISL_413589       ------------------------------------------------------------
EPI_ISL_424221       ------------------------------------------------------------
EPI_ISL_424233       ------------------------------------------------------------
EPI_ISL_424236       ------------------------------------------------------------
EPI_ISL_424238       ------------------------------------------------------------
EPI_ISL_424248       ------------------------------------------------------------
EPI_ISL_424254       ------------------------------------------------------------
EPI_ISL_425548       ------------------------------------------------------------
EPI_ISL_425581       ------------------------------------------------------------
EPI_ISL_426881       ------------------------------------------------------------
EPI_ISL_426883       ------------------------------------------------------------
EPI_ISL_426888       ------------------------------------------------------------
EPI_ISL_426900       ------------------------------------------------------------
EPI_ISL_426901       ------------------------------------------------------------
EPI_ISL_426903       ------------------------------------------------------------
EPI_ISL_426904       ------------------------------------------------------------
EPI_ISL_426907       ------------------------------------------------------------
EPI_ISL_426910       ------------------------------------------------------------
ORF1ab          3781 LVYCFLGYFCTCYFGLFCLLNRYFRLTLGVYDYLVSTQEFRYMNSQGLLPPKNSIDAFKL
EPI_ISL_413575       ------------------------------------------------------------
EPI_ISL_413588       ------------------------------------------------------------
EPI_ISL_413589       ------------------------------------------------------------
EPI_ISL_424221       ------------------------------------------------------------
EPI_ISL_424233       ------------------------------------------------------------
EPI_ISL_424236       ------------------------------------------------------------
EPI_ISL_424238       ------------------------------------------------------------
EPI_ISL_424248       ------------------------------------------------------------
EPI_ISL_424254       ------------------------------------------------------------
EPI_ISL_425548       ------------------------------------------------------------
EPI_ISL_425581       ------------------------------------------------------------
EPI_ISL_426881       ------------------------------------------------------------
EPI_ISL_426883       ------------------------------------------------------------
EPI_ISL_426888       ------------------------------------------------------------
EPI_ISL_426900       ------------------------------------------------------------
EPI_ISL_426901       ------------------------------------------------------------
EPI_ISL_426903       ------------------------------------------------------------
EPI_ISL_426904       ------------------------------------------------------------
EPI_ISL_426907       ------------------------------------------------------------
EPI_ISL_426910       ------------------------------------------------------------
```

```
ORF1ab          3841 NIKLLGVGGKPCIKVATVQSKMSDVKCTSVVLLSVLQQLRVESSSKLWAQCVQLHNDILL
EPI_ISL_413575       ------------------------------------------------------------
EPI_ISL_413588       ------------------------------------------------------------
EPI_ISL_413589       ------------------------------------------------------------
EPI_ISL_424221       ------------------------------------------------------------
EPI_ISL_424233       ------------------------------------------------------------
EPI_ISL_424236       ------------------------------------------------------------
EPI_ISL_424238       ------------------------------------------------------------
EPI_ISL_424248       ------------------------------------------------------------
EPI_ISL_424254       ------------------------------------------------------------
EPI_ISL_425548       ------------------------------------------------------------
EPI_ISL_425581       ------------------------------------------------------------
EPI_ISL_426881       ------------------------------------------------------------
EPI_ISL_426883       ------------------------------------------------------------
EPI_ISL_426888       ------------------------------------------------------------
EPI_ISL_426900       ------------------------------------------------------------
EPI_ISL_426901       ------------------------------------------------------------
EPI_ISL_426903       ------------------------------------------------------------
EPI_ISL_426904       ------------------------------------------------------------
EPI_ISL_426907       ------------------------------------------------------------
EPI_ISL_426910       ------------------------------------------------------------
```

```
ORF1ab          3841 NIKLLGVGGKPCIKVATVQSKMSDVKCTSVVLLSVLQQLRVESSSKLWAQCVQLHNDILL
EPI_ISL_413575       ------------------------------------------------------------
EPI_ISL_413588       ------------------------------------------------------------
EPI_ISL_413589       ------------------------------------------------------------
EPI_ISL_424221       ------------------------------------------------------------
EPI_ISL_424233       ------------------------------------------------------------
EPI_ISL_424236       ------------------------------------------------------------
EPI_ISL_424238       ------------------------------------------------------------
EPI_ISL_424248       ------------------------------------------------------------
EPI_ISL_424254       ------------------------------------------------------------
EPI_ISL_425548       ------------------------------------------------------------
EPI_ISL_425581       ------------------------------------------------------------
EPI_ISL_426881       ------------------------------------------------------------
EPI_ISL_426883       ------------------------------------------------------------
EPI_ISL_426888       ------------------------------------------------------------
EPI_ISL_426900       ------------------------------------------------------------
EPI_ISL_426901       ------------------------------------------------------------
EPI_ISL_426903       ------------------------------------------------------------
EPI_ISL_426904       ------------------------------------------------------------
EPI_ISL_426907       ------------------------------------------------------------
EPI_ISL_426910       ------------------------------------------------------------
```

```
ORF1ab         3901 AKDTTEAFEKMVSLLSVLLSMQGAVDINKLCEEMLDNRATLQAIASEFSSLPSYAAFATA
EPI_ISL_413575      ------------------------------------------------------------
EPI_ISL_413588      ------------------------------------------------------------
EPI_ISL_413589      ------------------------------------------------------------
EPI_ISL_424221      ------------------------------------------------------------
EPI_ISL_424233      ------------------------------------------------------------
EPI_ISL_424236      ------------------------------------------------------------
EPI_ISL_424238      ------------------------------------------------------------
EPI_ISL_424248      ------------------------------------------------------------
EPI_ISL_424254      ------------------------------------------------------------
EPI_ISL_425548      ------------------------------------------------------------
EPI_ISL_425581      ------------------------------------------------------------
EPI_ISL_426881      ------------------------------------------------------------
EPI_ISL_426883      ------------------------------------------------------------
EPI_ISL_426888      ------------------------------------------------------------
EPI_ISL_426900      ------------------------------------------------------------
EPI_ISL_426901      ------------------------------------------------------------
EPI_ISL_426903      ------------------------------------------------------------
EPI_ISL_426904      ------------------------------------------------------------
EPI_ISL_426907      ------------------------------------------------------------
EPI_ISL_426910      ------------------------------------------------------------
```

```
ORF1ab         3901 AKDTTEAFEKMVSLLSVLLSMQGAVDINKLCEEMLDNRATLQAIASEFSSLPSYAAFATA
EPI_ISL_413575      ------------------------------------------------------------
EPI_ISL_413588      ------------------------------------------------------------
EPI_ISL_413589      ------------------------------------------------------------
EPI_ISL_424221      ------------------------------------------------------------
EPI_ISL_424233      ------------------------------------------------------------
EPI_ISL_424236      ------------------------------------------------------------
EPI_ISL_424238      ------------------------------------------------------------
EPI_ISL_424248      ------------------------------------------------------------
EPI_ISL_424254      ------------------------------------------------------------
EPI_ISL_425548      ------------------------------------------------------------
EPI_ISL_425581      ------------------------------------------------------------
EPI_ISL_426881      ------------------------------------------------------------
EPI_ISL_426883      ------------------------------------------------------------
EPI_ISL_426888      ------------------------------------------------------------
EPI_ISL_426900      ------------------------------------------------------------
EPI_ISL_426901      ------------------------------------------------------------
EPI_ISL_426903      ------------------------------------------------------------
EPI_ISL_426904      ------------------------------------------------------------
EPI_ISL_426907      ------------------------------------------------------------
EPI_ISL_426910      ------------------------------------------------------------
```

```
ORF1ab         3961 QEAYEQAVANGDSEVVLKKLKKSLNVAKSEFDRDAAMQRKLEKMADQAMTQMYKQARSED
EPI_ISL_413575      ------------------------------------------------------------
EPI_ISL_413588      ------------------------------------------------------------
EPI_ISL_413589      ------------------------------------------------------------
EPI_ISL_424221      ------------------------------------------------------------
EPI_ISL_424233      ------------------------------------------------------------
EPI_ISL_424236      ------------------------------------------------------------
EPI_ISL_424238      ------------------------------------------------------------
EPI_ISL_424248      ------------------------------------------------------------
EPI_ISL_424254      ------------------------------------------------------------
EPI_ISL_425548      ------------------------------------------------------------
EPI_ISL_425581      ------------------------------------------------------------
EPI_ISL_426881      ------------------------------------------------------------
EPI_ISL_426883      ------------------------------------------------------------
EPI_ISL_426888      ------------------------------------------------------------
EPI_ISL_426900      ------------------------------------------------------------
EPI_ISL_426901      ------------------------------------------------------------
EPI_ISL_426903      ------------------------------------------------------------
EPI_ISL_426904      ------------------------------------------------------------
EPI_ISL_426907      ------------------------------------------------------------
EPI_ISL_426910      ------------------------------------------------------------
ORF1ab         3961 QEAYEQAVANGDSEVVLKKLKKSLNVAKSEFDRDAAMQRKLEKMADQAMTQMYKQARSED
EPI_ISL_413575      ------------------------------------------------------------
EPI_ISL_413588      ------------------------------------------------------------
EPI_ISL_413589      ------------------------------------------------------------
EPI_ISL_424221      ------------------------------------------------------------
EPI_ISL_424233      ------------------------------------------------------------
EPI_ISL_424236      ------------------------------------------------------------
EPI_ISL_424238      ------------------------------------------------------------
EPI_ISL_424248      ------------------------------------------------------------
EPI_ISL_424254      ------------------------------------------------------------
EPI_ISL_425548      ------------------------------------------------------------
EPI_ISL_425581      ------------------------------------------------------------
EPI_ISL_426881      ------------------------------------------------------------
EPI_ISL_426883      ------------------------------------------------------------
EPI_ISL_426888      ------------------------------------------------------------
EPI_ISL_426900      ------------------------------------------------------------
EPI_ISL_426901      ------------------------------------------------------------
EPI_ISL_426903      ------------------------------------------------------------
EPI_ISL_426904      ------------------------------------------------------------
EPI_ISL_426907      ------------------------------------------------------------
EPI_ISL_426910      ------------------------------------------------------------
```

```
ORF1ab         4021 KRAKVTSAMQTMLFTMLRKLDNDALNNIINNARDGCVPLNIIPLTTAAKLMVVIPDYNTY
EPI_ISL_413575      ------------------------------------------------------------
EPI_ISL_413588      ------------------------------------------------------------
EPI_ISL_413589      ------------------------------------------------------------
EPI_ISL_424221      ------------------------------------------------------------
EPI_ISL_424233      ------------------------------------------------------------
EPI_ISL_424236      ------------------------------------------------------------
EPI_ISL_424238      ------------------------------------------------------------
EPI_ISL_424248      ------------------------------------------------------------
EPI_ISL_424254      ------------------------------------------------------------
EPI_ISL_425548      ------------------------------------------------------------
EPI_ISL_425581      ------------------------------------------------------------
EPI_ISL_426881      ------------------------------------------------------------
EPI_ISL_426883      ------------------------------------------------------------
EPI_ISL_426888      ------------------------------------------------------------
EPI_ISL_426900      ------------------------------------------------------------
EPI_ISL_426901      ------------------------------------------------------------
EPI_ISL_426903      ------------------------------------------------------------
EPI_ISL_426904      ------------------------------------------------------------
EPI_ISL_426907      ------------------------------------------------------------
EPI_ISL_426910      ------------------------------------------------------------
```


```
ORF1ab         4021 KRAKVTSAMQTMLFTMLRKLDNDALNNIINNARDGCVPLNIIPLTTAAKLMVVIPDYNTY
EPI_ISL_413575      ------------------------------------------------------------
EPI_ISL_413588      ------------------------------------------------------------
EPI_ISL_413589      ------------------------------------------------------------
EPI_ISL_424221      ------------------------------------------------------------
EPI_ISL_424233      ------------------------------------------------------------
EPI_ISL_424236      ------------------------------------------------------------
EPI_ISL_424238      ------------------------------------------------------------
EPI_ISL_424248      ------------------------------------------------------------
EPI_ISL_424254      ------------------------------------------------------------
EPI_ISL_425548      ------------------------------------------------------------
EPI_ISL_425581      ------------------------------------------------------------
EPI_ISL_426881      ------------------------------------------------------------
EPI_ISL_426883      ------------------------------------------------------------
EPI_ISL_426888      ------------------------------------------------------------
EPI_ISL_426900      ------------------------------------------------------------
EPI_ISL_426901      ------------------------------------------------------------
EPI_ISL_426903      ------------------------------------------------------------
EPI_ISL_426904      ------------------------------------------------------------
EPI_ISL_426907      ------------------------------------------------------------
EPI_ISL_426910      ------------------------------------------------------------
```

```
ORF1ab         4081 KNTCDGTTFTYASALWEIQQVVDADSKIVQLSEISMDNSPNLAWPLIVTALRANSAVKLQ
EPI_ISL_413575      ------------------------------------------------------------
EPI_ISL_413588      ------------------------------------------------------------
EPI_ISL_413589      ------------------------------------------------------------
EPI_ISL_424221      ------------------------------------------------------------
EPI_ISL_424233      ------------------------------------------------------------
EPI_ISL_424236      ------------------------------------------------------------
EPI_ISL_424238      ------------------------------------------------------------
EPI_ISL_424248      ------------------------------------------------------------
EPI_ISL_424254      ------------------------------------------------------------
EPI_ISL_425548      ------------------------------------------------------------
EPI_ISL_425581      ------------------------------------------------------------
EPI_ISL_426881      ------------------------------------------------------------
EPI_ISL_426883      ------------------------------------------------------------
EPI_ISL_426888      ------------------------------------------------------------
EPI_ISL_426900      ------------------------------------------------------------
EPI_ISL_426901      ------------------------------------------------------------
EPI_ISL_426903      ------------------------------------------------------------
EPI_ISL_426904      ------------------------------------------------------------
EPI_ISL_426907      ------------------------------------------------------------
EPI_ISL_426910      ------------------------------------------------------------
```


```
ORF1ab         4081 KNTCDGTTFTYASALWEIQQVVDADSKIVQLSEISMDNSPNLAWPLIVTALRANSAVKLQ
EPI_ISL_413575      ------------------------------------------------------------
EPI_ISL_413588      ------------------------------------------------------------
EPI_ISL_413589      ------------------------------------------------------------
EPI_ISL_424221      ------------------------------------------------------------
EPI_ISL_424233      ------------------------------------------------------------
EPI_ISL_424236      ------------------------------------------------------------
EPI_ISL_424238      ------------------------------------------------------------
EPI_ISL_424248      ------------------------------------------------------------
EPI_ISL_424254      ------------------------------------------------------------
EPI_ISL_425548      ------------------------------------------------------------
EPI_ISL_425581      ------------------------------------------------------------
EPI_ISL_426881      ------------------------------------------------------------
EPI_ISL_426883      ------------------------------------------------------------
EPI_ISL_426888      ------------------------------------------------------------
EPI_ISL_426900      ------------------------------------------------------------
EPI_ISL_426901      ------------------------------------------------------------
EPI_ISL_426903      ------------------------------------------------------------
EPI_ISL_426904      ------------------------------------------------------------
EPI_ISL_426907      ------------------------------------------------------------
EPI_ISL_426910      ------------------------------------------------------------
```

```
ORF1ab         4141 NNELSPVALRQMSCAAGTTQTACTDDNALAYYNTTKGGRFVLALLSDLQDLKWARFPKSD
EPI_ISL_413575      ------------------------------------------------------------
EPI_ISL_413588      ------------------------------------------------------------
EPI_ISL_413589      ------------------------------------------------------------
EPI_ISL_424221      ------------------------------------------------------------
EPI_ISL_424233      ------------------------------------------------------------
EPI_ISL_424236      ------------------------------------------------------------
EPI_ISL_424238      ------------------------------------------------------------
EPI_ISL_424248      ------------------------------------------------------------
EPI_ISL_424254      ------------------------------------------------------------
EPI_ISL_425548      ------------------------------------------------------------
EPI_ISL_425581      ------------------------------------------------------------
EPI_ISL_426881      ------------------------------------------------------------
EPI_ISL_426883      ------------------------------------------------------------
EPI_ISL_426888      ------------------------------------------------------------
EPI_ISL_426900      ------------------------------------------------------------
EPI_ISL_426901      ------------------------------------------------------------
EPI_ISL_426903      ------------------------------------------------------------
EPI_ISL_426904      ------------------------------------------------------------
EPI_ISL_426907      ------------------------------------------------------------
EPI_ISL_426910      ------------------------------------------------------------
```

```
ORF1ab         4141 NNELSPVALRQMSCAAGTTQTACTDDNALAYYNTTKGGRFVLALLSDLQDLKWARFPKSD
EPI_ISL_413575      ------------------------------------------------------------
EPI_ISL_413588      ------------------------------------------------------------
EPI_ISL_413589      ------------------------------------------------------------
EPI_ISL_424221      ------------------------------------------------------------
EPI_ISL_424233      ------------------------------------------------------------
EPI_ISL_424236      ------------------------------------------------------------
EPI_ISL_424238      ------------------------------------------------------------
EPI_ISL_424248      ------------------------------------------------------------
EPI_ISL_424254      ------------------------------------------------------------
EPI_ISL_425548      ------------------------------------------------------------
EPI_ISL_425581      ------------------------------------------------------------
EPI_ISL_426881      ------------------------------------------------------------
EPI_ISL_426883      ------------------------------------------------------------
EPI_ISL_426888      ------------------------------------------------------------
EPI_ISL_426900      ------------------------------------------------------------
EPI_ISL_426901      ------------------------------------------------------------
EPI_ISL_426903      ------------------------------------------------------------
EPI_ISL_426904      ------------------------------------------------------------
EPI_ISL_426907      ------------------------------------------------------------
EPI_ISL_426910      ------------------------------------------------------------
```

```
ORF1ab        4201 GTGTIYTELEPPCRFVTDTPKGPKVKYLYFIKGLNNLNRGMVLGSLAATVRLQAGNATEV
EPI_ISL_413575     ------------------------------------------------------------
EPI_ISL_413588     ------------------------------------------------------------
EPI_ISL_413589     ------------------------------------------------------------
EPI_ISL_424221     ------------------------------------------------------------
EPI_ISL_424233     ------------------------------------------------------------
EPI_ISL_424236     ------------------------------------------------------------
EPI_ISL_424238     ------------------------------------------------------------
EPI_ISL_424248     ------------------------------------------------------------
EPI_ISL_424254     ------------------------------------------------------------
EPI_ISL_425548     ------------------------------------------------------------
EPI_ISL_425581     ------------------------------------------------------------
EPI_ISL_426881     ------------------------------------------------------------
EPI_ISL_426883     ------------------------------------------------------------
EPI_ISL_426888     ------------------------------------------------------------
EPI_ISL_426900     ------------------------------------------------------------
EPI_ISL_426901     ------------------------------------------------------------
EPI_ISL_426903     ------------------------------------------------------------
EPI_ISL_426904     ------------------------------------------------------------
EPI_ISL_426907     ------------------------------------------------------------
EPI_ISL_426910     ------------------------------------------------------------
```


```
ORF1ab        4201 GTGTIYTELEPPCRFVTDTPKGPKVKYLYFIKGLNNLNRGMVLGSLAATVRLQAGNATEV
EPI_ISL_413575     ------------------------------------------------------------
EPI_ISL_413588     ------------------------------------------------------------
EPI_ISL_413589     ------------------------------------------------------------
EPI_ISL_424221     ------------------------------------------------------------
EPI_ISL_424233     ------------------------------------------------------------
EPI_ISL_424236     ------------------------------------------------------------
EPI_ISL_424238     ------------------------------------------------------------
EPI_ISL_424248     ------------------------------------------------------------
EPI_ISL_424254     ------------------------------------------------------------
EPI_ISL_425548     ------------------------------------------------------------
EPI_ISL_425581     ------------------------------------------------------------
EPI_ISL_426881     ------------------------------------------------------------
EPI_ISL_426883     ------------------------------------------------------------
EPI_ISL_426888     ------------------------------------------------------------
EPI_ISL_426900     ------------------------------------------------------------
EPI_ISL_426901     ------------------------------------------------------------
EPI_ISL_426903     ------------------------------------------------------------
EPI_ISL_426904     ------------------------------------------------------------
EPI_ISL_426907     ------------------------------------------------------------
EPI_ISL_426910     ------------------------------------------------------------
```

```
ORF1ab          4261 PANSTVLSFCAFAVDAAKAYKDYLASGGQPITNCVKMLCTHTGTGQAITVTPEANMDQES
EPI_ISL_413575       ------------------------------------------------------------
EPI_ISL_413588       ------------------------------------------------------------
EPI_ISL_413589       ------------------------------------------------------------
EPI_ISL_424221       ------------------------------------------------------------
EPI_ISL_424233       ------------------------------------------------------------
EPI_ISL_424236       ------------------------------------------------------------
EPI_ISL_424238       ------------------------------------------------------------
EPI_ISL_424248       ------------------------------------------------------------
EPI_ISL_424254       ------------------------------------------------------------
EPI_ISL_425548       ------------------------------------------------------------
EPI_ISL_425581       ------------------------------------------------------------
EPI_ISL_426881       ------------------------------------------------------------
EPI_ISL_426883       ------------------------------------------------------------
EPI_ISL_426888       ------------------------------------------------------------
EPI_ISL_426900       ------------------------------------------------------------
EPI_ISL_426901       ------------------------------------------------------------
EPI_ISL_426903       ------------------------------------------------------------
EPI_ISL_426904       ------------------------------------------------------------
EPI_ISL_426907       ------------------------------------------------------------
EPI_ISL_426910       ------------------------------------------------------------
```

```
ORF1ab          4261 PANSTVLSFCAFAVDAAKAYKDYLASGGQPITNCVKMLCTHTGTGQAITVTPEANMDQES
EPI_ISL_413575       ------------------------------------------------------------
EPI_ISL_413588       ------------------------------------------------------------
EPI_ISL_413589       ------------------------------------------------------------
EPI_ISL_424221       ------------------------------------------------------------
EPI_ISL_424233       ------------------------------------------------------------
EPI_ISL_424236       ------------------------------------------------------------
EPI_ISL_424238       ------------------------------------------------------------
EPI_ISL_424248       ------------------------------------------------------------
EPI_ISL_424254       ------------------------------------------------------------
EPI_ISL_425548       ------------------------------------------------------------
EPI_ISL_425581       ------------------------------------------------------------
EPI_ISL_426881       ------------------------------------------------------------
EPI_ISL_426883       ------------------------------------------------------------
EPI_ISL_426888       ------------------------------------------------------------
EPI_ISL_426900       ------------------------------------------------------------
EPI_ISL_426901       ------------------------------------------------------------
EPI_ISL_426903       ------------------------------------------------------------
EPI_ISL_426904       ------------------------------------------------------------
EPI_ISL_426907       ------------------------------------------------------------
EPI_ISL_426910       ------------------------------------------------------------
```

```
ORF1ab         4321 FGGASCCLYCRCHIDHPNPKGFCDLKGKYVQIPTTCANDPVGFTLKNTVCTVCGMWKGYG
EPI_ISL_413575      ------------------------------------------------------------
EPI_ISL_413588      ------------------------------------------------------------
EPI_ISL_413589      ------------------------------------------------------------
EPI_ISL_424221      ------------------------------------------------------------
EPI_ISL_424233      ------------------------------------------------------------
EPI_ISL_424236      ------------------------------------------------------------
EPI_ISL_424238      ------------------------------------------------------------
EPI_ISL_424248      ------------------------------------------------------------
EPI_ISL_424254      ------------------------------------------------------------
EPI_ISL_425548      ------------------------------------------------------------
EPI_ISL_425581      ------------------------------------------------------------
EPI_ISL_426881      ------------------------------------------------------------
EPI_ISL_426883      ------------------------------------------------------------
EPI_ISL_426888      ------------------------------------------------------------
EPI_ISL_426900      ------------------------------------------------------------
EPI_ISL_426901      ------------------------------------------------------------
EPI_ISL_426903      ------------------------------------------------------------
EPI_ISL_426904      ------------------------------------------------------------
EPI_ISL_426907      ------------------------------------------------------------
EPI_ISL_426910      ------------------------------------------------------------
```

```
ORF1ab         4321 FGGASCCLYCRCHIDHPNPKGFCDLKGKYVQIPTTCANDPVGFTLKNTVCTVCGMWKGYG
EPI_ISL_413575      ------------------------------------------------------------
EPI_ISL_413588      ------------------------------------------------------------
EPI_ISL_413589      ------------------------------------------------------------
EPI_ISL_424221      ------------------------------------------------------------
EPI_ISL_424233      ------------------------------------------------------------
EPI_ISL_424236      ------------------------------------------------------------
EPI_ISL_424238      ------------------------------------------------------------
EPI_ISL_424248      ------------------------------------------------------------
EPI_ISL_424254      ------------------------------------------------------------
EPI_ISL_425548      ------------------------------------------------------------
EPI_ISL_425581      ------------------------------------------------------------
EPI_ISL_426881      ------------------------------------------------------------
EPI_ISL_426883      ------------------------------------------------------------
EPI_ISL_426888      ------------------------------------------------------------
EPI_ISL_426900      ------------------------------------------------------------
EPI_ISL_426901      ------------------------------------------------------------
EPI_ISL_426903      ------------------------------------------------------------
EPI_ISL_426904      ------------------------------------------------------------
EPI_ISL_426907      ------------------------------------------------------------
EPI_ISL_426910      ------------------------------------------------------------
```

```
ORF1ab         4381 CSCDQLREPMLQSADAQSFLNRVCGVSAARLTPCGTGTSTDVVYRAFDIYNDKVAGFAKF
EPI_ISL_413575      ------------------------------------------------------------
EPI_ISL_413588      ------------------------------------------------------------
EPI_ISL_413589      ------------------------------------------------------------
EPI_ISL_424221      ------------------------------------------------------------
EPI_ISL_424233      ------------------------------------------------------------
EPI_ISL_424236      ------------------------------------------------------------
EPI_ISL_424238      ------------------------------------------------------------
EPI_ISL_424248      ------------------------------------------------------------
EPI_ISL_424254      ------------------------------------------------------------
EPI_ISL_425548      ------------------------------------------------------------
EPI_ISL_425581      ------------------------------------------------------------
EPI_ISL_426881      ------------------------------------------------------------
EPI_ISL_426883      ------------------------------------------------------------
EPI_ISL_426888      ------------------------------------------------------------
EPI_ISL_426900      ------------------------------------------------------------
EPI_ISL_426901      ------------------------------------------------------------
EPI_ISL_426903      ------------------------------------------------------------
EPI_ISL_426904      ------------------------------------------------------------
EPI_ISL_426907      ------------------------------------------------------------
EPI_ISL_426910      ------------------------------------------------------------
```

```
ORF1ab         4381 CSCDQLREPMLQSADAQSFLNRVCGVSAARLTPCGTGTSTDVVYRAFDIYNDKVAGFAKF
EPI_ISL_413575      ------------------------------------------------------------
EPI_ISL_413588      ------------------------------------------------------------
EPI_ISL_413589      ------------------------------------------------------------
EPI_ISL_424221      ------------------------------------------------------------
EPI_ISL_424233      ------------------------------------------------------------
EPI_ISL_424236      ------------------------------------------------------------
EPI_ISL_424238      ------------------------------------------------------------
EPI_ISL_424248      ------------------------------------------------------------
EPI_ISL_424254      ------------------------------------------------------------
EPI_ISL_425548      ------------------------------------------------------------
EPI_ISL_425581      ------------------------------------------------------------
EPI_ISL_426881      ------------------------------------------------------------
EPI_ISL_426883      ------------------------------------------------------------
EPI_ISL_426888      ------------------------------------------------------------
EPI_ISL_426900      ------------------------------------------------------------
EPI_ISL_426901      ------------------------------------------------------------
EPI_ISL_426903      ------------------------------------------------------------
EPI_ISL_426904      ------------------------------------------------------------
EPI_ISL_426907      ------------------------------------------------------------
EPI_ISL_426910      ------------------------------------------------------------
```

```
ORF1ab          4441 LKTNCCRFQEKDEDDNLIDSYFVVKRHTFSNYQHEETIYNLLKDCPAVAKHDFFKFRIDG
EPI_ISL_413575       ------------------------------------------------------------
EPI_ISL_413588       ------------------------------------------------------------
EPI_ISL_413589       ------------------------------------------------------------
EPI_ISL_424221       ------------------------------------------------------------
EPI_ISL_424233       ------------------------------------------------------------
EPI_ISL_424236       ------------------------------------------------------------
EPI_ISL_424238       ------------------------------------------------------------
EPI_ISL_424248       ------------------------------------------------------------
EPI_ISL_424254       ------------------------------------------------------------
EPI_ISL_425548       ------------------------------------------------------------
EPI_ISL_425581       ------------------------------------------------------------
EPI_ISL_426881       ------------------------------------------------------------
EPI_ISL_426883       ------------------------------------------------------------
EPI_ISL_426888       ------------------------------------------------------------
EPI_ISL_426900       ------------------------------------------------------------
EPI_ISL_426901       ------------------------------------------------------------
EPI_ISL_426903       ------------------------------------------------------------
EPI_ISL_426904       ------------------------------------------------------------
EPI_ISL_426907       ------------------------------------------------------------
EPI_ISL_426910       ------------------------------------------------------------
ORF1ab          4441 LKTNCCRFQEKDEDDNLIDSYFVVKRHTFSNYQHEETIYNLLKDCPAVAKHDFFKFRIDG
EPI_ISL_413575       ------------------------------------------------------------
EPI_ISL_413588       ------------------------------------------------------------
EPI_ISL_413589       ------------------------------------------------------------
EPI_ISL_424221       ------------------------------------------------------------
EPI_ISL_424233       ------------------------------------------------------------
EPI_ISL_424236       ------------------------------------------------------------
EPI_ISL_424238       ------------------------------------------------------------
EPI_ISL_424248       ------------------------------------------------------------
EPI_ISL_424254       ------------------------------------------------------------
EPI_ISL_425548       ------------------------------------------------------------
EPI_ISL_425581       ------------------------------------------------------------
EPI_ISL_426881       ------------------------------------------------------------
EPI_ISL_426883       ------------------------------------------------------------
EPI_ISL_426888       ------------------------------------------------------------
EPI_ISL_426900       ------------------------------------------------------------
EPI_ISL_426901       ------------------------------------------------------------
EPI_ISL_426903       ------------------------------------------------------------
EPI_ISL_426904       ------------------------------------------------------------
EPI_ISL_426907       ------------------------------------------------------------
EPI_ISL_426910       ------------------------------------------------------------
```

```
ORF1ab         4501 DMVPHISRQRLTKYTMADLVYALRHFDEGNCDTLKEILVTYNCCDDDYFNKKDWYDFVEN
EPI_ISL_413575      ------------------------------------------------------------
EPI_ISL_413588      ------------------------------------------------------------
EPI_ISL_413589      ------------------------------------------------------------
EPI_ISL_424221      ------------------------------------------------------------
EPI_ISL_424233      ------------------------------------------------------------
EPI_ISL_424236      ------------------------------------------------------------
EPI_ISL_424238      ------------------------------------------------------------
EPI_ISL_424248      ------------------------------------------------------------
EPI_ISL_424254      ------------------------------------------------------------
EPI_ISL_425548      ------------------------------------------------------------
EPI_ISL_425581      ------------------------------------------------------------
EPI_ISL_426881      ------------------------------------------------------------
EPI_ISL_426883      ------------------------------------------------------------
EPI_ISL_426888      ------------------------------------------------------------
EPI_ISL_426900      ------------------------------------------------------------
EPI_ISL_426901      ------------------------------------------------------------
EPI_ISL_426903      ------------------------------------------------------------
EPI_ISL_426904      ------------------------------------------------------------
EPI_ISL_426907      ------------------------------------------------------------
EPI_ISL_426910      ------------------------------------------------------------
```

```
ORF1ab         4501 DMVPHISRQRLTKYTMADLVYALRHFDEGNCDTLKEILVTYNCCDDDYFNKKDWYDFVEN
EPI_ISL_413575      ------------------------------------------------------------
EPI_ISL_413588      ------------------------------------------------------------
EPI_ISL_413589      ------------------------------------------------------------
EPI_ISL_424221      ------------------------------------------------------------
EPI_ISL_424233      ------------------------------------------------------------
EPI_ISL_424236      ------------------------------------------------------------
EPI_ISL_424238      ------------------------------------------------------------
EPI_ISL_424248      ------------------------------------------------------------
EPI_ISL_424254      ------------------------------------------------------------
EPI_ISL_425548      ------------------------------------------------------------
EPI_ISL_425581      ------------------------------------------------------------
EPI_ISL_426881      ------------------------------------------------------------
EPI_ISL_426883      ------------------------------------------------------------
EPI_ISL_426888      ------------------------------------------------------------
EPI_ISL_426900      ------------------------------------------------------------
EPI_ISL_426901      ------------------------------------------------------------
EPI_ISL_426903      ------------------------------------------------------------
EPI_ISL_426904      ------------------------------------------------------------
EPI_ISL_426907      ------------------------------------------------------------
EPI_ISL_426910      ------------------------------------------------------------
```

```
ORF1ab         4561 PDILRVYANLGERVRQALLKTVQFCDAMRNAGIVGVLTLDNQDLNGNWYDFGDFIQTTPG
EPI_ISL_413575      ------------------------------------------------------------
EPI_ISL_413588      ------------------------------------------------------------
EPI_ISL_413589      ------------------------------------------------------------
EPI_ISL_424221      ------------------------------------------------------------
EPI_ISL_424233      ------------------------------------------------------------
EPI_ISL_424236      ------------------------------------------------------------
EPI_ISL_424238      ------------------------------------------------------------
EPI_ISL_424248      ------------------------------------------------------------
EPI_ISL_424254      ------------------------------------------------------------
EPI_ISL_425548      ------------------------------------------------------------
EPI_ISL_425581      ------------------------------------------------------------
EPI_ISL_426881      ------------------------------------------------------------
EPI_ISL_426883      ------------------------------------------------------------
EPI_ISL_426888      ------------------------------------------------------------
EPI_ISL_426900      ------------------------------------------------------------
EPI_ISL_426901      ------------------------------------------------------------
EPI_ISL_426903      ------------------------------------------------------------
EPI_ISL_426904      ------------------------------------------------------------
EPI_ISL_426907      ------------------------------------------------------------
EPI_ISL_426910      ------------------------------------------------------------
```

```
ORF1ab         4561 PDILRVYANLGERVRQALLKTVQFCDAMRNAGIVGVLTLDNQDLNGNWYDFGDFIQTTPG
EPI_ISL_413575      ------------------------------------------------------------
EPI_ISL_413588      ------------------------------------------------------------
EPI_ISL_413589      ------------------------------------------------------------
EPI_ISL_424221      ------------------------------------------------------------
EPI_ISL_424233      ------------------------------------------------------------
EPI_ISL_424236      ------------------------------------------------------------
EPI_ISL_424238      ------------------------------------------------------------
EPI_ISL_424248      ------------------------------------------------------------
EPI_ISL_424254      ------------------------------------------------------------
EPI_ISL_425548      ------------------------------------------------------------
EPI_ISL_425581      ------------------------------------------------------------
EPI_ISL_426881      ------------------------------------------------------------
EPI_ISL_426883      ------------------------------------------------------------
EPI_ISL_426888      ------------------------------------------------------------
EPI_ISL_426900      ------------------------------------------------------------
EPI_ISL_426901      ------------------------------------------------------------
EPI_ISL_426903      ------------------------------------------------------------
EPI_ISL_426904      ------------------------------------------------------------
EPI_ISL_426907      ------------------------------------------------------------
EPI_ISL_426910      ------------------------------------------------------------
```

```
ORF1ab         4621 SGVPVVDSYYSLLMPILTLTRALTAESHVDTDLTKPYIKWDLLKYDFTEERLKLFDRYFK
EPI_ISL_413575      ------------------------------------------------------------
EPI_ISL_413588      ------------------------------------------------------------
EPI_ISL_413589      ------------------------------------------------------------
EPI_ISL_424221      ------------------------------------------------------------
EPI_ISL_424233      ------------------------------------------------------------
EPI_ISL_424236      ------------------------------------------------------------
EPI_ISL_424238      ------------------------------------------------------------
EPI_ISL_424248      ------------------------------------------------------------
EPI_ISL_424254      ------------------------------------------------------------
EPI_ISL_425548      ------------------------------------------------------------
EPI_ISL_425581      ------------------------------------------------------------
EPI_ISL_426881      ------------------------------------------------------------
EPI_ISL_426883      ------------------------------------------------------------
EPI_ISL_426888      ------------------------------------------------------------
EPI_ISL_426900      ------------------------------------------------------------
EPI_ISL_426901      ------------------------------------------------------------
EPI_ISL_426903      ------------------------------------------------------------
EPI_ISL_426904      ------------------------------------------------------------
EPI_ISL_426907      ------------------------------------------------------------
EPI_ISL_426910      ------------------------------------------------------------
```


```
ORF1ab         4621 SGVPVVDSYYSLLMPILTLTRALTAESHVDTDLTKPYIKWDLLKYDFTEERLKLFDRYFK
EPI_ISL_413575      ------------------------------------------------------------
EPI_ISL_413588      ------------------------------------------------------------
EPI_ISL_413589      ------------------------------------------------------------
EPI_ISL_424221      ------------------------------------------------------------
EPI_ISL_424233      ------------------------------------------------------------
EPI_ISL_424236      ------------------------------------------------------------
EPI_ISL_424238      ------------------------------------------------------------
EPI_ISL_424248      ------------------------------------------------------------
EPI_ISL_424254      ------------------------------------------------------------
EPI_ISL_425548      ------------------------------------------------------------
EPI_ISL_425581      ------------------------------------------------------------
EPI_ISL_426881      ------------------------------------------------------------
EPI_ISL_426883      ------------------------------------------------------------
EPI_ISL_426888      ------------------------------------------------------------
EPI_ISL_426900      ------------------------------------------------------------
EPI_ISL_426901      ------------------------------------------------------------
EPI_ISL_426903      ------------------------------------------------------------
EPI_ISL_426904      ------------------------------------------------------------
EPI_ISL_426907      ------------------------------------------------------------
EPI_ISL_426910      ------------------------------------------------------------
```

```
ORF1ab         4681 YWDQTYHPNCVNCLDDRCILHCANFNVLFSTVFPPTSFGPLVRKIFVDGVPFVVSTGYHF
EPI_ISL_413575      ------------------------------------------------------------
EPI_ISL_413588      ------------------------------------------------------------
EPI_ISL_413589      ------------------------------------------------------------
EPI_ISL_424221      ------------------------------------------------------------
EPI_ISL_424233      ------------------------------------------------------------
EPI_ISL_424236      ------------------------------------------------------------
EPI_ISL_424238      ------------------------------------------------------------
EPI_ISL_424248      ------------------------------------------------------------
EPI_ISL_424254      ------------------------------------------------------------
EPI_ISL_425548      ------------------------------------------------------------
EPI_ISL_425581      ------------------------------------------------------------
EPI_ISL_426881      ------------------------------------------------------------
EPI_ISL_426883      ------------------------------------------------------------
EPI_ISL_426888      ------------------------------------------------------------
EPI_ISL_426900      ------------------------------------------------------------
EPI_ISL_426901      ------------------------------------------------------------
EPI_ISL_426903      ------------------------------------------------------------
EPI_ISL_426904      ------------------------------------------------------------
EPI_ISL_426907      ------------------------------------------------------------
EPI_ISL_426910      ------------------------------------------------------------
```

```
ORF1ab         4681 YWDQTYHPNCVNCLDDRCILHCANFNVLFSTVFPPTSFGPLVRKIFVDGVPFVVSTGYHF
EPI_ISL_413575      ------------------------------------------------------------
EPI_ISL_413588      ------------------------------------------------------------
EPI_ISL_413589      ------------------------------------------------------------
EPI_ISL_424221      ------------------------------------------------------------
EPI_ISL_424233      ------------------------------------------------------------
EPI_ISL_424236      ------------------------------------------------------------
EPI_ISL_424238      ------------------------------------------------------------
EPI_ISL_424248      ------------------------------------------------------------
EPI_ISL_424254      ------------------------------------------------------------
EPI_ISL_425548      ------------------------------------------------------------
EPI_ISL_425581      ------------------------------------------------------------
EPI_ISL_426881      ------------------------------------------------------------
EPI_ISL_426883      ------------------------------------------------------------
EPI_ISL_426888      ------------------------------------------------------------
EPI_ISL_426900      ------------------------------------------------------------
EPI_ISL_426901      ------------------------------------------------------------
EPI_ISL_426903      ------------------------------------------------------------
EPI_ISL_426904      ------------------------------------------------------------
EPI_ISL_426907      ------------------------------------------------------------
EPI_ISL_426910      ------------------------------------------------------------
```

```
ORF1ab         4741 RELGVVHNQDVNLHSSRLSFKELLVYAADPAMHAASGNLLLDKRTTCFSVAALTNNVAFQ
EPI_ISL_413575      ------------------------------------------------------------
EPI_ISL_413588      ------------------------------------------------------------
EPI_ISL_413589      ------------------------------------------------------------
EPI_ISL_424221      ------------------------------------------------------------
EPI_ISL_424233      ------------------------------------------------------------
EPI_ISL_424236      ------------------------------------------------------------
EPI_ISL_424238      ------------------------------------------------------------
EPI_ISL_424248      ------------------------------------------------------------
EPI_ISL_424254      ------------------------------------------------------------
EPI_ISL_425548      ------------------------------------------------------------
EPI_ISL_425581      ------------------------------------------------------------
EPI_ISL_426881      ------------------------------------------------------------
EPI_ISL_426883      ------------------------------------------------------------
EPI_ISL_426888      ------------------------------------------------------------
EPI_ISL_426900      ------------------------------------------------------------
EPI_ISL_426901      ------------------------------------------------------------
EPI_ISL_426903      ------------------------------------------------------------
EPI_ISL_426904      ------------------------------------------------------------
EPI_ISL_426907      ------------------------------------------------------------
EPI_ISL_426910      ------------------------------------------------------------
```

```
ORF1ab         4741 RELGVVHNQDVNLHSSRLSFKELLVYAADPAMHAASGNLLLDKRTTCFSVAALTNNVAFQ
EPI_ISL_413575      ------------------------------------------------------------
EPI_ISL_413588      ------------------------------------------------------------
EPI_ISL_413589      ------------------------------------------------------------
EPI_ISL_424221      ------------------------------------------------------------
EPI_ISL_424233      ------------------------------------------------------------
EPI_ISL_424236      ------------------------------------------------------------
EPI_ISL_424238      ------------------------------------------------------------
EPI_ISL_424248      ------------------------------------------------------------
EPI_ISL_424254      ------------------------------------------------------------
EPI_ISL_425548      ------------------------------------------------------------
EPI_ISL_425581      ------------------------------------------------------------
EPI_ISL_426881      ------------------------------------------------------------
EPI_ISL_426883      ------------------------------------------------------------
EPI_ISL_426888      ------------------------------------------------------------
EPI_ISL_426900      ------------------------------------------------------------
EPI_ISL_426901      ------------------------------------------------------------
EPI_ISL_426903      ------------------------------------------------------------
EPI_ISL_426904      ------------------------------------------------------------
EPI_ISL_426907      ------------------------------------------------------------
EPI_ISL_426910      ------------------------------------------------------------
```

```
ORF1ab          4801 TVKPGNFNKDFYDFAVSKGFFKEGSSVELKHFFFAQDGNAAISDYDYYRYNLPTMCDIRQ
EPI_ISL_413575       ------------------------------------------------------------
EPI_ISL_413588       ------------------------------------------------------------
EPI_ISL_413589       ------------------------------------------------------------
EPI_ISL_424221       ------------------------------------------------------------
EPI_ISL_424233       ------------------------------------------------------------
EPI_ISL_424236       ------------------------------------------------------------
EPI_ISL_424238       ------------------------------------------------------------
EPI_ISL_424248       ------------------------------------------------------------
EPI_ISL_424254       ------------------------------------------------------------
EPI_ISL_425548       ------------------------------------------------------------
EPI_ISL_425581       ------------------------------------------------------------
EPI_ISL_426881       ------------------------------------------------------------
EPI_ISL_426883       ------------------------------------------------------------
EPI_ISL_426888       ------------------------------------------------------------
EPI_ISL_426900       ------------------------------------------------------------
EPI_ISL_426901       ------------------------------------------------------------
EPI_ISL_426903       ------------------------------------------------------------
EPI_ISL_426904       ------------------------------------------------------------
EPI_ISL_426907       ------------------------------------------------------------
EPI_ISL_426910       ------------------------------------------------------------
```


```
ORF1ab          4801 TVKPGNFNKDFYDFAVSKGFFKEGSSVELKHFFFAQDGNAAISDYDYYRYNLPTMCDIRQ
EPI_ISL_413575       ------------------------------------------------------------
EPI_ISL_413588       ------------------------------------------------------------
EPI_ISL_413589       ------------------------------------------------------------
EPI_ISL_424221       ------------------------------------------------------------
EPI_ISL_424233       ------------------------------------------------------------
EPI_ISL_424236       ------------------------------------------------------------
EPI_ISL_424238       ------------------------------------------------------------
EPI_ISL_424248       ------------------------------------------------------------
EPI_ISL_424254       ------------------------------------------------------------
EPI_ISL_425548       ------------------------------------------------------------
EPI_ISL_425581       ------------------------------------------------------------
EPI_ISL_426881       ------------------------------------------------------------
EPI_ISL_426883       ------------------------------------------------------------
EPI_ISL_426888       ------------------------------------------------------------
EPI_ISL_426900       ------------------------------------------------------------
EPI_ISL_426901       ------------------------------------------------------------
EPI_ISL_426903       ------------------------------------------------------------
EPI_ISL_426904       ------------------------------------------------------------
EPI_ISL_426907       ------------------------------------------------------------
EPI_ISL_426910       ------------------------------------------------------------
```

```
ORF1ab          4861 LLFVVEVVDKYFDCYDGGCINANQVIVNNLDKSAGFPFNKWGKARLYYDSMSYEDQDALF
EPI_ISL_413575       ------------------------------------------------------------
EPI_ISL_413588       ------------------------------------------------------------
EPI_ISL_413589       ------------------------------------------------------------
EPI_ISL_424221       ------------------------------------------------------------
EPI_ISL_424233       ------------------------------------------------------------
EPI_ISL_424236       ------------------------------------------------------------
EPI_ISL_424238       ------------------------------------------------------------
EPI_ISL_424248       ------------------------------------------------------------
EPI_ISL_424254       ------------------------------------------------------------
EPI_ISL_425548       ------------------------------------------------------------
EPI_ISL_425581       ------------------------------------------------------------
EPI_ISL_426881       ------------------------------------------------------------
EPI_ISL_426883       ------------------------------------------------------------
EPI_ISL_426888       ------------------------------------------------------------
EPI_ISL_426900       ------------------------------------------------------------
EPI_ISL_426901       ------------------------------------------------------------
EPI_ISL_426903       ------------------------------------------------------------
EPI_ISL_426904       ------------------------------------------------------------
EPI_ISL_426907       ------------------------------------------------------------
EPI_ISL_426910       ------------------------------------------------------------
```


```
ORF1ab          4861 LLFVVEVVDKYFDCYDGGCINANQVIVNNLDKSAGFPFNKWGKARLYYDSMSYEDQDALF
EPI_ISL_413575       ------------------------------------------------------------
EPI_ISL_413588       ------------------------------------------------------------
EPI_ISL_413589       ------------------------------------------------------------
EPI_ISL_424221       ------------------------------------------------------------
EPI_ISL_424233       ------------------------------------------------------------
EPI_ISL_424236       ------------------------------------------------------------
EPI_ISL_424238       ------------------------------------------------------------
EPI_ISL_424248       ------------------------------------------------------------
EPI_ISL_424254       ------------------------------------------------------------
EPI_ISL_425548       ------------------------------------------------------------
EPI_ISL_425581       ------------------------------------------------------------
EPI_ISL_426881       ------------------------------------------------------------
EPI_ISL_426883       ------------------------------------------------------------
EPI_ISL_426888       ------------------------------------------------------------
EPI_ISL_426900       ------------------------------------------------------------
EPI_ISL_426901       ------------------------------------------------------------
EPI_ISL_426903       ------------------------------------------------------------
EPI_ISL_426904       ------------------------------------------------------------
EPI_ISL_426907       ------------------------------------------------------------
EPI_ISL_426910       ------------------------------------------------------------
```

```
ORF1ab         4921 AYTKRNVIPTITQMNLKYAISAKNRARTVAGVSICSTMTNRQFHQKLLKSIAATRGATVV
EPI_ISL_413575      ------------------------------------------------------------
EPI_ISL_413588      ------------------------------------------------------------
EPI_ISL_413589      ------------------------------------------------------------
EPI_ISL_424221      ------------------------------------------------------------
EPI_ISL_424233      ------------------------------------------------------------
EPI_ISL_424236      ------------------------------------------------------------
EPI_ISL_424238      ------------------------------------------------------------
EPI_ISL_424248      ------------------------------------------------------------
EPI_ISL_424254      ------------------------------------------------------------
EPI_ISL_425548      ------------------------------------------------------------
EPI_ISL_425581      ------------------------------------------------------------
EPI_ISL_426881      ------------------------------------------------------------
EPI_ISL_426883      ------------------------------------------------------------
EPI_ISL_426888      ------------------------------------------------------------
EPI_ISL_426900      ------------------------------------------------------------
EPI_ISL_426901      ------------------------------------------------------------
EPI_ISL_426903      ------------------------------------------------------------
EPI_ISL_426904      ------------------------------------------------------------
EPI_ISL_426907      ------------------------------------------------------------
EPI_ISL_426910      ------------------------------------------------------------
```

```
ORF1ab         4921 AYTKRNVIPTITQMNLKYAISAKNRARTVAGVSICSTMTNRQFHQKLLKSIAATRGATVV
EPI_ISL_413575      ------------------------------------------------------------
EPI_ISL_413588      ------------------------------------------------------------
EPI_ISL_413589      ------------------------------------------------------------
EPI_ISL_424221      ------------------------------------------------------------
EPI_ISL_424233      ------------------------------------------------------------
EPI_ISL_424236      ------------------------------------------------------------
EPI_ISL_424238      ------------------------------------------------------------
EPI_ISL_424248      ------------------------------------------------------------
EPI_ISL_424254      ------------------------------------------------------------
EPI_ISL_425548      ------------------------------------------------------------
EPI_ISL_425581      ------------------------------------------------------------
EPI_ISL_426881      ------------------------------------------------------------
EPI_ISL_426883      ------------------------------------------------------------
EPI_ISL_426888      ------------------------------------------------------------
EPI_ISL_426900      ------------------------------------------------------------
EPI_ISL_426901      ------------------------------------------------------------
EPI_ISL_426903      ------------------------------------------------------------
EPI_ISL_426904      ------------------------------------------------------------
EPI_ISL_426907      ------------------------------------------------------------
EPI_ISL_426910      ------------------------------------------------------------
```

```
ORF1ab         4981 IGTSKFYGGWHNMLKTVYSDVENPHLMGWDYPKCDRAMPNMLRIMASLVLARKHTTCCSL
EPI_ISL_413575      ------------------------------------------------------------
EPI_ISL_413588      ------------------------------------------------------------
EPI_ISL_413589      ------------------------------------------------------------
EPI_ISL_424221      ------------------------------------------------------------
EPI_ISL_424233      ------------------------------------------------------------
EPI_ISL_424236      ------------------------------------------------------------
EPI_ISL_424238      ------------------------------------------------------------
EPI_ISL_424248      ------------------------------------------------------------
EPI_ISL_424254      ------------------------------------------------------------
EPI_ISL_425548      ------------------------------------------------------------
EPI_ISL_425581      ------------------------------------------------------------
EPI_ISL_426881      ------------------------------------------------------------
EPI_ISL_426883      ------------------------------------------------------------
EPI_ISL_426888      ------------------------------------------------------------
EPI_ISL_426900      ------------------------------------------------------------
EPI_ISL_426901      ------------------------------------------------------------
EPI_ISL_426903      ------------------------------------------------------------
EPI_ISL_426904      ------------------------------------------------------------
EPI_ISL_426907      ------------------------------------------------------------
EPI_ISL_426910      ------------------------------------------------------------
```


```
ORF1ab         4981 IGTSKFYGGWHNMLKTVYSDVENPHLMGWDYPKCDRAMPNMLRIMASLVLARKHTTCCSL
EPI_ISL_413575      ------------------------------------------------------------
EPI_ISL_413588      ------------------------------------------------------------
EPI_ISL_413589      ------------------------------------------------------------
EPI_ISL_424221      ------------------------------------------------------------
EPI_ISL_424233      ------------------------------------------------------------
EPI_ISL_424236      ------------------------------------------------------------
EPI_ISL_424238      ------------------------------------------------------------
EPI_ISL_424248      ------------------------------------------------------------
EPI_ISL_424254      ------------------------------------------------------------
EPI_ISL_425548      ------------------------------------------------------------
EPI_ISL_425581      ------------------------------------------------------------
EPI_ISL_426881      ------------------------------------------------------------
EPI_ISL_426883      ------------------------------------------------------------
EPI_ISL_426888      ------------------------------------------------------------
EPI_ISL_426900      ------------------------------------------------------------
EPI_ISL_426901      ------------------------------------------------------------
EPI_ISL_426903      ------------------------------------------------------------
EPI_ISL_426904      ------------------------------------------------------------
EPI_ISL_426907      ------------------------------------------------------------
EPI_ISL_426910      ------------------------------------------------------------
```

```
ORF1ab        5041 SHRFYRLANECAQVLSEMVMCGGSLYVKPGGTSSGDATTAYANSVFNICQAVTANVNALL
EPI_ISL_413575     ------------------------------------------------------------
EPI_ISL_413588     ------------------------------------------------------------
EPI_ISL_413589     ------------------------------------------------------------
EPI_ISL_424221     ------------------------------------------------------------
EPI_ISL_424233     ------------------------------------------------------------
EPI_ISL_424236     ------------------------------------------------------------
EPI_ISL_424238     ------------------------------------------------------------
EPI_ISL_424248     ------------------------------------------------------------
EPI_ISL_424254     ------------------------------------------------------------
EPI_ISL_425548     ------------------------------------------------------------
EPI_ISL_425581     ------------------------------------------------------------
EPI_ISL_426881     ------------------------------------------------------------
EPI_ISL_426883     ------------------------------------------------------------
EPI_ISL_426888     ------------------------------------------------------------
EPI_ISL_426900     ------------------------------------------------------------
EPI_ISL_426901     ------------------------------------------------------------
EPI_ISL_426903     ------------------------------------------------------------
EPI_ISL_426904     ------------------------------------------------------------
EPI_ISL_426907     ------------------------------------------------------------
EPI_ISL_426910     ------------------------------------------------------------
ORF1ab        5041 SHRFYRLANECAQVLSEMVMCGGSLYVKPGGTSSGDATTAYANSVFNICQAVTANVNALL
EPI_ISL_413575     ------------------------------------------------------------
EPI_ISL_413588     ------------------------------------------------------------
EPI_ISL_413589     ------------------------------------------------------------
EPI_ISL_424221     ------------------------------------------------------------
EPI_ISL_424233     ------------------------------------------------------------
EPI_ISL_424236     ------------------------------------------------------------
EPI_ISL_424238     ------------------------------------------------------------
EPI_ISL_424248     ------------------------------------------------------------
EPI_ISL_424254     ------------------------------------------------------------
EPI_ISL_425548     ------------------------------------------------------------
EPI_ISL_425581     ------------------------------------------------------------
EPI_ISL_426881     ------------------------------------------------------------
EPI_ISL_426883     ------------------------------------------------------------
EPI_ISL_426888     ------------------------------------------------------------
EPI_ISL_426900     ------------------------------------------------------------
EPI_ISL_426901     ------------------------------------------------------------
EPI_ISL_426903     ------------------------------------------------------------
EPI_ISL_426904     ------------------------------------------------------------
EPI_ISL_426907     ------------------------------------------------------------
EPI_ISL_426910     ------------------------------------------------------------
```

```
ORF1ab          5101 STDGNKIADKYVRNLQHRLYECLYRNRDVDTDFVNEFYAYLRKHFSMMILSDDAVVCFNS
EPI_ISL_413575       ------------------------------------------------------------
EPI_ISL_413588       ------------------------------------------------------------
EPI_ISL_413589       ------------------------------------------------------------
EPI_ISL_424221       ------------------------------------------------------------
EPI_ISL_424233       ------------------------------------------------------------
EPI_ISL_424236       ------------------------------------------------------------
EPI_ISL_424238       ------------------------------------------------------------
EPI_ISL_424248       ------------------------------------------------------------
EPI_ISL_424254       ------------------------------------------------------------
EPI_ISL_425548       ------------------------------------------------------------
EPI_ISL_425581       ------------------------------------------------------------
EPI_ISL_426881       ------------------------------------------------------------
EPI_ISL_426883       ------------------------------------------------------------
EPI_ISL_426888       ------------------------------------------------------------
EPI_ISL_426900       ------------------------------------------------------------
EPI_ISL_426901       ------------------------------------------------------------
EPI_ISL_426903       ------------------------------------------------------------
EPI_ISL_426904       ------------------------------------------------------------
EPI_ISL_426907       ------------------------------------------------------------
EPI_ISL_426910       ------------------------------------------------------------
```

```
ORF1ab          5101 STDGNKIADKYVRNLQHRLYECLYRNRDVDTDFVNEFYAYLRKHFSMMILSDDAVVCFNS
EPI_ISL_413575       ------------------------------------------------------------
EPI_ISL_413588       ------------------------------------------------------------
EPI_ISL_413589       ------------------------------------------------------------
EPI_ISL_424221       ------------------------------------------------------------
EPI_ISL_424233       ------------------------------------------------------------
EPI_ISL_424236       ------------------------------------------------------------
EPI_ISL_424238       ------------------------------------------------------------
EPI_ISL_424248       ------------------------------------------------------------
EPI_ISL_424254       ------------------------------------------------------------
EPI_ISL_425548       ------------------------------------------------------------
EPI_ISL_425581       ------------------------------------------------------------
EPI_ISL_426881       ------------------------------------------------------------
EPI_ISL_426883       ------------------------------------------------------------
EPI_ISL_426888       ------------------------------------------------------------
EPI_ISL_426900       ------------------------------------------------------------
EPI_ISL_426901       ------------------------------------------------------------
EPI_ISL_426903       ------------------------------------------------------------
EPI_ISL_426904       ------------------------------------------------------------
EPI_ISL_426907       ------------------------------------------------------------
EPI_ISL_426910       ------------------------------------------------------------
```

```
ORF1ab         5161 TYASQGLVASIKNFKSVLYYQNNVFMSEAKCWTETDLTKGPHEFCSQHTMLVKQGDDYVY
EPI_ISL_413575      ------------------------------------------------------------
EPI_ISL_413588      ------------------------------------------------------------
EPI_ISL_413589      ------------------------------------------------------------
EPI_ISL_424221      ------------------------------------------------------------
EPI_ISL_424233      ------------------------------------------------------------
EPI_ISL_424236      ------------------------------------------------------------
EPI_ISL_424238      ------------------------------------------------------------
EPI_ISL_424248      ------------------------------------------------------------
EPI_ISL_424254      ------------------------------------------------------------
EPI_ISL_425548      ------------------------------------------------------------
EPI_ISL_425581      ------------------------------------------------------------
EPI_ISL_426881      ------------------------------------------------------------
EPI_ISL_426883      ------------------------------------------------------------
EPI_ISL_426888      ------------------------------------------------------------
EPI_ISL_426900      ------------------------------------------------------------
EPI_ISL_426901      ------------------------------------------------------------
EPI_ISL_426903      ------------------------------------------------------------
EPI_ISL_426904      ------------------------------------------------------------
EPI_ISL_426907      ------------------------------------------------------------
EPI_ISL_426910      ------------------------------------------------------------
```

```
ORF1ab         5161 TYASQGLVASIKNFKSVLYYQNNVFMSEAKCWTETDLTKGPHEFCSQHTMLVKQGDDYVY
EPI_ISL_413575      ------------------------------------------------------------
EPI_ISL_413588      ------------------------------------------------------------
EPI_ISL_413589      ------------------------------------------------------------
EPI_ISL_424221      ------------------------------------------------------------
EPI_ISL_424233      ------------------------------------------------------------
EPI_ISL_424236      ------------------------------------------------------------
EPI_ISL_424238      ------------------------------------------------------------
EPI_ISL_424248      ------------------------------------------------------------
EPI_ISL_424254      ------------------------------------------------------------
EPI_ISL_425548      ------------------------------------------------------------
EPI_ISL_425581      ------------------------------------------------------------
EPI_ISL_426881      ------------------------------------------------------------
EPI_ISL_426883      ------------------------------------------------------------
EPI_ISL_426888      ------------------------------------------------------------
EPI_ISL_426900      ------------------------------------------------------------
EPI_ISL_426901      ------------------------------------------------------------
EPI_ISL_426903      ------------------------------------------------------------
EPI_ISL_426904      ------------------------------------------------------------
EPI_ISL_426907      ------------------------------------------------------------
EPI_ISL_426910      ------------------------------------------------------------
```

```
ORF1ab          5221 LPYPDPSRILGAGCFVDDIVKTDGTLMIERFVSLAIDAYPLTKHPNQEYADVFHLYLQYI
EPI_ISL_413575       ------------------------------------------------------------
EPI_ISL_413588       ------------------------------------------------------------
EPI_ISL_413589       ------------------------------------------------------------
EPI_ISL_424221       ------------------------------------------------------------
EPI_ISL_424233       ------------------------------------------------------------
EPI_ISL_424236       ------------------------------------------------------------
EPI_ISL_424238       ------------------------------------------------------------
EPI_ISL_424248       ------------------------------------------------------------
EPI_ISL_424254       ------------------------------------------------------------
EPI_ISL_425548       ------------------------------------------------------------
EPI_ISL_425581       ------------------------------------------------------------
EPI_ISL_426881       ------------------------------------------------------------
EPI_ISL_426883       ------------------------------------------------------------
EPI_ISL_426888       ------------------------------------------------------------
EPI_ISL_426900       ------------------------------------------------------------
EPI_ISL_426901       ------------------------------------------------------------
EPI_ISL_426903       ------------------------------------------------------------
EPI_ISL_426904       ------------------------------------------------------------
EPI_ISL_426907       ------------------------------------------------------------
EPI_ISL_426910       ------------------------------------------------------------
```


```
ORF1ab          5221 LPYPDPSRILGAGCFVDDIVKTDGTLMIERFVSLAIDAYPLTKHPNQEYADVFHLYLQYI
EPI_ISL_413575       ------------------------------------------------------------
EPI_ISL_413588       ------------------------------------------------------------
EPI_ISL_413589       ------------------------------------------------------------
EPI_ISL_424221       ------------------------------------------------------------
EPI_ISL_424233       ------------------------------------------------------------
EPI_ISL_424236       ------------------------------------------------------------
EPI_ISL_424238       ------------------------------------------------------------
EPI_ISL_424248       ------------------------------------------------------------
EPI_ISL_424254       ------------------------------------------------------------
EPI_ISL_425548       ------------------------------------------------------------
EPI_ISL_425581       ------------------------------------------------------------
EPI_ISL_426881       ------------------------------------------------------------
EPI_ISL_426883       ------------------------------------------------------------
EPI_ISL_426888       ------------------------------------------------------------
EPI_ISL_426900       ------------------------------------------------------------
EPI_ISL_426901       ------------------------------------------------------------
EPI_ISL_426903       ------------------------------------------------------------
EPI_ISL_426904       ------------------------------------------------------------
EPI_ISL_426907       ------------------------------------------------------------
EPI_ISL_426910       ------------------------------------------------------------
```

```
ORF1ab          5281 RKLHDELTGHMLDMYSVMLTNDNTSRYWEPEFYEAMYTPHTVLQAVGACVLCNSQTSLRC
EPI_ISL_413575       ------------------------------------------------------------
EPI_ISL_413588       ------------------------------------------------------------
EPI_ISL_413589       ------------------------------------------------------------
EPI_ISL_424221       ------------------------------------------------------------
EPI_ISL_424233       ------------------------------------------------------------
EPI_ISL_424236       ------------------------------------------------------------
EPI_ISL_424238       ------------------------------------------------------------
EPI_ISL_424248       ------------------------------------------------------------
EPI_ISL_424254       ------------------------------------------------------------
EPI_ISL_425548       ------------------------------------------------------------
EPI_ISL_425581       ------------------------------------------------------------
EPI_ISL_426881       ------------------------------------------------------------
EPI_ISL_426883       ------------------------------------------------------------
EPI_ISL_426888       ------------------------------------------------------------
EPI_ISL_426900       ------------------------------------------------------------
EPI_ISL_426901       ------------------------------------------------------------
EPI_ISL_426903       ------------------------------------------------------------
EPI_ISL_426904       ------------------------------------------------------------
EPI_ISL_426907       ------------------------------------------------------------
EPI_ISL_426910       ------------------------------------------------------------
```


```
ORF1ab          5281 RKLHDELTGHMLDMYSVMLTNDNTSRYWEPEFYEAMYTPHTVLQAVGACVLCNSQTSLRC
EPI_ISL_413575       ------------------------------------------------------------
EPI_ISL_413588       ------------------------------------------------------------
EPI_ISL_413589       ------------------------------------------------------------
EPI_ISL_424221       ------------------------------------------------------------
EPI_ISL_424233       ------------------------------------------------------------
EPI_ISL_424236       ------------------------------------------------------------
EPI_ISL_424238       ------------------------------------------------------------
EPI_ISL_424248       ------------------------------------------------------------
EPI_ISL_424254       ------------------------------------------------------------
EPI_ISL_425548       ------------------------------------------------------------
EPI_ISL_425581       ------------------------------------------------------------
EPI_ISL_426881       ------------------------------------------------------------
EPI_ISL_426883       ------------------------------------------------------------
EPI_ISL_426888       ------------------------------------------------------------
EPI_ISL_426900       ------------------------------------------------------------
EPI_ISL_426901       ------------------------------------------------------------
EPI_ISL_426903       ------------------------------------------------------------
EPI_ISL_426904       ------------------------------------------------------------
EPI_ISL_426907       ------------------------------------------------------------
EPI_ISL_426910       ------------------------------------------------------------
```

```
ORF1ab         5341 GACIRRPFLCCKCCYDHVISTSHKLVLSVNPYVCNAPGCDVTDVTQLYLGGMSYYCKSHK
EPI_ISL_413575      ------------------------------------------------------------
EPI_ISL_413588      ------------------------------------------------------------
EPI_ISL_413589      ------------------------------------------------------------
EPI_ISL_424221      ------------------------------------------------------------
EPI_ISL_424233      ------------------------------------------------------------
EPI_ISL_424236      ------------------------------------------------------------
EPI_ISL_424238      ------------------------------------------------------------
EPI_ISL_424248      ------------------------------------------------------------
EPI_ISL_424254      ------------------------------------------------------------
EPI_ISL_425548      ------------------------------------------------------------
EPI_ISL_425581      ------------------------------------------------------------
EPI_ISL_426881      ------------------------------------------------------------
EPI_ISL_426883      ------------------------------------------------------------
EPI_ISL_426888      ------------------------------------------------------------
EPI_ISL_426900      ------------------------------------------------------------
EPI_ISL_426901      ------------------------------------------------------------
EPI_ISL_426903      ------------------------------------------------------------
EPI_ISL_426904      ------------------------------------------------------------
EPI_ISL_426907      ------------------------------------------------------------
EPI_ISL_426910      ------------------------------------------------------------
```

```
ORF1ab         5341 GACIRRPFLCCKCCYDHVISTSHKLVLSVNPYVCNAPGCDVTDVTQLYLGGMSYYCKSHK
EPI_ISL_413575      ------------------------------------------------------------
EPI_ISL_413588      ------------------------------------------------------------
EPI_ISL_413589      ------------------------------------------------------------
EPI_ISL_424221      ------------------------------------------------------------
EPI_ISL_424233      ------------------------------------------------------------
EPI_ISL_424236      ------------------------------------------------------------
EPI_ISL_424238      ------------------------------------------------------------
EPI_ISL_424248      ------------------------------------------------------------
EPI_ISL_424254      ------------------------------------------------------------
EPI_ISL_425548      ------------------------------------------------------------
EPI_ISL_425581      ------------------------------------------------------------
EPI_ISL_426881      ------------------------------------------------------------
EPI_ISL_426883      ------------------------------------------------------------
EPI_ISL_426888      ------------------------------------------------------------
EPI_ISL_426900      ------------------------------------------------------------
EPI_ISL_426901      ------------------------------------------------------------
EPI_ISL_426903      ------------------------------------------------------------
EPI_ISL_426904      ------------------------------------------------------------
EPI_ISL_426907      ------------------------------------------------------------
EPI_ISL_426910      ------------------------------------------------------------
```

```
ORF1ab         5401 PPISFPLCANGQVFGLYKNTCVGSDNVTDFNAIATCDWTNAGDYILANTCTERLKLFAAE
EPI_ISL_413575      ------------------------------------------------------------
EPI_ISL_413588      ------------------------------------------------------------
EPI_ISL_413589      ------------------------------------------------------------
EPI_ISL_424221      ------------------------------------------------------------
EPI_ISL_424233      ------------------------------------------------------------
EPI_ISL_424236      ------------------------------------------------------------
EPI_ISL_424238      ------------------------------------------------------------
EPI_ISL_424248      ------------------------------------------------------------
EPI_ISL_424254      ------------------------------------------------------------
EPI_ISL_425548      ------------------------------------------------------------
EPI_ISL_425581      ------------------------------------------------------------
EPI_ISL_426881      ------------------------------------------------------------
EPI_ISL_426883      ------------------------------------------------------------
EPI_ISL_426888      ------------------------------------------------------------
EPI_ISL_426900      ------------------------------------------------------------
EPI_ISL_426901      ------------------------------------------------------------
EPI_ISL_426903      ------------------------------------------------------------
EPI_ISL_426904      ------------------------------------------------------------
EPI_ISL_426907      ------------------------------------------------------------
EPI_ISL_426910      ------------------------------------------------------------
```

```
ORF1ab         5401 PPISFPLCANGQVFGLYKNTCVGSDNVTDFNAIATCDWTNAGDYILANTCTERLKLFAAE
EPI_ISL_413575      ------------------------------------------------------------
EPI_ISL_413588      ------------------------------------------------------------
EPI_ISL_413589      ------------------------------------------------------------
EPI_ISL_424221      ------------------------------------------------------------
EPI_ISL_424233      ------------------------------------------------------------
EPI_ISL_424236      ------------------------------------------------------------
EPI_ISL_424238      ------------------------------------------------------------
EPI_ISL_424248      ------------------------------------------------------------
EPI_ISL_424254      ------------------------------------------------------------
EPI_ISL_425548      ------------------------------------------------------------
EPI_ISL_425581      ------------------------------------------------------------
EPI_ISL_426881      ------------------------------------------------------------
EPI_ISL_426883      ------------------------------------------------------------
EPI_ISL_426888      ------------------------------------------------------------
EPI_ISL_426900      ------------------------------------------------------------
EPI_ISL_426901      ------------------------------------------------------------
EPI_ISL_426903      ------------------------------------------------------------
EPI_ISL_426904      ------------------------------------------------------------
EPI_ISL_426907      ------------------------------------------------------------
EPI_ISL_426910      ------------------------------------------------------------
```

```
ORF1ab         5461 TLKATEETFKLSYGIATVREVLSDRELHLSWEVGKPRPPLNRNYVFTGYRVTKNSKVQIG
EPI_ISL_413575      ------------------------------------------------------------
EPI_ISL_413588      ------------------------------------------------------------
EPI_ISL_413589      ------------------------------------------------------------
EPI_ISL_424221      ------------------------------------------------------------
EPI_ISL_424233      ------------------------------------------------------------
EPI_ISL_424236      ------------------------------------------------------------
EPI_ISL_424238      ------------------------------------------------------------
EPI_ISL_424248      ------------------------------------------------------------
EPI_ISL_424254      ------------------------------------------------------------
EPI_ISL_425548      ------------------------------------------------------------
EPI_ISL_425581      ------------------------------------------------------------
EPI_ISL_426881      ------------------------------------------------------------
EPI_ISL_426883      ------------------------------------------------------------
EPI_ISL_426888      ------------------------------------------------------------
EPI_ISL_426900      ------------------------------------------------------------
EPI_ISL_426901      ------------------------------------------------------------
EPI_ISL_426903      ------------------------------------------------------------
EPI_ISL_426904      ------------------------------------------------------------
EPI_ISL_426907      ------------------------------------------------------------
EPI_ISL_426910      ------------------------------------------------------------
```


```
ORF1ab         5461 TLKATEETFKLSYGIATVREVLSDRELHLSWEVGKPRPPLNRNYVFTGYRVTKNSKVQIG
EPI_ISL_413575      ------------------------------------------------------------
EPI_ISL_413588      ------------------------------------------------------------
EPI_ISL_413589      ------------------------------------------------------------
EPI_ISL_424221      ------------------------------------------------------------
EPI_ISL_424233      ------------------------------------------------------------
EPI_ISL_424236      ------------------------------------------------------------
EPI_ISL_424238      ------------------------------------------------------------
EPI_ISL_424248      ------------------------------------------------------------
EPI_ISL_424254      ------------------------------------------------------------
EPI_ISL_425548      ------------------------------------------------------------
EPI_ISL_425581      ------------------------------------------------------------
EPI_ISL_426881      ------------------------------------------------------------
EPI_ISL_426883      ------------------------------------------------------------
EPI_ISL_426888      ------------------------------------------------------------
EPI_ISL_426900      ------------------------------------------------------------
EPI_ISL_426901      ------------------------------------------------------------
EPI_ISL_426903      ------------------------------------------------------------
EPI_ISL_426904      ------------------------------------------------------------
EPI_ISL_426907      ------------------------------------------------------------
EPI_ISL_426910      ------------------------------------------------------------
```

```
ORF1ab         5521 EYTFEKGDYGDAVVYRGTTTYKLNVGDYFVLTSHTVMPLSAPTLVPQEHYVRITGLYPTL
EPI_ISL_413575      ------------------------------------------------------------
EPI_ISL_413588      ------------------------------------------------------------
EPI_ISL_413589      ------------------------------------------------------------
EPI_ISL_424221      ------------------------------------------------------------
EPI_ISL_424233      ------------------------------------------------------------
EPI_ISL_424236      ------------------------------------------------------------
EPI_ISL_424238      ------------------------------------------------------------
EPI_ISL_424248      ------------------------------------------------------------
EPI_ISL_424254      ------------------------------------------------------------
EPI_ISL_425548      ------------------------------------------------------------
EPI_ISL_425581      ------------------------------------------------------------
EPI_ISL_426881      ------------------------------------------------------------
EPI_ISL_426883      ------------------------------------------------------------
EPI_ISL_426888      ------------------------------------------------------------
EPI_ISL_426900      ------------------------------------------------------------
EPI_ISL_426901      ------------------------------------------------------------
EPI_ISL_426903      ------------------------------------------------------------
EPI_ISL_426904      ------------------------------------------------------------
EPI_ISL_426907      ------------------------------------------------------------
EPI_ISL_426910      ------------------------------------------------------------
ORF1ab         5521 EYTFEKGDYGDAVVYRGTTTYKLNVGDYFVLTSHTVMPLSAPTLVPQEHYVRITGLYPTL
EPI_ISL_413575      ------------------------------------------------------------
EPI_ISL_413588      ------------------------------------------------------------
EPI_ISL_413589      ------------------------------------------------------------
EPI_ISL_424221      ------------------------------------------------------------
EPI_ISL_424233      ------------------------------------------------------------
EPI_ISL_424236      ------------------------------------------------------------
EPI_ISL_424238      ------------------------------------------------------------
EPI_ISL_424248      ------------------------------------------------------------
EPI_ISL_424254      ------------------------------------------------------------
EPI_ISL_425548      ------------------------------------------------------------
EPI_ISL_425581      ------------------------------------------------------------
EPI_ISL_426881      ------------------------------------------------------------
EPI_ISL_426883      ------------------------------------------------------------
EPI_ISL_426888      ------------------------------------------------------------
EPI_ISL_426900      ------------------------------------------------------------
EPI_ISL_426901      ------------------------------------------------------------
EPI_ISL_426903      ------------------------------------------------------------
EPI_ISL_426904      ------------------------------------------------------------
EPI_ISL_426907      ------------------------------------------------------------
EPI_ISL_426910      ------------------------------------------------------------
```

```
ORF1ab          5581 NISDEFSSNVANYQKVGMQKYSTLQGPPGTGKSHFAIGLALYYPSARIVYTACSHAAVDA
EPI_ISL_413575       ------------------------------------------------------------
EPI_ISL_413588       ------------------------------------------------------------
EPI_ISL_413589       ------------------------------------------------------------
EPI_ISL_424221       ------------------------------------------------------------
EPI_ISL_424233       ------------------------------------------------------------
EPI_ISL_424236       ------------------------------------------------------------
EPI_ISL_424238       ------------------------------------------------------------
EPI_ISL_424248       ------------------------------------------------------------
EPI_ISL_424254       ------------------------------------------------------------
EPI_ISL_425548       ------------------------------------------------------------
EPI_ISL_425581       ------------------------------------------------------------
EPI_ISL_426881       ------------------------------------------------------------
EPI_ISL_426883       ------------------------------------------------------------
EPI_ISL_426888       ------------------------------------------------------------
EPI_ISL_426900       ------------------------------------------------------------
EPI_ISL_426901       ------------------------------------------------------------
EPI_ISL_426903       ------------------------------------------------------------
EPI_ISL_426904       ------------------------------------------------------------
EPI_ISL_426907       ------------------------------------------------------------
EPI_ISL_426910       ------------------------------------------------------------
```

```
ORF1ab          5581 NISDEFSSNVANYQKVGMQKYSTLQGPPGTGKSHFAIGLALYYPSARIVYTACSHAAVDA
EPI_ISL_413575       ------------------------------------------------------------
EPI_ISL_413588       ------------------------------------------------------------
EPI_ISL_413589       ------------------------------------------------------------
EPI_ISL_424221       ------------------------------------------------------------
EPI_ISL_424233       ------------------------------------------------------------
EPI_ISL_424236       ------------------------------------------------------------
EPI_ISL_424238       ------------------------------------------------------------
EPI_ISL_424248       ------------------------------------------------------------
EPI_ISL_424254       ------------------------------------------------------------
EPI_ISL_425548       ------------------------------------------------------------
EPI_ISL_425581       ------------------------------------------------------------
EPI_ISL_426881       ------------------------------------------------------------
EPI_ISL_426883       ------------------------------------------------------------
EPI_ISL_426888       ------------------------------------------------------------
EPI_ISL_426900       ------------------------------------------------------------
EPI_ISL_426901       ------------------------------------------------------------
EPI_ISL_426903       ------------------------------------------------------------
EPI_ISL_426904       ------------------------------------------------------------
EPI_ISL_426907       ------------------------------------------------------------
EPI_ISL_426910       ------------------------------------------------------------
```

```
ORF1ab         5641 LCEKALKYLPIDKCSRIIPARARVECFDKFKVNSTLEQYVFCTVNALPETTADIVVFDEI
EPI_ISL_413575      ------------------------------------------------------------
EPI_ISL_413588      ------------------------------------------------------------
EPI_ISL_413589      ------------------------------------------------------------
EPI_ISL_424221      ------------------------------------------------------------
EPI_ISL_424233      ------------------------------------------------------------
EPI_ISL_424236      ------------------------------------------------------------
EPI_ISL_424238      ------------------------------------------------------------
EPI_ISL_424248      ------------------------------------------------------------
EPI_ISL_424254      ------------------------------------------------------------
EPI_ISL_425548      ------------------------------------------------------------
EPI_ISL_425581      ------------------------------------------------------------
EPI_ISL_426881      ------------------------------------------------------------
EPI_ISL_426883      ------------------------------------------------------------
EPI_ISL_426888      ------------------------------------------------------------
EPI_ISL_426900      ------------------------------------------------------------
EPI_ISL_426901      ------------------------------------------------------------
EPI_ISL_426903      ------------------------------------------------------------
EPI_ISL_426904      ------------------------------------------------------------
EPI_ISL_426907      ------------------------------------------------------------
EPI_ISL_426910      ------------------------------------------------------------
```


```
ORF1ab         5641 LCEKALKYLPIDKCSRIIPARARVECFDKFKVNSTLEQYVFCTVNALPETTADIVVFDEI
EPI_ISL_413575      ------------------------------------------------------------
EPI_ISL_413588      ------------------------------------------------------------
EPI_ISL_413589      ------------------------------------------------------------
EPI_ISL_424221      ------------------------------------------------------------
EPI_ISL_424233      ------------------------------------------------------------
EPI_ISL_424236      ------------------------------------------------------------
EPI_ISL_424238      ------------------------------------------------------------
EPI_ISL_424248      ------------------------------------------------------------
EPI_ISL_424254      ------------------------------------------------------------
EPI_ISL_425548      ------------------------------------------------------------
EPI_ISL_425581      ------------------------------------------------------------
EPI_ISL_426881      ------------------------------------------------------------
EPI_ISL_426883      ------------------------------------------------------------
EPI_ISL_426888      ------------------------------------------------------------
EPI_ISL_426900      ------------------------------------------------------------
EPI_ISL_426901      ------------------------------------------------------------
EPI_ISL_426903      ------------------------------------------------------------
EPI_ISL_426904      ------------------------------------------------------------
EPI_ISL_426907      ------------------------------------------------------------
EPI_ISL_426910      ------------------------------------------------------------
```

```
ORF1ab         5701 SMATNYDLSVVNARLRAKHYVYIGDPAQLPAPRTLLTKGTLEPEYFNSVCRLMKTIGPDM
EPI_ISL_413575      ------------------------------------------------------------
EPI_ISL_413588      ------------------------------------------------------------
EPI_ISL_413589      ------------------------------------------------------------
EPI_ISL_424221      ------------------------------------------------------------
EPI_ISL_424233      ------------------------------------------------------------
EPI_ISL_424236      ------------------------------------------------------------
EPI_ISL_424238      ------------------------------------------------------------
EPI_ISL_424248      ------------------------------------------------------------
EPI_ISL_424254      ------------------------------------------------------------
EPI_ISL_425548      ------------------------------------------------------------
EPI_ISL_425581      ------------------------------------------------------------
EPI_ISL_426881      ------------------------------------------------------------
EPI_ISL_426883      ------------------------------------------------------------
EPI_ISL_426888      ------------------------------------------------------------
EPI_ISL_426900      ------------------------------------------------------------
EPI_ISL_426901      ------------------------------------------------------------
EPI_ISL_426903      ------------------------------------------------------------
EPI_ISL_426904      ------------------------------------------------------------
EPI_ISL_426907      ------------------------------------------------------------
EPI_ISL_426910      ------------------------------------------------------------
```

```
ORF1ab         5701 SMATNYDLSVVNARLRAKHYVYIGDPAQLPAPRTLLTKGTLEPEYFNSVCRLMKTIGPDM
EPI_ISL_413575      ------------------------------------------------------------
EPI_ISL_413588      ------------------------------------------------------------
EPI_ISL_413589      ------------------------------------------------------------
EPI_ISL_424221      ------------------------------------------------------------
EPI_ISL_424233      ------------------------------------------------------------
EPI_ISL_424236      ------------------------------------------------------------
EPI_ISL_424238      ------------------------------------------------------------
EPI_ISL_424248      ------------------------------------------------------------
EPI_ISL_424254      ------------------------------------------------------------
EPI_ISL_425548      ------------------------------------------------------------
EPI_ISL_425581      ------------------------------------------------------------
EPI_ISL_426881      ------------------------------------------------------------
EPI_ISL_426883      ------------------------------------------------------------
EPI_ISL_426888      ------------------------------------------------------------
EPI_ISL_426900      ------------------------------------------------------------
EPI_ISL_426901      ------------------------------------------------------------
EPI_ISL_426903      ------------------------------------------------------------
EPI_ISL_426904      ------------------------------------------------------------
EPI_ISL_426907      ------------------------------------------------------------
EPI_ISL_426910      ------------------------------------------------------------
```

```
ORF1ab          5761 FLGTCRRCPAEIVDTVSALVYDNKLKAHKDKSAQCFKMFYKGVITHDVSSAINRPQIGVV
EPI_ISL_413575       ------------------------------------------------------------
EPI_ISL_413588       ------------------------------------------------------------
EPI_ISL_413589       ------------------------------------------------------------
EPI_ISL_424221       ------------------------------------------------------------
EPI_ISL_424233       ------------------------------------------------------------
EPI_ISL_424236       ------------------------------------------------------------
EPI_ISL_424238       ------------------------------------------------------------
EPI_ISL_424248       ------------------------------------------------------------
EPI_ISL_424254       ------------------------------------------------------------
EPI_ISL_425548       ------------------------------------------------------------
EPI_ISL_425581       ------------------------------------------------------------
EPI_ISL_426881       ------------------------------------------------------------
EPI_ISL_426883       ------------------------------------------------------------
EPI_ISL_426888       ------------------------------------------------------------
EPI_ISL_426900       ------------------------------------------------------------
EPI_ISL_426901       ------------------------------------------------------------
EPI_ISL_426903       ------------------------------------------------------------
EPI_ISL_426904       ------------------------------------------------------------
EPI_ISL_426907       ------------------------------------------------------------
EPI_ISL_426910       ------------------------------------------------------------
ORF1ab          5761 FLGTCRRCPAEIVDTVSALVYDNKLKAHKDKSAQCFKMFYKGVITHDVSSAINRPQIGVV
EPI_ISL_413575       ------------------------------------------------------------
EPI_ISL_413588       ------------------------------------------------------------
EPI_ISL_413589       ------------------------------------------------------------
EPI_ISL_424221       ------------------------------------------------------------
EPI_ISL_424233       ------------------------------------------------------------
EPI_ISL_424236       ------------------------------------------------------------
EPI_ISL_424238       ------------------------------------------------------------
EPI_ISL_424248       ------------------------------------------------------------
EPI_ISL_424254       ------------------------------------------------------------
EPI_ISL_425548       ------------------------------------------------------------
EPI_ISL_425581       ------------------------------------------------------------
EPI_ISL_426881       ------------------------------------------------------------
EPI_ISL_426883       ------------------------------------------------------------
EPI_ISL_426888       ------------------------------------------------------------
EPI_ISL_426900       ------------------------------------------------------------
EPI_ISL_426901       ------------------------------------------------------------
EPI_ISL_426903       ------------------------------------------------------------
EPI_ISL_426904       ------------------------------------------------------------
EPI_ISL_426907       ------------------------------------------------------------
EPI_ISL_426910       ------------------------------------------------------------
```

```
ORF1ab          5821 REFLTRNPAWRKAVFISPYNSQNAVASKILGLPTQTVDSSQGSEYDYVIFTQTTETAHSC
EPI_ISL_413575       ------------------------------------------------------------
EPI_ISL_413588       ------------------------------------------------------------
EPI_ISL_413589       ------------------------------------------------------------
EPI_ISL_424221       ------------------------------------------------------------
EPI_ISL_424233       ------------------------------------------------------------
EPI_ISL_424236       ------------------------------------------------------------
EPI_ISL_424238       ------------------------------------------------------------
EPI_ISL_424248       ------------------------------------------------------------
EPI_ISL_424254       ------------------------------------------------------------
EPI_ISL_425548       ------------------------------------------------------------
EPI_ISL_425581       ------------------------------------------------------------
EPI_ISL_426881       ------------------------------------------------------------
EPI_ISL_426883       ------------------------------------------------------------
EPI_ISL_426888       ------------------------------------------------------------
EPI_ISL_426900       ------------------------------------------------------------
EPI_ISL_426901       ------------------------------------------------------------
EPI_ISL_426903       ------------------------------------------------------------
EPI_ISL_426904       ------------------------------------------------------------
EPI_ISL_426907       ------------------------------------------------------------
EPI_ISL_426910       ------------------------------------------------------------
ORF1ab          5821 REFLTRNPAWRKAVFISPYNSQNAVASKILGLPTQTVDSSQGSEYDYVIFTQTTETAHSC
EPI_ISL_413575       ------------------------------------------------------------
EPI_ISL_413588       ------------------------------------------------------------
EPI_ISL_413589       ------------------------------------------------------------
EPI_ISL_424221       ------------------------------------------------------------
EPI_ISL_424233       ------------------------------------------------------------
EPI_ISL_424236       ------------------------------------------------------------
EPI_ISL_424238       ------------------------------------------------------------
EPI_ISL_424248       ------------------------------------------------------------
EPI_ISL_424254       ------------------------------------------------------------
EPI_ISL_425548       ------------------------------------------------------------
EPI_ISL_425581       ------------------------------------------------------------
EPI_ISL_426881       ------------------------------------------------------------
EPI_ISL_426883       ------------------------------------------------------------
EPI_ISL_426888       ------------------------------------------------------------
EPI_ISL_426900       ------------------------------------------------------------
EPI_ISL_426901       ------------------------------------------------------------
EPI_ISL_426903       ------------------------------------------------------------
EPI_ISL_426904       ------------------------------------------------------------
EPI_ISL_426907       ------------------------------------------------------------
EPI_ISL_426910       ------------------------------------------------------------
```

```
ORF1ab         5881 NVNRFNVAITRAKVGILCIMSDRDLYDKLQFTSLEIPRRNVATLQAENVTGLFKDCSKVI
EPI_ISL_413575      ------------------------------------------------------------
EPI_ISL_413588      ------------------------------------------------------------
EPI_ISL_413589      ------------------------------------------------------------
EPI_ISL_424221      ------------------------------------------------------------
EPI_ISL_424233      ------------------------------------------------------------
EPI_ISL_424236      ------------------------------------------------------------
EPI_ISL_424238      ------------------------------------------------------------
EPI_ISL_424248      ------------------------------------------------------------
EPI_ISL_424254      ------------------------------------------------------------
EPI_ISL_425548      ------------------------------------------------------------
EPI_ISL_425581      ------------------------------------------------------------
EPI_ISL_426881      ------------------------------------------------------------
EPI_ISL_426883      ------------------------------------------------------------
EPI_ISL_426888      ------------------------------------------------------------
EPI_ISL_426900      ------------------------------------------------------------
EPI_ISL_426901      ------------------------------------------------------------
EPI_ISL_426903      ------------------------------------------------------------
EPI_ISL_426904      ------------------------------------------------------------
EPI_ISL_426907      ------------------------------------------------------------
EPI_ISL_426910      ------------------------------------------------------------
```

```
ORF1ab         5881 NVNRFNVAITRAKVGILCIMSDRDLYDKLQFTSLEIPRRNVATLQAENVTGLFKDCSKVI
EPI_ISL_413575      ------------------------------------------------------------
EPI_ISL_413588      ------------------------------------------------------------
EPI_ISL_413589      ------------------------------------------------------------
EPI_ISL_424221      ------------------------------------------------------------
EPI_ISL_424233      ------------------------------------------------------------
EPI_ISL_424236      ------------------------------------------------------------
EPI_ISL_424238      ------------------------------------------------------------
EPI_ISL_424248      ------------------------------------------------------------
EPI_ISL_424254      ------------------------------------------------------------
EPI_ISL_425548      ------------------------------------------------------------
EPI_ISL_425581      ------------------------------------------------------------
EPI_ISL_426881      ------------------------------------------------------------
EPI_ISL_426883      ------------------------------------------------------------
EPI_ISL_426888      ------------------------------------------------------------
EPI_ISL_426900      ------------------------------------------------------------
EPI_ISL_426901      ------------------------------------------------------------
EPI_ISL_426903      ------------------------------------------------------------
EPI_ISL_426904      ------------------------------------------------------------
EPI_ISL_426907      ------------------------------------------------------------
EPI_ISL_426910      ------------------------------------------------------------
```

```
ORF1ab          5941 TGLHPTQAPTHLSVDTKFKTEGLCVDIPGIPKDMTYRRLISMMGFKMNYQVNGYPNMFIT
EPI_ISL_413575       ------------------------------------------------------------
EPI_ISL_413588       ------------------------------------------------------------
EPI_ISL_413589       ------------------------------------------------------------
EPI_ISL_424221       ------------------------------------------------------------
EPI_ISL_424233       ------------------------------------------------------------
EPI_ISL_424236       ------------------------------------------------------------
EPI_ISL_424238       ------------------------------------------------------------
EPI_ISL_424248       ------------------------------------------------------------
EPI_ISL_424254       ------------------------------------------------------------
EPI_ISL_425548       ------------------------------------------------------------
EPI_ISL_425581       ------------------------------------------------------------
EPI_ISL_426881       ------------------------------------------------------------
EPI_ISL_426883       ------------------------------------------------------------
EPI_ISL_426888       ------------------------------------------------------------
EPI_ISL_426900       ------------------------------------------------------------
EPI_ISL_426901       ------------------------------------------------------------
EPI_ISL_426903       ------------------------------------------------------------
EPI_ISL_426904       ------------------------------------------------------------
EPI_ISL_426907       ------------------------------------------------------------
EPI_ISL_426910       ------------------------------------------------------------
```

```
ORF1ab          5941 TGLHPTQAPTHLSVDTKFKTEGLCVDIPGIPKDMTYRRLISMMGFKMNYQVNGYPNMFIT
EPI_ISL_413575       ------------------------------------------------------------
EPI_ISL_413588       ------------------------------------------------------------
EPI_ISL_413589       ------------------------------------------------------------
EPI_ISL_424221       ------------------------------------------------------------
EPI_ISL_424233       ------------------------------------------------------------
EPI_ISL_424236       ------------------------------------------------------------
EPI_ISL_424238       ------------------------------------------------------------
EPI_ISL_424248       ------------------------------------------------------------
EPI_ISL_424254       ------------------------------------------------------------
EPI_ISL_425548       ------------------------------------------------------------
EPI_ISL_425581       ------------------------------------------------------------
EPI_ISL_426881       ------------------------------------------------------------
EPI_ISL_426883       ------------------------------------------------------------
EPI_ISL_426888       ------------------------------------------------------------
EPI_ISL_426900       ------------------------------------------------------------
EPI_ISL_426901       ------------------------------------------------------------
EPI_ISL_426903       ------------------------------------------------------------
EPI_ISL_426904       ------------------------------------------------------------
EPI_ISL_426907       ------------------------------------------------------------
EPI_ISL_426910       ------------------------------------------------------------
```

```
ORF1ab          6001 REEAIRHVRAWIGFDVEGCHATREAVGTNLPLQLGFSTGVNLVAVPTGYVDTPNNTDFSR
EPI_ISL_413575       ------------------------------------------------------------
EPI_ISL_413588       ------------------------------------------------------------
EPI_ISL_413589       ------------------------------------------------------------
EPI_ISL_424221       ------------------------------------------------------------
EPI_ISL_424233       ------------------------------------------------------------
EPI_ISL_424236       ------------------------------------------------------------
EPI_ISL_424238       ------------------------------------------------------------
EPI_ISL_424248       ------------------------------------------------------------
EPI_ISL_424254       ------------------------------------------------------------
EPI_ISL_425548       ------------------------------------------------------------
EPI_ISL_425581       ------------------------------------------------------------
EPI_ISL_426881       ------------------------------------------------------------
EPI_ISL_426883       ------------------------------------------------------------
EPI_ISL_426888       ------------------------------------------------------------
EPI_ISL_426900       ------------------------------------------------------------
EPI_ISL_426901       ------------------------------------------------------------
EPI_ISL_426903       ------------------------------------------------------------
EPI_ISL_426904       ------------------------------------------------------------
EPI_ISL_426907       ------------------------------------------------------------
EPI_ISL_426910       ------------------------------------------------------------
```


```
ORF1ab          6001 REEAIRHVRAWIGFDVEGCHATREAVGTNLPLQLGFSTGVNLVAVPTGYVDTPNNTDFSR
EPI_ISL_413575       ------------------------------------------------------------
EPI_ISL_413588       ------------------------------------------------------------
EPI_ISL_413589       ------------------------------------------------------------
EPI_ISL_424221       ------------------------------------------------------------
EPI_ISL_424233       ------------------------------------------------------------
EPI_ISL_424236       ------------------------------------------------------------
EPI_ISL_424238       ------------------------------------------------------------
EPI_ISL_424248       ------------------------------------------------------------
EPI_ISL_424254       ------------------------------------------------------------
EPI_ISL_425548       ------------------------------------------------------------
EPI_ISL_425581       ------------------------------------------------------------
EPI_ISL_426881       ------------------------------------------------------------
EPI_ISL_426883       ------------------------------------------------------------
EPI_ISL_426888       ------------------------------------------------------------
EPI_ISL_426900       ------------------------------------------------------------
EPI_ISL_426901       ------------------------------------------------------------
EPI_ISL_426903       ------------------------------------------------------------
EPI_ISL_426904       ------------------------------------------------------------
EPI_ISL_426907       ------------------------------------------------------------
EPI_ISL_426910       ------------------------------------------------------------
```

```
ORF1ab          6061 VSAKPPPGDQFKHLIPLMYKGLPWNVVRIKIVQMLSDTLKNLSDRVVFVLWAHGFELTSM
EPI_ISL_413575       ------------------------------------------------------------
EPI_ISL_413588       ------------------------------------------------------------
EPI_ISL_413589       ------------------------------------------------------------
EPI_ISL_424221       ------------------------------------------------------------
EPI_ISL_424233       ------------------------------------------------------------
EPI_ISL_424236       ------------------------------------------------------------
EPI_ISL_424238       ------------------------------------------------------------
EPI_ISL_424248       ------------------------------------------------------------
EPI_ISL_424254       ------------------------------------------------------------
EPI_ISL_425548       ------------------------------------------------------------
EPI_ISL_425581       ------------------------------------------------------------
EPI_ISL_426881       ------------------------------------------------------------
EPI_ISL_426883       ------------------------------------------------------------
EPI_ISL_426888       ------------------------------------------------------------
EPI_ISL_426900       ------------------------------------------------------------
EPI_ISL_426901       ------------------------------------------------------------
EPI_ISL_426903       ------------------------------------------------------------
EPI_ISL_426904       ------------------------------------------------------------
EPI_ISL_426907       ------------------------------------------------------------
EPI_ISL_426910       ------------------------------------------------------------
```


```
ORF1ab          6061 VSAKPPPGDQFKHLIPLMYKGLPWNVVRIKIVQMLSDTLKNLSDRVVFVLWAHGFELTSM
EPI_ISL_413575       ------------------------------------------------------------
EPI_ISL_413588       ------------------------------------------------------------
EPI_ISL_413589       ------------------------------------------------------------
EPI_ISL_424221       ------------------------------------------------------------
EPI_ISL_424233       ------------------------------------------------------------
EPI_ISL_424236       ------------------------------------------------------------
EPI_ISL_424238       ------------------------------------------------------------
EPI_ISL_424248       ------------------------------------------------------------
EPI_ISL_424254       ------------------------------------------------------------
EPI_ISL_425548       ------------------------------------------------------------
EPI_ISL_425581       ------------------------------------------------------------
EPI_ISL_426881       ------------------------------------------------------------
EPI_ISL_426883       ------------------------------------------------------------
EPI_ISL_426888       ------------------------------------------------------------
EPI_ISL_426900       ------------------------------------------------------------
EPI_ISL_426901       ------------------------------------------------------------
EPI_ISL_426903       ------------------------------------------------------------
EPI_ISL_426904       ------------------------------------------------------------
EPI_ISL_426907       ------------------------------------------------------------
EPI_ISL_426910       ------------------------------------------------------------
```

```
ORF1ab         6121 KYFVKIGPERTCCLCDRRATCFSTASDTYACWHHSIGFDYVYNPFMIDVQQWGFTGNLQS
EPI_ISL_413575      ------------------------------------------------------------
EPI_ISL_413588      ------------------------------------------------------------
EPI_ISL_413589      ------------------------------------------------------------
EPI_ISL_424221      ------------------------------------------------------------
EPI_ISL_424233      ------------------------------------------------------------
EPI_ISL_424236      ------------------------------------------------------------
EPI_ISL_424238      ------------------------------------------------------------
EPI_ISL_424248      ------------------------------------------------------------
EPI_ISL_424254      ------------------------------------------------------------
EPI_ISL_425548      ------------------------------------------------------------
EPI_ISL_425581      ------------------------------------------------------------
EPI_ISL_426881      ------------------------------------------------------------
EPI_ISL_426883      ------------------------------------------------------------
EPI_ISL_426888      ------------------------------------------------------------
EPI_ISL_426900      ------------------------------------------------------------
EPI_ISL_426901      ------------------------------------------------------------
EPI_ISL_426903      ------------------------------------------------------------
EPI_ISL_426904      ------------------------------------------------------------
EPI_ISL_426907      ------------------------------------------------------------
EPI_ISL_426910      ------------------------------------------------------------
ORF1ab         6121 KYFVKIGPERTCCLCDRRATCFSTASDTYACWHHSIGFDYVYNPFMIDVQQWGFTGNLQS
EPI_ISL_413575      ------------------------------------------------------------
EPI_ISL_413588      ------------------------------------------------------------
EPI_ISL_413589      ------------------------------------------------------------
EPI_ISL_424221      ------------------------------------------------------------
EPI_ISL_424233      ------------------------------------------------------------
EPI_ISL_424236      ------------------------------------------------------------
EPI_ISL_424238      ------------------------------------------------------------
EPI_ISL_424248      ------------------------------------------------------------
EPI_ISL_424254      ------------------------------------------------------------
EPI_ISL_425548      ------------------------------------------------------------
EPI_ISL_425581      ------------------------------------------------------------
EPI_ISL_426881      ------------------------------------------------------------
EPI_ISL_426883      ------------------------------------------------------------
EPI_ISL_426888      ------------------------------------------------------------
EPI_ISL_426900      ------------------------------------------------------------
EPI_ISL_426901      ------------------------------------------------------------
EPI_ISL_426903      ------------------------------------------------------------
EPI_ISL_426904      ------------------------------------------------------------
EPI_ISL_426907      ------------------------------------------------------------
EPI_ISL_426910      ------------------------------------------------------------
```

```
ORF1ab         6181 NHDLYCQVHGNAHVASCDAIMTRCLAVHECFVKRVDWTIEYPIIGDELKINAACRKVQHM
EPI_ISL_413575      ------------------------------------------------------------
EPI_ISL_413588      ------------------------------------------------------------
EPI_ISL_413589      ------------------------------------------------------------
EPI_ISL_424221      ------------------------------------------------------------
EPI_ISL_424233      ------------------------------------------------------------
EPI_ISL_424236      ------------------------------------------------------------
EPI_ISL_424238      ------------------------------------------------------------
EPI_ISL_424248      ------------------------------------------------------------
EPI_ISL_424254      ------------------------------------------------------------
EPI_ISL_425548      ------------------------------------------------------------
EPI_ISL_425581      ------------------------------------------------------------
EPI_ISL_426881      ------------------------------------------------------------
EPI_ISL_426883      ------------------------------------------------------------
EPI_ISL_426888      ------------------------------------------------------------
EPI_ISL_426900      ------------------------------------------------------------
EPI_ISL_426901      ------------------------------------------------------------
EPI_ISL_426903      ------------------------------------------------------------
EPI_ISL_426904      ------------------------------------------------------------
EPI_ISL_426907      ------------------------------------------------------------
EPI_ISL_426910      ------------------------------------------------------------
```


```
ORF1ab         6181 NHDLYCQVHGNAHVASCDAIMTRCLAVHECFVKRVDWTIEYPIIGDELKINAACRKVQHM
EPI_ISL_413575      ------------------------------------------------------------
EPI_ISL_413588      ------------------------------------------------------------
EPI_ISL_413589      ------------------------------------------------------------
EPI_ISL_424221      ------------------------------------------------------------
EPI_ISL_424233      ------------------------------------------------------------
EPI_ISL_424236      ------------------------------------------------------------
EPI_ISL_424238      ------------------------------------------------------------
EPI_ISL_424248      ------------------------------------------------------------
EPI_ISL_424254      ------------------------------------------------------------
EPI_ISL_425548      ------------------------------------------------------------
EPI_ISL_425581      ------------------------------------------------------------
EPI_ISL_426881      ------------------------------------------------------------
EPI_ISL_426883      ------------------------------------------------------------
EPI_ISL_426888      ------------------------------------------------------------
EPI_ISL_426900      ------------------------------------------------------------
EPI_ISL_426901      ------------------------------------------------------------
EPI_ISL_426903      ------------------------------------------------------------
EPI_ISL_426904      ------------------------------------------------------------
EPI_ISL_426907      ------------------------------------------------------------
EPI_ISL_426910      ------------------------------------------------------------
```

```
ORF1ab          6241 VVKAALLADKFPVLHDIGNPKAIKCVPQADVEWKFYDAQPCSDKAYKIEELFYSYATHSD
EPI_ISL_413575       ------------------------------------------------------------
EPI_ISL_413588       ------------------------------------------------------------
EPI_ISL_413589       ------------------------------------------------------------
EPI_ISL_424221       ------------------------------------------------------------
EPI_ISL_424233       ------------------------------------------------------------
EPI_ISL_424236       ------------------------------------------------------------
EPI_ISL_424238       ------------------------------------------------------------
EPI_ISL_424248       ------------------------------------------------------------
EPI_ISL_424254       ------------------------------------------------------------
EPI_ISL_425548       ------------------------------------------------------------
EPI_ISL_425581       ------------------------------------------------------------
EPI_ISL_426881       ------------------------------------------------------------
EPI_ISL_426883       ------------------------------------------------------------
EPI_ISL_426888       ------------------------------------------------------------
EPI_ISL_426900       ------------------------------------------------------------
EPI_ISL_426901       ------------------------------------------------------------
EPI_ISL_426903       ------------------------------------------------------------
EPI_ISL_426904       ------------------------------------------------------------
EPI_ISL_426907       ------------------------------------------------------------
EPI_ISL_426910       ------------------------------------------------------------
ORF1ab          6241 VVKAALLADKFPVLHDIGNPKAIKCVPQADVEWKFYDAQPCSDKAYKIEELFYSYATHSD
EPI_ISL_413575       ------------------------------------------------------------
EPI_ISL_413588       ------------------------------------------------------------
EPI_ISL_413589       ------------------------------------------------------------
EPI_ISL_424221       ------------------------------------------------------------
EPI_ISL_424233       ------------------------------------------------------------
EPI_ISL_424236       ------------------------------------------------------------
EPI_ISL_424238       ------------------------------------------------------------
EPI_ISL_424248       ------------------------------------------------------------
EPI_ISL_424254       ------------------------------------------------------------
EPI_ISL_425548       ------------------------------------------------------------
EPI_ISL_425581       ------------------------------------------------------------
EPI_ISL_426881       ------------------------------------------------------------
EPI_ISL_426883       ------------------------------------------------------------
EPI_ISL_426888       ------------------------------------------------------------
EPI_ISL_426900       ------------------------------------------------------------
EPI_ISL_426901       ------------------------------------------------------------
EPI_ISL_426903       ------------------------------------------------------------
EPI_ISL_426904       ------------------------------------------------------------
EPI_ISL_426907       ------------------------------------------------------------
EPI_ISL_426910       ------------------------------------------------------------
```

```
ORF1ab          6301 KFTDGVCLFWNCNVDRYPANSIVCRFDTRVLSNLNLPGCDGGSLYVNKHAFHTPAFDKSA
EPI_ISL_413575       ------------------------------------------------------------
EPI_ISL_413588       ------------------------------------------------------------
EPI_ISL_413589       ------------------------------------------------------------
EPI_ISL_424221       ------------------------------------------------------------
EPI_ISL_424233       ------------------------------------------------------------
EPI_ISL_424236       ------------------------------------------------------------
EPI_ISL_424238       ------------------------------------------------------------
EPI_ISL_424248       ------------------------------------------------------------
EPI_ISL_424254       ------------------------------------------------------------
EPI_ISL_425548       ------------------------------------------------------------
EPI_ISL_425581       ------------------------------------------------------------
EPI_ISL_426881       ------------------------------------------------------------
EPI_ISL_426883       ------------------------------------------------------------
EPI_ISL_426888       ------------------------------------------------------------
EPI_ISL_426900       ------------------------------------------------------------
EPI_ISL_426901       ------------------------------------------------------------
EPI_ISL_426903       ------------------------------------------------------------
EPI_ISL_426904       ------------------------------------------------------------
EPI_ISL_426907       ------------------------------------------------------------
EPI_ISL_426910       ------------------------------------------------------------
```


```
ORF1ab          6301 KFTDGVCLFWNCNVDRYPANSIVCRFDTRVLSNLNLPGCDGGSLYVNKHAFHTPAFDKSA
EPI_ISL_413575       ------------------------------------------------------------
EPI_ISL_413588       ------------------------------------------------------------
EPI_ISL_413589       ------------------------------------------------------------
EPI_ISL_424221       ------------------------------------------------------------
EPI_ISL_424233       ------------------------------------------------------------
EPI_ISL_424236       ------------------------------------------------------------
EPI_ISL_424238       ------------------------------------------------------------
EPI_ISL_424248       ------------------------------------------------------------
EPI_ISL_424254       ------------------------------------------------------------
EPI_ISL_425548       ------------------------------------------------------------
EPI_ISL_425581       ------------------------------------------------------------
EPI_ISL_426881       ------------------------------------------------------------
EPI_ISL_426883       ------------------------------------------------------------
EPI_ISL_426888       ------------------------------------------------------------
EPI_ISL_426900       ------------------------------------------------------------
EPI_ISL_426901       ------------------------------------------------------------
EPI_ISL_426903       ------------------------------------------------------------
EPI_ISL_426904       ------------------------------------------------------------
EPI_ISL_426907       ------------------------------------------------------------
EPI_ISL_426910       ------------------------------------------------------------
```

```
ORF1ab         6361 FVNLKQLPFFYYSDSPCESHGKQVVSDIDYVPLKSATCITRCNLGGAVCRHHANEYRLYL
EPI_ISL_413575      ------------------------------------------------------------
EPI_ISL_413588      ------------------------------------------------------------
EPI_ISL_413589      ------------------------------------------------------------
EPI_ISL_424221      ------------------------------------------------------------
EPI_ISL_424233      ------------------------------------------------------------
EPI_ISL_424236      ------------------------------------------------------------
EPI_ISL_424238      ------------------------------------------------------------
EPI_ISL_424248      ------------------------------------------------------------
EPI_ISL_424254      ------------------------------------------------------------
EPI_ISL_425548      ------------------------------------------------------------
EPI_ISL_425581      ------------------------------------------------------------
EPI_ISL_426881      ------------------------------------------------------------
EPI_ISL_426883      ------------------------------------------------------------
EPI_ISL_426888      ------------------------------------------------------------
EPI_ISL_426900      ------------------------------------------------------------
EPI_ISL_426901      ------------------------------------------------------------
EPI_ISL_426903      ------------------------------------------------------------
EPI_ISL_426904      ------------------------------------------------------------
EPI_ISL_426907      ------------------------------------------------------------
EPI_ISL_426910      ------------------------------------------------------------
ORF1ab         6361 FVNLKQLPFFYYSDSPCESHGKQVVSDIDYVPLKSATCITRCNLGGAVCRHHANEYRLYL
EPI_ISL_413575      ------------------------------------------------------------
EPI_ISL_413588      ------------------------------------------------------------
EPI_ISL_413589      ------------------------------------------------------------
EPI_ISL_424221      ------------------------------------------------------------
EPI_ISL_424233      ------------------------------------------------------------
EPI_ISL_424236      ------------------------------------------------------------
EPI_ISL_424238      ------------------------------------------------------------
EPI_ISL_424248      ------------------------------------------------------------
EPI_ISL_424254      ------------------------------------------------------------
EPI_ISL_425548      ------------------------------------------------------------
EPI_ISL_425581      ------------------------------------------------------------
EPI_ISL_426881      ------------------------------------------------------------
EPI_ISL_426883      ------------------------------------------------------------
EPI_ISL_426888      ------------------------------------------------------------
EPI_ISL_426900      ------------------------------------------------------------
EPI_ISL_426901      ------------------------------------------------------------
EPI_ISL_426903      ------------------------------------------------------------
EPI_ISL_426904      ------------------------------------------------------------
EPI_ISL_426907      ------------------------------------------------------------
EPI_ISL_426910      ------------------------------------------------------------
```

```
ORF1ab         6421 DAYNMMISAGFSLWVYKQFDTYNLWNTFTRLQSLENVAFNVVNKGHFDGQQGEVPVSIIN
EPI_ISL_413575      ------------------------------------------------------------
EPI_ISL_413588      ------------------------------------------------------------
EPI_ISL_413589      ------------------------------------------------------------
EPI_ISL_424221      ------------------------------------------------------------
EPI_ISL_424233      ------------------------------------------------------------
EPI_ISL_424236      ------------------------------------------------------------
EPI_ISL_424238      ------------------------------------------------------------
EPI_ISL_424248      ------------------------------------------------------------
EPI_ISL_424254      ------------------------------------------------------------
EPI_ISL_425548      ------------------------------------------------------------
EPI_ISL_425581      ------------------------------------------------------------
EPI_ISL_426881      ------------------------------------------------------------
EPI_ISL_426883      ------------------------------------------------------------
EPI_ISL_426888      ------------------------------------------------------------
EPI_ISL_426900      ------------------------------------------------------------
EPI_ISL_426901      ------------------------------------------------------------
EPI_ISL_426903      ------------------------------------------------------------
EPI_ISL_426904      ------------------------------------------------------------
EPI_ISL_426907      ------------------------------------------------------------
EPI_ISL_426910      ------------------------------------------------------------
```


```
ORF1ab         6421 DAYNMMISAGFSLWVYKQFDTYNLWNTFTRLQSLENVAFNVVNKGHFDGQQGEVPVSIIN
EPI_ISL_413575      ------------------------------------------------------------
EPI_ISL_413588      ------------------------------------------------------------
EPI_ISL_413589      ------------------------------------------------------------
EPI_ISL_424221      ------------------------------------------------------------
EPI_ISL_424233      ------------------------------------------------------------
EPI_ISL_424236      ------------------------------------------------------------
EPI_ISL_424238      ------------------------------------------------------------
EPI_ISL_424248      ------------------------------------------------------------
EPI_ISL_424254      ------------------------------------------------------------
EPI_ISL_425548      ------------------------------------------------------------
EPI_ISL_425581      ------------------------------------------------------------
EPI_ISL_426881      ------------------------------------------------------------
EPI_ISL_426883      ------------------------------------------------------------
EPI_ISL_426888      ------------------------------------------------------------
EPI_ISL_426900      ------------------------------------------------------------
EPI_ISL_426901      ------------------------------------------------------------
EPI_ISL_426903      ------------------------------------------------------------
EPI_ISL_426904      ------------------------------------------------------------
EPI_ISL_426907      ------------------------------------------------------------
EPI_ISL_426910      ------------------------------------------------------------
```

```
ORF1ab          6481 NTVYTKVDGVDVELFENKTTLPVNVAFELWAKRNIKPVPEVKILNNLGVDIAANTVIWDY
EPI_ISL_413575       ------------------------------------------------------------
EPI_ISL_413588       ------------------------------------------------------------
EPI_ISL_413589       ------------------------------------------------------------
EPI_ISL_424221       ------------------------------------------------------------
EPI_ISL_424233       ------------------------------------------------------------
EPI_ISL_424236       ------------------------------------------------------------
EPI_ISL_424238       ------------------------------------------------------------
EPI_ISL_424248       ------------------------------------------------------------
EPI_ISL_424254       ------------------------------------------------------------
EPI_ISL_425548       ------------------------------------------------------------
EPI_ISL_425581       ------------------------------------------------------------
EPI_ISL_426881       ------------------------------------------------------------
EPI_ISL_426883       ------------------------------------------------------------
EPI_ISL_426888       ------------------------------------------------------------
EPI_ISL_426900       ------------------------------------------------------------
EPI_ISL_426901       ------------------------------------------------------------
EPI_ISL_426903       ------------------------------------------------------------
EPI_ISL_426904       ------------------------------------------------------------
EPI_ISL_426907       ------------------------------------------------------------
EPI_ISL_426910       ------------------------------------------------------------
```


```
ORF1ab          6481 NTVYTKVDGVDVELFENKTTLPVNVAFELWAKRNIKPVPEVKILNNLGVDIAANTVIWDY
EPI_ISL_413575       ------------------------------------------------------------
EPI_ISL_413588       ------------------------------------------------------------
EPI_ISL_413589       ------------------------------------------------------------
EPI_ISL_424221       ------------------------------------------------------------
EPI_ISL_424233       ------------------------------------------------------------
EPI_ISL_424236       ------------------------------------------------------------
EPI_ISL_424238       ------------------------------------------------------------
EPI_ISL_424248       ------------------------------------------------------------
EPI_ISL_424254       ------------------------------------------------------------
EPI_ISL_425548       ------------------------------------------------------------
EPI_ISL_425581       ------------------------------------------------------------
EPI_ISL_426881       ------------------------------------------------------------
EPI_ISL_426883       ------------------------------------------------------------
EPI_ISL_426888       ------------------------------------------------------------
EPI_ISL_426900       ------------------------------------------------------------
EPI_ISL_426901       ------------------------------------------------------------
EPI_ISL_426903       ------------------------------------------------------------
EPI_ISL_426904       ------------------------------------------------------------
EPI_ISL_426907       ------------------------------------------------------------
EPI_ISL_426910       ------------------------------------------------------------
```

```
ORF1ab         6541 KRDAPAHISTIGVCSMTDIAKKPTETICAPLTVFFDGRVDGQVDLFRNARNGVLITEGSV
EPI_ISL_413575      ------------------------------------------------------------
EPI_ISL_413588      ------------------------------------------------------------
EPI_ISL_413589      ------------------------------------------------------------
EPI_ISL_424221      ------------------------------------------------------------
EPI_ISL_424233      ------------------------------------------------------------
EPI_ISL_424236      ------------------------------------------------------------
EPI_ISL_424238      ------------------------------------------------------------
EPI_ISL_424248      ------------------------------------------------------------
EPI_ISL_424254      ------------------------------------------------------------
EPI_ISL_425548      ------------------------------------------------------------
EPI_ISL_425581      ------------------------------------------------------------
EPI_ISL_426881      ------------------------------------------------------------
EPI_ISL_426883      ------------------------------------------------------------
EPI_ISL_426888      ------------------------------------------------------------
EPI_ISL_426900      ------------------------------------------------------------
EPI_ISL_426901      ------------------------------------------------------------
EPI_ISL_426903      ------------------------------------------------------------
EPI_ISL_426904      ------------------------------------------------------------
EPI_ISL_426907      ------------------------------------------------------------
EPI_ISL_426910      ------------------------------------------------------------
ORF1ab         6541 KRDAPAHISTIGVCSMTDIAKKPTETICAPLTVFFDGRVDGQVDLFRNARNGVLITEGSV
EPI_ISL_413575      ------------------------------------------------------------
EPI_ISL_413588      ------------------------------------------------------------
EPI_ISL_413589      ------------------------------------------------------------
EPI_ISL_424221      ------------------------------------------------------------
EPI_ISL_424233      ------------------------------------------------------------
EPI_ISL_424236      ------------------------------------------------------------
EPI_ISL_424238      ------------------------------------------------------------
EPI_ISL_424248      ------------------------------------------------------------
EPI_ISL_424254      ------------------------------------------------------------
EPI_ISL_425548      ------------------------------------------------------------
EPI_ISL_425581      ------------------------------------------------------------
EPI_ISL_426881      ------------------------------------------------------------
EPI_ISL_426883      ------------------------------------------------------------
EPI_ISL_426888      ------------------------------------------------------------
EPI_ISL_426900      ------------------------------------------------------------
EPI_ISL_426901      ------------------------------------------------------------
EPI_ISL_426903      ------------------------------------------------------------
EPI_ISL_426904      ------------------------------------------------------------
EPI_ISL_426907      ------------------------------------------------------------
EPI_ISL_426910      ------------------------------------------------------------
```

```
ORF1ab         6601 KGLQPSVGPKQASLNGVTLIGEAVKTQFNYYKKVDGVVQQLPETYFTQSRNLQEFKPRSQ
EPI_ISL_413575      ------------------------------------------------------------
EPI_ISL_413588      ------------------------------------------------------------
EPI_ISL_413589      ------------------------------------------------------------
EPI_ISL_424221      ------------------------------------------------------------
EPI_ISL_424233      ------------------------------------------------------------
EPI_ISL_424236      ------------------------------------------------------------
EPI_ISL_424238      ------------------------------------------------------------
EPI_ISL_424248      ------------------------------------------------------------
EPI_ISL_424254      ------------------------------------------------------------
EPI_ISL_425548      ------------------------------------------------------------
EPI_ISL_425581      ------------------------------------------------------------
EPI_ISL_426881      ------------------------------------------------------------
EPI_ISL_426883      ------------------------------------------------------------
EPI_ISL_426888      ------------------------------------------------------------
EPI_ISL_426900      ------------------------------------------------------------
EPI_ISL_426901      ------------------------------------------------------------
EPI_ISL_426903      ------------------------------------------------------------
EPI_ISL_426904      ------------------------------------------------------------
EPI_ISL_426907      ------------------------------------------------------------
EPI_ISL_426910      ------------------------------------------------------------
```

```
ORF1ab         6601 KGLQPSVGPKQASLNGVTLIGEAVKTQFNYYKKVDGVVQQLPETYFTQSRNLQEFKPRSQ
EPI_ISL_413575      ------------------------------------------------------------
EPI_ISL_413588      ------------------------------------------------------------
EPI_ISL_413589      ------------------------------------------------------------
EPI_ISL_424221      ------------------------------------------------------------
EPI_ISL_424233      ------------------------------------------------------------
EPI_ISL_424236      ------------------------------------------------------------
EPI_ISL_424238      ------------------------------------------------------------
EPI_ISL_424248      ------------------------------------------------------------
EPI_ISL_424254      ------------------------------------------------------------
EPI_ISL_425548      ------------------------------------------------------------
EPI_ISL_425581      ------------------------------------------------------------
EPI_ISL_426881      ------------------------------------------------------------
EPI_ISL_426883      ------------------------------------------------------------
EPI_ISL_426888      ------------------------------------------------------------
EPI_ISL_426900      ------------------------------------------------------------
EPI_ISL_426901      ------------------------------------------------------------
EPI_ISL_426903      ------------------------------------------------------------
EPI_ISL_426904      ------------------------------------------------------------
EPI_ISL_426907      ------------------------------------------------------------
EPI_ISL_426910      ------------------------------------------------------------
```

```
ORF1ab          6661 MEIDFLELAMDEFIERYKLEGYAFEHIVYGDFSHSQLGGLHLLIGLAKRFKESPFELEDF
EPI_ISL_413575       ------------------------------------------------------------
EPI_ISL_413588       ------------------------------------------------------------
EPI_ISL_413589       ------------------------------------------------------------
EPI_ISL_424221       ------------------------------------------------------------
EPI_ISL_424233       ------------------------------------------------------------
EPI_ISL_424236       ------------------------------------------------------------
EPI_ISL_424238       ------------------------------------------------------------
EPI_ISL_424248       ------------------------------------------------------------
EPI_ISL_424254       ------------------------------------------------------------
EPI_ISL_425548       ------------------------------------------------------------
EPI_ISL_425581       ------------------------------------------------------------
EPI_ISL_426881       ------------------------------------------------------------
EPI_ISL_426883       ------------------------------------------------------------
EPI_ISL_426888       ------------------------------------------------------------
EPI_ISL_426900       ------------------------------------------------------------
EPI_ISL_426901       ------------------------------------------------------------
EPI_ISL_426903       ------------------------------------------------------------
EPI_ISL_426904       ------------------------------------------------------------
EPI_ISL_426907       ------------------------------------------------------------
EPI_ISL_426910       ------------------------------------------------------------
```

```
ORF1ab          6661 MEIDFLELAMDEFIERYKLEGYAFEHIVYGDFSHSQLGGLHLLIGLAKRFKESPFELEDF
EPI_ISL_413575       ------------------------------------------------------------
EPI_ISL_413588       ------------------------------------------------------------
EPI_ISL_413589       ------------------------------------------------------------
EPI_ISL_424221       ------------------------------------------------------------
EPI_ISL_424233       ------------------------------------------------------------
EPI_ISL_424236       ------------------------------------------------------------
EPI_ISL_424238       ------------------------------------------------------------
EPI_ISL_424248       ------------------------------------------------------------
EPI_ISL_424254       ------------------------------------------------------------
EPI_ISL_425548       ------------------------------------------------------------
EPI_ISL_425581       ------------------------------------------------------------
EPI_ISL_426881       ------------------------------------------------------------
EPI_ISL_426883       ------------------------------------------------------------
EPI_ISL_426888       ------------------------------------------------------------
EPI_ISL_426900       ------------------------------------------------------------
EPI_ISL_426901       ------------------------------------------------------------
EPI_ISL_426903       ------------------------------------------------------------
EPI_ISL_426904       ------------------------------------------------------------
EPI_ISL_426907       ------------------------------------------------------------
EPI_ISL_426910       ------------------------------------------------------------
```

```
ORF1ab         6721 IPMDSTVKNYFITDAQTGSSKCVCSVIDLLLDDFVEIIKSQDLSVVSKVVKVTIDYTEIS
EPI_ISL_413575      ------------------------------------------------------------
EPI_ISL_413588      ------------------------------------------------------------
EPI_ISL_413589      ------------------------------------------------------------
EPI_ISL_424221      ------------------------------------------------------------
EPI_ISL_424233      ------------------------------------------------------------
EPI_ISL_424236      ------------------------------------------------------------
EPI_ISL_424238      ------------------------------------------------------------
EPI_ISL_424248      ------------------------------------------------------------
EPI_ISL_424254      ------------------------------------------------------------
EPI_ISL_425548      ------------------------------------------------------------
EPI_ISL_425581      ------------------------------------------------------------
EPI_ISL_426881      ------------------------------------------------------------
EPI_ISL_426883      ------------------------------------------------------------
EPI_ISL_426888      ------------------------------------------------------------
EPI_ISL_426900      ------------------------------------------------------------
EPI_ISL_426901      ------------------------------------------------------------
EPI_ISL_426903      ------------------------------------------------------------
EPI_ISL_426904      ------------------------------------------------------------
EPI_ISL_426907      ------------------------------------------------------------
EPI_ISL_426910      ------------------------------------------------------------
```

```
ORF1ab         6721 IPMDSTVKNYFITDAQTGSSKCVCSVIDLLLDDFVEIIKSQDLSVVSKVVKVTIDYTEIS
EPI_ISL_413575      ------------------------------------------------------------
EPI_ISL_413588      ------------------------------------------------------------
EPI_ISL_413589      ------------------------------------------------------------
EPI_ISL_424221      ------------------------------------------------------------
EPI_ISL_424233      ------------------------------------------------------------
EPI_ISL_424236      ------------------------------------------------------------
EPI_ISL_424238      ------------------------------------------------------------
EPI_ISL_424248      ------------------------------------------------------------
EPI_ISL_424254      ------------------------------------------------------------
EPI_ISL_425548      ------------------------------------------------------------
EPI_ISL_425581      ------------------------------------------------------------
EPI_ISL_426881      ------------------------------------------------------------
EPI_ISL_426883      ------------------------------------------------------------
EPI_ISL_426888      ------------------------------------------------------------
EPI_ISL_426900      ------------------------------------------------------------
EPI_ISL_426901      ------------------------------------------------------------
EPI_ISL_426903      ------------------------------------------------------------
EPI_ISL_426904      ------------------------------------------------------------
EPI_ISL_426907      ------------------------------------------------------------
EPI_ISL_426910      ------------------------------------------------------------
```

```
ORF1ab         6781 FMLWCKDGHVETFYPKLQSSQAWQPGVAMPNLYKMQRMLLEKCDLQNYGDSATLPKGIMM
EPI_ISL_413575      ------------------------------------------------------------
EPI_ISL_413588      ------------------------------------------------------------
EPI_ISL_413589      ------------------------------------------------------------
EPI_ISL_424221      ------------------------------------------------------------
EPI_ISL_424233      ------------------------------------------------------------
EPI_ISL_424236      ------------------------------------------------------------
EPI_ISL_424238      ------------------------------------------------------------
EPI_ISL_424248      ------------------------------------------------------------
EPI_ISL_424254      ------------------------------------------------------------
EPI_ISL_425548      ------------------------------------------------------------
EPI_ISL_425581      ------------------------------------------------------------
EPI_ISL_426881      ------------------------------------------------------------
EPI_ISL_426883      ------------------------------------------------------------
EPI_ISL_426888      ------------------------------------------------------------
EPI_ISL_426900      ------------------------------------------------------------
EPI_ISL_426901      ------------------------------------------------------------
EPI_ISL_426903      ------------------------------------------------------------
EPI_ISL_426904      ------------------------------------------------------------
EPI_ISL_426907      ------------------------------------------------------------
EPI_ISL_426910      ------------------------------------------------------------
```

```
ORF1ab         6781 FMLWCKDGHVETFYPKLQSSQAWQPGVAMPNLYKMQRMLLEKCDLQNYGDSATLPKGIMM
EPI_ISL_413575      ------------------------------------------------------------
EPI_ISL_413588      ------------------------------------------------------------
EPI_ISL_413589      ------------------------------------------------------------
EPI_ISL_424221      ------------------------------------------------------------
EPI_ISL_424233      ------------------------------------------------------------
EPI_ISL_424236      ------------------------------------------------------------
EPI_ISL_424238      ------------------------------------------------------------
EPI_ISL_424248      ------------------------------------------------------------
EPI_ISL_424254      ------------------------------------------------------------
EPI_ISL_425548      ------------------------------------------------------------
EPI_ISL_425581      ------------------------------------------------------------
EPI_ISL_426881      ------------------------------------------------------------
EPI_ISL_426883      ------------------------------------------------------------
EPI_ISL_426888      ------------------------------------------------------------
EPI_ISL_426900      ------------------------------------------------------------
EPI_ISL_426901      ------------------------------------------------------------
EPI_ISL_426903      ------------------------------------------------------------
EPI_ISL_426904      ------------------------------------------------------------
EPI_ISL_426907      ------------------------------------------------------------
EPI_ISL_426910      ------------------------------------------------------------
```

```
ORF1ab         6841 NVAKYTQLCQYLNTLTLAVPYNMRVIHFGAGSDKGVAPGTAVLRQWLPTGTLLVDSDLND
EPI_ISL_413575      ------------------------------------------------------------
EPI_ISL_413588      ------------------------------------------------------------
EPI_ISL_413589      ------------------------------------------------------------
EPI_ISL_424221      ------------------------------------------------------------
EPI_ISL_424233      ------------------------------------------------------------
EPI_ISL_424236      ------------------------------------------------------------
EPI_ISL_424238      ------------------------------------------------------------
EPI_ISL_424248      ------------------------------------------------------------
EPI_ISL_424254      ------------------------------------------------------------
EPI_ISL_425548      ------------------------------------------------------------
EPI_ISL_425581      ------------------------------------------------------------
EPI_ISL_426881      ------------------------------------------------------------
EPI_ISL_426883      ------------------------------------------------------------
EPI_ISL_426888      ------------------------------------------------------------
EPI_ISL_426900      ------------------------------------------------------------
EPI_ISL_426901      ------------------------------------------------------------
EPI_ISL_426903      ------------------------------------------------------------
EPI_ISL_426904      ------------------------------------------------------------
EPI_ISL_426907      ------------------------------------------------------------
EPI_ISL_426910      ------------------------------------------------------------
```

```
ORF1ab         6841 NVAKYTQLCQYLNTLTLAVPYNMRVIHFGAGSDKGVAPGTAVLRQWLPTGTLLVDSDLND
EPI_ISL_413575      ------------------------------------------------------------
EPI_ISL_413588      ------------------------------------------------------------
EPI_ISL_413589      ------------------------------------------------------------
EPI_ISL_424221      ------------------------------------------------------------
EPI_ISL_424233      ------------------------------------------------------------
EPI_ISL_424236      ------------------------------------------------------------
EPI_ISL_424238      ------------------------------------------------------------
EPI_ISL_424248      ------------------------------------------------------------
EPI_ISL_424254      ------------------------------------------------------------
EPI_ISL_425548      ------------------------------------------------------------
EPI_ISL_425581      ------------------------------------------------------------
EPI_ISL_426881      ------------------------------------------------------------
EPI_ISL_426883      ------------------------------------------------------------
EPI_ISL_426888      ------------------------------------------------------------
EPI_ISL_426900      ------------------------------------------------------------
EPI_ISL_426901      ------------------------------------------------------------
EPI_ISL_426903      ------------------------------------------------------------
EPI_ISL_426904      ------------------------------------------------------------
EPI_ISL_426907      ------------------------------------------------------------
EPI_ISL_426910      ------------------------------------------------------------
```

```
ORF1ab         6901 FVSDADSTLIGDCATVHTANKWDLIISDMYDPKTKNVTKENDSKEGFFTYICGFIQQKLA
EPI_ISL_413575      ------------------------------------------------------------
EPI_ISL_413588      ------------------------------------------------------------
EPI_ISL_413589      ------------------------------------------------------------
EPI_ISL_424221      ------------------------------------------------------------
EPI_ISL_424233      ------------------------------------------------------------
EPI_ISL_424236      ------------------------------------------------------------
EPI_ISL_424238      ------------------------------------------------------------
EPI_ISL_424248      ------------------------------------------------------------
EPI_ISL_424254      ------------------------------------------------------------
EPI_ISL_425548      ------------------------------------------------------------
EPI_ISL_425581      ------------------------------------------------------------
EPI_ISL_426881      ------------------------------------------------------------
EPI_ISL_426883      ------------------------------------------------------------
EPI_ISL_426888      ------------------------------------------------------------
EPI_ISL_426900      ------------------------------------------------------------
EPI_ISL_426901      ------------------------------------------------------------
EPI_ISL_426903      ------------------------------------------------------------
EPI_ISL_426904      ------------------------------------------------------------
EPI_ISL_426907      ------------------------------------------------------------
EPI_ISL_426910      ------------------------------------------------------------
```

```
ORF1ab         6901 FVSDADSTLIGDCATVHTANKWDLIISDMYDPKTKNVTKENDSKEGFFTYICGFIQQKLA
EPI_ISL_413575      ------------------------------------------------------------
EPI_ISL_413588      ------------------------------------------------------------
EPI_ISL_413589      ------------------------------------------------------------
EPI_ISL_424221      ------------------------------------------------------------
EPI_ISL_424233      ------------------------------------------------------------
EPI_ISL_424236      ------------------------------------------------------------
EPI_ISL_424238      ------------------------------------------------------------
EPI_ISL_424248      ------------------------------------------------------------
EPI_ISL_424254      ------------------------------------------------------------
EPI_ISL_425548      ------------------------------------------------------------
EPI_ISL_425581      ------------------------------------------------------------
EPI_ISL_426881      ------------------------------------------------------------
EPI_ISL_426883      ------------------------------------------------------------
EPI_ISL_426888      ------------------------------------------------------------
EPI_ISL_426900      ------------------------------------------------------------
EPI_ISL_426901      ------------------------------------------------------------
EPI_ISL_426903      ------------------------------------------------------------
EPI_ISL_426904      ------------------------------------------------------------
EPI_ISL_426907      ------------------------------------------------------------
EPI_ISL_426910      ------------------------------------------------------------
```

```
ORF1ab         6961 LGGSVAIKITEHSWNADLYKLMGHFAWWTAFVTNVNASSSEAFLIGCNYLGKPREQIDGY
EPI_ISL_413575      ------------------------------------------------------------
EPI_ISL_413588      ------------------------------------------------------------
EPI_ISL_413589      ------------------------------------------------------------
EPI_ISL_424221      ------------------------------------------------------------
EPI_ISL_424233      ------------------------------------------------------------
EPI_ISL_424236      ------------------------------------------------------------
EPI_ISL_424238      ------------------------------------------------------------
EPI_ISL_424248      ------------------------------------------------------------
EPI_ISL_424254      ------------------------------------------------------------
EPI_ISL_425548      ------------------------------------------------------------
EPI_ISL_425581      ------------------------------------------------------------
EPI_ISL_426881      ------------------------------------------------------------
EPI_ISL_426883      ------------------------------------------------------------
EPI_ISL_426888      ------------------------------------------------------------
EPI_ISL_426900      ------------------------------------------------------------
EPI_ISL_426901      ------------------------------------------------------------
EPI_ISL_426903      ------------------------------------------------------------
EPI_ISL_426904      ------------------------------------------------------------
EPI_ISL_426907      ------------------------------------------------------------
EPI_ISL_426910      ------------------------------------------------------------
```


```
ORF1ab         6961 LGGSVAIKITEHSWNADLYKLMGHFAWWTAFVTNVNASSSEAFLIGCNYLGKPREQIDGY
EPI_ISL_413575      ------------------------------------------------------------
EPI_ISL_413588      ------------------------------------------------------------
EPI_ISL_413589      ------------------------------------------------------------
EPI_ISL_424221      ------------------------------------------------------------
EPI_ISL_424233      ------------------------------------------------------------
EPI_ISL_424236      ------------------------------------------------------------
EPI_ISL_424238      ------------------------------------------------------------
EPI_ISL_424248      ------------------------------------------------------------
EPI_ISL_424254      ------------------------------------------------------------
EPI_ISL_425548      ------------------------------------------------------------
EPI_ISL_425581      ------------------------------------------------------------
EPI_ISL_426881      ------------------------------------------------------------
EPI_ISL_426883      ------------------------------------------------------------
EPI_ISL_426888      ------------------------------------------------------------
EPI_ISL_426900      ------------------------------------------------------------
EPI_ISL_426901      ------------------------------------------------------------
EPI_ISL_426903      ------------------------------------------------------------
EPI_ISL_426904      ------------------------------------------------------------
EPI_ISL_426907      ------------------------------------------------------------
EPI_ISL_426910      ------------------------------------------------------------
```

```
ORF1ab          7021 VMHANYIFWRNTNPIQLSSYSLFDMSKFPLKLRGTAVMSLKEGQINDMILSLLSKGRLII
EPI_ISL_413575       ------------------------------------------------------------
EPI_ISL_413588       ------------------------------------------------------------
EPI_ISL_413589       ------------------------------------------------------------
EPI_ISL_424221       ------------------------------------------------------------
EPI_ISL_424233       ------------------------------------------------------------
EPI_ISL_424236       ------------------------------------------------------------
EPI_ISL_424238       ------------------------------------------------------------
EPI_ISL_424248       ------------------------------------------------------------
EPI_ISL_424254       ------------------------------------------------------------
EPI_ISL_425548       ------------------------------------------------------------
EPI_ISL_425581       ------------------------------------------------------------
EPI_ISL_426881       ------------------------------------------------------------
EPI_ISL_426883       ------------------------------------------------------------
EPI_ISL_426888       ------------------------------------------------------------
EPI_ISL_426900       ------------------------------------------------------------
EPI_ISL_426901       ------------------------------------------------------------
EPI_ISL_426903       ------------------------------------------------------------
EPI_ISL_426904       ------------------------------------------------------------
EPI_ISL_426907       ------------------------------------------------------------
EPI_ISL_426910       ------------------------------------------------------------
```

```
ORF1ab          7021 VMHANYIFWRNTNPIQLSSYSLFDMSKFPLKLRGTAVMSLKEGQINDMILSLLSKGRLII
EPI_ISL_413575       ------------------------------------------------------------
EPI_ISL_413588       ------------------------------------------------------------
EPI_ISL_413589       ------------------------------------------------------------
EPI_ISL_424221       ------------------------------------------------------------
EPI_ISL_424233       ------------------------------------------------------------
EPI_ISL_424236       ------------------------------------------------------------
EPI_ISL_424238       ------------------------------------------------------------
EPI_ISL_424248       ------------------------------------------------------------
EPI_ISL_424254       ------------------------------------------------------------
EPI_ISL_425548       ------------------------------------------------------------
EPI_ISL_425581       ------------------------------------------------------------
EPI_ISL_426881       ------------------------------------------------------------
EPI_ISL_426883       ------------------------------------------------------------
EPI_ISL_426888       ------------------------------------------------------------
EPI_ISL_426900       ------------------------------------------------------------
EPI_ISL_426901       ------------------------------------------------------------
EPI_ISL_426903       ------------------------------------------------------------
EPI_ISL_426904       ------------------------------------------------------------
EPI_ISL_426907       ------------------------------------------------------------
EPI_ISL_426910       ------------------------------------------------------------
```

```
ORF1ab         7081 RENNRVVISSDVLVNN
EPI_ISL_413575      ----------------
EPI_ISL_413588      ----------------
EPI_ISL_413589      ----------------
EPI_ISL_424221      ----------------
EPI_ISL_424233      ----------------
EPI_ISL_424236      ----------------
EPI_ISL_424238      ----------------
EPI_ISL_424248      ----------------
EPI_ISL_424254      ----------------
EPI_ISL_425548      ----------------
EPI_ISL_425581      ----------------
EPI_ISL_426881      ----------------
EPI_ISL_426883      ----------------
EPI_ISL_426888      ----------------
EPI_ISL_426900      ----------------
EPI_ISL_426901      ----------------
EPI_ISL_426903      ----------------
EPI_ISL_426904      ----------------
EPI_ISL_426907      ----------------
EPI_ISL_426910      ----------------
ORF1ab         7081 RENNRVVISSDVLVNN
EPI_ISL_413575      ----------------
EPI_ISL_413588      ----------------
EPI_ISL_413589      ----------------
EPI_ISL_424221      ----------------
EPI_ISL_424233      ----------------
EPI_ISL_424236      ----------------
EPI_ISL_424238      ----------------
EPI_ISL_424248      ----------------
EPI_ISL_424254      ----------------
EPI_ISL_425548      ----------------
EPI_ISL_425581      ----------------
EPI_ISL_426881      ----------------
EPI_ISL_426883      ----------------
EPI_ISL_426888      ----------------
EPI_ISL_426900      ----------------
EPI_ISL_426901      ----------------
EPI_ISL_426903      ----------------
EPI_ISL_426904      ----------------
EPI_ISL_426907      ----------------
EPI_ISL_426910      ----------------
```

Clade GH

```
ORF1ab            1 MESLVPGFNEKTHVQLSLPVLQVRDVLVRGFGDSVEEVLSEARQHLKDGTCGLVEVEKGV
EPI_ISL_413575    1 ------------------------------------------------------------
EPI_ISL_413588    1 ------------------------------------------------------------
EPI_ISL_413589    1 ------------------------------------------------------------
EPI_ISL_424221    1 ------------------------------------------------------------
EPI_ISL_424233    1 ------------------------------------------------------------
EPI_ISL_424236    1 ------------------------------------------------------------
EPI_ISL_424238    1 ------------------------------------------------------------
EPI_ISL_424248    1 ------------------------------------------------------------
EPI_ISL_424254    1 ------------------------------------------------------------
EPI_ISL_425548    1 ------------------------------------------------------------
EPI_ISL_425581    1 ------------------------------------------------------------
EPI_ISL_426881    1 ------------------------------------------------------------
EPI_ISL_426883    1 ------------------------------------------------------------
EPI_ISL_426888    1 ------------------------------------------------------------
EPI_ISL_426900    1 ------------------------------------------------------------
EPI_ISL_426901    1 ------------------------------------------------------------
EPI_ISL_426903    1 ------------------------------------------------------------
EPI_ISL_426904    1 ------------------------------------------------------------
EPI_ISL_426907    1 ------------------------------------------------------------
EPI_ISL_426910    1 ------------------------------------------------------------
```


```
ORF1ab            1 MESLVPGFNEKTHVQLSLPVLQVRDVLVRGFGDSVEEVLSEARQHLKDGTCGLVEVEKGV
EPI_ISL_413575    1 ------------------------------------------------------------
EPI_ISL_413588    1 ------------------------------------------------------------
EPI_ISL_413589    1 ------------------------------------------------------------
EPI_ISL_424221    1 ------------------------------------------------------------
EPI_ISL_424233    1 ------------------------------------------------------------
EPI_ISL_424236    1 ------------------------------------------------------------
EPI_ISL_424238    1 ------------------------------------------------------------
EPI_ISL_424248    1 ------------------------------------------------------------
EPI_ISL_424254    1 ------------------------------------------------------------
EPI_ISL_425548    1 ------------------------------------------------------------
EPI_ISL_425581    1 ------------------------------------------------------------
EPI_ISL_426881    1 ------------------------------------------------------------
EPI_ISL_426883    1 ------------------------------------------------------------
EPI_ISL_426888    1 ------------------------------------------------------------
EPI_ISL_426900    1 ------------------------------------------------------------
EPI_ISL_426901    1 ------------------------------------------------------------
EPI_ISL_426903    1 ------------------------------------------------------------
EPI_ISL_426904    1 ------------------------------------------------------------
EPI_ISL_426907    1 ------------------------------------------------------------
EPI_ISL_426910    1 ------------------------------------------------------------
```

```
ORF1ab           61 LPQLEQPYVFIKRSDARTAPHGHVMVELVAELEGIQYGRSGETLGVLVPHVGEIPVAYRK
EPI_ISL_413575    1 ------------------------------------------------------------
EPI_ISL_413588    1 ------------------------------------------------------------
EPI_ISL_413589    1 ------------------------------------------------------------
EPI_ISL_424221    1 ------------------------------------------------------------
EPI_ISL_424233    1 ------------------------------------------------------------
EPI_ISL_424236    1 ------------------------------------------------------------
EPI_ISL_424238    1 ------------------------------------------------------------
EPI_ISL_424248    1 ------------------------------------------------------------
EPI_ISL_424254    1 ------------------------------------------------------------
EPI_ISL_425548    1 ------------------------------------------------------------
EPI_ISL_425581    1 ------------------------------------------------------------
EPI_ISL_426881    1 ------------------------------------------------------------
EPI_ISL_426883    1 ------------------------------------------------------------
EPI_ISL_426888    1 ------------------------------------------------------------
EPI_ISL_426900    1 ------------------------------------------------------------
EPI_ISL_426901    1 ------------------------------------------------------------
EPI_ISL_426903    1 ------------------------------------------------------------
EPI_ISL_426904    1 ------------------------------------------------------------
EPI_ISL_426907    1 ------------------------------------------------------------
EPI_ISL_426910    1 ------------------------------------------------------------
```

```
ORF1ab           61 LPQLEQPYVFIKRSDARTAPHGHVMVELVAELEGIQYGRSGETLGVLVPHVGEIPVAYRK
EPI_ISL_413575    1 ------------------------------------------------------------
EPI_ISL_413588    1 ------------------------------------------------------------
EPI_ISL_413589    1 ------------------------------------------------------------
EPI_ISL_424221    1 ------------------------------------------------------------
EPI_ISL_424233    1 ------------------------------------------------------------
EPI_ISL_424236    1 ------------------------------------------------------------
EPI_ISL_424238    1 ------------------------------------------------------------
EPI_ISL_424248    1 ------------------------------------------------------------
EPI_ISL_424254    1 ------------------------------------------------------------
EPI_ISL_425548    1 ------------------------------------------------------------
EPI_ISL_425581    1 ------------------------------------------------------------
EPI_ISL_426881    1 ------------------------------------------------------------
EPI_ISL_426883    1 ------------------------------------------------------------
EPI_ISL_426888    1 ------------------------------------------------------------
EPI_ISL_426900    1 ------------------------------------------------------------
EPI_ISL_426901    1 ------------------------------------------------------------
EPI_ISL_426903    1 ------------------------------------------------------------
EPI_ISL_426904    1 ------------------------------------------------------------
EPI_ISL_426907    1 ------------------------------------------------------------
EPI_ISL_426910    1 ------------------------------------------------------------
```

```
ORF1ab          121 VLLRKNGNKGAGGHSYGADLKSFDLGDELGTDPYEDFQENWNTKHSSGVTRELMRELNGG
EPI_ISL_413575    1 ------------------------------------------------------------
EPI_ISL_413588    1 ------------------------------------------------------------
EPI_ISL_413589    1 ------------------------------------------------------------
EPI_ISL_424221    1 ------------------------------------------------------------
EPI_ISL_424233    1 ------------------------------------------------------------
EPI_ISL_424236    1 ------------------------------------------------------------
EPI_ISL_424238    1 ------------------------------------------------------------
EPI_ISL_424248    1 ------------------------------------------------------------
EPI_ISL_424254    1 ------------------------------------------------------------
EPI_ISL_425548    1 ------------------------------------------------------------
EPI_ISL_425581    1 ------------------------------------------------------------
EPI_ISL_426881    1 ------------------------------------------------------------
EPI_ISL_426883    1 ------------------------------------------------------------
EPI_ISL_426888    1 ------------------------------------------------------------
EPI_ISL_426900    1 ------------------------------------------------------------
EPI_ISL_426901    1 ------------------------------------------------------------
EPI_ISL_426903    1 ------------------------------------------------------------
EPI_ISL_426904    1 ------------------------------------------------------------
EPI_ISL_426907    1 ------------------------------------------------------------
EPI_ISL_426910    1 ------------------------------------------------------------
```

```
ORF1ab          121 VLLRKNGNKGAGGHSYGADLKSFDLGDELGTDPYEDFQENWNTKHSSGVTRELMRELNGG
EPI_ISL_413575    1 ------------------------------------------------------------
EPI_ISL_413588    1 ------------------------------------------------------------
EPI_ISL_413589    1 ------------------------------------------------------------
EPI_ISL_424221    1 ------------------------------------------------------------
EPI_ISL_424233    1 ------------------------------------------------------------
EPI_ISL_424236    1 ------------------------------------------------------------
EPI_ISL_424238    1 ------------------------------------------------------------
EPI_ISL_424248    1 ------------------------------------------------------------
EPI_ISL_424254    1 ------------------------------------------------------------
EPI_ISL_425548    1 ------------------------------------------------------------
EPI_ISL_425581    1 ------------------------------------------------------------
EPI_ISL_426881    1 ------------------------------------------------------------
EPI_ISL_426883    1 ------------------------------------------------------------
EPI_ISL_426888    1 ------------------------------------------------------------
EPI_ISL_426900    1 ------------------------------------------------------------
EPI_ISL_426901    1 ------------------------------------------------------------
EPI_ISL_426903    1 ------------------------------------------------------------
EPI_ISL_426904    1 ------------------------------------------------------------
EPI_ISL_426907    1 ------------------------------------------------------------
EPI_ISL_426910    1 ------------------------------------------------------------
```

```
ORF1ab          181 AYTRYVDNNFCGPDGYPLECIKDLLARAGKASCTLSEQLDFIDTKRGVYCCREHEHEIAW
EPI_ISL_413575    1 ------------------------------------------------------------
EPI_ISL_413588    1 ------------------------------------------------------------
EPI_ISL_413589    1 ------------------------------------------------------------
EPI_ISL_424221    1 ------------------------------------------------------------
EPI_ISL_424233    1 ------------------------------------------------------------
EPI_ISL_424236    1 ------------------------------------------------------------
EPI_ISL_424238    1 ------------------------------------------------------------
EPI_ISL_424248    1 ------------------------------------------------------------
EPI_ISL_424254    1 ------------------------------------------------------------
EPI_ISL_425548    1 ------------------------------------------------------------
EPI_ISL_425581    1 ------------------------------------------------------------
EPI_ISL_426881    1 ------------------------------------------------------------
EPI_ISL_426883    1 ------------------------------------------------------------
EPI_ISL_426888    1 ------------------------------------------------------------
EPI_ISL_426900    1 ------------------------------------------------------------
EPI_ISL_426901    1 ------------------------------------------------------------
EPI_ISL_426903    1 ------------------------------------------------------------
EPI_ISL_426904    1 ------------------------------------------------------------
EPI_ISL_426907    1 ------------------------------------------------------------
EPI_ISL_426910    1 ------------------------------------------------------------
```

```
ORF1ab          181 AYTRYVDNNFCGPDGYPLECIKDLLARAGKASCTLSEQLDFIDTKRGVYCCREHEHEIAW
EPI_ISL_413575    1 ------------------------------------------------------------
EPI_ISL_413588    1 ------------------------------------------------------------
EPI_ISL_413589    1 ------------------------------------------------------------
EPI_ISL_424221    1 ------------------------------------------------------------
EPI_ISL_424233    1 ------------------------------------------------------------
EPI_ISL_424236    1 ------------------------------------------------------------
EPI_ISL_424238    1 ------------------------------------------------------------
EPI_ISL_424248    1 ------------------------------------------------------------
EPI_ISL_424254    1 ------------------------------------------------------------
EPI_ISL_425548    1 ------------------------------------------------------------
EPI_ISL_425581    1 ------------------------------------------------------------
EPI_ISL_426881    1 ------------------------------------------------------------
EPI_ISL_426883    1 ------------------------------------------------------------
EPI_ISL_426888    1 ------------------------------------------------------------
EPI_ISL_426900    1 ------------------------------------------------------------
EPI_ISL_426901    1 ------------------------------------------------------------
EPI_ISL_426903    1 ------------------------------------------------------------
EPI_ISL_426904    1 ------------------------------------------------------------
EPI_ISL_426907    1 ------------------------------------------------------------
EPI_ISL_426910    1 ------------------------------------------------------------
```

```
ORF1ab          241 YTERSEKSYELQTPFEIKLAKKFDTFNGECPNFVFPLNSIIKTIQPRVEKKKLDGFMGRI
EPI_ISL_413575    1 ------------------------------------------------------------
EPI_ISL_413588    1 ------------------------------------------------------------
EPI_ISL_413589    1 ------------------------------------------------------------
EPI_ISL_424221    1 ------------------------------------------------------------
EPI_ISL_424233    1 ------------------------------------------------------------
EPI_ISL_424236    1 ------------------------------------------------------------
EPI_ISL_424238    1 ------------------------------------------------------------
EPI_ISL_424248    1 ------------------------------------------------------------
EPI_ISL_424254    1 ------------------------------------------------------------
EPI_ISL_425548    1 ------------------------------------------------------------
EPI_ISL_425581    1 ------------------------------------------------------------
EPI_ISL_426881    1 ------------------------------------------------------------
EPI_ISL_426883    1 ------------------------------------------------------------
EPI_ISL_426888    1 ------------------------------------------------------------
EPI_ISL_426900    1 ------------------------------------------------------------
EPI_ISL_426901    1 ------------------------------------------------------------
EPI_ISL_426903    1 ------------------------------------------------------------
EPI_ISL_426904    1 ------------------------------------------------------------
EPI_ISL_426907    1 ------------------------------------------------------------
EPI_ISL_426910    1 ------------------------------------------------------------
```


```
ORF1ab          241 YTERSEKSYELQTPFEIKLAKKFDTFNGECPNFVFPLNSIIKTIQPRVEKKKLDGFMGRI
EPI_ISL_413575    1 ------------------------------------------------------------
EPI_ISL_413588    1 ------------------------------------------------------------
EPI_ISL_413589    1 ------------------------------------------------------------
EPI_ISL_424221    1 ------------------------------------------------------------
EPI_ISL_424233    1 ------------------------------------------------------------
EPI_ISL_424236    1 ------------------------------------------------------------
EPI_ISL_424238    1 ------------------------------------------------------------
EPI_ISL_424248    1 ------------------------------------------------------------
EPI_ISL_424254    1 ------------------------------------------------------------
EPI_ISL_425548    1 ------------------------------------------------------------
EPI_ISL_425581    1 ------------------------------------------------------------
EPI_ISL_426881    1 ------------------------------------------------------------
EPI_ISL_426883    1 ------------------------------------------------------------
EPI_ISL_426888    1 ------------------------------------------------------------
EPI_ISL_426900    1 ------------------------------------------------------------
EPI_ISL_426901    1 ------------------------------------------------------------
EPI_ISL_426903    1 ------------------------------------------------------------
EPI_ISL_426904    1 ------------------------------------------------------------
EPI_ISL_426907    1 ------------------------------------------------------------
EPI_ISL_426910    1 ------------------------------------------------------------
```

```
ORF1ab           301 RSVYPVASPNECNQMCLSTLMKCDHCGETSWQTGDFVKATCEFCGTENLTKEGATTCGYL
EPI_ISL_413575     1 ------------------------------------------------------------
EPI_ISL_413588     1 ------------------------------------------------------------
EPI_ISL_413589     1 ------------------------------------------------------------
EPI_ISL_424221     1 ------------------------------------------------------------
EPI_ISL_424233     1 ------------------------------------------------------------
EPI_ISL_424236     1 ------------------------------------------------------------
EPI_ISL_424238     1 ------------------------------------------------------------
EPI_ISL_424248     1 ------------------------------------------------------------
EPI_ISL_424254     1 ------------------------------------------------------------
EPI_ISL_425548     1 ------------------------------------------------------------
EPI_ISL_425581     1 ------------------------------------------------------------
EPI_ISL_426881     1 ------------------------------------------------------------
EPI_ISL_426883     1 ------------------------------------------------------------
EPI_ISL_426888     1 ------------------------------------------------------------
EPI_ISL_426900     1 ------------------------------------------------------------
EPI_ISL_426901     1 ------------------------------------------------------------
EPI_ISL_426903     1 ------------------------------------------------------------
EPI_ISL_426904     1 ------------------------------------------------------------
EPI_ISL_426907     1 ------------------------------------------------------------
EPI_ISL_426910     1 ------------------------------------------------------------
```


```
ORF1ab           301 RSVYPVASPNECNQMCLSTLMKCDHCGETSWQTGDFVKATCEFCGTENLTKEGATTCGYL
EPI_ISL_413575     1 ------------------------------------------------------------
EPI_ISL_413588     1 ------------------------------------------------------------
EPI_ISL_413589     1 ------------------------------------------------------------
EPI_ISL_424221     1 ------------------------------------------------------------
EPI_ISL_424233     1 ------------------------------------------------------------
EPI_ISL_424236     1 ------------------------------------------------------------
EPI_ISL_424238     1 ------------------------------------------------------------
EPI_ISL_424248     1 ------------------------------------------------------------
EPI_ISL_424254     1 ------------------------------------------------------------
EPI_ISL_425548     1 ------------------------------------------------------------
EPI_ISL_425581     1 ------------------------------------------------------------
EPI_ISL_426881     1 ------------------------------------------------------------
EPI_ISL_426883     1 ------------------------------------------------------------
EPI_ISL_426888     1 ------------------------------------------------------------
EPI_ISL_426900     1 ------------------------------------------------------------
EPI_ISL_426901     1 ------------------------------------------------------------
EPI_ISL_426903     1 ------------------------------------------------------------
EPI_ISL_426904     1 ------------------------------------------------------------
EPI_ISL_426907     1 ------------------------------------------------------------
EPI_ISL_426910     1 ------------------------------------------------------------
```

```
ORF1ab          361 PQNAVVKIYCPACHNSEVGPEHSLAEYHNESGLKTILRKGGRTIAFGGCVFSYVGCHNKC
EPI_ISL_413575    1 ------------------------------------------------------------
EPI_ISL_413588    1 ------------------------------------------------------------
EPI_ISL_413589    1 ------------------------------------------------------------
EPI_ISL_424221    1 ------------------------------------------------------------
EPI_ISL_424233    1 ------------------------------------------------------------
EPI_ISL_424236    1 ------------------------------------------------------------
EPI_ISL_424238    1 ------------------------------------------------------------
EPI_ISL_424248    1 ------------------------------------------------------------
EPI_ISL_424254    1 ------------------------------------------------------------
EPI_ISL_425548    1 ------------------------------------------------------------
EPI_ISL_425581    1 ------------------------------------------------------------
EPI_ISL_426881    1 ------------------------------------------------------------
EPI_ISL_426883    1 ------------------------------------------------------------
EPI_ISL_426888    1 ------------------------------------------------------------
EPI_ISL_426900    1 ------------------------------------------------------------
EPI_ISL_426901    1 ------------------------------------------------------------
EPI_ISL_426903    1 ------------------------------------------------------------
EPI_ISL_426904    1 ------------------------------------------------------------
EPI_ISL_426907    1 ------------------------------------------------------------
EPI_ISL_426910    1 ------------------------------------------------------------
```

```
ORF1ab          361 PQNAVVKIYCPACHNSEVGPEHSLAEYHNESGLKTILRKGGRTIAFGGCVFSYVGCHNKC
EPI_ISL_413575    1 ------------------------------------------------------------
EPI_ISL_413588    1 ------------------------------------------------------------
EPI_ISL_413589    1 ------------------------------------------------------------
EPI_ISL_424221    1 ------------------------------------------------------------
EPI_ISL_424233    1 ------------------------------------------------------------
EPI_ISL_424236    1 ------------------------------------------------------------
EPI_ISL_424238    1 ------------------------------------------------------------
EPI_ISL_424248    1 ------------------------------------------------------------
EPI_ISL_424254    1 ------------------------------------------------------------
EPI_ISL_425548    1 ------------------------------------------------------------
EPI_ISL_425581    1 ------------------------------------------------------------
EPI_ISL_426881    1 ------------------------------------------------------------
EPI_ISL_426883    1 ------------------------------------------------------------
EPI_ISL_426888    1 ------------------------------------------------------------
EPI_ISL_426900    1 ------------------------------------------------------------
EPI_ISL_426901    1 ------------------------------------------------------------
EPI_ISL_426903    1 ------------------------------------------------------------
EPI_ISL_426904    1 ------------------------------------------------------------
EPI_ISL_426907    1 ------------------------------------------------------------
EPI_ISL_426910    1 ------------------------------------------------------------
```

```
ORF1ab          421 AYWVPRASANIGCNHTGVVGEGSEGLNDNLLEILQKEKVNINIVGDFKLNEEIAIILASF
EPI_ISL_413575    1 ------------------------------------------------------------
EPI_ISL_413588    1 ------------------------------------------------------------
EPI_ISL_413589    1 ------------------------------------------------------------
EPI_ISL_424221    1 ------------------------------------------------------------
EPI_ISL_424233    1 ------------------------------------------------------------
EPI_ISL_424236    1 ------------------------------------------------------------
EPI_ISL_424238    1 ------------------------------------------------------------
EPI_ISL_424248    1 ------------------------------------------------------------
EPI_ISL_424254    1 ------------------------------------------------------------
EPI_ISL_425548    1 ------------------------------------------------------------
EPI_ISL_425581    1 ------------------------------------------------------------
EPI_ISL_426881    1 ------------------------------------------------------------
EPI_ISL_426883    1 ------------------------------------------------------------
EPI_ISL_426888    1 ------------------------------------------------------------
EPI_ISL_426900    1 ------------------------------------------------------------
EPI_ISL_426901    1 ------------------------------------------------------------
EPI_ISL_426903    1 ------------------------------------------------------------
EPI_ISL_426904    1 ------------------------------------------------------------
EPI_ISL_426907    1 ------------------------------------------------------------
EPI_ISL_426910    1 ------------------------------------------------------------
```

```
ORF1ab          421 AYWVPRASANIGCNHTGVVGEGSEGLNDNLLEILQKEKVNINIVGDFKLNEEIAIILASF
EPI_ISL_413575    1 ------------------------------------------------------------
EPI_ISL_413588    1 ------------------------------------------------------------
EPI_ISL_413589    1 ------------------------------------------------------------
EPI_ISL_424221    1 ------------------------------------------------------------
EPI_ISL_424233    1 ------------------------------------------------------------
EPI_ISL_424236    1 ------------------------------------------------------------
EPI_ISL_424238    1 ------------------------------------------------------------
EPI_ISL_424248    1 ------------------------------------------------------------
EPI_ISL_424254    1 ------------------------------------------------------------
EPI_ISL_425548    1 ------------------------------------------------------------
EPI_ISL_425581    1 ------------------------------------------------------------
EPI_ISL_426881    1 ------------------------------------------------------------
EPI_ISL_426883    1 ------------------------------------------------------------
EPI_ISL_426888    1 ------------------------------------------------------------
EPI_ISL_426900    1 ------------------------------------------------------------
EPI_ISL_426901    1 ------------------------------------------------------------
EPI_ISL_426903    1 ------------------------------------------------------------
EPI_ISL_426904    1 ------------------------------------------------------------
EPI_ISL_426907    1 ------------------------------------------------------------
EPI_ISL_426910    1 ------------------------------------------------------------
```

```
ORF1ab          481 SASTSAFVETVKGLDYKAFKQIVESCGNFKVTKGKAKKGAWNIGEQKSILSPLYAFASEA
EPI_ISL_413575    1 ------------------------------------------------------------
EPI_ISL_413588    1 ------------------------------------------------------------
EPI_ISL_413589    1 ------------------------------------------------------------
EPI_ISL_424221    1 ------------------------------------------------------------
EPI_ISL_424233    1 ------------------------------------------------------------
EPI_ISL_424236    1 ------------------------------------------------------------
EPI_ISL_424238    1 ------------------------------------------------------------
EPI_ISL_424248    1 ------------------------------------------------------------
EPI_ISL_424254    1 ------------------------------------------------------------
EPI_ISL_425548    1 ------------------------------------------------------------
EPI_ISL_425581    1 ------------------------------------------------------------
EPI_ISL_426881    1 ------------------------------------------------------------
EPI_ISL_426883    1 ------------------------------------------------------------
EPI_ISL_426888    1 ------------------------------------------------------------
EPI_ISL_426900    1 ------------------------------------------------------------
EPI_ISL_426901    1 ------------------------------------------------------------
EPI_ISL_426903    1 ------------------------------------------------------------
EPI_ISL_426904    1 ------------------------------------------------------------
EPI_ISL_426907    1 ------------------------------------------------------------
EPI_ISL_426910    1 ------------------------------------------------------------
ORF1ab          481 SASTSAFVETVKGLDYKAFKQIVESCGNFKVTKGKAKKGAWNIGEQKSILSPLYAFASEA
EPI_ISL_413575    1 ------------------------------------------------------------
EPI_ISL_413588    1 ------------------------------------------------------------
EPI_ISL_413589    1 ------------------------------------------------------------
EPI_ISL_424221    1 ------------------------------------------------------------
EPI_ISL_424233    1 ------------------------------------------------------------
EPI_ISL_424236    1 ------------------------------------------------------------
EPI_ISL_424238    1 ------------------------------------------------------------
EPI_ISL_424248    1 ------------------------------------------------------------
EPI_ISL_424254    1 ------------------------------------------------------------
EPI_ISL_425548    1 ------------------------------------------------------------
EPI_ISL_425581    1 ------------------------------------------------------------
EPI_ISL_426881    1 ------------------------------------------------------------
EPI_ISL_426883    1 ------------------------------------------------------------
EPI_ISL_426888    1 ------------------------------------------------------------
EPI_ISL_426900    1 ------------------------------------------------------------
EPI_ISL_426901    1 ------------------------------------------------------------
EPI_ISL_426903    1 ------------------------------------------------------------
EPI_ISL_426904    1 ------------------------------------------------------------
EPI_ISL_426907    1 ------------------------------------------------------------
EPI_ISL_426910    1 ------------------------------------------------------------
```

```
ORF1ab           541 ARVVRSIFSRTLETAQNSVRVLQKAAITILDGISQYSLRLIDAMMFTSDLATNNLVVMAY
EPI_ISL_413575     1 ------------------------------------------------------------
EPI_ISL_413588     1 ------------------------------------------------------------
EPI_ISL_413589     1 ------------------------------------------------------------
EPI_ISL_424221     1 ------------------------------------------------------------
EPI_ISL_424233     1 ------------------------------------------------------------
EPI_ISL_424236     1 ------------------------------------------------------------
EPI_ISL_424238     1 ------------------------------------------------------------
EPI_ISL_424248     1 ------------------------------------------------------------
EPI_ISL_424254     1 ------------------------------------------------------------
EPI_ISL_425548     1 ------------------------------------------------------------
EPI_ISL_425581     1 ------------------------------------------------------------
EPI_ISL_426881     1 ------------------------------------------------------------
EPI_ISL_426883     1 ------------------------------------------------------------
EPI_ISL_426888     1 ------------------------------------------------------------
EPI_ISL_426900     1 ------------------------------------------------------------
EPI_ISL_426901     1 ------------------------------------------------------------
EPI_ISL_426903     1 ------------------------------------------------------------
EPI_ISL_426904     1 ------------------------------------------------------------
EPI_ISL_426907     1 ------------------------------------------------------------
EPI_ISL_426910     1 ------------------------------------------------------------
ORF1ab           541 ARVVRSIFSRTLETAQNSVRVLQKAAITILDGISQYSLRLIDAMMFTSDLATNNLVVMAY
EPI_ISL_413575     1 ------------------------------------------------------------
EPI_ISL_413588     1 ------------------------------------------------------------
EPI_ISL_413589     1 ------------------------------------------------------------
EPI_ISL_424221     1 ------------------------------------------------------------
EPI_ISL_424233     1 ------------------------------------------------------------
EPI_ISL_424236     1 ------------------------------------------------------------
EPI_ISL_424238     1 ------------------------------------------------------------
EPI_ISL_424248     1 ------------------------------------------------------------
EPI_ISL_424254     1 ------------------------------------------------------------
EPI_ISL_425548     1 ------------------------------------------------------------
EPI_ISL_425581     1 ------------------------------------------------------------
EPI_ISL_426881     1 ------------------------------------------------------------
EPI_ISL_426883     1 ------------------------------------------------------------
EPI_ISL_426888     1 ------------------------------------------------------------
EPI_ISL_426900     1 ------------------------------------------------------------
EPI_ISL_426901     1 ------------------------------------------------------------
EPI_ISL_426903     1 ------------------------------------------------------------
EPI_ISL_426904     1 ------------------------------------------------------------
EPI_ISL_426907     1 ------------------------------------------------------------
EPI_ISL_426910     1 ------------------------------------------------------------
```

```
ORF1ab          601 ITGGVVQLTSQWLTNIFGTVYEKLKPVLDWLEEKFKEGVEFLRDGWEIVKFISTCACEIV
EPI_ISL_413575    1 ------------------------------------------------------------
EPI_ISL_413588    1 ------------------------------------------------------------
EPI_ISL_413589    1 ------------------------------------------------------------
EPI_ISL_424221    1 ------------------------------------------------------------
EPI_ISL_424233    1 ------------------------------------------------------------
EPI_ISL_424236    1 ------------------------------------------------------------
EPI_ISL_424238    1 ------------------------------------------------------------
EPI_ISL_424248    1 ------------------------------------------------------------
EPI_ISL_424254    1 ------------------------------------------------------------
EPI_ISL_425548    1 ------------------------------------------------------------
EPI_ISL_425581    1 ------------------------------------------------------------
EPI_ISL_426881    1 ------------------------------------------------------------
EPI_ISL_426883    1 ------------------------------------------------------------
EPI_ISL_426888    1 ------------------------------------------------------------
EPI_ISL_426900    1 ------------------------------------------------------------
EPI_ISL_426901    1 ------------------------------------------------------------
EPI_ISL_426903    1 ------------------------------------------------------------
EPI_ISL_426904    1 ------------------------------------------------------------
EPI_ISL_426907    1 ------------------------------------------------------------
EPI_ISL_426910    1 ------------------------------------------------------------
```

```
ORF1ab          601 ITGGVVQLTSQWLTNIFGTVYEKLKPVLDWLEEKFKEGVEFLRDGWEIVKFISTCACEIV
EPI_ISL_413575    1 ------------------------------------------------------------
EPI_ISL_413588    1 ------------------------------------------------------------
EPI_ISL_413589    1 ------------------------------------------------------------
EPI_ISL_424221    1 ------------------------------------------------------------
EPI_ISL_424233    1 ------------------------------------------------------------
EPI_ISL_424236    1 ------------------------------------------------------------
EPI_ISL_424238    1 ------------------------------------------------------------
EPI_ISL_424248    1 ------------------------------------------------------------
EPI_ISL_424254    1 ------------------------------------------------------------
EPI_ISL_425548    1 ------------------------------------------------------------
EPI_ISL_425581    1 ------------------------------------------------------------
EPI_ISL_426881    1 ------------------------------------------------------------
EPI_ISL_426883    1 ------------------------------------------------------------
EPI_ISL_426888    1 ------------------------------------------------------------
EPI_ISL_426900    1 ------------------------------------------------------------
EPI_ISL_426901    1 ------------------------------------------------------------
EPI_ISL_426903    1 ------------------------------------------------------------
EPI_ISL_426904    1 ------------------------------------------------------------
EPI_ISL_426907    1 ------------------------------------------------------------
EPI_ISL_426910    1 ------------------------------------------------------------
```

```
ORF1ab          661 GGQIVTCAKEIKESVQTFFKLVNKFLALCADSIIIGGAKLKALNLGETFVTHSKGLYRKC
EPI_ISL_413575    1 ------------------------------------------------------------
EPI_ISL_413588    1 ------------------------------------------------------------
EPI_ISL_413589    1 ------------------------------------------------------------
EPI_ISL_424221    1 ------------------------------------------------------------
EPI_ISL_424233    1 ------------------------------------------------------------
EPI_ISL_424236    1 ------------------------------------------------------------
EPI_ISL_424238    1 ------------------------------------------------------------
EPI_ISL_424248    1 ------------------------------------------------------------
EPI_ISL_424254    1 ------------------------------------------------------------
EPI_ISL_425548    1 ------------------------------------------------------------
EPI_ISL_425581    1 ------------------------------------------------------------
EPI_ISL_426881    1 ------------------------------------------------------------
EPI_ISL_426883    1 ------------------------------------------------------------
EPI_ISL_426888    1 ------------------------------------------------------------
EPI_ISL_426900    1 ------------------------------------------------------------
EPI_ISL_426901    1 ------------------------------------------------------------
EPI_ISL_426903    1 ------------------------------------------------------------
EPI_ISL_426904    1 ------------------------------------------------------------
EPI_ISL_426907    1 ------------------------------------------------------------
EPI_ISL_426910    1 ------------------------------------------------------------
```


```
ORF1ab          661 GGQIVTCAKEIKESVQTFFKLVNKFLALCADSIIIGGAKLKALNLGETFVTHSKGLYRKC
EPI_ISL_413575    1 ------------------------------------------------------------
EPI_ISL_413588    1 ------------------------------------------------------------
EPI_ISL_413589    1 ------------------------------------------------------------
EPI_ISL_424221    1 ------------------------------------------------------------
EPI_ISL_424233    1 ------------------------------------------------------------
EPI_ISL_424236    1 ------------------------------------------------------------
EPI_ISL_424238    1 ------------------------------------------------------------
EPI_ISL_424248    1 ------------------------------------------------------------
EPI_ISL_424254    1 ------------------------------------------------------------
EPI_ISL_425548    1 ------------------------------------------------------------
EPI_ISL_425581    1 ------------------------------------------------------------
EPI_ISL_426881    1 ------------------------------------------------------------
EPI_ISL_426883    1 ------------------------------------------------------------
EPI_ISL_426888    1 ------------------------------------------------------------
EPI_ISL_426900    1 ------------------------------------------------------------
EPI_ISL_426901    1 ------------------------------------------------------------
EPI_ISL_426903    1 ------------------------------------------------------------
EPI_ISL_426904    1 ------------------------------------------------------------
EPI_ISL_426907    1 ------------------------------------------------------------
EPI_ISL_426910    1 ------------------------------------------------------------
```

```
ORF1ab          721 VKSREETGLLMPLKAPKEIIFLEGETLPTEVLTEEVVLKTGDLQPLEQPTSEAVEAPLVG
EPI_ISL_413575    1 ------------------------------------------------------------
EPI_ISL_413588    1 ------------------------------------------------------------
EPI_ISL_413589    1 ------------------------------------------------------------
EPI_ISL_424221    1 ------------------------------------------------------------
EPI_ISL_424233    1 ------------------------------------------------------------
EPI_ISL_424236    1 ------------------------------------------------------------
EPI_ISL_424238    1 ------------------------------------------------------------
EPI_ISL_424248    1 ------------------------------------------------------------
EPI_ISL_424254    1 ------------------------------------------------------------
EPI_ISL_425548    1 ------------------------------------------------------------
EPI_ISL_425581    1 ------------------------------------------------------------
EPI_ISL_426881    1 ------------------------------------------------------------
EPI_ISL_426883    1 ------------------------------------------------------------
EPI_ISL_426888    1 ------------------------------------------------------------
EPI_ISL_426900    1 ------------------------------------------------------------
EPI_ISL_426901    1 ------------------------------------------------------------
EPI_ISL_426903    1 ------------------------------------------------------------
EPI_ISL_426904    1 ------------------------------------------------------------
EPI_ISL_426907    1 ------------------------------------------------------------
EPI_ISL_426910    1 ------------------------------------------------------------
```


```
ORF1ab          721 VKSREETGLLMPLKAPKEIIFLEGETLPTEVLTEEVVLKTGDLQPLEQPTSEAVEAPLVG
EPI_ISL_413575    1 ------------------------------------------------------------
EPI_ISL_413588    1 ------------------------------------------------------------
EPI_ISL_413589    1 ------------------------------------------------------------
EPI_ISL_424221    1 ------------------------------------------------------------
EPI_ISL_424233    1 ------------------------------------------------------------
EPI_ISL_424236    1 ------------------------------------------------------------
EPI_ISL_424238    1 ------------------------------------------------------------
EPI_ISL_424248    1 ------------------------------------------------------------
EPI_ISL_424254    1 ------------------------------------------------------------
EPI_ISL_425548    1 ------------------------------------------------------------
EPI_ISL_425581    1 ------------------------------------------------------------
EPI_ISL_426881    1 ------------------------------------------------------------
EPI_ISL_426883    1 ------------------------------------------------------------
EPI_ISL_426888    1 ------------------------------------------------------------
EPI_ISL_426900    1 ------------------------------------------------------------
EPI_ISL_426901    1 ------------------------------------------------------------
EPI_ISL_426903    1 ------------------------------------------------------------
EPI_ISL_426904    1 ------------------------------------------------------------
EPI_ISL_426907    1 ------------------------------------------------------------
EPI_ISL_426910    1 ------------------------------------------------------------
```

```
ORF1ab          781 TPVCINGLMLLEIKDTEKYCALAPNMMVTNNTFTLKGGAPTKVTFGDDTVIEVQGYKSVN
EPI_ISL_413575    1 --------------------------------------APTKVTFGDDTVIEVQGYKSVN
EPI_ISL_413588    1 --------------------------------------APTKVTFGDDTVIEVQGYKSVN
EPI_ISL_413589    1 --------------------------------------APTKVTFGDDTVIEVQGYKSVN
EPI_ISL_424221    1 --------------------------------------APTKVTFGDDTVIEVQGYKSVN
EPI_ISL_424233    1 --------------------------------------APTKVTFGDDTVIEVQGYKSVN
EPI_ISL_424236    1 --------------------------------------APTKVTFGDDTVIEVQGYKSVN
EPI_ISL_424238    1 --------------------------------------APTKVTFGDDTVIEVQGYKSVN
EPI_ISL_424248    1 --------------------------------------APTKVTFGDDTVIEVQGYKSVN
EPI_ISL_424254    1 --------------------------------------APTKVTFGDDTVIEVQGYKSVN
EPI_ISL_425548    1 --------------------------------------APTKVTFGDDTVIEVQGYKSVN
EPI_ISL_425581    1 --------------------------------------APTKVTFGDDTVIEVQGYKSVN
EPI_ISL_426881    1 --------------------------------------APTKVTFGDDTVIEVQGYKSVN
EPI_ISL_426883    1 --------------------------------------APTKVTFGDDTVIEVQGYKSVN
EPI_ISL_426888    1 --------------------------------------APTKVTFGDDTVIEVQGYKSVN
EPI_ISL_426900    1 --------------------------------------APTKVTFGDDTVIEVQGYKSVN
EPI_ISL_426901    1 --------------------------------------APTKVTFGDDTVIEVQGYKSVN
EPI_ISL_426903    1 --------------------------------------APTKVTFGDDTVIEVQGYKSVN
EPI_ISL_426904    1 --------------------------------------APTKVTFGDDTVIEVQGYKSVN
EPI_ISL_426907    1 --------------------------------------APTKVTFGDDTVIEVQGYKSVN
EPI_ISL_426910    1 --------------------------------------APTKVTFGDDTVIEVQGYKSVN
```


```
ORF1ab          781 TPVCINGLMLLEIKDTEKYCALAPNMMVTNNTFTLKGGAPTKVTFGDDTVIEVQGYKSVN
EPI_ISL_413575    1 --------------------------------------APTKVTFGDDTVIEVQGYKSVN
EPI_ISL_413588    1 --------------------------------------APTKVTFGDDTVIEVQGYKSVN
EPI_ISL_413589    1 --------------------------------------APTKVTFGDDTVIEVQGYKSVN
EPI_ISL_424221    1 --------------------------------------APTKVTFGDDTVIEVQGYKSVN
EPI_ISL_424233    1 --------------------------------------APTKVTFGDDTVIEVQGYKSVN
EPI_ISL_424236    1 --------------------------------------APTKVTFGDDTVIEVQGYKSVN
EPI_ISL_424238    1 --------------------------------------APTKVTFGDDTVIEVQGYKSVN
EPI_ISL_424248    1 --------------------------------------APTKVTFGDDTVIEVQGYKSVN
EPI_ISL_424254    1 --------------------------------------APTKVTFGDDTVIEVQGYKSVN
EPI_ISL_425548    1 --------------------------------------APTKVTFGDDTVIEVQGYKSVN
EPI_ISL_425581    1 --------------------------------------APTKVTFGDDTVIEVQGYKSVN
EPI_ISL_426881    1 --------------------------------------APTKVTFGDDTVIEVQGYKSVN
EPI_ISL_426883    1 --------------------------------------APTKVTFGDDTVIEVQGYKSVN
EPI_ISL_426888    1 --------------------------------------APTKVTFGDDTVIEVQGYKSVN
EPI_ISL_426900    1 --------------------------------------APTKVTFGDDTVIEVQGYKSVN
EPI_ISL_426901    1 --------------------------------------APTKVTFGDDTVIEVQGYKSVN
EPI_ISL_426903    1 --------------------------------------APTKVTFGDDTVIEVQGYKSVN
EPI_ISL_426904    1 --------------------------------------APTKVTFGDDTVIEVQGYKSVN
EPI_ISL_426907    1 --------------------------------------APTKVTFGDDTVIEVQGYKSVN
EPI_ISL_426910    1 --------------------------------------APTKVTFGDDTVIEVQGYKSVN
```

ORF1ab	841	ITFELDERIDKVLNEKCSAYTVELGTEVNEFACVVADAVIKTLQPVSELLTPLGIDLDEW
EPI_ISL_413575	23	ITFELDERIDKVLNEKCSAYTVELGTEVNEFACVVADAVIKTLQPVSELLTPLGIDLDEW
EPI_ISL_413588	23	ITFELDERIDKVLNEKCSAYTVELGTEVNEFACVVADAVIKTLQPVSELLTPLGIDLDEW
EPI_ISL_413589	23	ITFELDERIDKVLNEKCSAYTVELGTEVNEFACVVADAVIKTLQPVSELLTPLGIDLDEW
EPI_ISL_424221	23	ITFELDERIDKVLNEKCSAYTVELGTEVNEFACVVADAVIKTLQPVSELLTPLGIDLDEW
EPI_ISL_424233	23	ITFELDERIDKVLNEKCSAYTVELGTEVNEFACVVADAVIKTLQPVSELLTPLGIDLDEW
EPI_ISL_424236	23	ITFELDERIDKVLNEKCSAYTVELGTEVNEFACVVADAVIKTLQPVSELLTPLGIDLDEW
EPI_ISL_424238	23	ITFELDERIDKVLNEKCSAYTVELGTEVNEFACVVADAVIKTLQPVSELLTPLGIDLDEW
EPI_ISL_424248	23	ITFELDERIDKVLNEKCSAYTVELGTEVNEFACVVADAVIKTLQPVSELLTPLGIDLDEW
EPI_ISL_424254	23	ITFELDERIDKVLNEKCSAYTVELGTEVNEFACVVADAVIKTLQPVSELLTPLGIDLDEW
EPI_ISL_425548	23	ITFELDERIDKVLNEKCSAYTVELGTEVNEFACVVADAVIKTLQPVSELLTPLGIDLDEW
EPI_ISL_425581	23	ITFELDERIDKVLNEKCSAYTVELGTEVNEFACVVADAVIKTLQPVSELLTPLGIDLDEW
EPI_ISL_426881	23	ITFELDERIDKVLNEKCSAYTVELGTEVNEFACVVADAVIKTLQPVSELLTPLGIDLDEW
EPI_ISL_426883	23	ITFELDERIDKVLNEKCSAYTVELGTEVNEFACVVADAVIKTLQPVSELLTPLGIDLDEW
EPI_ISL_426888	23	ITFELDERIDKVLNEKCSAYTVELGTEVNEFACVVADAVIKTLQPVSELLTPLGIDLDEW
EPI_ISL_426900	23	ITFELDERIDKVLNEKCSAYTVELGTEVNEFACVVADAVIKTLQPVSELLTPLGIDLDEW
EPI_ISL_426901	23	ITFELDERIDKVLNEKCSAYTVELGTEVNEFACVVADAVIKTLQPVSELLTPLGIDLDEW
EPI_ISL_426903	23	ITFELDERIDKVLNEKCSAYTVELGTEVNEFACVVADAVIKTLQPVSELLTPLGIDLDEW
EPI_ISL_426904	23	ITFELDERIDKVLNEKCSAYTVELGTEVNEFACVVADAVIKTLQPVSELLTPLGIDLDEW
EPI_ISL_426907	23	ITFELDERIDKVLNEKCSAYTVELGTEVNEFACVVADAVIKTLQPVSELLTPLGIDLDEW
EPI_ISL_426910	23	ITFELDERIDKVLNEKCSAYTVELGTEVNEFACVVADAVIKTLQPVSELLTPLGIDLDEW
ORF1ab	841	ITFELDERIDKVLNEKCSAYTVELGTEVNEFACVVADAVIKTLQPVSELLTPLGIDLDEW
EPI_ISL_413575	23	ITFELDERIDKVLNEKCSAYTVELGTEVNEFACVVADAVIKTLQPVSELLTPLGIDLDEW
EPI_ISL_413588	23	ITFELDERIDKVLNEKCSAYTVELGTEVNEFACVVADAVIKTLQPVSELLTPLGIDLDEW
EPI_ISL_413589	23	ITFELDERIDKVLNEKCSAYTVELGTEVNEFACVVADAVIKTLQPVSELLTPLGIDLDEW
EPI_ISL_424221	23	ITFELDERIDKVLNEKCSAYTVELGTEVNEFACVVADAVIKTLQPVSELLTPLGIDLDEW
EPI_ISL_424233	23	ITFELDERIDKVLNEKCSAYTVELGTEVNEFACVVADAVIKTLQPVSELLTPLGIDLDEW
EPI_ISL_424236	23	ITFELDERIDKVLNEKCSAYTVELGTEVNEFACVVADAVIKTLQPVSELLTPLGIDLDEW
EPI_ISL_424238	23	ITFELDERIDKVLNEKCSAYTVELGTEVNEFACVVADAVIKTLQPVSELLTPLGIDLDEW
EPI_ISL_424248	23	ITFELDERIDKVLNEKCSAYTVELGTEVNEFACVVADAVIKTLQPVSELLTPLGIDLDEW
EPI_ISL_424254	23	ITFELDERIDKVLNEKCSAYTVELGTEVNEFACVVADAVIKTLQPVSELLTPLGIDLDEW
EPI_ISL_425548	23	ITFELDERIDKVLNEKCSAYTVELGTEVNEFACVVADAVIKTLQPVSELLTPLGIDLDEW
EPI_ISL_425581	23	ITFELDERIDKVLNEKCSAYTVELGTEVNEFACVVADAVIKTLQPVSELLTPLGIDLDEW
EPI_ISL_426881	23	ITFELDERIDKVLNEKCSAYTVELGTEVNEFACVVADAVIKTLQPVSELLTPLGIDLDEW
EPI_ISL_426883	23	ITFELDERIDKVLNEKCSAYTVELGTEVNEFACVVADAVIKTLQPVSELLTPLGIDLDEW
EPI_ISL_426888	23	ITFELDERIDKVLNEKCSAYTVELGTEVNEFACVVADAVIKTLQPVSELLTPLGIDLDEW
EPI_ISL_426900	23	ITFELDERIDKVLNEKCSAYTVELGTEVNEFACVVADAVIKTLQPVSELLTPLGIDLDEW
EPI_ISL_426901	23	ITFELDERIDKVLNEKCSAYTVELGTEVNEFACVVADAVIKTLQPVSELLTPLGIDLDEW
EPI_ISL_426903	23	ITFELDERIDKVLNEKCSAYTVELGTEVNEFACVVADAVIKTLQPVSELLTPLGIDLDEW
EPI_ISL_426904	23	ITFELDERIDKVLNEKCSAYTVELGTEVNEFACVVADAVIKTLQPVSELLTPLGIDLDEW
EPI_ISL_426907	23	ITFELDERIDKVLNEKCSAYTVELGTEVNEFACVVADAVIKTLQPVSELLTPLGIDLDEW
EPI_ISL_426910	23	ITFELDERIDKVLNEKCSAYTVELGTEVNEFACVVADAVIKTLQPVSELLTPLGIDLDEW

ORF1ab	901	SMATYYLFDESGEFKLASHMYCSFYPPDEDEEEGDCEEEEFEPSTQYEYGTEDDYQGKPL
EPI_ISL_413575	83	SMATYYLFDESGEFKLASHMYCSFYPPDEDEEEGDCEEEEFEPSTQYEYGTEDDYQGKPL
EPI_ISL_413588	83	SMATYYLFDESGEFKLASHMYCSFYPPDEDEEEGDCEEEEFEPSTQYEYGTEDDYQGKPL
EPI_ISL_413589	83	SMATYYLFDESGEFKLASHMYCSFYPPDEDEEEGDCEEEEFEPSTQYEYGTEDDYQGKPL
EPI_ISL_424221	83	SMATYYLFDESGEFKLASHMYCSFYPPDEDEEEGDCEEEEFEPSTQYEYGTEDDYQGKPL
EPI_ISL_424233	83	SMATYYLFDESGEFKLASHMYCSFYPPDEDEEEGDCEEEEFEPSTQYEYGTEDDYQGKPL
EPI_ISL_424236	83	SMATYYLFDESGEFKLASHMYCSFYPPDEDEEEGDCEEEEFEPSTQYEYGTEDDYQGKPL
EPI_ISL_424238	83	SMATYYLFDESGEFKLASHMYCSFYPPDEDEEEGDCEEEEFEPSTQYEYGTEDDYQGKPL
EPI_ISL_424248	83	SMATYYLFDESGEFKLASHMYCSFYPPDEDEEEGDCEEEEFEPSTQYEYGTEDDYQGKPL
EPI_ISL_424254	83	SMATYYLFDESGEFKLASHMYCSFYPPDEDEEEGDCEEEEFEPSTQYEYGTEDDYQGKPL
EPI_ISL_425548	83	SMATYYLFDESGEFKLASHMYCSFYPPDEDEEEGDCEEEEFEPSTQYEYGTEDDYQGKPL
EPI_ISL_425581	83	SMATYYLFDESGEFKLASHMYCSFYPPDEDEEEGDCEEEEFEPSTQYEYGTEDDYQGKPL
EPI_ISL_426881	83	SMATYYLFDESGEFKLASHMYCSFYPPDEDEEEGDCEEEEFEPSTQYEYGTEDDYQGKPL
EPI_ISL_426883	83	SMATYYLFDESGEFKLASHMYCSFYPPDEDEEEGDCEEEEFEPSTQYEYGTEDDYQGKPL
EPI_ISL_426888	83	SMATYYLFDESGEFKLASHMYCSFYPPDEDEEEGDCEEEEFEPSTQYEYGTEDDYQGKPL
EPI_ISL_426900	83	SMATYYLFDESGEFKLASHMYCSFYPPDEDEEEGDCEEEEFEPSTQYEYGTEDDYQGKPL
EPI_ISL_426901	83	SMATYYLFDESGEFKLASHMYCSFYPPDEDEEEGDCEEEEFEPSTQYEYGTEDDYQGKPL
EPI_ISL_426903	83	SMATYYLFDESGEFKLASHMYCSFYPPDEDEEEGDCEEEEFEPSTQYEYGTEDDYQGKPL
EPI_ISL_426904	83	SMATYYLFDESGEFKLASHMYCSFYPPDEDEEEGDCEEEEFEPSTQYEYGTEDDYQGKPL
EPI_ISL_426907	83	SMATYYLFDESGEFKLASHMYCSFYPPDEDEEEGDCEEEEFEPSTQYEYGTEDDYQGKPL
EPI_ISL_426910	83	SMATYYLFDESGEFKLASHMYCSFYPPDEDEEEGDCEEEEFEPSTQYEYGTEDDYQGKPL
ORF1ab	901	SMATYYLFDESGEFKLASHMYCSFYPPDEDEEEGDCEEEEFEPSTQYEYGTEDDYQGKPL
EPI_ISL_413575	83	SMATYYLFDESGEFKLASHMYCSFYPPDEDEEEGDCEEEEFEPSTQYEYGTEDDYQGKPL
EPI_ISL_413588	83	SMATYYLFDESGEFKLASHMYCSFYPPDEDEEEGDCEEEEFEPSTQYEYGTEDDYQGKPL
EPI_ISL_413589	83	SMATYYLFDESGEFKLASHMYCSFYPPDEDEEEGDCEEEEFEPSTQYEYGTEDDYQGKPL
EPI_ISL_424221	83	SMATYYLFDESGEFKLASHMYCSFYPPDEDEEEGDCEEEEFEPSTQYEYGTEDDYQGKPL
EPI_ISL_424233	83	SMATYYLFDESGEFKLASHMYCSFYPPDEDEEEGDCEEEEFEPSTQYEYGTEDDYQGKPL
EPI_ISL_424236	83	SMATYYLFDESGEFKLASHMYCSFYPPDEDEEEGDCEEEEFEPSTQYEYGTEDDYQGKPL
EPI_ISL_424238	83	SMATYYLFDESGEFKLASHMYCSFYPPDEDEEEGDCEEEEFEPSTQYEYGTEDDYQGKPL
EPI_ISL_424248	83	SMATYYLFDESGEFKLASHMYCSFYPPDEDEEEGDCEEEEFEPSTQYEYGTEDDYQGKPL
EPI_ISL_424254	83	SMATYYLFDESGEFKLASHMYCSFYPPDEDEEEGDCEEEEFEPSTQYEYGTEDDYQGKPL
EPI_ISL_425548	83	SMATYYLFDESGEFKLASHMYCSFYPPDEDEEEGDCEEEEFEPSTQYEYGTEDDYQGKPL
EPI_ISL_425581	83	SMATYYLFDESGEFKLASHMYCSFYPPDEDEEEGDCEEEEFEPSTQYEYGTEDDYQGKPL
EPI_ISL_426881	83	SMATYYLFDESGEFKLASHMYCSFYPPDEDEEEGDCEEEEFEPSTQYEYGTEDDYQGKPL
EPI_ISL_426883	83	SMATYYLFDESGEFKLASHMYCSFYPPDEDEEEGDCEEEEFEPSTQYEYGTEDDYQGKPL
EPI_ISL_426888	83	SMATYYLFDESGEFKLASHMYCSFYPPDEDEEEGDCEEEEFEPSTQYEYGTEDDYQGKPL
EPI_ISL_426900	83	SMATYYLFDESGEFKLASHMYCSFYPPDEDEEEGDCEEEEFEPSTQYEYGTEDDYQGKPL
EPI_ISL_426901	83	SMATYYLFDESGEFKLASHMYCSFYPPDEDEEEGDCEEEEFEPSTQYEYGTEDDYQGKPL
EPI_ISL_426903	83	SMATYYLFDESGEFKLASHMYCSFYPPDEDEEEGDCEEEEFEPSTQYEYGTEDDYQGKPL
EPI_ISL_426904	83	SMATYYLFDESGEFKLASHMYCSFYPPDEDEEEGDCEEEEFEPSTQYEYGTEDDYQGKPL
EPI_ISL_426907	83	SMATYYLFDESGEFKLASHMYCSFYPPDEDEEEGDCEEEEFEPSTQYEYGTEDDYQGKPL
EPI_ISL_426910	83	SMATYYLFDESGEFKLASHMYCSFYPPDEDEEEGDCEEEEFEPSTQYEYGTEDDYQGKPL

```
ORF1ab          961 EFGATSAALQPEEEQEEDWLDDDSQQTVGQQDGSEDNQTTTIQTIVEVQPQLEMELTPVV
EPI_ISL_413575  143 EFGATSAALQHEEEQEEDWLDDDSQQTVGQQDGSEDNQTTTIQTIVEVQPQLEMELTPVV
EPI_ISL_413588  143 EFGATSAALQHEEEQEEDWLDDDSQQTVGQQDGSEDNQTTTIQTIVEVQPQLEMELTPVV
EPI_ISL_413589  143 EFGATSAALQPEEEQEEDWLDDDSQQTVGQQDGSEDNQTTTIQTIVEVQPQLEMELTPVV
EPI_ISL_424221  143 EFGATSAALQPEEEQEEDWLDDDSQQTVGQQDGSEDNQTTTIQTIVEVQPQLEMELTPVV
EPI_ISL_424233  143 EFGATSAALQPEEEQEEDWLDDDSQQTVGQQDGSEDNQTTTIQTIVEVQPQLEMELTPVV
EPI_ISL_424236  143 EFGATSAALQPEEEQEEDWLDDDSQQTVGQQDGSEDNQTTTIQTIVEVQPQLEMELTPVV
EPI_ISL_424238  143 EFGATSAALQPEEEQEEDWLDDDSQQTVGQQDGSEDNQTTTIQTIVEVQPQLEMELTPVV
EPI_ISL_424248  143 EFGATSAALQPEEEQEEDWLDDDSQQTVGQQDGSEDNQTTTIQTIVEVQPQLEMELTPVV
EPI_ISL_424254  143 EFGATSAALQPEEEQEEDWLDDDSQQTVGQQDGSEDNQTTTIQTIVEVQPQLEMELTPVV
EPI_ISL_425548  143 EFGATSAALQPEEEQEEDWLDDDSQQTVGQQDGSEDNQTTTIQTIVEVQPQLEMELTPVV
EPI_ISL_425581  143 EFGATSAALQPEEEQEEDWLDDDSQQTVGQQDGSEDNQTTTIQTIVEVQPQLEMELTPVV
EPI_ISL_426881  143 EFGATSAALQPEEEQEEDWLDDDSQQTVGQQDGSEDNQTTTIQTIVEVQPQLEMELTPVV
EPI_ISL_426883  143 EFGATSAALQPEEEQEEDWLDDDSQQTVGQQDGSEDNQTTTIQTIVEVQPQLEMELTPVV
EPI_ISL_426888  143 EFGATSAALQPEEEQEEDWLDDDSQQTVGQQDGSEDNQTTTIQTIVEVQPQLEMELTPVV
EPI_ISL_426900  143 EFGATSAALQPEEEQEEDWLDDDSQQTVGQQDGSEDNQTTTIQTIVEVQPQLEMELTPVV
EPI_ISL_426901  143 EFGATSAALQPEEEQEEDWLDDDSQQTVGQQDGSEDNQTTTIQTIVEVQPQLEMELTPVV
EPI_ISL_426903  143 EFGATSAALQPEEEQEEDWLDDDSQQTVGQQDGSEDNQTTTIQTIVEVQPQLEMELTPVV
EPI_ISL_426904  143 EFGATSAALQPEEEQEEDWLDDDSQQTVGQQDGSEDNQTTTIQTIVEVQPQLEMELTPVV
EPI_ISL_426907  143 EFGATSAALQPEEEQEEDWLDDDSQQTVGQQDGSEDNQTTTIQTIVEVQPQLEMELTPVV
EPI_ISL_426910  143 EFGATSAALQPEEEQEEDWLDDDSQQTVGQQDGSEDNQTTTIQTIVEVQPQLEMELTPVV
```


```
ORF1ab          961 EFGATSAALQPEEEQEEDWLDDDSQQTVGQQDGSEDNQTTTIQTIVEVQPQLEMELTPVV
EPI_ISL_413575  143 EFGATSAALQHEEEQEEDWLDDDSQQTVGQQDGSEDNQTTTIQTIVEVQPQLEMELTPVV
EPI_ISL_413588  143 EFGATSAALQHEEEQEEDWLDDDSQQTVGQQDGSEDNQTTTIQTIVEVQPQLEMELTPVV
EPI_ISL_413589  143 EFGATSAALQPEEEQEEDWLDDDSQQTVGQQDGSEDNQTTTIQTIVEVQPQLEMELTPVV
EPI_ISL_424221  143 EFGATSAALQPEEEQEEDWLDDDSQQTVGQQDGSEDNQTTTIQTIVEVQPQLEMELTPVV
EPI_ISL_424233  143 EFGATSAALQPEEEQEEDWLDDDSQQTVGQQDGSEDNQTTTIQTIVEVQPQLEMELTPVV
EPI_ISL_424236  143 EFGATSAALQPEEEQEEDWLDDDSQQTVGQQDGSEDNQTTTIQTIVEVQPQLEMELTPVV
EPI_ISL_424238  143 EFGATSAALQPEEEQEEDWLDDDSQQTVGQQDGSEDNQTTTIQTIVEVQPQLEMELTPVV
EPI_ISL_424248  143 EFGATSAALQPEEEQEEDWLDDDSQQTVGQQDGSEDNQTTTIQTIVEVQPQLEMELTPVV
EPI_ISL_424254  143 EFGATSAALQPEEEQEEDWLDDDSQQTVGQQDGSEDNQTTTIQTIVEVQPQLEMELTPVV
EPI_ISL_425548  143 EFGATSAALQPEEEQEEDWLDDDSQQTVGQQDGSEDNQTTTIQTIVEVQPQLEMELTPVV
EPI_ISL_425581  143 EFGATSAALQPEEEQEEDWLDDDSQQTVGQQDGSEDNQTTTIQTIVEVQPQLEMELTPVV
EPI_ISL_426881  143 EFGATSAALQPEEEQEEDWLDDDSQQTVGQQDGSEDNQTTTIQTIVEVQPQLEMELTPVV
EPI_ISL_426883  143 EFGATSAALQPEEEQEEDWLDDDSQQTVGQQDGSEDNQTTTIQTIVEVQPQLEMELTPVV
EPI_ISL_426888  143 EFGATSAALQPEEEQEEDWLDDDSQQTVGQQDGSEDNQTTTIQTIVEVQPQLEMELTPVV
EPI_ISL_426900  143 EFGATSAALQPEEEQEEDWLDDDSQQTVGQQDGSEDNQTTTIQTIVEVQPQLEMELTPVV
EPI_ISL_426901  143 EFGATSAALQPEEEQEEDWLDDDSQQTVGQQDGSEDNQTTTIQTIVEVQPQLEMELTPVV
EPI_ISL_426903  143 EFGATSAALQPEEEQEEDWLDDDSQQTVGQQDGSEDNQTTTIQTIVEVQPQLEMELTPVV
EPI_ISL_426904  143 EFGATSAALQPEEEQEEDWLDDDSQQTVGQQDGSEDNQTTTIQTIVEVQPQLEMELTPVV
EPI_ISL_426907  143 EFGATSAALQPEEEQEEDWLDDDSQQTVGQQDGSEDNQTTTIQTIVEVQPQLEMELTPVV
EPI_ISL_426910  143 EFGATSAALQPEEEQEEDWLDDDSQQTVGQQDGSEDNQTTTIQTIVEVQPQLEMELTPVV
```

```
ORF1ab          1021 QTIEVNSFSGYLKLTDNVYIKNADIVEEAKKVKPTVVVNAANVYLKHGGGVAGALNKATN
EPI_ISL_413575   203 QTIEVNSFSGYLKLTDNVYIKNADIVEEAKKVKPTVVVNAANVYLKHGGGVAGALNKATN
EPI_ISL_413588   203 QTIEVNSFSGYLKLTDNVYIKNADIVEEAKKVKPTVVVNAANVYLKHGGGVAGALNKATN
EPI_ISL_413589   203 QTIEVNSFSGYLKLTDNVYIKNADIVEEAKKVKPTVVVNAANVYLKHGGGVAGALNKATN
EPI_ISL_424221   203 QTIEVNSFSGYLKLTDNVYIKNADIVEEAKKVKPTVVVNAANVYLKHGGGVAGALNKATN
EPI_ISL_424233   203 QTIEVNSFSGYLKLTDNVYIKNADIVEEAKKVKPTVVVNAANVYLKHGGGVAGALNKATN
EPI_ISL_424236   203 QTIEVNSFSGYLKLTDNVYIKNADIVEEAKKVKPTVVVNAANVYLKHGGGVAGALNKATN
EPI_ISL_424238   203 QTIEVNSFSGYLKLTDNVYIKNADIVEEAKKVKPTVVVNAANVYLKHGGGVAGALNKATN
EPI_ISL_424248   203 QTIEVNSFSGYLKLTDNVYIKNADIVEEAKKVKPTVVVNAANVYLKHGGGVAGALNKATN
EPI_ISL_424254   203 QTIEVNSFSGYLKLTDNVYIKNADIVEEAKKVKPTVVVNAANVYLKHGGGVAGALNKATN
EPI_ISL_425548   203 QTIEVNSFSGYLKLTDNVYIKNADIVEEAKKVKPTVVVNAANVYLKHGGGVAGALNKATN
EPI_ISL_425581   203 QTIEVNSFSGYLKLTDNVYIKNADIVEEAKKVKPTVVVNAANVYLKHGGGVAGALNKATN
EPI_ISL_426881   203 QTIEVNSFSGYLKLTDNVYIKNADIVEEAKKVKPTVVVNAANVYLKHGGGVAGALNKATN
EPI_ISL_426883   203 QTIEVNSFSGYLKLTDNVYIKNADIVEEAKKVKPTVVVNAANVYLKHGGGVAGALNKATN
EPI_ISL_426888   203 QTIEVNSFSGYLKLTDNVYIKNADIVEEAKKVKPTVVVNAANVYLKHGGGVAGALNKATN
EPI_ISL_426900   203 QTIEVNSFSGYLKLTDNVYIKNADIVEEAKKVKPTVVVNAANVYLKHGGGVAGALNKATN
EPI_ISL_426901   203 QTIEVNSFSGYLKLTDNVYIKNADIVEEAKKVKPTVVVNAANVYLKHGGGVAGALNKATN
EPI_ISL_426903   203 QTIEVNSFSGYLKLTDNVYIKNADIVEEAKKVKPTVVVNAANVYLKHGGGVAGALNKATN
EPI_ISL_426904   203 QTIEVNSFSGYLKLTDNVYIKNADIVEEAKKVKPTVVVNAANVYLKHGGGVAGALNKATN
EPI_ISL_426907   203 QTIEVNSFSGYLKLTDNVYIKNADIVEEAKKVKPTVVVNAANVYLKHGGGVAGALNKATN
EPI_ISL_426910   203 QTIEVNSFSGYLKLTDNVYIKNADIVEEAKKVKPTVVVNAANVYLKHGGGVAGALNKATN
ORF1ab          1021 QTIEVNSFSGYLKLTDNVYIKNADIVEEAKKVKPTVVVNAANVYLKHGGGVAGALNKATN
EPI_ISL_413575   203 QTIEVNSFSGYLKLTDNVYIKNADIVEEAKKVKPTVVVNAANVYLKHGGGVAGALNKATN
EPI_ISL_413588   203 QTIEVNSFSGYLKLTDNVYIKNADIVEEAKKVKPTVVVNAANVYLKHGGGVAGALNKATN
EPI_ISL_413589   203 QTIEVNSFSGYLKLTDNVYIKNADIVEEAKKVKPTVVVNAANVYLKHGGGVAGALNKATN
EPI_ISL_424221   203 QTIEVNSFSGYLKLTDNVYIKNADIVEEAKKVKPTVVVNAANVYLKHGGGVAGALNKATN
EPI_ISL_424233   203 QTIEVNSFSGYLKLTDNVYIKNADIVEEAKKVKPTVVVNAANVYLKHGGGVAGALNKATN
EPI_ISL_424236   203 QTIEVNSFSGYLKLTDNVYIKNADIVEEAKKVKPTVVVNAANVYLKHGGGVAGALNKATN
EPI_ISL_424238   203 QTIEVNSFSGYLKLTDNVYIKNADIVEEAKKVKPTVVVNAANVYLKHGGGVAGALNKATN
EPI_ISL_424248   203 QTIEVNSFSGYLKLTDNVYIKNADIVEEAKKVKPTVVVNAANVYLKHGGGVAGALNKATN
EPI_ISL_424254   203 QTIEVNSFSGYLKLTDNVYIKNADIVEEAKKVKPTVVVNAANVYLKHGGGVAGALNKATN
EPI_ISL_425548   203 QTIEVNSFSGYLKLTDNVYIKNADIVEEAKKVKPTVVVNAANVYLKHGGGVAGALNKATN
EPI_ISL_425581   203 QTIEVNSFSGYLKLTDNVYIKNADIVEEAKKVKPTVVVNAANVYLKHGGGVAGALNKATN
EPI_ISL_426881   203 QTIEVNSFSGYLKLTDNVYIKNADIVEEAKKVKPTVVVNAANVYLKHGGGVAGALNKATN
EPI_ISL_426883   203 QTIEVNSFSGYLKLTDNVYIKNADIVEEAKKVKPTVVVNAANVYLKHGGGVAGALNKATN
EPI_ISL_426888   203 QTIEVNSFSGYLKLTDNVYIKNADIVEEAKKVKPTVVVNAANVYLKHGGGVAGALNKATN
EPI_ISL_426900   203 QTIEVNSFSGYLKLTDNVYIKNADIVEEAKKVKPTVVVNAANVYLKHGGGVAGALNKATN
EPI_ISL_426901   203 QTIEVNSFSGYLKLTDNVYIKNADIVEEAKKVKPTVVVNAANVYLKHGGGVAGALNKATN
EPI_ISL_426903   203 QTIEVNSFSGYLKLTDNVYIKNADIVEEAKKVKPTVVVNAANVYLKHGGGVAGALNKATN
EPI_ISL_426904   203 QTIEVNSFSGYLKLTDNVYIKNADIVEEAKKVKPTVVVNAANVYLKHGGGVAGALNKATN
EPI_ISL_426907   203 QTIEVNSFSGYLKLTDNVYIKNADIVEEAKKVKPTVVVNAANVYLKHGGGVAGALNKATN
EPI_ISL_426910   203 QTIEVNSFSGYLKLTDNVYIKNADIVEEAKKVKPTVVVNAANVYLKHGGGVAGALNKATN
```

```
ORF1ab          1081 NAMQVESDDYIATNGPLKVGGSCVLSGHNLAKHCLHVVGPNVNKGEDIQLLKSAYENFNQ
EPI_ISL_413575   263 NAMQVESDDYIATNGPLKVGGSCVLSGHNLAKHCLHVVGPNVNKGEDIQLLKSAYENFNQ
EPI_ISL_413588   263 NAMQVESDDYIATNGPLKVGGSCVLSGHNLAKHCLHVVGPNVNKGEDIQLLKSAYENFNQ
EPI_ISL_413589   263 NAMQVESDDYIATNGPLKVGGSCVLSGHNLAKHCLHVVGPNVNKGEDIQLLKSAYENFNQ
EPI_ISL_424221   263 NAMQVESDDYIATNGPLKVGGSCVLSGHNLAKHCLHVVGPNVNKGEDIQLLKSAYENFNQ
EPI_ISL_424233   263 NAMQVESDDYIATNGPLKVGGSCVLSGHNLAKHCLHVVGPNVNKGEDIQLLKSAYENFNQ
EPI_ISL_424236   263 NAMQVESDDYIATNGPLKVGGSCVLSGHNLAKHCLHVVGPNVNKGEDIQLLKSAYENFNQ
EPI_ISL_424238   263 NAMQVESDDYIATNGPLKVGGSCVLSGHNLAKHCLHVVGPNVNKGEDIQLLKSAYENFNQ
EPI_ISL_424248   263 NAMQVESDDYIATNGPLKVGGSCVLSGHNLAKHCLHVVGPNVNKGEDIQLLKSAYENFNQ
EPI_ISL_424254   263 NAMQVESDDYIATNGPLKVGGSCVLSGHNLAKHCLHVVGPNVNKGEDIQLLKSAYENFNQ
EPI_ISL_425548   263 NAMQVESDDYIATNGPLKVGGSCVLSGHNLAKHCLHVVGPNVNKGEDIQLLKSAYENFNQ
EPI_ISL_425581   263 NAMQVESDDYIATNGPLKVGGSCVLSGHNLAKHCLHVVGPNVNKGEDIQLLKSAYENFNQ
EPI_ISL_426881   263 NAMQVESDDYIATNGPLKVGGSCVLSGHNLAKHCLHVVGPNVNKGEDIQLLKSAYENFNQ
EPI_ISL_426883   263 NAMQVESDDYIATNGPLKVGGSCVLSGHNLAKHCLHVVGPNVNKGEDIQLLKSAYENFNQ
EPI_ISL_426888   263 NAMQVESDDYIATNGPLKVGGSCVLSGHNLAKHCLHVVGPNVNKGEDIQLLKSAYENFNQ
EPI_ISL_426900   263 NAMQVESDDYIATNGPLKVGGSCVLSGHNLAKHCLHVVGPNVNKGEDIQLLKSAYENFNQ
EPI_ISL_426901   263 NAMQVESDDYIATNGPLKVGGSCVLSGHNLAKHCLHVVGPNVNKGEDIQLLKSAYENFNQ
EPI_ISL_426903   263 NAMQVESDDYIATNGPLKVGGSCVLSGHNLAKHCLHVVGPNVNKGEDIQLLKSAYENFNQ
EPI_ISL_426904   263 NAMQVESDDYIATNGPLKVGGSCVLSGHNLAKHCLHVVGPNVNKGEDIQLLKSAYENFNQ
EPI_ISL_426907   263 NAMQVESDDYIATNGPLKVGGSCVLSGHNLAKHCLHVVGPNVNKGEDIQLLKSAYENFNQ
EPI_ISL_426910   263 NAMQVESDDYIATNGPLKVGGSCVLSGHNLAKHCLHVVGPNVNKGEDIQLLKSAYENFNQ
ORF1ab          1081 NAMQVESDDYIATNGPLKVGGSCVLSGHNLAKHCLHVVGPNVNKGEDIQLLKSAYENFNQ
EPI_ISL_413575   263 NAMQVESDDYIATNGPLKVGGSCVLSGHNLAKHCLHVVGPNVNKGEDIQLLKSAYENFNQ
EPI_ISL_413588   263 NAMQVESDDYIATNGPLKVGGSCVLSGHNLAKHCLHVVGPNVNKGEDIQLLKSAYENFNQ
EPI_ISL_413589   263 NAMQVESDDYIATNGPLKVGGSCVLSGHNLAKHCLHVVGPNVNKGEDIQLLKSAYENFNQ
EPI_ISL_424221   263 NAMQVESDDYIATNGPLKVGGSCVLSGHNLAKHCLHVVGPNVNKGEDIQLLKSAYENFNQ
EPI_ISL_424233   263 NAMQVESDDYIATNGPLKVGGSCVLSGHNLAKHCLHVVGPNVNKGEDIQLLKSAYENFNQ
EPI_ISL_424236   263 NAMQVESDDYIATNGPLKVGGSCVLSGHNLAKHCLHVVGPNVNKGEDIQLLKSAYENFNQ
EPI_ISL_424238   263 NAMQVESDDYIATNGPLKVGGSCVLSGHNLAKHCLHVVGPNVNKGEDIQLLKSAYENFNQ
EPI_ISL_424248   263 NAMQVESDDYIATNGPLKVGGSCVLSGHNLAKHCLHVVGPNVNKGEDIQLLKSAYENFNQ
EPI_ISL_424254   263 NAMQVESDDYIATNGPLKVGGSCVLSGHNLAKHCLHVVGPNVNKGEDIQLLKSAYENFNQ
EPI_ISL_425548   263 NAMQVESDDYIATNGPLKVGGSCVLSGHNLAKHCLHVVGPNVNKGEDIQLLKSAYENFNQ
EPI_ISL_425581   263 NAMQVESDDYIATNGPLKVGGSCVLSGHNLAKHCLHVVGPNVNKGEDIQLLKSAYENFNQ
EPI_ISL_426881   263 NAMQVESDDYIATNGPLKVGGSCVLSGHNLAKHCLHVVGPNVNKGEDIQLLKSAYENFNQ
EPI_ISL_426883   263 NAMQVESDDYIATNGPLKVGGSCVLSGHNLAKHCLHVVGPNVNKGEDIQLLKSAYENFNQ
EPI_ISL_426888   263 NAMQVESDDYIATNGPLKVGGSCVLSGHNLAKHCLHVVGPNVNKGEDIQLLKSAYENFNQ
EPI_ISL_426900   263 NAMQVESDDYIATNGPLKVGGSCVLSGHNLAKHCLHVVGPNVNKGEDIQLLKSAYENFNQ
EPI_ISL_426901   263 NAMQVESDDYIATNGPLKVGGSCVLSGHNLAKHCLHVVGPNVNKGEDIQLLKSAYENFNQ
EPI_ISL_426903   263 NAMQVESDDYIATNGPLKVGGSCVLSGHNLAKHCLHVVGPNVNKGEDIQLLKSAYENFNQ
EPI_ISL_426904   263 NAMQVESDDYIATNGPLKVGGSCVLSGHNLAKHCLHVVGPNVNKGEDIQLLKSAYENFNQ
EPI_ISL_426907   263 NAMQVESDDYIATNGPLKVGGSCVLSGHNLAKHCLHVVGPNVNKGEDIQLLKSAYENFNQ
EPI_ISL_426910   263 NAMQVESDDYIATNGPLKVGGSCVLSGHNLAKHCLHVVGPNVNKGEDIQLLKSAYENFNQ
```

```
ORF1ab         1141 HEVLLAPLLSAGIFGADPIHSLRVCVDTVRTNVYLAVFDKNLYDKLVSSFLEMKSEKQVE
EPI_ISL_413575  323 HEVLLAPLLSAGIFGADPIHSLRVCVDTVRTNVYLAVFDKNLYDKLVSSFLEMKSEKQVE
EPI_ISL_413588  323 HEVLLAPLLSAGIFGADPIHSLRVCVDTVRTNVYLAVFDKNLYDKLVSSFLEMKSEKQVE
EPI_ISL_413589  323 HEVLLAPLLSAGIFGADPIHSLRVCVDTVRTNVYLAVFDKNLYDKLVSSFLEMKSEKQVE
EPI_ISL_424221  323 HEVLLAPLLSAGIFGADPIHSLRVCVDTVRTNVYLAVFDKNLYDKLVSSFLEMKSEKQVE
EPI_ISL_424233  323 HEVLLAPLLSAGIFGADPIHSLRVCVDTVRTNVYLAVFDKNLYDKLVSSFLEMKSEKQVE
EPI_ISL_424236  323 HEVLLAPLLSAGIFGADPIHSLRVCVDTVRTNVYLAVFDKNLYDKLVSSFLEMKSEKQVE
EPI_ISL_424238  323 HEVLLAPLLSAGIFGADPIHSLRVCVDTVRTNVYLAVFDKNLYDKLVSSFLEMKSEKQVE
EPI_ISL_424248  323 HEVLLAPLLSAGIFGADPIHSLRVCVDTVRTNVYLAVFDKNLYDKLVSSFLEMKSEKQVE
EPI_ISL_424254  323 HEVLLAPLLSAGIFGADPIHSLRVCVDTVRTNVYLAVFDKNLYDKLVSSFLEMKSEKQVE
EPI_ISL_425548  323 HEVLLAPLLSAGIFGADPIHSLRVCVDTVRTNVYLAVFDKNLYDKLVSSFLEMKSEKQVE
EPI_ISL_425581  323 HEVLLAPLLSAGIFGADPIHSLRVCVDTVRTNVYLAVFDKNLYDKLVSSFLEMKSEKQVE
EPI_ISL_426881  323 HEVLLAPLLSAGIFGADPIHSLRVCVDTVRTNVYLAVFDKNLYDKLVSSFLEMKSEKQVE
EPI_ISL_426883  323 HEVLLAPLLSAGIFGADPIHSLRVCVDTVRTNVYLAVFDKNLYDKLVSSFLEMKSEKQVE
EPI_ISL_426888  323 HEVLLAPLLSAGIFGADPIHSLRVCVDTVRTNVYLAVFDKNLYDKLVSSFLEMKSEKQVE
EPI_ISL_426900  323 HEVLLAPLLSAGIFGADPIHSLRVCVDTVRTNVYLAVFDKNLYDKLVSSFLEMKSEKQVE
EPI_ISL_426901  323 HEVLLAPLLSAGIFGADPIHSLRVCVDTVRTNVYLAVFDKNLYDKLVSSFLEMKSEKQVE
EPI_ISL_426903  323 HEVLLAPLLSAGIFGADPIHSLRVCVDTVRTNVYLAVFDKNLYDKLVSSFLEMKSEKQVE
EPI_ISL_426904  323 HEVLLAPLLSAGIFGADPIHSLRVCVDTVRTNVYLAVFDKNLYDKLVSSFLEMKSEKQVE
EPI_ISL_426907  323 HEVLLAPLLSAGIFGADPIHSLRVCVDTVRTNVYLAVFDKNLYDKLVSSFLEMKSEKQVE
EPI_ISL_426910  323 HEVLLAPLLSAGIFGADPIHSLRVCVDTVRTNVYLAVFDKNLYDKLVSSFLEMKSEKQVE
ORF1ab         1141 HEVLLAPLLSAGIFGADPIHSLRVCVDTVRTNVYLAVFDKNLYDKLVSSFLEMKSEKQVE
EPI_ISL_413575  323 HEVLLAPLLSAGIFGADPIHSLRVCVDTVRTNVYLAVFDKNLYDKLVSSFLEMKSEKQVE
EPI_ISL_413588  323 HEVLLAPLLSAGIFGADPIHSLRVCVDTVRTNVYLAVFDKNLYDKLVSSFLEMKSEKQVE
EPI_ISL_413589  323 HEVLLAPLLSAGIFGADPIHSLRVCVDTVRTNVYLAVFDKNLYDKLVSSFLEMKSEKQVE
EPI_ISL_424221  323 HEVLLAPLLSAGIFGADPIHSLRVCVDTVRTNVYLAVFDKNLYDKLVSSFLEMKSEKQVE
EPI_ISL_424233  323 HEVLLAPLLSAGIFGADPIHSLRVCVDTVRTNVYLAVFDKNLYDKLVSSFLEMKSEKQVE
EPI_ISL_424236  323 HEVLLAPLLSAGIFGADPIHSLRVCVDTVRTNVYLAVFDKNLYDKLVSSFLEMKSEKQVE
EPI_ISL_424238  323 HEVLLAPLLSAGIFGADPIHSLRVCVDTVRTNVYLAVFDKNLYDKLVSSFLEMKSEKQVE
EPI_ISL_424248  323 HEVLLAPLLSAGIFGADPIHSLRVCVDTVRTNVYLAVFDKNLYDKLVSSFLEMKSEKQVE
EPI_ISL_424254  323 HEVLLAPLLSAGIFGADPIHSLRVCVDTVRTNVYLAVFDKNLYDKLVSSFLEMKSEKQVE
EPI_ISL_425548  323 HEVLLAPLLSAGIFGADPIHSLRVCVDTVRTNVYLAVFDKNLYDKLVSSFLEMKSEKQVE
EPI_ISL_425581  323 HEVLLAPLLSAGIFGADPIHSLRVCVDTVRTNVYLAVFDKNLYDKLVSSFLEMKSEKQVE
EPI_ISL_426881  323 HEVLLAPLLSAGIFGADPIHSLRVCVDTVRTNVYLAVFDKNLYDKLVSSFLEMKSEKQVE
EPI_ISL_426883  323 HEVLLAPLLSAGIFGADPIHSLRVCVDTVRTNVYLAVFDKNLYDKLVSSFLEMKSEKQVE
EPI_ISL_426888  323 HEVLLAPLLSAGIFGADPIHSLRVCVDTVRTNVYLAVFDKNLYDKLVSSFLEMKSEKQVE
EPI_ISL_426900  323 HEVLLAPLLSAGIFGADPIHSLRVCVDTVRTNVYLAVFDKNLYDKLVSSFLEMKSEKQVE
EPI_ISL_426901  323 HEVLLAPLLSAGIFGADPIHSLRVCVDTVRTNVYLAVFDKNLYDKLVSSFLEMKSEKQVE
EPI_ISL_426903  323 HEVLLAPLLSAGIFGADPIHSLRVCVDTVRTNVYLAVFDKNLYDKLVSSFLEMKSEKQVE
EPI_ISL_426904  323 HEVLLAPLLSAGIFGADPIHSLRVCVDTVRTNVYLAVFDKNLYDKLVSSFLEMKSEKQVE
EPI_ISL_426907  323 HEVLLAPLLSAGIFGADPIHSLRVCVDTVRTNVYLAVFDKNLYDKLVSSFLEMKSEKQVE
EPI_ISL_426910  323 HEVLLAPLLSAGIFGADPIHSLRVCVDTVRTNVYLAVFDKNLYDKLVSSFLEMKSEKQVE
```

```
ORF1ab         1201 QKIAEIPKEEVKPFITESKPSVEQRKQDDKKIKACVEEVTTTLEETKFLTENLLLYIDIN
EPI_ISL_413575  383 QKIAEIPKEEVKPFITESKPSVEQRKQDDKKIKACVEEVTTTLEETKFLTENLLLYIDIN
EPI_ISL_413588  383 QKIAEIPKEEVKPFITESKPSVEQRKQDDKKIKACVEEVTTTLEETKFLTENLLLYIDIN
EPI_ISL_413589  383 QKIAEIPKEEVKPFITESKPSVEQRKQDDKKIKACVEEVTTTLEETKFLTENLLLYIDIN
EPI_ISL_424221  383 QKIAEIPKEEVKPFITESKPSVEQRKQDDKKIKACVEEVTTTLEETKFLTENLLLYIDIN
EPI_ISL_424233  383 QKIAEIPKEEVKPFITESKPSVEQRKQDDKKIKACVEEVTTTLEETKFLTENLLLYIDIN
EPI_ISL_424236  383 QKIAEIPKEEVKPFITESKPSVEQRKQDDKKIKACVEEVTTTLEETKFLTENLLLYIDIN
EPI_ISL_424238  383 QKIAEIPKEEVKPFITESKPSVEQRKQDDKKIKACVEEVTTTLEETKFLTENLLLYIDIN
EPI_ISL_424248  383 QKIAEIPKEEVKPFITESKPSVEQRKQDDKKIKACVEEVTTTLEETKFLTENLLLYIDIN
EPI_ISL_424254  383 QKIAEIPKEEVKPFITESKPSVEQRKQDDKKIKACVEEVTTTLEETKFLTENLLLYIDIN
EPI_ISL_425548  383 QKIAEIPKEEVKPFITESKPSVEQRKQDDKKIKACVEEVTTTLEETKFLTENLLLYIDIN
EPI_ISL_425581  383 QKIAEIPKEEVKPFITESKPSVEQRKQDDKKIKACVEEVTTTLEETKFLTENLLLYIDIN
EPI_ISL_426881  383 QKIAEIPKEEVKPFITESKPSVEQRKQDDKKIKACVEEVTTTLEETKFLTENLLLYIDIN
EPI_ISL_426883  383 QKIAEIPKEEVKPFITESKPSVEQRKQDDKKIKACVEEVTTTLEETKFLTENLLLYIDIN
EPI_ISL_426888  383 QKIAEIPKEEVKPFITESKPSVEQRKQDDKKIKACVEEVTTTLEETKFLTENLLLYIDIN
EPI_ISL_426900  383 QKIAEIPKEEVKPFITESKPSVEQRKQDDKKIKACVEEVTTTLEETKFLTENLLLYIDIN
EPI_ISL_426901  383 QKIAEIPKEEVKPFITESKPSVEQRKQDDKKIKACVEEVTTTLEETKFLTENLLLYIDIN
EPI_ISL_426903  383 QKIAEIPKEEVKPFITESKPSVEQRKQDDKKIKACVEEVTTTLEETKFLTENLLLYIDIN
EPI_ISL_426904  383 QKIAEIPKEEVKPFITESKPSVEQRKQDDKKIKACVEEVTTTLEETKFLTENLLLYIDIN
EPI_ISL_426907  383 QKIAEIPKEEVKPFITESKPSVEQRKQDDKKIKACVEEVTTTLEETKFLTENLLLYIDIN
EPI_ISL_426910  383 QKIAEIPKEEVKPFITESKPSVEQRKQDDKKIKACVEEVTTTLEETKFLTENLLLYIDIN
```


```
ORF1ab         1201 QKIAEIPKEEVKPFITESKPSVEQRKQDDKKIKACVEEVTTTLEETKFLTENLLLYIDIN
EPI_ISL_413575  383 QKIAEIPKEEVKPFITESKPSVEQRKQDDKKIKACVEEVTTTLEETKFLTENLLLYIDIN
EPI_ISL_413588  383 QKIAEIPKEEVKPFITESKPSVEQRKQDDKKIKACVEEVTTTLEETKFLTENLLLYIDIN
EPI_ISL_413589  383 QKIAEIPKEEVKPFITESKPSVEQRKQDDKKIKACVEEVTTTLEETKFLTENLLLYIDIN
EPI_ISL_424221  383 QKIAEIPKEEVKPFITESKPSVEQRKQDDKKIKACVEEVTTTLEETKFLTENLLLYIDIN
EPI_ISL_424233  383 QKIAEIPKEEVKPFITESKPSVEQRKQDDKKIKACVEEVTTTLEETKFLTENLLLYIDIN
EPI_ISL_424236  383 QKIAEIPKEEVKPFITESKPSVEQRKQDDKKIKACVEEVTTTLEETKFLTENLLLYIDIN
EPI_ISL_424238  383 QKIAEIPKEEVKPFITESKPSVEQRKQDDKKIKACVEEVTTTLEETKFLTENLLLYIDIN
EPI_ISL_424248  383 QKIAEIPKEEVKPFITESKPSVEQRKQDDKKIKACVEEVTTTLEETKFLTENLLLYIDIN
EPI_ISL_424254  383 QKIAEIPKEEVKPFITESKPSVEQRKQDDKKIKACVEEVTTTLEETKFLTENLLLYIDIN
EPI_ISL_425548  383 QKIAEIPKEEVKPFITESKPSVEQRKQDDKKIKACVEEVTTTLEETKFLTENLLLYIDIN
EPI_ISL_425581  383 QKIAEIPKEEVKPFITESKPSVEQRKQDDKKIKACVEEVTTTLEETKFLTENLLLYIDIN
EPI_ISL_426881  383 QKIAEIPKEEVKPFITESKPSVEQRKQDDKKIKACVEEVTTTLEETKFLTENLLLYIDIN
EPI_ISL_426883  383 QKIAEIPKEEVKPFITESKPSVEQRKQDDKKIKACVEEVTTTLEETKFLTENLLLYIDIN
EPI_ISL_426888  383 QKIAEIPKEEVKPFITESKPSVEQRKQDDKKIKACVEEVTTTLEETKFLTENLLLYIDIN
EPI_ISL_426900  383 QKIAEIPKEEVKPFITESKPSVEQRKQDDKKIKACVEEVTTTLEETKFLTENLLLYIDIN
EPI_ISL_426901  383 QKIAEIPKEEVKPFITESKPSVEQRKQDDKKIKACVEEVTTTLEETKFLTENLLLYIDIN
EPI_ISL_426903  383 QKIAEIPKEEVKPFITESKPSVEQRKQDDKKIKACVEEVTTTLEETKFLTENLLLYIDIN
EPI_ISL_426904  383 QKIAEIPKEEVKPFITESKPSVEQRKQDDKKIKACVEEVTTTLEETKFLTENLLLYIDIN
EPI_ISL_426907  383 QKIAEIPKEEVKPFITESKPSVEQRKQDDKKIKACVEEVTTTLEETKFLTENLLLYIDIN
EPI_ISL_426910  383 QKIAEIPKEEVKPFITESKPSVEQRKQDDKKIKACVEEVTTTLEETKFLTENLLLYIDIN
```

```
ORF1ab          1261 GNLHPDSATLVSDIDITFLKKDAPYIVGDVVQEGVLTAVVIPTKKAGGTTEMLAKALRKV
EPI_ISL_413575   443 GNLHPDSATLVSDIDITFLKKDAPYIVGDVVQEGVLTAVVIPTKKAGGTTEMLAKALRKV
EPI_ISL_413588   443 GNLHPDSATLVSDIDITFLKKDAPYIVGDVVQEGVLTAVVIPTKKAGGTTEMLAKALRKV
EPI_ISL_413589   443 GNLHPDSATLVSDIDITFLKKDAPYIVGDVVQEGVLTAVVIPTKKAGGTTEMLAKALRKV
EPI_ISL_424221   443 GNLHPDSATLVSDIDITFLKKDAPYIVGDVVQEGVLTAVVIPTKKAGGTTEMLAKALRKV
EPI_ISL_424233   443 GNLHPDSATLVSDIDITFLKKDAPYIVGDVVQEGVLTAVVIPTKKAGGTTEMLAKALRKV
EPI_ISL_424236   443 GNLHPDSATLVSDIDITFLKKDAPYIVGDVVQEGVLTAVVIPTKKAGGTTEMLAKALRKV
EPI_ISL_424238   443 GNLHPDSATLVSDIDITFLKKDAPYIVGDVVQEGVLTAVVIPTKKAGGTTEMLAKALRKV
EPI_ISL_424248   443 GNLHPDSATLVSDIDITFLKKDAPYIVGDVVQEGVLTAVVIPTKKAGGTTEMLAKALRKV
EPI_ISL_424254   443 GNLHPDSATLVSDIDITFLKKDAPYIVGDVVQEGVLTAVVIPTKKAGGTTEMLAKALRKV
EPI_ISL_425548   443 GNLHPDSATLVSDIDITFLKKDAPYIVGDVVQEGVLTAVVIPTKKAGGTTEMLAKALRKV
EPI_ISL_425581   443 GNLHPDSATLVSDIDITFLKKDAPYIVGDVVQEGVLTAVVIPTKKAGGTTEMLAKALRKV
EPI_ISL_426881   443 GNLHPDSATLVSDIDITFLKKDAPYIVGDVVQEGVLTAVVIPTKKAGGTTEMLAKALRKV
EPI_ISL_426883   443 GNLHPDSATLVSDIDITFLKKDAPYIVGDVVQEGVLTAVVIPTKKAGGTTEMLAKALRKV
EPI_ISL_426888   443 GNLHPDSATLVSDIDITFLKKDAPYIVGDVVQEGVLTAVVIPTKKAGGTTEMLAKALRKV
EPI_ISL_426900   443 GNLHPDSATLVSDIDITFLKKDAPYIVGDVVQEGVLTAVVIPTKKAGGTTEMLAKALRKV
EPI_ISL_426901   443 GNLHPDSATLVSDIDITFLKKDAPYIVGDVVQEGVLTAVVIPTKKAGGTTEMLAKALRKV
EPI_ISL_426903   443 GNLHPDSATLVSDIDITFLKKDAPYIVGDVVQEGVLTAVVIPTKKAGGTTEMLAKALRKV
EPI_ISL_426904   443 GNLHPDSATLVSDIDITFLKKDAPYIVGDVVQEGVLTAVVIPTKKAGGTTEMLAKALRKV
EPI_ISL_426907   443 GNLHPDSATLVSDIDITFLKKDAPYIVGDVVQEGVLTAVVIPTKKAGGTTEMLAKALRKV
EPI_ISL_426910   443 GNLHPDSATLVSDIDITFLKKDAPYIVGDVVQEGVLTAVVIPTKKAGGTTEMLAKALRKV
ORF1ab          1261 GNLHPDSATLVSDIDITFLKKDAPYIVGDVVQEGVLTAVVIPTKKAGGTTEMLAKALRKV
EPI_ISL_413575   443 GNLHPDSATLVSDIDITFLKKDAPYIVGDVVQEGVLTAVVIPTKKAGGTTEMLAKALRKV
EPI_ISL_413588   443 GNLHPDSATLVSDIDITFLKKDAPYIVGDVVQEGVLTAVVIPTKKAGGTTEMLAKALRKV
EPI_ISL_413589   443 GNLHPDSATLVSDIDITFLKKDAPYIVGDVVQEGVLTAVVIPTKKAGGTTEMLAKALRKV
EPI_ISL_424221   443 GNLHPDSATLVSDIDITFLKKDAPYIVGDVVQEGVLTAVVIPTKKAGGTTEMLAKALRKV
EPI_ISL_424233   443 GNLHPDSATLVSDIDITFLKKDAPYIVGDVVQEGVLTAVVIPTKKAGGTTEMLAKALRKV
EPI_ISL_424236   443 GNLHPDSATLVSDIDITFLKKDAPYIVGDVVQEGVLTAVVIPTKKAGGTTEMLAKALRKV
EPI_ISL_424238   443 GNLHPDSATLVSDIDITFLKKDAPYIVGDVVQEGVLTAVVIPTKKAGGTTEMLAKALRKV
EPI_ISL_424248   443 GNLHPDSATLVSDIDITFLKKDAPYIVGDVVQEGVLTAVVIPTKKAGGTTEMLAKALRKV
EPI_ISL_424254   443 GNLHPDSATLVSDIDITFLKKDAPYIVGDVVQEGVLTAVVIPTKKAGGTTEMLAKALRKV
EPI_ISL_425548   443 GNLHPDSATLVSDIDITFLKKDAPYIVGDVVQEGVLTAVVIPTKKAGGTTEMLAKALRKV
EPI_ISL_425581   443 GNLHPDSATLVSDIDITFLKKDAPYIVGDVVQEGVLTAVVIPTKKAGGTTEMLAKALRKV
EPI_ISL_426881   443 GNLHPDSATLVSDIDITFLKKDAPYIVGDVVQEGVLTAVVIPTKKAGGTTEMLAKALRKV
EPI_ISL_426883   443 GNLHPDSATLVSDIDITFLKKDAPYIVGDVVQEGVLTAVVIPTKKAGGTTEMLAKALRKV
EPI_ISL_426888   443 GNLHPDSATLVSDIDITFLKKDAPYIVGDVVQEGVLTAVVIPTKKAGGTTEMLAKALRKV
EPI_ISL_426900   443 GNLHPDSATLVSDIDITFLKKDAPYIVGDVVQEGVLTAVVIPTKKAGGTTEMLAKALRKV
EPI_ISL_426901   443 GNLHPDSATLVSDIDITFLKKDAPYIVGDVVQEGVLTAVVIPTKKAGGTTEMLAKALRKV
EPI_ISL_426903   443 GNLHPDSATLVSDIDITFLKKDAPYIVGDVVQEGVLTAVVIPTKKAGGTTEMLAKALRKV
EPI_ISL_426904   443 GNLHPDSATLVSDIDITFLKKDAPYIVGDVVQEGVLTAVVIPTKKAGGTTEMLAKALRKV
EPI_ISL_426907   443 GNLHPDSATLVSDIDITFLKKDAPYIVGDVVQEGVLTAVVIPTKKAGGTTEMLAKALRKV
EPI_ISL_426910   443 GNLHPDSATLVSDIDITFLKKDAPYIVGDVVQEGVLTAVVIPTKKAGGTTEMLAKALRKV
```

```
ORF1ab          1321 PTDNYITTYPGQGLNGYTVEEAKTVLKKCKSAFYILPSIISNEKQEILGTVSWNLREMLA
EPI_ISL_413575   503 PTDNYITTYPGQGLNGYTVEEAKTVLKKCKSAFYILPSIISNEKQEILGTVSWNLREMLA
EPI_ISL_413588   503 PTDNYITTYPGQGLNGYTVEEAKTVLKKCKSAFYILPSIISNEKQEILGTVSWNLREMLA
EPI_ISL_413589   503 PTDNYITTYPGQGLNGYTVEEAKTVLKKCKSAFYILPSIISNEKQEILGTVSWNLREMLA
EPI_ISL_424221   503 PTDNYITTYPGQGLNGYTVEEAKTVLKKCKSAFYILPSIISNEKQEILGTVSWNLREMLA
EPI_ISL_424233   503 PTDNYITTYPGQGLNGYTVEEAKTVLKKCKSAFYILPSIISNEKQEILGTVSWNLREMLA
EPI_ISL_424236   503 PTDNYITTYPGQGLNGYTVEEAKTVLKKCKSAFYILPSIISNEKQEILGTVSWNLREMLA
EPI_ISL_424238   503 PTDNYITTYPGQGLNGYTVEEAKTVLKKCKSAFYILPSIISNEKQEILGTVSWNLREMLA
EPI_ISL_424248   503 PTDNYITTYPGQGLNGYTVEEAKTVLKKCKSAFYILPSIISNEKQEILGTVSWNLREMLA
EPI_ISL_424254   503 PTDNYITTYPGQGLNGYTVEEAKTVLKKCKSAFYILPSIISNEKQEILGTVSWNLREMLA
EPI_ISL_425548   503 PTDNYITTYPGQGLNGYTVEEAKTVLKKCKSAFYILPSIISNEKQEILGTVSWNLREMLA
EPI_ISL_425581   503 PTDNYITTYPGQGLNGYTVEEAKTVLKKCKSAFYILPSIISNEKQEILGTVSWNLREMLA
EPI_ISL_426881   503 PTDNYITTYPGQGLNGYTVEEAKTVLKKCKSAFYILPSIISNEKQEILGTVSWNLREMLA
EPI_ISL_426883   503 PTDNYITTYPGQGLNGYTVEEAKTVLKKCKSAFYILPSIISNEKQEILGTVSWNLREMLA
EPI_ISL_426888   503 PTDNYITTYPGQGLNGYTVEEAKTVLKKCKSAFYILPSIISNEKQEILGTVSWNLREMLA
EPI_ISL_426900   503 PTDNYITTYPGQGLNGYTVEEAKTVLKKCKSAFYILPSIISNEKQEILGTVSWNLREMLA
EPI_ISL_426901   503 PTDNYITTYPGQGLNGYTVEEAKTVLKKCKSAFYILPSIISNEKQEILGTVSWNLREMLA
EPI_ISL_426903   503 PTDNYITTYPGQGLNGYTVEEAKTVLKKCKSAFYILPSIISNEKQEILGTVSWNLREMLA
EPI_ISL_426904   503 PTDNYITTYPGQGLNGYTVEEAKTVLKKCKSAFYILPSIISNEKQEILGTVSWNLREMLA
EPI_ISL_426907   503 PTDNYITTYPGQGLNGYTVEEAKTVLKKCKSAFYILPSIISNEKQEILGTVSWNLREMLA
EPI_ISL_426910   503 PTDNYITTYPGQGLNGYTVEEAKTVLKKCKSAFYILPSIISNEKQEILGTVSWNLREMLA
ORF1ab          1321 PTDNYITTYPGQGLNGYTVEEAKTVLKKCKSAFYILPSIISNEKQEILGTVSWNLREMLA
EPI_ISL_413575   503 PTDNYITTYPGQGLNGYTVEEAKTVLKKCKSAFYILPSIISNEKQEILGTVSWNLREMLA
EPI_ISL_413588   503 PTDNYITTYPGQGLNGYTVEEAKTVLKKCKSAFYILPSIISNEKQEILGTVSWNLREMLA
EPI_ISL_413589   503 PTDNYITTYPGQGLNGYTVEEAKTVLKKCKSAFYILPSIISNEKQEILGTVSWNLREMLA
EPI_ISL_424221   503 PTDNYITTYPGQGLNGYTVEEAKTVLKKCKSAFYILPSIISNEKQEILGTVSWNLREMLA
EPI_ISL_424233   503 PTDNYITTYPGQGLNGYTVEEAKTVLKKCKSAFYILPSIISNEKQEILGTVSWNLREMLA
EPI_ISL_424236   503 PTDNYITTYPGQGLNGYTVEEAKTVLKKCKSAFYILPSIISNEKQEILGTVSWNLREMLA
EPI_ISL_424238   503 PTDNYITTYPGQGLNGYTVEEAKTVLKKCKSAFYILPSIISNEKQEILGTVSWNLREMLA
EPI_ISL_424248   503 PTDNYITTYPGQGLNGYTVEEAKTVLKKCKSAFYILPSIISNEKQEILGTVSWNLREMLA
EPI_ISL_424254   503 PTDNYITTYPGQGLNGYTVEEAKTVLKKCKSAFYILPSIISNEKQEILGTVSWNLREMLA
EPI_ISL_425548   503 PTDNYITTYPGQGLNGYTVEEAKTVLKKCKSAFYILPSIISNEKQEILGTVSWNLREMLA
EPI_ISL_425581   503 PTDNYITTYPGQGLNGYTVEEAKTVLKKCKSAFYILPSIISNEKQEILGTVSWNLREMLA
EPI_ISL_426881   503 PTDNYITTYPGQGLNGYTVEEAKTVLKKCKSAFYILPSIISNEKQEILGTVSWNLREMLA
EPI_ISL_426883   503 PTDNYITTYPGQGLNGYTVEEAKTVLKKCKSAFYILPSIISNEKQEILGTVSWNLREMLA
EPI_ISL_426888   503 PTDNYITTYPGQGLNGYTVEEAKTVLKKCKSAFYILPSIISNEKQEILGTVSWNLREMLA
EPI_ISL_426900   503 PTDNYITTYPGQGLNGYTVEEAKTVLKKCKSAFYILPSIISNEKQEILGTVSWNLREMLA
EPI_ISL_426901   503 PTDNYITTYPGQGLNGYTVEEAKTVLKKCKSAFYILPSIISNEKQEILGTVSWNLREMLA
EPI_ISL_426903   503 PTDNYITTYPGQGLNGYTVEEAKTVLKKCKSAFYILPSIISNEKQEILGTVSWNLREMLA
EPI_ISL_426904   503 PTDNYITTYPGQGLNGYTVEEAKTVLKKCKSAFYILPSIISNEKQEILGTVSWNLREMLA
EPI_ISL_426907   503 PTDNYITTYPGQGLNGYTVEEAKTVLKKCKSAFYILPSIISNEKQEILGTVSWNLREMLA
EPI_ISL_426910   503 PTDNYITTYPGQGLNGYTVEEAKTVLKKCKSAFYILPSIISNEKQEILGTVSWNLREMLA
```

ORF1ab	1381	HAEETRKLMPVCVETKAIVSTIQRKYKGIKIQEGVVDYGARFYFYTSKTTVASLINTLND
EPI_ISL_413575	563	HAEETRKLMPVCVETKAIVSTIQRKYKGIKIQEGVVDYGARFYFYTSKTTVASLINTLND
EPI_ISL_413588	563	HAEETRKLMPVCVETKAIVSTIQRKYKGIKIQEGVVDYGARFYFYTSKTTVASLINTLND
EPI_ISL_413589	563	HAEETRKLMPVCVETKAIVSTIQRKYKGIKIQEGVVDYGARFYFYTSKTTVASLINTLND
EPI_ISL_424221	563	HAEETRKLMPVCVETKAIVSTIQRKYKGIKIQEGVVDYGARFYFYTSKTTVASLINTLND
EPI_ISL_424233	563	HAEETRKLMPVCVETKAIVSTIQRKYKGIKIQEGVVDYGARFYFYTSKTTVASLINTLND
EPI_ISL_424236	563	HAEETRKLMPVCVETKAIVSTIQRKYKGIKIQEGVVDYGARFYFYTSKTTVASLINTLND
EPI_ISL_424238	563	HAEETRKLMPVCVETKAIVSTIQRKYKGIKIQEGVVDYGARFYFYTSKTTVASLINTLND
EPI_ISL_424248	563	HAEETRKLMPVCVETKAIVSTIQRKYKGIKIQEGVVDYGARFYFYTSKTTVASLINTLND
EPI_ISL_424254	563	HAEETRKLMPVCVETKAIVSTIQRKYKGIKIQEGVVDYGARFYFYTSKTTVASLINTLND
EPI_ISL_425548	563	HAEETRKLMPVCVETKAIVSTIQRKYKGIKIQEGVVDYGARFYFYTSKTTVASLINTLND
EPI_ISL_425581	563	HAEETRKLMPVCVETKAIVSTIQRKYKGIKIQEGVVDYGARFYFYTSKTTVASLINTLND
EPI_ISL_426881	563	HAEETRKLMPVCVETKAIVSTIQRKYKGIKIQEGVVDYGARFYFYTSKTTVASLINTLND
EPI_ISL_426883	563	HAEETRKLMPVCVETKAIVSTIQRKYKGIKIQEGVVDYGARFYFYTSKTTVASLINTLND
EPI_ISL_426888	563	HAEETRKLMPVCVETKAIVSTIQRKYKGIKIQEGVVDYGARFYFYTSKTTVASLINTLND
EPI_ISL_426900	563	HAEETRKLMPVCVETKAIVSTIQRKYKGIKIQEGVVDYGARFYFYTSKTTVASLINTLND
EPI_ISL_426901	563	HAEETRKLMPVCVETKAIVSTIQRKYKGIKIQEGVVDYGARFYFYTSKTTVASLINTLND
EPI_ISL_426903	563	HAEETRKLMPVCVETKAIVSTIQRKYKGIKIQEGVVDYGARFYFYTSKTTVASLINTLND
EPI_ISL_426904	563	HAEETRKLMPVCVETKAIVSTIQRKYKGIKIQEGVVDYGARFYFYTSKTTVASLINTLND
EPI_ISL_426907	563	HAEETRKLMPVCVETKAIVSTIQRKYKGIKIQEGVVDYGARFYFYTSKTTVASLINTLND
EPI_ISL_426910	563	HAEETRKLMPVCVETKAIVSTIQRKYKGIKIQEGVVDYGARFYFYTSKTTVASLINTLND
ORF1ab	1381	HAEETRKLMPVCVETKAIVSTIQRKYKGIKIQEGVVDYGARFYFYTSKTTVASLINTLND
EPI_ISL_413575	563	HAEETRKLMPVCVETKAIVSTIQRKYKGIKIQEGVVDYGARFYFYTSKTTVASLINTLND
EPI_ISL_413588	563	HAEETRKLMPVCVETKAIVSTIQRKYKGIKIQEGVVDYGARFYFYTSKTTVASLINTLND
EPI_ISL_413589	563	HAEETRKLMPVCVETKAIVSTIQRKYKGIKIQEGVVDYGARFYFYTSKTTVASLINTLND
EPI_ISL_424221	563	HAEETRKLMPVCVETKAIVSTIQRKYKGIKIQEGVVDYGARFYFYTSKTTVASLINTLND
EPI_ISL_424233	563	HAEETRKLMPVCVETKAIVSTIQRKYKGIKIQEGVVDYGARFYFYTSKTTVASLINTLND
EPI_ISL_424236	563	HAEETRKLMPVCVETKAIVSTIQRKYKGIKIQEGVVDYGARFYFYTSKTTVASLINTLND
EPI_ISL_424238	563	HAEETRKLMPVCVETKAIVSTIQRKYKGIKIQEGVVDYGARFYFYTSKTTVASLINTLND
EPI_ISL_424248	563	HAEETRKLMPVCVETKAIVSTIQRKYKGIKIQEGVVDYGARFYFYTSKTTVASLINTLND
EPI_ISL_424254	563	HAEETRKLMPVCVETKAIVSTIQRKYKGIKIQEGVVDYGARFYFYTSKTTVASLINTLND
EPI_ISL_425548	563	HAEETRKLMPVCVETKAIVSTIQRKYKGIKIQEGVVDYGARFYFYTSKTTVASLINTLND
EPI_ISL_425581	563	HAEETRKLMPVCVETKAIVSTIQRKYKGIKIQEGVVDYGARFYFYTSKTTVASLINTLND
EPI_ISL_426881	563	HAEETRKLMPVCVETKAIVSTIQRKYKGIKIQEGVVDYGARFYFYTSKTTVASLINTLND
EPI_ISL_426883	563	HAEETRKLMPVCVETKAIVSTIQRKYKGIKIQEGVVDYGARFYFYTSKTTVASLINTLND
EPI_ISL_426888	563	HAEETRKLMPVCVETKAIVSTIQRKYKGIKIQEGVVDYGARFYFYTSKTTVASLINTLND
EPI_ISL_426900	563	HAEETRKLMPVCVETKAIVSTIQRKYKGIKIQEGVVDYGARFYFYTSKTTVASLINTLND
EPI_ISL_426901	563	HAEETRKLMPVCVETKAIVSTIQRKYKGIKIQEGVVDYGARFYFYTSKTTVASLINTLND
EPI_ISL_426903	563	HAEETRKLMPVCVETKAIVSTIQRKYKGIKIQEGVVDYGARFYFYTSKTTVASLINTLND
EPI_ISL_426904	563	HAEETRKLMPVCVETKAIVSTIQRKYKGIKIQEGVVDYGARFYFYTSKTTVASLINTLND
EPI_ISL_426907	563	HAEETRKLMPVCVETKAIVSTIQRKYKGIKIQEGVVDYGARFYFYTSKTTVASLINTLND
EPI_ISL_426910	563	HAEETRKLMPVCVETKAIVSTIQRKYKGIKIQEGVVDYGARFYFYTSKTTVASLINTLND

```
ORF1ab          1441 LNETLVTMPLGYVTHGLNLEEAARYMRSLKVPATVSVSSPDAVTAYNGYLTSSSKTPEEH
EPI_ISL_413575   623 LNETLVTMPLGYVTHGLNLEEAARYMRSLKVPATVSVSSPDAVTAYNGYLTSSSKTPEEH
EPI_ISL_413588   623 LNETLVTMPLGYVTHGLNLEEAARYMRSLKVPATVSVSSPDAVTAYNGYLTSSSKTPEEH
EPI_ISL_413589   623 LNETLVTMPLGYVTHGLNLEEAARYMRSLKVPATVSVSSPDAVTAYNGYLTSSSKTPEEH
EPI_ISL_424221   623 LNETLVTMPLGYVTHGLNLEEAARYMRSLKVPATVSVSSPDAVTAYNGYLTSSSKTPEEH
EPI_ISL_424233   623 LNETLVTMPLGYVTHGLNLEEAARYMRSLKVPATVSVSSPDAVTAYNGYLTSSSKTPEEH
EPI_ISL_424236   623 LNETLVTMPLGYVTHGLNLEEAARYMRSLKVPATVSVSSPDAVTAYNGYLTSSSKTPEEH
EPI_ISL_424238   623 LNETLVTMPLGYVTHGLNLEEAARYMRSLKVPATVSVSSPDAVTAYNGYLTSSSKTPEEH
EPI_ISL_424248   623 LNETLVTMPLGYVTHGLNLEEAARYMRSLKVPATVSVSSPDAVTAYNGYLTSSSKTPEEH
EPI_ISL_424254   623 LNETLVTMPLGYVTHGLNLEEAARYMRSLKVPATVSVSSPDAVTAYNGYLTSSSKTPEEH
EPI_ISL_425548   623 LNETLVTMPLGYVTHGLNLEEAARYMRSLKVPATVSVSSPDAVTAYNGYLTSSSKTPEEH
EPI_ISL_425581   623 LNETLVTMPLGYVTHGLNLEEAARYMRSLKVPATVSVSSPDAVTAYNGYLTSSSKTPEEH
EPI_ISL_426881   623 LNETLVTMPLGYVTHGLNLEEAARYMRSLKVPATVSVSSPDAVTAYNGYLTSSSKTPEEH
EPI_ISL_426883   623 LNETLVTMPLGYVTHGLNLEEAARYMRSLKVPATVSVSSPDAVTAYNGYLTSSSKTPEEH
EPI_ISL_426888   623 LNETLVTMPLGYVTHGLNLEEAARYMRSLKVPATVSVSSPDAVTAYNGYLTSSSKTPEEH
EPI_ISL_426900   623 LNETLVTMPLGYVTHGLNLEEAARYMRSLKVPATVSVSSPDAVTAYNGYLTSSSKTPEEH
EPI_ISL_426901   623 LNETLVTMPLGYVTHGLNLEEAARYMRSLKVPATVSVSSPDAVTAYNGYLTSSSKTPEEH
EPI_ISL_426903   623 LNETLVTMPLGYVTHGLNLEEAARYMRSLKVPATVSVSSPDAVTAYNGYLTSSSKTPEEH
EPI_ISL_426904   623 LNETLVTMPLGYVTHGLNLEEAARYMRSLKVPATVSVSSPDAVTAYNGYLTSSSKTPEEH
EPI_ISL_426907   623 LNETLVTMPLGYVTHGLNLEEAARYMRSLKVPATVSVSSPDAVTAYNGYLTSSSKTPEEH
EPI_ISL_426910   623 LNETLVTMPLGYVTHGLNLEEAARYMRSLKVPATVSVSSPDAVTAYNGYLTSSSKTPEEH
ORF1ab          1441 LNETLVTMPLGYVTHGLNLEEAARYMRSLKVPATVSVSSPDAVTAYNGYLTSSSKTPEEH
EPI_ISL_413575   623 LNETLVTMPLGYVTHGLNLEEAARYMRSLKVPATVSVSSPDAVTAYNGYLTSSSKTPEEH
EPI_ISL_413588   623 LNETLVTMPLGYVTHGLNLEEAARYMRSLKVPATVSVSSPDAVTAYNGYLTSSSKTPEEH
EPI_ISL_413589   623 LNETLVTMPLGYVTHGLNLEEAARYMRSLKVPATVSVSSPDAVTAYNGYLTSSSKTPEEH
EPI_ISL_424221   623 LNETLVTMPLGYVTHGLNLEEAARYMRSLKVPATVSVSSPDAVTAYNGYLTSSSKTPEEH
EPI_ISL_424233   623 LNETLVTMPLGYVTHGLNLEEAARYMRSLKVPATVSVSSPDAVTAYNGYLTSSSKTPEEH
EPI_ISL_424236   623 LNETLVTMPLGYVTHGLNLEEAARYMRSLKVPATVSVSSPDAVTAYNGYLTSSSKTPEEH
EPI_ISL_424238   623 LNETLVTMPLGYVTHGLNLEEAARYMRSLKVPATVSVSSPDAVTAYNGYLTSSSKTPEEH
EPI_ISL_424248   623 LNETLVTMPLGYVTHGLNLEEAARYMRSLKVPATVSVSSPDAVTAYNGYLTSSSKTPEEH
EPI_ISL_424254   623 LNETLVTMPLGYVTHGLNLEEAARYMRSLKVPATVSVSSPDAVTAYNGYLTSSSKTPEEH
EPI_ISL_425548   623 LNETLVTMPLGYVTHGLNLEEAARYMRSLKVPATVSVSSPDAVTAYNGYLTSSSKTPEEH
EPI_ISL_425581   623 LNETLVTMPLGYVTHGLNLEEAARYMRSLKVPATVSVSSPDAVTAYNGYLTSSSKTPEEH
EPI_ISL_426881   623 LNETLVTMPLGYVTHGLNLEEAARYMRSLKVPATVSVSSPDAVTAYNGYLTSSSKTPEEH
EPI_ISL_426883   623 LNETLVTMPLGYVTHGLNLEEAARYMRSLKVPATVSVSSPDAVTAYNGYLTSSSKTPEEH
EPI_ISL_426888   623 LNETLVTMPLGYVTHGLNLEEAARYMRSLKVPATVSVSSPDAVTAYNGYLTSSSKTPEEH
EPI_ISL_426900   623 LNETLVTMPLGYVTHGLNLEEAARYMRSLKVPATVSVSSPDAVTAYNGYLTSSSKTPEEH
EPI_ISL_426901   623 LNETLVTMPLGYVTHGLNLEEAARYMRSLKVPATVSVSSPDAVTAYNGYLTSSSKTPEEH
EPI_ISL_426903   623 LNETLVTMPLGYVTHGLNLEEAARYMRSLKVPATVSVSSPDAVTAYNGYLTSSSKTPEEH
EPI_ISL_426904   623 LNETLVTMPLGYVTHGLNLEEAARYMRSLKVPATVSVSSPDAVTAYNGYLTSSSKTPEEH
EPI_ISL_426907   623 LNETLVTMPLGYVTHGLNLEEAARYMRSLKVPATVSVSSPDAVTAYNGYLTSSSKTPEEH
EPI_ISL_426910   623 LNETLVTMPLGYVTHGLNLEEAARYMRSLKVPATVSVSSPDAVTAYNGYLTSSSKTPEEH
```

```
ORF1ab          1501 FIETISLAGSYKDWSYSGQSTQLGIEFLKRGDKSVYYTSNPTTFHLDGEVITFDNLKTLL
EPI_ISL_413575   683 FIETISLAGSYKDWSYSGQSTQLGIEFLKRGDKSVYYTSNPTTFHLDGEVITFDNLKTLL
EPI_ISL_413588   683 FIETISLAGSYKDWSYSGQSTQLGIEFLKRGDKSVYYTSNPTTFHLDGEVITFDNLKTLL
EPI_ISL_413589   683 FIETISLAGSYKDWSYSGQSTQLGIEFLKRGDKSVYYTSNPTTFHLDGEVITFDNLKTLL
EPI_ISL_424221   683 FIETISLAGSYKDWSYSGQSTQLGIEFLKRGDKSVYYTSNPTTFHLDGEVITFDNLKTLL
EPI_ISL_424233   683 FIETISLAGSYKDWSYSGQSTQLGIEFLKRGDKSVYYTSNPTTFHLDGEVITFDNLKTLL
EPI_ISL_424236   683 FIETISLAGSYKDWSYSGQSTQLGIEFLKRGDKSVYYTSNPTTFHLDGEVITFDNLKTLL
EPI_ISL_424238   683 FIETISLAGSYKDWSYSGQSTQLGIEFLKRGDKSVYYTSNPTTFHLDGEVITFDNLKTLL
EPI_ISL_424248   683 FIETISLAGSYKDWSYSGQSTQLGIEFLKRGDKSVYYTSNPTTFHLDGEVITFDNLKTLL
EPI_ISL_424254   683 FIETISLAGSYKDWSYSGQSTQLGIEFLKRGDKSVYYTSNPTTFHLDGEVITFDNLKTLL
EPI_ISL_425548   683 FIETISLAGSYKDWSYSGQSTQLGIEFLKRGDKSVYYTSNPTTFHLDGEVITFDNLKTLL
EPI_ISL_425581   683 FIETISLAGSYKDWSYSGQSTQLGIEFLKRGDKSVYYTSNPTTFHLDGEVITFDNLKTLL
EPI_ISL_426881   683 FIETISLAGSYKDWSYSGQSTQLGIEFLKRGDKSVYYTSNPTTFHLDGEVITFDNLKTLL
EPI_ISL_426883   683 FIETISLAGSYKDWSYSGQSTQLGIEFLKRGDKSVYYTSNPTTFHLDGEVITFDNLKTLL
EPI_ISL_426888   683 FIETISLAGSYKDWSYSGQSTQLGIEFLKRGDKSVYYTSNPTTFHLDGEVITFDNLKTLL
EPI_ISL_426900   683 FIETISLAGSYKDWSYSGQSTQLGIEFLKRGDKSVYYTSNPTTFHLDGEVITFDNLKTLL
EPI_ISL_426901   683 FIETISLAGSYKDWSYSGQSTQLGIEFLKRGDKSVYYTSNPTTFHLDGEVITFDNLKTLL
EPI_ISL_426903   683 FIETISLAGSYKDWSYSGQSTQLGIEFLKRGDKSVYYTSNPTTFHLDGEVITFDNLKTLL
EPI_ISL_426904   683 FIETISLAGSYKDWSYSGQSTQLGIEFLKRGDKSVYYTSNPTTFHLDGEVITFDNLKTLL
EPI_ISL_426907   683 FIETISLAGSYKDWSYSGQSTQLGIEFLKRGDKSVYYTSNPTTFHLDGEVITFDNLKTLL
EPI_ISL_426910   683 FIETISLAGSYKDWSYSGQSTQLGIEFLKRGDKSVYYTSNPTTFHLDGEVITFDNLKTLL
ORF1ab          1501 FIETISLAGSYKDWSYSGQSTQLGIEFLKRGDKSVYYTSNPTTFHLDGEVITFDNLKTLL
EPI_ISL_413575   683 FIETISLAGSYKDWSYSGQSTQLGIEFLKRGDKSVYYTSNPTTFHLDGEVITFDNLKTLL
EPI_ISL_413588   683 FIETISLAGSYKDWSYSGQSTQLGIEFLKRGDKSVYYTSNPTTFHLDGEVITFDNLKTLL
EPI_ISL_413589   683 FIETISLAGSYKDWSYSGQSTQLGIEFLKRGDKSVYYTSNPTTFHLDGEVITFDNLKTLL
EPI_ISL_424221   683 FIETISLAGSYKDWSYSGQSTQLGIEFLKRGDKSVYYTSNPTTFHLDGEVITFDNLKTLL
EPI_ISL_424233   683 FIETISLAGSYKDWSYSGQSTQLGIEFLKRGDKSVYYTSNPTTFHLDGEVITFDNLKTLL
EPI_ISL_424236   683 FIETISLAGSYKDWSYSGQSTQLGIEFLKRGDKSVYYTSNPTTFHLDGEVITFDNLKTLL
EPI_ISL_424238   683 FIETISLAGSYKDWSYSGQSTQLGIEFLKRGDKSVYYTSNPTTFHLDGEVITFDNLKTLL
EPI_ISL_424248   683 FIETISLAGSYKDWSYSGQSTQLGIEFLKRGDKSVYYTSNPTTFHLDGEVITFDNLKTLL
EPI_ISL_424254   683 FIETISLAGSYKDWSYSGQSTQLGIEFLKRGDKSVYYTSNPTTFHLDGEVITFDNLKTLL
EPI_ISL_425548   683 FIETISLAGSYKDWSYSGQSTQLGIEFLKRGDKSVYYTSNPTTFHLDGEVITFDNLKTLL
EPI_ISL_425581   683 FIETISLAGSYKDWSYSGQSTQLGIEFLKRGDKSVYYTSNPTTFHLDGEVITFDNLKTLL
EPI_ISL_426881   683 FIETISLAGSYKDWSYSGQSTQLGIEFLKRGDKSVYYTSNPTTFHLDGEVITFDNLKTLL
EPI_ISL_426883   683 FIETISLAGSYKDWSYSGQSTQLGIEFLKRGDKSVYYTSNPTTFHLDGEVITFDNLKTLL
EPI_ISL_426888   683 FIETISLAGSYKDWSYSGQSTQLGIEFLKRGDKSVYYTSNPTTFHLDGEVITFDNLKTLL
EPI_ISL_426900   683 FIETISLAGSYKDWSYSGQSTQLGIEFLKRGDKSVYYTSNPTTFHLDGEVITFDNLKTLL
EPI_ISL_426901   683 FIETISLAGSYKDWSYSGQSTQLGIEFLKRGDKSVYYTSNPTTFHLDGEVITFDNLKTLL
EPI_ISL_426903   683 FIETISLAGSYKDWSYSGQSTQLGIEFLKRGDKSVYYTSNPTTFHLDGEVITFDNLKTLL
EPI_ISL_426904   683 FIETISLAGSYKDWSYSGQSTQLGIEFLKRGDKSVYYTSNPTTFHLDGEVITFDNLKTLL
EPI_ISL_426907   683 FIETISLAGSYKDWSYSGQSTQLGIEFLKRGDKSVYYTSNPTTFHLDGEVITFDNLKTLL
EPI_ISL_426910   683 FIETISLAGSYKDWSYSGQSTQLGIEFLKRGDKSVYYTSNPTTFHLDGEVITFDNLKTLL
```

ORF1ab	1561	SLREVRTIKVFTTVDNINLHTQVVDMSMTYGQQFGPTYLDGADVTKIKPHNSHEGKTFYV
EPI_ISL_413575	743	SLREVRTIKVFTTVDNINLHTQVVDMSMTYGQQFGPTYLDGADVTKIKPHNSHEGKTFYV
EPI_ISL_413588	743	SLREVRTIKVFTTVDNINLHTQVVDMSMTYGQQFGPTYLDGADVTKIKPHNSHEGKTFYV
EPI_ISL_413589	743	SLREVRTIKVFTTVDNINLHTQVVDMSMTYGQQFGPTYLDGADVTKIKPHNSHEGKTFYV
EPI_ISL_424221	743	SLREVRTIKVFTTVDNINLHTQVVDMSMTYGQQFGPTYLDGADVTKIKPHNSHEGKTFYV
EPI_ISL_424233	743	SLREVRTIKVFTTVDNINLHTQVVDMSMTYGQQFGPTYLDGADVTKIKPHNSHEGKTFYV
EPI_ISL_424236	743	SLREVRTIKVFTTVDNINLHTQVVDMSMTYGQQFGPTYLDGADVTKIKPHNSHEGKTFYV
EPI_ISL_424238	743	SLREVRTIKVFTTVDNINLHTQVVDMSMTYGQQFGPTYLDGADVTKIKPHNSHEGKTFYV
EPI_ISL_424248	743	SLREVRTIKVFTTVDNINLHTQVVDMSMTYGQQFGPTYLDGADVTKIKPHNSHEGKTFYV
EPI_ISL_424254	743	SLREVRTIKVFTTVDNINLHTQVVDMSMTYGQQFGPTYLDGADVTKIKPHNSHEGKTFYV
EPI_ISL_425548	743	SLREVRTIKVFTTVDNINLHTQVVDMSMTYGQQFGPTYLDGADVTKIKPHNSHEGKTFYV
EPI_ISL_425581	743	SLREVRTIKVFTTVDNINLHTQVVDMSMTYGQQFGPTYLDGADVTKIKPHNSHEGKTFYV
EPI_ISL_426881	743	SLREVRTIKVFTTVDNINLHTQVVDMSMTYGQQFGPTYLDGADVTKIKPHNSHEGKTFYV
EPI_ISL_426883	743	SLREVRTIKVFTTVDNINLHTQVVDMSMTYGQQFGPTYLDGADVTKIKPHNSHEGKTFYV
EPI_ISL_426888	743	SLREVRTIKVFTTVDNINLHTQVVDMSMTYGQQFGPTYLDGADVTKIKPHNSHEGKTFYV
EPI_ISL_426900	743	SLREVRTIKVFTTVDNINLHTQVVDMSMTYGQQFGPTYLDGADVTKIKPHNSHEGKTFYV
EPI_ISL_426901	743	SLREVRTIKVFTTVDNINLHTQVVDMSMTYGQQFGPTYLDGADVTKIKPHNSHEGKTFYV
EPI_ISL_426903	743	SLREVRTIKVFTTVDNINLHTQVVDMSMTYGQQFGPTYLDGADVTKIKPHNSHEGKTFYV
EPI_ISL_426904	743	SLREVRTIKVFTTVDNINLHTQVVDMSMTYGQQFGPTYLDGADVTKIKPHNSHEGKTFYV
EPI_ISL_426907	743	SLREVRTIKVFTTVDNINLHTQVVDMSMTYGQQFGPTYLDGADVTKIKPHNSHEGKTFYV
EPI_ISL_426910	743	SLREVRTIKVFTTVDNINLHTQVVDMSMTYGQQFGPTYLDGADVTKIKPHNSHEGKTFYV
ORF1ab	1561	SLREVRTIKVFTTVDNINLHTQVVDMSMTYGQQFGPTYLDGADVTKIKPHNSHEGKTFYV
EPI_ISL_413575	743	SLREVRTIKVFTTVDNINLHTQVVDMSMTYGQQFGPTYLDGADVTKIKPHNSHEGKTFYV
EPI_ISL_413588	743	SLREVRTIKVFTTVDNINLHTQVVDMSMTYGQQFGPTYLDGADVTKIKPHNSHEGKTFYV
EPI_ISL_413589	743	SLREVRTIKVFTTVDNINLHTQVVDMSMTYGQQFGPTYLDGADVTKIKPHNSHEGKTFYV
EPI_ISL_424221	743	SLREVRTIKVFTTVDNINLHTQVVDMSMTYGQQFGPTYLDGADVTKIKPHNSHEGKTFYV
EPI_ISL_424233	743	SLREVRTIKVFTTVDNINLHTQVVDMSMTYGQQFGPTYLDGADVTKIKPHNSHEGKTFYV
EPI_ISL_424236	743	SLREVRTIKVFTTVDNINLHTQVVDMSMTYGQQFGPTYLDGADVTKIKPHNSHEGKTFYV
EPI_ISL_424238	743	SLREVRTIKVFTTVDNINLHTQVVDMSMTYGQQFGPTYLDGADVTKIKPHNSHEGKTFYV
EPI_ISL_424248	743	SLREVRTIKVFTTVDNINLHTQVVDMSMTYGQQFGPTYLDGADVTKIKPHNSHEGKTFYV
EPI_ISL_424254	743	SLREVRTIKVFTTVDNINLHTQVVDMSMTYGQQFGPTYLDGADVTKIKPHNSHEGKTFYV
EPI_ISL_425548	743	SLREVRTIKVFTTVDNINLHTQVVDMSMTYGQQFGPTYLDGADVTKIKPHNSHEGKTFYV
EPI_ISL_425581	743	SLREVRTIKVFTTVDNINLHTQVVDMSMTYGQQFGPTYLDGADVTKIKPHNSHEGKTFYV
EPI_ISL_426881	743	SLREVRTIKVFTTVDNINLHTQVVDMSMTYGQQFGPTYLDGADVTKIKPHNSHEGKTFYV
EPI_ISL_426883	743	SLREVRTIKVFTTVDNINLHTQVVDMSMTYGQQFGPTYLDGADVTKIKPHNSHEGKTFYV
EPI_ISL_426888	743	SLREVRTIKVFTTVDNINLHTQVVDMSMTYGQQFGPTYLDGADVTKIKPHNSHEGKTFYV
EPI_ISL_426900	743	SLREVRTIKVFTTVDNINLHTQVVDMSMTYGQQFGPTYLDGADVTKIKPHNSHEGKTFYV
EPI_ISL_426901	743	SLREVRTIKVFTTVDNINLHTQVVDMSMTYGQQFGPTYLDGADVTKIKPHNSHEGKTFYV
EPI_ISL_426903	743	SLREVRTIKVFTTVDNINLHTQVVDMSMTYGQQFGPTYLDGADVTKIKPHNSHEGKTFYV
EPI_ISL_426904	743	SLREVRTIKVFTTVDNINLHTQVVDMSMTYGQQFGPTYLDGADVTKIKPHNSHEGKTFYV
EPI_ISL_426907	743	SLREVRTIKVFTTVDNINLHTQVVDMSMTYGQQFGPTYLDGADVTKIKPHNSHEGKTFYV
EPI_ISL_426910	743	SLREVRTIKVFTTVDNINLHTQVVDMSMTYGQQFGPTYLDGADVTKIKPHNSHEGKTFYV

ORF1ab	1621	LPNDDTLRVEAFEYYHTTDPSFLGRYMSALNHTKKWKYPQVNGLTSIKWADNNCYLATAL
EPI_ISL_413575	803	LPNDDTLRVEAFEYYHTTDPSFLGRYMSALNHTKKWKYPQVNGLTSIKWADNNCYLATAL
EPI_ISL_413588	803	LPNDDTLRVEAFEYYHTTDPSFLGRYMSALNHTKKWKYPQVNGLTSIKWADNNCYLATAL
EPI_ISL_413589	803	LPNDDTLRVEAFEYYHTTDPSFLGRYMSALNHTKKWKYPQVNGLTSIKWADNNCYLATAL
EPI_ISL_424221	803	LPNDDTLRVEAFEYYHTTDPSFLGRYMSALNHTKKWKYPQVNGLTSIKWADNNCYLATAL
EPI_ISL_424233	803	LPNDDTLRVEAFEYYHTTDPSFLGRYMSALNHTKKWKYPQVNGLTSIKWADNNCYLATAL
EPI_ISL_424236	803	LPNDDTLRVEAFEYYHTTDPSFLGRYMSALNHTKKWKYPQVNGLTSIKWADNNCYLATAL
EPI_ISL_424238	803	LPNDDTLRVEAFEYYHTTDPSFLGRYMSALNHTKKWKYPQVNGLTSIKWADNNCYLATAL
EPI_ISL_424248	803	LPNDDTLRVEAFEYYHTTDPSFLGRYMSALNHTKKWKYPQVNGLTSIKWADNNCYLATAL
EPI_ISL_424254	803	LPNDDTLRVEAFEYYHTTDPSFLGRYMSALNHTKKWKYPQVNGLTSIKWADNNCYLATAL
EPI_ISL_425548	803	LPNDDTLRVEAFEYYHTTDPSFLGRYMSALNHTKKWKYPQVNGLTSIKWADNNCYLATAL
EPI_ISL_425581	803	LPNDDTLRVEAFEYYHTTDPSFLGRYMSALNHTKKWKYPQVNGLTSIKWADNNCYLATAL
EPI_ISL_426881	803	LPNDDTLRVEAFEYYHTTDPSFLGRYMSALNHTKKWKYPQVNGLTSIKWADNNCYLATAL
EPI_ISL_426883	803	LPNDDTLRVEAFEYYHTTDPSFLGRYMSALNHTKKWKYPQVNGLTSIKWADNNCYLATAL
EPI_ISL_426888	803	LPNDDTLRVEAFEYYHTTDPSFLGRYMSALNHTKKWKYPQVNGLTSIKWADNNCYLATAL
EPI_ISL_426900	803	LPNDDTLRVEAFEYYHTTDPSFLGRYMSALNHTKKWKYPQVNGLTSIKWADNNCYLATAL
EPI_ISL_426901	803	LPNDDTLRVEAFEYYHTTDPSFLGRYMSALNHTKKWKYPQVNGLTSIKWADNNCYLATAL
EPI_ISL_426903	803	LPNDDTLRVEAFEYYHTTDPSFLGRYMSALNHTKKWKYPQVNGLTSIKWADNNCYLATAL
EPI_ISL_426904	803	LPNDDTLRVEAFEYYHTTDPSFLGRYMSALNHTKKWKYPQVNGLTSIKWADNNCYLATAL
EPI_ISL_426907	803	LPNDDTLRVEAFEYYHTTDPSFLGRYMSALNHTKKWKYPQVNGLTSIKWADNNCYLATAL
EPI_ISL_426910	803	LPNDDTLRVEAFEYYHTTDPSFLGRYMSALNHTKKWKYPQVNGLTSIKWADNNCYLATAL
ORF1ab	1621	LPNDDTLRVEAFEYYHTTDPSFLGRYMSALNHTKKWKYPQVNGLTSIKWADNNCYLATAL
EPI_ISL_413575	803	LPNDDTLRVEAFEYYHTTDPSFLGRYMSALNHTKKWKYPQVNGLTSIKWADNNCYLATAL
EPI_ISL_413588	803	LPNDDTLRVEAFEYYHTTDPSFLGRYMSALNHTKKWKYPQVNGLTSIKWADNNCYLATAL
EPI_ISL_413589	803	LPNDDTLRVEAFEYYHTTDPSFLGRYMSALNHTKKWKYPQVNGLTSIKWADNNCYLATAL
EPI_ISL_424221	803	LPNDDTLRVEAFEYYHTTDPSFLGRYMSALNHTKKWKYPQVNGLTSIKWADNNCYLATAL
EPI_ISL_424233	803	LPNDDTLRVEAFEYYHTTDPSFLGRYMSALNHTKKWKYPQVNGLTSIKWADNNCYLATAL
EPI_ISL_424236	803	LPNDDTLRVEAFEYYHTTDPSFLGRYMSALNHTKKWKYPQVNGLTSIKWADNNCYLATAL
EPI_ISL_424238	803	LPNDDTLRVEAFEYYHTTDPSFLGRYMSALNHTKKWKYPQVNGLTSIKWADNNCYLATAL
EPI_ISL_424248	803	LPNDDTLRVEAFEYYHTTDPSFLGRYMSALNHTKKWKYPQVNGLTSIKWADNNCYLATAL
EPI_ISL_424254	803	LPNDDTLRVEAFEYYHTTDPSFLGRYMSALNHTKKWKYPQVNGLTSIKWADNNCYLATAL
EPI_ISL_425548	803	LPNDDTLRVEAFEYYHTTDPSFLGRYMSALNHTKKWKYPQVNGLTSIKWADNNCYLATAL
EPI_ISL_425581	803	LPNDDTLRVEAFEYYHTTDPSFLGRYMSALNHTKKWKYPQVNGLTSIKWADNNCYLATAL
EPI_ISL_426881	803	LPNDDTLRVEAFEYYHTTDPSFLGRYMSALNHTKKWKYPQVNGLTSIKWADNNCYLATAL
EPI_ISL_426883	803	LPNDDTLRVEAFEYYHTTDPSFLGRYMSALNHTKKWKYPQVNGLTSIKWADNNCYLATAL
EPI_ISL_426888	803	LPNDDTLRVEAFEYYHTTDPSFLGRYMSALNHTKKWKYPQVNGLTSIKWADNNCYLATAL
EPI_ISL_426900	803	LPNDDTLRVEAFEYYHTTDPSFLGRYMSALNHTKKWKYPQVNGLTSIKWADNNCYLATAL
EPI_ISL_426901	803	LPNDDTLRVEAFEYYHTTDPSFLGRYMSALNHTKKWKYPQVNGLTSIKWADNNCYLATAL
EPI_ISL_426903	803	LPNDDTLRVEAFEYYHTTDPSFLGRYMSALNHTKKWKYPQVNGLTSIKWADNNCYLATAL
EPI_ISL_426904	803	LPNDDTLRVEAFEYYHTTDPSFLGRYMSALNHTKKWKYPQVNGLTSIKWADNNCYLATAL
EPI_ISL_426907	803	LPNDDTLRVEAFEYYHTTDPSFLGRYMSALNHTKKWKYPQVNGLTSIKWADNNCYLATAL
EPI_ISL_426910	803	LPNDDTLRVEAFEYYHTTDPSFLGRYMSALNHTKKWKYPQVNGLTSIKWADNNCYLATAL

```
ORF1ab          1681 LTLQQIELKFNPPALQDAYYRARAGEAANFCALILAYCNKTVGELGDVRETMSYLFQHAN
EPI_ISL_413575   863 LTLQQIELKFNPPALQDAYYRARAGEAANFCALILAYCNKTVGELGDVRETMSYLFQHAN
EPI_ISL_413588   863 LTLQQIELKFNPPALQDAYYRARAGEAANFCALILAYCNKTVGELGDVRETMSYLFQHAN
EPI_ISL_413589   863 LTLQQIELKFNPPALQDAYYRARAGEAANFCALILAYCNKTVGELGDVRETMSYLFQHAN
EPI_ISL_424221   863 LTLQQIELKFNPPALQDAYYRARAGEAANFCALILAYCNKTVGELGDVRETMSYLFQHAN
EPI_ISL_424233   863 LTLQQIELKFNPPALQDAYYRARAGEAANFCALILAYCNKTVGELGDVRETMSYLFQHAN
EPI_ISL_424236   863 LTLQQIELKFNPPALQDAYYRARAGEAANFCALILAYCNKTVGELGDVRETMSYLFQHAN
EPI_ISL_424238   863 LTLQQIELKFNPPALQDAYYRARAGEAANFCALILAYCNKTVGELGDVRETMSYLFQHAN
EPI_ISL_424248   863 LTLQQIELKFNPPALQDAYYRARAGEAANFCALILAYCNKTVGELGDVRETMSYLFQHAN
EPI_ISL_424254   863 LTLQQIELKFNPPALQDAYYRARAGEAANFCALILAYCNKTVGELGDVRETMSYLFQHAN
EPI_ISL_425548   863 LTLQQIELKFNPPALQDAYYRARAGEAANFCALILAYCNKTVGELGDVRETMSYLFQHAN
EPI_ISL_425581   863 LTLQQIELKFNPPALQDAYYRARAGEAANFCALILAYCNKTVGELGDVRETMSYLFQHAN
EPI_ISL_426881   863 LTLQQIELKFNPPALQDAYYRARAGEAANFCALILAYCNKTVGELGDVRETMSYLFQHAN
EPI_ISL_426883   863 LTLQQIELKFNPPALQDAYYRARAGEAANFCALILAYCNKTVGELGDVRETMSYLFQHAN
EPI_ISL_426888   863 LTLQQIELKFNPPALQDAYYRARAGEAANFCALILAYCNKTVGELGDVRETMSYLFQHAN
EPI_ISL_426900   863 LTLQQIELKFNPPALQDAYYRARAGEAANFCALILAYCNKTVGELGDVRETMSYLFQHAN
EPI_ISL_426901   863 LTLQQIELKFNPPALQDAYYRARAGEAANFCALILAYCNKTVGELGDVRETMSYLFQHAN
EPI_ISL_426903   863 LTLQQIELKFNPPALQDAYYRARAGEAANFCALILAYCNKTVGELGDVRETMSYLFQHAN
EPI_ISL_426904   863 LTLQQIELKFNPPALQDAYYRARAGEAANFCALILAYCNKTVGELGDVRETMSYLFQHAN
EPI_ISL_426907   863 LTLQQIELKFNPPALQDAYYRARAGEAANFCALILAYCNKTVGELGDVRETMSYLFQHAN
EPI_ISL_426910   863 LTLQQIELKFNPPALQDAYYRARAGEAANFCALILAYCNKTVGELGDVRETMSYLFQHAN
ORF1ab          1681 LTLQQIELKFNPPALQDAYYRARAGEAANFCALILAYCNKTVGELGDVRETMSYLFQHAN
EPI_ISL_413575   863 LTLQQIELKFNPPALQDAYYRARAGEAANFCALILAYCNKTVGELGDVRETMSYLFQHAN
EPI_ISL_413588   863 LTLQQIELKFNPPALQDAYYRARAGEAANFCALILAYCNKTVGELGDVRETMSYLFQHAN
EPI_ISL_413589   863 LTLQQIELKFNPPALQDAYYRARAGEAANFCALILAYCNKTVGELGDVRETMSYLFQHAN
EPI_ISL_424221   863 LTLQQIELKFNPPALQDAYYRARAGEAANFCALILAYCNKTVGELGDVRETMSYLFQHAN
EPI_ISL_424233   863 LTLQQIELKFNPPALQDAYYRARAGEAANFCALILAYCNKTVGELGDVRETMSYLFQHAN
EPI_ISL_424236   863 LTLQQIELKFNPPALQDAYYRARAGEAANFCALILAYCNKTVGELGDVRETMSYLFQHAN
EPI_ISL_424238   863 LTLQQIELKFNPPALQDAYYRARAGEAANFCALILAYCNKTVGELGDVRETMSYLFQHAN
EPI_ISL_424248   863 LTLQQIELKFNPPALQDAYYRARAGEAANFCALILAYCNKTVGELGDVRETMSYLFQHAN
EPI_ISL_424254   863 LTLQQIELKFNPPALQDAYYRARAGEAANFCALILAYCNKTVGELGDVRETMSYLFQHAN
EPI_ISL_425548   863 LTLQQIELKFNPPALQDAYYRARAGEAANFCALILAYCNKTVGELGDVRETMSYLFQHAN
EPI_ISL_425581   863 LTLQQIELKFNPPALQDAYYRARAGEAANFCALILAYCNKTVGELGDVRETMSYLFQHAN
EPI_ISL_426881   863 LTLQQIELKFNPPALQDAYYRARAGEAANFCALILAYCNKTVGELGDVRETMSYLFQHAN
EPI_ISL_426883   863 LTLQQIELKFNPPALQDAYYRARAGEAANFCALILAYCNKTVGELGDVRETMSYLFQHAN
EPI_ISL_426888   863 LTLQQIELKFNPPALQDAYYRARAGEAANFCALILAYCNKTVGELGDVRETMSYLFQHAN
EPI_ISL_426900   863 LTLQQIELKFNPPALQDAYYRARAGEAANFCALILAYCNKTVGELGDVRETMSYLFQHAN
EPI_ISL_426901   863 LTLQQIELKFNPPALQDAYYRARAGEAANFCALILAYCNKTVGELGDVRETMSYLFQHAN
EPI_ISL_426903   863 LTLQQIELKFNPPALQDAYYRARAGEAANFCALILAYCNKTVGELGDVRETMSYLFQHAN
EPI_ISL_426904   863 LTLQQIELKFNPPALQDAYYRARAGEAANFCALILAYCNKTVGELGDVRETMSYLFQHAN
EPI_ISL_426907   863 LTLQQIELKFNPPALQDAYYRARAGEAANFCALILAYCNKTVGELGDVRETMSYLFQHAN
EPI_ISL_426910   863 LTLQQIELKFNPPALQDAYYRARAGEAANFCALILAYCNKTVGELGDVRETMSYLFQHAN
```

```
ORF1ab          1741 LDSCKRVLNVVCKTCGQQQTTLKGVEAVMYMGTLSYEQFKKGVQIPCTCGKQATKYLVQQ
EPI_ISL_413575   923 LDSCKRVLNVVCKTCGQQQTTLKGVEAVMYMGTLSYEQFKKGVQIPCTCGKQATKYLVQQ
EPI_ISL_413588   923 LDSCKRVLNVVCKTCGQQQTTLKGVEAVMYMGTLSYEQFKKGVQIPCTCGKQATKYLVQQ
EPI_ISL_413589   923 LDSCKRVLNVVCKTCGQQQTTLKGVEAVMYMGTLSYEQFKKGVQIPCTCGKQATKYLVQQ
EPI_ISL_424221   923 LDSCKRVLNVVCKTCGQQQTTLKGVEAVMYMGTLSYEQFKKGVQIPCTCGKQATKYLVQQ
EPI_ISL_424233   923 LDSCKRVLNVVCKTCGQQQTTLKGVEAVMYMGTLSYEQFKKGVQIPCTCGKQATKYLVQQ
EPI_ISL_424236   923 LDSCKRVLNVVCKTCGQQQTTLKGVEAVMYMGTLSYEQFKKGVQIPCTCGKQATKYLVQQ
EPI_ISL_424238   923 LDSCKRVLNVVCKTCGQQQTTLKGVEAVMYMGTLSYEQFKKGVQIPCTCGKQATKYLVQQ
EPI_ISL_424248   923 LDSCKRVLNVVCKTCGQQQTTLKGVEAVMYMGTLSYEQFKKGVQIPCTCGKQATKYLVQQ
EPI_ISL_424254   923 LDSCKRVLNVVCKTCGQQQTTLKGVEAVMYMGTLSYEQFKKGVQIPCTCGKQATKYLVQQ
EPI_ISL_425548   923 LDSCKRVLNVVCKTCGQQQTTLKGVEAVMYMGTLSYEQFKKGVQIPCTCGKQATKYLVQQ
EPI_ISL_425581   923 LDSCKRVLNVVCKTCGQQQTTLKGVEAVMYMGTLSYEQFKKGVQIPCTCGKQATKYLVQQ
EPI_ISL_426881   923 LDSCKRVLNVVCKTCGQQQTTLKGVEAVMYMGTLSYEQFKKGVQIPCTCGKQATKYLVQQ
EPI_ISL_426883   923 LDSCKRVLNVVCKTCGQQQTTLKGVEAVMYMGTLSYEQFKKGVQIPCTCGKQATKYLVQQ
EPI_ISL_426888   923 LDSCKRVLNVVCKTCGQQQTTLKGVEAVMYMGTLSYEQFKKGVQIPCTCGKQATKYLVQQ
EPI_ISL_426900   923 LDSCKRVLNVVCKTCGQQQTTLKGVEAVMYMGTLSYEQFKKGVQIPCTCGKQATKYLVQQ
EPI_ISL_426901   923 LDSCKRVLNVVCKTCGQQQTTLKGVEAVMYMGTLSYEQFKKGVQIPCTCGKQATKYLVQQ
EPI_ISL_426903   923 LDSCKRVLNVVCKTCGQQQTTLKGVEAVMYMGTLSYEQFKKGVQIPCTCGKQATKYLVQQ
EPI_ISL_426904   923 LDSCKRVLNVVCKTCGQQQTTLKGVEAVMYMGTLSYEQFKKGVQIPCTCGKQATKYLVQQ
EPI_ISL_426907   923 LDSCKRVLNVVCKTCGQQQTTLKGVEAVMYMGTLSYEQFKKGVQIPCTCGKQATKYLVQQ
EPI_ISL_426910   923 LDSCKRVLNVVCKTCGQQQTTLKGVEAVMYMGTLSYEQFKKGVQIPCTCGKQATKYLVQQ
ORF1ab          1741 LDSCKRVLNVVCKTCGQQQTTLKGVEAVMYMGTLSYEQFKKGVQIPCTCGKQATKYLVQQ
EPI_ISL_413575   923 LDSCKRVLNVVCKTCGQQQTTLKGVEAVMYMGTLSYEQFKKGVQIPCTCGKQATKYLVQQ
EPI_ISL_413588   923 LDSCKRVLNVVCKTCGQQQTTLKGVEAVMYMGTLSYEQFKKGVQIPCTCGKQATKYLVQQ
EPI_ISL_413589   923 LDSCKRVLNVVCKTCGQQQTTLKGVEAVMYMGTLSYEQFKKGVQIPCTCGKQATKYLVQQ
EPI_ISL_424221   923 LDSCKRVLNVVCKTCGQQQTTLKGVEAVMYMGTLSYEQFKKGVQIPCTCGKQATKYLVQQ
EPI_ISL_424233   923 LDSCKRVLNVVCKTCGQQQTTLKGVEAVMYMGTLSYEQFKKGVQIPCTCGKQATKYLVQQ
EPI_ISL_424236   923 LDSCKRVLNVVCKTCGQQQTTLKGVEAVMYMGTLSYEQFKKGVQIPCTCGKQATKYLVQQ
EPI_ISL_424238   923 LDSCKRVLNVVCKTCGQQQTTLKGVEAVMYMGTLSYEQFKKGVQIPCTCGKQATKYLVQQ
EPI_ISL_424248   923 LDSCKRVLNVVCKTCGQQQTTLKGVEAVMYMGTLSYEQFKKGVQIPCTCGKQATKYLVQQ
EPI_ISL_424254   923 LDSCKRVLNVVCKTCGQQQTTLKGVEAVMYMGTLSYEQFKKGVQIPCTCGKQATKYLVQQ
EPI_ISL_425548   923 LDSCKRVLNVVCKTCGQQQTTLKGVEAVMYMGTLSYEQFKKGVQIPCTCGKQATKYLVQQ
EPI_ISL_425581   923 LDSCKRVLNVVCKTCGQQQTTLKGVEAVMYMGTLSYEQFKKGVQIPCTCGKQATKYLVQQ
EPI_ISL_426881   923 LDSCKRVLNVVCKTCGQQQTTLKGVEAVMYMGTLSYEQFKKGVQIPCTCGKQATKYLVQQ
EPI_ISL_426883   923 LDSCKRVLNVVCKTCGQQQTTLKGVEAVMYMGTLSYEQFKKGVQIPCTCGKQATKYLVQQ
EPI_ISL_426888   923 LDSCKRVLNVVCKTCGQQQTTLKGVEAVMYMGTLSYEQFKKGVQIPCTCGKQATKYLVQQ
EPI_ISL_426900   923 LDSCKRVLNVVCKTCGQQQTTLKGVEAVMYMGTLSYEQFKKGVQIPCTCGKQATKYLVQQ
EPI_ISL_426901   923 LDSCKRVLNVVCKTCGQQQTTLKGVEAVMYMGTLSYEQFKKGVQIPCTCGKQATKYLVQQ
EPI_ISL_426903   923 LDSCKRVLNVVCKTCGQQQTTLKGVEAVMYMGTLSYEQFKKGVQIPCTCGKQATKYLVQQ
EPI_ISL_426904   923 LDSCKRVLNVVCKTCGQQQTTLKGVEAVMYMGTLSYEQFKKGVQIPCTCGKQATKYLVQQ
EPI_ISL_426907   923 LDSCKRVLNVVCKTCGQQQTTLKGVEAVMYMGTLSYEQFKKGVQIPCTCGKQATKYLVQQ
EPI_ISL_426910   923 LDSCKRVLNVVCKTCGQQQTTLKGVEAVMYMGTLSYEQFKKGVQIPCTCGKQATKYLVQQ
```

```
ORF1ab         1801 ESPFVMMSAPPAQYELKHGTFTCASEYTGNYQCGHYKHITSKETLYCIDGALLTKSSEYK
EPI_ISL_413575  983 ESPFVMMSAPPAQYELKHGTFTCASEYTGNYQCGHYKHITSKETLYCIDGALLTKSSEYK
EPI_ISL_413588  983 ESPFVMMSAPPAQYELKHGTFTCASEYTGNYQCGHYKHITSKETLYCIDGALLTKSSEYK
EPI_ISL_413589  983 ESPFVMMSAPPAQYELKHGTFTCASEYTGNYQCGHYKHITSKETLYCIDGALLTKSSEYK
EPI_ISL_424221  983 ESPFVMMSAPPAQYELKHGTFTCASEYTGNYQCGHYKHITSKETLYCIDGALLTKSSEYK
EPI_ISL_424233  983 ESPFVMMSAPPAQYELKHGTFTCASEYTGNYQCGHYKHITSKETLYCIDGALLTKSSEYK
EPI_ISL_424236  983 ESPFVMMSAPPAQYELKHGTFTCASEYTGNYQCGHYKHITSKETLYCIDGALLTKSSEYK
EPI_ISL_424238  983 ESPFVMMSAPPAQYELKHGTFTCASEYTGNYQCGHYKHITSKETLYCIDGALLTKSSEYK
EPI_ISL_424248  983 ESPFVMMSAPPAQYELKHGTFTCASEYTGNYQCGHYKHITSKETLYCIDGALLTKSSEYK
EPI_ISL_424254  983 ESPFVMMSAPPAQYELKHGTFTCASEYTGNYQCGHYKHITSKETLYCIDGALLTKSSEYK
EPI_ISL_425548  983 ESPFVMMSAPPAQYELKHGTFTCASEYTGNYQCGHYKHITSKETLYCIDGALLTKSSEYK
EPI_ISL_425581  983 ESPFVMMSAPPAQYELKHGTFTCASEYTGNYQCGHYKHITSKETLYCIDGALLTKSSEYK
EPI_ISL_426881  983 ESPFVMMSAPPAQYELKHGTFTCASEYTGNYQCGHYKHITSKETLYCIDGALLTKSSEYK
EPI_ISL_426883  983 ESPFVMMSAPPAQYELKHGTFTCASEYTGNYQCGHYKHITSKETLYCIDGALLTKSSEYK
EPI_ISL_426888  983 ESPFVMMSAPPAQYELKHGTFTCASEYTGNYQCGHYKHITSKETLYCIDGALLTKSSEYK
EPI_ISL_426900  983 ESPFVMMSAPPAQYELKHGTFTCASEYTGNYQCGHYKHITSKETLYCIDGALLTKSSEYK
EPI_ISL_426901  983 ESPFVMMSAPPAQYELKHGTFTCASEYTGNYQCGHYKHITSKETLYCIDGALLTKSSEYK
EPI_ISL_426903  983 ESPFVMMSAPPAQYELKHGTFTCASEYTGNYQCGHYKHITSKETLYCIDGALLTKSSEYK
EPI_ISL_426904  983 ESPFVMMSAPPAQYELKHGTFTCASEYTGNYQCGHYKHITSKETLYCIDGALLTKSSEYK
EPI_ISL_426907  983 ESPFVMMSAPPAQYELKHGTFTCASEYTGNYQCGHYKHITSKETLYCIDGALLTKSSEYK
EPI_ISL_426910  983 ESPFVMMSAPPAQYELKHGTFTCASEYTGNYQCGHYKHITSKETLYCIDGALLTKSSEYK
ORF1ab         1801 ESPFVMMSAPPAQYELKHGTFTCASEYTGNYQCGHYKHITSKETLYCIDGALLTKSSEYK
EPI_ISL_413575  983 ESPFVMMSAPPAQYELKHGTFTCASEYTGNYQCGHYKHITSKETLYCIDGALLTKSSEYK
EPI_ISL_413588  983 ESPFVMMSAPPAQYELKHGTFTCASEYTGNYQCGHYKHITSKETLYCIDGALLTKSSEYK
EPI_ISL_413589  983 ESPFVMMSAPPAQYELKHGTFTCASEYTGNYQCGHYKHITSKETLYCIDGALLTKSSEYK
EPI_ISL_424221  983 ESPFVMMSAPPAQYELKHGTFTCASEYTGNYQCGHYKHITSKETLYCIDGALLTKSSEYK
EPI_ISL_424233  983 ESPFVMMSAPPAQYELKHGTFTCASEYTGNYQCGHYKHITSKETLYCIDGALLTKSSEYK
EPI_ISL_424236  983 ESPFVMMSAPPAQYELKHGTFTCASEYTGNYQCGHYKHITSKETLYCIDGALLTKSSEYK
EPI_ISL_424238  983 ESPFVMMSAPPAQYELKHGTFTCASEYTGNYQCGHYKHITSKETLYCIDGALLTKSSEYK
EPI_ISL_424248  983 ESPFVMMSAPPAQYELKHGTFTCASEYTGNYQCGHYKHITSKETLYCIDGALLTKSSEYK
EPI_ISL_424254  983 ESPFVMMSAPPAQYELKHGTFTCASEYTGNYQCGHYKHITSKETLYCIDGALLTKSSEYK
EPI_ISL_425548  983 ESPFVMMSAPPAQYELKHGTFTCASEYTGNYQCGHYKHITSKETLYCIDGALLTKSSEYK
EPI_ISL_425581  983 ESPFVMMSAPPAQYELKHGTFTCASEYTGNYQCGHYKHITSKETLYCIDGALLTKSSEYK
EPI_ISL_426881  983 ESPFVMMSAPPAQYELKHGTFTCASEYTGNYQCGHYKHITSKETLYCIDGALLTKSSEYK
EPI_ISL_426883  983 ESPFVMMSAPPAQYELKHGTFTCASEYTGNYQCGHYKHITSKETLYCIDGALLTKSSEYK
EPI_ISL_426888  983 ESPFVMMSAPPAQYELKHGTFTCASEYTGNYQCGHYKHITSKETLYCIDGALLTKSSEYK
EPI_ISL_426900  983 ESPFVMMSAPPAQYELKHGTFTCASEYTGNYQCGHYKHITSKETLYCIDGALLTKSSEYK
EPI_ISL_426901  983 ESPFVMMSAPPAQYELKHGTFTCASEYTGNYQCGHYKHITSKETLYCIDGALLTKSSEYK
EPI_ISL_426903  983 ESPFVMMSAPPAQYELKHGTFTCASEYTGNYQCGHYKHITSKETLYCIDGALLTKSSEYK
EPI_ISL_426904  983 ESPFVMMSAPPAQYELKHGTFTCASEYTGNYQCGHYKHITSKETLYCIDGALLTKSSEYK
EPI_ISL_426907  983 ESPFVMMSAPPAQYELKHGTFTCASEYTGNYQCGHYKHITSKETLYCIDGALLTKSSEYK
EPI_ISL_426910  983 ESPFVMMSAPPAQYELKHGTFTCASEYTGNYQCGHYKHITSKETLYCIDGALLTKSSEYK
```

```
ORF1ab         1861 GPITDVFYKENSYTTTIKPVTYKLDGVVCTEIDPKLDNYYKKDNSYFTEQPIDLVPNQPY
EPI_ISL_413575 1043 GPITDVFYKENSYTTTIKPVTYKLDGVVCTEIDPKLDNYYKKDNSYFTEQPIDLVPNQPY
EPI_ISL_413588 1043 GPITDVFYKENSYTTTIKPVTYKLDGVVCTEIDPKLDNYYKKDNSYFTEQPIDLVPNQPY
EPI_ISL_413589 1043 GPITDVFYKENSYTTTIKPVTYKLDGVVCTEIDPKLDNYYKKDNSYFTEQPIDLVPNQPY
EPI_ISL_424221 1043 GPITDVFYKENSYTTTIKPVTYKLDGVVCTEIDPKLDNYYKKDNSYFTEQPIDLVPNQPY
EPI_ISL_424233 1043 GPITDVFYKENSYTTTIKPVTYKLDGVVCTEIDPKLDNYYKKDNSYFTEQPIDLVPNQPY
EPI_ISL_424236 1043 GPITDVFYKENSYTTTIKPVTYKLDGVVCTEIDPKLDNYYKKDNSYFTEQPIDLVPNQPY
EPI_ISL_424238 1043 GPITDVFYKENSYTTTIKPVTYKLDGVVCTEIDPKLDNYYKKDNSYFTEQPIDLVPNQPY
EPI_ISL_424248 1043 GPITDVFYKENSYTTTIKPVTYKLDGVVCTEIDPKLDNYYKKDNSYFTEQPIDLVPNQPY
EPI_ISL_424254 1043 GPITDVFYKENSYTTTIKPVTYKLDGVVCTEIDPKLDNYYKKDNSYFTEQPIDLVPNQPY
EPI_ISL_425548 1043 GPITDVFYKENSYTTTIKPVTYKLDGVVCTEIDPKLDNYYKKDNSYFTEQPIDLVPNQPY
EPI_ISL_425581 1043 GPITDVFYKENSYTTTIKPVTYKLDGVVCTEIDPKLDNYYKKDNSYFTEQPIDLVPNQPY
EPI_ISL_426881 1043 GPITDVFYKENSYTTTIKPVTYKLDGVVCTEIDPKLDNYYKKDNSYFTEQPIDLVPNQPY
EPI_ISL_426883 1043 GPITDVFYKENSYTTTIKPVTYKLDGVVCTEIDPKLDNYYKKDNSYFTEQPIDLVPNQPY
EPI_ISL_426888 1043 GPITDVFYKENSYTTTIKPVTYKLDGVVCTEIDPKLDNYYKKDNSYFTEQPIDLVPNQPY
EPI_ISL_426900 1043 GPITDVFYKENSYTTTIKPVTYKLDGVVCTEIDPKLDNYYKKDNSYFTEQPIDLVPNQPY
EPI_ISL_426901 1043 GPITDVFYKENSYTTTIKPVTYKLDGVVCTEIDPKLDNYYKKDNSYFTEQPIDLVPNQPY
EPI_ISL_426903 1043 GPITDVFYKENSYTTTIKPVTYKLDGVVCTEIDPKLDNYYKKDNSYFTEQPIDLVPNQPY
EPI_ISL_426904 1043 GPITDVFYKENSYTTTIKPVTYKLDGVVCTEIDPKLDNYYKKDNSYFTEQPIDLVPNQPY
EPI_ISL_426907 1043 GPITDVFYKENSYTTTIKPVTYKLDGVVCTEIDPKLDNYYKKDNSYFTEQPIDLVPNQPY
EPI_ISL_426910 1043 GPITDVFYKENSYTTTIKPVTYKLDGVVCTEIDPKLDNYYKKDNSYFTEQPIDLVPNQPY
ORF1ab         1861 GPITDVFYKENSYTTTIKPVTYKLDGVVCTEIDPKLDNYYKKDNSYFTEQPIDLVPNQPY
EPI_ISL_413575 1043 GPITDVFYKENSYTTTIKPVTYKLDGVVCTEIDPKLDNYYKKDNSYFTEQPIDLVPNQPY
EPI_ISL_413588 1043 GPITDVFYKENSYTTTIKPVTYKLDGVVCTEIDPKLDNYYKKDNSYFTEQPIDLVPNQPY
EPI_ISL_413589 1043 GPITDVFYKENSYTTTIKPVTYKLDGVVCTEIDPKLDNYYKKDNSYFTEQPIDLVPNQPY
EPI_ISL_424221 1043 GPITDVFYKENSYTTTIKPVTYKLDGVVCTEIDPKLDNYYKKDNSYFTEQPIDLVPNQPY
EPI_ISL_424233 1043 GPITDVFYKENSYTTTIKPVTYKLDGVVCTEIDPKLDNYYKKDNSYFTEQPIDLVPNQPY
EPI_ISL_424236 1043 GPITDVFYKENSYTTTIKPVTYKLDGVVCTEIDPKLDNYYKKDNSYFTEQPIDLVPNQPY
EPI_ISL_424238 1043 GPITDVFYKENSYTTTIKPVTYKLDGVVCTEIDPKLDNYYKKDNSYFTEQPIDLVPNQPY
EPI_ISL_424248 1043 GPITDVFYKENSYTTTIKPVTYKLDGVVCTEIDPKLDNYYKKDNSYFTEQPIDLVPNQPY
EPI_ISL_424254 1043 GPITDVFYKENSYTTTIKPVTYKLDGVVCTEIDPKLDNYYKKDNSYFTEQPIDLVPNQPY
EPI_ISL_425548 1043 GPITDVFYKENSYTTTIKPVTYKLDGVVCTEIDPKLDNYYKKDNSYFTEQPIDLVPNQPY
EPI_ISL_425581 1043 GPITDVFYKENSYTTTIKPVTYKLDGVVCTEIDPKLDNYYKKDNSYFTEQPIDLVPNQPY
EPI_ISL_426881 1043 GPITDVFYKENSYTTTIKPVTYKLDGVVCTEIDPKLDNYYKKDNSYFTEQPIDLVPNQPY
EPI_ISL_426883 1043 GPITDVFYKENSYTTTIKPVTYKLDGVVCTEIDPKLDNYYKKDNSYFTEQPIDLVPNQPY
EPI_ISL_426888 1043 GPITDVFYKENSYTTTIKPVTYKLDGVVCTEIDPKLDNYYKKDNSYFTEQPIDLVPNQPY
EPI_ISL_426900 1043 GPITDVFYKENSYTTTIKPVTYKLDGVVCTEIDPKLDNYYKKDNSYFTEQPIDLVPNQPY
EPI_ISL_426901 1043 GPITDVFYKENSYTTTIKPVTYKLDGVVCTEIDPKLDNYYKKDNSYFTEQPIDLVPNQPY
EPI_ISL_426903 1043 GPITDVFYKENSYTTTIKPVTYKLDGVVCTEIDPKLDNYYKKDNSYFTEQPIDLVPNQPY
EPI_ISL_426904 1043 GPITDVFYKENSYTTTIKPVTYKLDGVVCTEIDPKLDNYYKKDNSYFTEQPIDLVPNQPY
EPI_ISL_426907 1043 GPITDVFYKENSYTTTIKPVTYKLDGVVCTEIDPKLDNYYKKDNSYFTEQPIDLVPNQPY
EPI_ISL_426910 1043 GPITDVFYKENSYTTTIKPVTYKLDGVVCTEIDPKLDNYYKKDNSYFTEQPIDLVPNQPY
```

```
ORF1ab         1921 PNASFDNFKFVCDNIKFADDLNQLTGYKKPASRELKVTFFPDLNGDVVAIDYKHYTPSFK
EPI_ISL_413575 1103 PNASFDNFKFVCDNIKFADDLNQLTGYKKPASRELKVTFFPDLNGDVVAIDYKHYTPSFK
EPI_ISL_413588 1103 PNASFDNFKFVCDNIKFADDLNQLTGYKKPASRELKVTFFPDLNGDVVAIDYKHYTPSFK
EPI_ISL_413589 1103 PNASFDNFKFVCDNIKFADDLNQLTGYKKPASRELKVTFFPDLNGDVVAIDYKHYTPSFK
EPI_ISL_424221 1103 PNASFDNFKFVCDNIKFADDLNQLTGYKKPASRELKVTFFPDLNGDVVAIDYKHYTPSFK
EPI_ISL_424233 1103 PNASFDNFKFVCDNIKFADDLNQLTGYKKPASRELKVTFFPDLNGDVVAIDYKHYTPSFK
EPI_ISL_424236 1103 PNASFDNFKFVCDNIKFADDLNQLTGYKKPASRELKVTFFPDLNGDVVAIDYKHYTPSFK
EPI_ISL_424238 1103 PNASFDNFKFVCDNIKFADDLNQLTGYKKPASRELKVTFFPDLNGDVVAIDYKHYTPSFK
EPI_ISL_424248 1103 PNASFDNFKFVCDNIKFADDLNQLTGYKKPASRELKVTFFPDLNGDVVAIDYKHYTPSFK
EPI_ISL_424254 1103 PNASFDNFKFVCDNIKFADDLNQLTGYKKPASRELKVTFFPDLNGDVVAIDYKHYTPSFK
EPI_ISL_425548 1103 PNASFDNFKFVCDNIKFADDLNQLTGYKKPASRELKVTFFPDLNGDVVAIDYKHYTPSFK
EPI_ISL_425581 1103 PNASFDNFKFVCDNIKFADDLNQLTGYKKPASRELKVTFFPDLNGDVVAIDYKHYTPSFK
EPI_ISL_426881 1103 PNASFDNFKFVCDNIKFADDLNQLTGYKKPASRELKVTFFPDLNGDVVAIDYKHYTPSFK
EPI_ISL_426883 1103 PNASFDNFKFVCDNIKFADDLNQLTGYKKPASRELKVTFFPDLNGDVVAIDYKHYTPSFK
EPI_ISL_426888 1103 PNASFDNFKFVCDNIKFADDLNQLTGYKKPASRELKVTFFPDLNGDVVAIDYKHYTPSFK
EPI_ISL_426900 1103 PNASFDNFKFVCDNIKFADDLNQLTGYKKPASRELKVTFFPDLNGDVVAIDYKHYTPSFK
EPI_ISL_426901 1103 PNASFDNFKFVCDNIKFADDLNQLTGYKKPASRELKVTFFPDLNGDVVAIDYKHYTPSFK
EPI_ISL_426903 1103 PNASFDNFKFVCDNIKFADDLNQLTGYKKPASRELKVTFFPDLNGDVVAIDYKHYTPSFK
EPI_ISL_426904 1103 PNASFDNFKFVCDNIKFADDLNQLTGYKKPASRELKVTFFPDLNGDVVAIDYKHYTPSFK
EPI_ISL_426907 1103 PNASFDNFKFVCDNIKFADDLNQLTGYKKPASRELKVTFFPDLNGDVVAIDYKHYTPSFK
EPI_ISL_426910 1103 PNASFDNFKFVCDNIKFADDLNQLTGYKKPASRELKVTFFPDLNGDVVAIDYKHYTPSFK
ORF1ab         1921 PNASFDNFKFVCDNIKFADDLNQLTGYKKPASRELKVTFFPDLNGDVVAIDYKHYTPSFK
EPI_ISL_413575 1103 PNASFDNFKFVCDNIKFADDLNQLTGYKKPASRELKVTFFPDLNGDVVAIDYKHYTPSFK
EPI_ISL_413588 1103 PNASFDNFKFVCDNIKFADDLNQLTGYKKPASRELKVTFFPDLNGDVVAIDYKHYTPSFK
EPI_ISL_413589 1103 PNASFDNFKFVCDNIKFADDLNQLTGYKKPASRELKVTFFPDLNGDVVAIDYKHYTPSFK
EPI_ISL_424221 1103 PNASFDNFKFVCDNIKFADDLNQLTGYKKPASRELKVTFFPDLNGDVVAIDYKHYTPSFK
EPI_ISL_424233 1103 PNASFDNFKFVCDNIKFADDLNQLTGYKKPASRELKVTFFPDLNGDVVAIDYKHYTPSFK
EPI_ISL_424236 1103 PNASFDNFKFVCDNIKFADDLNQLTGYKKPASRELKVTFFPDLNGDVVAIDYKHYTPSFK
EPI_ISL_424238 1103 PNASFDNFKFVCDNIKFADDLNQLTGYKKPASRELKVTFFPDLNGDVVAIDYKHYTPSFK
EPI_ISL_424248 1103 PNASFDNFKFVCDNIKFADDLNQLTGYKKPASRELKVTFFPDLNGDVVAIDYKHYTPSFK
EPI_ISL_424254 1103 PNASFDNFKFVCDNIKFADDLNQLTGYKKPASRELKVTFFPDLNGDVVAIDYKHYTPSFK
EPI_ISL_425548 1103 PNASFDNFKFVCDNIKFADDLNQLTGYKKPASRELKVTFFPDLNGDVVAIDYKHYTPSFK
EPI_ISL_425581 1103 PNASFDNFKFVCDNIKFADDLNQLTGYKKPASRELKVTFFPDLNGDVVAIDYKHYTPSFK
EPI_ISL_426881 1103 PNASFDNFKFVCDNIKFADDLNQLTGYKKPASRELKVTFFPDLNGDVVAIDYKHYTPSFK
EPI_ISL_426883 1103 PNASFDNFKFVCDNIKFADDLNQLTGYKKPASRELKVTFFPDLNGDVVAIDYKHYTPSFK
EPI_ISL_426888 1103 PNASFDNFKFVCDNIKFADDLNQLTGYKKPASRELKVTFFPDLNGDVVAIDYKHYTPSFK
EPI_ISL_426900 1103 PNASFDNFKFVCDNIKFADDLNQLTGYKKPASRELKVTFFPDLNGDVVAIDYKHYTPSFK
EPI_ISL_426901 1103 PNASFDNFKFVCDNIKFADDLNQLTGYKKPASRELKVTFFPDLNGDVVAIDYKHYTPSFK
EPI_ISL_426903 1103 PNASFDNFKFVCDNIKFADDLNQLTGYKKPASRELKVTFFPDLNGDVVAIDYKHYTPSFK
EPI_ISL_426904 1103 PNASFDNFKFVCDNIKFADDLNQLTGYKKPASRELKVTFFPDLNGDVVAIDYKHYTPSFK
EPI_ISL_426907 1103 PNASFDNFKFVCDNIKFADDLNQLTGYKKPASRELKVTFFPDLNGDVVAIDYKHYTPSFK
EPI_ISL_426910 1103 PNASFDNFKFVCDNIKFADDLNQLTGYKKPASRELKVTFFPDLNGDVVAIDYKHYTPSFK
```

```
ORF1ab         1981 KGAKLLHKPIVWHVNNATNKATYKPNTWCIRCLWSTKPVETSNSFDVLKSEDAQGMDNLA
EPI_ISL_413575 1163 KGAKLLHKPIVWHVNNATNKATYKPNTWCIRCLWSTKPVETSNSFDVLKSEDAQGMDNLA
EPI_ISL_413588 1163 KGAKLLHKPIVWHVNNATNKATYKPNTWCIRCLWSTKPVETSNSFDVLKSEDAQGMDNLA
EPI_ISL_413589 1163 KGAKLLHKPIVWHVNNATNKATYKPNTWCIRCLWSTKPVETSNSFDVLKSEDAQGMDNLA
EPI_ISL_424221 1163 KGAKLLHKPIVWHVNNATNKATYKPNTWCIRCLWSTKPVETSNSFDVLKSEDAQGMDNLA
EPI_ISL_424233 1163 KGAKLLHKPIVWHVNNATNKATYKPNTWCIRCLWSTKPVETSNSFDVLKSEDAQGMDNLA
EPI_ISL_424236 1163 KGAKLLHKPIVWHVNNATNKATYKPNTWCIRCLWSTKPVETSNSFDVLKSEDAQGMDNLA
EPI_ISL_424238 1163 KGAKLLHKPIVWHVNNATNKATYKPNTWCIRCLWSTKPVETSNSFDVLKSEDAQGMDNLA
EPI_ISL_424248 1163 KGAKLLHKPIVWHVNNATNKATYKPNTWCIRCLWSTKPVETSNSFDVLKSEDAQGMDNLA
EPI_ISL_424254 1163 KGAKLLHKPIVWHVNNATNKATYKPNTWCIRCLWSTKPVETSNSFDVLKSEDAQGMDNLA
EPI_ISL_425548 1163 KGAKLLHKPIVWHVNNATNKATYKPNTWCIRCLWSTKPVETSNSFDVLKSEDAQGMDNLA
EPI_ISL_425581 1163 KGAKLLHKPIVWHVNNATNKATYKPNTWCIRCLWSTKPVETSNSFDVLKSEDAQGMDNLA
EPI_ISL_426881 1163 KGAKLLHKPIVWHVNNATNKATYKPNTWCIRCLWSTKPVETSNSFDVLKSEDAQGMDNLA
EPI_ISL_426883 1163 KGAKLLHKPIVWHVNNATNKATYKPNTWCIRCLWSTKPVETSNSFDVLKSEDAQGMDNLA
EPI_ISL_426888 1163 KGAKLLHKPIVWHVNNATNKATYKPNTWCIRCLWSTKPVETSNSFDVLKSEDAQGMDNLA
EPI_ISL_426900 1163 KGAKLLHKPIVWHVNNATNKATYKPNTWCIRCLWSTKPVETSNSFDVLKSEDAQGMDNLA
EPI_ISL_426901 1163 KGAKLLHKPIVWHVNNATNKATYKPNTWCIRCLWSTKPVETSNSFDVLKSEDAQGMDNLA
EPI_ISL_426903 1163 KGAKLLHKPIVWHVNNATNKATYKPNTWCIRCLWSTKPVETSNSFDVLKSEDAQGMDNLA
EPI_ISL_426904 1163 KGAKLLHKPIVWHVNNATNKATYKPNTWCIRCLWSTKPVETSNSFDVLKSEDAQGMDNLA
EPI_ISL_426907 1163 KGAKLLHKPIVWHVNNATNKATYKPNTWCIRCLWSTKPVETSNSFDVLKSEDAQGMDNLA
EPI_ISL_426910 1163 KGAKLLHKPIVWHVNNATNKATYKPNTWCIRCLWSTKPVETSNSFDVLKSEDAQGMDNLA
```


```
ORF1ab         1981 KGAKLLHKPIVWHVNNATNKATYKPNTWCIRCLWSTKPVETSNSFDVLKSEDAQGMDNLA
EPI_ISL_413575 1163 KGAKLLHKPIVWHVNNATNKATYKPNTWCIRCLWSTKPVETSNSFDVLKSEDAQGMDNLA
EPI_ISL_413588 1163 KGAKLLHKPIVWHVNNATNKATYKPNTWCIRCLWSTKPVETSNSFDVLKSEDAQGMDNLA
EPI_ISL_413589 1163 KGAKLLHKPIVWHVNNATNKATYKPNTWCIRCLWSTKPVETSNSFDVLKSEDAQGMDNLA
EPI_ISL_424221 1163 KGAKLLHKPIVWHVNNATNKATYKPNTWCIRCLWSTKPVETSNSFDVLKSEDAQGMDNLA
EPI_ISL_424233 1163 KGAKLLHKPIVWHVNNATNKATYKPNTWCIRCLWSTKPVETSNSFDVLKSEDAQGMDNLA
EPI_ISL_424236 1163 KGAKLLHKPIVWHVNNATNKATYKPNTWCIRCLWSTKPVETSNSFDVLKSEDAQGMDNLA
EPI_ISL_424238 1163 KGAKLLHKPIVWHVNNATNKATYKPNTWCIRCLWSTKPVETSNSFDVLKSEDAQGMDNLA
EPI_ISL_424248 1163 KGAKLLHKPIVWHVNNATNKATYKPNTWCIRCLWSTKPVETSNSFDVLKSEDAQGMDNLA
EPI_ISL_424254 1163 KGAKLLHKPIVWHVNNATNKATYKPNTWCIRCLWSTKPVETSNSFDVLKSEDAQGMDNLA
EPI_ISL_425548 1163 KGAKLLHKPIVWHVNNATNKATYKPNTWCIRCLWSTKPVETSNSFDVLKSEDAQGMDNLA
EPI_ISL_425581 1163 KGAKLLHKPIVWHVNNATNKATYKPNTWCIRCLWSTKPVETSNSFDVLKSEDAQGMDNLA
EPI_ISL_426881 1163 KGAKLLHKPIVWHVNNATNKATYKPNTWCIRCLWSTKPVETSNSFDVLKSEDAQGMDNLA
EPI_ISL_426883 1163 KGAKLLHKPIVWHVNNATNKATYKPNTWCIRCLWSTKPVETSNSFDVLKSEDAQGMDNLA
EPI_ISL_426888 1163 KGAKLLHKPIVWHVNNATNKATYKPNTWCIRCLWSTKPVETSNSFDVLKSEDAQGMDNLA
EPI_ISL_426900 1163 KGAKLLHKPIVWHVNNATNKATYKPNTWCIRCLWSTKPVETSNSFDVLKSEDAQGMDNLA
EPI_ISL_426901 1163 KGAKLLHKPIVWHVNNATNKATYKPNTWCIRCLWSTKPVETSNSFDVLKSEDAQGMDNLA
EPI_ISL_426903 1163 KGAKLLHKPIVWHVNNATNKATYKPNTWCIRCLWSTKPVETSNSFDVLKSEDAQGMDNLA
EPI_ISL_426904 1163 KGAKLLHKPIVWHVNNATNKATYKPNTWCIRCLWSTKPVETSNSFDVLKSEDAQGMDNLA
EPI_ISL_426907 1163 KGAKLLHKPIVWHVNNATNKATYKPNTWCIRCLWSTKPVETSNSFDVLKSEDAQGMDNLA
EPI_ISL_426910 1163 KGAKLLHKPIVWHVNNATNKATYKPNTWCIRCLWSTKPVETSNSFDVLKSEDAQGMDNLA
```

```
ORF1ab         2041 CEDLKPVSEEVVENPTIQKDVLECNVKTTEVVGDIILKPANNSLKITEEVGHTDLMAAYV
EPI_ISL_413575 1223 CEDLKPVSEEVVENPTIQKDVLECNVKTTEVVGDIILKPANNSLKITEEVGHTDLMAAYV
EPI_ISL_413588 1223 CEDLKPVSEEVVENPTIQKDVLECNVKTTEVVGDIILKPANNSLKITEEVGHTDLMAAYV
EPI_ISL_413589 1223 CEDLKPVSEEVVENPTIQKDVLECNVKTTEVVGDIILKPANNSLKITEEVGHTDLMAAYV
EPI_ISL_424221 1223 CEDLKPVSEEVVENPTIQKDVLECNVKTTEVVGDIILKPANNSLKITEEVGHTDLMAAYV
EPI_ISL_424233 1223 CEDLKPVSEEVVENPTIQKDVLECNVKTTEVVGDIILKPANNSLKITEEVGHTDLMAAYV
EPI_ISL_424236 1223 CEDLKPVSEEVVENPTIQKDVLECNVKTTEVVGDIILKPANNSLKITEEVGHTDLMAAYV
EPI_ISL_424238 1223 CEDLKPVSEEVVENPTIQKDVLECNVKTTEVVGDIILKPANNSLKITEEVGHTDLMAAYV
EPI_ISL_424248 1223 CEDLKPVSEEVVENPTIQKDVLECNVKTTEVVGDIILKPANNSLKITEEVGHTDLMAAYV
EPI_ISL_424254 1223 CEDLKPVSEEVVENPTIQKDVLECNVKTTEVVGDIILKPANNSLKITEEVGHTDLMAAYV
EPI_ISL_425548 1223 CEDLKPVSEEVVENPTIQKDVLECNVKTTEVVGDIILKPANNSLKITEEVGHTDLMAAYV
EPI_ISL_425581 1223 CEDLKPVSEEVVENPTIQKDVLECNVKTTEVVGDIILKPANNSLKITEEVGHTDLMAAYV
EPI_ISL_426881 1223 CEDLKPVSEEVVENPTIQKDVLECNVKTTEVVGDIILKPANNSLKITEEVGHTDLMAAYV
EPI_ISL_426883 1223 CEDLKPVSEEVVENPTIQKDVLECNVKTTEVVGDIILKPANNSLKITEEVGHTDLMAAYV
EPI_ISL_426888 1223 CEDLKPVSEEVVENPTIQKDVLECNVKTTEVVGDIILKPANNSLKITEEVGHTDLMAAYV
EPI_ISL_426900 1223 CEDLKPVSEEVVENPTIQKDVLECNVKTTEVVGDIILKPANNSLKITEEVGHTDLMAAYV
EPI_ISL_426901 1223 CEDLKPVSEEVVENPTIQKDVLECNVKTTEVVGDIILKPANNSLKITEEVGHTDLMAAYV
EPI_ISL_426903 1223 CEDLKPVSEEVVENPTIQKDVLECNVKTTEVVGDIILKPANNSLKITEEVGHTDLMAAYV
EPI_ISL_426904 1223 CEDLKPVSEEVVENPTIQKDVLECNVKTTEVVGDIILKPANNSLKITEEVGHTDLMAAYV
EPI_ISL_426907 1223 CEDLKPVSEEVVENPTIQKDVLECNVKTTEVVGDIILKPANNSLKITEEVGHTDLMAAYV
EPI_ISL_426910 1223 CEDLKPVSEEVVENPTIQKDVLECNVKTTEVVGDIILKPANNSLKITEEVGHTDLMAAYV
ORF1ab         2041 CEDLKPVSEEVVENPTIQKDVLECNVKTTEVVGDIILKPANNSLKITEEVGHTDLMAAYV
EPI_ISL_413575 1223 CEDLKPVSEEVVENPTIQKDVLECNVKTTEVVGDIILKPANNSLKITEEVGHTDLMAAYV
EPI_ISL_413588 1223 CEDLKPVSEEVVENPTIQKDVLECNVKTTEVVGDIILKPANNSLKITEEVGHTDLMAAYV
EPI_ISL_413589 1223 CEDLKPVSEEVVENPTIQKDVLECNVKTTEVVGDIILKPANNSLKITEEVGHTDLMAAYV
EPI_ISL_424221 1223 CEDLKPVSEEVVENPTIQKDVLECNVKTTEVVGDIILKPANNSLKITEEVGHTDLMAAYV
EPI_ISL_424233 1223 CEDLKPVSEEVVENPTIQKDVLECNVKTTEVVGDIILKPANNSLKITEEVGHTDLMAAYV
EPI_ISL_424236 1223 CEDLKPVSEEVVENPTIQKDVLECNVKTTEVVGDIILKPANNSLKITEEVGHTDLMAAYV
EPI_ISL_424238 1223 CEDLKPVSEEVVENPTIQKDVLECNVKTTEVVGDIILKPANNSLKITEEVGHTDLMAAYV
EPI_ISL_424248 1223 CEDLKPVSEEVVENPTIQKDVLECNVKTTEVVGDIILKPANNSLKITEEVGHTDLMAAYV
EPI_ISL_424254 1223 CEDLKPVSEEVVENPTIQKDVLECNVKTTEVVGDIILKPANNSLKITEEVGHTDLMAAYV
EPI_ISL_425548 1223 CEDLKPVSEEVVENPTIQKDVLECNVKTTEVVGDIILKPANNSLKITEEVGHTDLMAAYV
EPI_ISL_425581 1223 CEDLKPVSEEVVENPTIQKDVLECNVKTTEVVGDIILKPANNSLKITEEVGHTDLMAAYV
EPI_ISL_426881 1223 CEDLKPVSEEVVENPTIQKDVLECNVKTTEVVGDIILKPANNSLKITEEVGHTDLMAAYV
EPI_ISL_426883 1223 CEDLKPVSEEVVENPTIQKDVLECNVKTTEVVGDIILKPANNSLKITEEVGHTDLMAAYV
EPI_ISL_426888 1223 CEDLKPVSEEVVENPTIQKDVLECNVKTTEVVGDIILKPANNSLKITEEVGHTDLMAAYV
EPI_ISL_426900 1223 CEDLKPVSEEVVENPTIQKDVLECNVKTTEVVGDIILKPANNSLKITEEVGHTDLMAAYV
EPI_ISL_426901 1223 CEDLKPVSEEVVENPTIQKDVLECNVKTTEVVGDIILKPANNSLKITEEVGHTDLMAAYV
EPI_ISL_426903 1223 CEDLKPVSEEVVENPTIQKDVLECNVKTTEVVGDIILKPANNSLKITEEVGHTDLMAAYV
EPI_ISL_426904 1223 CEDLKPVSEEVVENPTIQKDVLECNVKTTEVVGDIILKPANNSLKITEEVGHTDLMAAYV
EPI_ISL_426907 1223 CEDLKPVSEEVVENPTIQKDVLECNVKTTEVVGDIILKPANNSLKITEEVGHTDLMAAYV
EPI_ISL_426910 1223 CEDLKPVSEEVVENPTIQKDVLECNVKTTEVVGDIILKPANNSLKITEEVGHTDLMAAYV
```

```
ORF1ab         2101 DNSSLTIKKPNELSRVLGLKTLATHGLAAVNSVPWDTIANYAKPFLNKVVSTTTNIVTRC
EPI_ISL_413575 1283 DNSSLTIKKPNELSRVLGLKTLATHGLAAVNSVPWDTIANYAKPFLNKVVSTTTNIVTRC
EPI_ISL_413588 1283 DNSSLTIKKPNELSRVLGLKTLATHGLAAVNSVPWDTIANYAKPFLNKVVSTTTNIVTRC
EPI_ISL_413589 1283 DNSSLTIKKPNELSRVLGLKTLATHGLAAVNSVPWDTIANYAKPFLNKVVSTTTNIVTRC
EPI_ISL_424221 1283 DNSSLTIKKPNELSRVLGLKTLATHGLAAVNSVPWDTIANYAKPFLNKVVSTTTNIVTRC
EPI_ISL_424233 1283 DNSSLTIKKPNELSRVLGLKTLATHGLAAVNSVPWDTIANYAKPFLNKVVSTTTNIVTRC
EPI_ISL_424236 1283 DNSSLTIKKPNELSRVLGLKTLATHGLAAVNSVPWDTIANYAKPFLNKVVSTTTNIVTRC
EPI_ISL_424238 1283 DNSSLTIKKPNELSRVLGLKTLATHGLAAVNSVPWDTIANYAKPFLNKVVSTTTNIVTRC
EPI_ISL_424248 1283 DNSSLTIKKPNELSRVLGLKTLATHGLAAVNSVPWDTIANYAKPFLNKVVSTTTNIVTRC
EPI_ISL_424254 1283 DNSSLTIKKPNELSRVLGLKTLATHGLAAVNSVPWDTIANYAKPFLNKVVSTTTNIVTRC
EPI_ISL_425548 1283 DNSSLTIKKPNELSRVLGLKTLATHGLAAVNSVPWDTIANYAKPFLNKVVSTTTNIVTRC
EPI_ISL_425581 1283 DNSSLTIKKPNELSRVLGLKTLATHGLAAVNSVPWDTIANYAKPFLNKVVSTTTNIVTRC
EPI_ISL_426881 1283 DNSSLTIKKPNELSRVLGLKTLATHGLAAVNSVPWDTIANYAKPFLNKVVSTTTNIVTRC
EPI_ISL_426883 1283 DNSSLTIKKPNELSRVLGLKTLATHGLAAVNSVPWDTIANYAKPFLNKVVSTTTNIVTRC
EPI_ISL_426888 1283 DNSSLTIKKPNELSRVLGLKTLATHGLAAVNSVPWDTIANYAKPFLNKVVSTTTNIVTRC
EPI_ISL_426900 1283 DNSSLTIKKPNELSRVLGLKTLATHGLAAVNSVPWDTIANYAKPFLNKVVSTTTNIVTRC
EPI_ISL_426901 1283 DNSSLTIKKPNELSRVLGLKTLATHGLAAVNSVPWDTIANYAKPFLNKVVSTTTNIVTRC
EPI_ISL_426903 1283 DNSSLTIKKPNELSRVLGLKTLATHGLAAVNSVPWDTIANYAKPFLNKVVSTTTNIVTRC
EPI_ISL_426904 1283 DNSSLTIKKPNELSRVLGLKTLATHGLAAVNSVPWDTIANYAKPFLNKVVSTTTNIVTRC
EPI_ISL_426907 1283 DNSSLTIKKPNELSRVLGLKTLATHGLAAVNSVPWDTIANYAKPFLNKVVSTTTNIVTRC
EPI_ISL_426910 1283 DNSSLTIKKPNELSRVLGLKTLATHGLAAVNSVPWDTIANYAKPFLNKVVSTTTNIVTRC
```


```
ORF1ab         2101 DNSSLTIKKPNELSRVLGLKTLATHGLAAVNSVPWDTIANYAKPFLNKVVSTTTNIVTRC
EPI_ISL_413575 1283 DNSSLTIKKPNELSRVLGLKTLATHGLAAVNSVPWDTIANYAKPFLNKVVSTTTNIVTRC
EPI_ISL_413588 1283 DNSSLTIKKPNELSRVLGLKTLATHGLAAVNSVPWDTIANYAKPFLNKVVSTTTNIVTRC
EPI_ISL_413589 1283 DNSSLTIKKPNELSRVLGLKTLATHGLAAVNSVPWDTIANYAKPFLNKVVSTTTNIVTRC
EPI_ISL_424221 1283 DNSSLTIKKPNELSRVLGLKTLATHGLAAVNSVPWDTIANYAKPFLNKVVSTTTNIVTRC
EPI_ISL_424233 1283 DNSSLTIKKPNELSRVLGLKTLATHGLAAVNSVPWDTIANYAKPFLNKVVSTTTNIVTRC
EPI_ISL_424236 1283 DNSSLTIKKPNELSRVLGLKTLATHGLAAVNSVPWDTIANYAKPFLNKVVSTTTNIVTRC
EPI_ISL_424238 1283 DNSSLTIKKPNELSRVLGLKTLATHGLAAVNSVPWDTIANYAKPFLNKVVSTTTNIVTRC
EPI_ISL_424248 1283 DNSSLTIKKPNELSRVLGLKTLATHGLAAVNSVPWDTIANYAKPFLNKVVSTTTNIVTRC
EPI_ISL_424254 1283 DNSSLTIKKPNELSRVLGLKTLATHGLAAVNSVPWDTIANYAKPFLNKVVSTTTNIVTRC
EPI_ISL_425548 1283 DNSSLTIKKPNELSRVLGLKTLATHGLAAVNSVPWDTIANYAKPFLNKVVSTTTNIVTRC
EPI_ISL_425581 1283 DNSSLTIKKPNELSRVLGLKTLATHGLAAVNSVPWDTIANYAKPFLNKVVSTTTNIVTRC
EPI_ISL_426881 1283 DNSSLTIKKPNELSRVLGLKTLATHGLAAVNSVPWDTIANYAKPFLNKVVSTTTNIVTRC
EPI_ISL_426883 1283 DNSSLTIKKPNELSRVLGLKTLATHGLAAVNSVPWDTIANYAKPFLNKVVSTTTNIVTRC
EPI_ISL_426888 1283 DNSSLTIKKPNELSRVLGLKTLATHGLAAVNSVPWDTIANYAKPFLNKVVSTTTNIVTRC
EPI_ISL_426900 1283 DNSSLTIKKPNELSRVLGLKTLATHGLAAVNSVPWDTIANYAKPFLNKVVSTTTNIVTRC
EPI_ISL_426901 1283 DNSSLTIKKPNELSRVLGLKTLATHGLAAVNSVPWDTIANYAKPFLNKVVSTTTNIVTRC
EPI_ISL_426903 1283 DNSSLTIKKPNELSRVLGLKTLATHGLAAVNSVPWDTIANYAKPFLNKVVSTTTNIVTRC
EPI_ISL_426904 1283 DNSSLTIKKPNELSRVLGLKTLATHGLAAVNSVPWDTIANYAKPFLNKVVSTTTNIVTRC
EPI_ISL_426907 1283 DNSSLTIKKPNELSRVLGLKTLATHGLAAVNSVPWDTIANYAKPFLNKVVSTTTNIVTRC
EPI_ISL_426910 1283 DNSSLTIKKPNELSRVLGLKTLATHGLAAVNSVPWDTIANYAKPFLNKVVSTTTNIVTRC
```

ORF1ab	2161	LNRVCTNYMPYFFTLLLQLCTFTRSTNSRIKASMPTTIAKNTVKSVGKFCLEASFNYLKS
EPI_ISL_413575	1343	LNRVCTNYMPYFFTLLLQLCTFTRSTNSRIKAFMPTTIAKNTVKSVGKFCLEASFNYLKS
EPI_ISL_413588	1343	LNRVCTNYMPYFFTLLLQLCTFTRSTNSRIKAFMPTTIAKNTVKSVGKFCLEASFNYLKS
EPI_ISL_413589	1343	LNRVCTNYMPYFFTLLLQLCTFTRSTNSRIKASMPTTIAKNTVKSVGKFCLEASFNYLKS
EPI_ISL_424221	1343	LNRVCTNYMPYFFTLLLQLCTFTRSTNSRIKASMPTTIAKNTVKSVGKFCLEASFNYLKS
EPI_ISL_424233	1343	LNRVCTNYMPYFFTLLLQLCTFTRSTNSRIKASMPTTIAKNTVKSVGKFCLEASFNYLKS
EPI_ISL_424236	1343	LNRVCTNYMPYFFTLLLQLCTFTRSTNSRIKASMPTTIAKNTVKSVGKFCLEASFNYLKS
EPI_ISL_424238	1343	LNRVCTNYMPYFFTLLLQLCTFTRSTNSRIKASMPTTIAKNTVKSVGKFCLEASFNYLKS
EPI_ISL_424248	1343	LNRVCTNYMPYFFTLLLQLCTFTRSTNSRIKASMPTTIAKNTVKSVGKFCLEASFNYLKS
EPI_ISL_424254	1343	LNRVCTNYMPYFFTLLLQLCTFTRSTNSRIKASMPTTIAKNTVKSVGKFCLEASFNYLKS
EPI_ISL_425548	1343	LNRVCTNYMPYFFTLLLQLCTFTRSTNSRIKASMPTTIAKNTVKSVGKFCLEASFNYLKS
EPI_ISL_425581	1343	LNRVCTNYMPYFFTLLLQLCTFTRSTNSRIKASMPTTIAKNTVKSVGKFCLEASFNYLKS
EPI_ISL_426881	1343	LNRVCTNYMPYFFTLLLQLCTFTRSTNSRIKASMPTTIAKNTVKSVGKFCLEASFNYLKS
EPI_ISL_426883	1343	LNRVCTNYMPYFFTLLLQLCTFTRSTNSRIKASMPTTIAKNTVKSVGKFCLEASFNYLKS
EPI_ISL_426888	1343	LNRVCTNYMPYFFTLLLQLCTFTRSTNSRIKASMPTTIAKNTVKSVGKFCLEASFNYLKS
EPI_ISL_426900	1343	LNRVCTNYMPYFFTLLLQLCTFTRSTNSRIKASMPTTIAKNTVKSVGKFCLEASFNYLKS
EPI_ISL_426901	1343	LNRVCTNYMPYFFTLLLQLCTFTRSTNSRIKASMPTTIAKNTVKSVGKFCLEASFNYLKS
EPI_ISL_426903	1343	LNRVCTNYMPYFFTLLLQLCTFTRSTNSRIKASMPTTIAKNTVKSVGKFCLEASFNYLKS
EPI_ISL_426904	1343	LNRVCTNYMPYFFTLLLQLCTFTRSTNSRIKASMPTTIAKNTVKSVGKFCLEASFNYLKS
EPI_ISL_426907	1343	LNRVCTNYMPYFFTLLLQLCTFTRSTNSRIKASMPTTIAKNTVKSVGKFCLEASFNYLKS
EPI_ISL_426910	1343	LNRVCTNYMPYFFTLLLQLCTFTRSTNSRIKASMPTTIAKNTVKSVGKFCLEASFNYLKS

ORF1ab	2161	LNRVCTNYMPYFFTLLLQLCTFTRSTNSRIKASMPTTIAKNTVKSVGKFCLEASFNYLKS
EPI_ISL_413575	1343	LNRVCTNYMPYFFTLLLQLCTFTRSTNSRIKAFMPTTIAKNTVKSVGKFCLEASFNYLKS
EPI_ISL_413588	1343	LNRVCTNYMPYFFTLLLQLCTFTRSTNSRIKAFMPTTIAKNTVKSVGKFCLEASFNYLKS
EPI_ISL_413589	1343	LNRVCTNYMPYFFTLLLQLCTFTRSTNSRIKASMPTTIAKNTVKSVGKFCLEASFNYLKS
EPI_ISL_424221	1343	LNRVCTNYMPYFFTLLLQLCTFTRSTNSRIKASMPTTIAKNTVKSVGKFCLEASFNYLKS
EPI_ISL_424233	1343	LNRVCTNYMPYFFTLLLQLCTFTRSTNSRIKASMPTTIAKNTVKSVGKFCLEASFNYLKS
EPI_ISL_424236	1343	LNRVCTNYMPYFFTLLLQLCTFTRSTNSRIKASMPTTIAKNTVKSVGKFCLEASFNYLKS
EPI_ISL_424238	1343	LNRVCTNYMPYFFTLLLQLCTFTRSTNSRIKASMPTTIAKNTVKSVGKFCLEASFNYLKS
EPI_ISL_424248	1343	LNRVCTNYMPYFFTLLLQLCTFTRSTNSRIKASMPTTIAKNTVKSVGKFCLEASFNYLKS
EPI_ISL_424254	1343	LNRVCTNYMPYFFTLLLQLCTFTRSTNSRIKASMPTTIAKNTVKSVGKFCLEASFNYLKS
EPI_ISL_425548	1343	LNRVCTNYMPYFFTLLLQLCTFTRSTNSRIKASMPTTIAKNTVKSVGKFCLEASFNYLKS
EPI_ISL_425581	1343	LNRVCTNYMPYFFTLLLQLCTFTRSTNSRIKASMPTTIAKNTVKSVGKFCLEASFNYLKS
EPI_ISL_426881	1343	LNRVCTNYMPYFFTLLLQLCTFTRSTNSRIKASMPTTIAKNTVKSVGKFCLEASFNYLKS
EPI_ISL_426883	1343	LNRVCTNYMPYFFTLLLQLCTFTRSTNSRIKASMPTTIAKNTVKSVGKFCLEASFNYLKS
EPI_ISL_426888	1343	LNRVCTNYMPYFFTLLLQLCTFTRSTNSRIKASMPTTIAKNTVKSVGKFCLEASFNYLKS
EPI_ISL_426900	1343	LNRVCTNYMPYFFTLLLQLCTFTRSTNSRIKASMPTTIAKNTVKSVGKFCLEASFNYLKS
EPI_ISL_426901	1343	LNRVCTNYMPYFFTLLLQLCTFTRSTNSRIKASMPTTIAKNTVKSVGKFCLEASFNYLKS
EPI_ISL_426903	1343	LNRVCTNYMPYFFTLLLQLCTFTRSTNSRIKASMPTTIAKNTVKSVGKFCLEASFNYLKS
EPI_ISL_426904	1343	LNRVCTNYMPYFFTLLLQLCTFTRSTNSRIKASMPTTIAKNTVKSVGKFCLEASFNYLKS
EPI_ISL_426907	1343	LNRVCTNYMPYFFTLLLQLCTFTRSTNSRIKASMPTTIAKNTVKSVGKFCLEASFNYLKS
EPI_ISL_426910	1343	LNRVCTNYMPYFFTLLLQLCTFTRSTNSRIKASMPTTIAKNTVKSVGKFCLEASFNYLKS

```
ORF1ab         2221 PNFSKLINIIIWFLLLSVCLGSLIYSTAALGVLMSNLGMPSYCTGYREGYLNSTNVTIAT
EPI_ISL_413575 1403 PNFSKLINIIIWFLLLSVCLGSLIYSTAALGVLMSNLGMPSYCTGYREGYLNSTNVTIAT
EPI_ISL_413588 1403 PNFSKLINIIIWFLLLSVCLGSLIYSTAALGVLMSNLGMPSYCTGYREGYLNSTNVTIAT
EPI_ISL_413589 1403 PNFSKLINIIIWFLLLSVCLGSLIYSTAALGVLMSNLGMPSYCTGYREGYLNSTNVTIAT
EPI_ISL_424221 1403 PNFSKLINIIIWFLLLSVCLGSLIYSTAALGVLMSNLGMPSYCTGYREGYLNSTNVTIAT
EPI_ISL_424233 1403 PNFSKLINIIIWFLLLSVCLGSLIYSTAALGVLMSNLGMPSYCTGYREGYLNSTNVTIAT
EPI_ISL_424236 1403 PNFSKLINIIIWFLLLSVCLGSLIYSTAALGVLMSNLGMPSYCTGYREGYLNSTNVTIAT
EPI_ISL_424238 1403 PNFSKLINIIIWFLLLSVCLGSLIYSTAALGVLMSNLGMPSYCTGYREGYLNSTNVTIAT
EPI_ISL_424248 1403 PNFSKLINIIIWFLLLSVCLGSLIYSTAALGVLMSNLGMPSYCTGYREGYLNSTNVTIAT
EPI_ISL_424254 1403 PNFSKLINIIIWFLLLSVCLGSLIYSTAALGVLMSNLGMPSYCTGYREGYLNSTNVTIAT
EPI_ISL_425548 1403 PNFSKLINIIIWFLLLSVCLGSLIYSTAALGVLMSNLGMPSYCTGYREGYLNSTNVTIAT
EPI_ISL_425581 1403 PNFSKLINIIIWFLLLSVCLGSLIYSTAALGVLMSNLGMPSYCTGYREGYLNSTNVTIAT
EPI_ISL_426881 1403 PNFSKLINIIIWFLLLSVCLGSLIYSTAALGVLMSNLGMPSYCTGYREGYLNSTNVTIAT
EPI_ISL_426883 1403 PNFSKLINIIIWFLLLSVCLGSLIYSTAALGVLMSNLGMPSYCTGYREGYLNSTNVTIAT
EPI_ISL_426888 1403 PNFSKLINIIIWFLLLSVCLGSLIYSTAALGVLMSNLGMPSYCTGYREGYLNSTNVTIAT
EPI_ISL_426900 1403 PNFSKLINIIIWFLLLSVCLGSLIYSTAALGVLMSNLGMPSYCTGYREGYLNSTNVTIAT
EPI_ISL_426901 1403 PNFSKLINIIIWFLLLSVCLGSLIYSTAALGVLMSNLGMPSYCTGYREGYLNSTNVTIAT
EPI_ISL_426903 1403 PNFSKLINIIIWFLLLSVCLGSLIYSTAALGVLMSNLGMPSYCTGYREGYLNSTNVTIAT
EPI_ISL_426904 1403 PNFSKLINIIIWFLLLSVCLGSLIYSTAALGVLMSNLGMPSYCTGYREGYLNSTNVTIAT
EPI_ISL_426907 1403 PNFSKLINIIIWFLLLSVCLGSLIYSTAALGVLMSNLGMPSYCTGYREGYLNSTNVTIAT
EPI_ISL_426910 1403 PNFSKLINIIIWFLLLSVCLGSLIYSTAALGVLMSNLGMPSYCTGYREGYLNSTNVTIAT
ORF1ab         2221 PNFSKLINIIIWFLLLSVCLGSLIYSTAALGVLMSNLGMPSYCTGYREGYLNSTNVTIAT
EPI_ISL_413575 1403 PNFSKLINIIIWFLLLSVCLGSLIYSTAALGVLMSNLGMPSYCTGYREGYLNSTNVTIAT
EPI_ISL_413588 1403 PNFSKLINIIIWFLLLSVCLGSLIYSTAALGVLMSNLGMPSYCTGYREGYLNSTNVTIAT
EPI_ISL_413589 1403 PNFSKLINIIIWFLLLSVCLGSLIYSTAALGVLMSNLGMPSYCTGYREGYLNSTNVTIAT
EPI_ISL_424221 1403 PNFSKLINIIIWFLLLSVCLGSLIYSTAALGVLMSNLGMPSYCTGYREGYLNSTNVTIAT
EPI_ISL_424233 1403 PNFSKLINIIIWFLLLSVCLGSLIYSTAALGVLMSNLGMPSYCTGYREGYLNSTNVTIAT
EPI_ISL_424236 1403 PNFSKLINIIIWFLLLSVCLGSLIYSTAALGVLMSNLGMPSYCTGYREGYLNSTNVTIAT
EPI_ISL_424238 1403 PNFSKLINIIIWFLLLSVCLGSLIYSTAALGVLMSNLGMPSYCTGYREGYLNSTNVTIAT
EPI_ISL_424248 1403 PNFSKLINIIIWFLLLSVCLGSLIYSTAALGVLMSNLGMPSYCTGYREGYLNSTNVTIAT
EPI_ISL_424254 1403 PNFSKLINIIIWFLLLSVCLGSLIYSTAALGVLMSNLGMPSYCTGYREGYLNSTNVTIAT
EPI_ISL_425548 1403 PNFSKLINIIIWFLLLSVCLGSLIYSTAALGVLMSNLGMPSYCTGYREGYLNSTNVTIAT
EPI_ISL_425581 1403 PNFSKLINIIIWFLLLSVCLGSLIYSTAALGVLMSNLGMPSYCTGYREGYLNSTNVTIAT
EPI_ISL_426881 1403 PNFSKLINIIIWFLLLSVCLGSLIYSTAALGVLMSNLGMPSYCTGYREGYLNSTNVTIAT
EPI_ISL_426883 1403 PNFSKLINIIIWFLLLSVCLGSLIYSTAALGVLMSNLGMPSYCTGYREGYLNSTNVTIAT
EPI_ISL_426888 1403 PNFSKLINIIIWFLLLSVCLGSLIYSTAALGVLMSNLGMPSYCTGYREGYLNSTNVTIAT
EPI_ISL_426900 1403 PNFSKLINIIIWFLLLSVCLGSLIYSTAALGVLMSNLGMPSYCTGYREGYLNSTNVTIAT
EPI_ISL_426901 1403 PNFSKLINIIIWFLLLSVCLGSLIYSTAALGVLMSNLGMPSYCTGYREGYLNSTNVTIAT
EPI_ISL_426903 1403 PNFSKLINIIIWFLLLSVCLGSLIYSTAALGVLMSNLGMPSYCTGYREGYLNSTNVTIAT
EPI_ISL_426904 1403 PNFSKLINIIIWFLLLSVCLGSLIYSTAALGVLMSNLGMPSYCTGYREGYLNSTNVTIAT
EPI_ISL_426907 1403 PNFSKLINIIIWFLLLSVCLGSLIYSTAALGVLMSNLGMPSYCTGYREGYLNSTNVTIAT
EPI_ISL_426910 1403 PNFSKLINIIIWFLLLSVCLGSLIYSTAALGVLMSNLGMPSYCTGYREGYLNSTNVTIAT
```

ORF1ab	2281	YCTGSIPCSVCLSGLDSLDTYPSLETIQITISSFKWDLTAFGLVAEWFLAYILFTRFFYV
EPI_ISL_413575	1463	YCTGSIPCSVCLSGLDSLDTYPSLETIQITISSFKWDLTAFGLVAEWFLAYILFTRFFYV
EPI_ISL_413588	1463	YCTGSIPCSVCLSGLDSLDTYPSLETIQITISSFKWDLTAFGLVAEWFLAYILFTRFFYV
EPI_ISL_413589	1463	YCTGSIPCSVCLSGLDSLDTYPSLETIQITISSFKWDLTAFGLVAEWFLAYILFTRFFYV
EPI_ISL_424221	1463	YCTGSIPCSVCLSGLDSLDTYPSLETIQITISSFKWDLTAFGLVAEWFLAYILFTRFFYV
EPI_ISL_424233	1463	YCTGSIPCSVCLSGLDSLDTYPSLETIQITISSFKWDLTAFGLVAEWFLAYILFTRFFYV
EPI_ISL_424236	1463	YCTGSIPCSVCLSGLDSLDTYPSLETIQITISSFKWDLTAFGLVAEWFLAYILFTRFFYV
EPI_ISL_424238	1463	YCTGSIPCSVCLSGLDSLDTYPSLETIQITISSFKWDLTAFGLVAEWFLAYILFTRFFYV
EPI_ISL_424248	1463	YCTGSIPCSVCLSGLDSLDTYPSLETIQITISSFKWDLTAFGLVAEWFLAYILFTRFFYV
EPI_ISL_424254	1463	YCTGSIPCSVCLSGLDSLDTYPSLETIQITISSFKWDLTAFGLVAEWFLAYILFTRFFYV
EPI_ISL_425548	1463	YCTGSIPCSVCLSGLDSLDTYPSLETIQITISSFKWDLTAFGLVAEWFLAYILFTRFFYV
EPI_ISL_425581	1463	YCTGSIPCSVCLSGLDSLDTYPSLETIQITISSFKWDLTAFGLVAEWFLAYILFTRFFYV
EPI_ISL_426881	1463	YCTGSIPCSVCLSGLDSLDTYPSLETIQITISSFKWDLTAFGLVAEWFLAYILFTRFFYV
EPI_ISL_426883	1463	YCTGSIPCSVCLSGLDSLDTYPSLETIQITISSFKWDLTAFGLVAEWFLAYILFTRFFYV
EPI_ISL_426888	1463	YCTGSIPCSVCLSGLDSLDTYPSLETIQITISSFKWDLTAFGLVAEWFLAYILFTRFFYV
EPI_ISL_426900	1463	YCTGSIPCSVCLSGLDSLDTYPSLETIQITISSFKWDLTAFGLVAEWFLAYILFTRFFYV
EPI_ISL_426901	1463	YCTGSIPCSVCLSGLDSLDTYPSLETIQITISSFKWDLTAFGLVAEWFLAYILFTRFFYV
EPI_ISL_426903	1463	YCTGSIPCSVCLSGLDSLDTYPSLETIQITISSFKWDLTAFGLVAEWFLAYILFTRFFYV
EPI_ISL_426904	1463	YCTGSIPCSVCLSGLDSLDTYPSLETIQITISSFKWDLTAFGLVAEWFLAYILFTRFFYV
EPI_ISL_426907	1463	YCTGSIPCSVCLSGLDSLDTYPSLETIQITISSFKWDLTAFGLVAEWFLAYILFTRFFYV
EPI_ISL_426910	1463	YCTGSIPCSVCLSGLDSLDTYPSLETIQITISSFKWDLTAFGLVAEWFLAYILFTRFFYV
ORF1ab	2281	YCTGSIPCSVCLSGLDSLDTYPSLETIQITISSFKWDLTAFGLVAEWFLAYILFTRFFYV
EPI_ISL_413575	1463	YCTGSIPCSVCLSGLDSLDTYPSLETIQITISSFKWDLTAFGLVAEWFLAYILFTRFFYV
EPI_ISL_413588	1463	YCTGSIPCSVCLSGLDSLDTYPSLETIQITISSFKWDLTAFGLVAEWFLAYILFTRFFYV
EPI_ISL_413589	1463	YCTGSIPCSVCLSGLDSLDTYPSLETIQITISSFKWDLTAFGLVAEWFLAYILFTRFFYV
EPI_ISL_424221	1463	YCTGSIPCSVCLSGLDSLDTYPSLETIQITISSFKWDLTAFGLVAEWFLAYILFTRFFYV
EPI_ISL_424233	1463	YCTGSIPCSVCLSGLDSLDTYPSLETIQITISSFKWDLTAFGLVAEWFLAYILFTRFFYV
EPI_ISL_424236	1463	YCTGSIPCSVCLSGLDSLDTYPSLETIQITISSFKWDLTAFGLVAEWFLAYILFTRFFYV
EPI_ISL_424238	1463	YCTGSIPCSVCLSGLDSLDTYPSLETIQITISSFKWDLTAFGLVAEWFLAYILFTRFFYV
EPI_ISL_424248	1463	YCTGSIPCSVCLSGLDSLDTYPSLETIQITISSFKWDLTAFGLVAEWFLAYILFTRFFYV
EPI_ISL_424254	1463	YCTGSIPCSVCLSGLDSLDTYPSLETIQITISSFKWDLTAFGLVAEWFLAYILFTRFFYV
EPI_ISL_425548	1463	YCTGSIPCSVCLSGLDSLDTYPSLETIQITISSFKWDLTAFGLVAEWFLAYILFTRFFYV
EPI_ISL_425581	1463	YCTGSIPCSVCLSGLDSLDTYPSLETIQITISSFKWDLTAFGLVAEWFLAYILFTRFFYV
EPI_ISL_426881	1463	YCTGSIPCSVCLSGLDSLDTYPSLETIQITISSFKWDLTAFGLVAEWFLAYILFTRFFYV
EPI_ISL_426883	1463	YCTGSIPCSVCLSGLDSLDTYPSLETIQITISSFKWDLTAFGLVAEWFLAYILFTRFFYV
EPI_ISL_426888	1463	YCTGSIPCSVCLSGLDSLDTYPSLETIQITISSFKWDLTAFGLVAEWFLAYILFTRFFYV
EPI_ISL_426900	1463	YCTGSIPCSVCLSGLDSLDTYPSLETIQITISSFKWDLTAFGLVAEWFLAYILFTRFFYV
EPI_ISL_426901	1463	YCTGSIPCSVCLSGLDSLDTYPSLETIQITISSFKWDLTAFGLVAEWFLAYILFTRFFYV
EPI_ISL_426903	1463	YCTGSIPCSVCLSGLDSLDTYPSLETIQITISSFKWDLTAFGLVAEWFLAYILFTRFFYV
EPI_ISL_426904	1463	YCTGSIPCSVCLSGLDSLDTYPSLETIQITISSFKWDLTAFGLVAEWFLAYILFTRFFYV
EPI_ISL_426907	1463	YCTGSIPCSVCLSGLDSLDTYPSLETIQITISSFKWDLTAFGLVAEWFLAYILFTRFFYV
EPI_ISL_426910	1463	YCTGSIPCSVCLSGLDSLDTYPSLETIQITISSFKWDLTAFGLVAEWFLAYILFTRFFYV

```
ORF1ab         2341 LGLAAIMQLFFSYFAVHFISNSWLMWLIINLVQMAPISAMVRMYIFFASFYYVWKSYVHV
EPI_ISL_413575 1523 LGLAAIMQLFFSYFAVHFISNSWLMWLIINLVQMAPISAMVRMYIFFASFYYVWKSYVHV
EPI_ISL_413588 1523 LGLAAIMQLFFSYFAVHFISNSWLMWLIINLVQMAPISAMVRMYIFFASFYYVWKSYVHV
EPI_ISL_413589 1523 LGLAAIMQLFFSYFAVHFISNSWLMWLIINLVQMAPISAMVRMYIFFASFYYVWKSYVHV
EPI_ISL_424221 1523 LGLAAIMQLFFSYFAVHFISNSWLMWLIINLVQMAPISAMVRMYIFFASFYYVWKSYVHV
EPI_ISL_424233 1523 LGLAAIMQLFFSYFAVHFISNSWLMWLIINLVQMAPISAMVRMYIFFASFYYVWKSYVHV
EPI_ISL_424236 1523 LGLAAIMQLFFSYFAVHFISNSWLMWLIINLVQMAPISAMVRMYIFFASFYYVWKSYVHV
EPI_ISL_424238 1523 LGLAAIMQLFFSYFAVHFISNSWLMWLIINLVQMAPISAMVRMYIFFASFYYVWKSYVHV
EPI_ISL_424248 1523 LGLAAIMQLFFSYFAVHFISNSWLMWLIINLVQMAPISAMVRMYIFFASFYYVWKSYVHV
EPI_ISL_424254 1523 LGLAAIMQLFFSYFAVHFISNSWLMWLIINLVQMAPISAMVRMYIFFASFYYVWKSYVHV
EPI_ISL_425548 1523 LGLAAIMQLFFSYFAVHFISNSWLMWLIINLVQMAPISAMVRMYIFFASFYYVWKSYVHV
EPI_ISL_425581 1523 LGLAAIMQLFFSYFAVHFISNSWLMWLIINLVQMAPISAMVRMYIFFASFYYVWKSYVHV
EPI_ISL_426881 1523 LGLAAIMQLFFSYFAVHFISNSWLMWLIINLVQMAPISAMVRMYIFFASFYYVWKSYVHV
EPI_ISL_426883 1523 LGLAAIMQLFFSYFAVHFISNSWLMWLIINLVQMAPISAMVRMYIFFASFYYVWKSYVHV
EPI_ISL_426888 1523 LGLAAIMQLFFSYFAVHFISNSWLMWLIINLVQMAPISAMVRMYIFFASFYYVWKSYVHV
EPI_ISL_426900 1523 LGLAAIMQLFFSYFAVHFISNSWLMWLIINLVQMAPISAMVRMYIFFASFYYVWKSYVHV
EPI_ISL_426901 1523 LGLAAIMQLFFSYFAVHFISNSWLMWLIINLVQMAPISAMVRMYIFFASFYYVWKSYVHV
EPI_ISL_426903 1523 LGLAAIMQLFFSYFAVHFISNSWLMWLIINLVQMAPISAMVRMYIFFASFYYVWKSYVHV
EPI_ISL_426904 1523 LGLAAIMQLFFSYFAVHFISNSWLMWLIINLVQMAPISAMVRMYIFFASFYYVWKSYVHV
EPI_ISL_426907 1523 LGLAAIMQLFFSYFAVHFISNSWLMWLIINLVQMAPISAMVRMYIFFASFYYVWKSYVHV
EPI_ISL_426910 1523 LGLAAIMQLFFSYFAVHFISNSWLMWLIINLVQMAPISAMVRMYIFFASFYYVWKSYVHV
```


```
ORF1ab         2341 LGLAAIMQLFFSYFAVHFISNSWLMWLIINLVQMAPISAMVRMYIFFASFYYVWKSYVHV
EPI_ISL_413575 1523 LGLAAIMQLFFSYFAVHFISNSWLMWLIINLVQMAPISAMVRMYIFFASFYYVWKSYVHV
EPI_ISL_413588 1523 LGLAAIMQLFFSYFAVHFISNSWLMWLIINLVQMAPISAMVRMYIFFASFYYVWKSYVHV
EPI_ISL_413589 1523 LGLAAIMQLFFSYFAVHFISNSWLMWLIINLVQMAPISAMVRMYIFFASFYYVWKSYVHV
EPI_ISL_424221 1523 LGLAAIMQLFFSYFAVHFISNSWLMWLIINLVQMAPISAMVRMYIFFASFYYVWKSYVHV
EPI_ISL_424233 1523 LGLAAIMQLFFSYFAVHFISNSWLMWLIINLVQMAPISAMVRMYIFFASFYYVWKSYVHV
EPI_ISL_424236 1523 LGLAAIMQLFFSYFAVHFISNSWLMWLIINLVQMAPISAMVRMYIFFASFYYVWKSYVHV
EPI_ISL_424238 1523 LGLAAIMQLFFSYFAVHFISNSWLMWLIINLVQMAPISAMVRMYIFFASFYYVWKSYVHV
EPI_ISL_424248 1523 LGLAAIMQLFFSYFAVHFISNSWLMWLIINLVQMAPISAMVRMYIFFASFYYVWKSYVHV
EPI_ISL_424254 1523 LGLAAIMQLFFSYFAVHFISNSWLMWLIINLVQMAPISAMVRMYIFFASFYYVWKSYVHV
EPI_ISL_425548 1523 LGLAAIMQLFFSYFAVHFISNSWLMWLIINLVQMAPISAMVRMYIFFASFYYVWKSYVHV
EPI_ISL_425581 1523 LGLAAIMQLFFSYFAVHFISNSWLMWLIINLVQMAPISAMVRMYIFFASFYYVWKSYVHV
EPI_ISL_426881 1523 LGLAAIMQLFFSYFAVHFISNSWLMWLIINLVQMAPISAMVRMYIFFASFYYVWKSYVHV
EPI_ISL_426883 1523 LGLAAIMQLFFSYFAVHFISNSWLMWLIINLVQMAPISAMVRMYIFFASFYYVWKSYVHV
EPI_ISL_426888 1523 LGLAAIMQLFFSYFAVHFISNSWLMWLIINLVQMAPISAMVRMYIFFASFYYVWKSYVHV
EPI_ISL_426900 1523 LGLAAIMQLFFSYFAVHFISNSWLMWLIINLVQMAPISAMVRMYIFFASFYYVWKSYVHV
EPI_ISL_426901 1523 LGLAAIMQLFFSYFAVHFISNSWLMWLIINLVQMAPISAMVRMYIFFASFYYVWKSYVHV
EPI_ISL_426903 1523 LGLAAIMQLFFSYFAVHFISNSWLMWLIINLVQMAPISAMVRMYIFFASFYYVWKSYVHV
EPI_ISL_426904 1523 LGLAAIMQLFFSYFAVHFISNSWLMWLIINLVQMAPISAMVRMYIFFASFYYVWKSYVHV
EPI_ISL_426907 1523 LGLAAIMQLFFSYFAVHFISNSWLMWLIINLVQMAPISAMVRMYIFFASFYYVWKSYVHV
EPI_ISL_426910 1523 LGLAAIMQLFFSYFAVHFISNSWLMWLIINLVQMAPISAMVRMYIFFASFYYVWKSYVHV
```

```
ORF1ab         2401 VDGCNSSTCMMCYKRNRATRVECTTIVNGVRRSFYVYANGGKGFCKLHNWNCVNCDTFCA
EPI_ISL_413575 1583 VDGCNSSTCMMCYKRNRATRVECTTIVNGVRRSFYVYANGGKGFCKLHNWNCVNCDTFCA
EPI_ISL_413588 1583 VDGCNSSTCMMCYKRNRATRVECTTIVNGVRRSFYVYANGGKGFCKLHNWNCVNCDTFCA
EPI_ISL_413589 1583 VDGCNSSTCMMCYKRNRATRVECTTIVNGVRRSFYVYANGGKGFCKLHNWNCVNCDTFCA
EPI_ISL_424221 1583 VDGCNSSTCMMCYKRNRATRVECTTIVNGVRRSFYVYANGGKGFCKLHNWNCVNCDTFCA
EPI_ISL_424233 1583 VDGCNSSTCMMCYKRNRATRVECTTIVNGVRRSFYVYANGGKGFCKLHNWNCVNCDTFCA
EPI_ISL_424236 1583 VDGCNSSTCMMCYKRNRATRVECTTIVNGVRRSFYVYANGGKGFCKLHNWNCVNCDTFCA
EPI_ISL_424238 1583 VDGCNSSTCMMCYKRNRATRVECTTIVNGVRRSFYVYANGGKGFCKLHNWNCVNCDTFCA
EPI_ISL_424248 1583 VDGCNSSTCMMCYKRNRATRVECTTIVNGVRRSFYVYANGGKGFCKLHNWNCVNCDTFCA
EPI_ISL_424254 1583 VDGCNSSTCMMCYKRNRATRVECTTIVNGVRRSFYVYANGGKGFCKLHNWNCVNCDTFCA
EPI_ISL_425548 1583 VDGCNSSTCMMCYKRNRATRVECTTIVNGVRRSFYVYANGGKGFCKLHNWNCVNCDTFCA
EPI_ISL_425581 1583 VDGCNSSTCMMCYKRNRATRVECTTIVNGVRRSFYVYANGGKGFCKLHNWNCVNCDTFCA
EPI_ISL_426881 1583 VDGCNSSTCMMCYKRNRATRVECTTIVNGVRRSFYVYANGGKGFCKLHNWNCVNCDTFCA
EPI_ISL_426883 1583 VDGCNSSTCMMCYKRNRATRVECTTIVNGVRRSFYVYANGGKGFCKLHNWNCVNCDTFCA
EPI_ISL_426888 1583 VDGCNSSTCMMCYKRNRATRVECTTIVNGVRRSFYVYANGGKGFCKLHNWNCVNCDTFCA
EPI_ISL_426900 1583 VDGCNSSTCMMCYKRNRATRVECTTIVNGVRRSFYVYANGGKGFCKLHNWNCVNCDTFCA
EPI_ISL_426901 1583 VDGCNSSTCMMCYKRNRATRVECTTIVNGVRRSFYVYANGGKGFCKLHNWNCVNCDTFCA
EPI_ISL_426903 1583 VDGCNSSTCMMCYKRNRATRVECTTIVNGVRRSFYVYANGGKGFCKLHNWNCVNCDTFCA
EPI_ISL_426904 1583 VDGCNSSTCMMCYKRNRATRVECTTIVNGVRRSFYVYANGGKGFCKLHNWNCVNCDTFCA
EPI_ISL_426907 1583 VDGCNSSTCMMCYKRNRATRVECTTIVNGVRRSFYVYANGGKGFCKLHNWNCVNCDTFCA
EPI_ISL_426910 1583 VDGCNSSTCMMCYKRNRATRVECTTIVNGVRRSFYVYANGGKGFCKLHNWNCVNCDTFCA
```


```
ORF1ab         2401 VDGCNSSTCMMCYKRNRATRVECTTIVNGVRRSFYVYANGGKGFCKLHNWNCVNCDTFCA
EPI_ISL_413575 1583 VDGCNSSTCMMCYKRNRATRVECTTIVNGVRRSFYVYANGGKGFCKLHNWNCVNCDTFCA
EPI_ISL_413588 1583 VDGCNSSTCMMCYKRNRATRVECTTIVNGVRRSFYVYANGGKGFCKLHNWNCVNCDTFCA
EPI_ISL_413589 1583 VDGCNSSTCMMCYKRNRATRVECTTIVNGVRRSFYVYANGGKGFCKLHNWNCVNCDTFCA
EPI_ISL_424221 1583 VDGCNSSTCMMCYKRNRATRVECTTIVNGVRRSFYVYANGGKGFCKLHNWNCVNCDTFCA
EPI_ISL_424233 1583 VDGCNSSTCMMCYKRNRATRVECTTIVNGVRRSFYVYANGGKGFCKLHNWNCVNCDTFCA
EPI_ISL_424236 1583 VDGCNSSTCMMCYKRNRATRVECTTIVNGVRRSFYVYANGGKGFCKLHNWNCVNCDTFCA
EPI_ISL_424238 1583 VDGCNSSTCMMCYKRNRATRVECTTIVNGVRRSFYVYANGGKGFCKLHNWNCVNCDTFCA
EPI_ISL_424248 1583 VDGCNSSTCMMCYKRNRATRVECTTIVNGVRRSFYVYANGGKGFCKLHNWNCVNCDTFCA
EPI_ISL_424254 1583 VDGCNSSTCMMCYKRNRATRVECTTIVNGVRRSFYVYANGGKGFCKLHNWNCVNCDTFCA
EPI_ISL_425548 1583 VDGCNSSTCMMCYKRNRATRVECTTIVNGVRRSFYVYANGGKGFCKLHNWNCVNCDTFCA
EPI_ISL_425581 1583 VDGCNSSTCMMCYKRNRATRVECTTIVNGVRRSFYVYANGGKGFCKLHNWNCVNCDTFCA
EPI_ISL_426881 1583 VDGCNSSTCMMCYKRNRATRVECTTIVNGVRRSFYVYANGGKGFCKLHNWNCVNCDTFCA
EPI_ISL_426883 1583 VDGCNSSTCMMCYKRNRATRVECTTIVNGVRRSFYVYANGGKGFCKLHNWNCVNCDTFCA
EPI_ISL_426888 1583 VDGCNSSTCMMCYKRNRATRVECTTIVNGVRRSFYVYANGGKGFCKLHNWNCVNCDTFCA
EPI_ISL_426900 1583 VDGCNSSTCMMCYKRNRATRVECTTIVNGVRRSFYVYANGGKGFCKLHNWNCVNCDTFCA
EPI_ISL_426901 1583 VDGCNSSTCMMCYKRNRATRVECTTIVNGVRRSFYVYANGGKGFCKLHNWNCVNCDTFCA
EPI_ISL_426903 1583 VDGCNSSTCMMCYKRNRATRVECTTIVNGVRRSFYVYANGGKGFCKLHNWNCVNCDTFCA
EPI_ISL_426904 1583 VDGCNSSTCMMCYKRNRATRVECTTIVNGVRRSFYVYANGGKGFCKLHNWNCVNCDTFCA
EPI_ISL_426907 1583 VDGCNSSTCMMCYKRNRATRVECTTIVNGVRRSFYVYANGGKGFCKLHNWNCVNCDTFCA
EPI_ISL_426910 1583 VDGCNSSTCMMCYKRNRATRVECTTIVNGVRRSFYVYANGGKGFCKLHNWNCVNCDTFCA
```

```
ORF1ab         2461 GSTFISDEVARDLSLQFKRPINPTDQSSYIVDSVTVKNGSIHLYFDKAGQKTYERHSLSH
EPI_ISL_413575 1643 GSTFISDEVARDLSLQFKRPINPTDQSSYIVDSVTVKNGSIHLYFDKAGQKTYERHSLSH
EPI_ISL_413588 1643 GSTFISDEVARDLSLQFKRPINPTDQSSYIVDSVTVKNGSIHLYFDKAGQKTYERHSLSH
EPI_ISL_413589 1643 GSTFISDEVARDLSLQFKRPINPTDQSSYIVDSVTVKNGSIHLYFDKAGQKTYERHSLSH
EPI_ISL_424221 1643 GSTFISDEVARDLSLQFKRPINPTDQSSYIVDSVTVKNGSIHLYFDKAGQKTYERHSLSH
EPI_ISL_424233 1643 GSTFISDEVARDLSLQFKRPINPTDQSSYIVDSVTVKNGSIHLYFDKAGQKTYERHSLSH
EPI_ISL_424236 1643 GSTFISDEVARDLSLQFKRPINPTDQSSYIVDSVTVKNGSIHLYFDKAGQKTYERHSLSH
EPI_ISL_424238 1643 GSTFISDEVARDLSLQFKRPINPTDQSSYIVDSVTVKNGSIHLYFDKAGQKTYERHSLSH
EPI_ISL_424248 1643 GSTFISDEVARDLSLQFKRPINPTDQSSYIVDSVTVKNGSIHLYFDKAGQKTYERHSLSH
EPI_ISL_424254 1643 GSTFISDEVARDLSLQFKRPINPTDQSSYIVDSVTVKNGSIHLYFDKAGQKTYERHSLSH
EPI_ISL_425548 1643 GSTFISDEVARDLSLQFKRPINPTDQSSYIVDSVTVKNGSIHLYFDKAGQKTYERHSLSH
EPI_ISL_425581 1643 GSTFISDEVARDLSLQFKRPINPTDQSSYIVDSVTVKNGSIHLYFDKAGQKTYERHSLSH
EPI_ISL_426881 1643 GSTFISDEVARDLSLQFKRPINPTDQSSYIVDSVTVKNGSIHLYFDKAGQKTYERHSLSH
EPI_ISL_426883 1643 GSTFISDEVARDLSLQFKRPINPTDQSSYIVDSVTVKNGSIHLYFDKAGQKTYERHSLSH
EPI_ISL_426888 1643 GSTFISDEVARDLSLQFKRPINPTDQSSYIVDSVTVKNGSIHLYFDKAGQKTYERHSLSH
EPI_ISL_426900 1643 GSTFISDEVARDLSLQFKRPINPTDQSSYIVDSVTVKNGSIHLYFDKAGQKTYERHSLSH
EPI_ISL_426901 1643 GSTFISDEVARDLSLQFKRPINPTDQSSYIVDSVTVKNGSIHLYFDKAGQKTYERHSLSH
EPI_ISL_426903 1643 GSTFISDEVARDLSLQFKRPINPTDQSSYIVDSVTVKNGSIHLYFDKAGQKTYERHSLSH
EPI_ISL_426904 1643 GSTFISDEVARDLSLQFKRPINPTDQSSYIVDSVTVKNGSIHLYFDKAGQKTYERHSLSH
EPI_ISL_426907 1643 GSTFISDEVARDLSLQFKRPINPTDQSSYIVDSVTVKNGSIHLYFDKAGQKTYERHSLSH
EPI_ISL_426910 1643 GSTFISDEVARDLSLQFKRPINPTDQSSYIVDSVTVKNGSIHLYFDKAGQKTYERHSLSH
ORF1ab         2461 GSTFISDEVARDLSLQFKRPINPTDQSSYIVDSVTVKNGSIHLYFDKAGQKTYERHSLSH
EPI_ISL_413575 1643 GSTFISDEVARDLSLQFKRPINPTDQSSYIVDSVTVKNGSIHLYFDKAGQKTYERHSLSH
EPI_ISL_413588 1643 GSTFISDEVARDLSLQFKRPINPTDQSSYIVDSVTVKNGSIHLYFDKAGQKTYERHSLSH
EPI_ISL_413589 1643 GSTFISDEVARDLSLQFKRPINPTDQSSYIVDSVTVKNGSIHLYFDKAGQKTYERHSLSH
EPI_ISL_424221 1643 GSTFISDEVARDLSLQFKRPINPTDQSSYIVDSVTVKNGSIHLYFDKAGQKTYERHSLSH
EPI_ISL_424233 1643 GSTFISDEVARDLSLQFKRPINPTDQSSYIVDSVTVKNGSIHLYFDKAGQKTYERHSLSH
EPI_ISL_424236 1643 GSTFISDEVARDLSLQFKRPINPTDQSSYIVDSVTVKNGSIHLYFDKAGQKTYERHSLSH
EPI_ISL_424238 1643 GSTFISDEVARDLSLQFKRPINPTDQSSYIVDSVTVKNGSIHLYFDKAGQKTYERHSLSH
EPI_ISL_424248 1643 GSTFISDEVARDLSLQFKRPINPTDQSSYIVDSVTVKNGSIHLYFDKAGQKTYERHSLSH
EPI_ISL_424254 1643 GSTFISDEVARDLSLQFKRPINPTDQSSYIVDSVTVKNGSIHLYFDKAGQKTYERHSLSH
EPI_ISL_425548 1643 GSTFISDEVARDLSLQFKRPINPTDQSSYIVDSVTVKNGSIHLYFDKAGQKTYERHSLSH
EPI_ISL_425581 1643 GSTFISDEVARDLSLQFKRPINPTDQSSYIVDSVTVKNGSIHLYFDKAGQKTYERHSLSH
EPI_ISL_426881 1643 GSTFISDEVARDLSLQFKRPINPTDQSSYIVDSVTVKNGSIHLYFDKAGQKTYERHSLSH
EPI_ISL_426883 1643 GSTFISDEVARDLSLQFKRPINPTDQSSYIVDSVTVKNGSIHLYFDKAGQKTYERHSLSH
EPI_ISL_426888 1643 GSTFISDEVARDLSLQFKRPINPTDQSSYIVDSVTVKNGSIHLYFDKAGQKTYERHSLSH
EPI_ISL_426900 1643 GSTFISDEVARDLSLQFKRPINPTDQSSYIVDSVTVKNGSIHLYFDKAGQKTYERHSLSH
EPI_ISL_426901 1643 GSTFISDEVARDLSLQFKRPINPTDQSSYIVDSVTVKNGSIHLYFDKAGQKTYERHSLSH
EPI_ISL_426903 1643 GSTFISDEVARDLSLQFKRPINPTDQSSYIVDSVTVKNGSIHLYFDKAGQKTYERHSLSH
EPI_ISL_426904 1643 GSTFISDEVARDLSLQFKRPINPTDQSSYIVDSVTVKNGSIHLYFDKAGQKTYERHSLSH
EPI_ISL_426907 1643 GSTFISDEVARDLSLQFKRPINPTDQSSYIVDSVTVKNGSIHLYFDKAGQKTYERHSLSH
EPI_ISL_426910 1643 GSTFISDEVARDLSLQFKRPINPTDQSSYIVDSVTVKNGSIHLYFDKAGQKTYERHSLSH
```

```
ORF1ab         2521 FVNLDNLRANNTKGSLPINVIVFDGKSKCEESSAKSASVYYSQLMCQPILLLDQALVSDV
EPI_ISL_413575 1703 FVNLDNLRANNTKGSLPINVIVFDGKSKCEESSAKSASVYYSQLMCQPILLLDQALVSDV
EPI_ISL_413588 1703 FVNLDNLRANNTKGSLPINVIVFDGKSKCEESSAKSASVYYSQLMCQPILLLDQALVSDV
EPI_ISL_413589 1703 FVNLDNLRANNTKGSLPINVIVFDGKSKCEESSAKSASVYYSQLMCQPILLLDQALVSDV
EPI_ISL_424221 1703 FVNLDNLRANNTKGSLPINVIVFDGKSKCEESSAKSASVYYSQLMCQPILLLDQALVSDV
EPI_ISL_424233 1703 FVNLDNLRANNTKGSLPINVIVFDGKSKCEESSAKSASVYYSQLMCQPILLLDQALVSDV
EPI_ISL_424236 1703 FVNLDNLRANNTKGSLPINVIVFDGKSKCEESSAKSASVYYSQLMCQPILLLDQALVSDV
EPI_ISL_424238 1703 FVNLDNLRANNTKGSLPINVIVFDGKSKCEESSAKSASVYYSQLMCQPILLLDQALVSDV
EPI_ISL_424248 1703 FVNLDNLRANNTKGSLPINVIVFDGKSKCEESSAKSASVYYSQLMCQPILLLDQALVSDV
EPI_ISL_424254 1703 FVNLDNLRANNTKGSLPINVIVFDGKSKCEESSAKSASVYYSQLMCQPILLLDQALVSDV
EPI_ISL_425548 1703 FVNLDNLRANNTKGSLPINVIVFDGKSKCEESSAKSASVYYSQLMCQPILLLDQALVSDV
EPI_ISL_425581 1703 FVNLDNLRANNTKGSLPINVIVFDGKSKCEESSAKSASVYYSQLMCQPILLLDQALVSDV
EPI_ISL_426881 1703 FVNLDNLRANNTKGSLPINVIVFDGKSKCEESSAKSASVYYSQLMCQPILLLDQALVSDV
EPI_ISL_426883 1703 FVNLDNLRANNTKGSLPINVIVFDGKSKCEESSAKSASVYYSQLMCQPILLLDQALVSDV
EPI_ISL_426888 1703 FVNLDNLRANNTKGSLPINVIVFDGKSKCEESSAKSASVYYSQLMCQPILLLDQALVSDV
EPI_ISL_426900 1703 FVNLDNLRANNTKGSLPINVIVFDGKSKCEESSAKSASVYYSQLMCQPILLLDQALVSDV
EPI_ISL_426901 1703 FVNLDNLRANNTKGSLPINVIVFDGKSKCEESSAKSASVYYSQLMCQPILLLDQALVSDV
EPI_ISL_426903 1703 FVNLDNLRANNTKGSLPINVIVFDGKSKCEESSAKSASVYYSQLMCQPILLLDQALVSDV
EPI_ISL_426904 1703 FVNLDNLRANNTKGSLPINVIVFDGKSKCEESSAKSASVYYSQLMCQPILLLDQALVSDV
EPI_ISL_426907 1703 FVNLDNLRANNTKGSLPVNVIVFDGKSKCEESSAKSASVYYSQLMCQPILLLDQALVSDV
EPI_ISL_426910 1703 FVNLDNLRANNTKGSLPVNVIVFDGKSKCEESSAKSASVYYSQLMCQPILLLDQALVSDV
```


```
ORF1ab         2521 FVNLDNLRANNTKGSLPINVIVFDGKSKCEESSAKSASVYYSQLMCQPILLLDQALVSDV
EPI_ISL_413575 1703 FVNLDNLRANNTKGSLPINVIVFDGKSKCEESSAKSASVYYSQLMCQPILLLDQALVSDV
EPI_ISL_413588 1703 FVNLDNLRANNTKGSLPINVIVFDGKSKCEESSAKSASVYYSQLMCQPILLLDQALVSDV
EPI_ISL_413589 1703 FVNLDNLRANNTKGSLPINVIVFDGKSKCEESSAKSASVYYSQLMCQPILLLDQALVSDV
EPI_ISL_424221 1703 FVNLDNLRANNTKGSLPINVIVFDGKSKCEESSAKSASVYYSQLMCQPILLLDQALVSDV
EPI_ISL_424233 1703 FVNLDNLRANNTKGSLPINVIVFDGKSKCEESSAKSASVYYSQLMCQPILLLDQALVSDV
EPI_ISL_424236 1703 FVNLDNLRANNTKGSLPINVIVFDGKSKCEESSAKSASVYYSQLMCQPILLLDQALVSDV
EPI_ISL_424238 1703 FVNLDNLRANNTKGSLPINVIVFDGKSKCEESSAKSASVYYSQLMCQPILLLDQALVSDV
EPI_ISL_424248 1703 FVNLDNLRANNTKGSLPINVIVFDGKSKCEESSAKSASVYYSQLMCQPILLLDQALVSDV
EPI_ISL_424254 1703 FVNLDNLRANNTKGSLPINVIVFDGKSKCEESSAKSASVYYSQLMCQPILLLDQALVSDV
EPI_ISL_425548 1703 FVNLDNLRANNTKGSLPINVIVFDGKSKCEESSAKSASVYYSQLMCQPILLLDQALVSDV
EPI_ISL_425581 1703 FVNLDNLRANNTKGSLPINVIVFDGKSKCEESSAKSASVYYSQLMCQPILLLDQALVSDV
EPI_ISL_426881 1703 FVNLDNLRANNTKGSLPINVIVFDGKSKCEESSAKSASVYYSQLMCQPILLLDQALVSDV
EPI_ISL_426883 1703 FVNLDNLRANNTKGSLPINVIVFDGKSKCEESSAKSASVYYSQLMCQPILLLDQALVSDV
EPI_ISL_426888 1703 FVNLDNLRANNTKGSLPINVIVFDGKSKCEESSAKSASVYYSQLMCQPILLLDQALVSDV
EPI_ISL_426900 1703 FVNLDNLRANNTKGSLPINVIVFDGKSKCEESSAKSASVYYSQLMCQPILLLDQALVSDV
EPI_ISL_426901 1703 FVNLDNLRANNTKGSLPINVIVFDGKSKCEESSAKSASVYYSQLMCQPILLLDQALVSDV
EPI_ISL_426903 1703 FVNLDNLRANNTKGSLPINVIVFDGKSKCEESSAKSASVYYSQLMCQPILLLDQALVSDV
EPI_ISL_426904 1703 FVNLDNLRANNTKGSLPINVIVFDGKSKCEESSAKSASVYYSQLMCQPILLLDQALVSDV
EPI_ISL_426907 1703 FVNLDNLRANNTKGSLPVNVIVFDGKSKCEESSAKSASVYYSQLMCQPILLLDQALVSDV
EPI_ISL_426910 1703 FVNLDNLRANNTKGSLPVNVIVFDGKSKCEESSAKSASVYYSQLMCQPILLLDQALVSDV
```

```
ORF1ab         2581 GDSAEVAVKMFDAYVNTFSSTFNVPMEKLKTLVATAEAELAKNVSLDNVLSTFISAARQG
EPI_ISL_413575 1763 GDSAEVAVKMFDAYVNTFSSTFNVPMEKLKTLVATAEAELAKNVSLDNVLSTFISAARQG
EPI_ISL_413588 1763 GDSAEVAVKMFDAYVNTFSSTFNVPMEKLKTLVATAEAELAKNVSLDNVLSTFISAARQG
EPI_ISL_413589 1763 GDSAEVAVKMFDAYVNTFSSTFNVPMEKLKTLVATAEAELAKNVSLDNVLSTFISAARQG
EPI_ISL_424221 1763 GDSAEVAVKMFDAYVNTFSSTFNVPMEKLKTLVATAEAELAKNVSLDNVLSTFISAARQG
EPI_ISL_424233 1763 GDSAEVAVKMFDAYVNTFSSTFNVPMEKLKTLVATAEAELAKNVSLDNVLSTFISAARQG
EPI_ISL_424236 1763 GDSAEVAVKMFDAYVNTFSSTFNVPMEKLKTLVATAEAELAKNVSLDNVLSTFISAARQG
EPI_ISL_424238 1763 GDSAEVAVKMFDAYVNTFSSTFNVPMEKLKTLVATAEAELAKNVSLDNVLSTFISAARQG
EPI_ISL_424248 1763 GDSAEVAVKMFDAYVNTFSSTFNVPMEKLKTLVATAEAELAKNVSLDNVLSTFISAARQG
EPI_ISL_424254 1763 GDSAEVAVKMFDAYVNTFSSTFNVPMEKLKTLVATAEAELAKNVSLDNVLSTFISAARQG
EPI_ISL_425548 1763 GDSAEVAVKMFDAYVNTFSSTFNVPMEKLKTLVATAEAELAKNVSLDNVLSTFISAARQG
EPI_ISL_425581 1763 GDSAEVAVKMFDAYVNTFSSTFNVPMEKLKTLVATAEAELAKNVSLDNVLSTFISAARQG
EPI_ISL_426881 1763 GDSAEVAVKMFDAYVNTFSSTFNVPMEKLKTLVATAEAELAKNVSLDNVLSTFISAARQG
EPI_ISL_426883 1763 GDSAEVAVKMFDAYVNTFSSTFNVPMEKLKTLVATAEAELAKNVSLDNVLSTFISAARQG
EPI_ISL_426888 1763 GDSAEVAVKMFDAYVNTFSSTFNVPMEKLKTLVATAEAELAKNVSLDNVLSTFISAARQG
EPI_ISL_426900 1763 GDSAEVAVKMFDAYVNTFSSTFNVPMEKLKTLVATAEAELAKNVSLDNVLSTFISAARQG
EPI_ISL_426901 1763 GDSAEVAVKMFDAYVNTFSSTFNVPMEKLKTLVATAEAELAKNVSLDNVLSTFISAARQG
EPI_ISL_426903 1763 GDSAEVAVKMFDAYVNTFSSTFNVPMEKLKTLVATAEAELAKNVSLDNVLSTFISAARQG
EPI_ISL_426904 1763 GDSAEVAVKMFDAYVNTFSSTFNVPMEKLKTLVATAEAELAKNVSLDNVLSTFISAARQG
EPI_ISL_426907 1763 GDSAEVAVKMFDAYVNTFSSTFNVPMEKLKTLVATAEAELAKNVSLDNVLSTFISAARQG
EPI_ISL_426910 1763 GDSAEVAVKMFDAYVNTFSSTFNVPMEKLKTLVATAEAELAKNVSLDNVLSTFISAARQG
```


```
ORF1ab         2581 GDSAEVAVKMFDAYVNTFSSTFNVPMEKLKTLVATAEAELAKNVSLDNVLSTFISAARQG
EPI_ISL_413575 1763 GDSAEVAVKMFDAYVNTFSSTFNVPMEKLKTLVATAEAELAKNVSLDNVLSTFISAARQG
EPI_ISL_413588 1763 GDSAEVAVKMFDAYVNTFSSTFNVPMEKLKTLVATAEAELAKNVSLDNVLSTFISAARQG
EPI_ISL_413589 1763 GDSAEVAVKMFDAYVNTFSSTFNVPMEKLKTLVATAEAELAKNVSLDNVLSTFISAARQG
EPI_ISL_424221 1763 GDSAEVAVKMFDAYVNTFSSTFNVPMEKLKTLVATAEAELAKNVSLDNVLSTFISAARQG
EPI_ISL_424233 1763 GDSAEVAVKMFDAYVNTFSSTFNVPMEKLKTLVATAEAELAKNVSLDNVLSTFISAARQG
EPI_ISL_424236 1763 GDSAEVAVKMFDAYVNTFSSTFNVPMEKLKTLVATAEAELAKNVSLDNVLSTFISAARQG
EPI_ISL_424238 1763 GDSAEVAVKMFDAYVNTFSSTFNVPMEKLKTLVATAEAELAKNVSLDNVLSTFISAARQG
EPI_ISL_424248 1763 GDSAEVAVKMFDAYVNTFSSTFNVPMEKLKTLVATAEAELAKNVSLDNVLSTFISAARQG
EPI_ISL_424254 1763 GDSAEVAVKMFDAYVNTFSSTFNVPMEKLKTLVATAEAELAKNVSLDNVLSTFISAARQG
EPI_ISL_425548 1763 GDSAEVAVKMFDAYVNTFSSTFNVPMEKLKTLVATAEAELAKNVSLDNVLSTFISAARQG
EPI_ISL_425581 1763 GDSAEVAVKMFDAYVNTFSSTFNVPMEKLKTLVATAEAELAKNVSLDNVLSTFISAARQG
EPI_ISL_426881 1763 GDSAEVAVKMFDAYVNTFSSTFNVPMEKLKTLVATAEAELAKNVSLDNVLSTFISAARQG
EPI_ISL_426883 1763 GDSAEVAVKMFDAYVNTFSSTFNVPMEKLKTLVATAEAELAKNVSLDNVLSTFISAARQG
EPI_ISL_426888 1763 GDSAEVAVKMFDAYVNTFSSTFNVPMEKLKTLVATAEAELAKNVSLDNVLSTFISAARQG
EPI_ISL_426900 1763 GDSAEVAVKMFDAYVNTFSSTFNVPMEKLKTLVATAEAELAKNVSLDNVLSTFISAARQG
EPI_ISL_426901 1763 GDSAEVAVKMFDAYVNTFSSTFNVPMEKLKTLVATAEAELAKNVSLDNVLSTFISAARQG
EPI_ISL_426903 1763 GDSAEVAVKMFDAYVNTFSSTFNVPMEKLKTLVATAEAELAKNVSLDNVLSTFISAARQG
EPI_ISL_426904 1763 GDSAEVAVKMFDAYVNTFSSTFNVPMEKLKTLVATAEAELAKNVSLDNVLSTFISAARQG
EPI_ISL_426907 1763 GDSAEVAVKMFDAYVNTFSSTFNVPMEKLKTLVATAEAELAKNVSLDNVLSTFISAARQG
EPI_ISL_426910 1763 GDSAEVAVKMFDAYVNTFSSTFNVPMEKLKTLVATAEAELAKNVSLDNVLSTFISAARQG
```

```
ORF1ab          2641 FVDSDVETKDVVECLKLSHQSDIEVTGDSCNNYMLTYNKVENMTPRDLGACIDCSARHIN
EPI_ISL_413575 1823 FVDSDVETKDVVECLKLSHQSDIEVTGDSCNNYMLTYNKVENMTPRDLGACIDCSARHIN
EPI_ISL_413588 1823 FVDSDVETKDVVECLKLSHQSDIEVTGDSCNNYMLTYNKVENMTPRDLGACIDCSARHIN
EPI_ISL_413589 1823 FVDSDVETKDVVECLKLSHQSDIEVTGDSCNNYMLTYNKVENMTPRDLGACIDCSARHIN
EPI_ISL_424221 1823 FVDSDVETKDVVECLKLSHQSDIEVTGDSCNNYMLTYNKVENMTPRDLGACIDCSARHIN
EPI_ISL_424233 1823 FVDSDVETKDVVECLKLSHQSDIEVTGDSCNNYMLTYNKVENMTPRDLGACIDCSARHIN
EPI_ISL_424236 1823 FVDSDVETKDVVECLKLSHQSDIEVTGDSCNNYMLTYNKVENMTPRDLGACIDCSARHIN
EPI_ISL_424238 1823 FVDSDVETKDVVECLKLSHQSDIEVTGDSCNNYMLTYNKVENMTPRDLGACIDCSARHIN
EPI_ISL_424248 1823 FVDSDVETKDVVECLKLSHQSDIEVTGDSCNNYMLTYNKVENMTPRDLGACIDCSARHIN
EPI_ISL_424254 1823 FVDSDVETKDVVECLKLSHQSDIEVTGDSCNNYMLTYNKVENMTPRDLGACIDCSARHIN
EPI_ISL_425548 1823 FVDSDVETKDVVECLKLSHQSDIEVTGDSCNNYMLTYNKVENMTPRDLGACIDCSARHIN
EPI_ISL_425581 1823 FVDSDVETKDVVECLKLSHQSDIEVTGDSCNNYMLTYNKVENMTPRDLGACIDCSARHIN
EPI_ISL_426881 1823 FVDSDVETKDVVECLKLSHQSDIEVTGDSCNNYMLTYNKVENMTPRDLGACIDCSARHIN
EPI_ISL_426883 1823 FVDSDVETKDVVECLKLSHQSDIEVTGDSCNNYMLTYNKVENMTPRDLGACIDCSARHIN
EPI_ISL_426888 1823 FVDSDVETKDVVECLKLSHQSDIEVTGDSCNNYMLTYNKVENMTPRDLGACIDCSARHIN
EPI_ISL_426900 1823 FVDSDVETKDVVECLKLSHQSDIEVTGDSCNNYMLTYNKVENMTPRDLGACIDCSARHIN
EPI_ISL_426901 1823 FVDSDVETKDVVECLKLSHQSDIEVTGDSCNNYMLTYNKVENMTPRDLGACIDCSARHIN
EPI_ISL_426903 1823 FVDSDVETKDVVECLKLSHQSDIEVTGDSCNNYMLTYNKVENMTPRDLGACIDCSARHIN
EPI_ISL_426904 1823 FVDSDVETKDVVECLKLSHQSDIEVTGDSCNNYMLTYNKVENMTPRDLGACIDCSARHIN
EPI_ISL_426907 1823 FVDSDVETKDVVECLKLSHQSDIEVTGDSCNNYMLTYNKVENMTPRDLGACIDCSARHIN
EPI_ISL_426910 1823 FVDSDVETKDVVECLKLSHQSDIEVTGDSCNNYMLTYNKVENMTPRDLGACIDCSARHIN
ORF1ab          2641 FVDSDVETKDVVECLKLSHQSDIEVTGDSCNNYMLTYNKVENMTPRDLGACIDCSARHIN
EPI_ISL_413575 1823 FVDSDVETKDVVECLKLSHQSDIEVTGDSCNNYMLTYNKVENMTPRDLGACIDCSARHIN
EPI_ISL_413588 1823 FVDSDVETKDVVECLKLSHQSDIEVTGDSCNNYMLTYNKVENMTPRDLGACIDCSARHIN
EPI_ISL_413589 1823 FVDSDVETKDVVECLKLSHQSDIEVTGDSCNNYMLTYNKVENMTPRDLGACIDCSARHIN
EPI_ISL_424221 1823 FVDSDVETKDVVECLKLSHQSDIEVTGDSCNNYMLTYNKVENMTPRDLGACIDCSARHIN
EPI_ISL_424233 1823 FVDSDVETKDVVECLKLSHQSDIEVTGDSCNNYMLTYNKVENMTPRDLGACIDCSARHIN
EPI_ISL_424236 1823 FVDSDVETKDVVECLKLSHQSDIEVTGDSCNNYMLTYNKVENMTPRDLGACIDCSARHIN
EPI_ISL_424238 1823 FVDSDVETKDVVECLKLSHQSDIEVTGDSCNNYMLTYNKVENMTPRDLGACIDCSARHIN
EPI_ISL_424248 1823 FVDSDVETKDVVECLKLSHQSDIEVTGDSCNNYMLTYNKVENMTPRDLGACIDCSARHIN
EPI_ISL_424254 1823 FVDSDVETKDVVECLKLSHQSDIEVTGDSCNNYMLTYNKVENMTPRDLGACIDCSARHIN
EPI_ISL_425548 1823 FVDSDVETKDVVECLKLSHQSDIEVTGDSCNNYMLTYNKVENMTPRDLGACIDCSARHIN
EPI_ISL_425581 1823 FVDSDVETKDVVECLKLSHQSDIEVTGDSCNNYMLTYNKVENMTPRDLGACIDCSARHIN
EPI_ISL_426881 1823 FVDSDVETKDVVECLKLSHQSDIEVTGDSCNNYMLTYNKVENMTPRDLGACIDCSARHIN
EPI_ISL_426883 1823 FVDSDVETKDVVECLKLSHQSDIEVTGDSCNNYMLTYNKVENMTPRDLGACIDCSARHIN
EPI_ISL_426888 1823 FVDSDVETKDVVECLKLSHQSDIEVTGDSCNNYMLTYNKVENMTPRDLGACIDCSARHIN
EPI_ISL_426900 1823 FVDSDVETKDVVECLKLSHQSDIEVTGDSCNNYMLTYNKVENMTPRDLGACIDCSARHIN
EPI_ISL_426901 1823 FVDSDVETKDVVECLKLSHQSDIEVTGDSCNNYMLTYNKVENMTPRDLGACIDCSARHIN
EPI_ISL_426903 1823 FVDSDVETKDVVECLKLSHQSDIEVTGDSCNNYMLTYNKVENMTPRDLGACIDCSARHIN
EPI_ISL_426904 1823 FVDSDVETKDVVECLKLSHQSDIEVTGDSCNNYMLTYNKVENMTPRDLGACIDCSARHIN
EPI_ISL_426907 1823 FVDSDVETKDVVECLKLSHQSDIEVTGDSCNNYMLTYNKVENMTPRDLGACIDCSARHIN
EPI_ISL_426910 1823 FVDSDVETKDVVECLKLSHQSDIEVTGDSCNNYMLTYNKVENMTPRDLGACIDCSARHIN
```

```
ORF1ab         2701 AQVAKSHNIALIWNVKDFMSLSEQLRKQIRSAAKKNNLPFKLTCATTRQVVNVVTTKIAL
EPI_ISL_413575 1883 AQVAKSHNIALIWNVKDFMSLSEQLRKQIRSAAKKNNLPFKLTCATTRQVVNVVTTKIAL
EPI_ISL_413588 1883 AQVAKSHNIALIWNVKDFMSLSEQLRKQIRSAAKKNNLPFKLTCATTRQVVNVVTTKIAL
EPI_ISL_413589 1883 AQVAKSHNIALIWNVKDFMSLSEQLRKQIRSAAKKNNLPFKLTCATTRQVVNVVTTKIAL
EPI_ISL_424221 1883 AQVAKSHNIALIWNVKDFMSLSEQLRKQIRSAAKKNNLPFKLTCATTRQVVNVVTTKIAL
EPI_ISL_424233 1883 AQVAKSHNIALIWNVKDFMSLSEQLRKQIRSAAKKNNLPFKLTCATTRQVVNVVTTKIAL
EPI_ISL_424236 1883 AQVAKSHNIALIWNVKDFMSLSEQLRKQIRSAAKKNNLPFKLTCATTRQVVNVVTTKIAL
EPI_ISL_424238 1883 AQVAKSHNIALIWNVKDFMSLSEQLRKQIRSAAKKNNLPFKLTCATTRQVVNVVTTKIAL
EPI_ISL_424248 1883 AQVAKSHNIALIWNVKDFMSLSEQLRKQIRSAAKKNNLPFKLTCATTRQVVNVVTTKIAL
EPI_ISL_424254 1883 AQVAKSHNIALIWNVKDFMSLSEQLRKQIRSAAKKNNLPFKLTCATTRQVVNVVTTKIAL
EPI_ISL_425548 1883 AQVAKSHNIALIWNVKDFMSLSEQLRKQIRSAAKKNNLPFKLTCATTRQVVNVVTTKIAL
EPI_ISL_425581 1883 AQVAKSHNIALIWNVKDFMSLSEQLRKQIRSAAKKNNLPFKLTCATTRQVVNVVTTKIAL
EPI_ISL_426881 1883 AQVAKSHNIALIWNVKDFMSLSEQLRKQIRSAAKKNNLPFKLTCATTRQVVNVVTTKIAL
EPI_ISL_426883 1883 AQVAKSHNIALIWNVKDFMSLSEQLRKQIRSAAKKNNLPFKLTCATTRQVVNVVTTKIAL
EPI_ISL_426888 1883 AQVAKSHNIALIWNVKDFMSLSEQLRKQIRSAAKKNNLPFKLTCATTRQVVNVVTTKIAL
EPI_ISL_426900 1883 AQVAKSHNIALIWNVKDFMSLSEQLRKQIRSAAKKNNLPFKLTCATTRQVVNVVTTKIAL
EPI_ISL_426901 1883 AQVAKSHNIALIWNVKDFMSLSEQLRKQIRSAAKKNNLPFKLTCATTRQVVNVVTTKIAL
EPI_ISL_426903 1883 AQVAKSHNIALIWNVKDFMSLSEQLRKQIRSAAKKNNLPFKLTCATTRQVVNVVTTKIAL
EPI_ISL_426904 1883 AQVAKSHNIALIWNVKDFMSLSEQLRKQIRSAAKKNNLPFKLTCATTRQVVNVVTTKIAL
EPI_ISL_426907 1883 AQVAKSHNIALIWNVKDFMSLSEQLRKQIRSAAKKNNLPFKLTCATTRQVVNVVTTKIAL
EPI_ISL_426910 1883 AQVAKSHNIALIWNVKDFMSLSEQLRKQIRSAAKKNNLPFKLTCATTRQVVNVVTTKIAL
ORF1ab         2701 AQVAKSHNIALIWNVKDFMSLSEQLRKQIRSAAKKNNLPFKLTCATTRQVVNVVTTKIAL
EPI_ISL_413575 1883 AQVAKSHNIALIWNVKDFMSLSEQLRKQIRSAAKKNNLPFKLTCATTRQVVNVVTTKIAL
EPI_ISL_413588 1883 AQVAKSHNIALIWNVKDFMSLSEQLRKQIRSAAKKNNLPFKLTCATTRQVVNVVTTKIAL
EPI_ISL_413589 1883 AQVAKSHNIALIWNVKDFMSLSEQLRKQIRSAAKKNNLPFKLTCATTRQVVNVVTTKIAL
EPI_ISL_424221 1883 AQVAKSHNIALIWNVKDFMSLSEQLRKQIRSAAKKNNLPFKLTCATTRQVVNVVTTKIAL
EPI_ISL_424233 1883 AQVAKSHNIALIWNVKDFMSLSEQLRKQIRSAAKKNNLPFKLTCATTRQVVNVVTTKIAL
EPI_ISL_424236 1883 AQVAKSHNIALIWNVKDFMSLSEQLRKQIRSAAKKNNLPFKLTCATTRQVVNVVTTKIAL
EPI_ISL_424238 1883 AQVAKSHNIALIWNVKDFMSLSEQLRKQIRSAAKKNNLPFKLTCATTRQVVNVVTTKIAL
EPI_ISL_424248 1883 AQVAKSHNIALIWNVKDFMSLSEQLRKQIRSAAKKNNLPFKLTCATTRQVVNVVTTKIAL
EPI_ISL_424254 1883 AQVAKSHNIALIWNVKDFMSLSEQLRKQIRSAAKKNNLPFKLTCATTRQVVNVVTTKIAL
EPI_ISL_425548 1883 AQVAKSHNIALIWNVKDFMSLSEQLRKQIRSAAKKNNLPFKLTCATTRQVVNVVTTKIAL
EPI_ISL_425581 1883 AQVAKSHNIALIWNVKDFMSLSEQLRKQIRSAAKKNNLPFKLTCATTRQVVNVVTTKIAL
EPI_ISL_426881 1883 AQVAKSHNIALIWNVKDFMSLSEQLRKQIRSAAKKNNLPFKLTCATTRQVVNVVTTKIAL
EPI_ISL_426883 1883 AQVAKSHNIALIWNVKDFMSLSEQLRKQIRSAAKKNNLPFKLTCATTRQVVNVVTTKIAL
EPI_ISL_426888 1883 AQVAKSHNIALIWNVKDFMSLSEQLRKQIRSAAKKNNLPFKLTCATTRQVVNVVTTKIAL
EPI_ISL_426900 1883 AQVAKSHNIALIWNVKDFMSLSEQLRKQIRSAAKKNNLPFKLTCATTRQVVNVVTTKIAL
EPI_ISL_426901 1883 AQVAKSHNIALIWNVKDFMSLSEQLRKQIRSAAKKNNLPFKLTCATTRQVVNVVTTKIAL
EPI_ISL_426903 1883 AQVAKSHNIALIWNVKDFMSLSEQLRKQIRSAAKKNNLPFKLTCATTRQVVNVVTTKIAL
EPI_ISL_426904 1883 AQVAKSHNIALIWNVKDFMSLSEQLRKQIRSAAKKNNLPFKLTCATTRQVVNVVTTKIAL
EPI_ISL_426907 1883 AQVAKSHNIALIWNVKDFMSLSEQLRKQIRSAAKKNNLPFKLTCATTRQVVNVVTTKIAL
EPI_ISL_426910 1883 AQVAKSHNIALIWNVKDFMSLSEQLRKQIRSAAKKNNLPFKLTCATTRQVVNVVTTKIAL
```

```
ORF1ab         2761 KGGKIVNNWLKQLIKVTLVFLFVAAIFYLITPVHVMSKHTDFSSEIIGYKAIDGGVTRDI
EPI_ISL_413575 1943 KGG---------------------------------------------------------
EPI_ISL_413588 1943 KGG---------------------------------------------------------
EPI_ISL_413589 1943 KGG---------------------------------------------------------
EPI_ISL_424221 1943 KGG---------------------------------------------------------
EPI_ISL_424233 1943 KGG---------------------------------------------------------
EPI_ISL_424236 1943 KGG---------------------------------------------------------
EPI_ISL_424238 1943 KGG---------------------------------------------------------
EPI_ISL_424248 1943 KGG---------------------------------------------------------
EPI_ISL_424254 1943 KGG---------------------------------------------------------
EPI_ISL_425548 1943 KGG---------------------------------------------------------
EPI_ISL_425581 1943 KGG---------------------------------------------------------
EPI_ISL_426881 1943 KGG---------------------------------------------------------
EPI_ISL_426883 1943 KGG---------------------------------------------------------
EPI_ISL_426888 1943 KGG---------------------------------------------------------
EPI_ISL_426900 1943 KGG---------------------------------------------------------
EPI_ISL_426901 1943 KGG---------------------------------------------------------
EPI_ISL_426903 1943 KGG---------------------------------------------------------
EPI_ISL_426904 1943 KGG---------------------------------------------------------
EPI_ISL_426907 1943 KGG---------------------------------------------------------
EPI_ISL_426910 1943 KGG---------------------------------------------------------
ORF1ab         2761 KGGKIVNNWLKQLIKVTLVFLFVAAIFYLITPVHVMSKHTDFSSEIIGYKAIDGGVTRDI
EPI_ISL_413575 1943 KGG---------------------------------------------------------
EPI_ISL_413588 1943 KGG---------------------------------------------------------
EPI_ISL_413589 1943 KGG---------------------------------------------------------
EPI_ISL_424221 1943 KGG---------------------------------------------------------
EPI_ISL_424233 1943 KGG---------------------------------------------------------
EPI_ISL_424236 1943 KGG---------------------------------------------------------
EPI_ISL_424238 1943 KGG---------------------------------------------------------
EPI_ISL_424248 1943 KGG---------------------------------------------------------
EPI_ISL_424254 1943 KGG---------------------------------------------------------
EPI_ISL_425548 1943 KGG---------------------------------------------------------
EPI_ISL_425581 1943 KGG---------------------------------------------------------
EPI_ISL_426881 1943 KGG---------------------------------------------------------
EPI_ISL_426883 1943 KGG---------------------------------------------------------
EPI_ISL_426888 1943 KGG---------------------------------------------------------
EPI_ISL_426900 1943 KGG---------------------------------------------------------
EPI_ISL_426901 1943 KGG---------------------------------------------------------
EPI_ISL_426903 1943 KGG---------------------------------------------------------
EPI_ISL_426904 1943 KGG---------------------------------------------------------
EPI_ISL_426907 1943 KGG---------------------------------------------------------
EPI_ISL_426910 1943 KGG---------------------------------------------------------
```

```
ORF1ab         2821 ASTDTCFANKHADFDTWFSQRGGSYTNDKACPLIAAVITREVGFVVPGLPGTILRTTNGD
EPI_ISL_413575      ------------------------------------------------------------
EPI_ISL_413588      ------------------------------------------------------------
EPI_ISL_413589      ------------------------------------------------------------
EPI_ISL_424221      ------------------------------------------------------------
EPI_ISL_424233      ------------------------------------------------------------
EPI_ISL_424236      ------------------------------------------------------------
EPI_ISL_424238      ------------------------------------------------------------
EPI_ISL_424248      ------------------------------------------------------------
EPI_ISL_424254      ------------------------------------------------------------
EPI_ISL_425548      ------------------------------------------------------------
EPI_ISL_425581      ------------------------------------------------------------
EPI_ISL_426881      ------------------------------------------------------------
EPI_ISL_426883      ------------------------------------------------------------
EPI_ISL_426888      ------------------------------------------------------------
EPI_ISL_426900      ------------------------------------------------------------
EPI_ISL_426901      ------------------------------------------------------------
EPI_ISL_426903      ------------------------------------------------------------
EPI_ISL_426904      ------------------------------------------------------------
EPI_ISL_426907      ------------------------------------------------------------
EPI_ISL_426910      ------------------------------------------------------------
```


```
ORF1ab         2821 ASTDTCFANKHADFDTWFSQRGGSYTNDKACPLIAAVITREVGFVVPGLPGTILRTTNGD
EPI_ISL_413575      ------------------------------------------------------------
EPI_ISL_413588      ------------------------------------------------------------
EPI_ISL_413589      ------------------------------------------------------------
EPI_ISL_424221      ------------------------------------------------------------
EPI_ISL_424233      ------------------------------------------------------------
EPI_ISL_424236      ------------------------------------------------------------
EPI_ISL_424238      ------------------------------------------------------------
EPI_ISL_424248      ------------------------------------------------------------
EPI_ISL_424254      ------------------------------------------------------------
EPI_ISL_425548      ------------------------------------------------------------
EPI_ISL_425581      ------------------------------------------------------------
EPI_ISL_426881      ------------------------------------------------------------
EPI_ISL_426883      ------------------------------------------------------------
EPI_ISL_426888      ------------------------------------------------------------
EPI_ISL_426900      ------------------------------------------------------------
EPI_ISL_426901      ------------------------------------------------------------
EPI_ISL_426903      ------------------------------------------------------------
EPI_ISL_426904      ------------------------------------------------------------
EPI_ISL_426907      ------------------------------------------------------------
EPI_ISL_426910      ------------------------------------------------------------
```

```
ORF1ab         2881 FLHFLPRVFSAVGNICYTPSKLIEYTDFATSACVLAAECTIFKDASGKPVPYCYDTNVLE
EPI_ISL_413575      ------------------------------------------------------------
EPI_ISL_413588      ------------------------------------------------------------
EPI_ISL_413589      ------------------------------------------------------------
EPI_ISL_424221      ------------------------------------------------------------
EPI_ISL_424233      ------------------------------------------------------------
EPI_ISL_424236      ------------------------------------------------------------
EPI_ISL_424238      ------------------------------------------------------------
EPI_ISL_424248      ------------------------------------------------------------
EPI_ISL_424254      ------------------------------------------------------------
EPI_ISL_425548      ------------------------------------------------------------
EPI_ISL_425581      ------------------------------------------------------------
EPI_ISL_426881      ------------------------------------------------------------
EPI_ISL_426883      ------------------------------------------------------------
EPI_ISL_426888      ------------------------------------------------------------
EPI_ISL_426900      ------------------------------------------------------------
EPI_ISL_426901      ------------------------------------------------------------
EPI_ISL_426903      ------------------------------------------------------------
EPI_ISL_426904      ------------------------------------------------------------
EPI_ISL_426907      ------------------------------------------------------------
EPI_ISL_426910      ------------------------------------------------------------
```

```
ORF1ab         2881 FLHFLPRVFSAVGNICYTPSKLIEYTDFATSACVLAAECTIFKDASGKPVPYCYDTNVLE
EPI_ISL_413575      ------------------------------------------------------------
EPI_ISL_413588      ------------------------------------------------------------
EPI_ISL_413589      ------------------------------------------------------------
EPI_ISL_424221      ------------------------------------------------------------
EPI_ISL_424233      ------------------------------------------------------------
EPI_ISL_424236      ------------------------------------------------------------
EPI_ISL_424238      ------------------------------------------------------------
EPI_ISL_424248      ------------------------------------------------------------
EPI_ISL_424254      ------------------------------------------------------------
EPI_ISL_425548      ------------------------------------------------------------
EPI_ISL_425581      ------------------------------------------------------------
EPI_ISL_426881      ------------------------------------------------------------
EPI_ISL_426883      ------------------------------------------------------------
EPI_ISL_426888      ------------------------------------------------------------
EPI_ISL_426900      ------------------------------------------------------------
EPI_ISL_426901      ------------------------------------------------------------
EPI_ISL_426903      ------------------------------------------------------------
EPI_ISL_426904      ------------------------------------------------------------
EPI_ISL_426907      ------------------------------------------------------------
EPI_ISL_426910      ------------------------------------------------------------
```

```
ORF1ab         2941 GSVAYESLRPDTRYVLMDGSIIQFPNTYLEGSVRVVTTFDSEYCRHGTCERSEAGVCVST
EPI_ISL_413575      ------------------------------------------------------------
EPI_ISL_413588      ------------------------------------------------------------
EPI_ISL_413589      ------------------------------------------------------------
EPI_ISL_424221      ------------------------------------------------------------
EPI_ISL_424233      ------------------------------------------------------------
EPI_ISL_424236      ------------------------------------------------------------
EPI_ISL_424238      ------------------------------------------------------------
EPI_ISL_424248      ------------------------------------------------------------
EPI_ISL_424254      ------------------------------------------------------------
EPI_ISL_425548      ------------------------------------------------------------
EPI_ISL_425581      ------------------------------------------------------------
EPI_ISL_426881      ------------------------------------------------------------
EPI_ISL_426883      ------------------------------------------------------------
EPI_ISL_426888      ------------------------------------------------------------
EPI_ISL_426900      ------------------------------------------------------------
EPI_ISL_426901      ------------------------------------------------------------
EPI_ISL_426903      ------------------------------------------------------------
EPI_ISL_426904      ------------------------------------------------------------
EPI_ISL_426907      ------------------------------------------------------------
EPI_ISL_426910      ------------------------------------------------------------
```


```
ORF1ab         2941 GSVAYESLRPDTRYVLMDGSIIQFPNTYLEGSVRVVTTFDSEYCRHGTCERSEAGVCVST
EPI_ISL_413575      ------------------------------------------------------------
EPI_ISL_413588      ------------------------------------------------------------
EPI_ISL_413589      ------------------------------------------------------------
EPI_ISL_424221      ------------------------------------------------------------
EPI_ISL_424233      ------------------------------------------------------------
EPI_ISL_424236      ------------------------------------------------------------
EPI_ISL_424238      ------------------------------------------------------------
EPI_ISL_424248      ------------------------------------------------------------
EPI_ISL_424254      ------------------------------------------------------------
EPI_ISL_425548      ------------------------------------------------------------
EPI_ISL_425581      ------------------------------------------------------------
EPI_ISL_426881      ------------------------------------------------------------
EPI_ISL_426883      ------------------------------------------------------------
EPI_ISL_426888      ------------------------------------------------------------
EPI_ISL_426900      ------------------------------------------------------------
EPI_ISL_426901      ------------------------------------------------------------
EPI_ISL_426903      ------------------------------------------------------------
EPI_ISL_426904      ------------------------------------------------------------
EPI_ISL_426907      ------------------------------------------------------------
EPI_ISL_426910      ------------------------------------------------------------
```

```
ORF1ab         3001 SGRWVLNNDYYRSLPGVFCGVDAVNLLTNMFTPLIQPIGALDISASIVAGGIVAIVVTCL
EPI_ISL_413575      ------------------------------------------------------------
EPI_ISL_413588      ------------------------------------------------------------
EPI_ISL_413589      ------------------------------------------------------------
EPI_ISL_424221      ------------------------------------------------------------
EPI_ISL_424233      ------------------------------------------------------------
EPI_ISL_424236      ------------------------------------------------------------
EPI_ISL_424238      ------------------------------------------------------------
EPI_ISL_424248      ------------------------------------------------------------
EPI_ISL_424254      ------------------------------------------------------------
EPI_ISL_425548      ------------------------------------------------------------
EPI_ISL_425581      ------------------------------------------------------------
EPI_ISL_426881      ------------------------------------------------------------
EPI_ISL_426883      ------------------------------------------------------------
EPI_ISL_426888      ------------------------------------------------------------
EPI_ISL_426900      ------------------------------------------------------------
EPI_ISL_426901      ------------------------------------------------------------
EPI_ISL_426903      ------------------------------------------------------------
EPI_ISL_426904      ------------------------------------------------------------
EPI_ISL_426907      ------------------------------------------------------------
EPI_ISL_426910      ------------------------------------------------------------
```

```
ORF1ab         3001 SGRWVLNNDYYRSLPGVFCGVDAVNLLTNMFTPLIQPIGALDISASIVAGGIVAIVVTCL
EPI_ISL_413575      ------------------------------------------------------------
EPI_ISL_413588      ------------------------------------------------------------
EPI_ISL_413589      ------------------------------------------------------------
EPI_ISL_424221      ------------------------------------------------------------
EPI_ISL_424233      ------------------------------------------------------------
EPI_ISL_424236      ------------------------------------------------------------
EPI_ISL_424238      ------------------------------------------------------------
EPI_ISL_424248      ------------------------------------------------------------
EPI_ISL_424254      ------------------------------------------------------------
EPI_ISL_425548      ------------------------------------------------------------
EPI_ISL_425581      ------------------------------------------------------------
EPI_ISL_426881      ------------------------------------------------------------
EPI_ISL_426883      ------------------------------------------------------------
EPI_ISL_426888      ------------------------------------------------------------
EPI_ISL_426900      ------------------------------------------------------------
EPI_ISL_426901      ------------------------------------------------------------
EPI_ISL_426903      ------------------------------------------------------------
EPI_ISL_426904      ------------------------------------------------------------
EPI_ISL_426907      ------------------------------------------------------------
EPI_ISL_426910      ------------------------------------------------------------
```

```
ORF1ab         3061 AYYFMRFRRAFGEYSHVVAFNTLLFLMSFTVLCLTPVYSFLPGVYSVIYLYLTFYLTNDV
EPI_ISL_413575      ------------------------------------------------------------
EPI_ISL_413588      ------------------------------------------------------------
EPI_ISL_413589      ------------------------------------------------------------
EPI_ISL_424221      ------------------------------------------------------------
EPI_ISL_424233      ------------------------------------------------------------
EPI_ISL_424236      ------------------------------------------------------------
EPI_ISL_424238      ------------------------------------------------------------
EPI_ISL_424248      ------------------------------------------------------------
EPI_ISL_424254      ------------------------------------------------------------
EPI_ISL_425548      ------------------------------------------------------------
EPI_ISL_425581      ------------------------------------------------------------
EPI_ISL_426881      ------------------------------------------------------------
EPI_ISL_426883      ------------------------------------------------------------
EPI_ISL_426888      ------------------------------------------------------------
EPI_ISL_426900      ------------------------------------------------------------
EPI_ISL_426901      ------------------------------------------------------------
EPI_ISL_426903      ------------------------------------------------------------
EPI_ISL_426904      ------------------------------------------------------------
EPI_ISL_426907      ------------------------------------------------------------
EPI_ISL_426910      ------------------------------------------------------------
```

```
ORF1ab         3061 AYYFMRFRRAFGEYSHVVAFNTLLFLMSFTVLCLTPVYSFLPGVYSVIYLYLTFYLTNDV
EPI_ISL_413575      ------------------------------------------------------------
EPI_ISL_413588      ------------------------------------------------------------
EPI_ISL_413589      ------------------------------------------------------------
EPI_ISL_424221      ------------------------------------------------------------
EPI_ISL_424233      ------------------------------------------------------------
EPI_ISL_424236      ------------------------------------------------------------
EPI_ISL_424238      ------------------------------------------------------------
EPI_ISL_424248      ------------------------------------------------------------
EPI_ISL_424254      ------------------------------------------------------------
EPI_ISL_425548      ------------------------------------------------------------
EPI_ISL_425581      ------------------------------------------------------------
EPI_ISL_426881      ------------------------------------------------------------
EPI_ISL_426883      ------------------------------------------------------------
EPI_ISL_426888      ------------------------------------------------------------
EPI_ISL_426900      ------------------------------------------------------------
EPI_ISL_426901      ------------------------------------------------------------
EPI_ISL_426903      ------------------------------------------------------------
EPI_ISL_426904      ------------------------------------------------------------
EPI_ISL_426907      ------------------------------------------------------------
EPI_ISL_426910      ------------------------------------------------------------
```

```
ORF1ab           3121 SFLAHIQWMVMFTPLVPFWITIAYIICISTKHFYWFFSNYLKRRVVFNGVSFSTFEEAAL
EPI_ISL_413575        ------------------------------------------------------------
EPI_ISL_413588        ------------------------------------------------------------
EPI_ISL_413589        ------------------------------------------------------------
EPI_ISL_424221        ------------------------------------------------------------
EPI_ISL_424233        ------------------------------------------------------------
EPI_ISL_424236        ------------------------------------------------------------
EPI_ISL_424238        ------------------------------------------------------------
EPI_ISL_424248        ------------------------------------------------------------
EPI_ISL_424254        ------------------------------------------------------------
EPI_ISL_425548        ------------------------------------------------------------
EPI_ISL_425581        ------------------------------------------------------------
EPI_ISL_426881        ------------------------------------------------------------
EPI_ISL_426883        ------------------------------------------------------------
EPI_ISL_426888        ------------------------------------------------------------
EPI_ISL_426900        ------------------------------------------------------------
EPI_ISL_426901        ------------------------------------------------------------
EPI_ISL_426903        ------------------------------------------------------------
EPI_ISL_426904        ------------------------------------------------------------
EPI_ISL_426907        ------------------------------------------------------------
EPI_ISL_426910        ------------------------------------------------------------
ORF1ab           3121 SFLAHIQWMVMFTPLVPFWITIAYIICISTKHFYWFFSNYLKRRVVFNGVSFSTFEEAAL
EPI_ISL_413575        ------------------------------------------------------------
EPI_ISL_413588        ------------------------------------------------------------
EPI_ISL_413589        ------------------------------------------------------------
EPI_ISL_424221        ------------------------------------------------------------
EPI_ISL_424233        ------------------------------------------------------------
EPI_ISL_424236        ------------------------------------------------------------
EPI_ISL_424238        ------------------------------------------------------------
EPI_ISL_424248        ------------------------------------------------------------
EPI_ISL_424254        ------------------------------------------------------------
EPI_ISL_425548        ------------------------------------------------------------
EPI_ISL_425581        ------------------------------------------------------------
EPI_ISL_426881        ------------------------------------------------------------
EPI_ISL_426883        ------------------------------------------------------------
EPI_ISL_426888        ------------------------------------------------------------
EPI_ISL_426900        ------------------------------------------------------------
EPI_ISL_426901        ------------------------------------------------------------
EPI_ISL_426903        ------------------------------------------------------------
EPI_ISL_426904        ------------------------------------------------------------
EPI_ISL_426907        ------------------------------------------------------------
EPI_ISL_426910        ------------------------------------------------------------
```

```
ORF1ab         3181 CTFLLNKEMYLKLRSDVLLPLTQYNRYLALYNKYKYFSGAMDTTSYREAACCHLAKALND
EPI_ISL_413575      ------------------------------------------------------------
EPI_ISL_413588      ------------------------------------------------------------
EPI_ISL_413589      ------------------------------------------------------------
EPI_ISL_424221      ------------------------------------------------------------
EPI_ISL_424233      ------------------------------------------------------------
EPI_ISL_424236      ------------------------------------------------------------
EPI_ISL_424238      ------------------------------------------------------------
EPI_ISL_424248      ------------------------------------------------------------
EPI_ISL_424254      ------------------------------------------------------------
EPI_ISL_425548      ------------------------------------------------------------
EPI_ISL_425581      ------------------------------------------------------------
EPI_ISL_426881      ------------------------------------------------------------
EPI_ISL_426883      ------------------------------------------------------------
EPI_ISL_426888      ------------------------------------------------------------
EPI_ISL_426900      ------------------------------------------------------------
EPI_ISL_426901      ------------------------------------------------------------
EPI_ISL_426903      ------------------------------------------------------------
EPI_ISL_426904      ------------------------------------------------------------
EPI_ISL_426907      ------------------------------------------------------------
EPI_ISL_426910      ------------------------------------------------------------
```


```
ORF1ab         3181 CTFLLNKEMYLKLRSDVLLPLTQYNRYLALYNKYKYFSGAMDTTSYREAACCHLAKALND
EPI_ISL_413575      ------------------------------------------------------------
EPI_ISL_413588      ------------------------------------------------------------
EPI_ISL_413589      ------------------------------------------------------------
EPI_ISL_424221      ------------------------------------------------------------
EPI_ISL_424233      ------------------------------------------------------------
EPI_ISL_424236      ------------------------------------------------------------
EPI_ISL_424238      ------------------------------------------------------------
EPI_ISL_424248      ------------------------------------------------------------
EPI_ISL_424254      ------------------------------------------------------------
EPI_ISL_425548      ------------------------------------------------------------
EPI_ISL_425581      ------------------------------------------------------------
EPI_ISL_426881      ------------------------------------------------------------
EPI_ISL_426883      ------------------------------------------------------------
EPI_ISL_426888      ------------------------------------------------------------
EPI_ISL_426900      ------------------------------------------------------------
EPI_ISL_426901      ------------------------------------------------------------
EPI_ISL_426903      ------------------------------------------------------------
EPI_ISL_426904      ------------------------------------------------------------
EPI_ISL_426907      ------------------------------------------------------------
EPI_ISL_426910      ------------------------------------------------------------
```

```
ORF1ab         3241 FSNSGSDVLYQPPQTSITSAVLQSGFRKMAFPSGKVEGCMVQVTCGTTTLNGLWLDDVVY
EPI_ISL_413575      ------------------------------------------------------------
EPI_ISL_413588      ------------------------------------------------------------
EPI_ISL_413589      ------------------------------------------------------------
EPI_ISL_424221      ------------------------------------------------------------
EPI_ISL_424233      ------------------------------------------------------------
EPI_ISL_424236      ------------------------------------------------------------
EPI_ISL_424238      ------------------------------------------------------------
EPI_ISL_424248      ------------------------------------------------------------
EPI_ISL_424254      ------------------------------------------------------------
EPI_ISL_425548      ------------------------------------------------------------
EPI_ISL_425581      ------------------------------------------------------------
EPI_ISL_426881      ------------------------------------------------------------
EPI_ISL_426883      ------------------------------------------------------------
EPI_ISL_426888      ------------------------------------------------------------
EPI_ISL_426900      ------------------------------------------------------------
EPI_ISL_426901      ------------------------------------------------------------
EPI_ISL_426903      ------------------------------------------------------------
EPI_ISL_426904      ------------------------------------------------------------
EPI_ISL_426907      ------------------------------------------------------------
EPI_ISL_426910      ------------------------------------------------------------
ORF1ab         3241 FSNSGSDVLYQPPQTSITSAVLQSGFRKMAFPSGKVEGCMVQVTCGTTTLNGLWLDDVVY
EPI_ISL_413575      ------------------------------------------------------------
EPI_ISL_413588      ------------------------------------------------------------
EPI_ISL_413589      ------------------------------------------------------------
EPI_ISL_424221      ------------------------------------------------------------
EPI_ISL_424233      ------------------------------------------------------------
EPI_ISL_424236      ------------------------------------------------------------
EPI_ISL_424238      ------------------------------------------------------------
EPI_ISL_424248      ------------------------------------------------------------
EPI_ISL_424254      ------------------------------------------------------------
EPI_ISL_425548      ------------------------------------------------------------
EPI_ISL_425581      ------------------------------------------------------------
EPI_ISL_426881      ------------------------------------------------------------
EPI_ISL_426883      ------------------------------------------------------------
EPI_ISL_426888      ------------------------------------------------------------
EPI_ISL_426900      ------------------------------------------------------------
EPI_ISL_426901      ------------------------------------------------------------
EPI_ISL_426903      ------------------------------------------------------------
EPI_ISL_426904      ------------------------------------------------------------
EPI_ISL_426907      ------------------------------------------------------------
EPI_ISL_426910      ------------------------------------------------------------
```

```
ORF1ab         3301 CPRHVICTSEDMLNPNYEDLLIRKSNHNFLVQAGNVQLRVIGHSMQNCVLKLKVDTANPK
EPI_ISL_413575      ------------------------------------------------------------
EPI_ISL_413588      ------------------------------------------------------------
EPI_ISL_413589      ------------------------------------------------------------
EPI_ISL_424221      ------------------------------------------------------------
EPI_ISL_424233      ------------------------------------------------------------
EPI_ISL_424236      ------------------------------------------------------------
EPI_ISL_424238      ------------------------------------------------------------
EPI_ISL_424248      ------------------------------------------------------------
EPI_ISL_424254      ------------------------------------------------------------
EPI_ISL_425548      ------------------------------------------------------------
EPI_ISL_425581      ------------------------------------------------------------
EPI_ISL_426881      ------------------------------------------------------------
EPI_ISL_426883      ------------------------------------------------------------
EPI_ISL_426888      ------------------------------------------------------------
EPI_ISL_426900      ------------------------------------------------------------
EPI_ISL_426901      ------------------------------------------------------------
EPI_ISL_426903      ------------------------------------------------------------
EPI_ISL_426904      ------------------------------------------------------------
EPI_ISL_426907      ------------------------------------------------------------
EPI_ISL_426910      ------------------------------------------------------------
```


```
ORF1ab         3301 CPRHVICTSEDMLNPNYEDLLIRKSNHNFLVQAGNVQLRVIGHSMQNCVLKLKVDTANPK
EPI_ISL_413575      ------------------------------------------------------------
EPI_ISL_413588      ------------------------------------------------------------
EPI_ISL_413589      ------------------------------------------------------------
EPI_ISL_424221      ------------------------------------------------------------
EPI_ISL_424233      ------------------------------------------------------------
EPI_ISL_424236      ------------------------------------------------------------
EPI_ISL_424238      ------------------------------------------------------------
EPI_ISL_424248      ------------------------------------------------------------
EPI_ISL_424254      ------------------------------------------------------------
EPI_ISL_425548      ------------------------------------------------------------
EPI_ISL_425581      ------------------------------------------------------------
EPI_ISL_426881      ------------------------------------------------------------
EPI_ISL_426883      ------------------------------------------------------------
EPI_ISL_426888      ------------------------------------------------------------
EPI_ISL_426900      ------------------------------------------------------------
EPI_ISL_426901      ------------------------------------------------------------
EPI_ISL_426903      ------------------------------------------------------------
EPI_ISL_426904      ------------------------------------------------------------
EPI_ISL_426907      ------------------------------------------------------------
EPI_ISL_426910      ------------------------------------------------------------
```

```
ORF1ab         3361 TPKYKFVRIQPGQTFSVLACYNGSPSGVYQCAMRPNFTIKGSFLNGSCGSVGFNIDYDCV
EPI_ISL_413575      ------------------------------------------------------------
EPI_ISL_413588      ------------------------------------------------------------
EPI_ISL_413589      ------------------------------------------------------------
EPI_ISL_424221      ------------------------------------------------------------
EPI_ISL_424233      ------------------------------------------------------------
EPI_ISL_424236      ------------------------------------------------------------
EPI_ISL_424238      ------------------------------------------------------------
EPI_ISL_424248      ------------------------------------------------------------
EPI_ISL_424254      ------------------------------------------------------------
EPI_ISL_425548      ------------------------------------------------------------
EPI_ISL_425581      ------------------------------------------------------------
EPI_ISL_426881      ------------------------------------------------------------
EPI_ISL_426883      ------------------------------------------------------------
EPI_ISL_426888      ------------------------------------------------------------
EPI_ISL_426900      ------------------------------------------------------------
EPI_ISL_426901      ------------------------------------------------------------
EPI_ISL_426903      ------------------------------------------------------------
EPI_ISL_426904      ------------------------------------------------------------
EPI_ISL_426907      ------------------------------------------------------------
EPI_ISL_426910      ------------------------------------------------------------
ORF1ab         3361 TPKYKFVRIQPGQTFSVLACYNGSPSGVYQCAMRPNFTIKGSFLNGSCGSVGFNIDYDCV
EPI_ISL_413575      ------------------------------------------------------------
EPI_ISL_413588      ------------------------------------------------------------
EPI_ISL_413589      ------------------------------------------------------------
EPI_ISL_424221      ------------------------------------------------------------
EPI_ISL_424233      ------------------------------------------------------------
EPI_ISL_424236      ------------------------------------------------------------
EPI_ISL_424238      ------------------------------------------------------------
EPI_ISL_424248      ------------------------------------------------------------
EPI_ISL_424254      ------------------------------------------------------------
EPI_ISL_425548      ------------------------------------------------------------
EPI_ISL_425581      ------------------------------------------------------------
EPI_ISL_426881      ------------------------------------------------------------
EPI_ISL_426883      ------------------------------------------------------------
EPI_ISL_426888      ------------------------------------------------------------
EPI_ISL_426900      ------------------------------------------------------------
EPI_ISL_426901      ------------------------------------------------------------
EPI_ISL_426903      ------------------------------------------------------------
EPI_ISL_426904      ------------------------------------------------------------
EPI_ISL_426907      ------------------------------------------------------------
EPI_ISL_426910      ------------------------------------------------------------
```

```
ORF1ab         3421 SFCYMHHMELPTGVHAGTDLEGNFYGPFVDRQTAQAAGTDTTITVNVLAWLYAAVINGDR
EPI_ISL_413575      ------------------------------------------------------------
EPI_ISL_413588      ------------------------------------------------------------
EPI_ISL_413589      ------------------------------------------------------------
EPI_ISL_424221      ------------------------------------------------------------
EPI_ISL_424233      ------------------------------------------------------------
EPI_ISL_424236      ------------------------------------------------------------
EPI_ISL_424238      ------------------------------------------------------------
EPI_ISL_424248      ------------------------------------------------------------
EPI_ISL_424254      ------------------------------------------------------------
EPI_ISL_425548      ------------------------------------------------------------
EPI_ISL_425581      ------------------------------------------------------------
EPI_ISL_426881      ------------------------------------------------------------
EPI_ISL_426883      ------------------------------------------------------------
EPI_ISL_426888      ------------------------------------------------------------
EPI_ISL_426900      ------------------------------------------------------------
EPI_ISL_426901      ------------------------------------------------------------
EPI_ISL_426903      ------------------------------------------------------------
EPI_ISL_426904      ------------------------------------------------------------
EPI_ISL_426907      ------------------------------------------------------------
EPI_ISL_426910      ------------------------------------------------------------
```

```
ORF1ab         3421 SFCYMHHMELPTGVHAGTDLEGNFYGPFVDRQTAQAAGTDTTITVNVLAWLYAAVINGDR
EPI_ISL_413575      ------------------------------------------------------------
EPI_ISL_413588      ------------------------------------------------------------
EPI_ISL_413589      ------------------------------------------------------------
EPI_ISL_424221      ------------------------------------------------------------
EPI_ISL_424233      ------------------------------------------------------------
EPI_ISL_424236      ------------------------------------------------------------
EPI_ISL_424238      ------------------------------------------------------------
EPI_ISL_424248      ------------------------------------------------------------
EPI_ISL_424254      ------------------------------------------------------------
EPI_ISL_425548      ------------------------------------------------------------
EPI_ISL_425581      ------------------------------------------------------------
EPI_ISL_426881      ------------------------------------------------------------
EPI_ISL_426883      ------------------------------------------------------------
EPI_ISL_426888      ------------------------------------------------------------
EPI_ISL_426900      ------------------------------------------------------------
EPI_ISL_426901      ------------------------------------------------------------
EPI_ISL_426903      ------------------------------------------------------------
EPI_ISL_426904      ------------------------------------------------------------
EPI_ISL_426907      ------------------------------------------------------------
EPI_ISL_426910      ------------------------------------------------------------
```

```
ORF1ab         3481 WFLNRFTTTLNDFNLVAMKYNYEPLTQDHVDILGPLSAQTGIAVLDMCASLKELLQNGMN
EPI_ISL_413575      ------------------------------------------------------------
EPI_ISL_413588      ------------------------------------------------------------
EPI_ISL_413589      ------------------------------------------------------------
EPI_ISL_424221      ------------------------------------------------------------
EPI_ISL_424233      ------------------------------------------------------------
EPI_ISL_424236      ------------------------------------------------------------
EPI_ISL_424238      ------------------------------------------------------------
EPI_ISL_424248      ------------------------------------------------------------
EPI_ISL_424254      ------------------------------------------------------------
EPI_ISL_425548      ------------------------------------------------------------
EPI_ISL_425581      ------------------------------------------------------------
EPI_ISL_426881      ------------------------------------------------------------
EPI_ISL_426883      ------------------------------------------------------------
EPI_ISL_426888      ------------------------------------------------------------
EPI_ISL_426900      ------------------------------------------------------------
EPI_ISL_426901      ------------------------------------------------------------
EPI_ISL_426903      ------------------------------------------------------------
EPI_ISL_426904      ------------------------------------------------------------
EPI_ISL_426907      ------------------------------------------------------------
EPI_ISL_426910      ------------------------------------------------------------
```

```
ORF1ab         3481 WFLNRFTTTLNDFNLVAMKYNYEPLTQDHVDILGPLSAQTGIAVLDMCASLKELLQNGMN
EPI_ISL_413575      ------------------------------------------------------------
EPI_ISL_413588      ------------------------------------------------------------
EPI_ISL_413589      ------------------------------------------------------------
EPI_ISL_424221      ------------------------------------------------------------
EPI_ISL_424233      ------------------------------------------------------------
EPI_ISL_424236      ------------------------------------------------------------
EPI_ISL_424238      ------------------------------------------------------------
EPI_ISL_424248      ------------------------------------------------------------
EPI_ISL_424254      ------------------------------------------------------------
EPI_ISL_425548      ------------------------------------------------------------
EPI_ISL_425581      ------------------------------------------------------------
EPI_ISL_426881      ------------------------------------------------------------
EPI_ISL_426883      ------------------------------------------------------------
EPI_ISL_426888      ------------------------------------------------------------
EPI_ISL_426900      ------------------------------------------------------------
EPI_ISL_426901      ------------------------------------------------------------
EPI_ISL_426903      ------------------------------------------------------------
EPI_ISL_426904      ------------------------------------------------------------
EPI_ISL_426907      ------------------------------------------------------------
EPI_ISL_426910      ------------------------------------------------------------
```

```
ORF1ab         3541 GRTILGSALLEDEFTPFDVVRQCSGVTFQSAVKRTIKGTHHWLLLTILTSLLVLVQSTQW
EPI_ISL_413575      ------------------------------------------------------------
EPI_ISL_413588      ------------------------------------------------------------
EPI_ISL_413589      ------------------------------------------------------------
EPI_ISL_424221      ------------------------------------------------------------
EPI_ISL_424233      ------------------------------------------------------------
EPI_ISL_424236      ------------------------------------------------------------
EPI_ISL_424238      ------------------------------------------------------------
EPI_ISL_424248      ------------------------------------------------------------
EPI_ISL_424254      ------------------------------------------------------------
EPI_ISL_425548      ------------------------------------------------------------
EPI_ISL_425581      ------------------------------------------------------------
EPI_ISL_426881      ------------------------------------------------------------
EPI_ISL_426883      ------------------------------------------------------------
EPI_ISL_426888      ------------------------------------------------------------
EPI_ISL_426900      ------------------------------------------------------------
EPI_ISL_426901      ------------------------------------------------------------
EPI_ISL_426903      ------------------------------------------------------------
EPI_ISL_426904      ------------------------------------------------------------
EPI_ISL_426907      ------------------------------------------------------------
EPI_ISL_426910      ------------------------------------------------------------
ORF1ab         3541 GRTILGSALLEDEFTPFDVVRQCSGVTFQSAVKRTIKGTHHWLLLTILTSLLVLVQSTQW
EPI_ISL_413575      ------------------------------------------------------------
EPI_ISL_413588      ------------------------------------------------------------
EPI_ISL_413589      ------------------------------------------------------------
EPI_ISL_424221      ------------------------------------------------------------
EPI_ISL_424233      ------------------------------------------------------------
EPI_ISL_424236      ------------------------------------------------------------
EPI_ISL_424238      ------------------------------------------------------------
EPI_ISL_424248      ------------------------------------------------------------
EPI_ISL_424254      ------------------------------------------------------------
EPI_ISL_425548      ------------------------------------------------------------
EPI_ISL_425581      ------------------------------------------------------------
EPI_ISL_426881      ------------------------------------------------------------
EPI_ISL_426883      ------------------------------------------------------------
EPI_ISL_426888      ------------------------------------------------------------
EPI_ISL_426900      ------------------------------------------------------------
EPI_ISL_426901      ------------------------------------------------------------
EPI_ISL_426903      ------------------------------------------------------------
EPI_ISL_426904      ------------------------------------------------------------
EPI_ISL_426907      ------------------------------------------------------------
EPI_ISL_426910      ------------------------------------------------------------
```

```
ORF1ab         3601 SLFFFLYENAFLPFAMGIIAMSAFAMMFVKHKHAFLCLFLLPSLATVAYFNMVYMPASWV
EPI_ISL_413575      ------------------------------------------------------------
EPI_ISL_413588      ------------------------------------------------------------
EPI_ISL_413589      ------------------------------------------------------------
EPI_ISL_424221      ------------------------------------------------------------
EPI_ISL_424233      ------------------------------------------------------------
EPI_ISL_424236      ------------------------------------------------------------
EPI_ISL_424238      ------------------------------------------------------------
EPI_ISL_424248      ------------------------------------------------------------
EPI_ISL_424254      ------------------------------------------------------------
EPI_ISL_425548      ------------------------------------------------------------
EPI_ISL_425581      ------------------------------------------------------------
EPI_ISL_426881      ------------------------------------------------------------
EPI_ISL_426883      ------------------------------------------------------------
EPI_ISL_426888      ------------------------------------------------------------
EPI_ISL_426900      ------------------------------------------------------------
EPI_ISL_426901      ------------------------------------------------------------
EPI_ISL_426903      ------------------------------------------------------------
EPI_ISL_426904      ------------------------------------------------------------
EPI_ISL_426907      ------------------------------------------------------------
EPI_ISL_426910      ------------------------------------------------------------
```


```
ORF1ab         3601 SLFFFLYENAFLPFAMGIIAMSAFAMMFVKHKHAFLCLFLLPSLATVAYFNMVYMPASWV
EPI_ISL_413575      ------------------------------------------------------------
EPI_ISL_413588      ------------------------------------------------------------
EPI_ISL_413589      ------------------------------------------------------------
EPI_ISL_424221      ------------------------------------------------------------
EPI_ISL_424233      ------------------------------------------------------------
EPI_ISL_424236      ------------------------------------------------------------
EPI_ISL_424238      ------------------------------------------------------------
EPI_ISL_424248      ------------------------------------------------------------
EPI_ISL_424254      ------------------------------------------------------------
EPI_ISL_425548      ------------------------------------------------------------
EPI_ISL_425581      ------------------------------------------------------------
EPI_ISL_426881      ------------------------------------------------------------
EPI_ISL_426883      ------------------------------------------------------------
EPI_ISL_426888      ------------------------------------------------------------
EPI_ISL_426900      ------------------------------------------------------------
EPI_ISL_426901      ------------------------------------------------------------
EPI_ISL_426903      ------------------------------------------------------------
EPI_ISL_426904      ------------------------------------------------------------
EPI_ISL_426907      ------------------------------------------------------------
EPI_ISL_426910      ------------------------------------------------------------
```

```
ORF1ab          3661 MRIMTWLDMVDTSLSGFKLKDCVMYASAVVLLILMTARTVYDDGARRVWTLMNVLTLVYK
EPI_ISL_413575       ------------------------------------------------------------
EPI_ISL_413588       ------------------------------------------------------------
EPI_ISL_413589       ------------------------------------------------------------
EPI_ISL_424221       ------------------------------------------------------------
EPI_ISL_424233       ------------------------------------------------------------
EPI_ISL_424236       ------------------------------------------------------------
EPI_ISL_424238       ------------------------------------------------------------
EPI_ISL_424248       ------------------------------------------------------------
EPI_ISL_424254       ------------------------------------------------------------
EPI_ISL_425548       ------------------------------------------------------------
EPI_ISL_425581       ------------------------------------------------------------
EPI_ISL_426881       ------------------------------------------------------------
EPI_ISL_426883       ------------------------------------------------------------
EPI_ISL_426888       ------------------------------------------------------------
EPI_ISL_426900       ------------------------------------------------------------
EPI_ISL_426901       ------------------------------------------------------------
EPI_ISL_426903       ------------------------------------------------------------
EPI_ISL_426904       ------------------------------------------------------------
EPI_ISL_426907       ------------------------------------------------------------
EPI_ISL_426910       ------------------------------------------------------------
```


```
ORF1ab          3661 MRIMTWLDMVDTSLSGFKLKDCVMYASAVVLLILMTARTVYDDGARRVWTLMNVLTLVYK
EPI_ISL_413575       ------------------------------------------------------------
EPI_ISL_413588       ------------------------------------------------------------
EPI_ISL_413589       ------------------------------------------------------------
EPI_ISL_424221       ------------------------------------------------------------
EPI_ISL_424233       ------------------------------------------------------------
EPI_ISL_424236       ------------------------------------------------------------
EPI_ISL_424238       ------------------------------------------------------------
EPI_ISL_424248       ------------------------------------------------------------
EPI_ISL_424254       ------------------------------------------------------------
EPI_ISL_425548       ------------------------------------------------------------
EPI_ISL_425581       ------------------------------------------------------------
EPI_ISL_426881       ------------------------------------------------------------
EPI_ISL_426883       ------------------------------------------------------------
EPI_ISL_426888       ------------------------------------------------------------
EPI_ISL_426900       ------------------------------------------------------------
EPI_ISL_426901       ------------------------------------------------------------
EPI_ISL_426903       ------------------------------------------------------------
EPI_ISL_426904       ------------------------------------------------------------
EPI_ISL_426907       ------------------------------------------------------------
EPI_ISL_426910       ------------------------------------------------------------
```

```
ORF1ab         3721 VYYGNALDQAISMWALIISVTSNYSGVVTTVMFLARGIVFMCVEYCPIFFITGNTLQCIM
EPI_ISL_413575      ------------------------------------------------------------
EPI_ISL_413588      ------------------------------------------------------------
EPI_ISL_413589      ------------------------------------------------------------
EPI_ISL_424221      ------------------------------------------------------------
EPI_ISL_424233      ------------------------------------------------------------
EPI_ISL_424236      ------------------------------------------------------------
EPI_ISL_424238      ------------------------------------------------------------
EPI_ISL_424248      ------------------------------------------------------------
EPI_ISL_424254      ------------------------------------------------------------
EPI_ISL_425548      ------------------------------------------------------------
EPI_ISL_425581      ------------------------------------------------------------
EPI_ISL_426881      ------------------------------------------------------------
EPI_ISL_426883      ------------------------------------------------------------
EPI_ISL_426888      ------------------------------------------------------------
EPI_ISL_426900      ------------------------------------------------------------
EPI_ISL_426901      ------------------------------------------------------------
EPI_ISL_426903      ------------------------------------------------------------
EPI_ISL_426904      ------------------------------------------------------------
EPI_ISL_426907      ------------------------------------------------------------
EPI_ISL_426910      ------------------------------------------------------------
ORF1ab         3721 VYYGNALDQAISMWALIISVTSNYSGVVTTVMFLARGIVFMCVEYCPIFFITGNTLQCIM
EPI_ISL_413575      ------------------------------------------------------------
EPI_ISL_413588      ------------------------------------------------------------
EPI_ISL_413589      ------------------------------------------------------------
EPI_ISL_424221      ------------------------------------------------------------
EPI_ISL_424233      ------------------------------------------------------------
EPI_ISL_424236      ------------------------------------------------------------
EPI_ISL_424238      ------------------------------------------------------------
EPI_ISL_424248      ------------------------------------------------------------
EPI_ISL_424254      ------------------------------------------------------------
EPI_ISL_425548      ------------------------------------------------------------
EPI_ISL_425581      ------------------------------------------------------------
EPI_ISL_426881      ------------------------------------------------------------
EPI_ISL_426883      ------------------------------------------------------------
EPI_ISL_426888      ------------------------------------------------------------
EPI_ISL_426900      ------------------------------------------------------------
EPI_ISL_426901      ------------------------------------------------------------
EPI_ISL_426903      ------------------------------------------------------------
EPI_ISL_426904      ------------------------------------------------------------
EPI_ISL_426907      ------------------------------------------------------------
EPI_ISL_426910      ------------------------------------------------------------
```

```
ORF1ab         3781 LVYCFLGYFCTCYFGLFCLLNRYFRLTLGVYDYLVSTQEFRYMNSQGLLPPKNSIDAFKL
EPI_ISL_413575      ------------------------------------------------------------
EPI_ISL_413588      ------------------------------------------------------------
EPI_ISL_413589      ------------------------------------------------------------
EPI_ISL_424221      ------------------------------------------------------------
EPI_ISL_424233      ------------------------------------------------------------
EPI_ISL_424236      ------------------------------------------------------------
EPI_ISL_424238      ------------------------------------------------------------
EPI_ISL_424248      ------------------------------------------------------------
EPI_ISL_424254      ------------------------------------------------------------
EPI_ISL_425548      ------------------------------------------------------------
EPI_ISL_425581      ------------------------------------------------------------
EPI_ISL_426881      ------------------------------------------------------------
EPI_ISL_426883      ------------------------------------------------------------
EPI_ISL_426888      ------------------------------------------------------------
EPI_ISL_426900      ------------------------------------------------------------
EPI_ISL_426901      ------------------------------------------------------------
EPI_ISL_426903      ------------------------------------------------------------
EPI_ISL_426904      ------------------------------------------------------------
EPI_ISL_426907      ------------------------------------------------------------
EPI_ISL_426910      ------------------------------------------------------------
```

```
ORF1ab         3781 LVYCFLGYFCTCYFGLFCLLNRYFRLTLGVYDYLVSTQEFRYMNSQGLLPPKNSIDAFKL
EPI_ISL_413575      ------------------------------------------------------------
EPI_ISL_413588      ------------------------------------------------------------
EPI_ISL_413589      ------------------------------------------------------------
EPI_ISL_424221      ------------------------------------------------------------
EPI_ISL_424233      ------------------------------------------------------------
EPI_ISL_424236      ------------------------------------------------------------
EPI_ISL_424238      ------------------------------------------------------------
EPI_ISL_424248      ------------------------------------------------------------
EPI_ISL_424254      ------------------------------------------------------------
EPI_ISL_425548      ------------------------------------------------------------
EPI_ISL_425581      ------------------------------------------------------------
EPI_ISL_426881      ------------------------------------------------------------
EPI_ISL_426883      ------------------------------------------------------------
EPI_ISL_426888      ------------------------------------------------------------
EPI_ISL_426900      ------------------------------------------------------------
EPI_ISL_426901      ------------------------------------------------------------
EPI_ISL_426903      ------------------------------------------------------------
EPI_ISL_426904      ------------------------------------------------------------
EPI_ISL_426907      ------------------------------------------------------------
EPI_ISL_426910      ------------------------------------------------------------
```

```
ORF1ab         3841 NIKLLGVGGKPCIKVATVQSKMSDVKCTSVVLLSVLQQLRVESSSKLWAQCVQLHNDILL
EPI_ISL_413575      ------------------------------------------------------------
EPI_ISL_413588      ------------------------------------------------------------
EPI_ISL_413589      ------------------------------------------------------------
EPI_ISL_424221      ------------------------------------------------------------
EPI_ISL_424233      ------------------------------------------------------------
EPI_ISL_424236      ------------------------------------------------------------
EPI_ISL_424238      ------------------------------------------------------------
EPI_ISL_424248      ------------------------------------------------------------
EPI_ISL_424254      ------------------------------------------------------------
EPI_ISL_425548      ------------------------------------------------------------
EPI_ISL_425581      ------------------------------------------------------------
EPI_ISL_426881      ------------------------------------------------------------
EPI_ISL_426883      ------------------------------------------------------------
EPI_ISL_426888      ------------------------------------------------------------
EPI_ISL_426900      ------------------------------------------------------------
EPI_ISL_426901      ------------------------------------------------------------
EPI_ISL_426903      ------------------------------------------------------------
EPI_ISL_426904      ------------------------------------------------------------
EPI_ISL_426907      ------------------------------------------------------------
EPI_ISL_426910      ------------------------------------------------------------
ORF1ab         3841 NIKLLGVGGKPCIKVATVQSKMSDVKCTSVVLLSVLQQLRVESSSKLWAQCVQLHNDILL
EPI_ISL_413575      ------------------------------------------------------------
EPI_ISL_413588      ------------------------------------------------------------
EPI_ISL_413589      ------------------------------------------------------------
EPI_ISL_424221      ------------------------------------------------------------
EPI_ISL_424233      ------------------------------------------------------------
EPI_ISL_424236      ------------------------------------------------------------
EPI_ISL_424238      ------------------------------------------------------------
EPI_ISL_424248      ------------------------------------------------------------
EPI_ISL_424254      ------------------------------------------------------------
EPI_ISL_425548      ------------------------------------------------------------
EPI_ISL_425581      ------------------------------------------------------------
EPI_ISL_426881      ------------------------------------------------------------
EPI_ISL_426883      ------------------------------------------------------------
EPI_ISL_426888      ------------------------------------------------------------
EPI_ISL_426900      ------------------------------------------------------------
EPI_ISL_426901      ------------------------------------------------------------
EPI_ISL_426903      ------------------------------------------------------------
EPI_ISL_426904      ------------------------------------------------------------
EPI_ISL_426907      ------------------------------------------------------------
EPI_ISL_426910      ------------------------------------------------------------
```

```
ORF1ab          3901 AKDTTEAFEKMVSLLSVLLSMQGAVDINKLCEEMLDNRATLQAIASEFSSLPSYAAFATA
EPI_ISL_413575       ------------------------------------------------------------
EPI_ISL_413588       ------------------------------------------------------------
EPI_ISL_413589       ------------------------------------------------------------
EPI_ISL_424221       ------------------------------------------------------------
EPI_ISL_424233       ------------------------------------------------------------
EPI_ISL_424236       ------------------------------------------------------------
EPI_ISL_424238       ------------------------------------------------------------
EPI_ISL_424248       ------------------------------------------------------------
EPI_ISL_424254       ------------------------------------------------------------
EPI_ISL_425548       ------------------------------------------------------------
EPI_ISL_425581       ------------------------------------------------------------
EPI_ISL_426881       ------------------------------------------------------------
EPI_ISL_426883       ------------------------------------------------------------
EPI_ISL_426888       ------------------------------------------------------------
EPI_ISL_426900       ------------------------------------------------------------
EPI_ISL_426901       ------------------------------------------------------------
EPI_ISL_426903       ------------------------------------------------------------
EPI_ISL_426904       ------------------------------------------------------------
EPI_ISL_426907       ------------------------------------------------------------
EPI_ISL_426910       ------------------------------------------------------------
```


```
ORF1ab          3901 AKDTTEAFEKMVSLLSVLLSMQGAVDINKLCEEMLDNRATLQAIASEFSSLPSYAAFATA
EPI_ISL_413575       ------------------------------------------------------------
EPI_ISL_413588       ------------------------------------------------------------
EPI_ISL_413589       ------------------------------------------------------------
EPI_ISL_424221       ------------------------------------------------------------
EPI_ISL_424233       ------------------------------------------------------------
EPI_ISL_424236       ------------------------------------------------------------
EPI_ISL_424238       ------------------------------------------------------------
EPI_ISL_424248       ------------------------------------------------------------
EPI_ISL_424254       ------------------------------------------------------------
EPI_ISL_425548       ------------------------------------------------------------
EPI_ISL_425581       ------------------------------------------------------------
EPI_ISL_426881       ------------------------------------------------------------
EPI_ISL_426883       ------------------------------------------------------------
EPI_ISL_426888       ------------------------------------------------------------
EPI_ISL_426900       ------------------------------------------------------------
EPI_ISL_426901       ------------------------------------------------------------
EPI_ISL_426903       ------------------------------------------------------------
EPI_ISL_426904       ------------------------------------------------------------
EPI_ISL_426907       ------------------------------------------------------------
EPI_ISL_426910       ------------------------------------------------------------
```

```
ORF1ab         3961 QEAYEQAVANGDSEVVLKKLKKSLNVAKSEFDRDAAMQRKLEKMADQAMTQMYKQARSED
EPI_ISL_413575      ------------------------------------------------------------
EPI_ISL_413588      ------------------------------------------------------------
EPI_ISL_413589      ------------------------------------------------------------
EPI_ISL_424221      ------------------------------------------------------------
EPI_ISL_424233      ------------------------------------------------------------
EPI_ISL_424236      ------------------------------------------------------------
EPI_ISL_424238      ------------------------------------------------------------
EPI_ISL_424248      ------------------------------------------------------------
EPI_ISL_424254      ------------------------------------------------------------
EPI_ISL_425548      ------------------------------------------------------------
EPI_ISL_425581      ------------------------------------------------------------
EPI_ISL_426881      ------------------------------------------------------------
EPI_ISL_426883      ------------------------------------------------------------
EPI_ISL_426888      ------------------------------------------------------------
EPI_ISL_426900      ------------------------------------------------------------
EPI_ISL_426901      ------------------------------------------------------------
EPI_ISL_426903      ------------------------------------------------------------
EPI_ISL_426904      ------------------------------------------------------------
EPI_ISL_426907      ------------------------------------------------------------
EPI_ISL_426910      ------------------------------------------------------------
```


```
ORF1ab         3961 QEAYEQAVANGDSEVVLKKLKKSLNVAKSEFDRDAAMQRKLEKMADQAMTQMYKQARSED
EPI_ISL_413575      ------------------------------------------------------------
EPI_ISL_413588      ------------------------------------------------------------
EPI_ISL_413589      ------------------------------------------------------------
EPI_ISL_424221      ------------------------------------------------------------
EPI_ISL_424233      ------------------------------------------------------------
EPI_ISL_424236      ------------------------------------------------------------
EPI_ISL_424238      ------------------------------------------------------------
EPI_ISL_424248      ------------------------------------------------------------
EPI_ISL_424254      ------------------------------------------------------------
EPI_ISL_425548      ------------------------------------------------------------
EPI_ISL_425581      ------------------------------------------------------------
EPI_ISL_426881      ------------------------------------------------------------
EPI_ISL_426883      ------------------------------------------------------------
EPI_ISL_426888      ------------------------------------------------------------
EPI_ISL_426900      ------------------------------------------------------------
EPI_ISL_426901      ------------------------------------------------------------
EPI_ISL_426903      ------------------------------------------------------------
EPI_ISL_426904      ------------------------------------------------------------
EPI_ISL_426907      ------------------------------------------------------------
EPI_ISL_426910      ------------------------------------------------------------
```

```
ORF1ab         4021 KRAKVTSAMQTMLFTMLRKLDNDALNNIINNARDGCVPLNIIPLTTAAKLMVVIPDYNTY
EPI_ISL_413575      ------------------------------------------------------------
EPI_ISL_413588      ------------------------------------------------------------
EPI_ISL_413589      ------------------------------------------------------------
EPI_ISL_424221      ------------------------------------------------------------
EPI_ISL_424233      ------------------------------------------------------------
EPI_ISL_424236      ------------------------------------------------------------
EPI_ISL_424238      ------------------------------------------------------------
EPI_ISL_424248      ------------------------------------------------------------
EPI_ISL_424254      ------------------------------------------------------------
EPI_ISL_425548      ------------------------------------------------------------
EPI_ISL_425581      ------------------------------------------------------------
EPI_ISL_426881      ------------------------------------------------------------
EPI_ISL_426883      ------------------------------------------------------------
EPI_ISL_426888      ------------------------------------------------------------
EPI_ISL_426900      ------------------------------------------------------------
EPI_ISL_426901      ------------------------------------------------------------
EPI_ISL_426903      ------------------------------------------------------------
EPI_ISL_426904      ------------------------------------------------------------
EPI_ISL_426907      ------------------------------------------------------------
EPI_ISL_426910      ------------------------------------------------------------
ORF1ab         4021 KRAKVTSAMQTMLFTMLRKLDNDALNNIINNARDGCVPLNIIPLTTAAKLMVVIPDYNTY
EPI_ISL_413575      ------------------------------------------------------------
EPI_ISL_413588      ------------------------------------------------------------
EPI_ISL_413589      ------------------------------------------------------------
EPI_ISL_424221      ------------------------------------------------------------
EPI_ISL_424233      ------------------------------------------------------------
EPI_ISL_424236      ------------------------------------------------------------
EPI_ISL_424238      ------------------------------------------------------------
EPI_ISL_424248      ------------------------------------------------------------
EPI_ISL_424254      ------------------------------------------------------------
EPI_ISL_425548      ------------------------------------------------------------
EPI_ISL_425581      ------------------------------------------------------------
EPI_ISL_426881      ------------------------------------------------------------
EPI_ISL_426883      ------------------------------------------------------------
EPI_ISL_426888      ------------------------------------------------------------
EPI_ISL_426900      ------------------------------------------------------------
EPI_ISL_426901      ------------------------------------------------------------
EPI_ISL_426903      ------------------------------------------------------------
EPI_ISL_426904      ------------------------------------------------------------
EPI_ISL_426907      ------------------------------------------------------------
EPI_ISL_426910      ------------------------------------------------------------
```

```
ORF1ab         4081 KNTCDGTTFTYASALWEIQQVVDADSKIVQLSEISMDNSPNLAWPLIVTALRANSAVKLQ
EPI_ISL_413575      ------------------------------------------------------------
EPI_ISL_413588      ------------------------------------------------------------
EPI_ISL_413589      ------------------------------------------------------------
EPI_ISL_424221      ------------------------------------------------------------
EPI_ISL_424233      ------------------------------------------------------------
EPI_ISL_424236      ------------------------------------------------------------
EPI_ISL_424238      ------------------------------------------------------------
EPI_ISL_424248      ------------------------------------------------------------
EPI_ISL_424254      ------------------------------------------------------------
EPI_ISL_425548      ------------------------------------------------------------
EPI_ISL_425581      ------------------------------------------------------------
EPI_ISL_426881      ------------------------------------------------------------
EPI_ISL_426883      ------------------------------------------------------------
EPI_ISL_426888      ------------------------------------------------------------
EPI_ISL_426900      ------------------------------------------------------------
EPI_ISL_426901      ------------------------------------------------------------
EPI_ISL_426903      ------------------------------------------------------------
EPI_ISL_426904      ------------------------------------------------------------
EPI_ISL_426907      ------------------------------------------------------------
EPI_ISL_426910      ------------------------------------------------------------
```

```
ORF1ab         4081 KNTCDGTTFTYASALWEIQQVVDADSKIVQLSEISMDNSPNLAWPLIVTALRANSAVKLQ
EPI_ISL_413575      ------------------------------------------------------------
EPI_ISL_413588      ------------------------------------------------------------
EPI_ISL_413589      ------------------------------------------------------------
EPI_ISL_424221      ------------------------------------------------------------
EPI_ISL_424233      ------------------------------------------------------------
EPI_ISL_424236      ------------------------------------------------------------
EPI_ISL_424238      ------------------------------------------------------------
EPI_ISL_424248      ------------------------------------------------------------
EPI_ISL_424254      ------------------------------------------------------------
EPI_ISL_425548      ------------------------------------------------------------
EPI_ISL_425581      ------------------------------------------------------------
EPI_ISL_426881      ------------------------------------------------------------
EPI_ISL_426883      ------------------------------------------------------------
EPI_ISL_426888      ------------------------------------------------------------
EPI_ISL_426900      ------------------------------------------------------------
EPI_ISL_426901      ------------------------------------------------------------
EPI_ISL_426903      ------------------------------------------------------------
EPI_ISL_426904      ------------------------------------------------------------
EPI_ISL_426907      ------------------------------------------------------------
EPI_ISL_426910      ------------------------------------------------------------
```

```
ORF1ab         4141 NNELSPVALRQMSCAAGTTQTACTDDNALAYYNTTKGGRFVLALLSDLQDLKWARFPKSD
EPI_ISL_413575      ------------------------------------------------------------
EPI_ISL_413588      ------------------------------------------------------------
EPI_ISL_413589      ------------------------------------------------------------
EPI_ISL_424221      ------------------------------------------------------------
EPI_ISL_424233      ------------------------------------------------------------
EPI_ISL_424236      ------------------------------------------------------------
EPI_ISL_424238      ------------------------------------------------------------
EPI_ISL_424248      ------------------------------------------------------------
EPI_ISL_424254      ------------------------------------------------------------
EPI_ISL_425548      ------------------------------------------------------------
EPI_ISL_425581      ------------------------------------------------------------
EPI_ISL_426881      ------------------------------------------------------------
EPI_ISL_426883      ------------------------------------------------------------
EPI_ISL_426888      ------------------------------------------------------------
EPI_ISL_426900      ------------------------------------------------------------
EPI_ISL_426901      ------------------------------------------------------------
EPI_ISL_426903      ------------------------------------------------------------
EPI_ISL_426904      ------------------------------------------------------------
EPI_ISL_426907      ------------------------------------------------------------
EPI_ISL_426910      ------------------------------------------------------------
```


```
ORF1ab         4141 NNELSPVALRQMSCAAGTTQTACTDDNALAYYNTTKGGRFVLALLSDLQDLKWARFPKSD
EPI_ISL_413575      ------------------------------------------------------------
EPI_ISL_413588      ------------------------------------------------------------
EPI_ISL_413589      ------------------------------------------------------------
EPI_ISL_424221      ------------------------------------------------------------
EPI_ISL_424233      ------------------------------------------------------------
EPI_ISL_424236      ------------------------------------------------------------
EPI_ISL_424238      ------------------------------------------------------------
EPI_ISL_424248      ------------------------------------------------------------
EPI_ISL_424254      ------------------------------------------------------------
EPI_ISL_425548      ------------------------------------------------------------
EPI_ISL_425581      ------------------------------------------------------------
EPI_ISL_426881      ------------------------------------------------------------
EPI_ISL_426883      ------------------------------------------------------------
EPI_ISL_426888      ------------------------------------------------------------
EPI_ISL_426900      ------------------------------------------------------------
EPI_ISL_426901      ------------------------------------------------------------
EPI_ISL_426903      ------------------------------------------------------------
EPI_ISL_426904      ------------------------------------------------------------
EPI_ISL_426907      ------------------------------------------------------------
EPI_ISL_426910      ------------------------------------------------------------
```

```
ORF1ab          4201 GTGTIYTELEPPCRFVTDTPKGPKVKYLYFIKGLNNLNRGMVLGSLAATVRLQAGNATEV
EPI_ISL_413575       ------------------------------------------------------------
EPI_ISL_413588       ------------------------------------------------------------
EPI_ISL_413589       ------------------------------------------------------------
EPI_ISL_424221       ------------------------------------------------------------
EPI_ISL_424233       ------------------------------------------------------------
EPI_ISL_424236       ------------------------------------------------------------
EPI_ISL_424238       ------------------------------------------------------------
EPI_ISL_424248       ------------------------------------------------------------
EPI_ISL_424254       ------------------------------------------------------------
EPI_ISL_425548       ------------------------------------------------------------
EPI_ISL_425581       ------------------------------------------------------------
EPI_ISL_426881       ------------------------------------------------------------
EPI_ISL_426883       ------------------------------------------------------------
EPI_ISL_426888       ------------------------------------------------------------
EPI_ISL_426900       ------------------------------------------------------------
EPI_ISL_426901       ------------------------------------------------------------
EPI_ISL_426903       ------------------------------------------------------------
EPI_ISL_426904       ------------------------------------------------------------
EPI_ISL_426907       ------------------------------------------------------------
EPI_ISL_426910       ------------------------------------------------------------
```


```
ORF1ab          4201 GTGTIYTELEPPCRFVTDTPKGPKVKYLYFIKGLNNLNRGMVLGSLAATVRLQAGNATEV
EPI_ISL_413575       ------------------------------------------------------------
EPI_ISL_413588       ------------------------------------------------------------
EPI_ISL_413589       ------------------------------------------------------------
EPI_ISL_424221       ------------------------------------------------------------
EPI_ISL_424233       ------------------------------------------------------------
EPI_ISL_424236       ------------------------------------------------------------
EPI_ISL_424238       ------------------------------------------------------------
EPI_ISL_424248       ------------------------------------------------------------
EPI_ISL_424254       ------------------------------------------------------------
EPI_ISL_425548       ------------------------------------------------------------
EPI_ISL_425581       ------------------------------------------------------------
EPI_ISL_426881       ------------------------------------------------------------
EPI_ISL_426883       ------------------------------------------------------------
EPI_ISL_426888       ------------------------------------------------------------
EPI_ISL_426900       ------------------------------------------------------------
EPI_ISL_426901       ------------------------------------------------------------
EPI_ISL_426903       ------------------------------------------------------------
EPI_ISL_426904       ------------------------------------------------------------
EPI_ISL_426907       ------------------------------------------------------------
EPI_ISL_426910       ------------------------------------------------------------
```

```
ORF1ab         4261 PANSTVLSFCAFAVDAAKAYKDYLASGGQPITNCVKMLCTHTGTGQAITVTPEANMDQES
EPI_ISL_413575      ------------------------------------------------------------
EPI_ISL_413588      ------------------------------------------------------------
EPI_ISL_413589      ------------------------------------------------------------
EPI_ISL_424221      ------------------------------------------------------------
EPI_ISL_424233      ------------------------------------------------------------
EPI_ISL_424236      ------------------------------------------------------------
EPI_ISL_424238      ------------------------------------------------------------
EPI_ISL_424248      ------------------------------------------------------------
EPI_ISL_424254      ------------------------------------------------------------
EPI_ISL_425548      ------------------------------------------------------------
EPI_ISL_425581      ------------------------------------------------------------
EPI_ISL_426881      ------------------------------------------------------------
EPI_ISL_426883      ------------------------------------------------------------
EPI_ISL_426888      ------------------------------------------------------------
EPI_ISL_426900      ------------------------------------------------------------
EPI_ISL_426901      ------------------------------------------------------------
EPI_ISL_426903      ------------------------------------------------------------
EPI_ISL_426904      ------------------------------------------------------------
EPI_ISL_426907      ------------------------------------------------------------
EPI_ISL_426910      ------------------------------------------------------------
```


```
ORF1ab         4261 PANSTVLSFCAFAVDAAKAYKDYLASGGQPITNCVKMLCTHTGTGQAITVTPEANMDQES
EPI_ISL_413575      ------------------------------------------------------------
EPI_ISL_413588      ------------------------------------------------------------
EPI_ISL_413589      ------------------------------------------------------------
EPI_ISL_424221      ------------------------------------------------------------
EPI_ISL_424233      ------------------------------------------------------------
EPI_ISL_424236      ------------------------------------------------------------
EPI_ISL_424238      ------------------------------------------------------------
EPI_ISL_424248      ------------------------------------------------------------
EPI_ISL_424254      ------------------------------------------------------------
EPI_ISL_425548      ------------------------------------------------------------
EPI_ISL_425581      ------------------------------------------------------------
EPI_ISL_426881      ------------------------------------------------------------
EPI_ISL_426883      ------------------------------------------------------------
EPI_ISL_426888      ------------------------------------------------------------
EPI_ISL_426900      ------------------------------------------------------------
EPI_ISL_426901      ------------------------------------------------------------
EPI_ISL_426903      ------------------------------------------------------------
EPI_ISL_426904      ------------------------------------------------------------
EPI_ISL_426907      ------------------------------------------------------------
EPI_ISL_426910      ------------------------------------------------------------
```

```
ORF1ab         4321 FGGASCCLYCRCHIDHPNPKGFCDLKGKYVQIPTTCANDPVGFTLKNTVCTVCGMWKGYG
EPI_ISL_413575      ------------------------------------------------------------
EPI_ISL_413588      ------------------------------------------------------------
EPI_ISL_413589      ------------------------------------------------------------
EPI_ISL_424221      ------------------------------------------------------------
EPI_ISL_424233      ------------------------------------------------------------
EPI_ISL_424236      ------------------------------------------------------------
EPI_ISL_424238      ------------------------------------------------------------
EPI_ISL_424248      ------------------------------------------------------------
EPI_ISL_424254      ------------------------------------------------------------
EPI_ISL_425548      ------------------------------------------------------------
EPI_ISL_425581      ------------------------------------------------------------
EPI_ISL_426881      ------------------------------------------------------------
EPI_ISL_426883      ------------------------------------------------------------
EPI_ISL_426888      ------------------------------------------------------------
EPI_ISL_426900      ------------------------------------------------------------
EPI_ISL_426901      ------------------------------------------------------------
EPI_ISL_426903      ------------------------------------------------------------
EPI_ISL_426904      ------------------------------------------------------------
EPI_ISL_426907      ------------------------------------------------------------
EPI_ISL_426910      ------------------------------------------------------------
```

```
ORF1ab         4321 FGGASCCLYCRCHIDHPNPKGFCDLKGKYVQIPTTCANDPVGFTLKNTVCTVCGMWKGYG
EPI_ISL_413575      ------------------------------------------------------------
EPI_ISL_413588      ------------------------------------------------------------
EPI_ISL_413589      ------------------------------------------------------------
EPI_ISL_424221      ------------------------------------------------------------
EPI_ISL_424233      ------------------------------------------------------------
EPI_ISL_424236      ------------------------------------------------------------
EPI_ISL_424238      ------------------------------------------------------------
EPI_ISL_424248      ------------------------------------------------------------
EPI_ISL_424254      ------------------------------------------------------------
EPI_ISL_425548      ------------------------------------------------------------
EPI_ISL_425581      ------------------------------------------------------------
EPI_ISL_426881      ------------------------------------------------------------
EPI_ISL_426883      ------------------------------------------------------------
EPI_ISL_426888      ------------------------------------------------------------
EPI_ISL_426900      ------------------------------------------------------------
EPI_ISL_426901      ------------------------------------------------------------
EPI_ISL_426903      ------------------------------------------------------------
EPI_ISL_426904      ------------------------------------------------------------
EPI_ISL_426907      ------------------------------------------------------------
EPI_ISL_426910      ------------------------------------------------------------
```

```
ORF1ab         4381 CSCDQLREPMLQSADAQSFLNRVCGVSAARLTPCGTGTSTDVVYRAFDIYNDKVAGFAKF
EPI_ISL_413575      ------------------------------------------------------------
EPI_ISL_413588      ------------------------------------------------------------
EPI_ISL_413589      ------------------------------------------------------------
EPI_ISL_424221      ------------------------------------------------------------
EPI_ISL_424233      ------------------------------------------------------------
EPI_ISL_424236      ------------------------------------------------------------
EPI_ISL_424238      ------------------------------------------------------------
EPI_ISL_424248      ------------------------------------------------------------
EPI_ISL_424254      ------------------------------------------------------------
EPI_ISL_425548      ------------------------------------------------------------
EPI_ISL_425581      ------------------------------------------------------------
EPI_ISL_426881      ------------------------------------------------------------
EPI_ISL_426883      ------------------------------------------------------------
EPI_ISL_426888      ------------------------------------------------------------
EPI_ISL_426900      ------------------------------------------------------------
EPI_ISL_426901      ------------------------------------------------------------
EPI_ISL_426903      ------------------------------------------------------------
EPI_ISL_426904      ------------------------------------------------------------
EPI_ISL_426907      ------------------------------------------------------------
EPI_ISL_426910      ------------------------------------------------------------
```


```
ORF1ab         4381 CSCDQLREPMLQSADAQSFLNRVCGVSAARLTPCGTGTSTDVVYRAFDIYNDKVAGFAKF
EPI_ISL_413575      ------------------------------------------------------------
EPI_ISL_413588      ------------------------------------------------------------
EPI_ISL_413589      ------------------------------------------------------------
EPI_ISL_424221      ------------------------------------------------------------
EPI_ISL_424233      ------------------------------------------------------------
EPI_ISL_424236      ------------------------------------------------------------
EPI_ISL_424238      ------------------------------------------------------------
EPI_ISL_424248      ------------------------------------------------------------
EPI_ISL_424254      ------------------------------------------------------------
EPI_ISL_425548      ------------------------------------------------------------
EPI_ISL_425581      ------------------------------------------------------------
EPI_ISL_426881      ------------------------------------------------------------
EPI_ISL_426883      ------------------------------------------------------------
EPI_ISL_426888      ------------------------------------------------------------
EPI_ISL_426900      ------------------------------------------------------------
EPI_ISL_426901      ------------------------------------------------------------
EPI_ISL_426903      ------------------------------------------------------------
EPI_ISL_426904      ------------------------------------------------------------
EPI_ISL_426907      ------------------------------------------------------------
EPI_ISL_426910      ------------------------------------------------------------
```

```
ORF1ab         4441 LKTNCCRFQEKDEDDNLIDSYFVVKRHTFSNYQHEETIYNLLKDCPAVAKHDFFKFRIDG
EPI_ISL_413575      ------------------------------------------------------------
EPI_ISL_413588      ------------------------------------------------------------
EPI_ISL_413589      ------------------------------------------------------------
EPI_ISL_424221      ------------------------------------------------------------
EPI_ISL_424233      ------------------------------------------------------------
EPI_ISL_424236      ------------------------------------------------------------
EPI_ISL_424238      ------------------------------------------------------------
EPI_ISL_424248      ------------------------------------------------------------
EPI_ISL_424254      ------------------------------------------------------------
EPI_ISL_425548      ------------------------------------------------------------
EPI_ISL_425581      ------------------------------------------------------------
EPI_ISL_426881      ------------------------------------------------------------
EPI_ISL_426883      ------------------------------------------------------------
EPI_ISL_426888      ------------------------------------------------------------
EPI_ISL_426900      ------------------------------------------------------------
EPI_ISL_426901      ------------------------------------------------------------
EPI_ISL_426903      ------------------------------------------------------------
EPI_ISL_426904      ------------------------------------------------------------
EPI_ISL_426907      ------------------------------------------------------------
EPI_ISL_426910      ------------------------------------------------------------
```


```
ORF1ab         4441 LKTNCCRFQEKDEDDNLIDSYFVVKRHTFSNYQHEETIYNLLKDCPAVAKHDFFKFRIDG
EPI_ISL_413575      ------------------------------------------------------------
EPI_ISL_413588      ------------------------------------------------------------
EPI_ISL_413589      ------------------------------------------------------------
EPI_ISL_424221      ------------------------------------------------------------
EPI_ISL_424233      ------------------------------------------------------------
EPI_ISL_424236      ------------------------------------------------------------
EPI_ISL_424238      ------------------------------------------------------------
EPI_ISL_424248      ------------------------------------------------------------
EPI_ISL_424254      ------------------------------------------------------------
EPI_ISL_425548      ------------------------------------------------------------
EPI_ISL_425581      ------------------------------------------------------------
EPI_ISL_426881      ------------------------------------------------------------
EPI_ISL_426883      ------------------------------------------------------------
EPI_ISL_426888      ------------------------------------------------------------
EPI_ISL_426900      ------------------------------------------------------------
EPI_ISL_426901      ------------------------------------------------------------
EPI_ISL_426903      ------------------------------------------------------------
EPI_ISL_426904      ------------------------------------------------------------
EPI_ISL_426907      ------------------------------------------------------------
EPI_ISL_426910      ------------------------------------------------------------
```

```
ORF1ab         4501 DMVPHISRQRLTKYTMADLVYALRHFDEGNCDTLKEILVTYNCCDDDYFNKKDWYDFVEN
EPI_ISL_413575      ------------------------------------------------------------
EPI_ISL_413588      ------------------------------------------------------------
EPI_ISL_413589      ------------------------------------------------------------
EPI_ISL_424221      ------------------------------------------------------------
EPI_ISL_424233      ------------------------------------------------------------
EPI_ISL_424236      ------------------------------------------------------------
EPI_ISL_424238      ------------------------------------------------------------
EPI_ISL_424248      ------------------------------------------------------------
EPI_ISL_424254      ------------------------------------------------------------
EPI_ISL_425548      ------------------------------------------------------------
EPI_ISL_425581      ------------------------------------------------------------
EPI_ISL_426881      ------------------------------------------------------------
EPI_ISL_426883      ------------------------------------------------------------
EPI_ISL_426888      ------------------------------------------------------------
EPI_ISL_426900      ------------------------------------------------------------
EPI_ISL_426901      ------------------------------------------------------------
EPI_ISL_426903      ------------------------------------------------------------
EPI_ISL_426904      ------------------------------------------------------------
EPI_ISL_426907      ------------------------------------------------------------
EPI_ISL_426910      ------------------------------------------------------------
```

```
ORF1ab         4501 DMVPHISRQRLTKYTMADLVYALRHFDEGNCDTLKEILVTYNCCDDDYFNKKDWYDFVEN
EPI_ISL_413575      ------------------------------------------------------------
EPI_ISL_413588      ------------------------------------------------------------
EPI_ISL_413589      ------------------------------------------------------------
EPI_ISL_424221      ------------------------------------------------------------
EPI_ISL_424233      ------------------------------------------------------------
EPI_ISL_424236      ------------------------------------------------------------
EPI_ISL_424238      ------------------------------------------------------------
EPI_ISL_424248      ------------------------------------------------------------
EPI_ISL_424254      ------------------------------------------------------------
EPI_ISL_425548      ------------------------------------------------------------
EPI_ISL_425581      ------------------------------------------------------------
EPI_ISL_426881      ------------------------------------------------------------
EPI_ISL_426883      ------------------------------------------------------------
EPI_ISL_426888      ------------------------------------------------------------
EPI_ISL_426900      ------------------------------------------------------------
EPI_ISL_426901      ------------------------------------------------------------
EPI_ISL_426903      ------------------------------------------------------------
EPI_ISL_426904      ------------------------------------------------------------
EPI_ISL_426907      ------------------------------------------------------------
EPI_ISL_426910      ------------------------------------------------------------
```

```
ORF1ab          4561 PDILRVYANLGERVRQALLKTVQFCDAMRNAGIVGVLTLDNQDLNGNWYDFGDFIQTTPG
EPI_ISL_413575       ------------------------------------------------------------
EPI_ISL_413588       ------------------------------------------------------------
EPI_ISL_413589       ------------------------------------------------------------
EPI_ISL_424221       ------------------------------------------------------------
EPI_ISL_424233       ------------------------------------------------------------
EPI_ISL_424236       ------------------------------------------------------------
EPI_ISL_424238       ------------------------------------------------------------
EPI_ISL_424248       ------------------------------------------------------------
EPI_ISL_424254       ------------------------------------------------------------
EPI_ISL_425548       ------------------------------------------------------------
EPI_ISL_425581       ------------------------------------------------------------
EPI_ISL_426881       ------------------------------------------------------------
EPI_ISL_426883       ------------------------------------------------------------
EPI_ISL_426888       ------------------------------------------------------------
EPI_ISL_426900       ------------------------------------------------------------
EPI_ISL_426901       ------------------------------------------------------------
EPI_ISL_426903       ------------------------------------------------------------
EPI_ISL_426904       ------------------------------------------------------------
EPI_ISL_426907       ------------------------------------------------------------
EPI_ISL_426910       ------------------------------------------------------------
ORF1ab          4561 PDILRVYANLGERVRQALLKTVQFCDAMRNAGIVGVLTLDNQDLNGNWYDFGDFIQTTPG
EPI_ISL_413575       ------------------------------------------------------------
EPI_ISL_413588       ------------------------------------------------------------
EPI_ISL_413589       ------------------------------------------------------------
EPI_ISL_424221       ------------------------------------------------------------
EPI_ISL_424233       ------------------------------------------------------------
EPI_ISL_424236       ------------------------------------------------------------
EPI_ISL_424238       ------------------------------------------------------------
EPI_ISL_424248       ------------------------------------------------------------
EPI_ISL_424254       ------------------------------------------------------------
EPI_ISL_425548       ------------------------------------------------------------
EPI_ISL_425581       ------------------------------------------------------------
EPI_ISL_426881       ------------------------------------------------------------
EPI_ISL_426883       ------------------------------------------------------------
EPI_ISL_426888       ------------------------------------------------------------
EPI_ISL_426900       ------------------------------------------------------------
EPI_ISL_426901       ------------------------------------------------------------
EPI_ISL_426903       ------------------------------------------------------------
EPI_ISL_426904       ------------------------------------------------------------
EPI_ISL_426907       ------------------------------------------------------------
EPI_ISL_426910       ------------------------------------------------------------
```

```
ORF1ab         4621 SGVPVVDSYYSLLMPILTLTRALTAESHVDTDLTKPYIKWDLLKYDFTEERLKLFDRYFK
EPI_ISL_413575      ------------------------------------------------------------
EPI_ISL_413588      ------------------------------------------------------------
EPI_ISL_413589      ------------------------------------------------------------
EPI_ISL_424221      ------------------------------------------------------------
EPI_ISL_424233      ------------------------------------------------------------
EPI_ISL_424236      ------------------------------------------------------------
EPI_ISL_424238      ------------------------------------------------------------
EPI_ISL_424248      ------------------------------------------------------------
EPI_ISL_424254      ------------------------------------------------------------
EPI_ISL_425548      ------------------------------------------------------------
EPI_ISL_425581      ------------------------------------------------------------
EPI_ISL_426881      ------------------------------------------------------------
EPI_ISL_426883      ------------------------------------------------------------
EPI_ISL_426888      ------------------------------------------------------------
EPI_ISL_426900      ------------------------------------------------------------
EPI_ISL_426901      ------------------------------------------------------------
EPI_ISL_426903      ------------------------------------------------------------
EPI_ISL_426904      ------------------------------------------------------------
EPI_ISL_426907      ------------------------------------------------------------
EPI_ISL_426910      ------------------------------------------------------------
```

```
ORF1ab         4621 SGVPVVDSYYSLLMPILTLTRALTAESHVDTDLTKPYIKWDLLKYDFTEERLKLFDRYFK
EPI_ISL_413575      ------------------------------------------------------------
EPI_ISL_413588      ------------------------------------------------------------
EPI_ISL_413589      ------------------------------------------------------------
EPI_ISL_424221      ------------------------------------------------------------
EPI_ISL_424233      ------------------------------------------------------------
EPI_ISL_424236      ------------------------------------------------------------
EPI_ISL_424238      ------------------------------------------------------------
EPI_ISL_424248      ------------------------------------------------------------
EPI_ISL_424254      ------------------------------------------------------------
EPI_ISL_425548      ------------------------------------------------------------
EPI_ISL_425581      ------------------------------------------------------------
EPI_ISL_426881      ------------------------------------------------------------
EPI_ISL_426883      ------------------------------------------------------------
EPI_ISL_426888      ------------------------------------------------------------
EPI_ISL_426900      ------------------------------------------------------------
EPI_ISL_426901      ------------------------------------------------------------
EPI_ISL_426903      ------------------------------------------------------------
EPI_ISL_426904      ------------------------------------------------------------
EPI_ISL_426907      ------------------------------------------------------------
EPI_ISL_426910      ------------------------------------------------------------
```

```
ORF1ab         4681 YWDQTYHPNCVNCLDDRCILHCANFNVLFSTVFPPTSFGPLVRKIFVDGVPFVVSTGYHF
EPI_ISL_413575      ------------------------------------------------------------
EPI_ISL_413588      ------------------------------------------------------------
EPI_ISL_413589      ------------------------------------------------------------
EPI_ISL_424221      ------------------------------------------------------------
EPI_ISL_424233      ------------------------------------------------------------
EPI_ISL_424236      ------------------------------------------------------------
EPI_ISL_424238      ------------------------------------------------------------
EPI_ISL_424248      ------------------------------------------------------------
EPI_ISL_424254      ------------------------------------------------------------
EPI_ISL_425548      ------------------------------------------------------------
EPI_ISL_425581      ------------------------------------------------------------
EPI_ISL_426881      ------------------------------------------------------------
EPI_ISL_426883      ------------------------------------------------------------
EPI_ISL_426888      ------------------------------------------------------------
EPI_ISL_426900      ------------------------------------------------------------
EPI_ISL_426901      ------------------------------------------------------------
EPI_ISL_426903      ------------------------------------------------------------
EPI_ISL_426904      ------------------------------------------------------------
EPI_ISL_426907      ------------------------------------------------------------
EPI_ISL_426910      ------------------------------------------------------------
```

```
ORF1ab         4681 YWDQTYHPNCVNCLDDRCILHCANFNVLFSTVFPPTSFGPLVRKIFVDGVPFVVSTGYHF
EPI_ISL_413575      ------------------------------------------------------------
EPI_ISL_413588      ------------------------------------------------------------
EPI_ISL_413589      ------------------------------------------------------------
EPI_ISL_424221      ------------------------------------------------------------
EPI_ISL_424233      ------------------------------------------------------------
EPI_ISL_424236      ------------------------------------------------------------
EPI_ISL_424238      ------------------------------------------------------------
EPI_ISL_424248      ------------------------------------------------------------
EPI_ISL_424254      ------------------------------------------------------------
EPI_ISL_425548      ------------------------------------------------------------
EPI_ISL_425581      ------------------------------------------------------------
EPI_ISL_426881      ------------------------------------------------------------
EPI_ISL_426883      ------------------------------------------------------------
EPI_ISL_426888      ------------------------------------------------------------
EPI_ISL_426900      ------------------------------------------------------------
EPI_ISL_426901      ------------------------------------------------------------
EPI_ISL_426903      ------------------------------------------------------------
EPI_ISL_426904      ------------------------------------------------------------
EPI_ISL_426907      ------------------------------------------------------------
EPI_ISL_426910      ------------------------------------------------------------
```

```
ORF1ab         4741 RELGVVHNQDVNLHSSRLSFKELLVYAADPAMHAASGNLLLDKRTTCFSVAALTNNVAFQ
EPI_ISL_413575      ------------------------------------------------------------
EPI_ISL_413588      ------------------------------------------------------------
EPI_ISL_413589      ------------------------------------------------------------
EPI_ISL_424221      ------------------------------------------------------------
EPI_ISL_424233      ------------------------------------------------------------
EPI_ISL_424236      ------------------------------------------------------------
EPI_ISL_424238      ------------------------------------------------------------
EPI_ISL_424248      ------------------------------------------------------------
EPI_ISL_424254      ------------------------------------------------------------
EPI_ISL_425548      ------------------------------------------------------------
EPI_ISL_425581      ------------------------------------------------------------
EPI_ISL_426881      ------------------------------------------------------------
EPI_ISL_426883      ------------------------------------------------------------
EPI_ISL_426888      ------------------------------------------------------------
EPI_ISL_426900      ------------------------------------------------------------
EPI_ISL_426901      ------------------------------------------------------------
EPI_ISL_426903      ------------------------------------------------------------
EPI_ISL_426904      ------------------------------------------------------------
EPI_ISL_426907      ------------------------------------------------------------
EPI_ISL_426910      ------------------------------------------------------------
```


```
ORF1ab         4741 RELGVVHNQDVNLHSSRLSFKELLVYAADPAMHAASGNLLLDKRTTCFSVAALTNNVAFQ
EPI_ISL_413575      ------------------------------------------------------------
EPI_ISL_413588      ------------------------------------------------------------
EPI_ISL_413589      ------------------------------------------------------------
EPI_ISL_424221      ------------------------------------------------------------
EPI_ISL_424233      ------------------------------------------------------------
EPI_ISL_424236      ------------------------------------------------------------
EPI_ISL_424238      ------------------------------------------------------------
EPI_ISL_424248      ------------------------------------------------------------
EPI_ISL_424254      ------------------------------------------------------------
EPI_ISL_425548      ------------------------------------------------------------
EPI_ISL_425581      ------------------------------------------------------------
EPI_ISL_426881      ------------------------------------------------------------
EPI_ISL_426883      ------------------------------------------------------------
EPI_ISL_426888      ------------------------------------------------------------
EPI_ISL_426900      ------------------------------------------------------------
EPI_ISL_426901      ------------------------------------------------------------
EPI_ISL_426903      ------------------------------------------------------------
EPI_ISL_426904      ------------------------------------------------------------
EPI_ISL_426907      ------------------------------------------------------------
EPI_ISL_426910      ------------------------------------------------------------
```

```
ORF1ab         4801 TVKPGNFNKDFYDFAVSKGFFKEGSSVELKHFFFAQDGNAAISDYDYYRYNLPTMCDIRQ
EPI_ISL_413575      ------------------------------------------------------------
EPI_ISL_413588      ------------------------------------------------------------
EPI_ISL_413589      ------------------------------------------------------------
EPI_ISL_424221      ------------------------------------------------------------
EPI_ISL_424233      ------------------------------------------------------------
EPI_ISL_424236      ------------------------------------------------------------
EPI_ISL_424238      ------------------------------------------------------------
EPI_ISL_424248      ------------------------------------------------------------
EPI_ISL_424254      ------------------------------------------------------------
EPI_ISL_425548      ------------------------------------------------------------
EPI_ISL_425581      ------------------------------------------------------------
EPI_ISL_426881      ------------------------------------------------------------
EPI_ISL_426883      ------------------------------------------------------------
EPI_ISL_426888      ------------------------------------------------------------
EPI_ISL_426900      ------------------------------------------------------------
EPI_ISL_426901      ------------------------------------------------------------
EPI_ISL_426903      ------------------------------------------------------------
EPI_ISL_426904      ------------------------------------------------------------
EPI_ISL_426907      ------------------------------------------------------------
EPI_ISL_426910      ------------------------------------------------------------
```

```
ORF1ab         4801 TVKPGNFNKDFYDFAVSKGFFKEGSSVELKHFFFAQDGNAAISDYDYYRYNLPTMCDIRQ
EPI_ISL_413575      ------------------------------------------------------------
EPI_ISL_413588      ------------------------------------------------------------
EPI_ISL_413589      ------------------------------------------------------------
EPI_ISL_424221      ------------------------------------------------------------
EPI_ISL_424233      ------------------------------------------------------------
EPI_ISL_424236      ------------------------------------------------------------
EPI_ISL_424238      ------------------------------------------------------------
EPI_ISL_424248      ------------------------------------------------------------
EPI_ISL_424254      ------------------------------------------------------------
EPI_ISL_425548      ------------------------------------------------------------
EPI_ISL_425581      ------------------------------------------------------------
EPI_ISL_426881      ------------------------------------------------------------
EPI_ISL_426883      ------------------------------------------------------------
EPI_ISL_426888      ------------------------------------------------------------
EPI_ISL_426900      ------------------------------------------------------------
EPI_ISL_426901      ------------------------------------------------------------
EPI_ISL_426903      ------------------------------------------------------------
EPI_ISL_426904      ------------------------------------------------------------
EPI_ISL_426907      ------------------------------------------------------------
EPI_ISL_426910      ------------------------------------------------------------
```

```
ORF1ab         4861 LLFVVEVVDKYFDCYDGGCINANQVIVNNLDKSAGFPFNKWGKARLYYDSMSYEDQDALF
EPI_ISL_413575      ------------------------------------------------------------
EPI_ISL_413588      ------------------------------------------------------------
EPI_ISL_413589      ------------------------------------------------------------
EPI_ISL_424221      ------------------------------------------------------------
EPI_ISL_424233      ------------------------------------------------------------
EPI_ISL_424236      ------------------------------------------------------------
EPI_ISL_424238      ------------------------------------------------------------
EPI_ISL_424248      ------------------------------------------------------------
EPI_ISL_424254      ------------------------------------------------------------
EPI_ISL_425548      ------------------------------------------------------------
EPI_ISL_425581      ------------------------------------------------------------
EPI_ISL_426881      ------------------------------------------------------------
EPI_ISL_426883      ------------------------------------------------------------
EPI_ISL_426888      ------------------------------------------------------------
EPI_ISL_426900      ------------------------------------------------------------
EPI_ISL_426901      ------------------------------------------------------------
EPI_ISL_426903      ------------------------------------------------------------
EPI_ISL_426904      ------------------------------------------------------------
EPI_ISL_426907      ------------------------------------------------------------
EPI_ISL_426910      ------------------------------------------------------------
```

```
ORF1ab         4861 LLFVVEVVDKYFDCYDGGCINANQVIVNNLDKSAGFPFNKWGKARLYYDSMSYEDQDALF
EPI_ISL_413575      ------------------------------------------------------------
EPI_ISL_413588      ------------------------------------------------------------
EPI_ISL_413589      ------------------------------------------------------------
EPI_ISL_424221      ------------------------------------------------------------
EPI_ISL_424233      ------------------------------------------------------------
EPI_ISL_424236      ------------------------------------------------------------
EPI_ISL_424238      ------------------------------------------------------------
EPI_ISL_424248      ------------------------------------------------------------
EPI_ISL_424254      ------------------------------------------------------------
EPI_ISL_425548      ------------------------------------------------------------
EPI_ISL_425581      ------------------------------------------------------------
EPI_ISL_426881      ------------------------------------------------------------
EPI_ISL_426883      ------------------------------------------------------------
EPI_ISL_426888      ------------------------------------------------------------
EPI_ISL_426900      ------------------------------------------------------------
EPI_ISL_426901      ------------------------------------------------------------
EPI_ISL_426903      ------------------------------------------------------------
EPI_ISL_426904      ------------------------------------------------------------
EPI_ISL_426907      ------------------------------------------------------------
EPI_ISL_426910      ------------------------------------------------------------
```

```
ORF1ab         4921 AYTKRNVIPTITQMNLKYAISAKNRARTVAGVSICSTMTNRQFHQKLLKSIAATRGATVV
EPI_ISL_413575      ------------------------------------------------------------
EPI_ISL_413588      ------------------------------------------------------------
EPI_ISL_413589      ------------------------------------------------------------
EPI_ISL_424221      ------------------------------------------------------------
EPI_ISL_424233      ------------------------------------------------------------
EPI_ISL_424236      ------------------------------------------------------------
EPI_ISL_424238      ------------------------------------------------------------
EPI_ISL_424248      ------------------------------------------------------------
EPI_ISL_424254      ------------------------------------------------------------
EPI_ISL_425548      ------------------------------------------------------------
EPI_ISL_425581      ------------------------------------------------------------
EPI_ISL_426881      ------------------------------------------------------------
EPI_ISL_426883      ------------------------------------------------------------
EPI_ISL_426888      ------------------------------------------------------------
EPI_ISL_426900      ------------------------------------------------------------
EPI_ISL_426901      ------------------------------------------------------------
EPI_ISL_426903      ------------------------------------------------------------
EPI_ISL_426904      ------------------------------------------------------------
EPI_ISL_426907      ------------------------------------------------------------
EPI_ISL_426910      ------------------------------------------------------------
ORF1ab         4921 AYTKRNVIPTITQMNLKYAISAKNRARTVAGVSICSTMTNRQFHQKLLKSIAATRGATVV
EPI_ISL_413575      ------------------------------------------------------------
EPI_ISL_413588      ------------------------------------------------------------
EPI_ISL_413589      ------------------------------------------------------------
EPI_ISL_424221      ------------------------------------------------------------
EPI_ISL_424233      ------------------------------------------------------------
EPI_ISL_424236      ------------------------------------------------------------
EPI_ISL_424238      ------------------------------------------------------------
EPI_ISL_424248      ------------------------------------------------------------
EPI_ISL_424254      ------------------------------------------------------------
EPI_ISL_425548      ------------------------------------------------------------
EPI_ISL_425581      ------------------------------------------------------------
EPI_ISL_426881      ------------------------------------------------------------
EPI_ISL_426883      ------------------------------------------------------------
EPI_ISL_426888      ------------------------------------------------------------
EPI_ISL_426900      ------------------------------------------------------------
EPI_ISL_426901      ------------------------------------------------------------
EPI_ISL_426903      ------------------------------------------------------------
EPI_ISL_426904      ------------------------------------------------------------
EPI_ISL_426907      ------------------------------------------------------------
EPI_ISL_426910      ------------------------------------------------------------
```

```
ORF1ab         4981 IGTSKFYGGWHNMLKTVYSDVENPHLMGWDYPKCDRAMPNMLRIMASLVLARKHTTCCSL
EPI_ISL_413575      ------------------------------------------------------------
EPI_ISL_413588      ------------------------------------------------------------
EPI_ISL_413589      ------------------------------------------------------------
EPI_ISL_424221      ------------------------------------------------------------
EPI_ISL_424233      ------------------------------------------------------------
EPI_ISL_424236      ------------------------------------------------------------
EPI_ISL_424238      ------------------------------------------------------------
EPI_ISL_424248      ------------------------------------------------------------
EPI_ISL_424254      ------------------------------------------------------------
EPI_ISL_425548      ------------------------------------------------------------
EPI_ISL_425581      ------------------------------------------------------------
EPI_ISL_426881      ------------------------------------------------------------
EPI_ISL_426883      ------------------------------------------------------------
EPI_ISL_426888      ------------------------------------------------------------
EPI_ISL_426900      ------------------------------------------------------------
EPI_ISL_426901      ------------------------------------------------------------
EPI_ISL_426903      ------------------------------------------------------------
EPI_ISL_426904      ------------------------------------------------------------
EPI_ISL_426907      ------------------------------------------------------------
EPI_ISL_426910      ------------------------------------------------------------
```


```
ORF1ab         4981 IGTSKFYGGWHNMLKTVYSDVENPHLMGWDYPKCDRAMPNMLRIMASLVLARKHTTCCSL
EPI_ISL_413575      ------------------------------------------------------------
EPI_ISL_413588      ------------------------------------------------------------
EPI_ISL_413589      ------------------------------------------------------------
EPI_ISL_424221      ------------------------------------------------------------
EPI_ISL_424233      ------------------------------------------------------------
EPI_ISL_424236      ------------------------------------------------------------
EPI_ISL_424238      ------------------------------------------------------------
EPI_ISL_424248      ------------------------------------------------------------
EPI_ISL_424254      ------------------------------------------------------------
EPI_ISL_425548      ------------------------------------------------------------
EPI_ISL_425581      ------------------------------------------------------------
EPI_ISL_426881      ------------------------------------------------------------
EPI_ISL_426883      ------------------------------------------------------------
EPI_ISL_426888      ------------------------------------------------------------
EPI_ISL_426900      ------------------------------------------------------------
EPI_ISL_426901      ------------------------------------------------------------
EPI_ISL_426903      ------------------------------------------------------------
EPI_ISL_426904      ------------------------------------------------------------
EPI_ISL_426907      ------------------------------------------------------------
EPI_ISL_426910      ------------------------------------------------------------
```

```
ORF1ab         5041 SHRFYRLANECAQVLSEMVMCGGSLYVKPGGTSSGDATTAYANSVFNICQAVTANVNALL
EPI_ISL_413575      ------------------------------------------------------------
EPI_ISL_413588      ------------------------------------------------------------
EPI_ISL_413589      ------------------------------------------------------------
EPI_ISL_424221      ------------------------------------------------------------
EPI_ISL_424233      ------------------------------------------------------------
EPI_ISL_424236      ------------------------------------------------------------
EPI_ISL_424238      ------------------------------------------------------------
EPI_ISL_424248      ------------------------------------------------------------
EPI_ISL_424254      ------------------------------------------------------------
EPI_ISL_425548      ------------------------------------------------------------
EPI_ISL_425581      ------------------------------------------------------------
EPI_ISL_426881      ------------------------------------------------------------
EPI_ISL_426883      ------------------------------------------------------------
EPI_ISL_426888      ------------------------------------------------------------
EPI_ISL_426900      ------------------------------------------------------------
EPI_ISL_426901      ------------------------------------------------------------
EPI_ISL_426903      ------------------------------------------------------------
EPI_ISL_426904      ------------------------------------------------------------
EPI_ISL_426907      ------------------------------------------------------------
EPI_ISL_426910      ------------------------------------------------------------
ORF1ab         5041 SHRFYRLANECAQVLSEMVMCGGSLYVKPGGTSSGDATTAYANSVFNICQAVTANVNALL
EPI_ISL_413575      ------------------------------------------------------------
EPI_ISL_413588      ------------------------------------------------------------
EPI_ISL_413589      ------------------------------------------------------------
EPI_ISL_424221      ------------------------------------------------------------
EPI_ISL_424233      ------------------------------------------------------------
EPI_ISL_424236      ------------------------------------------------------------
EPI_ISL_424238      ------------------------------------------------------------
EPI_ISL_424248      ------------------------------------------------------------
EPI_ISL_424254      ------------------------------------------------------------
EPI_ISL_425548      ------------------------------------------------------------
EPI_ISL_425581      ------------------------------------------------------------
EPI_ISL_426881      ------------------------------------------------------------
EPI_ISL_426883      ------------------------------------------------------------
EPI_ISL_426888      ------------------------------------------------------------
EPI_ISL_426900      ------------------------------------------------------------
EPI_ISL_426901      ------------------------------------------------------------
EPI_ISL_426903      ------------------------------------------------------------
EPI_ISL_426904      ------------------------------------------------------------
EPI_ISL_426907      ------------------------------------------------------------
EPI_ISL_426910      ------------------------------------------------------------
```

```
ORF1ab         5101 STDGNKIADKYVRNLQHRLYECLYRNRDVDTDFVNEFYAYLRKHFSMMILSDDAVVCFNS
EPI_ISL_413575      ------------------------------------------------------------
EPI_ISL_413588      ------------------------------------------------------------
EPI_ISL_413589      ------------------------------------------------------------
EPI_ISL_424221      ------------------------------------------------------------
EPI_ISL_424233      ------------------------------------------------------------
EPI_ISL_424236      ------------------------------------------------------------
EPI_ISL_424238      ------------------------------------------------------------
EPI_ISL_424248      ------------------------------------------------------------
EPI_ISL_424254      ------------------------------------------------------------
EPI_ISL_425548      ------------------------------------------------------------
EPI_ISL_425581      ------------------------------------------------------------
EPI_ISL_426881      ------------------------------------------------------------
EPI_ISL_426883      ------------------------------------------------------------
EPI_ISL_426888      ------------------------------------------------------------
EPI_ISL_426900      ------------------------------------------------------------
EPI_ISL_426901      ------------------------------------------------------------
EPI_ISL_426903      ------------------------------------------------------------
EPI_ISL_426904      ------------------------------------------------------------
EPI_ISL_426907      ------------------------------------------------------------
EPI_ISL_426910      ------------------------------------------------------------
ORF1ab         5101 STDGNKIADKYVRNLQHRLYECLYRNRDVDTDFVNEFYAYLRKHFSMMILSDDAVVCFNS
EPI_ISL_413575      ------------------------------------------------------------
EPI_ISL_413588      ------------------------------------------------------------
EPI_ISL_413589      ------------------------------------------------------------
EPI_ISL_424221      ------------------------------------------------------------
EPI_ISL_424233      ------------------------------------------------------------
EPI_ISL_424236      ------------------------------------------------------------
EPI_ISL_424238      ------------------------------------------------------------
EPI_ISL_424248      ------------------------------------------------------------
EPI_ISL_424254      ------------------------------------------------------------
EPI_ISL_425548      ------------------------------------------------------------
EPI_ISL_425581      ------------------------------------------------------------
EPI_ISL_426881      ------------------------------------------------------------
EPI_ISL_426883      ------------------------------------------------------------
EPI_ISL_426888      ------------------------------------------------------------
EPI_ISL_426900      ------------------------------------------------------------
EPI_ISL_426901      ------------------------------------------------------------
EPI_ISL_426903      ------------------------------------------------------------
EPI_ISL_426904      ------------------------------------------------------------
EPI_ISL_426907      ------------------------------------------------------------
EPI_ISL_426910      ------------------------------------------------------------
```

```
ORF1ab          5161 TYASQGLVASIKNFKSVLYYQNNVFMSEAKCWTETDLTKGPHEFCSQHTMLVKQGDDYVY
EPI_ISL_413575       ------------------------------------------------------------
EPI_ISL_413588       ------------------------------------------------------------
EPI_ISL_413589       ------------------------------------------------------------
EPI_ISL_424221       ------------------------------------------------------------
EPI_ISL_424233       ------------------------------------------------------------
EPI_ISL_424236       ------------------------------------------------------------
EPI_ISL_424238       ------------------------------------------------------------
EPI_ISL_424248       ------------------------------------------------------------
EPI_ISL_424254       ------------------------------------------------------------
EPI_ISL_425548       ------------------------------------------------------------
EPI_ISL_425581       ------------------------------------------------------------
EPI_ISL_426881       ------------------------------------------------------------
EPI_ISL_426883       ------------------------------------------------------------
EPI_ISL_426888       ------------------------------------------------------------
EPI_ISL_426900       ------------------------------------------------------------
EPI_ISL_426901       ------------------------------------------------------------
EPI_ISL_426903       ------------------------------------------------------------
EPI_ISL_426904       ------------------------------------------------------------
EPI_ISL_426907       ------------------------------------------------------------
EPI_ISL_426910       ------------------------------------------------------------
ORF1ab          5161 TYASQGLVASIKNFKSVLYYQNNVFMSEAKCWTETDLTKGPHEFCSQHTMLVKQGDDYVY
EPI_ISL_413575       ------------------------------------------------------------
EPI_ISL_413588       ------------------------------------------------------------
EPI_ISL_413589       ------------------------------------------------------------
EPI_ISL_424221       ------------------------------------------------------------
EPI_ISL_424233       ------------------------------------------------------------
EPI_ISL_424236       ------------------------------------------------------------
EPI_ISL_424238       ------------------------------------------------------------
EPI_ISL_424248       ------------------------------------------------------------
EPI_ISL_424254       ------------------------------------------------------------
EPI_ISL_425548       ------------------------------------------------------------
EPI_ISL_425581       ------------------------------------------------------------
EPI_ISL_426881       ------------------------------------------------------------
EPI_ISL_426883       ------------------------------------------------------------
EPI_ISL_426888       ------------------------------------------------------------
EPI_ISL_426900       ------------------------------------------------------------
EPI_ISL_426901       ------------------------------------------------------------
EPI_ISL_426903       ------------------------------------------------------------
EPI_ISL_426904       ------------------------------------------------------------
EPI_ISL_426907       ------------------------------------------------------------
EPI_ISL_426910       ------------------------------------------------------------
```

```
ORF1ab         5221 LPYPDPSRILGAGCFVDDIVKTDGTLMIERFVSLAIDAYPLTKHPNQEYADVFHLYLQYI
EPI_ISL_413575      ------------------------------------------------------------
EPI_ISL_413588      ------------------------------------------------------------
EPI_ISL_413589      ------------------------------------------------------------
EPI_ISL_424221      ------------------------------------------------------------
EPI_ISL_424233      ------------------------------------------------------------
EPI_ISL_424236      ------------------------------------------------------------
EPI_ISL_424238      ------------------------------------------------------------
EPI_ISL_424248      ------------------------------------------------------------
EPI_ISL_424254      ------------------------------------------------------------
EPI_ISL_425548      ------------------------------------------------------------
EPI_ISL_425581      ------------------------------------------------------------
EPI_ISL_426881      ------------------------------------------------------------
EPI_ISL_426883      ------------------------------------------------------------
EPI_ISL_426888      ------------------------------------------------------------
EPI_ISL_426900      ------------------------------------------------------------
EPI_ISL_426901      ------------------------------------------------------------
EPI_ISL_426903      ------------------------------------------------------------
EPI_ISL_426904      ------------------------------------------------------------
EPI_ISL_426907      ------------------------------------------------------------
EPI_ISL_426910      ------------------------------------------------------------
```

```
ORF1ab         5221 LPYPDPSRILGAGCFVDDIVKTDGTLMIERFVSLAIDAYPLTKHPNQEYADVFHLYLQYI
EPI_ISL_413575      ------------------------------------------------------------
EPI_ISL_413588      ------------------------------------------------------------
EPI_ISL_413589      ------------------------------------------------------------
EPI_ISL_424221      ------------------------------------------------------------
EPI_ISL_424233      ------------------------------------------------------------
EPI_ISL_424236      ------------------------------------------------------------
EPI_ISL_424238      ------------------------------------------------------------
EPI_ISL_424248      ------------------------------------------------------------
EPI_ISL_424254      ------------------------------------------------------------
EPI_ISL_425548      ------------------------------------------------------------
EPI_ISL_425581      ------------------------------------------------------------
EPI_ISL_426881      ------------------------------------------------------------
EPI_ISL_426883      ------------------------------------------------------------
EPI_ISL_426888      ------------------------------------------------------------
EPI_ISL_426900      ------------------------------------------------------------
EPI_ISL_426901      ------------------------------------------------------------
EPI_ISL_426903      ------------------------------------------------------------
EPI_ISL_426904      ------------------------------------------------------------
EPI_ISL_426907      ------------------------------------------------------------
EPI_ISL_426910      ------------------------------------------------------------
```

```
ORF1ab         5281 RKLHDELTGHMLDMYSVMLTNDNTSRYWEPEFYEAMYTPHTVLQAVGACVLCNSQTSLRC
EPI_ISL_413575      ------------------------------------------------------------
EPI_ISL_413588      ------------------------------------------------------------
EPI_ISL_413589      ------------------------------------------------------------
EPI_ISL_424221      ------------------------------------------------------------
EPI_ISL_424233      ------------------------------------------------------------
EPI_ISL_424236      ------------------------------------------------------------
EPI_ISL_424238      ------------------------------------------------------------
EPI_ISL_424248      ------------------------------------------------------------
EPI_ISL_424254      ------------------------------------------------------------
EPI_ISL_425548      ------------------------------------------------------------
EPI_ISL_425581      ------------------------------------------------------------
EPI_ISL_426881      ------------------------------------------------------------
EPI_ISL_426883      ------------------------------------------------------------
EPI_ISL_426888      ------------------------------------------------------------
EPI_ISL_426900      ------------------------------------------------------------
EPI_ISL_426901      ------------------------------------------------------------
EPI_ISL_426903      ------------------------------------------------------------
EPI_ISL_426904      ------------------------------------------------------------
EPI_ISL_426907      ------------------------------------------------------------
EPI_ISL_426910      ------------------------------------------------------------
```

```
ORF1ab         5281 RKLHDELTGHMLDMYSVMLTNDNTSRYWEPEFYEAMYTPHTVLQAVGACVLCNSQTSLRC
EPI_ISL_413575      ------------------------------------------------------------
EPI_ISL_413588      ------------------------------------------------------------
EPI_ISL_413589      ------------------------------------------------------------
EPI_ISL_424221      ------------------------------------------------------------
EPI_ISL_424233      ------------------------------------------------------------
EPI_ISL_424236      ------------------------------------------------------------
EPI_ISL_424238      ------------------------------------------------------------
EPI_ISL_424248      ------------------------------------------------------------
EPI_ISL_424254      ------------------------------------------------------------
EPI_ISL_425548      ------------------------------------------------------------
EPI_ISL_425581      ------------------------------------------------------------
EPI_ISL_426881      ------------------------------------------------------------
EPI_ISL_426883      ------------------------------------------------------------
EPI_ISL_426888      ------------------------------------------------------------
EPI_ISL_426900      ------------------------------------------------------------
EPI_ISL_426901      ------------------------------------------------------------
EPI_ISL_426903      ------------------------------------------------------------
EPI_ISL_426904      ------------------------------------------------------------
EPI_ISL_426907      ------------------------------------------------------------
EPI_ISL_426910      ------------------------------------------------------------
```

```
ORF1ab          5341 GACIRRPFLCCKCCYDHVISTSHKLVLSVNPYVCNAPGCDVTDVTQLYLGGMSYYCKSHK
EPI_ISL_413575       ------------------------------------------------------------
EPI_ISL_413588       ------------------------------------------------------------
EPI_ISL_413589       ------------------------------------------------------------
EPI_ISL_424221       ------------------------------------------------------------
EPI_ISL_424233       ------------------------------------------------------------
EPI_ISL_424236       ------------------------------------------------------------
EPI_ISL_424238       ------------------------------------------------------------
EPI_ISL_424248       ------------------------------------------------------------
EPI_ISL_424254       ------------------------------------------------------------
EPI_ISL_425548       ------------------------------------------------------------
EPI_ISL_425581       ------------------------------------------------------------
EPI_ISL_426881       ------------------------------------------------------------
EPI_ISL_426883       ------------------------------------------------------------
EPI_ISL_426888       ------------------------------------------------------------
EPI_ISL_426900       ------------------------------------------------------------
EPI_ISL_426901       ------------------------------------------------------------
EPI_ISL_426903       ------------------------------------------------------------
EPI_ISL_426904       ------------------------------------------------------------
EPI_ISL_426907       ------------------------------------------------------------
EPI_ISL_426910       ------------------------------------------------------------
```

```
ORF1ab          5341 GACIRRPFLCCKCCYDHVISTSHKLVLSVNPYVCNAPGCDVTDVTQLYLGGMSYYCKSHK
EPI_ISL_413575       ------------------------------------------------------------
EPI_ISL_413588       ------------------------------------------------------------
EPI_ISL_413589       ------------------------------------------------------------
EPI_ISL_424221       ------------------------------------------------------------
EPI_ISL_424233       ------------------------------------------------------------
EPI_ISL_424236       ------------------------------------------------------------
EPI_ISL_424238       ------------------------------------------------------------
EPI_ISL_424248       ------------------------------------------------------------
EPI_ISL_424254       ------------------------------------------------------------
EPI_ISL_425548       ------------------------------------------------------------
EPI_ISL_425581       ------------------------------------------------------------
EPI_ISL_426881       ------------------------------------------------------------
EPI_ISL_426883       ------------------------------------------------------------
EPI_ISL_426888       ------------------------------------------------------------
EPI_ISL_426900       ------------------------------------------------------------
EPI_ISL_426901       ------------------------------------------------------------
EPI_ISL_426903       ------------------------------------------------------------
EPI_ISL_426904       ------------------------------------------------------------
EPI_ISL_426907       ------------------------------------------------------------
EPI_ISL_426910       ------------------------------------------------------------
```

```
ORF1ab          5401 PPISFPLCANGQVFGLYKNTCVGSDNVTDFNAIATCDWTNAGDYILANTCTERLKLFAAE
EPI_ISL_413575       ------------------------------------------------------------
EPI_ISL_413588       ------------------------------------------------------------
EPI_ISL_413589       ------------------------------------------------------------
EPI_ISL_424221       ------------------------------------------------------------
EPI_ISL_424233       ------------------------------------------------------------
EPI_ISL_424236       ------------------------------------------------------------
EPI_ISL_424238       ------------------------------------------------------------
EPI_ISL_424248       ------------------------------------------------------------
EPI_ISL_424254       ------------------------------------------------------------
EPI_ISL_425548       ------------------------------------------------------------
EPI_ISL_425581       ------------------------------------------------------------
EPI_ISL_426881       ------------------------------------------------------------
EPI_ISL_426883       ------------------------------------------------------------
EPI_ISL_426888       ------------------------------------------------------------
EPI_ISL_426900       ------------------------------------------------------------
EPI_ISL_426901       ------------------------------------------------------------
EPI_ISL_426903       ------------------------------------------------------------
EPI_ISL_426904       ------------------------------------------------------------
EPI_ISL_426907       ------------------------------------------------------------
EPI_ISL_426910       ------------------------------------------------------------
```

```
ORF1ab          5401 PPISFPLCANGQVFGLYKNTCVGSDNVTDFNAIATCDWTNAGDYILANTCTERLKLFAAE
EPI_ISL_413575       ------------------------------------------------------------
EPI_ISL_413588       ------------------------------------------------------------
EPI_ISL_413589       ------------------------------------------------------------
EPI_ISL_424221       ------------------------------------------------------------
EPI_ISL_424233       ------------------------------------------------------------
EPI_ISL_424236       ------------------------------------------------------------
EPI_ISL_424238       ------------------------------------------------------------
EPI_ISL_424248       ------------------------------------------------------------
EPI_ISL_424254       ------------------------------------------------------------
EPI_ISL_425548       ------------------------------------------------------------
EPI_ISL_425581       ------------------------------------------------------------
EPI_ISL_426881       ------------------------------------------------------------
EPI_ISL_426883       ------------------------------------------------------------
EPI_ISL_426888       ------------------------------------------------------------
EPI_ISL_426900       ------------------------------------------------------------
EPI_ISL_426901       ------------------------------------------------------------
EPI_ISL_426903       ------------------------------------------------------------
EPI_ISL_426904       ------------------------------------------------------------
EPI_ISL_426907       ------------------------------------------------------------
EPI_ISL_426910       ------------------------------------------------------------
```

```
ORF1ab         5461 TLKATEETFKLSYGIATVREVLSDRELHLSWEVGKPRPPLNRNYVFTGYRVTKNSKVQIG
EPI_ISL_413575      ------------------------------------------------------------
EPI_ISL_413588      ------------------------------------------------------------
EPI_ISL_413589      ------------------------------------------------------------
EPI_ISL_424221      ------------------------------------------------------------
EPI_ISL_424233      ------------------------------------------------------------
EPI_ISL_424236      ------------------------------------------------------------
EPI_ISL_424238      ------------------------------------------------------------
EPI_ISL_424248      ------------------------------------------------------------
EPI_ISL_424254      ------------------------------------------------------------
EPI_ISL_425548      ------------------------------------------------------------
EPI_ISL_425581      ------------------------------------------------------------
EPI_ISL_426881      ------------------------------------------------------------
EPI_ISL_426883      ------------------------------------------------------------
EPI_ISL_426888      ------------------------------------------------------------
EPI_ISL_426900      ------------------------------------------------------------
EPI_ISL_426901      ------------------------------------------------------------
EPI_ISL_426903      ------------------------------------------------------------
EPI_ISL_426904      ------------------------------------------------------------
EPI_ISL_426907      ------------------------------------------------------------
EPI_ISL_426910      ------------------------------------------------------------
```

```
ORF1ab         5461 TLKATEETFKLSYGIATVREVLSDRELHLSWEVGKPRPPLNRNYVFTGYRVTKNSKVQIG
EPI_ISL_413575      ------------------------------------------------------------
EPI_ISL_413588      ------------------------------------------------------------
EPI_ISL_413589      ------------------------------------------------------------
EPI_ISL_424221      ------------------------------------------------------------
EPI_ISL_424233      ------------------------------------------------------------
EPI_ISL_424236      ------------------------------------------------------------
EPI_ISL_424238      ------------------------------------------------------------
EPI_ISL_424248      ------------------------------------------------------------
EPI_ISL_424254      ------------------------------------------------------------
EPI_ISL_425548      ------------------------------------------------------------
EPI_ISL_425581      ------------------------------------------------------------
EPI_ISL_426881      ------------------------------------------------------------
EPI_ISL_426883      ------------------------------------------------------------
EPI_ISL_426888      ------------------------------------------------------------
EPI_ISL_426900      ------------------------------------------------------------
EPI_ISL_426901      ------------------------------------------------------------
EPI_ISL_426903      ------------------------------------------------------------
EPI_ISL_426904      ------------------------------------------------------------
EPI_ISL_426907      ------------------------------------------------------------
EPI_ISL_426910      ------------------------------------------------------------
```

```
ORF1ab         5521 EYTFEKGDYGDAVVYRGTTTYKLNVGDYFVLTSHTVMPLSAPTLVPQEHYVRITGLYPTL
EPI_ISL_413575      ------------------------------------------------------------
EPI_ISL_413588      ------------------------------------------------------------
EPI_ISL_413589      ------------------------------------------------------------
EPI_ISL_424221      ------------------------------------------------------------
EPI_ISL_424233      ------------------------------------------------------------
EPI_ISL_424236      ------------------------------------------------------------
EPI_ISL_424238      ------------------------------------------------------------
EPI_ISL_424248      ------------------------------------------------------------
EPI_ISL_424254      ------------------------------------------------------------
EPI_ISL_425548      ------------------------------------------------------------
EPI_ISL_425581      ------------------------------------------------------------
EPI_ISL_426881      ------------------------------------------------------------
EPI_ISL_426883      ------------------------------------------------------------
EPI_ISL_426888      ------------------------------------------------------------
EPI_ISL_426900      ------------------------------------------------------------
EPI_ISL_426901      ------------------------------------------------------------
EPI_ISL_426903      ------------------------------------------------------------
EPI_ISL_426904      ------------------------------------------------------------
EPI_ISL_426907      ------------------------------------------------------------
EPI_ISL_426910      ------------------------------------------------------------
```

```
ORF1ab         5521 EYTFEKGDYGDAVVYRGTTTYKLNVGDYFVLTSHTVMPLSAPTLVPQEHYVRITGLYPTL
EPI_ISL_413575      ------------------------------------------------------------
EPI_ISL_413588      ------------------------------------------------------------
EPI_ISL_413589      ------------------------------------------------------------
EPI_ISL_424221      ------------------------------------------------------------
EPI_ISL_424233      ------------------------------------------------------------
EPI_ISL_424236      ------------------------------------------------------------
EPI_ISL_424238      ------------------------------------------------------------
EPI_ISL_424248      ------------------------------------------------------------
EPI_ISL_424254      ------------------------------------------------------------
EPI_ISL_425548      ------------------------------------------------------------
EPI_ISL_425581      ------------------------------------------------------------
EPI_ISL_426881      ------------------------------------------------------------
EPI_ISL_426883      ------------------------------------------------------------
EPI_ISL_426888      ------------------------------------------------------------
EPI_ISL_426900      ------------------------------------------------------------
EPI_ISL_426901      ------------------------------------------------------------
EPI_ISL_426903      ------------------------------------------------------------
EPI_ISL_426904      ------------------------------------------------------------
EPI_ISL_426907      ------------------------------------------------------------
EPI_ISL_426910      ------------------------------------------------------------
```

```
ORF1ab         5581 NISDEFSSNVANYQKVGMQKYSTLQGPPGTGKSHFAIGLALYYPSARIVYTACSHAAVDA
EPI_ISL_413575      ------------------------------------------------------------
EPI_ISL_413588      ------------------------------------------------------------
EPI_ISL_413589      ------------------------------------------------------------
EPI_ISL_424221      ------------------------------------------------------------
EPI_ISL_424233      ------------------------------------------------------------
EPI_ISL_424236      ------------------------------------------------------------
EPI_ISL_424238      ------------------------------------------------------------
EPI_ISL_424248      ------------------------------------------------------------
EPI_ISL_424254      ------------------------------------------------------------
EPI_ISL_425548      ------------------------------------------------------------
EPI_ISL_425581      ------------------------------------------------------------
EPI_ISL_426881      ------------------------------------------------------------
EPI_ISL_426883      ------------------------------------------------------------
EPI_ISL_426888      ------------------------------------------------------------
EPI_ISL_426900      ------------------------------------------------------------
EPI_ISL_426901      ------------------------------------------------------------
EPI_ISL_426903      ------------------------------------------------------------
EPI_ISL_426904      ------------------------------------------------------------
EPI_ISL_426907      ------------------------------------------------------------
EPI_ISL_426910      ------------------------------------------------------------
```


```
ORF1ab         5581 NISDEFSSNVANYQKVGMQKYSTLQGPPGTGKSHFAIGLALYYPSARIVYTACSHAAVDA
EPI_ISL_413575      ------------------------------------------------------------
EPI_ISL_413588      ------------------------------------------------------------
EPI_ISL_413589      ------------------------------------------------------------
EPI_ISL_424221      ------------------------------------------------------------
EPI_ISL_424233      ------------------------------------------------------------
EPI_ISL_424236      ------------------------------------------------------------
EPI_ISL_424238      ------------------------------------------------------------
EPI_ISL_424248      ------------------------------------------------------------
EPI_ISL_424254      ------------------------------------------------------------
EPI_ISL_425548      ------------------------------------------------------------
EPI_ISL_425581      ------------------------------------------------------------
EPI_ISL_426881      ------------------------------------------------------------
EPI_ISL_426883      ------------------------------------------------------------
EPI_ISL_426888      ------------------------------------------------------------
EPI_ISL_426900      ------------------------------------------------------------
EPI_ISL_426901      ------------------------------------------------------------
EPI_ISL_426903      ------------------------------------------------------------
EPI_ISL_426904      ------------------------------------------------------------
EPI_ISL_426907      ------------------------------------------------------------
EPI_ISL_426910      ------------------------------------------------------------
```

```
ORF1ab         5641 LCEKALKYLPIDKCSRIIPARARVECFDKFKVNSTLEQYVFCTVNALPETTADIVVFDEI
EPI_ISL_413575      ------------------------------------------------------------
EPI_ISL_413588      ------------------------------------------------------------
EPI_ISL_413589      ------------------------------------------------------------
EPI_ISL_424221      ------------------------------------------------------------
EPI_ISL_424233      ------------------------------------------------------------
EPI_ISL_424236      ------------------------------------------------------------
EPI_ISL_424238      ------------------------------------------------------------
EPI_ISL_424248      ------------------------------------------------------------
EPI_ISL_424254      ------------------------------------------------------------
EPI_ISL_425548      ------------------------------------------------------------
EPI_ISL_425581      ------------------------------------------------------------
EPI_ISL_426881      ------------------------------------------------------------
EPI_ISL_426883      ------------------------------------------------------------
EPI_ISL_426888      ------------------------------------------------------------
EPI_ISL_426900      ------------------------------------------------------------
EPI_ISL_426901      ------------------------------------------------------------
EPI_ISL_426903      ------------------------------------------------------------
EPI_ISL_426904      ------------------------------------------------------------
EPI_ISL_426907      ------------------------------------------------------------
EPI_ISL_426910      ------------------------------------------------------------
```

```
ORF1ab         5641 LCEKALKYLPIDKCSRIIPARARVECFDKFKVNSTLEQYVFCTVNALPETTADIVVFDEI
EPI_ISL_413575      ------------------------------------------------------------
EPI_ISL_413588      ------------------------------------------------------------
EPI_ISL_413589      ------------------------------------------------------------
EPI_ISL_424221      ------------------------------------------------------------
EPI_ISL_424233      ------------------------------------------------------------
EPI_ISL_424236      ------------------------------------------------------------
EPI_ISL_424238      ------------------------------------------------------------
EPI_ISL_424248      ------------------------------------------------------------
EPI_ISL_424254      ------------------------------------------------------------
EPI_ISL_425548      ------------------------------------------------------------
EPI_ISL_425581      ------------------------------------------------------------
EPI_ISL_426881      ------------------------------------------------------------
EPI_ISL_426883      ------------------------------------------------------------
EPI_ISL_426888      ------------------------------------------------------------
EPI_ISL_426900      ------------------------------------------------------------
EPI_ISL_426901      ------------------------------------------------------------
EPI_ISL_426903      ------------------------------------------------------------
EPI_ISL_426904      ------------------------------------------------------------
EPI_ISL_426907      ------------------------------------------------------------
EPI_ISL_426910      ------------------------------------------------------------
```

```
ORF1ab         5701 SMATNYDLSVVNARLRAKHYVYIGDPAQLPAPRTLLTKGTLEPEYFNSVCRLMKTIGPDM
EPI_ISL_413575      ------------------------------------------------------------
EPI_ISL_413588      ------------------------------------------------------------
EPI_ISL_413589      ------------------------------------------------------------
EPI_ISL_424221      ------------------------------------------------------------
EPI_ISL_424233      ------------------------------------------------------------
EPI_ISL_424236      ------------------------------------------------------------
EPI_ISL_424238      ------------------------------------------------------------
EPI_ISL_424248      ------------------------------------------------------------
EPI_ISL_424254      ------------------------------------------------------------
EPI_ISL_425548      ------------------------------------------------------------
EPI_ISL_425581      ------------------------------------------------------------
EPI_ISL_426881      ------------------------------------------------------------
EPI_ISL_426883      ------------------------------------------------------------
EPI_ISL_426888      ------------------------------------------------------------
EPI_ISL_426900      ------------------------------------------------------------
EPI_ISL_426901      ------------------------------------------------------------
EPI_ISL_426903      ------------------------------------------------------------
EPI_ISL_426904      ------------------------------------------------------------
EPI_ISL_426907      ------------------------------------------------------------
EPI_ISL_426910      ------------------------------------------------------------
ORF1ab         5701 SMATNYDLSVVNARLRAKHYVYIGDPAQLPAPRTLLTKGTLEPEYFNSVCRLMKTIGPDM
EPI_ISL_413575      ------------------------------------------------------------
EPI_ISL_413588      ------------------------------------------------------------
EPI_ISL_413589      ------------------------------------------------------------
EPI_ISL_424221      ------------------------------------------------------------
EPI_ISL_424233      ------------------------------------------------------------
EPI_ISL_424236      ------------------------------------------------------------
EPI_ISL_424238      ------------------------------------------------------------
EPI_ISL_424248      ------------------------------------------------------------
EPI_ISL_424254      ------------------------------------------------------------
EPI_ISL_425548      ------------------------------------------------------------
EPI_ISL_425581      ------------------------------------------------------------
EPI_ISL_426881      ------------------------------------------------------------
EPI_ISL_426883      ------------------------------------------------------------
EPI_ISL_426888      ------------------------------------------------------------
EPI_ISL_426900      ------------------------------------------------------------
EPI_ISL_426901      ------------------------------------------------------------
EPI_ISL_426903      ------------------------------------------------------------
EPI_ISL_426904      ------------------------------------------------------------
EPI_ISL_426907      ------------------------------------------------------------
EPI_ISL_426910      ------------------------------------------------------------
```

```
ORF1ab          5761 FLGTCRRCPAEIVDTVSALVYDNKLKAHKDKSAQCFKMFYKGVITHDVSSAINRPQIGVV
EPI_ISL_413575       ------------------------------------------------------------
EPI_ISL_413588       ------------------------------------------------------------
EPI_ISL_413589       ------------------------------------------------------------
EPI_ISL_424221       ------------------------------------------------------------
EPI_ISL_424233       ------------------------------------------------------------
EPI_ISL_424236       ------------------------------------------------------------
EPI_ISL_424238       ------------------------------------------------------------
EPI_ISL_424248       ------------------------------------------------------------
EPI_ISL_424254       ------------------------------------------------------------
EPI_ISL_425548       ------------------------------------------------------------
EPI_ISL_425581       ------------------------------------------------------------
EPI_ISL_426881       ------------------------------------------------------------
EPI_ISL_426883       ------------------------------------------------------------
EPI_ISL_426888       ------------------------------------------------------------
EPI_ISL_426900       ------------------------------------------------------------
EPI_ISL_426901       ------------------------------------------------------------
EPI_ISL_426903       ------------------------------------------------------------
EPI_ISL_426904       ------------------------------------------------------------
EPI_ISL_426907       ------------------------------------------------------------
EPI_ISL_426910       ------------------------------------------------------------
ORF1ab          5761 FLGTCRRCPAEIVDTVSALVYDNKLKAHKDKSAQCFKMFYKGVITHDVSSAINRPQIGVV
EPI_ISL_413575       ------------------------------------------------------------
EPI_ISL_413588       ------------------------------------------------------------
EPI_ISL_413589       ------------------------------------------------------------
EPI_ISL_424221       ------------------------------------------------------------
EPI_ISL_424233       ------------------------------------------------------------
EPI_ISL_424236       ------------------------------------------------------------
EPI_ISL_424238       ------------------------------------------------------------
EPI_ISL_424248       ------------------------------------------------------------
EPI_ISL_424254       ------------------------------------------------------------
EPI_ISL_425548       ------------------------------------------------------------
EPI_ISL_425581       ------------------------------------------------------------
EPI_ISL_426881       ------------------------------------------------------------
EPI_ISL_426883       ------------------------------------------------------------
EPI_ISL_426888       ------------------------------------------------------------
EPI_ISL_426900       ------------------------------------------------------------
EPI_ISL_426901       ------------------------------------------------------------
EPI_ISL_426903       ------------------------------------------------------------
EPI_ISL_426904       ------------------------------------------------------------
EPI_ISL_426907       ------------------------------------------------------------
EPI_ISL_426910       ------------------------------------------------------------
```

```
ORF1ab          5821 REFLTRNPAWRKAVFISPYNSQNAVASKILGLPTQTVDSSQGSEYDYVIFTQTTETAHSC
EPI_ISL_413575       ------------------------------------------------------------
EPI_ISL_413588       ------------------------------------------------------------
EPI_ISL_413589       ------------------------------------------------------------
EPI_ISL_424221       ------------------------------------------------------------
EPI_ISL_424233       ------------------------------------------------------------
EPI_ISL_424236       ------------------------------------------------------------
EPI_ISL_424238       ------------------------------------------------------------
EPI_ISL_424248       ------------------------------------------------------------
EPI_ISL_424254       ------------------------------------------------------------
EPI_ISL_425548       ------------------------------------------------------------
EPI_ISL_425581       ------------------------------------------------------------
EPI_ISL_426881       ------------------------------------------------------------
EPI_ISL_426883       ------------------------------------------------------------
EPI_ISL_426888       ------------------------------------------------------------
EPI_ISL_426900       ------------------------------------------------------------
EPI_ISL_426901       ------------------------------------------------------------
EPI_ISL_426903       ------------------------------------------------------------
EPI_ISL_426904       ------------------------------------------------------------
EPI_ISL_426907       ------------------------------------------------------------
EPI_ISL_426910       ------------------------------------------------------------
ORF1ab          5821 REFLTRNPAWRKAVFISPYNSQNAVASKILGLPTQTVDSSQGSEYDYVIFTQTTETAHSC
EPI_ISL_413575       ------------------------------------------------------------
EPI_ISL_413588       ------------------------------------------------------------
EPI_ISL_413589       ------------------------------------------------------------
EPI_ISL_424221       ------------------------------------------------------------
EPI_ISL_424233       ------------------------------------------------------------
EPI_ISL_424236       ------------------------------------------------------------
EPI_ISL_424238       ------------------------------------------------------------
EPI_ISL_424248       ------------------------------------------------------------
EPI_ISL_424254       ------------------------------------------------------------
EPI_ISL_425548       ------------------------------------------------------------
EPI_ISL_425581       ------------------------------------------------------------
EPI_ISL_426881       ------------------------------------------------------------
EPI_ISL_426883       ------------------------------------------------------------
EPI_ISL_426888       ------------------------------------------------------------
EPI_ISL_426900       ------------------------------------------------------------
EPI_ISL_426901       ------------------------------------------------------------
EPI_ISL_426903       ------------------------------------------------------------
EPI_ISL_426904       ------------------------------------------------------------
EPI_ISL_426907       ------------------------------------------------------------
EPI_ISL_426910       ------------------------------------------------------------
```

```
ORF1ab          5881 NVNRFNVAITRAKVGILCIMSDRDLYDKLQFTSLEIPRRNVATLQAENVTGLFKDCSKVI
EPI_ISL_413575       ------------------------------------------------------------
EPI_ISL_413588       ------------------------------------------------------------
EPI_ISL_413589       ------------------------------------------------------------
EPI_ISL_424221       ------------------------------------------------------------
EPI_ISL_424233       ------------------------------------------------------------
EPI_ISL_424236       ------------------------------------------------------------
EPI_ISL_424238       ------------------------------------------------------------
EPI_ISL_424248       ------------------------------------------------------------
EPI_ISL_424254       ------------------------------------------------------------
EPI_ISL_425548       ------------------------------------------------------------
EPI_ISL_425581       ------------------------------------------------------------
EPI_ISL_426881       ------------------------------------------------------------
EPI_ISL_426883       ------------------------------------------------------------
EPI_ISL_426888       ------------------------------------------------------------
EPI_ISL_426900       ------------------------------------------------------------
EPI_ISL_426901       ------------------------------------------------------------
EPI_ISL_426903       ------------------------------------------------------------
EPI_ISL_426904       ------------------------------------------------------------
EPI_ISL_426907       ------------------------------------------------------------
EPI_ISL_426910       ------------------------------------------------------------
```

```
ORF1ab          5881 NVNRFNVAITRAKVGILCIMSDRDLYDKLQFTSLEIPRRNVATLQAENVTGLFKDCSKVI
EPI_ISL_413575       ------------------------------------------------------------
EPI_ISL_413588       ------------------------------------------------------------
EPI_ISL_413589       ------------------------------------------------------------
EPI_ISL_424221       ------------------------------------------------------------
EPI_ISL_424233       ------------------------------------------------------------
EPI_ISL_424236       ------------------------------------------------------------
EPI_ISL_424238       ------------------------------------------------------------
EPI_ISL_424248       ------------------------------------------------------------
EPI_ISL_424254       ------------------------------------------------------------
EPI_ISL_425548       ------------------------------------------------------------
EPI_ISL_425581       ------------------------------------------------------------
EPI_ISL_426881       ------------------------------------------------------------
EPI_ISL_426883       ------------------------------------------------------------
EPI_ISL_426888       ------------------------------------------------------------
EPI_ISL_426900       ------------------------------------------------------------
EPI_ISL_426901       ------------------------------------------------------------
EPI_ISL_426903       ------------------------------------------------------------
EPI_ISL_426904       ------------------------------------------------------------
EPI_ISL_426907       ------------------------------------------------------------
EPI_ISL_426910       ------------------------------------------------------------
```

```
ORF1ab         5941 TGLHPTQAPTHLSVDTKFKTEGLCVDIPGIPKDMTYRRLISMMGFKMNYQVNGYPNMFIT
EPI_ISL_413575      ------------------------------------------------------------
EPI_ISL_413588      ------------------------------------------------------------
EPI_ISL_413589      ------------------------------------------------------------
EPI_ISL_424221      ------------------------------------------------------------
EPI_ISL_424233      ------------------------------------------------------------
EPI_ISL_424236      ------------------------------------------------------------
EPI_ISL_424238      ------------------------------------------------------------
EPI_ISL_424248      ------------------------------------------------------------
EPI_ISL_424254      ------------------------------------------------------------
EPI_ISL_425548      ------------------------------------------------------------
EPI_ISL_425581      ------------------------------------------------------------
EPI_ISL_426881      ------------------------------------------------------------
EPI_ISL_426883      ------------------------------------------------------------
EPI_ISL_426888      ------------------------------------------------------------
EPI_ISL_426900      ------------------------------------------------------------
EPI_ISL_426901      ------------------------------------------------------------
EPI_ISL_426903      ------------------------------------------------------------
EPI_ISL_426904      ------------------------------------------------------------
EPI_ISL_426907      ------------------------------------------------------------
EPI_ISL_426910      ------------------------------------------------------------
ORF1ab         5941 TGLHPTQAPTHLSVDTKFKTEGLCVDIPGIPKDMTYRRLISMMGFKMNYQVNGYPNMFIT
EPI_ISL_413575      ------------------------------------------------------------
EPI_ISL_413588      ------------------------------------------------------------
EPI_ISL_413589      ------------------------------------------------------------
EPI_ISL_424221      ------------------------------------------------------------
EPI_ISL_424233      ------------------------------------------------------------
EPI_ISL_424236      ------------------------------------------------------------
EPI_ISL_424238      ------------------------------------------------------------
EPI_ISL_424248      ------------------------------------------------------------
EPI_ISL_424254      ------------------------------------------------------------
EPI_ISL_425548      ------------------------------------------------------------
EPI_ISL_425581      ------------------------------------------------------------
EPI_ISL_426881      ------------------------------------------------------------
EPI_ISL_426883      ------------------------------------------------------------
EPI_ISL_426888      ------------------------------------------------------------
EPI_ISL_426900      ------------------------------------------------------------
EPI_ISL_426901      ------------------------------------------------------------
EPI_ISL_426903      ------------------------------------------------------------
EPI_ISL_426904      ------------------------------------------------------------
EPI_ISL_426907      ------------------------------------------------------------
EPI_ISL_426910      ------------------------------------------------------------
```

```
ORF1ab        6001 REEAIRHVRAWIGFDVEGCHATREAVGTNLPLQLGFSTGVNLVAVPTGYVDTPNNTDFSR
EPI_ISL_413575     ------------------------------------------------------------
EPI_ISL_413588     ------------------------------------------------------------
EPI_ISL_413589     ------------------------------------------------------------
EPI_ISL_424221     ------------------------------------------------------------
EPI_ISL_424233     ------------------------------------------------------------
EPI_ISL_424236     ------------------------------------------------------------
EPI_ISL_424238     ------------------------------------------------------------
EPI_ISL_424248     ------------------------------------------------------------
EPI_ISL_424254     ------------------------------------------------------------
EPI_ISL_425548     ------------------------------------------------------------
EPI_ISL_425581     ------------------------------------------------------------
EPI_ISL_426881     ------------------------------------------------------------
EPI_ISL_426883     ------------------------------------------------------------
EPI_ISL_426888     ------------------------------------------------------------
EPI_ISL_426900     ------------------------------------------------------------
EPI_ISL_426901     ------------------------------------------------------------
EPI_ISL_426903     ------------------------------------------------------------
EPI_ISL_426904     ------------------------------------------------------------
EPI_ISL_426907     ------------------------------------------------------------
EPI_ISL_426910     ------------------------------------------------------------
```


```
ORF1ab        6001 REEAIRHVRAWIGFDVEGCHATREAVGTNLPLQLGFSTGVNLVAVPTGYVDTPNNTDFSR
EPI_ISL_413575     ------------------------------------------------------------
EPI_ISL_413588     ------------------------------------------------------------
EPI_ISL_413589     ------------------------------------------------------------
EPI_ISL_424221     ------------------------------------------------------------
EPI_ISL_424233     ------------------------------------------------------------
EPI_ISL_424236     ------------------------------------------------------------
EPI_ISL_424238     ------------------------------------------------------------
EPI_ISL_424248     ------------------------------------------------------------
EPI_ISL_424254     ------------------------------------------------------------
EPI_ISL_425548     ------------------------------------------------------------
EPI_ISL_425581     ------------------------------------------------------------
EPI_ISL_426881     ------------------------------------------------------------
EPI_ISL_426883     ------------------------------------------------------------
EPI_ISL_426888     ------------------------------------------------------------
EPI_ISL_426900     ------------------------------------------------------------
EPI_ISL_426901     ------------------------------------------------------------
EPI_ISL_426903     ------------------------------------------------------------
EPI_ISL_426904     ------------------------------------------------------------
EPI_ISL_426907     ------------------------------------------------------------
EPI_ISL_426910     ------------------------------------------------------------
```

```
ORF1ab         6061 VSAKPPPGDQFKHLIPLMYKGLPWNVVRIKIVQMLSDTLKNLSDRVVFVLWAHGFELTSM
EPI_ISL_413575      ------------------------------------------------------------
EPI_ISL_413588      ------------------------------------------------------------
EPI_ISL_413589      ------------------------------------------------------------
EPI_ISL_424221      ------------------------------------------------------------
EPI_ISL_424233      ------------------------------------------------------------
EPI_ISL_424236      ------------------------------------------------------------
EPI_ISL_424238      ------------------------------------------------------------
EPI_ISL_424248      ------------------------------------------------------------
EPI_ISL_424254      ------------------------------------------------------------
EPI_ISL_425548      ------------------------------------------------------------
EPI_ISL_425581      ------------------------------------------------------------
EPI_ISL_426881      ------------------------------------------------------------
EPI_ISL_426883      ------------------------------------------------------------
EPI_ISL_426888      ------------------------------------------------------------
EPI_ISL_426900      ------------------------------------------------------------
EPI_ISL_426901      ------------------------------------------------------------
EPI_ISL_426903      ------------------------------------------------------------
EPI_ISL_426904      ------------------------------------------------------------
EPI_ISL_426907      ------------------------------------------------------------
EPI_ISL_426910      ------------------------------------------------------------
```


```
ORF1ab         6061 VSAKPPPGDQFKHLIPLMYKGLPWNVVRIKIVQMLSDTLKNLSDRVVFVLWAHGFELTSM
EPI_ISL_413575      ------------------------------------------------------------
EPI_ISL_413588      ------------------------------------------------------------
EPI_ISL_413589      ------------------------------------------------------------
EPI_ISL_424221      ------------------------------------------------------------
EPI_ISL_424233      ------------------------------------------------------------
EPI_ISL_424236      ------------------------------------------------------------
EPI_ISL_424238      ------------------------------------------------------------
EPI_ISL_424248      ------------------------------------------------------------
EPI_ISL_424254      ------------------------------------------------------------
EPI_ISL_425548      ------------------------------------------------------------
EPI_ISL_425581      ------------------------------------------------------------
EPI_ISL_426881      ------------------------------------------------------------
EPI_ISL_426883      ------------------------------------------------------------
EPI_ISL_426888      ------------------------------------------------------------
EPI_ISL_426900      ------------------------------------------------------------
EPI_ISL_426901      ------------------------------------------------------------
EPI_ISL_426903      ------------------------------------------------------------
EPI_ISL_426904      ------------------------------------------------------------
EPI_ISL_426907      ------------------------------------------------------------
EPI_ISL_426910      ------------------------------------------------------------
```

```
ORF1ab         6121 KYFVKIGPERTCCLCDRRATCFSTASDTYACWHHSIGFDYVYNPFMIDVQQWGFTGNLQS
EPI_ISL_413575      ------------------------------------------------------------
EPI_ISL_413588      ------------------------------------------------------------
EPI_ISL_413589      ------------------------------------------------------------
EPI_ISL_424221      ------------------------------------------------------------
EPI_ISL_424233      ------------------------------------------------------------
EPI_ISL_424236      ------------------------------------------------------------
EPI_ISL_424238      ------------------------------------------------------------
EPI_ISL_424248      ------------------------------------------------------------
EPI_ISL_424254      ------------------------------------------------------------
EPI_ISL_425548      ------------------------------------------------------------
EPI_ISL_425581      ------------------------------------------------------------
EPI_ISL_426881      ------------------------------------------------------------
EPI_ISL_426883      ------------------------------------------------------------
EPI_ISL_426888      ------------------------------------------------------------
EPI_ISL_426900      ------------------------------------------------------------
EPI_ISL_426901      ------------------------------------------------------------
EPI_ISL_426903      ------------------------------------------------------------
EPI_ISL_426904      ------------------------------------------------------------
EPI_ISL_426907      ------------------------------------------------------------
EPI_ISL_426910      ------------------------------------------------------------
```

```
ORF1ab         6121 KYFVKIGPERTCCLCDRRATCFSTASDTYACWHHSIGFDYVYNPFMIDVQQWGFTGNLQS
EPI_ISL_413575      ------------------------------------------------------------
EPI_ISL_413588      ------------------------------------------------------------
EPI_ISL_413589      ------------------------------------------------------------
EPI_ISL_424221      ------------------------------------------------------------
EPI_ISL_424233      ------------------------------------------------------------
EPI_ISL_424236      ------------------------------------------------------------
EPI_ISL_424238      ------------------------------------------------------------
EPI_ISL_424248      ------------------------------------------------------------
EPI_ISL_424254      ------------------------------------------------------------
EPI_ISL_425548      ------------------------------------------------------------
EPI_ISL_425581      ------------------------------------------------------------
EPI_ISL_426881      ------------------------------------------------------------
EPI_ISL_426883      ------------------------------------------------------------
EPI_ISL_426888      ------------------------------------------------------------
EPI_ISL_426900      ------------------------------------------------------------
EPI_ISL_426901      ------------------------------------------------------------
EPI_ISL_426903      ------------------------------------------------------------
EPI_ISL_426904      ------------------------------------------------------------
EPI_ISL_426907      ------------------------------------------------------------
EPI_ISL_426910      ------------------------------------------------------------
```

```
ORF1ab         6181 NHDLYCQVHGNAHVASCDAIMTRCLAVHECFVKRVDWTIEYPIIGDELKINAACRKVQHM
EPI_ISL_413575      ------------------------------------------------------------
EPI_ISL_413588      ------------------------------------------------------------
EPI_ISL_413589      ------------------------------------------------------------
EPI_ISL_424221      ------------------------------------------------------------
EPI_ISL_424233      ------------------------------------------------------------
EPI_ISL_424236      ------------------------------------------------------------
EPI_ISL_424238      ------------------------------------------------------------
EPI_ISL_424248      ------------------------------------------------------------
EPI_ISL_424254      ------------------------------------------------------------
EPI_ISL_425548      ------------------------------------------------------------
EPI_ISL_425581      ------------------------------------------------------------
EPI_ISL_426881      ------------------------------------------------------------
EPI_ISL_426883      ------------------------------------------------------------
EPI_ISL_426888      ------------------------------------------------------------
EPI_ISL_426900      ------------------------------------------------------------
EPI_ISL_426901      ------------------------------------------------------------
EPI_ISL_426903      ------------------------------------------------------------
EPI_ISL_426904      ------------------------------------------------------------
EPI_ISL_426907      ------------------------------------------------------------
EPI_ISL_426910      ------------------------------------------------------------
ORF1ab         6181 NHDLYCQVHGNAHVASCDAIMTRCLAVHECFVKRVDWTIEYPIIGDELKINAACRKVQHM
EPI_ISL_413575      ------------------------------------------------------------
EPI_ISL_413588      ------------------------------------------------------------
EPI_ISL_413589      ------------------------------------------------------------
EPI_ISL_424221      ------------------------------------------------------------
EPI_ISL_424233      ------------------------------------------------------------
EPI_ISL_424236      ------------------------------------------------------------
EPI_ISL_424238      ------------------------------------------------------------
EPI_ISL_424248      ------------------------------------------------------------
EPI_ISL_424254      ------------------------------------------------------------
EPI_ISL_425548      ------------------------------------------------------------
EPI_ISL_425581      ------------------------------------------------------------
EPI_ISL_426881      ------------------------------------------------------------
EPI_ISL_426883      ------------------------------------------------------------
EPI_ISL_426888      ------------------------------------------------------------
EPI_ISL_426900      ------------------------------------------------------------
EPI_ISL_426901      ------------------------------------------------------------
EPI_ISL_426903      ------------------------------------------------------------
EPI_ISL_426904      ------------------------------------------------------------
EPI_ISL_426907      ------------------------------------------------------------
EPI_ISL_426910      ------------------------------------------------------------
```

```
ORF1ab         6241 VVKAALLADKFPVLHDIGNPKAIKCVPQADVEWKFYDAQPCSDKAYKIEELFYSYATHSD
EPI_ISL_413575      ------------------------------------------------------------
EPI_ISL_413588      ------------------------------------------------------------
EPI_ISL_413589      ------------------------------------------------------------
EPI_ISL_424221      ------------------------------------------------------------
EPI_ISL_424233      ------------------------------------------------------------
EPI_ISL_424236      ------------------------------------------------------------
EPI_ISL_424238      ------------------------------------------------------------
EPI_ISL_424248      ------------------------------------------------------------
EPI_ISL_424254      ------------------------------------------------------------
EPI_ISL_425548      ------------------------------------------------------------
EPI_ISL_425581      ------------------------------------------------------------
EPI_ISL_426881      ------------------------------------------------------------
EPI_ISL_426883      ------------------------------------------------------------
EPI_ISL_426888      ------------------------------------------------------------
EPI_ISL_426900      ------------------------------------------------------------
EPI_ISL_426901      ------------------------------------------------------------
EPI_ISL_426903      ------------------------------------------------------------
EPI_ISL_426904      ------------------------------------------------------------
EPI_ISL_426907      ------------------------------------------------------------
EPI_ISL_426910      ------------------------------------------------------------
ORF1ab         6241 VVKAALLADKFPVLHDIGNPKAIKCVPQADVEWKFYDAQPCSDKAYKIEELFYSYATHSD
EPI_ISL_413575      ------------------------------------------------------------
EPI_ISL_413588      ------------------------------------------------------------
EPI_ISL_413589      ------------------------------------------------------------
EPI_ISL_424221      ------------------------------------------------------------
EPI_ISL_424233      ------------------------------------------------------------
EPI_ISL_424236      ------------------------------------------------------------
EPI_ISL_424238      ------------------------------------------------------------
EPI_ISL_424248      ------------------------------------------------------------
EPI_ISL_424254      ------------------------------------------------------------
EPI_ISL_425548      ------------------------------------------------------------
EPI_ISL_425581      ------------------------------------------------------------
EPI_ISL_426881      ------------------------------------------------------------
EPI_ISL_426883      ------------------------------------------------------------
EPI_ISL_426888      ------------------------------------------------------------
EPI_ISL_426900      ------------------------------------------------------------
EPI_ISL_426901      ------------------------------------------------------------
EPI_ISL_426903      ------------------------------------------------------------
EPI_ISL_426904      ------------------------------------------------------------
EPI_ISL_426907      ------------------------------------------------------------
EPI_ISL_426910      ------------------------------------------------------------
```

```
ORF1ab         6301 KFTDGVCLFWNCNVDRYPANSIVCRFDTRVLSNLNLPGCDGGSLYVNKHAFHTPAFDKSA
EPI_ISL_413575      ------------------------------------------------------------
EPI_ISL_413588      ------------------------------------------------------------
EPI_ISL_413589      ------------------------------------------------------------
EPI_ISL_424221      ------------------------------------------------------------
EPI_ISL_424233      ------------------------------------------------------------
EPI_ISL_424236      ------------------------------------------------------------
EPI_ISL_424238      ------------------------------------------------------------
EPI_ISL_424248      ------------------------------------------------------------
EPI_ISL_424254      ------------------------------------------------------------
EPI_ISL_425548      ------------------------------------------------------------
EPI_ISL_425581      ------------------------------------------------------------
EPI_ISL_426881      ------------------------------------------------------------
EPI_ISL_426883      ------------------------------------------------------------
EPI_ISL_426888      ------------------------------------------------------------
EPI_ISL_426900      ------------------------------------------------------------
EPI_ISL_426901      ------------------------------------------------------------
EPI_ISL_426903      ------------------------------------------------------------
EPI_ISL_426904      ------------------------------------------------------------
EPI_ISL_426907      ------------------------------------------------------------
EPI_ISL_426910      ------------------------------------------------------------
```


```
ORF1ab         6301 KFTDGVCLFWNCNVDRYPANSIVCRFDTRVLSNLNLPGCDGGSLYVNKHAFHTPAFDKSA
EPI_ISL_413575      ------------------------------------------------------------
EPI_ISL_413588      ------------------------------------------------------------
EPI_ISL_413589      ------------------------------------------------------------
EPI_ISL_424221      ------------------------------------------------------------
EPI_ISL_424233      ------------------------------------------------------------
EPI_ISL_424236      ------------------------------------------------------------
EPI_ISL_424238      ------------------------------------------------------------
EPI_ISL_424248      ------------------------------------------------------------
EPI_ISL_424254      ------------------------------------------------------------
EPI_ISL_425548      ------------------------------------------------------------
EPI_ISL_425581      ------------------------------------------------------------
EPI_ISL_426881      ------------------------------------------------------------
EPI_ISL_426883      ------------------------------------------------------------
EPI_ISL_426888      ------------------------------------------------------------
EPI_ISL_426900      ------------------------------------------------------------
EPI_ISL_426901      ------------------------------------------------------------
EPI_ISL_426903      ------------------------------------------------------------
EPI_ISL_426904      ------------------------------------------------------------
EPI_ISL_426907      ------------------------------------------------------------
EPI_ISL_426910      ------------------------------------------------------------
```

```
ORF1ab         6361 FVNLKQLPFFYYSDSPCESHGKQVVSDIDYVPLKSATCITRCNLGGAVCRHHANEYRLYL
EPI_ISL_413575      ------------------------------------------------------------
EPI_ISL_413588      ------------------------------------------------------------
EPI_ISL_413589      ------------------------------------------------------------
EPI_ISL_424221      ------------------------------------------------------------
EPI_ISL_424233      ------------------------------------------------------------
EPI_ISL_424236      ------------------------------------------------------------
EPI_ISL_424238      ------------------------------------------------------------
EPI_ISL_424248      ------------------------------------------------------------
EPI_ISL_424254      ------------------------------------------------------------
EPI_ISL_425548      ------------------------------------------------------------
EPI_ISL_425581      ------------------------------------------------------------
EPI_ISL_426881      ------------------------------------------------------------
EPI_ISL_426883      ------------------------------------------------------------
EPI_ISL_426888      ------------------------------------------------------------
EPI_ISL_426900      ------------------------------------------------------------
EPI_ISL_426901      ------------------------------------------------------------
EPI_ISL_426903      ------------------------------------------------------------
EPI_ISL_426904      ------------------------------------------------------------
EPI_ISL_426907      ------------------------------------------------------------
EPI_ISL_426910      ------------------------------------------------------------
```


```
ORF1ab         6361 FVNLKQLPFFYYSDSPCESHGKQVVSDIDYVPLKSATCITRCNLGGAVCRHHANEYRLYL
EPI_ISL_413575      ------------------------------------------------------------
EPI_ISL_413588      ------------------------------------------------------------
EPI_ISL_413589      ------------------------------------------------------------
EPI_ISL_424221      ------------------------------------------------------------
EPI_ISL_424233      ------------------------------------------------------------
EPI_ISL_424236      ------------------------------------------------------------
EPI_ISL_424238      ------------------------------------------------------------
EPI_ISL_424248      ------------------------------------------------------------
EPI_ISL_424254      ------------------------------------------------------------
EPI_ISL_425548      ------------------------------------------------------------
EPI_ISL_425581      ------------------------------------------------------------
EPI_ISL_426881      ------------------------------------------------------------
EPI_ISL_426883      ------------------------------------------------------------
EPI_ISL_426888      ------------------------------------------------------------
EPI_ISL_426900      ------------------------------------------------------------
EPI_ISL_426901      ------------------------------------------------------------
EPI_ISL_426903      ------------------------------------------------------------
EPI_ISL_426904      ------------------------------------------------------------
EPI_ISL_426907      ------------------------------------------------------------
EPI_ISL_426910      ------------------------------------------------------------
```

```
ORF1ab         6421 DAYNMMISAGFSLWVYKQFDTYNLWNTFTRLQSLENVAFNVVNKGHFDGQQGEVPVSIIN
EPI_ISL_413575      ------------------------------------------------------------
EPI_ISL_413588      ------------------------------------------------------------
EPI_ISL_413589      ------------------------------------------------------------
EPI_ISL_424221      ------------------------------------------------------------
EPI_ISL_424233      ------------------------------------------------------------
EPI_ISL_424236      ------------------------------------------------------------
EPI_ISL_424238      ------------------------------------------------------------
EPI_ISL_424248      ------------------------------------------------------------
EPI_ISL_424254      ------------------------------------------------------------
EPI_ISL_425548      ------------------------------------------------------------
EPI_ISL_425581      ------------------------------------------------------------
EPI_ISL_426881      ------------------------------------------------------------
EPI_ISL_426883      ------------------------------------------------------------
EPI_ISL_426888      ------------------------------------------------------------
EPI_ISL_426900      ------------------------------------------------------------
EPI_ISL_426901      ------------------------------------------------------------
EPI_ISL_426903      ------------------------------------------------------------
EPI_ISL_426904      ------------------------------------------------------------
EPI_ISL_426907      ------------------------------------------------------------
EPI_ISL_426910      ------------------------------------------------------------
ORF1ab         6421 DAYNMMISAGFSLWVYKQFDTYNLWNTFTRLQSLENVAFNVVNKGHFDGQQGEVPVSIIN
EPI_ISL_413575      ------------------------------------------------------------
EPI_ISL_413588      ------------------------------------------------------------
EPI_ISL_413589      ------------------------------------------------------------
EPI_ISL_424221      ------------------------------------------------------------
EPI_ISL_424233      ------------------------------------------------------------
EPI_ISL_424236      ------------------------------------------------------------
EPI_ISL_424238      ------------------------------------------------------------
EPI_ISL_424248      ------------------------------------------------------------
EPI_ISL_424254      ------------------------------------------------------------
EPI_ISL_425548      ------------------------------------------------------------
EPI_ISL_425581      ------------------------------------------------------------
EPI_ISL_426881      ------------------------------------------------------------
EPI_ISL_426883      ------------------------------------------------------------
EPI_ISL_426888      ------------------------------------------------------------
EPI_ISL_426900      ------------------------------------------------------------
EPI_ISL_426901      ------------------------------------------------------------
EPI_ISL_426903      ------------------------------------------------------------
EPI_ISL_426904      ------------------------------------------------------------
EPI_ISL_426907      ------------------------------------------------------------
EPI_ISL_426910      ------------------------------------------------------------
```

```
ORF1ab         6481 NTVYTKVDGVDVELFENKTTLPVNVAFELWAKRNIKPVPEVKILNNLGVDIAANTVIWDY
EPI_ISL_413575      ------------------------------------------------------------
EPI_ISL_413588      ------------------------------------------------------------
EPI_ISL_413589      ------------------------------------------------------------
EPI_ISL_424221      ------------------------------------------------------------
EPI_ISL_424233      ------------------------------------------------------------
EPI_ISL_424236      ------------------------------------------------------------
EPI_ISL_424238      ------------------------------------------------------------
EPI_ISL_424248      ------------------------------------------------------------
EPI_ISL_424254      ------------------------------------------------------------
EPI_ISL_425548      ------------------------------------------------------------
EPI_ISL_425581      ------------------------------------------------------------
EPI_ISL_426881      ------------------------------------------------------------
EPI_ISL_426883      ------------------------------------------------------------
EPI_ISL_426888      ------------------------------------------------------------
EPI_ISL_426900      ------------------------------------------------------------
EPI_ISL_426901      ------------------------------------------------------------
EPI_ISL_426903      ------------------------------------------------------------
EPI_ISL_426904      ------------------------------------------------------------
EPI_ISL_426907      ------------------------------------------------------------
EPI_ISL_426910      ------------------------------------------------------------
```


```
ORF1ab         6481 NTVYTKVDGVDVELFENKTTLPVNVAFELWAKRNIKPVPEVKILNNLGVDIAANTVIWDY
EPI_ISL_413575      ------------------------------------------------------------
EPI_ISL_413588      ------------------------------------------------------------
EPI_ISL_413589      ------------------------------------------------------------
EPI_ISL_424221      ------------------------------------------------------------
EPI_ISL_424233      ------------------------------------------------------------
EPI_ISL_424236      ------------------------------------------------------------
EPI_ISL_424238      ------------------------------------------------------------
EPI_ISL_424248      ------------------------------------------------------------
EPI_ISL_424254      ------------------------------------------------------------
EPI_ISL_425548      ------------------------------------------------------------
EPI_ISL_425581      ------------------------------------------------------------
EPI_ISL_426881      ------------------------------------------------------------
EPI_ISL_426883      ------------------------------------------------------------
EPI_ISL_426888      ------------------------------------------------------------
EPI_ISL_426900      ------------------------------------------------------------
EPI_ISL_426901      ------------------------------------------------------------
EPI_ISL_426903      ------------------------------------------------------------
EPI_ISL_426904      ------------------------------------------------------------
EPI_ISL_426907      ------------------------------------------------------------
EPI_ISL_426910      ------------------------------------------------------------
```

```
ORF1ab         6541 KRDAPAHISTIGVCSMTDIAKKPTETICAPLTVFFDGRVDGQVDLFRNARNGVLITEGSV
EPI_ISL_413575      ------------------------------------------------------------
EPI_ISL_413588      ------------------------------------------------------------
EPI_ISL_413589      ------------------------------------------------------------
EPI_ISL_424221      ------------------------------------------------------------
EPI_ISL_424233      ------------------------------------------------------------
EPI_ISL_424236      ------------------------------------------------------------
EPI_ISL_424238      ------------------------------------------------------------
EPI_ISL_424248      ------------------------------------------------------------
EPI_ISL_424254      ------------------------------------------------------------
EPI_ISL_425548      ------------------------------------------------------------
EPI_ISL_425581      ------------------------------------------------------------
EPI_ISL_426881      ------------------------------------------------------------
EPI_ISL_426883      ------------------------------------------------------------
EPI_ISL_426888      ------------------------------------------------------------
EPI_ISL_426900      ------------------------------------------------------------
EPI_ISL_426901      ------------------------------------------------------------
EPI_ISL_426903      ------------------------------------------------------------
EPI_ISL_426904      ------------------------------------------------------------
EPI_ISL_426907      ------------------------------------------------------------
EPI_ISL_426910      ------------------------------------------------------------
```


```
ORF1ab         6541 KRDAPAHISTIGVCSMTDIAKKPTETICAPLTVFFDGRVDGQVDLFRNARNGVLITEGSV
EPI_ISL_413575      ------------------------------------------------------------
EPI_ISL_413588      ------------------------------------------------------------
EPI_ISL_413589      ------------------------------------------------------------
EPI_ISL_424221      ------------------------------------------------------------
EPI_ISL_424233      ------------------------------------------------------------
EPI_ISL_424236      ------------------------------------------------------------
EPI_ISL_424238      ------------------------------------------------------------
EPI_ISL_424248      ------------------------------------------------------------
EPI_ISL_424254      ------------------------------------------------------------
EPI_ISL_425548      ------------------------------------------------------------
EPI_ISL_425581      ------------------------------------------------------------
EPI_ISL_426881      ------------------------------------------------------------
EPI_ISL_426883      ------------------------------------------------------------
EPI_ISL_426888      ------------------------------------------------------------
EPI_ISL_426900      ------------------------------------------------------------
EPI_ISL_426901      ------------------------------------------------------------
EPI_ISL_426903      ------------------------------------------------------------
EPI_ISL_426904      ------------------------------------------------------------
EPI_ISL_426907      ------------------------------------------------------------
EPI_ISL_426910      ------------------------------------------------------------
```

```
ORF1ab          6601 KGLQPSVGPKQASLNGVTLIGEAVKTQFNYYKKVDGVVQQLPETYFTQSRNLQEFKPRSQ
EPI_ISL_413575       ------------------------------------------------------------
EPI_ISL_413588       ------------------------------------------------------------
EPI_ISL_413589       ------------------------------------------------------------
EPI_ISL_424221       ------------------------------------------------------------
EPI_ISL_424233       ------------------------------------------------------------
EPI_ISL_424236       ------------------------------------------------------------
EPI_ISL_424238       ------------------------------------------------------------
EPI_ISL_424248       ------------------------------------------------------------
EPI_ISL_424254       ------------------------------------------------------------
EPI_ISL_425548       ------------------------------------------------------------
EPI_ISL_425581       ------------------------------------------------------------
EPI_ISL_426881       ------------------------------------------------------------
EPI_ISL_426883       ------------------------------------------------------------
EPI_ISL_426888       ------------------------------------------------------------
EPI_ISL_426900       ------------------------------------------------------------
EPI_ISL_426901       ------------------------------------------------------------
EPI_ISL_426903       ------------------------------------------------------------
EPI_ISL_426904       ------------------------------------------------------------
EPI_ISL_426907       ------------------------------------------------------------
EPI_ISL_426910       ------------------------------------------------------------
ORF1ab          6601 KGLQPSVGPKQASLNGVTLIGEAVKTQFNYYKKVDGVVQQLPETYFTQSRNLQEFKPRSQ
EPI_ISL_413575       ------------------------------------------------------------
EPI_ISL_413588       ------------------------------------------------------------
EPI_ISL_413589       ------------------------------------------------------------
EPI_ISL_424221       ------------------------------------------------------------
EPI_ISL_424233       ------------------------------------------------------------
EPI_ISL_424236       ------------------------------------------------------------
EPI_ISL_424238       ------------------------------------------------------------
EPI_ISL_424248       ------------------------------------------------------------
EPI_ISL_424254       ------------------------------------------------------------
EPI_ISL_425548       ------------------------------------------------------------
EPI_ISL_425581       ------------------------------------------------------------
EPI_ISL_426881       ------------------------------------------------------------
EPI_ISL_426883       ------------------------------------------------------------
EPI_ISL_426888       ------------------------------------------------------------
EPI_ISL_426900       ------------------------------------------------------------
EPI_ISL_426901       ------------------------------------------------------------
EPI_ISL_426903       ------------------------------------------------------------
EPI_ISL_426904       ------------------------------------------------------------
EPI_ISL_426907       ------------------------------------------------------------
EPI_ISL_426910       ------------------------------------------------------------
```

```
ORF1ab         6661 MEIDFLELAMDEFIERYKLEGYAFEHIVYGDFSHSQLGGLHLLIGLAKRFKESPFELEDF
EPI_ISL_413575      ------------------------------------------------------------
EPI_ISL_413588      ------------------------------------------------------------
EPI_ISL_413589      ------------------------------------------------------------
EPI_ISL_424221      ------------------------------------------------------------
EPI_ISL_424233      ------------------------------------------------------------
EPI_ISL_424236      ------------------------------------------------------------
EPI_ISL_424238      ------------------------------------------------------------
EPI_ISL_424248      ------------------------------------------------------------
EPI_ISL_424254      ------------------------------------------------------------
EPI_ISL_425548      ------------------------------------------------------------
EPI_ISL_425581      ------------------------------------------------------------
EPI_ISL_426881      ------------------------------------------------------------
EPI_ISL_426883      ------------------------------------------------------------
EPI_ISL_426888      ------------------------------------------------------------
EPI_ISL_426900      ------------------------------------------------------------
EPI_ISL_426901      ------------------------------------------------------------
EPI_ISL_426903      ------------------------------------------------------------
EPI_ISL_426904      ------------------------------------------------------------
EPI_ISL_426907      ------------------------------------------------------------
EPI_ISL_426910      ------------------------------------------------------------
```


```
ORF1ab         6661 MEIDFLELAMDEFIERYKLEGYAFEHIVYGDFSHSQLGGLHLLIGLAKRFKESPFELEDF
EPI_ISL_413575      ------------------------------------------------------------
EPI_ISL_413588      ------------------------------------------------------------
EPI_ISL_413589      ------------------------------------------------------------
EPI_ISL_424221      ------------------------------------------------------------
EPI_ISL_424233      ------------------------------------------------------------
EPI_ISL_424236      ------------------------------------------------------------
EPI_ISL_424238      ------------------------------------------------------------
EPI_ISL_424248      ------------------------------------------------------------
EPI_ISL_424254      ------------------------------------------------------------
EPI_ISL_425548      ------------------------------------------------------------
EPI_ISL_425581      ------------------------------------------------------------
EPI_ISL_426881      ------------------------------------------------------------
EPI_ISL_426883      ------------------------------------------------------------
EPI_ISL_426888      ------------------------------------------------------------
EPI_ISL_426900      ------------------------------------------------------------
EPI_ISL_426901      ------------------------------------------------------------
EPI_ISL_426903      ------------------------------------------------------------
EPI_ISL_426904      ------------------------------------------------------------
EPI_ISL_426907      ------------------------------------------------------------
EPI_ISL_426910      ------------------------------------------------------------
```

```
ORF1ab         6721 IPMDSTVKNYFITDAQTGSSKCVCSVIDLLLDDFVEIIKSQDLSVVSKVVKVTIDYTEIS
EPI_ISL_413575      ------------------------------------------------------------
EPI_ISL_413588      ------------------------------------------------------------
EPI_ISL_413589      ------------------------------------------------------------
EPI_ISL_424221      ------------------------------------------------------------
EPI_ISL_424233      ------------------------------------------------------------
EPI_ISL_424236      ------------------------------------------------------------
EPI_ISL_424238      ------------------------------------------------------------
EPI_ISL_424248      ------------------------------------------------------------
EPI_ISL_424254      ------------------------------------------------------------
EPI_ISL_425548      ------------------------------------------------------------
EPI_ISL_425581      ------------------------------------------------------------
EPI_ISL_426881      ------------------------------------------------------------
EPI_ISL_426883      ------------------------------------------------------------
EPI_ISL_426888      ------------------------------------------------------------
EPI_ISL_426900      ------------------------------------------------------------
EPI_ISL_426901      ------------------------------------------------------------
EPI_ISL_426903      ------------------------------------------------------------
EPI_ISL_426904      ------------------------------------------------------------
EPI_ISL_426907      ------------------------------------------------------------
EPI_ISL_426910      ------------------------------------------------------------
```


```
ORF1ab         6721 IPMDSTVKNYFITDAQTGSSKCVCSVIDLLLDDFVEIIKSQDLSVVSKVVKVTIDYTEIS
EPI_ISL_413575      ------------------------------------------------------------
EPI_ISL_413588      ------------------------------------------------------------
EPI_ISL_413589      ------------------------------------------------------------
EPI_ISL_424221      ------------------------------------------------------------
EPI_ISL_424233      ------------------------------------------------------------
EPI_ISL_424236      ------------------------------------------------------------
EPI_ISL_424238      ------------------------------------------------------------
EPI_ISL_424248      ------------------------------------------------------------
EPI_ISL_424254      ------------------------------------------------------------
EPI_ISL_425548      ------------------------------------------------------------
EPI_ISL_425581      ------------------------------------------------------------
EPI_ISL_426881      ------------------------------------------------------------
EPI_ISL_426883      ------------------------------------------------------------
EPI_ISL_426888      ------------------------------------------------------------
EPI_ISL_426900      ------------------------------------------------------------
EPI_ISL_426901      ------------------------------------------------------------
EPI_ISL_426903      ------------------------------------------------------------
EPI_ISL_426904      ------------------------------------------------------------
EPI_ISL_426907      ------------------------------------------------------------
EPI_ISL_426910      ------------------------------------------------------------
```

```
ORF1ab         6781 FMLWCKDGHVETFYPKLQSSQAWQPGVAMPNLYKMQRMLLEKCDLQNYGDSATLPKGIMM
EPI_ISL_413575      ------------------------------------------------------------
EPI_ISL_413588      ------------------------------------------------------------
EPI_ISL_413589      ------------------------------------------------------------
EPI_ISL_424221      ------------------------------------------------------------
EPI_ISL_424233      ------------------------------------------------------------
EPI_ISL_424236      ------------------------------------------------------------
EPI_ISL_424238      ------------------------------------------------------------
EPI_ISL_424248      ------------------------------------------------------------
EPI_ISL_424254      ------------------------------------------------------------
EPI_ISL_425548      ------------------------------------------------------------
EPI_ISL_425581      ------------------------------------------------------------
EPI_ISL_426881      ------------------------------------------------------------
EPI_ISL_426883      ------------------------------------------------------------
EPI_ISL_426888      ------------------------------------------------------------
EPI_ISL_426900      ------------------------------------------------------------
EPI_ISL_426901      ------------------------------------------------------------
EPI_ISL_426903      ------------------------------------------------------------
EPI_ISL_426904      ------------------------------------------------------------
EPI_ISL_426907      ------------------------------------------------------------
EPI_ISL_426910      ------------------------------------------------------------
```

```
ORF1ab         6781 FMLWCKDGHVETFYPKLQSSQAWQPGVAMPNLYKMQRMLLEKCDLQNYGDSATLPKGIMM
EPI_ISL_413575      ------------------------------------------------------------
EPI_ISL_413588      ------------------------------------------------------------
EPI_ISL_413589      ------------------------------------------------------------
EPI_ISL_424221      ------------------------------------------------------------
EPI_ISL_424233      ------------------------------------------------------------
EPI_ISL_424236      ------------------------------------------------------------
EPI_ISL_424238      ------------------------------------------------------------
EPI_ISL_424248      ------------------------------------------------------------
EPI_ISL_424254      ------------------------------------------------------------
EPI_ISL_425548      ------------------------------------------------------------
EPI_ISL_425581      ------------------------------------------------------------
EPI_ISL_426881      ------------------------------------------------------------
EPI_ISL_426883      ------------------------------------------------------------
EPI_ISL_426888      ------------------------------------------------------------
EPI_ISL_426900      ------------------------------------------------------------
EPI_ISL_426901      ------------------------------------------------------------
EPI_ISL_426903      ------------------------------------------------------------
EPI_ISL_426904      ------------------------------------------------------------
EPI_ISL_426907      ------------------------------------------------------------
EPI_ISL_426910      ------------------------------------------------------------
```

```
ORF1ab          6841 NVAKYTQLCQYLNTLTLAVPYNMRVIHFGAGSDKGVAPGTAVLRQWLPTGTLLVDSDLND
EPI_ISL_413575       ------------------------------------------------------------
EPI_ISL_413588       ------------------------------------------------------------
EPI_ISL_413589       ------------------------------------------------------------
EPI_ISL_424221       ------------------------------------------------------------
EPI_ISL_424233       ------------------------------------------------------------
EPI_ISL_424236       ------------------------------------------------------------
EPI_ISL_424238       ------------------------------------------------------------
EPI_ISL_424248       ------------------------------------------------------------
EPI_ISL_424254       ------------------------------------------------------------
EPI_ISL_425548       ------------------------------------------------------------
EPI_ISL_425581       ------------------------------------------------------------
EPI_ISL_426881       ------------------------------------------------------------
EPI_ISL_426883       ------------------------------------------------------------
EPI_ISL_426888       ------------------------------------------------------------
EPI_ISL_426900       ------------------------------------------------------------
EPI_ISL_426901       ------------------------------------------------------------
EPI_ISL_426903       ------------------------------------------------------------
EPI_ISL_426904       ------------------------------------------------------------
EPI_ISL_426907       ------------------------------------------------------------
EPI_ISL_426910       ------------------------------------------------------------
```

```
ORF1ab          6841 NVAKYTQLCQYLNTLTLAVPYNMRVIHFGAGSDKGVAPGTAVLRQWLPTGTLLVDSDLND
EPI_ISL_413575       ------------------------------------------------------------
EPI_ISL_413588       ------------------------------------------------------------
EPI_ISL_413589       ------------------------------------------------------------
EPI_ISL_424221       ------------------------------------------------------------
EPI_ISL_424233       ------------------------------------------------------------
EPI_ISL_424236       ------------------------------------------------------------
EPI_ISL_424238       ------------------------------------------------------------
EPI_ISL_424248       ------------------------------------------------------------
EPI_ISL_424254       ------------------------------------------------------------
EPI_ISL_425548       ------------------------------------------------------------
EPI_ISL_425581       ------------------------------------------------------------
EPI_ISL_426881       ------------------------------------------------------------
EPI_ISL_426883       ------------------------------------------------------------
EPI_ISL_426888       ------------------------------------------------------------
EPI_ISL_426900       ------------------------------------------------------------
EPI_ISL_426901       ------------------------------------------------------------
EPI_ISL_426903       ------------------------------------------------------------
EPI_ISL_426904       ------------------------------------------------------------
EPI_ISL_426907       ------------------------------------------------------------
EPI_ISL_426910       ------------------------------------------------------------
```

```
ORF1ab          6901 FVSDADSTLIGDCATVHTANKWDLIISDMYDPKTKNVTKENDSKEGFFTYICGFIQQKLA
EPI_ISL_413575       ------------------------------------------------------------
EPI_ISL_413588       ------------------------------------------------------------
EPI_ISL_413589       ------------------------------------------------------------
EPI_ISL_424221       ------------------------------------------------------------
EPI_ISL_424233       ------------------------------------------------------------
EPI_ISL_424236       ------------------------------------------------------------
EPI_ISL_424238       ------------------------------------------------------------
EPI_ISL_424248       ------------------------------------------------------------
EPI_ISL_424254       ------------------------------------------------------------
EPI_ISL_425548       ------------------------------------------------------------
EPI_ISL_425581       ------------------------------------------------------------
EPI_ISL_426881       ------------------------------------------------------------
EPI_ISL_426883       ------------------------------------------------------------
EPI_ISL_426888       ------------------------------------------------------------
EPI_ISL_426900       ------------------------------------------------------------
EPI_ISL_426901       ------------------------------------------------------------
EPI_ISL_426903       ------------------------------------------------------------
EPI_ISL_426904       ------------------------------------------------------------
EPI_ISL_426907       ------------------------------------------------------------
EPI_ISL_426910       ------------------------------------------------------------
ORF1ab          6901 FVSDADSTLIGDCATVHTANKWDLIISDMYDPKTKNVTKENDSKEGFFTYICGFIQQKLA
EPI_ISL_413575       ------------------------------------------------------------
EPI_ISL_413588       ------------------------------------------------------------
EPI_ISL_413589       ------------------------------------------------------------
EPI_ISL_424221       ------------------------------------------------------------
EPI_ISL_424233       ------------------------------------------------------------
EPI_ISL_424236       ------------------------------------------------------------
EPI_ISL_424238       ------------------------------------------------------------
EPI_ISL_424248       ------------------------------------------------------------
EPI_ISL_424254       ------------------------------------------------------------
EPI_ISL_425548       ------------------------------------------------------------
EPI_ISL_425581       ------------------------------------------------------------
EPI_ISL_426881       ------------------------------------------------------------
EPI_ISL_426883       ------------------------------------------------------------
EPI_ISL_426888       ------------------------------------------------------------
EPI_ISL_426900       ------------------------------------------------------------
EPI_ISL_426901       ------------------------------------------------------------
EPI_ISL_426903       ------------------------------------------------------------
EPI_ISL_426904       ------------------------------------------------------------
EPI_ISL_426907       ------------------------------------------------------------
EPI_ISL_426910       ------------------------------------------------------------
```

```
ORF1ab         6961 LGGSVAIKITEHSWNADLYKLMGHFAWWTAFVTNVNASSSEAFLIGCNYLGKPREQIDGY
EPI_ISL_413575      ------------------------------------------------------------
EPI_ISL_413588      ------------------------------------------------------------
EPI_ISL_413589      ------------------------------------------------------------
EPI_ISL_424221      ------------------------------------------------------------
EPI_ISL_424233      ------------------------------------------------------------
EPI_ISL_424236      ------------------------------------------------------------
EPI_ISL_424238      ------------------------------------------------------------
EPI_ISL_424248      ------------------------------------------------------------
EPI_ISL_424254      ------------------------------------------------------------
EPI_ISL_425548      ------------------------------------------------------------
EPI_ISL_425581      ------------------------------------------------------------
EPI_ISL_426881      ------------------------------------------------------------
EPI_ISL_426883      ------------------------------------------------------------
EPI_ISL_426888      ------------------------------------------------------------
EPI_ISL_426900      ------------------------------------------------------------
EPI_ISL_426901      ------------------------------------------------------------
EPI_ISL_426903      ------------------------------------------------------------
EPI_ISL_426904      ------------------------------------------------------------
EPI_ISL_426907      ------------------------------------------------------------
EPI_ISL_426910      ------------------------------------------------------------
```

```
ORF1ab         6961 LGGSVAIKITEHSWNADLYKLMGHFAWWTAFVTNVNASSSEAFLIGCNYLGKPREQIDGY
EPI_ISL_413575      ------------------------------------------------------------
EPI_ISL_413588      ------------------------------------------------------------
EPI_ISL_413589      ------------------------------------------------------------
EPI_ISL_424221      ------------------------------------------------------------
EPI_ISL_424233      ------------------------------------------------------------
EPI_ISL_424236      ------------------------------------------------------------
EPI_ISL_424238      ------------------------------------------------------------
EPI_ISL_424248      ------------------------------------------------------------
EPI_ISL_424254      ------------------------------------------------------------
EPI_ISL_425548      ------------------------------------------------------------
EPI_ISL_425581      ------------------------------------------------------------
EPI_ISL_426881      ------------------------------------------------------------
EPI_ISL_426883      ------------------------------------------------------------
EPI_ISL_426888      ------------------------------------------------------------
EPI_ISL_426900      ------------------------------------------------------------
EPI_ISL_426901      ------------------------------------------------------------
EPI_ISL_426903      ------------------------------------------------------------
EPI_ISL_426904      ------------------------------------------------------------
EPI_ISL_426907      ------------------------------------------------------------
EPI_ISL_426910      ------------------------------------------------------------
```

```
ORF1ab         7021 VMHANYIFWRNTNPIQLSSYSLFDMSKFPLKLRGTAVMSLKEGQINDMILSLLSKGRLII
EPI_ISL_413575      ------------------------------------------------------------
EPI_ISL_413588      ------------------------------------------------------------
EPI_ISL_413589      ------------------------------------------------------------
EPI_ISL_424221      ------------------------------------------------------------
EPI_ISL_424233      ------------------------------------------------------------
EPI_ISL_424236      ------------------------------------------------------------
EPI_ISL_424238      ------------------------------------------------------------
EPI_ISL_424248      ------------------------------------------------------------
EPI_ISL_424254      ------------------------------------------------------------
EPI_ISL_425548      ------------------------------------------------------------
EPI_ISL_425581      ------------------------------------------------------------
EPI_ISL_426881      ------------------------------------------------------------
EPI_ISL_426883      ------------------------------------------------------------
EPI_ISL_426888      ------------------------------------------------------------
EPI_ISL_426900      ------------------------------------------------------------
EPI_ISL_426901      ------------------------------------------------------------
EPI_ISL_426903      ------------------------------------------------------------
EPI_ISL_426904      ------------------------------------------------------------
EPI_ISL_426907      ------------------------------------------------------------
EPI_ISL_426910      ------------------------------------------------------------
```


```
ORF1ab         7021 VMHANYIFWRNTNPIQLSSYSLFDMSKFPLKLRGTAVMSLKEGQINDMILSLLSKGRLII
EPI_ISL_413575      ------------------------------------------------------------
EPI_ISL_413588      ------------------------------------------------------------
EPI_ISL_413589      ------------------------------------------------------------
EPI_ISL_424221      ------------------------------------------------------------
EPI_ISL_424233      ------------------------------------------------------------
EPI_ISL_424236      ------------------------------------------------------------
EPI_ISL_424238      ------------------------------------------------------------
EPI_ISL_424248      ------------------------------------------------------------
EPI_ISL_424254      ------------------------------------------------------------
EPI_ISL_425548      ------------------------------------------------------------
EPI_ISL_425581      ------------------------------------------------------------
EPI_ISL_426881      ------------------------------------------------------------
EPI_ISL_426883      ------------------------------------------------------------
EPI_ISL_426888      ------------------------------------------------------------
EPI_ISL_426900      ------------------------------------------------------------
EPI_ISL_426901      ------------------------------------------------------------
EPI_ISL_426903      ------------------------------------------------------------
EPI_ISL_426904      ------------------------------------------------------------
EPI_ISL_426907      ------------------------------------------------------------
EPI_ISL_426910      ------------------------------------------------------------
```

```
ORF1ab          7081 RENNRVVISSDVLVNN
EPI_ISL_413575       ----------------
EPI_ISL_413588       ----------------
EPI_ISL_413589       ----------------
EPI_ISL_424221       ----------------
EPI_ISL_424233       ----------------
EPI_ISL_424236       ----------------
EPI_ISL_424238       ----------------
EPI_ISL_424248       ----------------
EPI_ISL_424254       ----------------
EPI_ISL_425548       ----------------
EPI_ISL_425581       ----------------
EPI_ISL_426881       ----------------
EPI_ISL_426883       ----------------
EPI_ISL_426888       ----------------
EPI_ISL_426900       ----------------
EPI_ISL_426901       ----------------
EPI_ISL_426903       ----------------
EPI_ISL_426904       ----------------
EPI_ISL_426907       ----------------
EPI_ISL_426910       ----------------
ORF1ab          7081 RENNRVVISSDVLVNN
EPI_ISL_413575       ----------------
EPI_ISL_413588       ----------------
EPI_ISL_413589       ----------------
EPI_ISL_424221       ----------------
EPI_ISL_424233       ----------------
EPI_ISL_424236       ----------------
EPI_ISL_424238       ----------------
EPI_ISL_424248       ----------------
EPI_ISL_424254       ----------------
EPI_ISL_425548       ----------------
EPI_ISL_425581       ----------------
EPI_ISL_426881       ----------------
EPI_ISL_426883       ----------------
EPI_ISL_426888       ----------------
EPI_ISL_426900       ----------------
EPI_ISL_426901       ----------------
EPI_ISL_426903       ----------------
EPI_ISL_426904       ----------------
EPI_ISL_426907       ----------------
EPI_ISL_426910       ----------------
```

Clade GR

```
ORF1ab             1 MESLVPGFNEKTHVQLSLPVLQVRDVLVRGFGDSVEEVLSEARQHLKDGTCGLVEVEKGV
EPI_ISL_413571     1 ------------------------------------------------------------
EPI_ISL_413574     1 ------------------------------------------------------------
EPI_ISL_413579     1 ------------------------------------------------------------
EPI_ISL_413584     1 ------------------------------------------------------------
EPI_ISL_413587     1 ------------------------------------------------------------
EPI_ISL_424257     1 ------------------------------------------------------------
EPI_ISL_425565     1 ------------------------------------------------------------
EPI_ISL_425575     1 ------------------------------------------------------------
EPI_ISL_426822     1 ------------------------------------------------------------
EPI_ISL_426833     1 ------------------------------------------------------------
EPI_ISL_426885     1 ------------------------------------------------------------
EPI_ISL_426886     1 ------------------------------------------------------------
EPI_ISL_426889     1 ------------------------------------------------------------
EPI_ISL_426891     1 ------------------------------------------------------------
EPI_ISL_426892     1 ------------------------------------------------------------
EPI_ISL_426894     1 ------------------------------------------------------------
EPI_ISL_426896     1 ------------------------------------------------------------
EPI_ISL_426897     1 ------------------------------------------------------------
EPI_ISL_426899     1 ------------------------------------------------------------
EPI_ISL_426906     1 ------------------------------------------------------------

ORF1ab            61 LPQLEQPYVFIKRSDARTAPHGHVMVELVAELEGIQYGRSGETLGVLVPHVGEIPVAYRK
EPI_ISL_413571     1 ------------------------------------------------------------
EPI_ISL_413574     1 ------------------------------------------------------------
EPI_ISL_413579     1 ------------------------------------------------------------
EPI_ISL_413584     1 ------------------------------------------------------------
EPI_ISL_413587     1 ------------------------------------------------------------
EPI_ISL_424257     1 ------------------------------------------------------------
EPI_ISL_425565     1 ------------------------------------------------------------
EPI_ISL_425575     1 ------------------------------------------------------------
EPI_ISL_426822     1 ------------------------------------------------------------
EPI_ISL_426833     1 ------------------------------------------------------------
EPI_ISL_426885     1 ------------------------------------------------------------
EPI_ISL_426886     1 ------------------------------------------------------------
EPI_ISL_426889     1 ------------------------------------------------------------
EPI_ISL_426891     1 ------------------------------------------------------------
EPI_ISL_426892     1 ------------------------------------------------------------
EPI_ISL_426894     1 ------------------------------------------------------------
EPI_ISL_426896     1 ------------------------------------------------------------
EPI_ISL_426897     1 ------------------------------------------------------------
EPI_ISL_426899     1 ------------------------------------------------------------
EPI_ISL_426906     1 ------------------------------------------------------------

ORF1ab           121 VLLRKNGNKGAGGHSYGADLKSFDLGDELGTDPYEDFQENWNTKHSSGVTRELMRELNGG
EPI_ISL_413571     1 ------------------------------------------------------------
EPI_ISL_413574     1 ------------------------------------------------------------
EPI_ISL_413579     1 ------------------------------------------------------------
EPI_ISL_413584     1 ------------------------------------------------------------
EPI_ISL_413587     1 ------------------------------------------------------------
EPI_ISL_424257     1 ------------------------------------------------------------
EPI_ISL_425565     1 ------------------------------------------------------------
EPI_ISL_425575     1 ------------------------------------------------------------
EPI_ISL_426822     1 ------------------------------------------------------------
EPI_ISL_426833     1 ------------------------------------------------------------
EPI_ISL_426885     1 ------------------------------------------------------------
EPI_ISL_426886     1 ------------------------------------------------------------
EPI_ISL_426889     1 ------------------------------------------------------------
EPI_ISL_426891     1 ------------------------------------------------------------
EPI_ISL_426892     1 ------------------------------------------------------------
```

```
EPI_ISL_426894    1 ------------------------------------------------------------
EPI_ISL_426896    1 ------------------------------------------------------------
EPI_ISL_426897    1 ------------------------------------------------------------
EPI_ISL_426899    1 ------------------------------------------------------------
EPI_ISL_426906    1 ------------------------------------------------------------
```

```
ORF1ab          181 AYTRYVDNNFCGPDGYPLECIKDLLARAGKASCTLSEQLDFIDTKRGVYCCREHEHEIAW
EPI_ISL_413571    1 ------------------------------------------------------------
EPI_ISL_413574    1 ------------------------------------------------------------
EPI_ISL_413579    1 ------------------------------------------------------------
EPI_ISL_413584    1 ------------------------------------------------------------
EPI_ISL_413587    1 ------------------------------------------------------------
EPI_ISL_424257    1 ------------------------------------------------------------
EPI_ISL_425565    1 ------------------------------------------------------------
EPI_ISL_425575    1 ------------------------------------------------------------
EPI_ISL_426822    1 ------------------------------------------------------------
EPI_ISL_426833    1 ------------------------------------------------------------
EPI_ISL_426885    1 ------------------------------------------------------------
EPI_ISL_426886    1 ------------------------------------------------------------
EPI_ISL_426889    1 ------------------------------------------------------------
EPI_ISL_426891    1 ------------------------------------------------------------
EPI_ISL_426892    1 ------------------------------------------------------------
EPI_ISL_426894    1 ------------------------------------------------------------
EPI_ISL_426896    1 ------------------------------------------------------------
EPI_ISL_426897    1 ------------------------------------------------------------
EPI_ISL_426899    1 ------------------------------------------------------------
EPI_ISL_426906    1 ------------------------------------------------------------

ORF1ab          241 YTERSEKSYELQTPFEIKLAKKFDTFNGECPNFVFPLNSIIKTIQPRVEKKKLDGFMGRI
EPI_ISL_413571    1 ------------------------------------------------------------
EPI_ISL_413574    1 ------------------------------------------------------------
EPI_ISL_413579    1 ------------------------------------------------------------
EPI_ISL_413584    1 ------------------------------------------------------------
EPI_ISL_413587    1 ------------------------------------------------------------
EPI_ISL_424257    1 ------------------------------------------------------------
EPI_ISL_425565    1 ------------------------------------------------------------
EPI_ISL_425575    1 ------------------------------------------------------------
EPI_ISL_426822    1 ------------------------------------------------------------
EPI_ISL_426833    1 ------------------------------------------------------------
EPI_ISL_426885    1 ------------------------------------------------------------
EPI_ISL_426886    1 ------------------------------------------------------------
EPI_ISL_426889    1 ------------------------------------------------------------
EPI_ISL_426891    1 ------------------------------------------------------------
EPI_ISL_426892    1 ------------------------------------------------------------
EPI_ISL_426894    1 ------------------------------------------------------------
EPI_ISL_426896    1 ------------------------------------------------------------
EPI_ISL_426897    1 ------------------------------------------------------------
EPI_ISL_426899    1 ------------------------------------------------------------
EPI_ISL_426906    1 ------------------------------------------------------------

ORF1ab          301 RSVYPVASPNECNQMCLSTLMKCDHCGETSWQTGDFVKATCEFCGTENLTKEGATTCGYL
EPI_ISL_413571    1 ------------------------------------------------------------
EPI_ISL_413574    1 ------------------------------------------------------------
EPI_ISL_413579    1 ------------------------------------------------------------
EPI_ISL_413584    1 ------------------------------------------------------------
EPI_ISL_413587    1 ------------------------------------------------------------
EPI_ISL_424257    1 ------------------------------------------------------------
EPI_ISL_425565    1 ------------------------------------------------------------
EPI_ISL_425575    1 ------------------------------------------------------------
EPI_ISL_426822    1 ------------------------------------------------------------
EPI_ISL_426833    1 ------------------------------------------------------------
EPI_ISL_426885    1 ------------------------------------------------------------
EPI_ISL_426886    1 ------------------------------------------------------------
EPI_ISL_426889    1 ------------------------------------------------------------
EPI_ISL_426891    1 ------------------------------------------------------------
EPI_ISL_426892    1 ------------------------------------------------------------
EPI_ISL_426894    1 ------------------------------------------------------------
EPI_ISL_426896    1 ------------------------------------------------------------
EPI_ISL_426897    1 ------------------------------------------------------------
EPI_ISL_426899    1 ------------------------------------------------------------
```

EPI_ISL_426906   1 --

```
ORF1ab          361 PQNAVVKIYCPACHNSEVGPEHSLAEYHNESGLKTILRKGGRTIAFGGCVFSYVGCHNKC
EPI_ISL_413571    1 ------------------------------------------------------------
EPI_ISL_413574    1 ------------------------------------------------------------
EPI_ISL_413579    1 ------------------------------------------------------------
EPI_ISL_413584    1 ------------------------------------------------------------
EPI_ISL_413587    1 ------------------------------------------------------------
EPI_ISL_424257    1 ------------------------------------------------------------
EPI_ISL_425565    1 ------------------------------------------------------------
EPI_ISL_425575    1 ------------------------------------------------------------
EPI_ISL_426822    1 ------------------------------------------------------------
EPI_ISL_426833    1 ------------------------------------------------------------
EPI_ISL_426885    1 ------------------------------------------------------------
EPI_ISL_426886    1 ------------------------------------------------------------
EPI_ISL_426889    1 ------------------------------------------------------------
EPI_ISL_426891    1 ------------------------------------------------------------
EPI_ISL_426892    1 ------------------------------------------------------------
EPI_ISL_426894    1 ------------------------------------------------------------
EPI_ISL_426896    1 ------------------------------------------------------------
EPI_ISL_426897    1 ------------------------------------------------------------
EPI_ISL_426899    1 ------------------------------------------------------------
EPI_ISL_426906    1 ------------------------------------------------------------

ORF1ab          421 AYWVPRASANIGCNHTGVVGEGSEGLNDNLLEILQKEKVNINIVGDFKLNEEIAIILASF
EPI_ISL_413571    1 ------------------------------------------------------------
EPI_ISL_413574    1 ------------------------------------------------------------
EPI_ISL_413579    1 ------------------------------------------------------------
EPI_ISL_413584    1 ------------------------------------------------------------
EPI_ISL_413587    1 ------------------------------------------------------------
EPI_ISL_424257    1 ------------------------------------------------------------
EPI_ISL_425565    1 ------------------------------------------------------------
EPI_ISL_425575    1 ------------------------------------------------------------
EPI_ISL_426822    1 ------------------------------------------------------------
EPI_ISL_426833    1 ------------------------------------------------------------
EPI_ISL_426885    1 ------------------------------------------------------------
EPI_ISL_426886    1 ------------------------------------------------------------
EPI_ISL_426889    1 ------------------------------------------------------------
EPI_ISL_426891    1 ------------------------------------------------------------
EPI_ISL_426892    1 ------------------------------------------------------------
EPI_ISL_426894    1 ------------------------------------------------------------
EPI_ISL_426896    1 ------------------------------------------------------------
EPI_ISL_426897    1 ------------------------------------------------------------
EPI_ISL_426899    1 ------------------------------------------------------------
EPI_ISL_426906    1 ------------------------------------------------------------

ORF1ab          481 SASTSAFVETVKGLDYKAFKQIVESCGNFKVTKGKAKKGAWNIGEQKSILSPLYAFASEA
EPI_ISL_413571    1 ------------------------------------------------------------
EPI_ISL_413574    1 ------------------------------------------------------------
EPI_ISL_413579    1 ------------------------------------------------------------
EPI_ISL_413584    1 ------------------------------------------------------------
EPI_ISL_413587    1 ------------------------------------------------------------
EPI_ISL_424257    1 ------------------------------------------------------------
EPI_ISL_425565    1 ------------------------------------------------------------
EPI_ISL_425575    1 ------------------------------------------------------------
EPI_ISL_426822    1 ------------------------------------------------------------
EPI_ISL_426833    1 ------------------------------------------------------------
EPI_ISL_426885    1 ------------------------------------------------------------
EPI_ISL_426886    1 ------------------------------------------------------------
EPI_ISL_426889    1 ------------------------------------------------------------
EPI_ISL_426891    1 ------------------------------------------------------------
EPI_ISL_426892    1 ------------------------------------------------------------
EPI_ISL_426894    1 ------------------------------------------------------------
EPI_ISL_426896    1 ------------------------------------------------------------
EPI_ISL_426897    1 ------------------------------------------------------------
EPI_ISL_426899    1 ------------------------------------------------------------
```

```
EPI_ISL_426906     1 ------------------------------------------------------------
```

```
ORF1ab          541 ARVVRSIFSRTLETAQNSVRVLQKAAITILDGISQYSLRLIDAMMFTSDLATNNLVVMAY
EPI_ISL_413571    1 ------------------------------------------------------------
EPI_ISL_413574    1 ------------------------------------------------------------
EPI_ISL_413579    1 ------------------------------------------------------------
EPI_ISL_413584    1 ------------------------------------------------------------
EPI_ISL_413587    1 ------------------------------------------------------------
EPI_ISL_424257    1 ------------------------------------------------------------
EPI_ISL_425565    1 ------------------------------------------------------------
EPI_ISL_425575    1 ------------------------------------------------------------
EPI_ISL_426822    1 ------------------------------------------------------------
EPI_ISL_426833    1 ------------------------------------------------------------
EPI_ISL_426885    1 ------------------------------------------------------------
EPI_ISL_426886    1 ------------------------------------------------------------
EPI_ISL_426889    1 ------------------------------------------------------------
EPI_ISL_426891    1 ------------------------------------------------------------
EPI_ISL_426892    1 ------------------------------------------------------------
EPI_ISL_426894    1 ------------------------------------------------------------
EPI_ISL_426896    1 ------------------------------------------------------------
EPI_ISL_426897    1 ------------------------------------------------------------
EPI_ISL_426899    1 ------------------------------------------------------------
EPI_ISL_426906    1 ------------------------------------------------------------

ORF1ab          601 ITGGVVQLTSQWLTNIFGTVYEKLKPVLDWLEEKFKEGVEFLRDGWEIVKFISTCACEIV
EPI_ISL_413571    1 ------------------------------------------------------------
EPI_ISL_413574    1 ------------------------------------------------------------
EPI_ISL_413579    1 ------------------------------------------------------------
EPI_ISL_413584    1 ------------------------------------------------------------
EPI_ISL_413587    1 ------------------------------------------------------------
EPI_ISL_424257    1 ------------------------------------------------------------
EPI_ISL_425565    1 ------------------------------------------------------------
EPI_ISL_425575    1 ------------------------------------------------------------
EPI_ISL_426822    1 ------------------------------------------------------------
EPI_ISL_426833    1 ------------------------------------------------------------
EPI_ISL_426885    1 ------------------------------------------------------------
EPI_ISL_426886    1 ------------------------------------------------------------
EPI_ISL_426889    1 ------------------------------------------------------------
EPI_ISL_426891    1 ------------------------------------------------------------
EPI_ISL_426892    1 ------------------------------------------------------------
EPI_ISL_426894    1 ------------------------------------------------------------
EPI_ISL_426896    1 ------------------------------------------------------------
EPI_ISL_426897    1 ------------------------------------------------------------
EPI_ISL_426899    1 ------------------------------------------------------------
EPI_ISL_426906    1 ------------------------------------------------------------

ORF1ab          661 GGQIVTCAKEIKESVQTFFKLVNKFLALCADSIIIGGAKLKALNLGETFVTHSKGLYRKC
EPI_ISL_413571    1 ------------------------------------------------------------
EPI_ISL_413574    1 ------------------------------------------------------------
EPI_ISL_413579    1 ------------------------------------------------------------
EPI_ISL_413584    1 ------------------------------------------------------------
EPI_ISL_413587    1 ------------------------------------------------------------
EPI_ISL_424257    1 ------------------------------------------------------------
EPI_ISL_425565    1 ------------------------------------------------------------
EPI_ISL_425575    1 ------------------------------------------------------------
EPI_ISL_426822    1 ------------------------------------------------------------
EPI_ISL_426833    1 ------------------------------------------------------------
EPI_ISL_426885    1 ------------------------------------------------------------
EPI_ISL_426886    1 ------------------------------------------------------------
EPI_ISL_426889    1 ------------------------------------------------------------
EPI_ISL_426891    1 ------------------------------------------------------------
EPI_ISL_426892    1 ------------------------------------------------------------
EPI_ISL_426894    1 ------------------------------------------------------------
EPI_ISL_426896    1 ------------------------------------------------------------
EPI_ISL_426897    1 ------------------------------------------------------------
EPI_ISL_426899    1 ------------------------------------------------------------
```

EPI_ISL_426906   1 --

```
ORF1ab           721 VKSREETGLLMPLKAPKEIIFLEGETLPTEVLTEEVVLKTGDLQPLEQPTSEAVEAPLVG
EPI_ISL_413571     1 ------------------------------------------------------------
EPI_ISL_413574     1 ------------------------------------------------------------
EPI_ISL_413579     1 ------------------------------------------------------------
EPI_ISL_413584     1 ------------------------------------------------------------
EPI_ISL_413587     1 ------------------------------------------------------------
EPI_ISL_424257     1 ------------------------------------------------------------
EPI_ISL_425565     1 ------------------------------------------------------------
EPI_ISL_425575     1 ------------------------------------------------------------
EPI_ISL_426822     1 ------------------------------------------------------------
EPI_ISL_426833     1 ------------------------------------------------------------
EPI_ISL_426885     1 ------------------------------------------------------------
EPI_ISL_426886     1 ------------------------------------------------------------
EPI_ISL_426889     1 ------------------------------------------------------------
EPI_ISL_426891     1 ------------------------------------------------------------
EPI_ISL_426892     1 ------------------------------------------------------------
EPI_ISL_426894     1 ------------------------------------------------------------
EPI_ISL_426896     1 ------------------------------------------------------------
EPI_ISL_426897     1 ------------------------------------------------------------
EPI_ISL_426899     1 ------------------------------------------------------------
EPI_ISL_426906     1 ------------------------------------------------------------

ORF1ab           781 TPVCINGLMLLEIKDTEKYCALAPNMMVTNNTFTLKGGAPTKVTFGDDTVIEVQGYKSVN
EPI_ISL_413571     1 --------------------------------------APTKVTFGDDTVIEVQGYKSVN
EPI_ISL_413574     1 --------------------------------------APTKVTFGDDTVIEVQGYKSVN
EPI_ISL_413579     1 --------------------------------------APTKVTFGDDTVIEVQGYKSVN
EPI_ISL_413584     1 --------------------------------------APTKVTFGDDTVIEVQGYKSVN
EPI_ISL_413587     1 --------------------------------------APTKVTFGDDTVIEVQGYKSVN
EPI_ISL_424257     1 --------------------------------------APTKVTFGDDTVIEVQGYKSVN
EPI_ISL_425565     1 --------------------------------------APTKVTFGDDTVIEVQGYKSVN
EPI_ISL_425575     1 --------------------------------------APTKVTFGDDTVIEVQGYKSVN
EPI_ISL_426822     1 --------------------------------------APTKVTFGDDTVIEVQGYKSVN
EPI_ISL_426833     1 --------------------------------------APTKVTFGDDTVIEVQGYKSVN
EPI_ISL_426885     1 --------------------------------------APTKVTFGDDTVIEVQGYKSVN
EPI_ISL_426886     1 --------------------------------------APTKVTFGDDTVIEVQGYKSVN
EPI_ISL_426889     1 --------------------------------------APTKVTFGDDTVIEVQGYKSVN
EPI_ISL_426891     1 --------------------------------------APTKVTFGDDTVIEVQGYKSVN
EPI_ISL_426892     1 --------------------------------------APTKVTFGDDTVIEVQGYKSVN
EPI_ISL_426894     1 --------------------------------------APTKVTFGDDTVIEVQGYKSVN
EPI_ISL_426896     1 --------------------------------------APTKVTFGDDTVIEVQGYKSVN
EPI_ISL_426897     1 --------------------------------------APTKVTFGDDTVIEVQGYKSVN
EPI_ISL_426899     1 --------------------------------------APTKVTFGDDTVIEVQGYKSVN
EPI_ISL_426906     1 --------------------------------------APTKVTFGDDTVIEVQGYKSVN

ORF1ab           841 ITFELDERIDKVLNEKCSAYTVELGTEVNEFACVVADAVIKTLQPVSELLTPLGIDLDEW
EPI_ISL_413571    23 ITFELDERIDKVLNEKCSAYTVELGTEVNEFACVVADAVIKTLQPVSELLTPLGIDLDEW
EPI_ISL_413574    23 ITFELDERIDKVLNEKCSAYTVELGTEVNEFACVVADAVIKTLQPVSELLTPLGIDLDEW
EPI_ISL_413579    23 ITFELDERIDKVLNEKCSAYTVELGTEVNEFACVVADAVIKTLQPVSELLTPLGIDLDEW
EPI_ISL_413584    23 ITFELDERIDKVLNEKCSAYTVELGTEVNEFACVVADAVIKTLQPVSELLTPLGIDLDEW
EPI_ISL_413587    23 ITFELDERIDKVLNEKCSAYTVELGTEVNEFACVVADAVIKTLQPVSELLTPLGIDLDEW
EPI_ISL_424257    23 ITFELDERIDKVLNEKCSAYTVELGTEVNEFACVVADAVIKTLQPVSELLTPLGIDLDEW
EPI_ISL_425565    23 ITFELDERIDKVLNEKCSAYTVELGTEVNEFACVVADAVIKTLQPVSELLTPLGIDLDEW
EPI_ISL_425575    23 ITFELDERIDKVLNEKCSAYTVELGTEVNEFACVVADAVIKTLQPVSELLTPLGIDLDEW
EPI_ISL_426822    23 ITFELDERIDKVLNEKCSAYTVELGTEVNEFACVVADAVIKTLQPVSELLTPLGIDLDEW
EPI_ISL_426833    23 ITFELDERIDKVLNEKCSAYTVELGTEVNEFACVVADAVIKTLQPVSELLTPLGIDLDEW
EPI_ISL_426885    23 ITFELDERIDKVLNEKCSAYTVELGTEVNEFACVVADAVIKTLQPVSELLTPLGIDLDEW
EPI_ISL_426886    23 ITFELDERIDKVLNEKCSAYTVELGTEVNEFACVVADAVIKTLQPVSELLTPLGIDLDEW
EPI_ISL_426889    23 ITFELDERIDKVLNEKCSAYTVELGTEVNEFACVVADAVIKTLQPVSELLTPLGIDLDEW
EPI_ISL_426891    23 ITFELDERIDKVLNEKCSAYTVELGTEVNEFACVVADAVIKTLQPVSELLTPLGIDLDEW
EPI_ISL_426892    23 ITFELDERIDKVLNEKCSAYTVELGTEVNEFACVVADAVIKTLQPVSELLTPLGIDLDEW
EPI_ISL_426894    23 ITFELDERIDKVLNEKCSAYTVELGTEVNEFACVVADAVIKTLQPVSELLTPLGIDLDEW
EPI_ISL_426896    23 ITFELDERIDKVLNEKCSAYTVELGTEVNEFACVVADAVIKTLQPVSELLTPLGIDLDEW
EPI_ISL_426897    23 ITFELDERIDKVLNEKCSAYTVELGTEVNEFACVVADAVIKTLQPVSELLTPLGIDLDEW
EPI_ISL_426899    23 ITFELDERIDKVLNEKCSAYTVELGTEVNEFACVVADAVIKTLQPVSELLTPLGIDLDEW
```

EPI_ISL_426906 23 ITFELDERIDKVLNEKCSAYTVELGTEVNEFACVVADAVIKTLQPVSELLTPLGIDLDEW

```
ORF1ab          901 SMATYYLFDESGEFKLASHMYCSFYPPDEDEEEGDCEEEEFEPSTQYEYGTEDDYQGKPL
EPI_ISL_413571   83 SMATYYLFDESGEFKLASHMYCSFYPPDEDEEEGDCEEEEFEPSTQYEYGTEDDYQGKPL
EPI_ISL_413574   83 SMATYYLFDESGEFKLASHMYCSFYPPDEDEEEGDCEEEEFEPSTQYEYGTEDDYQGKPL
EPI_ISL_413579   83 SMATYYLFDESGEFKLASHMYCSFYPPDEDEEEGDCEEEEFEPSTQYEYGTEDDYQGKPL
EPI_ISL_413584   83 SMATYYLFDESGEFKLASHMYCSFYPPDEDEEEGDCEEEEFEPSTQYEYGTEDDYQGKPL
EPI_ISL_413587   83 SMATYYLFDESGEFKLASHMYCSFYPPDEDEEEGDCEEEEFEPSTQYEYGTEDDYQGKPL
EPI_ISL_424257   83 SMATYYLFDESGEFKLASHMYCSFYPPDEDEEEGDCEEEEFEPSTQYEYGTEDDYQGKPL
EPI_ISL_425565   83 SMATYYLFDESGEFKLASHMYCSFYPPDEDEEEGDCEEEEFEPSTQYEYGTEDDYQGKPL
EPI_ISL_425575   83 SMATYYLFDESGEFKLASHMYCSFYPPDEDEEEGDCEEEEFEPSTQYEYGTEDDYQGKPL
EPI_ISL_426822   83 SMATYYLFDESGEFKLASHMYCSFYPPDEDEEEGDCEEEEFEPSTQYEYGTEDDYQGKPL
EPI_ISL_426833   83 SMATYYLFDESGEFKLASHMYCSFYPPDEDEEEGDCEEEEFEPSTQYEYGTEDDYQGKPL
EPI_ISL_426885   83 SMATYYLFDESGEFKLASHMYCSFYPPDEDEEEGDCEEEEFEPSTQYEYGTEDDYQGKPL
EPI_ISL_426886   83 SMATYYLFDESGEFKLASHMYCSFYPPDEDEEEGDCEEEEFEPSTQYEYGTEDDYQGKPL
EPI_ISL_426889   83 SMATYYLFDESGEFKLASHMYCSFYPPDEDEEEGDCEEEEFEPSTQYEYGTEDDYQGKPL
EPI_ISL_426891   83 SMATYYLFDESGEFKLASHMYCSFYPPDEDEEEGDCEEEEFEPSTQYEYGTEDDYQGKPL
EPI_ISL_426892   83 SMATYYLFDESGEFKLASHMYCSFYPPDEDEEEGDCEEEEFEPSTQYEYGTEDDYQGKPL
EPI_ISL_426894   83 SMATYYLFDESGEFKLASHMYCSFYPPDEDEEEGDCEEEEFEPSTQYEYGTEDDYQGKPL
EPI_ISL_426896   83 SMATYYLFDESGEFKLASHMYCSFYPPDEDEEEGDCEEEEFEPSTQYEYGTEDDYQGKPL
EPI_ISL_426897   83 SMATYYLFDESGEFKLASHMYCSFYPPDEDEEEGDCEEEEFEPSTQYEYGTEDDYQGKPL
EPI_ISL_426899   83 SMATYYLFDESGEFKLASHMYCSFYPPDEDEEEGDCEEEEFEPSTQYEYGTEDDYQGKPL
EPI_ISL_426906   83 SMATYYLFDESGEFKLASHMYCSFYPPDEDEEEGDCEEEEFEPSTQYEYGTEDDYQGKPL

ORF1ab          961 EFGATSAALQPEEEQEEDWLDDDSQQTVGQQDGSEDNQTTTIQTIVEVQPQLEMELTPVV
EPI_ISL_413571  143 EFGATSAALQPEEEQEEDWLDDDSQQTVGQQDGSEDNQTTTIQTIVEVQPQLEMELTPVV
EPI_ISL_413574  143 EFGATSAALQPEEEQEEDWLDDDSQQTVGQQDGSEDNQTTTIQTIVEVQPQLEMELTPVV
EPI_ISL_413579  143 EFGATSAALQPEEEQEEDWLDDDSQQTVGQQDGSEDNQTTTIQTIVEVQPQLEMELTPVV
EPI_ISL_413584  143 EFGATSAALQPEEEQEEDWLDDDSQQTVGQQDGSEDNQTTTIQTIVEVQPQLEMELTPVV
EPI_ISL_413587  143 EFGATSAALQPEEEQEEDWLDDDSQQTVGQQDGSEDNQTTTIQTIVEVQPQLEMELTPVV
EPI_ISL_424257  143 EFGATSAALQPEEEQEEDWLDDDSQQTVGQQDGSEDNQTTTIQTIVEVQPQLEMELTPVV
EPI_ISL_425565  143 EFGATSAALQPEEEQEEDWLDDDSQQTVGQQDGSEDNQTTTIQTIVEVQPQLEMELTPVV
EPI_ISL_425575  143 EFGATSAALQPEEEQEEDWLDDDSQQTVGQQDGSEDNQTTTIQTIVEVQPQLEMELTPVV
EPI_ISL_426822  143 EFGATSAALQPEEEQEEDWLDDDSQQTVGQQDGSEDNQTTTIQTIVEVQPQLEMELTPVV
EPI_ISL_426833  143 EFGATSAALQPEEEQEEDWLDDDSQQTVGQQDGSEDNQTTTIQTIVEVQPQLEMELTPVV
EPI_ISL_426885  143 EFGATSAALQPEEEQEEDWLDDDSQQTVGQQDGSEDNQTTTIQTIVEVQPQLEMELTPVV
EPI_ISL_426886  143 EFGATSAALQPEEEQEEDWLDDDSQQTVGQQDGSEDNQTTTIQTIVEVQPQLEMELTPVV
EPI_ISL_426889  143 EFGATSAALQPEEEQEEDWLDDDSQQTVGQQDGSEDNQTTTIQTIVEVQPQLEMELTPVV
EPI_ISL_426891  143 EFGATSAALQPEEEQEEDWLDDDSQQTVGQQDGSEDNQTTTIQTIVEVQPQLEMELTPVV
EPI_ISL_426892  143 EFGATSAALQPEEEQEEDWLDDDSQQTVGQQDGSEDNQTTTIQTIVEVQPQLEMELTPVV
EPI_ISL_426894  143 EFGATSAALQPEEEQEEDWLDDDSQQTVGQQDGSEDNQTTTIQTIVEVQPQLEMELTPVV
EPI_ISL_426896  143 EFGATSAALQPEEEQEEDWLDDDSQQTVGQQDGSEDNQTTTIQTIVEVQPQLEMELTPVV
EPI_ISL_426897  143 EFGATSAALQPEEEQEEDWLDDDSQQTVGQQDGSEDNQTTTIQTIVEVQPQLEMELTPVV
EPI_ISL_426899  143 EFGATSAALQPEEEQEEDWLDDDSQQTVGQQDGSEDNQTTTIQTIVEVQPQLEMELTPVV
EPI_ISL_426906  143 EFGATSAALQPEEEQEEDWLDDDSQQTVGQQDGSEDNQTTTIQTIVEVQPQLEMELTPVV

ORF1ab         1021 QTIEVNSFSGYLKLTDNVYIKNADIVEEAKKVKPTVVVNAANVYLKHGGGVAGALNKATN
EPI_ISL_413571  203 QTIEVNSFSGYLKLTDNVYIKNADIVEEAKKVKPTVVVNAANVYLKHGGGVAGALNKATN
EPI_ISL_413574  203 QTIEVNSFSGYLKLTDNVYIKNADIVEEAKKVKPTVVVNAANVYLKHGGGVAGALNKATN
EPI_ISL_413579  203 QTIEVNSFSGYLKLTDNVYIKNADIVEEAKKVKPTVVVNAANVYLKHGGGVAGALNKATN
EPI_ISL_413584  203 QTIEVNSFSGYLKLTDNVYIKNADIVEEAKKVKPTVVVNAANVYLKHGGGVAGALNKATN
EPI_ISL_413587  203 QTIEVNSFSGYLKLTDNVYIKNADIVEEAKKVKPTVVVNAANVYLKHGGGVAGALNKATN
EPI_ISL_424257  203 QTIEVNSFSGYLKLTDNVYIKNADIVEEAKKVKPTVVVNAANVYLKHGGGVAGALNKATN
EPI_ISL_425565  203 QTIEVNSFSGYLKLTDNVYIKNADIVEEAKKVKPTVVVNAANVYLKHGGGVAGALNKATN
EPI_ISL_425575  203 QTIEVNSFSGYLKLTDNVYIKNADIVEEAKKVKPTVVVNAANVYLKHGGGVAGALNKATN
EPI_ISL_426822  203 QTIEVNSFSGYLKLTDNVYIKNADIVEEAKKVKPTVVVNAANVYLKHGGGVAGALNKATN
EPI_ISL_426833  203 QTIEVNSFSGYLKLTDNVYIKNADIVEEAKKVKPTVVVNAANVYLKHGGGVAGALNKATN
EPI_ISL_426885  203 QTIEVNSFSGYLKLTDNVYIKNADIVEEAKKVKPTVVVNAANVYLKHGGGVAGALNKATN
EPI_ISL_426886  203 QTIEVNSFSGYLKLTDNVYIKNADIVEEAKKVKPTVVVNAANVYLKHGGGVAGALNKATN
EPI_ISL_426889  203 QTIEVNSFSGYLKLTDNVYIKNADIVEEAKKVKPTVVVNAANVYLKHGGGVAGALNKATN
EPI_ISL_426891  203 QTIEVNSFSGYLKLTDNVYIKNADIVEEAKKVKPTVVVNAANVYLKHGGGVAGALNKATN
EPI_ISL_426892  203 QTIEVNSFSGYLKLTDNVYIKNADIVEEAKKVKPTVVVNAANVYLKHGGGVAGALNKATN
EPI_ISL_426894  203 QTIEVNSFSGYLKLTDNVYIKNADIVEEAKKVKPTVVVNAANVYLKHGGGVAGALNKATN
EPI_ISL_426896  203 QTIEVNSFSGYLKLTDNVYIKNADIVEEAKKVKPTVVVNAANVYLKHGGGVAGALNKATN
EPI_ISL_426897  203 QTIEVNSFSGYLKLTDNVYIKNADIVEEAKKVKPTVVVNAANVYLKHGGGVAGALNKATN
EPI_ISL_426899  203 QTIEVNSFSGYLKLTDNVYIKNADIVEEAKKVKPTVVVNAANVYLKHGGGVAGALNKATN
```

EPI_ISL_426906 203 QTIEVNSFSGYLKLTDNVYIKNADIVEEAKKVKPTVVVNAANVYLKHGGGVAGALNKATN

```
ORF1ab          1081 NAMQVESDDYIATNGPLKVGGSCVLSGHNLAKHCLHVVGPNVNKGEDIQLLKSAYENFNQ
EPI_ISL_413571   263 NAMQVESDDYIATNGPLKVGGSCVLSGHNLAKHCLHVVGPNVNKGEDIQLLKSAYENFNQ
EPI_ISL_413574   263 NAMQVESDDYIATNGPLKVGGSCVLSGHNLAKHCLHVVGPNVNKGEDIQLLKSAYENFNQ
EPI_ISL_413579   263 NAMQVESDDYIATNGPLKVGGSCVLSGHNLAKHCLHVVGPNVNKGEDIQLLKSAYENFNQ
EPI_ISL_413584   263 NAMQVESDDYIATNGPLKVGGSCVLSGHNLAKHCLHVVGPNVNKGEDIQLLKSAYENFNQ
EPI_ISL_413587   263 NAMQVESDDYIATNGPLKVGGSCVLSGHNLAKHCLHVVGPNVNKGEDIQLLKSAYENFNQ
EPI_ISL_424257   263 NAMQVESDDYIATNGPLKVGGSCVLSGHNLAKHCLHVVGPNVNKGEDIQLLKSAYENFNQ
EPI_ISL_425565   263 NAMQVESDDYIATNGPLKVGGSCVLSGHNLAKHCLHVVGPNVNKGEDIQLLKSAYENFNQ
EPI_ISL_425575   263 NAMQVESDDYIATNGPLKVGGSCVLSGHNLAKHCLHVVGPNVNKGEDIQLLKSAYENFNQ
EPI_ISL_426822   263 NAMQVESDDYIATNGPLKVVGSCVLSGHNLAKHCLHVVGPNVNKGEDIQLLKSAYENFNQ
EPI_ISL_426833   263 NAMQVESDDYIATNGPLKVVGSCVLSGHNLAKHCLHVVGPNVNKGEDIQLLKSAYENFNQ
EPI_ISL_426885   263 NAMQVESDDYIATNGPLKVGGSCVLSGHNLAKHCLHVVGPNVNKGEDIQLLKSAYENFNQ
EPI_ISL_426886   263 NAMQVESDDYIATNGPLKVGGSCVLSGHNLAKHCLHVVGPNVNKGEDIQLLKSAYENFNQ
EPI_ISL_426889   263 NAMQVESDDYIATNGPLKVGGSCVLSGHNLAKHCLHVVGPNVNKGEDIQLLKSAYENFNQ
EPI_ISL_426891   263 NAMQVESDDYIATNGPLKVGGSCVLSGHNLAKHCLHVVGPNVNKGEDIQLLKSAYENFNQ
EPI_ISL_426892   263 NAMQVESDDYIATNGPLKVGGSCVLSGHNLAKHCLHVVGPNVNKGEDIQLLKSAYENFNQ
EPI_ISL_426894   263 NAMQVESDDYIATNGPLKVGGSCVLSGHNLAKHCLHVVGPNVNKGEDIQLLKSAYENFNQ
EPI_ISL_426896   263 NAMQVESDDYIATNGPLKVGGSCVLSGHNLAKHCLHVVGPNVNKGEDIQLLKSAYENFNQ
EPI_ISL_426897   263 NAMQVESDDYIATNGPLKVGGSCVLSGHNLAKHCLHVVGPNVNKGEDIQLLKSAYENFNQ
EPI_ISL_426899   263 NAMQVESDDYIATNGPLKVGGSCVLSGHNLAKHCLHVVGPNVNKGEDIQLLKSAYENFNQ
EPI_ISL_426906   263 NAMQVESDDYIATNGPLKVGGSCVLSGHNLAKHCLHVVGPNVNKGEDIQLLKSAYENFNQ

ORF1ab          1141 HEVLLAPLLSAGIFGADPIHSLRVCVDTVRTNVYLAVFDKNLYDKLVSSFLEMKSEKQVE
EPI_ISL_413571   323 HEVLLAPLLSAGIFGADPIHSLRVCVDTVRTNVYLAVFDKNLYDKLVSSFLEMKSEKQVE
EPI_ISL_413574   323 HEVLLAPLLSAGIFGADPIHSLRVCVDTVRTNVYLAVFDKNLYDKLVSSFLEMKSEKQVE
EPI_ISL_413579   323 HEVLLAPLLSAGIFGADPIHSLRVCVDTVRTNVYLAVFDKNLYDKLVSSFLEMKSEKQVE
EPI_ISL_413584   323 HEVLLAPLLSAGIFGADPIHSLRVCVDTVRTNVYLAVFDKNLYDKLVSSFLEMKSEKQVE
EPI_ISL_413587   323 HEVLLAPLLSAGIFGADPIHSLRVCVDTVRTNVYLAVFDKNLYDKLVSSFLEMKSEKQVE
EPI_ISL_424257   323 HEVLLAPLLSAGIFGADPIHSLRVCVDTVRTNVYLAVFDKNLYDKLVSSFLEMKSEKQVE
EPI_ISL_425565   323 HEVLLAPLLSAGIFGADPIHSLRVCVDTVRTNVYLAVFDKNLYDKLVSSFLEMKSEKQVE
EPI_ISL_425575   323 HEVLLAPLLSAGIFGADPIHSLRVCVDTVRTNVYLAVFDKNLYDKLVSSFLEMKSEKQVE
EPI_ISL_426822   323 HEVLLAPLLSAGIFGADPIHSLRVCVDTVRTNVYLAVFDKNLYDKLVSSFLEMKSEKQVE
EPI_ISL_426833   323 HEVLLAPLLSAGIFGADPIHSLRVCVDTVRTNVYLAVFDKNLYDKLVSSFLEMKSEKQVE
EPI_ISL_426885   323 HEVLLAPLLSAGIFGADPIHSLRVCVDTVRTNVYLAVFDKNLYDKLVSSFLEMKSEKQVE
EPI_ISL_426886   323 HEVLLAPLLSAGIFGADPIHSLRVCVDTVRTNVYLAVFDKNLYDKLVSSFLEMKSEKQVE
EPI_ISL_426889   323 HEVLLAPLLSAGIFGADPIHSLRVCVDTVRTNVYLAVFDKNLYDKLVSSFLEMKSEKQVE
EPI_ISL_426891   323 HEVLLAPLLSAGIFGADPIHSLRVCVDTVRTNVYLAVFDKNLYDKLVSSFLEMKSEKQVE
EPI_ISL_426892   323 HEVLLAPLLSAGIFGADPIHSLRVCVDTVRTNVYLAVFDKNLYDKLVSSFLEMKSEKQVE
EPI_ISL_426894   323 HEVLLAPLLSAGIFGADPIHSLRVCVDTVRTNVYLAVFDKNLYDKLVSSFLEMKSEKQVE
EPI_ISL_426896   323 HEVLLAPLLSAGIFGADPIHSLRVCVDTVRTNVYLAVFDKNLYDKLVSSFLEMKSEKQVE
EPI_ISL_426897   323 HEVLLAPLLSAGIFGADPIHSLRVCVDTVRTNVYLAVFDKNLYDKLVSSFLEMKSEKQVE
EPI_ISL_426899   323 HEVLLAPLLSAGIFGADPIHSLRVCVDTVRTNVYLAVFDKNLYDKLVSSFLEMKSEKQVE
EPI_ISL_426906   323 HEVLLAPLLSAGIFGADPIHSLRVCVDTVRTNVYLAVFDKNLYDKLVSSFLEMKSEKQVE

ORF1ab          1201 QKIAEIPKEEVKPFITESKPSVEQRKQDDKKIKACVEEVTTTLEETKFLTENLLLYIDIN
EPI_ISL_413571   383 QKIAEIPKEEVKPFITESKPSVEQRKQDDNKIKACVEEVTTTLEETKFLTENLLLYIDIN
EPI_ISL_413574   383 QKIAEIPKEEVKPFITESKPSVEQRKQDDNKIKACVEEVTTTLEETKFLTENLLLYIDIN
EPI_ISL_413579   383 QKIAEIPKEEVKPFITESKPSVEQRKQDDNKIKACVEEVTTTLEETKFLTENLLLYIDIN
EPI_ISL_413584   383 QKIAEIPKEEVKPFITESKPSVEQRKQDDKKIKACVEEVTTTLEETKFLTENLLLYIDIN
EPI_ISL_413587   383 QKIAEIPKEEVKPFITESKPSVEQRKQDDKKIKACVEEVTTTLEETKFLTENLLLYIDIN
EPI_ISL_424257   383 QKIAEIPKEEVKPFITESKPSVEQRKQDDKKIKACVEEVTTTLEETKFLTENLLLYIDIN
EPI_ISL_425565   383 QKIAEIPKEEVKPFITESKPSVEQRKQDDKKIKACVEEVTTTLEETKFLTENLLLYIDIN
EPI_ISL_425575   383 QKIAEIPKEEVKPFITESKPSVEQRKQDDKKIKACVEEVTTTLEETKFLTENLLLYIDIN
EPI_ISL_426822   383 QKIAEIPKEEVKPFITESKPSVEQRKQDDKKIKACVEEVTTTLEETKFLTENLLLYIDIN
EPI_ISL_426833   383 QKIAEIPKEEVKPFITESKPSVEQRKQDDKKIKACVEEVTTTLEETKFLTENLLLYIDIN
EPI_ISL_426885   383 QKIAEIPKEEVKPFITESKPSVEQRKQDDKKIKACVEEVTTTLEETKFLTENLLLYIDIN
EPI_ISL_426886   383 QKIAEIPKEEVKPFITESKPSVEQRKQDDKKIKACVEEVTTTLEETKFLTENLLLYIDIN
EPI_ISL_426889   383 QKIAEIPKEEVKPFITESKPSVEQRKQDDKKIKACVEEVTTTLEETKFLTENLLLYIDIN
EPI_ISL_426891   383 QKIAEIPKEEVKPFITESKPSVEQRKQDDKKIKACVEEVTTTLEETKFLTENLLLYIDIN
EPI_ISL_426892   383 QKIAEIPKEEVKPFITESKPSVEQRKQDDKKIKACVEEVTTTLEETKFLTENLLLYIDIN
EPI_ISL_426894   383 QKIAEIPKEEVKPFITESKPSVEQRKQDDKKIKACVEEVTTTLEETKFLTENLLLYIDIN
EPI_ISL_426896   383 QKIAEIPKEEVKPFITESKPSVEQRKQDDKKIKACVEEVTTTLEETKFLTENLLLYIDIN
EPI_ISL_426897   383 QKIAEIPKEEVKPFITESKPSVEQRKQDDKKIKACVEEVTTTLEETKFLTENLLLYIDIN
EPI_ISL_426899   383 QKIAEIPKEEVKPFITESKPSVEQRKQDDKKIKACVEEVTTTLEETKFLTENLLLYIDIN
```

EPI_ISL_426906 383 QKIAEIPKEEVKPFITESKPSVEQRKQDDKKIKACVEEVTTTLEETKFLTENLLLYIDIN

```
ORF1ab          1261 GNLHPDSATLVSDIDITFLKKDAPYIVGDVVQEGVLTAVVIPTKKAGGTTEMLAKALRKV
EPI_ISL_413571   443 GNLHPDSATLVSDIDITFLKKDAPYIVGDVVQEGVLTAVVIPTKKAGGTTEMLAKALRKV
EPI_ISL_413574   443 GNLHPDSATLVSDIDITFLKKDAPYIVGDVVQEGVLTAVVIPTKKAGGTTEMLAKALRKV
EPI_ISL_413579   443 GNLHPDSATLVSDIDITFLKKDAPYIVGDVVQEGVLTAVVIPTKKAGGTTEMLAKALRKV
EPI_ISL_413584   443 GNLHPDSATLVSDIDITFLKKDAPYIVGDVVQEGVLTAVVIPTKKAGGTTEMLAKALRKV
EPI_ISL_413587   443 GNLHPDSATLVSDIDITFLKKDAPYIVGDVVQEGVLTAVVIPTKKAGGTTEMLAKALRKV
EPI_ISL_424257   443 GNLHPDSATLVSDIDITFLKKDAPYIVGDVVQEGVLTAVVIPTKKAGGTTEMLAKALRKV
EPI_ISL_425565   443 GNLHPDSATLVSDIDITFLKKDAPYIVGDVVQEGVLTAVVIPTKKAGGTTEMLAKALRKV
EPI_ISL_425575   443 GNLHPDSATLVSDIDITFLKKDAPYIVGDVVQEGVLTAVVIPTKKAGGTTEMLAKALRKV
EPI_ISL_426822   443 GNLHPDSATLVSDIDITFLKKDAPYIVGDVVQEGVLTAVVIPTKKAGGTTEMLAKALRKV
EPI_ISL_426833   443 GNLHPDSATLVSDIDITFLKKDAPYIVGDVVQEGVLTAVVIPTKKAGGTTEMLAKALRKV
EPI_ISL_426885   443 GNLHPDSATLVSDIDITFLKKDAPYIVGDVVQEGVLTAVVIPTKKAGGTTEMLAKALRKV
EPI_ISL_426886   443 GNLHPDSATLVSDIDITFLKKDAPYIVGDVVQEGVLTAVVIPTKKAGGTTEMLAKALRKV
EPI_ISL_426889   443 GNLHPDSATLVSDIDITFLKKDAPYIVGDVVQEGVLTAVVIPTKKAGGTTEMLAKALRKV
EPI_ISL_426891   443 GNLHPDSATLVSDIDITFLKKDAPYIVGDVVQEGVLTAVVIPTKKAGGTTEMLAKALRKV
EPI_ISL_426892   443 GNLHPDSATLVSDIDITFLKKDAPYIVGDVVQEGVLTAVVIPTKKAGGTTEMLAKALRKV
EPI_ISL_426894   443 GNLHPDSATLVSDIDITFLKKDAPYIVGDVVQEGVLTAVVIPTKKAGGTTEMLAKALRKV
EPI_ISL_426896   443 GNLHPDSATLVSDIDITFLKKDAPYIVGDVVQEGVLTAVVIPTKKAGGTTEMLAKALRKV
EPI_ISL_426897   443 GNLHPDSATLVSDIDITFLKKDAPYIVGDVVQEGVLTAVVIPTKKAGGTTEMLAKALRKV
EPI_ISL_426899   443 GNLHPDSATLVSDIDITFLKKDAPYIVGDVVQEGVLTAVVIPTKKAGGTTEMLAKALRKV
EPI_ISL_426906   443 GNLHPDSATLVSDIDITFLKKDAPYIVGDVVQEGVLTAVVIPTKKAGGTTEMLAKALRKV

ORF1ab          1321 PTDNYITTYPGQGLNGYTVEEAKTVLKKCKSAFYILPSIISNEKQEILGTVSWNLREMLA
EPI_ISL_413571   503 PTDNYITTYPGQGLNGYTVEEAKTVLKKCKSAFYILPSIISNEKQEILGTVSWNLREMLA
EPI_ISL_413574   503 PTDNYITTYPGQGLNGYTVEEAKTVLKKCKSAFYILPSIISNEKQEILGTVSWNLREMLA
EPI_ISL_413579   503 PTDNYITTYPGQGLNGYTVEEAKTVLKKCKSAFYILPSIISNEKQEILGTVSWNLREMLA
EPI_ISL_413584   503 PTDNYITTYPGQGLNGYTVEEAKTVLKKCKSAFYILPSIISNEKQEILGTVSWNLREMLA
EPI_ISL_413587   503 PTDNYITTYPGQGLNGYTVEEAKTVLKKCKSAFYILPSIISNEKQEILGTVSWNLREMLA
EPI_ISL_424257   503 PTDNYITTYPGQGLNGYTVEEAKTVLKKCKSAFYILPSIISNEKQEILGTVSWNLREMLA
EPI_ISL_425565   503 PTDNYITTYPGQGLNGYTVEEAKTVLKKCKSAFYILPSIISNEKQEILGTVSWNLREMLA
EPI_ISL_425575   503 PTDNYITTYPGQGLNGYTVEEAKTVLKKCKSAFYILPSIISNEKQEILGTVSWNLREMLA
EPI_ISL_426822   503 PTDNYITTYPGQGLNGYTVEEAKTVLKKCKSAFYILPSIISNEKQEILGTVSWNLREMLA
EPI_ISL_426833   503 PTDNYITTYPGQGLNGYTVEEAKTVLKKCKSAFYILPSIISNEKQEILGTVSWNLREMLA
EPI_ISL_426885   503 PTDNYITTYPGQGLNGYTVEEAKTVLKKCKSAFYILPSIISNEKQEILGTVSWNLREMLA
EPI_ISL_426886   503 PTDNYITTYPGQGLNGYTVEEAKTVLKKCKSAFYILPSIISNEKQEILGTVSWNLREMLA
EPI_ISL_426889   503 PTDNYITTYPGQGLNGYTVEEAKTVLKKCKSAFYILPSIISNEKQEILGTVSWNLREMLA
EPI_ISL_426891   503 PTDNYITTYPGQGLNGYTVEEAKTVLKKCKSAFYILPSIISNEKQEILGTVSWNLREMLA
EPI_ISL_426892   503 PTDNYITTYPGQGLNGYTVEEAKTVLKKCKSAFYILPSIISNEKQEILGTVSWNLREMLA
EPI_ISL_426894   503 PTDNYITTYPGQGLNGYTVEEAKTVLKKCKSAFYILPSIISNEKQEILGTVSWNLREMLA
EPI_ISL_426896   503 PTDNYITTYPGQGLNGYTVEEAKTVLKKCKSAFYILPSIISNEKQEILGTVSWNLREMLA
EPI_ISL_426897   503 PTDNYITTYPGQGLNGYTVEEAKTVLKKCKSAFYILPSIISNEKQEILGTVSWNLREMLA
EPI_ISL_426899   503 PTDNYITTYPGQGLNGYTVEEAKTVLKKCKSAFYILPSIISNEKQEILGTVSWNLREMLA
EPI_ISL_426906   503 PTDNYITTYPGQGLNGYTVEEAKTVLKKCKSAFYILPSIISNEKQEILGTVSWNLREMLA

ORF1ab          1381 HAEETRKLMPVCVETKAIVSTIQRKYKGIKIQEGVVDYGARFYFYTSKTTVASLINTLND
EPI_ISL_413571   563 HAEETRKLMPVCVETKAIVSTIQRKYKGIKIQEGVVDYGARFYFYTSKTTVASLINTLND
EPI_ISL_413574   563 HAEETRKLMPVCVETKAIVSTIQRKYKGIKIQEGVVDYGARFYFYTSKTTVASLINTLND
EPI_ISL_413579   563 HAEETRKLMPVCVETKAIVSTIQRKYKGIKIQEGVVDYGARFYFYTSKTTVASLINTLND
EPI_ISL_413584   563 HAEETRKLMPVCVETKAIVSTIQRKYKGIKIQEGVVDYGARFYFYTSKTTVASLINTLND
EPI_ISL_413587   563 HAEETRKLMPVCVETKAIVSTIQRKYKGIKIQEGVVDYGARFYFYTSKTTVASLINTLND
EPI_ISL_424257   563 HAEETRKLMPVCVETKAIVSTIQRKYKGIKIQEGVVDYGARFYFYTSKTTVASLINTLND
EPI_ISL_425565   563 HAEETRKLMPVCVETKAIVSTIQRKYKGIKIQEGVVDYGARFYFYTSKTTVASLINTLND
EPI_ISL_425575   563 HAEETRKLMPVCVETKAIVSTIQRKYKGIKIQEGVVDYGARFYFYTSKTTVVSLINTLND
EPI_ISL_426822   563 HAEETRKLMPVCVETKAIVSTIQRKYKGIKIQEGVVDYGARFYFYTSKTTVASLINTLND
EPI_ISL_426833   563 HAEETRKLMPVCVETKAIVSTIQRKYKGIKIQEGVVDYGARFYFYTSKTTVASLINTLND
EPI_ISL_426885   563 HAEETRKLMPVCVETKAIVSTIQRKYKGIKIQEGVVDYGARFYFYTSKTTVASLINTLND
EPI_ISL_426886   563 HAEETRKLMPVCVETKAIVSTIQRKYKGIKIQEGVVDYGARFYFYTSKTTVASLINTLND
EPI_ISL_426889   563 HAEETRKLMPVCVETKAIVSTIQRKYKGIKIQEGVVDYGARFYFYTSKTTVASLINTLND
EPI_ISL_426891   563 HAEETRKLMPVCVETKAIVSTIQRKYKGIKIQEGVVDYGARFYFYTSKTTVASLINTLND
EPI_ISL_426892   563 HAEETRKLMPVCVETKAIVSTIQRKYKGIKIQEGVVDYGARFYFYTSKTTVASLINTLND
EPI_ISL_426894   563 HAEETRKLMPVCVETKAIVSTIQRKYKGIKIQEGVVDYGARFYFYTSKTTVASLINTLND
EPI_ISL_426896   563 HAEETRKLMPVCVETKAIVSTIQRKYKGIKIQEGVVDYGARFYFYTSKTTVASLINTLND
EPI_ISL_426897   563 HAEETRKLMPVCVETKAIVSTIQRKYKGIKIQEGVVDYGARFYFYTSKTTVASLINTLND
EPI_ISL_426899   563 HAEETRKLMPVCVETKAIVSTIQRKYKGIKIQEGVVDYGARFYFYTSKTTVASLINTLND
```

EPI_ISL_426906 563 HAEETRKLMPVCVETKAIVSTIQRKYKGIKIQEGVVDYGARFYFYTSKTTVASLINTLND

```
ORF1ab          1441 LNETLVTMPLGYVTHGLNLEEAARYMRSLKVPATVSVSSPDAVTAYNGYLTSSSKTPEEH
EPI_ISL_413571   623 LNETLVTMPLGYVTHGLNLEEAARYMRSLKVPATVSVSSPDAVTAYNGYLTSSSKTPEEH
EPI_ISL_413574   623 LNETLVTMPLGYVTHGLNLEEAARYMRSLKVPATVSVSSPDAVTAYNGYLTSSSKTPEEH
EPI_ISL_413579   623 LNETLVTMPLGYVTHGLNLEEAARYMRSLKVPATVSVSSPDAVTAYNGYLTSSSKTPEEH
EPI_ISL_413584   623 LNETLVTMPLGYVTHGLNLEEAARYMRSLKVPATVSVSSPDAVTAYNGYLTSSSKTPEEH
EPI_ISL_413587   623 LNETLVTMPLGYVTHGLNLEEAARYMRSLKVPATVSVSSPDAVTAYNGYLTSSSKTPEEH
EPI_ISL_424257   623 LNETLVTMPLGYVTHGLNLEEAARYMRSLKVPATVSVSSPDAVTAYNGYLTSSSKTPEEH
EPI_ISL_425565   623 LNETLVTMPLGYVTHGLNLEEAARYMRSLKVPATVSVSSPDAVTAYNGYLTSSSKTPEEH
EPI_ISL_425575   623 LNETLVTMPLGYVTHGLNLEEAARYMRSLKVPATVSVSSPDAVTAYNGYLTSSSKTPEEH
EPI_ISL_426822   623 LNETLVTMPLGYVTHGLNLEEAARYMRSLKVPATVSVSSPDAVTAYNGYLTSSSKTPEEH
EPI_ISL_426833   623 LNETLVTMPLGYVTHGLNLEEAARYMRSLKVPATVSVSSPDAVTAYNGYLTSSSKTPEEH
EPI_ISL_426885   623 LNETLVTMPLGYVTHGLNLEEAARYMRSLKVPATVSVSSPDAVTAYNGYLTSSSKTPEEH
EPI_ISL_426886   623 LNETLVTMPLGYVTHGLNLEEAARYMRSLKVPATVSVSSPDAVTAYNGYLTSSSKTPEEH
EPI_ISL_426889   623 LNETLVTMPLGYVTHGLNLEEAARYMRSLKVPATVSVSSPDAVTAYNGYLTSSSKTPEEH
EPI_ISL_426891   623 LNETLVTMPLGYVTHGLNLEEAARYMRSLKVPATVSVSSPDAVTAYNGYLTSSSKTPEEH
EPI_ISL_426892   623 LNETLVTMPLGYVTHGLNLEEAARYMRSLKVPATVSVSSPDAVTAYNGYLTSSSKTPEEH
EPI_ISL_426894   623 LNETLVTMPLGYVTHGLNLEEAARYMRSLKVPATVSVSSPDAVTAYNGYLTSSSKTPEEH
EPI_ISL_426896   623 LNETLVTMPLGYVTHGLNLEEAARYMRSLKVPATVSVSSPDAVTAYNGYLTSSSKTPEEH
EPI_ISL_426897   623 LNETLVTMPLGYVTHGLNLEEAARYMRSLKVPATVSVSSPDAVTAYNGYLTSSSKTPEEH
EPI_ISL_426899   623 LNETLVTMPLGYVTHGLNLEEAARYMRSLKVPATVSVSSPDAVTAYNGYLTSSSKTPEEH
EPI_ISL_426906   623 LNETLVTMPLGYVTHGLNLEEAARYMRSLKVPATVSVSSPDAVTAYNGYLTSSSKTPEEH

ORF1ab          1501 FIETISLAGSYKDWSYSGQSTQLGIEFLKRGDKSVYYTSNPTTFHLDGEVITFDNLKTLL
EPI_ISL_413571   683 FIETISLAGSYKDWSYSGQSTQLGIEFLKRGDKSVYYTSNPTTFHLDGEVITFDNLKTFL
EPI_ISL_413574   683 FIETISLAGSYKDWSYSGQSTQLGIEFLKRGDKSVYYTSNPTTFHLDGEVITFDNLKTLL
EPI_ISL_413579   683 FIETISLAGSYKDWSYSGQSTQLGIEFLKRGDKSVYYTSNPTTFHLDGEVITFDNLKTLL
EPI_ISL_413584   683 FIETISLAGSYKDWSYSGQSTQLGIEFLKRGDKSVYYTSNPTTFHLDGEVITFDNLKTLL
EPI_ISL_413587   683 FIETISLAGSYKDWSYSGQSTQLGIEFLKRGDKSVYYTSNPTTFHLDGEVITFDNLKTLL
EPI_ISL_424257   683 FIETISLAGSYKDWSYSGQSTQLGIEFLKRGDKSVYYTSNPTTFHLDGEVITFDNLKTLL
EPI_ISL_425565   683 FIETISLAGSYKDWSYSGQSTQLGIEFLKRGDKSVYYTSNPTTFHLDGEVITFDNLKTLL
EPI_ISL_425575   683 FIETISLAGSYKDWSYSGQSTQLGIEFLKRGDKSVYYTSNPTTFHLDGEVITFDNLKTLL
EPI_ISL_426822   683 FIETISLAGSYKDWSYSGQSTQLGIEFLKRGDKSVYYTSNPTTFHLDGEVITFDNLKTLL
EPI_ISL_426833   683 FIETISLAGSYKDWSYSGQSTQLGIEFLKRGDKSVYYTSNPTTFHLDGEVITFDNLKTLL
EPI_ISL_426885   683 FIETISLAGSYKDWSYSGQSTQLGIEFLKRGDKSVYYTSNPTTFHLDGEVITFDNLKTLL
EPI_ISL_426886   683 FIETISLAGSYKDWSYSGQSTQLGIEFLKRGDKSVYYTSNPTTFHLDGEVITFDNLKTLL
EPI_ISL_426889   683 FIETISLAGSYKDWSYSGQSTQLGIEFLKRGDKSVYYTSNPTTFHLDGEVITFDNLKTLL
EPI_ISL_426891   683 FIETISLAGSYKDWSYSGQSTQLGIEFLKRGDKSVYYTSNPTTFHLDGEVITFDNLKTLL
EPI_ISL_426892   683 FIETISLAGSYKDWSYSGQSTQLGIEFLKRGDKSVYYTSNPTTFHLDGEVITFDNLKTLL
EPI_ISL_426894   683 FIETISLAGSYKDWSYSGQSTQLGIEFLKRGDKSVYYTSNPTTFHLDGEVITFDNLKTLL
EPI_ISL_426896   683 FIETISLAGSYKDWSYSGQSTQLGIEFLKRGDKSVYYTSNPTTFHLDGEVITFDNLKTLL
EPI_ISL_426897   683 FIETISLAGSYKDWSYSGQSTQLGIEFLKRGDKSVYYTSNPTTFHLDGEVITFDNLKTLL
EPI_ISL_426899   683 FIETISLAGSYKDWSYSGQSTQLGIEFLKRGDKSVYYTSNPTTFHLDGEVITFDNLKTLL
EPI_ISL_426906   683 FIETISLAGSYKDWSYSGQSTQLGIEFLKRGDKSVYYTSNPTTFHLDGEVITFDNLKTLL

ORF1ab          1561 SLREVRTIKVFTTVDNINLHTQVVDMSMTYGQQFGPTYLDGADVTKIKPHNSHEGKTFYV
EPI_ISL_413571   743 SLREVRTIKVFTTVDNINLHTQVVDMSMTYGQQFGPTYLDGADVTKIKPHNSHEGKTFYV
EPI_ISL_413574   743 SLREVRTIKVFTTVDNINLHTQVVDMSMTYGQQFGPTYLDGADVTKIKPHNSHEGKTFYV
EPI_ISL_413579   743 SLREVRTIKVFTTVDNINLHTQVVDMSMTYGQQFGPTYLDGADVTKIKPHNSHEGKTFYV
EPI_ISL_413584   743 SLREVRTIKVFTTVDNINLHTQVVDMSMTYGQQFGPTYLDGADVTKIKPHNSHEGKTFYV
EPI_ISL_413587   743 SLREVRTIKVFTTVDNINLHTQVVDMSMTYGQQFGPTYLDGADVTKIKPHNSHEGKTFYV
EPI_ISL_424257   743 SLREVRTIKVFTTVDNINLHTQVVDMSMTYGQQFGPTYLDGADVTKIKPHNSHEGKTFYV
EPI_ISL_425565   743 SLREVRTIKVFTTVDNINLHTQVVDMSMTYGQQFGPTYLDGADVTKIKPHNSHEGKTFYV
EPI_ISL_425575   743 SLREVRTIKVFTTVDNINLHTQVVDMSMTYGQQFGPTYLDGADVTKIKPHNSHEGKTFYV
EPI_ISL_426822   743 SLREVRTIKVFTTVDNINLHTQVVDMSMTYGQQFGPTYLDGADVTKIKPHNSHEGKTFYV
EPI_ISL_426833   743 SLREVRTIKVFTTVDNINLHTQVVDMSMTYGQQFGPTYLDGADVTKIKPHNSHEGKTFYV
EPI_ISL_426885   743 SLREVRTIKVFTTVDNINLHTQVVDMSMTYGQQFGPTYLDGADVTKIKPHNSHEGKTFYV
EPI_ISL_426886   743 SLREVRTIKVFTTVDNINLHTQVVDMSMTYGQQFGPTYLDGADVTKIKPHNSHEGKTFYV
EPI_ISL_426889   743 SLREVRTIKVFTTVDNINLHTQVVDMSMTYGQQFGPTYLDGADVTKIKPHNSHEGKTFYV
EPI_ISL_426891   743 SLREVRTIKVFTTVDNINLHTQVVDMSMTYGQQFGPTYLDGADVTKIKPHNSHEGKTFYV
EPI_ISL_426892   743 SLREVRTIKVFTTVDNINLHTQVVDMSMTYGQQFGPTYLDGADVTKIKPHNSHEGKTFYV
EPI_ISL_426894   743 SLREVRTIKVFTTVDNINLHTQVVDMSMTYGQQFGPTYLDGADVTKIKPHNSHEGKTFYV
EPI_ISL_426896   743 SLREVRTIKVFTTVDNINLHTQVVDMSMTYGQQFGPTYLDGADVTKIKPHNSHEGKTFYV
EPI_ISL_426897   743 SLREVRTIKVFTTVDNINLHTQVVDMSMTYGQQFGPTYLDGADVTKIKPHNSHEGKTFYV
EPI_ISL_426899   743 SLREVRTIKVFTTVDNINLHTQVVDMSMTYGQQFGPTYLDGADVTKIKPHNSHEGKTFYV
```

EPI_ISL_426906 743 SLREVRTIKVFTTVDNINLHTQVVDMSMTYGQQFGPTYLDGADVTKIKPHNSHEGKTFYV

```
ORF1ab         1621 LPNDDTLRVEAFEYYHTTDPSFLGRYMSALNHTKKWKYPQVNGLTSIKWADNNCYLATAL
EPI_ISL_413571  803 LPNDDTLRVEAFEYYHTTDPSFLGRYMSALNHTKKWKYPQVNGLTSIKWADNNCYLATAL
EPI_ISL_413574  803 LPNDDTLRVEAFEYYHTTDPSFLGRYMSALNHTKKWKYPQVNGLTSIKWADNNCYLATAL
EPI_ISL_413579  803 LPNDDTLRVEAFEYYHTTDPSFLGRYMSALNHTKKWKYPQVNGLTSIKWADNNCYLATAL
EPI_ISL_413584  803 LPNDDTLRVEAFEYYHTTDPSFLGRYMSALNHTKKWKYPQVNGLTSIKWADNNCYLATAL
EPI_ISL_413587  803 LPNDDTLRVEAFEYYHTTDPSFLGRYMSALNHTKKWKYPQVNGLTSIKWADNNCYLATAL
EPI_ISL_424257  803 LPNDDTLRVEAFEYYHTTDPSFLGRYMSALNHTKKWKYPQVNGLTSIKWADNNCYLATAL
EPI_ISL_425565  803 LPNDDTLRVEAFEYYHTTDPSFLGRYMSALNHTKKWKYPQVNGLTSIKWADNNCYLATAL
EPI_ISL_425575  803 LPNDDTLRVEAFEYYHTTDPSFLGRYMSALNHTKKWKYPQVNGLTSIKWADNNCYLATAL
EPI_ISL_426822  803 LPNDDTLRVEAFEYYHTTDPSFLGRYMSALNHTKKWKYPQVNGLTSIKWADNNCYLATAL
EPI_ISL_426833  803 LPNDDTLRVEAFEYYHTTDPSFLGRYMSALNHTKKWKYPQVNGLTSIKWADNNCYLATAL
EPI_ISL_426885  803 LPNDDTLRVEAFEYYHTTDPSFLGRYMSALNHTKKWKYPQVNGLTSIKWADNNCYLATAL
EPI_ISL_426886  803 LPNDDTLRVEAFEYYHTTDPSFLGRYMSALNHTKKWKYPQVNGLTSIKWADNNCYLATAL
EPI_ISL_426889  803 LPNDDTLRVEAFEYYHTTDPSFLGRYMSALNHTKKWKYPQVNGLTSIKWADNNCYLATAL
EPI_ISL_426891  803 LPNDDTLRVEAFEYYHTTDPSFLGRYMSALNHTKKWKYPQVNGLTSIKWADNNCYLATAL
EPI_ISL_426892  803 LPNDDTLRVEAFEYYHTTDPSFLGRYMSALNHTKKWKYPQVNGLTSIKWADNNCYLATAL
EPI_ISL_426894  803 LPNDDTLRVEAFEYYHTTDPSFLGRYMSALNHTKKWKYPQVNGLTSIKWADNNCYLATAL
EPI_ISL_426896  803 LPNDDTLRVEAFEYYHTTDPSFLGRYMSALNHTKKWKYPQVNGLTSIKWADNNCYLATAL
EPI_ISL_426897  803 LPNDDTLRVEAFEYYHTTDPSFLGRYMSALNHTKKWKYPQVNGLTSIKWADNNCYLATAL
EPI_ISL_426899  803 LPNDDTLRVEAFEYYHTTDPSFLGRYMSALNHTKKWKYPQVNGLTSIKWADNNCYLATAL
EPI_ISL_426906  803 LPNDDTLRVEAFEYYHTTDPSFLGRYMSALNHTKKWKYPQVNGLTSIKWADNNCYLATAL

ORF1ab         1681 LTLQQIELKFNPPALQDAYYRARAGEAANFCALILAYCNKTVGELGDVRETMSYLFQHAN
EPI_ISL_413571  863 LTLQQIELKFNPPALQDAYYRARAGEAANFCALILAYCNKTVGELGDVRETMSYLFQHAN
EPI_ISL_413574  863 LTLQQIELKFNPPALQDAYYRARAGEAANFCALILAYCNKTVGELGDVRETMSYLFQHAN
EPI_ISL_413579  863 LTLQQIELKFNPPALQDAYYRARAGEAANFCALILAYCNKTVGELGDVRETMSYLFQHAN
EPI_ISL_413584  863 LTLQQIELKFNPPALQDAYYRARAGEAANFCALILAYCNKTVGELGDVRETMSYLFQHAN
EPI_ISL_413587  863 LTLQQIELKFNPPALQDAYYRARAGEAANFCALILAYCNKTVGELGDVRETMSYLFQHAN
EPI_ISL_424257  863 LTLQQIELKFNPPALQDAYYRARAGEAANFCALILAYCNKTVGELGDVRETMSYLFQHAN
EPI_ISL_425565  863 LTLQQIELKFNPPALQDAYYRARAGEAANFCALILAYCNKTVGELGDVRETMSYLFQHAN
EPI_ISL_425575  863 LTLQQIELKFNPPALQDAYYRARAGEAANFCALILAYCNKTVGELGDVRETMSYLFQHAN
EPI_ISL_426822  863 LTLQQIELKFNPPALQDAYYRARAGEAANFCALILAYCNKTVGELGDVRETMSYLFQHAN
EPI_ISL_426833  863 LTLQQIELKFNPPALQDAYYRARAGEAANFCALILAYCNKTVGELGDVRETMSYLFQHAN
EPI_ISL_426885  863 LTLQQIELKFNPPALQDAYYRARAGEAANFCALILAYCNKTVGELGDVRETMSYLFQHAN
EPI_ISL_426886  863 LTLQQIELKFNPPALQDAYYRARAGEAANFCALILAYCNKTVGELGDVRETMSYLFQHAN
EPI_ISL_426889  863 LTLQQIELKFNPPALQDAYYRARAGEAANFCALILAYCNKTVGELGDVRETMSYLFQHAN
EPI_ISL_426891  863 LTLQQIELKFNPPALQDAYYRARAGEAANFCALILAYCNKTVGELGDVRETMSYLFQHAN
EPI_ISL_426892  863 LTLQQIELKFNPPALQDAYYRARAGEAANFCALILAYCNKTVGELGDVRETMSYLFQHAN
EPI_ISL_426894  863 LTLQQIELKFNPPALQDAYYRARAGEAANFCALILAYCNKTVGELGDVRETMSYLFQHAN
EPI_ISL_426896  863 LTLQQIELKFNPPALQDAYYRARAGEAANFCALILAYCNKTVGELGDVRETMSYLFQHAN
EPI_ISL_426897  863 LTLQQIELKFNPPALQDAYYRARAGEAANFCALILAYCNKTVGELGDVRETMSYLFQHAN
EPI_ISL_426899  863 LTLQQIELKFNPPALQDAYYRARAGEAANFCALILAYCNKTVGELGDVRETMSYLFQHAN
EPI_ISL_426906  863 LTLQQIELKFNPPALQDAYYRARAGEAANFCALILAYCNKTVGELGDVRETMSYLFQHAN

ORF1ab         1741 LDSCKRVLNVVCKTCGQQQTTLKGVEAVMYMGTLSYEQFKKGVQIPCTCGKQATKYLVQQ
EPI_ISL_413571  923 LDSCKRVLNVVCKTCGQQQTTLKGVEAVMYMGTLSYEQFKKGVQIPCTCGKQATKYLVQQ
EPI_ISL_413574  923 LDSCKRVLNVVCKTCGQQQTTLKGVEAVMYMGTLSYEQFKKGVQIPCTCGKQATKYLVQQ
EPI_ISL_413579  923 LDSCKRVLNVVCKTCGQQQTTLKGVEAVMYMGTLSYEQFKKGVQIPCTCGKQATKYLVQQ
EPI_ISL_413584  923 LDSCKRVLNVVCKTCGQQQTTLKGVEAVMYMGTLSYEQFKKGVQIPCTCGKQATKYLVQQ
EPI_ISL_413587  923 LDSCKRVLNVVCKTCGQQQTTLKGVEAVMYMGTLSYEQFKKGVQIPCTCGKQATKYLVQQ
EPI_ISL_424257  923 LDSCKRVLNVVCKTCGQQQTTLKGVEAVMYMGTLSYEQFKKGVQIPCTCGKQATKYLVQQ
EPI_ISL_425565  923 LDSCKRVLNVVCKTCGQQQTTLKGVEAVMYMGTLSYEQFKKGVQIPCTCGKQATKYLVQQ
EPI_ISL_425575  923 LDSCKRVLNVVCKTCGQQQTTLKGVEAVMYMGTLSYEQFKKGVQIPCTCGKQATKYLVQQ
EPI_ISL_426822  923 LDSCKRVLNVVCKTCGQQQTTLKGVEAVMYMGTLSYEQFKKGVQIPCTCGKQATKYLVQQ
EPI_ISL_426833  923 LDSCKRVLNVVCKTCGQQQTTLKGVEAVMYMGTLSYEQFKKGVQIPCTCGKQATKYLVQQ
EPI_ISL_426885  923 LDSCKRVLNVVCKTCGQQQTTLKGVEAVMYMGTLSYEQFKKGVQIPCTCGKQATKYLVQQ
EPI_ISL_426886  923 LDSCKRVLNVVCKTCGQQQTTLKGVEAVMYMGTLSYEQFKKGVQIPCTCGKQATKYLVQQ
EPI_ISL_426889  923 LDSCKRVLNVVCKTCGQQQTTLKGVEAVMYMGTLSYEQFKKGVQIPCTCGKQATKYLVQQ
EPI_ISL_426891  923 LDSCKRVLNVVCKTCGQQQTTLKGVEAVMYMGTLSYEQFKKGVQIPCTCGKQATKYLVQQ
EPI_ISL_426892  923 LDSCKRVLNVVCKTCGQQQTTLKGVEAVMYMGTLSYEQFKKGVQIPCTCGKQATKYLVQQ
EPI_ISL_426894  923 LDSCKRVLNVVCKTCGQQQTTLKGVEAVMYMGTLSYEQFKKGVQIPCTCGKQATKYLVQQ
EPI_ISL_426896  923 LDSCKRVLNVVCKTCGQQQTTLKGVEAVMYMGTLSYEQFKKGVQIPCTCGKQATKYLVQQ
EPI_ISL_426897  923 LDSCKRVLNVVCKTCGQQQTTLKGVEAVMYMGTLSYEQFKKGVQIPCTCGKQATKYLVQQ
EPI_ISL_426899  923 LDSCKRVLNVVCKTCGQQQTTLKGVEAVMYMGTLSYEQFKKGVQIPCTCGKQATKYLVQQ
```

EPI_ISL_426906 923 LDSCKRVLNVVCKTCGQQQTTLKGVEAVMYMGTLSYEQFKKGVQIPCTCGKQATKYLVQQ

```
ORF1ab          1801 ESPFVMMSAPPAQYELKHGTFTCASEYTGNYQCGHYKHITSKETLYCIDGALLTKSSEYK
EPI_ISL_413571   983 ESPFVMMSAPPAQYELKHGTFTCASEYTGNYQCGHYKHITSKETLYCIDGALLTKSSEYK
EPI_ISL_413574   983 ESPFVMMSAPPAQYELKHGTFTCASEYTGNYQCGHYKHITSKETLYCIDGALLTKSSEYK
EPI_ISL_413579   983 ESPFVMMSAPPAQYELKHGTFTCASEYTGNYQCGHYKHITSKETLYCIDGALLTKSSEYK
EPI_ISL_413584   983 ESPFVMMSAPPAQYELKHGTFTCASEYTGNYQCGHYKHITSKETLYCIDGALLTKSSEYK
EPI_ISL_413587   983 ESPFVMMSAPPAQYELKHGTFTCASEYTGNYQCGHYKHITSKETLYCIDGALLTKSSEYK
EPI_ISL_424257   983 ESPFVMMSAPPAQYELKHGTFTCASEYTGNYQCGHYKHITSKETLYCIDGALLTKSSEYK
EPI_ISL_425565   983 ESPFVMMSAPPAQYELKHGTFTCASEYTGNYQCGHYKHITSKETLYCIDGALLTKSSEYK
EPI_ISL_425575   983 ESPFVMMSAPPAQYELKHGTFTCASEYTGNYQCGHYKHITSKETLYCIDGALLTKSSEYK
EPI_ISL_426822   983 ESPFVMMSAPPAQYELKHGTFTCASEYTGNYQCGHYKHITSKETLYCIDGALLTKSSEYK
EPI_ISL_426833   983 ESPFVMMSAPPAQYELKHGTFTCASEYTGNYQCGHYKHITSKETLYCIDGALLTKSSEYK
EPI_ISL_426885   983 ESPFVMMSAPPAQYELKHGTFTCASEYTGNYQCGHYKHITSKETLYCIDGALLTKSSEYK
EPI_ISL_426886   983 ESPFVMMSAPPAQYELKHGTFTCASEYTGNYQCGHYKHITSKETLYCIDGALLTKSSEYK
EPI_ISL_426889   983 ESPFVMMSAPPAQYELKHGTFTCASEYTGNYQCGHYKHITSKETLYCIDGALLTKSSEYK
EPI_ISL_426891   983 ESPFVMMSAPPAQYELKHGTFTCASEYTGNYQCGHYKHITSKETLYCIDGALLTKSSEYK
EPI_ISL_426892   983 ESPFVMMSAPPAQYELKHGTFTCASEYTGNYQCGHYKHITSKETLYCIDGALLTKSSEYK
EPI_ISL_426894   983 ESPFVMMSAPPAQYELKHGTFTCASEYTGNYQCGHYKHITSKETLYCIDGALLTKSSEYK
EPI_ISL_426896   983 ESPFVMMSAPPAQYELKHGTFTCASEYTGNYQCGHYKHITSKETLYCIDGALLTKSSEYK
EPI_ISL_426897   983 ESPFVMMSAPPAQYELKHGTFTCASEYTGNYQCGHYKHITSKETLYCIDGALLTKSSEYK
EPI_ISL_426899   983 ESPFVMMSAPPAQYELKHGTFTCASEYTGNYQCGHYKHITSKETLYCIDGALLTKSSEYK
EPI_ISL_426906   983 ESPFVMMSAPPAQYELKHGTFTCASEYTGNYQCGHYKHITSKETLYCIDGALLTKSSEYK

ORF1ab          1861 GPITDVFYKENSYTTTIKPVTYKLDGVVCTEIDPKLDNYYKKDNSYFTEQPIDLVPNQPY
EPI_ISL_413571  1043 GPITDVFYKENSYTTTIKPVTYKLDGVVCTEIDPKLDNYYKKDNSYFTEQPIDLVPNQPY
EPI_ISL_413574  1043 GPITDVFYKENSYTTTIKPVTYKLDGVVCTEIDPKLDNYYKKDNSYFTEQPIDLVPNQPY
EPI_ISL_413579  1043 GPITDVFYKENSYTTTIKPVTYKLDGVVCTEIDPKLDNYYKKDNSYFTEQPIDLVPNQPY
EPI_ISL_413584  1043 GPITDVFYKENSYTTTIKPVTYKLDGVVCTEIDPKLDNYYKKDNSYFTEQPIDLVPNQPY
EPI_ISL_413587  1043 GPITDVFYKENSYTTTIKPVTYKLDGVVCTEIDPKLDNYYKKDNSYFTEQPIDLVPNQPY
EPI_ISL_424257  1043 GPITDVFYKENSYTTTIKPVTYKLDGVVCTEIDPKLDNYYKKDNSYFTEQPIDLVPNQPY
EPI_ISL_425565  1043 GPITDVFYKENSYTTTIKPVTYKLDGVVCTEIDPKLDNYYKKDNSYFTEQPIDLVPNQPY
EPI_ISL_425575  1043 GPITDVFYKENSYTTTIKPVTYKLDGVVCTEIDPKLDNYYKKDNSYFTEQPIDLVPNQPY
EPI_ISL_426822  1043 GPITDVFYKENSYTTTIKPVTYKLDGVVCTEIDPKLDNYYKKDNSYFTEQPIDLVPNQPY
EPI_ISL_426833  1043 GPITDVFYKENSYTTTIKPVTYKLDGVVCTEIDPKLDNYYKKDNSYFTEQPIDLVPNQPY
EPI_ISL_426885  1043 GPITDVFYKENSYTTTIKPVTYKLDGVVCTEIDPKLDNYYKKDNSYFTEQPIDLVPNQPY
EPI_ISL_426886  1043 GPITDVFYKENSYTTTIKPVTYKLDGVVCTEIDPKLDNYYKKDNSYFTEQPIDLVPNQPY
EPI_ISL_426889  1043 GPITDVFYKENSYTTTIKPVTYKLDGVVCTEIDPKLDNYYKKDNSYFTEQPIDLVPNQPY
EPI_ISL_426891  1043 GPITDVFYKENSYTTTIKPVTYKLDGVVCTEIDPKLDNYYKKDNSYFTEQPIDLVPNQPY
EPI_ISL_426892  1043 GPITDVFYKENSYTTTIKPVTYKLDGVVCTEIDPKLDNYYKKDNSYFTEQPIDLVPNQPY
EPI_ISL_426894  1043 GPITDVFYKENSYTTTIKPVTYKLDGVVCTEIDPKLDNYYKKDNSYFTEQPIDLVPNQPY
EPI_ISL_426896  1043 GPITDVFYKENSYTTTIKPVTYKLDGVVCTEIDPKLDNYYKKDNSYFTEQPIDLVPNQPY
EPI_ISL_426897  1043 GPITDVFYKENSYTTTIKPVTYKLDGVVCTEIDPKLDNYYKKDNSYFTEQPIDLVPNQPY
EPI_ISL_426899  1043 GPITDVFYKENSYTTTIKPVTYKLDGVVCTEIDPKLDNYYKKDNSYFTEQPIDLVPNQPY
EPI_ISL_426906  1043 GPITDVFYKENSYTTTIKPVTYKLDGVVCTEIDPKLDNYYKKDNSYFTEQPIDLVPNQPY

ORF1ab          1921 PNASFDNFKFVCDNIKFADDLNQLTGYKKPASRELKVTFFPDLNGDVVAIDYKHYTPSFK
EPI_ISL_413571  1103 PNASFDNFKFVCDNIKFADDLNQLTGYKKPASRELKVTFFPDLNGDVVAIDYKHYTPSFK
EPI_ISL_413574  1103 PNASFDNFKFVCDNIKFADDLNQLTGYKKPASRELKVTFFPDLNGDVVAIDYKHYTPSFK
EPI_ISL_413579  1103 PNASFDNFKFVCDNIKFADDLNQLTGYKKPASRELKVTFFPDLNGDVVAIDYKHYTPSFK
EPI_ISL_413584  1103 SNASFDNFKFVCDNIKFADDLNQLTGYKKPASRELKVTFFPDLNGDVVAIDYKHYTPSFK
EPI_ISL_413587  1103 SNASFDNFKFVCDNIKFADDLNQLTGYKKPASRELKVTFFPDLNGDVVAIDYKHYTPSFK
EPI_ISL_424257  1103 SNASFDNFKFVCDNIKFADDLNQLTGYKKPASRELKVTFFPDLNGDVVAIDYKHYTPSFK
EPI_ISL_425565  1103 PNASFDNFKFVCDNIKFADDLNQLTGYKKPASRELKVTFFPDLNGDVVAIDYKHYTPSFK
EPI_ISL_425575  1103 PNASFDNFKFVCDNIKFADDLNQLTGYKKPASRELKVTFFPDLNGDVVAIDYKHYTPSFK
EPI_ISL_426822  1103 PNASFDNFKFVCDNIKFADDLNQLTGYKKPASRELKVTFFPDLNGDVVAIDYKHYTPSFK
EPI_ISL_426833  1103 PNASFDNFKFVCDNIKFADDLNQLTGYKKPASRELKVTFFPDLNGDVVAIDYKHYTPSFK
EPI_ISL_426885  1103 PNASFDNFKFVCDNIKFADDLNQLTGYKKPASRELKVTFFPDLNGDVVAIDYKHYTPSFK
EPI_ISL_426886  1103 PNASFDNFKFVCDNIKFADDLNQLTGYKKPASRELKVTFFPDLNGDVVAIDYKHYTPSFK
EPI_ISL_426889  1103 PNASFDNFKFVCDNIKFADDLNQLTGYKKPASRELKVTFFPDLNGDVVAIDYKHYTPSFK
EPI_ISL_426891  1103 PNASFDNFKFVCDNIKFADDLNQLTGYKKPASRELKVTFFPDLNGDVVAIDYKHYTPSFK
EPI_ISL_426892  1103 PNASFDNFKFVCDNIKFADDLNQLTGYKKPASRELKVTFFPDLNGDVVAIDYKHYTPSFK
EPI_ISL_426894  1103 PNASFDNFKFVCDNIKFADDLNQLTGYKKPASRELKVTFFPDLNGDVVAIDYKHYTPSFK
EPI_ISL_426896  1103 PNASFDNFKFVCDNIKFADDLNQLTGYKKPASRELKVTFFPDLNGDVVAIDYKHYTPSFK
EPI_ISL_426897  1103 PNASFDNFKFVCDNIKFADDLNQLTGYKKPASRELKVTFFPDLNGDVVAIDYKHYTPSFK
EPI_ISL_426899  1103 PNASFDNFKFVCDNIKFADDLNQLTGYKKPASRELKVTFFPDLNGDVVAIDYKHYTPSFK
```

EPI_ISL_426906 1103 PNASFDNFKFVCDNIKFADDLNQLTGYKKPASRELKVTFFPDLNGDVVAIDYKHYTPSFK

```
ORF1ab         1981 KGAKLLHKPIVWHVNNATNKATYKPNTWCIRCLWSTKPVETSNSFDVLKSEDAQGMDNLA
EPI_ISL_413571 1163 KGAKLLHKPIVWHVNNATNKATYKPNTWCIRCLWSTKPVETSNSFDVLKSEDAQGMDNLA
EPI_ISL_413574 1163 KGAKLLHKPIVWHVNNATNKATYKPNTWCIRCLWSTKPVETSNSFDVLKSEDAQGMDNLA
EPI_ISL_413579 1163 KGAKLLHKPIVWHVNNATNKATYKPNTWCIRCLWSTKPVETSNSFDVLKSEDAQGMDNLA
EPI_ISL_413584 1163 KGAKLLHKPIVWHVNNATNKATYKPNTWCIRCLWSTKPVETSNSFDVLKSEDAQGMDNLA
EPI_ISL_413587 1163 KGAKLLHKPIVWHVNNATNKATYKPNTWCIRCLWSTKPVETSNSFDVLKSEDAQGMDNLA
EPI_ISL_424257 1163 KGAKLLHKPIVWHVNNATNKATYKPNTWCIRCLWSTKPVETSNSFDVLKSEDAQGMDNLA
EPI_ISL_425565 1163 KGAKLLHKPIVWHVNNATNKATYKPNTWCIRCLWSTKPVETSNSFDVLKSEDAQGMDNLA
EPI_ISL_425575 1163 KGAKLLHKPIVWHVNNATNKATYKPNTWCIRCLWSTKPVETSNSFDVLKSEDAQGMDNLA
EPI_ISL_426822 1163 KGAKLLHKPIVWHVNNATNKATYKPNTWCIRCLWSTKPVETSNSFDVLKSEDAQGMDNLA
EPI_ISL_426833 1163 KGAKLLHKPIVWHVNNATNKATYKPNTWCIRCLWSTKPVETSNSFDVLKSEDAQGMDNLA
EPI_ISL_426885 1163 KGAKLLHKPIVWHVNNATNKATYKPNTWCIRCLWSTKPVETSNSFDVLKSEDAQGMDNLA
EPI_ISL_426886 1163 KGAKLLHKPIVWHVNNATNKATYKPNTWCIRCLWSTKPVETSNSFDVLKSEDAQGMDNLA
EPI_ISL_426889 1163 KGAKLLHKPIVWHVNNATNKATYKPNTWCIRCLWSTKPVETSNSFDVLKSEDAQGMDNLA
EPI_ISL_426891 1163 KGAKLLHKPIVWHVNNATNKATYKPNTWCIRCLWSTKPVETSNSFDVLKSEDAQGMDNLA
EPI_ISL_426892 1163 KGAKLLHKPIVWHVNNATNKATYKPNTWCIRCLWSTKPVETSNSFDVLKSEDAQGMDNLA
EPI_ISL_426894 1163 KGAKLLHKPIVWHVNNATNKATYKPNTWCIRCLWSTKPVETSNSFDVLKSEDAQGMDNLA
EPI_ISL_426896 1163 KGAKLLHKPIVWHVNNATNKATYKPNTWCIRCLWSTKPVETSNSFDVLKSEDAQGMDNLA
EPI_ISL_426897 1163 KGAKLLHKPIVWHVNNATNKATYKPNTWCIRCLWSTKPVETSNSFDVLKSEDAQGMDNLA
EPI_ISL_426899 1163 KGAKLLHKPIVWHVNNATNKATYKPNTWCIRCLWSTKPVETSNSFDVLKSEDAQGMDNLA
EPI_ISL_426906 1163 KGAKLLHKPIVWHVNNATNKATYKPNTWCIRCLWSTKPVETSNSFDVLKSEDAQGMDNLA

ORF1ab         2041 CEDLKPVSEEVVENPTIQKDVLECNVKTTEVVGDIILKPANNSLKITEEVGHTDLMAAYV
EPI_ISL_413571 1223 CEDLKPVSEEVVENPTIQKDVLECNVKTTEVVGDIILKPANNSLKITEEVGHTDLMAAYV
EPI_ISL_413574 1223 CEDLKPVSEEVVENPTIQKDVLECNVKTTEVVGDIILKPANNSLKITEEVGHTDLMAAYV
EPI_ISL_413579 1223 CEDLKPVSEEVVENPTIQKDVLECNVKTTEVVGDIILKPANNSLKITEEVGHTDLMAAYV
EPI_ISL_413584 1223 CEDLKPVSEEVVENPTIQKDVLECNVKTTEVVGDIILKPANNSLKITEEVGHTDLMAAYV
EPI_ISL_413587 1223 CEDLKPVSEEVVENPTIQKDVLECNVKTTEVVGDIILKPANNSLKITEEVGHTDLMAAYV
EPI_ISL_424257 1223 CEDLKPVSEEVVENPTIQKDVLECNVKTTEVVGDIILKPANNSLKITEEVGHTDLMAAYV
EPI_ISL_425565 1223 CEDLKPVSEEVVENPTIQKDVLECNVKTTEVVGDIILKPANNSLKITEEVGHTDLMAAYV
EPI_ISL_425575 1223 CEDLKPVSEEVVENPTIQKDVLECNVKTTEVVGDIILKPANNSLKITEEVGHTDLMAAYV
EPI_ISL_426822 1223 CEDLKPVSEEVVENPTIQKDVLECNVKTTEVVGDIILKPANNSLKITEEVGHTDLMAAYV
EPI_ISL_426833 1223 CEDLKPVSEEVVENPTIQKDVLECNVKTTEVVGDIILKPANNSLKITEEVGHTDLMAAYV
EPI_ISL_426885 1223 CEDLKPVSEEVVENPTIQKDVLECNVKTTEVVGDIILKPANNSLKITEEVGHTDLMAAYV
EPI_ISL_426886 1223 CEDLKPVSEEVVENPTIQKDVLECNVKTTEVVGDIILKPANNSLKITEEVGHTDLMAAYV
EPI_ISL_426889 1223 CEDLKPVSEEVVENPTIQKDVLECNVKTTEVVGDIILKPANNSLKITEEVGHTDLMAAYV
EPI_ISL_426891 1223 CEDLKPVSEEVVENPTIQKDVLECNVKTTEVVGDIILKPANNSLKITEEVGHTDLMAAYV
EPI_ISL_426892 1223 CEDLKPVSEEVVENPTIQKDVLECNVKTTEVVGDIILKPANNSLKITEEVGHTDLMAAYV
EPI_ISL_426894 1223 CEDLKPVSEEVVENPTIQKDVLECNVKTTEVVGDIILKPANNSLKITEEVGHTDLMAAYV
EPI_ISL_426896 1223 CEDLKPVSEEVVENPTIQKDVLECNVKTTEVVGDIILKPANNSLKITEEVGHTDLMAAYV
EPI_ISL_426897 1223 CEDLKPVSEEVVENPTIQKDVLECNVKTTEVVGDIILKPANNSLKITEEVGHTDLMAAYV
EPI_ISL_426899 1223 CEDLKPVSEEVVENPTIQKDVLECNVKTTEVVGDIILKPANNSLKITEEVGHTDLMAAYV
EPI_ISL_426906 1223 CEDLKPVSEEVVENPTIQKDVLECNVKTTEVVGDIILKPANNSLKITEEVGHTDLMAAYV

ORF1ab         2101 DNSSLTIKKPNELSRVLGLKTLATHGLAAVNSVPWDTIANYAKPFLNKVVSTTTNIVTRC
EPI_ISL_413571 1283 DNSSLTIKKPNELSRVLGLKTLATHGLAAVNSVPWDTIANYAKPFLNKVVSTTTNIVTRC
EPI_ISL_413574 1283 DNSSLTIKKPNELSRVLGLKTLATHGLAAVNSVPWDTIANYAKPFLNKVVSTTTNIVTRC
EPI_ISL_413579 1283 DNSSLTIKKPNELSRVLGLKTLATHGLAAVNSVPWDTIANYAKPFLNKVVSTTTNIVTRC
EPI_ISL_413584 1283 DNSSLTIKKPNELSRVLGLKTLATHGLAAVNSVPWDTIANYAKPFLNKVVSTTTNIVTRC
EPI_ISL_413587 1283 DNSSLTIKKPNELSRVLGLKTLATHGLAAVNSVPWDTIANYAKPFLNKVVSTTTNIVTRC
EPI_ISL_424257 1283 DNSSLTIKKPNELSRVLGLKTLATHGLAAVNSVPWDTIANYAKPFLNKVVSTTTNIVTRC
EPI_ISL_425565 1283 DNSSLTIKKPNELSRVLGLKTLATHGLAAVNSVPWDTIANYAKPFLNKVVSTTTNIVTRC
EPI_ISL_425575 1283 DNSSLTIKKPNELSRVLGLKTLATHGLAAVNSVPWDTIANYAKPFLNKVVSTTTNIVTRC
EPI_ISL_426822 1283 DNSSLTIKKPNELSRVLGLKTLATHGLAAVNSVPWDTIANYAKPFLNKVVSTTTNIVTRC
EPI_ISL_426833 1283 DNSSLTIKKPNELSRVLGLKTLATHGLAAVNSVPWDTIANYAKPFLNKVVSTTTNIVTRC
EPI_ISL_426885 1283 DNSSLTIKKPNELSRVLGLKTLATHGLAAVNSVPWDTIANYAKPFLNKVVSTTTNIVTRC
EPI_ISL_426886 1283 DNSSLTIKKPNELSRVLGLKTLATHGLAAVNSVPWDTIANYAKPFLNKVVSTTTNIVTRC
EPI_ISL_426889 1283 DNSSLTIKKPNELSRVLGLKTLATHGLAAVNSVPWDTIANYAKPFLNKVVSTTTNIVTRC
EPI_ISL_426891 1283 DNSSLTIKKPNELSRVLGLKTLATHGLAAVNSVPWDTIANYAKPFLNKVVSTTTNIVTRC
EPI_ISL_426892 1283 DNSSLTIKKPNELSRVLGLKTLATHGLAAVNSVPWDTIANYAKPFLNKVVSTTTNIVTRC
EPI_ISL_426894 1283 DNSSLTIKKPNELSRVLGLKTLATHGLAAVNSVPWDTIANYAKPFLNKVVSTTTNIVTRC
EPI_ISL_426896 1283 DNSSLTIKKPNELSRVLGLKTLATHGLAAVNSVPWDTIANYAKPFLNKVVSTTTNIVTRC
EPI_ISL_426897 1283 DNSSLTIKKPNELSRVLGLKTLATHGLAAVNSVPWDTIANYAKPFLNKVVSTTTNIVTRC
EPI_ISL_426899 1283 DNSSLTIKKPNELSRVLGLKTLATHGLAAVNSVPWDTIANYAKPFLNKVVSTTTNIVTRC
```

EPI_ISL_426906 1283 DNSSLTIKKPNELSRVLGLKTLATHGLAAVNSVPWDTIANYAKPFLNKVVSTTTNIVTRC

```
ORF1ab          2161 LNRVCTNYMPYFFTLLLQLCTFTRSTNSRIKASMPTTIAKNTVKSVGKFCLEASFNYLKS
EPI_ISL_413571 1343 LNRVCTNYMPYFFTLLLQLCTFTRSTNSRIKASMPTTIAKNTVKSVGKFCLEASFNYLKS
EPI_ISL_413574 1343 LNRVCTNYMPYFFTLLLQLCTFTRSTNSRIKASMPTTIAKNTVKSVGKFCLEASFNYLKS
EPI_ISL_413579 1343 LNRVCTNYMPYFFTLLLQLCTFTRSTNSRIKASMPTTIAKNTVKSVGKFCLEASFNYLKS
EPI_ISL_413584 1343 LNRVCTNYMPYFFTLLLQLCTFTRSTNSRIKASMPTTIAKNTVKSVGKFCLEASFNYLKS
EPI_ISL_413587 1343 LNRVCTNYMPYFFTLLLQLCTFTRSTNSRIKASMPTTIAKNTVKSVGKFCLEASFNYLKS
EPI_ISL_424257 1343 LNRVCTNYMPYFFTLLLQLCTFTRSTNSRIKASMPTTIAKNTVKSVGKFCLEASFNYLKS
EPI_ISL_425565 1343 LNRVCTNYMPYFFTLLLQLCTFTRSTNSRIKASMPTTIAKNTVKSVGKFCLEASFNYLKS
EPI_ISL_425575 1343 LNRVCTNYMPYFFTLLLQLCTFTRSTNSRIKASMPTTIAKNTVKSVGKFCLEASFNYLKS
EPI_ISL_426822 1343 LNRVCTNYMPYFFTLLLQLCTFTRSTNSRIKASMPTTIAKNTVKSVGKFCLEASFNYLKS
EPI_ISL_426833 1343 LNRVCTNYMPYFFTLLLQLCTFTRSTNSRIKASMPTTIAKNTVKSVGKFCLEASFNYLKS
EPI_ISL_426885 1343 LNRVCTNYMPYFFTLLLQLCTFTRSTNSRIKASMPTTIAKNTVKSVGKFCLEASFNYLKS
EPI_ISL_426886 1343 LNRVCTNYMPYFFTLLLQLCTFTRSTNSRIKASMPTTIAKNTVKSVGKFCLEASFNYLKS
EPI_ISL_426889 1343 LNRVCTNYMPYFFTLLLQLCTFTRSTNSRIKASMPTTIAKNTVKSVGKFCLEASFNYLKS
EPI_ISL_426891 1343 LNRVCTNYMPYFFTLLLQLCTFTRSTNSRIKASMPTTIAKNTVKSVGKFCLEASFNYLKS
EPI_ISL_426892 1343 LNRVCTNYMPYFFTLLLQLCTFTRSTNSRIKASMPTTIAKNTVKSVGKFCLEASFNYLKS
EPI_ISL_426894 1343 LNRVCTNYMPYFFTLLLQLCTFTRSTNSRIKASMPTTIAKNTVKSVGKFCLEASFNYLKS
EPI_ISL_426896 1343 LNRVCTNYMPYFFTLLLQLCTFTRSTNSRIKASMPTTIAKNTVKSVGKFCLEASFNYLKS
EPI_ISL_426897 1343 LNRVCTNYMPYFFTLLLQLCTFTRSTNSRIKASMPTTIAKNTVKSVGKFCLEASFNYLKS
EPI_ISL_426899 1343 LNRVCTNYMPYFFTLLLQLCTFTRSTNSRIKASMPTTIAKNTVKSVGKFCLEASFNYLKS
EPI_ISL_426906 1343 LNRVCTNYMPYFFTLLLQLCTFTRSTNSRIKASMPTTIAKNTVKSVGKFCLEASFNYLKS

ORF1ab          2221 PNFSKLINIIIWFLLLSVCLGSLIYSTAALGVLMSNLGMPSYCTGYREGYLNSTNVTIAT
EPI_ISL_413571 1403 PNFSKLINIIIWFLLLSVCLGSLIYSTAALGVLMSNLGMPSYCTGYREGYLNSTNVTIAT
EPI_ISL_413574 1403 PNFSKLINIIIWFLLLSVCLGSLIYSTAALGVLMSNLGMPSYCTGYREGYLNSTNVTIAT
EPI_ISL_413579 1403 PNFSKLINIIIWFLLLSVCLGSLIYSTAALGVLMSNLGMPSYCTGYREGYLNSTNVTIAT
EPI_ISL_413584 1403 PNFSKLINIIIWFLLLSVCLGSLIYSTAALGVLMSNLGMPSYCTGYREGYLNSTNVTIAT
EPI_ISL_413587 1403 PNFSKLINIIIWFLLLSVCLGSLIYSTAALGVLMSNLGMPSYCTGYREGYLNSTNVTIAT
EPI_ISL_424257 1403 PNFSKLINIIIWFLLLSVCLGSLIYSTAALGVLMSNLGMPSYCTGYREGYLNSTNVTIAT
EPI_ISL_425565 1403 PNFSKLINIIIWFLLLSVCLGSLIYSTAALGVLMSNLGMPSYCTGYREGYLNSTNVTIAT
EPI_ISL_425575 1403 PNFSKLINIIIWFLLLSVCLGSLIYSTAALGVLMSNLGMPSYCTGYREGYLNSTNVTIAT
EPI_ISL_426822 1403 PNFSKLINIIIWFLLLSVCLGSLIYSTAALGVLMSNLGMPSYCTGYREGYLNSTNVTIAT
EPI_ISL_426833 1403 PNFSKLINIIIWFLLLSVCLGSLIYSTAALGVLMSNLGMPSYCTGYREGYLNSTNVTIAT
EPI_ISL_426885 1403 PNFSKLINIIIWFLLLSVCLGSLIYSTAALGVLMSNLGMPSYCTGYREGYLNSTNVTIAT
EPI_ISL_426886 1403 PNFSKLINIIIWFLLLSVCLGSLIYSTAALGVLMSNLGMPSYCTGYREGYLNSTNVTIAT
EPI_ISL_426889 1403 PNFSKLINIIIWFLLLSVCLGSLIYSTAALGVLMSNLGMPSYCTGYREGYLNSTNVTIAT
EPI_ISL_426891 1403 PNFSKLINIIIWFLLLSVCLGSLIYSTAALGVLMSNLGMPSYCTGYREGYLNSTNVTIAT
EPI_ISL_426892 1403 PNFSKLINIIIWFLLLSVCLGSLIYSTAALGVLMSNLGMPSYCTGYREGYLNSTNVTIAT
EPI_ISL_426894 1403 PNFSKLINIIIWFLLLSVCLGSLIYSTAALGVLMSNLGMPSYCTGYREGYLNSTNVTIAT
EPI_ISL_426896 1403 PNFSKLINIIIWFLLLSVCLGSLIYSTAALGVLMSNLGMPSYCTGYREGYLNSTNVTIAT
EPI_ISL_426897 1403 PNFSKLINIIIWFLLLSVCLGSLIYSTAALGVLMSNLGMPSYCTGYREGYLNSTNVTIAT
EPI_ISL_426899 1403 PNFSKLINIIIWFLLLSVCLGSLIYSTAALGVLMSNLGMPSYCTGYREGYLNSTNVTIAT
EPI_ISL_426906 1403 PNFSKLINIIIWFLLLSVCLGSLIYSTAALGVLMSNLGMPSYCTGYREGYLNSTNVTIAT

ORF1ab          2281 YCTGSIPCSVCLSGLDSLDTYPSLETIQITISSFKWDLTAFGLVAEWFLAYILFTRFFYV
EPI_ISL_413571 1463 YCTGSIPCSVCLSGLDSLDTYPSLETIQITISSFKWDLTAFGLVAEWFLAYILFTRFFYV
EPI_ISL_413574 1463 YCTGSIPCSVCLSGLDSLDTYPSLETIQITISSFKWDLTAFGLVAEWFLAYILFTRFFYV
EPI_ISL_413579 1463 YCTGSIPCSVCLSGLDSLDTYPSLETIQITISSFKWDLTAFGLVAEWFLAYILFTRFFYV
EPI_ISL_413584 1463 YCTGSIPCSVCLSGLDSLDTYPSLETIQITISSFKWDLTAFGLVAEWFLAYILFTRFFYV
EPI_ISL_413587 1463 YCTGSIPCSVCLSGLDSLDTYPSLETIQITISSFKWDLTAFGLVAEWFLAYILFTRFFYV
EPI_ISL_424257 1463 YCTGSIPCSVCLSGLDSLDTYPSLETIQITISSFKWDLTAFGLVAEWFLAYILFTRFFYV
EPI_ISL_425565 1463 YCTGSIPCSVCLSGLDSLDTYPSLETIQITISSFKWDLTAFGLVAEWFLAYILFTRFFYV
EPI_ISL_425575 1463 YCTGSIPCSVCLSGLDSLDTYPSLETIQITISSFKWDLTAFGLVAEWFLAYILFTRFFYV
EPI_ISL_426822 1463 YCTGSIPCSVCLSGLDSLDTYPSLETIQITISSFKWDLTAFGLVAEWFLAYILFTRFFYV
EPI_ISL_426833 1463 YCTGSIPCSVCLSGLDSLDTYPSLETIQITISSFKWDLTAFGLVAEWFLAYILFTRFFYV
EPI_ISL_426885 1463 YCTGSIPCSVCLSGLDSLDTYPSLETIQITISSFKWDLTAFGLVAEWFLAYILFTRFFYV
EPI_ISL_426886 1463 YCTGSIPCSVCLSGLDSLDTYPSLETIQITISSFKWDLTAFGLVAEWFLAYILFTRFFYV
EPI_ISL_426889 1463 YCTGSIPCSVCLSGLDSLDTYPSLETIQITISSFKWDLTAFGLVAEWFLAYILFTRFFYV
EPI_ISL_426891 1463 YCTGSIPCSVCLSGLDSLDTYPSLETIQITISSFKWDLTAFGLVAEWFLAYILFTRFFYV
EPI_ISL_426892 1463 YCTGSIPCSVCLSGLDSLDTYPSLETIQITISSFKWDLTAFGLVAEWFLAYILFTRFFYV
EPI_ISL_426894 1463 YCTGSIPCSVCLSGLDSLDTYPSLETIQITISSFKWDLTAFGLVAEWFLAYILFTRFFYV
EPI_ISL_426896 1463 YCTGSIPCSVCLSGLDSLDTYPSLETIQITISSFKWDLTAFGLVAEWFLAYILFTRFFYV
EPI_ISL_426897 1463 YCTGSIPCSVCLSGLDSLDTYPSLETIQITISSFKWDLTAFGLVAEWFLAYILFTRFFYV
EPI_ISL_426899 1463 YCTGSIPCSVCLSGLDSLDTYPSLETIQITISSFKWDLTAFGLVAEWFLAYILFTRFFYV
```

EPI_ISL_426906 1463 YCTGSIPCSVCLSGLDSLDTYPSLETIQITISSFKWDLTAFGLVAEWFLAYILFTRFFYV

```
ORF1ab          2341 LGLAAIMQLFFSYFAVHFISNSWLMWLIINLVQMAPISAMVRMYIFFASFYYVWKSYVHV
EPI_ISL_413571 1523 LGLAAIMQLFFSYFAVHFISNSWLMWLIINLVQMAPISAMVRMYIFFASFYYVWKSYVHV
EPI_ISL_413574 1523 LGLAAIMQLFFSYFAVHFISNSWLMWLIINLVQMAPISAMVRMYIFFASFYYVWKSYVHV
EPI_ISL_413579 1523 LGLAAIMQLFFSYFAVHFISNSWLMWLIINLVQMAPISAMVRMYIFFASFYYVWKSYVHV
EPI_ISL_413584 1523 LGLAAIMQLFFSYFAVHFISNSWLMWLIINLVQMAPISAMVRMYIFFASFYYVWKSYVHV
EPI_ISL_413587 1523 LGLAAIMQLFFSYFAVHFISNSWLMWLIINLVQMAPISAMVRMYIFFASFYYVWKSYVHV
EPI_ISL_424257 1523 LGLAAIMQLFFSYFAVHFISNSWLMWLIINLVQMAPISAMVRMYIFFASFYYVWKSYVHV
EPI_ISL_425565 1523 LGLAAIMQLFFSYFAVHFISNSWLMWLIINLVQMAPISAMVRMYIFFASFYYVWKSYVHV
EPI_ISL_425575 1523 LGLAAIMQLFFSYFAVHFISNSWLMWLIINLVQMAPISAMVRMYIFFASFYYVWKSYVHV
EPI_ISL_426822 1523 LGLAAIMQLFFSYFAVHFISNSWLMWLIINLVQMAPISAMVRMYIFFASFYYVWKSYVHV
EPI_ISL_426833 1523 LGLAAIMQLFFSYFAVHFISNSWLMWLIINLVQMAPISAMVRMYIFFASFYYVWKSYVHV
EPI_ISL_426885 1523 LGLAAIMQLFFSYFAVHFISNSWLMWLIINLVQMAPISAMVRMYIFFASFYYVWKSYVHV
EPI_ISL_426886 1523 LGLAAIMQLFFSYFAVHFISNSWLMWLIINLVQMAPISAMVRMYIFFASFYYVWKSYVHV
EPI_ISL_426889 1523 LGLAAIMQLFFSYFAVHFISNSWLMWLIINLVQMAPISAMVRMYIFFASFYYVWKSYVHV
EPI_ISL_426891 1523 LGLAAIMQLFFSYFAVHFISNSWLMWLIINLVQMAPISAMVRMYIFFASFYYVWKSYVHV
EPI_ISL_426892 1523 LGLAAIMQLFFSYFAVHFISNSWLMWLIINLVQMAPISAMVRMYIFFASFYYVWKSYVHV
EPI_ISL_426894 1523 LGLAAIMQLFFSYFAVHFISNSWLMWLIINLVQMAPISAMVRMYIFFASFYYVWKSYVHV
EPI_ISL_426896 1523 LGLAAIMQLFFSYFAVHFISNSWLMWLIINLVQMAPISAMVRMYIFFASFYYVWKSYVHV
EPI_ISL_426897 1523 LGLAAIMQLFFSYFAVHFISNSWLMWLIINLVQMAPISAMVRMYIFFASFYYVWKSYVHV
EPI_ISL_426899 1523 LGLAAIMQLFFSYFAVHFISNSWLMWLIINLVQMAPISAMVRMYIFFASFYYVWKSYVHV
EPI_ISL_426906 1523 LGLAAIMQLFFSYFAVHFISNSWLMWLIINLVQMAPISAMVRMYIFFASFYYVWKSYVHV

ORF1ab          2401 VDGCNSSTCMMCYKRNRATRVECTTIVNGVRRSFYVYANGGKGFCKLHNWNCVNCDTFCA
EPI_ISL_413571 1583 VDGCNSSTCMMCYKRNRATRVECTTIVNGVRRSFYVYANGGKGFCKLHNWNCVNCDTFCA
EPI_ISL_413574 1583 VDGCNSSTCMMCYKRNRATRVECTTIVNGVRRSFYVYANGGKGFCKLHNWNCVNCDTFCA
EPI_ISL_413579 1583 VDGCNSSTCMMCYKRNRATRVECTTIVNGVRRSFYVYANGGKGFCKLHNWNCVNCDTFCA
EPI_ISL_413584 1583 VDGCNSSTCMMCYKRNRATRVECTTIVNGVRRSFYVYANGGKGFCKLHNWNCVNCDTFCA
EPI_ISL_413587 1583 VDGCNSSTCMMCYKRNRATRVECTTIVNGVRRSFYVYANGGKGFCKLHNWNCVNCDTFCA
EPI_ISL_424257 1583 VDGCNSSTCMMCYKRNRATRVECTTIVNGVRRSFYVYANGGKGFCKLHNWNCVNCDTFCA
EPI_ISL_425565 1583 VDGCNSSTCMMCYKRNRATRVECTTIVNGVRRSFYVYANGGKGFCKLHNWNCVNCDTFCA
EPI_ISL_425575 1583 VDGCNSSTCMMCYKRNRATRVECTTIVNGVRRSFYVYANGGKGFCKLHNWNCVNCDTFCA
EPI_ISL_426822 1583 VDGCNSSTCMMCYKRNRATRVECTTIVNGVRRSFYVYANGGKGFCKLHNWNCVNCDTFCA
EPI_ISL_426833 1583 VDGCNSSTCMMCYKRNRATRVECTTIVNGVRRSFYVYANGGKGFCKLHNWNCVNCDTFCA
EPI_ISL_426885 1583 VDGCNSSTCMMCYKRNRATRVECTTIVNGVRRSFYVYANGGKGFCKLHNWNCVNCDTFCA
EPI_ISL_426886 1583 VDGCNSSTCMMCYKRNRATRVECTTIVNGVRRSFYVYANGGKGFCKLHNWNCVNCDTFCA
EPI_ISL_426889 1583 VDGCNSSTCMMCYKRNRATRVECTTIVNGVRRSFYVYANGGKGFCKLHNWNCVNCDTFCA
EPI_ISL_426891 1583 VDGCNSSTCMMCYKRNRATRVECTTIVNGVRRSFYVYANGGKGFCKLHNWNCVNCDTFCA
EPI_ISL_426892 1583 VDGCNSSTCMMCYKRNRATRVECTTIVNGVRRSFYVYANGGKGFCKLHNWNCVNCDTFCA
EPI_ISL_426894 1583 VDGCNSSTCMMCYKRNRATRVECTTIVNGVRRSFYVYANGGKGFCKLHNWNCVNCDTFCA
EPI_ISL_426896 1583 VDGCNSSTCMMCYKRNRATRVECTTIVNGVRRSFYVYANGGKGFCKLHNWNCVNCDTFCA
EPI_ISL_426897 1583 VDGCNSSTCMMCYKRNRATRVECTTIVNGVRRSFYVYANGGKGFCKLHNWNCVNCDTFCA
EPI_ISL_426899 1583 VDGCNSSTCMMCYKRNRATRVECTTIVNGVRRSFYVYANGGKGFCKLHNWNCVNCDTFCA
EPI_ISL_426906 1583 VDGCNSSTCMMCYKRNRATRVECTTIVNGVRRSFYVYANGGKGFCKLHNWNCVNCDTFCA

ORF1ab          2461 GSTFISDEVARDLSLQFKRPINPTDQSSYIVDSVTVKNGSIHLYFDKAGQKTYERHSLSH
EPI_ISL_413571 1643 GSTFISDEVARDLSLQFKRPINPTDQSSYIVDSVTVKNGSIHLYFDKAGQKTYERHSLSH
EPI_ISL_413574 1643 GSTFISDEVARDLSLQFKRPINPTDQSSYIVDSVTVKNGSIHLYFDKAGQKTYERHSLSH
EPI_ISL_413579 1643 GSTFISDEVARDLSLQFKRPINPTDQSSYIVDSVTVKNGSIHLYFDKAGQKTYERHSLSH
EPI_ISL_413584 1643 GSTFISDEVARDLSLQFKRPINPTDQSSYIVDSVTVKNGSIHLYFDKAGQKTYERHSLSH
EPI_ISL_413587 1643 GSTFISDEVARDLSLQFKRPINPTDQSSYIVDSVTVKNGSIHLYFDKAGQKTYERHSLSH
EPI_ISL_424257 1643 GSTFISDEVARDLSLQFKRPINPTDQSSYIVDSVTVKNGSIHLYFDKAGQKTYERHSLSH
EPI_ISL_425565 1643 GSTFISDEVARDLSLQFKRPINPTDQSSYIVDSVTVKNGSIHLYFDKAGQKTYERHSLSH
EPI_ISL_425575 1643 GSTFISDEVARDLSLQFKRPINPTDQSSYIVDSVTVKNGSIHLYFDKAGQKTYERHSLSH
EPI_ISL_426822 1643 GSTFISDEVARDLSLQFKRPINPTDQSSYIVDSVTVKNGSIHLYFDKAGQKTYERHSLSH
EPI_ISL_426833 1643 GSTFISDEVARDLSLQFKRPINPTDQSSYIVDSVTVKNGSIHLYFDKAGQKTYERHSLSH
EPI_ISL_426885 1643 GSTFISDEVARDLSLQFKRPINPTDQSSYIVDSVTVKNGSIHLYFDKAGQKTYERHSLSH
EPI_ISL_426886 1643 GSTFISDEVARDLSLQFKRPINPTDQSSYIVDSVTVKNGSIHLYFDKAGQKTYERHSLSH
EPI_ISL_426889 1643 GSTFISDEVARDLSLQFKRPINPTDQSSYIVDSVTVKNGSIHLYFDKAGQKTYERHSLSH
EPI_ISL_426891 1643 GSTFISDEVARDLSLQFKRPINPTDQSSYIVDSVTVKNGSIHLYFDKAGQKTYERHSLSH
EPI_ISL_426892 1643 GSTFISDEVARDLSLQFKRPINPTDQSSYIVDSVTVKNGSIHLYFDKAGQKTYERHSLSH
EPI_ISL_426894 1643 GSTFISDEVARDLSLQFKRPINPTDQSSYIVDSVTVKNGSIHLYFDKAGQKTYERHSLSH
EPI_ISL_426896 1643 GSTFISDEVARDLSLQFKRPINPTDQSSYIVDSVTVKNGSIHLYFDKAGQKTYERHSLSH
EPI_ISL_426897 1643 GSTFISDEVARDLSLQFKRPINPTDQSSYIVDSVTVKNGSIHLYFDKAGQKTYERHSLSH
EPI_ISL_426899 1643 GSTFISDEVARDLSLQFKRPINPTDQSSYIVDSVTVKNGSIHLYFDKAGQKTYERHSLSH
```

EPI_ISL_426906 1643 GSTFISDEVARDLSLQFKRPINPTDQSSYIVDSVTVKNGSIHLYFDKAGQKTYERHSLSH

```
ORF1ab         2521 FVNLDNLRANNTKGSLPINVIVFDGKSKCEESSAKSASVYYSQLMCQPILLLDQALVSDV
EPI_ISL_413571 1703 FVNLDNLRANNTKGSLPINVIVFDGKSKCEESSAKSASVYYSQLMCQPILLLDQALVSDV
EPI_ISL_413574 1703 FVNLDNLRANNTKGSLPINVIVFDGKSKCEESSAKSASVYYSQLMCQPILLLDQALVSDV
EPI_ISL_413579 1703 FVNLDNLRANNTKGSLPINVIVFDGKSKCEESSAKSASVYYSQLMCQPILLLDQALVSDV
EPI_ISL_413584 1703 FVNLDNLRANNTKGSLPINVIVFDGKSKCEESSAKSASVYYSQLMCQPILLLDQALVSDV
EPI_ISL_413587 1703 FVNLDNLRANNTKGSLPINVIVFDGKSKCEESSAKSASVYYSQLMCQPILLLDQALVSDV
EPI_ISL_424257 1703 FVNLDNLRANNTKGSLPINVIVFDGKSKCEESSAKSASVYYSQLMCQPILLLDQALVSDV
EPI_ISL_425565 1703 FVNLDNLRANNTKGSLPINVIVFDGKSKCEESSAKSASVYYSQLMCQPILLLDQALVSDV
EPI_ISL_425575 1703 FVNLDNLRANNTKGSLPINVIVFDGKSKCEESSAKSASVYYSQLMCQPILLLDQALVSDV
EPI_ISL_426822 1703 FVNLDNLRANNTKGSLPINVIVFDGKSKCEESSAKSASVYYSQLMCQPILLLDQALVSDV
EPI_ISL_426833 1703 FVNLDNLRANNTKGSLPINVIVFDGKSKCEESSAKSASVYYSQLMCQPILLLDQALVSDV
EPI_ISL_426885 1703 FVNLDNLRANNTKGSLPINVIVFDGKSKCEESSAKSASVYYSQLMCQPILLLDQALVSDV
EPI_ISL_426886 1703 FVNLDNLRANNTKGSLPINVIVFDGKSKCEESSAKSASVYYSQLMCQPILLLDQALVSDV
EPI_ISL_426889 1703 FVNLDNLRANNTKGSLPINVIVFDGKSKCEESSAKSASVYYSQLMCQPILLLDQALVSDV
EPI_ISL_426891 1703 FVNLDNLRANNTKGSLPINVIVFDGKSKCEESSAKSASVYYSQLMCQPILLLDQALVSDV
EPI_ISL_426892 1703 FVNLDNLRANNTKGSLPINVIVFDGKSKCEESSAKSASVYYSQLMCQPILLLDQALVSDV
EPI_ISL_426894 1703 FVNLDNLRANNTKGSLPINVIVFDGKSKCEESSAKSASVYYSQLMCQPILLLDQALVSDV
EPI_ISL_426896 1703 FVNLDNLRANNTKGSLPINVIVFDGKSKCEESSAKSASVYYSQLMCQPILLLDQALVSDV
EPI_ISL_426897 1703 FVNLDNLRANNTKGSLPINVIVFDGKSKCEESSAKSASVYYSQLMCQPILLLDQALVSDV
EPI_ISL_426899 1703 FVNLDNLRANNTKGSLPINVIVFDGKSKCEESSAKSASVYYSQLMCQPILLLDQALVSDV
EPI_ISL_426906 1703 FVNLDNLRANNTKGSLPINVIVFDGKSKCEESSAKSASVYYSQLMCQPILLLDQALVSDV

ORF1ab         2581 GDSAEVAVKMFDAYVNTFSSTFNVPMEKLKTLVATAEAELAKNVSLDNVLSTFISAARQG
EPI_ISL_413571 1763 GDSAEVAVKMFDAYVNTFSSTFNVPMEKLKTLVATAEAELAKNVSLDNVLSTFISAARQG
EPI_ISL_413574 1763 GDSAEVAVKMFDAYVNTFSSTFNVPMEKLKTLVATAEAELAKNVSLDNVLSTFISAARQG
EPI_ISL_413579 1763 GDSAEVAVKMFDAYVNTFSSTFNVPMEKLKTLVATAEAELAKNVSLDNVLSTFISAARQG
EPI_ISL_413584 1763 GDSAEVAVKMFDAYVNTFSSTFNVPMEKLKTLVATAEAELAKNVSLDNVLSTFISAARQG
EPI_ISL_413587 1763 GDSAEVAVKMFDAYVNTFSSTFNVPMEKLKTLVATAEAELAKNVSLDNVLSTFISAARQG
EPI_ISL_424257 1763 GDSAEVAVKMFDAYVNTFSSTFNVPMEKLKTLVATAEAELAKNVSLDNVLSTFISAARQG
EPI_ISL_425565 1763 GDSAEVAVKMFDAYVDTFSSTFNVPMEKLKTLVATAEAELAKNVSLDNVLSTFISAARQG
EPI_ISL_425575 1763 GDSAEVAVKMFDAYVNTFSSTFNVPMEKLKTLVATAEAELAKNVSLDNVLSTFISAARQG
EPI_ISL_426822 1763 GDSAEVAVKMFDAYVNTFSSTFNVPMEKLKTLVATAEAELAKNVSLDNVLSTFISAARQG
EPI_ISL_426833 1763 GDSAEVAVKMFDAYVNTFSSTFNVPMEKLKTLVATAEAELAKNVSLDNVLSTFISAARQG
EPI_ISL_426885 1763 GDSAEVAVKMFDAYVNTFSSTFNVPMEKLKTLVATAEAELAKNVSLDNVLSTFISAARQG
EPI_ISL_426886 1763 GDSAEVAVKMFDAYVNTFSSTFNVPMEKLKTLVATAEAELAKNVSLDNVLSTFISAARQG
EPI_ISL_426889 1763 GDSAEVAVKMFDAYVNTFSSTFNVPMEKLKTLVATAEAELAKNVSLDNVLSTFISAARQG
EPI_ISL_426891 1763 GDSAEVAVKMFDAYVNTFSSTFNVPMEKLKTLVATAEAELAKNVSLDNVLSTFISAARQG
EPI_ISL_426892 1763 GDSAEVAVKMFDAYVNTFSSTFNVPMEKLKTLVATAEAELAKNVSLDNVLSTFISAARQG
EPI_ISL_426894 1763 GDSAEVAVKMFDAYVNTFSSTFNVPMEKLKTLVATAEAELAKNVSLDNVLSTFISAARQG
EPI_ISL_426896 1763 GDSAEVAVKMFDAYVNTFSSTFNVPMEKLKTLVATAEAELAKNVSLDNVLSTFISAARQG
EPI_ISL_426897 1763 GDSAEVAVKMFDAYVNTFSSTFNVPMEKLKTLVATAEAELAKNVSLDNVLSTFISAARQG
EPI_ISL_426899 1763 GDSAEVAVKMFDAYVNTFSSTFNVPMEKLKTLVATAEAELAKNVSLDNVLSTFISAARQG
EPI_ISL_426906 1763 GDSAEVAVKMFDAYVNTFSSTFNVPMEKLKTLVATAEAELAKNVSLDNVLSTFISAARQG

ORF1ab         2641 FVDSDVETKDVVECLKLSHQSDIEVTGDSCNNYMLTYNKVENMTPRDLGACIDCSARHIN
EPI_ISL_413571 1823 FVDSDVETKDVVECLKLSHQSDIEVTGDSCNNYMLTYNKVENMTPRDLGACIDCSARHIN
EPI_ISL_413574 1823 FVDSDVETKDVVECLKLSHQSDIEVTGDSCNNYMLTYNKVENMTPRDLGACIDCSARHIN
EPI_ISL_413579 1823 FVDSDVETKDVVECLKLSHQSDIEVTGDSCNNYMLTYNKVENMTPRDLGACIDCSARHIN
EPI_ISL_413584 1823 FVDSDVETKDVVECLKLSHQSDIEVTGDSCNNYMLTYNKVENMTPRDLGACIDCSARHIN
EPI_ISL_413587 1823 FVDSDVETKDVVECLKLSHQSDIEVTGDSCNNYMLTYNKVENMTPRDLGACIDCSARHIN
EPI_ISL_424257 1823 FVDSDVETKDVVECLKLSHQSDIEVTGDSCNNYMLTYNKVENMTPRDLGACIDCSARHIN
EPI_ISL_425565 1823 FVDSDVETKDVVECLKLSHQSDIEVTGDSCNNYMLTYNKVENMTPRDLGACIDCSARHIN
EPI_ISL_425575 1823 FVDSDVETKDVVECLKLSHQSDIEVTGDSCNNYMLTYNKVENMTPRDLGACIDCSARHIN
EPI_ISL_426822 1823 FVDSDVETKDVVECLKLSHQSDIEVTGDSCNNYMLTYNKVENMTPRDLGACIDCSARHIN
EPI_ISL_426833 1823 FVDSDVETKDVVECLKLSHQSDIEVTGDSCNNYMLTYNKVENMTPRDLGACIDCSARHIN
EPI_ISL_426885 1823 FVDSDVETKDVVECLKLSHQSDIEVTGDSCNNYMLTYNKVENMTPRDLGACIDCSARHIN
EPI_ISL_426886 1823 FVDSDVETKDVVECLKLSHQSDIEVTGDSCNNYMLTYNKVENMTPRDLGACIDCSARHIN
EPI_ISL_426889 1823 FVDSDVETKDVVECLKLSHQSDIEVTGDSCNNYMLTYNKVENMTPRDLGACIDCSARHIN
EPI_ISL_426891 1823 FVDSDVETKDVVECLKLSHQSDIEVTGDSCNNYMLTYNKVENMTPRDLGACIDCSARHIN
EPI_ISL_426892 1823 FVDSDVETKDVVECLKLSHQSDIEVTGDSCNNYMLTYNKVENMTPRDLGACIDCSARHIN
EPI_ISL_426894 1823 FVDSDVETKDVVECLKLSHQSDIEVTGDSCNNYMLTYNKVENMTPRDLGACIDCSARHIN
EPI_ISL_426896 1823 FVDSDVETKDVVECLKLSHQSDIEVTGDSCNNYMLTYNKVENMTPRDLGACIDCSARHIN
EPI_ISL_426897 1823 FVDSDVETKDVVECLKLSHQSDIEVTGDSCNNYMLTYNKVENMTPRDLGACIDCSARHIN
EPI_ISL_426899 1823 FVDSDVETKDVVECLKLSHQSDIEVTGDSCNNYMLTYNKVENMTPRDLGACIDCSARHIN
```

EPI_ISL_426906 1823 FVDSDVETKDVVECLKLSHQSDIEVTGDSCNNYMLTYNKVENMTPRDLGACIDCSARHIN

```
ORF1ab         2701 AQVAKSHNIALIWNVKDFMSLSEQLRKQIRSAAKKNNLPFKLTCATTRQVVNVVTTKIAL
EPI_ISL_413571 1883 AQVAKSHNIALIWNVKDFMSLSEQLRKQIRSAAKKNNLPFKLTCATTRQVVNVVTTKIAL
EPI_ISL_413574 1883 AQVAKSHNIALIWNVKDFMSLSEQLRKQIRSAAKKNNLPFKLTCATTRQVVNVVTTKIAL
EPI_ISL_413579 1883 AQVAKSHNIALIWNVKDFMSLSEQLRKQIRSAAKKNNLPFKLTCATTRQVVNVVTTKIAL
EPI_ISL_413584 1883 AQVAKSHNIALIWNVKDFMSLSEQLRKQIRSAAKKNNLPFKLTCATTRQVVNVVTTKIAL
EPI_ISL_413587 1883 AQVAKSHNIALIWNVKDFMSLSEQLRKQIRSAAKKNNLPFKLTCATTRQVVNVVTTKIAL
EPI_ISL_424257 1883 AQVAKSHNIALIWNVKDFMSLSEQLRKQIRSAAKKNNLPFKLTCATTRQVVNVVTTKIAL
EPI_ISL_425565 1883 AQVAKSHNIALIWNVKDFMSLSEQLRKQIRSAAKKNNLPFKLTCATTRQVVNVVTTKIAL
EPI_ISL_425575 1883 AQVAKSHNIALIWNVKDFMSLSEQLRKQIRSAAKKNNLPFKLTCATTRQVVNVVTTKIAL
EPI_ISL_426822 1883 AQVAKSHNIALIWNVKDFMSLSEQLRKQIRSAAKKNNLPFKLTCATTRQVVNVVTTKIAL
EPI_ISL_426833 1883 AQVAKSHNIALIWNVKDFMSLSEQLRKQIRSAAKKNNLPFKLTCATTRQVVNVVTTKIAL
EPI_ISL_426885 1883 AQVAKSHNIALIWNVKDFMSLSEQLRKQIRSAAKKNNLPFKLTCATTRQVVNVVTTKIAL
EPI_ISL_426886 1883 AQVAKSHNIALIWNVKDFMSLSEQLRKQIRSAAKKNNLPFKLTCATTRQVVNVVTTKIAL
EPI_ISL_426889 1883 AQVAKSHNIALIWNVKDFMSLSEQLRKQIRSAAKKNNLPFKLTCATTRQVVNVVTTKIAL
EPI_ISL_426891 1883 AQVAKSHNIALIWNVKDFMSLSEQLRKQIRSAAKKNNLPFKLTCATTRQVVNVVTTKIAL
EPI_ISL_426892 1883 AQVAKSHNIALIWNVKDFMSLSEQLRKQIRSAAKKNNLPFKLTCATTRQVVNVVTTKIAL
EPI_ISL_426894 1883 AQVAKSHNIALIWNVKDFMSLSEQLRKQIRSAAKKNNLPFKLTCATTRQVVNVVTTKIAL
EPI_ISL_426896 1883 AQVAKSHNIALIWNVKDFMSLSEQLRKQIRSAAKKNNLPFKLTCATTRQVVNVVTTKIAL
EPI_ISL_426897 1883 AQVAKSHNIALIWNVKDFMSLSEQLRKQIRSAAKKNNLPFKLTCATTRQVVNVVTTKIAL
EPI_ISL_426899 1883 AQVAKSHNIALIWNVKDFMSLSEQLRKQIRSAAKKNNLPFKLTCATTRQVVNVVTTKIAL
EPI_ISL_426906 1883 AQVAKSHNIALIWNVKDFMSLSEQLRKQIRSAAKKNNLPFKLTCATTRQVVNVVTTKIAL

ORF1ab         2761 KGGKIVNNWLKQLIKVTLVFLFVAAIFYLITPVHVMSKHTDFSSEIIGYKAIDGGVTRDI
EPI_ISL_413571 1943 KGG---------------------------------------------------------
EPI_ISL_413574 1943 KGG---------------------------------------------------------
EPI_ISL_413579 1943 KGG---------------------------------------------------------
EPI_ISL_413584 1943 KGG---------------------------------------------------------
EPI_ISL_413587 1943 KGG---------------------------------------------------------
EPI_ISL_424257 1943 KGG---------------------------------------------------------
EPI_ISL_425565 1943 KGG---------------------------------------------------------
EPI_ISL_425575 1943 KGG---------------------------------------------------------
EPI_ISL_426822 1943 KGG---------------------------------------------------------
EPI_ISL_426833 1943 KGG---------------------------------------------------------
EPI_ISL_426885 1943 KGG---------------------------------------------------------
EPI_ISL_426886 1943 KGG---------------------------------------------------------
EPI_ISL_426889 1943 KGG---------------------------------------------------------
EPI_ISL_426891 1943 KGG---------------------------------------------------------
EPI_ISL_426892 1943 KGG---------------------------------------------------------
EPI_ISL_426894 1943 KGG---------------------------------------------------------
EPI_ISL_426896 1943 KGG---------------------------------------------------------
EPI_ISL_426897 1943 KGG---------------------------------------------------------
EPI_ISL_426899 1943 KGG---------------------------------------------------------
EPI_ISL_426906 1943 KGG---------------------------------------------------------

ORF1ab         2821 ASTDTCFANKHADFDTWFSQRGGSYTNDKACPLIAAVITREVGFVVPGLPGTILRTTNGD
EPI_ISL_413571      ------------------------------------------------------------
EPI_ISL_413574      ------------------------------------------------------------
EPI_ISL_413579      ------------------------------------------------------------
EPI_ISL_413584      ------------------------------------------------------------
EPI_ISL_413587      ------------------------------------------------------------
EPI_ISL_424257      ------------------------------------------------------------
EPI_ISL_425565      ------------------------------------------------------------
EPI_ISL_425575      ------------------------------------------------------------
EPI_ISL_426822      ------------------------------------------------------------
EPI_ISL_426833      ------------------------------------------------------------
EPI_ISL_426885      ------------------------------------------------------------
EPI_ISL_426886      ------------------------------------------------------------
EPI_ISL_426889      ------------------------------------------------------------
EPI_ISL_426891      ------------------------------------------------------------
EPI_ISL_426892      ------------------------------------------------------------
EPI_ISL_426894      ------------------------------------------------------------
EPI_ISL_426896      ------------------------------------------------------------
EPI_ISL_426897      ------------------------------------------------------------
EPI_ISL_426899      ------------------------------------------------------------
```

EPI_ISL_426906   --

```
ORF1ab          2881 FLHFLPRVFSAVGNICYTPSKLIEYTDFATSACVLAAECTIFKDASGKPVPYCYDTNVLE
EPI_ISL_413571       ------------------------------------------------------------
EPI_ISL_413574       ------------------------------------------------------------
EPI_ISL_413579       ------------------------------------------------------------
EPI_ISL_413584       ------------------------------------------------------------
EPI_ISL_413587       ------------------------------------------------------------
EPI_ISL_424257       ------------------------------------------------------------
EPI_ISL_425565       ------------------------------------------------------------
EPI_ISL_425575       ------------------------------------------------------------
EPI_ISL_426822       ------------------------------------------------------------
EPI_ISL_426833       ------------------------------------------------------------
EPI_ISL_426885       ------------------------------------------------------------
EPI_ISL_426886       ------------------------------------------------------------
EPI_ISL_426889       ------------------------------------------------------------
EPI_ISL_426891       ------------------------------------------------------------
EPI_ISL_426892       ------------------------------------------------------------
EPI_ISL_426894       ------------------------------------------------------------
EPI_ISL_426896       ------------------------------------------------------------
EPI_ISL_426897       ------------------------------------------------------------
EPI_ISL_426899       ------------------------------------------------------------
EPI_ISL_426906       ------------------------------------------------------------

ORF1ab          2941 GSVAYESLRPDTRYVLMDGSIIQFPNTYLEGSVRVVTTFDSEYCRHGTCERSEAGVCVST
EPI_ISL_413571       ------------------------------------------------------------
EPI_ISL_413574       ------------------------------------------------------------
EPI_ISL_413579       ------------------------------------------------------------
EPI_ISL_413584       ------------------------------------------------------------
EPI_ISL_413587       ------------------------------------------------------------
EPI_ISL_424257       ------------------------------------------------------------
EPI_ISL_425565       ------------------------------------------------------------
EPI_ISL_425575       ------------------------------------------------------------
EPI_ISL_426822       ------------------------------------------------------------
EPI_ISL_426833       ------------------------------------------------------------
EPI_ISL_426885       ------------------------------------------------------------
EPI_ISL_426886       ------------------------------------------------------------
EPI_ISL_426889       ------------------------------------------------------------
EPI_ISL_426891       ------------------------------------------------------------
EPI_ISL_426892       ------------------------------------------------------------
EPI_ISL_426894       ------------------------------------------------------------
EPI_ISL_426896       ------------------------------------------------------------
EPI_ISL_426897       ------------------------------------------------------------
EPI_ISL_426899       ------------------------------------------------------------
EPI_ISL_426906       ------------------------------------------------------------

ORF1ab          3001 SGRWVLNNDYYRSLPGVFCGVDAVNLLTNMFTPLIQPIGALDISASIVAGGIVAIVVTCL
EPI_ISL_413571       ------------------------------------------------------------
EPI_ISL_413574       ------------------------------------------------------------
EPI_ISL_413579       ------------------------------------------------------------
EPI_ISL_413584       ------------------------------------------------------------
EPI_ISL_413587       ------------------------------------------------------------
EPI_ISL_424257       ------------------------------------------------------------
EPI_ISL_425565       ------------------------------------------------------------
EPI_ISL_425575       ------------------------------------------------------------
EPI_ISL_426822       ------------------------------------------------------------
EPI_ISL_426833       ------------------------------------------------------------
EPI_ISL_426885       ------------------------------------------------------------
EPI_ISL_426886       ------------------------------------------------------------
EPI_ISL_426889       ------------------------------------------------------------
EPI_ISL_426891       ------------------------------------------------------------
EPI_ISL_426892       ------------------------------------------------------------
EPI_ISL_426894       ------------------------------------------------------------
EPI_ISL_426896       ------------------------------------------------------------
EPI_ISL_426897       ------------------------------------------------------------
EPI_ISL_426899       ------------------------------------------------------------
```

EPI_ISL_426906 --

```
ORF1ab          3061 AYYFMRFRRAFGEYSHVVAFNTLLFLMSFTVLCLTPVYSFLPGVYSVIYLYLTFYLTNDV
EPI_ISL_413571       ------------------------------------------------------------
EPI_ISL_413574       ------------------------------------------------------------
EPI_ISL_413579       ------------------------------------------------------------
EPI_ISL_413584       ------------------------------------------------------------
EPI_ISL_413587       ------------------------------------------------------------
EPI_ISL_424257       ------------------------------------------------------------
EPI_ISL_425565       ------------------------------------------------------------
EPI_ISL_425575       ------------------------------------------------------------
EPI_ISL_426822       ------------------------------------------------------------
EPI_ISL_426833       ------------------------------------------------------------
EPI_ISL_426885       ------------------------------------------------------------
EPI_ISL_426886       ------------------------------------------------------------
EPI_ISL_426889       ------------------------------------------------------------
EPI_ISL_426891       ------------------------------------------------------------
EPI_ISL_426892       ------------------------------------------------------------
EPI_ISL_426894       ------------------------------------------------------------
EPI_ISL_426896       ------------------------------------------------------------
EPI_ISL_426897       ------------------------------------------------------------
EPI_ISL_426899       ------------------------------------------------------------
EPI_ISL_426906       ------------------------------------------------------------
```

```
ORF1ab          3121 SFLAHIQWMVMFTPLVPFWITIAYIICISTKHFYWFFSNYLKRRVVFNGVSFSTFEEAAL
EPI_ISL_413571       ------------------------------------------------------------
EPI_ISL_413574       ------------------------------------------------------------
EPI_ISL_413579       ------------------------------------------------------------
EPI_ISL_413584       ------------------------------------------------------------
EPI_ISL_413587       ------------------------------------------------------------
EPI_ISL_424257       ------------------------------------------------------------
EPI_ISL_425565       ------------------------------------------------------------
EPI_ISL_425575       ------------------------------------------------------------
EPI_ISL_426822       ------------------------------------------------------------
EPI_ISL_426833       ------------------------------------------------------------
EPI_ISL_426885       ------------------------------------------------------------
EPI_ISL_426886       ------------------------------------------------------------
EPI_ISL_426889       ------------------------------------------------------------
EPI_ISL_426891       ------------------------------------------------------------
EPI_ISL_426892       ------------------------------------------------------------
EPI_ISL_426894       ------------------------------------------------------------
EPI_ISL_426896       ------------------------------------------------------------
EPI_ISL_426897       ------------------------------------------------------------
EPI_ISL_426899       ------------------------------------------------------------
EPI_ISL_426906       ------------------------------------------------------------
```

```
ORF1ab          3181 CTFLLNKEMYLKLRSDVLLPLTQYNRYLALYNKYKYFSGAMDTTSYREAACCHLAKALND
EPI_ISL_413571       ------------------------------------------------------------
EPI_ISL_413574       ------------------------------------------------------------
EPI_ISL_413579       ------------------------------------------------------------
EPI_ISL_413584       ------------------------------------------------------------
EPI_ISL_413587       ------------------------------------------------------------
EPI_ISL_424257       ------------------------------------------------------------
EPI_ISL_425565       ------------------------------------------------------------
EPI_ISL_425575       ------------------------------------------------------------
EPI_ISL_426822       ------------------------------------------------------------
EPI_ISL_426833       ------------------------------------------------------------
EPI_ISL_426885       ------------------------------------------------------------
EPI_ISL_426886       ------------------------------------------------------------
EPI_ISL_426889       ------------------------------------------------------------
EPI_ISL_426891       ------------------------------------------------------------
EPI_ISL_426892       ------------------------------------------------------------
EPI_ISL_426894       ------------------------------------------------------------
EPI_ISL_426896       ------------------------------------------------------------
EPI_ISL_426897       ------------------------------------------------------------
EPI_ISL_426899       ------------------------------------------------------------
```

EPI_ISL_426906 --

```
ORF1ab         3241 FSNSGSDVLYQPPQTSITSAVLQSGFRKMAFPSGKVEGCMVQVTCGTTTLNGLWLDDVVY
EPI_ISL_413571      ------------------------------------------------------------
EPI_ISL_413574      ------------------------------------------------------------
EPI_ISL_413579      ------------------------------------------------------------
EPI_ISL_413584      ------------------------------------------------------------
EPI_ISL_413587      ------------------------------------------------------------
EPI_ISL_424257      ------------------------------------------------------------
EPI_ISL_425565      ------------------------------------------------------------
EPI_ISL_425575      ------------------------------------------------------------
EPI_ISL_426822      ------------------------------------------------------------
EPI_ISL_426833      ------------------------------------------------------------
EPI_ISL_426885      ------------------------------------------------------------
EPI_ISL_426886      ------------------------------------------------------------
EPI_ISL_426889      ------------------------------------------------------------
EPI_ISL_426891      ------------------------------------------------------------
EPI_ISL_426892      ------------------------------------------------------------
EPI_ISL_426894      ------------------------------------------------------------
EPI_ISL_426896      ------------------------------------------------------------
EPI_ISL_426897      ------------------------------------------------------------
EPI_ISL_426899      ------------------------------------------------------------
EPI_ISL_426906      ------------------------------------------------------------

ORF1ab         3301 CPRHVICTSEDMLNPNYEDLLIRKSNHNFLVQAGNVQLRVIGHSMQNCVLKLKVDTANPK
EPI_ISL_413571      ------------------------------------------------------------
EPI_ISL_413574      ------------------------------------------------------------
EPI_ISL_413579      ------------------------------------------------------------
EPI_ISL_413584      ------------------------------------------------------------
EPI_ISL_413587      ------------------------------------------------------------
EPI_ISL_424257      ------------------------------------------------------------
EPI_ISL_425565      ------------------------------------------------------------
EPI_ISL_425575      ------------------------------------------------------------
EPI_ISL_426822      ------------------------------------------------------------
EPI_ISL_426833      ------------------------------------------------------------
EPI_ISL_426885      ------------------------------------------------------------
EPI_ISL_426886      ------------------------------------------------------------
EPI_ISL_426889      ------------------------------------------------------------
EPI_ISL_426891      ------------------------------------------------------------
EPI_ISL_426892      ------------------------------------------------------------
EPI_ISL_426894      ------------------------------------------------------------
EPI_ISL_426896      ------------------------------------------------------------
EPI_ISL_426897      ------------------------------------------------------------
EPI_ISL_426899      ------------------------------------------------------------
EPI_ISL_426906      ------------------------------------------------------------

ORF1ab         3361 TPKYKFVRIQPGQTFSVLACYNGSPSGVYQCAMRPNFTIKGSFLNGSCGSVGFNIDYDCV
EPI_ISL_413571      ------------------------------------------------------------
EPI_ISL_413574      ------------------------------------------------------------
EPI_ISL_413579      ------------------------------------------------------------
EPI_ISL_413584      ------------------------------------------------------------
EPI_ISL_413587      ------------------------------------------------------------
EPI_ISL_424257      ------------------------------------------------------------
EPI_ISL_425565      ------------------------------------------------------------
EPI_ISL_425575      ------------------------------------------------------------
EPI_ISL_426822      ------------------------------------------------------------
EPI_ISL_426833      ------------------------------------------------------------
EPI_ISL_426885      ------------------------------------------------------------
EPI_ISL_426886      ------------------------------------------------------------
EPI_ISL_426889      ------------------------------------------------------------
EPI_ISL_426891      ------------------------------------------------------------
EPI_ISL_426892      ------------------------------------------------------------
EPI_ISL_426894      ------------------------------------------------------------
EPI_ISL_426896      ------------------------------------------------------------
EPI_ISL_426897      ------------------------------------------------------------
EPI_ISL_426899      ------------------------------------------------------------
```

EPI_ISL_426906   --

```
ORF1ab          3421 SFCYMHHMELPTGVHAGTDLEGNFYGPFVDRQTAQAAGTDTTITVNVLAWLYAAVINGDR
EPI_ISL_413571       ------------------------------------------------------------
EPI_ISL_413574       ------------------------------------------------------------
EPI_ISL_413579       ------------------------------------------------------------
EPI_ISL_413584       ------------------------------------------------------------
EPI_ISL_413587       ------------------------------------------------------------
EPI_ISL_424257       ------------------------------------------------------------
EPI_ISL_425565       ------------------------------------------------------------
EPI_ISL_425575       ------------------------------------------------------------
EPI_ISL_426822       ------------------------------------------------------------
EPI_ISL_426833       ------------------------------------------------------------
EPI_ISL_426885       ------------------------------------------------------------
EPI_ISL_426886       ------------------------------------------------------------
EPI_ISL_426889       ------------------------------------------------------------
EPI_ISL_426891       ------------------------------------------------------------
EPI_ISL_426892       ------------------------------------------------------------
EPI_ISL_426894       ------------------------------------------------------------
EPI_ISL_426896       ------------------------------------------------------------
EPI_ISL_426897       ------------------------------------------------------------
EPI_ISL_426899       ------------------------------------------------------------
EPI_ISL_426906       ------------------------------------------------------------

ORF1ab          3481 WFLNRFTTTLNDFNLVAMKYNYEPLTQDHVDILGPLSAQTGIAVLDMCASLKELLQNGMN
EPI_ISL_413571       ------------------------------------------------------------
EPI_ISL_413574       ------------------------------------------------------------
EPI_ISL_413579       ------------------------------------------------------------
EPI_ISL_413584       ------------------------------------------------------------
EPI_ISL_413587       ------------------------------------------------------------
EPI_ISL_424257       ------------------------------------------------------------
EPI_ISL_425565       ------------------------------------------------------------
EPI_ISL_425575       ------------------------------------------------------------
EPI_ISL_426822       ------------------------------------------------------------
EPI_ISL_426833       ------------------------------------------------------------
EPI_ISL_426885       ------------------------------------------------------------
EPI_ISL_426886       ------------------------------------------------------------
EPI_ISL_426889       ------------------------------------------------------------
EPI_ISL_426891       ------------------------------------------------------------
EPI_ISL_426892       ------------------------------------------------------------
EPI_ISL_426894       ------------------------------------------------------------
EPI_ISL_426896       ------------------------------------------------------------
EPI_ISL_426897       ------------------------------------------------------------
EPI_ISL_426899       ------------------------------------------------------------
EPI_ISL_426906       ------------------------------------------------------------

ORF1ab          3541 GRTILGSALLEDEFTPFDVVRQCSGVTFQSAVKRTIKGTHHWLLLTILTSLLVLVQSTQW
EPI_ISL_413571       ------------------------------------------------------------
EPI_ISL_413574       ------------------------------------------------------------
EPI_ISL_413579       ------------------------------------------------------------
EPI_ISL_413584       ------------------------------------------------------------
EPI_ISL_413587       ------------------------------------------------------------
EPI_ISL_424257       ------------------------------------------------------------
EPI_ISL_425565       ------------------------------------------------------------
EPI_ISL_425575       ------------------------------------------------------------
EPI_ISL_426822       ------------------------------------------------------------
EPI_ISL_426833       ------------------------------------------------------------
EPI_ISL_426885       ------------------------------------------------------------
EPI_ISL_426886       ------------------------------------------------------------
EPI_ISL_426889       ------------------------------------------------------------
EPI_ISL_426891       ------------------------------------------------------------
EPI_ISL_426892       ------------------------------------------------------------
EPI_ISL_426894       ------------------------------------------------------------
EPI_ISL_426896       ------------------------------------------------------------
EPI_ISL_426897       ------------------------------------------------------------
EPI_ISL_426899       ------------------------------------------------------------
```

EPI_ISL_426906 --

```
ORF1ab         3601 SLFFFLYENAFLPFAMGIIAMSAFAMMFVKHKHAFLCLFLLPSLATVAYFNMVYMPASWV
EPI_ISL_413571      ------------------------------------------------------------
EPI_ISL_413574      ------------------------------------------------------------
EPI_ISL_413579      ------------------------------------------------------------
EPI_ISL_413584      ------------------------------------------------------------
EPI_ISL_413587      ------------------------------------------------------------
EPI_ISL_424257      ------------------------------------------------------------
EPI_ISL_425565      ------------------------------------------------------------
EPI_ISL_425575      ------------------------------------------------------------
EPI_ISL_426822      ------------------------------------------------------------
EPI_ISL_426833      ------------------------------------------------------------
EPI_ISL_426885      ------------------------------------------------------------
EPI_ISL_426886      ------------------------------------------------------------
EPI_ISL_426889      ------------------------------------------------------------
EPI_ISL_426891      ------------------------------------------------------------
EPI_ISL_426892      ------------------------------------------------------------
EPI_ISL_426894      ------------------------------------------------------------
EPI_ISL_426896      ------------------------------------------------------------
EPI_ISL_426897      ------------------------------------------------------------
EPI_ISL_426899      ------------------------------------------------------------
EPI_ISL_426906      ------------------------------------------------------------

ORF1ab         3661 MRIMTWLDMVDTSLSGFKLKDCVMYASAVVLLILMTARTVYDDGARRVWTLMNVLTLVYK
EPI_ISL_413571      ------------------------------------------------------------
EPI_ISL_413574      ------------------------------------------------------------
EPI_ISL_413579      ------------------------------------------------------------
EPI_ISL_413584      ------------------------------------------------------------
EPI_ISL_413587      ------------------------------------------------------------
EPI_ISL_424257      ------------------------------------------------------------
EPI_ISL_425565      ------------------------------------------------------------
EPI_ISL_425575      ------------------------------------------------------------
EPI_ISL_426822      ------------------------------------------------------------
EPI_ISL_426833      ------------------------------------------------------------
EPI_ISL_426885      ------------------------------------------------------------
EPI_ISL_426886      ------------------------------------------------------------
EPI_ISL_426889      ------------------------------------------------------------
EPI_ISL_426891      ------------------------------------------------------------
EPI_ISL_426892      ------------------------------------------------------------
EPI_ISL_426894      ------------------------------------------------------------
EPI_ISL_426896      ------------------------------------------------------------
EPI_ISL_426897      ------------------------------------------------------------
EPI_ISL_426899      ------------------------------------------------------------
EPI_ISL_426906      ------------------------------------------------------------

ORF1ab         3721 VYYGNALDQAISMWALIISVTSNYSGVVTTVMFLARGIVFMCVEYCPIFFITGNTLQCIM
EPI_ISL_413571      ------------------------------------------------------------
EPI_ISL_413574      ------------------------------------------------------------
EPI_ISL_413579      ------------------------------------------------------------
EPI_ISL_413584      ------------------------------------------------------------
EPI_ISL_413587      ------------------------------------------------------------
EPI_ISL_424257      ------------------------------------------------------------
EPI_ISL_425565      ------------------------------------------------------------
EPI_ISL_425575      ------------------------------------------------------------
EPI_ISL_426822      ------------------------------------------------------------
EPI_ISL_426833      ------------------------------------------------------------
EPI_ISL_426885      ------------------------------------------------------------
EPI_ISL_426886      ------------------------------------------------------------
EPI_ISL_426889      ------------------------------------------------------------
EPI_ISL_426891      ------------------------------------------------------------
EPI_ISL_426892      ------------------------------------------------------------
EPI_ISL_426894      ------------------------------------------------------------
EPI_ISL_426896      ------------------------------------------------------------
EPI_ISL_426897      ------------------------------------------------------------
EPI_ISL_426899      ------------------------------------------------------------
```

EPI_ISL_426906 --

```
ORF1ab               3781 LVYCFLGYFCTCYFGLFCLLNRYFRLTLGVYDYLVSTQEFRYMNSQGLLPPKNSIDAFKL
EPI_ISL_413571            ------------------------------------------------------------
EPI_ISL_413574            ------------------------------------------------------------
EPI_ISL_413579            ------------------------------------------------------------
EPI_ISL_413584            ------------------------------------------------------------
EPI_ISL_413587            ------------------------------------------------------------
EPI_ISL_424257            ------------------------------------------------------------
EPI_ISL_425565            ------------------------------------------------------------
EPI_ISL_425575            ------------------------------------------------------------
EPI_ISL_426822            ------------------------------------------------------------
EPI_ISL_426833            ------------------------------------------------------------
EPI_ISL_426885            ------------------------------------------------------------
EPI_ISL_426886            ------------------------------------------------------------
EPI_ISL_426889            ------------------------------------------------------------
EPI_ISL_426891            ------------------------------------------------------------
EPI_ISL_426892            ------------------------------------------------------------
EPI_ISL_426894            ------------------------------------------------------------
EPI_ISL_426896            ------------------------------------------------------------
EPI_ISL_426897            ------------------------------------------------------------
EPI_ISL_426899            ------------------------------------------------------------
EPI_ISL_426906            ------------------------------------------------------------

ORF1ab               3841 NIKLLGVGGKPCIKVATVQSKMSDVKCTSVVLLSVLQQLRVESSSKLWAQCVQLHNDILL
EPI_ISL_413571            ------------------------------------------------------------
EPI_ISL_413574            ------------------------------------------------------------
EPI_ISL_413579            ------------------------------------------------------------
EPI_ISL_413584            ------------------------------------------------------------
EPI_ISL_413587            ------------------------------------------------------------
EPI_ISL_424257            ------------------------------------------------------------
EPI_ISL_425565            ------------------------------------------------------------
EPI_ISL_425575            ------------------------------------------------------------
EPI_ISL_426822            ------------------------------------------------------------
EPI_ISL_426833            ------------------------------------------------------------
EPI_ISL_426885            ------------------------------------------------------------
EPI_ISL_426886            ------------------------------------------------------------
EPI_ISL_426889            ------------------------------------------------------------
EPI_ISL_426891            ------------------------------------------------------------
EPI_ISL_426892            ------------------------------------------------------------
EPI_ISL_426894            ------------------------------------------------------------
EPI_ISL_426896            ------------------------------------------------------------
EPI_ISL_426897            ------------------------------------------------------------
EPI_ISL_426899            ------------------------------------------------------------
EPI_ISL_426906            ------------------------------------------------------------

ORF1ab               3901 AKDTTEAFEKMVSLLSVLLSMQGAVDINKLCEEMLDNRATLQAIASEFSSLPSYAAFATA
EPI_ISL_413571            ------------------------------------------------------------
EPI_ISL_413574            ------------------------------------------------------------
EPI_ISL_413579            ------------------------------------------------------------
EPI_ISL_413584            ------------------------------------------------------------
EPI_ISL_413587            ------------------------------------------------------------
EPI_ISL_424257            ------------------------------------------------------------
EPI_ISL_425565            ------------------------------------------------------------
EPI_ISL_425575            ------------------------------------------------------------
EPI_ISL_426822            ------------------------------------------------------------
EPI_ISL_426833            ------------------------------------------------------------
EPI_ISL_426885            ------------------------------------------------------------
EPI_ISL_426886            ------------------------------------------------------------
EPI_ISL_426889            ------------------------------------------------------------
EPI_ISL_426891            ------------------------------------------------------------
EPI_ISL_426892            ------------------------------------------------------------
EPI_ISL_426894            ------------------------------------------------------------
EPI_ISL_426896            ------------------------------------------------------------
EPI_ISL_426897            ------------------------------------------------------------
EPI_ISL_426899            ------------------------------------------------------------
```

EPI_ISL_426906   --

```
ORF1ab         3961 QEAYEQAVANGDSEVVLKKLKKSLNVAKSEFDRDAAMQRKLEKMADQAMTQMYKQARSED
EPI_ISL_413571      ------------------------------------------------------------
EPI_ISL_413574      ------------------------------------------------------------
EPI_ISL_413579      ------------------------------------------------------------
EPI_ISL_413584      ------------------------------------------------------------
EPI_ISL_413587      ------------------------------------------------------------
EPI_ISL_424257      ------------------------------------------------------------
EPI_ISL_425565      ------------------------------------------------------------
EPI_ISL_425575      ------------------------------------------------------------
EPI_ISL_426822      ------------------------------------------------------------
EPI_ISL_426833      ------------------------------------------------------------
EPI_ISL_426885      ------------------------------------------------------------
EPI_ISL_426886      ------------------------------------------------------------
EPI_ISL_426889      ------------------------------------------------------------
EPI_ISL_426891      ------------------------------------------------------------
EPI_ISL_426892      ------------------------------------------------------------
EPI_ISL_426894      ------------------------------------------------------------
EPI_ISL_426896      ------------------------------------------------------------
EPI_ISL_426897      ------------------------------------------------------------
EPI_ISL_426899      ------------------------------------------------------------
EPI_ISL_426906      ------------------------------------------------------------

ORF1ab         4021 KRAKVTSAMQTMLFTMLRKLDNDALNNIINNARDGCVPLNIIPLTTAAKLMVVIPDYNTY
EPI_ISL_413571      ------------------------------------------------------------
EPI_ISL_413574      ------------------------------------------------------------
EPI_ISL_413579      ------------------------------------------------------------
EPI_ISL_413584      ------------------------------------------------------------
EPI_ISL_413587      ------------------------------------------------------------
EPI_ISL_424257      ------------------------------------------------------------
EPI_ISL_425565      ------------------------------------------------------------
EPI_ISL_425575      ------------------------------------------------------------
EPI_ISL_426822      ------------------------------------------------------------
EPI_ISL_426833      ------------------------------------------------------------
EPI_ISL_426885      ------------------------------------------------------------
EPI_ISL_426886      ------------------------------------------------------------
EPI_ISL_426889      ------------------------------------------------------------
EPI_ISL_426891      ------------------------------------------------------------
EPI_ISL_426892      ------------------------------------------------------------
EPI_ISL_426894      ------------------------------------------------------------
EPI_ISL_426896      ------------------------------------------------------------
EPI_ISL_426897      ------------------------------------------------------------
EPI_ISL_426899      ------------------------------------------------------------
EPI_ISL_426906      ------------------------------------------------------------

ORF1ab         4081 KNTCDGTTFTYASALWEIQQVVDADSKIVQLSEISMDNSPNLAWPLIVTALRANSAVKLQ
EPI_ISL_413571      ------------------------------------------------------------
EPI_ISL_413574      ------------------------------------------------------------
EPI_ISL_413579      ------------------------------------------------------------
EPI_ISL_413584      ------------------------------------------------------------
EPI_ISL_413587      ------------------------------------------------------------
EPI_ISL_424257      ------------------------------------------------------------
EPI_ISL_425565      ------------------------------------------------------------
EPI_ISL_425575      ------------------------------------------------------------
EPI_ISL_426822      ------------------------------------------------------------
EPI_ISL_426833      ------------------------------------------------------------
EPI_ISL_426885      ------------------------------------------------------------
EPI_ISL_426886      ------------------------------------------------------------
EPI_ISL_426889      ------------------------------------------------------------
EPI_ISL_426891      ------------------------------------------------------------
EPI_ISL_426892      ------------------------------------------------------------
EPI_ISL_426894      ------------------------------------------------------------
EPI_ISL_426896      ------------------------------------------------------------
EPI_ISL_426897      ------------------------------------------------------------
EPI_ISL_426899      ------------------------------------------------------------
```

EPI_ISL_426906 --

```
ORF1ab         4141 NNELSPVALRQMSCAAGTTQTACTDDNALAYYNTTKGGRFVLALLSDLQDLKWARFPKSD
EPI_ISL_413571      ------------------------------------------------------------
EPI_ISL_413574      ------------------------------------------------------------
EPI_ISL_413579      ------------------------------------------------------------
EPI_ISL_413584      ------------------------------------------------------------
EPI_ISL_413587      ------------------------------------------------------------
EPI_ISL_424257      ------------------------------------------------------------
EPI_ISL_425565      ------------------------------------------------------------
EPI_ISL_425575      ------------------------------------------------------------
EPI_ISL_426822      ------------------------------------------------------------
EPI_ISL_426833      ------------------------------------------------------------
EPI_ISL_426885      ------------------------------------------------------------
EPI_ISL_426886      ------------------------------------------------------------
EPI_ISL_426889      ------------------------------------------------------------
EPI_ISL_426891      ------------------------------------------------------------
EPI_ISL_426892      ------------------------------------------------------------
EPI_ISL_426894      ------------------------------------------------------------
EPI_ISL_426896      ------------------------------------------------------------
EPI_ISL_426897      ------------------------------------------------------------
EPI_ISL_426899      ------------------------------------------------------------
EPI_ISL_426906      ------------------------------------------------------------

ORF1ab         4201 GTGTIYTELEPPCRFVTDTPKGPKVKYLYFIKGLNNLNRGMVLGSLAATVRLQAGNATEV
EPI_ISL_413571      ------------------------------------------------------------
EPI_ISL_413574      ------------------------------------------------------------
EPI_ISL_413579      ------------------------------------------------------------
EPI_ISL_413584      ------------------------------------------------------------
EPI_ISL_413587      ------------------------------------------------------------
EPI_ISL_424257      ------------------------------------------------------------
EPI_ISL_425565      ------------------------------------------------------------
EPI_ISL_425575      ------------------------------------------------------------
EPI_ISL_426822      ------------------------------------------------------------
EPI_ISL_426833      ------------------------------------------------------------
EPI_ISL_426885      ------------------------------------------------------------
EPI_ISL_426886      ------------------------------------------------------------
EPI_ISL_426889      ------------------------------------------------------------
EPI_ISL_426891      ------------------------------------------------------------
EPI_ISL_426892      ------------------------------------------------------------
EPI_ISL_426894      ------------------------------------------------------------
EPI_ISL_426896      ------------------------------------------------------------
EPI_ISL_426897      ------------------------------------------------------------
EPI_ISL_426899      ------------------------------------------------------------
EPI_ISL_426906      ------------------------------------------------------------

ORF1ab         4261 PANSTVLSFCAFAVDAAKAYKDYLASGGQPITNCVKMLCTHTGTGQAITVTPEANMDQES
EPI_ISL_413571      ------------------------------------------------------------
EPI_ISL_413574      ------------------------------------------------------------
EPI_ISL_413579      ------------------------------------------------------------
EPI_ISL_413584      ------------------------------------------------------------
EPI_ISL_413587      ------------------------------------------------------------
EPI_ISL_424257      ------------------------------------------------------------
EPI_ISL_425565      ------------------------------------------------------------
EPI_ISL_425575      ------------------------------------------------------------
EPI_ISL_426822      ------------------------------------------------------------
EPI_ISL_426833      ------------------------------------------------------------
EPI_ISL_426885      ------------------------------------------------------------
EPI_ISL_426886      ------------------------------------------------------------
EPI_ISL_426889      ------------------------------------------------------------
EPI_ISL_426891      ------------------------------------------------------------
EPI_ISL_426892      ------------------------------------------------------------
EPI_ISL_426894      ------------------------------------------------------------
EPI_ISL_426896      ------------------------------------------------------------
EPI_ISL_426897      ------------------------------------------------------------
EPI_ISL_426899      ------------------------------------------------------------
```

EPI_ISL_426906 --

```
ORF1ab          4321 FGGASCCLYCRCHIDHPNPKGFCDLKGKYVQIPTTCANDPVGFTLKNTVCTVCGMWKGYG
EPI_ISL_413571       ------------------------------------------------------------
EPI_ISL_413574       ------------------------------------------------------------
EPI_ISL_413579       ------------------------------------------------------------
EPI_ISL_413584       ------------------------------------------------------------
EPI_ISL_413587       ------------------------------------------------------------
EPI_ISL_424257       ------------------------------------------------------------
EPI_ISL_425565       ------------------------------------------------------------
EPI_ISL_425575       ------------------------------------------------------------
EPI_ISL_426822       ------------------------------------------------------------
EPI_ISL_426833       ------------------------------------------------------------
EPI_ISL_426885       ------------------------------------------------------------
EPI_ISL_426886       ------------------------------------------------------------
EPI_ISL_426889       ------------------------------------------------------------
EPI_ISL_426891       ------------------------------------------------------------
EPI_ISL_426892       ------------------------------------------------------------
EPI_ISL_426894       ------------------------------------------------------------
EPI_ISL_426896       ------------------------------------------------------------
EPI_ISL_426897       ------------------------------------------------------------
EPI_ISL_426899       ------------------------------------------------------------
EPI_ISL_426906       ------------------------------------------------------------

ORF1ab          4381 CSCDQLREPMLQSADAQSFLNRVCGVSAARLTPCGTGTSTDVVYRAFDIYNDKVAGFAKF
EPI_ISL_413571       ------------------------------------------------------------
EPI_ISL_413574       ------------------------------------------------------------
EPI_ISL_413579       ------------------------------------------------------------
EPI_ISL_413584       ------------------------------------------------------------
EPI_ISL_413587       ------------------------------------------------------------
EPI_ISL_424257       ------------------------------------------------------------
EPI_ISL_425565       ------------------------------------------------------------
EPI_ISL_425575       ------------------------------------------------------------
EPI_ISL_426822       ------------------------------------------------------------
EPI_ISL_426833       ------------------------------------------------------------
EPI_ISL_426885       ------------------------------------------------------------
EPI_ISL_426886       ------------------------------------------------------------
EPI_ISL_426889       ------------------------------------------------------------
EPI_ISL_426891       ------------------------------------------------------------
EPI_ISL_426892       ------------------------------------------------------------
EPI_ISL_426894       ------------------------------------------------------------
EPI_ISL_426896       ------------------------------------------------------------
EPI_ISL_426897       ------------------------------------------------------------
EPI_ISL_426899       ------------------------------------------------------------
EPI_ISL_426906       ------------------------------------------------------------

ORF1ab          4441 LKTNCCRFQEKDEDDNLIDSYFVVKRHTFSNYQHEETIYNLLKDCPAVAKHDFFKFRIDG
EPI_ISL_413571       ------------------------------------------------------------
EPI_ISL_413574       ------------------------------------------------------------
EPI_ISL_413579       ------------------------------------------------------------
EPI_ISL_413584       ------------------------------------------------------------
EPI_ISL_413587       ------------------------------------------------------------
EPI_ISL_424257       ------------------------------------------------------------
EPI_ISL_425565       ------------------------------------------------------------
EPI_ISL_425575       ------------------------------------------------------------
EPI_ISL_426822       ------------------------------------------------------------
EPI_ISL_426833       ------------------------------------------------------------
EPI_ISL_426885       ------------------------------------------------------------
EPI_ISL_426886       ------------------------------------------------------------
EPI_ISL_426889       ------------------------------------------------------------
EPI_ISL_426891       ------------------------------------------------------------
EPI_ISL_426892       ------------------------------------------------------------
EPI_ISL_426894       ------------------------------------------------------------
EPI_ISL_426896       ------------------------------------------------------------
EPI_ISL_426897       ------------------------------------------------------------
EPI_ISL_426899       ------------------------------------------------------------
```

EPI_ISL_426906 --

```
ORF1ab         4501 DMVPHISRQRLTKYTMADLVYALRHFDEGNCDTLKEILVTYNCCDDDYFNKKDWYDFVEN
EPI_ISL_413571      ------------------------------------------------------------
EPI_ISL_413574      ------------------------------------------------------------
EPI_ISL_413579      ------------------------------------------------------------
EPI_ISL_413584      ------------------------------------------------------------
EPI_ISL_413587      ------------------------------------------------------------
EPI_ISL_424257      ------------------------------------------------------------
EPI_ISL_425565      ------------------------------------------------------------
EPI_ISL_425575      ------------------------------------------------------------
EPI_ISL_426822      ------------------------------------------------------------
EPI_ISL_426833      ------------------------------------------------------------
EPI_ISL_426885      ------------------------------------------------------------
EPI_ISL_426886      ------------------------------------------------------------
EPI_ISL_426889      ------------------------------------------------------------
EPI_ISL_426891      ------------------------------------------------------------
EPI_ISL_426892      ------------------------------------------------------------
EPI_ISL_426894      ------------------------------------------------------------
EPI_ISL_426896      ------------------------------------------------------------
EPI_ISL_426897      ------------------------------------------------------------
EPI_ISL_426899      ------------------------------------------------------------
EPI_ISL_426906      ------------------------------------------------------------

ORF1ab         4561 PDILRVYANLGERVRQALLKTVQFCDAMRNAGIVGVLTLDNQDLNGNWYDFGDFIQTTPG
EPI_ISL_413571      ------------------------------------------------------------
EPI_ISL_413574      ------------------------------------------------------------
EPI_ISL_413579      ------------------------------------------------------------
EPI_ISL_413584      ------------------------------------------------------------
EPI_ISL_413587      ------------------------------------------------------------
EPI_ISL_424257      ------------------------------------------------------------
EPI_ISL_425565      ------------------------------------------------------------
EPI_ISL_425575      ------------------------------------------------------------
EPI_ISL_426822      ------------------------------------------------------------
EPI_ISL_426833      ------------------------------------------------------------
EPI_ISL_426885      ------------------------------------------------------------
EPI_ISL_426886      ------------------------------------------------------------
EPI_ISL_426889      ------------------------------------------------------------
EPI_ISL_426891      ------------------------------------------------------------
EPI_ISL_426892      ------------------------------------------------------------
EPI_ISL_426894      ------------------------------------------------------------
EPI_ISL_426896      ------------------------------------------------------------
EPI_ISL_426897      ------------------------------------------------------------
EPI_ISL_426899      ------------------------------------------------------------
EPI_ISL_426906      ------------------------------------------------------------

ORF1ab         4621 SGVPVVDSYYSLLMPILTLTRALTAESHVDTDLTKPYIKWDLLKYDFTEERLKLFDRYFK
EPI_ISL_413571      ------------------------------------------------------------
EPI_ISL_413574      ------------------------------------------------------------
EPI_ISL_413579      ------------------------------------------------------------
EPI_ISL_413584      ------------------------------------------------------------
EPI_ISL_413587      ------------------------------------------------------------
EPI_ISL_424257      ------------------------------------------------------------
EPI_ISL_425565      ------------------------------------------------------------
EPI_ISL_425575      ------------------------------------------------------------
EPI_ISL_426822      ------------------------------------------------------------
EPI_ISL_426833      ------------------------------------------------------------
EPI_ISL_426885      ------------------------------------------------------------
EPI_ISL_426886      ------------------------------------------------------------
EPI_ISL_426889      ------------------------------------------------------------
EPI_ISL_426891      ------------------------------------------------------------
EPI_ISL_426892      ------------------------------------------------------------
EPI_ISL_426894      ------------------------------------------------------------
EPI_ISL_426896      ------------------------------------------------------------
EPI_ISL_426897      ------------------------------------------------------------
EPI_ISL_426899      ------------------------------------------------------------
```

EPI_ISL_426906 --

```
ORF1ab          4681 YWDQTYHPNCVNCLDDRCILHCANFNVLFSTVFPPTSFGPLVRKIFVDGVPFVVSTGYHF
EPI_ISL_413571       ------------------------------------------------------------
EPI_ISL_413574       ------------------------------------------------------------
EPI_ISL_413579       ------------------------------------------------------------
EPI_ISL_413584       ------------------------------------------------------------
EPI_ISL_413587       ------------------------------------------------------------
EPI_ISL_424257       ------------------------------------------------------------
EPI_ISL_425565       ------------------------------------------------------------
EPI_ISL_425575       ------------------------------------------------------------
EPI_ISL_426822       ------------------------------------------------------------
EPI_ISL_426833       ------------------------------------------------------------
EPI_ISL_426885       ------------------------------------------------------------
EPI_ISL_426886       ------------------------------------------------------------
EPI_ISL_426889       ------------------------------------------------------------
EPI_ISL_426891       ------------------------------------------------------------
EPI_ISL_426892       ------------------------------------------------------------
EPI_ISL_426894       ------------------------------------------------------------
EPI_ISL_426896       ------------------------------------------------------------
EPI_ISL_426897       ------------------------------------------------------------
EPI_ISL_426899       ------------------------------------------------------------
EPI_ISL_426906       ------------------------------------------------------------

ORF1ab          4741 RELGVVHNQDVNLHSSRLSFKELLVYAADPAMHAASGNLLLDKRTTCFSVAALTNNVAFQ
EPI_ISL_413571       ------------------------------------------------------------
EPI_ISL_413574       ------------------------------------------------------------
EPI_ISL_413579       ------------------------------------------------------------
EPI_ISL_413584       ------------------------------------------------------------
EPI_ISL_413587       ------------------------------------------------------------
EPI_ISL_424257       ------------------------------------------------------------
EPI_ISL_425565       ------------------------------------------------------------
EPI_ISL_425575       ------------------------------------------------------------
EPI_ISL_426822       ------------------------------------------------------------
EPI_ISL_426833       ------------------------------------------------------------
EPI_ISL_426885       ------------------------------------------------------------
EPI_ISL_426886       ------------------------------------------------------------
EPI_ISL_426889       ------------------------------------------------------------
EPI_ISL_426891       ------------------------------------------------------------
EPI_ISL_426892       ------------------------------------------------------------
EPI_ISL_426894       ------------------------------------------------------------
EPI_ISL_426896       ------------------------------------------------------------
EPI_ISL_426897       ------------------------------------------------------------
EPI_ISL_426899       ------------------------------------------------------------
EPI_ISL_426906       ------------------------------------------------------------

ORF1ab          4801 TVKPGNFNKDFYDFAVSKGFFKEGSSVELKHFFFAQDGNAAISDYDYYRYNLPTMCDIRQ
EPI_ISL_413571       ------------------------------------------------------------
EPI_ISL_413574       ------------------------------------------------------------
EPI_ISL_413579       ------------------------------------------------------------
EPI_ISL_413584       ------------------------------------------------------------
EPI_ISL_413587       ------------------------------------------------------------
EPI_ISL_424257       ------------------------------------------------------------
EPI_ISL_425565       ------------------------------------------------------------
EPI_ISL_425575       ------------------------------------------------------------
EPI_ISL_426822       ------------------------------------------------------------
EPI_ISL_426833       ------------------------------------------------------------
EPI_ISL_426885       ------------------------------------------------------------
EPI_ISL_426886       ------------------------------------------------------------
EPI_ISL_426889       ------------------------------------------------------------
EPI_ISL_426891       ------------------------------------------------------------
EPI_ISL_426892       ------------------------------------------------------------
EPI_ISL_426894       ------------------------------------------------------------
EPI_ISL_426896       ------------------------------------------------------------
EPI_ISL_426897       ------------------------------------------------------------
EPI_ISL_426899       ------------------------------------------------------------
```

EPI_ISL_426906 --

```
ORF1ab         4861 LLFVVEVVDKYFDCYDGGCINANQVIVNNLDKSAGFPFNKWGKARLYYDSMSYEDQDALF
EPI_ISL_413571      ------------------------------------------------------------
EPI_ISL_413574      ------------------------------------------------------------
EPI_ISL_413579      ------------------------------------------------------------
EPI_ISL_413584      ------------------------------------------------------------
EPI_ISL_413587      ------------------------------------------------------------
EPI_ISL_424257      ------------------------------------------------------------
EPI_ISL_425565      ------------------------------------------------------------
EPI_ISL_425575      ------------------------------------------------------------
EPI_ISL_426822      ------------------------------------------------------------
EPI_ISL_426833      ------------------------------------------------------------
EPI_ISL_426885      ------------------------------------------------------------
EPI_ISL_426886      ------------------------------------------------------------
EPI_ISL_426889      ------------------------------------------------------------
EPI_ISL_426891      ------------------------------------------------------------
EPI_ISL_426892      ------------------------------------------------------------
EPI_ISL_426894      ------------------------------------------------------------
EPI_ISL_426896      ------------------------------------------------------------
EPI_ISL_426897      ------------------------------------------------------------
EPI_ISL_426899      ------------------------------------------------------------
EPI_ISL_426906      ------------------------------------------------------------

ORF1ab         4921 AYTKRNVIPTITQMNLKYAISAKNRARTVAGVSICSTMTNRQFHQKLLKSIAATRGATVV
EPI_ISL_413571      ------------------------------------------------------------
EPI_ISL_413574      ------------------------------------------------------------
EPI_ISL_413579      ------------------------------------------------------------
EPI_ISL_413584      ------------------------------------------------------------
EPI_ISL_413587      ------------------------------------------------------------
EPI_ISL_424257      ------------------------------------------------------------
EPI_ISL_425565      ------------------------------------------------------------
EPI_ISL_425575      ------------------------------------------------------------
EPI_ISL_426822      ------------------------------------------------------------
EPI_ISL_426833      ------------------------------------------------------------
EPI_ISL_426885      ------------------------------------------------------------
EPI_ISL_426886      ------------------------------------------------------------
EPI_ISL_426889      ------------------------------------------------------------
EPI_ISL_426891      ------------------------------------------------------------
EPI_ISL_426892      ------------------------------------------------------------
EPI_ISL_426894      ------------------------------------------------------------
EPI_ISL_426896      ------------------------------------------------------------
EPI_ISL_426897      ------------------------------------------------------------
EPI_ISL_426899      ------------------------------------------------------------
EPI_ISL_426906      ------------------------------------------------------------

ORF1ab         4981 IGTSKFYGGWHNMLKTVYSDVENPHLMGWDYPKCDRAMPNMLRIMASLVLARKHTTCCSL
EPI_ISL_413571      ------------------------------------------------------------
EPI_ISL_413574      ------------------------------------------------------------
EPI_ISL_413579      ------------------------------------------------------------
EPI_ISL_413584      ------------------------------------------------------------
EPI_ISL_413587      ------------------------------------------------------------
EPI_ISL_424257      ------------------------------------------------------------
EPI_ISL_425565      ------------------------------------------------------------
EPI_ISL_425575      ------------------------------------------------------------
EPI_ISL_426822      ------------------------------------------------------------
EPI_ISL_426833      ------------------------------------------------------------
EPI_ISL_426885      ------------------------------------------------------------
EPI_ISL_426886      ------------------------------------------------------------
EPI_ISL_426889      ------------------------------------------------------------
EPI_ISL_426891      ------------------------------------------------------------
EPI_ISL_426892      ------------------------------------------------------------
EPI_ISL_426894      ------------------------------------------------------------
EPI_ISL_426896      ------------------------------------------------------------
EPI_ISL_426897      ------------------------------------------------------------
EPI_ISL_426899      ------------------------------------------------------------
```

EPI_ISL_426906 --

```
ORF1ab          5041 SHRFYRLANECAQVLSEMVMCGGSLYVKPGGTSSGDATTAYANSVFNICQAVTANVNALL
EPI_ISL_413571       ------------------------------------------------------------
EPI_ISL_413574       ------------------------------------------------------------
EPI_ISL_413579       ------------------------------------------------------------
EPI_ISL_413584       ------------------------------------------------------------
EPI_ISL_413587       ------------------------------------------------------------
EPI_ISL_424257       ------------------------------------------------------------
EPI_ISL_425565       ------------------------------------------------------------
EPI_ISL_425575       ------------------------------------------------------------
EPI_ISL_426822       ------------------------------------------------------------
EPI_ISL_426833       ------------------------------------------------------------
EPI_ISL_426885       ------------------------------------------------------------
EPI_ISL_426886       ------------------------------------------------------------
EPI_ISL_426889       ------------------------------------------------------------
EPI_ISL_426891       ------------------------------------------------------------
EPI_ISL_426892       ------------------------------------------------------------
EPI_ISL_426894       ------------------------------------------------------------
EPI_ISL_426896       ------------------------------------------------------------
EPI_ISL_426897       ------------------------------------------------------------
EPI_ISL_426899       ------------------------------------------------------------
EPI_ISL_426906       ------------------------------------------------------------

ORF1ab          5101 STDGNKIADKYVRNLQHRLYECLYRNRDVDTDFVNEFYAYLRKHFSMMILSDDAVVCFNS
EPI_ISL_413571       ------------------------------------------------------------
EPI_ISL_413574       ------------------------------------------------------------
EPI_ISL_413579       ------------------------------------------------------------
EPI_ISL_413584       ------------------------------------------------------------
EPI_ISL_413587       ------------------------------------------------------------
EPI_ISL_424257       ------------------------------------------------------------
EPI_ISL_425565       ------------------------------------------------------------
EPI_ISL_425575       ------------------------------------------------------------
EPI_ISL_426822       ------------------------------------------------------------
EPI_ISL_426833       ------------------------------------------------------------
EPI_ISL_426885       ------------------------------------------------------------
EPI_ISL_426886       ------------------------------------------------------------
EPI_ISL_426889       ------------------------------------------------------------
EPI_ISL_426891       ------------------------------------------------------------
EPI_ISL_426892       ------------------------------------------------------------
EPI_ISL_426894       ------------------------------------------------------------
EPI_ISL_426896       ------------------------------------------------------------
EPI_ISL_426897       ------------------------------------------------------------
EPI_ISL_426899       ------------------------------------------------------------
EPI_ISL_426906       ------------------------------------------------------------

ORF1ab          5161 TYASQGLVASIKNFKSVLYYQNNVFMSEAKCWTETDLTKGPHEFCSQHTMLVKQGDDYVY
EPI_ISL_413571       ------------------------------------------------------------
EPI_ISL_413574       ------------------------------------------------------------
EPI_ISL_413579       ------------------------------------------------------------
EPI_ISL_413584       ------------------------------------------------------------
EPI_ISL_413587       ------------------------------------------------------------
EPI_ISL_424257       ------------------------------------------------------------
EPI_ISL_425565       ------------------------------------------------------------
EPI_ISL_425575       ------------------------------------------------------------
EPI_ISL_426822       ------------------------------------------------------------
EPI_ISL_426833       ------------------------------------------------------------
EPI_ISL_426885       ------------------------------------------------------------
EPI_ISL_426886       ------------------------------------------------------------
EPI_ISL_426889       ------------------------------------------------------------
EPI_ISL_426891       ------------------------------------------------------------
EPI_ISL_426892       ------------------------------------------------------------
EPI_ISL_426894       ------------------------------------------------------------
EPI_ISL_426896       ------------------------------------------------------------
EPI_ISL_426897       ------------------------------------------------------------
EPI_ISL_426899       ------------------------------------------------------------
```

EPI_ISL_426906 --

```
ORF1ab          5221 LPYPDPSRILGAGCFVDDIVKTDGTLMIERFVSLAIDAYPLTKHPNQEYADVFHLYLQYI
EPI_ISL_413571       ------------------------------------------------------------
EPI_ISL_413574       ------------------------------------------------------------
EPI_ISL_413579       ------------------------------------------------------------
EPI_ISL_413584       ------------------------------------------------------------
EPI_ISL_413587       ------------------------------------------------------------
EPI_ISL_424257       ------------------------------------------------------------
EPI_ISL_425565       ------------------------------------------------------------
EPI_ISL_425575       ------------------------------------------------------------
EPI_ISL_426822       ------------------------------------------------------------
EPI_ISL_426833       ------------------------------------------------------------
EPI_ISL_426885       ------------------------------------------------------------
EPI_ISL_426886       ------------------------------------------------------------
EPI_ISL_426889       ------------------------------------------------------------
EPI_ISL_426891       ------------------------------------------------------------
EPI_ISL_426892       ------------------------------------------------------------
EPI_ISL_426894       ------------------------------------------------------------
EPI_ISL_426896       ------------------------------------------------------------
EPI_ISL_426897       ------------------------------------------------------------
EPI_ISL_426899       ------------------------------------------------------------
EPI_ISL_426906       ------------------------------------------------------------

ORF1ab          5281 RKLHDELTGHMLDMYSVMLTNDNTSRYWEPEFYEAMYTPHTVLQAVGACVLCNSQTSLRC
EPI_ISL_413571       ------------------------------------------------------------
EPI_ISL_413574       ------------------------------------------------------------
EPI_ISL_413579       ------------------------------------------------------------
EPI_ISL_413584       ------------------------------------------------------------
EPI_ISL_413587       ------------------------------------------------------------
EPI_ISL_424257       ------------------------------------------------------------
EPI_ISL_425565       ------------------------------------------------------------
EPI_ISL_425575       ------------------------------------------------------------
EPI_ISL_426822       ------------------------------------------------------------
EPI_ISL_426833       ------------------------------------------------------------
EPI_ISL_426885       ------------------------------------------------------------
EPI_ISL_426886       ------------------------------------------------------------
EPI_ISL_426889       ------------------------------------------------------------
EPI_ISL_426891       ------------------------------------------------------------
EPI_ISL_426892       ------------------------------------------------------------
EPI_ISL_426894       ------------------------------------------------------------
EPI_ISL_426896       ------------------------------------------------------------
EPI_ISL_426897       ------------------------------------------------------------
EPI_ISL_426899       ------------------------------------------------------------
EPI_ISL_426906       ------------------------------------------------------------

ORF1ab          5341 GACIRRPFLCCKCCYDHVISTSHKLVLSVNPYVCNAPGCDVTDVTQLYLGGMSYYCKSHK
EPI_ISL_413571       ------------------------------------------------------------
EPI_ISL_413574       ------------------------------------------------------------
EPI_ISL_413579       ------------------------------------------------------------
EPI_ISL_413584       ------------------------------------------------------------
EPI_ISL_413587       ------------------------------------------------------------
EPI_ISL_424257       ------------------------------------------------------------
EPI_ISL_425565       ------------------------------------------------------------
EPI_ISL_425575       ------------------------------------------------------------
EPI_ISL_426822       ------------------------------------------------------------
EPI_ISL_426833       ------------------------------------------------------------
EPI_ISL_426885       ------------------------------------------------------------
EPI_ISL_426886       ------------------------------------------------------------
EPI_ISL_426889       ------------------------------------------------------------
EPI_ISL_426891       ------------------------------------------------------------
EPI_ISL_426892       ------------------------------------------------------------
EPI_ISL_426894       ------------------------------------------------------------
EPI_ISL_426896       ------------------------------------------------------------
EPI_ISL_426897       ------------------------------------------------------------
EPI_ISL_426899       ------------------------------------------------------------
```

EPI_ISL_426906 --

```
ORF1ab         5401 PPISFPLCANGQVFGLYKNTCVGSDNVTDFNAIATCDWTNAGDYILANTCTERLKLFAAE
EPI_ISL_413571      ------------------------------------------------------------
EPI_ISL_413574      ------------------------------------------------------------
EPI_ISL_413579      ------------------------------------------------------------
EPI_ISL_413584      ------------------------------------------------------------
EPI_ISL_413587      ------------------------------------------------------------
EPI_ISL_424257      ------------------------------------------------------------
EPI_ISL_425565      ------------------------------------------------------------
EPI_ISL_425575      ------------------------------------------------------------
EPI_ISL_426822      ------------------------------------------------------------
EPI_ISL_426833      ------------------------------------------------------------
EPI_ISL_426885      ------------------------------------------------------------
EPI_ISL_426886      ------------------------------------------------------------
EPI_ISL_426889      ------------------------------------------------------------
EPI_ISL_426891      ------------------------------------------------------------
EPI_ISL_426892      ------------------------------------------------------------
EPI_ISL_426894      ------------------------------------------------------------
EPI_ISL_426896      ------------------------------------------------------------
EPI_ISL_426897      ------------------------------------------------------------
EPI_ISL_426899      ------------------------------------------------------------
EPI_ISL_426906      ------------------------------------------------------------

ORF1ab         5461 TLKATEETFKLSYGIATVREVLSDRELHLSWEVGKPRPPLNRNYVFTGYRVTKNSKVQIG
EPI_ISL_413571      ------------------------------------------------------------
EPI_ISL_413574      ------------------------------------------------------------
EPI_ISL_413579      ------------------------------------------------------------
EPI_ISL_413584      ------------------------------------------------------------
EPI_ISL_413587      ------------------------------------------------------------
EPI_ISL_424257      ------------------------------------------------------------
EPI_ISL_425565      ------------------------------------------------------------
EPI_ISL_425575      ------------------------------------------------------------
EPI_ISL_426822      ------------------------------------------------------------
EPI_ISL_426833      ------------------------------------------------------------
EPI_ISL_426885      ------------------------------------------------------------
EPI_ISL_426886      ------------------------------------------------------------
EPI_ISL_426889      ------------------------------------------------------------
EPI_ISL_426891      ------------------------------------------------------------
EPI_ISL_426892      ------------------------------------------------------------
EPI_ISL_426894      ------------------------------------------------------------
EPI_ISL_426896      ------------------------------------------------------------
EPI_ISL_426897      ------------------------------------------------------------
EPI_ISL_426899      ------------------------------------------------------------
EPI_ISL_426906      ------------------------------------------------------------

ORF1ab         5521 EYTFEKGDYGDAVVYRGTTTYKLNVGDYFVLTSHTVMPLSAPTLVPQEHYVRITGLYPTL
EPI_ISL_413571      ------------------------------------------------------------
EPI_ISL_413574      ------------------------------------------------------------
EPI_ISL_413579      ------------------------------------------------------------
EPI_ISL_413584      ------------------------------------------------------------
EPI_ISL_413587      ------------------------------------------------------------
EPI_ISL_424257      ------------------------------------------------------------
EPI_ISL_425565      ------------------------------------------------------------
EPI_ISL_425575      ------------------------------------------------------------
EPI_ISL_426822      ------------------------------------------------------------
EPI_ISL_426833      ------------------------------------------------------------
EPI_ISL_426885      ------------------------------------------------------------
EPI_ISL_426886      ------------------------------------------------------------
EPI_ISL_426889      ------------------------------------------------------------
EPI_ISL_426891      ------------------------------------------------------------
EPI_ISL_426892      ------------------------------------------------------------
EPI_ISL_426894      ------------------------------------------------------------
EPI_ISL_426896      ------------------------------------------------------------
EPI_ISL_426897      ------------------------------------------------------------
EPI_ISL_426899      ------------------------------------------------------------
```

EPI_ISL_426906 --

```
ORF1ab          5581 NISDEFSSNVANYQKVGMQKYSTLQGPPGTGKSHFAIGLALYYPSARIVYTACSHAAVDA
EPI_ISL_413571       ------------------------------------------------------------
EPI_ISL_413574       ------------------------------------------------------------
EPI_ISL_413579       ------------------------------------------------------------
EPI_ISL_413584       ------------------------------------------------------------
EPI_ISL_413587       ------------------------------------------------------------
EPI_ISL_424257       ------------------------------------------------------------
EPI_ISL_425565       ------------------------------------------------------------
EPI_ISL_425575       ------------------------------------------------------------
EPI_ISL_426822       ------------------------------------------------------------
EPI_ISL_426833       ------------------------------------------------------------
EPI_ISL_426885       ------------------------------------------------------------
EPI_ISL_426886       ------------------------------------------------------------
EPI_ISL_426889       ------------------------------------------------------------
EPI_ISL_426891       ------------------------------------------------------------
EPI_ISL_426892       ------------------------------------------------------------
EPI_ISL_426894       ------------------------------------------------------------
EPI_ISL_426896       ------------------------------------------------------------
EPI_ISL_426897       ------------------------------------------------------------
EPI_ISL_426899       ------------------------------------------------------------
EPI_ISL_426906       ------------------------------------------------------------

ORF1ab          5641 LCEKALKYLPIDKCSRIIPARARVECFDKFKVNSTLEQYVFCTVNALPETTADIVVFDEI
EPI_ISL_413571       ------------------------------------------------------------
EPI_ISL_413574       ------------------------------------------------------------
EPI_ISL_413579       ------------------------------------------------------------
EPI_ISL_413584       ------------------------------------------------------------
EPI_ISL_413587       ------------------------------------------------------------
EPI_ISL_424257       ------------------------------------------------------------
EPI_ISL_425565       ------------------------------------------------------------
EPI_ISL_425575       ------------------------------------------------------------
EPI_ISL_426822       ------------------------------------------------------------
EPI_ISL_426833       ------------------------------------------------------------
EPI_ISL_426885       ------------------------------------------------------------
EPI_ISL_426886       ------------------------------------------------------------
EPI_ISL_426889       ------------------------------------------------------------
EPI_ISL_426891       ------------------------------------------------------------
EPI_ISL_426892       ------------------------------------------------------------
EPI_ISL_426894       ------------------------------------------------------------
EPI_ISL_426896       ------------------------------------------------------------
EPI_ISL_426897       ------------------------------------------------------------
EPI_ISL_426899       ------------------------------------------------------------
EPI_ISL_426906       ------------------------------------------------------------

ORF1ab          5701 SMATNYDLSVVNARLRAKHYVYIGDPAQLPAPRTLLTKGTLEPEYFNSVCRLMKTIGPDM
EPI_ISL_413571       ------------------------------------------------------------
EPI_ISL_413574       ------------------------------------------------------------
EPI_ISL_413579       ------------------------------------------------------------
EPI_ISL_413584       ------------------------------------------------------------
EPI_ISL_413587       ------------------------------------------------------------
EPI_ISL_424257       ------------------------------------------------------------
EPI_ISL_425565       ------------------------------------------------------------
EPI_ISL_425575       ------------------------------------------------------------
EPI_ISL_426822       ------------------------------------------------------------
EPI_ISL_426833       ------------------------------------------------------------
EPI_ISL_426885       ------------------------------------------------------------
EPI_ISL_426886       ------------------------------------------------------------
EPI_ISL_426889       ------------------------------------------------------------
EPI_ISL_426891       ------------------------------------------------------------
EPI_ISL_426892       ------------------------------------------------------------
EPI_ISL_426894       ------------------------------------------------------------
EPI_ISL_426896       ------------------------------------------------------------
EPI_ISL_426897       ------------------------------------------------------------
EPI_ISL_426899       ------------------------------------------------------------
```

EPI_ISL_426906 --

```
ORF1ab         5761 FLGTCRRCPAEIVDTVSALVYDNKLKAHKDKSAQCFKMFYKGVITHDVSSAINRPQIGVV
EPI_ISL_413571      ------------------------------------------------------------
EPI_ISL_413574      ------------------------------------------------------------
EPI_ISL_413579      ------------------------------------------------------------
EPI_ISL_413584      ------------------------------------------------------------
EPI_ISL_413587      ------------------------------------------------------------
EPI_ISL_424257      ------------------------------------------------------------
EPI_ISL_425565      ------------------------------------------------------------
EPI_ISL_425575      ------------------------------------------------------------
EPI_ISL_426822      ------------------------------------------------------------
EPI_ISL_426833      ------------------------------------------------------------
EPI_ISL_426885      ------------------------------------------------------------
EPI_ISL_426886      ------------------------------------------------------------
EPI_ISL_426889      ------------------------------------------------------------
EPI_ISL_426891      ------------------------------------------------------------
EPI_ISL_426892      ------------------------------------------------------------
EPI_ISL_426894      ------------------------------------------------------------
EPI_ISL_426896      ------------------------------------------------------------
EPI_ISL_426897      ------------------------------------------------------------
EPI_ISL_426899      ------------------------------------------------------------
EPI_ISL_426906      ------------------------------------------------------------

ORF1ab         5821 REFLTRNPAWRKAVFISPYNSQNAVASKILGLPTQTVDSSQGSEYDYVIFTQTTETAHSC
EPI_ISL_413571      ------------------------------------------------------------
EPI_ISL_413574      ------------------------------------------------------------
EPI_ISL_413579      ------------------------------------------------------------
EPI_ISL_413584      ------------------------------------------------------------
EPI_ISL_413587      ------------------------------------------------------------
EPI_ISL_424257      ------------------------------------------------------------
EPI_ISL_425565      ------------------------------------------------------------
EPI_ISL_425575      ------------------------------------------------------------
EPI_ISL_426822      ------------------------------------------------------------
EPI_ISL_426833      ------------------------------------------------------------
EPI_ISL_426885      ------------------------------------------------------------
EPI_ISL_426886      ------------------------------------------------------------
EPI_ISL_426889      ------------------------------------------------------------
EPI_ISL_426891      ------------------------------------------------------------
EPI_ISL_426892      ------------------------------------------------------------
EPI_ISL_426894      ------------------------------------------------------------
EPI_ISL_426896      ------------------------------------------------------------
EPI_ISL_426897      ------------------------------------------------------------
EPI_ISL_426899      ------------------------------------------------------------
EPI_ISL_426906      ------------------------------------------------------------

ORF1ab         5881 NVNRFNVAITRAKVGILCIMSDRDLYDKLQFTSLEIPRRNVATLQAENVTGLFKDCSKVI
EPI_ISL_413571      ------------------------------------------------------------
EPI_ISL_413574      ------------------------------------------------------------
EPI_ISL_413579      ------------------------------------------------------------
EPI_ISL_413584      ------------------------------------------------------------
EPI_ISL_413587      ------------------------------------------------------------
EPI_ISL_424257      ------------------------------------------------------------
EPI_ISL_425565      ------------------------------------------------------------
EPI_ISL_425575      ------------------------------------------------------------
EPI_ISL_426822      ------------------------------------------------------------
EPI_ISL_426833      ------------------------------------------------------------
EPI_ISL_426885      ------------------------------------------------------------
EPI_ISL_426886      ------------------------------------------------------------
EPI_ISL_426889      ------------------------------------------------------------
EPI_ISL_426891      ------------------------------------------------------------
EPI_ISL_426892      ------------------------------------------------------------
EPI_ISL_426894      ------------------------------------------------------------
EPI_ISL_426896      ------------------------------------------------------------
EPI_ISL_426897      ------------------------------------------------------------
EPI_ISL_426899      ------------------------------------------------------------
```

EPI_ISL_426906 --

```
ORF1ab         5941 TGLHPTQAPTHLSVDTKFKTEGLCVDIPGIPKDMTYRRLISMMGFKMNYQVNGYPNMFIT
EPI_ISL_413571      ------------------------------------------------------------
EPI_ISL_413574      ------------------------------------------------------------
EPI_ISL_413579      ------------------------------------------------------------
EPI_ISL_413584      ------------------------------------------------------------
EPI_ISL_413587      ------------------------------------------------------------
EPI_ISL_424257      ------------------------------------------------------------
EPI_ISL_425565      ------------------------------------------------------------
EPI_ISL_425575      ------------------------------------------------------------
EPI_ISL_426822      ------------------------------------------------------------
EPI_ISL_426833      ------------------------------------------------------------
EPI_ISL_426885      ------------------------------------------------------------
EPI_ISL_426886      ------------------------------------------------------------
EPI_ISL_426889      ------------------------------------------------------------
EPI_ISL_426891      ------------------------------------------------------------
EPI_ISL_426892      ------------------------------------------------------------
EPI_ISL_426894      ------------------------------------------------------------
EPI_ISL_426896      ------------------------------------------------------------
EPI_ISL_426897      ------------------------------------------------------------
EPI_ISL_426899      ------------------------------------------------------------
EPI_ISL_426906      ------------------------------------------------------------

ORF1ab         6001 REEAIRHVRAWIGFDVEGCHATREAVGTNLPLQLGFSTGVNLVAVPTGYVDTPNNTDFSR
EPI_ISL_413571      ------------------------------------------------------------
EPI_ISL_413574      ------------------------------------------------------------
EPI_ISL_413579      ------------------------------------------------------------
EPI_ISL_413584      ------------------------------------------------------------
EPI_ISL_413587      ------------------------------------------------------------
EPI_ISL_424257      ------------------------------------------------------------
EPI_ISL_425565      ------------------------------------------------------------
EPI_ISL_425575      ------------------------------------------------------------
EPI_ISL_426822      ------------------------------------------------------------
EPI_ISL_426833      ------------------------------------------------------------
EPI_ISL_426885      ------------------------------------------------------------
EPI_ISL_426886      ------------------------------------------------------------
EPI_ISL_426889      ------------------------------------------------------------
EPI_ISL_426891      ------------------------------------------------------------
EPI_ISL_426892      ------------------------------------------------------------
EPI_ISL_426894      ------------------------------------------------------------
EPI_ISL_426896      ------------------------------------------------------------
EPI_ISL_426897      ------------------------------------------------------------
EPI_ISL_426899      ------------------------------------------------------------
EPI_ISL_426906      ------------------------------------------------------------

ORF1ab         6061 VSAKPPPGDQFKHLIPLMYKGLPWNVVRIKIVQMLSDTLKNLSDRVVFVLWAHGFELTSM
EPI_ISL_413571      ------------------------------------------------------------
EPI_ISL_413574      ------------------------------------------------------------
EPI_ISL_413579      ------------------------------------------------------------
EPI_ISL_413584      ------------------------------------------------------------
EPI_ISL_413587      ------------------------------------------------------------
EPI_ISL_424257      ------------------------------------------------------------
EPI_ISL_425565      ------------------------------------------------------------
EPI_ISL_425575      ------------------------------------------------------------
EPI_ISL_426822      ------------------------------------------------------------
EPI_ISL_426833      ------------------------------------------------------------
EPI_ISL_426885      ------------------------------------------------------------
EPI_ISL_426886      ------------------------------------------------------------
EPI_ISL_426889      ------------------------------------------------------------
EPI_ISL_426891      ------------------------------------------------------------
EPI_ISL_426892      ------------------------------------------------------------
EPI_ISL_426894      ------------------------------------------------------------
EPI_ISL_426896      ------------------------------------------------------------
EPI_ISL_426897      ------------------------------------------------------------
EPI_ISL_426899      ------------------------------------------------------------
```

EPI_ISL_426906   --

```
ORF1ab          6121 KYFVKIGPERTCCLCDRRATCFSTASDTYACWHHSIGFDYVYNPFMIDVQQWGFTGNLQS
EPI_ISL_413571       ------------------------------------------------------------
EPI_ISL_413574       ------------------------------------------------------------
EPI_ISL_413579       ------------------------------------------------------------
EPI_ISL_413584       ------------------------------------------------------------
EPI_ISL_413587       ------------------------------------------------------------
EPI_ISL_424257       ------------------------------------------------------------
EPI_ISL_425565       ------------------------------------------------------------
EPI_ISL_425575       ------------------------------------------------------------
EPI_ISL_426822       ------------------------------------------------------------
EPI_ISL_426833       ------------------------------------------------------------
EPI_ISL_426885       ------------------------------------------------------------
EPI_ISL_426886       ------------------------------------------------------------
EPI_ISL_426889       ------------------------------------------------------------
EPI_ISL_426891       ------------------------------------------------------------
EPI_ISL_426892       ------------------------------------------------------------
EPI_ISL_426894       ------------------------------------------------------------
EPI_ISL_426896       ------------------------------------------------------------
EPI_ISL_426897       ------------------------------------------------------------
EPI_ISL_426899       ------------------------------------------------------------
EPI_ISL_426906       ------------------------------------------------------------

ORF1ab          6181 NHDLYCQVHGNAHVASCDAIMTRCLAVHECFVKRVDWTIEYPIIGDELKINAACRKVQHM
EPI_ISL_413571       ------------------------------------------------------------
EPI_ISL_413574       ------------------------------------------------------------
EPI_ISL_413579       ------------------------------------------------------------
EPI_ISL_413584       ------------------------------------------------------------
EPI_ISL_413587       ------------------------------------------------------------
EPI_ISL_424257       ------------------------------------------------------------
EPI_ISL_425565       ------------------------------------------------------------
EPI_ISL_425575       ------------------------------------------------------------
EPI_ISL_426822       ------------------------------------------------------------
EPI_ISL_426833       ------------------------------------------------------------
EPI_ISL_426885       ------------------------------------------------------------
EPI_ISL_426886       ------------------------------------------------------------
EPI_ISL_426889       ------------------------------------------------------------
EPI_ISL_426891       ------------------------------------------------------------
EPI_ISL_426892       ------------------------------------------------------------
EPI_ISL_426894       ------------------------------------------------------------
EPI_ISL_426896       ------------------------------------------------------------
EPI_ISL_426897       ------------------------------------------------------------
EPI_ISL_426899       ------------------------------------------------------------
EPI_ISL_426906       ------------------------------------------------------------

ORF1ab          6241 VVKAALLADKFPVLHDIGNPKAIKCVPQADVEWKFYDAQPCSDKAYKIEELFYSYATHSD
EPI_ISL_413571       ------------------------------------------------------------
EPI_ISL_413574       ------------------------------------------------------------
EPI_ISL_413579       ------------------------------------------------------------
EPI_ISL_413584       ------------------------------------------------------------
EPI_ISL_413587       ------------------------------------------------------------
EPI_ISL_424257       ------------------------------------------------------------
EPI_ISL_425565       ------------------------------------------------------------
EPI_ISL_425575       ------------------------------------------------------------
EPI_ISL_426822       ------------------------------------------------------------
EPI_ISL_426833       ------------------------------------------------------------
EPI_ISL_426885       ------------------------------------------------------------
EPI_ISL_426886       ------------------------------------------------------------
EPI_ISL_426889       ------------------------------------------------------------
EPI_ISL_426891       ------------------------------------------------------------
EPI_ISL_426892       ------------------------------------------------------------
EPI_ISL_426894       ------------------------------------------------------------
EPI_ISL_426896       ------------------------------------------------------------
EPI_ISL_426897       ------------------------------------------------------------
EPI_ISL_426899       ------------------------------------------------------------
```

EPI_ISL_426906 --

```
ORF1ab         6301 KFTDGVCLFWNCNVDRYPANSIVCRFDTRVLSNLNLPGCDGGSLYVNKHAFHTPAFDKSA
EPI_ISL_413571      ------------------------------------------------------------
EPI_ISL_413574      ------------------------------------------------------------
EPI_ISL_413579      ------------------------------------------------------------
EPI_ISL_413584      ------------------------------------------------------------
EPI_ISL_413587      ------------------------------------------------------------
EPI_ISL_424257      ------------------------------------------------------------
EPI_ISL_425565      ------------------------------------------------------------
EPI_ISL_425575      ------------------------------------------------------------
EPI_ISL_426822      ------------------------------------------------------------
EPI_ISL_426833      ------------------------------------------------------------
EPI_ISL_426885      ------------------------------------------------------------
EPI_ISL_426886      ------------------------------------------------------------
EPI_ISL_426889      ------------------------------------------------------------
EPI_ISL_426891      ------------------------------------------------------------
EPI_ISL_426892      ------------------------------------------------------------
EPI_ISL_426894      ------------------------------------------------------------
EPI_ISL_426896      ------------------------------------------------------------
EPI_ISL_426897      ------------------------------------------------------------
EPI_ISL_426899      ------------------------------------------------------------
EPI_ISL_426906      ------------------------------------------------------------

ORF1ab         6361 FVNLKQLPFFYYSDSPCESHGKQVVSDIDYVPLKSATCITRCNLGGAVCRHHANEYRLYL
EPI_ISL_413571      ------------------------------------------------------------
EPI_ISL_413574      ------------------------------------------------------------
EPI_ISL_413579      ------------------------------------------------------------
EPI_ISL_413584      ------------------------------------------------------------
EPI_ISL_413587      ------------------------------------------------------------
EPI_ISL_424257      ------------------------------------------------------------
EPI_ISL_425565      ------------------------------------------------------------
EPI_ISL_425575      ------------------------------------------------------------
EPI_ISL_426822      ------------------------------------------------------------
EPI_ISL_426833      ------------------------------------------------------------
EPI_ISL_426885      ------------------------------------------------------------
EPI_ISL_426886      ------------------------------------------------------------
EPI_ISL_426889      ------------------------------------------------------------
EPI_ISL_426891      ------------------------------------------------------------
EPI_ISL_426892      ------------------------------------------------------------
EPI_ISL_426894      ------------------------------------------------------------
EPI_ISL_426896      ------------------------------------------------------------
EPI_ISL_426897      ------------------------------------------------------------
EPI_ISL_426899      ------------------------------------------------------------
EPI_ISL_426906      ------------------------------------------------------------

ORF1ab         6421 DAYNMMISAGFSLWVYKQFDTYNLWNTFTRLQSLENVAFNVVNKGHFDGQQGEVPVSIIN
EPI_ISL_413571      ------------------------------------------------------------
EPI_ISL_413574      ------------------------------------------------------------
EPI_ISL_413579      ------------------------------------------------------------
EPI_ISL_413584      ------------------------------------------------------------
EPI_ISL_413587      ------------------------------------------------------------
EPI_ISL_424257      ------------------------------------------------------------
EPI_ISL_425565      ------------------------------------------------------------
EPI_ISL_425575      ------------------------------------------------------------
EPI_ISL_426822      ------------------------------------------------------------
EPI_ISL_426833      ------------------------------------------------------------
EPI_ISL_426885      ------------------------------------------------------------
EPI_ISL_426886      ------------------------------------------------------------
EPI_ISL_426889      ------------------------------------------------------------
EPI_ISL_426891      ------------------------------------------------------------
EPI_ISL_426892      ------------------------------------------------------------
EPI_ISL_426894      ------------------------------------------------------------
EPI_ISL_426896      ------------------------------------------------------------
EPI_ISL_426897      ------------------------------------------------------------
EPI_ISL_426899      ------------------------------------------------------------
```

EPI_ISL_426906 --

```
ORF1ab         6481 NTVYTKVDGVDVELFENKTTLPVNVAFELWAKRNIKPVPEVKILNNLGVDIAANTVIWDY
EPI_ISL_413571      ------------------------------------------------------------
EPI_ISL_413574      ------------------------------------------------------------
EPI_ISL_413579      ------------------------------------------------------------
EPI_ISL_413584      ------------------------------------------------------------
EPI_ISL_413587      ------------------------------------------------------------
EPI_ISL_424257      ------------------------------------------------------------
EPI_ISL_425565      ------------------------------------------------------------
EPI_ISL_425575      ------------------------------------------------------------
EPI_ISL_426822      ------------------------------------------------------------
EPI_ISL_426833      ------------------------------------------------------------
EPI_ISL_426885      ------------------------------------------------------------
EPI_ISL_426886      ------------------------------------------------------------
EPI_ISL_426889      ------------------------------------------------------------
EPI_ISL_426891      ------------------------------------------------------------
EPI_ISL_426892      ------------------------------------------------------------
EPI_ISL_426894      ------------------------------------------------------------
EPI_ISL_426896      ------------------------------------------------------------
EPI_ISL_426897      ------------------------------------------------------------
EPI_ISL_426899      ------------------------------------------------------------
EPI_ISL_426906      ------------------------------------------------------------

ORF1ab         6541 KRDAPAHISTIGVCSMTDIAKKPTETICAPLTVFFDGRVDGQVDLFRNARNGVLITEGSV
EPI_ISL_413571      ------------------------------------------------------------
EPI_ISL_413574      ------------------------------------------------------------
EPI_ISL_413579      ------------------------------------------------------------
EPI_ISL_413584      ------------------------------------------------------------
EPI_ISL_413587      ------------------------------------------------------------
EPI_ISL_424257      ------------------------------------------------------------
EPI_ISL_425565      ------------------------------------------------------------
EPI_ISL_425575      ------------------------------------------------------------
EPI_ISL_426822      ------------------------------------------------------------
EPI_ISL_426833      ------------------------------------------------------------
EPI_ISL_426885      ------------------------------------------------------------
EPI_ISL_426886      ------------------------------------------------------------
EPI_ISL_426889      ------------------------------------------------------------
EPI_ISL_426891      ------------------------------------------------------------
EPI_ISL_426892      ------------------------------------------------------------
EPI_ISL_426894      ------------------------------------------------------------
EPI_ISL_426896      ------------------------------------------------------------
EPI_ISL_426897      ------------------------------------------------------------
EPI_ISL_426899      ------------------------------------------------------------
EPI_ISL_426906      ------------------------------------------------------------

ORF1ab         6601 KGLQPSVGPKQASLNGVTLIGEAVKTQFNYYKKVDGVVQQLPETYFTQSRNLQEFKPRSQ
EPI_ISL_413571      ------------------------------------------------------------
EPI_ISL_413574      ------------------------------------------------------------
EPI_ISL_413579      ------------------------------------------------------------
EPI_ISL_413584      ------------------------------------------------------------
EPI_ISL_413587      ------------------------------------------------------------
EPI_ISL_424257      ------------------------------------------------------------
EPI_ISL_425565      ------------------------------------------------------------
EPI_ISL_425575      ------------------------------------------------------------
EPI_ISL_426822      ------------------------------------------------------------
EPI_ISL_426833      ------------------------------------------------------------
EPI_ISL_426885      ------------------------------------------------------------
EPI_ISL_426886      ------------------------------------------------------------
EPI_ISL_426889      ------------------------------------------------------------
EPI_ISL_426891      ------------------------------------------------------------
EPI_ISL_426892      ------------------------------------------------------------
EPI_ISL_426894      ------------------------------------------------------------
EPI_ISL_426896      ------------------------------------------------------------
EPI_ISL_426897      ------------------------------------------------------------
EPI_ISL_426899      ------------------------------------------------------------
```

EPI_ISL_426906 --

```
ORF1ab         6661 MEIDFLELAMDEFIERYKLEGYAFEHIVYGDFSHSQLGGLHLLIGLAKRFKESPFELEDF
EPI_ISL_413571      ------------------------------------------------------------
EPI_ISL_413574      ------------------------------------------------------------
EPI_ISL_413579      ------------------------------------------------------------
EPI_ISL_413584      ------------------------------------------------------------
EPI_ISL_413587      ------------------------------------------------------------
EPI_ISL_424257      ------------------------------------------------------------
EPI_ISL_425565      ------------------------------------------------------------
EPI_ISL_425575      ------------------------------------------------------------
EPI_ISL_426822      ------------------------------------------------------------
EPI_ISL_426833      ------------------------------------------------------------
EPI_ISL_426885      ------------------------------------------------------------
EPI_ISL_426886      ------------------------------------------------------------
EPI_ISL_426889      ------------------------------------------------------------
EPI_ISL_426891      ------------------------------------------------------------
EPI_ISL_426892      ------------------------------------------------------------
EPI_ISL_426894      ------------------------------------------------------------
EPI_ISL_426896      ------------------------------------------------------------
EPI_ISL_426897      ------------------------------------------------------------
EPI_ISL_426899      ------------------------------------------------------------
EPI_ISL_426906      ------------------------------------------------------------

ORF1ab         6721 IPMDSTVKNYFITDAQTGSSKCVCSVIDLLLDDFVEIIKSQDLSVVSKVVKVTIDYTEIS
EPI_ISL_413571      ------------------------------------------------------------
EPI_ISL_413574      ------------------------------------------------------------
EPI_ISL_413579      ------------------------------------------------------------
EPI_ISL_413584      ------------------------------------------------------------
EPI_ISL_413587      ------------------------------------------------------------
EPI_ISL_424257      ------------------------------------------------------------
EPI_ISL_425565      ------------------------------------------------------------
EPI_ISL_425575      ------------------------------------------------------------
EPI_ISL_426822      ------------------------------------------------------------
EPI_ISL_426833      ------------------------------------------------------------
EPI_ISL_426885      ------------------------------------------------------------
EPI_ISL_426886      ------------------------------------------------------------
EPI_ISL_426889      ------------------------------------------------------------
EPI_ISL_426891      ------------------------------------------------------------
EPI_ISL_426892      ------------------------------------------------------------
EPI_ISL_426894      ------------------------------------------------------------
EPI_ISL_426896      ------------------------------------------------------------
EPI_ISL_426897      ------------------------------------------------------------
EPI_ISL_426899      ------------------------------------------------------------
EPI_ISL_426906      ------------------------------------------------------------

ORF1ab         6781 FMLWCKDGHVETFYPKLQSSQAWQPGVAMPNLYKMQRMLLEKCDLQNYGDSATLPKGIMM
EPI_ISL_413571      ------------------------------------------------------------
EPI_ISL_413574      ------------------------------------------------------------
EPI_ISL_413579      ------------------------------------------------------------
EPI_ISL_413584      ------------------------------------------------------------
EPI_ISL_413587      ------------------------------------------------------------
EPI_ISL_424257      ------------------------------------------------------------
EPI_ISL_425565      ------------------------------------------------------------
EPI_ISL_425575      ------------------------------------------------------------
EPI_ISL_426822      ------------------------------------------------------------
EPI_ISL_426833      ------------------------------------------------------------
EPI_ISL_426885      ------------------------------------------------------------
EPI_ISL_426886      ------------------------------------------------------------
EPI_ISL_426889      ------------------------------------------------------------
EPI_ISL_426891      ------------------------------------------------------------
EPI_ISL_426892      ------------------------------------------------------------
EPI_ISL_426894      ------------------------------------------------------------
EPI_ISL_426896      ------------------------------------------------------------
EPI_ISL_426897      ------------------------------------------------------------
EPI_ISL_426899      ------------------------------------------------------------
```

EPI_ISL_426906 --

```
ORF1ab          6841 NVAKYTQLCQYLNTLTLAVPYNMRVIHFGAGSDKGVAPGTAVLRQWLPTGTLLVDSDLND
EPI_ISL_413571       ------------------------------------------------------------
EPI_ISL_413574       ------------------------------------------------------------
EPI_ISL_413579       ------------------------------------------------------------
EPI_ISL_413584       ------------------------------------------------------------
EPI_ISL_413587       ------------------------------------------------------------
EPI_ISL_424257       ------------------------------------------------------------
EPI_ISL_425565       ------------------------------------------------------------
EPI_ISL_425575       ------------------------------------------------------------
EPI_ISL_426822       ------------------------------------------------------------
EPI_ISL_426833       ------------------------------------------------------------
EPI_ISL_426885       ------------------------------------------------------------
EPI_ISL_426886       ------------------------------------------------------------
EPI_ISL_426889       ------------------------------------------------------------
EPI_ISL_426891       ------------------------------------------------------------
EPI_ISL_426892       ------------------------------------------------------------
EPI_ISL_426894       ------------------------------------------------------------
EPI_ISL_426896       ------------------------------------------------------------
EPI_ISL_426897       ------------------------------------------------------------
EPI_ISL_426899       ------------------------------------------------------------
EPI_ISL_426906       ------------------------------------------------------------

ORF1ab          6901 FVSDADSTLIGDCATVHTANKWDLIISDMYDPKTKNVTKENDSKEGFFTYICGFIQQKLA
EPI_ISL_413571       ------------------------------------------------------------
EPI_ISL_413574       ------------------------------------------------------------
EPI_ISL_413579       ------------------------------------------------------------
EPI_ISL_413584       ------------------------------------------------------------
EPI_ISL_413587       ------------------------------------------------------------
EPI_ISL_424257       ------------------------------------------------------------
EPI_ISL_425565       ------------------------------------------------------------
EPI_ISL_425575       ------------------------------------------------------------
EPI_ISL_426822       ------------------------------------------------------------
EPI_ISL_426833       ------------------------------------------------------------
EPI_ISL_426885       ------------------------------------------------------------
EPI_ISL_426886       ------------------------------------------------------------
EPI_ISL_426889       ------------------------------------------------------------
EPI_ISL_426891       ------------------------------------------------------------
EPI_ISL_426892       ------------------------------------------------------------
EPI_ISL_426894       ------------------------------------------------------------
EPI_ISL_426896       ------------------------------------------------------------
EPI_ISL_426897       ------------------------------------------------------------
EPI_ISL_426899       ------------------------------------------------------------
EPI_ISL_426906       ------------------------------------------------------------

ORF1ab          6961 LGGSVAIKITEHSWNADLYKLMGHFAWWTAFVTNVNASSSEAFLIGCNYLGKPREQIDGY
EPI_ISL_413571       ------------------------------------------------------------
EPI_ISL_413574       ------------------------------------------------------------
EPI_ISL_413579       ------------------------------------------------------------
EPI_ISL_413584       ------------------------------------------------------------
EPI_ISL_413587       ------------------------------------------------------------
EPI_ISL_424257       ------------------------------------------------------------
EPI_ISL_425565       ------------------------------------------------------------
EPI_ISL_425575       ------------------------------------------------------------
EPI_ISL_426822       ------------------------------------------------------------
EPI_ISL_426833       ------------------------------------------------------------
EPI_ISL_426885       ------------------------------------------------------------
EPI_ISL_426886       ------------------------------------------------------------
EPI_ISL_426889       ------------------------------------------------------------
EPI_ISL_426891       ------------------------------------------------------------
EPI_ISL_426892       ------------------------------------------------------------
EPI_ISL_426894       ------------------------------------------------------------
EPI_ISL_426896       ------------------------------------------------------------
EPI_ISL_426897       ------------------------------------------------------------
EPI_ISL_426899       ------------------------------------------------------------
```

EPI_ISL_426906 --

```
ORF1ab         7021 VMHANYIFWRNTNPIQLSSYSLFDMSKFPLKLRGTAVMSLKEGQINDMILSLLSKGRLII
EPI_ISL_413571      ------------------------------------------------------------
EPI_ISL_413574      ------------------------------------------------------------
EPI_ISL_413579      ------------------------------------------------------------
EPI_ISL_413584      ------------------------------------------------------------
EPI_ISL_413587      ------------------------------------------------------------
EPI_ISL_424257      ------------------------------------------------------------
EPI_ISL_425565      ------------------------------------------------------------
EPI_ISL_425575      ------------------------------------------------------------
EPI_ISL_426822      ------------------------------------------------------------
EPI_ISL_426833      ------------------------------------------------------------
EPI_ISL_426885      ------------------------------------------------------------
EPI_ISL_426886      ------------------------------------------------------------
EPI_ISL_426889      ------------------------------------------------------------
EPI_ISL_426891      ------------------------------------------------------------
EPI_ISL_426892      ------------------------------------------------------------
EPI_ISL_426894      ------------------------------------------------------------
EPI_ISL_426896      ------------------------------------------------------------
EPI_ISL_426897      ------------------------------------------------------------
EPI_ISL_426899      ------------------------------------------------------------
EPI_ISL_426906      ------------------------------------------------------------

ORF1ab         7081 RENNRVVISSDVLVNN
EPI_ISL_413571      ----------------
EPI_ISL_413574      ----------------
EPI_ISL_413579      ----------------
EPI_ISL_413584      ----------------
EPI_ISL_413587      ----------------
EPI_ISL_424257      ----------------
EPI_ISL_425565      ----------------
EPI_ISL_425575      ----------------
EPI_ISL_426822      ----------------
EPI_ISL_426833      ----------------
EPI_ISL_426885      ----------------
EPI_ISL_426886      ----------------
EPI_ISL_426889      ----------------
EPI_ISL_426891      ----------------
EPI_ISL_426892      ----------------
EPI_ISL_426894      ----------------
EPI_ISL_426896      ----------------
EPI_ISL_426897      ----------------
EPI_ISL_426899      ----------------
EPI_ISL_426906      ----------------
```

Clade L

```
ORF1ab              1 MESLVPGFNEKTHVQLSLPVLQVRDVLVRGFGDSVEEVLSEARQHLKDGTCGLVEVEKGV
EPI_ISL_411066      1 ------------------------------------------------------------
EPI_ISL_413522      1 ------------------------------------------------------------
EPI_ISL_413558      1 ------------------------------------------------------------
EPI_ISL_413559      1 ------------------------------------------------------------
EPI_ISL_413561      1 ------------------------------------------------------------
EPI_ISL_413692      1 ------------------------------------------------------------
EPI_ISL_413693      1 ------------------------------------------------------------
EPI_ISL_413694      1 ------------------------------------------------------------
EPI_ISL_414692      1 ------------------------------------------------------------
EPI_ISL_424359      1 ------------------------------------------------------------
EPI_ISL_424396      1 ------------------------------------------------------------
EPI_ISL_424399      1 ------------------------------------------------------------
EPI_ISL_425530      1 ------------------------------------------------------------
EPI_ISL_425561      1 ------------------------------------------------------------
EPI_ISL_425614      1 ------------------------------------------------------------
EPI_ISL_425635      1 ------------------------------------------------------------
EPI_ISL_426848      1 ------------------------------------------------------------
EPI_ISL_426850      1 ------------------------------------------------------------
EPI_ISL_426955      1 ------------------------------------------------------------
EPI_ISL_426963      1 ------------------------------------------------------------

ORF1ab             61 LPQLEQPYVFIKRSDARTAPHGHVMVELVAELEGIQYGRSGETLGVLVPHVGEIPVAYRK
EPI_ISL_411066      1 ------------------------------------------------------------
EPI_ISL_413522      1 ------------------------------------------------------------
EPI_ISL_413558      1 ------------------------------------------------------------
EPI_ISL_413559      1 ------------------------------------------------------------
EPI_ISL_413561      1 ------------------------------------------------------------
EPI_ISL_413692      1 ------------------------------------------------------------
EPI_ISL_413693      1 ------------------------------------------------------------
EPI_ISL_413694      1 ------------------------------------------------------------
EPI_ISL_414692      1 ------------------------------------------------------------
EPI_ISL_424359      1 ------------------------------------------------------------
EPI_ISL_424396      1 ------------------------------------------------------------
EPI_ISL_424399      1 ------------------------------------------------------------
EPI_ISL_425530      1 ------------------------------------------------------------
EPI_ISL_425561      1 ------------------------------------------------------------
EPI_ISL_425614      1 ------------------------------------------------------------
EPI_ISL_425635      1 ------------------------------------------------------------
EPI_ISL_426848      1 ------------------------------------------------------------
EPI_ISL_426850      1 ------------------------------------------------------------
EPI_ISL_426955      1 ------------------------------------------------------------
EPI_ISL_426963      1 ------------------------------------------------------------

ORF1ab            121 VLLRKNGNKGAGGHSYGADLKSFDLGDELGTDPYEDFQENWNTKHSSGVTRELMRELNGG
EPI_ISL_411066      1 ------------------------------------------------------------
EPI_ISL_413522      1 ------------------------------------------------------------
EPI_ISL_413558      1 ------------------------------------------------------------
EPI_ISL_413559      1 ------------------------------------------------------------
EPI_ISL_413561      1 ------------------------------------------------------------
EPI_ISL_413692      1 ------------------------------------------------------------
EPI_ISL_413693      1 ------------------------------------------------------------
EPI_ISL_413694      1 ------------------------------------------------------------
EPI_ISL_414692      1 ------------------------------------------------------------
EPI_ISL_424359      1 ------------------------------------------------------------
EPI_ISL_424396      1 ------------------------------------------------------------
EPI_ISL_424399      1 ------------------------------------------------------------
EPI_ISL_425530      1 ------------------------------------------------------------
EPI_ISL_425561      1 ------------------------------------------------------------
EPI_ISL_425614      1 ------------------------------------------------------------
```

```
EPI_ISL_425635    1 ------------------------------------------------------------
EPI_ISL_426848    1 ------------------------------------------------------------
EPI_ISL_426850    1 ------------------------------------------------------------
EPI_ISL_426955    1 ------------------------------------------------------------
EPI_ISL_426963    1 ------------------------------------------------------------
```

```
ORF1ab          181 AYTRYVDNNFCGPDGYPLECIKDLLARAGKASCTLSEQLDFIDTKRGVYCCREHEHEIAW
EPI_ISL_411066    1 ------------------------------------------------------------
EPI_ISL_413522    1 ------------------------------------------------------------
EPI_ISL_413558    1 ------------------------------------------------------------
EPI_ISL_413559    1 ------------------------------------------------------------
EPI_ISL_413561    1 ------------------------------------------------------------
EPI_ISL_413692    1 ------------------------------------------------------------
EPI_ISL_413693    1 ------------------------------------------------------------
EPI_ISL_413694    1 ------------------------------------------------------------
EPI_ISL_414692    1 ------------------------------------------------------------
EPI_ISL_424359    1 ------------------------------------------------------------
EPI_ISL_424396    1 ------------------------------------------------------------
EPI_ISL_424399    1 ------------------------------------------------------------
EPI_ISL_425530    1 ------------------------------------------------------------
EPI_ISL_425561    1 ------------------------------------------------------------
EPI_ISL_425614    1 ------------------------------------------------------------
EPI_ISL_425635    1 ------------------------------------------------------------
EPI_ISL_426848    1 ------------------------------------------------------------
EPI_ISL_426850    1 ------------------------------------------------------------
EPI_ISL_426955    1 ------------------------------------------------------------
EPI_ISL_426963    1 ------------------------------------------------------------

ORF1ab          241 YTERSEKSYELQTPFEIKLAKKFDTFNGECPNFVFPLNSIIKTIQPRVEKKKLDGFMGRI
EPI_ISL_411066    1 ------------------------------------------------------------
EPI_ISL_413522    1 ------------------------------------------------------------
EPI_ISL_413558    1 ------------------------------------------------------------
EPI_ISL_413559    1 ------------------------------------------------------------
EPI_ISL_413561    1 ------------------------------------------------------------
EPI_ISL_413692    1 ------------------------------------------------------------
EPI_ISL_413693    1 ------------------------------------------------------------
EPI_ISL_413694    1 ------------------------------------------------------------
EPI_ISL_414692    1 ------------------------------------------------------------
EPI_ISL_424359    1 ------------------------------------------------------------
EPI_ISL_424396    1 ------------------------------------------------------------
EPI_ISL_424399    1 ------------------------------------------------------------
EPI_ISL_425530    1 ------------------------------------------------------------
EPI_ISL_425561    1 ------------------------------------------------------------
EPI_ISL_425614    1 ------------------------------------------------------------
EPI_ISL_425635    1 ------------------------------------------------------------
EPI_ISL_426848    1 ------------------------------------------------------------
EPI_ISL_426850    1 ------------------------------------------------------------
EPI_ISL_426955    1 ------------------------------------------------------------
EPI_ISL_426963    1 ------------------------------------------------------------

ORF1ab          301 RSVYPVASPNECNQMCLSTLMKCDHCGETSWQTGDFVKATCEFCGTENLTKEGATTCGYL
EPI_ISL_411066    1 ------------------------------------------------------------
EPI_ISL_413522    1 ------------------------------------------------------------
EPI_ISL_413558    1 ------------------------------------------------------------
EPI_ISL_413559    1 ------------------------------------------------------------
EPI_ISL_413561    1 ------------------------------------------------------------
EPI_ISL_413692    1 ------------------------------------------------------------
EPI_ISL_413693    1 ------------------------------------------------------------
EPI_ISL_413694    1 ------------------------------------------------------------
EPI_ISL_414692    1 ------------------------------------------------------------
EPI_ISL_424359    1 ------------------------------------------------------------
EPI_ISL_424396    1 ------------------------------------------------------------
EPI_ISL_424399    1 ------------------------------------------------------------
EPI_ISL_425530    1 ------------------------------------------------------------
EPI_ISL_425561    1 ------------------------------------------------------------
EPI_ISL_425614    1 ------------------------------------------------------------
EPI_ISL_425635    1 ------------------------------------------------------------
EPI_ISL_426848    1 ------------------------------------------------------------
EPI_ISL_426850    1 ------------------------------------------------------------
EPI_ISL_426955    1 ------------------------------------------------------------
```

EPI_ISL_426963   1 --

```
ORF1ab         361 PQNAVVKIYCPACHNSEVGPEHSLAEYHNESGLKTILRKGGRTIAFGGCVFSYVGCHNKC
EPI_ISL_411066   1 ------------------------------------------------------------
EPI_ISL_413522   1 ------------------------------------------------------------
EPI_ISL_413558   1 ------------------------------------------------------------
EPI_ISL_413559   1 ------------------------------------------------------------
EPI_ISL_413561   1 ------------------------------------------------------------
EPI_ISL_413692   1 ------------------------------------------------------------
EPI_ISL_413693   1 ------------------------------------------------------------
EPI_ISL_413694   1 ------------------------------------------------------------
EPI_ISL_414692   1 ------------------------------------------------------------
EPI_ISL_424359   1 ------------------------------------------------------------
EPI_ISL_424396   1 ------------------------------------------------------------
EPI_ISL_424399   1 ------------------------------------------------------------
EPI_ISL_425530   1 ------------------------------------------------------------
EPI_ISL_425561   1 ------------------------------------------------------------
EPI_ISL_425614   1 ------------------------------------------------------------
EPI_ISL_425635   1 ------------------------------------------------------------
EPI_ISL_426848   1 ------------------------------------------------------------
EPI_ISL_426850   1 ------------------------------------------------------------
EPI_ISL_426955   1 ------------------------------------------------------------
EPI_ISL_426963   1 ------------------------------------------------------------
```

```
ORF1ab         421 AYWVPRASANIGCNHTGVVGEGSEGLNDNLLEILQKEKVNINIVGDFKLNEEIAIILASF
EPI_ISL_411066   1 ------------------------------------------------------------
EPI_ISL_413522   1 ------------------------------------------------------------
EPI_ISL_413558   1 ------------------------------------------------------------
EPI_ISL_413559   1 ------------------------------------------------------------
EPI_ISL_413561   1 ------------------------------------------------------------
EPI_ISL_413692   1 ------------------------------------------------------------
EPI_ISL_413693   1 ------------------------------------------------------------
EPI_ISL_413694   1 ------------------------------------------------------------
EPI_ISL_414692   1 ------------------------------------------------------------
EPI_ISL_424359   1 ------------------------------------------------------------
EPI_ISL_424396   1 ------------------------------------------------------------
EPI_ISL_424399   1 ------------------------------------------------------------
EPI_ISL_425530   1 ------------------------------------------------------------
EPI_ISL_425561   1 ------------------------------------------------------------
EPI_ISL_425614   1 ------------------------------------------------------------
EPI_ISL_425635   1 ------------------------------------------------------------
EPI_ISL_426848   1 ------------------------------------------------------------
EPI_ISL_426850   1 ------------------------------------------------------------
EPI_ISL_426955   1 ------------------------------------------------------------
EPI_ISL_426963   1 ------------------------------------------------------------
```

```
ORF1ab         481 SASTSAFVETVKGLDYKAFKQIVESCGNFKVTKGKAKKGAWNIGEQKSILSPLYAFASEA
EPI_ISL_411066   1 ------------------------------------------------------------
EPI_ISL_413522   1 ------------------------------------------------------------
EPI_ISL_413558   1 ------------------------------------------------------------
EPI_ISL_413559   1 ------------------------------------------------------------
EPI_ISL_413561   1 ------------------------------------------------------------
EPI_ISL_413692   1 ------------------------------------------------------------
EPI_ISL_413693   1 ------------------------------------------------------------
EPI_ISL_413694   1 ------------------------------------------------------------
EPI_ISL_414692   1 ------------------------------------------------------------
EPI_ISL_424359   1 ------------------------------------------------------------
EPI_ISL_424396   1 ------------------------------------------------------------
EPI_ISL_424399   1 ------------------------------------------------------------
EPI_ISL_425530   1 ------------------------------------------------------------
EPI_ISL_425561   1 ------------------------------------------------------------
EPI_ISL_425614   1 ------------------------------------------------------------
EPI_ISL_425635   1 ------------------------------------------------------------
EPI_ISL_426848   1 ------------------------------------------------------------
EPI_ISL_426850   1 ------------------------------------------------------------
EPI_ISL_426955   1 ------------------------------------------------------------
```

EPI_ISL_426963   1 --

```
ORF1ab          541 ARVVRSIFSRTLETAQNSVRVLQKAAITILDGISQYSLRLIDAMMFTSDLATNNLVVMAY
EPI_ISL_411066    1 ------------------------------------------------------------
EPI_ISL_413522    1 ------------------------------------------------------------
EPI_ISL_413558    1 ------------------------------------------------------------
EPI_ISL_413559    1 ------------------------------------------------------------
EPI_ISL_413561    1 ------------------------------------------------------------
EPI_ISL_413692    1 ------------------------------------------------------------
EPI_ISL_413693    1 ------------------------------------------------------------
EPI_ISL_413694    1 ------------------------------------------------------------
EPI_ISL_414692    1 ------------------------------------------------------------
EPI_ISL_424359    1 ------------------------------------------------------------
EPI_ISL_424396    1 ------------------------------------------------------------
EPI_ISL_424399    1 ------------------------------------------------------------
EPI_ISL_425530    1 ------------------------------------------------------------
EPI_ISL_425561    1 ------------------------------------------------------------
EPI_ISL_425614    1 ------------------------------------------------------------
EPI_ISL_425635    1 ------------------------------------------------------------
EPI_ISL_426848    1 ------------------------------------------------------------
EPI_ISL_426850    1 ------------------------------------------------------------
EPI_ISL_426955    1 ------------------------------------------------------------
EPI_ISL_426963    1 ------------------------------------------------------------

ORF1ab          601 ITGGVVQLTSQWLTNIFGTVYEKLKPVLDWLEEKFKEGVEFLRDGWEIVKFISTCACEIV
EPI_ISL_411066    1 ------------------------------------------------------------
EPI_ISL_413522    1 ------------------------------------------------------------
EPI_ISL_413558    1 ------------------------------------------------------------
EPI_ISL_413559    1 ------------------------------------------------------------
EPI_ISL_413561    1 ------------------------------------------------------------
EPI_ISL_413692    1 ------------------------------------------------------------
EPI_ISL_413693    1 ------------------------------------------------------------
EPI_ISL_413694    1 ------------------------------------------------------------
EPI_ISL_414692    1 ------------------------------------------------------------
EPI_ISL_424359    1 ------------------------------------------------------------
EPI_ISL_424396    1 ------------------------------------------------------------
EPI_ISL_424399    1 ------------------------------------------------------------
EPI_ISL_425530    1 ------------------------------------------------------------
EPI_ISL_425561    1 ------------------------------------------------------------
EPI_ISL_425614    1 ------------------------------------------------------------
EPI_ISL_425635    1 ------------------------------------------------------------
EPI_ISL_426848    1 ------------------------------------------------------------
EPI_ISL_426850    1 ------------------------------------------------------------
EPI_ISL_426955    1 ------------------------------------------------------------
EPI_ISL_426963    1 ------------------------------------------------------------

ORF1ab          661 GGQIVTCAKEIKESVQTFFKLVNKFLALCADSIIIGGAKLKALNLGETFVTHSKGLYRKC
EPI_ISL_411066    1 ------------------------------------------------------------
EPI_ISL_413522    1 ------------------------------------------------------------
EPI_ISL_413558    1 ------------------------------------------------------------
EPI_ISL_413559    1 ------------------------------------------------------------
EPI_ISL_413561    1 ------------------------------------------------------------
EPI_ISL_413692    1 ------------------------------------------------------------
EPI_ISL_413693    1 ------------------------------------------------------------
EPI_ISL_413694    1 ------------------------------------------------------------
EPI_ISL_414692    1 ------------------------------------------------------------
EPI_ISL_424359    1 ------------------------------------------------------------
EPI_ISL_424396    1 ------------------------------------------------------------
EPI_ISL_424399    1 ------------------------------------------------------------
EPI_ISL_425530    1 ------------------------------------------------------------
EPI_ISL_425561    1 ------------------------------------------------------------
EPI_ISL_425614    1 ------------------------------------------------------------
EPI_ISL_425635    1 ------------------------------------------------------------
EPI_ISL_426848    1 ------------------------------------------------------------
EPI_ISL_426850    1 ------------------------------------------------------------
EPI_ISL_426955    1 ------------------------------------------------------------
```

EPI_ISL_426963   1 --

```
ORF1ab          721 VKSREETGLLMPLKAPKEIIFLEGETLPTEVLTEEVVLKTGDLQPLEQPTSEAVEAPLVG
EPI_ISL_411066    1 ------------------------------------------------------------
EPI_ISL_413522    1 ------------------------------------------------------------
EPI_ISL_413558    1 ------------------------------------------------------------
EPI_ISL_413559    1 ------------------------------------------------------------
EPI_ISL_413561    1 ------------------------------------------------------------
EPI_ISL_413692    1 ------------------------------------------------------------
EPI_ISL_413693    1 ------------------------------------------------------------
EPI_ISL_413694    1 ------------------------------------------------------------
EPI_ISL_414692    1 ------------------------------------------------------------
EPI_ISL_424359    1 ------------------------------------------------------------
EPI_ISL_424396    1 ------------------------------------------------------------
EPI_ISL_424399    1 ------------------------------------------------------------
EPI_ISL_425530    1 ------------------------------------------------------------
EPI_ISL_425561    1 ------------------------------------------------------------
EPI_ISL_425614    1 ------------------------------------------------------------
EPI_ISL_425635    1 ------------------------------------------------------------
EPI_ISL_426848    1 ------------------------------------------------------------
EPI_ISL_426850    1 ------------------------------------------------------------
EPI_ISL_426955    1 ------------------------------------------------------------
EPI_ISL_426963    1 ------------------------------------------------------------

ORF1ab          781 TPVCINGLMLLEIKDTEKYCALAPNMMVTNNTFTLKGGAPTKVTFGDDTVIEVQGYKSVN
EPI_ISL_411066    1 --------------------------------------APTKVTFGDDTVIEVQGYKSVN
EPI_ISL_413522    1 --------------------------------------APTKVTFGDDTVIEVQGYKSVN
EPI_ISL_413558    1 --------------------------------------APTKVTFGDDTVIEVQGYKSVN
EPI_ISL_413559    1 --------------------------------------APTKVTFGDDTVIEVQGYKSVN
EPI_ISL_413561    1 --------------------------------------APTKVTFGDDTVIEVQGYKSVN
EPI_ISL_413692    1 --------------------------------------APTKVTFGDDTVIEVQGYKSVN
EPI_ISL_413693    1 --------------------------------------APTKVTFGDDTVIEVQGYKSVN
EPI_ISL_413694    1 --------------------------------------APTKVTFGDDTVIEVQGYKSVN
EPI_ISL_414692    1 --------------------------------------APTKVTFGDDTVIEVQGYKSVN
EPI_ISL_424359    1 --------------------------------------APTKVTFGDDTVIEVQGYKSVN
EPI_ISL_424396    1 --------------------------------------APTKVTFGDDTVIEVQGYKSVN
EPI_ISL_424399    1 --------------------------------------APTKVTFGDDTVIEVQGYKSVN
EPI_ISL_425530    1 --------------------------------------APTKVTFGDDTVIEVQGYKSVN
EPI_ISL_425561    1 --------------------------------------APTKVTFGDDTVIEVQGYKSVN
EPI_ISL_425614    1 --------------------------------------APTKVTFGDDTVIEVQGYKSVN
EPI_ISL_425635    1 --------------------------------------APTKVTFGDDTVIEVQGYKSVN
EPI_ISL_426848    1 --------------------------------------APTKVTFGDDTVIEVQGYKSVN
EPI_ISL_426850    1 --------------------------------------APTKVTFGDDTVIEVQGYKSVN
EPI_ISL_426955    1 --------------------------------------APTKVTFGDDTVIEVQGYKSVN
EPI_ISL_426963    1 --------------------------------------APTKVTFGDDTVIEVQGYKSVN

ORF1ab          841 ITFELDERIDKVLNEKCSAYTVELGTEVNEFACVVADAVIKTLQPVSELLTPLGIDLDEW
EPI_ISL_411066   23 ITFELDERIDKVLNEKCSAYTVELGTEVNEFACVVADAVIKTLQPVSELLTPLGIDLDEW
EPI_ISL_413522   23 ITFELDERIDKVLNEKCSAYTVELGTEVNEFACVVADAVIKTLQPVSELLTPLGIDLDEW
EPI_ISL_413558   23 ITFELDERIDKVLNEKCSAYTVELGTEVNEFACVVADAVIKTLQPVSELLTPLGIDLDEW
EPI_ISL_413559   23 ITFELDERIDKVLNEKCSAYTVELGTEVNEFACVVADAVIKTLQPVSELLTPLGIDLDEW
EPI_ISL_413561   23 ITFELDERIDKVLNEKCSAYTVELGTEVNEFACVVTDAVIKTLQPVSELLTPLGIDLDEW
EPI_ISL_413692   23 ITFELDERIDKVLNEKCSAYTVELGTEVNEFACVVADAVIKTLQPVSELLTPLGIDLDEW
EPI_ISL_413693   23 ITFELDERIDKVLNEKCSAYTVELGTEVNEFACVVADAVIKTLQPVSELLTPLGIDLDEW
EPI_ISL_413694   23 ITFELDERIDKVLNEKCSAYTVELGTEVNEFACVVADAVIKTLQPVSELLTPLGIDLDEW
EPI_ISL_414692   23 ITFELDERIDKVLNEKCSAYTVELGTEVNEFACVVADAVIKTLQPVSELLTPLGIDLDEW
EPI_ISL_424359   23 ITFELDERIDKVLNEKCSAYTVELGTEVNEFACVVADAVIKTLQPVSELLTPLGIDLDEW
EPI_ISL_424396   23 ITFELDERIDKVLNEKCSAYTVELGTEVNEFACVVADAVIKTLQPVSELLTPLGIDLDEW
EPI_ISL_424399   23 ITFELDERIDKVLNEKCSAYTVELGTEVNEFACVVADAVIKTLQPVSELLTPLGIDLDEW
EPI_ISL_425530   23 ITFELDERIDKVLNEKCSAYTVELGTEVNEFACVVTDAVIKTLQPVSELLTPLGIDLDEW
EPI_ISL_425561   23 ITFELDERIDKVLNEKCSAYTVELGTEVNEFACVVTDAVIKTLQPVSELLTPLGIDLDEW
EPI_ISL_425614   23 ITFELDERIDKVLNEKCSAYTVELGTEVNEFACVVTDAVIKTLQPVSELLTPLGIDLDEW
EPI_ISL_425635   23 ITFELDERIDKVLNEKCSAYTVELGTEVNEFACVVTDAVIKTLQPVSELLTPLGIDLDEW
EPI_ISL_426848   23 ITFELDERIDKVLNEKCSAYTVELGTEVNEFACVVTDAVIKTLQPVSELLTPLGIDLDEW
EPI_ISL_426850   23 ITFELDERIDKVLNEKCSAYTVELGTEVNEFACVVTDAVIKTLQPVSELLTPLGIDLDEW
EPI_ISL_426955   23 ITFELDERIDKVLNEKCSAYTVELGTEVNEFACVVTDAVIKTLQPVSELLTPLGIDLDEW
```

EPI_ISL_426963 23 ITFELDERIDKVLNEKCSAYTVELGTEVNEFACVVTDAVIKTLQPVSELLTPLGIDLDEW

```
ORF1ab          901  SMATYYLFDESGEFKLASHMYCSFYPPDEDEEEGDCEEEEFEPSTQYEYGTEDDYQGKPL
EPI_ISL_411066   83  SMATYYLFDESGEFKLASHMYCSFYPPDEDEEEGDCEEEEFEPSTQYEYGTEDDYQGKPL
EPI_ISL_413522   83  SMATYYLFDESGEFKLASHMYCSFYPPDEDEEEGDCEEEEFEPSTQYEYGTEDDYQGKPL
EPI_ISL_413558   83  SMATYYLFDESGEFKLASHMYCSFYPPDEDEEEGDCEEEEFEPSTQYEYGTEDDYQGKPL
EPI_ISL_413559   83  SMATYYLFDESGEFKLASHMYCSFYPPDEDEEEGDCEEEEFEPSTQYEYGTEDDYQGKPL
EPI_ISL_413561   83  SMATYYLFDESGEFKLASHMYCSFYPPDEDEEEGDCEEEEFEPSTQYEYGTEDDYQGKPL
EPI_ISL_413692   83  SMATYYLFDESGEFKLASHMYCSFYPPDEDEEEGDCEEEEFEPSTQYEYGTEDDYQGKPL
EPI_ISL_413693   83  SMATYYLFDESGEFKLASHMYCSFYPPDEDEEEGDCEEEEFEPSTQYEYGTEDDYQGKPL
EPI_ISL_413694   83  SMATYYLFDESGEFKLASHMYCSFYPPDEDEEEGDCEEEEFEPSTQYEYGTEDDYQGKPL
EPI_ISL_414692   83  SMATYYLFDESGEFKLASHMYCSFYPPDEDEEEGDCEEEEFEPSTQYEYGTEDDYQGKPL
EPI_ISL_424359   83  SMATYYLFDESGEFKLASHMYCSFYPPDEDEEEGDCEEEEFEPSTQYEYGTEDDYQGKPL
EPI_ISL_424396   83  SMATYYLFDESGEFKLASHMYCSFYPPDEDEEEGDCEEEEFEPSTQYEYGTEDDYQGKPL
EPI_ISL_424399   83  SMATYYLFDESGEFKLASHMYCSFYPPDEDEEEGDCEEEEFEPSTQYEYGTEDDYQGKPL
EPI_ISL_425530   83  SMATYYLFDESGEFKLASHMYCSFYPPDEDEEEGDCEEEEFEPSTQYEYGTEDDYQGKPL
EPI_ISL_425561   83  SMATYYLFDESGEFKLASHMYCSFYPPDEDEEEGDCEEEEFEPSTQYEYGTEDDYQGKPL
EPI_ISL_425614   83  SMATYYLFDESGEFKLASHMYCSFYPPDEDEEEGDCEEEEFEPSTQYEYGTEDDYQGKPL
EPI_ISL_425635   83  SMATYYLFDESGEFKLASHMYCSFYPPDEDEEEGDCEEEEFEPSTQYEYGTEDDYQGKPL
EPI_ISL_426848   83  SMATYYLFDESGEFKLASHMYCSFYPPDEDEEEGDCEEEEFEPSTQYEYGTEDDYQGKPL
EPI_ISL_426850   83  SMATYYLFDESGEFKLASHMYCSFYPPDEDEEEGDCEEEEFEPSTQYEYGTEDDYQGKPL
EPI_ISL_426955   83  SMATYYLFDESGEFKLASHMYCSFYPPDEDEEEGDCEEEEFEPSTQYEYGTEDDYQGKPL
EPI_ISL_426963   83  SMATYYLFDESGEFKLASHMYCSFYPPDEDEEEGDCEEEEFEPSTQYEYGTEDDYQGKPL
```

```
ORF1ab          961  EFGATSAALQPEEEQEEDWLDDDSQQTVGQQDGSEDNQTTTIQTIVEVQPQLEMELTPVV
EPI_ISL_411066  143  EFGATSAALQPEEEQEEDWLDDDSQQTVGQQDGSEDNQTTTIQTIVEVQPQLEMELTPVV
EPI_ISL_413522  143  EFGATSAALQPEEEQEEDWLDDDSQQTVGQQDGSEDNQTTTIQTIVEVQPQLEMELTPVV
EPI_ISL_413558  143  EFGATSAALQPEEEQEEDWLDDDSQQTVGQQDGSEDNQTTTIQTIVEVQPQLEMELTPVV
EPI_ISL_413559  143  EFGATSAALQPEEEQEEDWLDDDSQQTVGQQDGSEDNQTTTIQTIVEVQPQLEMELTPVV
EPI_ISL_413561  143  EFGATSAALQPEEEQEEDWLDDDSQQTVGQQDGSEDNQTTTIQTIVEVQPQLEMELTPVV
EPI_ISL_413692  143  EFGATSAALQPEEEQEEDWLDDDSQQTVGQQDGSEDNQTTTIQTIVEVQPQLEMELTPVV
EPI_ISL_413693  143  EFGATSAALQPEEEQEEDWLDDDSQQTVGQQDGSEDNQTTTIQTIVEVQPQLEMELTPVV
EPI_ISL_413694  143  EFGATSAALQPEEEQEEDWLDDDSQQTVGQQDGSEDNQTTTIQTIVEVQPQLEMELTPVV
EPI_ISL_414692  143  EFGATSAALQPEEEQEEDWLDDDSQQTVGQQDGSEDNQTTTIQTIVEVQPQLEMELTPVV
EPI_ISL_424359  143  EFGATSAALQPEEEQEEDWLDDDSQQTVGQQDGSEDNQTTTIQTIVEVQPQLEMELTPVV
EPI_ISL_424396  143  EFGATSAALQPEEEQEEDWLDDDSQQTVGQQDGSEDNQTTTIQTIVEVQPQLEMELTPVV
EPI_ISL_424399  143  EFGATSAALQPEEEQEEDWLDDDSQQTVGQQDGSEDNQTTTIQTIVEVQPQLEMELTPVV
EPI_ISL_425530  143  EFGATSAALQPEEEQEEDWLDDDSQQTVGQQDGSEDNQTTTIQTIVEVQPQLEMELTPVV
EPI_ISL_425561  143  EFGATSAALQPEEEQEEDWLDDDSQQTVGQQDGSEDNQTTTIQTIVEVQPQLEMELTPVV
EPI_ISL_425614  143  EFGATSAALQPEEEQEEDWLDDDSQQTVGQQDGSEDNQTTTIQTIVEVQPQLEMELTPVV
EPI_ISL_425635  143  EFGATSAALQPEEEQEEDWLDDDSQQTVGQQDGSEDNQTTTIQTIVEVQPQLEMELTPVV
EPI_ISL_426848  143  EFGATSAALQPEEEQEEDWLDDDSQQTVGQQDGSEDNQTTTIQTIVEVQPQLEMELTPVV
EPI_ISL_426850  143  EFGATSAALQPEEEQEEDWLDDDSQQTVGQQDGSEDNQTTTIQTIVEVQPQLEMELTPVV
EPI_ISL_426955  143  EFGATSAALQPEEEQEEDWLDDDSQQTVGQQDGSEDNQTTTIQTIVEVQPQLEMELTPVV
EPI_ISL_426963  143  EFGATSAALQPEEEQEEDWLDDDSQQTVGQQDGSEDNQTTTIQTIVEVQPQLEMELTPVV
```

```
ORF1ab         1021  QTIEVNSFSGYLKLTDNVYIKNADIVEEAKKVKPTVVVNAANVYLKHGGGVAGALNKATN
EPI_ISL_411066  203  QTIEVNSFSGYLKLTDNVYIKNADIVEEAKKVKPTVVVNAANVYLKHGGGVAGALNKATN
EPI_ISL_413522  203  QTIEVNSFSGYLKLTDNVYIKNADIVEEAKKVKPTVVVNAANVYLKHGGGVAGALNKATN
EPI_ISL_413558  203  QTIEVNSFSGYLKLTDNVYIKNADIVEEAKKVKPTVVVNAANVYLKHGGGVAGALNKATN
EPI_ISL_413559  203  QTIEVNSFSGYLKLTDNVYIKNADIVEEAKKVKPTVVVNAANVYLKHGGGVAGALNKATN
EPI_ISL_413561  203  QTIEVNSFSGYLKLTDNVYIKNADIVEEAKKVKPTVVVNAANVYLKHGGGVAGALNKATN
EPI_ISL_413692  203  QTIEVNSFSGYLKLTDNVYIKNADIVEEAKKVKPTVVVNAANVYLKHGGGVAGALNKATN
EPI_ISL_413693  203  QTIEVNSFSGYLKLTDNVYIKNADIVEEAKKVKPTVVVNAANVYLKHGGGVAGALNKATN
EPI_ISL_413694  203  QTIEVNSFSGYLKLTDNVYIKNADIVEEAKKVKPTVVVNAANVYLKHGGGVAGALNKATN
EPI_ISL_414692  203  QTIEVNSFSGYLKLTDNVYIKNADIVEEAKKVKPTVVVNAANVYLKHGGGVAGALNKATN
EPI_ISL_424359  203  QTIEVNSFSGYLKLTDNVYIKNADIVEEAKKVKPTVVVNAANVYLKHGGGVAGALNKATN
EPI_ISL_424396  203  QTIEVNSFSGYLKLTDNVYIKNADIVEEAKKVKPTVVVNAANVYLKHGGGVAGALNKATN
EPI_ISL_424399  203  QTIEVNSFSGYLKLTDNVYIKNADIVEEAKKVKPTVVVNAANVYLKHGGGVAGALNKATN
EPI_ISL_425530  203  QTIEVNSFSGYLKLTDNVYIKNADIVEEAKKVKPTVVVNAANVYLKHGGGVAGALNKATN
EPI_ISL_425561  203  QTIEVNSFSGYLKLTDNVYIKNADIVEEAKKVKPTVVVNAANVYLKHGGGVAGALNKATN
EPI_ISL_425614  203  QTIEVNSFSGYLKLTDNVYIKNADIVEEAKKVKPTVVVNAANVYLKHGGGVAGALNKATN
EPI_ISL_425635  203  QTIEVNSFSGYLKLTDNVYIKNADIVEEAKKVKPTVVVNAANVYLKHGGGVAGALNKATN
EPI_ISL_426848  203  QTIEVNSFSGYLKLTDNVYIKNADIVEEAKKVKPTVVVNAANVYLKHGGGVAGALNKATN
EPI_ISL_426850  203  QTIEVNSFSGYLKLTDNVYIKNADIVEEAKKVKPTVVVNAANVYLKHGGGVAGALNKATN
EPI_ISL_426955  203  QTIEVNSFSGYLKLTDNVYIKNADIVEEAKKVKPTVVVNAANVYLKHGGGVAGALNKATN
```

EPI_ISL_426963 203 QTIEVNSFSGYLKLTDNVYIKNADIVEEAKKVKPTVVVNAANVYLKHGGGVAGALNKATN

```
ORF1ab          1081 NAMQVESDDYIATNGPLKVGGSCVLSGHNLAKHCLHVVGPNVNKGEDIQLLKSAYENFNQ
EPI_ISL_411066   263 NAMQVESDDYIATNGPLKVGGSCVLSGHNLAKHCLHVVGPNVNKGEDIQLLKSAYENFNQ
EPI_ISL_413522   263 NAMQVESDDYIATNGPLKVGGSCVLSGHNLAKHCLHVVGPNVNKGEDIQLLKSAYENFNQ
EPI_ISL_413558   263 NAMQVESDDYIATNGPLKVGGSCVLSGHNLAKHCLHVVGPNVNKGEDIQLLKSAYENFNQ
EPI_ISL_413559   263 NAMQVESDDYIATNGPLKVGGSCVLSGHNLAKHCLHVVGPNVNKGEDIQLLKSAYENFNQ
EPI_ISL_413561   263 NAMQVESDDYIATNGPLKVGGSCVLSGHNLAKHCLHVVGPNVNKGEDIQLLKSAYENFNQ
EPI_ISL_413692   263 NAMQVESDDYIATNGPLKVGGSCVLSGHNLAKHCLHVVGPNVNKGEDIQLLKSAYENFNQ
EPI_ISL_413693   263 NAMQVESDDYIATNGPLKVGGSCVLSGHNLAKHCLHVVGPNVNKGEDIQLLKSAYENFNQ
EPI_ISL_413694   263 NAMQVESDDYIATNGPLKVGGSCVLSGHNLAKHCLHVVGPNVNKGEDIQLLKSAYENFNQ
EPI_ISL_414692   263 NAMQVESDDYIATNGPLKVGGSCVLSGHNLAKHCLHVVGPNVNKGEDIQLLKSAYENFNQ
EPI_ISL_424359   263 NAMQVESDDYIATNGPLKVGGSCVLSGHNLAKHCLHVVGPNVNKGEDIQLLKSAYENFNQ
EPI_ISL_424396   263 NAMQVESDDYIATNGPLKVGGSCVLSGHNLAKHCLHVVGPNVNKGEDIQLLKSAYENFNQ
EPI_ISL_424399   263 NAMQVESDDYIATNGPLKVGGSCVLSGHNLAKHCLHVVGPNVNKGEDIQLLKSAYENFNQ
EPI_ISL_425530   263 NAMQVESDDYIATNGPLKVVGSCVLSGHNLAKHCLHVVGPNVNKGEDIQLLKSAYENFNQ
EPI_ISL_425561   263 NAMQVESDDYIATNGPLKVGGSCVLSGHNLAKHCLHVVGPNVNKGEDIQLLKSAYENFNQ
EPI_ISL_425614   263 NAMQVESDDYIATNGPLKVGGSCVLSGHNLAKHCLHVVGPNVNKGEDIQLLKSAYENFNQ
EPI_ISL_425635   263 NAMQVESDDYIATNGPLKVGGSCVLSGHNLAKHCLHVVGPNVNKGEDIQLLKSAYENFNQ
EPI_ISL_426848   263 NAMQVESDDYIATNGPLKVGGSCVLSGHNLAKHCLHVVGPNVNKGEDIQLLKSAYENFNQ
EPI_ISL_426850   263 NAMQVESDDYIATNGPLKVGGSCVLSGHNLAKHCLHVVGPNVNKGEDIQLLKSAYENFNQ
EPI_ISL_426955   263 NAMQVESDDYIATNGPLKVGGSCVLSGHNLAKHCLHVVGPNVNKGEDIQLLKSAYENFNQ
EPI_ISL_426963   263 NAMQVESDDYIATNGPLKVGGSCVLSGHNLAKHCLHVVGPNVNKGEDIQLLKSAYENFNQ
```

```
ORF1ab          1141 HEVLLAPLLSAGIFGADPIHSLRVCVDTVRTNVYLAVFDKNLYDKLVSSFLEMKSEKQVE
EPI_ISL_411066   323 HEVLLAPLLSAGIFGADPIHSLRVCVDTVRTNVYLAVFDKNLYDKLVSSFLEMKSEKQVE
EPI_ISL_413522   323 HEVLLAPLLSAGIFGADPIHSLRVCVDTVRTNVYLAVFDKNLYDKLVSSFLEMKSEKQVE
EPI_ISL_413558   323 HEVLLAPLLSAGIFGADPIHSLRVCVDTVRTNVYLAVFDKNLYDKLVSSFLEMKSEKQVE
EPI_ISL_413559   323 HEVLLAPLLSAGIFGADPIHSLRVCVDTVRTNVYLAVFDKNLYDKLVSSFLEMKSEKQVE
EPI_ISL_413561   323 HEVLLAPLLSAGIFGADPIHSLRVCVDTVRTNVYLAVFDKNLYDKLVSSFLEMKSEKQVE
EPI_ISL_413692   323 HEVLLAPLLSAGIFGADPIHSLRVCVDTVRTNVYLAVFDKNLYDKLVSSFLEMKSEKQVE
EPI_ISL_413693   323 HEVLLAPLLSAGIFGADPIHSLRVCVDTVRTNVYLAVFDKNLYDKLVSSFLEMKSEKQVE
EPI_ISL_413694   323 HEVLLAPLLSAGIFGADPIHSLRVCVDTVRTNVYLAVFDKNLYDKLVSSFLEMKSEKQVE
EPI_ISL_414692   323 HEVLLAPLLSAGIFGADPIHSLRVCVDTVRTNVYLAVFDKNLYDKLVSSFLEMKSEKQVE
EPI_ISL_424359   323 HEVLLAPLLSAGIFGADPIHSLRVCVDTVRTNVYLAVFDKNLYDKLVSSFLEMKSEKQVE
EPI_ISL_424396   323 HEVLLAPLLSAGIFGADPIHSLRVCVDTVRTNVYLAVFDKNLYDKLVSSFLEMKSEKQVE
EPI_ISL_424399   323 HEVLLAPLLSAGIFGADPIHSLRVCVDTVRTNVYLAVFDKNLYDKLVSSFLEMKSEKQVE
EPI_ISL_425530   323 HEVLLAPLLSAGIFGADPIHSLRVCVDTVRTNVYLAVFDKNLYDKLVSSFLEMKSEKQVE
EPI_ISL_425561   323 HEVLLAPLLSAGIFGADPIHSLRVCVDTVRTNVYLAVFDKNLYDKLVSSFLEMKSEKQVE
EPI_ISL_425614   323 HEVLLAPLLSAGIFGADPIHSLRVCVDTVRTNVYLAVFDKNLYDKLVSSFLEMKSEKQVE
EPI_ISL_425635   323 HEVLLAPLLSAGIFGADPIHSLRVCVDTVRTNVYLAVFDKNLYDKLVSSFLEMKSEKQVE
EPI_ISL_426848   323 HEVLLAPLLSAGIFGADPIHSLRVCVDTVRTNVYLAVFDKNLYDKLVSSFLEMKSEKQVE
EPI_ISL_426850   323 HEVLLAPLLSAGIFGADPIHSLRVCVDTVRTNVYLAVFDKNLYDKLVSSFLEMKSEKQVE
EPI_ISL_426955   323 HEVLLAPLLSAGIFGADPIHSLRVCVDTVRTNVYLAVFDKNLYDKLVSSFLEMKSEKQVE
EPI_ISL_426963   323 HEVLLAPLLSAGIFGADPIHSLRVCVDTVRTNVYLAVFDKNLYDKLVSSFLEMKSEKQVE
```

```
ORF1ab          1201 QKIAEIPKEEVKPFITESKPSVEQRKQDDKKIKACVEEVTTTLEETKFLTENLLLYIDIN
EPI_ISL_411066   383 QKIAEIPKEEVKPFITESKPSVEQRKQDDKKIKACVEEVTTTLEETKFLTENLLLYIDIN
EPI_ISL_413522   383 QKIAEIPKEEVKPFITESKPSVEQRKQDDKKIKACVEEVTTTLEETKFLTENLLLYIDIN
EPI_ISL_413558   383 QKIAEIPKEEVKPFITESKPSVEQRKQDDKKIKACVEEVTTTLEETKFLTENLLLYIDIN
EPI_ISL_413559   383 QKIAEIPKEEVKPFITESKPSVEQRKQDDKKIKACVEEVTTTLEETKFLTENLLLYIDIN
EPI_ISL_413561   383 QKIAEIPKEEVKPFITESKPSVEQRKQDDKKIKACVEEVTTTLEETKFLTENLLLYIDIN
EPI_ISL_413692   383 QKIAEIPKEEVKPFITESKPSVEQRKQDDKKIKACVEEVTTTLEETKFLTENLLLYIDIN
EPI_ISL_413693   383 QKIAEIPKEEVKPFITESKPSVEQRKQDDKKIKACVEEVTTTLEETKFLTENLLLYIDIN
EPI_ISL_413694   383 QKIAEIPKEEVKPFITESKPSVEQRKQDDKKIKACVEEVTTTLEETKFLTENLLLYIDIN
EPI_ISL_414692   383 QKIAEIPKEEVKPFITESKPSVEQRKQDDKKIKACVEEVTTTLEETKFLTENLLLYIDIN
EPI_ISL_424359   383 QKIAEIPKEEVKPFITESKPSVEQRKQDDKKIKACVEEVTTTLEETKFLTENLLLYIDIN
EPI_ISL_424396   383 QKIAEIPKEEVKPFITESKPSVEQRKQDDKKIKACVEEVTTTLEETKFLTENLLLYIDIN
EPI_ISL_424399   383 QKIAEIPKEEVKPFITESKPSVEQRKQDDKKIKACVEEVTTTLEETKFLTENLLLYIDIN
EPI_ISL_425530   383 QKIAEIPKEEVKPFITESKPSVEQRKQDDKKIKACVEEVTTTLEETKFLTENLLLYIDIN
EPI_ISL_425561   383 QKIAEIPKEEVKPFITESKPSVEQRKQDDKKIKACVEEVTTTLEETKFLTENLLLYIDIN
EPI_ISL_425614   383 QKIAEIPKEEVKPFITESKPSVEQRKQDDKKIKACVEEVTTTLEETKFLTENLLLYIDIN
EPI_ISL_425635   383 QKIAEIPKEEVKPFITESKPSVEQRKQDDKKIKACVEEVTTTLEETKFLTENLLLYIDIN
EPI_ISL_426848   383 QKIAEIPKEEVKPFITESKPSVEQRKQDDKKIKACVEEVTTTLEETKFLTENLLLYIDIN
EPI_ISL_426850   383 QKIAEIPKEEVKPFITESKPSVEQRKQDDKKIKACVEEVTTTLEETKFLTENLLLYIDIN
EPI_ISL_426955   383 QKIAEIPKEEVKPFITESKPSVEQRKQDDKKIKACVEEVTTTLEETKFLTENLLLYIDIN
```

EPI_ISL_426963 383 QKIAEIPKEEVKPFITESKPSVEQRKQDDKKIKACVEEVTTTLEETKFLTENLLLYIDIN

```
ORF1ab          1261 GNLHPDSATLVSDIDITFLKKDAPYIVGDVVQEGVLTAVVIPTKKAGGTTEMLAKALRKV
EPI_ISL_411066   443 GNLHPDSATLVSDIDITFLKKDAPYIVGDVVQEGVLTAVVIPTKKAGGTTEMLAKALRKV
EPI_ISL_413522   443 GNLHPDSATLVSDIDITFLKKDAPYIVGDVVQEGVLTAVVIPTKKAGGTTEMLAKALRKV
EPI_ISL_413558   443 GNLHPDSATLVSDIDITFLKKDAPYIVGDVVQEGVLTAVVIPTKKAGGTTEMLAKALRKV
EPI_ISL_413559   443 GNLHPDSATLVSDIDITFLKKDAPYIVGDVVQEGVLTAVVIPTKKAGGTTEMLAKALRKV
EPI_ISL_413561   443 GNLHPDSATLVSDIDITFLKKDAPYIVGDVVQEGVLTAVVIPTKKAGGTTEMLAKALRKV
EPI_ISL_413692   443 GNLHPDSATLVSDIDITFLKKDAPYIVGDVVQEGVLTAVVIPTKKAGGTTEMLAKALRKV
EPI_ISL_413693   443 GNLHPDSATLVSDIDITFLKKDAPYIVGDVVQEGVLTAVVIPTKKAGGTTEMLAKALRKV
EPI_ISL_413694   443 GNLHPDSATLVSDIDITFLKKDAPYIVGDVVQEGVLTAVVIPTKKAGGTTEMLAKALRKV
EPI_ISL_414692   443 GNLHPDSATLVSDIDITFLKKDAPYIVGDVVQEGVLTAVVIPTKKAGGTTEMLAKALRKV
EPI_ISL_424359   443 GNLHPDSATLVSDIDITFLKKDAPYIVGDVVQEGVLTAVVIPTKKAGGTTEMLAKALRKV
EPI_ISL_424396   443 GNLHPDSATLVSDIDITFLKKDAPYIVGDVVQEGVLTAVVIPTKKAGGTTEMLAKALRKV
EPI_ISL_424399   443 GNLHPDSATLVSDIDITFLKKDAPYIVGDVVQEGVLTAVVIPTKKAGGTTEMLAKALRKV
EPI_ISL_425530   443 GNLHPDSATLVSDIDITFLKKDAPYIVGDVVQEGVLTAVVIPTKKAGGTTEMLAKALRKV
EPI_ISL_425561   443 GNLHPDSATLVSDIDITFLKKDAPYIVGDVVREGVLTAVVIPTKKAGGTTEMLAKALRKV
EPI_ISL_425614   443 GNLHPDSATLVSDIDITFLKKDAPYIVGDVVQEGVLTAVVIPTKKAGGTTEMLAKALRKV
EPI_ISL_425635   443 GNLHPDSATLVSDIDITFLKKDAPYIVGDVVQEGVLTAVVIPTKKAGGTTEMLAKALRKV
EPI_ISL_426848   443 GNLHPDSATLVSDIDITFLKKDAPYIVGDVVQEGVLTAVVIPTKKAGGTTEMLAKALRKV
EPI_ISL_426850   443 GNLHPDSATLVSDIDITFLKKDAPYIVGDVVQEGVLTAVVIPTKKAGGTTEMLAKALRKV
EPI_ISL_426955   443 GNLHPDSATLVSDIDITFLKKDAPYIVGDVVQEGVLTAVVIPTKKAGGTTEMLAKALRKV
EPI_ISL_426963   443 GNLHPDSATLVSDIDITFLKKDAPYIVGDVVQEGVLTAVVIPTKKAGGTTEMLAKALRKV
```

```
ORF1ab          1321 PTDNYITTYPGQGLNGYTVEEAKTVLKKCKSAFYILPSIISNEKQEILGTVSWNLREMLA
EPI_ISL_411066   503 PTDNYITTYPGQGLNGYTVEEAKTVLKKCKSAFYILPSIISNEKQEILGTVSWNLREMLA
EPI_ISL_413522   503 PTDNYITTYPGQGLNGYTVEEAKTVLKKCKSAFYILPSIISNEKQEILGTVSWNLREMLA
EPI_ISL_413558   503 PTDNYITTYPGQGLNGYTVEEAKTVLKKCKSAFYILPSIISNEKQEILGTVSWNLREMLA
EPI_ISL_413559   503 PTDNYITTYPGQGLNGYTVEEAKTVLKKCKSAFYILPSIISNEKQEILGTVSWNLREMLA
EPI_ISL_413561   503 PTDNYITTYPGQGLNGYTVEEAKTVLKKCKSAFYILPSIISNEKQEILGTVSWNLREMLA
EPI_ISL_413692   503 PTDNYITTYPGQGLNGYTVEEAKTVLKKCKSAFYILPSIISNEKQEILGTVSWNLREMLA
EPI_ISL_413693   503 PTDNYITTYPGQGLNGYTVEEAKTVLKKCKSAFYILPSIISNEKQEILGTVSWNLREMLA
EPI_ISL_413694   503 PTDNYITTYPGQGLNGYTVEEAKTVLKKCKSAFYILPSIISNEKQEILGTVSWNLREMLA
EPI_ISL_414692   503 PTDNYITTYPGQGLNGYTVEEAKTVLKKCKSAFYILPSIISNEKQEILGTVSWNLREMLA
EPI_ISL_424359   503 PTDNYITTYPGQGLNGYTVEEAKTVLKKCKSAFYILPSIISNEKQEILGTVSWNLREMLA
EPI_ISL_424396   503 PTDNYITTYPGQGLNGYTVEEAKTVLKKCKSAFYILPSIISNEKQEILGTVSWNLREMLA
EPI_ISL_424399   503 PTDNYITTYPGQGLNGYTVEEAKTVLKKCKSAFYILPSIISNEKQEILGTVSWNLREMLA
EPI_ISL_425530   503 PTDNYITTYPGQGLNGYTVEEAKTVLKKCKSAFYILPSIISNEKQEILGTVSWNLREMLA
EPI_ISL_425561   503 PTDNYITTYPGQGLNGYTVEEAKTVLKKCKSAFYILPSIISNEKQEILGTVSWNLREMLA
EPI_ISL_425614   503 PTDNYITTYPGQGLNGYTVEEAKTVLKKCKSAFYILPSIISNEKQEILGTVSWNLREMLA
EPI_ISL_425635   503 PTDNYITTYPGQGLNGYTVEEAKTVLKKCKSAFYILPSIISNEKQEILGTVSWNLREMLA
EPI_ISL_426848   503 PTDNYITTYPGQGLNGYTVEEAKTVLKKCKSAFYILPSIISNEKQEILGTVSWNLREMLA
EPI_ISL_426850   503 PTDNYITTYPGQGLNGYTVEEAKTVLKKCKSAFYILPSIISNEKQEILGTVSWNLREMLA
EPI_ISL_426955   503 PTDNYITTYPGQGLNGYTVEEAKTVLKKCKSAFYILPSIISNEKQEILGTVSWNLREMLA
EPI_ISL_426963   503 PTDNYITTYPGQGLNGYTVEEAKTVLKKCKSAFYILPSIISNEKQEILGTVSWNLREMLA
```

```
ORF1ab          1381 HAEETRKLMPVCVETKAIVSTIQRKYKGIKIQEGVVDYGARFYFYTSKTTVASLINTLND
EPI_ISL_411066   563 HAEETRKLMPVCVETKAIVSTIQRKYKGIKIQEGVVDYGARFYFYTSKTTVASLINTLND
EPI_ISL_413522   563 HAEETRKLMPVCVETKAIVSTIQRKYKGIKIQEGVVDYGARFYFYTSKTTVASLINTLND
EPI_ISL_413558   563 HAEETRKLMPVCVETKAIVSTIQRKYKGIKIQEGVVDYGARFYFYTSKTTVASLINTLND
EPI_ISL_413559   563 HAEETRKLMPVCVETKAIVSTIQRKYKGIKIQEGVVDYGARFYFYTSKTTVASLINTLND
EPI_ISL_413561   563 HAEETRKLMPVCVETKAIVSTIQRKYKGIKIQEGVVDYGARFYFYTSKTTVASLINTLND
EPI_ISL_413692   563 HAEETRKLMPVCVETKAIVSTIQRKYKGIKIQEGVVDYGARFYFYTSKTTVASLINTLND
EPI_ISL_413693   563 HAEETRKLMPVCVETKAIVSTIQRKYKGIKIQEGVVDYGARFYFYTSKTTVASLINTLND
EPI_ISL_413694   563 HAEETRKLMPVCVETKAIVSTIQRKYKGIKIQEGVVDYGARFYFYTSKTTVASLINTLND
EPI_ISL_414692   563 HAEETRKLMPVCVETKAIVSTIQRKYKGIKIQEGVVDYGARFYFYTSKTTVASLINTLND
EPI_ISL_424359   563 HAEETRKLMPVCVETKAIVSTIQRKYKGIKIQEGVVDYGARFYFYTSKTTVASLINTLND
EPI_ISL_424396   563 HAEETRKLMPVCVETKAIVSTIQRKYKGIKIQEGVVDYGARFYFYTSKTTVASLINTLND
EPI_ISL_424399   563 HAEETRKLMPVCVETKAIVSTIQRKYKGIKIQEGVVDYGARFYFYTSKTTVASLINTLND
EPI_ISL_425530   563 HAEETRKLMPVCVETKAIVSTIQRKYKGIKIQEGVVDYGARFYFYTSKTTVASLINTLND
EPI_ISL_425561   563 HAEETRKLMPVCVETKAIVSTIQRKYKGIKIQEGVVDYGARFYFYTSKTTVASLINTLND
EPI_ISL_425614   563 HAEETRKLMPVCVETKAIVSTIQRKYKGIKIQEGVVDYGARFYFYTSKTTVASLINTLND
EPI_ISL_425635   563 HAEETRKLMPVCVETKAIVSTIQRKYKGIKIQEGVVDYGARFYFYTSKTTVASLINTLND
EPI_ISL_426848   563 HAEETRKLMPVCVETKAIVSTIQRKYKGIKIQEGVVDYGARFYFYTSKTTVASLINTLND
EPI_ISL_426850   563 HAEETRKLMPVCVETKAIVSTIQRKYKGIKIQEGVVDYGARFYFYTSKTTVASLINTLND
EPI_ISL_426955   563 HAEETRKLMPVCVETKAIVSTIQRKYKGIKIQEGVVDYGARFYFYTSKTTVASLINTLND
```

EPI_ISL_426963 563 HAEETRKLMPVCVETKAIVSTIQRKYKGIKIQEGVVDYGARFYFYTSKTTVASLINTLND

```
ORF1ab          1441 LNETLVTMPLGYVTHGLNLEEAARYMRSLKVPATVSVSSPDAVTAYNGYLTSSSKTPEEH
EPI_ISL_411066   623 LNETLVTMPLGYVTHGLNLEEAARYMRSLKVPATVSVSSPDAVTAYNGYLTSSSKTPEEH
EPI_ISL_413522   623 LNETLVTMPLGYVTHGLNLEEAARYMRSLKVPATVSVSSPDAVTAYNGYLTSSSKTPEEH
EPI_ISL_413558   623 LNETLVTMPLGYVTHGLNLEEAARYMRSLKVPATVSVSSPDAVTAYNGYLTSSSKTPEEH
EPI_ISL_413559   623 LNETLVTMPLGYVTHGLNLEEAARYMRSLKVPATVSVSSPDAVTAYNGYLTSSSKTPEEH
EPI_ISL_413561   623 LNETLVTMPLGYVTHGLNLEEAARYMRSLKVPATVSVSSPDAVTAYNGYLTSSSKTPEEH
EPI_ISL_413692   623 LNETLVTMPLGYVTHGLNLEEAARYMRSLKVPATVSVSSPDAVTAYNGYLTSSSKTPEEH
EPI_ISL_413693   623 LNETLVTMPLGYVTHGLNLEEAARYMRSLKVPATVSVSSPDAVTAYNGYLTSSSKTPEEH
EPI_ISL_413694   623 LNETLVTMPLGYVTHGLNLEEAARYMRSLKVPATVSVSSPDAVTAYNGYLTSSSKTPEEH
EPI_ISL_414692   623 LNETLVTMPLGYVTHGLNLEEAARYMRSLKVPATVSVSSPDAVTAYNGYLTSSSKTPEEH
EPI_ISL_424359   623 LNETLVTMPLGYVTHGLNLEEAARYMRSLKVPATVSVSSPDAVTAYNGYLTSSSKTPEEH
EPI_ISL_424396   623 LNETLVTMPLGYVTHGLNLEEAARYMRSLKVPATVSVSSPDAVTAYNGYLTSSSKTPEEH
EPI_ISL_424399   623 LNETLVTMPLGYVTHGLNLEEAARYMRSLKVPATVSVSSPDAVTAYNGYLTSSSKTPEEH
EPI_ISL_425530   623 LNETLVTMPLGYVTHGLNLEEAARYMRSLKVPATVSVSSPDAVTAYNGYLTSSSKTPEEH
EPI_ISL_425561   623 LNETLVTMPLGYVTHGLNLEEAARYMRSLKVPATVSVSSPDAVTAYNGYLTSSSKTPEEH
EPI_ISL_425614   623 LNETLVTMPLGYVTHGLNLEEAARYMRFLKVPATVSVSSPDAVTAYNGYLTSSSKTPEEH
EPI_ISL_425635   623 LNETLVTMPLGYVTHGLNLEEAARYMRSLKVPATVSVSSPDAVTAYNGYLTSSSKTPEEH
EPI_ISL_426848   623 LNETLVTMPLGYVTHGLNLEEAARYMRSLKVPATVSVSSPDAVTAYNGYLTSSSKTPEEH
EPI_ISL_426850   623 LNETLVTMPLGYVTHGLNLEEAARYMRSLKVPATVSVSSPDAVTAYNGYLTSSSKTPEEH
EPI_ISL_426955   623 LNETLVTMPLGYVTHGLNLEEAARYMRSLKVPATVSVSSPDAVTAYNGYLTSSSKTPEEH
EPI_ISL_426963   623 LNETLVTMPLGYVTHGLNLEEAARYMRSLKVPATVSVSSPDAVTAYNGYLTSSSKTPEEH

ORF1ab          1501 FIETISLAGSYKDWSYSGQSTQLGIEFLKRGDKSVYYTSNPTTFHLDGEVITFDNLKTLL
EPI_ISL_411066   683 FIETISLAGSYKDWSYSGQSTQLGIEFLKRGDKSVYYTSNPTTFHLDGEVITFDNLKTLL
EPI_ISL_413522   683 FIETISLAGSYKDWSYSGQSTQLGIEFLKRGDKSVYYTSNPTTFHLDGEVITFDNLKTLL
EPI_ISL_413558   683 FIETISLAGSYKDWSYSGQSTQLGIEFLKRGDKSVYYTSNPTTFHLDGEVITFDNLKTLL
EPI_ISL_413559   683 FIETISLAGSYKDWSYSGQSTQLGIEFLKRGDKSVYYTSNPTTFHLDGEVITFDNLKTLL
EPI_ISL_413561   683 FIETISLAGSYKDWSYSGQSTQLGIEFLKRGDKSVYYTSNPTTFHLDGEVITFDNLKTLL
EPI_ISL_413692   683 FIETISLAGSYKDWSYSGQSTQLGIEFLKRGDKSVYYTSNPTTFHLDGEVITFDNLKTLL
EPI_ISL_413693   683 FIETISLAGSYKDWSYSGQSTQLGIEFLKRGDKSVYYTSNPTTFHLDGEVITFDNLKTLL
EPI_ISL_413694   683 FIETISLAGSYKDWSYSGQSTQLGIEFLKRGDKSVYYTSNPTTFHLDGEVITFDNLKTLL
EPI_ISL_414692   683 FIETISLAGSYKDWSYSGQSTQLGIEFLKRGDKSVYYTSNPTTFHLDGEVITFDNLKTLL
EPI_ISL_424359   683 FIETISLAGSYKDWSYSGQSTQLGIEFLKRGDKSVYYTSNPTTFHLDGEVITFDNLKTLL
EPI_ISL_424396   683 FIETISLAGSYKDWSYSGQSTQLGIEFLKRGDKSVYYTSNPTTFHLDGEVITFDNLKTLL
EPI_ISL_424399   683 FIETISLAGSYKDWSYSGQSTQLGIEFLKRGDKSVYYTSNPTTFHLDGEVITFDNLKTLL
EPI_ISL_425530   683 FIETISLAGSYKDWSYSGQSTQLGIEFLKRGDKSVYYTSNPTTFHLDGEVITFDNLKTLL
EPI_ISL_425561   683 FIETISLAGSYKDWSYSGQSTQLGIEFLKRGDKSVYYTSNPTTFHLDGEVITFDNLKTLL
EPI_ISL_425614   683 FIETISLAGSYKDWSYSGQSTQLGIEFLKRGDKSVYYTSNPTTFHLDGEVITFDNLKTLL
EPI_ISL_425635   683 FIETISLAGSYKDWSYSGQSTQLGIEFLKRGDKSVYYTSNPTTFHLDGEVITFDNLKTLL
EPI_ISL_426848   683 FIETISLAGSYKDWSYSGQSTQLGIEFLKRGDKSVYYTSNPTTFHLDGEVITFDNLKTLL
EPI_ISL_426850   683 FIETISLAGSYKDWSYSGQSTQLGIEFLKRGDKSVYYTSNPTTFHLDGEVITFDNLKTLL
EPI_ISL_426955   683 FIETISLAGSYKDWSYSGQSTQLGIEFLKRGDKSVYYTSNPTTFHLDGEVITFDNLKTLL
EPI_ISL_426963   683 FIETISLAGSYKDWSYSGQSTQLGIEFLKRGDKSVYYTSNPTTFHLDGEVITFDNLKTLL

ORF1ab          1561 SLREVRTIKVFTTVDNINLHTQVVDMSMTYGQQFGPTYLDGADVTKIKPHNSHEGKTFYV
EPI_ISL_411066   743 SLREVRTIKVFTTVDNINLHTQVVDMSMTYGQQFGPTYLDGADVTKIKPHNSHEGKTFYV
EPI_ISL_413522   743 SLREVRTIKVFTTVDNINLHTQVVDMSMTYGQQFGPTYLDGADVTKIKPHNSHEGKTFYV
EPI_ISL_413558   743 SLREVRTIKVFTTVDNINLHTQVVDMSMTYGQQFGPTYLDGADVTKIKPHNSHEGKTFYV
EPI_ISL_413559   743 SLREVRTIKVFTTVDNINLHTQVVDMSMTYGQQFGPTYLDGADVTKIKPHNSHEGKTFYV
EPI_ISL_413561   743 SLREVRTIKVFTTVDNINLHTQVVDMSMTYGQQFGPTYLDGADVTKIKPHNSHEGKTFYV
EPI_ISL_413692   743 SLREVRTIKVFTTVDNINLHTQVVDMSMTYGQQFGPTYLDGADVTKIKPHNSHEGKTFYV
EPI_ISL_413693   743 SLREVRTIKVFTTVDNINLHTQVVDMSMTYGQQFGPTYLDGADVTKIKPHNSHEGKTFYV
EPI_ISL_413694   743 SLREVRTIKVFTTVDNINLHTQVVDMSMTYGQQFGPTYLDGADVTKIKPHNSHEGKTFYV
EPI_ISL_414692   743 SLREVRTIKVFTTVDNINLHTQVVDMSMTYGQQFGPTYLDGADVTKIKPHNSHEGKTFYV
EPI_ISL_424359   743 SLREVRTIKVFTTVDNINLHTQVVDMSMTYGQQFGPTYLDGADVTKIKPHNSHEGKTFYV
EPI_ISL_424396   743 SLREVRTIKVFTTVDNINLHTQVVDMSMTYGQQFGPTYLDGADVTKIKPHNSHEGKTFYV
EPI_ISL_424399   743 SLREVRTIKVFTTVDNINLHTQVVDMSMTYGQQFGPTYLDGADVTKIKPHNSHEGKTFYV
EPI_ISL_425530   743 SLREVRTIKVFTTVDNINLHTQVVDMSMTYGQQFGPTYLDGADVTKIKPHNSHEGKTFYV
EPI_ISL_425561   743 SLREVRTIKVFTTVDNINLHTQVVDMSMTYGQQFGPTYLDGADVTKIKPHNSHEGKTFYV
EPI_ISL_425614   743 SLREVRTIKVFTTVDNINLHTQVVDMSMTYGQQFGPTYLDGADVTKIKPHNSHEGKTFYV
EPI_ISL_425635   743 SLREVRTIKVFTTVDNINLHTQVVDMSMTYGQQFGPTYLDGADVTKIKPHNSHEGKTFYV
EPI_ISL_426848   743 SLREVRTIKVFTTVDNINLHTQVVDMSMTYGQQFGPTYLDGADVTKIKPHNSHEGKTFYV
EPI_ISL_426850   743 SLREVRTIKVFTTVDNINLHTQVVDMSMTYGQQFGPTYLDGADVTKIKPHNSHEGKTFYV
EPI_ISL_426955   743 SLREVRTIKVFTTVDNINLHTQVVDMSMTYGQQFGPTYLDGADVTKIKPHNSHEGKTFYV
```

EPI_ISL_426963 743 SLREVRTIKVFTTVDNINLHTQVVDMSMTYGQQFGPTYLDGADVTKIKPHNSHEGKTFYV

```
ORF1ab          1621 LPNDDTLRVEAFEYYHTTDPSFLGRYMSALNHTKKWKYPQVNGLTSIKWADNNCYLATAL
EPI_ISL_411066   803 LPNDDTLRVEAFEYYHTTDPSFLGRYMSALNHTKKWKYPQVNGLTSIKWADNNCYLATAL
EPI_ISL_413522   803 LPNDDTLRVEAFEYYHTTDPSFLGRYMSALNHTKKWKYPQVNGLTSIKWADNNCYLATAL
EPI_ISL_413558   803 LPNDDTLRVEAFEYYHTTDPSFLGRYMSALNHTKKWKYPQVNGLTSIKWADNNCYLATAL
EPI_ISL_413559   803 LPNDDTLRVEAFEYYHTTDPSFLGRYMSALNHTKKWKYPQVNGLTSIKWADNNCYLATAL
EPI_ISL_413561   803 LPNDDTLRVEAFEYYHTTDPSFLGRYMSALNHTKKWKYPQVNGLTSIKWADNNCYLATAL
EPI_ISL_413692   803 LPNDDTLRVEAFEYYHTTDPSFLGRYMSALNHTKKWKYPQVNGLTSIKWADNNCYLATAL
EPI_ISL_413693   803 LPNDDTLRVEAFEYYHTTDPSFLGRYMSALNHTKKWKYPQVNGLTSIKWADNNCYLATAL
EPI_ISL_413694   803 LPNDDTLRVEAFEYYHTTDPSFLGRYMSALNHTKKWKYPQVNGLTSIKWADNNCYLATAL
EPI_ISL_414692   803 LPNDDTLRVEAFEYYHTTDPSFLGRYMSALNHTKKWKYPQVNGLTSIKWADNNCYLATAL
EPI_ISL_424359   803 LPNDDTLRVEAFEYYHTTDPSFLGRYMSALNHTKKWKYPQVNGLTSIKWADNNCYLATAL
EPI_ISL_424396   803 LPNDDTLRVEAFEYYHTTDPSFLGRYMSALNHTKKWKYPQVNGLTSIKWADNNCYLATAL
EPI_ISL_424399   803 LPNDDTLRVEAFEYYHTTDPSFLGRYMSALNHTKKWKYPQVNGLTSIKWADNNCYLATAL
EPI_ISL_425530   803 LPNDDTLRVEAFEYYHTTDPSFLGRYMSALNHTKKWKYPQVNGLTSIKWADNNCYLATAL
EPI_ISL_425561   803 LPNDDTLRVEAFEYYHTTDPSFLGRYMSALNHTKKWKYPQVNGLTSIKWADNNCYLATAL
EPI_ISL_425614   803 LPNDDTLRVEAFEYYHTTDPSFLGRYMSALNHTKKWKYPQVNGLTSIKWADNNCYLATAL
EPI_ISL_425635   803 LPNDDTLRVEAFEYYHTTDPSFLGRYMSALNHTKKWKYPQVNGLTSIKWADNNCYLATAL
EPI_ISL_426848   803 LPNDDTLRVEAFEYYHTTDPSFLGRYMSALNHTKKWKYPQVNGLTSIKWADNNCYLATAL
EPI_ISL_426850   803 LPNDDTLRVEAFEYYHTTDPSFLGRYMSALNHTKKWKYPQVNGLTSIKWADNNCYLATAL
EPI_ISL_426955   803 LPNDDTLRVEAFEYYHTTDPSFLGRYMSALNHTKKWKYPQVNGLTSIKWADNNCYLATAL
EPI_ISL_426963   803 LPNDDTLRVEAFEYYHTTDPSFLGRYMSALNHTKKWKYPQVNGLTSIKWADNNCYLATAL

ORF1ab          1681 LTLQQIELKFNPPALQDAYYRARAGEAANFCALILAYCNKTVGELGDVRETMSYLFQHAN
EPI_ISL_411066   863 LTLQQIELKFNPPALQDAYYRARAGEAANFCALILAYCNKTVGELGDVRETMSYLFQHAN
EPI_ISL_413522   863 LTLQQIELKFNPPALQDAYYRARAGEAANFCALILAYCNKTVGELGDVRETMSYLFQHAN
EPI_ISL_413558   863 LTLQQIELKFNPPALQDAYYRARAGEAANFCALILAYCNKTVGELGDVRETMSYLFQHAN
EPI_ISL_413559   863 LTLQQIELKFNPPALQDAYYRARAGEAANFCALILAYCNKTVGELGDVRETMSYLFQHAN
EPI_ISL_413561   863 LTLQQIELKFNPPALQDAYYRARAGEAANFCALILAYCNKTVGELGDVRETMSYLFQHAN
EPI_ISL_413692   863 LTLQQIELKFNPPALQDAYYRARAGEAANFCALILAYCNKTVGELGDVRETMSYLFQHAN
EPI_ISL_413693   863 LTLQQIELKFNPPALQDAYYRARAGEAANFCALILAYCNKTVGELGDVRETMSYLFQHAN
EPI_ISL_413694   863 LTLQQIELKFNPPALQDAYYRARAGEAANFCALILAYCNKTVGELGDVRETMSYLFQHAN
EPI_ISL_414692   863 LTLQQIELKFNPPALQDAYYRARAGEAANFCALILAYCNKTVGELGDVRETMSYLFQHAN
EPI_ISL_424359   863 LTLQQIELKFNPPALQDAYYRARAGEAANFCALILAYCNKTVGELGDVRETMSYLFQHAN
EPI_ISL_424396   863 LTLQQIELKFNPPALQDAYYRARAGEAANFCALILAYCNKTVGELGDVRETMSYLFQHAN
EPI_ISL_424399   863 LTLQQIELKFNPPALQDAYYRARAGEAANFCALILAYCNKTVGELGDVRETMSYLFQHAN
EPI_ISL_425530   863 LTLQQIELKFNPPALQDAYYRARAGEAANFCALILAYCNKTVGELGDVRETMSYLFQHAN
EPI_ISL_425561   863 LTLQQIELKFNPPALQDAYYRARAGEAANFCALILAYCNKTVGELGDVRETMSYLFQHAN
EPI_ISL_425614   863 LTLQQIELKFNPPALQDAYYRARAGEAANFCALILAYCNKTVGELGDVRETMSYLFQHAN
EPI_ISL_425635   863 LTLQQIELKFNPPALQDAYYRARAGEAANFCALILAYCNKTVGELGDVRETMSYLFQHAN
EPI_ISL_426848   863 LTLQQIELKFNPPALQDAYYRARAGEAANFCALILAYCNKTVGELGDVRETMSYLFQHAN
EPI_ISL_426850   863 LTLQQIELKFNPPALQDAYYRARAGEAANFCALILAYCNKTVGELGDVRETMSYLFQHAN
EPI_ISL_426955   863 LTLQQIELKFNPPALQDAYYRARAGEAANFCALILAYCNKTVGELGDVRETMSYLFQHAN
EPI_ISL_426963   863 LTLQQIELKFNPPALQDAYYRARAGEAANFCALILAYCNKTVGELGDVRETMSYLFQHAN

ORF1ab          1741 LDSCKRVLNVVCKTCGQQQTTLKGVEAVMYMGTLSYEQFKKGVQIPCTCGKQATKYLVQQ
EPI_ISL_411066   923 LDSCKRVLNVVCKTCGQQQTTLKGVEAVMYMGTLSYEQFKKGVQIPCTCGKQATKYLVQQ
EPI_ISL_413522   923 LDSCKRVLNVVCKTCGQQQTTLKGVEAVMYMGTLSYEQFKKGVQIPCTCGKQATKYLVQQ
EPI_ISL_413558   923 LDSCKRVLNVVCKTCGQQQTTLKGVEAVMYMGTLSYEQFKKGVQIPCTCGKQATKYLVQQ
EPI_ISL_413559   923 LDSCKRVLNVVCKTCGQQQTTLKGVEAVMYMGTLSYEQFKKGVQIPCTCGKQATKYLVQQ
EPI_ISL_413561   923 LDSCKRVLNVVCKTCGQQQTTLKGVEAVMYMGTLSYEQFKKGVQIPCTCGKQATKYLVQQ
EPI_ISL_413692   923 LDSCKRVLNVVCKTCGQQQTTLKGVEAVMYMGTLSYEQFKKGVQIPCTCGKQATKYLVQQ
EPI_ISL_413693   923 LDSCKRVLNVVCKTCGQQQTTLKGVEAVMYMGTLSYEQFKKGVQIPCTCGKQATKYLVQQ
EPI_ISL_413694   923 LDSCKRVLNVVCKTCGQQQTTLKGVEAVMYMGTLSYEQFKKGVQIPCTCGKQATKYLVQQ
EPI_ISL_414692   923 LDSCKRVLNVVCKTCGQQQTTLKGVEAVMYMGTLSYEQFKKGVQIPCTCGKQATKYLVQQ
EPI_ISL_424359   923 LDSCKRVLNVVCKTCGQQQTTLKGVEAVMYMGTLSYEQFKKGVQIPCTCGKQATKYLVQQ
EPI_ISL_424396   923 LDSCKRVLNVVCKTCGQQQTTLKGVEAVMYMGTLSYEQFKKGVQIPCTCGKQATKYLVQQ
EPI_ISL_424399   923 LDSCKRVLNVVCKTCGQQQTTLKGVEAVMYMGTLSYEQFKKGVQIPCTCGKQATKYLVQQ
EPI_ISL_425530   923 LDSCKRVLNVVCKTCGQQQTTLKGVEAVMYMGTLSYEQFKKGVQIPCTCGKQATKYLVQQ
EPI_ISL_425561   923 LDSCKRVLNVVCKTCGQQQTTLKGVEAVMYMGTLSYEQFKKGVQIPCTCGKQATKYLVQQ
EPI_ISL_425614   923 LDSCKRVLNVVCKTCGQQQTTLKGVEAVMYMGTLSYEQFKKGVQIPCTCGKQATKYLVQQ
EPI_ISL_425635   923 LDSCKRVLNVVCKTCGQQQTTLKGVEAVMYMGTLSYEQFKKGVQIPCTCGKQATKYLVQQ
EPI_ISL_426848   923 LDSCKRVLNVVCKTCGQQQTTLKGVEAVMYMGTLSYEQFKKGVQIPCTCGKQATKYLVQQ
EPI_ISL_426850   923 LDSCKRVLNVVCKTCGQQQTTLKGVEAVMYMGTLSYEQFKKGVQIPCTCGKQATKYLVQQ
EPI_ISL_426955   923 LDSCKRVLNVVCKTCGQQQTTLKGVEAVMYMGTLSYEQFKKGVQIPCTCGKQATKYLVQQ
```

EPI_ISL_426963 923 LDSCKRVLNVVCKTCGQQQTTLKGVEAVMYMGTLSYEQFKKGVQIPCTCGKQATKYLVQQ

```
ORF1ab          1801 ESPFVMMSAPPAQYELKHGTFTCASEYTGNYQCGHYKHITSKETLYCIDGALLTKSSEYK
EPI_ISL_411066   983 ESPFVMMSAPPAQYELKHGTFTCASEYTGNYQCGHYKHITSKETLYCIDGALLTKSSEYK
EPI_ISL_413522   983 ESPFVMMSAPPAQYELKHGTFTCASEYTGNYQCGHYKHITSKETLYCIDGALLTKSSEYK
EPI_ISL_413558   983 ESPFVMMSAPPAQYELKHGTFTCASEYTGNYQCGHYKHITSKETLYCIDGALLTKSSEYK
EPI_ISL_413559   983 ESPFVMMSAPPAQYELKHGTFTCASEYTGNYQCGHYKHITSKETLYCIDGALLTKSSEYK
EPI_ISL_413561   983 ESPFVMMSAPPAQYELKHGTFTCASEYTGNYQCGHYKHITSKETLYCIDGALLTKSSEYK
EPI_ISL_413692   983 ESPFVMMSAPPAQYELKHGTFTCASEYTGNYQCGHYKHITSKETLYCIDGALLTKSSEYK
EPI_ISL_413693   983 ESPFVMMSAPPAQYELKHGTFTCASEYTGNYQCGHYKHITSKETLYCIDGALLTKSSEYK
EPI_ISL_413694   983 ESPFVMMSAPPAQYELKHGTFTCASEYTGNYQCGHYKHITSKETLYCIDGALLTKSSEYK
EPI_ISL_414692   983 ESPFVMMSAPPAQYELKHGTFTCASEYTGNYQCGHYKHITSKETLYCIDGALLTKSSEYK
EPI_ISL_424359   983 ESPFVMMSAPPAQYELKHGTFTCASEYTGNYQCGHYKHITSKETLYCIDGALLTKSSEYK
EPI_ISL_424396   983 ESPFVMMSAPPAQYELKHGTFTCASEYTGNYQCGHYKHITSKETLYCIDGALLTKSSEYK
EPI_ISL_424399   983 ESPFVMMSAPPAQYELKHGTFTCASEYTGNYQCGHYKHITSKETLYCIDGALLTKSSEYK
EPI_ISL_425530   983 ESPFVMMSAPPAQYELKHGTFTCASEYTGNYQCGHYKHITSKETLYCIDGALLTKSSEYK
EPI_ISL_425561   983 ESPFVMMSAPPAQYELKHGTFTCASEYTGNYQCGHYKHITSKETLYCIDGALLTKSSEYK
EPI_ISL_425614   983 ESPFVMMSAPPAQYELKHGTFTCASEYTGNYQCGHYKHITSKETLYCIDGALLTKSSEYK
EPI_ISL_425635   983 ESPFVMMSAPPAQYELKHGTFTCASEYTGNYQCGHYKHITSKETLYCIDGALLTKSSEYK
EPI_ISL_426848   983 ESPFVMMSAPPAQYELKHGTFTCASEYTGNYQCGHYKHITSKETLYCIDGALLTKSSEYK
EPI_ISL_426850   983 ESPFVMMSAPPAQYELKHGTFTCASEYTGNYQCGHYKHITSKETLYCIDGALLTKSSEYK
EPI_ISL_426955   983 ESPFVMMSAPPAQYELKHGTFTCASEYTGNYQCGHYKHITSKETLYCIDGALLTKSSEYK
EPI_ISL_426963   983 ESPFVMMSAPPAQYELKHGTFTCASEYTGNYQCGHYKHITSKETLYCIDGALLTKSSEYK

ORF1ab          1861 GPITDVFYKENSYTTTIKPVTYKLDGVVCTEIDPKLDNYYKKDNSYFTEQPIDLVPNQPY
EPI_ISL_411066  1043 GPITDVFYKENSYTTTIKPVTYKLDGVVCTEIDPKLDNYYKKDNSYFTEQPIDLVPNQPY
EPI_ISL_413522  1043 GPITDVFYKENSYTTTIKPVTYKLDGVVCTEIDPKLDNYYKKDNSYFTEQPIDLVPNQPY
EPI_ISL_413558  1043 GPITDVFYKENSYTTTIKPVTYKLDGVVCTEIDPKLDNYYKKDNSYFTEQPIDLVPNQPY
EPI_ISL_413559  1043 GPITDVFYKENSYTTTIKPVTYKLDGVVCTEIDPKLDNYYKKDNSYFTEQPIDLVPNQPY
EPI_ISL_413561  1043 GPITDVFYKENSYTTTIKPVTYKLDGVVCTEIDPKLDNYYKKDNSYFTEQPIDLVPNQPY
EPI_ISL_413692  1043 GPITDVFYKENSYTTTIKPVTYKLDGVVCTEIDPKLDNYYKKDNSYFTEQPIDLVPNQPY
EPI_ISL_413693  1043 GPITDVFYKENSYTTTIKPVTYKLDGVVCTEIDPKLDNYYKKDNSYFTEQPIDLVPNQPY
EPI_ISL_413694  1043 GPITDVFYKENSYTTTIKPVTYKLDGVVCTEIDPKLDNYYKKDNSYFTEQPIDLVPNQPY
EPI_ISL_414692  1043 GPITDVFYKENSYTTTIKPVTYKLDGVVCTEIDPKLDNYYKKDNSYFTEQPIDLVPNQPY
EPI_ISL_424359  1043 GPITDVFYKENSYTTTIKPVTYKLDGVVCTEIDPKLDNYYKKDNSYFTEQPIDLVPNQPY
EPI_ISL_424396  1043 GPITDVFYKENSYTTTIKPVTYKLDGVVCTEIDPKLDNYYKKDNSYFTEQPIDLVPNQPY
EPI_ISL_424399  1043 GPITDVFYKENSYTTTIKPVTYKLDGVVCTEIDPKLDNYYKKDNSYFTEQPIDLVPNQPY
EPI_ISL_425530  1043 GPITDVFYKENSYTTTIKPVTYKLDGVVCTEIDPKLDNYYKKDNSYFTEQPIDLVPNQPY
EPI_ISL_425561  1043 GPITDVFYKENSYTTTIKPVTYKLDGVVCTEIDPKLDNYYKKDNSYFTEQPIDLVPNQPY
EPI_ISL_425614  1043 GPITDVFYKENSYTTTIKPVTYKLDGVVCTEIDPKLDNYYKKDNSYFTEQPIDLVPNQPY
EPI_ISL_425635  1043 GPITDVFYKENSYTTTIKPVTYKLDGVVCTEIDPKLDNYYKKDNSYFTEQPIDLVPNQPY
EPI_ISL_426848  1043 GPITDVFYKENSYTTTIKPVTYKLDGVVCTEIDPKLDNYYKKDNSYFTEQPIDLVPNQPY
EPI_ISL_426850  1043 GPITDVFYKENSYTTTIKPVTYKLDGVVCTEIDPKLDNYYKKDNSYFTEQPIDLVPNQPY
EPI_ISL_426955  1043 GPITDVFYKENSYTTTIKPVTYKLDGVVCTEIDPKLDNYYKKDNSYFTEQPIDLVPNQPY
EPI_ISL_426963  1043 GPITDVFYKENSYTTTIKPVTYKLDGVVCTEIDPKLDNYYKKDNSYFTEQPIDLVPNQPY

ORF1ab          1921 PNASFDNFKFVCDNIKFADDLNQLTGYKKPASRELKVTFFPDLNGDVVAIDYKHYTPSFK
EPI_ISL_411066  1103 PNASFDNFKFVCDNIKFADDLNQLTGYKKPASRELKVTFFPDLNGDVVAIDYKHYTPSFK
EPI_ISL_413522  1103 PNASFDNFKFVCDNIKFADDLNQLTGYKKPASRELKVTFFPDLNGDVVAIDYKHYTPSFK
EPI_ISL_413558  1103 PNASFDNFKFVCDNIKFADDLNQLTGYKKPASRELKVTFFPDLNGDVVAIDYKHYTPSFK
EPI_ISL_413559  1103 PNASFDNFKFVCDNIKFADDLNQLTGYKKPASRELKVTFFPDLNGDVVAIDYKHYTPSFK
EPI_ISL_413561  1103 PNASFDNFKFVCDNIKFADDLNQLTGYKKPASRELKVTFFPDLNGDVVAIDYKHYTPSFK
EPI_ISL_413692  1103 PNASFDNFKFVCDNIKFADDLNQLTGYKKPASRELKVTFFPDLNGDVVAIDYKHYTPSFK
EPI_ISL_413693  1103 PNASFDNFKFVCDNIKFADDLNQLTGYKKPASRELKVTFFPDLNGDVVAIDYKHYTPSFK
EPI_ISL_413694  1103 PNASFDNFKFVCDNIKFADDLNQLTGYKKPASRELKVTFFPDLNGDVVAIDYKHYTPSFK
EPI_ISL_414692  1103 PNASFDNFKFVCDNIKFADDLNQLTGYKKPASRELKVTFFPDLNGDVVAIDYKHYTPSFK
EPI_ISL_424359  1103 PNASFDNFKFVCDNIKFADDLNQLTGYKKPASRELKVTFFPDLNGDVVAIDYKHYTPSFK
EPI_ISL_424396  1103 PNASFDNFKFVCDNIKFADDLNQLTGYKKPASRELKVTFFPDLNGDVVAIDYKHYTPSFK
EPI_ISL_424399  1103 PNASFDNFKFVCDNIKFADDLNQLTGYKKPASRELKVTFFPDLNGDVVAIDYKHYTPSFK
EPI_ISL_425530  1103 PNASFDNFKFVCDNIKFADDLNQLTGYKKPASRELKVTFFPDLNGDVVAIDYKHYTPSFK
EPI_ISL_425561  1103 PNASFDNFKFVCDNIKFADDLNQLTGYKKPASRELKVTFFPDLNGDVVAIDYKHYTPSFK
EPI_ISL_425614  1103 PNASFDNFKFVCDNIKFADDLNQLTGYKKPASRELKVTFFPDLNGDVVAIDYKHYTPSFK
EPI_ISL_425635  1103 PNASFDNFKFVCDNIKFADDLNQLTGYKKPASRELKVTFFPDLNGDVVAIDYKHYTPSFK
EPI_ISL_426848  1103 PNASFDNFKFVCDNIKFADDLNQLTGYKKPASRELKVTFFPDLNGDVVAIDYKHYTPSFK
EPI_ISL_426850  1103 PNASFDNFKFVCDNIKFADDLNQLTGYKKPASRELKVTFFPDLNGDVVAIDYKHYTPSFK
EPI_ISL_426955  1103 PNASFDNFKFVCDNIKFADDLNQLTGYKKPASRELKVTFFPDLNGDVVAIDYKHYTPSFK
```

EPI_ISL_426963 1103 PNASFDNFKFVCDNIKFADDLNQLTGYKKPASRELKVTFFPDLNGDVVAIDYKHYTPSFK

```
ORF1ab         1981 KGAKLLHKPIVWHVNNATNKATYKPNTWCIRCLWSTKPVETSNSFDVLKSEDAQGMDNLA
EPI_ISL_411066 1163 KGAKLLHKPIVWHVNNATNKATYKPNTWCIRCLWSTKPVETSNSFDVLKSEDAQGMDNLA
EPI_ISL_413522 1163 KGAKLLHKPIVWHVNNATNKATYKPNTWCIRCLWSTKPVETSNSFDVLKSEDAQGMDNLA
EPI_ISL_413558 1163 KGAKLLHKPIVWHVNNATNKATYKPNTWCIRCLWSTKPVETSNSFDVLKSEDAQGMDNLA
EPI_ISL_413559 1163 KGAKLLHKPIVWHVNNATNKATYKPNTWCIRCLWSTKPVETSNSFDVLKSEDAQGMDNLA
EPI_ISL_413561 1163 KGAKLLHKPIVWHVNNATNKATYKPNTWCIRCLWSTKPVETSNSFDVLKSEDAQGMDNLA
EPI_ISL_413692 1163 KGAKLLHKPIVWHVNNATNKATYKPNTWCIRCLWSTKPVETSNSFDVLKSEDAQGMDNLA
EPI_ISL_413693 1163 KGAKLLHKPIVWHVNNATNKATYKPNTWCIRCLWSTKPVETSNSFDVLKSEDAQGMDNLA
EPI_ISL_413694 1163 KGAKLLHKPIVWHVNNATNKATYKPNTWCIRCLWSTKPVETSNSFDVLKSEDAQGMDNLA
EPI_ISL_414692 1163 KGAKLLHKPIVWHVNNATNKATYKPNTWCIRCLWSTKPVETSNSFDVLKSEDAQGMDNLA
EPI_ISL_424359 1163 KGAKLLHKPIVWHVNNATNKATYKPNTWCIRCLWSTKPVETSNSFDVLKSEDAQGMDNLA
EPI_ISL_424396 1163 KGAKLLHKPIVWHVNNATNKATYKPNTWCIRCLWSTKPVETSNSFDVLKSEDAQGMDNLA
EPI_ISL_424399 1163 KGAKLLHKPIVWHVNNATNKATYKPNTWCIRCLWSTKPVETSNSFDVLKSEDAQGMDNLA
EPI_ISL_425530 1163 KGAKLLHKPIVWHVNNATNKATYKPNTWCIRCLWSTKPVETSNSFDVLKSEDAQGMDNLA
EPI_ISL_425561 1163 KGAKLLHKPIVWHVNNATNKATYKPNTWCIRCLWSTKPVETSNSFDVLKSEDAQGMDNLA
EPI_ISL_425614 1163 KGAKLLHKPIVWHVNNATNKATYKPNTWCIRCLWSTKPVETSNSFDVLKSEDAQGMDNLA
EPI_ISL_425635 1163 KGAKLLHKPIVWHVNNATNKATYKPNTWCIRCLWSTKPVETSNSFDVLKSEDAQGMDNLA
EPI_ISL_426848 1163 KGAKLLHKPIVWHVNNATNKATYKPNTWCIRCLWSTKPVETSNSFDVLKSEDAQGMDNLA
EPI_ISL_426850 1163 KGAKLLHKPIVWHVNNATNKATYKPNTWCIRCLWSTKPVETSNSFDVLKSEDAQGMDNLA
EPI_ISL_426955 1163 KGAKLLHKPIVWHVNNATNKATYKPNTWCIRCLWSTKPVETSNSFDVLKSEDAQGMDNLA
EPI_ISL_426963 1163 KGAKLLHKPIVWHVNNATNKATYKPNTWCIRCLWSTKPVETSNSFDVLKSEDAQGMDNLA

ORF1ab         2041 CEDLKPVSEEVVENPTIQKDVLECNVKTTEVVGDIILKPANNSLKITEEVGHTDLMAAYV
EPI_ISL_411066 1223 CEDLKPVSEEVVENPTIQKDVLECNVKTTEVVGDIILKPANNSLKITEEVGHTDLMAAYV
EPI_ISL_413522 1223 CEDLKPVSEEVVENPTIQKDVLECNVKTTEVVGDIILKPANNSLKITEEVGHTDLMAAYV
EPI_ISL_413558 1223 CEDLKPVSEEVVENPTIQKDVLECNVKTTEVVGDIILKPANNSLKITEEVGHTDLMAAYV
EPI_ISL_413559 1223 CEDLKPVSEEVVENPTIQKDVLECNVKTTEVVGDIILKPANNSLKITEEVGHTDLMAAYV
EPI_ISL_413561 1223 CEDLKPVSEEVVENPTIQKDVLECNVKTTEVVGDIILKPANNSLKITEEVGHTDLMAAYV
EPI_ISL_413692 1223 CEDLKPVSEEVVENPTIQKDVLECNVKTTEVVGDIILKPANNSLKITEEVGHTDLMAAYV
EPI_ISL_413693 1223 CEDLKPVSEEVVENPTIQKDVLECNVKTTEVVGDIILKPANNSLKITEEVGHTDLMAAYV
EPI_ISL_413694 1223 CEDLKPVSEEVVENPTIQKDVLECNVKTTEVVGDIILKPANNSLKITEEVGHTDLMAAYV
EPI_ISL_414692 1223 CEDLKPVSEEVVENPTIQKDVLECNVKTTEVVGDIILKPANNSLKITEEVGHTDLMAAYV
EPI_ISL_424359 1223 CEDLKPVSEEVVENPTIQKDVLECNVKTTEVVGDIILKPANNSLKITEEVGHTDLMAAYV
EPI_ISL_424396 1223 CEDLKPVSEEVVENPTIQKDVLECNVKTTEVVGDIILKPANNSLKITEEVGHTDLMAAYV
EPI_ISL_424399 1223 CEDLKPVSEEVVENPTIQKDVLECNVKTTEVVGDIILKPANNSLKITEEVGHTDLMAAYV
EPI_ISL_425530 1223 CEDLKPVSEEVVENPTIQKDVLECNVKTTEVVGDIILKPANNSLKITEEVGHTDLMAAYV
EPI_ISL_425561 1223 CEDLKPVSEEVVENPTIQKDVLECNVKTTEVVGDIILKPANNSLKITEEVGHTDLMAAYV
EPI_ISL_425614 1223 CEDLKPVSEEVVENPTIQKDVLECNVKTTEVVGDIILKPANNSLKITEEVGHTDLMAAYV
EPI_ISL_425635 1223 CEDLKPVSEEVVENPTIQKDVLECNVKTTEVVGDIILKPANNSLKITEEVGHTDLMAAYV
EPI_ISL_426848 1223 CEDLKPVSEEVVENPTIQKDVLECNVKTTEVVGDIILKPANNSLKITEEVGHTDLMAAYV
EPI_ISL_426850 1223 CEDLKPVSEEVVENPTIQKDVLECNVKTTEVVGDIILKPANNSLKITEEVGHTDLMAAYV
EPI_ISL_426955 1223 CEDLKPVSEEVVENPTIQKDVLECNVKTTEVVGDIILKPANNSLKITEEVGHTDLMAAYV
EPI_ISL_426963 1223 CEDLKPVSEEVVENPTIQKDVLECNVKTTEVVGDIILKPANNSLKITEEVGHTDLMAAYV

ORF1ab         2101 DNSSLTIKKPNELSRVLGLKTLATHGLAAVNSVPWDTIANYAKPFLNKVVSTTTNIVTRC
EPI_ISL_411066 1283 DNSSLTIKKPNELSRVLGLKTLATHGLAAVNSVPWDTIANYAKPFLNKVVSTTTNIVTRC
EPI_ISL_413522 1283 DNSSLTIKKPNELSRVLGLKTLATHGLAAVNSVPWDTIANYAKPFLNKVVSTTTNIVTRC
EPI_ISL_413558 1283 DNSSLTIKKPNELSRVLGLKTLATHGLAAVNSVPWDTIANYAKSFLNKVVSTTTNIVTRC
EPI_ISL_413559 1283 DNSSLTIKKPNELSRVLGLKTLATHGLAAVNSVPWDTIANYAKPFLNKVVSTTTNIVTRC
EPI_ISL_413561 1283 DNSSLTIKKPNELSRVLGLKTLATHGLAAVNSVPWDTIANYAKPFLNKVVSTTTNIVTRC
EPI_ISL_413692 1283 DNSSLTIKKPNELSRVLGLKTLATHGLAAVNSVPWDTIANYAKPFLNKVVSTTTNIVTRC
EPI_ISL_413693 1283 DNSSLTIKKPNELSRVLGLKTLATHGLAAVNSVPWDTIANYAKPFLNKVVSTTTNIVTRC
EPI_ISL_413694 1283 DNSSLTIKKPNELSRVLGLKTLATHGLAAVNSVPWDTIANYAKPFLNKVVSTTTNIVTRC
EPI_ISL_414692 1283 DNSSLTIKKPNELSRVLGLKTLATHGLAAVNSVPWDTIANYAKPFLNKVVSTTTNIVTRC
EPI_ISL_424359 1283 DNSSLTIKKPNELSRVLGLKTLATHGLAAVNSVPWDTIANYAKPFLNKVVSTTTNIVTRC
EPI_ISL_424396 1283 DNSSLTIKKPNELSRVLGLKTLATHGLAAVNSVPWDTIANYAKPFLNKVVSTTTNIVTRC
EPI_ISL_424399 1283 DNSSLTIKKPNELSRVLGLKTLATHGLAAVNSVPWDTIANYAKPFLNKVVSTTTNIVTRC
EPI_ISL_425530 1283 DNSSLTIKKPNELSRVLGLKTLATHGLAAVNSVPWDTIANYAKPFLNKVVSTTTNIVTRC
EPI_ISL_425561 1283 DNSSLTIKKPNELSRVLGLKTLATHGLAAVNSVPWDTIANYAKPFLNKVVSTTTNIVTRC
EPI_ISL_425614 1283 DNSSLTIKKPNELSRVLGLKTLATHGLAAVNSVPWDTIANYAKPFLNKVVSTTTNIVTRC
EPI_ISL_425635 1283 DNSSLTIKKPNELSRVLGLKTLATHGLAAVNSVPWDTIANYAKPFLNKVVSTTTNIVTRC
EPI_ISL_426848 1283 DNSSLTIKKPNELSRVLGLKTLATHGLAAVNSVPWDTIANYAKPFLNKVVSTTTNIVTRC
EPI_ISL_426850 1283 DNSSLTIKKPNELSRVLGLKTLATHGLAAVNSVPWDTIANYAKPFLNKVVSTTTNIVTRC
EPI_ISL_426955 1283 DNSSLTIKKPNELSRVLGLKTLATHGLAAVNSVPWDTIANYAKPFLNKVVSTTTNIVTRC
```

EPI_ISL_426963 1283 DNSSLTIKKPNELSRVLGLKTLATHGLAAVNSVPWDTIANYAKPFLNKVVSTTTNIVTRC

```
ORF1ab         2161 LNRVCTNYMPYFFTLLLQLCTFTRSTNSRIKASMPTTIAKNTVKSVGKFCLEASFNYLKS
EPI_ISL_411066 1343 LNRVCTNYMPYFFTLLLQLCTFTRSTNSRIKASMPTTIAKNTVKSVGKFCLEASFNYLKS
EPI_ISL_413522 1343 LNRVCTNYMPYFFTLXLXLXXFTRSTNSRIKASMPTTIAKNTVKSVGKFCLEASFNYLKS
EPI_ISL_413558 1343 LNRVCTNYMPYFFTLLLQLCTFTRSTNSRIKASMPTTIAKNTVKSVGKFCLEASFNYLKS
EPI_ISL_413559 1343 LNRVCTNYMPYFFTLLLQLCTFTRSTNSRIKASMPTTIAKNTVKSVGKFCLEASFNYLKS
EPI_ISL_413561 1343 LNRVCTNYMPYFFTLLLQLCTFTRSTNSRIKASMPTTIAKNTVKSVGKFCLEASFNYLKS
EPI_ISL_413692 1343 LNRVCTNYMPYFFTLLLQLCTFTRSTNSRIKASMPTTIAKNTVKSVGKFCLEASFNYLKS
EPI_ISL_413693 1343 LNRVCTNYMPYFFTLLLQLCTFTRSTNSRIKASMPTTIAKNTVKSVGKFCLEASFNYLKS
EPI_ISL_413694 1343 LNRVCTNYMPYFFTLLLQLCTFTRSTNSRIKASMPTTIAKNTVKSVGKFCLEASFNYLKS
EPI_ISL_414692 1343 LNRVCTNYMPYFFTLLLQLCTFTRSTNSRIKASMPTTIAKNTVKSVGKFCLEASFNYLKS
EPI_ISL_424359 1343 LNRVCTNYMPYFFTLLLQLCTFTRSTNSRIKASMPTTIAKNTVKSVGKFCLEASFNYLKS
EPI_ISL_424396 1343 LNRVCTNYMPYFFTLLLQLCTFTRSTNSRIKASMPTTIAKNTVKSVGKFCLEASFNYLKS
EPI_ISL_424399 1343 LNRVCTNYMPYFFTLLLQLCTFTRSTNSRIKASMPTTIAKNTVKSVGKFCLEASFNYLKS
EPI_ISL_425530 1343 LNRVCTNYMPYFFTLLLQLCTFTRSTNSRIKASMPTTIAKNTVKSVGKFCLEASFNYLKS
EPI_ISL_425561 1343 LNRVCTNYMPYFFTLLLQLCTFTRSTNSRIKASMPTTIAKNTVKSVGKFCLEASFNYLKS
EPI_ISL_425614 1343 LNRVCTNYMPYFFTLLLQLCTFTRSTNSRIKASMPTTIAKNTVKSVGKFCLEASFNYLKS
EPI_ISL_425635 1343 LNRVCTNYMPYFFTLLLQLCTFTRSTNSRIKASMPTTIAKNTVKSVGKFCLEASFNYLKS
EPI_ISL_426848 1343 LNRVCTNYMPYFFTLLLQLCTFTRSTNSRIKASMPTTIAKNTVKSVGKFCLEASFNYLKS
EPI_ISL_426850 1343 LNRVCTNYMPYFFTLLLQLCTFTRSTNSRIKASMPTTIAKNTVKSVGKFCLEASFNYLKS
EPI_ISL_426955 1343 LNRVCTNYMPYFFTLLLQLCTFTRSTNSRIKASMPTTIAKNTVKSVGKFCLEASFNYLKS
EPI_ISL_426963 1343 LNRVCTNYMPYFFTLLLQLCTFTRSTNSRIKASMPTTIAKNTVKSVGKFCLEASFNYLKS

ORF1ab         2221 PNFSKLINIIIWFLLLSVCLGSLIYSTAALGVLMSNLGMPSYCTGYREGYLNSTNVTIAT
EPI_ISL_411066 1403 PNFSKLXXXXXXFLLXSVCLGSLIYSTAALGVLMSNLGMPSYCTGYREGYLNSTNVTIAT
EPI_ISL_413522 1403 PNFSKLINIIIWFLLLSVCLGSLIYSTAALGVLMSNLGMPSYCTGYREGYLNSTNVTIAT
EPI_ISL_413558 1403 PNFSKLINIIIWFLLLSVCLGSLIYSTAALGVLMSNLGMPSYCTGYREGYLNSTNVTIAT
EPI_ISL_413559 1403 PNFSKLINIIIWFLLLSVCLGSLIYSTAALGVLMSNLGMPSYCTGYREGYLNPTNVTIAT
EPI_ISL_413561 1403 PNFSKLVNIIIWFLLLSVCLGSLIYSTAALGVLMSNLGMPSYCTGYREGYLNSTNVTIAT
EPI_ISL_413692 1403 PNFSKLINIIIWFLLLSVCLGSLIYSTAALGVLMSNLGMPSYCTGYREGYLNSTNVTIAT
EPI_ISL_413693 1403 PNFSKLINIIIWFLLLSVCLGSLIYSTAALGVLMSNLGMPSYCTGYREGYLNSTNVTIAT
EPI_ISL_413694 1403 PNFSKLINIIIWFLLLSVCLGSLIYSTAALGVLMSNLGMPSYCTGYREGYLNSTNVTIAT
EPI_ISL_414692 1403 PNFSKLINIIIWFLLLSVCLGSLIYSTAALGVLMSNLGMPSYCTGYREGYLNSTNVTIAT
EPI_ISL_424359 1403 PNFSKLINIIIWFLLLSVCLGSLIYSTAALGVLMSNLGMPSYCTGYREGYLNSTNVTIAT
EPI_ISL_424396 1403 PNFSKLINIIIWFLLLSVCLGSLIYSTAALGVLMSNLGMPSYCTGYREGYLNSTNVTIAT
EPI_ISL_424399 1403 PNFSKLINIIIWFLLLSVCLGSLIYSTAALGVLMSNLGMPSYCTGYREGYLNSTNVTIAT
EPI_ISL_425530 1403 PNFSKLINIIIWFLLLSVCLGSLIYSTAALGVLMSNLGMPSYCTGYREGYLNSTNVTIAT
EPI_ISL_425561 1403 PNFSKLINIIIWFLLLSVCLGSLIYSTAALGVLMSNLGMPSYCTGYREGYLNSTNVTIAT
EPI_ISL_425614 1403 PNFSKLINIIIWFLLLSVCLGSLIYSTAALGVLMSNLGMPSYCTGYREGYLNSTNVTIAT
EPI_ISL_425635 1403 PNFSKLINIIIWFLLLSVCLGSLIYSTAALGVLMSNLGMPSYCTGYREGYLNSTNVTIAT
EPI_ISL_426848 1403 PNFSKLINIIIWFLLLSVCLGSLIYSTAALGVLMSNLGMPSYCTGYREGYLNSTNVTIAT
EPI_ISL_426850 1403 PNFSKLINIIIWFLLLSVCLGSLIYSTAALGVLMSNLGMPSYCTGYREGYLNSTNVTIAT
EPI_ISL_426955 1403 PNFSKLINIIIWFLLLSVCLGSLIYSTAALGVLMSNLGMPSYCTGYREGYLNSTNVTIAT
EPI_ISL_426963 1403 PNFSKLINIIIWFLLLSVCLGSLIYSTAALGVLMSNLGMPSYCTGYREGYLNSTNVTIAT

ORF1ab         2281 YCTGSIPCSVCLSGLDSLDTYPSLETIQITISSFKWDLTAFGLVAEWFLAYILFTRFFYV
EPI_ISL_411066 1463 YCTGSIPCSVCLSGLDSLDTYPSLETIQITISSFKWDLTAFGLVAEWFLAYILFTRFFYV
EPI_ISL_413522 1463 YCTGSIPCSVCLSGLDSLDTYPSLETIQITISSFKWDLTAFGLVAEWFLAYILFTRFFYV
EPI_ISL_413558 1463 YCTGSIPCSVCLSGLDSLDTYPSLETIQITISSFKWDLTAFGLVAEWFLAYILFTRFFYV
EPI_ISL_413559 1463 YCTGSIPCSVCLSGLDSLDTYPSLETIQITISSFKWDLTAFGLVAEWFLAYILFTRFFYV
EPI_ISL_413561 1463 YCTGSIPCSVCLSGLDSLDTYPSLETIQITISSFKWDLTAFGLVAEWFLAYILFTRFFYV
EPI_ISL_413692 1463 YCTGSIPCSVCLSGLDSLDTYPSLETIQITISSFKWDLTAFGLVAEWFLAYILFTRFFYV
EPI_ISL_413693 1463 YCTGSIPCSVCLSGLDSLDTYPSLETIQITISSFKWDLTAFGLVAEWFLAYILFTRFFYV
EPI_ISL_413694 1463 YCTGSIPCSVCLSGLDSLDTYPSLETIQITISSFKWDLTAFGLVAEWFLAYILFTRFFYV
EPI_ISL_414692 1463 YCTGSIPCSVCLSGLDSLDTYPSLETIQITISSFKWDLTAFGLVAEWFLAYILFTRFFYV
EPI_ISL_424359 1463 YCTGSIPCSVCLSGLDSLDTYPSLETIQITISSFKWDLTAFGLVAEWFLAYILFTRFFYV
EPI_ISL_424396 1463 YCTGSIPCSVCLSGLDSLDTYPSLETIQITISSFKWDLTAFGLVAEWFLAYILFTRFFYV
EPI_ISL_424399 1463 YCTGSIPCSVCLSGLDSLDTYPSLETIQITISSFKWDLTAFGLVAEWFLAYILFTRFFYV
EPI_ISL_425530 1463 YCTGSIPCSVCLSGLDSLDTYPSLETIQITISSFKWDLTAFGLVAEWFLAYILFTRFFYV
EPI_ISL_425561 1463 YCTGSIPCSVCLSGLDSLDTYPSLETIQITISSFKWDLTAFGLVAEWFLAYILFTRFFYV
EPI_ISL_425614 1463 YCTGSIPCSVCLSGLDSLDTYPSLETIQITISSFKWDLTAFGLVAEWFLAYILFTRFFYV
EPI_ISL_425635 1463 YCTGSIPCSVCLSGLDSLDTYPSLETIQITISSFKWDLTAFGLVAEWFLAYILFTRFFYV
EPI_ISL_426848 1463 YCTGSIPCSVCLSGLDSLDTYPSLETIQITISSFKWDLTAFGLVAEWFLAYILFTRFFYV
EPI_ISL_426850 1463 YCTGSIPCSVCLSGLDSLDTYPSLETIQITISSFKWDLTAFGLVAEWFLAYILFTRFFYV
EPI_ISL_426955 1463 YCTGSIPCSVCLSGLDSLDTYPSLETIQITISSFKWDLTAFGLVAEWFLAYILFTRFFYV
```

EPI_ISL_426963 1463 YCTGSIPCSVCLSGLDSLDTYPSLETIQITISSFKWDLTAFGLVAEWFLAYILFTRFFYV

```
ORF1ab         2341 LGLAAIMQLFFSYFAVHFISNSWLMWLIINLVQMAPISAMVRMYIFFASFYYVWKSYVHV
EPI_ISL_411066 1523 LGLAAIMQLFFSYFAVHFISNSWLMWLIINLVQMAPISAMVRMYIFFASFYYVWKSYVHV
EPI_ISL_413522 1523 LGLAAIMQLFFSYFAVHFISNSWLMWLIINLVQMAPISAMVRMYIFFASFYYVWKSYVHV
EPI_ISL_413558 1523 LGLAAIMQLFFSYFAVHFISNSWLMWLIINLVQMAPISAMVRMYIFFASFYYVWKSYVHV
EPI_ISL_413559 1523 LGLAAIMQLFFSYFAVHFISNSWLMWLIINLVQMAPISAMVRMYIFFASFYYVWKSYVHV
EPI_ISL_413561 1523 LGLAAIMQLFFSYFAVHFISNSWLMWLIINLVQMAPISAMVRMYIFFASFYYVWKSYVHV
EPI_ISL_413692 1523 LGLAAIMQLFFSYFAVHFISNSWLMWLIINLVQMAPISAMVRMYIFFASFYYVWKSYVHV
EPI_ISL_413693 1523 LGLAAIMQLFFSYFAVHFISNSWLMWLIINLVQMAPISAMVRMYIFFASFYYVWKSYVHV
EPI_ISL_413694 1523 LGLAAIMQLFFSYFAVHFISNSWLMWLIINLVQMAPISAMVRMYIFFASFYYVWKSYVHV
EPI_ISL_414692 1523 LGLAAIMQLFFSYFAVHFISNSWLMWLIINLVQMAPISAMVRMYIFFASFYYVWKSYVHV
EPI_ISL_424359 1523 LGLAAIMQLFFSYFAVHFISNSWLMWLIINLVQMAPISAMVRMYIFFASFYYVWKSYVHV
EPI_ISL_424396 1523 LGLAAIMQLFFSYFAVHFISNSWLMWLIINLVQMAPISAMVRMYIFFASFYYVWKSYVHV
EPI_ISL_424399 1523 LGLAAIMQLFFSYFAVHFISNSWLMWLIINLVQMAPISAMVRMYIFFASFYYVWKSYVHV
EPI_ISL_425530 1523 LGLAAIMQLFFSYFAVHFISNSWLMWLIINLVQMAPISAMVRMYIFFASFYYVWKSYVHV
EPI_ISL_425561 1523 LGLAAIMQLFFSYFAVHFISNSWLMWLIINLVQMAPISAMVRMYIFFASFYYVWKSYVHV
EPI_ISL_425614 1523 LGLAAIMQLFFSYFAVHFISNSWLMWLIINLVQMAPISAMVRMYIFFASFYYVWKSYVHV
EPI_ISL_425635 1523 LGLAAIMQLFFSYFAVHFISNSWLMWLIINLVQMAPISAMVRMYIFFASFYYVWKSYVHV
EPI_ISL_426848 1523 LGLAAIMQLFFSYFAVHFISNSWLMWLIINLVQMAPISAMVRMYIFFASFYYVWKSYVHV
EPI_ISL_426850 1523 LGLAAIMQLFFSYFAVHFISNSWLMWLIINLVQMAPISAMVRMYIFFASFYYVWKSYVHV
EPI_ISL_426955 1523 LGLAAIMQLFFSYFAVHFISNSWLMWLIINLVQMAPISAMVRMYIFFASFYYVWKSYVHV
EPI_ISL_426963 1523 LGLAAIMQLFFSYFAVHFISNSWLMWLIINLVQMAPISAMVRMYIFFASFYYVWKSYVHV

ORF1ab         2401 VDGCNSSTCMMCYKRNRATRVECTTIVNGVRRSFYVYANGGKGFCKLHNWNCVNCDTFCA
EPI_ISL_411066 1583 VDGCNSSTCMMCYKRNRATRVECTTIVNGVRRSFYVYANGGKGFCKLHNWNCVNCDTFCA
EPI_ISL_413522 1583 VDGCNSSTCMMCYKRNRATRVECTTIVNGVRRSFYVYANGGKGFCKLHNWNCVNCDTFCA
EPI_ISL_413558 1583 VDGCNSSTCMMCYKRNRATRVECTTIVNGVRRSFYVYANGGKGFCKLHNWNCVNCDTFCA
EPI_ISL_413559 1583 VDGCNSSTCMMCYKRNRATRVECTTIVNGVRRSFYVYANGGKGFCKLHNWNCVNCDTFCA
EPI_ISL_413561 1583 VDGCNSSTCMMCYKRNRATRVECTTIVNGVRRSFYVYANGGKGFCKLHNWNCVNCDTFCA
EPI_ISL_413692 1583 VDGCNSSTCMMCYKRNRATRVECTTIVNGVRRSFYVYANGGKGFCKLHNWNCVNCDTFCA
EPI_ISL_413693 1583 VDGCNSSTCMMCYKRNRATRVECTTIVNGVRRSFYVYANGGKGFCKLHNWNCVNCDTFCA
EPI_ISL_413694 1583 VDGCNSSTCMMCYKRNRATRVECTTIVNGVRRSFYVYANGGKGFCKLHNWNCVNCDTFCA
EPI_ISL_414692 1583 VDGCNSSTCMMCYKRNRATRVECTTIVNGVRRSFYVYANGGKGFCKLHNWNCVNCDTFCA
EPI_ISL_424359 1583 VDGCNSSTCMMCYKRNRATRVECTTIVNGVRRSFYVYANGGKGFCKLHNWNCVNCDTFCA
EPI_ISL_424396 1583 VDGCNSSTCMMCYKRNRATRVECTTIVNGVRRSFYVYANGGKGFCKLHNWNCVNCDTFCA
EPI_ISL_424399 1583 VDGCNSSTCMMCYKRNRATRVECTTIVNGVRRSFYVYANGGKGFCKLHNWNCVNCDTFCA
EPI_ISL_425530 1583 VDGCNSSTCMMCYKRNRATRVECTTIVNGVRRSFYVYANGGKGFCKLHNWNCVNCDTFCA
EPI_ISL_425561 1583 VDGCNSSTCMMCYKRNRATRVECTTIVNGVRRSFYVYANGGKGFCKLHNWNCVNCDTFCA
EPI_ISL_425614 1583 VDGCNSSTCMMCYKRNRATRVECTTIVNGVRRSFYVYANGGKGFCKLHNWNCVNCDTFCA
EPI_ISL_425635 1583 VDGCNSSTCMMCYKRNRATRVECTTIVNGVRRSFYVYANGGKGFCKLHNWNCVNCDTFCA
EPI_ISL_426848 1583 VDGCNSSTCMMCYKRNRATRVECTTIVNGVRRSFYVYANGGKGFCKLHNWNCVNCDTFCA
EPI_ISL_426850 1583 VDGCNSSTCMMCYKRNRATRVECTTIVNGVRRSFYVYANGGKGFCKLHNWNCVNCDTFCA
EPI_ISL_426955 1583 VDGCNSSTCMMCYKRNRATRVECTTIVNGVRRSFYVYANGGKGFCKLHNWNCVNCDTFCA
EPI_ISL_426963 1583 VDGCNSSTCMMCYKRNRATRVECTTIVNGVRRSFYVYANGGKGFCKLHNWNCVNCDTFCA

ORF1ab         2461 GSTFISDEVARDLSLQFKRPINPTDQSSYIVDSVTVKNGSIHLYFDKAGQKTYERHSLSH
EPI_ISL_411066 1643 GSTFISDEVARDLSLQFKRPINPTDQSSYIVDSVTVKNGSIHLYFDKAGQKTYERHSLSH
EPI_ISL_413522 1643 GSTFISDEVARDLSLQFKRPINPTDQSSYIVDSVTVKNGSIHLYFDKAGQKTYERHSLSH
EPI_ISL_413558 1643 GSTFISDEVARDLSLQFKRPINPTDQSSYIVDSVTVKNGSIHLYFDKAGQKTYERHSLSH
EPI_ISL_413559 1643 GSTFISDEVARDLSLQFKRPINPTDQSSYIVDSVTVKNGSIHLYFDKAGQKTYERHSLSH
EPI_ISL_413561 1643 GSTFISDEVARDLSLQFKRPINPTDQSSYIVDSVTVKNGSIHLYFDKAGQKTYERHSLSH
EPI_ISL_413692 1643 GSTFISDEVARDLSLQFKRPINPTDQSSYIVDSVTVKNGSIHLYFDKAGQKTYERHSLSH
EPI_ISL_413693 1643 GSTFISDEVARDLSLQFKRPINPTDQSSYIVDSVTVKNGSIHLYFDKAGQKTYERHSLSH
EPI_ISL_413694 1643 GSTFISDEVARDLSLQFKRPINPTDQSSYIVDSVTVKNGSIHLYFDKAGQKTYERHSLSH
EPI_ISL_414692 1643 GSTFISDEVARDLSLQFKRPINPTDQSSYIVDSVTVKNGSIHLYFDKAGQKTYERHSLSH
EPI_ISL_424359 1643 GSTFISDEVARDLSLQFKRPINPTDQSSYIVDSVTVKNGSIHLYFDKAGQKTYERHSLSH
EPI_ISL_424396 1643 GSTFISDEVARDLSLQFKRPINPTDQSSYIVDSVTVKNGSIHLYFDKAGQKTYERHSLSH
EPI_ISL_424399 1643 GSTFISDEVARDLSLQFKRPINPTDQSSYIVDSVTVKNGSIHLYFDKAGQKTYERHSLSH
EPI_ISL_425530 1643 GSTFISDEVARDLSLQFKRPINPTDQSSYIVDSVTVKNGSIHLYFDKAGQKTYERHSLSH
EPI_ISL_425561 1643 GSTFISDEVARDLSLQFKRPINPTDQSSYIVDSVTVKNGSIHLYFDKAGQKTYERHSLSH
EPI_ISL_425614 1643 GSTFISDEVARDLSLQFKRPINPTDQSSYIVDSVTVKNGSIHLYFDKAGQKTYERHSLSH
EPI_ISL_425635 1643 GSTFISDEVARDLSLQFKRPINPTDQSSYIVDSVTVKNGSIHLYFDKAGQKTYERHSLSH
EPI_ISL_426848 1643 GSTFISDEVARDLSLQFKRPINPTDQSSYIVDSVTVKNGSIHLYFDKAGQKTYERHSLSH
EPI_ISL_426850 1643 GSTFISDEVARDLSLQFKRPINPTDQSSYIVDSVTVKNGSIHLYFDKAGQKTYERHSLSH
EPI_ISL_426955 1643 GSTFISDEVARDLSLQFKRPINPTDQSSYIVDSVTVKNGSIHLYFDKAGQKTYERHSLSH
```

EPI_ISL_426963 1643 GSTFISDEVARDLSLQFKRPINPTDQSSYIVDSVTVKNGSIHLYFDKAGQKTYERHSLSH

```
ORF1ab         2521 FVNLDNLRANNTKGSLPINVIVFDGKSKCEESSAKSASVYYSQLMCQPILLLDQALVSDV
EPI_ISL_411066 1703 FVNLDNLRANNTKGSLPINVIVFDGKSKCEESSAKSASVYYSQLMCQPILLLDQALVSDV
EPI_ISL_413522 1703 FVNLDNLRANNTKGSLPINVIVFDGKSKCEESSAKSASVYYSQLMCQPILLLDQALVSDV
EPI_ISL_413558 1703 FVNLDNLRANNTKGSLPINVIVFDGKSKCEESSAKSASVYYSQLMCQPILLLDQALVSDV
EPI_ISL_413559 1703 FVNLDNLRANNTKGSLPINVIVFDGKSKCEESSAKSASVYYSQLMCQPILLLDQALVSDV
EPI_ISL_413561 1703 FVNLDNLRANNTKGSLPINVIVFDGKSKCEESSAKSASVYYSQLMCQPILLLDQALVSDV
EPI_ISL_413692 1703 FVNLDNLRANNTKGSLPINVIVFDGKSKCEESSAKSASVYYSQLMCQPILLLDQALVSDV
EPI_ISL_413693 1703 FVNLDNLRANNTKGSLPINVIVFDGKSKCEESSAKSASVYYSQLMCQPILLLDQALVSDV
EPI_ISL_413694 1703 FVNLDNLRANNTKGSLPINVIVFDGKSKCEESSAKSASVYYSQLMCQPILLLDQALVSDV
EPI_ISL_414692 1703 FVNLDNLRANNTKGSLPINVIVFDGKSKCEESSAKSASVYYSQLMCQPILLLDQALVSDV
EPI_ISL_424359 1703 FVNLDNLRANNTKGSLPINVIVFDGKSKCEESSAKSASVYYSQLMCQPILLLDQALVSDV
EPI_ISL_424396 1703 FVNLDNLRANNTKGSLPINVIVFDGKSKCEESSAKSASVYYSQLMCQPILLLDQALVSDV
EPI_ISL_424399 1703 FVNLDNLRANNTKGSLPINVIVFDGKSKCEESSAKSASVYYSQLMCQPILLLDQALVSDV
EPI_ISL_425530 1703 FVNLDNLRANNTKGSLPINVIVFDGKSKCEESSAKSASVYYSQLMCQPILLLDQALVSDV
EPI_ISL_425561 1703 FVNLDNLRANNTKGSLPINVIVFDGKSKCEESSAKSASVYYSQLMCQPILLLDQALVSDV
EPI_ISL_425614 1703 FVNLDNLRANNTKGSLPINVIVFDGKSKCEESSAKSASVYYSQLMCQPILLLDQALVSDV
EPI_ISL_425635 1703 FVNLDNLRANNTKGSLPINVIVFDGKSKCEESSAKSASVYYSQLMCQPILLLDQALVSDV
EPI_ISL_426848 1703 FVNLDNLRANNTKGSLPINVIVFDGKSKCEESSAKSASVYYSQLMCQPILLLDQALVSDV
EPI_ISL_426850 1703 FVNLDNLRANNTKGSLPINVIVFDGKSKCEESSAKSASVYYSQLMCQPILLLDQALVSDV
EPI_ISL_426955 1703 FVNLDNLRANNTKGSLPINVIVFDGKSKCEESSAKSASVYYSQLMCQPILLLDQALVSDV
EPI_ISL_426963 1703 FVNLDNLRANNTKGSLPINVIVFDGKSKCEESSAKSASVYYSQLMCQPILLLDQALVSDV
```

```
ORF1ab         2581 GDSAEVAVKMFDAYVNTFSSTFNVPMEKLKTLVATAEAELAKNVSLDNVLSTFISAARQG
EPI_ISL_411066 1763 GDSAEVAVKMFDAYVNTFSSTFNVPMEKLKTLVATAEAELAKNVSLDNVLSTFISAARQG
EPI_ISL_413522 1763 GDSAEVAVKMFDAYVNTFSSTFNVPMEKLKTLVATAEAELAKNVSLDNVLSTFISAARQG
EPI_ISL_413558 1763 GDSAEVAVKMFDAYVNTFSSTFNVPMEKLKTLVATAEAELAKNVSLDNVLSTFISAARQG
EPI_ISL_413559 1763 GDSAEVAVKMFDAYVNTFSSTFNVPMEKLKTLVATAEAELAKNVSLDNVLSTFISAARQG
EPI_ISL_413561 1763 GDSAEVAVKMFDAYVNTFSSTFNVPMEKLKTLVATAEAELAKNVSLDNVLSTFISAARQG
EPI_ISL_413692 1763 GDSAEVAVKMFDAYVNTFSSTFNVPMEKLKTLVATAEAELAKNVSLDNVLSTFISAARQG
EPI_ISL_413693 1763 GDSAEVAVKMFDAYVNTFSSTFNVPMEKLKTLVATAEAELAKNVSLDNVLSTFISAARQG
EPI_ISL_413694 1763 GDSAEVAVKMFDAYVNTFSSTFNVPMEKLKTLVATAEAELAKNVSLDNVLSTFISAARQG
EPI_ISL_414692 1763 GDSAEVAVKMFDAYVNTFSSTFNVPMEKLKTLVATAEAELAKNVSLDNVLSTFISAARQG
EPI_ISL_424359 1763 GDSAEVAVKMFDAYVNTFSSTFNVPMEKLKTLVATAEAELAKNVSLDNVLSTFISAARQG
EPI_ISL_424396 1763 GDSAEVAVKMFDAYVNTFSSTFNVPMEKLKTLVATAEAELAKNVSLDNVLSTFISAARQG
EPI_ISL_424399 1763 GDSAEVAVKMFDAYVNTFSSTFNVPMEKLKTLVATAEAELAKNVSLDNVLSTFISAARQG
EPI_ISL_425530 1763 GDSAEVAVKMFDAYVNTFSSTFNVPMEKLKTLVATAEAELAKNVSLDNVLSTFISAARQG
EPI_ISL_425561 1763 GDSAEVAVKMFDAYVNTFSSTFNVPMEKLKTLVATAEAELAKNVSLDNVLSTFISAARQG
EPI_ISL_425614 1763 GDSAEVAVKMFDAYVNTFSSTFNVPMEKLKTLVATAEAELAKNVSLDNVLSTFISAARQG
EPI_ISL_425635 1763 GDSAEVAVKMFDAYVNTFSSTFNVPMEKLKTLVATAEAELAKNVSLDNVLSTFISAARQG
EPI_ISL_426848 1763 GDSAEVAVKMFDAYVNTFSSTFNVPMEKLKTLVATAEAELAKNVSLDNVLSTFISAARQG
EPI_ISL_426850 1763 GDSAEVAVKMFDAYVNTFSSTFNVPMEKLKTLVATAEAELAKNVSLDNVLSTFISAARQG
EPI_ISL_426955 1763 GDSAEVAVKMFDAYVNTFSSTFNVPMEKLKTLVATAEAELAKNVSLDNVLSTFISAARQG
EPI_ISL_426963 1763 GDSAEVAVKMFDAYVNTFSSTFNVPMEKLKTLVATAEAELAKNVSLDNVLSTFISAARQG
```

```
ORF1ab         2641 FVDSDVETKDVVECLKLSHQSDIEVTGDSCNNYMLTYNKVENMTPRDLGACIDCSARHIN
EPI_ISL_411066 1823 FVDSDVETKDVVECLKLSHQSDIEVTGDSCNNYMLTYNKVENMTPRDLGACIDCSARHIN
EPI_ISL_413522 1823 FVDSDVETKDVVECLKLSHQSDIEVTGDSCNNYMLTYNKVENMTPRDLGACIDCSARHIN
EPI_ISL_413558 1823 FVDSDVETKDVVECLKLSHQSDIEVTGDSCNNYMLTYNKVENMTPRDLGACIDCSARHIN
EPI_ISL_413559 1823 FVDSDVETKDVVECLKLSHQSDIEVTGDSCNNYMLTYNKVENMTPRDLGACIDCSARHIN
EPI_ISL_413561 1823 FVDSDVETKDVVECLKLSHQSDIEVTGDSCNNYMLTYNKVENMTPRDLGACIDCSARHIN
EPI_ISL_413692 1823 FVDSDVETKDVVECLKLSHQSDIEVTGDSCNNYMLTYNKVENMTPRDLGACIDCSARHIN
EPI_ISL_413693 1823 FVDSDVETKDVVECLKLSHQSDIEVTGDSCNNYMLTYNKVENMTPRDLGACIDCSARHIN
EPI_ISL_413694 1823 FVDSDVETKDVVECLKLSHQSDIEVTGDSCNNYMLTYNKVENMTPRDLGACIDCSARHIN
EPI_ISL_414692 1823 FVDSDVETKDVVECLKLSHQSDIEVTGDSCNNYMLTYNKVENMTPRDLGACIDCSARHIN
EPI_ISL_424359 1823 FVDSDVETKDVVECLKLSHQSDIEVTGDSCNNYMLTYNKVENMTPRDLGACIDCSARHIN
EPI_ISL_424396 1823 FVDSDVETKDVVECLKLSHQSDIEVTGDSCNNYMLTYNKVENMTPRDLGACIDCSARHIN
EPI_ISL_424399 1823 FVDSDVETKDVVECLKLSHQSDIEVTGDSCNNYMLTYNKVENMTPRDLGACIDCSARHIN
EPI_ISL_425530 1823 FVDSDVETKDVVECLKLSHQSDIEVTGDSCNNYMLTYNKVENMTPRDLGACIDCSARHIN
EPI_ISL_425561 1823 FVDSDVETKDVVECLKLSHQSDIEVTGDSCNNYMLTYNKVENMTPRDLGACIDCSARHIN
EPI_ISL_425614 1823 FVDSDVETKDVVECLKLSHQSDIEVTGDSCNNYMLTYNKVENMTPRDLGACIDCSARHIN
EPI_ISL_425635 1823 FVDSDVETKDVVECLKLSHQSDIEVTGDSCNNYMLTYNKVENMTPRDLGACIDCSARHIN
EPI_ISL_426848 1823 FVDSDVETKDVVECLKLSHQSDIEVTGDSCNNYMLTYNKVENMTPRDLGACIDCSARHIN
EPI_ISL_426850 1823 FVDSDVETKDVVECLKLSHQSDIEVTGDSCNNYMLTYNKVENMTPRDLGACIDCSARHIN
EPI_ISL_426955 1823 FVDSDVETKDVVECLKLSHQSDIEVTGDSCNNYMLTYNKVENMTPRDLGACIDCSARHIN
```

EPI_ISL_426963 1823 FVDSDVETKDVVECLKLSHQSDIEVTGDSCNNYMLTYNKVENMTPRDLGACIDCSARHIN

```
ORF1ab         2701 AQVAKSHNIALIWNVKDFMSLSEQLRKQIRSAAKKNNLPFKLTCATTRQVVNVVTTKIAL
EPI_ISL_411066 1883 AQVAKSHNIALIWNVKDFMSLSEQLRKQIRSAAKKNNLPFKLTCATTRQVVNVVTTKIAL
EPI_ISL_413522 1883 AQVAKSHNIALIWNVKDFMSLSEQLRKQIRSAAKKNNLPFKLTCATTRQVVNVVTTKIAL
EPI_ISL_413558 1883 AQVAKSHNIALIWNVKDFMSLSEQLRKQIRSAAKKNNLPFKLTCATTRQVVNVVTTKIAL
EPI_ISL_413559 1883 AQVAKSHNIALIWNVKDFMSLSEQLRKQIRSAAKKNNLPFKLTCATTRQVVNVVTTKIAL
EPI_ISL_413561 1883 AQVAKSHNIALIWNVKDFMSLSEQLRKQIRSAAKKNNLPFKLTCATTRQVVNVVTTKIAL
EPI_ISL_413692 1883 AQVAKSHNIALIWNVKDFMSLSEQLRKQIRSAAKKNNLPFKLTCATTRQVVNVVTTKIAL
EPI_ISL_413693 1883 AQVAKSHNIALIWNVKDFMSLSEQLRKQIRSAAKKNNLPFKLTCATTRQVVNVVTTKIAL
EPI_ISL_413694 1883 AQVAKSHNIALIWNVKDFMSLSEQLRKQIRSAAKKNNLPFKLTCATTRQVVNVVTTKIAL
EPI_ISL_414692 1883 AQVAKSHNIALIWNVKDFMSLSEQLRKQIRSAAKKNNLPFKLTCATTRQVVNVVTTKIAL
EPI_ISL_424359 1883 AQVAKSHNIALIWNVKDFMSLSEQLRKQIRSAAKKNNLPFKLTCATTRQVVNVVTTKIAL
EPI_ISL_424396 1883 AQVAKSHNIALIWNVKDFMSLSEQLRKQIRSAAKKNNLPFKLTCATTRQVVNVVTTKIAL
EPI_ISL_424399 1883 AQVAKSHNIALIWNVKDFMSLSEQLRKQIRSAAKKNNLPFKLTCATTRQVVNVVTTKIAL
EPI_ISL_425530 1883 AQVAKSHNIALIWNVKDFMSLSEQLRKQIRSAAKKNNLPFKLTCATTRQVVNVVTTKIAL
EPI_ISL_425561 1883 AQVAKSHNIALIWNVKDFMSLSEQLRKQIRSAAKKNNLPFKLTCATTRQVVNVVTTKIAL
EPI_ISL_425614 1883 AQVAKSHNIALIWNVKDFMSLSEQLRKQIRSAAKKNNLPFKLTCATTRQVVNVVTTKIAL
EPI_ISL_425635 1883 AQVAKSHNIALIWNVKDFMSLSEQLRKQIRSAAKKNNLPFKLTCATTRQVVNVVTTKIAL
EPI_ISL_426848 1883 AQVAKSHNIALIWNVKDFMSLSEQLRKQIRSAAKKNNLPFKLTCATTRQVVNVVTTKIAL
EPI_ISL_426850 1883 AQVAKSHNIALIWNVKDFMSLSEQLRKQIRSAAKKNNLPFKLTCATTRQVVNVVTTKIAL
EPI_ISL_426955 1883 AQVAKSHNIALIWNVKDFMSLSEQLRKQIRSAAKKNNLPFKLTCATTRQVVNVVTTKIAL
EPI_ISL_426963 1883 AQVAKSHNIALIWNVKDFMSLSEQLRKQIRSAAKKNNLPFKLTCATTRQVVNVVTTKIAL

ORF1ab         2761 KGGKIVNNWLKQLIKVTLVFLFVAAIFYLITPVHVMSKHTDFSSEIIGYKAIDGGVTRDI
EPI_ISL_411066 1943 KGG---------------------------------------------------------
EPI_ISL_413522 1943 KGG---------------------------------------------------------
EPI_ISL_413558 1943 KGG---------------------------------------------------------
EPI_ISL_413559 1943 KGG---------------------------------------------------------
EPI_ISL_413561 1943 KGG---------------------------------------------------------
EPI_ISL_413692 1943 KGG---------------------------------------------------------
EPI_ISL_413693 1943 KGG---------------------------------------------------------
EPI_ISL_413694 1943 KGG---------------------------------------------------------
EPI_ISL_414692 1943 KGG---------------------------------------------------------
EPI_ISL_424359 1943 KGG---------------------------------------------------------
EPI_ISL_424396 1943 KGG---------------------------------------------------------
EPI_ISL_424399 1943 KGG---------------------------------------------------------
EPI_ISL_425530 1943 KGG---------------------------------------------------------
EPI_ISL_425561 1943 KGG---------------------------------------------------------
EPI_ISL_425614 1943 KGG---------------------------------------------------------
EPI_ISL_425635 1943 KGG---------------------------------------------------------
EPI_ISL_426848 1943 KGG---------------------------------------------------------
EPI_ISL_426850 1943 KGG---------------------------------------------------------
EPI_ISL_426955 1943 KGG---------------------------------------------------------
EPI_ISL_426963 1943 KGG---------------------------------------------------------

ORF1ab         2821 ASTDTCFANKHADFDTWFSQRGGSYTNDKACPLIAAVITREVGFVVPGLPGTILRTTNGD
EPI_ISL_411066      ------------------------------------------------------------
EPI_ISL_413522      ------------------------------------------------------------
EPI_ISL_413558      ------------------------------------------------------------
EPI_ISL_413559      ------------------------------------------------------------
EPI_ISL_413561      ------------------------------------------------------------
EPI_ISL_413692      ------------------------------------------------------------
EPI_ISL_413693      ------------------------------------------------------------
EPI_ISL_413694      ------------------------------------------------------------
EPI_ISL_414692      ------------------------------------------------------------
EPI_ISL_424359      ------------------------------------------------------------
EPI_ISL_424396      ------------------------------------------------------------
EPI_ISL_424399      ------------------------------------------------------------
EPI_ISL_425530      ------------------------------------------------------------
EPI_ISL_425561      ------------------------------------------------------------
EPI_ISL_425614      ------------------------------------------------------------
EPI_ISL_425635      ------------------------------------------------------------
EPI_ISL_426848      ------------------------------------------------------------
EPI_ISL_426850      ------------------------------------------------------------
EPI_ISL_426955      ------------------------------------------------------------
```

EPI_ISL_426963 --

```
ORF1ab          2881 FLHFLPRVFSAVGNICYTPSKLIEYTDFATSACVLAAECTIFKDASGKPVPYCYDTNVLE
EPI_ISL_411066       ------------------------------------------------------------
EPI_ISL_413522       ------------------------------------------------------------
EPI_ISL_413558       ------------------------------------------------------------
EPI_ISL_413559       ------------------------------------------------------------
EPI_ISL_413561       ------------------------------------------------------------
EPI_ISL_413692       ------------------------------------------------------------
EPI_ISL_413693       ------------------------------------------------------------
EPI_ISL_413694       ------------------------------------------------------------
EPI_ISL_414692       ------------------------------------------------------------
EPI_ISL_424359       ------------------------------------------------------------
EPI_ISL_424396       ------------------------------------------------------------
EPI_ISL_424399       ------------------------------------------------------------
EPI_ISL_425530       ------------------------------------------------------------
EPI_ISL_425561       ------------------------------------------------------------
EPI_ISL_425614       ------------------------------------------------------------
EPI_ISL_425635       ------------------------------------------------------------
EPI_ISL_426848       ------------------------------------------------------------
EPI_ISL_426850       ------------------------------------------------------------
EPI_ISL_426955       ------------------------------------------------------------
EPI_ISL_426963       ------------------------------------------------------------

ORF1ab          2941 GSVAYESLRPDTRYVLMDGSIIQFPNTYLEGSVRVVTTFDSEYCRHGTCERSEAGVCVST
EPI_ISL_411066       ------------------------------------------------------------
EPI_ISL_413522       ------------------------------------------------------------
EPI_ISL_413558       ------------------------------------------------------------
EPI_ISL_413559       ------------------------------------------------------------
EPI_ISL_413561       ------------------------------------------------------------
EPI_ISL_413692       ------------------------------------------------------------
EPI_ISL_413693       ------------------------------------------------------------
EPI_ISL_413694       ------------------------------------------------------------
EPI_ISL_414692       ------------------------------------------------------------
EPI_ISL_424359       ------------------------------------------------------------
EPI_ISL_424396       ------------------------------------------------------------
EPI_ISL_424399       ------------------------------------------------------------
EPI_ISL_425530       ------------------------------------------------------------
EPI_ISL_425561       ------------------------------------------------------------
EPI_ISL_425614       ------------------------------------------------------------
EPI_ISL_425635       ------------------------------------------------------------
EPI_ISL_426848       ------------------------------------------------------------
EPI_ISL_426850       ------------------------------------------------------------
EPI_ISL_426955       ------------------------------------------------------------
EPI_ISL_426963       ------------------------------------------------------------

ORF1ab          3001 SGRWVLNNDYYRSLPGVFCGVDAVNLLTNMFTPLIQPIGALDISASIVAGGIVAIVVTCL
EPI_ISL_411066       ------------------------------------------------------------
EPI_ISL_413522       ------------------------------------------------------------
EPI_ISL_413558       ------------------------------------------------------------
EPI_ISL_413559       ------------------------------------------------------------
EPI_ISL_413561       ------------------------------------------------------------
EPI_ISL_413692       ------------------------------------------------------------
EPI_ISL_413693       ------------------------------------------------------------
EPI_ISL_413694       ------------------------------------------------------------
EPI_ISL_414692       ------------------------------------------------------------
EPI_ISL_424359       ------------------------------------------------------------
EPI_ISL_424396       ------------------------------------------------------------
EPI_ISL_424399       ------------------------------------------------------------
EPI_ISL_425530       ------------------------------------------------------------
EPI_ISL_425561       ------------------------------------------------------------
EPI_ISL_425614       ------------------------------------------------------------
EPI_ISL_425635       ------------------------------------------------------------
EPI_ISL_426848       ------------------------------------------------------------
EPI_ISL_426850       ------------------------------------------------------------
EPI_ISL_426955       ------------------------------------------------------------
```

EPI_ISL_426963 --

```
ORF1ab          3061 AYYFMRFRRAFGEYSHVVAFNTLLFLMSFTVLCLTPVYSFLPGVYSVIYLYLTFYLTNDV
EPI_ISL_411066       ------------------------------------------------------------
EPI_ISL_413522       ------------------------------------------------------------
EPI_ISL_413558       ------------------------------------------------------------
EPI_ISL_413559       ------------------------------------------------------------
EPI_ISL_413561       ------------------------------------------------------------
EPI_ISL_413692       ------------------------------------------------------------
EPI_ISL_413693       ------------------------------------------------------------
EPI_ISL_413694       ------------------------------------------------------------
EPI_ISL_414692       ------------------------------------------------------------
EPI_ISL_424359       ------------------------------------------------------------
EPI_ISL_424396       ------------------------------------------------------------
EPI_ISL_424399       ------------------------------------------------------------
EPI_ISL_425530       ------------------------------------------------------------
EPI_ISL_425561       ------------------------------------------------------------
EPI_ISL_425614       ------------------------------------------------------------
EPI_ISL_425635       ------------------------------------------------------------
EPI_ISL_426848       ------------------------------------------------------------
EPI_ISL_426850       ------------------------------------------------------------
EPI_ISL_426955       ------------------------------------------------------------
EPI_ISL_426963       ------------------------------------------------------------

ORF1ab          3121 SFLAHIQWMVMFTPLVPFWITIAYIICISTKHFYWFFSNYLKRRVVFNGVSFSTFEEAAL
EPI_ISL_411066       ------------------------------------------------------------
EPI_ISL_413522       ------------------------------------------------------------
EPI_ISL_413558       ------------------------------------------------------------
EPI_ISL_413559       ------------------------------------------------------------
EPI_ISL_413561       ------------------------------------------------------------
EPI_ISL_413692       ------------------------------------------------------------
EPI_ISL_413693       ------------------------------------------------------------
EPI_ISL_413694       ------------------------------------------------------------
EPI_ISL_414692       ------------------------------------------------------------
EPI_ISL_424359       ------------------------------------------------------------
EPI_ISL_424396       ------------------------------------------------------------
EPI_ISL_424399       ------------------------------------------------------------
EPI_ISL_425530       ------------------------------------------------------------
EPI_ISL_425561       ------------------------------------------------------------
EPI_ISL_425614       ------------------------------------------------------------
EPI_ISL_425635       ------------------------------------------------------------
EPI_ISL_426848       ------------------------------------------------------------
EPI_ISL_426850       ------------------------------------------------------------
EPI_ISL_426955       ------------------------------------------------------------
EPI_ISL_426963       ------------------------------------------------------------

ORF1ab          3181 CTFLLNKEMYLKLRSDVLLPLTQYNRYLALYNKYKYFSGAMDTTSYREAACCHLAKALND
EPI_ISL_411066       ------------------------------------------------------------
EPI_ISL_413522       ------------------------------------------------------------
EPI_ISL_413558       ------------------------------------------------------------
EPI_ISL_413559       ------------------------------------------------------------
EPI_ISL_413561       ------------------------------------------------------------
EPI_ISL_413692       ------------------------------------------------------------
EPI_ISL_413693       ------------------------------------------------------------
EPI_ISL_413694       ------------------------------------------------------------
EPI_ISL_414692       ------------------------------------------------------------
EPI_ISL_424359       ------------------------------------------------------------
EPI_ISL_424396       ------------------------------------------------------------
EPI_ISL_424399       ------------------------------------------------------------
EPI_ISL_425530       ------------------------------------------------------------
EPI_ISL_425561       ------------------------------------------------------------
EPI_ISL_425614       ------------------------------------------------------------
EPI_ISL_425635       ------------------------------------------------------------
EPI_ISL_426848       ------------------------------------------------------------
EPI_ISL_426850       ------------------------------------------------------------
EPI_ISL_426955       ------------------------------------------------------------
```

EPI_ISL_426963 --

```
ORF1ab          3241 FSNSGSDVLYQPPQTSITSAVLQSGFRKMAFPSGKVEGCMVQVTCGTTTLNGLWLDDVVY
EPI_ISL_411066       ------------------------------------------------------------
EPI_ISL_413522       ------------------------------------------------------------
EPI_ISL_413558       ------------------------------------------------------------
EPI_ISL_413559       ------------------------------------------------------------
EPI_ISL_413561       ------------------------------------------------------------
EPI_ISL_413692       ------------------------------------------------------------
EPI_ISL_413693       ------------------------------------------------------------
EPI_ISL_413694       ------------------------------------------------------------
EPI_ISL_414692       ------------------------------------------------------------
EPI_ISL_424359       ------------------------------------------------------------
EPI_ISL_424396       ------------------------------------------------------------
EPI_ISL_424399       ------------------------------------------------------------
EPI_ISL_425530       ------------------------------------------------------------
EPI_ISL_425561       ------------------------------------------------------------
EPI_ISL_425614       ------------------------------------------------------------
EPI_ISL_425635       ------------------------------------------------------------
EPI_ISL_426848       ------------------------------------------------------------
EPI_ISL_426850       ------------------------------------------------------------
EPI_ISL_426955       ------------------------------------------------------------
EPI_ISL_426963       ------------------------------------------------------------

ORF1ab          3301 CPRHVICTSEDMLNPNYEDLLIRKSNHNFLVQAGNVQLRVIGHSMQNCVLKLKVDTANPK
EPI_ISL_411066       ------------------------------------------------------------
EPI_ISL_413522       ------------------------------------------------------------
EPI_ISL_413558       ------------------------------------------------------------
EPI_ISL_413559       ------------------------------------------------------------
EPI_ISL_413561       ------------------------------------------------------------
EPI_ISL_413692       ------------------------------------------------------------
EPI_ISL_413693       ------------------------------------------------------------
EPI_ISL_413694       ------------------------------------------------------------
EPI_ISL_414692       ------------------------------------------------------------
EPI_ISL_424359       ------------------------------------------------------------
EPI_ISL_424396       ------------------------------------------------------------
EPI_ISL_424399       ------------------------------------------------------------
EPI_ISL_425530       ------------------------------------------------------------
EPI_ISL_425561       ------------------------------------------------------------
EPI_ISL_425614       ------------------------------------------------------------
EPI_ISL_425635       ------------------------------------------------------------
EPI_ISL_426848       ------------------------------------------------------------
EPI_ISL_426850       ------------------------------------------------------------
EPI_ISL_426955       ------------------------------------------------------------
EPI_ISL_426963       ------------------------------------------------------------

ORF1ab          3361 TPKYKFVRIQPGQTFSVLACYNGSPSGVYQCAMRPNFTIKGSFLNGSCGSVGFNIDYDCV
EPI_ISL_411066       ------------------------------------------------------------
EPI_ISL_413522       ------------------------------------------------------------
EPI_ISL_413558       ------------------------------------------------------------
EPI_ISL_413559       ------------------------------------------------------------
EPI_ISL_413561       ------------------------------------------------------------
EPI_ISL_413692       ------------------------------------------------------------
EPI_ISL_413693       ------------------------------------------------------------
EPI_ISL_413694       ------------------------------------------------------------
EPI_ISL_414692       ------------------------------------------------------------
EPI_ISL_424359       ------------------------------------------------------------
EPI_ISL_424396       ------------------------------------------------------------
EPI_ISL_424399       ------------------------------------------------------------
EPI_ISL_425530       ------------------------------------------------------------
EPI_ISL_425561       ------------------------------------------------------------
EPI_ISL_425614       ------------------------------------------------------------
EPI_ISL_425635       ------------------------------------------------------------
EPI_ISL_426848       ------------------------------------------------------------
EPI_ISL_426850       ------------------------------------------------------------
EPI_ISL_426955       ------------------------------------------------------------
```

EPI_ISL_426963   --

```
ORF1ab          3421 SFCYMHHMELPTGVHAGTDLEGNFYGPFVDRQTAQAAGTDTTITVNVLAWLYAAVINGDR
EPI_ISL_411066       ------------------------------------------------------------
EPI_ISL_413522       ------------------------------------------------------------
EPI_ISL_413558       ------------------------------------------------------------
EPI_ISL_413559       ------------------------------------------------------------
EPI_ISL_413561       ------------------------------------------------------------
EPI_ISL_413692       ------------------------------------------------------------
EPI_ISL_413693       ------------------------------------------------------------
EPI_ISL_413694       ------------------------------------------------------------
EPI_ISL_414692       ------------------------------------------------------------
EPI_ISL_424359       ------------------------------------------------------------
EPI_ISL_424396       ------------------------------------------------------------
EPI_ISL_424399       ------------------------------------------------------------
EPI_ISL_425530       ------------------------------------------------------------
EPI_ISL_425561       ------------------------------------------------------------
EPI_ISL_425614       ------------------------------------------------------------
EPI_ISL_425635       ------------------------------------------------------------
EPI_ISL_426848       ------------------------------------------------------------
EPI_ISL_426850       ------------------------------------------------------------
EPI_ISL_426955       ------------------------------------------------------------
EPI_ISL_426963       ------------------------------------------------------------

ORF1ab          3481 WFLNRFTTTLNDFNLVAMKYNYEPLTQDHVDILGPLSAQTGIAVLDMCASLKELLQNGMN
EPI_ISL_411066       ------------------------------------------------------------
EPI_ISL_413522       ------------------------------------------------------------
EPI_ISL_413558       ------------------------------------------------------------
EPI_ISL_413559       ------------------------------------------------------------
EPI_ISL_413561       ------------------------------------------------------------
EPI_ISL_413692       ------------------------------------------------------------
EPI_ISL_413693       ------------------------------------------------------------
EPI_ISL_413694       ------------------------------------------------------------
EPI_ISL_414692       ------------------------------------------------------------
EPI_ISL_424359       ------------------------------------------------------------
EPI_ISL_424396       ------------------------------------------------------------
EPI_ISL_424399       ------------------------------------------------------------
EPI_ISL_425530       ------------------------------------------------------------
EPI_ISL_425561       ------------------------------------------------------------
EPI_ISL_425614       ------------------------------------------------------------
EPI_ISL_425635       ------------------------------------------------------------
EPI_ISL_426848       ------------------------------------------------------------
EPI_ISL_426850       ------------------------------------------------------------
EPI_ISL_426955       ------------------------------------------------------------
EPI_ISL_426963       ------------------------------------------------------------

ORF1ab          3541 GRTILGSALLEDEFTPFDVVRQCSGVTFQSAVKRTIKGTHHWLLLTILTSLLVLVQSTQW
EPI_ISL_411066       ------------------------------------------------------------
EPI_ISL_413522       ------------------------------------------------------------
EPI_ISL_413558       ------------------------------------------------------------
EPI_ISL_413559       ------------------------------------------------------------
EPI_ISL_413561       ------------------------------------------------------------
EPI_ISL_413692       ------------------------------------------------------------
EPI_ISL_413693       ------------------------------------------------------------
EPI_ISL_413694       ------------------------------------------------------------
EPI_ISL_414692       ------------------------------------------------------------
EPI_ISL_424359       ------------------------------------------------------------
EPI_ISL_424396       ------------------------------------------------------------
EPI_ISL_424399       ------------------------------------------------------------
EPI_ISL_425530       ------------------------------------------------------------
EPI_ISL_425561       ------------------------------------------------------------
EPI_ISL_425614       ------------------------------------------------------------
EPI_ISL_425635       ------------------------------------------------------------
EPI_ISL_426848       ------------------------------------------------------------
EPI_ISL_426850       ------------------------------------------------------------
EPI_ISL_426955       ------------------------------------------------------------
```

EPI_ISL_426963   --

```
ORF1ab         3601 SLFFFLYENAFLPFAMGIIAMSAFAMMFVKHKHAFLCLFLLPSLATVAYFNMVYMPASWV
EPI_ISL_411066      ------------------------------------------------------------
EPI_ISL_413522      ------------------------------------------------------------
EPI_ISL_413558      ------------------------------------------------------------
EPI_ISL_413559      ------------------------------------------------------------
EPI_ISL_413561      ------------------------------------------------------------
EPI_ISL_413692      ------------------------------------------------------------
EPI_ISL_413693      ------------------------------------------------------------
EPI_ISL_413694      ------------------------------------------------------------
EPI_ISL_414692      ------------------------------------------------------------
EPI_ISL_424359      ------------------------------------------------------------
EPI_ISL_424396      ------------------------------------------------------------
EPI_ISL_424399      ------------------------------------------------------------
EPI_ISL_425530      ------------------------------------------------------------
EPI_ISL_425561      ------------------------------------------------------------
EPI_ISL_425614      ------------------------------------------------------------
EPI_ISL_425635      ------------------------------------------------------------
EPI_ISL_426848      ------------------------------------------------------------
EPI_ISL_426850      ------------------------------------------------------------
EPI_ISL_426955      ------------------------------------------------------------
EPI_ISL_426963      ------------------------------------------------------------

ORF1ab         3661 MRIMTWLDMVDTSLSGFKLKDCVMYASAVVLLILMTARTVYDDGARRVWTLMNVLTLVYK
EPI_ISL_411066      ------------------------------------------------------------
EPI_ISL_413522      ------------------------------------------------------------
EPI_ISL_413558      ------------------------------------------------------------
EPI_ISL_413559      ------------------------------------------------------------
EPI_ISL_413561      ------------------------------------------------------------
EPI_ISL_413692      ------------------------------------------------------------
EPI_ISL_413693      ------------------------------------------------------------
EPI_ISL_413694      ------------------------------------------------------------
EPI_ISL_414692      ------------------------------------------------------------
EPI_ISL_424359      ------------------------------------------------------------
EPI_ISL_424396      ------------------------------------------------------------
EPI_ISL_424399      ------------------------------------------------------------
EPI_ISL_425530      ------------------------------------------------------------
EPI_ISL_425561      ------------------------------------------------------------
EPI_ISL_425614      ------------------------------------------------------------
EPI_ISL_425635      ------------------------------------------------------------
EPI_ISL_426848      ------------------------------------------------------------
EPI_ISL_426850      ------------------------------------------------------------
EPI_ISL_426955      ------------------------------------------------------------
EPI_ISL_426963      ------------------------------------------------------------

ORF1ab         3721 VYYGNALDQAISMWALIISVTSNYSGVVTTVMFLARGIVFMCVEYCPIFFITGNTLQCIM
EPI_ISL_411066      ------------------------------------------------------------
EPI_ISL_413522      ------------------------------------------------------------
EPI_ISL_413558      ------------------------------------------------------------
EPI_ISL_413559      ------------------------------------------------------------
EPI_ISL_413561      ------------------------------------------------------------
EPI_ISL_413692      ------------------------------------------------------------
EPI_ISL_413693      ------------------------------------------------------------
EPI_ISL_413694      ------------------------------------------------------------
EPI_ISL_414692      ------------------------------------------------------------
EPI_ISL_424359      ------------------------------------------------------------
EPI_ISL_424396      ------------------------------------------------------------
EPI_ISL_424399      ------------------------------------------------------------
EPI_ISL_425530      ------------------------------------------------------------
EPI_ISL_425561      ------------------------------------------------------------
EPI_ISL_425614      ------------------------------------------------------------
EPI_ISL_425635      ------------------------------------------------------------
EPI_ISL_426848      ------------------------------------------------------------
EPI_ISL_426850      ------------------------------------------------------------
EPI_ISL_426955      ------------------------------------------------------------
```

EPI_ISL_426963 --

```
ORF1ab          3781 LVYCFLGYFCTCYFGLFCLLNRYFRLTLGVYDYLVSTQEFRYMNSQGLLPPKNSIDAFKL
EPI_ISL_411066       ------------------------------------------------------------
EPI_ISL_413522       ------------------------------------------------------------
EPI_ISL_413558       ------------------------------------------------------------
EPI_ISL_413559       ------------------------------------------------------------
EPI_ISL_413561       ------------------------------------------------------------
EPI_ISL_413692       ------------------------------------------------------------
EPI_ISL_413693       ------------------------------------------------------------
EPI_ISL_413694       ------------------------------------------------------------
EPI_ISL_414692       ------------------------------------------------------------
EPI_ISL_424359       ------------------------------------------------------------
EPI_ISL_424396       ------------------------------------------------------------
EPI_ISL_424399       ------------------------------------------------------------
EPI_ISL_425530       ------------------------------------------------------------
EPI_ISL_425561       ------------------------------------------------------------
EPI_ISL_425614       ------------------------------------------------------------
EPI_ISL_425635       ------------------------------------------------------------
EPI_ISL_426848       ------------------------------------------------------------
EPI_ISL_426850       ------------------------------------------------------------
EPI_ISL_426955       ------------------------------------------------------------
EPI_ISL_426963       ------------------------------------------------------------

ORF1ab          3841 NIKLLGVGGKPCIKVATVQSKMSDVKCTSVVLLSVLQQLRVESSSKLWAQCVQLHNDILL
EPI_ISL_411066       ------------------------------------------------------------
EPI_ISL_413522       ------------------------------------------------------------
EPI_ISL_413558       ------------------------------------------------------------
EPI_ISL_413559       ------------------------------------------------------------
EPI_ISL_413561       ------------------------------------------------------------
EPI_ISL_413692       ------------------------------------------------------------
EPI_ISL_413693       ------------------------------------------------------------
EPI_ISL_413694       ------------------------------------------------------------
EPI_ISL_414692       ------------------------------------------------------------
EPI_ISL_424359       ------------------------------------------------------------
EPI_ISL_424396       ------------------------------------------------------------
EPI_ISL_424399       ------------------------------------------------------------
EPI_ISL_425530       ------------------------------------------------------------
EPI_ISL_425561       ------------------------------------------------------------
EPI_ISL_425614       ------------------------------------------------------------
EPI_ISL_425635       ------------------------------------------------------------
EPI_ISL_426848       ------------------------------------------------------------
EPI_ISL_426850       ------------------------------------------------------------
EPI_ISL_426955       ------------------------------------------------------------
EPI_ISL_426963       ------------------------------------------------------------

ORF1ab          3901 AKDTTEAFEKMVSLLSVLLSMQGAVDINKLCEEMLDNRATLQAIASEFSSLPSYAAFATA
EPI_ISL_411066       ------------------------------------------------------------
EPI_ISL_413522       ------------------------------------------------------------
EPI_ISL_413558       ------------------------------------------------------------
EPI_ISL_413559       ------------------------------------------------------------
EPI_ISL_413561       ------------------------------------------------------------
EPI_ISL_413692       ------------------------------------------------------------
EPI_ISL_413693       ------------------------------------------------------------
EPI_ISL_413694       ------------------------------------------------------------
EPI_ISL_414692       ------------------------------------------------------------
EPI_ISL_424359       ------------------------------------------------------------
EPI_ISL_424396       ------------------------------------------------------------
EPI_ISL_424399       ------------------------------------------------------------
EPI_ISL_425530       ------------------------------------------------------------
EPI_ISL_425561       ------------------------------------------------------------
EPI_ISL_425614       ------------------------------------------------------------
EPI_ISL_425635       ------------------------------------------------------------
EPI_ISL_426848       ------------------------------------------------------------
EPI_ISL_426850       ------------------------------------------------------------
EPI_ISL_426955       ------------------------------------------------------------
```

EPI_ISL_426963 --

```
ORF1ab          3961 QEAYEQAVANGDSEVVLKKLKKSLNVAKSEFDRDAAMQRKLEKMADQAMTQMYKQARSED
EPI_ISL_411066       ------------------------------------------------------------
EPI_ISL_413522       ------------------------------------------------------------
EPI_ISL_413558       ------------------------------------------------------------
EPI_ISL_413559       ------------------------------------------------------------
EPI_ISL_413561       ------------------------------------------------------------
EPI_ISL_413692       ------------------------------------------------------------
EPI_ISL_413693       ------------------------------------------------------------
EPI_ISL_413694       ------------------------------------------------------------
EPI_ISL_414692       ------------------------------------------------------------
EPI_ISL_424359       ------------------------------------------------------------
EPI_ISL_424396       ------------------------------------------------------------
EPI_ISL_424399       ------------------------------------------------------------
EPI_ISL_425530       ------------------------------------------------------------
EPI_ISL_425561       ------------------------------------------------------------
EPI_ISL_425614       ------------------------------------------------------------
EPI_ISL_425635       ------------------------------------------------------------
EPI_ISL_426848       ------------------------------------------------------------
EPI_ISL_426850       ------------------------------------------------------------
EPI_ISL_426955       ------------------------------------------------------------
EPI_ISL_426963       ------------------------------------------------------------

ORF1ab          4021 KRAKVTSAMQTMLFTMLRKLDNDALNNIINNARDGCVPLNIIPLTTAAKLMVVIPDYNTY
EPI_ISL_411066       ------------------------------------------------------------
EPI_ISL_413522       ------------------------------------------------------------
EPI_ISL_413558       ------------------------------------------------------------
EPI_ISL_413559       ------------------------------------------------------------
EPI_ISL_413561       ------------------------------------------------------------
EPI_ISL_413692       ------------------------------------------------------------
EPI_ISL_413693       ------------------------------------------------------------
EPI_ISL_413694       ------------------------------------------------------------
EPI_ISL_414692       ------------------------------------------------------------
EPI_ISL_424359       ------------------------------------------------------------
EPI_ISL_424396       ------------------------------------------------------------
EPI_ISL_424399       ------------------------------------------------------------
EPI_ISL_425530       ------------------------------------------------------------
EPI_ISL_425561       ------------------------------------------------------------
EPI_ISL_425614       ------------------------------------------------------------
EPI_ISL_425635       ------------------------------------------------------------
EPI_ISL_426848       ------------------------------------------------------------
EPI_ISL_426850       ------------------------------------------------------------
EPI_ISL_426955       ------------------------------------------------------------
EPI_ISL_426963       ------------------------------------------------------------

ORF1ab          4081 KNTCDGTTFTYASALWEIQQVVDADSKIVQLSEISMDNSPNLAWPLIVTALRANSAVKLQ
EPI_ISL_411066       ------------------------------------------------------------
EPI_ISL_413522       ------------------------------------------------------------
EPI_ISL_413558       ------------------------------------------------------------
EPI_ISL_413559       ------------------------------------------------------------
EPI_ISL_413561       ------------------------------------------------------------
EPI_ISL_413692       ------------------------------------------------------------
EPI_ISL_413693       ------------------------------------------------------------
EPI_ISL_413694       ------------------------------------------------------------
EPI_ISL_414692       ------------------------------------------------------------
EPI_ISL_424359       ------------------------------------------------------------
EPI_ISL_424396       ------------------------------------------------------------
EPI_ISL_424399       ------------------------------------------------------------
EPI_ISL_425530       ------------------------------------------------------------
EPI_ISL_425561       ------------------------------------------------------------
EPI_ISL_425614       ------------------------------------------------------------
EPI_ISL_425635       ------------------------------------------------------------
EPI_ISL_426848       ------------------------------------------------------------
EPI_ISL_426850       ------------------------------------------------------------
EPI_ISL_426955       ------------------------------------------------------------
```

EPI_ISL_426963 --

```
ORF1ab          4141 NNELSPVALRQMSCAAGTTQTACTDDNALAYYNTTKGGRFVLALLSDLQDLKWARFPKSD
EPI_ISL_411066       ------------------------------------------------------------
EPI_ISL_413522       ------------------------------------------------------------
EPI_ISL_413558       ------------------------------------------------------------
EPI_ISL_413559       ------------------------------------------------------------
EPI_ISL_413561       ------------------------------------------------------------
EPI_ISL_413692       ------------------------------------------------------------
EPI_ISL_413693       ------------------------------------------------------------
EPI_ISL_413694       ------------------------------------------------------------
EPI_ISL_414692       ------------------------------------------------------------
EPI_ISL_424359       ------------------------------------------------------------
EPI_ISL_424396       ------------------------------------------------------------
EPI_ISL_424399       ------------------------------------------------------------
EPI_ISL_425530       ------------------------------------------------------------
EPI_ISL_425561       ------------------------------------------------------------
EPI_ISL_425614       ------------------------------------------------------------
EPI_ISL_425635       ------------------------------------------------------------
EPI_ISL_426848       ------------------------------------------------------------
EPI_ISL_426850       ------------------------------------------------------------
EPI_ISL_426955       ------------------------------------------------------------
EPI_ISL_426963       ------------------------------------------------------------

ORF1ab          4201 GTGTIYTELEPPCRFVTDTPKGPKVKYLYFIKGLNNLNRGMVLGSLAATVRLQAGNATEV
EPI_ISL_411066       ------------------------------------------------------------
EPI_ISL_413522       ------------------------------------------------------------
EPI_ISL_413558       ------------------------------------------------------------
EPI_ISL_413559       ------------------------------------------------------------
EPI_ISL_413561       ------------------------------------------------------------
EPI_ISL_413692       ------------------------------------------------------------
EPI_ISL_413693       ------------------------------------------------------------
EPI_ISL_413694       ------------------------------------------------------------
EPI_ISL_414692       ------------------------------------------------------------
EPI_ISL_424359       ------------------------------------------------------------
EPI_ISL_424396       ------------------------------------------------------------
EPI_ISL_424399       ------------------------------------------------------------
EPI_ISL_425530       ------------------------------------------------------------
EPI_ISL_425561       ------------------------------------------------------------
EPI_ISL_425614       ------------------------------------------------------------
EPI_ISL_425635       ------------------------------------------------------------
EPI_ISL_426848       ------------------------------------------------------------
EPI_ISL_426850       ------------------------------------------------------------
EPI_ISL_426955       ------------------------------------------------------------
EPI_ISL_426963       ------------------------------------------------------------

ORF1ab          4261 PANSTVLSFCAFAVDAAKAYKDYLASGGQPITNCVKMLCTHTGTGQAITVTPEANMDQES
EPI_ISL_411066       ------------------------------------------------------------
EPI_ISL_413522       ------------------------------------------------------------
EPI_ISL_413558       ------------------------------------------------------------
EPI_ISL_413559       ------------------------------------------------------------
EPI_ISL_413561       ------------------------------------------------------------
EPI_ISL_413692       ------------------------------------------------------------
EPI_ISL_413693       ------------------------------------------------------------
EPI_ISL_413694       ------------------------------------------------------------
EPI_ISL_414692       ------------------------------------------------------------
EPI_ISL_424359       ------------------------------------------------------------
EPI_ISL_424396       ------------------------------------------------------------
EPI_ISL_424399       ------------------------------------------------------------
EPI_ISL_425530       ------------------------------------------------------------
EPI_ISL_425561       ------------------------------------------------------------
EPI_ISL_425614       ------------------------------------------------------------
EPI_ISL_425635       ------------------------------------------------------------
EPI_ISL_426848       ------------------------------------------------------------
EPI_ISL_426850       ------------------------------------------------------------
EPI_ISL_426955       ------------------------------------------------------------
```

EPI_ISL_426963   --

```
ORF1ab          4321 FGGASCCLYCRCHIDHPNPKGFCDLKGKYVQIPTTCANDPVGFTLKNTVCTVCGMWKGYG
EPI_ISL_411066       ------------------------------------------------------------
EPI_ISL_413522       ------------------------------------------------------------
EPI_ISL_413558       ------------------------------------------------------------
EPI_ISL_413559       ------------------------------------------------------------
EPI_ISL_413561       ------------------------------------------------------------
EPI_ISL_413692       ------------------------------------------------------------
EPI_ISL_413693       ------------------------------------------------------------
EPI_ISL_413694       ------------------------------------------------------------
EPI_ISL_414692       ------------------------------------------------------------
EPI_ISL_424359       ------------------------------------------------------------
EPI_ISL_424396       ------------------------------------------------------------
EPI_ISL_424399       ------------------------------------------------------------
EPI_ISL_425530       ------------------------------------------------------------
EPI_ISL_425561       ------------------------------------------------------------
EPI_ISL_425614       ------------------------------------------------------------
EPI_ISL_425635       ------------------------------------------------------------
EPI_ISL_426848       ------------------------------------------------------------
EPI_ISL_426850       ------------------------------------------------------------
EPI_ISL_426955       ------------------------------------------------------------
EPI_ISL_426963       ------------------------------------------------------------

ORF1ab          4381 CSCDQLREPMLQSADAQSFLNRVCGVSAARLTPCGTGTSTDVVYRAFDIYNDKVAGFAKF
EPI_ISL_411066       ------------------------------------------------------------
EPI_ISL_413522       ------------------------------------------------------------
EPI_ISL_413558       ------------------------------------------------------------
EPI_ISL_413559       ------------------------------------------------------------
EPI_ISL_413561       ------------------------------------------------------------
EPI_ISL_413692       ------------------------------------------------------------
EPI_ISL_413693       ------------------------------------------------------------
EPI_ISL_413694       ------------------------------------------------------------
EPI_ISL_414692       ------------------------------------------------------------
EPI_ISL_424359       ------------------------------------------------------------
EPI_ISL_424396       ------------------------------------------------------------
EPI_ISL_424399       ------------------------------------------------------------
EPI_ISL_425530       ------------------------------------------------------------
EPI_ISL_425561       ------------------------------------------------------------
EPI_ISL_425614       ------------------------------------------------------------
EPI_ISL_425635       ------------------------------------------------------------
EPI_ISL_426848       ------------------------------------------------------------
EPI_ISL_426850       ------------------------------------------------------------
EPI_ISL_426955       ------------------------------------------------------------
EPI_ISL_426963       ------------------------------------------------------------

ORF1ab          4441 LKTNCCRFQEKDEDDNLIDSYFVVKRHTFSNYQHEETIYNLLKDCPAVAKHDFFKFRIDG
EPI_ISL_411066       ------------------------------------------------------------
EPI_ISL_413522       ------------------------------------------------------------
EPI_ISL_413558       ------------------------------------------------------------
EPI_ISL_413559       ------------------------------------------------------------
EPI_ISL_413561       ------------------------------------------------------------
EPI_ISL_413692       ------------------------------------------------------------
EPI_ISL_413693       ------------------------------------------------------------
EPI_ISL_413694       ------------------------------------------------------------
EPI_ISL_414692       ------------------------------------------------------------
EPI_ISL_424359       ------------------------------------------------------------
EPI_ISL_424396       ------------------------------------------------------------
EPI_ISL_424399       ------------------------------------------------------------
EPI_ISL_425530       ------------------------------------------------------------
EPI_ISL_425561       ------------------------------------------------------------
EPI_ISL_425614       ------------------------------------------------------------
EPI_ISL_425635       ------------------------------------------------------------
EPI_ISL_426848       ------------------------------------------------------------
EPI_ISL_426850       ------------------------------------------------------------
EPI_ISL_426955       ------------------------------------------------------------
```

EPI_ISL_426963 --

```
ORF1ab         4501 DMVPHISRQRLTKYTMADLVYALRHFDEGNCDTLKEILVTYNCCDDDYFNKKDWYDFVEN
EPI_ISL_411066      ------------------------------------------------------------
EPI_ISL_413522      ------------------------------------------------------------
EPI_ISL_413558      ------------------------------------------------------------
EPI_ISL_413559      ------------------------------------------------------------
EPI_ISL_413561      ------------------------------------------------------------
EPI_ISL_413692      ------------------------------------------------------------
EPI_ISL_413693      ------------------------------------------------------------
EPI_ISL_413694      ------------------------------------------------------------
EPI_ISL_414692      ------------------------------------------------------------
EPI_ISL_424359      ------------------------------------------------------------
EPI_ISL_424396      ------------------------------------------------------------
EPI_ISL_424399      ------------------------------------------------------------
EPI_ISL_425530      ------------------------------------------------------------
EPI_ISL_425561      ------------------------------------------------------------
EPI_ISL_425614      ------------------------------------------------------------
EPI_ISL_425635      ------------------------------------------------------------
EPI_ISL_426848      ------------------------------------------------------------
EPI_ISL_426850      ------------------------------------------------------------
EPI_ISL_426955      ------------------------------------------------------------
EPI_ISL_426963      ------------------------------------------------------------

ORF1ab         4561 PDILRVYANLGERVRQALLKTVQFCDAMRNAGIVGVLTLDNQDLNGNWYDFGDFIQTTPG
EPI_ISL_411066      ------------------------------------------------------------
EPI_ISL_413522      ------------------------------------------------------------
EPI_ISL_413558      ------------------------------------------------------------
EPI_ISL_413559      ------------------------------------------------------------
EPI_ISL_413561      ------------------------------------------------------------
EPI_ISL_413692      ------------------------------------------------------------
EPI_ISL_413693      ------------------------------------------------------------
EPI_ISL_413694      ------------------------------------------------------------
EPI_ISL_414692      ------------------------------------------------------------
EPI_ISL_424359      ------------------------------------------------------------
EPI_ISL_424396      ------------------------------------------------------------
EPI_ISL_424399      ------------------------------------------------------------
EPI_ISL_425530      ------------------------------------------------------------
EPI_ISL_425561      ------------------------------------------------------------
EPI_ISL_425614      ------------------------------------------------------------
EPI_ISL_425635      ------------------------------------------------------------
EPI_ISL_426848      ------------------------------------------------------------
EPI_ISL_426850      ------------------------------------------------------------
EPI_ISL_426955      ------------------------------------------------------------
EPI_ISL_426963      ------------------------------------------------------------

ORF1ab         4621 SGVPVVDSYYSLLMPILTLTRALTAESHVDTDLTKPYIKWDLLKYDFTEERLKLFDRYFK
EPI_ISL_411066      ------------------------------------------------------------
EPI_ISL_413522      ------------------------------------------------------------
EPI_ISL_413558      ------------------------------------------------------------
EPI_ISL_413559      ------------------------------------------------------------
EPI_ISL_413561      ------------------------------------------------------------
EPI_ISL_413692      ------------------------------------------------------------
EPI_ISL_413693      ------------------------------------------------------------
EPI_ISL_413694      ------------------------------------------------------------
EPI_ISL_414692      ------------------------------------------------------------
EPI_ISL_424359      ------------------------------------------------------------
EPI_ISL_424396      ------------------------------------------------------------
EPI_ISL_424399      ------------------------------------------------------------
EPI_ISL_425530      ------------------------------------------------------------
EPI_ISL_425561      ------------------------------------------------------------
EPI_ISL_425614      ------------------------------------------------------------
EPI_ISL_425635      ------------------------------------------------------------
EPI_ISL_426848      ------------------------------------------------------------
EPI_ISL_426850      ------------------------------------------------------------
EPI_ISL_426955      ------------------------------------------------------------
```

EPI_ISL_426963 --

```
ORF1ab          4681 YWDQTYHPNCVNCLDDRCILHCANFNVLFSTVFPPTSFGPLVRKIFVDGVPFVVSTGYHF
EPI_ISL_411066       ------------------------------------------------------------
EPI_ISL_413522       ------------------------------------------------------------
EPI_ISL_413558       ------------------------------------------------------------
EPI_ISL_413559       ------------------------------------------------------------
EPI_ISL_413561       ------------------------------------------------------------
EPI_ISL_413692       ------------------------------------------------------------
EPI_ISL_413693       ------------------------------------------------------------
EPI_ISL_413694       ------------------------------------------------------------
EPI_ISL_414692       ------------------------------------------------------------
EPI_ISL_424359       ------------------------------------------------------------
EPI_ISL_424396       ------------------------------------------------------------
EPI_ISL_424399       ------------------------------------------------------------
EPI_ISL_425530       ------------------------------------------------------------
EPI_ISL_425561       ------------------------------------------------------------
EPI_ISL_425614       ------------------------------------------------------------
EPI_ISL_425635       ------------------------------------------------------------
EPI_ISL_426848       ------------------------------------------------------------
EPI_ISL_426850       ------------------------------------------------------------
EPI_ISL_426955       ------------------------------------------------------------
EPI_ISL_426963       ------------------------------------------------------------

ORF1ab          4741 RELGVVHNQDVNLHSSRLSFKELLVYAADPAMHAASGNLLLDKRTTCFSVAALTNNVAFQ
EPI_ISL_411066       ------------------------------------------------------------
EPI_ISL_413522       ------------------------------------------------------------
EPI_ISL_413558       ------------------------------------------------------------
EPI_ISL_413559       ------------------------------------------------------------
EPI_ISL_413561       ------------------------------------------------------------
EPI_ISL_413692       ------------------------------------------------------------
EPI_ISL_413693       ------------------------------------------------------------
EPI_ISL_413694       ------------------------------------------------------------
EPI_ISL_414692       ------------------------------------------------------------
EPI_ISL_424359       ------------------------------------------------------------
EPI_ISL_424396       ------------------------------------------------------------
EPI_ISL_424399       ------------------------------------------------------------
EPI_ISL_425530       ------------------------------------------------------------
EPI_ISL_425561       ------------------------------------------------------------
EPI_ISL_425614       ------------------------------------------------------------
EPI_ISL_425635       ------------------------------------------------------------
EPI_ISL_426848       ------------------------------------------------------------
EPI_ISL_426850       ------------------------------------------------------------
EPI_ISL_426955       ------------------------------------------------------------
EPI_ISL_426963       ------------------------------------------------------------

ORF1ab          4801 TVKPGNFNKDFYDFAVSKGFFKEGSSVELKHFFFAQDGNAAISDYDYYRYNLPTMCDIRQ
EPI_ISL_411066       ------------------------------------------------------------
EPI_ISL_413522       ------------------------------------------------------------
EPI_ISL_413558       ------------------------------------------------------------
EPI_ISL_413559       ------------------------------------------------------------
EPI_ISL_413561       ------------------------------------------------------------
EPI_ISL_413692       ------------------------------------------------------------
EPI_ISL_413693       ------------------------------------------------------------
EPI_ISL_413694       ------------------------------------------------------------
EPI_ISL_414692       ------------------------------------------------------------
EPI_ISL_424359       ------------------------------------------------------------
EPI_ISL_424396       ------------------------------------------------------------
EPI_ISL_424399       ------------------------------------------------------------
EPI_ISL_425530       ------------------------------------------------------------
EPI_ISL_425561       ------------------------------------------------------------
EPI_ISL_425614       ------------------------------------------------------------
EPI_ISL_425635       ------------------------------------------------------------
EPI_ISL_426848       ------------------------------------------------------------
EPI_ISL_426850       ------------------------------------------------------------
EPI_ISL_426955       ------------------------------------------------------------
```

EPI_ISL_426963 --

```
ORF1ab         4861 LLFVVEVVDKYFDCYDGGCINANQVIVNNLDKSAGFPFNKWGKARLYYDSMSYEDQDALF
EPI_ISL_411066      ------------------------------------------------------------
EPI_ISL_413522      ------------------------------------------------------------
EPI_ISL_413558      ------------------------------------------------------------
EPI_ISL_413559      ------------------------------------------------------------
EPI_ISL_413561      ------------------------------------------------------------
EPI_ISL_413692      ------------------------------------------------------------
EPI_ISL_413693      ------------------------------------------------------------
EPI_ISL_413694      ------------------------------------------------------------
EPI_ISL_414692      ------------------------------------------------------------
EPI_ISL_424359      ------------------------------------------------------------
EPI_ISL_424396      ------------------------------------------------------------
EPI_ISL_424399      ------------------------------------------------------------
EPI_ISL_425530      ------------------------------------------------------------
EPI_ISL_425561      ------------------------------------------------------------
EPI_ISL_425614      ------------------------------------------------------------
EPI_ISL_425635      ------------------------------------------------------------
EPI_ISL_426848      ------------------------------------------------------------
EPI_ISL_426850      ------------------------------------------------------------
EPI_ISL_426955      ------------------------------------------------------------
EPI_ISL_426963      ------------------------------------------------------------

ORF1ab         4921 AYTKRNVIPTITQMNLKYAISAKNRARTVAGVSICSTMTNRQFHQKLLKSIAATRGATVV
EPI_ISL_411066      ------------------------------------------------------------
EPI_ISL_413522      ------------------------------------------------------------
EPI_ISL_413558      ------------------------------------------------------------
EPI_ISL_413559      ------------------------------------------------------------
EPI_ISL_413561      ------------------------------------------------------------
EPI_ISL_413692      ------------------------------------------------------------
EPI_ISL_413693      ------------------------------------------------------------
EPI_ISL_413694      ------------------------------------------------------------
EPI_ISL_414692      ------------------------------------------------------------
EPI_ISL_424359      ------------------------------------------------------------
EPI_ISL_424396      ------------------------------------------------------------
EPI_ISL_424399      ------------------------------------------------------------
EPI_ISL_425530      ------------------------------------------------------------
EPI_ISL_425561      ------------------------------------------------------------
EPI_ISL_425614      ------------------------------------------------------------
EPI_ISL_425635      ------------------------------------------------------------
EPI_ISL_426848      ------------------------------------------------------------
EPI_ISL_426850      ------------------------------------------------------------
EPI_ISL_426955      ------------------------------------------------------------
EPI_ISL_426963      ------------------------------------------------------------

ORF1ab         4981 IGTSKFYGGWHNMLKTVYSDVENPHLMGWDYPKCDRAMPNMLRIMASLVLARKHTTCCSL
EPI_ISL_411066      ------------------------------------------------------------
EPI_ISL_413522      ------------------------------------------------------------
EPI_ISL_413558      ------------------------------------------------------------
EPI_ISL_413559      ------------------------------------------------------------
EPI_ISL_413561      ------------------------------------------------------------
EPI_ISL_413692      ------------------------------------------------------------
EPI_ISL_413693      ------------------------------------------------------------
EPI_ISL_413694      ------------------------------------------------------------
EPI_ISL_414692      ------------------------------------------------------------
EPI_ISL_424359      ------------------------------------------------------------
EPI_ISL_424396      ------------------------------------------------------------
EPI_ISL_424399      ------------------------------------------------------------
EPI_ISL_425530      ------------------------------------------------------------
EPI_ISL_425561      ------------------------------------------------------------
EPI_ISL_425614      ------------------------------------------------------------
EPI_ISL_425635      ------------------------------------------------------------
EPI_ISL_426848      ------------------------------------------------------------
EPI_ISL_426850      ------------------------------------------------------------
EPI_ISL_426955      ------------------------------------------------------------
```

EPI_ISL_426963 --

```
ORF1ab          5041 SHRFYRLANECAQVLSEMVMCGGSLYVKPGGTSSGDATTAYANSVFNICQAVTANVNALL
EPI_ISL_411066       ------------------------------------------------------------
EPI_ISL_413522       ------------------------------------------------------------
EPI_ISL_413558       ------------------------------------------------------------
EPI_ISL_413559       ------------------------------------------------------------
EPI_ISL_413561       ------------------------------------------------------------
EPI_ISL_413692       ------------------------------------------------------------
EPI_ISL_413693       ------------------------------------------------------------
EPI_ISL_413694       ------------------------------------------------------------
EPI_ISL_414692       ------------------------------------------------------------
EPI_ISL_424359       ------------------------------------------------------------
EPI_ISL_424396       ------------------------------------------------------------
EPI_ISL_424399       ------------------------------------------------------------
EPI_ISL_425530       ------------------------------------------------------------
EPI_ISL_425561       ------------------------------------------------------------
EPI_ISL_425614       ------------------------------------------------------------
EPI_ISL_425635       ------------------------------------------------------------
EPI_ISL_426848       ------------------------------------------------------------
EPI_ISL_426850       ------------------------------------------------------------
EPI_ISL_426955       ------------------------------------------------------------
EPI_ISL_426963       ------------------------------------------------------------

ORF1ab          5101 STDGNKIADKYVRNLQHRLYECLYRNRDVDTDFVNEFYAYLRKHFSMMILSDDAVVCFNS
EPI_ISL_411066       ------------------------------------------------------------
EPI_ISL_413522       ------------------------------------------------------------
EPI_ISL_413558       ------------------------------------------------------------
EPI_ISL_413559       ------------------------------------------------------------
EPI_ISL_413561       ------------------------------------------------------------
EPI_ISL_413692       ------------------------------------------------------------
EPI_ISL_413693       ------------------------------------------------------------
EPI_ISL_413694       ------------------------------------------------------------
EPI_ISL_414692       ------------------------------------------------------------
EPI_ISL_424359       ------------------------------------------------------------
EPI_ISL_424396       ------------------------------------------------------------
EPI_ISL_424399       ------------------------------------------------------------
EPI_ISL_425530       ------------------------------------------------------------
EPI_ISL_425561       ------------------------------------------------------------
EPI_ISL_425614       ------------------------------------------------------------
EPI_ISL_425635       ------------------------------------------------------------
EPI_ISL_426848       ------------------------------------------------------------
EPI_ISL_426850       ------------------------------------------------------------
EPI_ISL_426955       ------------------------------------------------------------
EPI_ISL_426963       ------------------------------------------------------------

ORF1ab          5161 TYASQGLVASIKNFKSVLYYQNNVFMSEAKCWTETDLTKGPHEFCSQHTMLVKQGDDYVY
EPI_ISL_411066       ------------------------------------------------------------
EPI_ISL_413522       ------------------------------------------------------------
EPI_ISL_413558       ------------------------------------------------------------
EPI_ISL_413559       ------------------------------------------------------------
EPI_ISL_413561       ------------------------------------------------------------
EPI_ISL_413692       ------------------------------------------------------------
EPI_ISL_413693       ------------------------------------------------------------
EPI_ISL_413694       ------------------------------------------------------------
EPI_ISL_414692       ------------------------------------------------------------
EPI_ISL_424359       ------------------------------------------------------------
EPI_ISL_424396       ------------------------------------------------------------
EPI_ISL_424399       ------------------------------------------------------------
EPI_ISL_425530       ------------------------------------------------------------
EPI_ISL_425561       ------------------------------------------------------------
EPI_ISL_425614       ------------------------------------------------------------
EPI_ISL_425635       ------------------------------------------------------------
EPI_ISL_426848       ------------------------------------------------------------
EPI_ISL_426850       ------------------------------------------------------------
EPI_ISL_426955       ------------------------------------------------------------
```

EPI_ISL_426963 --

```
ORF1ab          5221 LPYPDPSRILGAGCFVDDIVKTDGTLMIERFVSLAIDAYPLTKHPNQEYADVFHLYLQYI
EPI_ISL_411066       ------------------------------------------------------------
EPI_ISL_413522       ------------------------------------------------------------
EPI_ISL_413558       ------------------------------------------------------------
EPI_ISL_413559       ------------------------------------------------------------
EPI_ISL_413561       ------------------------------------------------------------
EPI_ISL_413692       ------------------------------------------------------------
EPI_ISL_413693       ------------------------------------------------------------
EPI_ISL_413694       ------------------------------------------------------------
EPI_ISL_414692       ------------------------------------------------------------
EPI_ISL_424359       ------------------------------------------------------------
EPI_ISL_424396       ------------------------------------------------------------
EPI_ISL_424399       ------------------------------------------------------------
EPI_ISL_425530       ------------------------------------------------------------
EPI_ISL_425561       ------------------------------------------------------------
EPI_ISL_425614       ------------------------------------------------------------
EPI_ISL_425635       ------------------------------------------------------------
EPI_ISL_426848       ------------------------------------------------------------
EPI_ISL_426850       ------------------------------------------------------------
EPI_ISL_426955       ------------------------------------------------------------
EPI_ISL_426963       ------------------------------------------------------------

ORF1ab          5281 RKLHDELTGHMLDMYSVMLTNDNTSRYWEPEFYEAMYTPHTVLQAVGACVLCNSQTSLRC
EPI_ISL_411066       ------------------------------------------------------------
EPI_ISL_413522       ------------------------------------------------------------
EPI_ISL_413558       ------------------------------------------------------------
EPI_ISL_413559       ------------------------------------------------------------
EPI_ISL_413561       ------------------------------------------------------------
EPI_ISL_413692       ------------------------------------------------------------
EPI_ISL_413693       ------------------------------------------------------------
EPI_ISL_413694       ------------------------------------------------------------
EPI_ISL_414692       ------------------------------------------------------------
EPI_ISL_424359       ------------------------------------------------------------
EPI_ISL_424396       ------------------------------------------------------------
EPI_ISL_424399       ------------------------------------------------------------
EPI_ISL_425530       ------------------------------------------------------------
EPI_ISL_425561       ------------------------------------------------------------
EPI_ISL_425614       ------------------------------------------------------------
EPI_ISL_425635       ------------------------------------------------------------
EPI_ISL_426848       ------------------------------------------------------------
EPI_ISL_426850       ------------------------------------------------------------
EPI_ISL_426955       ------------------------------------------------------------
EPI_ISL_426963       ------------------------------------------------------------

ORF1ab          5341 GACIRRPFLCCKCCYDHVISTSHKLVLSVNPYVCNAPGCDVTDVTQLYLGGMSYYCKSHK
EPI_ISL_411066       ------------------------------------------------------------
EPI_ISL_413522       ------------------------------------------------------------
EPI_ISL_413558       ------------------------------------------------------------
EPI_ISL_413559       ------------------------------------------------------------
EPI_ISL_413561       ------------------------------------------------------------
EPI_ISL_413692       ------------------------------------------------------------
EPI_ISL_413693       ------------------------------------------------------------
EPI_ISL_413694       ------------------------------------------------------------
EPI_ISL_414692       ------------------------------------------------------------
EPI_ISL_424359       ------------------------------------------------------------
EPI_ISL_424396       ------------------------------------------------------------
EPI_ISL_424399       ------------------------------------------------------------
EPI_ISL_425530       ------------------------------------------------------------
EPI_ISL_425561       ------------------------------------------------------------
EPI_ISL_425614       ------------------------------------------------------------
EPI_ISL_425635       ------------------------------------------------------------
EPI_ISL_426848       ------------------------------------------------------------
EPI_ISL_426850       ------------------------------------------------------------
EPI_ISL_426955       ------------------------------------------------------------
```

EPI_ISL_426963 --

```
ORF1ab         5401 PPISFPLCANGQVFGLYKNTCVGSDNVTDFNAIATCDWTNAGDYILANTCTERLKLFAAE
EPI_ISL_411066      ------------------------------------------------------------
EPI_ISL_413522      ------------------------------------------------------------
EPI_ISL_413558      ------------------------------------------------------------
EPI_ISL_413559      ------------------------------------------------------------
EPI_ISL_413561      ------------------------------------------------------------
EPI_ISL_413692      ------------------------------------------------------------
EPI_ISL_413693      ------------------------------------------------------------
EPI_ISL_413694      ------------------------------------------------------------
EPI_ISL_414692      ------------------------------------------------------------
EPI_ISL_424359      ------------------------------------------------------------
EPI_ISL_424396      ------------------------------------------------------------
EPI_ISL_424399      ------------------------------------------------------------
EPI_ISL_425530      ------------------------------------------------------------
EPI_ISL_425561      ------------------------------------------------------------
EPI_ISL_425614      ------------------------------------------------------------
EPI_ISL_425635      ------------------------------------------------------------
EPI_ISL_426848      ------------------------------------------------------------
EPI_ISL_426850      ------------------------------------------------------------
EPI_ISL_426955      ------------------------------------------------------------
EPI_ISL_426963      ------------------------------------------------------------

ORF1ab         5461 TLKATEETFKLSYGIATVREVLSDRELHLSWEVGKPRPPLNRNYVFTGYRVTKNSKVQIG
EPI_ISL_411066      ------------------------------------------------------------
EPI_ISL_413522      ------------------------------------------------------------
EPI_ISL_413558      ------------------------------------------------------------
EPI_ISL_413559      ------------------------------------------------------------
EPI_ISL_413561      ------------------------------------------------------------
EPI_ISL_413692      ------------------------------------------------------------
EPI_ISL_413693      ------------------------------------------------------------
EPI_ISL_413694      ------------------------------------------------------------
EPI_ISL_414692      ------------------------------------------------------------
EPI_ISL_424359      ------------------------------------------------------------
EPI_ISL_424396      ------------------------------------------------------------
EPI_ISL_424399      ------------------------------------------------------------
EPI_ISL_425530      ------------------------------------------------------------
EPI_ISL_425561      ------------------------------------------------------------
EPI_ISL_425614      ------------------------------------------------------------
EPI_ISL_425635      ------------------------------------------------------------
EPI_ISL_426848      ------------------------------------------------------------
EPI_ISL_426850      ------------------------------------------------------------
EPI_ISL_426955      ------------------------------------------------------------
EPI_ISL_426963      ------------------------------------------------------------

ORF1ab         5521 EYTFEKGDYGDAVVYRGTTTYKLNVGDYFVLTSHTVMPLSAPTLVPQEHYVRITGLYPTL
EPI_ISL_411066      ------------------------------------------------------------
EPI_ISL_413522      ------------------------------------------------------------
EPI_ISL_413558      ------------------------------------------------------------
EPI_ISL_413559      ------------------------------------------------------------
EPI_ISL_413561      ------------------------------------------------------------
EPI_ISL_413692      ------------------------------------------------------------
EPI_ISL_413693      ------------------------------------------------------------
EPI_ISL_413694      ------------------------------------------------------------
EPI_ISL_414692      ------------------------------------------------------------
EPI_ISL_424359      ------------------------------------------------------------
EPI_ISL_424396      ------------------------------------------------------------
EPI_ISL_424399      ------------------------------------------------------------
EPI_ISL_425530      ------------------------------------------------------------
EPI_ISL_425561      ------------------------------------------------------------
EPI_ISL_425614      ------------------------------------------------------------
EPI_ISL_425635      ------------------------------------------------------------
EPI_ISL_426848      ------------------------------------------------------------
EPI_ISL_426850      ------------------------------------------------------------
EPI_ISL_426955      ------------------------------------------------------------
```

EPI_ISL_426963 --

```
ORF1ab          5581 NISDEFSSNVANYQKVGMQKYSTLQGPPGTGKSHFAIGLALYYPSARIVYTACSHAAVDA
EPI_ISL_411066       ------------------------------------------------------------
EPI_ISL_413522       ------------------------------------------------------------
EPI_ISL_413558       ------------------------------------------------------------
EPI_ISL_413559       ------------------------------------------------------------
EPI_ISL_413561       ------------------------------------------------------------
EPI_ISL_413692       ------------------------------------------------------------
EPI_ISL_413693       ------------------------------------------------------------
EPI_ISL_413694       ------------------------------------------------------------
EPI_ISL_414692       ------------------------------------------------------------
EPI_ISL_424359       ------------------------------------------------------------
EPI_ISL_424396       ------------------------------------------------------------
EPI_ISL_424399       ------------------------------------------------------------
EPI_ISL_425530       ------------------------------------------------------------
EPI_ISL_425561       ------------------------------------------------------------
EPI_ISL_425614       ------------------------------------------------------------
EPI_ISL_425635       ------------------------------------------------------------
EPI_ISL_426848       ------------------------------------------------------------
EPI_ISL_426850       ------------------------------------------------------------
EPI_ISL_426955       ------------------------------------------------------------
EPI_ISL_426963       ------------------------------------------------------------

ORF1ab          5641 LCEKALKYLPIDKCSRIIPARARVECFDKFKVNSTLEQYVFCTVNALPETTADIVVFDEI
EPI_ISL_411066       ------------------------------------------------------------
EPI_ISL_413522       ------------------------------------------------------------
EPI_ISL_413558       ------------------------------------------------------------
EPI_ISL_413559       ------------------------------------------------------------
EPI_ISL_413561       ------------------------------------------------------------
EPI_ISL_413692       ------------------------------------------------------------
EPI_ISL_413693       ------------------------------------------------------------
EPI_ISL_413694       ------------------------------------------------------------
EPI_ISL_414692       ------------------------------------------------------------
EPI_ISL_424359       ------------------------------------------------------------
EPI_ISL_424396       ------------------------------------------------------------
EPI_ISL_424399       ------------------------------------------------------------
EPI_ISL_425530       ------------------------------------------------------------
EPI_ISL_425561       ------------------------------------------------------------
EPI_ISL_425614       ------------------------------------------------------------
EPI_ISL_425635       ------------------------------------------------------------
EPI_ISL_426848       ------------------------------------------------------------
EPI_ISL_426850       ------------------------------------------------------------
EPI_ISL_426955       ------------------------------------------------------------
EPI_ISL_426963       ------------------------------------------------------------

ORF1ab          5701 SMATNYDLSVVNARLRAKHYVYIGDPAQLPAPRTLLTKGTLEPEYFNSVCRLMKTIGPDM
EPI_ISL_411066       ------------------------------------------------------------
EPI_ISL_413522       ------------------------------------------------------------
EPI_ISL_413558       ------------------------------------------------------------
EPI_ISL_413559       ------------------------------------------------------------
EPI_ISL_413561       ------------------------------------------------------------
EPI_ISL_413692       ------------------------------------------------------------
EPI_ISL_413693       ------------------------------------------------------------
EPI_ISL_413694       ------------------------------------------------------------
EPI_ISL_414692       ------------------------------------------------------------
EPI_ISL_424359       ------------------------------------------------------------
EPI_ISL_424396       ------------------------------------------------------------
EPI_ISL_424399       ------------------------------------------------------------
EPI_ISL_425530       ------------------------------------------------------------
EPI_ISL_425561       ------------------------------------------------------------
EPI_ISL_425614       ------------------------------------------------------------
EPI_ISL_425635       ------------------------------------------------------------
EPI_ISL_426848       ------------------------------------------------------------
EPI_ISL_426850       ------------------------------------------------------------
EPI_ISL_426955       ------------------------------------------------------------
```

EPI_ISL_426963   --

```
ORF1ab          5761 FLGTCRRCPAEIVDTVSALVYDNKLKAHKDKSAQCFKMFYKGVITHDVSSAINRPQIGVV
EPI_ISL_411066       ------------------------------------------------------------
EPI_ISL_413522       ------------------------------------------------------------
EPI_ISL_413558       ------------------------------------------------------------
EPI_ISL_413559       ------------------------------------------------------------
EPI_ISL_413561       ------------------------------------------------------------
EPI_ISL_413692       ------------------------------------------------------------
EPI_ISL_413693       ------------------------------------------------------------
EPI_ISL_413694       ------------------------------------------------------------
EPI_ISL_414692       ------------------------------------------------------------
EPI_ISL_424359       ------------------------------------------------------------
EPI_ISL_424396       ------------------------------------------------------------
EPI_ISL_424399       ------------------------------------------------------------
EPI_ISL_425530       ------------------------------------------------------------
EPI_ISL_425561       ------------------------------------------------------------
EPI_ISL_425614       ------------------------------------------------------------
EPI_ISL_425635       ------------------------------------------------------------
EPI_ISL_426848       ------------------------------------------------------------
EPI_ISL_426850       ------------------------------------------------------------
EPI_ISL_426955       ------------------------------------------------------------
EPI_ISL_426963       ------------------------------------------------------------

ORF1ab          5821 REFLTRNPAWRKAVFISPYNSQNAVASKILGLPTQTVDSSQGSEYDYVIFTQTTETAHSC
EPI_ISL_411066       ------------------------------------------------------------
EPI_ISL_413522       ------------------------------------------------------------
EPI_ISL_413558       ------------------------------------------------------------
EPI_ISL_413559       ------------------------------------------------------------
EPI_ISL_413561       ------------------------------------------------------------
EPI_ISL_413692       ------------------------------------------------------------
EPI_ISL_413693       ------------------------------------------------------------
EPI_ISL_413694       ------------------------------------------------------------
EPI_ISL_414692       ------------------------------------------------------------
EPI_ISL_424359       ------------------------------------------------------------
EPI_ISL_424396       ------------------------------------------------------------
EPI_ISL_424399       ------------------------------------------------------------
EPI_ISL_425530       ------------------------------------------------------------
EPI_ISL_425561       ------------------------------------------------------------
EPI_ISL_425614       ------------------------------------------------------------
EPI_ISL_425635       ------------------------------------------------------------
EPI_ISL_426848       ------------------------------------------------------------
EPI_ISL_426850       ------------------------------------------------------------
EPI_ISL_426955       ------------------------------------------------------------
EPI_ISL_426963       ------------------------------------------------------------

ORF1ab          5881 NVNRFNVAITRAKVGILCIMSDRDLYDKLQFTSLEIPRRNVATLQAENVTGLFKDCSKVI
EPI_ISL_411066       ------------------------------------------------------------
EPI_ISL_413522       ------------------------------------------------------------
EPI_ISL_413558       ------------------------------------------------------------
EPI_ISL_413559       ------------------------------------------------------------
EPI_ISL_413561       ------------------------------------------------------------
EPI_ISL_413692       ------------------------------------------------------------
EPI_ISL_413693       ------------------------------------------------------------
EPI_ISL_413694       ------------------------------------------------------------
EPI_ISL_414692       ------------------------------------------------------------
EPI_ISL_424359       ------------------------------------------------------------
EPI_ISL_424396       ------------------------------------------------------------
EPI_ISL_424399       ------------------------------------------------------------
EPI_ISL_425530       ------------------------------------------------------------
EPI_ISL_425561       ------------------------------------------------------------
EPI_ISL_425614       ------------------------------------------------------------
EPI_ISL_425635       ------------------------------------------------------------
EPI_ISL_426848       ------------------------------------------------------------
EPI_ISL_426850       ------------------------------------------------------------
EPI_ISL_426955       ------------------------------------------------------------
```

EPI_ISL_426963 --

```
ORF1ab          5941 TGLHPTQAPTHLSVDTKFKTEGLCVDIPGIPKDMTYRRLISMMGFKMNYQVNGYPNMFIT
EPI_ISL_411066       ------------------------------------------------------------
EPI_ISL_413522       ------------------------------------------------------------
EPI_ISL_413558       ------------------------------------------------------------
EPI_ISL_413559       ------------------------------------------------------------
EPI_ISL_413561       ------------------------------------------------------------
EPI_ISL_413692       ------------------------------------------------------------
EPI_ISL_413693       ------------------------------------------------------------
EPI_ISL_413694       ------------------------------------------------------------
EPI_ISL_414692       ------------------------------------------------------------
EPI_ISL_424359       ------------------------------------------------------------
EPI_ISL_424396       ------------------------------------------------------------
EPI_ISL_424399       ------------------------------------------------------------
EPI_ISL_425530       ------------------------------------------------------------
EPI_ISL_425561       ------------------------------------------------------------
EPI_ISL_425614       ------------------------------------------------------------
EPI_ISL_425635       ------------------------------------------------------------
EPI_ISL_426848       ------------------------------------------------------------
EPI_ISL_426850       ------------------------------------------------------------
EPI_ISL_426955       ------------------------------------------------------------
EPI_ISL_426963       ------------------------------------------------------------

ORF1ab          6001 REEAIRHVRAWIGFDVEGCHATREAVGTNLPLQLGFSTGVNLVAVPTGYVDTPNNTDFSR
EPI_ISL_411066       ------------------------------------------------------------
EPI_ISL_413522       ------------------------------------------------------------
EPI_ISL_413558       ------------------------------------------------------------
EPI_ISL_413559       ------------------------------------------------------------
EPI_ISL_413561       ------------------------------------------------------------
EPI_ISL_413692       ------------------------------------------------------------
EPI_ISL_413693       ------------------------------------------------------------
EPI_ISL_413694       ------------------------------------------------------------
EPI_ISL_414692       ------------------------------------------------------------
EPI_ISL_424359       ------------------------------------------------------------
EPI_ISL_424396       ------------------------------------------------------------
EPI_ISL_424399       ------------------------------------------------------------
EPI_ISL_425530       ------------------------------------------------------------
EPI_ISL_425561       ------------------------------------------------------------
EPI_ISL_425614       ------------------------------------------------------------
EPI_ISL_425635       ------------------------------------------------------------
EPI_ISL_426848       ------------------------------------------------------------
EPI_ISL_426850       ------------------------------------------------------------
EPI_ISL_426955       ------------------------------------------------------------
EPI_ISL_426963       ------------------------------------------------------------

ORF1ab          6061 VSAKPPPGDQFKHLIPLMYKGLPWNVVRIKIVQMLSDTLKNLSDRVVFVLWAHGFELTSM
EPI_ISL_411066       ------------------------------------------------------------
EPI_ISL_413522       ------------------------------------------------------------
EPI_ISL_413558       ------------------------------------------------------------
EPI_ISL_413559       ------------------------------------------------------------
EPI_ISL_413561       ------------------------------------------------------------
EPI_ISL_413692       ------------------------------------------------------------
EPI_ISL_413693       ------------------------------------------------------------
EPI_ISL_413694       ------------------------------------------------------------
EPI_ISL_414692       ------------------------------------------------------------
EPI_ISL_424359       ------------------------------------------------------------
EPI_ISL_424396       ------------------------------------------------------------
EPI_ISL_424399       ------------------------------------------------------------
EPI_ISL_425530       ------------------------------------------------------------
EPI_ISL_425561       ------------------------------------------------------------
EPI_ISL_425614       ------------------------------------------------------------
EPI_ISL_425635       ------------------------------------------------------------
EPI_ISL_426848       ------------------------------------------------------------
EPI_ISL_426850       ------------------------------------------------------------
EPI_ISL_426955       ------------------------------------------------------------
```

EPI_ISL_426963 --

```
ORF1ab          6121 KYFVKIGPERTCCLCDRRATCFSTASDTYACWHHSIGFDYVYNPFMIDVQQWGFTGNLQS
EPI_ISL_411066       ------------------------------------------------------------
EPI_ISL_413522       ------------------------------------------------------------
EPI_ISL_413558       ------------------------------------------------------------
EPI_ISL_413559       ------------------------------------------------------------
EPI_ISL_413561       ------------------------------------------------------------
EPI_ISL_413692       ------------------------------------------------------------
EPI_ISL_413693       ------------------------------------------------------------
EPI_ISL_413694       ------------------------------------------------------------
EPI_ISL_414692       ------------------------------------------------------------
EPI_ISL_424359       ------------------------------------------------------------
EPI_ISL_424396       ------------------------------------------------------------
EPI_ISL_424399       ------------------------------------------------------------
EPI_ISL_425530       ------------------------------------------------------------
EPI_ISL_425561       ------------------------------------------------------------
EPI_ISL_425614       ------------------------------------------------------------
EPI_ISL_425635       ------------------------------------------------------------
EPI_ISL_426848       ------------------------------------------------------------
EPI_ISL_426850       ------------------------------------------------------------
EPI_ISL_426955       ------------------------------------------------------------
EPI_ISL_426963       ------------------------------------------------------------

ORF1ab          6181 NHDLYCQVHGNAHVASCDAIMTRCLAVHECFVKRVDWTIEYPIIGDELKINAACRKVQHM
EPI_ISL_411066       ------------------------------------------------------------
EPI_ISL_413522       ------------------------------------------------------------
EPI_ISL_413558       ------------------------------------------------------------
EPI_ISL_413559       ------------------------------------------------------------
EPI_ISL_413561       ------------------------------------------------------------
EPI_ISL_413692       ------------------------------------------------------------
EPI_ISL_413693       ------------------------------------------------------------
EPI_ISL_413694       ------------------------------------------------------------
EPI_ISL_414692       ------------------------------------------------------------
EPI_ISL_424359       ------------------------------------------------------------
EPI_ISL_424396       ------------------------------------------------------------
EPI_ISL_424399       ------------------------------------------------------------
EPI_ISL_425530       ------------------------------------------------------------
EPI_ISL_425561       ------------------------------------------------------------
EPI_ISL_425614       ------------------------------------------------------------
EPI_ISL_425635       ------------------------------------------------------------
EPI_ISL_426848       ------------------------------------------------------------
EPI_ISL_426850       ------------------------------------------------------------
EPI_ISL_426955       ------------------------------------------------------------
EPI_ISL_426963       ------------------------------------------------------------

ORF1ab          6241 VVKAALLADKFPVLHDIGNPKAIKCVPQADVEWKFYDAQPCSDKAYKIEELFYSYATHSD
EPI_ISL_411066       ------------------------------------------------------------
EPI_ISL_413522       ------------------------------------------------------------
EPI_ISL_413558       ------------------------------------------------------------
EPI_ISL_413559       ------------------------------------------------------------
EPI_ISL_413561       ------------------------------------------------------------
EPI_ISL_413692       ------------------------------------------------------------
EPI_ISL_413693       ------------------------------------------------------------
EPI_ISL_413694       ------------------------------------------------------------
EPI_ISL_414692       ------------------------------------------------------------
EPI_ISL_424359       ------------------------------------------------------------
EPI_ISL_424396       ------------------------------------------------------------
EPI_ISL_424399       ------------------------------------------------------------
EPI_ISL_425530       ------------------------------------------------------------
EPI_ISL_425561       ------------------------------------------------------------
EPI_ISL_425614       ------------------------------------------------------------
EPI_ISL_425635       ------------------------------------------------------------
EPI_ISL_426848       ------------------------------------------------------------
EPI_ISL_426850       ------------------------------------------------------------
EPI_ISL_426955       ------------------------------------------------------------
```

EPI_ISL_426963 --

```
ORF1ab          6301 KFTDGVCLFWNCNVDRYPANSIVCRFDTRVLSNLNLPGCDGGSLYVNKHAFHTPAFDKSA
EPI_ISL_411066       ------------------------------------------------------------
EPI_ISL_413522       ------------------------------------------------------------
EPI_ISL_413558       ------------------------------------------------------------
EPI_ISL_413559       ------------------------------------------------------------
EPI_ISL_413561       ------------------------------------------------------------
EPI_ISL_413692       ------------------------------------------------------------
EPI_ISL_413693       ------------------------------------------------------------
EPI_ISL_413694       ------------------------------------------------------------
EPI_ISL_414692       ------------------------------------------------------------
EPI_ISL_424359       ------------------------------------------------------------
EPI_ISL_424396       ------------------------------------------------------------
EPI_ISL_424399       ------------------------------------------------------------
EPI_ISL_425530       ------------------------------------------------------------
EPI_ISL_425561       ------------------------------------------------------------
EPI_ISL_425614       ------------------------------------------------------------
EPI_ISL_425635       ------------------------------------------------------------
EPI_ISL_426848       ------------------------------------------------------------
EPI_ISL_426850       ------------------------------------------------------------
EPI_ISL_426955       ------------------------------------------------------------
EPI_ISL_426963       ------------------------------------------------------------

ORF1ab          6361 FVNLKQLPFFYYSDSPCESHGKQVVSDIDYVPLKSATCITRCNLGGAVCRHHANEYRLYL
EPI_ISL_411066       ------------------------------------------------------------
EPI_ISL_413522       ------------------------------------------------------------
EPI_ISL_413558       ------------------------------------------------------------
EPI_ISL_413559       ------------------------------------------------------------
EPI_ISL_413561       ------------------------------------------------------------
EPI_ISL_413692       ------------------------------------------------------------
EPI_ISL_413693       ------------------------------------------------------------
EPI_ISL_413694       ------------------------------------------------------------
EPI_ISL_414692       ------------------------------------------------------------
EPI_ISL_424359       ------------------------------------------------------------
EPI_ISL_424396       ------------------------------------------------------------
EPI_ISL_424399       ------------------------------------------------------------
EPI_ISL_425530       ------------------------------------------------------------
EPI_ISL_425561       ------------------------------------------------------------
EPI_ISL_425614       ------------------------------------------------------------
EPI_ISL_425635       ------------------------------------------------------------
EPI_ISL_426848       ------------------------------------------------------------
EPI_ISL_426850       ------------------------------------------------------------
EPI_ISL_426955       ------------------------------------------------------------
EPI_ISL_426963       ------------------------------------------------------------

ORF1ab          6421 DAYNMMISAGFSLWVYKQFDTYNLWNTFTRLQSLENVAFNVVNKGHFDGQQGEVPVSIIN
EPI_ISL_411066       ------------------------------------------------------------
EPI_ISL_413522       ------------------------------------------------------------
EPI_ISL_413558       ------------------------------------------------------------
EPI_ISL_413559       ------------------------------------------------------------
EPI_ISL_413561       ------------------------------------------------------------
EPI_ISL_413692       ------------------------------------------------------------
EPI_ISL_413693       ------------------------------------------------------------
EPI_ISL_413694       ------------------------------------------------------------
EPI_ISL_414692       ------------------------------------------------------------
EPI_ISL_424359       ------------------------------------------------------------
EPI_ISL_424396       ------------------------------------------------------------
EPI_ISL_424399       ------------------------------------------------------------
EPI_ISL_425530       ------------------------------------------------------------
EPI_ISL_425561       ------------------------------------------------------------
EPI_ISL_425614       ------------------------------------------------------------
EPI_ISL_425635       ------------------------------------------------------------
EPI_ISL_426848       ------------------------------------------------------------
EPI_ISL_426850       ------------------------------------------------------------
EPI_ISL_426955       ------------------------------------------------------------
```

EPI_ISL_426963 --

```
ORF1ab          6481 NTVYTKVDGVDVELFENKTTLPVNVAFELWAKRNIKPVPEVKILNNLGVDIAANTVIWDY
EPI_ISL_411066       ------------------------------------------------------------
EPI_ISL_413522       ------------------------------------------------------------
EPI_ISL_413558       ------------------------------------------------------------
EPI_ISL_413559       ------------------------------------------------------------
EPI_ISL_413561       ------------------------------------------------------------
EPI_ISL_413692       ------------------------------------------------------------
EPI_ISL_413693       ------------------------------------------------------------
EPI_ISL_413694       ------------------------------------------------------------
EPI_ISL_414692       ------------------------------------------------------------
EPI_ISL_424359       ------------------------------------------------------------
EPI_ISL_424396       ------------------------------------------------------------
EPI_ISL_424399       ------------------------------------------------------------
EPI_ISL_425530       ------------------------------------------------------------
EPI_ISL_425561       ------------------------------------------------------------
EPI_ISL_425614       ------------------------------------------------------------
EPI_ISL_425635       ------------------------------------------------------------
EPI_ISL_426848       ------------------------------------------------------------
EPI_ISL_426850       ------------------------------------------------------------
EPI_ISL_426955       ------------------------------------------------------------
EPI_ISL_426963       ------------------------------------------------------------

ORF1ab          6541 KRDAPAHISTIGVCSMTDIAKKPTETICAPLTVFFDGRVDGQVDLFRNARNGVLITEGSV
EPI_ISL_411066       ------------------------------------------------------------
EPI_ISL_413522       ------------------------------------------------------------
EPI_ISL_413558       ------------------------------------------------------------
EPI_ISL_413559       ------------------------------------------------------------
EPI_ISL_413561       ------------------------------------------------------------
EPI_ISL_413692       ------------------------------------------------------------
EPI_ISL_413693       ------------------------------------------------------------
EPI_ISL_413694       ------------------------------------------------------------
EPI_ISL_414692       ------------------------------------------------------------
EPI_ISL_424359       ------------------------------------------------------------
EPI_ISL_424396       ------------------------------------------------------------
EPI_ISL_424399       ------------------------------------------------------------
EPI_ISL_425530       ------------------------------------------------------------
EPI_ISL_425561       ------------------------------------------------------------
EPI_ISL_425614       ------------------------------------------------------------
EPI_ISL_425635       ------------------------------------------------------------
EPI_ISL_426848       ------------------------------------------------------------
EPI_ISL_426850       ------------------------------------------------------------
EPI_ISL_426955       ------------------------------------------------------------
EPI_ISL_426963       ------------------------------------------------------------

ORF1ab          6601 KGLQPSVGPKQASLNGVTLIGEAVKTQFNYYKKVDGVVQQLPETYFTQSRNLQEFKPRSQ
EPI_ISL_411066       ------------------------------------------------------------
EPI_ISL_413522       ------------------------------------------------------------
EPI_ISL_413558       ------------------------------------------------------------
EPI_ISL_413559       ------------------------------------------------------------
EPI_ISL_413561       ------------------------------------------------------------
EPI_ISL_413692       ------------------------------------------------------------
EPI_ISL_413693       ------------------------------------------------------------
EPI_ISL_413694       ------------------------------------------------------------
EPI_ISL_414692       ------------------------------------------------------------
EPI_ISL_424359       ------------------------------------------------------------
EPI_ISL_424396       ------------------------------------------------------------
EPI_ISL_424399       ------------------------------------------------------------
EPI_ISL_425530       ------------------------------------------------------------
EPI_ISL_425561       ------------------------------------------------------------
EPI_ISL_425614       ------------------------------------------------------------
EPI_ISL_425635       ------------------------------------------------------------
EPI_ISL_426848       ------------------------------------------------------------
EPI_ISL_426850       ------------------------------------------------------------
EPI_ISL_426955       ------------------------------------------------------------
```

EPI_ISL_426963 --

```
ORF1ab         6661 MEIDFLELAMDEFIERYKLEGYAFEHIVYGDFSHSQLGGLHLLIGLAKRFKESPFELEDF
EPI_ISL_411066      ------------------------------------------------------------
EPI_ISL_413522      ------------------------------------------------------------
EPI_ISL_413558      ------------------------------------------------------------
EPI_ISL_413559      ------------------------------------------------------------
EPI_ISL_413561      ------------------------------------------------------------
EPI_ISL_413692      ------------------------------------------------------------
EPI_ISL_413693      ------------------------------------------------------------
EPI_ISL_413694      ------------------------------------------------------------
EPI_ISL_414692      ------------------------------------------------------------
EPI_ISL_424359      ------------------------------------------------------------
EPI_ISL_424396      ------------------------------------------------------------
EPI_ISL_424399      ------------------------------------------------------------
EPI_ISL_425530      ------------------------------------------------------------
EPI_ISL_425561      ------------------------------------------------------------
EPI_ISL_425614      ------------------------------------------------------------
EPI_ISL_425635      ------------------------------------------------------------
EPI_ISL_426848      ------------------------------------------------------------
EPI_ISL_426850      ------------------------------------------------------------
EPI_ISL_426955      ------------------------------------------------------------
EPI_ISL_426963      ------------------------------------------------------------

ORF1ab         6721 IPMDSTVKNYFITDAQTGSSKCVCSVIDLLLDDFVEIIKSQDLSVVSKVVKVTIDYTEIS
EPI_ISL_411066      ------------------------------------------------------------
EPI_ISL_413522      ------------------------------------------------------------
EPI_ISL_413558      ------------------------------------------------------------
EPI_ISL_413559      ------------------------------------------------------------
EPI_ISL_413561      ------------------------------------------------------------
EPI_ISL_413692      ------------------------------------------------------------
EPI_ISL_413693      ------------------------------------------------------------
EPI_ISL_413694      ------------------------------------------------------------
EPI_ISL_414692      ------------------------------------------------------------
EPI_ISL_424359      ------------------------------------------------------------
EPI_ISL_424396      ------------------------------------------------------------
EPI_ISL_424399      ------------------------------------------------------------
EPI_ISL_425530      ------------------------------------------------------------
EPI_ISL_425561      ------------------------------------------------------------
EPI_ISL_425614      ------------------------------------------------------------
EPI_ISL_425635      ------------------------------------------------------------
EPI_ISL_426848      ------------------------------------------------------------
EPI_ISL_426850      ------------------------------------------------------------
EPI_ISL_426955      ------------------------------------------------------------
EPI_ISL_426963      ------------------------------------------------------------

ORF1ab         6781 FMLWCKDGHVETFYPKLQSSQAWQPGVAMPNLYKMQRMLLEKCDLQNYGDSATLPKGIMM
EPI_ISL_411066      ------------------------------------------------------------
EPI_ISL_413522      ------------------------------------------------------------
EPI_ISL_413558      ------------------------------------------------------------
EPI_ISL_413559      ------------------------------------------------------------
EPI_ISL_413561      ------------------------------------------------------------
EPI_ISL_413692      ------------------------------------------------------------
EPI_ISL_413693      ------------------------------------------------------------
EPI_ISL_413694      ------------------------------------------------------------
EPI_ISL_414692      ------------------------------------------------------------
EPI_ISL_424359      ------------------------------------------------------------
EPI_ISL_424396      ------------------------------------------------------------
EPI_ISL_424399      ------------------------------------------------------------
EPI_ISL_425530      ------------------------------------------------------------
EPI_ISL_425561      ------------------------------------------------------------
EPI_ISL_425614      ------------------------------------------------------------
EPI_ISL_425635      ------------------------------------------------------------
EPI_ISL_426848      ------------------------------------------------------------
EPI_ISL_426850      ------------------------------------------------------------
EPI_ISL_426955      ------------------------------------------------------------
```

EPI_ISL_426963 --

```
ORF1ab          6841 NVAKYTQLCQYLNTLTLAVPYNMRVIHFGAGSDKGVAPGTAVLRQWLPTGTLLVDSDLND
EPI_ISL_411066       ------------------------------------------------------------
EPI_ISL_413522       ------------------------------------------------------------
EPI_ISL_413558       ------------------------------------------------------------
EPI_ISL_413559       ------------------------------------------------------------
EPI_ISL_413561       ------------------------------------------------------------
EPI_ISL_413692       ------------------------------------------------------------
EPI_ISL_413693       ------------------------------------------------------------
EPI_ISL_413694       ------------------------------------------------------------
EPI_ISL_414692       ------------------------------------------------------------
EPI_ISL_424359       ------------------------------------------------------------
EPI_ISL_424396       ------------------------------------------------------------
EPI_ISL_424399       ------------------------------------------------------------
EPI_ISL_425530       ------------------------------------------------------------
EPI_ISL_425561       ------------------------------------------------------------
EPI_ISL_425614       ------------------------------------------------------------
EPI_ISL_425635       ------------------------------------------------------------
EPI_ISL_426848       ------------------------------------------------------------
EPI_ISL_426850       ------------------------------------------------------------
EPI_ISL_426955       ------------------------------------------------------------
EPI_ISL_426963       ------------------------------------------------------------

ORF1ab          6901 FVSDADSTLIGDCATVHTANKWDLIISDMYDPKTKNVTKENDSKEGFFTYICGFIQQKLA
EPI_ISL_411066       ------------------------------------------------------------
EPI_ISL_413522       ------------------------------------------------------------
EPI_ISL_413558       ------------------------------------------------------------
EPI_ISL_413559       ------------------------------------------------------------
EPI_ISL_413561       ------------------------------------------------------------
EPI_ISL_413692       ------------------------------------------------------------
EPI_ISL_413693       ------------------------------------------------------------
EPI_ISL_413694       ------------------------------------------------------------
EPI_ISL_414692       ------------------------------------------------------------
EPI_ISL_424359       ------------------------------------------------------------
EPI_ISL_424396       ------------------------------------------------------------
EPI_ISL_424399       ------------------------------------------------------------
EPI_ISL_425530       ------------------------------------------------------------
EPI_ISL_425561       ------------------------------------------------------------
EPI_ISL_425614       ------------------------------------------------------------
EPI_ISL_425635       ------------------------------------------------------------
EPI_ISL_426848       ------------------------------------------------------------
EPI_ISL_426850       ------------------------------------------------------------
EPI_ISL_426955       ------------------------------------------------------------
EPI_ISL_426963       ------------------------------------------------------------

ORF1ab          6961 LGGSVAIKITEHSWNADLYKLMGHFAWWTAFVTNVNASSSEAFLIGCNYLGKPREQIDGY
EPI_ISL_411066       ------------------------------------------------------------
EPI_ISL_413522       ------------------------------------------------------------
EPI_ISL_413558       ------------------------------------------------------------
EPI_ISL_413559       ------------------------------------------------------------
EPI_ISL_413561       ------------------------------------------------------------
EPI_ISL_413692       ------------------------------------------------------------
EPI_ISL_413693       ------------------------------------------------------------
EPI_ISL_413694       ------------------------------------------------------------
EPI_ISL_414692       ------------------------------------------------------------
EPI_ISL_424359       ------------------------------------------------------------
EPI_ISL_424396       ------------------------------------------------------------
EPI_ISL_424399       ------------------------------------------------------------
EPI_ISL_425530       ------------------------------------------------------------
EPI_ISL_425561       ------------------------------------------------------------
EPI_ISL_425614       ------------------------------------------------------------
EPI_ISL_425635       ------------------------------------------------------------
EPI_ISL_426848       ------------------------------------------------------------
EPI_ISL_426850       ------------------------------------------------------------
EPI_ISL_426955       ------------------------------------------------------------
```

EPI_ISL_426963   --

```
ORF1ab         7021 VMHANYIFWRNTNPIQLSSYSLFDMSKFPLKLRGTAVMSLKEGQINDMILSLLSKGRLII
EPI_ISL_411066      ------------------------------------------------------------
EPI_ISL_413522      ------------------------------------------------------------
EPI_ISL_413558      ------------------------------------------------------------
EPI_ISL_413559      ------------------------------------------------------------
EPI_ISL_413561      ------------------------------------------------------------
EPI_ISL_413692      ------------------------------------------------------------
EPI_ISL_413693      ------------------------------------------------------------
EPI_ISL_413694      ------------------------------------------------------------
EPI_ISL_414692      ------------------------------------------------------------
EPI_ISL_424359      ------------------------------------------------------------
EPI_ISL_424396      ------------------------------------------------------------
EPI_ISL_424399      ------------------------------------------------------------
EPI_ISL_425530      ------------------------------------------------------------
EPI_ISL_425561      ------------------------------------------------------------
EPI_ISL_425614      ------------------------------------------------------------
EPI_ISL_425635      ------------------------------------------------------------
EPI_ISL_426848      ------------------------------------------------------------
EPI_ISL_426850      ------------------------------------------------------------
EPI_ISL_426955      ------------------------------------------------------------
EPI_ISL_426963      ------------------------------------------------------------

ORF1ab         7081 RENNRVVISSDVLVNN
EPI_ISL_411066      ----------------
EPI_ISL_413522      ----------------
EPI_ISL_413558      ----------------
EPI_ISL_413559      ----------------
EPI_ISL_413561      ----------------
EPI_ISL_413692      ----------------
EPI_ISL_413693      ----------------
EPI_ISL_413694      ----------------
EPI_ISL_414692      ----------------
EPI_ISL_424359      ----------------
EPI_ISL_424396      ----------------
EPI_ISL_424399      ----------------
EPI_ISL_425530      ----------------
EPI_ISL_425561      ----------------
EPI_ISL_425614      ----------------
EPI_ISL_425635      ----------------
EPI_ISL_426848      ----------------
EPI_ISL_426850      ----------------
EPI_ISL_426955      ----------------
EPI_ISL_426963      ----------------
```

Clade O

```
ORF1ab              1 MESLVPGFNEKTHVQLSLPVLQVRDVLVRGFGDSVEEVLSEARQHLKDGTCGLVEVEKGV
EPI_ISL_413594      1 ------------------------------------------------------------
EPI_ISL_413595      1 ------------------------------------------------------------
EPI_ISL_413596      1 ------------------------------------------------------------
EPI_ISL_413597      1 ------------------------------------------------------------
EPI_ISL_413598      1 ------------------------------------------------------------
EPI_ISL_413599      1 ------------------------------------------------------------
EPI_ISL_413600      1 ------------------------------------------------------------
EPI_ISL_413601      1 ------------------------------------------------------------
EPI_ISL_424217      1 ------------------------------------------------------------
EPI_ISL_424218      1 ------------------------------------------------------------
EPI_ISL_424220      1 ------------------------------------------------------------
EPI_ISL_424232      1 ------------------------------------------------------------
EPI_ISL_424266      1 ------------------------------------------------------------
EPI_ISL_424272      1 ------------------------------------------------------------
EPI_ISL_424283      1 ------------------------------------------------------------
EPI_ISL_424288      1 ------------------------------------------------------------
EPI_ISL_424292      1 ------------------------------------------------------------
EPI_ISL_424295      1 ------------------------------------------------------------
EPI_ISL_426882      1 ------------------------------------------------------------

ORF1ab             61 LPQLEQPYVFIKRSDARTAPHGHVMVELVAELEGIQYGRSGETLGVLVPHVGEIPVAYRK
EPI_ISL_413594      1 ------------------------------------------------------------
EPI_ISL_413595      1 ------------------------------------------------------------
EPI_ISL_413596      1 ------------------------------------------------------------
EPI_ISL_413597      1 ------------------------------------------------------------
EPI_ISL_413598      1 ------------------------------------------------------------
EPI_ISL_413599      1 ------------------------------------------------------------
EPI_ISL_413600      1 ------------------------------------------------------------
EPI_ISL_413601      1 ------------------------------------------------------------
EPI_ISL_424217      1 ------------------------------------------------------------
EPI_ISL_424218      1 ------------------------------------------------------------
EPI_ISL_424220      1 ------------------------------------------------------------
EPI_ISL_424232      1 ------------------------------------------------------------
EPI_ISL_424266      1 ------------------------------------------------------------
EPI_ISL_424272      1 ------------------------------------------------------------
EPI_ISL_424283      1 ------------------------------------------------------------
EPI_ISL_424288      1 ------------------------------------------------------------
EPI_ISL_424292      1 ------------------------------------------------------------
EPI_ISL_424295      1 ------------------------------------------------------------
EPI_ISL_426882      1 ------------------------------------------------------------

ORF1ab            121 VLLRKNGNKGAGGHSYGADLKSFDLGDELGTDPYEDFQENWNTKHSSGVTRELMRELNGG
EPI_ISL_413594      1 ------------------------------------------------------------
EPI_ISL_413595      1 ------------------------------------------------------------
EPI_ISL_413596      1 ------------------------------------------------------------
EPI_ISL_413597      1 ------------------------------------------------------------
EPI_ISL_413598      1 ------------------------------------------------------------
EPI_ISL_413599      1 ------------------------------------------------------------
EPI_ISL_413600      1 ------------------------------------------------------------
EPI_ISL_413601      1 ------------------------------------------------------------
EPI_ISL_424217      1 ------------------------------------------------------------
EPI_ISL_424218      1 ------------------------------------------------------------
EPI_ISL_424220      1 ------------------------------------------------------------
EPI_ISL_424232      1 ------------------------------------------------------------
EPI_ISL_424266      1 ------------------------------------------------------------
EPI_ISL_424272      1 ------------------------------------------------------------
EPI_ISL_424283      1 ------------------------------------------------------------
EPI_ISL_424288      1 ------------------------------------------------------------
EPI_ISL_424292      1 ------------------------------------------------------------
```

```
EPI_ISL_424295    1 ------------------------------------------------------------
EPI_ISL_426882    1 ------------------------------------------------------------
```

```
ORF1ab          181 AYTRYVDNNFCGPDGYPLECIKDLLARAGKASCTLSEQLDFIDTKRGVYCCREHEHEIAW
EPI_ISL_413594    1 ------------------------------------------------------------
EPI_ISL_413595    1 ------------------------------------------------------------
EPI_ISL_413596    1 ------------------------------------------------------------
EPI_ISL_413597    1 ------------------------------------------------------------
EPI_ISL_413598    1 ------------------------------------------------------------
EPI_ISL_413599    1 ------------------------------------------------------------
EPI_ISL_413600    1 ------------------------------------------------------------
EPI_ISL_413601    1 ------------------------------------------------------------
EPI_ISL_424217    1 ------------------------------------------------------------
EPI_ISL_424218    1 ------------------------------------------------------------
EPI_ISL_424220    1 ------------------------------------------------------------
EPI_ISL_424232    1 ------------------------------------------------------------
EPI_ISL_424266    1 ------------------------------------------------------------
EPI_ISL_424272    1 ------------------------------------------------------------
EPI_ISL_424283    1 ------------------------------------------------------------
EPI_ISL_424288    1 ------------------------------------------------------------
EPI_ISL_424292    1 ------------------------------------------------------------
EPI_ISL_424295    1 ------------------------------------------------------------
EPI_ISL_426882    1 ------------------------------------------------------------

ORF1ab          241 YTERSEKSYELQTPFEIKLAKKFDTFNGECPNFVFPLNSIIKTIQPRVEKKKLDGFMGRI
EPI_ISL_413594    1 ------------------------------------------------------------
EPI_ISL_413595    1 ------------------------------------------------------------
EPI_ISL_413596    1 ------------------------------------------------------------
EPI_ISL_413597    1 ------------------------------------------------------------
EPI_ISL_413598    1 ------------------------------------------------------------
EPI_ISL_413599    1 ------------------------------------------------------------
EPI_ISL_413600    1 ------------------------------------------------------------
EPI_ISL_413601    1 ------------------------------------------------------------
EPI_ISL_424217    1 ------------------------------------------------------------
EPI_ISL_424218    1 ------------------------------------------------------------
EPI_ISL_424220    1 ------------------------------------------------------------
EPI_ISL_424232    1 ------------------------------------------------------------
EPI_ISL_424266    1 ------------------------------------------------------------
EPI_ISL_424272    1 ------------------------------------------------------------
EPI_ISL_424283    1 ------------------------------------------------------------
EPI_ISL_424288    1 ------------------------------------------------------------
EPI_ISL_424292    1 ------------------------------------------------------------
EPI_ISL_424295    1 ------------------------------------------------------------
EPI_ISL_426882    1 ------------------------------------------------------------

ORF1ab          301 RSVYPVASPNECNQMCLSTLMKCDHCGETSWQTGDFVKATCEFCGTENLTKEGATTCGYL
EPI_ISL_413594    1 ------------------------------------------------------------
EPI_ISL_413595    1 ------------------------------------------------------------
EPI_ISL_413596    1 ------------------------------------------------------------
EPI_ISL_413597    1 ------------------------------------------------------------
EPI_ISL_413598    1 ------------------------------------------------------------
EPI_ISL_413599    1 ------------------------------------------------------------
EPI_ISL_413600    1 ------------------------------------------------------------
EPI_ISL_413601    1 ------------------------------------------------------------
EPI_ISL_424217    1 ------------------------------------------------------------
EPI_ISL_424218    1 ------------------------------------------------------------
EPI_ISL_424220    1 ------------------------------------------------------------
EPI_ISL_424232    1 ------------------------------------------------------------
EPI_ISL_424266    1 ------------------------------------------------------------
EPI_ISL_424272    1 ------------------------------------------------------------
EPI_ISL_424283    1 ------------------------------------------------------------
EPI_ISL_424288    1 ------------------------------------------------------------
EPI_ISL_424292    1 ------------------------------------------------------------
EPI_ISL_424295    1 ------------------------------------------------------------
EPI_ISL_426882    1 ------------------------------------------------------------
```

```
ORF1ab          361 PQNAVVKIYCPACHNSEVGPEHSLAEYHNESGLKTILRKGGRTIAFGGCVFSYVGCHNKC
EPI_ISL_413594    1 ------------------------------------------------------------
EPI_ISL_413595    1 ------------------------------------------------------------
EPI_ISL_413596    1 ------------------------------------------------------------
EPI_ISL_413597    1 ------------------------------------------------------------
EPI_ISL_413598    1 ------------------------------------------------------------
EPI_ISL_413599    1 ------------------------------------------------------------
EPI_ISL_413600    1 ------------------------------------------------------------
EPI_ISL_413601    1 ------------------------------------------------------------
EPI_ISL_424217    1 ------------------------------------------------------------
EPI_ISL_424218    1 ------------------------------------------------------------
EPI_ISL_424220    1 ------------------------------------------------------------
EPI_ISL_424232    1 ------------------------------------------------------------
EPI_ISL_424266    1 ------------------------------------------------------------
EPI_ISL_424272    1 ------------------------------------------------------------
EPI_ISL_424283    1 ------------------------------------------------------------
EPI_ISL_424288    1 ------------------------------------------------------------
EPI_ISL_424292    1 ------------------------------------------------------------
EPI_ISL_424295    1 ------------------------------------------------------------
EPI_ISL_426882    1 ------------------------------------------------------------

ORF1ab          421 AYWVPRASANIGCNHTGVVGEGSEGLNDNLLEILQKEKVNINIVGDFKLNEEIAIILASF
EPI_ISL_413594    1 ------------------------------------------------------------
EPI_ISL_413595    1 ------------------------------------------------------------
EPI_ISL_413596    1 ------------------------------------------------------------
EPI_ISL_413597    1 ------------------------------------------------------------
EPI_ISL_413598    1 ------------------------------------------------------------
EPI_ISL_413599    1 ------------------------------------------------------------
EPI_ISL_413600    1 ------------------------------------------------------------
EPI_ISL_413601    1 ------------------------------------------------------------
EPI_ISL_424217    1 ------------------------------------------------------------
EPI_ISL_424218    1 ------------------------------------------------------------
EPI_ISL_424220    1 ------------------------------------------------------------
EPI_ISL_424232    1 ------------------------------------------------------------
EPI_ISL_424266    1 ------------------------------------------------------------
EPI_ISL_424272    1 ------------------------------------------------------------
EPI_ISL_424283    1 ------------------------------------------------------------
EPI_ISL_424288    1 ------------------------------------------------------------
EPI_ISL_424292    1 ------------------------------------------------------------
EPI_ISL_424295    1 ------------------------------------------------------------
EPI_ISL_426882    1 ------------------------------------------------------------

ORF1ab          481 SASTSAFVETVKGLDYKAFKQIVESCGNFKVTKGKAKKGAWNIGEQKSILSPLYAFASEA
EPI_ISL_413594    1 ------------------------------------------------------------
EPI_ISL_413595    1 ------------------------------------------------------------
EPI_ISL_413596    1 ------------------------------------------------------------
EPI_ISL_413597    1 ------------------------------------------------------------
EPI_ISL_413598    1 ------------------------------------------------------------
EPI_ISL_413599    1 ------------------------------------------------------------
EPI_ISL_413600    1 ------------------------------------------------------------
EPI_ISL_413601    1 ------------------------------------------------------------
EPI_ISL_424217    1 ------------------------------------------------------------
EPI_ISL_424218    1 ------------------------------------------------------------
EPI_ISL_424220    1 ------------------------------------------------------------
EPI_ISL_424232    1 ------------------------------------------------------------
EPI_ISL_424266    1 ------------------------------------------------------------
EPI_ISL_424272    1 ------------------------------------------------------------
EPI_ISL_424283    1 ------------------------------------------------------------
EPI_ISL_424288    1 ------------------------------------------------------------
EPI_ISL_424292    1 ------------------------------------------------------------
EPI_ISL_424295    1 ------------------------------------------------------------
EPI_ISL_426882    1 ------------------------------------------------------------
```

```
ORF1ab          541 ARVVRSIFSRTLETAQNSVRVLQKAAITILDGISQYSLRLIDAMMFTSDLATNNLVVMAY
EPI_ISL_413594    1 ------------------------------------------------------------
EPI_ISL_413595    1 ------------------------------------------------------------
EPI_ISL_413596    1 ------------------------------------------------------------
EPI_ISL_413597    1 ------------------------------------------------------------
EPI_ISL_413598    1 ------------------------------------------------------------
EPI_ISL_413599    1 ------------------------------------------------------------
EPI_ISL_413600    1 ------------------------------------------------------------
EPI_ISL_413601    1 ------------------------------------------------------------
EPI_ISL_424217    1 ------------------------------------------------------------
EPI_ISL_424218    1 ------------------------------------------------------------
EPI_ISL_424220    1 ------------------------------------------------------------
EPI_ISL_424232    1 ------------------------------------------------------------
EPI_ISL_424266    1 ------------------------------------------------------------
EPI_ISL_424272    1 ------------------------------------------------------------
EPI_ISL_424283    1 ------------------------------------------------------------
EPI_ISL_424288    1 ------------------------------------------------------------
EPI_ISL_424292    1 ------------------------------------------------------------
EPI_ISL_424295    1 ------------------------------------------------------------
EPI_ISL_426882    1 ------------------------------------------------------------

ORF1ab          601 ITGGVVQLTSQWLTNIFGTVYEKLKPVLDWLEEKFKEGVEFLRDGWEIVKFISTCACEIV
EPI_ISL_413594    1 ------------------------------------------------------------
EPI_ISL_413595    1 ------------------------------------------------------------
EPI_ISL_413596    1 ------------------------------------------------------------
EPI_ISL_413597    1 ------------------------------------------------------------
EPI_ISL_413598    1 ------------------------------------------------------------
EPI_ISL_413599    1 ------------------------------------------------------------
EPI_ISL_413600    1 ------------------------------------------------------------
EPI_ISL_413601    1 ------------------------------------------------------------
EPI_ISL_424217    1 ------------------------------------------------------------
EPI_ISL_424218    1 ------------------------------------------------------------
EPI_ISL_424220    1 ------------------------------------------------------------
EPI_ISL_424232    1 ------------------------------------------------------------
EPI_ISL_424266    1 ------------------------------------------------------------
EPI_ISL_424272    1 ------------------------------------------------------------
EPI_ISL_424283    1 ------------------------------------------------------------
EPI_ISL_424288    1 ------------------------------------------------------------
EPI_ISL_424292    1 ------------------------------------------------------------
EPI_ISL_424295    1 ------------------------------------------------------------
EPI_ISL_426882    1 ------------------------------------------------------------

ORF1ab          661 GGQIVTCAKEIKESVQTFFKLVNKFLALCADSIIIGGAKLKALNLGETFVTHSKGLYRKC
EPI_ISL_413594    1 ------------------------------------------------------------
EPI_ISL_413595    1 ------------------------------------------------------------
EPI_ISL_413596    1 ------------------------------------------------------------
EPI_ISL_413597    1 ------------------------------------------------------------
EPI_ISL_413598    1 ------------------------------------------------------------
EPI_ISL_413599    1 ------------------------------------------------------------
EPI_ISL_413600    1 ------------------------------------------------------------
EPI_ISL_413601    1 ------------------------------------------------------------
EPI_ISL_424217    1 ------------------------------------------------------------
EPI_ISL_424218    1 ------------------------------------------------------------
EPI_ISL_424220    1 ------------------------------------------------------------
EPI_ISL_424232    1 ------------------------------------------------------------
EPI_ISL_424266    1 ------------------------------------------------------------
EPI_ISL_424272    1 ------------------------------------------------------------
EPI_ISL_424283    1 ------------------------------------------------------------
EPI_ISL_424288    1 ------------------------------------------------------------
EPI_ISL_424292    1 ------------------------------------------------------------
EPI_ISL_424295    1 ------------------------------------------------------------
EPI_ISL_426882    1 ------------------------------------------------------------
```

```
ORF1ab           721 VKSREETGLLMPLKAPKEIIFLEGETLPTEVLTEEVVLKTGDLQPLEQPTSEAVEAPLVG
EPI_ISL_413594     1 ------------------------------------------------------------
EPI_ISL_413595     1 ------------------------------------------------------------
EPI_ISL_413596     1 ------------------------------------------------------------
EPI_ISL_413597     1 ------------------------------------------------------------
EPI_ISL_413598     1 ------------------------------------------------------------
EPI_ISL_413599     1 ------------------------------------------------------------
EPI_ISL_413600     1 ------------------------------------------------------------
EPI_ISL_413601     1 ------------------------------------------------------------
EPI_ISL_424217     1 ------------------------------------------------------------
EPI_ISL_424218     1 ------------------------------------------------------------
EPI_ISL_424220     1 ------------------------------------------------------------
EPI_ISL_424232     1 ------------------------------------------------------------
EPI_ISL_424266     1 ------------------------------------------------------------
EPI_ISL_424272     1 ------------------------------------------------------------
EPI_ISL_424283     1 ------------------------------------------------------------
EPI_ISL_424288     1 ------------------------------------------------------------
EPI_ISL_424292     1 ------------------------------------------------------------
EPI_ISL_424295     1 ------------------------------------------------------------
EPI_ISL_426882     1 ------------------------------------------------------------

ORF1ab           781 TPVCINGLMLLEIKDTEKYCALAPNMMVTNNTFTLKGGAPTKVTFGDDTVIEVQGYKSVN
EPI_ISL_413594     1 --------------------------------------APTKVTFGDDTVIEVQGYKSVN
EPI_ISL_413595     1 --------------------------------------APTKVTFGDDTVIEVQGYKSVN
EPI_ISL_413596     1 --------------------------------------APTKVTFGDDTVIEVQGYKSVN
EPI_ISL_413597     1 --------------------------------------APTKVTFGDDTVIEVQGYKSVN
EPI_ISL_413598     1 --------------------------------------APTKVTFGDDTVIEVQGYKSVN
EPI_ISL_413599     1 --------------------------------------APTKVTFGDDTVIEVQGYKSVN
EPI_ISL_413600     1 --------------------------------------APTKVTFGDDTVIEVQGYKSVN
EPI_ISL_413601     1 --------------------------------------APTKVTFGDDTVIEVQGYKSVN
EPI_ISL_424217     1 --------------------------------------APTKVTFGDDTVIEVQGYKSVN
EPI_ISL_424218     1 --------------------------------------APTKVTFGDDTVIEVQGYKSVN
EPI_ISL_424220     1 --------------------------------------APTKVTFGDDTVIEVQGYKSVN
EPI_ISL_424232     1 --------------------------------------APTKVTFGDDTVIEVQGYKSVN
EPI_ISL_424266     1 --------------------------------------APTKVTFGDDTVIEVQGYKSVN
EPI_ISL_424272     1 --------------------------------------APTKVTFGDDTVIEVQGYKSVN
EPI_ISL_424283     1 --------------------------------------APTKVTFGDDTVIEVQGYKSVN
EPI_ISL_424288     1 --------------------------------------APTKVTFGDDTVIEVQGYKSVN
EPI_ISL_424292     1 --------------------------------------APTKVTFGDDTVIEVQGYKSVN
EPI_ISL_424295     1 --------------------------------------APTKVTFGDDTVIEVQGYKSVN
EPI_ISL_426882     1 --------------------------------------APTKVTFGDDTVIEVQGYKSVN

ORF1ab           841 ITFELDERIDKVLNEKCSAYTVELGTEVNEFACVVADAVIKTLQPVSELLTPLGIDLDEW
EPI_ISL_413594    23 ITFELDERIDKVLNEKCSAYTVELGTEVNEFACVVADAVIKTLQPVSELLTPLGIDLDEW
EPI_ISL_413595    23 ITFELDERIDKVLNEKCSAYTVELGTEVNEFACVVADAVIKTLQPVSELLTPLGIDLDEW
EPI_ISL_413596    23 ITFELDERIDKVLNEKCSAYTVELGTEVNEFACVVADAVIKTLQPVSELLTPLGIDLDEW
EPI_ISL_413597    23 ITFELDERIDKVLNEKCSAYTVELGTEVNEFACVVADAVIKTLQPVSELLTPLGIDLDEW
EPI_ISL_413598    23 ITFELDERIDKVLNEKCSAYTVELGTEVNEFACVVADAVIKTLQPVSELLTPLGIDLDEW
EPI_ISL_413599    23 ITFELDERIDKVLNEKCSAYTVELGTEVNEFACVVADAVIKTLQPVSELLTPLGIDLDEW
EPI_ISL_413600    23 ITFELDERIDKVLNEKCSAYTVELGTEVNEFACVVADAVIKTLQPVSELLTPLGIDLDEW
EPI_ISL_413601    23 ITFELDERIDKVLNEKCSAYTVELGTEVNEFACVVADAVIKTLQPVSELLTPLGIDLDEW
EPI_ISL_424217    23 ITFELDERIDKVLNEKCSAYTVELGTEVNEFACVVADAVIKTLQPVSELLTPLGIDLDEW
EPI_ISL_424218    23 ITFELDERIDKVLNEKCSAYTVELGTEVNEFACVVADAVIKTLQPVSELLTPLGIDLDEW
EPI_ISL_424220    23 ITFELDERIDKVLNEKCSAYTVELGTEVNEFACVVADAVIKTLQPVSELLTPLGIDLDEW
EPI_ISL_424232    23 ITFELDERIDKVLNEKCSAYTVELGTEVNEFACVVADAVIKTLQPVSELLTPLGIDLDEW
EPI_ISL_424266    23 ITFELDERIDKVLNEKCSAYTVELGTEVNEFACVVADAVIKTLQPVSELLTPLGIDLDEW
EPI_ISL_424272    23 ITFELDERIDKVLNEKCSAYTVELGTEVNEFACVVADAVIKTLQPVSELLTPLGIDLDEW
EPI_ISL_424283    23 ITFELDERIDKVLNEKCSAYTVELGTEVNEFACVVADAVIKTLQPVSELLTPLGIDLDEW
EPI_ISL_424288    23 ITFELDERIDKVLNEKCSAYTVELGTEVNEFACVVADAVIKTLQPVSELLTPLGIDLDEW
EPI_ISL_424292    23 ITFELDERIDKVLNEKCSAYTVELGTEVNEFACVVADAVIKTLQPVSELLTPLGIDLDEW
EPI_ISL_424295    23 ITFELDERIDKVLNEKCSAYTVELGTEVNEFACVVADAVIKTLQPVSELLTPLGIDLDEW
EPI_ISL_426882    23 ITFELDERIDKVLNEKCSAYTVELGTEVNEFACVVADAVIKTLQPVSELLTPLGIDLDEW
```

```
ORF1ab           901  SMATYYLFDESGEFKLASHMYCSFYPPDEDEEEGDCEEEEFEPSTQYEYGTEDDYQGKPL
EPI_ISL_413594    83  SMATYYLFDESGEFKLASHMYCSFYPPDEDEEEGDCEEEEFEPSTQYEYGTEDDYQGKPL
EPI_ISL_413595    83  SMATYYLFDESGEFKLASHMYCSFYPPDEDEEEGDCEEEEFEPSTQYEYGTEDDYQGKPL
EPI_ISL_413596    83  SMATYYLFDESGEFKLASHMYCSFYPPDEDEEEGDCEEEEFEPSTQYEYGTEDDYQGKPL
EPI_ISL_413597    83  SMATYYLFDESGEFKLASHMYCSFYPPDEDEEEGDCEEEEFEPSTQYEYGTEDDYQGKPL
EPI_ISL_413598    83  SMATYYLFDESGEFKLASHMYCSFYPPDEDEEEGDCEEEEFEPSTQYEYGTEDDYQGKPL
EPI_ISL_413599    83  SMATYYLFDESGEFKLASHMYCSFYPPDEDEEEGDCEEEEFEPSTQYEYGTEDDYQGKPL
EPI_ISL_413600    83  SMATYYLFDESGEFKLASHMYCSFYPPDEDEEEGDCEEEEFEPSTQYEYGTEDDYQGKPL
EPI_ISL_413601    83  SMATYYLFDESGEFKLASHMYCSFYPPDEDEEEGDCEEEEFEPSTQYEYGTEDDYQGKPL
EPI_ISL_424217    83  SMATYYLFDESGEFKLASHMYCSFYPPDEDEEEGDCEEEEFEPSTQYEYGTEDDYQGKPL
EPI_ISL_424218    83  SMATYYLFDESGEFKLASHMYCSFYPPDEDEEEGDCEEEEFEPSTQYEYGTEDDYQGKPL
EPI_ISL_424220    83  SMATYYLFDESGEFKLASHMYCSFYPPDEDEEEGDCEEEEFEPSTQYEYGTEDDYQGKPL
EPI_ISL_424232    83  SMATYYLFDESGEFKLASHMYCSFYPPDEDEEEGDCEEEEFEPSTQYEYGTEDDYQGKPL
EPI_ISL_424266    83  SMATYYLFDESGEFKLASHMYCSFYPPDEDEEEGDCEEEEFEPSTQYEYGTEDDYQGKPL
EPI_ISL_424272    83  SMATYYLFDESGEFKLASHMYCSFYPPDEDEEEGDCEEEEFEPSTQYEYGTEDDYQGKPL
EPI_ISL_424283    83  SMATYYLFDESGEFKLASHMYCSFYPPDEDEEEGDCEEEEFEPSTQYEYGTEDDYQGKPL
EPI_ISL_424288    83  SMATYYLFDESGEFKLASHMYCSFYPPDEDEEEGDCEEEEFEPSTQYEYGTEDDYQGKPL
EPI_ISL_424292    83  SMATYYLFDESGEFKLASHMYCSFYPPDEDEEEGDCEEEEFEPSTQYEYGTEDDYQGKPL
EPI_ISL_424295    83  SMATYYLFDESGEFKLASHMYCSFYPPDEDEEEGDCEEEEFEPSTQYEYGTEDDYQGKPL
EPI_ISL_426882    83  SMATYYLFDESGEFKLASHMYCSFYPPDEDEEEGDCEEEEFEPSTQYEYGTEDDYQGKPL
```

```
ORF1ab           961  EFGATSAALQPEEEQEEDWLDDDSQQTVGQQDGSEDNQTTTIQTIVEVQPQLEMELTPVV
EPI_ISL_413594   143  EFGATSAALQPEEEQEEDWLDDDSQQTVGQQDGSEDNQTTTIQTIVEVQPQLEMELTPVV
EPI_ISL_413595   143  EFGATSAALQPEEEQEEDWLDDDSQQTVGQQDGSEDNQTTTIQTIVEVQPQLEMELTPVV
EPI_ISL_413596   143  EFGATSAALQPEEEQEEDWLDDDSQQTVGQQDGSEDNQTTTIQTIVEVQPQLEMELTPVV
EPI_ISL_413597   143  EFGATSAALQPEEEQEEDWLDDDSQQTVGQQDGSEDNQTTTIQTIVEVQPQLEMELTPVV
EPI_ISL_413598   143  EFGATSAALQPEEEQEEDWLDDDSQQTVGQQDGSEDNQTTTIQTIVEVQPQLEMELTPVV
EPI_ISL_413599   143  EFGATSAALQPEEEQEEDWLDDDSQQTVGQQDGSEDNQTTTIQTIVEVQPQLEMELTPVV
EPI_ISL_413600   143  EFGATSAALQPEEEQEEDWLDDDSQQTVGQQDGSEDNQTTTIQTIVEVQPQLEMELTPVV
EPI_ISL_413601   143  EFGATSAALQPEEEQEEDWLDDDSQQTVGQQDGSEDNQTTTIQTIVEVQPQLEMELTPVV
EPI_ISL_424217   143  EFGATSAALQPEEEQEEDWLDDDSQQTVGQQDGSEDNQTTTIQTIVEVQPQLEMELTPVV
EPI_ISL_424218   143  EFGATSAALQPEEEQEEDWLDDDSQQTVGQQDGSEDNQTTTIQTIVEVQPQLEMELTPVV
EPI_ISL_424220   143  EFGATSAALQPEEEQEEDWLDDDSQQTVGQQDGSEDNQTTTIQTIVEVQPQLEMELTPVV
EPI_ISL_424232   143  EFGATSAALQPEEEQEEDWLDDDSQQTVGQQDGSEDNQTTTIQTIVEVQPQLEMELTPVV
EPI_ISL_424266   143  EFGATSAALQPEEEQEEDWLDDDSQQTVGQQDGSEDNQTTTIQTIVEVQPQLEMELTPVV
EPI_ISL_424272   143  EFGATSAALQPEEEQEEDWLDDDSQQTVGQQDGSEDNQTTTIQTIVEVQPQLEMELTPVV
EPI_ISL_424283   143  EFGATSAALQPEEEQEEDWLDDDSQQTVGQQDGSEDNQTTTIQTIVEVQPQLEMELTPVV
EPI_ISL_424288   143  EFGATSAALQPEEEQEEDWLDDDSQQTVGQQDGSEDNQTTTIQTIVEVQPQLEMELTPVV
EPI_ISL_424292   143  EFGATSAALQPEEEQEEDWLDDDSQQTVGQQDGSEDNQTTTIQTIVEVQPQLEMELTPVV
EPI_ISL_424295   143  EFGATSAALQPEEEQEEDWLDDDSQQTVGQQDGSEDNQTTTIQTIVEVQPQLEMELTPVV
EPI_ISL_426882   143  EFGATSAALQPEEEQEEDWLDDDSQQTVGQQDGSEDNQTTTIQTIVEVQPQLEMELTPVV
```

```
ORF1ab          1021  QTIEVNSFSGYLKLTDNVYIKNADIVEEAKKVKPTVVVNAANVYLKHGGGVAGALNKATN
EPI_ISL_413594   203  QTIEVNSFSGYLKLTDNVYIKNADIVEEAKKVKPTVVVNAANVYLKHGGGVAGALNKATN
EPI_ISL_413595   203  QTIEVNSFSGYLKLTDNVYIKNADIVEEAKKVKPTVVVNAANVYLKHGGGVAGALNKATN
EPI_ISL_413596   203  QTIEVNSFSGYLKLTDNVYIKNADIVEEAKKVKPTVVVNAANVYLKHGGGVAGALNKATN
EPI_ISL_413597   203  QTIEVNSFSGYLKLTDNVYIKNADIVEEAKKVKPTVVVNAANVYLKHGGGVAGALNKATN
EPI_ISL_413598   203  QTIEVNSFSGYLKLTDNVYIKNADIVEEAKKVKPTVVVNAANVYLKHGGGVAGALNKATN
EPI_ISL_413599   203  QTIEVNSFSGYLKLTDNVYIKNADIVEEAKKVKPTVVVNAANVYLKHGGGVAGALNKATN
EPI_ISL_413600   203  QTIEVNSFSGYLKLTDNVYIKNADIVEEAKKVKPTVVVNAANVYLKHGGGVAGALNKATN
EPI_ISL_413601   203  QTIEVNSFSGYLKLTDNVYIKNADIVEEAKKVKPTVVVNAANVYLKHGGGVAGALNKATN
EPI_ISL_424217   203  QTIEVNSFSGYLKLTDNVYIKNADIVEEAKKVKPTVVVNAANVYLKHGGGVAGALNKATN
EPI_ISL_424218   203  QTIEVNSFSGYLKLTDNVYIKNADIVEEAKKVKPTVVVNAANVYLKHGGGVAGALNKATN
EPI_ISL_424220   203  QTIEVNSFSGYLKLTDNVYIKNADIVEEAKKVKPTVVVNAANVYLKHGGGVAGALNKATN
EPI_ISL_424232   203  QTIEVNSFSGYLKLTDNVYIKNADIVEEAKKVKPTVVVNAANVYLKHGGGVAGALNKATN
EPI_ISL_424266   203  QTIEVNSFSGYLKLTDNVYIKNADIVEEAKKVKPTVVVNAANVYLKHGGGVAGALNKATN
EPI_ISL_424272   203  QTIEVNSFSGYLKLTDNVYIKNADIVEEAKKVKPTVVVNAANVYLKHGGGVAGALNKATN
EPI_ISL_424283   203  QTIEVNSFSGYLKLTDNVYIKNADIVEEAKKVKPTVVVNAANVYLKHGGGVAGALNKATN
EPI_ISL_424288   203  QTIEVNSFSGYLKLTDNVYIKNADIVEEAKKVKPTVVVNAANVYLKHGGGVAGALNKATN
EPI_ISL_424292   203  QTIEVNSFSGYLKLTDNVYIKNADIVEEAKKVKPTVVVNAANVYLKHGGGVAGALNKATN
EPI_ISL_424295   203  QTIEVNSFSGYLKLTDNVYIKNADIVEEAKKVKPTVVVNAANVYLKHGGGVAGALNKATN
EPI_ISL_426882   203  QTIEVNSFSGYLKLTDNVYIKNADIVEEAKKVKPTVVVNAANVYLKHGGGVAGALNKATN
```

ORF1ab	1081	NAMQVESDDYIATNGPLKVGGSCVLSGHNLAKHCLHVVGPNVNKGEDIQLLKSAYENFNQ
EPI_ISL_413594	263	NAMQVESDDYIATNGPLKVGGSCVLSGHNLAKHCLHVVGPNVNKGEDIQLLKSAYENFNQ
EPI_ISL_413595	263	NAMQVESDDYIATNGPLKVGGSCVLSGHNLAKHCLHVVGPNVNKGEDIQLLKSAYENFNQ
EPI_ISL_413596	263	NAMQVESDDYIATNGPLKVGGSCVLSGHNLAKHCLHVVGPNVNKGEDIQLLKSAYENFNQ
EPI_ISL_413597	263	NAMQVESDDYIATNGPLKVGGSCVLSGHNLAKHCLHVVGPNVNKGEDIQLLKSAYENFNQ
EPI_ISL_413598	263	NAMQVESDDYIATNGPLKVGGSCVLSGHNLAKHCLHVVGPNVNKGEDIQLLKSAYENFNQ
EPI_ISL_413599	263	NAMQVESDDYIATNGPLKVGGSCVLSGHNLAKHCLHVVGPNVNKGEDIQLLKSAYENFNQ
EPI_ISL_413600	263	NAMQVESDDYIATNGPLKVGGSCVLSGHNLAKHCLHVVGPNVNKGEDIQLLKSAYENFNQ
EPI_ISL_413601	263	NAMQVESDDYIATNGPLKVGGSCVLSGHNLAKHCLHVVGPNVNKGEDIQLLKSAYENFNQ
EPI_ISL_424217	263	NAMQVESDDYIATNGPLKVGGSCVLSGHNLAKHCLHVVGPNVNKGEDIQLLKSAYENFNQ
EPI_ISL_424218	263	NAMQVESDDYIATNGPLKVGGSCVLSGHNLAKHCLHVVGPNVNKGEDIQLLKSAYENFNQ
EPI_ISL_424220	263	NAMQVESDDYIATNGPLKVGGSCVLSGHNLAKHCLHVVGPNVNKGEDIQLLKSAYENFNQ
EPI_ISL_424232	263	NAMQVESDDYIATNGPLKVGGSCVLSGHNLAKHCLHVVGPNVNKGEDIQLLKSAYENFNQ
EPI_ISL_424266	263	NAMQVESDDYIATNGPLKVGGSCVLSGHNLAKHCLHVVGPNVNKGEDIQLLKSAYENFNQ
EPI_ISL_424272	263	NAMQVESDDYIATNGPLKVGGSCVLSGHNLAKHCLHVVGPNVNKGEDIQLLKSAYENFNQ
EPI_ISL_424283	263	NAMQVESDDYIATNGPLKVGGSCVLSGHNLAKHCLHVVGPNVNKGEDIQLLKSAYENFNQ
EPI_ISL_424288	263	NAMQVESDDYIATNGPLKVGGSCVLSGHNLAKHCLHVVGPNVNKGEDIQLLKSAYENFNQ
EPI_ISL_424292	263	NAMQVESDDYIATNGPLKVGGSCVLSGHNLAKHCLHVVGPNVNKGEDIQLLKSAYENFNQ
EPI_ISL_424295	263	NAMQVESDDYIATNGPLKVGGSCVLSGHNLAKHCLHVVGPNVNKGEDIQLLKSAYENFNQ
EPI_ISL_426882	263	NAMQVESDDYIATNGPLKVGGSCVLSGHNLAKHCLHVVGPNVNKGEDIQLLKSAYENFNQ

ORF1ab	1141	HEVLLAPLLSAGIFGADPIHSLRVCVDTVRTNVYLAVFDKNLYDKLVSSFLEMKSEKQVE
EPI_ISL_413594	323	HEVLLAPLLSAGIFGADPIHSLRVCVDTVRTNVYLAVFDKNLYDKLVSSFLEMKSEKQVE
EPI_ISL_413595	323	HEVLLAPLLSAGIFGADPIHSLRVCVDTVRTNVYLAVFDKNLYDKLVSSFLEMKSEKQVE
EPI_ISL_413596	323	HEVLLAPLLSAGIFGADPIHSLRVCVDTVRTNVYLAVFDKNLYDKLVSSFLEMKSEKQVE
EPI_ISL_413597	323	HEVLLAPLLSAGIFGADPIHSLRVCVDTVRTNVYLAVFDKNLYDKLVSSFLEMKSEKQVE
EPI_ISL_413598	323	HEVLLAPLLSAGIFGADPIHSLRVCVDTVRTNVYLAVFDKNLYDKLVSSFLEMKSEKQVE
EPI_ISL_413599	323	HEVLLAPLLSAGIFGADPIHSLRVCVDTVRTNVYLAVFDKNLYDKLVSSFLEMKSEKQVE
EPI_ISL_413600	323	HEVLLAPLLSAGIFGADPIHSLRVCVDTVRTNVYLAVFDKNLYDKLVSSFLEMKSEKQVE
EPI_ISL_413601	323	HEVLLAPLLSAGIFGADPIHSLRVCVDTVRTNVYLAVFDKNLYDKLVSSFLEMKSEKQVE
EPI_ISL_424217	323	HEVLLAPLLSAGIFGADPIHSLRVCVDTVRTNVYLAVFDKNLYDKLVSSFLEMKSEKQVE
EPI_ISL_424218	323	HEVLLAPLLSAGIFGADPIHSLRVCVDTVRTNVYLAVFDKNLYDKLVSSFLEMKSEKQVE
EPI_ISL_424220	323	HEVLLAPLLSAGIFGADPIHSLRVCVDTVRTNVYLAVFDKNLYDKLVSSFLEMKSEKQVE
EPI_ISL_424232	323	HEVLLAPLLSAGIFGADPIHSLRVCVDTVRTNVYLAVFDKNLYDKLVSSFLEMKSEKQVE
EPI_ISL_424266	323	HEVLLAPLLSAGIFGADPIHSLRVCVDTVRTNVYLAVFDKNLYDKLVSSFLEMKSEKQVE
EPI_ISL_424272	323	HEVLLAPLLSAGIFGADPIHSLRVCVDTVRTNVYLAVFDKNLYDKLVSSFLEMKSEKQVE
EPI_ISL_424283	323	HEVLLAPLLSAGIFGADPIHSLRVCVDTVRTNVYLAVFDKNLYDKLVSSFLEMKSEKQVE
EPI_ISL_424288	323	HEVLLAPLLSAGIFGADPIHSLRVCVDTVRTNVYLAVFDKNLYDKLVSSFLEMKSEKQVE
EPI_ISL_424292	323	HEVLLAPLLSAGIFGADPIHSLRVCVDTVRTNVYLAVFDKNLYDKLVSSFLEMKSEKQVE
EPI_ISL_424295	323	HEVLLAPLLSAGIFGADPIHSLRVCVDTVRTNVYLAVFDKNLYDKLVSSFLEMKSEKQVE
EPI_ISL_426882	323	HEVLLAPLLSAGIFGADPIHSLRVCVDTVRTNVYLAVFDKNLYDKLVSSFLEMKSEKQVE

ORF1ab	1201	QKIAEIPKEEVKPFITESKPSVEQRKQDDKKIKACVEEVTTTLEETKFLTENLLLYIDIN
EPI_ISL_413594	383	QKIAEIPKEEVKPFITESKPSVEQRKQDDKKIKACVEEVTTTLEETKFLTENLLLYIDIN
EPI_ISL_413595	383	QKIAEIPKEEVKPFITESKPSVEQRKQDDKKIKACVEEVTTTLEETKFLTENLLLYIDIN
EPI_ISL_413596	383	QKIAEIPKEEVKPFITESKPSVEQRKQDDKKIKACVEEVTTTLEETKFLTENLLLYIDIN
EPI_ISL_413597	383	QKIAEIPKEEVKPFITESKPSVEQRKQDDKKIKACVEEVTTTLEETKFLTENLLLYIDIN
EPI_ISL_413598	383	QKIAEIPKEEVKPFITESKPSVEQRKQDDKKIKACVEEVTTTLEETKFLTENLLLYIDIN
EPI_ISL_413599	383	QKIAEIPKEEVKPFITESKPSVEQRKQDDKKIKACVEEVTTTLEETKFLTENLLLYIDIN
EPI_ISL_413600	383	QKIAEIPKEEVKPFITESKPSVEQRKQDDKKIKACVEEVTTTLEETKFLTENLLLYIDIN
EPI_ISL_413601	383	QKIAEIPKEEVKPFITESKPSVEQRKQDDKKIKACVEEVTTTLEETKFLTENLLLYIDIN
EPI_ISL_424217	383	QKIAEIPKEEVKPFITESKPSVEQRKQDDKKIKACVEEVTTTLEETKFLTENLLLYIDIN
EPI_ISL_424218	383	QKIAEIPKEEVKPFITESKPSVEQRKQDDKKIKACVEEVTTTLEETKFLTENLLLYIDIN
EPI_ISL_424220	383	QKIAEIPKEEVKPFITESKPSVEQRKQDDKKIKACVEEVTTTLEETKFLTENLLLYIDIN
EPI_ISL_424232	383	QKIAEIPKEEVKPFITESKPSVEQRKQDDKKIKACVEEVTTTLEETKFLTENLLLYIDIN
EPI_ISL_424266	383	QKIAEIPKEEVKPFITESKPSVEQRKQDDKKIKACVEEVTTTLEETKFLTENLLLYIDIN
EPI_ISL_424272	383	QKIAEIPKEEVKPFITESKPSVEQRKQDDKKIKACVEEVTTTLEETKFLTENLLLYIDIN
EPI_ISL_424283	383	QKIAEIPKEEVKPFITESKPSVEQRKQDDKKIKACVEEVTTTLEETKFLTENLLLYIDIN
EPI_ISL_424288	383	QKIAEIPKEEVKPFITESKPSVEQRKQDDKKIKACVEEVTTTLEETKFLTENLLLYIDIN
EPI_ISL_424292	383	QKIAEIPKEEVKPFITESKPSVEQRKQDDKKIKACVEEVTTTLEETKFLTENLLLYIDIN
EPI_ISL_424295	383	QKIAEIPKEEVKPFITESKPSVEQRKQDDKKIKACVEEVTTTLEETKFLTENLLLYIDIN
EPI_ISL_426882	383	QKIAEIPKEEVKPFITESKPSVEQRKQDDKKIKACVEEVTTTLEETKFLTENLLLYIDIN

ORF1ab	1261	GNLHPDSATLVSDIDITFLKKDAPYIVGDVVQEGVLTAVVIPTKKAGGTTEMLAKALRKV
EPI_ISL_413594	443	GNLHPDSATLVSDIDITFLKKDAPYIVGDVVQEGVLTAVVIPTKKAGGTTEMLAKALRKV
EPI_ISL_413595	443	GNLHPDSATLVSDIDITFLKKDAPYIVGDVVQEGVLTAVVIPTKKAGGTTEMLAKALRKV
EPI_ISL_413596	443	GNLHPDSATLVSDIDITFLKKDAPYIVGDVVQEGVLTAVVIPTKKAGGTTEMLAKALRKV
EPI_ISL_413597	443	GNLHPDSATLVSDIDITFLKKDAPYIVGDVVQEGVLTAVVIPTKKAGGTTEMLAKALRKV
EPI_ISL_413598	443	GNLHPDSATLVSDIDITFLKKDAPYIVGDVVQEGVLTAVVIPTKKAGGTTEMLAKALRKV
EPI_ISL_413599	443	GNLHPDSATLVSDIDITFLKKDAPYIVGDVVQEGVLTAVVIPTKKAGGTTEMLAKALRKV
EPI_ISL_413600	443	GNLHPDSATLVSDIDITFLKKDAPYIVGDVVQEGVLTAVVIPTKKAGGTTEMLAKALRKV
EPI_ISL_413601	443	GNLHPDSATLVSDIDITFLKKDAPYIVGDVVQEGVLTAVVIPTKKAGGTTEMLAKALRKV
EPI_ISL_424217	443	GNLHPDSATLVSDIDITFLKKDAPYIVGDVVQEGVLTAVVIPTKKAGGTTEMLAKALRKV
EPI_ISL_424218	443	GNLHPDSATLVSDIDITFLKKDAPYIVGDVVQEGVLTAVVIPTKKAGGTTEMLAKALRKV
EPI_ISL_424220	443	GNLHPDSATLVSDIDITFLKKDAPYIVGDVVQEGVLTAVVIPTKKAGGTTEMLAKALRKV
EPI_ISL_424232	443	GNLHPDSATLVSDIDITFLKKDAPYIVGDVVQEGVLTAVVIPTKKAGGTTEMLAKALRKV
EPI_ISL_424266	443	GNLHPDSATLVSDIDITFLKKDAPYIVGDVVQEGVLTAVVIPTKKAGGTTEMLAKALRKV
EPI_ISL_424272	443	GNLHPDSATLVSDIDITFLKKDAPYIVGDVVQEGVLTAVVIPTKKAGGTTEMLAKALRKV
EPI_ISL_424283	443	GNLHPDSATLVSDIDITFLKKDAPYIVGDVVQEGVLTAVVIPTKKAGGTTEMLAKALRKV
EPI_ISL_424288	443	GNLHPDSATLVSDIDITFLKKDAPYIVGDVVQEGVLTAVVIPTKKAGGTTEMLAKALRKV
EPI_ISL_424292	443	GNLHPDSATLVSDIDITFLKKDAPYIVGDVVQEGVLTAVVIPTKKAGGTTEMLAKALRKV
EPI_ISL_424295	443	GNLHPDSATLVSDIDITFLKKDAPYIVGDVVQEGVLTAVVIPTKKAGGTTEMLAKALRKV
EPI_ISL_426882	443	GNLHPDSATLVSDIDITFLKKDAPYIVGDVVQEGVLTAVVIPTKKAGGTTEMLAKALRKV

ORF1ab	1321	PTDNYITTYPGQGLNGYTVEEAKTVLKKCKSAFYILPSIISNEKQEILGTVSWNLREMLA
EPI_ISL_413594	503	PTDNYITTYPGQGLNGYTVEEAKTVLKKCKSAFYILPSIISNEKQEILGTVSWNLREMLA
EPI_ISL_413595	503	PTDNYITTYPGQGLNGYTVEEAKTVLKKCKSAFYILPSIISNEKQEILGTVSWNLREMLA
EPI_ISL_413596	503	PTDNYITTYPGQGLNGYTVEEAKTVLKKCKSAFYILPSIISNEKQEILGTVSWNLREMLA
EPI_ISL_413597	503	PTDNYITTYPGQGLNGYTVEEAKTVLKKCKSAFYILPSIISNEKQEILGTVSWNLREMLA
EPI_ISL_413598	503	PTDNYITTYPGQGLNGYTVEEAKTVLKKCKSAFYILPSIISNEKQEILGTVSWNLREMLA
EPI_ISL_413599	503	PTDNYITTYPGQGLNGYTVEEAKTVLKKCKSAFYILPSIISNEKQEILGTVSWNLREMLA
EPI_ISL_413600	503	PTDNYITTYPGQGLNGYTVEEAKTVLKKCKSAFYILPSIISNEKQEILGTVSWNLREMLA
EPI_ISL_413601	503	PTDNYITTYPGQGLNGYTVEEAKTVLKKCKSAFYILPSIISNEKQEILGTVSWNLREMLA
EPI_ISL_424217	503	PTDNYITTYPGQGLNGYTVEEAKTVLKKCKSAFYILPSIISNEKQEILGTVSWNLREMLA
EPI_ISL_424218	503	PTDNYITTYPGQGLNGYTVEEAKTVLKKCKSAFYILPSIISNEKQEILGTVSWNLREMLA
EPI_ISL_424220	503	PTDNYITTYPGQGLNGYTVEEAKTVLKKCKSAFYILPSIISNEKQEILGTVSWNLREMLA
EPI_ISL_424232	503	PTDNYITTYPGQGLNGYTVEEAKTVLKKCKSAFYILPSIISNEKQEILGTVSWNLREMLA
EPI_ISL_424266	503	PTDNYITTYPGQGLNGYTVEEAKTVLKKCKSAFYILPSIISNEKQEILGTVSWNLREMLA
EPI_ISL_424272	503	PTDNYITTYPGQGLNGYTVEEAKTVLKKCKSAFYILPSIISNEKQEILGTVSWNLREMLA
EPI_ISL_424283	503	PTDNYITTYPGQGLNGYTVEEAKTVLKKCKSAFYILPSIISNEKQEILGTVSWNLREMLA
EPI_ISL_424288	503	PTDNYITTYPGQGLNGYTVEEAKTVLKKCKSAFYILPSIISNEKQEILGTVSWNLREMLA
EPI_ISL_424292	503	PTDNYITTYPGQGLNGYTVEEAKTVLKKCKSAFYILPSIISNEKQEILGTVSWNLREMLA
EPI_ISL_424295	503	PTDNYITTYPGQGLNGYTVEEAKTVLKKCKSAFYILPSIISNEKQEILGTVSWNLREMLA
EPI_ISL_426882	503	PTDNYITTYPGQGLNGYTVEEAKTVLKKCKSAFYILPSIISNEKQEILGTVSWNLREMLA

ORF1ab	1381	HAEETRKLMPVCVETKAIVSTIQRKYKGIKIQEGVVDYGARFYFYTSKTTVASLINTLND
EPI_ISL_413594	563	HAEETRKLMPVCVETKAIVSTIQRKYKGIKIQEGVVDYGARFYFYTSKTTVASLINTLND
EPI_ISL_413595	563	HAEETRKLMPVCVETKAIVSTIQRKYKGIKIQEGVVDYGARFYFYTSKTTVASLINTLND
EPI_ISL_413596	563	HAEETRKLMPVCVETKAIVSTIQRKYKGIKIQEGVVDYGARFYFYTSKTTVASLINTLND
EPI_ISL_413597	563	HAEETRKLMPVCVETKAIVSTIQRKYKGIKIQEGVVDYGARFYFYTSKTTVASLINTLND
EPI_ISL_413598	563	HAEETRKLMPVCVETKAIVSTIQRKYKGIKIQEGVVDYGARFYFYTSKTTVASLINTLND
EPI_ISL_413599	563	HAEETRKLMPVCVETKAIVSTIQRKYKGIKIQEGVVDYGARFYFYTSKTTVASLINTLND
EPI_ISL_413600	563	HAEETRKLMPVCVETKAIVSTIQRKYKGIKIQEGVVDYGARFYFYTSKTTVASLINTLND
EPI_ISL_413601	563	HAEETRKLMPVCVETKAIVSTIQRKYKGIKIQEGVVDYGARFYFYTSKTTVASLINTLND
EPI_ISL_424217	563	HAEETRKLMPVCVETKAIVSTIQRKYKGIKIQEGVVDYGARFYFYTSKTTVASLINTLND
EPI_ISL_424218	563	HAEETRKLMPVCVETKAIVSTIQRKYKGIKIQEGVVDYGARFYFYTSKTTVASLINTLND
EPI_ISL_424220	563	HAEETRKLMPVCVETKAIVSTIQRKYKGIKIQEGVVDYGARFYFYTSKTTVASLINTLND
EPI_ISL_424232	563	HAEETRKLMPVCVETKAIVSTIQRKYKGIKIQEGVVDYGARFYFYTSKTTVASLINTLND
EPI_ISL_424266	563	HAEETRKLMPVCVETKAIVSTIQRKYKGIKIQEGVVDYGARFYFYTSKTTVASLINTLND
EPI_ISL_424272	563	HAEETRKLMPVCVETKAIVSTIQRKYKGIKIQEGVVDYGARFYFYTSKTTVASLINTLND
EPI_ISL_424283	563	HAEETRKLMPVCVETKAIVSTIQRKYKGIKIQEGVVDYGARFYFYTSKTTVASLINTLND
EPI_ISL_424288	563	HAEETRKLMPVCVETKAIVSTIQRKYKGIKIQEGVVDYGARFYFYTSKTTVASLINTLND
EPI_ISL_424292	563	HAEETRKLMPVCVETKAIVSTIQRKYKGIKIQEGVVDYGARFYFYTSKTTVASLINTLND
EPI_ISL_424295	563	HAEETRKLMPVCVETKAIVSTIQRKYKGIKIQEGVVDYGARFYFYTSKTTVASLINTLND
EPI_ISL_426882	563	HAEETRKLMPVCVETKAIVSTIQRKYKGIKIQEGVVDYGARFYFYTSKTTVASLINTLND

```
ORF1ab          1441 LNETLVTMPLGYVTHGLNLEEAARYMRSLKVPATVSVSSPDAVTAYNGYLTSSSKTPEEH
EPI_ISL_413594   623 LNETLVTMPLGYVTHGLNLEEAARYMRSLKVPATVSVSSPDAVTAYNGYLTSSSKTPEEH
EPI_ISL_413595   623 LNETLVTMPLGYVTHGLNLEEAARYMRSLKVPATVSVSSPDAVTAYNGYLTSSSKTPEEH
EPI_ISL_413596   623 LNETLVTMPLGYVTHGLNLEEAARYMRSLKVPATVSVSSPDAVTAYNGYLTSSSKTPEEH
EPI_ISL_413597   623 LNETLVTMPLGYVTHGLNLEEAARYMRSLKVPATVSVSSPDAVTAYNGYLTSSSKTPEEH
EPI_ISL_413598   623 LNETLVTMPLGYVTHGLNLEEAARYMRSLKVPATVSVSSPDAVTAYNGYLTSSSKTPEEH
EPI_ISL_413599   623 LNETLVTMPLGYVTHGLNLEEAARYMRSLKVPATVSVSSPDAVTAYNGYLTSSSKTPEEH
EPI_ISL_413600   623 LNETLVTMPLGYVTHGLNLEEAARYMRSLKVPATVSVSSPDAVTAYNGYLTSSSKTPEEH
EPI_ISL_413601   623 LNETLVTMPLGYVTHGLNLEEAARYMRSLKVPATVSVSSPDAVTAYNGYLTSSSKTPEEH
EPI_ISL_424217   623 LNETLVTMPLGYVTHGLNLEEAARYMRSLKVPATVSVSSPDAVTAYNGYLTSSSKTPEEH
EPI_ISL_424218   623 LNETLVTMPLGYVTHGLNLEEAARYMRSLKVPATVSVSSPDAVTAYNGYLTSSSKTPEEH
EPI_ISL_424220   623 LNETLVTMPLGYVTHGLNLEEAARYMRSLKVPATVSVSSPDAVTAYNGYLTSSSKTPEEH
EPI_ISL_424232   623 LNETLVTMPLGYVTHGLNLEEAARYMRSLKVPATVSVSSPDAVTAYNGYLTSSSKTPEEH
EPI_ISL_424266   623 LNETLVTMPLGYVTHGLNLEEAARYMRSLKVPATVSVSSPDAVTAYNGYLTSSSKTPEEH
EPI_ISL_424272   623 LNETLVTMPLGYVTHGLNLEEAARYMRSLKVPATVSVSSPDAVTAYNGYLTSSSKTPEEH
EPI_ISL_424283   623 LNETLVTMPLGYVTHGLNLEEAARYMRSLKVPATVSVSSPDAVTAYNGYLTSSSKTPEEH
EPI_ISL_424288   623 LNETLVTMPLGYVTHGLNLEEAARYMRSLKVPATVSVSSPDAVTAYNGYLTSSSKTPEEH
EPI_ISL_424292   623 LNETLVTMPLGYVTHGLNLEEAARYMRSLKVPATVSVSSPDAVTAYNGYLTSSSKTPEEH
EPI_ISL_424295   623 LNETLVTMPLGYVTHGLNLEEAARYMRSLKVPATVSVSSPDAVTAYNGYLTSSSKTPEEH
EPI_ISL_426882   623 LNETLVTMPLGYVTHGLNLEEAARYMRSLKVPATVSVSSPDAVTAYNGYLTSSSKTPEEH

ORF1ab          1501 FIETISLAGSYKDWSYSGQSTQLGIEFLKRGDKSVYYTSNPTTFHLDGEVITFDNLKTLL
EPI_ISL_413594   683 FIETISLAGSYKDWSYSGQSTQLGIEFLKRGDKSVYYTSNPTTFHLDGEVITFDNLKTLL
EPI_ISL_413595   683 FIETISLAGSYKDWSYSGQSTQLGIEFLKRGDKSVYYTSNPTTFHLDGEVITFDNLKTLL
EPI_ISL_413596   683 FIETISLAGSYKDWSYSGQSTQLGIEFLKRGDKSVYYTSNPTTFHLDGEVITFDNLKTLL
EPI_ISL_413597   683 FIETISLAGSYKDWSYSGQSTQLGIEFLKRGDKSVYYTSNPTTFHLDGEVITFDNLKTLL
EPI_ISL_413598   683 FIETISLAGSYKDWSYSGQSTQLGIEFLKRGDKSVYYTSNPTTFHLDGEVITFDNLKTLL
EPI_ISL_413599   683 FIETISLAGSYKDWSYSGQSTQLGIEFLKRGDKSVYYTSNPTTFHLDGEVITFDNLKTLL
EPI_ISL_413600   683 FIETISLAGSYKDWSYSGQSTQLGIEFLKRGDKSVYYTSNPTTFHLDGEVITFDNLKTLL
EPI_ISL_413601   683 FIETISLAGSYKDWSYSGQSTQLGIEFLKRGDKSVYYTSNPTTFHLDGEVITFDNLKTLL
EPI_ISL_424217   683 FIETISLAGSYKDWSYSGQSTQLGIEFLKRGDKSVYYTSNPTTFHLDGEVITFDNLKTLL
EPI_ISL_424218   683 FIETISLAGSYKDWSYSGQSTQLGIEFLKRGDKSVYYTSNPTTFHLDGEVITFDNLKTLL
EPI_ISL_424220   683 FIETISLAGSYKDWSYSGQSTQLGIEFLKRGDKSVYYTSNPTTFHLDGEVITFDNLKTLL
EPI_ISL_424232   683 FIETISLAGSYKDWSYSGQSTQLGIEFLKRGDKSVYYTSNPTTFHLDGEVITFDNLKTLL
EPI_ISL_424266   683 FIETISLAGSYKDWSYSGQSTQLGIEFLKRGDKSVYYTSNPTTFHLDGEVITFDNLKTLL
EPI_ISL_424272   683 FIETISLAGSYKDWSYSGQSTQLGIEFLKRGDKSVYYTSNPTTFHLDGEVITFDNLKTLL
EPI_ISL_424283   683 FIETISLAGSYKDWSYSGQSTQLGIEFLKRGDKSVYYTSNPTTFHLDGEVITFDNLKTLL
EPI_ISL_424288   683 FIETISLAGSYKDWSYSGQSTQLGIEFLKRGDKSVYYTSNPTTFHLDGEVITFDNLKTLL
EPI_ISL_424292   683 FIETISLAGSYKDWSYSGQSTQLGIEFLKRGDKSVYYTSNPTTFHLDGEVITFDNLKTLL
EPI_ISL_424295   683 FIETISLAGSYKDWSYSGQSTQLGIEFLKRGDKSVYYTSNPTTFHLDGEVITFDNLKTLL
EPI_ISL_426882   683 FIETISLAGSYKDWSYSGQSTQLGIEFLKRGDKSVYYTSNPTTFHLDGEVITFDNLKTLL

ORF1ab          1561 SLREVRTIKVFTTVDNINLHTQVVDMSMTYGQQFGPTYLDGADVTKIKPHNSHEGKTFYV
EPI_ISL_413594   743 SLREVRTIKVFTTVDNINLHTQVVDMSMTYGQQFGPTYLDGADVTKIKPHNSHEGKTFYV
EPI_ISL_413595   743 SLREVRTIKVFTTVDNINLHTQVVDMSMTYGQQFGPTYLDGADVTKIKPHNSHEGKTFYV
EPI_ISL_413596   743 SLREVRTIKVFTTVDNINLHTQVVDMSMTYGQQFGPTYLDGADVTKIKPHNSHEGKTFYV
EPI_ISL_413597   743 SLREVRTIKVFTTVDNINLHTQVVDMSMTYGQQFGPTYLDGADVTKIKPHNSHEGKTFYV
EPI_ISL_413598   743 SLREVRTIKVFTTVDNINLHTQVVDMSMTYGQQFGPTYLDGADVTKIKPHNSHEGKTFYV
EPI_ISL_413599   743 SLREVRTIKVFTTVDNINLHTQVVDMSMTYGQQFGPTYLDGADVTKIKPHNSHEGKTFYV
EPI_ISL_413600   743 SLREVRTIKVFTTVDNINLHTQVVDMSMTYGQQFGPTYLDGADVTKIKPHNSHEGKTFYV
EPI_ISL_413601   743 SLREVRTIKVFTTVDNINLHTQVVDMSMTYGQQFGPTYLDGADVTKIKPHNSHEGKTFYV
EPI_ISL_424217   743 SLREVRTIKVFTTVDNINLHTQVVDMSMTYGQQFGPTYLDGADVTKIKPHNSHEGKTFYV
EPI_ISL_424218   743 SLREVRTIKVFTTVDNINLHTQVVDMSMTYGQQFGPTYLDGADVTKIKPHNSHEGKTFYV
EPI_ISL_424220   743 SLREVRTIKVFTTVDNINLHTQVVDMSMTYGQQFGPTYLDGADVTKIKPHNSHEGKTFYV
EPI_ISL_424232   743 SLREVRTIKVFTTVDNINLHTQVVDMSMTYGQQFGPTYLDGADVTKIKPHNSHEGKTFYV
EPI_ISL_424266   743 SLREVRTIKVFTTVDNINLHTQVVDMSMTYGQQFGPTYLDGADVTKIKPHNSHEGKTFYV
EPI_ISL_424272   743 SLREVRTIKVFTTVDNINLHTQVVDMSMTYGQQFGPTYLDGADVTKIKPHNSHEGKTFYV
EPI_ISL_424283   743 SLREVRTIKVFTTVDNINLHTQVVDMSMTYGQQFGPTYLDGADVTKIKPHNSHEGKTFYV
EPI_ISL_424288   743 SLREVRTIKVFTTVDNINLHTQVVDMSMTYGQQFGPTYLDGADVTKIKPHNSHEGKTFYV
EPI_ISL_424292   743 SLREVRTIKVFTTVDNINLHTQVVDMSMTYGQQFGPTYLDGADVTKIKPHNSHEGKTFYV
EPI_ISL_424295   743 SLREVRTIKVFTTVDNINLHTQVVDMSMTYGQQFGPTYLDGADVTKIKPHNSHEGKTFYV
EPI_ISL_426882   743 SLREVRTIKVFTTVDNINLHTQVVDMSMTYGQQFGPTYLDGADVTKIKPHNSHEGKTFYV
```

```
ORF1ab          1621 LPNDDTLRVEAFEYYHTTDPSFLGRYMSALNHTKKWKYPQVNGLTSIKWADNNCYLATAL
EPI_ISL_413594   803 LPNDDTLRVEAFEYYHTTDPSFLGRYMSALNHTKKWKYPQVNGLTSIKWADNNCYLATAL
EPI_ISL_413595   803 LPNDDTLRVEAFEYYHTTDPSFLGRYMSALNHTKKWKYPQVNGLTSIKWADNNCYLATAL
EPI_ISL_413596   803 LPNDDTLRVEAFEYYHTTDPSFLGRYMSALNHTKKWKYPQVNGLTSIKWADNNCYLATAL
EPI_ISL_413597   803 LPNDDTLRVEAFEYYHTTDPSFLGRYMSALNHTKKWKYPQVNGLTSIKWADNNCYLATAL
EPI_ISL_413598   803 LPNDDTLRVEAFEYYHTTDPSFLGRYMSALNHTKKWKYPQVNGLTSIKWADNNCYLATAL
EPI_ISL_413599   803 LPNDDTLRVEAFEYYHTTDPSFLGRYMSALNHTKKWKYPQVNGLTSIKWADNNCYLATAL
EPI_ISL_413600   803 LPNDDTLRVEAFEYYHTTDPSFLGRYMSALNHTKKWKYPQVNGLTSIKWADNNCYLATAL
EPI_ISL_413601   803 LPNDDTLRVEAFEYYHTTDPSFLGRYMSALNHTKKWKYPQVNGLTSIKWADNNCYLATAL
EPI_ISL_424217   803 LPNDDTLRVEAFEYYHTTDPSFLGRYMSALNHTKKWKYPQVNGLTSIKWADNNCYLATAL
EPI_ISL_424218   803 LPNDDTLRVEAFEYYHTTDPSFLGRYMSALNHTKKWKYPQVNGLTSIKWADNNCYLATAL
EPI_ISL_424220   803 LPNDDTLRVEAFEYYHTTDPSFLGRYMSALNHTKKWKYPQVNGLTSIKWADNNCYLATAL
EPI_ISL_424232   803 LPNDDTLRVEAFEYYHTTDPSFLGRYMSALNHTKKWKYPQVNGLTSIKWADNNCYLATAL
EPI_ISL_424266   803 LPNDDTLRVEAFEYYHTTDPSFLGRYMSALNHTKKWKYPQVNGLTSIKWADNNCYLATAL
EPI_ISL_424272   803 LPNDDTLRVEAFEYYHTTDPSFLGRYMSALNHTKKWKYPQVNGLTSIKWADNNCYLATAL
EPI_ISL_424283   803 LPNDDTLRVEAFEYYHTTDPSFLGRYMSALNHTKKWKYPQVNGLTSIKWADNNCYLATAL
EPI_ISL_424288   803 LPNDDTLRVEAFEYYHTTDPSFLGRYMSALNHTKKWKYPQVNGLTSIKWADNNCYLATAL
EPI_ISL_424292   803 LPNDDTLRVEAFEYYHTTDPSFLGRYMSALNHTKKWKYPQVNGLTSIKWADNNCYLATAL
EPI_ISL_424295   803 LPNDDTLRVEAFEYYHTTDPSFLGRYMSALNHTKKWKYPQVNGLTSIKWADNNCYLATAL
EPI_ISL_426882   803 LPNDDTLRVEAFEYYHTTDPSFLGRYMSALNHTKKWKYPQVNGLTSIKWADNNCYLATAL

ORF1ab          1681 LTLQQIELKFNPPALQDAYYRARAGEAANFCALILAYCNKTVGELGDVRETMSYLFQHAN
EPI_ISL_413594   863 LTLQQIELKFNPPALQDAYYRARAGEAANFCALILAYCNKTVGELGDVRETMSYLFQHAN
EPI_ISL_413595   863 LTLQQIELKFNPPALQDAYYRARAGEAANFCALILAYCNKTVGELGDVRETMSYLFQHAN
EPI_ISL_413596   863 LTLQQIELKFNPPALQDAYYRARAGEAANFCALILAYCNKTVGELGDVRETMSYLFQHAN
EPI_ISL_413597   863 LTLQQIELKFNPPALQDAYYRARAGEAANFCALILAYCNKTVGELGDVRETMSYLFQHAN
EPI_ISL_413598   863 LTLQQIELKFNPPALQDAYYRARAGEAANFCALILAYCNKTVGELGDVRETMSYLFQHAN
EPI_ISL_413599   863 LTLQQIELKFNPPALQDAYYRARAGEAANFCALILAYCNKTVGELGDVRETMSYLFQHAN
EPI_ISL_413600   863 LTLQQIELKFNPPALQDAYYRARAGEAANFCALILAYCNKTVGELGDVRETMSYLFQHAN
EPI_ISL_413601   863 LTLQQIELKFNPPALQDAYYRARAGEAANFCALILAYCNKTVGELGDVRETMSYLFQHAN
EPI_ISL_424217   863 LTLQQIELKFNPPALQDAYYRARAGEAANFCALILAYCNKTVGELGDVRETMSYLFQHAN
EPI_ISL_424218   863 LTLQQIELKFNPPALQDAYYRARAGEAANFCALILAYCNKTVGELGDVRETMSYLFQHAN
EPI_ISL_424220   863 LTLQQIELKFNPPALQDAYYRARAGEAANFCALILAYCNKTVGELGDVRETMSYLFQHAN
EPI_ISL_424232   863 LTLQQIELKFNPPALQDAYYRARAGEAANFCALILAYCNKTVGELGDVRETMSYLFQHAN
EPI_ISL_424266   863 LTLQQIELKFNPPALQDAYYRARAGEAANFCALILAYCNKTVGELGDVRETMSYLFQHAN
EPI_ISL_424272   863 LTLQQIELKFNPPALQDAYYRARAGEAANFCALILAYCNKTVGELGDVRETMSYLFQHAN
EPI_ISL_424283   863 LTLQQIELKFNPPALQDAYYRARAGEAANFCALILAYCNKTVGELGDVRETMSYLFQHAN
EPI_ISL_424288   863 LTLQQIELKFNPPALQDAYYRARAGEAANFCALILAYCNKTVGELGDVRETMSYLFQHAN
EPI_ISL_424292   863 LTLQQIELKFNPPALQDAYYRARAGEAANFCALILAYCNKTVGELGDVRETMSYLFQHAN
EPI_ISL_424295   863 LTLQQIELKFNPPALQDAYYRARAGEAANFCALILAYCNKTVGELGDVRETMSYLFQHAN
EPI_ISL_426882   863 LTLQQIELKFNPPALQDAYYRARAGEAANFCALILAYCNKTVGELGDVRETMSYLFQHAN

ORF1ab          1741 LDSCKRVLNVVCKTCGQQQTTLKGVEAVMYMGTLSYEQFKKGVQIPCTCGKQATKYLVQQ
EPI_ISL_413594   923 LDSCKRVLNVVCKTCGQQQTTLKGVEAVMYMGTLSYEQFKKGVQIPCTCGKQATKYLVQQ
EPI_ISL_413595   923 LDSCKRVLNVVCKTCGQQQTTLKGVEAVMYMGTLSYEQFKKGVQIPCTCGKQATKYLVQQ
EPI_ISL_413596   923 LDSCKRVLNVVCKTCGQQQTTLKGVEAVMYMGTLSYEQFKKGVQIPCTCGKQATKYLVQQ
EPI_ISL_413597   923 LDSCKRVLNVVCKTCGQQQTTLKGVEAVMYMGTLSYEQFKKGVQIPCTCGKQATKYLVQQ
EPI_ISL_413598   923 LDSCKRVLNVVCKTCGQQQTTLKGVEAVMYMGTLSYEQFKKGVQIPCTCGKQATKYLVQQ
EPI_ISL_413599   923 LDSCKRVLNVVCKTCGQQQTTLKGVEAVMYMGTLSYEQFKKGVQIPCTCGKQATKYLVQQ
EPI_ISL_413600   923 LDSCKRVLNVVCKTCGQQQTTLKGVEAVMYMGTLSYEQFKKGVQIPCTCGKQATKYLVQQ
EPI_ISL_413601   923 LDSCKRVLNVVCKTCGQQQTTLKGVEAVMYMGTLSYEQFKKGVQIPCTCGKQATKYLVQQ
EPI_ISL_424217   923 LDSCKRVLNVVCKTCGQQQTTLKGVEAVMYMGTLSYEQFKKGVQIPCTCGKQATKYLVQQ
EPI_ISL_424218   923 LDSCKRVLNVVCKTCGQQQTTLKGVEAVMYMGTLSYEQFKKGVQIPCTCGKQATKYLVQQ
EPI_ISL_424220   923 LDSCKRVLNVVCKTCGQQQTTLKGVEAVMYMGTLSYEQFKKGVQIPCTCGKQATKYLVQQ
EPI_ISL_424232   923 LDSCKRVLNVVCKTCGQQQTTLKGVEAVMYMGTLSYEQFKKGVQIPCTCGKQATKYLVQQ
EPI_ISL_424266   923 LDSCKRVLNVVCKTCGQQQTTLKGVEAVMYMGTLSYEQFKKGVQIPCTCGKQATKYLVQQ
EPI_ISL_424272   923 LDSCKRVLNVVCKTCGQQQTTLKGVEAVMYMGTLSYEQFKKGVQIPCTCGKQATKYLVQQ
EPI_ISL_424283   923 LDSCKRVLNVVCKTCGQQQTTLKGVEAVMYMGTLSYEQFKKGVQIPCTCGKQATKYLVQQ
EPI_ISL_424288   923 LDSCKRVLNVVCKTCGQQQTTLKGVEAVMYMGTLSYEQFKKGVQIPCTCGKQATKYLVQQ
EPI_ISL_424292   923 LDSCKRVLNVVCKTCGQQQTTLKGVEAVMYMGTLSYEQFKKGVQIPCTCGKQATKYLVQQ
EPI_ISL_424295   923 LDSCKRVLNVVCKTCGQQQTTLKGVEAVMYMGTLSYEQFKKGVQIPCTCGKQATKYLVQQ
EPI_ISL_426882   923 LDSCKRVLNVVCKTCGQQQTTLKGVEAVMYMGTLSYEQFKKGVQIPCTCGKQATKYLVQQ
```

```
ORF1ab          1801 ESPFVMMSAPPAQYELKHGTFTCASEYTGNYQCGHYKHITSKETLYCIDGALLTKSSEYK
EPI_ISL_413594   983 ESPFVMMSAPPAQYELKHGTFTCASEYTGNYQCGHYKHITSKETLYCIDGALLTKSSEYK
EPI_ISL_413595   983 ESPFVMMSAPPAQYELKHGTFTCASEYTGNYQCGHYKHITSKETLYCIDGALLTKSSEYK
EPI_ISL_413596   983 ESPFVMMSAPPAQYELKHGTFTCASEYTGNYQCGHYKHITSKETLYCIDGALLTKSSEYK
EPI_ISL_413597   983 ESPFVMMSAPPAQYELKHGTFTCASEYTGNYQCGHYKHITSKETLYCIDGALLTKSSEYK
EPI_ISL_413598   983 ESPFVMMSAPPAQYELKHGTFTCASEYTGNYQCGHYKHITSKETLYCIDGALLTKSSEYK
EPI_ISL_413599   983 ESPFVMMSAPPAQYELKHGTFTCASEYTGNYQCGHYKHITSKETLYCIDGALLTKSSEYK
EPI_ISL_413600   983 ESPFVMMSAPPAQYELKHGTFTCASEYTGNYQCGHYKHITSKETLYCIDGALLTKSSEYK
EPI_ISL_413601   983 ESPFVMMSAPPAQYELKHGTFTCASEYTGNYQCGHYKHITSKETLYCIDGALLTKSSEYK
EPI_ISL_424217   983 ESPFVMMSAPPAQYELKHGTFTCASEYTGNYQCGHYKHITSKETLYCIDGALLTKSSEYK
EPI_ISL_424218   983 ESPFVMMSAPPAQYELKHGTFTCASEYTGNYQCGHYKHITSKETLYCIDGALLTKSSEYK
EPI_ISL_424220   983 ESPFVMMSAPPAQYELKHGTFTCASEYTGNYQCGHYKHITSKETLYCIDGALLTKSSEYK
EPI_ISL_424232   983 ESPFVMMSAPPAQYELKHGTFTCASEYTGNYQCGHYKHITSKETLYCIDGALLTKSSEYK
EPI_ISL_424266   983 ESPFVMMSAPPAQYELKHGTFTCASEYTGNYQCGHYKHITSKETLYCIDGALLTKSSEYK
EPI_ISL_424272   983 ESPFVMMSAPPAQYELKHGTFTCASEYTGNYQCGHYKHITSKETLYCIDGALLTKSSEYK
EPI_ISL_424283   983 ESPFVMMSAPPAQYELKHGTFTCASEYTGNYQCGHYKHITSKETLYCIDGALLTKSSEYK
EPI_ISL_424288   983 ESPFVMMSAPPAQYELKHGTFTCASEYTGNYQCGHYKHITSKETLYCIDGALLTKSSEYK
EPI_ISL_424292   983 ESPFVMMSAPPAQYELKHGTFTCASEYTGNYQCGHYKHITSKETLYCIDGALLTKSSEYK
EPI_ISL_424295   983 ESPFVMMSAPPAQYELKHGTFTCASEYTGNYQCGHYKHITSKETLYCIDGALLTKSSEYK
EPI_ISL_426882   983 ESPFVMMSAPPAQYELKHGTFTCASEYTGNYQCGHYKHITSKETLYCIDGALLTKSSEYK
```

```
ORF1ab          1861 GPITDVFYKENSYTTTIKPVTYKLDGVVCTEIDPKLDNYYKKDNSYFTEQPIDLVPNQPY
EPI_ISL_413594  1043 GPITDVFYKENSYTTTIKPVTYKLDGVVCTEIDPKLDNYYKKDNSYFTEQPIDLVPNQPY
EPI_ISL_413595  1043 GPITDVFYKENSYTTTIKPVTYKLDGVVCTEIDPKLDNYYKKDNSYFTEQPIDLVPNQPY
EPI_ISL_413596  1043 GPITDVFYKENSYTTTIKPVTYKLDGVVCTEIDPKLDNYYKKDNSYFTEQPIDLVPNQPY
EPI_ISL_413597  1043 GPITDVFYKENSYTTTIKPVTYKLDGVVCTEIDPKLDNYYKKDNSYFTEQPIDLVPNQPY
EPI_ISL_413598  1043 GPITDVFYKENSYTTTIKPVTYKLDGVVCTEIDPKLDNYYKKDNSYFTEQPIDLVPNQPY
EPI_ISL_413599  1043 GPITDVFYKENSYTTTIKPVTYKLDGVVCTEIDPKLDNYYKKDNSYFTEQPIDLVPNQPY
EPI_ISL_413600  1043 GPITDVFYKENSYTTTIKPVTYKLDGVVCTEIDPKLDNYYKKDNSYFTEQPIDLVPNQPY
EPI_ISL_413601  1043 GPITDVFYKENSYTTTIKPVTYKLDGVVCTEIDPKLDNYYKKDNSYFTEQPIDLVPNQPY
EPI_ISL_424217  1043 GPITDVFYKENSYTTTIKPVTYKLDGVVCTEIDPKLDNYYKKDNSYFTEQPIDLVPNQPY
EPI_ISL_424218  1043 GPITDVFYKENSYTTTIKPVTYKLDGVVCTEIDPKLDNYYKKDNSYFTEQPIDLVPNQPY
EPI_ISL_424220  1043 GPITDVFYKENSYTTTIKPVTYKLDGVVCTEIDPKLDNYYKKDNSYFTEQPIDLVPNQPY
EPI_ISL_424232  1043 GPITDVFYKENSYTTTIKPVTYKLDGVVCTEIDPKLDNYYKKDNSYFTEQPIDLVPNQPY
EPI_ISL_424266  1043 GPITDVFYKENSYTTTIKPVTYKLDGVVCTEIDPKLDNYYKKDNSYFTEQPIDLVPNQPY
EPI_ISL_424272  1043 GPITDVFYKENSYTTTIKPVTYKLDGVVCTEIDPKLDNYYKKDNSYFTEQPIDLVPNQPY
EPI_ISL_424283  1043 GPITDVFYKENSYTTTIKPVTYKLDGVVCTEIDPKLDNYYKKDNSYFTEQPIDLVPNQPY
EPI_ISL_424288  1043 GPITDVFYKENSYTTTIKPVTYKLDGVVCTEIDPKLDNYYKKDNSYFTEQPIDLVPNQPY
EPI_ISL_424292  1043 GPITDVFYKENSYTTTIKPVTYKLDGVVCTEIDPKLDNYYKKDNSYFTEQPIDLVPNQPY
EPI_ISL_424295  1043 GPITDVFYKENSYTTTIKPVTYKLDGVVCTEIDPKLDNYYKKDNSYFTEQPIDLVPNQPY
EPI_ISL_426882  1043 GPITDVFYKENSYTTTIKPVTYKLDGVVCTEIDPKLDNYYKKDNSYFTEQPIDLVPNQPY
```

```
ORF1ab          1921 PNASFDNFKFVCDNIKFADDLNQLTGYKKPASRELKVTFFPDLNGDVVAIDYKHYTPSFK
EPI_ISL_413594  1103 PNASFDNFKFVCDNIKFADDLNQLTGYKKPASRELKVTFFPDLNGDVVAIDYKHYTPSFK
EPI_ISL_413595  1103 PNASFDNFKFVCDNIKFADDLNQLTGYKKPASRELKVTFFPDLNGDVVAIDYKHYTPSFK
EPI_ISL_413596  1103 PNASXXXFKFVCDNIKFADDLNQLTGYKKPASRELKVTFFPDLNGDVVAIDYKHYTPSFK
EPI_ISL_413597  1103 PNASFDNFKFVCDNIKFADDLNQLTGYKKPASRELKVTFFPDLNGDVVAIDYKHYTPSFK
EPI_ISL_413598  1103 PNASFDNFKFVCDNIKFADDLNQLTGYKKPASRELKVTFFPDLNGDVVAIDYKHYTPSFK
EPI_ISL_413599  1103 PNASFDNFKFVCDNIKFADDLNQLTGYKKPASRELKVTFFPDLNGDVVAIDYKHYTPSFK
EPI_ISL_413600  1103 PNASFDNFKFVCDNIKFADDLNQLTGYKKPASRELKVTFFPDLNGDVVAIDYKHYTPSFK
EPI_ISL_413601  1103 PNASFDNFKFVCDNIKFADDLNQLTGYKKPASRELKVTFFPDLNGDVVAIDYKHYTPSFK
EPI_ISL_424217  1103 PNASFDNFKFVCDNIKFADDLNQLTGYKKPASRELKVTFFPDLNGDVVAIDYKHYTPSFK
EPI_ISL_424218  1103 PNASFDNFKFVCDNIKFADDLNQLTGYKKPASRELKVTFFPDLNGDVVAIDYKHYTPSFK
EPI_ISL_424220  1103 PNASFDNFKFVCDNIKFADDLNQLTGYKKPASRELKVTFFPDLNGDVVAIDYKHYTPSFK
EPI_ISL_424232  1103 PNASFDNFKFVCDNIKFADDLNQLTGYKKPASRELKVTFFPDLNGDVVAIDYKHYTPSFK
EPI_ISL_424266  1103 PNASFDNFKFVCDNIKFADDLNQLTGYKKPASRELKVTFFPDLNGDVVAIDYKHYTPSFK
EPI_ISL_424272  1103 PNASFDNFKFVCDNIKFADDLNQLTGYKKPASRELKVTFFPDLNGDVVAIDYKHYTPSFK
EPI_ISL_424283  1103 PNASFDNFKFVCDNIKFADDLNQLTGYKKPASRELKVTFFPDLNGDVVAIDYKHYTPSFK
EPI_ISL_424288  1103 PNASFDNFKFVCDNIKFADDLNQLTGYKKPASRELKVTFFPDLNGDVVAIDYKHYTPSFK
EPI_ISL_424292  1103 PNASFDNFKFVCDNIKFADDLNQLTGYKKPASRELKVTFFPDLNGDVVAIDYKHYTPSFK
EPI_ISL_424295  1103 PNASFDNFKFVCDNIKFADDLNQLTGYKKPASRELKVTFFPDLNGDVVAIDYKHYTPSFK
EPI_ISL_426882  1103 PNASFDNFKFVCDNIKFADDLNQLTGYKKPASRELKVTFFPDLNGDVVAIDYKHYTPSFK
```

```
ORF1ab         1981 KGAKLLHKPIVWHVNNATNKATYKPNTWCIRCLWSTKPVETSNSFDVLKSEDAQGMDNLA
EPI_ISL_413594 1163 KGAKLLHKPIVWHVNNATNKATYKPNTWCIRCLWSTKPVETSNSFDVLKSEDAQGMDNLA
EPI_ISL_413595 1163 KGAKLLHKPIVWHVNNATNKATYKPNTWCIRCLWSTKPVETSNSFDVLKSEDAQGMDNLA
EPI_ISL_413596 1163 KGAKLLHKPIVWHVNNATNKATYKPNTWCIRCLWSTKPVETSNSFDVLKSEDAQGMDNLA
EPI_ISL_413597 1163 KGAKLLHKPIVWHVNNATNKATYKPNTWCIRCLWRKKPVETSNSFDVLKSEDAQGMDNLA
EPI_ISL_413598 1163 KGAKLLHKPIVWHVNNATNKATYKPNTWCIRCLWSTKPVETSNSFDVLKSEDAQGMDNLA
EPI_ISL_413599 1163 KGAKLLHKPIVWHVNNATNKATYKPNTWCIRCLWSTKPVETSNSFDVLKSEDAQGMDNLA
EPI_ISL_413600 1163 KGAKLLHKPIVWHVNNATNKATYKPNTWCIRCLWSTKPVETSNSFDVLKSEDAQGMDNLA
EPI_ISL_413601 1163 KGAKLLHKPIVWHVNNATNKATYKPNTWCIRCLWSTKPVETSNSFDVLKSEDAQGMDNLA
EPI_ISL_424217 1163 KGAKLLHKPIVWHVNNATNKATYKPNTWCIRCLWSTKPVETSNSFDVLKSEDAQGMDNLA
EPI_ISL_424218 1163 KGAKLLHKPIVWHVNNATNKATYKPNTWCIRCLWSTKPVETSNSFDVLKSEDAQGMDNLA
EPI_ISL_424220 1163 KGAKLLHKPIVWHVNNATNKATYKPNTWCIRCLWSTKPVETSNSFDVLKSEDAQGMDNLA
EPI_ISL_424232 1163 KGAKLLHKPIVWHVNNATNKATYKPNTWCIRCLWSTKPVETSNSFDVLKSEDAQGMDNLA
EPI_ISL_424266 1163 KGAKLLHKPIVWHVNNATNKATYKPNTWCIRCLWSTKPVETSNSFDVLKSEDAQGMDNLA
EPI_ISL_424272 1163 KGAKLLHKPIVWHVNNATNKATYKPNTWCIRCLWSTKPVETSNSFDVLKSEDAQGMDNLA
EPI_ISL_424283 1163 KGAKLLHKPIVWHVNNATNKATYKPNTWCIRCLWSTKPVETSNSFDVLKSEDAQGMDNLA
EPI_ISL_424288 1163 KGAKLLHKPIVWHVNNATNKATYKPNTWCIRCLWSTKPVETSNSFDVLKSEDAQGMDNLA
EPI_ISL_424292 1163 KGAKLLHKPIVWHVNNATNKATYKPNTWCIRCLWSTKPVETSNSFDVLKSEDAQGMDNLA
EPI_ISL_424295 1163 KGAKLLHKPIVWHVNNATNKATYKPNTWCIRCLWSTKPVETSNSFDVLKSEDAQGMDNLA
EPI_ISL_426882 1163 KGAKLLHKPIVWHVNNATNKATYKPNTWCIRCLWSTKPVETSNSFDVLKSEDAQGMDNLA

ORF1ab         2041 CEDLKPVSEEVVENPTIQKDVLECNVKTTEVVGDIILKPANNSLKITEEVGHTDLMAAYV
EPI_ISL_413594 1223 CEDLKPVSEEVVENPTIQKDVLECNVKTTEVVGDIILKPANNSLKITEEVGHTDLMAAYV
EPI_ISL_413595 1223 CEDLKPVSEEVVENPTIQKDVLECNVKTTEVVGDIILKPANNSLKITEEVGHTDLMAAYV
EPI_ISL_413596 1223 CEDLKPVSEEVVENPTIQKDVLECNVKTTEVVGDIILKPANNSLKITEEVGHTDLMAAYV
EPI_ISL_413597 1223 CEDLKPVSEEVVENPTIQKDVLECNVKTTEVVGDIILKPANNSLKITEEVGHTDLMAAYV
EPI_ISL_413598 1223 CEDLKPVSEEVVENPTIQKDVLECNVKTTEVVGDIILKPANNSLKITEEVGHTDLMAAYV
EPI_ISL_413599 1223 CEDLKPVSEEVVENPTIQKDVLECNVKTTEVVGDIILKPANNSLKITEEVGHTDLMAAYV
EPI_ISL_413600 1223 CEDLKPVSEEVVENPTIQKDVLECNVKTTEVVGDIILKPANNSLKITEEVGHTDLMAAYV
EPI_ISL_413601 1223 CEDLKPVSEEVVENPTIQKDVLECNVKTTEVVGDIILKPANNSLKITEEVGHTDLMAAYV
EPI_ISL_424217 1223 CEDLKPVSEEVVENPTIQKDVLECNVKTTEVVGDIILKPANNSLKITEEVGHTDLMAAYV
EPI_ISL_424218 1223 CEDLKPVSEEVVENPTIQKDVLECNVKTTEVVGDIILKPANNSLKITEEVGHTDLMAAYV
EPI_ISL_424220 1223 CEDLKPVSEEVVENPTIQKDVLECNVKTTEVVGDIILKPANNSLKITEEVGHTDLMAAYV
EPI_ISL_424232 1223 CEDLKPVSEEVVENPTIQKDVLECNVKTTEVVGDIILKPANNSLKITEEVGHTDLMAAYV
EPI_ISL_424266 1223 CEDLKPVSEEVVENPTIQKDVLECNVKTTEVVGDIILKPANNSLKITEEVGHTDLMAAYV
EPI_ISL_424272 1223 CEDLKPVSEEVVENPTIQKDVLECNVKTTEVVGDIILKPANNSLKITEEVGHTDLMAAYV
EPI_ISL_424283 1223 CEDLKPVSEEVVENPTIQKDVLECNVKTTEVVGDIILKPANNSLKITEEVGHTDLMAAYV
EPI_ISL_424288 1223 CEDLKPVSEEVVENPTIQKDVLECNVKTTEVVGDIILKPANNSLKITEEVGHTDLMAAYV
EPI_ISL_424292 1223 CEDLKPVSEEVVENPTIQKDVLECNVKTTEVVGDIILKPANNSLKITEEVGHTDLMAAYV
EPI_ISL_424295 1223 CEDLKPVSEEVVENPTIQKDVLECNVKTTEVVGDIILKPANNSLKITEEVGHTDLMAAYV
EPI_ISL_426882 1223 CEDLKPVSEEVVENPTIQKDVLECNVKTTEVVGDIILKPANNSLKITEEVGHTDLMAAYV

ORF1ab         2101 DNSSLTIKKPNELSRVLGLKTLATHGLAAVNSVPWDTIANYAKPFLNKVVSTTTNIVTRC
EPI_ISL_413594 1283 DNSSLTIKKPNELSRVLGLKTLATHGLAAVNSVPWDTIANYAKPFLNKVVSTTTNIVTRC
EPI_ISL_413595 1283 DNSSLTIKKPNELSRVLGLKTLATHGLAAVNSVPWDTIANYAKPFLNKVVSTTTNIVTRC
EPI_ISL_413596 1283 DNSSLTIKKPNELSRVLGLKTLATHGLAAVNSVPWDTIANYAKPFLNKVVSTTTNIVTRC
EPI_ISL_413597 1283 DNSSLTIKKPNELSRVLGLKTLATHGLAAVNSVPWDTIANYAKPFLNKVVSTTTNIVTRC
EPI_ISL_413598 1283 DNSSLTIKKPNELSRVLGLKTLATHGLAAVNSVPWDTIANYAKPFLNKVVSTTTNIVTRC
EPI_ISL_413599 1283 DNSSLTIKKPNELSRVLGLKTLATHGLAAVNSVPWDTIANYAKPFLNKVVSTTTNIVTRC
EPI_ISL_413600 1283 DNSSLTIKKPNELSRVLGLKTLATHGLAAVNSVPWDTIANYAKPFLNKVVSTTTNIVTRC
EPI_ISL_413601 1283 DNSSLTIKKPNELSRVLGLKTLATHGLAAVNSVPWDTIANYAKPFLNKVVSTTTNIVTRC
EPI_ISL_424217 1283 DNSSLTIKKPNELSRVLGLKTLATHGLAAVNSVPWDTIANYAKPFLNKVVSTTTNIVTRC
EPI_ISL_424218 1283 DNSSLTIKKPNELSRVLGLKTLATHGLAAVNSVPWDTIANYAKPFLNKVVSTTTNIVTRC
EPI_ISL_424220 1283 DNSSLTIKKPNELSRVLGLKTLATHGLAAVNSVPWDTIANYAKPFLNKVVSTTTNIVTRC
EPI_ISL_424232 1283 DNSSLTIKKPNELSRVLGLKTLATHGLAAVNSVPWDTIANYAKPFLNKVVSTTTNIVTRC
EPI_ISL_424266 1283 DNSSLTIKKPNELSRVLGLKTLATHGLAAVNSVPWDTIANYAKPFLNKVVSTTTNIVTRC
EPI_ISL_424272 1283 DNSSLTIKKPNELSRVLGLKTLATHGLAAVNSVPWDTIANYAKPFLNKVVSTTTNIVTRC
EPI_ISL_424283 1283 DNSSLTIKKPNELSRVLGLKTLATHGLAAVNSVPWDTIANYAKPFLNKVVSTTTNIVTRC
EPI_ISL_424288 1283 DNSSLTIKKPNELSRVLGLKTLATHGLAAVNSVPWDTIANYAKPFLNKVVSTTTNIVTRC
EPI_ISL_424292 1283 DNSSLTIKKPNELSRVLGLKTLATHGLAAVNSVPWDTIANYAKPFLNKVVSTTTNIVTRC
EPI_ISL_424295 1283 DNSSLTIKKPNELSRVLGLKTLATHGLAAVNSVPWDTIANYAKPFLNKVVSTTTNIVTRC
EPI_ISL_426882 1283 DNSSLTIKKPNELSRVLGLKTLATHGLAAVNSVPWDTIANYAKPFLNKVVSTTTNIVTRC
```

```
ORF1ab          2161 LNRVCTNYMPYFFTLLLQLCTFTRSTNSRIKASMPTTIAKNTVKSVGKFCLEASFNYLKS
EPI_ISL_413594 1343 LNRVCTNYMPYFFTLLLQLCTFTRSTNSRIKASMPTTIAKNXXXXXXXXXXXXSFNYLKX
EPI_ISL_413595 1343 LNRVCTNYMPYFFTLLLQLCTFTRSTNSRIKASMPTTIXXXXXXXXXXXXXXXXXXXXX
EPI_ISL_413596 1343 LNRVCTNYMPYFFTLLLQLCTFTRSTNSRIKASMPTTIAKNTVKSVGKFCLEASFNYLKS
EPI_ISL_413597 1343 LNRVCTNYMPYFFTLLLQLCTFTRSTNSRIKASMPTTIAKNTVKSVGKFCLEASFNYLKS
EPI_ISL_413598 1343 LNRVCTNYMPYFFTLLLQLCTFTRSTNSRIKASMPTTIAKNTVKSVGKFCLEASFNYLKS
EPI_ISL_413599 1343 LNRVCTNYMPYFFTLLLQLCTFTRSTNSRIKASMPTTIAKNTVKSVGKFCLEASFNYLKS
EPI_ISL_413600 1343 LNRVCTNYMPYFFTLLLQLCTFTRSTNSRIKASMPTTIAKNTVKSVGKFCLEASFNYLKS
EPI_ISL_413601 1343 LNRVCTNYMPYFFTLLLQLCTFTRSTNSRIKASMPTTIAKNTVKSVGKFCLEASFNYLKS
EPI_ISL_424217 1343 LNRVCTNYMPYFFTLLLQLCTFTRSTNSRIKASMPTTIAKNTVKSVGKFCLEASFNYLKS
EPI_ISL_424218 1343 LNRVCTNYMPYFFTLLLQLCTFTRSTNSRIKASMPTTIAKNTVKSVGKFCLEASFNYLKS
EPI_ISL_424220 1343 LNRVCTNYMPYFFTLLLQLCTFTRSTNSRIKASMPTTIAKNTVKSVGKFCLEASFNYLKS
EPI_ISL_424232 1343 LNRVCTNYMPYFFTLLLQLCTFTRSTNSRIKASMPTTIAKNTVKSVGKFCLEASFNYLKS
EPI_ISL_424266 1343 LNRVCTNYMPYFFTLLLQLCTFTRSTNSRIKASMPTTIAKNTVKSVGKFCLEASFNYLKS
EPI_ISL_424272 1343 LNRVCTNYMPYFFTLLLQLCTFTRSTNSRIKASMPTTIAKNTVKSVGKFCLEASFNYLKS
EPI_ISL_424283 1343 LNRVCTNYMPYFFTLLLQLCTFTRSTNSRIKASMPTTIAKNTVKSVGKFCLEASFNYLKS
EPI_ISL_424288 1343 LNRVCTNYMPYFFTLLLQLCTFTRSTNSRIKASMPTTIAKNTVKSVGKFCLEASFNYLKS
EPI_ISL_424292 1343 LNRVCTNYMPYFFTLLLQLCTFTRSTNSRIKASMPTTIAKNTVKSVGKFCLEASFNYLKS
EPI_ISL_424295 1343 LNRVCTNYMPYFFTLLLQLCTFTRSTNSRIKASMPTTIAKNTVKSVGKFCLEASFNYLKS
EPI_ISL_426882 1343 LNRVCTNYMPYFFTLLLQLCTFTRSTNSRIKASMPTTIAKNTVKSVGKFCLEASFNYLKS

ORF1ab          2221 PNFSKLINIIIWFLLLSVCLGSLIYSTAALGVLMSNLGMPSYCTGYREGYLNSTNVTIAT
EPI_ISL_413594 1403 PNFSKXXXIIIWFLLLSVCLGSLIYSTAALGVLMSNLGMPSYCTGYREGYLNSTNVTIAT
EPI_ISL_413595 1403 XXXXKLINIIIWFLLLSVCLGSLIYSTAALGVLMSNLGMPSYCTGYREGYLNSTNVTIAT
EPI_ISL_413596 1403 PNFSKLINIIIWFLLLSVCLGSLIYSTAALGVLMSNLGMPSYCTGYREGYLNSTNVTIAT
EPI_ISL_413597 1403 PNFSKLINIIIWFLLLSVCLGSLIYSTAALGVLMSNLGMPSYCTGYREGYLNSTNVTIAT
EPI_ISL_413598 1403 PNFSKLINIIIWFLLLSVCLGSLIYSTAALGVLMSNLGMPSYCTGYREGYLNSTNVTIAT
EPI_ISL_413599 1403 PNFSKLINIIIWFLLLSVCLGSLIYSTAALGVLMSNLGMPSYCTGYREGYLNSTNVTIAT
EPI_ISL_413600 1403 PNFSKLINIIIWFLLLSVCLGSLIYSTAALGVLMSNLGMPSYCTGYREGYLNSTNVTIAT
EPI_ISL_413601 1403 PNFSKLINIIIWFLLLSVCLGSLIYSTAALGVLMSNLGMPSYCTGYREGYLNSTNVTIAT
EPI_ISL_424217 1403 PNFSKLINIIIWFLLLSVCLGSLIYSTAALGVLMSNLGMPSYCTGYREGYLNSTNVTIAT
EPI_ISL_424218 1403 PNFSKLINIIIWFLLLSVCLGSLIYSTAALGVLMSNLGMPSYCTGYREGYLNSTNVTIAT
EPI_ISL_424220 1403 PNFSKLINIIIWFLLLSVCLGSLIYSTAALGVLMSNLGMPSYCTGYREGYLNSTNVTIAT
EPI_ISL_424232 1403 PNFSKLINIIIWFLLLSVCLGSLIYSTAALGVLMSNLGMPSYCTGYREGYLNSTNVTIAT
EPI_ISL_424266 1403 PNFSKLINIIIWFLLLSVCLGSLIYSTAALGVLMSNLGMPSYCTGYREGYLNSTNVTIAT
EPI_ISL_424272 1403 PNFSKLINIIIWFLLLSVCLGSLIYSTAALGVLMSNLGMPSYCTGYREGYLNSTNVTIAT
EPI_ISL_424283 1403 PNFSKLINIIIWFLLLSVCLGSLIYSTAALGVLMSNLGMPSYCTGYREGYLNSTNVTIAT
EPI_ISL_424288 1403 PNFSKLINIIIWFLLLSVCLGSLIYSTAALGVLMSNLGMPSYCTGYREGYLNSTNVTIAT
EPI_ISL_424292 1403 PNFSKLINIIIWFLLLSVCLGSLIYSTAALGVLMSNLGMPSYCTGYREGYLNSTNVTIAT
EPI_ISL_424295 1403 PNFSKLINIIIWFLLLSVCLGSLIYSTAALGVLMSNLGMPSYCTGYREGYLNSTNVTIAT
EPI_ISL_426882 1403 PNFSKLINIIIWFLLLSVCLGSLIYSTAALGVLMSNLGMPSYCTGYREGYLNSTNVTIAT

ORF1ab          2281 YCTGSIPCSVCLSGLDSLDTYPSLETIQITISSFKWDLTAFGLVAEWFLAYILFTRFFYV
EPI_ISL_413594 1463 YCTGSIPCSVCLSGLDSLDTYPSLETIQITISSFKWDLTAFGLVAEWFLAYILFTRFFYV
EPI_ISL_413595 1463 YCTGSIPCSVCLSGLDSLDTYPSLETIQITISSFKWDLTAFGLVAEWFLAYILFTRFFYV
EPI_ISL_413596 1463 YCTGSIPCSVCLSGLDSLDTYPSLETIQITISSFKWDLTAFGLVAEWFLAYILFTRFFYV
EPI_ISL_413597 1463 YCTGSIPCSVCLSGLDSLDTYPSLETIQITISSFKWDLTAFGLVAEWFLAYILFTRFFYV
EPI_ISL_413598 1463 YCTGSIPCSVCLSGLDSLDTYPSLETIQITISSFKWDLTAFGLVAEWFLAYILFTRFFYV
EPI_ISL_413599 1463 YCTGSIPCSVCLSGLDSLDTYPSLETIQITISSFKWDLTAFGLVAEWFLAYILFTRFFYV
EPI_ISL_413600 1463 YCTGSIPCSVCLSGLDSLDTYPSLETIQITISSFKWDLTAFGLVAEWFLAYILFTRFFYV
EPI_ISL_413601 1463 YCTGSIPCSVCLSGLDSLDTYPSLETIQITISSFKWDLTAFGLVAEWFLAYILFTRFFYV
EPI_ISL_424217 1463 YCTGSIPCSVCLSGLDSLDTYPSLETIQITISSFKWDLTAFGLVAEWFLAYILFTRFFYV
EPI_ISL_424218 1463 YCTGSIPCSVCLSGLDSLDTYPSLETIQITISSFKWDLTAFGLVAEWFLAYILFTRFFYV
EPI_ISL_424220 1463 YCTGSIPCSVCLSGLDSLDTYPSLETIQITISSFKWDLTAFGLVAEWFLAYILFTRFFYV
EPI_ISL_424232 1463 YCTGSIPCSVCLSGLDSLDTYPSLETIQITISSFKWDLTAFGLVAEWFLAYILFTRFFYV
EPI_ISL_424266 1463 YCTGSIPCSVCLSGLDSLDTYPSLETIQITISSFKWDLTAFGLVAEWFLAYILFTRFFYV
EPI_ISL_424272 1463 YCTGSIPCSVCLSGLDSLDTYPSLETIQITISSFKWDLTAFGLVAEWFLAYILFTRFFYV
EPI_ISL_424283 1463 YCTGSIPCSVCLSGLDSLDTYPSLETIQITISSFKWDLTAFGLVAEWFLAYILFTRFFYV
EPI_ISL_424288 1463 YCTGSIPCSVCLSGLDSLDTYPSLETIQITISSFKWDLTAFGLVAEWFLAYILFTRFFYV
EPI_ISL_424292 1463 YCTGSIPCSVCLSGLDSLDTYPSLETIQITISSFKWDLTAFGLVAEWFLAYILFTRFFYV
EPI_ISL_424295 1463 YCTGSIPCSVCLSGLDSLDTYPSLETIQITISSFKWDLTAFGLVAEWFLAYILFTRFFYV
EPI_ISL_426882 1463 YCTGSIPCSVCLSGLDSLDTYPSLETIQITISSFKWDLTAFGLVAEWFLAYILFTRFFYV
```

```
ORF1ab         2341 LGLAAIMQLFFSYFAVHFISNSWLMWLIINLVQMAPISAMVRMYIFFASFYYVWKSYVHV
EPI_ISL_413594 1523 LGLAAIMQLFFSYFAVHFISNSWLMWLIINXXQMAPISXXVRMYIFFASFYYVWKSYVHV
EPI_ISL_413595 1523 LGLAAIMQLFFSYFAVHFISNSWLMWLIINLVQMAPISAMVRMYIFFASFYYVWKSYVHV
EPI_ISL_413596 1523 LGLAAIMQLFFSYFAVHFISNSWLMWLIINLVQMAPISAMVRMYIFFASFYYVWKSYVHV
EPI_ISL_413597 1523 LGLAAIMQLFFSYFAVHFISNSWLMWLIINLVQMAPISAMVRMYIFFASFYYVWKSYVHV
EPI_ISL_413598 1523 LGLAAIMQLFFSYFAVHFISNSWLMWLIINLVQMAPISAMVRMYIFFASFYYVWKSYVHV
EPI_ISL_413599 1523 LGLAAIMQLFFSYFAVHFISNSWLMWLIINLVQMAPISAMVRMYIFFASFYYVWKSYVHV
EPI_ISL_413600 1523 LGLAAIMQLFFSYFAVHFISNSWLMWLIINLVQMAPISAMVRMYIFFASFYYVWKSYVHV
EPI_ISL_413601 1523 LGLAAIMQLFFSYFAVHFISNSWLMWLIINLVQMAPISAMVRMYIFFASFYYVWKSYVHV
EPI_ISL_424217 1523 LGLAAIMQLFFSYFAVHFISNSWLMWLIINLVQMAPISAMVRMYIFFASFYYVWKSYVHV
EPI_ISL_424218 1523 LGLAAIMQLFFSYFAVHFISNSWLMWLIINLVQMAPISAMVRMYIFFASFYYVWKSYVHV
EPI_ISL_424220 1523 LGLAAIMQLFFSYFAVHFISNSWLMWLIINLVQMAPISAMVRMYIFFASFYYVWKSYVHV
EPI_ISL_424232 1523 LGLAAIMQLFFSYFAVHFISNSWLMWLIINLVQMAPISAMVRMYIFFASFYYVWKSYVHV
EPI_ISL_424266 1523 LGLAAIMQLFFSYFAVHFISNSWLMWLIINLVQMAPISAMVRMYIFFASFYYVWKSYVHV
EPI_ISL_424272 1523 LGLAAIMQLFFSYFAVHFISNSWLMWLIINLVQMAPISAMVRMYIFFASFYYVWKSYVHV
EPI_ISL_424283 1523 LGLAAIMQLFFSYFAVHFISNSWLMWLIINLVQMAPISAMVRMYIFFASFYYVWKSYVHV
EPI_ISL_424288 1523 LGLAAIMQLFFSYFAVHFISNSWLMWLIINLVQMAPISAMVRMYIFFASFYYVWKSYVHV
EPI_ISL_424292 1523 LGLAAIMQLFFSYFAVHFISNSWLMWLIINLVQMAPISAMVRMYIFFASFYYVWKSYVHV
EPI_ISL_424295 1523 LGLAAIMQLFFSYFAVHFISNSWLMWLIINLVQMAPISAMVRMYIFFASFYYVWKSYVHV
EPI_ISL_426882 1523 LGLAAIMQLFFSYFAVHFISNSWLMWLIINLVQMAPISAMVRMYIFFASFYYVWKSYVHV
```

```
ORF1ab         2401 VDGCNSSTCMMCYKRNRATRVECTTIVNGVRRSFYVYANGGKGFCKLHNWNCVNCDTFCA
EPI_ISL_413594 1583 VDGCNSSTCMMCYKRNRATRVECTTIVNGVRRSFYVYANGGKGFCKLHNWNCVNCDTFCA
EPI_ISL_413595 1583 VDGCNSSTCMMCYKRNRATRVECTTIVNGVRRSFYVYANGGKGFCKLHNWNCVNCDTFCA
EPI_ISL_413596 1583 VDGCNSSTCMMCYKRNRATRVECTTIVNGVRRSFYVYANGGKGFCKLHNWNCVNCDTFCA
EPI_ISL_413597 1583 VDGCNSSTCMMCYKRNRATRVECTTIVNGVRRSFYVYANGGKGFCKLHNWNCVNCDTFCA
EPI_ISL_413598 1583 VDGCNSSTCMMCYKRNRATRVECTTIVNGVRRSFYVYANGGKGFCKLHNWNCVNCDTFCA
EPI_ISL_413599 1583 VDGCNSSTCMMCYKRNRATRVECTTIVNGVRRSFYVYANGGKGFCKLHNWNCVNCDTFCA
EPI_ISL_413600 1583 VDGCNSSTCMMCYKRNRATRVECTTIVNGVRRSFYVYANGGKGFCKLHNWNCVNCDTFCA
EPI_ISL_413601 1583 VDGCNSSTCMMCYKRNRATRVECTTIVNGVRRSFYVYANGGKGFCKLHNWNCVNCDTFCA
EPI_ISL_424217 1583 VDGCNSSTCMMCYKRNRATRVECTTIVNGVRRSFYVYANGGKGFCKLHNWNCVNCDTFCA
EPI_ISL_424218 1583 VDGCNSSTCMMCYKRNRATRVECTTIVNGVRRSFYVYANGGKGFCKLHNWNCVNCDTFCA
EPI_ISL_424220 1583 VDGCNSSTCMMCYKRNRATRVECTTIVNGVRRSFYVYANGGKGFCKLHNWNCVNCDTFCA
EPI_ISL_424232 1583 VDGCNSSTCMMCYKRNRATRVECTTIVNGVRRSFYVYANGGKGFCKLHNWNCVNCDTFCA
EPI_ISL_424266 1583 VDGCNSSTCMMCYKRNRATRVECTTIVNGVRRSFYVYANGGKGFCKLHNWNCVNCDTFCA
EPI_ISL_424272 1583 VDGCNSSTCMMCYKRNRATRVECTTIVNGVRRSFYVYANGGKGFCKLHNWNCVNCDTFCA
EPI_ISL_424283 1583 VDGCNSSTCMMCYKRNRATRVECTTIVNGVRRSFYVYANGGKGFCKLHNWNCVNCDTFCA
EPI_ISL_424288 1583 VDGCNSSTCMMCYKRNRATRVECTTIVNGVRRSFYVYANGGKGFCKLHNWNCVNCDTFCA
EPI_ISL_424292 1583 VDGCNSSTCMMCYKRNRATRVECTTIVNGVRRSFYVYANGGKGFCKLHNWNCVNCDTFCA
EPI_ISL_424295 1583 VDGCNSSTCMMCYKRNRATRVECTTIVNGVRRSFYVYANGGKGFCKLHNWNCVNCDTFCA
EPI_ISL_426882 1583 VDGCNSSTCMMCYKRNRATRVECTTIVNGVRRSFYVYANGGKGFCKLHNWNCVNCDTFCA
```

```
ORF1ab         2461 GSTFISDEVARDLSLQFKRPINPTDQSSYIVDSVTVKNGSIHLYFDKAGQKTYERHSLSH
EPI_ISL_413594 1643 GSTFISDEVARDLSLQFKRPINPTDQSSYIVDSVTVKNGSIHLYFDKAGQKTYERHSLSH
EPI_ISL_413595 1643 GSTFISDEVARDLSLQFKRPINPTDQSSYIVDSVTVKNGSIHLYFDKAGQKTYERHSLSH
EPI_ISL_413596 1643 GSTFISDEVARDLSLQFKRPINPTDQSSYIVDSVTVKNGSIHLYFDKAGQKTYERHSLSH
EPI_ISL_413597 1643 GSTFISDEVARDLSLQFKRPINPTDQSSYIVDSVTVKNGSIHLYFDKAGQKTYERHSLSH
EPI_ISL_413598 1643 GSTFISDEVARDLSLQFKRPINPTDQSSYIVDSVTVKNGSIHLYFDKAGQKTYERHSLSH
EPI_ISL_413599 1643 GSTFISDEVARDLSLQFKRPINPTDQSSYIVDSVTVKNGSIHLYFDKAGQKTYERHSLSH
EPI_ISL_413600 1643 GSTFISDEVARDLSLQFKRPINPTDQSSYIVDSVTVKNGSIHLYFDKAGQKTYERHSLSH
EPI_ISL_413601 1643 GSTFISDEVARDLSLQFKRPINPTDQSSYIVDSVTVKNGSIHLYFDKAGQKTYERHSLSH
EPI_ISL_424217 1643 GSTFISDEVARDLSLQFKRPINPTDQSSYIVDSVTVKNGSIHLYFDKAGQKTYERHSLSH
EPI_ISL_424218 1643 GSTFISDEVARDLSLQFKRPINPTDQSSYIVDSVTVKNGSIHLYFDKAGQKTYERHSLSH
EPI_ISL_424220 1643 GSTFISDEVARDLSLQFKRPINPTDQSSYIVDSVTVKNGSIHLYFDKAGQKTYERHSLSH
EPI_ISL_424232 1643 GSTFISDEVARDLSLQFKRPINPTDQSSYIVDSVTVKNGSIHLYFDKAGQKTYERHSLSH
EPI_ISL_424266 1643 GSTFISDEVARDLSLQFKRPINPTDQSSYIVDSVTVKNGSIHLYFDKAGQKTYERHSLSH
EPI_ISL_424272 1643 GSTFISDEVARDLSLQFKRPINPTDQSSYIVDSVTVKNGSIHLYFDKAGQKTYERHSLSH
EPI_ISL_424283 1643 GSTFISDEVARDLSLQFKRPINPTDQSSYIVDSVTVKNGSIHLYFDKAGQKTYERHSLSH
EPI_ISL_424288 1643 GSTFISDEVARDLSLQFKRPINPTDQSSYIVDSVTVKNGSIHLYFDKAGQKTYERHSLSH
EPI_ISL_424292 1643 GSTFISDEVARDLSLQFKRPINPTDQSSYIVDSVTVKNGSIHLYFDKAGQKTYERHSLSH
EPI_ISL_424295 1643 GSTFISDEVARDLSLQFKRPINPTDQSSYIVDSVTVKNGSIHLYFDKAGQKTYERHSLSH
EPI_ISL_426882 1643 GSTFISDEVARDLSLQFKRPINPTDQSSYIVDSVTVKNGSIHLYFDKAGQKTYERHSLSH
```

```
ORF1ab         2521 FVNLDNLRANNTKGSLPINVIVFDGKSKCEESSAKSASVYYSQLMCQPILLLDQALVSDV
EPI_ISL_413594 1703 FVNLDNLRANNTKGSLPINVIVFDGKSKCEESSAKSASVYYSQLMCQPILLLDQALVSDV
EPI_ISL_413595 1703 FVNLDNLRANNTKGSLPINVIVFDGKSKCEESSAKSASVYYSQLMCQPILLLDQALVSDV
EPI_ISL_413596 1703 FVNLDNLRANNTKGSLPINVIVFDGKSKCEESSAKSASVYYSQLMCQPILLLDQALVSDV
EPI_ISL_413597 1703 FVNLDNLRANNTKGSLPINVIVFDGKSKCEESSAKSASVYYSQLMCQPILLLDQALVSDV
EPI_ISL_413598 1703 FVNLDNLRANNTKGSLPINVIVFDGKSKCEESSAKSASVYYSQLMCQPILLLDQALVSDV
EPI_ISL_413599 1703 FVNLDNLRANNTKGSLPINVIVFDGKSKCEESSAKSASVYYSQLMCQPILLLDQALVSDV
EPI_ISL_413600 1703 FVNLDNLRANNTKGSLPINVIVFDGKSKCEESSAKSASVYYSQLMCQPILLLDQALVSDV
EPI_ISL_413601 1703 FVNLDNLRANNTKGSLPINVIVFDGKSKCEESSAKSASVYYSQLMCQPILLLDQALVSDV
EPI_ISL_424217 1703 FVNLDNLRANNTKGSLPINVIVFDGKSKCEESSAKSASVYYSQLMCQPILLLDQALVSDV
EPI_ISL_424218 1703 FVNLDNLRANNTKGSLPINVIVFDGKSKCEESSAKSASVYYSQLMCQPILLLDQALVSDV
EPI_ISL_424220 1703 FVNLDNLRANNTKGSLPINVIVFDGKSKCEESSAKSASVYYSQLMCQPILLLDQALVSDV
EPI_ISL_424232 1703 FVNLDNLRANNTKGSLPINVIVFDGKSKCEESSAKSASVYYSQLMCQPILLLDQALVSDV
EPI_ISL_424266 1703 FVNLDNLRANNTKGSLPINVIVFDGKSKCEESSAKSASVYYSQLMCQPILLLDQALVSDV
EPI_ISL_424272 1703 FVNLDNLRANNTKGSLPINVIVFDGKSKCEESSAKSASVYYSQLMCQPILLLDQALVSDV
EPI_ISL_424283 1703 FVNLDNLRANNTKGSLPINVIVFDGKSKCEESSAKSASVYYSQLMCQPILLLDQALVSDV
EPI_ISL_424288 1703 FVNLDNLRANNTKGSLPINVIVFDGKSKCEESSAKSASVYYSQLMCQPILLLDQALVSDV
EPI_ISL_424292 1703 FVNLDNLRANNTKGSLPINVIVFDGKSKCEESSAKSASVYYSQLMCQPILLLDQALVSDV
EPI_ISL_424295 1703 FVNLDNLRANNTKGSLPINVIVFDGKSKCEESSAKSASVYYSQLMCQPILLLDQALVSDV
EPI_ISL_426882 1703 FVNLDNLRANNTKGSLPINVIVFDGKSKCEESSAKSASVYYSQLMCQPILLLDQALVSDV

ORF1ab         2581 GDSAEVAVKMFDAYVNTFSSTFNVPMEKLKTLVATAEAELAKNVSLDNVLSTFISAARQG
EPI_ISL_413594 1763 GDSAEVAVKMFDAYVNTFSSTFNVPMEKLKTLVATAEAELAKNVSLDNVLSTFISAARQG
EPI_ISL_413595 1763 GDSAEVAVKMFDAYVNTFSSTFNVPMEKLKTLVATAEAELAKNVSLDNVLSTFISAARQG
EPI_ISL_413596 1763 GDSAEVAVKMFDAYVNTFSSTFNVPMEKLKTLVATAEAELAKNVSLDNVLSTFISAARQG
EPI_ISL_413597 1763 GDSAEVAVKMFDAYVNTFSSTFNVPMEKLKTLVATAEAELAKNVSLDNVLSTFISAARQG
EPI_ISL_413598 1763 GDSAEVAVKMFDAYVNTFSSTFNVPMEKLKTLVATAEAELAKNVSLDNVLSTFISAARQG
EPI_ISL_413599 1763 GDSAEVAVKMFDAYVNTFSSTFNVPMEKLKTLVATAEAELAKNVSLDNVLSTFISAARQG
EPI_ISL_413600 1763 GDSAEVAVKMFDAYVNTFSSTFNVPMEKLKTLVATAEAELAKNVSLDNVLSTFISAARQG
EPI_ISL_413601 1763 GDSAEVAVKMFDAYVNTFSSTFNVPMEKLKTLVATAEAELAKNVSLDNVLSTFISAARQG
EPI_ISL_424217 1763 GDSAEVAVKMFDAYVNTFSSTFNVPMEKLKTLVATAEAELAKNVSLDNVLSTFISAARQG
EPI_ISL_424218 1763 GDSAEVAVKMFDAYVNTFSSTFNVPMEKLKTLVATAEAELAKNVSLDNVLSTFISAARQG
EPI_ISL_424220 1763 GDSAEVAVKMFDAYVNTFSSTFNVPMEKLKTLVATAEAELAKNVSLDNVLSTFISAARQG
EPI_ISL_424232 1763 GDSAEVAVKMFDAYVNTFSSTFNVPMEKLKTLVATAEAELAKNVSLDNVLSTFISAARQG
EPI_ISL_424266 1763 GDSAEVAVKMFDAYVNTFSSTFNVPMEKLKTLVATAEAELAKNVSLDNVLSTFISAARQG
EPI_ISL_424272 1763 GDSAEVAVKMFDAYVNTFSSTFNVPMEKLKTLVATAEAELAKNVSLDNVLSTFISAARQG
EPI_ISL_424283 1763 GDSAEVAVKMFDAYVNTFSSTFNVPMEKLKTLVATAEAELAKNVSLDNVLSTFISAARQG
EPI_ISL_424288 1763 GDSAEVAVKMFDAYVNTFSSTFNVPMEKLKTLVATAEAELAKNVSLDNVLSTFISAARQG
EPI_ISL_424292 1763 GDSAEVAVKMFDAYVNTFSSTFNVPMEKLKTLVATAEAELAKNVSLDNVLSTFISAARQG
EPI_ISL_424295 1763 GDSAEVAVKMFDAYVNTFSSTFNVPMEKLKTLVATAEAELAKNVSLDNVLSTFISAARQG
EPI_ISL_426882 1763 GDSAEVAVKMFDAYVNTFSSTFNVPMEKLKTLVATAEAELAKNVSLDNVLSTFISAARQG

ORF1ab         2641 FVDSDVETKDVVECLKLSHQSDIEVTGDSCNNYMLTYNKVENMTPRDLGACIDCSARHIN
EPI_ISL_413594 1823 FVDSDVETKDVVECLKLSHQSDIEVTGDSCNNYMLTYNKVENMTPRDLGACIDXXXXXIN
EPI_ISL_413595 1823 FVDSDVETKDVVECLKLSHQSDIEVTGDSCNNYMLTYNKVENMTPRDLGACIDCSARHIN
EPI_ISL_413596 1823 FVDSDVETKDVVECLKLSHQSDIEVTGDSCNNYMLTYNKVENMTPRDLGACIDCSARHIN
EPI_ISL_413597 1823 FVDSDVETKDVVECLKLSHQSDIEVTGDSCNNYMLTYNKVENMTPRDLGACIDCSARHIN
EPI_ISL_413598 1823 FVDSDVETKDVVECLKLSHQSDIEVTGDSCNNYMLTYNKVENMTPRDLGACIDCSARHIN
EPI_ISL_413599 1823 FVDSDVETKDVVECLKLSHQSDIEVTGDSCNNYMLTYNKVENMTPRDLGACIDCSARHIN
EPI_ISL_413600 1823 FVDSDVETKDVVECLKLSHQSDIEVTGDSCNNYMLTYNKVENMTPRDLGACIDCSARHIN
EPI_ISL_413601 1823 FVDSDVETKDVVECLKLSHQSDIEVTGDSCNNYMLTYNKVENMTPRDLGACIDCSARHIN
EPI_ISL_424217 1823 FVDSDVETKDVVECLKLSHQSDIEVTGDSCNNYMLTYNKVENMTPRDLGACIDCSARHIN
EPI_ISL_424218 1823 FVDSDVETKDVVECLKLSHQSDIEVTGDSCNNYMLTYNKVENMTPRDLGACIDCSARHIN
EPI_ISL_424220 1823 FVDSDVETKDVVECLKLSHQSDIEVTGDSCNNYMLTYNKVENMTPRDLGACIDCSARHIN
EPI_ISL_424232 1823 FVDSDVETKDVVECLKLSHQSDIEVTGDSCNNYMLTYNKVENMTPRDLGACIDCSARHIN
EPI_ISL_424266 1823 FVDSDVETKDVVECLKLSHQSDIEVTGDSCNNYMLTYNKVENMTPRDLGACIDCSARHIN
EPI_ISL_424272 1823 FVDSDVETKDVVECLKLSHQSDIEVTGDSCNNYMLTYNKVENMTPRDLGACIDCSARHIN
EPI_ISL_424283 1823 FVDSDVETKDVVECLKLSHQSDIEVTGDSCNNYMLTYNKVENMTPRDLGACIDCSARHIN
EPI_ISL_424288 1823 FVDSDVETKDVVECLKLSHQSDIEVTGDSCNNYMLTYNKVENMTPRDLGACIDCSARHIN
EPI_ISL_424292 1823 FVDSDVETKDVVECLKLSHQSDIEVTGDSCNNYMLTYNKVENMTPRDLGACIDCSARHIN
EPI_ISL_424295 1823 FVDSDVETKDVVECLKLSHQSDIEVTGDSCNNYMLTYNKVENMTPRDLGACIDCSARHIN
EPI_ISL_426882 1823 FVDSDVETKDVVECLKLSHQSDIEVTGDSCNNYMLTYNKVENMTPRDLGACIDCSARHIN
```

```
ORF1ab         2701 AQVAKSHNIALIWNVKDFMSLSEQLRKQIRSAAKKNNLPFKLTCATTRQVVNVVTTKIAL
EPI_ISL_413594 1883 AXVAKSHNIALIWNVKDFMSLSEQLRKQIRSAAKKNNLPFKLTCATTRQVVNVVTTKIAL
EPI_ISL_413595 1883 AQVAKSHNIALIWNVKDFMSLSEQLRKQIRSAAKKNNLPFKLTCATTRQVVNVVTTKIAL
EPI_ISL_413596 1883 AQVAKSHNIALIWNVKDFMSLSEQLRKQIRSAAKKNNLPFKLTCATTRQVVNVVTTKIAL
EPI_ISL_413597 1883 AQVAKSHNIALIWNVKDFMSLSEQLRKQIRSAAKKNNLPFKLTCATTRQVVNVVTTKIAL
EPI_ISL_413598 1883 AQVAKSHNIALIWNVKDFMSLSEQLRKQIRSAAKKNNLPFKLTCATTRQVVNVVTTKIAL
EPI_ISL_413599 1883 AQVAKSHNIALIWNVKDFMSLSEQLRKQIRSAAKKNNLPFKLTCATTRQVVNVVTTKIAL
EPI_ISL_413600 1883 AQVAKSHNIALIWNVKDFMSLSEQLRKQIRSAAKKNNLPFKLTCATTRQVVNVVTTKIAL
EPI_ISL_413601 1883 AQVAKSHNIALIWNVKDFMSLSEQLRKQIRSAAKKNNLPFKLTCATTRQVVNVVTTKIAL
EPI_ISL_424217 1883 AQVAKSHNIALIWNVKDFMSLSEQLRKQIRSAAKKNNLPFKLTCATTRQVVNVVTTKIAL
EPI_ISL_424218 1883 AQVAKSHNIALIWNVKDFMSLSEQLRKQIRSAAKKNNLPFKLTCATTRQVVNVVTTKIAL
EPI_ISL_424220 1883 AQVAKSHNIALIWNVKDFMSLSEQLRKQIRSAAKKNNLPFKLTCATTRQVVNVVTTKIAL
EPI_ISL_424232 1883 AQVAKSHNIALIWNVKDFMSLSEQLRKQIRSAAKKNNLPFKLTCATTRQVVNVVTTKIAL
EPI_ISL_424266 1883 AQVAKSHNIALIWNVKDFMSLSEQLRKQIRSAAKKNNLPFKLTCATTRQVVNVVTTKIAL
EPI_ISL_424272 1883 AQVAKSHNIALIWNVKDFMSLSEQLRKQIRSAAKKNNLPFKLTCATTRQVVNVVTTKIAL
EPI_ISL_424283 1883 AQVAKSHNIALIWNVKDFMSLSEQLRKQIRSAAKKNNLPFKLTCATTRQVVNVVTTKIAL
EPI_ISL_424288 1883 AQVAKSHNIALIWNVKDFMSLSEQLRKQIRSAAKKNNLPFKLTCATTRQVVNVVTTKIAL
EPI_ISL_424292 1883 AQVAKSHNIALIWNVKDFMSLSEQLRKQIRSAAKKNNLPFKLTCATTRQVVNVVTTKIAL
EPI_ISL_424295 1883 AQVAKSHNIALIWNVKDFMSLSEQLRKQIRSAAKKNNLPFKLTCATTRQVVNVVTTKIAL
EPI_ISL_426882 1883 AQVAKSHNIALIWNVKDFMSLSEQLRKQIRSAAKKNNLPFKLTCATTRQVVNVVTTKIAL
```

```
ORF1ab         2761 KGGKIVNNWLKQLIKVTLVFLFVAAIFYLITPVHVMSKHTDFSSEIIGYKAIDGGVTRDI
EPI_ISL_413594 1943 KGG---------------------------------------------------------
EPI_ISL_413595 1943 KGG---------------------------------------------------------
EPI_ISL_413596 1943 KGG---------------------------------------------------------
EPI_ISL_413597 1943 KGG---------------------------------------------------------
EPI_ISL_413598 1943 KGG---------------------------------------------------------
EPI_ISL_413599 1943 KGG---------------------------------------------------------
EPI_ISL_413600 1943 KGG---------------------------------------------------------
EPI_ISL_413601 1943 KGG---------------------------------------------------------
EPI_ISL_424217 1943 KGG---------------------------------------------------------
EPI_ISL_424218 1943 KGG---------------------------------------------------------
EPI_ISL_424220 1943 KGG---------------------------------------------------------
EPI_ISL_424232 1943 KGG---------------------------------------------------------
EPI_ISL_424266 1943 KGG---------------------------------------------------------
EPI_ISL_424272 1943 KGG---------------------------------------------------------
EPI_ISL_424283 1943 KGG---------------------------------------------------------
EPI_ISL_424288 1943 KGG---------------------------------------------------------
EPI_ISL_424292 1943 KGG---------------------------------------------------------
EPI_ISL_424295 1943 KGG---------------------------------------------------------
EPI_ISL_426882 1943 KGG---------------------------------------------------------
```

```
ORF1ab         2821 ASTDTCFANKHADFDTWFSQRGGSYTNDKACPLIAAVITREVGFVVPGLPGTILRTTNGD
EPI_ISL_413594      ------------------------------------------------------------
EPI_ISL_413595      ------------------------------------------------------------
EPI_ISL_413596      ------------------------------------------------------------
EPI_ISL_413597      ------------------------------------------------------------
EPI_ISL_413598      ------------------------------------------------------------
EPI_ISL_413599      ------------------------------------------------------------
EPI_ISL_413600      ------------------------------------------------------------
EPI_ISL_413601      ------------------------------------------------------------
EPI_ISL_424217      ------------------------------------------------------------
EPI_ISL_424218      ------------------------------------------------------------
EPI_ISL_424220      ------------------------------------------------------------
EPI_ISL_424232      ------------------------------------------------------------
EPI_ISL_424266      ------------------------------------------------------------
EPI_ISL_424272      ------------------------------------------------------------
EPI_ISL_424283      ------------------------------------------------------------
EPI_ISL_424288      ------------------------------------------------------------
EPI_ISL_424292      ------------------------------------------------------------
EPI_ISL_424295      ------------------------------------------------------------
EPI_ISL_426882      ------------------------------------------------------------
```

```
ORF1ab         2881 FLHFLPRVFSAVGNICYTPSKLIEYTDFATSACVLAAECTIFKDASGKPVPYCYDTNVLE
EPI_ISL_413594      ------------------------------------------------------------
EPI_ISL_413595      ------------------------------------------------------------
EPI_ISL_413596      ------------------------------------------------------------
EPI_ISL_413597      ------------------------------------------------------------
EPI_ISL_413598      ------------------------------------------------------------
EPI_ISL_413599      ------------------------------------------------------------
EPI_ISL_413600      ------------------------------------------------------------
EPI_ISL_413601      ------------------------------------------------------------
EPI_ISL_424217      ------------------------------------------------------------
EPI_ISL_424218      ------------------------------------------------------------
EPI_ISL_424220      ------------------------------------------------------------
EPI_ISL_424232      ------------------------------------------------------------
EPI_ISL_424266      ------------------------------------------------------------
EPI_ISL_424272      ------------------------------------------------------------
EPI_ISL_424283      ------------------------------------------------------------
EPI_ISL_424288      ------------------------------------------------------------
EPI_ISL_424292      ------------------------------------------------------------
EPI_ISL_424295      ------------------------------------------------------------
EPI_ISL_426882      ------------------------------------------------------------

ORF1ab         2941 GSVAYESLRPDTRYVLMDGSIIQFPNTYLEGSVRVVTTFDSEYCRHGTCERSEAGVCVST
EPI_ISL_413594      ------------------------------------------------------------
EPI_ISL_413595      ------------------------------------------------------------
EPI_ISL_413596      ------------------------------------------------------------
EPI_ISL_413597      ------------------------------------------------------------
EPI_ISL_413598      ------------------------------------------------------------
EPI_ISL_413599      ------------------------------------------------------------
EPI_ISL_413600      ------------------------------------------------------------
EPI_ISL_413601      ------------------------------------------------------------
EPI_ISL_424217      ------------------------------------------------------------
EPI_ISL_424218      ------------------------------------------------------------
EPI_ISL_424220      ------------------------------------------------------------
EPI_ISL_424232      ------------------------------------------------------------
EPI_ISL_424266      ------------------------------------------------------------
EPI_ISL_424272      ------------------------------------------------------------
EPI_ISL_424283      ------------------------------------------------------------
EPI_ISL_424288      ------------------------------------------------------------
EPI_ISL_424292      ------------------------------------------------------------
EPI_ISL_424295      ------------------------------------------------------------
EPI_ISL_426882      ------------------------------------------------------------

ORF1ab         3001 SGRWVLNNDYYRSLPGVFCGVDAVNLLTNMFTPLIQPIGALDISASIVAGGIVAIVVTCL
EPI_ISL_413594      ------------------------------------------------------------
EPI_ISL_413595      ------------------------------------------------------------
EPI_ISL_413596      ------------------------------------------------------------
EPI_ISL_413597      ------------------------------------------------------------
EPI_ISL_413598      ------------------------------------------------------------
EPI_ISL_413599      ------------------------------------------------------------
EPI_ISL_413600      ------------------------------------------------------------
EPI_ISL_413601      ------------------------------------------------------------
EPI_ISL_424217      ------------------------------------------------------------
EPI_ISL_424218      ------------------------------------------------------------
EPI_ISL_424220      ------------------------------------------------------------
EPI_ISL_424232      ------------------------------------------------------------
EPI_ISL_424266      ------------------------------------------------------------
EPI_ISL_424272      ------------------------------------------------------------
EPI_ISL_424283      ------------------------------------------------------------
EPI_ISL_424288      ------------------------------------------------------------
EPI_ISL_424292      ------------------------------------------------------------
EPI_ISL_424295      ------------------------------------------------------------
EPI_ISL_426882      ------------------------------------------------------------
```

```
ORF1ab          3061 AYYFMRFRRAFGEYSHVVAFNTLLFLMSFTVLCLTPVYSFLPGVYSVIYLYLTFYLTNDV
EPI_ISL_413594       ------------------------------------------------------------
EPI_ISL_413595       ------------------------------------------------------------
EPI_ISL_413596       ------------------------------------------------------------
EPI_ISL_413597       ------------------------------------------------------------
EPI_ISL_413598       ------------------------------------------------------------
EPI_ISL_413599       ------------------------------------------------------------
EPI_ISL_413600       ------------------------------------------------------------
EPI_ISL_413601       ------------------------------------------------------------
EPI_ISL_424217       ------------------------------------------------------------
EPI_ISL_424218       ------------------------------------------------------------
EPI_ISL_424220       ------------------------------------------------------------
EPI_ISL_424232       ------------------------------------------------------------
EPI_ISL_424266       ------------------------------------------------------------
EPI_ISL_424272       ------------------------------------------------------------
EPI_ISL_424283       ------------------------------------------------------------
EPI_ISL_424288       ------------------------------------------------------------
EPI_ISL_424292       ------------------------------------------------------------
EPI_ISL_424295       ------------------------------------------------------------
EPI_ISL_426882       ------------------------------------------------------------

ORF1ab          3121 SFLAHIQWMVMFTPLVPFWITIAYIICISTKHFYWFFSNYLKRRVVFNGVSFSTFEEAAL
EPI_ISL_413594       ------------------------------------------------------------
EPI_ISL_413595       ------------------------------------------------------------
EPI_ISL_413596       ------------------------------------------------------------
EPI_ISL_413597       ------------------------------------------------------------
EPI_ISL_413598       ------------------------------------------------------------
EPI_ISL_413599       ------------------------------------------------------------
EPI_ISL_413600       ------------------------------------------------------------
EPI_ISL_413601       ------------------------------------------------------------
EPI_ISL_424217       ------------------------------------------------------------
EPI_ISL_424218       ------------------------------------------------------------
EPI_ISL_424220       ------------------------------------------------------------
EPI_ISL_424232       ------------------------------------------------------------
EPI_ISL_424266       ------------------------------------------------------------
EPI_ISL_424272       ------------------------------------------------------------
EPI_ISL_424283       ------------------------------------------------------------
EPI_ISL_424288       ------------------------------------------------------------
EPI_ISL_424292       ------------------------------------------------------------
EPI_ISL_424295       ------------------------------------------------------------
EPI_ISL_426882       ------------------------------------------------------------

ORF1ab          3181 CTFLLNKEMYLKLRSDVLLPLTQYNRYLALYNKYKYFSGAMDTTSYREAACCHLAKALND
EPI_ISL_413594       ------------------------------------------------------------
EPI_ISL_413595       ------------------------------------------------------------
EPI_ISL_413596       ------------------------------------------------------------
EPI_ISL_413597       ------------------------------------------------------------
EPI_ISL_413598       ------------------------------------------------------------
EPI_ISL_413599       ------------------------------------------------------------
EPI_ISL_413600       ------------------------------------------------------------
EPI_ISL_413601       ------------------------------------------------------------
EPI_ISL_424217       ------------------------------------------------------------
EPI_ISL_424218       ------------------------------------------------------------
EPI_ISL_424220       ------------------------------------------------------------
EPI_ISL_424232       ------------------------------------------------------------
EPI_ISL_424266       ------------------------------------------------------------
EPI_ISL_424272       ------------------------------------------------------------
EPI_ISL_424283       ------------------------------------------------------------
EPI_ISL_424288       ------------------------------------------------------------
EPI_ISL_424292       ------------------------------------------------------------
EPI_ISL_424295       ------------------------------------------------------------
EPI_ISL_426882       ------------------------------------------------------------
```

```
ORF1ab          3241 FSNSGSDVLYQPPQTSITSAVLQSGFRKMAFPSGKVEGCMVQVTCGTTTLNGLWLDDVVY
EPI_ISL_413594       ------------------------------------------------------------
EPI_ISL_413595       ------------------------------------------------------------
EPI_ISL_413596       ------------------------------------------------------------
EPI_ISL_413597       ------------------------------------------------------------
EPI_ISL_413598       ------------------------------------------------------------
EPI_ISL_413599       ------------------------------------------------------------
EPI_ISL_413600       ------------------------------------------------------------
EPI_ISL_413601       ------------------------------------------------------------
EPI_ISL_424217       ------------------------------------------------------------
EPI_ISL_424218       ------------------------------------------------------------
EPI_ISL_424220       ------------------------------------------------------------
EPI_ISL_424232       ------------------------------------------------------------
EPI_ISL_424266       ------------------------------------------------------------
EPI_ISL_424272       ------------------------------------------------------------
EPI_ISL_424283       ------------------------------------------------------------
EPI_ISL_424288       ------------------------------------------------------------
EPI_ISL_424292       ------------------------------------------------------------
EPI_ISL_424295       ------------------------------------------------------------
EPI_ISL_426882       ------------------------------------------------------------

ORF1ab          3301 CPRHVICTSEDMLNPNYEDLLIRKSNHNFLVQAGNVQLRVIGHSMQNCVLKLKVDTANPK
EPI_ISL_413594       ------------------------------------------------------------
EPI_ISL_413595       ------------------------------------------------------------
EPI_ISL_413596       ------------------------------------------------------------
EPI_ISL_413597       ------------------------------------------------------------
EPI_ISL_413598       ------------------------------------------------------------
EPI_ISL_413599       ------------------------------------------------------------
EPI_ISL_413600       ------------------------------------------------------------
EPI_ISL_413601       ------------------------------------------------------------
EPI_ISL_424217       ------------------------------------------------------------
EPI_ISL_424218       ------------------------------------------------------------
EPI_ISL_424220       ------------------------------------------------------------
EPI_ISL_424232       ------------------------------------------------------------
EPI_ISL_424266       ------------------------------------------------------------
EPI_ISL_424272       ------------------------------------------------------------
EPI_ISL_424283       ------------------------------------------------------------
EPI_ISL_424288       ------------------------------------------------------------
EPI_ISL_424292       ------------------------------------------------------------
EPI_ISL_424295       ------------------------------------------------------------
EPI_ISL_426882       ------------------------------------------------------------

ORF1ab          3361 TPKYKFVRIQPGQTFSVLACYNGSPSGVYQCAMRPNFTIKGSFLNGSCGSVGFNIDYDCV
EPI_ISL_413594       ------------------------------------------------------------
EPI_ISL_413595       ------------------------------------------------------------
EPI_ISL_413596       ------------------------------------------------------------
EPI_ISL_413597       ------------------------------------------------------------
EPI_ISL_413598       ------------------------------------------------------------
EPI_ISL_413599       ------------------------------------------------------------
EPI_ISL_413600       ------------------------------------------------------------
EPI_ISL_413601       ------------------------------------------------------------
EPI_ISL_424217       ------------------------------------------------------------
EPI_ISL_424218       ------------------------------------------------------------
EPI_ISL_424220       ------------------------------------------------------------
EPI_ISL_424232       ------------------------------------------------------------
EPI_ISL_424266       ------------------------------------------------------------
EPI_ISL_424272       ------------------------------------------------------------
EPI_ISL_424283       ------------------------------------------------------------
EPI_ISL_424288       ------------------------------------------------------------
EPI_ISL_424292       ------------------------------------------------------------
EPI_ISL_424295       ------------------------------------------------------------
EPI_ISL_426882       ------------------------------------------------------------
```

```
ORF1ab          3421 SFCYMHHMELPTGVHAGTDLEGNFYGPFVDRQTAQAAGTDTTITVNVLAWLYAAVINGDR
EPI_ISL_413594       ------------------------------------------------------------
EPI_ISL_413595       ------------------------------------------------------------
EPI_ISL_413596       ------------------------------------------------------------
EPI_ISL_413597       ------------------------------------------------------------
EPI_ISL_413598       ------------------------------------------------------------
EPI_ISL_413599       ------------------------------------------------------------
EPI_ISL_413600       ------------------------------------------------------------
EPI_ISL_413601       ------------------------------------------------------------
EPI_ISL_424217       ------------------------------------------------------------
EPI_ISL_424218       ------------------------------------------------------------
EPI_ISL_424220       ------------------------------------------------------------
EPI_ISL_424232       ------------------------------------------------------------
EPI_ISL_424266       ------------------------------------------------------------
EPI_ISL_424272       ------------------------------------------------------------
EPI_ISL_424283       ------------------------------------------------------------
EPI_ISL_424288       ------------------------------------------------------------
EPI_ISL_424292       ------------------------------------------------------------
EPI_ISL_424295       ------------------------------------------------------------
EPI_ISL_426882       ------------------------------------------------------------

ORF1ab          3481 WFLNRFTTTLNDFNLVAMKYNYEPLTQDHVDILGPLSAQTGIAVLDMCASLKELLQNGMN
EPI_ISL_413594       ------------------------------------------------------------
EPI_ISL_413595       ------------------------------------------------------------
EPI_ISL_413596       ------------------------------------------------------------
EPI_ISL_413597       ------------------------------------------------------------
EPI_ISL_413598       ------------------------------------------------------------
EPI_ISL_413599       ------------------------------------------------------------
EPI_ISL_413600       ------------------------------------------------------------
EPI_ISL_413601       ------------------------------------------------------------
EPI_ISL_424217       ------------------------------------------------------------
EPI_ISL_424218       ------------------------------------------------------------
EPI_ISL_424220       ------------------------------------------------------------
EPI_ISL_424232       ------------------------------------------------------------
EPI_ISL_424266       ------------------------------------------------------------
EPI_ISL_424272       ------------------------------------------------------------
EPI_ISL_424283       ------------------------------------------------------------
EPI_ISL_424288       ------------------------------------------------------------
EPI_ISL_424292       ------------------------------------------------------------
EPI_ISL_424295       ------------------------------------------------------------
EPI_ISL_426882       ------------------------------------------------------------

ORF1ab          3541 GRTILGSALLEDEFTPFDVVRQCSGVTFQSAVKRTIKGTHHWLLLTILTSLLVLVQSTQW
EPI_ISL_413594       ------------------------------------------------------------
EPI_ISL_413595       ------------------------------------------------------------
EPI_ISL_413596       ------------------------------------------------------------
EPI_ISL_413597       ------------------------------------------------------------
EPI_ISL_413598       ------------------------------------------------------------
EPI_ISL_413599       ------------------------------------------------------------
EPI_ISL_413600       ------------------------------------------------------------
EPI_ISL_413601       ------------------------------------------------------------
EPI_ISL_424217       ------------------------------------------------------------
EPI_ISL_424218       ------------------------------------------------------------
EPI_ISL_424220       ------------------------------------------------------------
EPI_ISL_424232       ------------------------------------------------------------
EPI_ISL_424266       ------------------------------------------------------------
EPI_ISL_424272       ------------------------------------------------------------
EPI_ISL_424283       ------------------------------------------------------------
EPI_ISL_424288       ------------------------------------------------------------
EPI_ISL_424292       ------------------------------------------------------------
EPI_ISL_424295       ------------------------------------------------------------
EPI_ISL_426882       ------------------------------------------------------------
```

```
ORF1ab          3601 SLFFFLYENAFLPFAMGIIAMSAFAMMFVKHKHAFLCLFLLPSLATVAYFNMVYMPASWV
EPI_ISL_413594       ------------------------------------------------------------
EPI_ISL_413595       ------------------------------------------------------------
EPI_ISL_413596       ------------------------------------------------------------
EPI_ISL_413597       ------------------------------------------------------------
EPI_ISL_413598       ------------------------------------------------------------
EPI_ISL_413599       ------------------------------------------------------------
EPI_ISL_413600       ------------------------------------------------------------
EPI_ISL_413601       ------------------------------------------------------------
EPI_ISL_424217       ------------------------------------------------------------
EPI_ISL_424218       ------------------------------------------------------------
EPI_ISL_424220       ------------------------------------------------------------
EPI_ISL_424232       ------------------------------------------------------------
EPI_ISL_424266       ------------------------------------------------------------
EPI_ISL_424272       ------------------------------------------------------------
EPI_ISL_424283       ------------------------------------------------------------
EPI_ISL_424288       ------------------------------------------------------------
EPI_ISL_424292       ------------------------------------------------------------
EPI_ISL_424295       ------------------------------------------------------------
EPI_ISL_426882       ------------------------------------------------------------

ORF1ab          3661 MRIMTWLDMVDTSLSGFKLKDCVMYASAVVLLILMTARTVYDDGARRVWTLMNVLTLVYK
EPI_ISL_413594       ------------------------------------------------------------
EPI_ISL_413595       ------------------------------------------------------------
EPI_ISL_413596       ------------------------------------------------------------
EPI_ISL_413597       ------------------------------------------------------------
EPI_ISL_413598       ------------------------------------------------------------
EPI_ISL_413599       ------------------------------------------------------------
EPI_ISL_413600       ------------------------------------------------------------
EPI_ISL_413601       ------------------------------------------------------------
EPI_ISL_424217       ------------------------------------------------------------
EPI_ISL_424218       ------------------------------------------------------------
EPI_ISL_424220       ------------------------------------------------------------
EPI_ISL_424232       ------------------------------------------------------------
EPI_ISL_424266       ------------------------------------------------------------
EPI_ISL_424272       ------------------------------------------------------------
EPI_ISL_424283       ------------------------------------------------------------
EPI_ISL_424288       ------------------------------------------------------------
EPI_ISL_424292       ------------------------------------------------------------
EPI_ISL_424295       ------------------------------------------------------------
EPI_ISL_426882       ------------------------------------------------------------

ORF1ab          3721 VYYGNALDQAISMWALIISVTSNYSGVVTTVMFLARGIVFMCVEYCPIFFITGNTLQCIM
EPI_ISL_413594       ------------------------------------------------------------
EPI_ISL_413595       ------------------------------------------------------------
EPI_ISL_413596       ------------------------------------------------------------
EPI_ISL_413597       ------------------------------------------------------------
EPI_ISL_413598       ------------------------------------------------------------
EPI_ISL_413599       ------------------------------------------------------------
EPI_ISL_413600       ------------------------------------------------------------
EPI_ISL_413601       ------------------------------------------------------------
EPI_ISL_424217       ------------------------------------------------------------
EPI_ISL_424218       ------------------------------------------------------------
EPI_ISL_424220       ------------------------------------------------------------
EPI_ISL_424232       ------------------------------------------------------------
EPI_ISL_424266       ------------------------------------------------------------
EPI_ISL_424272       ------------------------------------------------------------
EPI_ISL_424283       ------------------------------------------------------------
EPI_ISL_424288       ------------------------------------------------------------
EPI_ISL_424292       ------------------------------------------------------------
EPI_ISL_424295       ------------------------------------------------------------
EPI_ISL_426882       ------------------------------------------------------------
```

```
ORF1ab         3781 LVYCFLGYFCTCYFGLFCLLNRYFRLTLGVYDYLVSTQEFRYMNSQGLLPPKNSIDAFKL
EPI_ISL_413594      ------------------------------------------------------------
EPI_ISL_413595      ------------------------------------------------------------
EPI_ISL_413596      ------------------------------------------------------------
EPI_ISL_413597      ------------------------------------------------------------
EPI_ISL_413598      ------------------------------------------------------------
EPI_ISL_413599      ------------------------------------------------------------
EPI_ISL_413600      ------------------------------------------------------------
EPI_ISL_413601      ------------------------------------------------------------
EPI_ISL_424217      ------------------------------------------------------------
EPI_ISL_424218      ------------------------------------------------------------
EPI_ISL_424220      ------------------------------------------------------------
EPI_ISL_424232      ------------------------------------------------------------
EPI_ISL_424266      ------------------------------------------------------------
EPI_ISL_424272      ------------------------------------------------------------
EPI_ISL_424283      ------------------------------------------------------------
EPI_ISL_424288      ------------------------------------------------------------
EPI_ISL_424292      ------------------------------------------------------------
EPI_ISL_424295      ------------------------------------------------------------
EPI_ISL_426882      ------------------------------------------------------------

ORF1ab         3841 NIKLLGVGGKPCIKVATVQSKMSDVKCTSVVLLSVLQQLRVESSSKLWAQCVQLHNDILL
EPI_ISL_413594      ------------------------------------------------------------
EPI_ISL_413595      ------------------------------------------------------------
EPI_ISL_413596      ------------------------------------------------------------
EPI_ISL_413597      ------------------------------------------------------------
EPI_ISL_413598      ------------------------------------------------------------
EPI_ISL_413599      ------------------------------------------------------------
EPI_ISL_413600      ------------------------------------------------------------
EPI_ISL_413601      ------------------------------------------------------------
EPI_ISL_424217      ------------------------------------------------------------
EPI_ISL_424218      ------------------------------------------------------------
EPI_ISL_424220      ------------------------------------------------------------
EPI_ISL_424232      ------------------------------------------------------------
EPI_ISL_424266      ------------------------------------------------------------
EPI_ISL_424272      ------------------------------------------------------------
EPI_ISL_424283      ------------------------------------------------------------
EPI_ISL_424288      ------------------------------------------------------------
EPI_ISL_424292      ------------------------------------------------------------
EPI_ISL_424295      ------------------------------------------------------------
EPI_ISL_426882      ------------------------------------------------------------

ORF1ab         3901 AKDTTEAFEKMVSLLSVLLSMQGAVDINKLCEEMLDNRATLQAIASEFSSLPSYAAFATA
EPI_ISL_413594      ------------------------------------------------------------
EPI_ISL_413595      ------------------------------------------------------------
EPI_ISL_413596      ------------------------------------------------------------
EPI_ISL_413597      ------------------------------------------------------------
EPI_ISL_413598      ------------------------------------------------------------
EPI_ISL_413599      ------------------------------------------------------------
EPI_ISL_413600      ------------------------------------------------------------
EPI_ISL_413601      ------------------------------------------------------------
EPI_ISL_424217      ------------------------------------------------------------
EPI_ISL_424218      ------------------------------------------------------------
EPI_ISL_424220      ------------------------------------------------------------
EPI_ISL_424232      ------------------------------------------------------------
EPI_ISL_424266      ------------------------------------------------------------
EPI_ISL_424272      ------------------------------------------------------------
EPI_ISL_424283      ------------------------------------------------------------
EPI_ISL_424288      ------------------------------------------------------------
EPI_ISL_424292      ------------------------------------------------------------
EPI_ISL_424295      ------------------------------------------------------------
EPI_ISL_426882      ------------------------------------------------------------
```

```
ORF1ab          3961 QEAYEQAVANGDSEVVLKKLKKSLNVAKSEFDRDAAMQRKLEKMADQAMTQMYKQARSED
EPI_ISL_413594       ------------------------------------------------------------
EPI_ISL_413595       ------------------------------------------------------------
EPI_ISL_413596       ------------------------------------------------------------
EPI_ISL_413597       ------------------------------------------------------------
EPI_ISL_413598       ------------------------------------------------------------
EPI_ISL_413599       ------------------------------------------------------------
EPI_ISL_413600       ------------------------------------------------------------
EPI_ISL_413601       ------------------------------------------------------------
EPI_ISL_424217       ------------------------------------------------------------
EPI_ISL_424218       ------------------------------------------------------------
EPI_ISL_424220       ------------------------------------------------------------
EPI_ISL_424232       ------------------------------------------------------------
EPI_ISL_424266       ------------------------------------------------------------
EPI_ISL_424272       ------------------------------------------------------------
EPI_ISL_424283       ------------------------------------------------------------
EPI_ISL_424288       ------------------------------------------------------------
EPI_ISL_424292       ------------------------------------------------------------
EPI_ISL_424295       ------------------------------------------------------------
EPI_ISL_426882       ------------------------------------------------------------

ORF1ab          4021 KRAKVTSAMQTMLFTMLRKLDNDALNNIINNARDGCVPLNIIPLTTAAKLMVVIPDYNTY
EPI_ISL_413594       ------------------------------------------------------------
EPI_ISL_413595       ------------------------------------------------------------
EPI_ISL_413596       ------------------------------------------------------------
EPI_ISL_413597       ------------------------------------------------------------
EPI_ISL_413598       ------------------------------------------------------------
EPI_ISL_413599       ------------------------------------------------------------
EPI_ISL_413600       ------------------------------------------------------------
EPI_ISL_413601       ------------------------------------------------------------
EPI_ISL_424217       ------------------------------------------------------------
EPI_ISL_424218       ------------------------------------------------------------
EPI_ISL_424220       ------------------------------------------------------------
EPI_ISL_424232       ------------------------------------------------------------
EPI_ISL_424266       ------------------------------------------------------------
EPI_ISL_424272       ------------------------------------------------------------
EPI_ISL_424283       ------------------------------------------------------------
EPI_ISL_424288       ------------------------------------------------------------
EPI_ISL_424292       ------------------------------------------------------------
EPI_ISL_424295       ------------------------------------------------------------
EPI_ISL_426882       ------------------------------------------------------------

ORF1ab          4081 KNTCDGTTFTYASALWEIQQVVDADSKIVQLSEISMDNSPNLAWPLIVTALRANSAVKLQ
EPI_ISL_413594       ------------------------------------------------------------
EPI_ISL_413595       ------------------------------------------------------------
EPI_ISL_413596       ------------------------------------------------------------
EPI_ISL_413597       ------------------------------------------------------------
EPI_ISL_413598       ------------------------------------------------------------
EPI_ISL_413599       ------------------------------------------------------------
EPI_ISL_413600       ------------------------------------------------------------
EPI_ISL_413601       ------------------------------------------------------------
EPI_ISL_424217       ------------------------------------------------------------
EPI_ISL_424218       ------------------------------------------------------------
EPI_ISL_424220       ------------------------------------------------------------
EPI_ISL_424232       ------------------------------------------------------------
EPI_ISL_424266       ------------------------------------------------------------
EPI_ISL_424272       ------------------------------------------------------------
EPI_ISL_424283       ------------------------------------------------------------
EPI_ISL_424288       ------------------------------------------------------------
EPI_ISL_424292       ------------------------------------------------------------
EPI_ISL_424295       ------------------------------------------------------------
EPI_ISL_426882       ------------------------------------------------------------
```

```
ORF1ab          4141 NNELSPVALRQMSCAAGTTQTACTDDNALAYYNTTKGGRFVLALLSDLQDLKWARFPKSD
EPI_ISL_413594       ------------------------------------------------------------
EPI_ISL_413595       ------------------------------------------------------------
EPI_ISL_413596       ------------------------------------------------------------
EPI_ISL_413597       ------------------------------------------------------------
EPI_ISL_413598       ------------------------------------------------------------
EPI_ISL_413599       ------------------------------------------------------------
EPI_ISL_413600       ------------------------------------------------------------
EPI_ISL_413601       ------------------------------------------------------------
EPI_ISL_424217       ------------------------------------------------------------
EPI_ISL_424218       ------------------------------------------------------------
EPI_ISL_424220       ------------------------------------------------------------
EPI_ISL_424232       ------------------------------------------------------------
EPI_ISL_424266       ------------------------------------------------------------
EPI_ISL_424272       ------------------------------------------------------------
EPI_ISL_424283       ------------------------------------------------------------
EPI_ISL_424288       ------------------------------------------------------------
EPI_ISL_424292       ------------------------------------------------------------
EPI_ISL_424295       ------------------------------------------------------------
EPI_ISL_426882       ------------------------------------------------------------

ORF1ab          4201 GTGTIYTELEPPCRFVTDTPKGPKVKYLYFIKGLNNLNRGMVLGSLAATVRLQAGNATEV
EPI_ISL_413594       ------------------------------------------------------------
EPI_ISL_413595       ------------------------------------------------------------
EPI_ISL_413596       ------------------------------------------------------------
EPI_ISL_413597       ------------------------------------------------------------
EPI_ISL_413598       ------------------------------------------------------------
EPI_ISL_413599       ------------------------------------------------------------
EPI_ISL_413600       ------------------------------------------------------------
EPI_ISL_413601       ------------------------------------------------------------
EPI_ISL_424217       ------------------------------------------------------------
EPI_ISL_424218       ------------------------------------------------------------
EPI_ISL_424220       ------------------------------------------------------------
EPI_ISL_424232       ------------------------------------------------------------
EPI_ISL_424266       ------------------------------------------------------------
EPI_ISL_424272       ------------------------------------------------------------
EPI_ISL_424283       ------------------------------------------------------------
EPI_ISL_424288       ------------------------------------------------------------
EPI_ISL_424292       ------------------------------------------------------------
EPI_ISL_424295       ------------------------------------------------------------
EPI_ISL_426882       ------------------------------------------------------------

ORF1ab          4261 PANSTVLSFCAFAVDAAKAYKDYLASGGQPITNCVKMLCTHTGTGQAITVTPEANMDQES
EPI_ISL_413594       ------------------------------------------------------------
EPI_ISL_413595       ------------------------------------------------------------
EPI_ISL_413596       ------------------------------------------------------------
EPI_ISL_413597       ------------------------------------------------------------
EPI_ISL_413598       ------------------------------------------------------------
EPI_ISL_413599       ------------------------------------------------------------
EPI_ISL_413600       ------------------------------------------------------------
EPI_ISL_413601       ------------------------------------------------------------
EPI_ISL_424217       ------------------------------------------------------------
EPI_ISL_424218       ------------------------------------------------------------
EPI_ISL_424220       ------------------------------------------------------------
EPI_ISL_424232       ------------------------------------------------------------
EPI_ISL_424266       ------------------------------------------------------------
EPI_ISL_424272       ------------------------------------------------------------
EPI_ISL_424283       ------------------------------------------------------------
EPI_ISL_424288       ------------------------------------------------------------
EPI_ISL_424292       ------------------------------------------------------------
EPI_ISL_424295       ------------------------------------------------------------
EPI_ISL_426882       ------------------------------------------------------------
```

```
ORF1ab         4321 FGGASCCLYCRCHIDHPNPKGFCDLKGKYVQIPTTCANDPVGFTLKNTVCTVCGMWKGYG
EPI_ISL_413594      ------------------------------------------------------------
EPI_ISL_413595      ------------------------------------------------------------
EPI_ISL_413596      ------------------------------------------------------------
EPI_ISL_413597      ------------------------------------------------------------
EPI_ISL_413598      ------------------------------------------------------------
EPI_ISL_413599      ------------------------------------------------------------
EPI_ISL_413600      ------------------------------------------------------------
EPI_ISL_413601      ------------------------------------------------------------
EPI_ISL_424217      ------------------------------------------------------------
EPI_ISL_424218      ------------------------------------------------------------
EPI_ISL_424220      ------------------------------------------------------------
EPI_ISL_424232      ------------------------------------------------------------
EPI_ISL_424266      ------------------------------------------------------------
EPI_ISL_424272      ------------------------------------------------------------
EPI_ISL_424283      ------------------------------------------------------------
EPI_ISL_424288      ------------------------------------------------------------
EPI_ISL_424292      ------------------------------------------------------------
EPI_ISL_424295      ------------------------------------------------------------
EPI_ISL_426882      ------------------------------------------------------------

ORF1ab         4381 CSCDQLREPMLQSADAQSFLNRVCGVSAARLTPCGTGTSTDVVYRAFDIYNDKVAGFAKF
EPI_ISL_413594      ------------------------------------------------------------
EPI_ISL_413595      ------------------------------------------------------------
EPI_ISL_413596      ------------------------------------------------------------
EPI_ISL_413597      ------------------------------------------------------------
EPI_ISL_413598      ------------------------------------------------------------
EPI_ISL_413599      ------------------------------------------------------------
EPI_ISL_413600      ------------------------------------------------------------
EPI_ISL_413601      ------------------------------------------------------------
EPI_ISL_424217      ------------------------------------------------------------
EPI_ISL_424218      ------------------------------------------------------------
EPI_ISL_424220      ------------------------------------------------------------
EPI_ISL_424232      ------------------------------------------------------------
EPI_ISL_424266      ------------------------------------------------------------
EPI_ISL_424272      ------------------------------------------------------------
EPI_ISL_424283      ------------------------------------------------------------
EPI_ISL_424288      ------------------------------------------------------------
EPI_ISL_424292      ------------------------------------------------------------
EPI_ISL_424295      ------------------------------------------------------------
EPI_ISL_426882      ------------------------------------------------------------

ORF1ab         4441 LKTNCCRFQEKDEDDNLIDSYFVVKRHTFSNYQHEETIYNLLKDCPAVAKHDFFKFRIDG
EPI_ISL_413594      ------------------------------------------------------------
EPI_ISL_413595      ------------------------------------------------------------
EPI_ISL_413596      ------------------------------------------------------------
EPI_ISL_413597      ------------------------------------------------------------
EPI_ISL_413598      ------------------------------------------------------------
EPI_ISL_413599      ------------------------------------------------------------
EPI_ISL_413600      ------------------------------------------------------------
EPI_ISL_413601      ------------------------------------------------------------
EPI_ISL_424217      ------------------------------------------------------------
EPI_ISL_424218      ------------------------------------------------------------
EPI_ISL_424220      ------------------------------------------------------------
EPI_ISL_424232      ------------------------------------------------------------
EPI_ISL_424266      ------------------------------------------------------------
EPI_ISL_424272      ------------------------------------------------------------
EPI_ISL_424283      ------------------------------------------------------------
EPI_ISL_424288      ------------------------------------------------------------
EPI_ISL_424292      ------------------------------------------------------------
EPI_ISL_424295      ------------------------------------------------------------
EPI_ISL_426882      ------------------------------------------------------------
```

```
ORF1ab         4501 DMVPHISRQRLTKYTMADLVYALRHFDEGNCDTLKEILVTYNCCDDDYFNKKDWYDFVEN
EPI_ISL_413594      ------------------------------------------------------------
EPI_ISL_413595      ------------------------------------------------------------
EPI_ISL_413596      ------------------------------------------------------------
EPI_ISL_413597      ------------------------------------------------------------
EPI_ISL_413598      ------------------------------------------------------------
EPI_ISL_413599      ------------------------------------------------------------
EPI_ISL_413600      ------------------------------------------------------------
EPI_ISL_413601      ------------------------------------------------------------
EPI_ISL_424217      ------------------------------------------------------------
EPI_ISL_424218      ------------------------------------------------------------
EPI_ISL_424220      ------------------------------------------------------------
EPI_ISL_424232      ------------------------------------------------------------
EPI_ISL_424266      ------------------------------------------------------------
EPI_ISL_424272      ------------------------------------------------------------
EPI_ISL_424283      ------------------------------------------------------------
EPI_ISL_424288      ------------------------------------------------------------
EPI_ISL_424292      ------------------------------------------------------------
EPI_ISL_424295      ------------------------------------------------------------
EPI_ISL_426882      ------------------------------------------------------------

ORF1ab         4561 PDILRVYANLGERVRQALLKTVQFCDAMRNAGIVGVLTLDNQDLNGNWYDFGDFIQTTPG
EPI_ISL_413594      ------------------------------------------------------------
EPI_ISL_413595      ------------------------------------------------------------
EPI_ISL_413596      ------------------------------------------------------------
EPI_ISL_413597      ------------------------------------------------------------
EPI_ISL_413598      ------------------------------------------------------------
EPI_ISL_413599      ------------------------------------------------------------
EPI_ISL_413600      ------------------------------------------------------------
EPI_ISL_413601      ------------------------------------------------------------
EPI_ISL_424217      ------------------------------------------------------------
EPI_ISL_424218      ------------------------------------------------------------
EPI_ISL_424220      ------------------------------------------------------------
EPI_ISL_424232      ------------------------------------------------------------
EPI_ISL_424266      ------------------------------------------------------------
EPI_ISL_424272      ------------------------------------------------------------
EPI_ISL_424283      ------------------------------------------------------------
EPI_ISL_424288      ------------------------------------------------------------
EPI_ISL_424292      ------------------------------------------------------------
EPI_ISL_424295      ------------------------------------------------------------
EPI_ISL_426882      ------------------------------------------------------------

ORF1ab         4621 SGVPVVDSYYSLLMPILTLTRALTAESHVDTDLTKPYIKWDLLKYDFTEERLKLFDRYFK
EPI_ISL_413594      ------------------------------------------------------------
EPI_ISL_413595      ------------------------------------------------------------
EPI_ISL_413596      ------------------------------------------------------------
EPI_ISL_413597      ------------------------------------------------------------
EPI_ISL_413598      ------------------------------------------------------------
EPI_ISL_413599      ------------------------------------------------------------
EPI_ISL_413600      ------------------------------------------------------------
EPI_ISL_413601      ------------------------------------------------------------
EPI_ISL_424217      ------------------------------------------------------------
EPI_ISL_424218      ------------------------------------------------------------
EPI_ISL_424220      ------------------------------------------------------------
EPI_ISL_424232      ------------------------------------------------------------
EPI_ISL_424266      ------------------------------------------------------------
EPI_ISL_424272      ------------------------------------------------------------
EPI_ISL_424283      ------------------------------------------------------------
EPI_ISL_424288      ------------------------------------------------------------
EPI_ISL_424292      ------------------------------------------------------------
EPI_ISL_424295      ------------------------------------------------------------
EPI_ISL_426882      ------------------------------------------------------------
```

```
ORF1ab          4681 YWDQTYHPNCVNCLDDRCILHCANFNVLFSTVFPPTSFGPLVRKIFVDGVPFVVSTGYHF
EPI_ISL_413594       ------------------------------------------------------------
EPI_ISL_413595       ------------------------------------------------------------
EPI_ISL_413596       ------------------------------------------------------------
EPI_ISL_413597       ------------------------------------------------------------
EPI_ISL_413598       ------------------------------------------------------------
EPI_ISL_413599       ------------------------------------------------------------
EPI_ISL_413600       ------------------------------------------------------------
EPI_ISL_413601       ------------------------------------------------------------
EPI_ISL_424217       ------------------------------------------------------------
EPI_ISL_424218       ------------------------------------------------------------
EPI_ISL_424220       ------------------------------------------------------------
EPI_ISL_424232       ------------------------------------------------------------
EPI_ISL_424266       ------------------------------------------------------------
EPI_ISL_424272       ------------------------------------------------------------
EPI_ISL_424283       ------------------------------------------------------------
EPI_ISL_424288       ------------------------------------------------------------
EPI_ISL_424292       ------------------------------------------------------------
EPI_ISL_424295       ------------------------------------------------------------
EPI_ISL_426882       ------------------------------------------------------------

ORF1ab          4741 RELGVVHNQDVNLHSSRLSFKELLVYAADPAMHAASGNLLLDKRTTCFSVAALTNNVAFQ
EPI_ISL_413594       ------------------------------------------------------------
EPI_ISL_413595       ------------------------------------------------------------
EPI_ISL_413596       ------------------------------------------------------------
EPI_ISL_413597       ------------------------------------------------------------
EPI_ISL_413598       ------------------------------------------------------------
EPI_ISL_413599       ------------------------------------------------------------
EPI_ISL_413600       ------------------------------------------------------------
EPI_ISL_413601       ------------------------------------------------------------
EPI_ISL_424217       ------------------------------------------------------------
EPI_ISL_424218       ------------------------------------------------------------
EPI_ISL_424220       ------------------------------------------------------------
EPI_ISL_424232       ------------------------------------------------------------
EPI_ISL_424266       ------------------------------------------------------------
EPI_ISL_424272       ------------------------------------------------------------
EPI_ISL_424283       ------------------------------------------------------------
EPI_ISL_424288       ------------------------------------------------------------
EPI_ISL_424292       ------------------------------------------------------------
EPI_ISL_424295       ------------------------------------------------------------
EPI_ISL_426882       ------------------------------------------------------------

ORF1ab          4801 TVKPGNFNKDFYDFAVSKGFFKEGSSVELKHFFFAQDGNAAISDYDYYRYNLPTMCDIRQ
EPI_ISL_413594       ------------------------------------------------------------
EPI_ISL_413595       ------------------------------------------------------------
EPI_ISL_413596       ------------------------------------------------------------
EPI_ISL_413597       ------------------------------------------------------------
EPI_ISL_413598       ------------------------------------------------------------
EPI_ISL_413599       ------------------------------------------------------------
EPI_ISL_413600       ------------------------------------------------------------
EPI_ISL_413601       ------------------------------------------------------------
EPI_ISL_424217       ------------------------------------------------------------
EPI_ISL_424218       ------------------------------------------------------------
EPI_ISL_424220       ------------------------------------------------------------
EPI_ISL_424232       ------------------------------------------------------------
EPI_ISL_424266       ------------------------------------------------------------
EPI_ISL_424272       ------------------------------------------------------------
EPI_ISL_424283       ------------------------------------------------------------
EPI_ISL_424288       ------------------------------------------------------------
EPI_ISL_424292       ------------------------------------------------------------
EPI_ISL_424295       ------------------------------------------------------------
EPI_ISL_426882       ------------------------------------------------------------
```

```
ORF1ab          4861 LLFVVEVVDKYFDCYDGGCINANQVIVNNLDKSAGFPFNKWGKARLYYDSMSYEDQDALF
EPI_ISL_413594       ------------------------------------------------------------
EPI_ISL_413595       ------------------------------------------------------------
EPI_ISL_413596       ------------------------------------------------------------
EPI_ISL_413597       ------------------------------------------------------------
EPI_ISL_413598       ------------------------------------------------------------
EPI_ISL_413599       ------------------------------------------------------------
EPI_ISL_413600       ------------------------------------------------------------
EPI_ISL_413601       ------------------------------------------------------------
EPI_ISL_424217       ------------------------------------------------------------
EPI_ISL_424218       ------------------------------------------------------------
EPI_ISL_424220       ------------------------------------------------------------
EPI_ISL_424232       ------------------------------------------------------------
EPI_ISL_424266       ------------------------------------------------------------
EPI_ISL_424272       ------------------------------------------------------------
EPI_ISL_424283       ------------------------------------------------------------
EPI_ISL_424288       ------------------------------------------------------------
EPI_ISL_424292       ------------------------------------------------------------
EPI_ISL_424295       ------------------------------------------------------------
EPI_ISL_426882       ------------------------------------------------------------

ORF1ab          4921 AYTKRNVIPTITQMNLKYAISAKNRARTVAGVSICSTMTNRQFHQKLLKSIAATRGATVV
EPI_ISL_413594       ------------------------------------------------------------
EPI_ISL_413595       ------------------------------------------------------------
EPI_ISL_413596       ------------------------------------------------------------
EPI_ISL_413597       ------------------------------------------------------------
EPI_ISL_413598       ------------------------------------------------------------
EPI_ISL_413599       ------------------------------------------------------------
EPI_ISL_413600       ------------------------------------------------------------
EPI_ISL_413601       ------------------------------------------------------------
EPI_ISL_424217       ------------------------------------------------------------
EPI_ISL_424218       ------------------------------------------------------------
EPI_ISL_424220       ------------------------------------------------------------
EPI_ISL_424232       ------------------------------------------------------------
EPI_ISL_424266       ------------------------------------------------------------
EPI_ISL_424272       ------------------------------------------------------------
EPI_ISL_424283       ------------------------------------------------------------
EPI_ISL_424288       ------------------------------------------------------------
EPI_ISL_424292       ------------------------------------------------------------
EPI_ISL_424295       ------------------------------------------------------------
EPI_ISL_426882       ------------------------------------------------------------

ORF1ab          4981 IGTSKFYGGWHNMLKTVYSDVENPHLMGWDYPKCDRAMPNMLRIMASLVLARKHTTCCSL
EPI_ISL_413594       ------------------------------------------------------------
EPI_ISL_413595       ------------------------------------------------------------
EPI_ISL_413596       ------------------------------------------------------------
EPI_ISL_413597       ------------------------------------------------------------
EPI_ISL_413598       ------------------------------------------------------------
EPI_ISL_413599       ------------------------------------------------------------
EPI_ISL_413600       ------------------------------------------------------------
EPI_ISL_413601       ------------------------------------------------------------
EPI_ISL_424217       ------------------------------------------------------------
EPI_ISL_424218       ------------------------------------------------------------
EPI_ISL_424220       ------------------------------------------------------------
EPI_ISL_424232       ------------------------------------------------------------
EPI_ISL_424266       ------------------------------------------------------------
EPI_ISL_424272       ------------------------------------------------------------
EPI_ISL_424283       ------------------------------------------------------------
EPI_ISL_424288       ------------------------------------------------------------
EPI_ISL_424292       ------------------------------------------------------------
EPI_ISL_424295       ------------------------------------------------------------
EPI_ISL_426882       ------------------------------------------------------------
```

```
ORF1ab          5041 SHRFYRLANECAQVLSEMVMCGGSLYVKPGGTSSGDATTAYANSVFNICQAVTANVNALL
EPI_ISL_413594       ------------------------------------------------------------
EPI_ISL_413595       ------------------------------------------------------------
EPI_ISL_413596       ------------------------------------------------------------
EPI_ISL_413597       ------------------------------------------------------------
EPI_ISL_413598       ------------------------------------------------------------
EPI_ISL_413599       ------------------------------------------------------------
EPI_ISL_413600       ------------------------------------------------------------
EPI_ISL_413601       ------------------------------------------------------------
EPI_ISL_424217       ------------------------------------------------------------
EPI_ISL_424218       ------------------------------------------------------------
EPI_ISL_424220       ------------------------------------------------------------
EPI_ISL_424232       ------------------------------------------------------------
EPI_ISL_424266       ------------------------------------------------------------
EPI_ISL_424272       ------------------------------------------------------------
EPI_ISL_424283       ------------------------------------------------------------
EPI_ISL_424288       ------------------------------------------------------------
EPI_ISL_424292       ------------------------------------------------------------
EPI_ISL_424295       ------------------------------------------------------------
EPI_ISL_426882       ------------------------------------------------------------

ORF1ab          5101 STDGNKIADKYVRNLQHRLYECLYRNRDVDTDFVNEFYAYLRKHFSMMILSDDAVVCFNS
EPI_ISL_413594       ------------------------------------------------------------
EPI_ISL_413595       ------------------------------------------------------------
EPI_ISL_413596       ------------------------------------------------------------
EPI_ISL_413597       ------------------------------------------------------------
EPI_ISL_413598       ------------------------------------------------------------
EPI_ISL_413599       ------------------------------------------------------------
EPI_ISL_413600       ------------------------------------------------------------
EPI_ISL_413601       ------------------------------------------------------------
EPI_ISL_424217       ------------------------------------------------------------
EPI_ISL_424218       ------------------------------------------------------------
EPI_ISL_424220       ------------------------------------------------------------
EPI_ISL_424232       ------------------------------------------------------------
EPI_ISL_424266       ------------------------------------------------------------
EPI_ISL_424272       ------------------------------------------------------------
EPI_ISL_424283       ------------------------------------------------------------
EPI_ISL_424288       ------------------------------------------------------------
EPI_ISL_424292       ------------------------------------------------------------
EPI_ISL_424295       ------------------------------------------------------------
EPI_ISL_426882       ------------------------------------------------------------

ORF1ab          5161 TYASQGLVASIKNFKSVLYYQNNVFMSEAKCWTETDLTKGPHEFCSQHTMLVKQGDDYVY
EPI_ISL_413594       ------------------------------------------------------------
EPI_ISL_413595       ------------------------------------------------------------
EPI_ISL_413596       ------------------------------------------------------------
EPI_ISL_413597       ------------------------------------------------------------
EPI_ISL_413598       ------------------------------------------------------------
EPI_ISL_413599       ------------------------------------------------------------
EPI_ISL_413600       ------------------------------------------------------------
EPI_ISL_413601       ------------------------------------------------------------
EPI_ISL_424217       ------------------------------------------------------------
EPI_ISL_424218       ------------------------------------------------------------
EPI_ISL_424220       ------------------------------------------------------------
EPI_ISL_424232       ------------------------------------------------------------
EPI_ISL_424266       ------------------------------------------------------------
EPI_ISL_424272       ------------------------------------------------------------
EPI_ISL_424283       ------------------------------------------------------------
EPI_ISL_424288       ------------------------------------------------------------
EPI_ISL_424292       ------------------------------------------------------------
EPI_ISL_424295       ------------------------------------------------------------
EPI_ISL_426882       ------------------------------------------------------------
```

```
ORF1ab         5221 LPYPDPSRILGAGCFVDDIVKTDGTLMIERFVSLAIDAYPLTKHPNQEYADVFHLYLQYI
EPI_ISL_413594      ------------------------------------------------------------
EPI_ISL_413595      ------------------------------------------------------------
EPI_ISL_413596      ------------------------------------------------------------
EPI_ISL_413597      ------------------------------------------------------------
EPI_ISL_413598      ------------------------------------------------------------
EPI_ISL_413599      ------------------------------------------------------------
EPI_ISL_413600      ------------------------------------------------------------
EPI_ISL_413601      ------------------------------------------------------------
EPI_ISL_424217      ------------------------------------------------------------
EPI_ISL_424218      ------------------------------------------------------------
EPI_ISL_424220      ------------------------------------------------------------
EPI_ISL_424232      ------------------------------------------------------------
EPI_ISL_424266      ------------------------------------------------------------
EPI_ISL_424272      ------------------------------------------------------------
EPI_ISL_424283      ------------------------------------------------------------
EPI_ISL_424288      ------------------------------------------------------------
EPI_ISL_424292      ------------------------------------------------------------
EPI_ISL_424295      ------------------------------------------------------------
EPI_ISL_426882      ------------------------------------------------------------

ORF1ab         5281 RKLHDELTGHMLDMYSVMLTNDNTSRYWEPEFYEAMYTPHTVLQAVGACVLCNSQTSLRC
EPI_ISL_413594      ------------------------------------------------------------
EPI_ISL_413595      ------------------------------------------------------------
EPI_ISL_413596      ------------------------------------------------------------
EPI_ISL_413597      ------------------------------------------------------------
EPI_ISL_413598      ------------------------------------------------------------
EPI_ISL_413599      ------------------------------------------------------------
EPI_ISL_413600      ------------------------------------------------------------
EPI_ISL_413601      ------------------------------------------------------------
EPI_ISL_424217      ------------------------------------------------------------
EPI_ISL_424218      ------------------------------------------------------------
EPI_ISL_424220      ------------------------------------------------------------
EPI_ISL_424232      ------------------------------------------------------------
EPI_ISL_424266      ------------------------------------------------------------
EPI_ISL_424272      ------------------------------------------------------------
EPI_ISL_424283      ------------------------------------------------------------
EPI_ISL_424288      ------------------------------------------------------------
EPI_ISL_424292      ------------------------------------------------------------
EPI_ISL_424295      ------------------------------------------------------------
EPI_ISL_426882      ------------------------------------------------------------

ORF1ab         5341 GACIRRPFLCCKCCYDHVISTSHKLVLSVNPYVCNAPGCDVTDVTQLYLGGMSYYCKSHK
EPI_ISL_413594      ------------------------------------------------------------
EPI_ISL_413595      ------------------------------------------------------------
EPI_ISL_413596      ------------------------------------------------------------
EPI_ISL_413597      ------------------------------------------------------------
EPI_ISL_413598      ------------------------------------------------------------
EPI_ISL_413599      ------------------------------------------------------------
EPI_ISL_413600      ------------------------------------------------------------
EPI_ISL_413601      ------------------------------------------------------------
EPI_ISL_424217      ------------------------------------------------------------
EPI_ISL_424218      ------------------------------------------------------------
EPI_ISL_424220      ------------------------------------------------------------
EPI_ISL_424232      ------------------------------------------------------------
EPI_ISL_424266      ------------------------------------------------------------
EPI_ISL_424272      ------------------------------------------------------------
EPI_ISL_424283      ------------------------------------------------------------
EPI_ISL_424288      ------------------------------------------------------------
EPI_ISL_424292      ------------------------------------------------------------
EPI_ISL_424295      ------------------------------------------------------------
EPI_ISL_426882      ------------------------------------------------------------
```

```
ORF1ab          5401 PPISFPLCANGQVFGLYKNTCVGSDNVTDFNAIATCDWTNAGDYILANTCTERLKLFAAE
EPI_ISL_413594       ------------------------------------------------------------
EPI_ISL_413595       ------------------------------------------------------------
EPI_ISL_413596       ------------------------------------------------------------
EPI_ISL_413597       ------------------------------------------------------------
EPI_ISL_413598       ------------------------------------------------------------
EPI_ISL_413599       ------------------------------------------------------------
EPI_ISL_413600       ------------------------------------------------------------
EPI_ISL_413601       ------------------------------------------------------------
EPI_ISL_424217       ------------------------------------------------------------
EPI_ISL_424218       ------------------------------------------------------------
EPI_ISL_424220       ------------------------------------------------------------
EPI_ISL_424232       ------------------------------------------------------------
EPI_ISL_424266       ------------------------------------------------------------
EPI_ISL_424272       ------------------------------------------------------------
EPI_ISL_424283       ------------------------------------------------------------
EPI_ISL_424288       ------------------------------------------------------------
EPI_ISL_424292       ------------------------------------------------------------
EPI_ISL_424295       ------------------------------------------------------------
EPI_ISL_426882       ------------------------------------------------------------

ORF1ab          5461 TLKATEETFKLSYGIATVREVLSDRELHLSWEVGKPRPPLNRNYVFTGYRVTKNSKVQIG
EPI_ISL_413594       ------------------------------------------------------------
EPI_ISL_413595       ------------------------------------------------------------
EPI_ISL_413596       ------------------------------------------------------------
EPI_ISL_413597       ------------------------------------------------------------
EPI_ISL_413598       ------------------------------------------------------------
EPI_ISL_413599       ------------------------------------------------------------
EPI_ISL_413600       ------------------------------------------------------------
EPI_ISL_413601       ------------------------------------------------------------
EPI_ISL_424217       ------------------------------------------------------------
EPI_ISL_424218       ------------------------------------------------------------
EPI_ISL_424220       ------------------------------------------------------------
EPI_ISL_424232       ------------------------------------------------------------
EPI_ISL_424266       ------------------------------------------------------------
EPI_ISL_424272       ------------------------------------------------------------
EPI_ISL_424283       ------------------------------------------------------------
EPI_ISL_424288       ------------------------------------------------------------
EPI_ISL_424292       ------------------------------------------------------------
EPI_ISL_424295       ------------------------------------------------------------
EPI_ISL_426882       ------------------------------------------------------------

ORF1ab          5521 EYTFEKGDYGDAVVYRGTTTYKLNVGDYFVLTSHTVMPLSAPTLVPQEHYVRITGLYPTL
EPI_ISL_413594       ------------------------------------------------------------
EPI_ISL_413595       ------------------------------------------------------------
EPI_ISL_413596       ------------------------------------------------------------
EPI_ISL_413597       ------------------------------------------------------------
EPI_ISL_413598       ------------------------------------------------------------
EPI_ISL_413599       ------------------------------------------------------------
EPI_ISL_413600       ------------------------------------------------------------
EPI_ISL_413601       ------------------------------------------------------------
EPI_ISL_424217       ------------------------------------------------------------
EPI_ISL_424218       ------------------------------------------------------------
EPI_ISL_424220       ------------------------------------------------------------
EPI_ISL_424232       ------------------------------------------------------------
EPI_ISL_424266       ------------------------------------------------------------
EPI_ISL_424272       ------------------------------------------------------------
EPI_ISL_424283       ------------------------------------------------------------
EPI_ISL_424288       ------------------------------------------------------------
EPI_ISL_424292       ------------------------------------------------------------
EPI_ISL_424295       ------------------------------------------------------------
EPI_ISL_426882       ------------------------------------------------------------
```

```
ORF1ab          5581 NISDEFSSNVANYQKVGMQKYSTLQGPPGTGKSHFAIGLALYYPSARIVYTACSHAAVDA
EPI_ISL_413594       ------------------------------------------------------------
EPI_ISL_413595       ------------------------------------------------------------
EPI_ISL_413596       ------------------------------------------------------------
EPI_ISL_413597       ------------------------------------------------------------
EPI_ISL_413598       ------------------------------------------------------------
EPI_ISL_413599       ------------------------------------------------------------
EPI_ISL_413600       ------------------------------------------------------------
EPI_ISL_413601       ------------------------------------------------------------
EPI_ISL_424217       ------------------------------------------------------------
EPI_ISL_424218       ------------------------------------------------------------
EPI_ISL_424220       ------------------------------------------------------------
EPI_ISL_424232       ------------------------------------------------------------
EPI_ISL_424266       ------------------------------------------------------------
EPI_ISL_424272       ------------------------------------------------------------
EPI_ISL_424283       ------------------------------------------------------------
EPI_ISL_424288       ------------------------------------------------------------
EPI_ISL_424292       ------------------------------------------------------------
EPI_ISL_424295       ------------------------------------------------------------
EPI_ISL_426882       ------------------------------------------------------------

ORF1ab          5641 LCEKALKYLPIDKCSRIIPARARVECFDKFKVNSTLEQYVFCTVNALPETTADIVVFDEI
EPI_ISL_413594       ------------------------------------------------------------
EPI_ISL_413595       ------------------------------------------------------------
EPI_ISL_413596       ------------------------------------------------------------
EPI_ISL_413597       ------------------------------------------------------------
EPI_ISL_413598       ------------------------------------------------------------
EPI_ISL_413599       ------------------------------------------------------------
EPI_ISL_413600       ------------------------------------------------------------
EPI_ISL_413601       ------------------------------------------------------------
EPI_ISL_424217       ------------------------------------------------------------
EPI_ISL_424218       ------------------------------------------------------------
EPI_ISL_424220       ------------------------------------------------------------
EPI_ISL_424232       ------------------------------------------------------------
EPI_ISL_424266       ------------------------------------------------------------
EPI_ISL_424272       ------------------------------------------------------------
EPI_ISL_424283       ------------------------------------------------------------
EPI_ISL_424288       ------------------------------------------------------------
EPI_ISL_424292       ------------------------------------------------------------
EPI_ISL_424295       ------------------------------------------------------------
EPI_ISL_426882       ------------------------------------------------------------

ORF1ab          5701 SMATNYDLSVVNARLRAKHYVYIGDPAQLPAPRTLLTKGTLEPEYFNSVCRLMKTIGPDM
EPI_ISL_413594       ------------------------------------------------------------
EPI_ISL_413595       ------------------------------------------------------------
EPI_ISL_413596       ------------------------------------------------------------
EPI_ISL_413597       ------------------------------------------------------------
EPI_ISL_413598       ------------------------------------------------------------
EPI_ISL_413599       ------------------------------------------------------------
EPI_ISL_413600       ------------------------------------------------------------
EPI_ISL_413601       ------------------------------------------------------------
EPI_ISL_424217       ------------------------------------------------------------
EPI_ISL_424218       ------------------------------------------------------------
EPI_ISL_424220       ------------------------------------------------------------
EPI_ISL_424232       ------------------------------------------------------------
EPI_ISL_424266       ------------------------------------------------------------
EPI_ISL_424272       ------------------------------------------------------------
EPI_ISL_424283       ------------------------------------------------------------
EPI_ISL_424288       ------------------------------------------------------------
EPI_ISL_424292       ------------------------------------------------------------
EPI_ISL_424295       ------------------------------------------------------------
EPI_ISL_426882       ------------------------------------------------------------
```

```
ORF1ab         5761 FLGTCRRCPAEIVDTVSALVYDNKLKAHKDKSAQCFKMFYKGVITHDVSSAINRPQIGVV
EPI_ISL_413594      ------------------------------------------------------------
EPI_ISL_413595      ------------------------------------------------------------
EPI_ISL_413596      ------------------------------------------------------------
EPI_ISL_413597      ------------------------------------------------------------
EPI_ISL_413598      ------------------------------------------------------------
EPI_ISL_413599      ------------------------------------------------------------
EPI_ISL_413600      ------------------------------------------------------------
EPI_ISL_413601      ------------------------------------------------------------
EPI_ISL_424217      ------------------------------------------------------------
EPI_ISL_424218      ------------------------------------------------------------
EPI_ISL_424220      ------------------------------------------------------------
EPI_ISL_424232      ------------------------------------------------------------
EPI_ISL_424266      ------------------------------------------------------------
EPI_ISL_424272      ------------------------------------------------------------
EPI_ISL_424283      ------------------------------------------------------------
EPI_ISL_424288      ------------------------------------------------------------
EPI_ISL_424292      ------------------------------------------------------------
EPI_ISL_424295      ------------------------------------------------------------
EPI_ISL_426882      ------------------------------------------------------------

ORF1ab         5821 REFLTRNPAWRKAVFISPYNSQNAVASKILGLPTQTVDSSQGSEYDYVIFTQTTETAHSC
EPI_ISL_413594      ------------------------------------------------------------
EPI_ISL_413595      ------------------------------------------------------------
EPI_ISL_413596      ------------------------------------------------------------
EPI_ISL_413597      ------------------------------------------------------------
EPI_ISL_413598      ------------------------------------------------------------
EPI_ISL_413599      ------------------------------------------------------------
EPI_ISL_413600      ------------------------------------------------------------
EPI_ISL_413601      ------------------------------------------------------------
EPI_ISL_424217      ------------------------------------------------------------
EPI_ISL_424218      ------------------------------------------------------------
EPI_ISL_424220      ------------------------------------------------------------
EPI_ISL_424232      ------------------------------------------------------------
EPI_ISL_424266      ------------------------------------------------------------
EPI_ISL_424272      ------------------------------------------------------------
EPI_ISL_424283      ------------------------------------------------------------
EPI_ISL_424288      ------------------------------------------------------------
EPI_ISL_424292      ------------------------------------------------------------
EPI_ISL_424295      ------------------------------------------------------------
EPI_ISL_426882      ------------------------------------------------------------

ORF1ab         5881 NVNRFNVAITRAKVGILCIMSDRDLYDKLQFTSLEIPRRNVATLQAENVTGLFKDCSKVI
EPI_ISL_413594      ------------------------------------------------------------
EPI_ISL_413595      ------------------------------------------------------------
EPI_ISL_413596      ------------------------------------------------------------
EPI_ISL_413597      ------------------------------------------------------------
EPI_ISL_413598      ------------------------------------------------------------
EPI_ISL_413599      ------------------------------------------------------------
EPI_ISL_413600      ------------------------------------------------------------
EPI_ISL_413601      ------------------------------------------------------------
EPI_ISL_424217      ------------------------------------------------------------
EPI_ISL_424218      ------------------------------------------------------------
EPI_ISL_424220      ------------------------------------------------------------
EPI_ISL_424232      ------------------------------------------------------------
EPI_ISL_424266      ------------------------------------------------------------
EPI_ISL_424272      ------------------------------------------------------------
EPI_ISL_424283      ------------------------------------------------------------
EPI_ISL_424288      ------------------------------------------------------------
EPI_ISL_424292      ------------------------------------------------------------
EPI_ISL_424295      ------------------------------------------------------------
EPI_ISL_426882      ------------------------------------------------------------
```

```
ORF1ab          5941 TGLHPTQAPTHLSVDTKFKTEGLCVDIPGIPKDMTYRRLISMMGFKMNYQVNGYPNMFIT
EPI_ISL_413594       ------------------------------------------------------------
EPI_ISL_413595       ------------------------------------------------------------
EPI_ISL_413596       ------------------------------------------------------------
EPI_ISL_413597       ------------------------------------------------------------
EPI_ISL_413598       ------------------------------------------------------------
EPI_ISL_413599       ------------------------------------------------------------
EPI_ISL_413600       ------------------------------------------------------------
EPI_ISL_413601       ------------------------------------------------------------
EPI_ISL_424217       ------------------------------------------------------------
EPI_ISL_424218       ------------------------------------------------------------
EPI_ISL_424220       ------------------------------------------------------------
EPI_ISL_424232       ------------------------------------------------------------
EPI_ISL_424266       ------------------------------------------------------------
EPI_ISL_424272       ------------------------------------------------------------
EPI_ISL_424283       ------------------------------------------------------------
EPI_ISL_424288       ------------------------------------------------------------
EPI_ISL_424292       ------------------------------------------------------------
EPI_ISL_424295       ------------------------------------------------------------
EPI_ISL_426882       ------------------------------------------------------------

ORF1ab          6001 REEAIRHVRAWIGFDVEGCHATREAVGTNLPLQLGFSTGVNLVAVPTGYVDTPNNTDFSR
EPI_ISL_413594       ------------------------------------------------------------
EPI_ISL_413595       ------------------------------------------------------------
EPI_ISL_413596       ------------------------------------------------------------
EPI_ISL_413597       ------------------------------------------------------------
EPI_ISL_413598       ------------------------------------------------------------
EPI_ISL_413599       ------------------------------------------------------------
EPI_ISL_413600       ------------------------------------------------------------
EPI_ISL_413601       ------------------------------------------------------------
EPI_ISL_424217       ------------------------------------------------------------
EPI_ISL_424218       ------------------------------------------------------------
EPI_ISL_424220       ------------------------------------------------------------
EPI_ISL_424232       ------------------------------------------------------------
EPI_ISL_424266       ------------------------------------------------------------
EPI_ISL_424272       ------------------------------------------------------------
EPI_ISL_424283       ------------------------------------------------------------
EPI_ISL_424288       ------------------------------------------------------------
EPI_ISL_424292       ------------------------------------------------------------
EPI_ISL_424295       ------------------------------------------------------------
EPI_ISL_426882       ------------------------------------------------------------

ORF1ab          6061 VSAKPPPGDQFKHLIPLMYKGLPWNVVRIKIVQMLSDTLKNLSDRVVFVLWAHGFELTSM
EPI_ISL_413594       ------------------------------------------------------------
EPI_ISL_413595       ------------------------------------------------------------
EPI_ISL_413596       ------------------------------------------------------------
EPI_ISL_413597       ------------------------------------------------------------
EPI_ISL_413598       ------------------------------------------------------------
EPI_ISL_413599       ------------------------------------------------------------
EPI_ISL_413600       ------------------------------------------------------------
EPI_ISL_413601       ------------------------------------------------------------
EPI_ISL_424217       ------------------------------------------------------------
EPI_ISL_424218       ------------------------------------------------------------
EPI_ISL_424220       ------------------------------------------------------------
EPI_ISL_424232       ------------------------------------------------------------
EPI_ISL_424266       ------------------------------------------------------------
EPI_ISL_424272       ------------------------------------------------------------
EPI_ISL_424283       ------------------------------------------------------------
EPI_ISL_424288       ------------------------------------------------------------
EPI_ISL_424292       ------------------------------------------------------------
EPI_ISL_424295       ------------------------------------------------------------
EPI_ISL_426882       ------------------------------------------------------------
```

```
ORF1ab          6121 KYFVKIGPERTCCLCDRRATCFSTASDTYACWHHSIGFDYVYNPFMIDVQQWGFTGNLQS
EPI_ISL_413594       ------------------------------------------------------------
EPI_ISL_413595       ------------------------------------------------------------
EPI_ISL_413596       ------------------------------------------------------------
EPI_ISL_413597       ------------------------------------------------------------
EPI_ISL_413598       ------------------------------------------------------------
EPI_ISL_413599       ------------------------------------------------------------
EPI_ISL_413600       ------------------------------------------------------------
EPI_ISL_413601       ------------------------------------------------------------
EPI_ISL_424217       ------------------------------------------------------------
EPI_ISL_424218       ------------------------------------------------------------
EPI_ISL_424220       ------------------------------------------------------------
EPI_ISL_424232       ------------------------------------------------------------
EPI_ISL_424266       ------------------------------------------------------------
EPI_ISL_424272       ------------------------------------------------------------
EPI_ISL_424283       ------------------------------------------------------------
EPI_ISL_424288       ------------------------------------------------------------
EPI_ISL_424292       ------------------------------------------------------------
EPI_ISL_424295       ------------------------------------------------------------
EPI_ISL_426882       ------------------------------------------------------------
```

```
ORF1ab          6181 NHDLYCQVHGNAHVASCDAIMTRCLAVHECFVKRVDWTIEYPIIGDELKINAACRKVQHM
EPI_ISL_413594       ------------------------------------------------------------
EPI_ISL_413595       ------------------------------------------------------------
EPI_ISL_413596       ------------------------------------------------------------
EPI_ISL_413597       ------------------------------------------------------------
EPI_ISL_413598       ------------------------------------------------------------
EPI_ISL_413599       ------------------------------------------------------------
EPI_ISL_413600       ------------------------------------------------------------
EPI_ISL_413601       ------------------------------------------------------------
EPI_ISL_424217       ------------------------------------------------------------
EPI_ISL_424218       ------------------------------------------------------------
EPI_ISL_424220       ------------------------------------------------------------
EPI_ISL_424232       ------------------------------------------------------------
EPI_ISL_424266       ------------------------------------------------------------
EPI_ISL_424272       ------------------------------------------------------------
EPI_ISL_424283       ------------------------------------------------------------
EPI_ISL_424288       ------------------------------------------------------------
EPI_ISL_424292       ------------------------------------------------------------
EPI_ISL_424295       ------------------------------------------------------------
EPI_ISL_426882       ------------------------------------------------------------
```

```
ORF1ab          6241 VVKAALLADKFPVLHDIGNPKAIKCVPQADVEWKFYDAQPCSDKAYKIEELFYSYATHSD
EPI_ISL_413594       ------------------------------------------------------------
EPI_ISL_413595       ------------------------------------------------------------
EPI_ISL_413596       ------------------------------------------------------------
EPI_ISL_413597       ------------------------------------------------------------
EPI_ISL_413598       ------------------------------------------------------------
EPI_ISL_413599       ------------------------------------------------------------
EPI_ISL_413600       ------------------------------------------------------------
EPI_ISL_413601       ------------------------------------------------------------
EPI_ISL_424217       ------------------------------------------------------------
EPI_ISL_424218       ------------------------------------------------------------
EPI_ISL_424220       ------------------------------------------------------------
EPI_ISL_424232       ------------------------------------------------------------
EPI_ISL_424266       ------------------------------------------------------------
EPI_ISL_424272       ------------------------------------------------------------
EPI_ISL_424283       ------------------------------------------------------------
EPI_ISL_424288       ------------------------------------------------------------
EPI_ISL_424292       ------------------------------------------------------------
EPI_ISL_424295       ------------------------------------------------------------
EPI_ISL_426882       ------------------------------------------------------------
```

```
ORF1ab         6301 KFTDGVCLFWNCNVDRYPANSIVCRFDTRVLSNLNLPGCDGGSLYVNKHAFHTPAFDKSA
EPI_ISL_413594      ------------------------------------------------------------
EPI_ISL_413595      ------------------------------------------------------------
EPI_ISL_413596      ------------------------------------------------------------
EPI_ISL_413597      ------------------------------------------------------------
EPI_ISL_413598      ------------------------------------------------------------
EPI_ISL_413599      ------------------------------------------------------------
EPI_ISL_413600      ------------------------------------------------------------
EPI_ISL_413601      ------------------------------------------------------------
EPI_ISL_424217      ------------------------------------------------------------
EPI_ISL_424218      ------------------------------------------------------------
EPI_ISL_424220      ------------------------------------------------------------
EPI_ISL_424232      ------------------------------------------------------------
EPI_ISL_424266      ------------------------------------------------------------
EPI_ISL_424272      ------------------------------------------------------------
EPI_ISL_424283      ------------------------------------------------------------
EPI_ISL_424288      ------------------------------------------------------------
EPI_ISL_424292      ------------------------------------------------------------
EPI_ISL_424295      ------------------------------------------------------------
EPI_ISL_426882      ------------------------------------------------------------

ORF1ab         6361 FVNLKQLPFFYYSDSPCESHGKQVVSDIDYVPLKSATCITRCNLGGAVCRHHANEYRLYL
EPI_ISL_413594      ------------------------------------------------------------
EPI_ISL_413595      ------------------------------------------------------------
EPI_ISL_413596      ------------------------------------------------------------
EPI_ISL_413597      ------------------------------------------------------------
EPI_ISL_413598      ------------------------------------------------------------
EPI_ISL_413599      ------------------------------------------------------------
EPI_ISL_413600      ------------------------------------------------------------
EPI_ISL_413601      ------------------------------------------------------------
EPI_ISL_424217      ------------------------------------------------------------
EPI_ISL_424218      ------------------------------------------------------------
EPI_ISL_424220      ------------------------------------------------------------
EPI_ISL_424232      ------------------------------------------------------------
EPI_ISL_424266      ------------------------------------------------------------
EPI_ISL_424272      ------------------------------------------------------------
EPI_ISL_424283      ------------------------------------------------------------
EPI_ISL_424288      ------------------------------------------------------------
EPI_ISL_424292      ------------------------------------------------------------
EPI_ISL_424295      ------------------------------------------------------------
EPI_ISL_426882      ------------------------------------------------------------

ORF1ab         6421 DAYNMMISAGFSLWVYKQFDTYNLWNTFTRLQSLENVAFNVVNKGHFDGQQGEVPVSIIN
EPI_ISL_413594      ------------------------------------------------------------
EPI_ISL_413595      ------------------------------------------------------------
EPI_ISL_413596      ------------------------------------------------------------
EPI_ISL_413597      ------------------------------------------------------------
EPI_ISL_413598      ------------------------------------------------------------
EPI_ISL_413599      ------------------------------------------------------------
EPI_ISL_413600      ------------------------------------------------------------
EPI_ISL_413601      ------------------------------------------------------------
EPI_ISL_424217      ------------------------------------------------------------
EPI_ISL_424218      ------------------------------------------------------------
EPI_ISL_424220      ------------------------------------------------------------
EPI_ISL_424232      ------------------------------------------------------------
EPI_ISL_424266      ------------------------------------------------------------
EPI_ISL_424272      ------------------------------------------------------------
EPI_ISL_424283      ------------------------------------------------------------
EPI_ISL_424288      ------------------------------------------------------------
EPI_ISL_424292      ------------------------------------------------------------
EPI_ISL_424295      ------------------------------------------------------------
EPI_ISL_426882      ------------------------------------------------------------
```

```
ORF1ab          6481 NTVYTKVDGVDVELFENKTTLPVNVAFELWAKRNIKPVPEVKILNNLGVDIAANTVIWDY
EPI_ISL_413594       ------------------------------------------------------------
EPI_ISL_413595       ------------------------------------------------------------
EPI_ISL_413596       ------------------------------------------------------------
EPI_ISL_413597       ------------------------------------------------------------
EPI_ISL_413598       ------------------------------------------------------------
EPI_ISL_413599       ------------------------------------------------------------
EPI_ISL_413600       ------------------------------------------------------------
EPI_ISL_413601       ------------------------------------------------------------
EPI_ISL_424217       ------------------------------------------------------------
EPI_ISL_424218       ------------------------------------------------------------
EPI_ISL_424220       ------------------------------------------------------------
EPI_ISL_424232       ------------------------------------------------------------
EPI_ISL_424266       ------------------------------------------------------------
EPI_ISL_424272       ------------------------------------------------------------
EPI_ISL_424283       ------------------------------------------------------------
EPI_ISL_424288       ------------------------------------------------------------
EPI_ISL_424292       ------------------------------------------------------------
EPI_ISL_424295       ------------------------------------------------------------
EPI_ISL_426882       ------------------------------------------------------------

ORF1ab          6541 KRDAPAHISTIGVCSMTDIAKKPTETICAPLTVFFDGRVDGQVDLFRNARNGVLITEGSV
EPI_ISL_413594       ------------------------------------------------------------
EPI_ISL_413595       ------------------------------------------------------------
EPI_ISL_413596       ------------------------------------------------------------
EPI_ISL_413597       ------------------------------------------------------------
EPI_ISL_413598       ------------------------------------------------------------
EPI_ISL_413599       ------------------------------------------------------------
EPI_ISL_413600       ------------------------------------------------------------
EPI_ISL_413601       ------------------------------------------------------------
EPI_ISL_424217       ------------------------------------------------------------
EPI_ISL_424218       ------------------------------------------------------------
EPI_ISL_424220       ------------------------------------------------------------
EPI_ISL_424232       ------------------------------------------------------------
EPI_ISL_424266       ------------------------------------------------------------
EPI_ISL_424272       ------------------------------------------------------------
EPI_ISL_424283       ------------------------------------------------------------
EPI_ISL_424288       ------------------------------------------------------------
EPI_ISL_424292       ------------------------------------------------------------
EPI_ISL_424295       ------------------------------------------------------------
EPI_ISL_426882       ------------------------------------------------------------

ORF1ab          6601 KGLQPSVGPKQASLNGVTLIGEAVKTQFNYYKKVDGVVQQLPETYFTQSRNLQEFKPRSQ
EPI_ISL_413594       ------------------------------------------------------------
EPI_ISL_413595       ------------------------------------------------------------
EPI_ISL_413596       ------------------------------------------------------------
EPI_ISL_413597       ------------------------------------------------------------
EPI_ISL_413598       ------------------------------------------------------------
EPI_ISL_413599       ------------------------------------------------------------
EPI_ISL_413600       ------------------------------------------------------------
EPI_ISL_413601       ------------------------------------------------------------
EPI_ISL_424217       ------------------------------------------------------------
EPI_ISL_424218       ------------------------------------------------------------
EPI_ISL_424220       ------------------------------------------------------------
EPI_ISL_424232       ------------------------------------------------------------
EPI_ISL_424266       ------------------------------------------------------------
EPI_ISL_424272       ------------------------------------------------------------
EPI_ISL_424283       ------------------------------------------------------------
EPI_ISL_424288       ------------------------------------------------------------
EPI_ISL_424292       ------------------------------------------------------------
EPI_ISL_424295       ------------------------------------------------------------
EPI_ISL_426882       ------------------------------------------------------------
```

```
ORF1ab          6661 MEIDFLELAMDEFIERYKLEGYAFEHIVYGDFSHSQLGGLHLLIGLAKRFKESPFELEDF
EPI_ISL_413594       ------------------------------------------------------------
EPI_ISL_413595       ------------------------------------------------------------
EPI_ISL_413596       ------------------------------------------------------------
EPI_ISL_413597       ------------------------------------------------------------
EPI_ISL_413598       ------------------------------------------------------------
EPI_ISL_413599       ------------------------------------------------------------
EPI_ISL_413600       ------------------------------------------------------------
EPI_ISL_413601       ------------------------------------------------------------
EPI_ISL_424217       ------------------------------------------------------------
EPI_ISL_424218       ------------------------------------------------------------
EPI_ISL_424220       ------------------------------------------------------------
EPI_ISL_424232       ------------------------------------------------------------
EPI_ISL_424266       ------------------------------------------------------------
EPI_ISL_424272       ------------------------------------------------------------
EPI_ISL_424283       ------------------------------------------------------------
EPI_ISL_424288       ------------------------------------------------------------
EPI_ISL_424292       ------------------------------------------------------------
EPI_ISL_424295       ------------------------------------------------------------
EPI_ISL_426882       ------------------------------------------------------------

ORF1ab          6721 IPMDSTVKNYFITDAQTGSSKCVCSVIDLLLDDFVEIIKSQDLSVVSKVVKVTIDYTEIS
EPI_ISL_413594       ------------------------------------------------------------
EPI_ISL_413595       ------------------------------------------------------------
EPI_ISL_413596       ------------------------------------------------------------
EPI_ISL_413597       ------------------------------------------------------------
EPI_ISL_413598       ------------------------------------------------------------
EPI_ISL_413599       ------------------------------------------------------------
EPI_ISL_413600       ------------------------------------------------------------
EPI_ISL_413601       ------------------------------------------------------------
EPI_ISL_424217       ------------------------------------------------------------
EPI_ISL_424218       ------------------------------------------------------------
EPI_ISL_424220       ------------------------------------------------------------
EPI_ISL_424232       ------------------------------------------------------------
EPI_ISL_424266       ------------------------------------------------------------
EPI_ISL_424272       ------------------------------------------------------------
EPI_ISL_424283       ------------------------------------------------------------
EPI_ISL_424288       ------------------------------------------------------------
EPI_ISL_424292       ------------------------------------------------------------
EPI_ISL_424295       ------------------------------------------------------------
EPI_ISL_426882       ------------------------------------------------------------

ORF1ab          6781 FMLWCKDGHVETFYPKLQSSQAWQPGVAMPNLYKMQRMLLEKCDLQNYGDSATLPKGIMM
EPI_ISL_413594       ------------------------------------------------------------
EPI_ISL_413595       ------------------------------------------------------------
EPI_ISL_413596       ------------------------------------------------------------
EPI_ISL_413597       ------------------------------------------------------------
EPI_ISL_413598       ------------------------------------------------------------
EPI_ISL_413599       ------------------------------------------------------------
EPI_ISL_413600       ------------------------------------------------------------
EPI_ISL_413601       ------------------------------------------------------------
EPI_ISL_424217       ------------------------------------------------------------
EPI_ISL_424218       ------------------------------------------------------------
EPI_ISL_424220       ------------------------------------------------------------
EPI_ISL_424232       ------------------------------------------------------------
EPI_ISL_424266       ------------------------------------------------------------
EPI_ISL_424272       ------------------------------------------------------------
EPI_ISL_424283       ------------------------------------------------------------
EPI_ISL_424288       ------------------------------------------------------------
EPI_ISL_424292       ------------------------------------------------------------
EPI_ISL_424295       ------------------------------------------------------------
EPI_ISL_426882       ------------------------------------------------------------
```

```
ORF1ab              6841 NVAKYTQLCQYLNTLTLAVPYNMRVIHFGAGSDKGVAPGTAVLRQWLPTGTLLVDSDLND
EPI_ISL_413594           ------------------------------------------------------------
EPI_ISL_413595           ------------------------------------------------------------
EPI_ISL_413596           ------------------------------------------------------------
EPI_ISL_413597           ------------------------------------------------------------
EPI_ISL_413598           ------------------------------------------------------------
EPI_ISL_413599           ------------------------------------------------------------
EPI_ISL_413600           ------------------------------------------------------------
EPI_ISL_413601           ------------------------------------------------------------
EPI_ISL_424217           ------------------------------------------------------------
EPI_ISL_424218           ------------------------------------------------------------
EPI_ISL_424220           ------------------------------------------------------------
EPI_ISL_424232           ------------------------------------------------------------
EPI_ISL_424266           ------------------------------------------------------------
EPI_ISL_424272           ------------------------------------------------------------
EPI_ISL_424283           ------------------------------------------------------------
EPI_ISL_424288           ------------------------------------------------------------
EPI_ISL_424292           ------------------------------------------------------------
EPI_ISL_424295           ------------------------------------------------------------
EPI_ISL_426882           ------------------------------------------------------------

ORF1ab              6901 FVSDADSTLIGDCATVHTANKWDLIISDMYDPKTKNVTKENDSKEGFFTYICGFIQQKLA
EPI_ISL_413594           ------------------------------------------------------------
EPI_ISL_413595           ------------------------------------------------------------
EPI_ISL_413596           ------------------------------------------------------------
EPI_ISL_413597           ------------------------------------------------------------
EPI_ISL_413598           ------------------------------------------------------------
EPI_ISL_413599           ------------------------------------------------------------
EPI_ISL_413600           ------------------------------------------------------------
EPI_ISL_413601           ------------------------------------------------------------
EPI_ISL_424217           ------------------------------------------------------------
EPI_ISL_424218           ------------------------------------------------------------
EPI_ISL_424220           ------------------------------------------------------------
EPI_ISL_424232           ------------------------------------------------------------
EPI_ISL_424266           ------------------------------------------------------------
EPI_ISL_424272           ------------------------------------------------------------
EPI_ISL_424283           ------------------------------------------------------------
EPI_ISL_424288           ------------------------------------------------------------
EPI_ISL_424292           ------------------------------------------------------------
EPI_ISL_424295           ------------------------------------------------------------
EPI_ISL_426882           ------------------------------------------------------------

ORF1ab              6961 LGGSVAIKITEHSWNADLYKLMGHFAWWTAFVTNVNASSSEAFLIGCNYLGKPREQIDGY
EPI_ISL_413594           ------------------------------------------------------------
EPI_ISL_413595           ------------------------------------------------------------
EPI_ISL_413596           ------------------------------------------------------------
EPI_ISL_413597           ------------------------------------------------------------
EPI_ISL_413598           ------------------------------------------------------------
EPI_ISL_413599           ------------------------------------------------------------
EPI_ISL_413600           ------------------------------------------------------------
EPI_ISL_413601           ------------------------------------------------------------
EPI_ISL_424217           ------------------------------------------------------------
EPI_ISL_424218           ------------------------------------------------------------
EPI_ISL_424220           ------------------------------------------------------------
EPI_ISL_424232           ------------------------------------------------------------
EPI_ISL_424266           ------------------------------------------------------------
EPI_ISL_424272           ------------------------------------------------------------
EPI_ISL_424283           ------------------------------------------------------------
EPI_ISL_424288           ------------------------------------------------------------
EPI_ISL_424292           ------------------------------------------------------------
EPI_ISL_424295           ------------------------------------------------------------
EPI_ISL_426882           ------------------------------------------------------------
```

```
ORF1ab          7021 VMHANYIFWRNTNPIQLSSYSLFDMSKFPLKLRGTAVMSLKEGQINDMILSLLSKGRLII
EPI_ISL_413594       ------------------------------------------------------------
EPI_ISL_413595       ------------------------------------------------------------
EPI_ISL_413596       ------------------------------------------------------------
EPI_ISL_413597       ------------------------------------------------------------
EPI_ISL_413598       ------------------------------------------------------------
EPI_ISL_413599       ------------------------------------------------------------
EPI_ISL_413600       ------------------------------------------------------------
EPI_ISL_413601       ------------------------------------------------------------
EPI_ISL_424217       ------------------------------------------------------------
EPI_ISL_424218       ------------------------------------------------------------
EPI_ISL_424220       ------------------------------------------------------------
EPI_ISL_424232       ------------------------------------------------------------
EPI_ISL_424266       ------------------------------------------------------------
EPI_ISL_424272       ------------------------------------------------------------
EPI_ISL_424283       ------------------------------------------------------------
EPI_ISL_424288       ------------------------------------------------------------
EPI_ISL_424292       ------------------------------------------------------------
EPI_ISL_424295       ------------------------------------------------------------
EPI_ISL_426882       ------------------------------------------------------------

ORF1ab          7081 RENNRVVISSDVLVNN
EPI_ISL_413594       ----------------
EPI_ISL_413595       ----------------
EPI_ISL_413596       ----------------
EPI_ISL_413597       ----------------
EPI_ISL_413598       ----------------
EPI_ISL_413599       ----------------
EPI_ISL_413600       ----------------
EPI_ISL_413601       ----------------
EPI_ISL_424217       ----------------
EPI_ISL_424218       ----------------
EPI_ISL_424220       ----------------
EPI_ISL_424232       ----------------
EPI_ISL_424266       ----------------
EPI_ISL_424272       ----------------
EPI_ISL_424283       ----------------
EPI_ISL_424288       ----------------
EPI_ISL_424292       ----------------
EPI_ISL_424295       ----------------
EPI_ISL_426882       ----------------
```

Clade S

```
ORF1ab             1 MESLVPGFNEKTHVQLSLPVLQVRDVLVRGFGDSVEEVLSEARQHLKDGTCGLVEVEKGV
EPI_ISL_424219     1 ------------------------------------------------------------
EPI_ISL_424222     1 ------------------------------------------------------------
EPI_ISL_424223     1 ------------------------------------------------------------
EPI_ISL_424226     1 ------------------------------------------------------------
EPI_ISL_424228     1 ------------------------------------------------------------
EPI_ISL_424230     1 ------------------------------------------------------------
EPI_ISL_424231     1 ------------------------------------------------------------
EPI_ISL_424240     1 ------------------------------------------------------------
EPI_ISL_424241     1 ------------------------------------------------------------
EPI_ISL_424243     1 ------------------------------------------------------------
EPI_ISL_424247     1 ------------------------------------------------------------
EPI_ISL_424249     1 ------------------------------------------------------------
EPI_ISL_424250     1 ------------------------------------------------------------
EPI_ISL_424252     1 ------------------------------------------------------------
EPI_ISL_424258     1 ------------------------------------------------------------
EPI_ISL_424268     1 ------------------------------------------------------------
EPI_ISL_424269     1 ------------------------------------------------------------
EPI_ISL_424275     1 ------------------------------------------------------------
EPI_ISL_424276     1 ------------------------------------------------------------
EPI_ISL_424279     1 ------------------------------------------------------------

ORF1ab            61 LPQLEQPYVFIKRSDARTAPHGHVMVELVAELEGIQYGRSGETLGVLVPHVGEIPVAYRK
EPI_ISL_424219     1 ------------------------------------------------------------
EPI_ISL_424222     1 ------------------------------------------------------------
EPI_ISL_424223     1 ------------------------------------------------------------
EPI_ISL_424226     1 ------------------------------------------------------------
EPI_ISL_424228     1 ------------------------------------------------------------
EPI_ISL_424230     1 ------------------------------------------------------------
EPI_ISL_424231     1 ------------------------------------------------------------
EPI_ISL_424240     1 ------------------------------------------------------------
EPI_ISL_424241     1 ------------------------------------------------------------
EPI_ISL_424243     1 ------------------------------------------------------------
EPI_ISL_424247     1 ------------------------------------------------------------
EPI_ISL_424249     1 ------------------------------------------------------------
EPI_ISL_424250     1 ------------------------------------------------------------
EPI_ISL_424252     1 ------------------------------------------------------------
EPI_ISL_424258     1 ------------------------------------------------------------
EPI_ISL_424268     1 ------------------------------------------------------------
EPI_ISL_424269     1 ------------------------------------------------------------
EPI_ISL_424275     1 ------------------------------------------------------------
EPI_ISL_424276     1 ------------------------------------------------------------
EPI_ISL_424279     1 ------------------------------------------------------------

ORF1ab           121 VLLRKNGNKGAGGHSYGADLKSFDLGDELGTDPYEDFQENWNTKHSSGVTRELMRELNGG
EPI_ISL_424219     1 ------------------------------------------------------------
EPI_ISL_424222     1 ------------------------------------------------------------
EPI_ISL_424223     1 ------------------------------------------------------------
EPI_ISL_424226     1 ------------------------------------------------------------
EPI_ISL_424228     1 ------------------------------------------------------------
EPI_ISL_424230     1 ------------------------------------------------------------
EPI_ISL_424231     1 ------------------------------------------------------------
EPI_ISL_424240     1 ------------------------------------------------------------
EPI_ISL_424241     1 ------------------------------------------------------------
EPI_ISL_424243     1 ------------------------------------------------------------
EPI_ISL_424247     1 ------------------------------------------------------------
EPI_ISL_424249     1 ------------------------------------------------------------
EPI_ISL_424250     1 ------------------------------------------------------------
EPI_ISL_424252     1 ------------------------------------------------------------
EPI_ISL_424258     1 ------------------------------------------------------------
```

```
EPI_ISL_424268    1 ------------------------------------------------------------
EPI_ISL_424269    1 ------------------------------------------------------------
EPI_ISL_424275    1 ------------------------------------------------------------
EPI_ISL_424276    1 ------------------------------------------------------------
EPI_ISL_424279    1 ------------------------------------------------------------
```

```
ORF1ab          181 AYTRYVDNNFCGPDGYPLECIKDLLARAGKASCTLSEQLDFIDTKRGVYCCREHEHEIAW
EPI_ISL_424219    1 ------------------------------------------------------------
EPI_ISL_424222    1 ------------------------------------------------------------
EPI_ISL_424223    1 ------------------------------------------------------------
EPI_ISL_424226    1 ------------------------------------------------------------
EPI_ISL_424228    1 ------------------------------------------------------------
EPI_ISL_424230    1 ------------------------------------------------------------
EPI_ISL_424231    1 ------------------------------------------------------------
EPI_ISL_424240    1 ------------------------------------------------------------
EPI_ISL_424241    1 ------------------------------------------------------------
EPI_ISL_424243    1 ------------------------------------------------------------
EPI_ISL_424247    1 ------------------------------------------------------------
EPI_ISL_424249    1 ------------------------------------------------------------
EPI_ISL_424250    1 ------------------------------------------------------------
EPI_ISL_424252    1 ------------------------------------------------------------
EPI_ISL_424258    1 ------------------------------------------------------------
EPI_ISL_424268    1 ------------------------------------------------------------
EPI_ISL_424269    1 ------------------------------------------------------------
EPI_ISL_424275    1 ------------------------------------------------------------
EPI_ISL_424276    1 ------------------------------------------------------------
EPI_ISL_424279    1 ------------------------------------------------------------

ORF1ab          241 YTERSEKSYELQTPFEIKLAKKFDTFNGECPNFVFPLNSIIKTIQPRVEKKKLDGFMGRI
EPI_ISL_424219    1 ------------------------------------------------------------
EPI_ISL_424222    1 ------------------------------------------------------------
EPI_ISL_424223    1 ------------------------------------------------------------
EPI_ISL_424226    1 ------------------------------------------------------------
EPI_ISL_424228    1 ------------------------------------------------------------
EPI_ISL_424230    1 ------------------------------------------------------------
EPI_ISL_424231    1 ------------------------------------------------------------
EPI_ISL_424240    1 ------------------------------------------------------------
EPI_ISL_424241    1 ------------------------------------------------------------
EPI_ISL_424243    1 ------------------------------------------------------------
EPI_ISL_424247    1 ------------------------------------------------------------
EPI_ISL_424249    1 ------------------------------------------------------------
EPI_ISL_424250    1 ------------------------------------------------------------
EPI_ISL_424252    1 ------------------------------------------------------------
EPI_ISL_424258    1 ------------------------------------------------------------
EPI_ISL_424268    1 ------------------------------------------------------------
EPI_ISL_424269    1 ------------------------------------------------------------
EPI_ISL_424275    1 ------------------------------------------------------------
EPI_ISL_424276    1 ------------------------------------------------------------
EPI_ISL_424279    1 ------------------------------------------------------------

ORF1ab          301 RSVYPVASPNECNQMCLSTLMKCDHCGETSWQTGDFVKATCEFCGTENLTKEGATTCGYL
EPI_ISL_424219    1 ------------------------------------------------------------
EPI_ISL_424222    1 ------------------------------------------------------------
EPI_ISL_424223    1 ------------------------------------------------------------
EPI_ISL_424226    1 ------------------------------------------------------------
EPI_ISL_424228    1 ------------------------------------------------------------
EPI_ISL_424230    1 ------------------------------------------------------------
EPI_ISL_424231    1 ------------------------------------------------------------
EPI_ISL_424240    1 ------------------------------------------------------------
EPI_ISL_424241    1 ------------------------------------------------------------
EPI_ISL_424243    1 ------------------------------------------------------------
EPI_ISL_424247    1 ------------------------------------------------------------
EPI_ISL_424249    1 ------------------------------------------------------------
EPI_ISL_424250    1 ------------------------------------------------------------
EPI_ISL_424252    1 ------------------------------------------------------------
EPI_ISL_424258    1 ------------------------------------------------------------
EPI_ISL_424268    1 ------------------------------------------------------------
EPI_ISL_424269    1 ------------------------------------------------------------
EPI_ISL_424275    1 ------------------------------------------------------------
EPI_ISL_424276    1 ------------------------------------------------------------
```

EPI_ISL_424279   1 --

```
ORF1ab          361 PQNAVVKIYCPACHNSEVGPEHSLAEYHNESGLKTILRKGGRTIAFGGCVFSYVGCHNKC
EPI_ISL_424219    1 ------------------------------------------------------------
EPI_ISL_424222    1 ------------------------------------------------------------
EPI_ISL_424223    1 ------------------------------------------------------------
EPI_ISL_424226    1 ------------------------------------------------------------
EPI_ISL_424228    1 ------------------------------------------------------------
EPI_ISL_424230    1 ------------------------------------------------------------
EPI_ISL_424231    1 ------------------------------------------------------------
EPI_ISL_424240    1 ------------------------------------------------------------
EPI_ISL_424241    1 ------------------------------------------------------------
EPI_ISL_424243    1 ------------------------------------------------------------
EPI_ISL_424247    1 ------------------------------------------------------------
EPI_ISL_424249    1 ------------------------------------------------------------
EPI_ISL_424250    1 ------------------------------------------------------------
EPI_ISL_424252    1 ------------------------------------------------------------
EPI_ISL_424258    1 ------------------------------------------------------------
EPI_ISL_424268    1 ------------------------------------------------------------
EPI_ISL_424269    1 ------------------------------------------------------------
EPI_ISL_424275    1 ------------------------------------------------------------
EPI_ISL_424276    1 ------------------------------------------------------------
EPI_ISL_424279    1 ------------------------------------------------------------

ORF1ab          421 AYWVPRASANIGCNHTGVVGEGSEGLNDNLLEILQKEKVNINIVGDFKLNEEIAIILASF
EPI_ISL_424219    1 ------------------------------------------------------------
EPI_ISL_424222    1 ------------------------------------------------------------
EPI_ISL_424223    1 ------------------------------------------------------------
EPI_ISL_424226    1 ------------------------------------------------------------
EPI_ISL_424228    1 ------------------------------------------------------------
EPI_ISL_424230    1 ------------------------------------------------------------
EPI_ISL_424231    1 ------------------------------------------------------------
EPI_ISL_424240    1 ------------------------------------------------------------
EPI_ISL_424241    1 ------------------------------------------------------------
EPI_ISL_424243    1 ------------------------------------------------------------
EPI_ISL_424247    1 ------------------------------------------------------------
EPI_ISL_424249    1 ------------------------------------------------------------
EPI_ISL_424250    1 ------------------------------------------------------------
EPI_ISL_424252    1 ------------------------------------------------------------
EPI_ISL_424258    1 ------------------------------------------------------------
EPI_ISL_424268    1 ------------------------------------------------------------
EPI_ISL_424269    1 ------------------------------------------------------------
EPI_ISL_424275    1 ------------------------------------------------------------
EPI_ISL_424276    1 ------------------------------------------------------------
EPI_ISL_424279    1 ------------------------------------------------------------

ORF1ab          481 SASTSAFVETVKGLDYKAFKQIVESCGNFKVTKGKAKKGAWNIGEQKSILSPLYAFASEA
EPI_ISL_424219    1 ------------------------------------------------------------
EPI_ISL_424222    1 ------------------------------------------------------------
EPI_ISL_424223    1 ------------------------------------------------------------
EPI_ISL_424226    1 ------------------------------------------------------------
EPI_ISL_424228    1 ------------------------------------------------------------
EPI_ISL_424230    1 ------------------------------------------------------------
EPI_ISL_424231    1 ------------------------------------------------------------
EPI_ISL_424240    1 ------------------------------------------------------------
EPI_ISL_424241    1 ------------------------------------------------------------
EPI_ISL_424243    1 ------------------------------------------------------------
EPI_ISL_424247    1 ------------------------------------------------------------
EPI_ISL_424249    1 ------------------------------------------------------------
EPI_ISL_424250    1 ------------------------------------------------------------
EPI_ISL_424252    1 ------------------------------------------------------------
EPI_ISL_424258    1 ------------------------------------------------------------
EPI_ISL_424268    1 ------------------------------------------------------------
EPI_ISL_424269    1 ------------------------------------------------------------
EPI_ISL_424275    1 ------------------------------------------------------------
EPI_ISL_424276    1 ------------------------------------------------------------
```

EPI_ISL_424279   1 --

```
ORF1ab           541 ARVVRSIFSRTLETAQNSVRVLQKAAITILDGISQYSLRLIDAMMFTSDLATNNLVVMAY
EPI_ISL_424219     1 ------------------------------------------------------------
EPI_ISL_424222     1 ------------------------------------------------------------
EPI_ISL_424223     1 ------------------------------------------------------------
EPI_ISL_424226     1 ------------------------------------------------------------
EPI_ISL_424228     1 ------------------------------------------------------------
EPI_ISL_424230     1 ------------------------------------------------------------
EPI_ISL_424231     1 ------------------------------------------------------------
EPI_ISL_424240     1 ------------------------------------------------------------
EPI_ISL_424241     1 ------------------------------------------------------------
EPI_ISL_424243     1 ------------------------------------------------------------
EPI_ISL_424247     1 ------------------------------------------------------------
EPI_ISL_424249     1 ------------------------------------------------------------
EPI_ISL_424250     1 ------------------------------------------------------------
EPI_ISL_424252     1 ------------------------------------------------------------
EPI_ISL_424258     1 ------------------------------------------------------------
EPI_ISL_424268     1 ------------------------------------------------------------
EPI_ISL_424269     1 ------------------------------------------------------------
EPI_ISL_424275     1 ------------------------------------------------------------
EPI_ISL_424276     1 ------------------------------------------------------------
EPI_ISL_424279     1 ------------------------------------------------------------

ORF1ab           601 ITGGVVQLTSQWLTNIFGTVYEKLKPVLDWLEEKFKEGVEFLRDGWEIVKFISTCACEIV
EPI_ISL_424219     1 ------------------------------------------------------------
EPI_ISL_424222     1 ------------------------------------------------------------
EPI_ISL_424223     1 ------------------------------------------------------------
EPI_ISL_424226     1 ------------------------------------------------------------
EPI_ISL_424228     1 ------------------------------------------------------------
EPI_ISL_424230     1 ------------------------------------------------------------
EPI_ISL_424231     1 ------------------------------------------------------------
EPI_ISL_424240     1 ------------------------------------------------------------
EPI_ISL_424241     1 ------------------------------------------------------------
EPI_ISL_424243     1 ------------------------------------------------------------
EPI_ISL_424247     1 ------------------------------------------------------------
EPI_ISL_424249     1 ------------------------------------------------------------
EPI_ISL_424250     1 ------------------------------------------------------------
EPI_ISL_424252     1 ------------------------------------------------------------
EPI_ISL_424258     1 ------------------------------------------------------------
EPI_ISL_424268     1 ------------------------------------------------------------
EPI_ISL_424269     1 ------------------------------------------------------------
EPI_ISL_424275     1 ------------------------------------------------------------
EPI_ISL_424276     1 ------------------------------------------------------------
EPI_ISL_424279     1 ------------------------------------------------------------

ORF1ab           661 GGQIVTCAKEIKESVQTFFKLVNKFLALCADSIIIGGAKLKALNLGETFVTHSKGLYRKC
EPI_ISL_424219     1 ------------------------------------------------------------
EPI_ISL_424222     1 ------------------------------------------------------------
EPI_ISL_424223     1 ------------------------------------------------------------
EPI_ISL_424226     1 ------------------------------------------------------------
EPI_ISL_424228     1 ------------------------------------------------------------
EPI_ISL_424230     1 ------------------------------------------------------------
EPI_ISL_424231     1 ------------------------------------------------------------
EPI_ISL_424240     1 ------------------------------------------------------------
EPI_ISL_424241     1 ------------------------------------------------------------
EPI_ISL_424243     1 ------------------------------------------------------------
EPI_ISL_424247     1 ------------------------------------------------------------
EPI_ISL_424249     1 ------------------------------------------------------------
EPI_ISL_424250     1 ------------------------------------------------------------
EPI_ISL_424252     1 ------------------------------------------------------------
EPI_ISL_424258     1 ------------------------------------------------------------
EPI_ISL_424268     1 ------------------------------------------------------------
EPI_ISL_424269     1 ------------------------------------------------------------
EPI_ISL_424275     1 ------------------------------------------------------------
EPI_ISL_424276     1 ------------------------------------------------------------
```

EPI_ISL_424279   1 --

```
ORF1ab          721 VKSREETGLLMPLKAPKEIIFLEGETLPTEVLTEEVVLKTGDLQPLEQPTSEAVEAPLVG
EPI_ISL_424219    1 ------------------------------------------------------------
EPI_ISL_424222    1 ------------------------------------------------------------
EPI_ISL_424223    1 ------------------------------------------------------------
EPI_ISL_424226    1 ------------------------------------------------------------
EPI_ISL_424228    1 ------------------------------------------------------------
EPI_ISL_424230    1 ------------------------------------------------------------
EPI_ISL_424231    1 ------------------------------------------------------------
EPI_ISL_424240    1 ------------------------------------------------------------
EPI_ISL_424241    1 ------------------------------------------------------------
EPI_ISL_424243    1 ------------------------------------------------------------
EPI_ISL_424247    1 ------------------------------------------------------------
EPI_ISL_424249    1 ------------------------------------------------------------
EPI_ISL_424250    1 ------------------------------------------------------------
EPI_ISL_424252    1 ------------------------------------------------------------
EPI_ISL_424258    1 ------------------------------------------------------------
EPI_ISL_424268    1 ------------------------------------------------------------
EPI_ISL_424269    1 ------------------------------------------------------------
EPI_ISL_424275    1 ------------------------------------------------------------
EPI_ISL_424276    1 ------------------------------------------------------------
EPI_ISL_424279    1 ------------------------------------------------------------

ORF1ab          781 TPVCINGLMLLEIKDTEKYCALAPNMMVTNNTFTLKGGAPTKVTFGDDTVIEVQGYKSVN
EPI_ISL_424219    1 --------------------------------------APTKVTFGDDTVIEVQGYKSVN
EPI_ISL_424222    1 --------------------------------------APTKVTFGDDTVIEVQGYKSVN
EPI_ISL_424223    1 --------------------------------------APTKVTFGDDTVIEVQGYKSVN
EPI_ISL_424226    1 --------------------------------------APTKVTFGDDTVIEVQGYKSVN
EPI_ISL_424228    1 --------------------------------------APTKVTFGDDTVIEVQGYKSVN
EPI_ISL_424230    1 --------------------------------------APTKVTFGDDTVIEVQGYKSVN
EPI_ISL_424231    1 --------------------------------------APTKVTFGDDTVIEVQGYKSVN
EPI_ISL_424240    1 --------------------------------------APTKVTFGDDTVIEVQGYKSVN
EPI_ISL_424241    1 --------------------------------------APTKVTFGDDTVIEVQGYKSVN
EPI_ISL_424243    1 --------------------------------------APTKVTFGDDTVIEVQGYKSVN
EPI_ISL_424247    1 --------------------------------------APTKVTFGDDTVIEVQGYKSVN
EPI_ISL_424249    1 --------------------------------------APTKVTFGDDTVIEVQGYKSVN
EPI_ISL_424250    1 --------------------------------------APTKVTFGDDTVIEVQGYKSVN
EPI_ISL_424252    1 --------------------------------------APTKVTFGDDTVIEVQGYKSVN
EPI_ISL_424258    1 --------------------------------------APTKVTFGDDTVIEVQGYKSVN
EPI_ISL_424268    1 --------------------------------------APTKVTFGDDTVIEVQGYKSVN
EPI_ISL_424269    1 --------------------------------------APTKVTFGDDTVIEVQGYKSVN
EPI_ISL_424275    1 --------------------------------------APTKVTFGDDTVIEVQGYKSVN
EPI_ISL_424276    1 --------------------------------------APTKVTFGDDTVIEVQGYKSVN
EPI_ISL_424279    1 --------------------------------------APTKVTFGDDTVIEVQGYKSVN

ORF1ab          841 ITFELDERIDKVLNEKCSAYTVELGTEVNEFACVVADAVIKTLQPVSELLTPLGIDLDEW
EPI_ISL_424219   23 ITFELDERIDKVLNEKCSAYTVELGTEVNEFACVVADAVIKTLQPVSELLTPLGIDLDEW
EPI_ISL_424222   23 ITFELDERIDKVLNEKCSAYTVELGTEVNEFACVVADAVIKTLQPVSELLTPLGIDLDEW
EPI_ISL_424223   23 ITFELDERIDKVLNEKCSAYTVELGTEVNEFACVVADAVIKTLQPVSELLTPLGIGLDEW
EPI_ISL_424226   23 ITFELDERIDKVLNEKCSAYTVELGTEVNEFACVVADAVIKTLQPVSELLTPLGIDLDEW
EPI_ISL_424228   23 ITFELDERIDKVLNEKCSAYTVELGTEVNEFACVVADAVIKTLQPVSELLTPLGIDLDEW
EPI_ISL_424230   23 ITFELDERIDKVLNEKCSAYTVELGTEVNEFACVVADAVIKTLQPVSELLTPLGIDLDEW
EPI_ISL_424231   23 ITFELDERIDKVLNEKCSAYTVELGTEVNEFACVVADAVIKTLQPVSELLTPLGIDLDEW
EPI_ISL_424240   23 ITFELDERIDKVLNEKCSAYTVELGTEVNEFACVVADAVIKTLQPVSELLTPLGIDLDEW
EPI_ISL_424241   23 ITFELDERIDKVLNEKCSAYTVELGTEVNEFACVVADAVIKTLQPVSELLTPLGIDLDEW
EPI_ISL_424243   23 ITFELDERIDKVLNEKCSAYTVELGTEVNEFACVVADAVIKTLQPVSELLTPLGIDLDEW
EPI_ISL_424247   23 ITFELDERIDKVLNEKCSAYTVELGTEVNEFACVVADAVIKTLQPVSELLTPLGIDLDEW
EPI_ISL_424249   23 ITFELDERIDKVLNEKCSAYTVELGTEVNEFACVVADAVIKTLQPVSELLTPLGIDLDEW
EPI_ISL_424250   23 ITFELDERIDKVLNEKCSAYTVELGTEVNEFACVVADAVIKTLQPVSELLTPLGIDLDEW
EPI_ISL_424252   23 ITFELDERIDKVLNEKCSAYTVELGTEVNEFACVVADAVIKTLQPVSELLTPLGIDLDEW
EPI_ISL_424258   23 ITFELDERIDKVLNEKCSAYTVELGTEVNEFACVVADAVIKTLQPVSELLTPLGIDLDEW
EPI_ISL_424268   23 ITFELDERIDKVLNEKCSAYTVELGTEVNEFACVVADAVIKTLQPVSELLTPLGIDLDEW
EPI_ISL_424269   23 ITFELDERIDKVLNEKCSAYTVELGTEVNEFACVVADAVIKTLQPVSELLTPLGIDLDEW
EPI_ISL_424275   23 ITFELDERIDKVLNEKCSAYTVELGTEVNEFACVVADAVIKTLQPVSELLTPLGIDLDEW
EPI_ISL_424276   23 ITFELDERIDKVLNEKCSAYTVELGTEVNEFACVVADAVIKTLQPVSELLTPLGIDLDEW
```

EPI_ISL_424279 23 ITFELDERIDKVLNEKCSAYTVELGTEVNEFACVVADAVIKTLQPVSELLTPLGIDLDEW

```
ORF1ab            901  SMATYYLFDESGEFKLASHMYCSFYPPDEDEEEGDCEEEEFEPSTQYEYGTEDDYQGKPL
EPI_ISL_424219     83  SMATYYLFDESGEFKLASHMYCSFYPPDEDEEEGDCEEEEFEPSTQYEYGTEDDYQGKPL
EPI_ISL_424222     83  SMATYYLFDESGEFKLASHMYCSFYPPDEDEEEGDCEEEEFEPSTQYEYGTEDDYQGKPL
EPI_ISL_424223     83  SMATYYLFDESGEFKLASHMYCSFYPPDEDEEEGDCEEEEFEPSTQYEYGTEDDYQGKPL
EPI_ISL_424226     83  SMATYYLFDESGEFKLASHMYCSFYPPDEDEEEGDCEEEEFEPSTQYEYGTEDDYQGKPL
EPI_ISL_424228     83  SMATYYLFDESGEFKLASHMYCSFYPPDEDEEEGDCEEEEFEPSTQYEYGTEDDYQGKPL
EPI_ISL_424230     83  SMATYYLFDESGEFKLASHMYCSFYPPDEDEEEGDCEEEEFEPSTQYEYGTEDDYQGKPL
EPI_ISL_424231     83  SMATYYLFDESGEFKLASHMYCSFYPPDEDEEEGDCEEEEFEPSTQYEYGTEDDYQGKPL
EPI_ISL_424240     83  SMATYYLFDESGEFKLASHMYCSFYPPDEDEEEGDCEEEEFEPSTQYEYGTEDDYQGKPL
EPI_ISL_424241     83  SMATYYLFDESGEFKLASHMYCSFYPPDEDEEEGDCEEEEFEPSTQYEYGTEDDYQGKPL
EPI_ISL_424243     83  SMATYYLFDESGEFKLASHMYCSFYPPDEDEEEGDCEEEEFEPSTQYEYGTEDDYQGKPL
EPI_ISL_424247     83  SMATYYLFDESGEFKLASHMYCSFYPPDEDEEEGDCEEEEFEPSTQYEYGTEDDYQGKPL
EPI_ISL_424249     83  SMATYYLFDESGEFKLASHMYCSFYPPDEDEEEGDCEEEEFEPSTQYEYGTEDDYQGKPL
EPI_ISL_424250     83  SMATYYLFDESGEFKLASHMYCSFYPPDEDEEEGDCEEEEFEPSTQYEYGTEDDYQGKPL
EPI_ISL_424252     83  SMATYYLFDESGEFKLASHMYCSFYPPDEDEEEGDCEEEEFEPSTQYEYGTEDDYQGKPL
EPI_ISL_424258     83  SMATYYLFDESGEFKLASHMYCSFYPPDEDEEEGDCEEEEFEPSTQYEYGTEDDYQGKPL
EPI_ISL_424268     83  SMATYYLFDESGEFKLASHMYCSFYPPDEDEEEGDCEEEEFEPSTQYEYGTEDDYQGKPL
EPI_ISL_424269     83  SMATYYLFDESGEFKLASHMYCSFYPPDEDEEEGDCEEEEFEPSTQYEYGTEDDYQGKPL
EPI_ISL_424275     83  SMATYYLFDESGEFKLASHMYCSFYPPDEDEEEGDCEEEEFEPSTQYEYGTEDDYQGKPL
EPI_ISL_424276     83  SMATYYLFDESGEFKLASHMYCSFYPPDEDEEEGDCEEEEFEPSTQYEYGTEDDYQGKPL
EPI_ISL_424279     83  SMATYYLFDESGEFKLASHMYCSFYPPDEDEEEGDCEEEEFEPSTQYEYGTEDDYQGKPL
```

```
ORF1ab            961  EFGATSAALQPEEEQEEDWLDDDSQQTVGQQDGSEDNQTTTIQTIVEVQPQLEMELTPVV
EPI_ISL_424219    143  EFGATSAALQPEEEQEEDWLDDDSQQTVGQQDGSEDNQTTTIQTIVEVQPQLEMELTPVV
EPI_ISL_424222    143  EFGATSAALQPEEEQEEDWLDDDSQQTVGQQDGSEDNQTTTIQTIVEVQPQLEMELTPVV
EPI_ISL_424223    143  EFGATSAALQPEEEQEEDWLDDDSQQTVGQQDGSEDNQTTTIQTIVEVQPQLEMELTPVV
EPI_ISL_424226    143  EFGATSAALQPEEEQEEDWLDDDSQQTVVQQDGSEDNQTTTIQTIVEVQPQLEMELTPVV
EPI_ISL_424228    143  EFGATSAALQPEEEQEEDWLDDDSQQTVVQQDGSEDNQTTTIQTIVEVQPQLEMELTPVV
EPI_ISL_424230    143  EFGATSAALQPEEEQEEDWLDDDSQQTVGQQDGSEDNQTTTIQTIVEVQPQLEMELTPVV
EPI_ISL_424231    143  EFGATSAALQPEEEQEEDWLDDDSQQTVGQQDGSEDNQTTTIQTIVEVQPQLEMELTPVV
EPI_ISL_424240    143  EFGATSAALQPEEEQEEDWLDDDSQQTVGQQDGSEDNQTTTIQTIVEVQPQLEMELTPVV
EPI_ISL_424241    143  EFGATSAALQPEEEQEEDWLDDDSQQTVGQQDGSEDNQTTTIQTIVEVQPQLEMELTPVV
EPI_ISL_424243    143  EFGATSAALQPEEEQEEDWLDDDSQQTVGQQDGSEDNQTTTIQTIVEVQPQLEMELTPVV
EPI_ISL_424247    143  EFGATSAALQPEEEQEEDWLDDDSQQTVGQQDGSEDNQTTTIQTIVEVQPQLEMELTPVV
EPI_ISL_424249    143  EFGATSAALQPEEEQEEDWLDDDSQQTVGQQDGSEDNQTTTIQTIVEVQPQLEMELTPVV
EPI_ISL_424250    143  EFGATSAALQPEEEQEEDWLDDDSQQTVGQQDGSEDNQTTTIQTIVEVQPQLEMELTPVV
EPI_ISL_424252    143  EFGATSAALQPEEEQEEDWLDDDSQQTVGQQDGSEDNQTTTIQTIVEVQPQLEMELTPVV
EPI_ISL_424258    143  EFGATSAALQPEEEQEEDWLDDDSQQTVGQQDGSEDNQTTTIQTIVEVQPQLEMELTPVV
EPI_ISL_424268    143  EFGATSAALQPEEEQEEDWLDDDSQQTVGQQDGSEDNQTTTIQTIVEVQPQLEMELTPVV
EPI_ISL_424269    143  EFGATSAALQPEEEQEEDWLDDDSQQTVGQQDGSEDNQTTTIQTIVEVQPQLEMELTPVV
EPI_ISL_424275    143  EFGATSAALQPEEEQEEDWLDDDSQQTVGQQDGSEDNQTTTIQTIVEVQPQLEMELTPVV
EPI_ISL_424276    143  EFGATSAALQPEEEQEEDWLDDDSQQTVGQQDGSEDNQTTTIQTIVEVQPQLEMELTPVV
EPI_ISL_424279    143  EFGATSAALQPEEEQEEDWLDDDSQQTVGQQDGSEDNQTTTIQTIVEVQPQLEMELTPVV
```

```
ORF1ab           1021  QTIEVNSFSGYLKLTDNVYIKNADIVEEAKKVKPTVVVNAANVYLKHGGGVAGALNKATN
EPI_ISL_424219    203  QTIEVNSFSGYLKLTDNVYIKNADIVEEAKKVKPTVVVNAANVYLKHGGGVAGALNKATN
EPI_ISL_424222    203  QTIEVNSFSGYLKLTDNVYIKNADIVEEAKKVKPTVVVNAANVYLKHGGGVAGALNKATN
EPI_ISL_424223    203  QTIEVNSFSGYLKLTDNVYIKNADIVEEAKKVKPTVVVNAANVYLKHGGGVAGALNKATN
EPI_ISL_424226    203  QTIEVNSFSGYLKLTDNVYIKNADIVEEAKKVKPTVVVNAANVYLKHGGGVAGALNKATN
EPI_ISL_424228    203  QTIEVNSFSGYLKLTDNVYIKNADIVEEAKKVKPTVVVNAANVYLKHGGGVAGALNKATN
EPI_ISL_424230    203  QTIEVNSFSGYLKLTDNVYIKNADIVEEAKKVKPTVVVNAANVYLKHGGGVAGALNKATN
EPI_ISL_424231    203  QTIEVNSFSGYLKLTDNVYIKNADIVEEAKKVKPTVVVNAANVYLKHGGGVAGALNKATN
EPI_ISL_424240    203  QTIEVNSFSGYLKLTDNVYIKNADIVEEAKKVKPTVVVNAANVYLKHGGGVAGALNKATN
EPI_ISL_424241    203  QTIEVNSFSGYLKLTDNVYIKNADIVEEAKKVKPTVVVNAANVYLKHGGGVAGALNKATN
EPI_ISL_424243    203  QTIEVNSFSGYLKLTDNVYIKNADIVEEAKKVKPTVVVNAANVYLKHGGGVAGALNKATN
EPI_ISL_424247    203  QTIEVNSFSGYLKLTDNVYIKNADIVEEAKKVKPTVVVNAANVYLKHGGGVAGALNKATN
EPI_ISL_424249    203  QTIEVNSFSGYLKLTDNVYIKNADIVEEAKKVKPTVVVNAANVYLKHGGGVAGALNKATN
EPI_ISL_424250    203  QTIEVNSFSGYLKLTDNVYIKNADIVEEAKKVKPTVVVNAANVYLKHGGGVAGALNKATN
EPI_ISL_424252    203  QTIEVNSFSGYLKLTDNVYIKNADIVEEAKKVKPTVVVNAANVYLKHGGGVAGALNKATN
EPI_ISL_424258    203  QTIEVNSFSGYLKLTDNVYIKNADIVEEAKKVKPTVVVNAANVYLKHGGGVAGALNKATN
EPI_ISL_424268    203  QTIEVNSFSGYLKLTDNVYIKNADIVEEAKKVKPTVVVNAANVYLKHGGGVAGALNKATN
EPI_ISL_424269    203  QTIEVNSFSGYLKLTDNVYIKNADIVEEAKKVKPTVVVNAANVYLKHGGGVAGALNKATN
EPI_ISL_424275    203  QTIEVNSFSGYLKLTDNVYIKNADIVEEAKKVKPTVVVNAANVYLKHGGGVAGALNKATN
EPI_ISL_424276    203  QTIEVNSFSGYLKLTDNVYIKNADIVEEAKKVKPTVVVNAANVYLKHGGGVAGALNKATN
```

EPI_ISL_424279 203 QTIEVNSFSGYLKLTDNVYIKNADIVEEAKKVKPTVVVNAANVYLKHGGGVAGALNKATN

```
ORF1ab          1081 NAMQVESDDYIATNGPLKVGGSCVLSGHNLAKHCLHVVGPNVNKGEDIQLLKSAYENFNQ
EPI_ISL_424219   263 NAMQVESDDYIATNGPLKVGGSCVLSGHNLAKHCLHVVGPNVNKGEDIQLLKSAYENFNQ
EPI_ISL_424222   263 NAMQVESDDYIATNGPLKVGGSCVLSGHNLAKHCLHVVGPNVNKGEDIQLLKSAYENFNQ
EPI_ISL_424223   263 NAMQVESDDYIATNGPLKVGGSCVLSGHNLAKHCLHVVGPNVNKGEDIQLLKSAYENFNQ
EPI_ISL_424226   263 NAMQVESDDYIATNGPLKVGGSCVLSGHNLAKHCLHVVGPNVNKGEDIQLLKSAYENFNQ
EPI_ISL_424228   263 NAMQVESDDYIATNGPLKVGGSCVLSGHNLAKHCLHVVGPNVNKGEDIQLLKSAYENFNQ
EPI_ISL_424230   263 NAMQVESDDYIATNGPLKVGGSCVLSGHNLAKHCLHVVGPNVNKGEDIQLLKSAYENFNQ
EPI_ISL_424231   263 NAMQVESDDYIATNGPLKVGGSCVLSGHNLAKHCLHVVGPNVNKGEDIQLLKSAYENFNQ
EPI_ISL_424240   263 NAMQVESDDYIATNGPLKVGGSCVLSGHNLAKHCLHVVGPNVNKGEDIQLLKSAYENFNQ
EPI_ISL_424241   263 NAMQVESDDYIATNGPLKVGGSCVLSGHNLAKHCLHVVGPNVNKGEDIQLLKSAYENFNQ
EPI_ISL_424243   263 NAMQVESDDYIATNGPLKVGGSCVLSGHNLAKHCLHVVGPNVNKGEDIQLLKSAYENFNQ
EPI_ISL_424247   263 NAMQVESDDYIATNGPLKVGGSCVLSGHNLAKHCLHVVGPNVNKGEDIQLLKSAYENFNQ
EPI_ISL_424249   263 NAMQVESDDYIATNGPLKVGGSCVLSGHNLAKHCLHVVGPNVNKGEDIQLLKSAYENFNQ
EPI_ISL_424250   263 NAMQVESDDYIATNGPLKVGGSCVLSGHNLAKHCLHVVGPNVNKGEDIQLLKSAYENFNQ
EPI_ISL_424252   263 NAMQVESDDYIATNGPLKVGGSCVLSGHNLAKHCLHVVGPNVNKGEDIQLLKSAYENFNQ
EPI_ISL_424258   263 NAMQVESDDYIATNGPLKVGGSCVLSGHNLAKHCLHVVGPNVNKGEDIQLLKSAYENFNQ
EPI_ISL_424268   263 NAMQVESDDYIATNGPLKVGGSCVLSGHNLAKHCLHVVGPNVNKGEDIQLLKSAYENFNQ
EPI_ISL_424269   263 NAMQVESDDYIATNGPLKVGGSCVLSGHNLAKHCLHVVGPNVNKGEDIQLLKSAYENFNQ
EPI_ISL_424275   263 NAMQVESDDYIATNGPLKVGGSCVLSGHNLAKHCLHVVGPNVNKGEDIQLLKSAYENFNQ
EPI_ISL_424276   263 NAMQVESDDYIATNGPLKVGGSCVLSGHNLAKHCLHVVGPNVNKGEDIQLLKSAYENFNQ
EPI_ISL_424279   263 NAMQVESDDYIATNGPLKVGGSCVLSGHNLAKHCLHVVGPNVNKGEDIQLLKSAYENFNQ

ORF1ab          1141 HEVLLAPLLSAGIFGADPIHSLRVCVDTVRTNVYLAVFDKNLYDKLVSSFLEMKSEKQVE
EPI_ISL_424219   323 HEVLLAPLLSAGIFGADPIHSLRVCVDTVRTNVYLAVFDKNLYDKLVSSFLEMKSEKQVE
EPI_ISL_424222   323 HEVLLAPLLSAGIFGADPIHSLRVCVDTVRTNVYLAVFDKNLYDKLVSSFLEMKSEKQVE
EPI_ISL_424223   323 HEVLLAPLLSAGIFGADPIHSLRVCVDTVRTNVYLAVFDKNLYDKLVSSFLEMKSEKQVE
EPI_ISL_424226   323 HEVLLAPLLSAGIFGADPIHSLRVCVDTVRTNVYLAVFDKNLYDKLVSSFLEMKSEKQVE
EPI_ISL_424228   323 HEVLLAPLLSAGIFGADPIHSLRVCVDTVRTNVYLAVFDKNLYDKLVSSFLEMKSEKQVE
EPI_ISL_424230   323 HEVLLAPLLSAGIFGADPIHSLRVCVDTVRTNVYLAVFDKNLYDKLVSSFLEMKSEKQVE
EPI_ISL_424231   323 HEVLLAPLLSAGIFGADPIHSLRVCVDTVRTNVYLAVFDKNLYDKLVSSFLEMKSEKQVE
EPI_ISL_424240   323 HEVLLAPLLSAGIFGADPIHSLRVCVDTVRTNVYLAVFDKNLYDKLVSSFLEMKSEKQVE
EPI_ISL_424241   323 HEVLLAPLLSAGIFGADPIHSLRVCVDTVRTNVYLAVFDKNLYDKLVSSFLEMKSEKQVE
EPI_ISL_424243   323 HEVLLAPLLSAGIFGADPIHSLRVCVDTVRTNVYLAVFDKNLYDKLVSSFLEMKSEKQVE
EPI_ISL_424247   323 HEVLLAPLLSAGIFGADPIHSLRVCVDTVRTNVYLAVFDKNLYDKLVSSFLEMKSEKQVE
EPI_ISL_424249   323 HEVLLAPLLSAGIFGADPIHSLRVCVDTVRTNVYLAVFDKNLYDKLVSSFLEMKSEKQVE
EPI_ISL_424250   323 HEVLLAPLLSAGIFGADPIHSLRVCVDTVRTNVYLAVFDKNLYDKLVSSFLEMKSEKQVE
EPI_ISL_424252   323 HEVLLAPLLSAGIFGADPIHSLRVCVDTVRTNVYLAVFDKNLYDKLVSSFLEMKSEKQVE
EPI_ISL_424258   323 HEVLLAPLLSAGIFGADPIHSLRVCVDTVRTNVYLAVFDKNLYDKLVSSFLEMKSEKQVE
EPI_ISL_424268   323 HEVLLAPLLSAGIFGADPIHSLRVCVDTVRTNVYLAVFDKNLYDKLVSSFLEMKSEKQVE
EPI_ISL_424269   323 HEVLLAPLLSAGIFGADPIHSLRVCVDTVRTNVYLAVFDKNLYDKLVSSFLEMKSEKQVE
EPI_ISL_424275   323 HEVLLAPLLSAGIFGADPIHSLRVCVDTVRTNVYLAVFDKNLYDKLVSSFLEMKSEKQVE
EPI_ISL_424276   323 HEVLLAPLLSAGIFGADPIHSLRVCVDTVRTNVYLAVFDKNLYDKLVSSFLEMKSEKQVE
EPI_ISL_424279   323 HEVLLAPLLSAGIFGADPIHSLRVCVDTVRTNVYLAVFDKNLYDKLVSSFLEMKSEKQVE

ORF1ab          1201 QKIAEIPKEEVKPFITESKPSVEQRKQDDKKIKACVEEVTTTLEETKFLTENLLLYIDIN
EPI_ISL_424219   383 QKIAEIPKEEVKPFITESKPSVEQRKQDDKKIKACVEEVTTTLEETKFLTENLLLYIDIN
EPI_ISL_424222   383 QKIAEIPKEEVKPFITESKPSVEQRKQDDKKIKACVEEVTTTLEETKFLTENLLLYIDIN
EPI_ISL_424223   383 QKIAEIPKEEVKPFITESKPSVEQRKQDDKKIKACVEEVTTTLEETKFLTENLLLYIDIN
EPI_ISL_424226   383 QKIAEIPKEEVKPFITESKPSVEQRKQDDKKIKACVEEVTTTLEETKFLTENLLLYIDIN
EPI_ISL_424228   383 QKIAEIPKEEVKPFITESKPSVEQRKQDDKKIKACVEEVTTTLEETKFLTENLLLYIDIN
EPI_ISL_424230   383 QKIAEIPKEEVKPFITESKPSVEQRKQDDKKIKACVEEVTTTLEETKFLTENLLLYIDIN
EPI_ISL_424231   383 QKIAEIPKEEVKPFITESKPSVEQRKQDDKKIKACVEEVTTTLEETKFLTENLLLYIDIN
EPI_ISL_424240   383 QKIAEIPKEEVKPFITESKPSVEQRKQDDKKIKACVEEVTTTLEETKFLTENLLLYIDIN
EPI_ISL_424241   383 QKIAEIPKEEVKPFITESKPSVEQRKQDDKKIKACVEEVTTTLEETKFLTENLLLYIDIN
EPI_ISL_424243   383 QKIAEIPKEEVKPFITESKPSVEQRKQDDKKIKACVEEVTTTLEETKFLTENLLLYIDIN
EPI_ISL_424247   383 QKIAEIPKEEVKPFITESKPSVEQRKQDDKKIKACVEEVTTTLEETKFLTENLLLYIDIN
EPI_ISL_424249   383 QKIAEIPKEEVKPFITESKPSVEQRKQDDKKIKACVEEVTTTLEETKFLTENLLLYIDIN
EPI_ISL_424250   383 QKIAEIPKEEVKPFITESKPSVEQRKQDDKKIKACVEEVTTTLEETKFLTENLLLYIDIN
EPI_ISL_424252   383 QKIAEIPKEEVKPFITESKPSVEQRKQDDKKIKACVEEVTTTLEETKFLTENLLLYIDIN
EPI_ISL_424258   383 QKIAEIPKEEVKPFITESKPSVEQRKQDDKKIKACVEEVTTTLEETKFLTENLLLYIDIN
EPI_ISL_424268   383 QKIAEIPKEEVKPFITESKPSVEQRKQDDKKIKACVEEVTTTLEETKFLTENLLLYIDIN
EPI_ISL_424269   383 QKIAEIPKEEVKPFITESKPSVEQRKQDDKKIKACVEEVTTTLEETKFLTENLLLYIDIN
EPI_ISL_424275   383 QKIAEIPKEEVKPFITESKPSVEQRKQDDKKIKACVEEVTTTLEETKFLTENLLLYIDIN
EPI_ISL_424276   383 QKIAEIPKEEVKPFITESKPSVEQRKQDDKKIKACVEEVTTTLEETKFLTENLLLYIDIN
```

EPI_ISL_424279 383 QKIAEIPKEEVKPFITESKPSVEQRKQDDKKIKACVEEVTTTLEETKFLTENLLLYIDIN

```
ORF1ab          1261 GNLHPDSATLVSDIDITFLKKDAPYIVGDVVQEGVLTAVVIPTKKAGGTTEMLAKALRKV
EPI_ISL_424219   443 GNLHPDSATLVSDIDITFLKKDAPYIVGDVVQEGVLTAVVIPTKKAGGTTEMLAKALRKV
EPI_ISL_424222   443 GNLHPDSATLVSDIDITFLKKDAPYIVGDVVQEGVLTAVVIPTKKAGGTTEMLAKALRKV
EPI_ISL_424223   443 GNLHPDSATLVSDIDITFLKKDAPYIVGDVVQEGVLTAVVIPTKKAGGTTEMLAKALRKV
EPI_ISL_424226   443 GNLHPDSATLVSDIDITFLKKDAPYIVGDVVQEGVLTAVVIPTKKAGGTTEMLAKALRKV
EPI_ISL_424228   443 GNLHPDSATLVSDIDITFLKKDAPYIVGDVVQEGVLTAVVIPTKKAGGTTEMLAKALRKV
EPI_ISL_424230   443 GNLHPDSATLVSDIDITFLKKDAPYIVGDVVQEGVLTAVVIPTKKAGGTTEMLAKALRKV
EPI_ISL_424231   443 GNLHPDSATLVSDIDITFLKKDAPYIVGDVVQEGVLTAVVIPTKKAGGTTEMLAKALRKV
EPI_ISL_424240   443 GNLHPDSATLVSDIDITFLKKDAPYIVGDVVQEGVLTAVVIPTKKAGGTTEMLAKALRKV
EPI_ISL_424241   443 GNLHPDSATLVSDIDITFLKKDAPYIVGDVVQEGVLTAVVIPTKKAGGTTEMLAKALRKV
EPI_ISL_424243   443 GNLHPDSATLVSDIDITFLKKDAPYIVGDVVQEGVLTAVVIPTKKAGGTTEMLAKALRKV
EPI_ISL_424247   443 GNLHPDSATLVSDIDITFLKKDAPYIVGDVVQEGVLTAVVIPTKKAGGTTEMLAKALRKV
EPI_ISL_424249   443 GNLHPDSATLVSDIDITFLKKDAPYIVGDVVQEGVLTAVVIPTKKAGGTTEMLAKALRKV
EPI_ISL_424250   443 GNLHPDSATLVSDIDITFLKKDAPYIVGDVVQEGVLTAVVIPTKKAGGTTEMLAKALRKV
EPI_ISL_424252   443 GNLHPDSATLVSDIDITFLKKDAPYIVGDVVQEGVLTAVVIPTKKAGGTTEMLAKALRKV
EPI_ISL_424258   443 GNLHPDSATLVSDIDITFLKKDAPYIVGDVVQEGVLTAVVIPTKKAGGTTEMLAKALRKV
EPI_ISL_424268   443 GNLHPDSATLVSDIDITFLKKDAPYIVGDVVQEGVLTAVVIPTKKAGGTTEMLAKALRKV
EPI_ISL_424269   443 GNLHPDSATLVSDIDITFLKKDAPYIVGDVVQEGVLTAVVIPTKKAGGTTEMLAKALRKV
EPI_ISL_424275   443 GNLHPDSATLVSDIDITFLKKDAPYIVGDVVQEGVLTAVVIPTKKAGGTTEMLAKALRKV
EPI_ISL_424276   443 GNLHPDSATLVSDIDITFLKKDAPYIVGDVVQEGVLTAVVIPTKKAGGTTEMLAKALRKV
EPI_ISL_424279   443 GNLHPDSATLVSDIDITFLKKDAPYIVGDVVQEGVLTAVVIPTKKAGGTTEMLAKALRKV

ORF1ab          1321 PTDNYITTYPGQGLNGYTVEEAKTVLKKCKSAFYILPSIISNEKQEILGTVSWNLREMLA
EPI_ISL_424219   503 PTDNYITTYPGQGLNGYTVEEAKTVLKKCKSAFYILPSIISNEKQEILGTVSWNLREMLA
EPI_ISL_424222   503 PTDNYITTYPGQGLNGYTVEEAKTVLKKCKSAFYILPSIISNEKQEILGTVSWNLREMLA
EPI_ISL_424223   503 PTDNYITTYPGQGLNGYTVEEAKTVLKKCKSAFYILPSIISNEKQEILGTVSWNLREMLA
EPI_ISL_424226   503 PTDNYITTYPGQGLNGYTVEEAKTVLKKCKSAFYILPSIISNEKQEILGTVSWNLREMLA
EPI_ISL_424228   503 PTDNYITTYPGQGLNGYTVEEAKTVLKKCKSAFYILPSIISNEKQEILGTVSWNLREMLA
EPI_ISL_424230   503 PTDNYITTYPGQGLNGYTVEEAKTVLKKCKSAFYILPSIISNEKQEILGTVSWNLREMLA
EPI_ISL_424231   503 PTDNYITTYPGQGLNGYTVEEAKTVLKKCKSAFYILPSIISNEKQEILGTVSWNLREMLA
EPI_ISL_424240   503 PTDNYITTYPGQGLNGYTVEEAKTVLKKCKSAFYILPSIISNEKQEILGTVSWNLREMLA
EPI_ISL_424241   503 PTDNYITTYPGQGLNGYTVEEAKTVLKKCKSAFYILPSIISNEKQEILGTVSWNLREMLA
EPI_ISL_424243   503 PTDNYITTYPGQGLNGYTVEEAKTVLKKCKSAFYILPSIISNEKQEILGTVSWNLREMLA
EPI_ISL_424247   503 PTDNYITTYPGQGLNGYTVEEAKTVLKKCKSAFYILPSIISNEKQEILGTVSWNLREMLA
EPI_ISL_424249   503 PTDNYITTYPGQGLNGYTVEEAKTVLKKCKSAFYILPSIISNEKQEILGTVSWNLREMLA
EPI_ISL_424250   503 PTDNYITTYPGQGLNGYTVEEAKTVLKKCKSAFYILPSIISNEKQEILGTVSWNLREMLA
EPI_ISL_424252   503 PTDNYITTYPGQGLNGYTVEEAKTVLKKCKSAFYILPSIISNEKQEILGTVSWNLREMLA
EPI_ISL_424258   503 PTDNYITTYPGQGLNGYTVEEAKTVLKKCKSAFYILPSIISNEKQEILGTVSWNLREMLA
EPI_ISL_424268   503 PTDNYITTYPGQGLNGYTVEEAKTVLKKCKSAFYILPSIISNEKQEILGTVSWNLREMLA
EPI_ISL_424269   503 PTDNYITTYPGQGLNGYTVEEAKTVLKKCKSAFYILPSIISNEKQEILGTVSWNLREMLA
EPI_ISL_424275   503 PTDNYITTYPGQGLNGYTVEEAKTVLKKCKSAFYILPSIISNEKQEILGTVSWNLREMLA
EPI_ISL_424276   503 PTDNYITTYPGQGLNGYTVEEAKTVLKKCKSAFYILPSIISNEKQEILGTVSWNLREMLA
EPI_ISL_424279   503 PTDNYITTYPGQGLNGYTVEEAKTVLKKCKSAFYILPSIISNEKQEILGTVSWNLREMLA

ORF1ab          1381 HAEETRKLMPVCVETKAIVSTIQRKYKGIKIQEGVVDYGARFYFYTSKTTVASLINTLND
EPI_ISL_424219   563 HAEETRKLMPVCVETKAIVSTIQRKYKGIKIQEGVVDYGARFYFYTSKTTVASLINTLND
EPI_ISL_424222   563 HAEETRKLMPVCVETKAIVSTIQRKYKGIKIQEGVVDYGARFYFYTSKTTVASLINTLND
EPI_ISL_424223   563 HAEETRKLMPVCVETKAIVSTIQRKYKGIKIQEGVVDYGARFYFYTSKTTVASLINTLND
EPI_ISL_424226   563 HAEETRKLMPVCVETKAIVSTIQRKYKGIKIQEGVVDYGARFYFYTSKTTVASLINTLND
EPI_ISL_424228   563 HAEETRKLMPVCVETKAIVSTIQRKYKGIKIQEGVVDYGARFYFYTSKTTVASLINTLND
EPI_ISL_424230   563 HAEETRKLMPVCVETKAIVSTIQRKYKGIKIQEGVVDYGARFYFYTSKTTVASLINTLND
EPI_ISL_424231   563 HAEETRKLMPVCVETKAIVSTIQRKYKGIKIQEGVVDYGARFYFYTSKTTVASLINTLND
EPI_ISL_424240   563 HAEETRKLMPVCVETKAIVSTIQRKYKGIKIQEGVVDYGARFYFYTSKTTVASLINTLND
EPI_ISL_424241   563 HAEETRKLMPVCVETKAIVSTIQRKYKGIKIQEGVVDYGARFYFYTSKTTVASLINTLND
EPI_ISL_424243   563 HAEETRKLMPVCVETKAIVSTIQRKYKGIKIQEGVVDYGARFYFYTSKTTVASLINTLND
EPI_ISL_424247   563 HAEETRKLMPVCVETKAIVSTIQRKYKGIKIQEGVVDYGARFYFYTSKTTVASLINTLND
EPI_ISL_424249   563 HAEETRKLMPVCVETKAIVSTIQRKYKGIKIQEGVVDYGARFYFYTSKTTVASLINTLND
EPI_ISL_424250   563 HAEETRKLMPVCVETKAIVSTIQRKYKGIKIQEGVVDYGARFYFYTSKTTVASLINTLND
EPI_ISL_424252   563 HAEETRKLMPVCVETKAIVSTIQRKYKGIKIQEGVVDYGARFYFYTSKTTVASLINTLND
EPI_ISL_424258   563 HAEETRKLMPVCVETKAIVSTIQRKYKGIKIQEGVVDYGARFYFYTSKTTVASLINTLND
EPI_ISL_424268   563 HAEETRKLMPVCVETKAIVSTIQRKYKGIKIQEGVVDYGARFYFYTSKTTVASLINTLND
EPI_ISL_424269   563 HAEETRKLMPVCVETKAIVSTIQRKYKGIKIQEGVVDYGARFYFYTSKTTVASLINTLND
EPI_ISL_424275   563 HAEETRKLMPVCVETKAIVSTIQRKYKGIKIQEGVVDYGARFYFYTSKTTVASLINTLND
EPI_ISL_424276   563 HAEETRKLMPVCVETKAIVSTIQRKYKGIKIQEGVVDYGARFYFYTSKTTVASLINTLND
```

EPI_ISL_424279 563 HAEETRKLMPVCVETKAIVSTIQRKYKGIKIQEGVVDYGARFYFYTSKTTVASLINTLND

```
ORF1ab          1441 LNETLVTMPLGYVTHGLNLEEAARYMRSLKVPATVSVSSPDAVTAYNGYLTSSSKTPEEH
EPI_ISL_424219   623 LNETLVTMPLGYVTHGLNLEEAARYMRSLKVPATVSVSSPDAVTAYNGYLTSSSKTPEEH
EPI_ISL_424222   623 LNETLVTMPLGYVTHGLNLEEAARYMRSLKVPATVSVSSPDAVTAYNGYLTSSSKTPEEH
EPI_ISL_424223   623 LNETLVTMPLGYVTHGLNLEEAARYMRSLKVPATVSVSSPDAVTAYNGYLTSSSKTPEEH
EPI_ISL_424226   623 LNETLVTMPLGYVTHGLNLEEAARYMRSLKVPATVSVSSPDAVTAYNGYLTSSSKTPEEH
EPI_ISL_424228   623 LNETLVTMPLGYVTHGLNLEEAARYMRSLKVPATVSVSSPDAVTAYNGYLTSSSKTPEEH
EPI_ISL_424230   623 LNETLVTMPLGYVTHGLNLEEAARYMRSLKVPATVSVSSPDAVTAYNGYLTSSSKTPEEH
EPI_ISL_424231   623 LNETLVTMPLGYVTHGLNLEEAARYMRSLKVPATVSVSSPDAVTAYNGYLTSSSKTPEEH
EPI_ISL_424240   623 LNETLVTMPLGYVTHGLNLEEAARYMRSLKVPATVSVSSPDAVTAYNGYLTSSSKTPEEH
EPI_ISL_424241   623 LNETLVTMPLGYVTHGLNLEEAARYMRSLKVPATVSVSSPDAVTAYNGYLTSSSKTPEEH
EPI_ISL_424243   623 LNETLVTMPLGYVTHGLNLEEAARYMRSLKVPATVSVSSPDAVTAYNGYLTSSSKTPEEH
EPI_ISL_424247   623 LNETLVTMPLGYVTHGLNLEEAARYMRSLKVPATVSVSSPDAVTAYNGYLTSSSKTPEEH
EPI_ISL_424249   623 LNETLVTMPLGYVTHGLNLEEAARYMRSLKVPATVSVSSPDAVTAYNGYLTSSSKTPEEH
EPI_ISL_424250   623 LNETLVTMPLGYVTHGLNLEEAARYMRSLKVPATVSVSSPDAVTAYNGYLTSSSKTPEEH
EPI_ISL_424252   623 LNETLVTMPLGYVTHGLNLEEAARYMRSLKVPATVSVSSPDAVTAYNGYLTSSSKTPEEH
EPI_ISL_424258   623 LNETLVTMPLGYVTHGLNLEEAARYMRSLKVPATVSVSSPDAVTAYNGYLTSSSKTPEEH
EPI_ISL_424268   623 LNETLVTMPLGYVTHGLNLEEAARYMRSLKVPATVSVSSPDAVTAYNGYLTSSSKTPEEH
EPI_ISL_424269   623 LNETLVTMPLGYVTHGLNLEEAARYMRSLKVPATVSVSSPDAVTAYNGYLTSSSKTPEEH
EPI_ISL_424275   623 LNETLVTMPLGYVTHGLNLEEAARYMRSLKVPATVSVSSPDAVTAYNGYLTSSSKTPEEH
EPI_ISL_424276   623 LNETLVTMPLGYVTHGLNLEEAARYMRSLKVPATVSVSSPDAVTAYNGYLTSSSKTPEEH
EPI_ISL_424279   623 LNETLVTMPLGYVTHGLNLEEAARYMRSLKVPATVSVSSPDAVTAYNGYLTSSSKTPEEH

ORF1ab          1501 FIETISLAGSYKDWSYSGQSTQLGIEFLKRGDKSVYYTSNPTTFHLDGEVITFDNLKTLL
EPI_ISL_424219   683 FIETISLAGSYKDWSYSGQSTQLGIEFLKRGDKSVYYTSNPTTFHLDGEVITFDNLKTLL
EPI_ISL_424222   683 FIETISLAGSYKDWSYSGQSTQLGIEFLKRGDKSVYYTSNPTTFHLDGEVITFDNLKTLL
EPI_ISL_424223   683 FIETISLAGSYKDWSYSGQSTQLGIEFLKRGDKSVYYTSNPTTFHLDGEVITFDNLKTLL
EPI_ISL_424226   683 FIETISLAGSYKDWSYSGQSTQLGIEFLKRGDKSVYYTSNPTTFHLDGEVITFDNLKTLL
EPI_ISL_424228   683 FIETISLAGSYKDWSYSGQSTQLGIEFLKRGDKSVYYTSNPTTFHLDGEVITFDNLKTLL
EPI_ISL_424230   683 FIETISLAGSYKDWSYSGQSTQLGIEFLKRGDKSVYYTSNPTTFHLDGEVITFDNLKTLL
EPI_ISL_424231   683 FIETISLAGSYKDWSYSGQSTQLGIEFLKRGDKSVYYTSNPTTFHLDGEVITFDNLKTLL
EPI_ISL_424240   683 FIETISLAGSYKDWSYSGQSTQLGIEFLKRGDKSVYYTSNPTTFHLDGEVITFDNLKTLL
EPI_ISL_424241   683 FIETISLAGSYKDWSYSGQSTQLGIEFLKRGDKSVYYTSNPTTFHLDGEVITFDNLKTLL
EPI_ISL_424243   683 FIETISLAGSYKDWSYSGQSTQLGIEFLKRGDKSVYYTSNPTTFHLDGEVITFDNLKTLL
EPI_ISL_424247   683 FIETISLAGSYKDWSYSGQSTQLGIEFLKRGDKSVYYTSNPTTFHLDGEVITFDNLKTLL
EPI_ISL_424249   683 FIETISLAGSYKDWSYSGQSTQLGIEFLKRGDKSVYYTSNPTTFHLDGEVITFDNLKTLL
EPI_ISL_424250   683 FIETISLAGSYKDWSYSGQSTQLGIEFLKRGDKSVYYTSNPTTFHLDGEVITFDNLKTLL
EPI_ISL_424252   683 FIETISLAGSYKDWSYSGQSTQLGIEFLKRGDKSVYYTSNPTTFHLDGEVITFDNLKTLL
EPI_ISL_424258   683 FIETISLAGSYKDWSYSGQSTQLGIEFLKRGDKSVYYTSNPTTFHLDGEVITFDNLKTLL
EPI_ISL_424268   683 FIETISLAGSYKDWSYSGQSTQLGIEFLKRGDKSVYYTSNPTTFHLDGEVITFDNLKTLL
EPI_ISL_424269   683 FIETISLAGSYKDWSYSGQSTQLGIEFLKRGDKSVYYTSNPTTFHLDGEVITFDNLKTLL
EPI_ISL_424275   683 FIETISLAGSYKDWSYSGQSTQLGIEFLKRGDKSVYYTSNPTTFHLDGEVITFDNLKTLL
EPI_ISL_424276   683 FIETISLAGSYKDWSYSGQSTQLGIEFLKRGDKSVYYTSNPTTFHLDGEVITFDNLKTLL
EPI_ISL_424279   683 FIETISLAGSYKDWSYSGQSTQLGIEFLKRGDKSVYYTSNPTTFHLDGEVITFDNLKTLL

ORF1ab          1561 SLREVRTIKVFTTVDNINLHTQVVDMSMTYGQQFGPTYLDGADVTKIKPHNSHEGKTFYV
EPI_ISL_424219   743 SLREVRTIKVFTTVDNINLHTQVVDMSMTYGQQFGPTYLDGADVTKIKPHNSHEGKTFYV
EPI_ISL_424222   743 SLREVRTIKVFTTVDNINLHTQVVDMSMTYGQQFGPTYLDGADVTKIKPHNSHEGKTFYV
EPI_ISL_424223   743 SLREVRTIKVFTTVDNINLHTQVVDMSMTYGQQFGPTYLDGADVTKIKPHNSHEGKTFYV
EPI_ISL_424226   743 SLREVRTIKVFTTVDNINLHTQVVDMSMTYGQQFGPTYLDGADVTKIKPHNSHEGKTFYV
EPI_ISL_424228   743 SLREVRTIKVFTTVDNINLHTQVVDMSMTYGQQFGPTYLDGADVTKIKPHNSHEGKTFYV
EPI_ISL_424230   743 SLREVRTIKVFTTVDNINLHTQVVDMSMTYGQQFGPTYLDGADVTKIKPHNSHEGKTFYV
EPI_ISL_424231   743 SLREVRTIKVFTTVDNINLHTQVVDMSMTYGQQFGPTYLDGADVTKIKPHNSHEGKTFYV
EPI_ISL_424240   743 SLREVRTIKVFTTVDNINLHTQVVDMSMTYGQQFGPTYLDGADVTKIKPHNSHEGKTFYV
EPI_ISL_424241   743 SLREVRTIKVFTTVDNINLHTQVVDMSMTYGQQFGPTYLDGADVTKIKPHNSHEGKTFYV
EPI_ISL_424243   743 SLREVRTIKVFTTVDNINLHTQVVDMSMTYGQQFGPTYLDGADVTKIKPHNSHEGKTFYV
EPI_ISL_424247   743 SLREVRTIKVFTTVDNINLHTQVVDMSMTYGQQFGPTYLDGADVTKIKPHNSHEGKTFYV
EPI_ISL_424249   743 SLREVRTIKVFTTVDNINLHTQVVDMSMTYGQQFGPTYLDGADVTKIKPHNSHEGKTFYV
EPI_ISL_424250   743 SLREVRTIKVFTTVDNINLHTQVVDMSMTYGQQFGPTYLDGADVTKIKPHNSHEGKTFYV
EPI_ISL_424252   743 SLREVRTIKVFTTVDNINLHTQVVDMSMTYGQQFGPTYLDGADVTKIKPHNSHEGKTFYV
EPI_ISL_424258   743 SLREVRTIKVFTTVDNINLHTQVVDMSMTYGQQFGPTYLDGADVTKIKPHNSHEGKTFYV
EPI_ISL_424268   743 SLREVRTIKVFTTVDNINLHTQVVDMSMTYGQQFGPTYLDGADVTKIKPHNSHEGKTFYV
EPI_ISL_424269   743 SLREVRTIKVFTTVDNINLHTQVVDMSMTYGQQFGPTYLDGADVTKIKPHNSHEGKTFYV
EPI_ISL_424275   743 SLREVRTIKVFTTVDNINLHTQVVDMSMTYGQQFGPTYLDGADVTKIKPHNSHEGKTFYV
EPI_ISL_424276   743 SLREVRTIKVFTTVDNINLHTQVVDMSMTYGQQFGPTYLDGADVTKIKPHNSHEGKTFYV
```

EPI_ISL_424279 743 SLREVRTIKVFTTVDNINLHTQVVDMSMTYGQQFGPTYLDGADVTKIKPHNSHEGKTFYV

```
ORF1ab          1621 LPNDDTLRVEAFEYYHTTDPSFLGRYMSALNHTKKWKYPQVNGLTSIKWADNNCYLATAL
EPI_ISL_424219   803 LPNDDTLRVEAFEYYHTTDPSFLGRYMSALNHTKKWKYPQVNGLTSIKWADNNCYLATAL
EPI_ISL_424222   803 LPNDDTLRVEAFEYYHTTDPSFLGRYMSALNHTKKWKYPQVNGLTSIKWADNNCYLATAL
EPI_ISL_424223   803 LPNDDTLRVEAFEYYHTTDPSFLGRYMSALNHTKKWKYPQVNGLTSIKWADNNCYLATAL
EPI_ISL_424226   803 LPNDDTLRVEAFEYYHTTDPSFLGRYMSALNHTKKWKYPQVNGLTSIKWADNNCYLATAL
EPI_ISL_424228   803 LPNDDTLRVEAFEYYHTTDPSFLGRYMSALNHTKKWKYPQVNGLTSIKWADNNCYLATAL
EPI_ISL_424230   803 LPNDDTLRVEAFEYYHTTDPSFLGRYMSALNHTKKWKYPQVNGLTSIKWADNNCYLATAL
EPI_ISL_424231   803 LPNDDTLRVEAFEYYHTTDPSFLGRYMSALNHTKKWKYPQVNGLTSIKWADNNCYLATAL
EPI_ISL_424240   803 LPNDDTLRVEAFEYYHTTDPSFLGRYMSALNHTKKWKYPQVNGLTSIKWADNNCYLATAL
EPI_ISL_424241   803 LPNDDTLRVEAFEYYHTTDPSFLGRYMSALNHTKKWKYPQVNGLTSIKWADNNCYLATAL
EPI_ISL_424243   803 LPNDDTLRVEAFEYYHTTDPSFLGRYMSALNHTKKWKYPQVNGLTSIKWADNNCYLATAL
EPI_ISL_424247   803 LPNDDTLRVEAFEYYHTTDPSFLGRYMSALNHTKKWKYPQVNGLTSIKWADNNCYLATAL
EPI_ISL_424249   803 LPNDDTLRVEAFEYYHTTDPSFLGRYMSALNHTKKWKYPQVNGLTSIKWADNNCYLATAL
EPI_ISL_424250   803 LPNDDTLRVEAFEYYHTTDPSFLGRYMSALNHTKKWKYPQVNGLTSIKWADNNCYLATAL
EPI_ISL_424252   803 LPNDDTLRVEAFEYYHTTDPSFLGRYMSALNHTKKWKYPQVNGLTSIKWADNNCYLATAL
EPI_ISL_424258   803 LPNDDTLRVEAFEYYHTTDPSFLGRYMSALNHTKKWKYPQVNGLTSIKWADNNCYLATAL
EPI_ISL_424268   803 LPNDDTLRVEAFEYYHTTDPSFLGRYMSALNHTKKWKYPQVNGLTSIKWADNNCYLATAL
EPI_ISL_424269   803 LPNDDTLRVEAFEYYHTTDPSFLGRYMSALNHTKKWKYPQVNGLTSIKWADNNCYLATAL
EPI_ISL_424275   803 LPNDDTLRVEAFEYYHTTDPSFLGRYMSALNHTKKWKYPQVNGLTSIKWADNNCYLATAL
EPI_ISL_424276   803 LPNDDTLRVEAFEYYHTTDPSFLGRYMSALNHTKKWKYPQVNGLTSIKWADNNCYLATAL
EPI_ISL_424279   803 LPNDDTLRVEAFEYYHTTDPSFLGRYMSALNHTKKWKYPQVNGLTSIKWADNNCYLATAL

ORF1ab          1681 LTLQQIELKFNPPALQDAYYRARAGEAANFCALILAYCNKTVGELGDVRETMSYLFQHAN
EPI_ISL_424219   863 LTLQQIELKFNPPALQDAYYRARAGEAANFCALILAYCNKTVGELGDVRETMSYLFQHAN
EPI_ISL_424222   863 LTLQQIELKFNPPALQDAYYRARAGEAANFCALILAYCNKTVGELGDVRETMSYLFQHAN
EPI_ISL_424223   863 LTLQQIELKFNPPALQDAYYRARAGEAANFCALILAYCNKTVGELGDVRETMSYLFQHAN
EPI_ISL_424226   863 LTLQQIELKFNPPALQDAYYRARAGEAANFCALILAYCNKTVGELGDVRETMSYLFQHAN
EPI_ISL_424228   863 LTLQQIELKFNPPALQDAYYRARAGEAANFCALILAYCNKTVGELGDVRETMSYLFQHAN
EPI_ISL_424230   863 LTLQQIELKFNPPALQDAYYRARAGEAANFCALILAYCNKTVGELGDVRETMSYLFQHAN
EPI_ISL_424231   863 LTLQQIELKFNPPALQDAYYRARAGEAANFCALILAYCNKTVGELGDVRETMSYLFQHAN
EPI_ISL_424240   863 LTLQQIELKFNPPALQDAYYRARAGEAANFCALILAYCNKTVGELGDVRETMSYLFQHAN
EPI_ISL_424241   863 LTLQQIELKFNPPALQDAYYRARAGEAANFCALILAYCNKTVGELGDVRETMSYLFQHAN
EPI_ISL_424243   863 LTLQQIELKFNPPALQDAYYRARAGEAANFCALILAYCNKTVGELGDVRETMSYLFQHAN
EPI_ISL_424247   863 LTLQQIELKFNPPALQDAYYRARAGEAANFCALILAYCNKTVGELGDVRETMSYLFQHAN
EPI_ISL_424249   863 LTLQQIELKFNPPALQDAYYRARAGEAANFCALILAYCNKTVGELGDVRETMSYLFQHAN
EPI_ISL_424250   863 LTLQQIELKFNPPALQDAYYRARAGEAANFCALILAYCNKTVGELGDVRETMSYLFQHAN
EPI_ISL_424252   863 LTLQQIELKFNPPALQDAYYRARAGEAANFCALILAYCNKTVGELGDVRETMSYLFQHAN
EPI_ISL_424258   863 LTLQQIELKFNPPALQDAYYRARAGEAANFCALILAYCNKTVGELGDVRETMSYLFQHAN
EPI_ISL_424268   863 LTLQQIELKFNPPALQDAYYRARAGEAANFCALILAYCNKTVGELGDVRETMSYLFQHAN
EPI_ISL_424269   863 LTLQQIELKFNPPALQDAYYRARAGEAANFCALILAYCNKTVGELGDVRETMSYLFQHAN
EPI_ISL_424275   863 LTLQQIELKFNPPALQDAYYRARAGEAANFCALILAYCNKTVGELGDVRETMSYLFQHAN
EPI_ISL_424276   863 LTLQQIELKFNPPALQDAYYRARAGEAANFCALILAYCNKTVGELGDVRETMSYLFQHAN
EPI_ISL_424279   863 LTLQQIELKFNPPALQDAYYRARAGEAANFCALILAYCNKTVGELGDVRETMSYLFQHAN

ORF1ab          1741 LDSCKRVLNVVCKTCGQQQTTLKGVEAVMYMGTLSYEQFKKGVQIPCTCGKQATKYLVQQ
EPI_ISL_424219   923 LDSCKRVLNVVCKTCGQQQTTLKGVEAVMYMGTLSYEQFKKGVQIPCTCGKQATKYLVQQ
EPI_ISL_424222   923 LDSCKRVLNVVCKTCGQQQTTLKGVEAVMYMGTLSYEQFKKGVQIPCTCGKQATKYLVQQ
EPI_ISL_424223   923 LDSCKRVLNVVCKTCGQQQTTLKGVEAVMYMGTLSYEQFKKGVQIPCTCGKQATKYLVQQ
EPI_ISL_424226   923 LDSCKRVLNVVCKTCGQQQTTLKGVEAVMYMGTLSYEQFKKGVQIPCTCGKQATKYLVQQ
EPI_ISL_424228   923 LDSCKRVLNVVCKTCGQQQTTLKGVEAVMYMGTLSYEQFKKGVQIPCTCGKQATKYLVQQ
EPI_ISL_424230   923 LDSCKRVLNVVCKTCGQQQTTLKGVEAVMYMGTLSYEQFKKGVQIPCTCGKQATKYLVQQ
EPI_ISL_424231   923 LDSCKRVLNVVCKTCGQQQTTLKGVEAVMYMGTLSYEQFKKGVQIPCTCGKQATKYLVQQ
EPI_ISL_424240   923 LDSCKRVLNVVCKTCGQQQTTLKGVEAVMYMGTLSYEQFKKGVQIPCTCGKQATKYLVQQ
EPI_ISL_424241   923 LDSCKRVLNVVCKTCGQQQTTLKGVEAVMYMGTLSYEQFKKGVQIPCTCGKQATKYLVQQ
EPI_ISL_424243   923 LDSCKRVLNVVCKTCGQQQTTLKGVEAVMYMGTLSYEQFKKGVQIPCTCGKQATKYLVQQ
EPI_ISL_424247   923 LDSCKRVLNVVCKTCGQQQTTLKGVEAVMYMGTLSYEQFKKGVQIPCTCGKQATKYLVQQ
EPI_ISL_424249   923 LDSCKRVLNVVCKTCGQQQTTLKGVEAVMYMGTLSYEQFKKGVQIPCTCGKQATKYLVQQ
EPI_ISL_424250   923 LDSCKRVLNVVCKTCGQQQTTLKGVEAVMYMGTLSYEQFKKGVQIPCTCGKQATKYLVQQ
EPI_ISL_424252   923 LDSCKRVLNVVCKTCGQQQTTLKGVEAVMYMGTLSYEQFKKGVQIPCTCGKQATKYLVQQ
EPI_ISL_424258   923 LDSCKRVLNVVCKTCGQQQTTLKGVEAVMYMGTLSYEQFKKGVQIPCTCGKQATKYLVQQ
EPI_ISL_424268   923 LDSCKRVLNVVCKTCGQQQTTLKGVEAVMYMGTLSYEQFKKGVQIPCTCGKQATKYLVQQ
EPI_ISL_424269   923 LDSCKRVLNVVCKTCGQQQTTLKGVEAVMYMGTLSYEQFKKGVQIPCTCGKQATKYLVQQ
EPI_ISL_424275   923 LDSCKRVLNVVCKTCGQQQTTLKGVEAVMYMGTLSYEQFKKGVQIPCTCGKQATKYLVQQ
EPI_ISL_424276   923 LDSCKRVLNVVCKTCGQQQTTLKGVEAVMYMGTLSYEQFKKGVQIPCTCGKQATKYLVQQ
```

EPI_ISL_424279 923 LDSCKRVLNVVCKTCGQQQTTLKGVEAVMYMGTLSYEQFKKGVQIPCTCGKQATKYLVQQ

```
ORF1ab         1801 ESPFVMMSAPPAQYELKHGTFTCASEYTGNYQCGHYKHITSKETLYCIDGALLTKSSEYK
EPI_ISL_424219  983 ESPFVMMSAPPAQYELKHGTFTCASEYTGNYQCGHYKHITSKETLYCIDGALLTKSSEYK
EPI_ISL_424222  983 ESPFVMMSAPPAQYELKHGTFTCASEYTGNYQCGHYKHITSKETLYCIDGALLTKSSEYK
EPI_ISL_424223  983 ESPFVMMSAPPAQYELKHGTFTCASEYTGNYQCGHYKHITSKETLYCIDGALLTKSSEYK
EPI_ISL_424226  983 ESPFVMMSAPPAQYELKHGTFTCASEYTGNYQCGHYKHITSKETLYCIDGALLTKSSEYK
EPI_ISL_424228  983 ESPFVMMSAPPAQYELKHGTFTCASEYTGNYQCGHYKHITSKETLYCIDGALLTKSSEYK
EPI_ISL_424230  983 ESPFVMMSAPPAQYELKHGTFTCASEYTGNYQCGHYKHIISKETLYCIDGALLTKSSEYK
EPI_ISL_424231  983 ESPFVMMSAPPAQYELKHGTFTCASEYTGNYQCGHYKHIISKETLYCIDGALLTKSSEYK
EPI_ISL_424240  983 ESPFVMMSAPPAQYELKHGTFTCASEYTGNYQCGHYKHITSKETLYCIDGALLTKSSEYK
EPI_ISL_424241  983 ESPFVMMSAPPAQYELKHGTFTCASEYTGNYQCGHYKHITSKETLYCIDGALLTKSSEYK
EPI_ISL_424243  983 ESPFVMMSAPPAQYELKHGTFTCASEYTGNYQCGHYKHITSKETLYCIDGALLTKSSEYK
EPI_ISL_424247  983 ESPFVMMSAPPAQYELKHGTFTCASEYTGNYQCGHYKHITSKETLYCIDGALLTKSSEYK
EPI_ISL_424249  983 ESPFVMMSAPPAQYELKHGTFTCASEYTGNYQCGHYKHITSKETLYCIDGALLTKSSEYK
EPI_ISL_424250  983 ESPFVMMSAPPAQYELKHGTFTCASEYTGNYQCGHYKHITSKETLYCIDGALLTKSSEYK
EPI_ISL_424252  983 ESPFVMMSAPPAQYELKHGTFTCASEYTGNYQCGHYKHITSKETLYCIDGALLTKSSEYK
EPI_ISL_424258  983 ESPFVMMSAPPAQYELKHGTFTCASEYTGNYQCGHYKHITSKETLYCIDGALLTKSSEYK
EPI_ISL_424268  983 ESPFVMMSAPPAQYELKHGTFTCASEYTGNYQCGHYKHITSKETLYCIDGALLTKSSEYK
EPI_ISL_424269  983 ESPFVMMSAPPAQYELKHGTFTCASEYTGNYQCGHYKHITSKETLYCIDGALLTKSSEYK
EPI_ISL_424275  983 ESPFVMMSAPPAQYELKHGTFTCASEYTGNYQCGHYKHITSKETLYCIDGALLTKSSEYK
EPI_ISL_424276  983 ESPFVMMSAPPAQYELKHGTFTCASEYTGNYQCGHYKHITSKETLYCIDGALLTKSSEYK
EPI_ISL_424279  983 ESPFVMMSAPPAQYELKHGTFTCASEYTGNYQCGHYKHITSKETLYCIDGALLTKSSEYK

ORF1ab         1861 GPITDVFYKENSYTTTIKPVTYKLDGVVCTEIDPKLDNYYKKDNSYFTEQPIDLVPNQPY
EPI_ISL_424219 1043 GPITDVFYKENSYTTTIKPVTYKLDGVVCTEIDPKLDNYYKKDNSYFTEQPIDLVPNQPY
EPI_ISL_424222 1043 GPITDVFYKENSYTTTIKPVTYKLDGVVCTEIDPKLDNYYKKDNSYFTEQPIDLVPNQPY
EPI_ISL_424223 1043 GPITDVFYKENSYTTTIKPVTYKLDGVVCTEIDPKLDNYYKKDNSYFTEQPIDLVPNQPY
EPI_ISL_424226 1043 GPITDVFYKENSYTTTIKPVTYKLDGVVCTEIDPKLDNYYKKDNSYFTEQPIDLVPNQPY
EPI_ISL_424228 1043 GPITDVFYKENSYTTTIKPVTYKLDGVVCTEIDPKLDNYYKKDNSYFTEQPIDLVPNQPY
EPI_ISL_424230 1043 GPITDVFYKENSYTTTIKPVTYKLDGVVCTEIDPKLDNYYKKDNSYFTEQPIDLVPNQPY
EPI_ISL_424231 1043 GPITDVFYKENSYTTTIKPVTYKLDGVVCTEIDPKLDNYYKKDNSYFTEQPIDLVPNQPY
EPI_ISL_424240 1043 GPITDVFYKENSYTTTIKPVTYKLDGVVCTEIDPKLDNYYKKDNSYFTEQPIDLVPNQPY
EPI_ISL_424241 1043 GPITDVFYKENSYTTTIKPVTYKLDGVVCTEIDPKLDNYYKKDNSYFTEQPIDLVPNQPY
EPI_ISL_424243 1043 GPITDVFYKENSYTTTIKPVTYKLDGVVCTEIDPKLDNYYKKDNSYFTEQPIDLVPNQPY
EPI_ISL_424247 1043 GPITDVFYKENSYTTTIKPVTYKLDGVVCTEIDPKLDNYYKKDNSYFTEQPIDLVPNQPY
EPI_ISL_424249 1043 GPITDVFYKENSYTTTIKPVTYKLDGVVCTEIDPKLDNYYKKDNSYFTEQPIDLVPNQPY
EPI_ISL_424250 1043 GPITDVFYKENSYTTTIKPVTYKLDGVVCTEIDPKLDNYYKKDNSYFTEQPIDLVPNQPY
EPI_ISL_424252 1043 GPITDVFYKENSYTTTIKPVTYKLDGVVCTEIDPKLDNYYKKDNSYFTEQPIDLVPNQPY
EPI_ISL_424258 1043 GPITDVFYKENSYTTTIKPVTYKLDGVVCTEIDPKLDNYYKKDNSYFTEQPIDLVPNQPY
EPI_ISL_424268 1043 GPITDVFYKENSYTTTIKPVTYKLDGVVCTEIDPKLDNYYKKDNSYFTEQPIDLVPNQPY
EPI_ISL_424269 1043 GPITDVFYKENSYTTTIKPVTYKLDGVVCTEIDPKLDNYYKKDNSYFTEQPIDLVPNQPY
EPI_ISL_424275 1043 GPITDVFYKENSYTTTIKPVTYKLDGVVCTEIDPKLDNYYKKDNSYFTEQPIDLVPNQPY
EPI_ISL_424276 1043 GPITDVFYKENSYTTTIKPVTYKLDGVVCTEIDPKLDNYYKKDNSYFTEQPIDLVPNQPY
EPI_ISL_424279 1043 GPITDVFYKENSYTTTIKPVTYKLDGVVCTEIDPKLDNYYKKDNSYFTEQPIDLVPNQPY

ORF1ab         1921 PNASFDNFKFVCDNIKFADDLNQLTGYKKPASRELKVTFFPDLNGDVVAIDYKHYTPSFK
EPI_ISL_424219 1103 PNASFDNFKFVCDNIKFADDLNQLTGYKKPASRELKVTFFPDLNGDVVAIDYKHYTPSFK
EPI_ISL_424222 1103 PNASFDNFKFVCDNIKFADDLNQLTGYKKPASRELKVTFFPDLNGDVVAIDYKHYTPSFK
EPI_ISL_424223 1103 PNASFDNFKFVCDNIKFADDLNQLTGYKKPASRELKVTFFPDLNGDVVAIDYKHYTPSFK
EPI_ISL_424226 1103 PNASFDNFKFVCDNIKFADDLNQLTGYKKPASRELKVTFFPDLNGDVVAIDYKHYTPSFK
EPI_ISL_424228 1103 PNASFDNFKFVCDNIKFADDLNQLTGYKKPASRELKVTFFPDLNGDVVAIDYKHYTPSFK
EPI_ISL_424230 1103 PNASFDNFKFVCDNIKFADDLNQLTGYKKPASRELKVTFFPDLNGDVVAIDYKHYTPSFK
EPI_ISL_424231 1103 PNASFDNFKFVCDNIKFADDLNQLTGYKKPASRELKVTFFPDLNGDVVAIDYKHYTPSFK
EPI_ISL_424240 1103 PNASFDNFKFVCDNIKFADDLNQLTGYKKPASRELKVTFFPDLNGDVVAIDYKHYTPSFK
EPI_ISL_424241 1103 PNASFDNFKFVCDNIKFADDLNQLTGYKKPASRELKVTFFPDLNGDVVAIDYKHYTPSFK
EPI_ISL_424243 1103 PNASFDNFKFVCDNIKFADDLNQLTGYKKPASRELKVTFFPDLNGDVVAIDYKHYTPSFK
EPI_ISL_424247 1103 PNASFDNFKFVCDNIKFADDLNQLTGYKKPASRELKVTFFPDLNGDVVAIDYKHYTPSFK
EPI_ISL_424249 1103 PNASFDNFKFVCDNIKFADDLNQLTGYKKPASRELKVTFFPDLNGDVVAIDYKHYTPSFK
EPI_ISL_424250 1103 PNASFDNFKFVCDNIKFADDLNQLTGYKKPASRELKVTFFPDLNGDVVAIDYKHYTPSFK
EPI_ISL_424252 1103 PNASFDNFKFVCDNIKFADDLNQLTGYKKPASRELKVTFFPDLNGDVVAIDYKHYTPSFK
EPI_ISL_424258 1103 PNASFDNFKFVCDNIKFADDLNQLTGYKKPASRELKVTFFPDLNGDVVAIDYKHYTPSFK
EPI_ISL_424268 1103 PNASFDNFKFVCDNIKFADDLNQLTGYKKPASRELKVTFFPDLNGDVVAIDYKHYTPSFK
EPI_ISL_424269 1103 PNASFDNFKFVCDNIKFADDLNQLTGYKKPASRELKVTFFPDLNGDVVAIDYKHYTPSFK
EPI_ISL_424275 1103 PNASFDNFKFVCDNIKFADDLNQLTGYKKPASRELKVTFFPDLNGDVVAIDYKHYTPSFK
EPI_ISL_424276 1103 PNASFDNFKFVCDNIKFADDLNQLTGYKKPASRELKVTFFPDLNGDVVAIDYKHYTPSFK
```

EPI_ISL_424279 1103 PNASFDNFKFVCDNIKFADDLNQLTGYKKPASRELKVTFFPDLNGDVVAIDYKHYTPSFK

```
ORF1ab         1981 KGAKLLHKPIVWHVNNATNKATYKPNTWCIRCLWSTKPVETSNSFDVLKSEDAQGMDNLA
EPI_ISL_424219 1163 KGAKLLHKPIVWHVNNATNKATYKPNTWCIRCLWSTKPVETSNSFDVLKSEDAQGMDNLA
EPI_ISL_424222 1163 KGAKLLHKPIVWHVNNATNKATYKPNTWCIRCLWSTKPVETSNSFDVLKSEDAQGMDNLA
EPI_ISL_424223 1163 KGAKLLHKPIVWHVNNATNKATYKPNTWCIRCLWSTKPVETSNSFDVLKSEDAQGMDNLA
EPI_ISL_424226 1163 KGAKLLHKPIVWHVNNATNKATYKPNTWCIRCLWSTKPVETSNSFDVLKSEDAQGMDNLA
EPI_ISL_424228 1163 KGAKLLHKPIVWHVNNATNKATYKPNTWCIRCLWSTKPVETSNSFDVLKSEDAQGMDNLA
EPI_ISL_424230 1163 KGAKLLHKPIVWHVNNATNKATYKPNTWCIRCLWSTKPVETSNSFDVLKSEDAQGMDNLA
EPI_ISL_424231 1163 KGAKLLHKPIVWHVNNATNKATYKPNTWCIRCLWSTKPVETSNSFDVLKSEDAQGMDNLA
EPI_ISL_424240 1163 KGAKLLHKPIVWHVNNATNKATYKPNTWCIRCLWSTKPVETSNSFDVLKSEDAQGMDNLA
EPI_ISL_424241 1163 KGAKLLHKPIVWHVNNATNKATYKPNTWCIRCLWSTKPVETSNSFDVLKSEDAQGMDNLA
EPI_ISL_424243 1163 KGAKLLHKPIVWHVNNATNKATYKPNTWCIRCLWSTKPVETSNSFDVLKSEDAQGMDNLA
EPI_ISL_424247 1163 KGAKLLHKPIVWHVNNATNKATYKPNTWCIRCLWSTKPVETSNSFDVLKSEDAQGMDNLA
EPI_ISL_424249 1163 KGAKLLHKPIVWHVNNATNKATYKPNTWCIRCLWSTKPVETSNSFDVLKSEDAQGMDNLA
EPI_ISL_424250 1163 KGAKLLHKPIVWHVNNATNKATYKPNTWCIRCLWSTKPVETSNSFDVLKSEDAQGMDNLA
EPI_ISL_424252 1163 KGAKLLHKPIVWHVNNATNKATYKPNTWCIRCLWSTKPVETSNSFDVLKSEDAQGMDNLA
EPI_ISL_424258 1163 KGAKLLHKPIVWHVNNATNKATYKPNTWCIRCLWSTKPVETSNSFDVLKSEDAQGMDNLA
EPI_ISL_424268 1163 KGAKLLHKPIVWHVNNATNKATYKPNTWCIRCLWSTKPVETSNSFDVLKSEDAQGMDNLA
EPI_ISL_424269 1163 KGAKLLHKPIVWHVNNATNKATYKPNTWCIRCLWSTKPVETSNSFDVLKSEDAQGMDNLA
EPI_ISL_424275 1163 KGAKLLHKPIVWHVNNATNKATYKPNTWCIRCLWSTKPVETSNSFDVLKSEDAQGMDNLA
EPI_ISL_424276 1163 KGAKLLHKPIVWHVNNATNKATYKPNTWCIRCLWSTKPVETSNSFDVLKSEDAQGMDNLA
EPI_ISL_424279 1163 KGAKLLHKPIVWHVNNATNKATYKPNTWCIRCLWSTKPVETSNSFDVLKSEDAQGMDNLA

ORF1ab         2041 CEDLKPVSEEVVENPTIQKDVLECNVKTTEVVGDIILKPANNSLKITEEVGHTDLMAAYV
EPI_ISL_424219 1223 CEDLKPVSEEVVENPTIQKDVLECNVKTTEVVGDIILKPANNSLKITEEVGHTDLMAAYV
EPI_ISL_424222 1223 CEDLKPVSEEVVENPTIQKDVLECNVKTTEVVGDIILKPANNSLKITEEVGHTDLMAAYV
EPI_ISL_424223 1223 CEDLKPVSEEVVENPTIQKDVLECNVKTTEVVGDIILKPANNSLKITEEVGHTDLMAAYV
EPI_ISL_424226 1223 CEDLKPVSEEVVENPTIQKDVLECNVKTTEVVGDIILKPANNSLKITEEVGHTDLMAAYV
EPI_ISL_424228 1223 CEDLKPVSEEVVENPTIQKDVLECNVKTTEVVGDIILKPANNSLKITEEVGHTDLMAAYV
EPI_ISL_424230 1223 CEDLKPVSEEVVENPTIQKDVLECNVKTTEVVGDIILKPANNSLKITEEVGHTDLMAAYV
EPI_ISL_424231 1223 CEDLKPVSEEVVENPTIQKDVLECNVKTTEVVGDIILKPANNSLKITEEVGHTDLMAAYV
EPI_ISL_424240 1223 CEDLKPVSEEVVENPTIQKDVLECNVKTTEVVGDIILKPANNSLKITEEVGHTDLMAAYV
EPI_ISL_424241 1223 CEDLKPVSEEVVENPTIQKDVLECNVKTTEVVGDIILKPANNSLKITEEVGHTDLMAAYV
EPI_ISL_424243 1223 CEDLKPVSEEVVENPTIQKDVLECNVKTTEVVGDIILKPANNSLKITEEVGHTDLMAAYV
EPI_ISL_424247 1223 CEDLKPVSEEVVENPTIQKDVLECNVKTTEVVGDIILKPANNSLKITEEVGHTDLMAAYV
EPI_ISL_424249 1223 CEDLKPVSEEVVENPTIQKDVLECNVKTTEVVGDIILKPANNSLKITEEVGHTDLMAAYV
EPI_ISL_424250 1223 CEDLKPVSEEVVENPTIQKDVLECNVKTTEVVGDIILKPANNSLKITEEVGHTDLMAAYV
EPI_ISL_424252 1223 CEDLKPVSEEVVENPTIQKDVLECNVKTTEVVGDIILKPANNSLKITEEVGHTDLMAAYV
EPI_ISL_424258 1223 CEDLKPVSEEVVENPTIQKDVLECNVKTTEVVGDIILKPANNSLKITEEVGHTDLMAAYV
EPI_ISL_424268 1223 CEDLKPVSEEVVENPTIQKDVLECNVKTTEVVGDIILKPANNSLKITEEVGHTDLMAAYV
EPI_ISL_424269 1223 CEDLKPVSEEVVENPTIQKDVLECNVKTTEVVGDIILKPANNSLKITEEVGHTDLMAAYV
EPI_ISL_424275 1223 CEDLKPVSEEVVENPTIQKDVLECNVKTTEVVGDIILKPANNSLKITEEVGHTDLMAAYV
EPI_ISL_424276 1223 CEDLKPVSEEVVENPTIQKDVLECNVKTTEVVGDIILKPANNSLKITEEVGHTDLMAAYV
EPI_ISL_424279 1223 CEDLKPVSEEVVENPTIQKDVLECNVKTTEVVGDIILKPANNSLKITEEVGHTDLMAAYV

ORF1ab         2101 DNSSLTIKKPNELSRVLGLKTLATHGLAAVNSVPWDTIANYAKPFLNKVVSTTTNIVTRC
EPI_ISL_424219 1283 DNSSLTIKKPNELSRVLGLKTLATHGLAAVNSVPWDTIANYARPFLNKVVSTTTNIVTRC
EPI_ISL_424222 1283 DNSSLTIKKPNELSRVLGLKTLATHGLAAVNSVPWDTIANYAKPFLNKVVSTTTNIVTRC
EPI_ISL_424223 1283 DNSSLTIKKPNELSRVLGLKTLATHGLAAVNSVPWDTIANYAKPFLNKVVSTTTNIVTRC
EPI_ISL_424226 1283 DNSSLTIKKPNELSRVLGLKTLATHGLAAVNSVPWDTIANYAKPFLNKVVSTTTNIVTRC
EPI_ISL_424228 1283 DNSSLTIKKPNELSRVLGLKTLATHGLAAVNSVPWDTIANYAKPFLNKVVSTTTNIVTRC
EPI_ISL_424230 1283 DNSSLTIKKPNELSRVLGLKTLATHGLAAVNSVPWDTIANYAKPFLNKVVSTTTNIVTRC
EPI_ISL_424231 1283 DNSSLTIKKPNELSRVLGLKTLATHGLAAVNSVPWDTIANYAKPFLNKVVSTTTNIVTRC
EPI_ISL_424240 1283 DNSSLTIKKPNELSRVLGLKTLATHGLAAVNSVPWDTIANYAKPFLNKVVSTTTNIVTRC
EPI_ISL_424241 1283 DNSSLTIKKPNELSRVLGLKTLATHGLAAVNSVPWDTIANYAKPFLNKVVSTTTNIVTRC
EPI_ISL_424243 1283 DNSSLTIKKPNELSRVLGLKTLATHGLAAVNSVPWDTIANYAKPFLNKVVSTTTNIVTRC
EPI_ISL_424247 1283 DNSSLTIKKPNELSRVLGLKTLATHGLAAVNSVPWDTIANYAKPFLNKVVSTTTNIVTRC
EPI_ISL_424249 1283 DNSSLTIKKPNELSRVLGLKTLATHGLAAVNSVPWDTIANYAKPFLNKVVSTTTNIVTRC
EPI_ISL_424250 1283 DNSSLTIKKPNELSRVLGLKTLATHGLAAVNSVPWDTIANYAKPFLNKVVSTTTNIVTRC
EPI_ISL_424252 1283 DNSSLTIKKPNELSRVLGLKTLATHGLAAVNSVPWDTIANYAKPFLNKVVSTTTNIVTRC
EPI_ISL_424258 1283 DNSSLTIKKPNELSRVLGLKTLATHGLAAVNSVPWDTIANYAKPFLNKVVSTTTNIVTRC
EPI_ISL_424268 1283 DNSSLTIKKPNELSRVLGLKTLATHGLAAVNSVPWDTIANYAKPFLNKVVSTTTNIVTRC
EPI_ISL_424269 1283 DNSSLTIKKPNELSRVLGLKTLATHGLAAVNSVPWDTIANYAKPFLNKVVSTTTNIVTRC
EPI_ISL_424275 1283 DNSSLTIKKPNELSRVLGLKTLATHGLAAVNSVPWDTIANYAKPFLNKVVSTTTNIVTRC
EPI_ISL_424276 1283 DNSSLTIKKPNELSRVLGLKTLATHGLAAVNSVPWDTIANYAKPFLNKVVSTTTNIVTRC
```

EPI_ISL_424279 1283 DNSSLTIKKPNELSRVLGLKTLATHGLAAVNSVPWDTIANYAKPFLNKVVSTTTNIVTRC

ORF1ab	2161	LNRVCTNYMPYFFTLLLQLCTFTRSTNSRIKASMPTTIAKNTVKSVGKFCLEASFNYLKS
EPI_ISL_424219	1343	LNRVCTNYMPYFFTLLLQLCTFTRSTNSRIKASMPTTIAKNTVKSVGKFCLEASFNYLKS
EPI_ISL_424222	1343	LNRVCTNYMPYFFTLLLQLCTFTRSANSRIKASMPTTIAKNTVKSVGKFCLEASFNYLKS
EPI_ISL_424223	1343	LNRVCTNYMPYFFTLLLQLCTFTRSTNSRIKASMPTTIAKNTVKSVGKFCLEASFNYLKS
EPI_ISL_424226	1343	LNRVCTNYMPYFFTLLLQLCTFTRSTNSRIKASMPTTIAKNTVKSVGKFCLEASFNYLKS
EPI_ISL_424228	1343	LNRVCTNYMPYFFTLLLQLCTFTRSTNSRIKASMPTTIAKNTVKSVGKFCLEASFNYLKS
EPI_ISL_424230	1343	LNRVCTNYMPYFFTLLLQLCTFTRSTNSRIKASMPTTIAKNTVKSVGKFCLEASFNYLKS
EPI_ISL_424231	1343	LNRVCTNYMPYFFTLLLQLCTFTRSTNSRIKASMPTTIAKNTVKSVGKFCLEASFNYLKS
EPI_ISL_424240	1343	LNRVCTNYMPYFFTLLLQLCTFTRSTNSRIKASMPTTIAKNTVKSVGKFCLEASFNYLKS
EPI_ISL_424241	1343	LNRVCTNYMPYFFTLLLQLCTFTRSTNSRIKASMPTTIAKNTVKSVGKFCLEASFNYLKS
EPI_ISL_424243	1343	LNRVCTNYMPYFFTLLLQLCTFTRSTNSRIKASMPTTIAKNTVKSVGKFCLEASFNYLKS
EPI_ISL_424247	1343	LNRVCTNYMPYFFTLLLQLCTFTRSTNSRIKASMPTTIAKNTVKSVGKFCLEASFNYLKS
EPI_ISL_424249	1343	LNRVCTNYMPYFFTLLLQLCTFTRSTNSRIKASMPTTIAKNTVKSVGKFCLEASFNYLKS
EPI_ISL_424250	1343	LNRVCTNYMPYFFTLLLQLCTFTRSTNSRIKASMPTTIAKNTVKSVGKFCLEASFNYLKS
EPI_ISL_424252	1343	LNRVCTNYMPYFFTLLLQLCTFTRSTNSRIKASMPTTIAKNTVKSVGKFCLEASFNYLKS
EPI_ISL_424258	1343	LNRVCTNYMPYFFTLLLQLCTFTRSTNSRIKASMPTTIAKNTVKSVGKFCLEASFNYLKS
EPI_ISL_424268	1343	LNRVCTNYMPYFFTLLLQLCTFTRSTNSRIKASMPTTIAKNTVKSVGKFCLEASFNYLKS
EPI_ISL_424269	1343	LNRVCTNYMPYFFTLLLQLCTFTRSTNSRIKASMPTTIAKNTVKSVGKFCLEASFNYLKS
EPI_ISL_424275	1343	LNRVCTNYMPYFFTLLLQLCTFTRSTNSRIKASMPTTIAKNTVKSVGKFCLEASFNYLKS
EPI_ISL_424276	1343	LNRVCTNYMPYFFTLLLQLCTFTRSTNSRIKASMPTTIAKNTVKSVGKFCLEASFNYLKS
EPI_ISL_424279	1343	LNRVCTNYMPYFFTLLLQLCTFTRSTNSRIKASMPTTIAKNTVKSVGKFCLEASFNYLKS

ORF1ab	2221	PNFSKLINIIIWFLLLSVCLGSLIYSTAALGVLMSNLGMPSYCTGYREGYLNSTNVTIAT
EPI_ISL_424219	1403	PNFSKLINIIIWFLLLSVCLGSLIYSTAALGVLMSNLGMPSYCTGYREGYLNSTNVTIAT
EPI_ISL_424222	1403	PNFSKLINIIIWFLLLSVCLGSLIYSTAALGVLMSNLGMPSYCTGYREGYLNSTNVTIAT
EPI_ISL_424223	1403	PNFSKLINIIIWFLLLSVCLGSLIYSTAALGVLMSNLGMPSYCTGYREGYLNSTNVTIAT
EPI_ISL_424226	1403	PNFSKLINIIIWFLLLSVCLGSLIYSTAALGVLMSNLGMPSYCTGYREGYLNSTNVTIAT
EPI_ISL_424228	1403	PNFSKLINIIIWFLLLSVCLGSLIYSTAALGVLMSNLGMPSYCTGYREGYLNSTNVTIAT
EPI_ISL_424230	1403	PNFSKLINIIIWFLLLSVCLGSLIYSTAALGVLMSNLGMPSYCTGYREGYLNSTNVTIAT
EPI_ISL_424231	1403	PNFSKLINIIIWFLLLSVCLGSLIYSTAALGVLMSNLGMPSYCTGYREGYLNSTNVTIAT
EPI_ISL_424240	1403	PNFSKLINIIIWFLLLSVCLGSLIYSTAALGVLMSNLGMPSYCTGYREGYLNSTNVTIAT
EPI_ISL_424241	1403	PNFSKLINIIIWFLLLSVCLGSLIYSTAALGVLMSNLGMPSYCTGYREGYLNSTNVTIAT
EPI_ISL_424243	1403	PNFSKLINIIIWFLLLSVCLGSLIYSTAALGVLMSNLGMPSYCTGYREGYLNSTNVTIAT
EPI_ISL_424247	1403	PNFSKLINIIIWFLLLSVCLGSLIYSTAALGVLMSNLGMPSYCTGYREGYLNSTNVTIAT
EPI_ISL_424249	1403	PNFSKLINIIIWFLLLSVCLGSLIYSTAALGVLMSNLGMPSYCTGYREGYLNSTNVTIAT
EPI_ISL_424250	1403	PNFSKLINIIIWFLLLSVCLGSLIYSTAALGVLMSNLGMPSYCTGYREGYLNSTNVTIAT
EPI_ISL_424252	1403	PNFSKLINIIIWFLLLSVCLGSLIYSTAALGVLMSNLGMPSYCTGYREGYLNSTNVTIAT
EPI_ISL_424258	1403	PNFSKLINIIIWFLLLSVCLGSLIYSTAALGVLMSNLGMPSYCTGYREGYLNSTNVTIAT
EPI_ISL_424268	1403	PNFSKLINIIIWFLLLSVCLGSLIYSTAALGVLMSNLGMPSYCTGYREGYLNSTNVTIAT
EPI_ISL_424269	1403	PNFSKLINIIIWFLLLSVCLGSLIYSTAALGVLMSNLGMPSYCTGYREGYLNSTNVTIAT
EPI_ISL_424275	1403	PNFSKLINIIIWFLLLSVCLGSLIYSTAALGVLMSNLGMPSYCTGYREGYLNSTNVTIAT
EPI_ISL_424276	1403	PNFSKLINIIIWFLLLSVCLGSLIYSTAALGVLMSNLGMPSYCTGYREGYLNSTNVTIAT
EPI_ISL_424279	1403	PNFSKLINIIIWFLLLSVCLGSLIYSTAALGVLMSNLGMPSYCTGYREGYLNSTNVTIAT

ORF1ab	2281	YCTGSIPCSVCLSGLDSLDTYPSLETIQITISSFKWDLTAFGLVAEWFLAYILFTRFFYV
EPI_ISL_424219	1463	YCTGSIPCSVCLSGLDSLDTYPSLETIQITISSFKWDLTAFGLVAEWFLAYILFTRFFYV
EPI_ISL_424222	1463	YCTGSIPCSVCLSGLDSLDTYPSLETIQITISSFKWDLTAFGLVAEWFLAYILFTRFFYV
EPI_ISL_424223	1463	YCTGSIPCSVCLSGLDSLDTYPSLETIQITISSFKWDLTAFGLVAEWFLAYILFTRFFYV
EPI_ISL_424226	1463	YCTGSIPCSVCLSGLDSLDTYPSLETIQITISSFKWDLTAFGLVAEWFLAYILFTRFFYV
EPI_ISL_424228	1463	YCTGSIPCSVCLSGLDSLDTYPSLETIQITISSFKWDLTAFGLVAEWFLAYILFTRFFYV
EPI_ISL_424230	1463	YCTGSIPCSVCLSGLDSLDTYPSLETIQITISSFKWDLTAFGLVAEWFLAYILFTRFFYV
EPI_ISL_424231	1463	YCTGSIPCSVCLSGLDSLDTYPSLETIQITISSFKWDLTAFGLVAEWFLAYILFTRFFYV
EPI_ISL_424240	1463	YCTGSIPCSVCLSGLDSLDTYPSLETIQITISSFKWDLTAFGLVAEWFLAYILFTRFFYV
EPI_ISL_424241	1463	YCTGSIPCSVCLSGLDSLDTYPSLETIQITISSFKWDLTAFGLVAEWFLAYILFTRFFYV
EPI_ISL_424243	1463	YCTGSIPCSVCLSGLDSLDTYPSLETIQITISSFKWDLTAFGLVAEWFLAYILFTRFFYV
EPI_ISL_424247	1463	YCTGSIPCSVCLSGLDSLDTYPSLETIQITISSFKWDLTAFGLVAEWFLAYILFTRFFYV
EPI_ISL_424249	1463	YCTGSIPCSVCLSGLDSLDTYPSLETIQITISSFKWDLTAFGLVAEWFLAYILFTRFFYV
EPI_ISL_424250	1463	YCTGSIPCSVCLSGLDSLDTYPSLETIQITISSFKWDLTAFGLVAEWFLAYILFTRFFYV
EPI_ISL_424252	1463	YCTGSIPCSVCLSGLDSLDTYPSLETIQITISSFKWDLTAFGLVAEWFLAYILFTRFFYV
EPI_ISL_424258	1463	YCTGSIPCSVCLSGLDSLDTYPSLETIQITISSFKWDLTAFGLVAEWFLAYILFTRFFYV
EPI_ISL_424268	1463	YCTGSIPCSVCLSGLDSLDTYPSLETIQITISSFKWDLTAFGLVAEWFLAYILFTRFFYV
EPI_ISL_424269	1463	YCTGSIPCSVCLSGLDSLDTYPSLETIQITISSFKWDLTAFGLVAEWFLAYILFTRFFYV
EPI_ISL_424275	1463	YCTGSIPCSVCLSGLDSLDTYPSLETIQITISSFKWDLTAFGLVAEWFLAYILFTRFFYV
EPI_ISL_424276	1463	YCTGSIPCSVCLSGLDSLDTYPSLETIQITISSFKWDLTAFGLVAEWFLAYILFTRFFYV

EPI_ISL_424279 1463 YCTGSIPCSVCLSGLDSLDTYPSLETIQITISSFKWDLTAFGLVAEWFLAYILFTRFFYV

```
ORF1ab          2341 LGLAAIMQLFFSYFAVHFISNSWLMWLIINLVQMAPISAMVRMYIFFASFYYVWKSYVHV
EPI_ISL_424219 1523 LGLAAIMQLFFSYFAVHFISNSWLMWLIINLVQMAPISAMVRMYIFFASFYYVWKSYVHV
EPI_ISL_424222 1523 LGLAAIMQLFFSYFAVHFISNSWLMWLIINLVQMAPISAMVRMYIFFASFYYVWKSYVHV
EPI_ISL_424223 1523 LGLAAIMQLFFSYFAVHFISNSWLMWLIINLVQMAPISAMVRMYIFFASFYYVWKSYVHV
EPI_ISL_424226 1523 LGLAAIMQLFFSYFAVHFISNSWLMWLIINLVQMAPISAMVRMYIFFASFYYVWKSYVHV
EPI_ISL_424228 1523 LGLAAIMQLFFSYFAVHFISNSWLMWLIINLVQMAPISAMVRMYIFFASFYYVWKSYVHV
EPI_ISL_424230 1523 LGLAAIMQLFFSYFAVHFISNSWLMWLIINLVQMAPISAMVRMYIFFASFYYVWKSYVHV
EPI_ISL_424231 1523 LGLAAIMQLFFSYFAVHFISNSWLMWLIINLVQMAPISAMVRMYIFFASFYYVWKSYVHV
EPI_ISL_424240 1523 LGLAAIMQLFFSYFAVHFISNSWLMWLIINLVQMAPISAMVRMYIFFASFYYVWKSYVHV
EPI_ISL_424241 1523 LGLAAIMQLFFSYFAVHFISNSWLMWLIINLVQMAPISAMVRMYIFFASFYYVWKSYVHV
EPI_ISL_424243 1523 LGLAAIMQLFFSYFAVHFISNSWLMWLIINLVQMAPISAMVRMYIFFASFYYVWKSYVHV
EPI_ISL_424247 1523 LGLAAIMQLFFSYFAVHFISNSWLMWLIINLVQMAPISAMVRMYIFFASFYYVWKSYVHV
EPI_ISL_424249 1523 LGLAAIMQLFFSYFAVHFISNSWLMWLIINLVQMAPISAMVRMYIFFASFYYVWKSYVHV
EPI_ISL_424250 1523 LGLAAIMQLFFSYFAVHFISNSWLMWLIINLVQMAPISAMVRMYIFFASFYYVWKSYVHV
EPI_ISL_424252 1523 LGLAAIMQLFFSYFAVHFISNSWLMWLIINLVQMAPISAMVRMYIFFASFYYVWKSYVHV
EPI_ISL_424258 1523 LGLAAIMQLFFSYFAVHFISNSWLMWLIINLVQMAPISAMVRMYIFFASFYYVWKSYVHV
EPI_ISL_424268 1523 LGLAAIMQLFFSYFAVHFISNSWLMWLIINLVQMAPISAMVRMYIFFASFYYVWKSYVHV
EPI_ISL_424269 1523 LGLAAIMQLFFSYFAVHFISNSWLMWLIINLVQMAPISAMVRMYIFFASFYYVWKSYVHV
EPI_ISL_424275 1523 LGLAAIMQLFFSYFAVHFISNSWLMWLIINLVQMAPISAMVRMYIFFASFYYVWKSYVHV
EPI_ISL_424276 1523 LGLAAIMQLFFSYFAVHFISNSWLMWLIINLVQMAPISAMVRMYIFFASFYYVWKSYVHV
EPI_ISL_424279 1523 LGLAAIMQLFFSYFAVHFISNSWLMWLIINLVQMAPISAMVRMYIFFASFYYVWKSYVHV
```

```
ORF1ab          2401 VDGCNSSTCMMCYKRNRATRVECTTIVNGVRRSFYVYANGGKGFCKLHNWNCVNCDTFCA
EPI_ISL_424219 1583 VDGCNSSTCMMCYKRNRATRVECTTIVNGVRRSFYVYANGGKGFCKLHNWNCVNCDTFCA
EPI_ISL_424222 1583 VDGCNSSTCMMCYKRNRATRVECTTIVNGVRRSFYVYANGGKGFCKLHNWNCVNCDTFCA
EPI_ISL_424223 1583 VDGCNSSTCMMCYKRNRATRVECTTIVNGVRRSFYVYANGGKGFCKLHNWNCVNCDTFCA
EPI_ISL_424226 1583 VDGCNSSTCMMCYKRNRATRVECTTIVNGVRRSFYVYANGGKGFCKLHNWNCVNCDTFCA
EPI_ISL_424228 1583 VDGCNSSTCMMCYKRNRATRVECTTIVNGVRRSFYVYANGGKGFCKLHNWNCVNCDTFCA
EPI_ISL_424230 1583 VDGCNSSTCMMCYKRNRATRVECTTIVNGVRRSFYVYANGGKGFCKLHNWNCVNCDTFCA
EPI_ISL_424231 1583 VDGCNSSTCMMCYKRNRATRVECTTIVNGVRRSFYVYANGGKGFCKLHNWNCVNCDTFCA
EPI_ISL_424240 1583 VDGCNSSTCMMCYKRNRATRVECTTIVNGVRRSFYVYANGGKGFCKLHNWNCVNCDTFCA
EPI_ISL_424241 1583 VDGCNSSTCMMCYKRNRATRVECTTIVNGVRRSFYVYANGGKGFCKLHNWNCVNCDTFCA
EPI_ISL_424243 1583 VDGCNSSTCMMCYKRNRATRVECTTIVNGVRRSFYVYANGGKGFCKLHNWNCVNCDTFCA
EPI_ISL_424247 1583 VDGCNSSTCMMCYKRNRATRVECTTIVNGVRRSFYVYANGGKGFCKLHNWNCVNCDTFCA
EPI_ISL_424249 1583 VDGCNSSTCMMCYKRNRATRVECTTIVNGVRRSFYVYANGGKGFCKLHNWNCVNCDTFCA
EPI_ISL_424250 1583 VDGCNSSTCMMCYKRNRATRVECTTIVNGVRRSFYVYANGGKGFCKLHNWNCVNCDTFCA
EPI_ISL_424252 1583 VDGCNSSTCMMCYKRNRATRVECTTIVNGVRRSFYVYANGGKGFCKLHNWNCVNCDTFCA
EPI_ISL_424258 1583 VDGCNSSTCMMCYKRNRATRVECTTIVNGVRRSFYVYANGGKGFCKLHNWNCVNCDTFCA
EPI_ISL_424268 1583 VDGCNSSTCMMCYKRNRATRVECTTIVNGVRRSFYVYANGGKGFCKLHNWNCVNCDTFCA
EPI_ISL_424269 1583 VDGCNSSTCMMCYKRNRATRVECTTIVNGVRRSFYVYANGGKGFCKLHNWNCVNCDTFCA
EPI_ISL_424275 1583 VDGCNSSTCMMCYKRNRATRVECTTIVNGVRRSFYVYANGGKGFCKLHNWNCVNCDTFCA
EPI_ISL_424276 1583 VDGCNSSTCMMCYKRNRATRVECTTIVNGVRRSFYVYANGGKGFCKLHNWNCVNCDTFCA
EPI_ISL_424279 1583 VDGCNSSTCMMCYKRNRATRVECTTIVNGVRRSFYVYANGGKGFCKLHNWNCVNCDTFCA
```

```
ORF1ab          2461 GSTFISDEVARDLSLQFKRPINPTDQSSYIVDSVTVKNGSIHLYFDKAGQKTYERHSLSH
EPI_ISL_424219 1643 GSTFISDEVARDLSLQFKRPINPTDQSSYIVDSVTVKNGSIHLYFDKAGQKTYERHSLSH
EPI_ISL_424222 1643 GSTFISDEVARDLSLQFKRPINPTDQSSYIVDSVTVKNGSIHLYFDKAGQKTYERHSLSH
EPI_ISL_424223 1643 GSTFISDEVARDLSLQFKRPINPTDQSSYIVDSVTVKNGSIHLYFDKAGQKTYERHSLSH
EPI_ISL_424226 1643 GSTFISDEVARDLSLQFKRPINPTDQSSYIVDSVTVKNGSIHLYFDKAGQKTYERHSLSH
EPI_ISL_424228 1643 GSTFISDEVARDLSLQFKRPINPTDQSSYIVDSVTVKNGSIHLYFDKAGQKTYERHSLSH
EPI_ISL_424230 1643 GSTFISDEVARDLSLQFKRPINPTDQSSYIVDSVTVKNGSIHLYFDKAGQKTYERHSLSH
EPI_ISL_424231 1643 GSTFISDEVARDLSLQFKRPINPTDQSSYIVDSVTVKNGSIHLYFDKAGQKTYERHSLSH
EPI_ISL_424240 1643 GSTFISDEVARDLSLQFKRPINPTDQSSYIVDSVTVKNGSIHLYFDKAGQKTYERHSLSH
EPI_ISL_424241 1643 GSTFISDEVARDLSLQFKRPINPTDQSSYIVDSVTVKNGSIHLYFDKAGQKTYERHSLSH
EPI_ISL_424243 1643 GSTFISDEVARDLSLQFKRPINPTDQSSYIVDSVTVKNGSIHLYFDKAGQKTYERHSLSH
EPI_ISL_424247 1643 GSTFISDEVARDLSLQFKRPINPTDQSSYIVDSVTVKNGSIHLYFDKAGQKTYERHSLSH
EPI_ISL_424249 1643 GSTFISDEVARDLSLQFKRPINPTDQSSYIVDSVTVKNGSIHLYFDKAGQKTYERHSLSH
EPI_ISL_424250 1643 GSTFISDEVARDLSLQFKRPINPTDQSSYIVDSVTVKNGSIHLYFDKAGQKTYERHSLSH
EPI_ISL_424252 1643 GSTFISDEVARDLSLQFKRPINPTDQSSYIVDSVTVKNGSIHLYFDKAGQKTYERHSLSH
EPI_ISL_424258 1643 GSTFISDEVARDLSLQFKRPINPTDQSSYIVDSVTVKNGSIHLYFDKAGQKTYERHSLSH
EPI_ISL_424268 1643 GSTFISDEVARDLSLQFKRPINPTDQSSYIVDSVTVKNGSIHLYFDKAGQKTYERHSLSH
EPI_ISL_424269 1643 GSTFISDEVARDLSLQFKRPINPTDQSSYIVDSVTVKNGSIHLYFDKAGQKTYERHSLSH
EPI_ISL_424275 1643 GSTFISDEVARDLSLQFKRPINPTDQSSYIVDSVTVKNGSIHLYFDKAGQKTYERHSLSH
EPI_ISL_424276 1643 GSTFISDEVARDLSLQFKRPINPTDQSSYIVDSVTVKNGSIHLYFDKAGQKTYERHSLSH
```

EPI_ISL_424279 1643 GSTFISDEVARDLSLQFKRPINPTDQSSYIVDSVTVKNGSIHLYFDKAGQKTYERHSLSH

```
ORF1ab         2521 FVNLDNLRANNTKGSLPINVIVFDGKSKCEESSAKSASVYYSQLMCQPILLLDQALVSDV
EPI_ISL_424219 1703 FVNLDNLRANNTKGSLPINVIVFDGKSKCEESSAKSASVYYSQLMCQPILLLDQALVSDV
EPI_ISL_424222 1703 FVNLDNLRANNTKGSLPINVIVFDGKSKCEESSAKSASVYYSQLMCQPILLLDQALVSDV
EPI_ISL_424223 1703 FVNLDNLRANNTKGSLPINVIVFDGKSKCEESSAKSASVYYSQLMCQPILLLDQALVSDV
EPI_ISL_424226 1703 FVNLDNLRANNTKGSLPINVIVFDGKSKCEESSAKSASVYYSQLMCQPILLLDQALVSDV
EPI_ISL_424228 1703 FVNLDNLRANNTKGSLPINVIVFDGKSKCEESSAKSASVYYSQLMCQPILLLDQALVSDV
EPI_ISL_424230 1703 FVNLDNLRANNTKGSLPINVIVFDGKSKCEESSAKSASVYYSQLMCQPILLLDQALVSDV
EPI_ISL_424231 1703 FVNLDNLRANNTKGSLPINVIVFDGKSKCEESSAKSASVYYSQLMCQPILLLDQALVSDV
EPI_ISL_424240 1703 FVNLDNLRANNTKGSLPINVIVFDGKSKCEESSAKSASVYYSQLMCQPILLLDQALVSDV
EPI_ISL_424241 1703 FVNLDNLRANNTKGSLPINVIVFDGKSKCEESSAKSASVYYSQLMCQPILLLDQALVSDV
EPI_ISL_424243 1703 FVNLDNLRANNTKGSLPINVIVFDGKSKCEESSAKSASVYYSQLMCQPILLLDQALVSDV
EPI_ISL_424247 1703 FVNLDNLRANNTKGSLPINVIVFDGKSKCEESSAKSASVYYSQLMCQPILLLDQALVSDV
EPI_ISL_424249 1703 FVNLDNLRANNTKGSLPINVIVFDGKSKCEESSAKSASVYYSQLMCQPILLLDQALVSDV
EPI_ISL_424250 1703 FVNLDNLRANNTKGSLPINVIVFDGKSKCEESSAKSASVYYSQLMCQPILLLDQALVSDV
EPI_ISL_424252 1703 FVNLDNLRANNTKGSLPINVIVFDGKSKCEESSAKSASVYYSQLMCQPILLLDQALVSDV
EPI_ISL_424258 1703 FVNLDNLRANNTKGSLPINVIVFDGKSKCEESSAKSASVYYSQLMCQPILLLDQALVSDV
EPI_ISL_424268 1703 FVNLDNLRANNTKGSLPINVIVFDGKSKCEESSAKSASVYYSQLMCQPILLLDQALVSDV
EPI_ISL_424269 1703 FVNLDNLRANNTKGSLPINVIVFDGKSKCEESSAKSASVYYSQLMCQPILLLDQALVSDV
EPI_ISL_424275 1703 FVNLDNLRANNTKGSLPINVIVFDGKSKCEESSAKSASVYYSQLMCQPILLLDQALVSDV
EPI_ISL_424276 1703 FVNLDNLRANNTKGSLPINVIVFDGKSKCEESSAKSASVYYSQLMCQPILLLDQALVSDV
EPI_ISL_424279 1703 FVNLDNLRANNTKGSLPINVIVFDGKSKCEESSAKSASVYYSQLMCQPILLLDQALVSDV

ORF1ab         2581 GDSAEVAVKMFDAYVNTFSSTFNVPMEKLKTLVATAEAELAKNVSLDNVLSTFISAARQG
EPI_ISL_424219 1763 GDSAEVAVKMFDAYVNTFSSTFNVPMEKLKTLVATAEAELAKNVSLDNVLSTFISAARQG
EPI_ISL_424222 1763 GDSAEVAVKMFDAYVNTFSSTFNVPMEKLKTLVATAEAELAKNVSLDNVLSTFISAARQG
EPI_ISL_424223 1763 GDSAEVAVKMFDAYVNTFSSTFNVPMEKLKTLVATAEAELAKNVSLDNVLSTFISAARQG
EPI_ISL_424226 1763 GDSAEVAVKMFDAYVNTFSSTFNVPMEKLKTLVATAEAELAKNVSLDNVLSTFISAARQG
EPI_ISL_424228 1763 GDSAEVAVKMFDAYVNTFSSTFNVPMEKLKTLVATAEAELAKNVSLDNVLSTFISAARQG
EPI_ISL_424230 1763 GDSAEVAVKMFDAYVNTFSSTFNVPMEKLKTLVATAEAELAKNVSLDNVLSTFISAARQG
EPI_ISL_424231 1763 GDSAEVAVKMFDAYVNTFSSTFNVPMEKLKTLVATAEAELAKNVSLDNVLSTFISAARQG
EPI_ISL_424240 1763 GDSAEVAVKMFDAYVNTFSSTFNVPMEKLKTLVATAEAELAKNVSLDNVLSTFISAARQG
EPI_ISL_424241 1763 GDSAEVAVKMFDAYVNTFSSTFNVPMEKLKTLVATAEAELAKNVSLDNVLSTFISAARQG
EPI_ISL_424243 1763 GDSAEVAVKMFDAYVNTFSSTFNVPMEKLKTLVATAEAELAKNVSLDNVLSTFISAARQG
EPI_ISL_424247 1763 GDSAEVAVKMFDAYVNTFSSTFNVPMEKLKTLVATAEAELAKNVSLDNVLSTFISAARQG
EPI_ISL_424249 1763 GDSAEVAVKMFDAYVNTFSSTFNVPMEKLKTLVATAEAELAKNVSLDNVLSTFISAARQG
EPI_ISL_424250 1763 GDSAEVAVKMFDAYVNTFSSTFNVPMEKLKTLVATAEAELAKNVSLDNVLSTFISAARQG
EPI_ISL_424252 1763 GDSAEVAVKMFDAYVNTFSSTFNVPMEKLKTLVATAEAELAKNVSLDNVLSTFISAARQG
EPI_ISL_424258 1763 GDSAEVAVKMFDAYVNTFSSTFNVPMEKLKTLVATAEAELAKNVSLDNVLSTFISAARQG
EPI_ISL_424268 1763 GDSAEVAVKMFDAYVNTFSSTFNVPMEKLKTLVATAEAELAKNVSLDNVLSTFISAARQG
EPI_ISL_424269 1763 GDSAEVAVKMFDAYVNTFSSTFNVPMEKLKTLVATAEAELAKNVSLDNVLSTFISAARQG
EPI_ISL_424275 1763 GDSAEVAVKMFDAYVNTFSSTFNVPMEKLKTLVATAEAELAKNVSLDNVLSTFISAARQG
EPI_ISL_424276 1763 GDSAEVAVKMFDAYVNTFSSTFNVPMEKLKTLVATAEAELAKNVSLDNVLSTFISAARQG
EPI_ISL_424279 1763 GDSAEVAVKMFDAYVNTFSSTFNVPMEKLKTLVATAEAELAKNVSLDNVLSTFISAARQG

ORF1ab         2641 FVDSDVETKDVVECLKLSHQSDIEVTGDSCNNYMLTYNKVENMTPRDLGACIDCSARHIN
EPI_ISL_424219 1823 FVDSDVETKDVVECLKLSHQSDIEVTGDSCNNYMLTYNKVENMTPRDLGACIDCSARHIN
EPI_ISL_424222 1823 FVDSDVETKDVVECLKLSHQSDIEVTGDSCNNYMLTYNKVENMTPRDLGACIDCSARHIN
EPI_ISL_424223 1823 FVDSDVETKDVVECLKLSHQSDIEVTGDSCNNYMLTYNKVENMTPRDLGACIDCSARHIN
EPI_ISL_424226 1823 FVDSDVETKDVVECLKLSHQSDIEVTGDSCNNYMLTYNKVENMTPRDLGACIDCSARHIN
EPI_ISL_424228 1823 FVDSDVETKDVVECLKLSHQSDIEVTGDSCNNYMLTYNKVENMTPRDLGACIDCSARHIN
EPI_ISL_424230 1823 FVDSDVETKDVVECLKLSHQSDIEVTGDSCNNYMLTYNKVENMTPRDLGACIDCSARHIN
EPI_ISL_424231 1823 FVDSDVETKDVVECLKLSHQSDIEVTGDSCNNYMLTYNKVENMTPRDLGACIDCSARHIN
EPI_ISL_424240 1823 FVDSDVETKDVVECLKLSHQSDIEVTGDSCNNYMLTYNKVENMTPRDLGACIDCSARHIN
EPI_ISL_424241 1823 FVDSDVETKDVVECLKLSHQSDIEVTGDSCNNYMLTYNKVENMTPRDLGACIDCSARHIN
EPI_ISL_424243 1823 FVDSDVETKDVVECLKLSHQSDIEVTGDSCNNYMLTYNKVENMTPRDLGACIDCSARHIN
EPI_ISL_424247 1823 FVDSDVETKDVVECLKLSHQSDIEVTGDSCNNYMLTYNKVENMTPRDLGACIDCSARHIN
EPI_ISL_424249 1823 FVDSDVETKDVVECLKLSHQSDIEVTGDSCNNYMLTYNKVENMTPRDLGACIDCSARHIN
EPI_ISL_424250 1823 FVDSDVETKDVVECLKLSHQSDIEVTGDSCNNYMLTYNKVENMTPRDLGACIDCSARHIN
EPI_ISL_424252 1823 FVDSDVETKDVVECLKLSHQSDIEVTGDSCNNYMLTYNKVENMTPRDLGACIDCSARHIN
EPI_ISL_424258 1823 FVDSDVETKDVVECLKLSHQSDIEVTGDSCNNYMLTYNKVENMTPRDLGACIDCSARHIN
EPI_ISL_424268 1823 FVDSDVETKDVVECLKLSHQSDIEVTGDSCNNYMLTYNKVENMTPRDLGACIDCSARHIN
EPI_ISL_424269 1823 FVDSDVETKDVVECLKLSHQSDIEVTGDSCNNYMLTYNKVENMTPRDLGACIDCSARHIN
EPI_ISL_424275 1823 FVDSDVETKDVVECLKLSHQSDIEVTGDSCNNYMLTYNKVENMTPRDLGACIDCSARHIN
EPI_ISL_424276 1823 FVDSDVETKDVVECLKLSHQSDIEVTGDSCNNYMLTYNKVENMTPRDLGACIDCSARHIN
```

EPI_ISL_424279 1823 FVDSDVETKDVVECLKLSHQSDIEVTGDSCNNYMLTYNKVENMTPRDLGACIDCSARHIN

```
ORF1ab         2701 AQVAKSHNIALIWNVKDFMSLSEQLRKQIRSAAKKNNLPFKLTCATTRQVVNVVTTKIAL
EPI_ISL_424219 1883 AQVAKSHNIALIWNVKDFMSLSEQLRKQIRSAAKKNNLPFKLTCATTRQVVNVVTTKIAL
EPI_ISL_424222 1883 AQVAKSHNIALIWNVKDFMSLSEQLRKQIRSAAKKNNLPFKLTCATTRQVVNVVTTKIAL
EPI_ISL_424223 1883 AQVAKSHNIALIWNVKDFMSLSEQLRKQIRSAAKKNNLPFKLTCATTRQVVNVVTTKIAL
EPI_ISL_424226 1883 AQVAKSHNIALIWNVKDFMSLSEQLRKQIRSAAKKNNLPFKLTCATTRQVVNVVTTKIAL
EPI_ISL_424228 1883 AQVAKSHNIALIWNVKDFMSLSEQLRKQIRSAAKKNNLPFKLTCATTRQVVNVVTTKIAL
EPI_ISL_424230 1883 AQVAKSHNIALIWNVKDFMSLSEQLRKQIRSAAKKNNLPFKLTCATTRQVVNVVTTKIAL
EPI_ISL_424231 1883 AQVAKSHNIALIWNVKDFMSLSEQLRKQIRSAAKKNNLPFKLTCATTRQVVNVVTTKIAL
EPI_ISL_424240 1883 AQVAKSHNIALIWNVKDFMSLSEQLRKQIRSAAKKNNLPFKLTCATTRQVVNVVTTKIAL
EPI_ISL_424241 1883 AQVAKSHNIALIWNVKDFMSLSEQLRKQIRSAAKKNNLPFKLTCATTRQVVNVVTTKIAL
EPI_ISL_424243 1883 AQVAKSHNIALIWNVKDFMSLSEQLRKQIRSAAKKNNLPFKLTCATTRQVVNVVTTKIAL
EPI_ISL_424247 1883 AQVAKSHNIALIWNVKDFMSLSEQLRKQIRSAAKKNNLPFKLTCATTRQVVNVVTTKIAL
EPI_ISL_424249 1883 AQVAKSHNIALIWNVKDFMSLSEQLRKQIRSAAKKNNLPFKLTCATTRQVVNVVTTKIAL
EPI_ISL_424250 1883 AQVAKSHNIALIWNVKDFMSLSEQLRKQIRSAAKKNNLPFKLTCATTRQVVNVVTTKIAL
EPI_ISL_424252 1883 AQVAKSHNIALIWNVKDFMSLSEQLRKQIRSAAKKNNLPFKLTCATTRQVVNVVTTKIAL
EPI_ISL_424258 1883 AQVAKSHNIALIWNVKDFMSLSEQLRKQIRSAAKKNNLPFKLTCATTRQVVNVVTTKIAL
EPI_ISL_424268 1883 AQVAKSHNIALIWNVKDFMSLSEQLRKQIRSAAKKNNLPFKLTCATTRQVVNVVTTKIAL
EPI_ISL_424269 1883 AQVAKSHNIALIWNVKDFMSLSEQLRKQIRSAAKKNNLPFKLTCATTRQVVNVVTTKIAL
EPI_ISL_424275 1883 AQVAKSHNIALIWNVKDFMSLSEQLRKQIRSAAKKNNLPFKLTCATTRQVVNVVTTKIAL
EPI_ISL_424276 1883 AQVAKSHNIALIWNVKDFMSLSEQLRKQIRSAAKKNNLPFKLTCATTRQVVNVVTTKIAL
EPI_ISL_424279 1883 AQVAKSHNIALIWNVKDFMSLSEQLRKQIRSAAKKNNLPFKLTCATTRQVVNVVTTKIAL

ORF1ab         2761 KGGKIVNNWLKQLIKVTLVFLFVAAIFYLITPVHVMSKHTDFSSEIIGYKAIDGGVTRDI
EPI_ISL_424219 1943 KGG---------------------------------------------------------
EPI_ISL_424222 1943 KGG---------------------------------------------------------
EPI_ISL_424223 1943 KGG---------------------------------------------------------
EPI_ISL_424226 1943 KGG---------------------------------------------------------
EPI_ISL_424228 1943 KGG---------------------------------------------------------
EPI_ISL_424230 1943 KGG---------------------------------------------------------
EPI_ISL_424231 1943 KGG---------------------------------------------------------
EPI_ISL_424240 1943 KGG---------------------------------------------------------
EPI_ISL_424241 1943 KGG---------------------------------------------------------
EPI_ISL_424243 1943 KGG---------------------------------------------------------
EPI_ISL_424247 1943 KGG---------------------------------------------------------
EPI_ISL_424249 1943 KGG---------------------------------------------------------
EPI_ISL_424250 1943 KGG---------------------------------------------------------
EPI_ISL_424252 1943 KGG---------------------------------------------------------
EPI_ISL_424258 1943 KGG---------------------------------------------------------
EPI_ISL_424268 1943 KGG---------------------------------------------------------
EPI_ISL_424269 1943 KGG---------------------------------------------------------
EPI_ISL_424275 1943 KGG---------------------------------------------------------
EPI_ISL_424276 1943 KGG---------------------------------------------------------
EPI_ISL_424279 1943 KGG---------------------------------------------------------

ORF1ab         2821 ASTDTCFANKHADFDTWFSQRGGSYTNDKACPLIAAVITREVGFVVPGLPGTILRTTNGD
EPI_ISL_424219      ------------------------------------------------------------
EPI_ISL_424222      ------------------------------------------------------------
EPI_ISL_424223      ------------------------------------------------------------
EPI_ISL_424226      ------------------------------------------------------------
EPI_ISL_424228      ------------------------------------------------------------
EPI_ISL_424230      ------------------------------------------------------------
EPI_ISL_424231      ------------------------------------------------------------
EPI_ISL_424240      ------------------------------------------------------------
EPI_ISL_424241      ------------------------------------------------------------
EPI_ISL_424243      ------------------------------------------------------------
EPI_ISL_424247      ------------------------------------------------------------
EPI_ISL_424249      ------------------------------------------------------------
EPI_ISL_424250      ------------------------------------------------------------
EPI_ISL_424252      ------------------------------------------------------------
EPI_ISL_424258      ------------------------------------------------------------
EPI_ISL_424268      ------------------------------------------------------------
EPI_ISL_424269      ------------------------------------------------------------
EPI_ISL_424275      ------------------------------------------------------------
EPI_ISL_424276      ------------------------------------------------------------
```

EPI_ISL_424279   --

```
ORF1ab          2881 FLHFLPRVFSAVGNICYTPSKLIEYTDFATSACVLAAECTIFKDASGKPVPYCYDTNVLE
EPI_ISL_424219       ------------------------------------------------------------
EPI_ISL_424222       ------------------------------------------------------------
EPI_ISL_424223       ------------------------------------------------------------
EPI_ISL_424226       ------------------------------------------------------------
EPI_ISL_424228       ------------------------------------------------------------
EPI_ISL_424230       ------------------------------------------------------------
EPI_ISL_424231       ------------------------------------------------------------
EPI_ISL_424240       ------------------------------------------------------------
EPI_ISL_424241       ------------------------------------------------------------
EPI_ISL_424243       ------------------------------------------------------------
EPI_ISL_424247       ------------------------------------------------------------
EPI_ISL_424249       ------------------------------------------------------------
EPI_ISL_424250       ------------------------------------------------------------
EPI_ISL_424252       ------------------------------------------------------------
EPI_ISL_424258       ------------------------------------------------------------
EPI_ISL_424268       ------------------------------------------------------------
EPI_ISL_424269       ------------------------------------------------------------
EPI_ISL_424275       ------------------------------------------------------------
EPI_ISL_424276       ------------------------------------------------------------
EPI_ISL_424279       ------------------------------------------------------------

ORF1ab          2941 GSVAYESLRPDTRYVLMDGSIIQFPNTYLEGSVRVVTTFDSEYCRHGTCERSEAGVCVST
EPI_ISL_424219       ------------------------------------------------------------
EPI_ISL_424222       ------------------------------------------------------------
EPI_ISL_424223       ------------------------------------------------------------
EPI_ISL_424226       ------------------------------------------------------------
EPI_ISL_424228       ------------------------------------------------------------
EPI_ISL_424230       ------------------------------------------------------------
EPI_ISL_424231       ------------------------------------------------------------
EPI_ISL_424240       ------------------------------------------------------------
EPI_ISL_424241       ------------------------------------------------------------
EPI_ISL_424243       ------------------------------------------------------------
EPI_ISL_424247       ------------------------------------------------------------
EPI_ISL_424249       ------------------------------------------------------------
EPI_ISL_424250       ------------------------------------------------------------
EPI_ISL_424252       ------------------------------------------------------------
EPI_ISL_424258       ------------------------------------------------------------
EPI_ISL_424268       ------------------------------------------------------------
EPI_ISL_424269       ------------------------------------------------------------
EPI_ISL_424275       ------------------------------------------------------------
EPI_ISL_424276       ------------------------------------------------------------
EPI_ISL_424279       ------------------------------------------------------------

ORF1ab          3001 SGRWVLNNDYYRSLPGVFCGVDAVNLLTNMFTPLIQPIGALDISASIVAGGIVAIVVTCL
EPI_ISL_424219       ------------------------------------------------------------
EPI_ISL_424222       ------------------------------------------------------------
EPI_ISL_424223       ------------------------------------------------------------
EPI_ISL_424226       ------------------------------------------------------------
EPI_ISL_424228       ------------------------------------------------------------
EPI_ISL_424230       ------------------------------------------------------------
EPI_ISL_424231       ------------------------------------------------------------
EPI_ISL_424240       ------------------------------------------------------------
EPI_ISL_424241       ------------------------------------------------------------
EPI_ISL_424243       ------------------------------------------------------------
EPI_ISL_424247       ------------------------------------------------------------
EPI_ISL_424249       ------------------------------------------------------------
EPI_ISL_424250       ------------------------------------------------------------
EPI_ISL_424252       ------------------------------------------------------------
EPI_ISL_424258       ------------------------------------------------------------
EPI_ISL_424268       ------------------------------------------------------------
EPI_ISL_424269       ------------------------------------------------------------
EPI_ISL_424275       ------------------------------------------------------------
EPI_ISL_424276       ------------------------------------------------------------
```

EPI_ISL_424279   --

```
ORF1ab         3061 AYYFMRFRRAFGEYSHVVAFNTLLFLMSFTVLCLTPVYSFLPGVYSVIYLYLTFYLTNDV
EPI_ISL_424219      ------------------------------------------------------------
EPI_ISL_424222      ------------------------------------------------------------
EPI_ISL_424223      ------------------------------------------------------------
EPI_ISL_424226      ------------------------------------------------------------
EPI_ISL_424228      ------------------------------------------------------------
EPI_ISL_424230      ------------------------------------------------------------
EPI_ISL_424231      ------------------------------------------------------------
EPI_ISL_424240      ------------------------------------------------------------
EPI_ISL_424241      ------------------------------------------------------------
EPI_ISL_424243      ------------------------------------------------------------
EPI_ISL_424247      ------------------------------------------------------------
EPI_ISL_424249      ------------------------------------------------------------
EPI_ISL_424250      ------------------------------------------------------------
EPI_ISL_424252      ------------------------------------------------------------
EPI_ISL_424258      ------------------------------------------------------------
EPI_ISL_424268      ------------------------------------------------------------
EPI_ISL_424269      ------------------------------------------------------------
EPI_ISL_424275      ------------------------------------------------------------
EPI_ISL_424276      ------------------------------------------------------------
EPI_ISL_424279      ------------------------------------------------------------

ORF1ab         3121 SFLAHIQWMVMFTPLVPFWITIAYIICISTKHFYWFFSNYLKRRVVFNGVSFSTFEEAAL
EPI_ISL_424219      ------------------------------------------------------------
EPI_ISL_424222      ------------------------------------------------------------
EPI_ISL_424223      ------------------------------------------------------------
EPI_ISL_424226      ------------------------------------------------------------
EPI_ISL_424228      ------------------------------------------------------------
EPI_ISL_424230      ------------------------------------------------------------
EPI_ISL_424231      ------------------------------------------------------------
EPI_ISL_424240      ------------------------------------------------------------
EPI_ISL_424241      ------------------------------------------------------------
EPI_ISL_424243      ------------------------------------------------------------
EPI_ISL_424247      ------------------------------------------------------------
EPI_ISL_424249      ------------------------------------------------------------
EPI_ISL_424250      ------------------------------------------------------------
EPI_ISL_424252      ------------------------------------------------------------
EPI_ISL_424258      ------------------------------------------------------------
EPI_ISL_424268      ------------------------------------------------------------
EPI_ISL_424269      ------------------------------------------------------------
EPI_ISL_424275      ------------------------------------------------------------
EPI_ISL_424276      ------------------------------------------------------------
EPI_ISL_424279      ------------------------------------------------------------

ORF1ab         3181 CTFLLNKEMYLKLRSDVLLPLTQYNRYLALYNKYKYFSGAMDTTSYREAACCHLAKALND
EPI_ISL_424219      ------------------------------------------------------------
EPI_ISL_424222      ------------------------------------------------------------
EPI_ISL_424223      ------------------------------------------------------------
EPI_ISL_424226      ------------------------------------------------------------
EPI_ISL_424228      ------------------------------------------------------------
EPI_ISL_424230      ------------------------------------------------------------
EPI_ISL_424231      ------------------------------------------------------------
EPI_ISL_424240      ------------------------------------------------------------
EPI_ISL_424241      ------------------------------------------------------------
EPI_ISL_424243      ------------------------------------------------------------
EPI_ISL_424247      ------------------------------------------------------------
EPI_ISL_424249      ------------------------------------------------------------
EPI_ISL_424250      ------------------------------------------------------------
EPI_ISL_424252      ------------------------------------------------------------
EPI_ISL_424258      ------------------------------------------------------------
EPI_ISL_424268      ------------------------------------------------------------
EPI_ISL_424269      ------------------------------------------------------------
EPI_ISL_424275      ------------------------------------------------------------
EPI_ISL_424276      ------------------------------------------------------------
```

EPI_ISL_424279   --

```
ORF1ab          3241 FSNSGSDVLYQPPQTSITSAVLQSGFRKMAFPSGKVEGCMVQVTCGTTTLNGLWLDDVVY
EPI_ISL_424219       ------------------------------------------------------------
EPI_ISL_424222       ------------------------------------------------------------
EPI_ISL_424223       ------------------------------------------------------------
EPI_ISL_424226       ------------------------------------------------------------
EPI_ISL_424228       ------------------------------------------------------------
EPI_ISL_424230       ------------------------------------------------------------
EPI_ISL_424231       ------------------------------------------------------------
EPI_ISL_424240       ------------------------------------------------------------
EPI_ISL_424241       ------------------------------------------------------------
EPI_ISL_424243       ------------------------------------------------------------
EPI_ISL_424247       ------------------------------------------------------------
EPI_ISL_424249       ------------------------------------------------------------
EPI_ISL_424250       ------------------------------------------------------------
EPI_ISL_424252       ------------------------------------------------------------
EPI_ISL_424258       ------------------------------------------------------------
EPI_ISL_424268       ------------------------------------------------------------
EPI_ISL_424269       ------------------------------------------------------------
EPI_ISL_424275       ------------------------------------------------------------
EPI_ISL_424276       ------------------------------------------------------------
EPI_ISL_424279       ------------------------------------------------------------

ORF1ab          3301 CPRHVICTSEDMLNPNYEDLLIRKSNHNFLVQAGNVQLRVIGHSMQNCVLKLKVDTANPK
EPI_ISL_424219       ------------------------------------------------------------
EPI_ISL_424222       ------------------------------------------------------------
EPI_ISL_424223       ------------------------------------------------------------
EPI_ISL_424226       ------------------------------------------------------------
EPI_ISL_424228       ------------------------------------------------------------
EPI_ISL_424230       ------------------------------------------------------------
EPI_ISL_424231       ------------------------------------------------------------
EPI_ISL_424240       ------------------------------------------------------------
EPI_ISL_424241       ------------------------------------------------------------
EPI_ISL_424243       ------------------------------------------------------------
EPI_ISL_424247       ------------------------------------------------------------
EPI_ISL_424249       ------------------------------------------------------------
EPI_ISL_424250       ------------------------------------------------------------
EPI_ISL_424252       ------------------------------------------------------------
EPI_ISL_424258       ------------------------------------------------------------
EPI_ISL_424268       ------------------------------------------------------------
EPI_ISL_424269       ------------------------------------------------------------
EPI_ISL_424275       ------------------------------------------------------------
EPI_ISL_424276       ------------------------------------------------------------
EPI_ISL_424279       ------------------------------------------------------------

ORF1ab          3361 TPKYKFVRIQPGQTFSVLACYNGSPSGVYQCAMRPNFTIKGSFLNGSCGSVGFNIDYDCV
EPI_ISL_424219       ------------------------------------------------------------
EPI_ISL_424222       ------------------------------------------------------------
EPI_ISL_424223       ------------------------------------------------------------
EPI_ISL_424226       ------------------------------------------------------------
EPI_ISL_424228       ------------------------------------------------------------
EPI_ISL_424230       ------------------------------------------------------------
EPI_ISL_424231       ------------------------------------------------------------
EPI_ISL_424240       ------------------------------------------------------------
EPI_ISL_424241       ------------------------------------------------------------
EPI_ISL_424243       ------------------------------------------------------------
EPI_ISL_424247       ------------------------------------------------------------
EPI_ISL_424249       ------------------------------------------------------------
EPI_ISL_424250       ------------------------------------------------------------
EPI_ISL_424252       ------------------------------------------------------------
EPI_ISL_424258       ------------------------------------------------------------
EPI_ISL_424268       ------------------------------------------------------------
EPI_ISL_424269       ------------------------------------------------------------
EPI_ISL_424275       ------------------------------------------------------------
EPI_ISL_424276       ------------------------------------------------------------
```

EPI_ISL_424279 --

```
ORF1ab          3421 SFCYMHHMELPTGVHAGTDLEGNFYGPFVDRQTAQAAGTDTTITVNVLAWLYAAVINGDR
EPI_ISL_424219       ------------------------------------------------------------
EPI_ISL_424222       ------------------------------------------------------------
EPI_ISL_424223       ------------------------------------------------------------
EPI_ISL_424226       ------------------------------------------------------------
EPI_ISL_424228       ------------------------------------------------------------
EPI_ISL_424230       ------------------------------------------------------------
EPI_ISL_424231       ------------------------------------------------------------
EPI_ISL_424240       ------------------------------------------------------------
EPI_ISL_424241       ------------------------------------------------------------
EPI_ISL_424243       ------------------------------------------------------------
EPI_ISL_424247       ------------------------------------------------------------
EPI_ISL_424249       ------------------------------------------------------------
EPI_ISL_424250       ------------------------------------------------------------
EPI_ISL_424252       ------------------------------------------------------------
EPI_ISL_424258       ------------------------------------------------------------
EPI_ISL_424268       ------------------------------------------------------------
EPI_ISL_424269       ------------------------------------------------------------
EPI_ISL_424275       ------------------------------------------------------------
EPI_ISL_424276       ------------------------------------------------------------
EPI_ISL_424279       ------------------------------------------------------------
```

```
ORF1ab          3481 WFLNRFTTTLNDFNLVAMKYNYEPLTQDHVDILGPLSAQTGIAVLDMCASLKELLQNGMN
EPI_ISL_424219       ------------------------------------------------------------
EPI_ISL_424222       ------------------------------------------------------------
EPI_ISL_424223       ------------------------------------------------------------
EPI_ISL_424226       ------------------------------------------------------------
EPI_ISL_424228       ------------------------------------------------------------
EPI_ISL_424230       ------------------------------------------------------------
EPI_ISL_424231       ------------------------------------------------------------
EPI_ISL_424240       ------------------------------------------------------------
EPI_ISL_424241       ------------------------------------------------------------
EPI_ISL_424243       ------------------------------------------------------------
EPI_ISL_424247       ------------------------------------------------------------
EPI_ISL_424249       ------------------------------------------------------------
EPI_ISL_424250       ------------------------------------------------------------
EPI_ISL_424252       ------------------------------------------------------------
EPI_ISL_424258       ------------------------------------------------------------
EPI_ISL_424268       ------------------------------------------------------------
EPI_ISL_424269       ------------------------------------------------------------
EPI_ISL_424275       ------------------------------------------------------------
EPI_ISL_424276       ------------------------------------------------------------
EPI_ISL_424279       ------------------------------------------------------------
```

```
ORF1ab          3541 GRTILGSALLEDEFTPFDVVRQCSGVTFQSAVKRTIKGTHHWLLLTILTSLLVLVQSTQW
EPI_ISL_424219       ------------------------------------------------------------
EPI_ISL_424222       ------------------------------------------------------------
EPI_ISL_424223       ------------------------------------------------------------
EPI_ISL_424226       ------------------------------------------------------------
EPI_ISL_424228       ------------------------------------------------------------
EPI_ISL_424230       ------------------------------------------------------------
EPI_ISL_424231       ------------------------------------------------------------
EPI_ISL_424240       ------------------------------------------------------------
EPI_ISL_424241       ------------------------------------------------------------
EPI_ISL_424243       ------------------------------------------------------------
EPI_ISL_424247       ------------------------------------------------------------
EPI_ISL_424249       ------------------------------------------------------------
EPI_ISL_424250       ------------------------------------------------------------
EPI_ISL_424252       ------------------------------------------------------------
EPI_ISL_424258       ------------------------------------------------------------
EPI_ISL_424268       ------------------------------------------------------------
EPI_ISL_424269       ------------------------------------------------------------
EPI_ISL_424275       ------------------------------------------------------------
EPI_ISL_424276       ------------------------------------------------------------
```

EPI_ISL_424279 --

```
ORF1ab          3601 SLFFFLYENAFLPFAMGIIAMSAFAMMFVKHKHAFLCLFLLPSLATVAYFNMVYMPASWV
EPI_ISL_424219       ------------------------------------------------------------
EPI_ISL_424222       ------------------------------------------------------------
EPI_ISL_424223       ------------------------------------------------------------
EPI_ISL_424226       ------------------------------------------------------------
EPI_ISL_424228       ------------------------------------------------------------
EPI_ISL_424230       ------------------------------------------------------------
EPI_ISL_424231       ------------------------------------------------------------
EPI_ISL_424240       ------------------------------------------------------------
EPI_ISL_424241       ------------------------------------------------------------
EPI_ISL_424243       ------------------------------------------------------------
EPI_ISL_424247       ------------------------------------------------------------
EPI_ISL_424249       ------------------------------------------------------------
EPI_ISL_424250       ------------------------------------------------------------
EPI_ISL_424252       ------------------------------------------------------------
EPI_ISL_424258       ------------------------------------------------------------
EPI_ISL_424268       ------------------------------------------------------------
EPI_ISL_424269       ------------------------------------------------------------
EPI_ISL_424275       ------------------------------------------------------------
EPI_ISL_424276       ------------------------------------------------------------
EPI_ISL_424279       ------------------------------------------------------------

ORF1ab          3661 MRIMTWLDMVDTSLSGFKLKDCVMYASAVVLLILMTARTVYDDGARRVWTLMNVLTLVYK
EPI_ISL_424219       ------------------------------------------------------------
EPI_ISL_424222       ------------------------------------------------------------
EPI_ISL_424223       ------------------------------------------------------------
EPI_ISL_424226       ------------------------------------------------------------
EPI_ISL_424228       ------------------------------------------------------------
EPI_ISL_424230       ------------------------------------------------------------
EPI_ISL_424231       ------------------------------------------------------------
EPI_ISL_424240       ------------------------------------------------------------
EPI_ISL_424241       ------------------------------------------------------------
EPI_ISL_424243       ------------------------------------------------------------
EPI_ISL_424247       ------------------------------------------------------------
EPI_ISL_424249       ------------------------------------------------------------
EPI_ISL_424250       ------------------------------------------------------------
EPI_ISL_424252       ------------------------------------------------------------
EPI_ISL_424258       ------------------------------------------------------------
EPI_ISL_424268       ------------------------------------------------------------
EPI_ISL_424269       ------------------------------------------------------------
EPI_ISL_424275       ------------------------------------------------------------
EPI_ISL_424276       ------------------------------------------------------------
EPI_ISL_424279       ------------------------------------------------------------

ORF1ab          3721 VYYGNALDQAISMWALIISVTSNYSGVVTTVMFLARGIVFMCVEYCPIFFITGNTLQCIM
EPI_ISL_424219       ------------------------------------------------------------
EPI_ISL_424222       ------------------------------------------------------------
EPI_ISL_424223       ------------------------------------------------------------
EPI_ISL_424226       ------------------------------------------------------------
EPI_ISL_424228       ------------------------------------------------------------
EPI_ISL_424230       ------------------------------------------------------------
EPI_ISL_424231       ------------------------------------------------------------
EPI_ISL_424240       ------------------------------------------------------------
EPI_ISL_424241       ------------------------------------------------------------
EPI_ISL_424243       ------------------------------------------------------------
EPI_ISL_424247       ------------------------------------------------------------
EPI_ISL_424249       ------------------------------------------------------------
EPI_ISL_424250       ------------------------------------------------------------
EPI_ISL_424252       ------------------------------------------------------------
EPI_ISL_424258       ------------------------------------------------------------
EPI_ISL_424268       ------------------------------------------------------------
EPI_ISL_424269       ------------------------------------------------------------
EPI_ISL_424275       ------------------------------------------------------------
EPI_ISL_424276       ------------------------------------------------------------
```

EPI_ISL_424279 --

```
ORF1ab         3781 LVYCFLGYFCTCYFGLFCLLNRYFRLTLGVYDYLVSTQEFRYMNSQGLLPPKNSIDAFKL
EPI_ISL_424219      ------------------------------------------------------------
EPI_ISL_424222      ------------------------------------------------------------
EPI_ISL_424223      ------------------------------------------------------------
EPI_ISL_424226      ------------------------------------------------------------
EPI_ISL_424228      ------------------------------------------------------------
EPI_ISL_424230      ------------------------------------------------------------
EPI_ISL_424231      ------------------------------------------------------------
EPI_ISL_424240      ------------------------------------------------------------
EPI_ISL_424241      ------------------------------------------------------------
EPI_ISL_424243      ------------------------------------------------------------
EPI_ISL_424247      ------------------------------------------------------------
EPI_ISL_424249      ------------------------------------------------------------
EPI_ISL_424250      ------------------------------------------------------------
EPI_ISL_424252      ------------------------------------------------------------
EPI_ISL_424258      ------------------------------------------------------------
EPI_ISL_424268      ------------------------------------------------------------
EPI_ISL_424269      ------------------------------------------------------------
EPI_ISL_424275      ------------------------------------------------------------
EPI_ISL_424276      ------------------------------------------------------------
EPI_ISL_424279      ------------------------------------------------------------

ORF1ab         3841 NIKLLGVGGKPCIKVATVQSKMSDVKCTSVVLLSVLQQLRVESSSKLWAQCVQLHNDILL
EPI_ISL_424219      ------------------------------------------------------------
EPI_ISL_424222      ------------------------------------------------------------
EPI_ISL_424223      ------------------------------------------------------------
EPI_ISL_424226      ------------------------------------------------------------
EPI_ISL_424228      ------------------------------------------------------------
EPI_ISL_424230      ------------------------------------------------------------
EPI_ISL_424231      ------------------------------------------------------------
EPI_ISL_424240      ------------------------------------------------------------
EPI_ISL_424241      ------------------------------------------------------------
EPI_ISL_424243      ------------------------------------------------------------
EPI_ISL_424247      ------------------------------------------------------------
EPI_ISL_424249      ------------------------------------------------------------
EPI_ISL_424250      ------------------------------------------------------------
EPI_ISL_424252      ------------------------------------------------------------
EPI_ISL_424258      ------------------------------------------------------------
EPI_ISL_424268      ------------------------------------------------------------
EPI_ISL_424269      ------------------------------------------------------------
EPI_ISL_424275      ------------------------------------------------------------
EPI_ISL_424276      ------------------------------------------------------------
EPI_ISL_424279      ------------------------------------------------------------

ORF1ab         3901 AKDTTEAFEKMVSLLSVLLSMQGAVDINKLCEEMLDNRATLQAIASEFSSLPSYAAFATA
EPI_ISL_424219      ------------------------------------------------------------
EPI_ISL_424222      ------------------------------------------------------------
EPI_ISL_424223      ------------------------------------------------------------
EPI_ISL_424226      ------------------------------------------------------------
EPI_ISL_424228      ------------------------------------------------------------
EPI_ISL_424230      ------------------------------------------------------------
EPI_ISL_424231      ------------------------------------------------------------
EPI_ISL_424240      ------------------------------------------------------------
EPI_ISL_424241      ------------------------------------------------------------
EPI_ISL_424243      ------------------------------------------------------------
EPI_ISL_424247      ------------------------------------------------------------
EPI_ISL_424249      ------------------------------------------------------------
EPI_ISL_424250      ------------------------------------------------------------
EPI_ISL_424252      ------------------------------------------------------------
EPI_ISL_424258      ------------------------------------------------------------
EPI_ISL_424268      ------------------------------------------------------------
EPI_ISL_424269      ------------------------------------------------------------
EPI_ISL_424275      ------------------------------------------------------------
EPI_ISL_424276      ------------------------------------------------------------
```

EPI_ISL_424279 --

```
ORF1ab          3961 QEAYEQAVANGDSEVVLKKLKKSLNVAKSEFDRDAAMQRKLEKMADQAMTQMYKQARSED
EPI_ISL_424219       ------------------------------------------------------------
EPI_ISL_424222       ------------------------------------------------------------
EPI_ISL_424223       ------------------------------------------------------------
EPI_ISL_424226       ------------------------------------------------------------
EPI_ISL_424228       ------------------------------------------------------------
EPI_ISL_424230       ------------------------------------------------------------
EPI_ISL_424231       ------------------------------------------------------------
EPI_ISL_424240       ------------------------------------------------------------
EPI_ISL_424241       ------------------------------------------------------------
EPI_ISL_424243       ------------------------------------------------------------
EPI_ISL_424247       ------------------------------------------------------------
EPI_ISL_424249       ------------------------------------------------------------
EPI_ISL_424250       ------------------------------------------------------------
EPI_ISL_424252       ------------------------------------------------------------
EPI_ISL_424258       ------------------------------------------------------------
EPI_ISL_424268       ------------------------------------------------------------
EPI_ISL_424269       ------------------------------------------------------------
EPI_ISL_424275       ------------------------------------------------------------
EPI_ISL_424276       ------------------------------------------------------------
EPI_ISL_424279       ------------------------------------------------------------

ORF1ab          4021 KRAKVTSAMQTMLFTMLRKLDNDALNNIINNARDGCVPLNIIPLTTAAKLMVVIPDYNTY
EPI_ISL_424219       ------------------------------------------------------------
EPI_ISL_424222       ------------------------------------------------------------
EPI_ISL_424223       ------------------------------------------------------------
EPI_ISL_424226       ------------------------------------------------------------
EPI_ISL_424228       ------------------------------------------------------------
EPI_ISL_424230       ------------------------------------------------------------
EPI_ISL_424231       ------------------------------------------------------------
EPI_ISL_424240       ------------------------------------------------------------
EPI_ISL_424241       ------------------------------------------------------------
EPI_ISL_424243       ------------------------------------------------------------
EPI_ISL_424247       ------------------------------------------------------------
EPI_ISL_424249       ------------------------------------------------------------
EPI_ISL_424250       ------------------------------------------------------------
EPI_ISL_424252       ------------------------------------------------------------
EPI_ISL_424258       ------------------------------------------------------------
EPI_ISL_424268       ------------------------------------------------------------
EPI_ISL_424269       ------------------------------------------------------------
EPI_ISL_424275       ------------------------------------------------------------
EPI_ISL_424276       ------------------------------------------------------------
EPI_ISL_424279       ------------------------------------------------------------

ORF1ab          4081 KNTCDGTTFTYASALWEIQQVVDADSKIVQLSEISMDNSPNLAWPLIVTALRANSAVKLQ
EPI_ISL_424219       ------------------------------------------------------------
EPI_ISL_424222       ------------------------------------------------------------
EPI_ISL_424223       ------------------------------------------------------------
EPI_ISL_424226       ------------------------------------------------------------
EPI_ISL_424228       ------------------------------------------------------------
EPI_ISL_424230       ------------------------------------------------------------
EPI_ISL_424231       ------------------------------------------------------------
EPI_ISL_424240       ------------------------------------------------------------
EPI_ISL_424241       ------------------------------------------------------------
EPI_ISL_424243       ------------------------------------------------------------
EPI_ISL_424247       ------------------------------------------------------------
EPI_ISL_424249       ------------------------------------------------------------
EPI_ISL_424250       ------------------------------------------------------------
EPI_ISL_424252       ------------------------------------------------------------
EPI_ISL_424258       ------------------------------------------------------------
EPI_ISL_424268       ------------------------------------------------------------
EPI_ISL_424269       ------------------------------------------------------------
EPI_ISL_424275       ------------------------------------------------------------
EPI_ISL_424276       ------------------------------------------------------------
```

EPI_ISL_424279 --

```
ORF1ab         4141 NNELSPVALRQMSCAAGTTQTACTDDNALAYYNTTKGGRFVLALLSDLQDLKWARFPKSD
EPI_ISL_424219      ------------------------------------------------------------
EPI_ISL_424222      ------------------------------------------------------------
EPI_ISL_424223      ------------------------------------------------------------
EPI_ISL_424226      ------------------------------------------------------------
EPI_ISL_424228      ------------------------------------------------------------
EPI_ISL_424230      ------------------------------------------------------------
EPI_ISL_424231      ------------------------------------------------------------
EPI_ISL_424240      ------------------------------------------------------------
EPI_ISL_424241      ------------------------------------------------------------
EPI_ISL_424243      ------------------------------------------------------------
EPI_ISL_424247      ------------------------------------------------------------
EPI_ISL_424249      ------------------------------------------------------------
EPI_ISL_424250      ------------------------------------------------------------
EPI_ISL_424252      ------------------------------------------------------------
EPI_ISL_424258      ------------------------------------------------------------
EPI_ISL_424268      ------------------------------------------------------------
EPI_ISL_424269      ------------------------------------------------------------
EPI_ISL_424275      ------------------------------------------------------------
EPI_ISL_424276      ------------------------------------------------------------
EPI_ISL_424279      ------------------------------------------------------------

ORF1ab         4201 GTGTIYTELEPPCRFVTDTPKGPKVKYLYFIKGLNNLNRGMVLGSLAATVRLQAGNATEV
EPI_ISL_424219      ------------------------------------------------------------
EPI_ISL_424222      ------------------------------------------------------------
EPI_ISL_424223      ------------------------------------------------------------
EPI_ISL_424226      ------------------------------------------------------------
EPI_ISL_424228      ------------------------------------------------------------
EPI_ISL_424230      ------------------------------------------------------------
EPI_ISL_424231      ------------------------------------------------------------
EPI_ISL_424240      ------------------------------------------------------------
EPI_ISL_424241      ------------------------------------------------------------
EPI_ISL_424243      ------------------------------------------------------------
EPI_ISL_424247      ------------------------------------------------------------
EPI_ISL_424249      ------------------------------------------------------------
EPI_ISL_424250      ------------------------------------------------------------
EPI_ISL_424252      ------------------------------------------------------------
EPI_ISL_424258      ------------------------------------------------------------
EPI_ISL_424268      ------------------------------------------------------------
EPI_ISL_424269      ------------------------------------------------------------
EPI_ISL_424275      ------------------------------------------------------------
EPI_ISL_424276      ------------------------------------------------------------
EPI_ISL_424279      ------------------------------------------------------------

ORF1ab         4261 PANSTVLSFCAFAVDAAKAYKDYLASGGQPITNCVKMLCTHTGTGQAITVTPEANMDQES
EPI_ISL_424219      ------------------------------------------------------------
EPI_ISL_424222      ------------------------------------------------------------
EPI_ISL_424223      ------------------------------------------------------------
EPI_ISL_424226      ------------------------------------------------------------
EPI_ISL_424228      ------------------------------------------------------------
EPI_ISL_424230      ------------------------------------------------------------
EPI_ISL_424231      ------------------------------------------------------------
EPI_ISL_424240      ------------------------------------------------------------
EPI_ISL_424241      ------------------------------------------------------------
EPI_ISL_424243      ------------------------------------------------------------
EPI_ISL_424247      ------------------------------------------------------------
EPI_ISL_424249      ------------------------------------------------------------
EPI_ISL_424250      ------------------------------------------------------------
EPI_ISL_424252      ------------------------------------------------------------
EPI_ISL_424258      ------------------------------------------------------------
EPI_ISL_424268      ------------------------------------------------------------
EPI_ISL_424269      ------------------------------------------------------------
EPI_ISL_424275      ------------------------------------------------------------
EPI_ISL_424276      ------------------------------------------------------------
```

EPI_ISL_424279   --

```
ORF1ab          4321 FGGASCCLYCRCHIDHPNPKGFCDLKGKYVQIPTTCANDPVGFTLKNTVCTVCGMWKGYG
EPI_ISL_424219       ------------------------------------------------------------
EPI_ISL_424222       ------------------------------------------------------------
EPI_ISL_424223       ------------------------------------------------------------
EPI_ISL_424226       ------------------------------------------------------------
EPI_ISL_424228       ------------------------------------------------------------
EPI_ISL_424230       ------------------------------------------------------------
EPI_ISL_424231       ------------------------------------------------------------
EPI_ISL_424240       ------------------------------------------------------------
EPI_ISL_424241       ------------------------------------------------------------
EPI_ISL_424243       ------------------------------------------------------------
EPI_ISL_424247       ------------------------------------------------------------
EPI_ISL_424249       ------------------------------------------------------------
EPI_ISL_424250       ------------------------------------------------------------
EPI_ISL_424252       ------------------------------------------------------------
EPI_ISL_424258       ------------------------------------------------------------
EPI_ISL_424268       ------------------------------------------------------------
EPI_ISL_424269       ------------------------------------------------------------
EPI_ISL_424275       ------------------------------------------------------------
EPI_ISL_424276       ------------------------------------------------------------
EPI_ISL_424279       ------------------------------------------------------------

ORF1ab          4381 CSCDQLREPMLQSADAQSFLNRVCGVSAARLTPCGTGTSTDVVYRAFDIYNDKVAGFAKF
EPI_ISL_424219       ------------------------------------------------------------
EPI_ISL_424222       ------------------------------------------------------------
EPI_ISL_424223       ------------------------------------------------------------
EPI_ISL_424226       ------------------------------------------------------------
EPI_ISL_424228       ------------------------------------------------------------
EPI_ISL_424230       ------------------------------------------------------------
EPI_ISL_424231       ------------------------------------------------------------
EPI_ISL_424240       ------------------------------------------------------------
EPI_ISL_424241       ------------------------------------------------------------
EPI_ISL_424243       ------------------------------------------------------------
EPI_ISL_424247       ------------------------------------------------------------
EPI_ISL_424249       ------------------------------------------------------------
EPI_ISL_424250       ------------------------------------------------------------
EPI_ISL_424252       ------------------------------------------------------------
EPI_ISL_424258       ------------------------------------------------------------
EPI_ISL_424268       ------------------------------------------------------------
EPI_ISL_424269       ------------------------------------------------------------
EPI_ISL_424275       ------------------------------------------------------------
EPI_ISL_424276       ------------------------------------------------------------
EPI_ISL_424279       ------------------------------------------------------------

ORF1ab          4441 LKTNCCRFQEKDEDDNLIDSYFVVKRHTFSNYQHEETIYNLLKDCPAVAKHDFFKFRIDG
EPI_ISL_424219       ------------------------------------------------------------
EPI_ISL_424222       ------------------------------------------------------------
EPI_ISL_424223       ------------------------------------------------------------
EPI_ISL_424226       ------------------------------------------------------------
EPI_ISL_424228       ------------------------------------------------------------
EPI_ISL_424230       ------------------------------------------------------------
EPI_ISL_424231       ------------------------------------------------------------
EPI_ISL_424240       ------------------------------------------------------------
EPI_ISL_424241       ------------------------------------------------------------
EPI_ISL_424243       ------------------------------------------------------------
EPI_ISL_424247       ------------------------------------------------------------
EPI_ISL_424249       ------------------------------------------------------------
EPI_ISL_424250       ------------------------------------------------------------
EPI_ISL_424252       ------------------------------------------------------------
EPI_ISL_424258       ------------------------------------------------------------
EPI_ISL_424268       ------------------------------------------------------------
EPI_ISL_424269       ------------------------------------------------------------
EPI_ISL_424275       ------------------------------------------------------------
EPI_ISL_424276       ------------------------------------------------------------
```

EPI_ISL_424279   --

```
ORF1ab          4501 DMVPHISRQRLTKYTMADLVYALRHFDEGNCDTLKEILVTYNCCDDDYFNKKDWYDFVEN
EPI_ISL_424219       ------------------------------------------------------------
EPI_ISL_424222       ------------------------------------------------------------
EPI_ISL_424223       ------------------------------------------------------------
EPI_ISL_424226       ------------------------------------------------------------
EPI_ISL_424228       ------------------------------------------------------------
EPI_ISL_424230       ------------------------------------------------------------
EPI_ISL_424231       ------------------------------------------------------------
EPI_ISL_424240       ------------------------------------------------------------
EPI_ISL_424241       ------------------------------------------------------------
EPI_ISL_424243       ------------------------------------------------------------
EPI_ISL_424247       ------------------------------------------------------------
EPI_ISL_424249       ------------------------------------------------------------
EPI_ISL_424250       ------------------------------------------------------------
EPI_ISL_424252       ------------------------------------------------------------
EPI_ISL_424258       ------------------------------------------------------------
EPI_ISL_424268       ------------------------------------------------------------
EPI_ISL_424269       ------------------------------------------------------------
EPI_ISL_424275       ------------------------------------------------------------
EPI_ISL_424276       ------------------------------------------------------------
EPI_ISL_424279       ------------------------------------------------------------

ORF1ab          4561 PDILRVYANLGERVRQALLKTVQFCDAMRNAGIVGVLTLDNQDLNGNWYDFGDFIQTTPG
EPI_ISL_424219       ------------------------------------------------------------
EPI_ISL_424222       ------------------------------------------------------------
EPI_ISL_424223       ------------------------------------------------------------
EPI_ISL_424226       ------------------------------------------------------------
EPI_ISL_424228       ------------------------------------------------------------
EPI_ISL_424230       ------------------------------------------------------------
EPI_ISL_424231       ------------------------------------------------------------
EPI_ISL_424240       ------------------------------------------------------------
EPI_ISL_424241       ------------------------------------------------------------
EPI_ISL_424243       ------------------------------------------------------------
EPI_ISL_424247       ------------------------------------------------------------
EPI_ISL_424249       ------------------------------------------------------------
EPI_ISL_424250       ------------------------------------------------------------
EPI_ISL_424252       ------------------------------------------------------------
EPI_ISL_424258       ------------------------------------------------------------
EPI_ISL_424268       ------------------------------------------------------------
EPI_ISL_424269       ------------------------------------------------------------
EPI_ISL_424275       ------------------------------------------------------------
EPI_ISL_424276       ------------------------------------------------------------
EPI_ISL_424279       ------------------------------------------------------------

ORF1ab          4621 SGVPVVDSYYSLLMPILTLTRALTAESHVDTDLTKPYIKWDLLKYDFTEERLKLFDRYFK
EPI_ISL_424219       ------------------------------------------------------------
EPI_ISL_424222       ------------------------------------------------------------
EPI_ISL_424223       ------------------------------------------------------------
EPI_ISL_424226       ------------------------------------------------------------
EPI_ISL_424228       ------------------------------------------------------------
EPI_ISL_424230       ------------------------------------------------------------
EPI_ISL_424231       ------------------------------------------------------------
EPI_ISL_424240       ------------------------------------------------------------
EPI_ISL_424241       ------------------------------------------------------------
EPI_ISL_424243       ------------------------------------------------------------
EPI_ISL_424247       ------------------------------------------------------------
EPI_ISL_424249       ------------------------------------------------------------
EPI_ISL_424250       ------------------------------------------------------------
EPI_ISL_424252       ------------------------------------------------------------
EPI_ISL_424258       ------------------------------------------------------------
EPI_ISL_424268       ------------------------------------------------------------
EPI_ISL_424269       ------------------------------------------------------------
EPI_ISL_424275       ------------------------------------------------------------
EPI_ISL_424276       ------------------------------------------------------------
```

EPI_ISL_424279   --

```
ORF1ab         4681 YWDQTYHPNCVNCLDDRCILHCANFNVLFSTVFPPTSFGPLVRKIFVDGVPFVVSTGYHF
EPI_ISL_424219      ------------------------------------------------------------
EPI_ISL_424222      ------------------------------------------------------------
EPI_ISL_424223      ------------------------------------------------------------
EPI_ISL_424226      ------------------------------------------------------------
EPI_ISL_424228      ------------------------------------------------------------
EPI_ISL_424230      ------------------------------------------------------------
EPI_ISL_424231      ------------------------------------------------------------
EPI_ISL_424240      ------------------------------------------------------------
EPI_ISL_424241      ------------------------------------------------------------
EPI_ISL_424243      ------------------------------------------------------------
EPI_ISL_424247      ------------------------------------------------------------
EPI_ISL_424249      ------------------------------------------------------------
EPI_ISL_424250      ------------------------------------------------------------
EPI_ISL_424252      ------------------------------------------------------------
EPI_ISL_424258      ------------------------------------------------------------
EPI_ISL_424268      ------------------------------------------------------------
EPI_ISL_424269      ------------------------------------------------------------
EPI_ISL_424275      ------------------------------------------------------------
EPI_ISL_424276      ------------------------------------------------------------
EPI_ISL_424279      ------------------------------------------------------------

ORF1ab         4741 RELGVVHNQDVNLHSSRLSFKELLVYAADPAMHAASGNLLLDKRTTCFSVAALTNNVAFQ
EPI_ISL_424219      ------------------------------------------------------------
EPI_ISL_424222      ------------------------------------------------------------
EPI_ISL_424223      ------------------------------------------------------------
EPI_ISL_424226      ------------------------------------------------------------
EPI_ISL_424228      ------------------------------------------------------------
EPI_ISL_424230      ------------------------------------------------------------
EPI_ISL_424231      ------------------------------------------------------------
EPI_ISL_424240      ------------------------------------------------------------
EPI_ISL_424241      ------------------------------------------------------------
EPI_ISL_424243      ------------------------------------------------------------
EPI_ISL_424247      ------------------------------------------------------------
EPI_ISL_424249      ------------------------------------------------------------
EPI_ISL_424250      ------------------------------------------------------------
EPI_ISL_424252      ------------------------------------------------------------
EPI_ISL_424258      ------------------------------------------------------------
EPI_ISL_424268      ------------------------------------------------------------
EPI_ISL_424269      ------------------------------------------------------------
EPI_ISL_424275      ------------------------------------------------------------
EPI_ISL_424276      ------------------------------------------------------------
EPI_ISL_424279      ------------------------------------------------------------

ORF1ab         4801 TVKPGNFNKDFYDFAVSKGFFKEGSSVELKHFFFAQDGNAAISDYDYYRYNLPTMCDIRQ
EPI_ISL_424219      ------------------------------------------------------------
EPI_ISL_424222      ------------------------------------------------------------
EPI_ISL_424223      ------------------------------------------------------------
EPI_ISL_424226      ------------------------------------------------------------
EPI_ISL_424228      ------------------------------------------------------------
EPI_ISL_424230      ------------------------------------------------------------
EPI_ISL_424231      ------------------------------------------------------------
EPI_ISL_424240      ------------------------------------------------------------
EPI_ISL_424241      ------------------------------------------------------------
EPI_ISL_424243      ------------------------------------------------------------
EPI_ISL_424247      ------------------------------------------------------------
EPI_ISL_424249      ------------------------------------------------------------
EPI_ISL_424250      ------------------------------------------------------------
EPI_ISL_424252      ------------------------------------------------------------
EPI_ISL_424258      ------------------------------------------------------------
EPI_ISL_424268      ------------------------------------------------------------
EPI_ISL_424269      ------------------------------------------------------------
EPI_ISL_424275      ------------------------------------------------------------
EPI_ISL_424276      ------------------------------------------------------------
```

EPI_ISL_424279 --

```
ORF1ab          4861 LLFVVEVVDKYFDCYDGGCINANQVIVNNLDKSAGFPFNKWGKARLYYDSMSYEDQDALF
EPI_ISL_424219       ------------------------------------------------------------
EPI_ISL_424222       ------------------------------------------------------------
EPI_ISL_424223       ------------------------------------------------------------
EPI_ISL_424226       ------------------------------------------------------------
EPI_ISL_424228       ------------------------------------------------------------
EPI_ISL_424230       ------------------------------------------------------------
EPI_ISL_424231       ------------------------------------------------------------
EPI_ISL_424240       ------------------------------------------------------------
EPI_ISL_424241       ------------------------------------------------------------
EPI_ISL_424243       ------------------------------------------------------------
EPI_ISL_424247       ------------------------------------------------------------
EPI_ISL_424249       ------------------------------------------------------------
EPI_ISL_424250       ------------------------------------------------------------
EPI_ISL_424252       ------------------------------------------------------------
EPI_ISL_424258       ------------------------------------------------------------
EPI_ISL_424268       ------------------------------------------------------------
EPI_ISL_424269       ------------------------------------------------------------
EPI_ISL_424275       ------------------------------------------------------------
EPI_ISL_424276       ------------------------------------------------------------
EPI_ISL_424279       ------------------------------------------------------------

ORF1ab          4921 AYTKRNVIPTITQMNLKYAISAKNRARTVAGVSICSTMTNRQFHQKLLKSIAATRGATVV
EPI_ISL_424219       ------------------------------------------------------------
EPI_ISL_424222       ------------------------------------------------------------
EPI_ISL_424223       ------------------------------------------------------------
EPI_ISL_424226       ------------------------------------------------------------
EPI_ISL_424228       ------------------------------------------------------------
EPI_ISL_424230       ------------------------------------------------------------
EPI_ISL_424231       ------------------------------------------------------------
EPI_ISL_424240       ------------------------------------------------------------
EPI_ISL_424241       ------------------------------------------------------------
EPI_ISL_424243       ------------------------------------------------------------
EPI_ISL_424247       ------------------------------------------------------------
EPI_ISL_424249       ------------------------------------------------------------
EPI_ISL_424250       ------------------------------------------------------------
EPI_ISL_424252       ------------------------------------------------------------
EPI_ISL_424258       ------------------------------------------------------------
EPI_ISL_424268       ------------------------------------------------------------
EPI_ISL_424269       ------------------------------------------------------------
EPI_ISL_424275       ------------------------------------------------------------
EPI_ISL_424276       ------------------------------------------------------------
EPI_ISL_424279       ------------------------------------------------------------

ORF1ab          4981 IGTSKFYGGWHNMLKTVYSDVENPHLMGWDYPKCDRAMPNMLRIMASLVLARKHTTCCSL
EPI_ISL_424219       ------------------------------------------------------------
EPI_ISL_424222       ------------------------------------------------------------
EPI_ISL_424223       ------------------------------------------------------------
EPI_ISL_424226       ------------------------------------------------------------
EPI_ISL_424228       ------------------------------------------------------------
EPI_ISL_424230       ------------------------------------------------------------
EPI_ISL_424231       ------------------------------------------------------------
EPI_ISL_424240       ------------------------------------------------------------
EPI_ISL_424241       ------------------------------------------------------------
EPI_ISL_424243       ------------------------------------------------------------
EPI_ISL_424247       ------------------------------------------------------------
EPI_ISL_424249       ------------------------------------------------------------
EPI_ISL_424250       ------------------------------------------------------------
EPI_ISL_424252       ------------------------------------------------------------
EPI_ISL_424258       ------------------------------------------------------------
EPI_ISL_424268       ------------------------------------------------------------
EPI_ISL_424269       ------------------------------------------------------------
EPI_ISL_424275       ------------------------------------------------------------
EPI_ISL_424276       ------------------------------------------------------------
```

EPI_ISL_424279 --

```
ORF1ab         5041 SHRFYRLANECAQVLSEMVMCGGSLYVKPGGTSSGDATTAYANSVFNICQAVTANVNALL
EPI_ISL_424219      ------------------------------------------------------------
EPI_ISL_424222      ------------------------------------------------------------
EPI_ISL_424223      ------------------------------------------------------------
EPI_ISL_424226      ------------------------------------------------------------
EPI_ISL_424228      ------------------------------------------------------------
EPI_ISL_424230      ------------------------------------------------------------
EPI_ISL_424231      ------------------------------------------------------------
EPI_ISL_424240      ------------------------------------------------------------
EPI_ISL_424241      ------------------------------------------------------------
EPI_ISL_424243      ------------------------------------------------------------
EPI_ISL_424247      ------------------------------------------------------------
EPI_ISL_424249      ------------------------------------------------------------
EPI_ISL_424250      ------------------------------------------------------------
EPI_ISL_424252      ------------------------------------------------------------
EPI_ISL_424258      ------------------------------------------------------------
EPI_ISL_424268      ------------------------------------------------------------
EPI_ISL_424269      ------------------------------------------------------------
EPI_ISL_424275      ------------------------------------------------------------
EPI_ISL_424276      ------------------------------------------------------------
EPI_ISL_424279      ------------------------------------------------------------

ORF1ab         5101 STDGNKIADKYVRNLQHRLYECLYRNRDVDTDFVNEFYAYLRKHFSMMILSDDAVVCFNS
EPI_ISL_424219      ------------------------------------------------------------
EPI_ISL_424222      ------------------------------------------------------------
EPI_ISL_424223      ------------------------------------------------------------
EPI_ISL_424226      ------------------------------------------------------------
EPI_ISL_424228      ------------------------------------------------------------
EPI_ISL_424230      ------------------------------------------------------------
EPI_ISL_424231      ------------------------------------------------------------
EPI_ISL_424240      ------------------------------------------------------------
EPI_ISL_424241      ------------------------------------------------------------
EPI_ISL_424243      ------------------------------------------------------------
EPI_ISL_424247      ------------------------------------------------------------
EPI_ISL_424249      ------------------------------------------------------------
EPI_ISL_424250      ------------------------------------------------------------
EPI_ISL_424252      ------------------------------------------------------------
EPI_ISL_424258      ------------------------------------------------------------
EPI_ISL_424268      ------------------------------------------------------------
EPI_ISL_424269      ------------------------------------------------------------
EPI_ISL_424275      ------------------------------------------------------------
EPI_ISL_424276      ------------------------------------------------------------
EPI_ISL_424279      ------------------------------------------------------------

ORF1ab         5161 TYASQGLVASIKNFKSVLYYQNNVFMSEAKCWTETDLTKGPHEFCSQHTMLVKQGDDYVY
EPI_ISL_424219      ------------------------------------------------------------
EPI_ISL_424222      ------------------------------------------------------------
EPI_ISL_424223      ------------------------------------------------------------
EPI_ISL_424226      ------------------------------------------------------------
EPI_ISL_424228      ------------------------------------------------------------
EPI_ISL_424230      ------------------------------------------------------------
EPI_ISL_424231      ------------------------------------------------------------
EPI_ISL_424240      ------------------------------------------------------------
EPI_ISL_424241      ------------------------------------------------------------
EPI_ISL_424243      ------------------------------------------------------------
EPI_ISL_424247      ------------------------------------------------------------
EPI_ISL_424249      ------------------------------------------------------------
EPI_ISL_424250      ------------------------------------------------------------
EPI_ISL_424252      ------------------------------------------------------------
EPI_ISL_424258      ------------------------------------------------------------
EPI_ISL_424268      ------------------------------------------------------------
EPI_ISL_424269      ------------------------------------------------------------
EPI_ISL_424275      ------------------------------------------------------------
EPI_ISL_424276      ------------------------------------------------------------
```

EPI_ISL_424279 --

```
ORF1ab         5221 LPYPDPSRILGAGCFVDDIVKTDGTLMIERFVSLAIDAYPLTKHPNQEYADVFHLYLQYI
EPI_ISL_424219      ------------------------------------------------------------
EPI_ISL_424222      ------------------------------------------------------------
EPI_ISL_424223      ------------------------------------------------------------
EPI_ISL_424226      ------------------------------------------------------------
EPI_ISL_424228      ------------------------------------------------------------
EPI_ISL_424230      ------------------------------------------------------------
EPI_ISL_424231      ------------------------------------------------------------
EPI_ISL_424240      ------------------------------------------------------------
EPI_ISL_424241      ------------------------------------------------------------
EPI_ISL_424243      ------------------------------------------------------------
EPI_ISL_424247      ------------------------------------------------------------
EPI_ISL_424249      ------------------------------------------------------------
EPI_ISL_424250      ------------------------------------------------------------
EPI_ISL_424252      ------------------------------------------------------------
EPI_ISL_424258      ------------------------------------------------------------
EPI_ISL_424268      ------------------------------------------------------------
EPI_ISL_424269      ------------------------------------------------------------
EPI_ISL_424275      ------------------------------------------------------------
EPI_ISL_424276      ------------------------------------------------------------
EPI_ISL_424279      ------------------------------------------------------------

ORF1ab         5281 RKLHDELTGHMLDMYSVMLTNDNTSRYWEPEFYEAMYTPHTVLQAVGACVLCNSQTSLRC
EPI_ISL_424219      ------------------------------------------------------------
EPI_ISL_424222      ------------------------------------------------------------
EPI_ISL_424223      ------------------------------------------------------------
EPI_ISL_424226      ------------------------------------------------------------
EPI_ISL_424228      ------------------------------------------------------------
EPI_ISL_424230      ------------------------------------------------------------
EPI_ISL_424231      ------------------------------------------------------------
EPI_ISL_424240      ------------------------------------------------------------
EPI_ISL_424241      ------------------------------------------------------------
EPI_ISL_424243      ------------------------------------------------------------
EPI_ISL_424247      ------------------------------------------------------------
EPI_ISL_424249      ------------------------------------------------------------
EPI_ISL_424250      ------------------------------------------------------------
EPI_ISL_424252      ------------------------------------------------------------
EPI_ISL_424258      ------------------------------------------------------------
EPI_ISL_424268      ------------------------------------------------------------
EPI_ISL_424269      ------------------------------------------------------------
EPI_ISL_424275      ------------------------------------------------------------
EPI_ISL_424276      ------------------------------------------------------------
EPI_ISL_424279      ------------------------------------------------------------

ORF1ab         5341 GACIRRPFLCCKCCYDHVISTSHKLVLSVNPYVCNAPGCDVTDVTQLYLGGMSYYCKSHK
EPI_ISL_424219      ------------------------------------------------------------
EPI_ISL_424222      ------------------------------------------------------------
EPI_ISL_424223      ------------------------------------------------------------
EPI_ISL_424226      ------------------------------------------------------------
EPI_ISL_424228      ------------------------------------------------------------
EPI_ISL_424230      ------------------------------------------------------------
EPI_ISL_424231      ------------------------------------------------------------
EPI_ISL_424240      ------------------------------------------------------------
EPI_ISL_424241      ------------------------------------------------------------
EPI_ISL_424243      ------------------------------------------------------------
EPI_ISL_424247      ------------------------------------------------------------
EPI_ISL_424249      ------------------------------------------------------------
EPI_ISL_424250      ------------------------------------------------------------
EPI_ISL_424252      ------------------------------------------------------------
EPI_ISL_424258      ------------------------------------------------------------
EPI_ISL_424268      ------------------------------------------------------------
EPI_ISL_424269      ------------------------------------------------------------
EPI_ISL_424275      ------------------------------------------------------------
EPI_ISL_424276      ------------------------------------------------------------
```

EPI_ISL_424279   --

```
ORF1ab          5401 PPISFPLCANGQVFGLYKNTCVGSDNVTDFNAIATCDWTNAGDYILANTCTERLKLFAAE
EPI_ISL_424219       ------------------------------------------------------------
EPI_ISL_424222       ------------------------------------------------------------
EPI_ISL_424223       ------------------------------------------------------------
EPI_ISL_424226       ------------------------------------------------------------
EPI_ISL_424228       ------------------------------------------------------------
EPI_ISL_424230       ------------------------------------------------------------
EPI_ISL_424231       ------------------------------------------------------------
EPI_ISL_424240       ------------------------------------------------------------
EPI_ISL_424241       ------------------------------------------------------------
EPI_ISL_424243       ------------------------------------------------------------
EPI_ISL_424247       ------------------------------------------------------------
EPI_ISL_424249       ------------------------------------------------------------
EPI_ISL_424250       ------------------------------------------------------------
EPI_ISL_424252       ------------------------------------------------------------
EPI_ISL_424258       ------------------------------------------------------------
EPI_ISL_424268       ------------------------------------------------------------
EPI_ISL_424269       ------------------------------------------------------------
EPI_ISL_424275       ------------------------------------------------------------
EPI_ISL_424276       ------------------------------------------------------------
EPI_ISL_424279       ------------------------------------------------------------

ORF1ab          5461 TLKATEETFKLSYGIATVREVLSDRELHLSWEVGKPRPPLNRNYVFTGYRVTKNSKVQIG
EPI_ISL_424219       ------------------------------------------------------------
EPI_ISL_424222       ------------------------------------------------------------
EPI_ISL_424223       ------------------------------------------------------------
EPI_ISL_424226       ------------------------------------------------------------
EPI_ISL_424228       ------------------------------------------------------------
EPI_ISL_424230       ------------------------------------------------------------
EPI_ISL_424231       ------------------------------------------------------------
EPI_ISL_424240       ------------------------------------------------------------
EPI_ISL_424241       ------------------------------------------------------------
EPI_ISL_424243       ------------------------------------------------------------
EPI_ISL_424247       ------------------------------------------------------------
EPI_ISL_424249       ------------------------------------------------------------
EPI_ISL_424250       ------------------------------------------------------------
EPI_ISL_424252       ------------------------------------------------------------
EPI_ISL_424258       ------------------------------------------------------------
EPI_ISL_424268       ------------------------------------------------------------
EPI_ISL_424269       ------------------------------------------------------------
EPI_ISL_424275       ------------------------------------------------------------
EPI_ISL_424276       ------------------------------------------------------------
EPI_ISL_424279       ------------------------------------------------------------

ORF1ab          5521 EYTFEKGDYGDAVVYRGTTTYKLNVGDYFVLTSHTVMPLSAPTLVPQEHYVRITGLYPTL
EPI_ISL_424219       ------------------------------------------------------------
EPI_ISL_424222       ------------------------------------------------------------
EPI_ISL_424223       ------------------------------------------------------------
EPI_ISL_424226       ------------------------------------------------------------
EPI_ISL_424228       ------------------------------------------------------------
EPI_ISL_424230       ------------------------------------------------------------
EPI_ISL_424231       ------------------------------------------------------------
EPI_ISL_424240       ------------------------------------------------------------
EPI_ISL_424241       ------------------------------------------------------------
EPI_ISL_424243       ------------------------------------------------------------
EPI_ISL_424247       ------------------------------------------------------------
EPI_ISL_424249       ------------------------------------------------------------
EPI_ISL_424250       ------------------------------------------------------------
EPI_ISL_424252       ------------------------------------------------------------
EPI_ISL_424258       ------------------------------------------------------------
EPI_ISL_424268       ------------------------------------------------------------
EPI_ISL_424269       ------------------------------------------------------------
EPI_ISL_424275       ------------------------------------------------------------
EPI_ISL_424276       ------------------------------------------------------------
```

EPI_ISL_424279   --

```
ORF1ab          5581 NISDEFSSNVANYQKVGMQKYSTLQGPPGTGKSHFAIGLALYYPSARIVYTACSHAAVDA
EPI_ISL_424219       ------------------------------------------------------------
EPI_ISL_424222       ------------------------------------------------------------
EPI_ISL_424223       ------------------------------------------------------------
EPI_ISL_424226       ------------------------------------------------------------
EPI_ISL_424228       ------------------------------------------------------------
EPI_ISL_424230       ------------------------------------------------------------
EPI_ISL_424231       ------------------------------------------------------------
EPI_ISL_424240       ------------------------------------------------------------
EPI_ISL_424241       ------------------------------------------------------------
EPI_ISL_424243       ------------------------------------------------------------
EPI_ISL_424247       ------------------------------------------------------------
EPI_ISL_424249       ------------------------------------------------------------
EPI_ISL_424250       ------------------------------------------------------------
EPI_ISL_424252       ------------------------------------------------------------
EPI_ISL_424258       ------------------------------------------------------------
EPI_ISL_424268       ------------------------------------------------------------
EPI_ISL_424269       ------------------------------------------------------------
EPI_ISL_424275       ------------------------------------------------------------
EPI_ISL_424276       ------------------------------------------------------------
EPI_ISL_424279       ------------------------------------------------------------

ORF1ab          5641 LCEKALKYLPIDKCSRIIPARARVECFDKFKVNSTLEQYVFCTVNALPETTADIVVFDEI
EPI_ISL_424219       ------------------------------------------------------------
EPI_ISL_424222       ------------------------------------------------------------
EPI_ISL_424223       ------------------------------------------------------------
EPI_ISL_424226       ------------------------------------------------------------
EPI_ISL_424228       ------------------------------------------------------------
EPI_ISL_424230       ------------------------------------------------------------
EPI_ISL_424231       ------------------------------------------------------------
EPI_ISL_424240       ------------------------------------------------------------
EPI_ISL_424241       ------------------------------------------------------------
EPI_ISL_424243       ------------------------------------------------------------
EPI_ISL_424247       ------------------------------------------------------------
EPI_ISL_424249       ------------------------------------------------------------
EPI_ISL_424250       ------------------------------------------------------------
EPI_ISL_424252       ------------------------------------------------------------
EPI_ISL_424258       ------------------------------------------------------------
EPI_ISL_424268       ------------------------------------------------------------
EPI_ISL_424269       ------------------------------------------------------------
EPI_ISL_424275       ------------------------------------------------------------
EPI_ISL_424276       ------------------------------------------------------------
EPI_ISL_424279       ------------------------------------------------------------

ORF1ab          5701 SMATNYDLSVVNARLRAKHYVYIGDPAQLPAPRTLLTKGTLEPEYFNSVCRLMKTIGPDM
EPI_ISL_424219       ------------------------------------------------------------
EPI_ISL_424222       ------------------------------------------------------------
EPI_ISL_424223       ------------------------------------------------------------
EPI_ISL_424226       ------------------------------------------------------------
EPI_ISL_424228       ------------------------------------------------------------
EPI_ISL_424230       ------------------------------------------------------------
EPI_ISL_424231       ------------------------------------------------------------
EPI_ISL_424240       ------------------------------------------------------------
EPI_ISL_424241       ------------------------------------------------------------
EPI_ISL_424243       ------------------------------------------------------------
EPI_ISL_424247       ------------------------------------------------------------
EPI_ISL_424249       ------------------------------------------------------------
EPI_ISL_424250       ------------------------------------------------------------
EPI_ISL_424252       ------------------------------------------------------------
EPI_ISL_424258       ------------------------------------------------------------
EPI_ISL_424268       ------------------------------------------------------------
EPI_ISL_424269       ------------------------------------------------------------
EPI_ISL_424275       ------------------------------------------------------------
EPI_ISL_424276       ------------------------------------------------------------
```

EPI_ISL_424279 --

```
ORF1ab          5761 FLGTCRRCPAEIVDTVSALVYDNKLKAHKDKSAQCFKMFYKGVITHDVSSAINRPQIGVV
EPI_ISL_424219       ------------------------------------------------------------
EPI_ISL_424222       ------------------------------------------------------------
EPI_ISL_424223       ------------------------------------------------------------
EPI_ISL_424226       ------------------------------------------------------------
EPI_ISL_424228       ------------------------------------------------------------
EPI_ISL_424230       ------------------------------------------------------------
EPI_ISL_424231       ------------------------------------------------------------
EPI_ISL_424240       ------------------------------------------------------------
EPI_ISL_424241       ------------------------------------------------------------
EPI_ISL_424243       ------------------------------------------------------------
EPI_ISL_424247       ------------------------------------------------------------
EPI_ISL_424249       ------------------------------------------------------------
EPI_ISL_424250       ------------------------------------------------------------
EPI_ISL_424252       ------------------------------------------------------------
EPI_ISL_424258       ------------------------------------------------------------
EPI_ISL_424268       ------------------------------------------------------------
EPI_ISL_424269       ------------------------------------------------------------
EPI_ISL_424275       ------------------------------------------------------------
EPI_ISL_424276       ------------------------------------------------------------
EPI_ISL_424279       ------------------------------------------------------------

ORF1ab          5821 REFLTRNPAWRKAVFISPYNSQNAVASKILGLPTQTVDSSQGSEYDYVIFTQTTETAHSC
EPI_ISL_424219       ------------------------------------------------------------
EPI_ISL_424222       ------------------------------------------------------------
EPI_ISL_424223       ------------------------------------------------------------
EPI_ISL_424226       ------------------------------------------------------------
EPI_ISL_424228       ------------------------------------------------------------
EPI_ISL_424230       ------------------------------------------------------------
EPI_ISL_424231       ------------------------------------------------------------
EPI_ISL_424240       ------------------------------------------------------------
EPI_ISL_424241       ------------------------------------------------------------
EPI_ISL_424243       ------------------------------------------------------------
EPI_ISL_424247       ------------------------------------------------------------
EPI_ISL_424249       ------------------------------------------------------------
EPI_ISL_424250       ------------------------------------------------------------
EPI_ISL_424252       ------------------------------------------------------------
EPI_ISL_424258       ------------------------------------------------------------
EPI_ISL_424268       ------------------------------------------------------------
EPI_ISL_424269       ------------------------------------------------------------
EPI_ISL_424275       ------------------------------------------------------------
EPI_ISL_424276       ------------------------------------------------------------
EPI_ISL_424279       ------------------------------------------------------------

ORF1ab          5881 NVNRFNVAITRAKVGILCIMSDRDLYDKLQFTSLEIPRRNVATLQAENVTGLFKDCSKVI
EPI_ISL_424219       ------------------------------------------------------------
EPI_ISL_424222       ------------------------------------------------------------
EPI_ISL_424223       ------------------------------------------------------------
EPI_ISL_424226       ------------------------------------------------------------
EPI_ISL_424228       ------------------------------------------------------------
EPI_ISL_424230       ------------------------------------------------------------
EPI_ISL_424231       ------------------------------------------------------------
EPI_ISL_424240       ------------------------------------------------------------
EPI_ISL_424241       ------------------------------------------------------------
EPI_ISL_424243       ------------------------------------------------------------
EPI_ISL_424247       ------------------------------------------------------------
EPI_ISL_424249       ------------------------------------------------------------
EPI_ISL_424250       ------------------------------------------------------------
EPI_ISL_424252       ------------------------------------------------------------
EPI_ISL_424258       ------------------------------------------------------------
EPI_ISL_424268       ------------------------------------------------------------
EPI_ISL_424269       ------------------------------------------------------------
EPI_ISL_424275       ------------------------------------------------------------
EPI_ISL_424276       ------------------------------------------------------------
```

EPI_ISL_424279 --

```
ORF1ab         5941 TGLHPTQAPTHLSVDTKFKTEGLCVDIPGIPKDMTYRRLISMMGFKMNYQVNGYPNMFIT
EPI_ISL_424219      ------------------------------------------------------------
EPI_ISL_424222      ------------------------------------------------------------
EPI_ISL_424223      ------------------------------------------------------------
EPI_ISL_424226      ------------------------------------------------------------
EPI_ISL_424228      ------------------------------------------------------------
EPI_ISL_424230      ------------------------------------------------------------
EPI_ISL_424231      ------------------------------------------------------------
EPI_ISL_424240      ------------------------------------------------------------
EPI_ISL_424241      ------------------------------------------------------------
EPI_ISL_424243      ------------------------------------------------------------
EPI_ISL_424247      ------------------------------------------------------------
EPI_ISL_424249      ------------------------------------------------------------
EPI_ISL_424250      ------------------------------------------------------------
EPI_ISL_424252      ------------------------------------------------------------
EPI_ISL_424258      ------------------------------------------------------------
EPI_ISL_424268      ------------------------------------------------------------
EPI_ISL_424269      ------------------------------------------------------------
EPI_ISL_424275      ------------------------------------------------------------
EPI_ISL_424276      ------------------------------------------------------------
EPI_ISL_424279      ------------------------------------------------------------

ORF1ab         6001 REEAIRHVRAWIGFDVEGCHATREAVGTNLPLQLGFSTGVNLVAVPTGYVDTPNNTDFSR
EPI_ISL_424219      ------------------------------------------------------------
EPI_ISL_424222      ------------------------------------------------------------
EPI_ISL_424223      ------------------------------------------------------------
EPI_ISL_424226      ------------------------------------------------------------
EPI_ISL_424228      ------------------------------------------------------------
EPI_ISL_424230      ------------------------------------------------------------
EPI_ISL_424231      ------------------------------------------------------------
EPI_ISL_424240      ------------------------------------------------------------
EPI_ISL_424241      ------------------------------------------------------------
EPI_ISL_424243      ------------------------------------------------------------
EPI_ISL_424247      ------------------------------------------------------------
EPI_ISL_424249      ------------------------------------------------------------
EPI_ISL_424250      ------------------------------------------------------------
EPI_ISL_424252      ------------------------------------------------------------
EPI_ISL_424258      ------------------------------------------------------------
EPI_ISL_424268      ------------------------------------------------------------
EPI_ISL_424269      ------------------------------------------------------------
EPI_ISL_424275      ------------------------------------------------------------
EPI_ISL_424276      ------------------------------------------------------------
EPI_ISL_424279      ------------------------------------------------------------

ORF1ab         6061 VSAKPPPGDQFKHLIPLMYKGLPWNVVRIKIVQMLSDTLKNLSDRVVFVLWAHGFELTSM
EPI_ISL_424219      ------------------------------------------------------------
EPI_ISL_424222      ------------------------------------------------------------
EPI_ISL_424223      ------------------------------------------------------------
EPI_ISL_424226      ------------------------------------------------------------
EPI_ISL_424228      ------------------------------------------------------------
EPI_ISL_424230      ------------------------------------------------------------
EPI_ISL_424231      ------------------------------------------------------------
EPI_ISL_424240      ------------------------------------------------------------
EPI_ISL_424241      ------------------------------------------------------------
EPI_ISL_424243      ------------------------------------------------------------
EPI_ISL_424247      ------------------------------------------------------------
EPI_ISL_424249      ------------------------------------------------------------
EPI_ISL_424250      ------------------------------------------------------------
EPI_ISL_424252      ------------------------------------------------------------
EPI_ISL_424258      ------------------------------------------------------------
EPI_ISL_424268      ------------------------------------------------------------
EPI_ISL_424269      ------------------------------------------------------------
EPI_ISL_424275      ------------------------------------------------------------
EPI_ISL_424276      ------------------------------------------------------------
```

EPI_ISL_424279 --

```
ORF1ab          6121 KYFVKIGPERTCCLCDRRATCFSTASDTYACWHHSIGFDYVYNPFMIDVQQWGFTGNLQS
EPI_ISL_424219       ------------------------------------------------------------
EPI_ISL_424222       ------------------------------------------------------------
EPI_ISL_424223       ------------------------------------------------------------
EPI_ISL_424226       ------------------------------------------------------------
EPI_ISL_424228       ------------------------------------------------------------
EPI_ISL_424230       ------------------------------------------------------------
EPI_ISL_424231       ------------------------------------------------------------
EPI_ISL_424240       ------------------------------------------------------------
EPI_ISL_424241       ------------------------------------------------------------
EPI_ISL_424243       ------------------------------------------------------------
EPI_ISL_424247       ------------------------------------------------------------
EPI_ISL_424249       ------------------------------------------------------------
EPI_ISL_424250       ------------------------------------------------------------
EPI_ISL_424252       ------------------------------------------------------------
EPI_ISL_424258       ------------------------------------------------------------
EPI_ISL_424268       ------------------------------------------------------------
EPI_ISL_424269       ------------------------------------------------------------
EPI_ISL_424275       ------------------------------------------------------------
EPI_ISL_424276       ------------------------------------------------------------
EPI_ISL_424279       ------------------------------------------------------------

ORF1ab          6181 NHDLYCQVHGNAHVASCDAIMTRCLAVHECFVKRVDWTIEYPIIGDELKINAACRKVQHM
EPI_ISL_424219       ------------------------------------------------------------
EPI_ISL_424222       ------------------------------------------------------------
EPI_ISL_424223       ------------------------------------------------------------
EPI_ISL_424226       ------------------------------------------------------------
EPI_ISL_424228       ------------------------------------------------------------
EPI_ISL_424230       ------------------------------------------------------------
EPI_ISL_424231       ------------------------------------------------------------
EPI_ISL_424240       ------------------------------------------------------------
EPI_ISL_424241       ------------------------------------------------------------
EPI_ISL_424243       ------------------------------------------------------------
EPI_ISL_424247       ------------------------------------------------------------
EPI_ISL_424249       ------------------------------------------------------------
EPI_ISL_424250       ------------------------------------------------------------
EPI_ISL_424252       ------------------------------------------------------------
EPI_ISL_424258       ------------------------------------------------------------
EPI_ISL_424268       ------------------------------------------------------------
EPI_ISL_424269       ------------------------------------------------------------
EPI_ISL_424275       ------------------------------------------------------------
EPI_ISL_424276       ------------------------------------------------------------
EPI_ISL_424279       ------------------------------------------------------------

ORF1ab          6241 VVKAALLADKFPVLHDIGNPKAIKCVPQADVEWKFYDAQPCSDKAYKIEELFYSYATHSD
EPI_ISL_424219       ------------------------------------------------------------
EPI_ISL_424222       ------------------------------------------------------------
EPI_ISL_424223       ------------------------------------------------------------
EPI_ISL_424226       ------------------------------------------------------------
EPI_ISL_424228       ------------------------------------------------------------
EPI_ISL_424230       ------------------------------------------------------------
EPI_ISL_424231       ------------------------------------------------------------
EPI_ISL_424240       ------------------------------------------------------------
EPI_ISL_424241       ------------------------------------------------------------
EPI_ISL_424243       ------------------------------------------------------------
EPI_ISL_424247       ------------------------------------------------------------
EPI_ISL_424249       ------------------------------------------------------------
EPI_ISL_424250       ------------------------------------------------------------
EPI_ISL_424252       ------------------------------------------------------------
EPI_ISL_424258       ------------------------------------------------------------
EPI_ISL_424268       ------------------------------------------------------------
EPI_ISL_424269       ------------------------------------------------------------
EPI_ISL_424275       ------------------------------------------------------------
EPI_ISL_424276       ------------------------------------------------------------
```

EPI_ISL_424279 --

```
ORF1ab          6301 KFTDGVCLFWNCNVDRYPANSIVCRFDTRVLSNLNLPGCDGGSLYVNKHAFHTPAFDKSA
EPI_ISL_424219       ------------------------------------------------------------
EPI_ISL_424222       ------------------------------------------------------------
EPI_ISL_424223       ------------------------------------------------------------
EPI_ISL_424226       ------------------------------------------------------------
EPI_ISL_424228       ------------------------------------------------------------
EPI_ISL_424230       ------------------------------------------------------------
EPI_ISL_424231       ------------------------------------------------------------
EPI_ISL_424240       ------------------------------------------------------------
EPI_ISL_424241       ------------------------------------------------------------
EPI_ISL_424243       ------------------------------------------------------------
EPI_ISL_424247       ------------------------------------------------------------
EPI_ISL_424249       ------------------------------------------------------------
EPI_ISL_424250       ------------------------------------------------------------
EPI_ISL_424252       ------------------------------------------------------------
EPI_ISL_424258       ------------------------------------------------------------
EPI_ISL_424268       ------------------------------------------------------------
EPI_ISL_424269       ------------------------------------------------------------
EPI_ISL_424275       ------------------------------------------------------------
EPI_ISL_424276       ------------------------------------------------------------
EPI_ISL_424279       ------------------------------------------------------------

ORF1ab          6361 FVNLKQLPFFYYSDSPCESHGKQVVSDIDYVPLKSATCITRCNLGGAVCRHHANEYRLYL
EPI_ISL_424219       ------------------------------------------------------------
EPI_ISL_424222       ------------------------------------------------------------
EPI_ISL_424223       ------------------------------------------------------------
EPI_ISL_424226       ------------------------------------------------------------
EPI_ISL_424228       ------------------------------------------------------------
EPI_ISL_424230       ------------------------------------------------------------
EPI_ISL_424231       ------------------------------------------------------------
EPI_ISL_424240       ------------------------------------------------------------
EPI_ISL_424241       ------------------------------------------------------------
EPI_ISL_424243       ------------------------------------------------------------
EPI_ISL_424247       ------------------------------------------------------------
EPI_ISL_424249       ------------------------------------------------------------
EPI_ISL_424250       ------------------------------------------------------------
EPI_ISL_424252       ------------------------------------------------------------
EPI_ISL_424258       ------------------------------------------------------------
EPI_ISL_424268       ------------------------------------------------------------
EPI_ISL_424269       ------------------------------------------------------------
EPI_ISL_424275       ------------------------------------------------------------
EPI_ISL_424276       ------------------------------------------------------------
EPI_ISL_424279       ------------------------------------------------------------

ORF1ab          6421 DAYNMMISAGFSLWVYKQFDTYNLWNTFTRLQSLENVAFNVVNKGHFDGQQGEVPVSIIN
EPI_ISL_424219       ------------------------------------------------------------
EPI_ISL_424222       ------------------------------------------------------------
EPI_ISL_424223       ------------------------------------------------------------
EPI_ISL_424226       ------------------------------------------------------------
EPI_ISL_424228       ------------------------------------------------------------
EPI_ISL_424230       ------------------------------------------------------------
EPI_ISL_424231       ------------------------------------------------------------
EPI_ISL_424240       ------------------------------------------------------------
EPI_ISL_424241       ------------------------------------------------------------
EPI_ISL_424243       ------------------------------------------------------------
EPI_ISL_424247       ------------------------------------------------------------
EPI_ISL_424249       ------------------------------------------------------------
EPI_ISL_424250       ------------------------------------------------------------
EPI_ISL_424252       ------------------------------------------------------------
EPI_ISL_424258       ------------------------------------------------------------
EPI_ISL_424268       ------------------------------------------------------------
EPI_ISL_424269       ------------------------------------------------------------
EPI_ISL_424275       ------------------------------------------------------------
EPI_ISL_424276       ------------------------------------------------------------
```

EPI_ISL_424279   --

```
ORF1ab         6481 NTVYTKVDGVDVELFENKTTLPVNVAFELWAKRNIKPVPEVKILNNLGVDIAANTVIWDY
EPI_ISL_424219      ------------------------------------------------------------
EPI_ISL_424222      ------------------------------------------------------------
EPI_ISL_424223      ------------------------------------------------------------
EPI_ISL_424226      ------------------------------------------------------------
EPI_ISL_424228      ------------------------------------------------------------
EPI_ISL_424230      ------------------------------------------------------------
EPI_ISL_424231      ------------------------------------------------------------
EPI_ISL_424240      ------------------------------------------------------------
EPI_ISL_424241      ------------------------------------------------------------
EPI_ISL_424243      ------------------------------------------------------------
EPI_ISL_424247      ------------------------------------------------------------
EPI_ISL_424249      ------------------------------------------------------------
EPI_ISL_424250      ------------------------------------------------------------
EPI_ISL_424252      ------------------------------------------------------------
EPI_ISL_424258      ------------------------------------------------------------
EPI_ISL_424268      ------------------------------------------------------------
EPI_ISL_424269      ------------------------------------------------------------
EPI_ISL_424275      ------------------------------------------------------------
EPI_ISL_424276      ------------------------------------------------------------
EPI_ISL_424279      ------------------------------------------------------------

ORF1ab         6541 KRDAPAHISTIGVCSMTDIAKKPTETICAPLTVFFDGRVDGQVDLFRNARNGVLITEGSV
EPI_ISL_424219      ------------------------------------------------------------
EPI_ISL_424222      ------------------------------------------------------------
EPI_ISL_424223      ------------------------------------------------------------
EPI_ISL_424226      ------------------------------------------------------------
EPI_ISL_424228      ------------------------------------------------------------
EPI_ISL_424230      ------------------------------------------------------------
EPI_ISL_424231      ------------------------------------------------------------
EPI_ISL_424240      ------------------------------------------------------------
EPI_ISL_424241      ------------------------------------------------------------
EPI_ISL_424243      ------------------------------------------------------------
EPI_ISL_424247      ------------------------------------------------------------
EPI_ISL_424249      ------------------------------------------------------------
EPI_ISL_424250      ------------------------------------------------------------
EPI_ISL_424252      ------------------------------------------------------------
EPI_ISL_424258      ------------------------------------------------------------
EPI_ISL_424268      ------------------------------------------------------------
EPI_ISL_424269      ------------------------------------------------------------
EPI_ISL_424275      ------------------------------------------------------------
EPI_ISL_424276      ------------------------------------------------------------
EPI_ISL_424279      ------------------------------------------------------------

ORF1ab         6601 KGLQPSVGPKQASLNGVTLIGEAVKTQFNYYKKVDGVVQQLPETYFTQSRNLQEFKPRSQ
EPI_ISL_424219      ------------------------------------------------------------
EPI_ISL_424222      ------------------------------------------------------------
EPI_ISL_424223      ------------------------------------------------------------
EPI_ISL_424226      ------------------------------------------------------------
EPI_ISL_424228      ------------------------------------------------------------
EPI_ISL_424230      ------------------------------------------------------------
EPI_ISL_424231      ------------------------------------------------------------
EPI_ISL_424240      ------------------------------------------------------------
EPI_ISL_424241      ------------------------------------------------------------
EPI_ISL_424243      ------------------------------------------------------------
EPI_ISL_424247      ------------------------------------------------------------
EPI_ISL_424249      ------------------------------------------------------------
EPI_ISL_424250      ------------------------------------------------------------
EPI_ISL_424252      ------------------------------------------------------------
EPI_ISL_424258      ------------------------------------------------------------
EPI_ISL_424268      ------------------------------------------------------------
EPI_ISL_424269      ------------------------------------------------------------
EPI_ISL_424275      ------------------------------------------------------------
EPI_ISL_424276      ------------------------------------------------------------
```

EPI_ISL_424279 --

```
ORF1ab         6661 MEIDFLELAMDEFIERYKLEGYAFEHIVYGDFSHSQLGGLHLLIGLAKRFKESPFELEDF
EPI_ISL_424219      ------------------------------------------------------------
EPI_ISL_424222      ------------------------------------------------------------
EPI_ISL_424223      ------------------------------------------------------------
EPI_ISL_424226      ------------------------------------------------------------
EPI_ISL_424228      ------------------------------------------------------------
EPI_ISL_424230      ------------------------------------------------------------
EPI_ISL_424231      ------------------------------------------------------------
EPI_ISL_424240      ------------------------------------------------------------
EPI_ISL_424241      ------------------------------------------------------------
EPI_ISL_424243      ------------------------------------------------------------
EPI_ISL_424247      ------------------------------------------------------------
EPI_ISL_424249      ------------------------------------------------------------
EPI_ISL_424250      ------------------------------------------------------------
EPI_ISL_424252      ------------------------------------------------------------
EPI_ISL_424258      ------------------------------------------------------------
EPI_ISL_424268      ------------------------------------------------------------
EPI_ISL_424269      ------------------------------------------------------------
EPI_ISL_424275      ------------------------------------------------------------
EPI_ISL_424276      ------------------------------------------------------------
EPI_ISL_424279      ------------------------------------------------------------
```

```
ORF1ab         6721 IPMDSTVKNYFITDAQTGSSKCVCSVIDLLLDDFVEIIKSQDLSVVSKVVKVTIDYTEIS
EPI_ISL_424219      ------------------------------------------------------------
EPI_ISL_424222      ------------------------------------------------------------
EPI_ISL_424223      ------------------------------------------------------------
EPI_ISL_424226      ------------------------------------------------------------
EPI_ISL_424228      ------------------------------------------------------------
EPI_ISL_424230      ------------------------------------------------------------
EPI_ISL_424231      ------------------------------------------------------------
EPI_ISL_424240      ------------------------------------------------------------
EPI_ISL_424241      ------------------------------------------------------------
EPI_ISL_424243      ------------------------------------------------------------
EPI_ISL_424247      ------------------------------------------------------------
EPI_ISL_424249      ------------------------------------------------------------
EPI_ISL_424250      ------------------------------------------------------------
EPI_ISL_424252      ------------------------------------------------------------
EPI_ISL_424258      ------------------------------------------------------------
EPI_ISL_424268      ------------------------------------------------------------
EPI_ISL_424269      ------------------------------------------------------------
EPI_ISL_424275      ------------------------------------------------------------
EPI_ISL_424276      ------------------------------------------------------------
EPI_ISL_424279      ------------------------------------------------------------
```

```
ORF1ab         6781 FMLWCKDGHVETFYPKLQSSQAWQPGVAMPNLYKMQRMLLEKCDLQNYGDSATLPKGIMM
EPI_ISL_424219      ------------------------------------------------------------
EPI_ISL_424222      ------------------------------------------------------------
EPI_ISL_424223      ------------------------------------------------------------
EPI_ISL_424226      ------------------------------------------------------------
EPI_ISL_424228      ------------------------------------------------------------
EPI_ISL_424230      ------------------------------------------------------------
EPI_ISL_424231      ------------------------------------------------------------
EPI_ISL_424240      ------------------------------------------------------------
EPI_ISL_424241      ------------------------------------------------------------
EPI_ISL_424243      ------------------------------------------------------------
EPI_ISL_424247      ------------------------------------------------------------
EPI_ISL_424249      ------------------------------------------------------------
EPI_ISL_424250      ------------------------------------------------------------
EPI_ISL_424252      ------------------------------------------------------------
EPI_ISL_424258      ------------------------------------------------------------
EPI_ISL_424268      ------------------------------------------------------------
EPI_ISL_424269      ------------------------------------------------------------
EPI_ISL_424275      ------------------------------------------------------------
EPI_ISL_424276      ------------------------------------------------------------
```

EPI_ISL_424279   --

```
ORF1ab          6841 NVAKYTQLCQYLNTLTLAVPYNMRVIHFGAGSDKGVAPGTAVLRQWLPTGTLLVDSDLND
EPI_ISL_424219       ------------------------------------------------------------
EPI_ISL_424222       ------------------------------------------------------------
EPI_ISL_424223       ------------------------------------------------------------
EPI_ISL_424226       ------------------------------------------------------------
EPI_ISL_424228       ------------------------------------------------------------
EPI_ISL_424230       ------------------------------------------------------------
EPI_ISL_424231       ------------------------------------------------------------
EPI_ISL_424240       ------------------------------------------------------------
EPI_ISL_424241       ------------------------------------------------------------
EPI_ISL_424243       ------------------------------------------------------------
EPI_ISL_424247       ------------------------------------------------------------
EPI_ISL_424249       ------------------------------------------------------------
EPI_ISL_424250       ------------------------------------------------------------
EPI_ISL_424252       ------------------------------------------------------------
EPI_ISL_424258       ------------------------------------------------------------
EPI_ISL_424268       ------------------------------------------------------------
EPI_ISL_424269       ------------------------------------------------------------
EPI_ISL_424275       ------------------------------------------------------------
EPI_ISL_424276       ------------------------------------------------------------
EPI_ISL_424279       ------------------------------------------------------------

ORF1ab          6901 FVSDADSTLIGDCATVHTANKWDLIISDMYDPKTKNVTKENDSKEGFFTYICGFIQQKLA
EPI_ISL_424219       ------------------------------------------------------------
EPI_ISL_424222       ------------------------------------------------------------
EPI_ISL_424223       ------------------------------------------------------------
EPI_ISL_424226       ------------------------------------------------------------
EPI_ISL_424228       ------------------------------------------------------------
EPI_ISL_424230       ------------------------------------------------------------
EPI_ISL_424231       ------------------------------------------------------------
EPI_ISL_424240       ------------------------------------------------------------
EPI_ISL_424241       ------------------------------------------------------------
EPI_ISL_424243       ------------------------------------------------------------
EPI_ISL_424247       ------------------------------------------------------------
EPI_ISL_424249       ------------------------------------------------------------
EPI_ISL_424250       ------------------------------------------------------------
EPI_ISL_424252       ------------------------------------------------------------
EPI_ISL_424258       ------------------------------------------------------------
EPI_ISL_424268       ------------------------------------------------------------
EPI_ISL_424269       ------------------------------------------------------------
EPI_ISL_424275       ------------------------------------------------------------
EPI_ISL_424276       ------------------------------------------------------------
EPI_ISL_424279       ------------------------------------------------------------

ORF1ab          6961 LGGSVAIKITEHSWNADLYKLMGHFAWWTAFVTNVNASSSEAFLIGCNYLGKPREQIDGY
EPI_ISL_424219       ------------------------------------------------------------
EPI_ISL_424222       ------------------------------------------------------------
EPI_ISL_424223       ------------------------------------------------------------
EPI_ISL_424226       ------------------------------------------------------------
EPI_ISL_424228       ------------------------------------------------------------
EPI_ISL_424230       ------------------------------------------------------------
EPI_ISL_424231       ------------------------------------------------------------
EPI_ISL_424240       ------------------------------------------------------------
EPI_ISL_424241       ------------------------------------------------------------
EPI_ISL_424243       ------------------------------------------------------------
EPI_ISL_424247       ------------------------------------------------------------
EPI_ISL_424249       ------------------------------------------------------------
EPI_ISL_424250       ------------------------------------------------------------
EPI_ISL_424252       ------------------------------------------------------------
EPI_ISL_424258       ------------------------------------------------------------
EPI_ISL_424268       ------------------------------------------------------------
EPI_ISL_424269       ------------------------------------------------------------
EPI_ISL_424275       ------------------------------------------------------------
EPI_ISL_424276       ------------------------------------------------------------
```

EPI_ISL_424279   --

```
ORF1ab         7021 VMHANYIFWRNTNPIQLSSYSLFDMSKFPLKLRGTAVMSLKEGQINDMILSLLSKGRLII
EPI_ISL_424219      ------------------------------------------------------------
EPI_ISL_424222      ------------------------------------------------------------
EPI_ISL_424223      ------------------------------------------------------------
EPI_ISL_424226      ------------------------------------------------------------
EPI_ISL_424228      ------------------------------------------------------------
EPI_ISL_424230      ------------------------------------------------------------
EPI_ISL_424231      ------------------------------------------------------------
EPI_ISL_424240      ------------------------------------------------------------
EPI_ISL_424241      ------------------------------------------------------------
EPI_ISL_424243      ------------------------------------------------------------
EPI_ISL_424247      ------------------------------------------------------------
EPI_ISL_424249      ------------------------------------------------------------
EPI_ISL_424250      ------------------------------------------------------------
EPI_ISL_424252      ------------------------------------------------------------
EPI_ISL_424258      ------------------------------------------------------------
EPI_ISL_424268      ------------------------------------------------------------
EPI_ISL_424269      ------------------------------------------------------------
EPI_ISL_424275      ------------------------------------------------------------
EPI_ISL_424276      ------------------------------------------------------------
EPI_ISL_424279      ------------------------------------------------------------

ORF1ab         7081 RENNRVVISSDVLVNN
EPI_ISL_424219      ----------------
EPI_ISL_424222      ----------------
EPI_ISL_424223      ----------------
EPI_ISL_424226      ----------------
EPI_ISL_424228      ----------------
EPI_ISL_424230      ----------------
EPI_ISL_424231      ----------------
EPI_ISL_424240      ----------------
EPI_ISL_424241      ----------------
EPI_ISL_424243      ----------------
EPI_ISL_424247      ----------------
EPI_ISL_424249      ----------------
EPI_ISL_424250      ----------------
EPI_ISL_424252      ----------------
EPI_ISL_424258      ----------------
EPI_ISL_424268      ----------------
EPI_ISL_424269      ----------------
EPI_ISL_424275      ----------------
EPI_ISL_424276      ----------------
EPI_ISL_424279      ----------------
```

Clade V

```
ORF1ab            1 MESLVPGFNEKTHVQLSLPVLQVRDVLVRGFGDSVEEVLSEARQHLKDGTCGLVEVEKGV
EPI_ISL_424224    1 ------------------------------------------------------------
EPI_ISL_424255    1 ------------------------------------------------------------
EPI_ISL_425501    1 ------------------------------------------------------------
EPI_ISL_425502    1 ------------------------------------------------------------
EPI_ISL_425504    1 ------------------------------------------------------------
EPI_ISL_425523    1 ------------------------------------------------------------
EPI_ISL_425535    1 ------------------------------------------------------------
EPI_ISL_425537    1 ------------------------------------------------------------
EPI_ISL_425549    1 ------------------------------------------------------------
EPI_ISL_425551    1 ------------------------------------------------------------
EPI_ISL_425562    1 ------------------------------------------------------------
EPI_ISL_425576    1 ------------------------------------------------------------
EPI_ISL_425586    1 ------------------------------------------------------------
EPI_ISL_425587    1 ------------------------------------------------------------
EPI_ISL_426817    1 ------------------------------------------------------------
EPI_ISL_426832    1 ------------------------------------------------------------

ORF1ab           61 LPQLEQPYVFIKRSDARTAPHGHVMVELVAELEGIQYGRSGETLGVLVPHVGEIPVAYRK
EPI_ISL_424224    1 ------------------------------------------------------------
EPI_ISL_424255    1 ------------------------------------------------------------
EPI_ISL_425501    1 ------------------------------------------------------------
EPI_ISL_425502    1 ------------------------------------------------------------
EPI_ISL_425504    1 ------------------------------------------------------------
EPI_ISL_425523    1 ------------------------------------------------------------
EPI_ISL_425535    1 ------------------------------------------------------------
EPI_ISL_425537    1 ------------------------------------------------------------
EPI_ISL_425549    1 ------------------------------------------------------------
EPI_ISL_425551    1 ------------------------------------------------------------
EPI_ISL_425562    1 ------------------------------------------------------------
EPI_ISL_425576    1 ------------------------------------------------------------
EPI_ISL_425586    1 ------------------------------------------------------------
EPI_ISL_425587    1 ------------------------------------------------------------
EPI_ISL_426817    1 ------------------------------------------------------------
EPI_ISL_426832    1 ------------------------------------------------------------

ORF1ab          121 VLLRKNGNKGAGGHSYGADLKSFDLGDELGTDPYEDFQENWNTKHSSGVTRELMRELNGG
EPI_ISL_424224    1 ------------------------------------------------------------
EPI_ISL_424255    1 ------------------------------------------------------------
EPI_ISL_425501    1 ------------------------------------------------------------
EPI_ISL_425502    1 ------------------------------------------------------------
EPI_ISL_425504    1 ------------------------------------------------------------
EPI_ISL_425523    1 ------------------------------------------------------------
EPI_ISL_425535    1 ------------------------------------------------------------
EPI_ISL_425537    1 ------------------------------------------------------------
EPI_ISL_425549    1 ------------------------------------------------------------
EPI_ISL_425551    1 ------------------------------------------------------------
EPI_ISL_425562    1 ------------------------------------------------------------
EPI_ISL_425576    1 ------------------------------------------------------------
EPI_ISL_425586    1 ------------------------------------------------------------
EPI_ISL_425587    1 ------------------------------------------------------------
EPI_ISL_426817    1 ------------------------------------------------------------
EPI_ISL_426832    1 ------------------------------------------------------------

ORF1ab          181 AYTRYVDNNFCGPDGYPLECIKDLLARAGKASCTLSEQLDFIDTKRGVYCCREHEHEIAW
EPI_ISL_424224    1 ------------------------------------------------------------
EPI_ISL_424255    1 ------------------------------------------------------------
EPI_ISL_425501    1 ------------------------------------------------------------
EPI_ISL_425502    1 ------------------------------------------------------------
```

```
EPI_ISL_425504     1 ------------------------------------------------------------
EPI_ISL_425523     1 ------------------------------------------------------------
EPI_ISL_425535     1 ------------------------------------------------------------
EPI_ISL_425537     1 ------------------------------------------------------------
EPI_ISL_425549     1 ------------------------------------------------------------
EPI_ISL_425551     1 ------------------------------------------------------------
EPI_ISL_425562     1 ------------------------------------------------------------
EPI_ISL_425576     1 ------------------------------------------------------------
EPI_ISL_425586     1 ------------------------------------------------------------
EPI_ISL_425587     1 ------------------------------------------------------------
EPI_ISL_426817     1 ------------------------------------------------------------
EPI_ISL_426832     1 ------------------------------------------------------------
```

```
ORF1ab          241 YTERSEKSYELQTPFEIKLAKKFDTFNGECPNFVFPLNSIIKTIQPRVEKKKLDGFMGRI
EPI_ISL_424224    1 ------------------------------------------------------------
EPI_ISL_424255    1 ------------------------------------------------------------
EPI_ISL_425501    1 ------------------------------------------------------------
EPI_ISL_425502    1 ------------------------------------------------------------
EPI_ISL_425504    1 ------------------------------------------------------------
EPI_ISL_425523    1 ------------------------------------------------------------
EPI_ISL_425535    1 ------------------------------------------------------------
EPI_ISL_425537    1 ------------------------------------------------------------
EPI_ISL_425549    1 ------------------------------------------------------------
EPI_ISL_425551    1 ------------------------------------------------------------
EPI_ISL_425562    1 ------------------------------------------------------------
EPI_ISL_425576    1 ------------------------------------------------------------
EPI_ISL_425586    1 ------------------------------------------------------------
EPI_ISL_425587    1 ------------------------------------------------------------
EPI_ISL_426817    1 ------------------------------------------------------------
EPI_ISL_426832    1 ------------------------------------------------------------

ORF1ab          301 RSVYPVASPNECNQMCLSTLMKCDHCGETSWQTGDFVKATCEFCGTENLTKEGATTCGYL
EPI_ISL_424224    1 ------------------------------------------------------------
EPI_ISL_424255    1 ------------------------------------------------------------
EPI_ISL_425501    1 ------------------------------------------------------------
EPI_ISL_425502    1 ------------------------------------------------------------
EPI_ISL_425504    1 ------------------------------------------------------------
EPI_ISL_425523    1 ------------------------------------------------------------
EPI_ISL_425535    1 ------------------------------------------------------------
EPI_ISL_425537    1 ------------------------------------------------------------
EPI_ISL_425549    1 ------------------------------------------------------------
EPI_ISL_425551    1 ------------------------------------------------------------
EPI_ISL_425562    1 ------------------------------------------------------------
EPI_ISL_425576    1 ------------------------------------------------------------
EPI_ISL_425586    1 ------------------------------------------------------------
EPI_ISL_425587    1 ------------------------------------------------------------
EPI_ISL_426817    1 ------------------------------------------------------------
EPI_ISL_426832    1 ------------------------------------------------------------

ORF1ab          361 PQNAVVKIYCPACHNSEVGPEHSLAEYHNESGLKTILRKGGRTIAFGGCVFSYVGCHNKC
EPI_ISL_424224    1 ------------------------------------------------------------
EPI_ISL_424255    1 ------------------------------------------------------------
EPI_ISL_425501    1 ------------------------------------------------------------
EPI_ISL_425502    1 ------------------------------------------------------------
EPI_ISL_425504    1 ------------------------------------------------------------
EPI_ISL_425523    1 ------------------------------------------------------------
EPI_ISL_425535    1 ------------------------------------------------------------
EPI_ISL_425537    1 ------------------------------------------------------------
EPI_ISL_425549    1 ------------------------------------------------------------
EPI_ISL_425551    1 ------------------------------------------------------------
EPI_ISL_425562    1 ------------------------------------------------------------
EPI_ISL_425576    1 ------------------------------------------------------------
EPI_ISL_425586    1 ------------------------------------------------------------
EPI_ISL_425587    1 ------------------------------------------------------------
EPI_ISL_426817    1 ------------------------------------------------------------
EPI_ISL_426832    1 ------------------------------------------------------------
```

```
ORF1ab          421 AYWVPRASANIGCNHTGVVGEGSEGLNDNLLEILQKEKVNINIVGDFKLNEEIAIILASF
EPI_ISL_424224    1 ------------------------------------------------------------
EPI_ISL_424255    1 ------------------------------------------------------------
EPI_ISL_425501    1 ------------------------------------------------------------
EPI_ISL_425502    1 ------------------------------------------------------------
EPI_ISL_425504    1 ------------------------------------------------------------
EPI_ISL_425523    1 ------------------------------------------------------------
EPI_ISL_425535    1 ------------------------------------------------------------
EPI_ISL_425537    1 ------------------------------------------------------------
EPI_ISL_425549    1 ------------------------------------------------------------
EPI_ISL_425551    1 ------------------------------------------------------------
EPI_ISL_425562    1 ------------------------------------------------------------
EPI_ISL_425576    1 ------------------------------------------------------------
EPI_ISL_425586    1 ------------------------------------------------------------
EPI_ISL_425587    1 ------------------------------------------------------------
EPI_ISL_426817    1 ------------------------------------------------------------
EPI_ISL_426832    1 ------------------------------------------------------------

ORF1ab          481 SASTSAFVETVKGLDYKAFKQIVESCGNFKVTKGKAKKGAWNIGEQKSILSPLYAFASEA
EPI_ISL_424224    1 ------------------------------------------------------------
EPI_ISL_424255    1 ------------------------------------------------------------
EPI_ISL_425501    1 ------------------------------------------------------------
EPI_ISL_425502    1 ------------------------------------------------------------
EPI_ISL_425504    1 ------------------------------------------------------------
EPI_ISL_425523    1 ------------------------------------------------------------
EPI_ISL_425535    1 ------------------------------------------------------------
EPI_ISL_425537    1 ------------------------------------------------------------
EPI_ISL_425549    1 ------------------------------------------------------------
EPI_ISL_425551    1 ------------------------------------------------------------
EPI_ISL_425562    1 ------------------------------------------------------------
EPI_ISL_425576    1 ------------------------------------------------------------
EPI_ISL_425586    1 ------------------------------------------------------------
EPI_ISL_425587    1 ------------------------------------------------------------
EPI_ISL_426817    1 ------------------------------------------------------------
EPI_ISL_426832    1 ------------------------------------------------------------

ORF1ab          541 ARVVRSIFSRTLETAQNSVRVLQKAAITILDGISQYSLRLIDAMMFTSDLATNNLVVMAY
EPI_ISL_424224    1 ------------------------------------------------------------
EPI_ISL_424255    1 ------------------------------------------------------------
EPI_ISL_425501    1 ------------------------------------------------------------
EPI_ISL_425502    1 ------------------------------------------------------------
EPI_ISL_425504    1 ------------------------------------------------------------
EPI_ISL_425523    1 ------------------------------------------------------------
EPI_ISL_425535    1 ------------------------------------------------------------
EPI_ISL_425537    1 ------------------------------------------------------------
EPI_ISL_425549    1 ------------------------------------------------------------
EPI_ISL_425551    1 ------------------------------------------------------------
EPI_ISL_425562    1 ------------------------------------------------------------
EPI_ISL_425576    1 ------------------------------------------------------------
EPI_ISL_425586    1 ------------------------------------------------------------
EPI_ISL_425587    1 ------------------------------------------------------------
EPI_ISL_426817    1 ------------------------------------------------------------
EPI_ISL_426832    1 ------------------------------------------------------------

ORF1ab          601 ITGGVVQLTSQWLTNIFGTVYEKLKPVLDWLEEKFKEGVEFLRDGWEIVKFISTCACEIV
EPI_ISL_424224    1 ------------------------------------------------------------
EPI_ISL_424255    1 ------------------------------------------------------------
EPI_ISL_425501    1 ------------------------------------------------------------
EPI_ISL_425502    1 ------------------------------------------------------------
EPI_ISL_425504    1 ------------------------------------------------------------
EPI_ISL_425523    1 ------------------------------------------------------------
EPI_ISL_425535    1 ------------------------------------------------------------
EPI_ISL_425537    1 ------------------------------------------------------------
```

EPI_ISL_425549   1 --
EPI_ISL_425551   1 --
EPI_ISL_425562   1 --
EPI_ISL_425576   1 --
EPI_ISL_425586   1 --
EPI_ISL_425587   1 --
EPI_ISL_426817   1 --
EPI_ISL_426832   1 --

```
ORF1ab          661 GGQIVTCAKEIKESVQTFFKLVNKFLALCADSIIIGGAKLKALNLGETFVTHSKGLYRKC
EPI_ISL_424224    1 ------------------------------------------------------------
EPI_ISL_424255    1 ------------------------------------------------------------
EPI_ISL_425501    1 ------------------------------------------------------------
EPI_ISL_425502    1 ------------------------------------------------------------
EPI_ISL_425504    1 ------------------------------------------------------------
EPI_ISL_425523    1 ------------------------------------------------------------
EPI_ISL_425535    1 ------------------------------------------------------------
EPI_ISL_425537    1 ------------------------------------------------------------
EPI_ISL_425549    1 ------------------------------------------------------------
EPI_ISL_425551    1 ------------------------------------------------------------
EPI_ISL_425562    1 ------------------------------------------------------------
EPI_ISL_425576    1 ------------------------------------------------------------
EPI_ISL_425586    1 ------------------------------------------------------------
EPI_ISL_425587    1 ------------------------------------------------------------
EPI_ISL_426817    1 ------------------------------------------------------------
EPI_ISL_426832    1 ------------------------------------------------------------

ORF1ab          721 VKSREETGLLMPLKAPKEIIFLEGETLPTEVLTEEVVLKTGDLQPLEQPTSEAVEAPLVG
EPI_ISL_424224    1 ------------------------------------------------------------
EPI_ISL_424255    1 ------------------------------------------------------------
EPI_ISL_425501    1 ------------------------------------------------------------
EPI_ISL_425502    1 ------------------------------------------------------------
EPI_ISL_425504    1 ------------------------------------------------------------
EPI_ISL_425523    1 ------------------------------------------------------------
EPI_ISL_425535    1 ------------------------------------------------------------
EPI_ISL_425537    1 ------------------------------------------------------------
EPI_ISL_425549    1 ------------------------------------------------------------
EPI_ISL_425551    1 ------------------------------------------------------------
EPI_ISL_425562    1 ------------------------------------------------------------
EPI_ISL_425576    1 ------------------------------------------------------------
EPI_ISL_425586    1 ------------------------------------------------------------
EPI_ISL_425587    1 ------------------------------------------------------------
EPI_ISL_426817    1 ------------------------------------------------------------
EPI_ISL_426832    1 ------------------------------------------------------------

ORF1ab          781 TPVCINGLMLLEIKDTEKYCALAPNMMVTNNTFTLKGGAPTKVTFGDDTVIEVQGYKSVN
EPI_ISL_424224    1 --------------------------------------APTKVTFGDDTVIEVQGYKSVN
EPI_ISL_424255    1 --------------------------------------APTKVTFGDDTVIEVQGYKSVN
EPI_ISL_425501    1 --------------------------------------APTKVTFGDDTVIEVQGYKSVN
EPI_ISL_425502    1 --------------------------------------APTKVTFGDDTVIEVQGYKSVN
EPI_ISL_425504    1 --------------------------------------APTKVTFGDDTVIEVQGYKSVN
EPI_ISL_425523    1 --------------------------------------APTKVTFGDDTVIEVQGYKSVN
EPI_ISL_425535    1 --------------------------------------APTKVTFGDDTVIEVQGYKSVN
EPI_ISL_425537    1 --------------------------------------APTKVTFGDDTVIEVQGYKSVN
EPI_ISL_425549    1 --------------------------------------APTKVTFGDDTVIEVQGYKSVN
EPI_ISL_425551    1 --------------------------------------APTKVTFGDDTVIEVQGYKSVN
EPI_ISL_425562    1 --------------------------------------APTKVTFGDDTVIEVQGYKSVN
EPI_ISL_425576    1 --------------------------------------APTKVTFGDDTVIEVQGYKSVN
EPI_ISL_425586    1 --------------------------------------APTKVTFGDDTVIEVQGYKSVN
EPI_ISL_425587    1 --------------------------------------APTKVTFGDDTVIEVQGYKSVN
EPI_ISL_426817    1 --------------------------------------APTKVTFGDDTVIEVQGYKSVN
EPI_ISL_426832    1 --------------------------------------APTKVTFGDDTVIEVQGYKSVN
```

```
ORF1ab          841 ITFELDERIDKVLNEKCSAYTVELGTEVNEFACVVADAVIKTLQPVSELLTPLGIDLDEW
EPI_ISL_424224   23 ITFELDERIDKVLNEKCSAYTVELGTEVNEFACVVADAVIKTLQPVSELLTPLGIGFYEW
EPI_ISL_424255   23 ITFELDERIDKVLNEKCSAYTVELGTEVNEFACVVADAVIKTLQPVSELLTPLGIDLDEW
EPI_ISL_425501   23 ITFELDERIDKVLNEKCSAYTVELGTEVNEFACVVADAVIKTLQPVSELLTPLGIDLDEW
EPI_ISL_425502   23 ITFELDERIDKVLNEKCSAYTVELGTEVNEFACVVADAVIKTLQPVSELLTPLGIDLDEW
EPI_ISL_425504   23 ITFELDERIDKVLNEKCSAYTVELGTEVNEFACVVADAVIKTLQPVSELLTPLGIDLDEW
EPI_ISL_425523   23 ITFELDERIDKVLNEKCSAYTVELGTEVNEFACVVADAVIKTLQPVSELLTPLGIDLDEW
EPI_ISL_425535   23 ITFELDERIDKVLNEKCSAYTVELGTEVNEFACVVADAVIKTLQPVSELLTPLGIDLDEW
EPI_ISL_425537   23 ITFELDERIDKVLNEKCSAYTVELGTEVNEFACVVADAVIKTLQPVSELLTPLGIDLDEW
EPI_ISL_425549   23 ITFELDERIDKVLNEKCSAYTVELGTEVNEFACVVADAVIKTLQPVSELLTPLGIDLDEW
EPI_ISL_425551   23 ITFELDERIDKVLNEKCSAYTVELGTEVNEFACVVADAVIKTLQPVSELLTPLGIDLDEW
EPI_ISL_425562   23 ITFELDERIDKVLNEKCSAYTVELGTEVNEFACVVADAVIKTLQPVSELLTPLGIDLDEW
EPI_ISL_425576   23 ITFELDERIDKVLNEKCSAYTVELGTEVNEFACVVADAVIKTLQPVSELLTPLGIDLDEW
EPI_ISL_425586   23 ITFELDERIDKVLNEKCSAYTVELGTEVNEFACVVADAVIKTLQPVSELLTPLGIDLDEW
EPI_ISL_425587   23 ITFELDERIDKVLNEKCSAYTVELGTEVNEFACVVADAVIKTLQPVSELLTPLGIDLDEW
EPI_ISL_426817   23 ITFELDERIDKVLNEKCSAYTVELGTEVNEFACVVADAVIKTLQPVSELLTPLGIDLDEW
EPI_ISL_426832   23 ITFELDERIDKVLNEKCSAYTVELGTEVNEFACVVADAVIKTLQPVSELLTPLGIDLDEW

ORF1ab          901 SMATYYLFDESGEFKLASHMYCSFYPPDEDEEEGDCEEEEFEPSTQYEYGTEDDYQGKPL
EPI_ISL_424224   83 SMATYYLFDESGEFKLASHMYCSFYPPDEDEEEGDCEEEEFEPSTQYEYGTEDDYQGKPL
EPI_ISL_424255   83 SMATYYLFDESGEFKLASHMYCSFYPPDEDEEEGDCEEEEFEPSTQYEYGTEDDYQGKPL
EPI_ISL_425501   83 SMATYYLFDESGEFKLASHMYCSFYPPDEDEEEGDCEEEEFEPSTQYEYGTEDDYQGKPL
EPI_ISL_425502   83 SMATYYLFDESGEFKLASHMYCSFYPPDEDEEEGDCEEEEFEPSTQYEYGTEDDYQGKPL
EPI_ISL_425504   83 SMATYYLFDESGEFKLASHMYCSFYPPDEDEEEGDCEEEEFEPSTQYEYGTEDDYQGKPL
EPI_ISL_425523   83 SMATYYLFDESGEFKLASHMYCSFYPPDEDEEEGDCEEEEFEPSTQYEYGTEDDYQGKPL
EPI_ISL_425535   83 SMATYYLFDESGEFKLASHMYCSFYPPDEDEEEGDCEEEEFEPSTQYEYGTEDDYQGKPL
EPI_ISL_425537   83 SMATYYLFDESGEFKLASHMYCSFYPPDEDEEEGDCEEEEFEPSTQYEYGTEDDYQGKPL
EPI_ISL_425549   83 SMATYYLFDESGEFKLASHMYCSFYPPDEDEEEGDCEEEEFEPSTQYEYGTEDDYQGKPL
EPI_ISL_425551   83 SMATYYLFDESGEFKLASHMYCSFYPPDEDEEEGDCEEEEFEPSTQYEYGTEDDYQGKPL
EPI_ISL_425562   83 SMATYYLFDESGEFKLASHMYCSFYPPDEDEEEGDCEEEEFEPSTQYEYGTEDDYQGKPL
EPI_ISL_425576   83 SMATYYLFDESGEFKLASHMYCSFYPPDEDEEEGDCEEEEFEPSTQYEYGTEDDYQGKPL
EPI_ISL_425586   83 SMATYYLFDESGEFKLASHMYCSFYPPDEDEEEGDCEEEEFEPSTQYEYGTEDDYQGKPL
EPI_ISL_425587   83 SMATYYLFDESGEFKLASHMYCSFYPPDEDEEEGDCEEEEFEPSTQYEYGTEDDYQGKPL
EPI_ISL_426817   83 SMATYYLFDESGEFKLASHMYCSFYPPDEDEEEGDCEEEEFEPSTQYEYGTEDDYQGKPL
EPI_ISL_426832   83 SMATYYLFDESGEFKLASHMYCSFYPPDEDEEEGDCEEEEFEPSTQYEYGTEDDYQGKPL

ORF1ab          961 EFGATSAALQPEEEQEEDWLDDDSQQTVGQQDGSEDNQTTTIQTIVEVQPQLEMELTPVV
EPI_ISL_424224  143 EFGATSAALQPEEEQEEDWLDDDSQQTVGQQDGSEDNQTTTIQTIVEVQPQLEMELTPVV
EPI_ISL_424255  143 EFGATSAALQPEEEQEEDWLDDDSQQTVGQQDGSEDNQTTTIQTIVEVQPQLEMELTPVV
EPI_ISL_425501  143 EFGATSAALQPEEEQEEDWLDDDSQQTVGQQDGSEDNQTTTIQTIVEVQPQLEMELTPVV
EPI_ISL_425502  143 EFGATSAALQPEEEQEEDWLDDDSQQTVGQQDGSEDNQTTTIQTIVEVQPQLEMELTPVV
EPI_ISL_425504  143 EFGATSAALQPEEEQEEDWLDDDSQQTVGQQDGSEDNQTTTIQTIVEVQPQLEMELTPVV
EPI_ISL_425523  143 EFGATSAALQPEEEQEEDWLDDDSQQTVGQQDGSEDNQTTTIQTIVEVQPQLEMELTPVV
EPI_ISL_425535  143 EFGATSAALQPEEEQEEDWLDDDSQQTVGQQDGSEDNQTTTIQTIVEVQPQLEMELTPVV
EPI_ISL_425537  143 EFGATSAALQPEEEQEEDWLDDDSQQTVGQQDGSEDNQTTTIQTIVEVQPQLEMELTPVV
EPI_ISL_425549  143 EFGATSAALQPEEEQEEDWLDDDSQQTVGQQDGSEDNQTTTIQTIVEVQPQLEMELTPVV
EPI_ISL_425551  143 EFGATSAALQPEEEQEEDWLDDDSQQTVGQQDGSEDNQTTTIQTIVEVQPQLEMELTPVV
EPI_ISL_425562  143 EFGATSAALQPEEEQEEDWLDDDSQQTVGQQDGSEDNQTTTIQTIVEVQPQLEMELTPVV
EPI_ISL_425576  143 EFGATSAALQPEEEQEEDWLDDDSQQTVGQQDGSEDNQTTTIQTIVEVQPQLEMELTPVV
EPI_ISL_425586  143 EFGATSAALQPEEEQEEDWLDDDSQQTVGQQDGSEDNQTTTIQTIVEVQPQLEMELTPVV
EPI_ISL_425587  143 EFGATSAALQPEEEQEEDWLDDDSQQTVGQQDGSEDNQTTTIQTIVEVQPQLEMELTPVV
EPI_ISL_426817  143 EFGATSAALQPEEEQEEDWLDDDSQQTVGQQDGSEDNQTTTIQTIVEVQPQLEMELTPVV
EPI_ISL_426832  143 EFGATSAALQPEEEQEEDWLDDDSQQTVGQQDGSEDNQTTTIQTIVEVQPQLEMELTPVV

ORF1ab         1021 QTIEVNSFSGYLKLTDNVYIKNADIVEEAKKVKPTVVVNAANVYLKHGGGVAGALNKATN
EPI_ISL_424224  203 QTIEVNSFSGYLKLTDNVYIKNADIVEEAKKVKPTVVVNAANVYLKHGGGVAGALNKATN
EPI_ISL_424255  203 QTIEVNSFSGYLKLTDNVYIKNADIVEEAKKVKPTVVVNAANVYLKHGGGVAGALNKATN
EPI_ISL_425501  203 QTIEVNSFSGYLKLTDNVYIKNADIVEEAKKVKPTVVVNAANVYLKHGGGVAGALNKATN
EPI_ISL_425502  203 QTIEVNSFSGYLKLTDNVYIKNADIVEEAKKVKPTVVVNAANVYLKHGGGVAGALNKATN
EPI_ISL_425504  203 QTIEVNSFSGYLKLTDNVYIKNADIVEEAKKVKPTVVVNAANVYLKHGGGVAGALNKATN
EPI_ISL_425523  203 QTIEVNSFSGYLKLTDNVYIKNADIVEEAKKVKPTVVVNAANVYLKHGGGVAGALNKATN
EPI_ISL_425535  203 QTIEVNSFSGYLKLTDNVYIKNADIVEEAKKVKPTVVVNAANVYLKHGGGVAGALNKATN
EPI_ISL_425537  203 QTIEVNSFSGYLKLTDNVYIKNADIVEEAKKVKPTVVVNAANVYLKHGGGVAGALNKATN
```

EPI_ISL_425549	203	QTIEVNSFSGYLKLTDNVYIKNADIVEEAKKVKPTVVVNAANVYLKHGGGVAGALNKATN
EPI_ISL_425551	203	QTIEVNSFSGYLKLTDNVYIKNADIVEEAKKVKPTVVVNAANVYLKHGGGVAGALNKATN
EPI_ISL_425562	203	QTIEVNSFSGYLKLTDNVYIKNADIVEEAKKVKPTVVVNAANVYLKHGGGVAGALNKATN
EPI_ISL_425576	203	QTIEVNSFSGYLKLTDNVYIKNADIVEEAKKVKPTVVVNAANVYLKHGGGVAGALNKATN
EPI_ISL_425586	203	QTIEVNSFSGYLKLTDNVYIKNADIVEEAKKVKPTVVVNAANVYLKHGGGVAGALNKATN
EPI_ISL_425587	203	QTIEVNSFSGYLKLTDNVYIKNADIVEEAKKVKPTVVVNAANVYLKHGGGVAGALNKATN
EPI_ISL_426817	203	QTIEVNSFSGYLKLTDNVYIKNADIVEEAKKVKPTVVVNAANVYLKHGGGVAGALNKATN
EPI_ISL_426832	203	QTIEVNSFSGYLKLTDNVYIKNADIVEEAKKVKPTVVVNAANVYLKHGGGVAGALNKATN

```
ORF1ab          1081 NAMQVESDDYIATNGPLKVGGSCVLSGHNLAKHCLHVVGPNVNKGEDIQLLKSAYENFNQ
EPI_ISL_424224   263 NAMQVESDDYIATNGPLKVGGSCVLSGHNLAKHCLHVVGPNVNKGEDIQLLKSAYENFNQ
EPI_ISL_424255   263 NAMQVESDDYIATNGPLKVXGSCVLSGHNLAKHCLHVVGPNVNKGEDIQLLKSAYENFNQ
EPI_ISL_425501   263 NAMQVESDDYIATNGPLKVGGSCVLSGHNLAKHCLHVVGPNVNKGEDIQLLKSAYENFNQ
EPI_ISL_425502   263 NAMQVESDDYIATNGPLKVGGSCVLSGHNLAKHCLHVVGPNVNKGEDIQLLKSAYENFNQ
EPI_ISL_425504   263 NAMQVESDDYIATNGPLKVGGSCVLSGHNLAKHCLHVVGPNVNKGEDIQLLKSAYENFNQ
EPI_ISL_425523   263 NAMQVESDDYIATNGPLKVGGSCVLSGHNLAKHCLHVVGPNVNKGEDIQLLKSAYENFNQ
EPI_ISL_425535   263 NAMQVESDDYIATNGPLKVGGSCVLSGHNLAKHCLHVVGPNVNKGEDIQLLKSAYENFNQ
EPI_ISL_425537   263 NAMQVESDDYIATNGPLKVGGSCVLSGHNLAKHCLHVVGPNVNKGEDIQLLKSAYENFNQ
EPI_ISL_425549   263 NAMQVESDDYIATNGPLKVGGSCVLSGHNLAKHCLHVVGPNVNKGEDIQLLKSAYENFNQ
EPI_ISL_425551   263 NAMQVESDDYIATNGPLKVGGSCVLSGHNLAKHCLHVVGPNVNKGEDIQLLKSAYENFNQ
EPI_ISL_425562   263 NAMQVESDDYIATNGPLKVGGSCVLSGHNLAKHCLHVVGPNVNKGEDIQLLKSAYENFNQ
EPI_ISL_425576   263 NAMQVESDDYIATNGPLKVGGSCVLSGHNLAKHCLHVVGPNVNKGEDIQLLKSAYENFNQ
EPI_ISL_425586   263 NAMQVESDDYIATNGPLKVGGSCVLSGHNLAKHCLHVVGPNVNKGEDIQLLKSAYENFNQ
EPI_ISL_425587   263 NAMQVESDDYIATNGPLKVGGSCVLSGHNLAKHCLHVVGPNVNKGEDIQLLKSAYENFNQ
EPI_ISL_426817   263 NAMQVESDDYIATNGPLKVGGSCVLSGHNLAKHCLHVVGPNVNKGEDIQLLKSAYENFNQ
EPI_ISL_426832   263 NAMQVESDDYIATNGPLKVGGSCVLSGHNLAKHCLHVVGPNVNKGEDIQLLKSAYENFNQ

ORF1ab          1141 HEVLLAPLLSAGIFGADPIHSLRVCVDTVRTNVYLAVFDKNLYDKLVSSFLEMKSEKQVE
EPI_ISL_424224   323 HEVLLAPLLSAGIFGADPIHSLRVCVDTVRTNVYLAVFDKNLYDKLVSSFLEMKSEKQVE
EPI_ISL_424255   323 HEVLLAPLLSAGIFGADPIHSLRVCVDTVRTNVYLAVFDKNLYDKLVSSFLEMKSEKQVE
EPI_ISL_425501   323 HEVLLAPLLSAGIFGADPIHSLRVCVDTVRTNVYLAVFDKNLYDKLVSSFLEMKSEKQVE
EPI_ISL_425502   323 HEVLLAPLLSAGIFGADPIHSLRVCVDTVRTNVYLAVFDKNLYDKLVSSFLEMKSEKQVE
EPI_ISL_425504   323 HEVLLAPLLSAGIFGADPIHSLRVCVDTVRTNVYLAVFDKNLYDKLVSSFLEMKSEKQVE
EPI_ISL_425523   323 HEVLLAPLLSAGIFGADPIHSLRVCVDTVRTNVYLAVFDKNLYDKLVSSFLEMKSEKQVE
EPI_ISL_425535   323 HEVLLAPLLSAGIFGADPIHSLRVCVDTVRTNVYLAVFDKNLYDKLVSSFLEMKSEKQVE
EPI_ISL_425537   323 HEVLLAPLLSAGIFGADPIHSLRVCVDTVRTNVYLAVFDKNLYDKLVSSFLEMKSEKQVE
EPI_ISL_425549   323 HEVLLAPLLSAGIFGADPIHSLRVCVDTVRTNVYLAVFDKNLYDKLVSSFLEMKSEKQVE
EPI_ISL_425551   323 HEVLLAPLLSAGIFGADPIHSLRVCVDTVRTNVYLAVFDKNLYDKLVSSFLEMKSEKQVE
EPI_ISL_425562   323 HEVLLAPLLSAGIFGADPIHSLRVCVDTVRTNVYLAVFDKNLYDKLVSSFLEMKSEKQVE
EPI_ISL_425576   323 HEVLLAPLLSAGIFGADPIHSLRVCVDTVRTNVYLAVFDKNLYDKLVSSFLEMKSEKQVE
EPI_ISL_425586   323 HEVLLAPLLSAGIFGADPIHSLRVCVDTVRTNVYLAVFDKNLYDKLVSSFLEMKSEKQVE
EPI_ISL_425587   323 HEVLLAPLLSAGIFGADPIHSLRVCVDTVRTNVYLAVFDKNLYDKLVSSFLEMKSEKQVE
EPI_ISL_426817   323 HEVLLAPLLSAGIFGADPIHSLRVCVDTVRTNVYLAVFDKNLYDKLVSSFLEMKSEKQVE
EPI_ISL_426832   323 HEVLLAPLLSAGIFGADPIHSLRVCVDTVRTNVYLAVFDKNLYDKLVSSFLEMKSEKQVE

ORF1ab          1201 QKIAEIPKEEVKPFITESKPSVEQRKQDDKKIKACVEEVTTTLEETKFLTENLLLYIDIN
EPI_ISL_424224   383 QKIAEIPKEEVKPFITESKPSVEQRKQDDKKIKACVEEVTTTLEETKFLTENLLLYIDIN
EPI_ISL_424255   383 QKIAEIPKEEVKPFITESKPSVEQRKQDDKKIKACVEEVTTTLEETKFLTENLLLYIDIN
EPI_ISL_425501   383 QKIAEIPKEEVKPFITESKPSVEQRKQDDKKIKACVEEVTTTLEETKFLTENLLLYIDIN
EPI_ISL_425502   383 QKIAEIPKEEVKPFITESKPSVEQRKQDDKKIKACVEEVTTTLEETKFLTENLLLYIDIN
EPI_ISL_425504   383 QKIAEIPKEEVKPFITESKPSVEQRKQDDKKIKACVEEVTTTLEETKFLTENLLLYIDIN
EPI_ISL_425523   383 QKIAEIPKEEVKPFITESKPSVEQRKQDDKKIKACVEEVTTTLEETKFLTENLLLYIDIN
EPI_ISL_425535   383 QKIAEIPKEEVKPFITESKPSVEQRKQDDKKIKACVEEVTTTLEETKFLTENLLLYIDIN
EPI_ISL_425537   383 QKIAEIPKEEVKPFITESKPSVEQRKQDDKKIKACVEEVTTTLEETKFLTENLLLYIDIN
EPI_ISL_425549   383 QKIAEIPKEEVKPFITESKPSVEQRKQDDKKIKACVEEVTTTLEETKFLTENLLLYIDIN
EPI_ISL_425551   383 QKIAEIPKEEVKPFITESKPSVEQRKQDDKKIKACVEEVTTTLEETKFLTENLLLYIDIN
EPI_ISL_425562   383 QKIAEIPKEEVKPFITESKPSVEQRKQDDKKIKACVEEVTTTLEETKFLTENLLLYIDIN
EPI_ISL_425576   383 QKIAEIPKEEVKPFITESKPSVEQRKQDDKKIKACVEEVTTTLEETKFLTENLLLYIDIN
EPI_ISL_425586   383 QKIAEIPKEEVKPFITESKPSVEQRKQDDKKIKACVEEVTTTLEETKFLTENLLLYIDIN
EPI_ISL_425587   383 QKIAEIPKEEVKPFITESKPSVEQRKQDDKKIKACVEEVTTTLEETKFLTENLLLYIDIN
EPI_ISL_426817   383 QKIAEIPKEEVKPFITESKPSVEQRKQDDKKIKACVEEVTTTLEETKFLTENLLLYIDIN
EPI_ISL_426832   383 QKIAEIPKEEVKPFITESKPSVEQRKQDDKKIKACVEEVTTTLEETKFLTENLLLYIDIN
```

```
ORF1ab           1261 GNLHPDSATLVSDIDITFLKKDAPYIVGDVVQEGVLTAVVIPTKKAGGTTEMLAKALRKV
EPI_ISL_424224    443 GNLHPDSATLVSDIDITFLKKDAPYIVGDVVQEGVLTAVVIPTKKAGGTTEMLAKALRKV
EPI_ISL_424255    443 GNLHPDSATLVSDIDITFLKKDAPYIVGDVVQEGVLTAVVIPTKKAGGTTEMLAKALRKV
EPI_ISL_425501    443 GNLHPDSATLVSDIDITFLKKDAPYIVGDVVQEGVLTAVVIPTKKAGGTTEMLAKALRKV
EPI_ISL_425502    443 GNLHPDSATLVSDIDITFLKKDAPYIVGDVVQEGVLTAVVIPTKKAGGTTEMLAKALRKV
EPI_ISL_425504    443 GNLHPDSATLVSDIDITFLKKDAPYIVGDVVQEGVLTAVVIPTKKAGGTTEMLAKALRKV
EPI_ISL_425523    443 GNLHPDSATLVSDIDITFLKKDAPYIVGDVVQEGVLTAVVIPTKKAGGTTEMLAKALRKV
EPI_ISL_425535    443 GNLHPDSATLVSDIDITFLKKDAPYIVGDVVQEGVLTAVVIPTKKAGGTTEMLAKALRKV
EPI_ISL_425537    443 GNLHPDSATLVSDIDITFLKKDAPYIVGDVVQEGVLTAVVIPTKKAGGTTEMLAKALRKV
EPI_ISL_425549    443 GNLHPDSATLVSDIDITFLKKDAPYIVGDVVQEGVLTAVVIPTKKAGGTTEMLAKALRKV
EPI_ISL_425551    443 GNLHPDSATLVSDIDITFLKKDAPYIVGDVVQEGVLTAVVIPTKKAGGTTEMLAKALRKV
EPI_ISL_425562    443 GNLHPDSATLVSDIDITFLKKDAPYIVGDVVQEGVLTAVVIPTKKAGGTTEMLAKALRKV
EPI_ISL_425576    443 GNLHPDSATLVSDIDITFLKKDAPYIVGDVVQEGVLTAVVIPTKKAGGTTEMLAKALRKV
EPI_ISL_425586    443 GNLHPDSATLVSDIDITFLKKDAPYIVGDVVQEGVLTAVVIPTKKAGGTTEMLAKALRKV
EPI_ISL_425587    443 GNLHPDSATLVSDIDITFLKKDAPYIVGDVVQEGVLTAVVIPTKKAGGTTEMLAKALRKV
EPI_ISL_426817    443 GNLHPDSATLVSDIDITFLKKDAPYIVGDVVQEGVLTAVVIPTKKAGGTTEMLAKALRKV
EPI_ISL_426832    443 GNLHPDSATLVSDIDITFLKKDAPYIVGDVVQEGVLTAVVIPTKKAGGTTEMLAKALRKV

ORF1ab           1321 PTDNYITTYPGQGLNGYTVEEAKTVLKKCKSAFYILPSIISNEKQEILGTVSWNLREMLA
EPI_ISL_424224    503 PTDSYITTYPGQGLNGYTVEEAKTVLKKCKSAFYILPSIISNEKQEILGTVSWNLREMLA
EPI_ISL_424255    503 PTDNYITTYPGQGLNGYTVEEAKTVLKKCKSAFYILPSIISNEKQEILGTVSWNLREMLA
EPI_ISL_425501    503 PTDSYITTYPGQGLNGYTVEEAKTVLKKCKSAFYILPSIISNEKQEILGTVSWNLREMLA
EPI_ISL_425502    503 PTDNYITTYPGQGLNGYTVEEAKTVLKKCKSAFYILPSIISNEKQEILGTVSWNLREMLA
EPI_ISL_425504    503 PTDNYITTYPGQGLNGYTVEEAKTVLKKCKSAFYILPSIISNEKQEILGTVSWNLREMLA
EPI_ISL_425523    503 PTDNYITTYPGQGLNGYTVEEAKTVLKKCKSAFYILPSIISNEKQEILGTVSWNLREMLA
EPI_ISL_425535    503 PTDNYITTYPGQGLNGYTVEEAKTVLKKCKSAFYILPSIISNEKQEILGTVSWNLREMLA
EPI_ISL_425537    503 PTDNYITTYPGQGLNGYTVEEAKTVLKKCKSAFYILPSIISNEKQEILGTVSWNLREMLA
EPI_ISL_425549    503 PTDNYITTYPGQGLNGYTVEEAKTVLKKCKSAFYILPSIISNEKQEILGTVSWNLREMLA
EPI_ISL_425551    503 PTDNYITTYPGQGLNGYTVEEAKTVLKKCKSAFYILPSIISNEKQEILGTVSWNLREMLA
EPI_ISL_425562    503 PTDNYITTYPGQGLNGYTVEEAKTVLKKCKSAFYILPSIISNEKQEILGTVSWNLREMLA
EPI_ISL_425576    503 PTDNYITTYPGQGLNGYTVEEAKTVLKKCKSAFYILPSIISNEKQEILGTVSWNLREMLA
EPI_ISL_425586    503 PTDNYITTYPGQGLNGYTVEEAKTVLKKCKSAFYILPSIISNEKQEILGTVSWNLREMLA
EPI_ISL_425587    503 PTDNYITTYPGQGLNGYTVEEAKTVLKKCKSAFYILPSIISNEKQEILGTVSWNLREMLA
EPI_ISL_426817    503 PTDNYITTYPGQGLNGYTVEEAKTVLKKCKSAFYILPSIISNEKQEILGTVSWNLREMLA
EPI_ISL_426832    503 PTDNYITTYPGQGLNGYTVEEAKTVLKKCKSAFYILPSIISNEKQEILGTVSWNLREMLA

ORF1ab           1381 HAEETRKLMPVCVETKAIVSTIQRKYKGIKIQEGVVDYGARFYFYTSKTTVASLINTLND
EPI_ISL_424224    563 HAEETRKLMPVCVETKAIVSTIQRKYKGIKIQEGVVDYGARFYFYTSKTTVASLINTLND
EPI_ISL_424255    563 HAEETRKLMPVCVETKAIVSTIQRKYKGIKIQEGVVDYGARFYFYTSKTTVASLINTLND
EPI_ISL_425501    563 HAEETRKLMPVCVETKAIVSTIQRKYKGIKIQEGVVDYGARFYFYTSKTTVASLINTLND
EPI_ISL_425502    563 HAEETRKLMPVCVETKAIVSTIQRKYKGIKIQEGVVDYGARFYFYTSKTTVASLINTLND
EPI_ISL_425504    563 HAEETRKLMPVCVETKAIVSTIQRKYKGIKIQEGVVDYGARFYFYTSKTTVASLINTLND
EPI_ISL_425523    563 HAEETRKLMPVCVETKAIVSTIQRKYKGIKIQEGVVDYGARFYFYTSKTTVASLINTLND
EPI_ISL_425535    563 HAEETRKLMPVCVETKAIVSTIQRKYKGIKIQEGVVDYGARFYFYTSKTTVASLINTLND
EPI_ISL_425537    563 HAEETRKLMPVCVETKAIVSTIQRKYKGIKIQEGVVDYGARFYFYTSKTTVASLINTLND
EPI_ISL_425549    563 HAEETRKLMPVCVETKAIVSTIQRKYKGIKIQEGVVDYGARFYFYTSKTTVASLINTLND
EPI_ISL_425551    563 HAEETRKLMPVCVETKAIVSTIQRKYKGIKIQEGVVDYGARFYFYTSKTTVASLINTLND
EPI_ISL_425562    563 HAEETRKLMPVCVETKAIVSTIQRKYKGIKIQEGVVDYGARFYFYTSKTTVASLINTLND
EPI_ISL_425576    563 HAEETRKLMPVCVETKAIVSTIQRKYKGIKIQEGVVDYGARFYFYTSKTTVASLINTLND
EPI_ISL_425586    563 HAEETRKLMPVCVETKAIVSTIQRKYKGIKIQEGVVDYGARFYFYTSKTTVASLINTLND
EPI_ISL_425587    563 HAEETRKLMPVCVETKAIVSTIQRKYKGIKIQEGVVDYGARFYFYTSKTTVASLINTLND
EPI_ISL_426817    563 HAEETRKLMPVCVETKAIVSTIQRKYKGIKIQEGVVDYGARFYFYTSKTTVASLINTLND
EPI_ISL_426832    563 HAEETRKLMPVCVETKAIVSTIQRKYKGIKIQEGVVDYGARFYFYTSKTTVASLINTLND

ORF1ab           1441 LNETLVTMPLGYVTHGLNLEEAARYMRSLKVPATVSVSSPDAVTAYNGYLTSSSKTPEEH
EPI_ISL_424224    623 LNETLVTMPLGYVTHGLNLEEAARYMRSLKVPATVSVSSPDAVTAYNGYLTSSSKTPEEH
EPI_ISL_424255    623 LNETLVTMPLGYVTHGLNLEEAARYMRSLKVPATVSVSSPDAVTAYNGYLTSSSKTPEEH
EPI_ISL_425501    623 LNETLVTMPLGYVTHGLNLEEAARYMRSLKVPATVSVSSPDAVTAYNGYLTSSSKTPEEH
EPI_ISL_425502    623 LNETLVTMPLGYVTHGLNLEEAARYMRSLKVPATVSVSSPDAVTAYNGYLTSSSKTPEEH
EPI_ISL_425504    623 LNETLVTMPLGYVTHGLNLEEAARYMRSLKVPATVSVSSPDAVTAYNGYLTSSSKTPEEH
EPI_ISL_425523    623 LNETLVTMPLGYVTHGLNLEEAARYMRSLKVPATVSVSSPDAVTAYNGYLTSSSKTPEEH
EPI_ISL_425535    623 LNETLVTMPLGYVTHGLNLEEAARYMRSLKVPATVSVSSPDAVTAYNGYLTSSSKTPEEH
EPI_ISL_425537    623 LNETLVTMPLGYVTHGLNLEEAARYMRSLKVPATVSVSSPDAVTAYNGYLTSSSKTPEEH
```

```
EPI_ISL_425549  623 LNETLVTMPLGYVTHGLNLEEAARYMRSLKVPATVSVSSPDAVTAYNGYLTSSSKTPEEH
EPI_ISL_425551  623 LNETLVTMPLGYVTHGLNLEEAARYMRSLKVPATVSVSSPDAVTAYNGYLTSSSKTPEEH
EPI_ISL_425562  623 LNETLVTMPLGYVTHGLNLEEAARYMRSLKVPATVSVSSPDAVTAYNGYLTSSSKTPEEH
EPI_ISL_425576  623 LNETLVTMPLGYVTHGLNLEEAARYMRSLKVPATVSVSSPDAVTAYNGYLTSSSKTPEEH
EPI_ISL_425586  623 LNETLVTMPLGYVTHGLNLEEAARYMRSLKVPATVSVSSPDAVTAYNGYLTSSSKTPEEH
EPI_ISL_425587  623 LNETLVTMPLGYVTHGLNLEEAARYMRSLKVPATVSVSSPDAVTAYNGYLTSSSKTPEEH
EPI_ISL_426817  623 LNETLVTMPLGYVTHGLNLEEAARYMRSLKVPATVSVSSPDAVTAYNGYLTSSSKTPEEH
EPI_ISL_426832  623 LNETLVTMPLGYVTHGLNLEEAARYMRSLKVPATVSVSSPDAVTAYNGYLTSSSKTPEEH
```

```
ORF1ab          1501 FIETISLAGSYKDWSYSGQSTQLGIEFLKRGDKSVYYTSNPTTFHLDGEVITFDNLKTLL
EPI_ISL_424224   683 FIETISLAGSYKDWSYSGQSTQLGIEFLKRGDKSVYYTSNPTTFHLDGEVITFDNLKTLL
EPI_ISL_424255   683 FIETISLAGSYKDWSYSGQSTQLGIEFLKRGDKSVYYTSNPTTFHLDGEVITFDNLKTLL
EPI_ISL_425501   683 FIETISLAGSYKDWSYSGQSTQLGIEFLKRGDKSVYYTSNPTTFHLDGEVITFDNLKTLL
EPI_ISL_425502   683 FIETISLAGSYKDWSYSGQSTQLGIEFLKRGDKSVYYTSNPTTFHLDGEVITFDNLKTLL
EPI_ISL_425504   683 FIETISLAGSYKDWSYSGQSTQLGIEFLKRGDKSVYYTSNPTTFHLDGEVITFDNLKTLL
EPI_ISL_425523   683 FIETISLAGSYKDWSYSGQSTQLGIEFLKRGDKSVYYTSNPTTFHLDGEVITFDNLKTLL
EPI_ISL_425535   683 FIETISLAGSYKDWSYSGQSTQLGIEFLKRGDKSVYYTSNPTTFHLDGEVITFDNLKTLL
EPI_ISL_425537   683 FIETISLAGSYKDWSYSGQSTQLGIEFLKRGDKSVYYTSNPTTFHLDGEVITFDNLKTLL
EPI_ISL_425549   683 FIETISLAGSYKDWSYSGQSTQLGIEFLKRGDKSVYYTSNPTTFHLDGEVITFDNLKTLL
EPI_ISL_425551   683 FIETISLAGSYKDWSYSGQSTQLGIEFLKRGDKSVYYTSNPTTFHLDGEVITFDNLKTLL
EPI_ISL_425562   683 FIETISLAGSYKDWSYSGQSTQLGIEFLKRGDKSVYYTSNPTTFHLDGEVITFDNLKTLL
EPI_ISL_425576   683 FIETISLAGSYKDWSYSGQSTQLGIEFLKRGDKSVYYTSNPTTFHLDGEVITFDNLKTLL
EPI_ISL_425586   683 FIETISLAGSYKDWSYSGQSTQLGIEFLKRGDKSVYYTSNPTTFHLDGEVITFDNLKTLL
EPI_ISL_425587   683 FIETISLAGSYKDWSYSGQSTQLGIEFLKRGDKSVYYTSNPTTFHLDGEVITFDNLKTLL
EPI_ISL_426817   683 FIETISLAGSYKDWSYSGQSTQLGIEFLKRGDKSVYYTSNPTTFHLDGEVITFDNLKTLL
EPI_ISL_426832   683 FIETISLAGSYKDWSYSGQSTQLGIEFLKRGDKSVYYTSNPTTFHLDGEVITFDNLKTLL
```

```
ORF1ab          1561 SLREVRTIKVFTTVDNINLHTQVVDMSMTYGQQFGPTYLDGADVTKIKPHNSHEGKTFYV
EPI_ISL_424224   743 SLREVRTIKVFTTVDNINLHTQVVDMSMTYGQQFGPTYLDGADVTKIKPHNSHEGKTFYV
EPI_ISL_424255   743 SLREVRTIKVFTTVDNINLHTQVVDMSMTYGQQFGPTYLDGADVTKIKPHNSHEGKTFYV
EPI_ISL_425501   743 SLREVRTIKVFTTVDNINLHTQVVDMSMTYGQQFGPTYLDGADVTKIKPHNSHEGKTFYV
EPI_ISL_425502   743 SLREVRTIKVFTTVDNINLHTQVVDMSMTYGQQFGPTYLDGADVTKIKPHNSHEGKTFYV
EPI_ISL_425504   743 SLREVRTIKVFTTVDNINLHTQVVDMSMTYGQQFGPTYLDGADVTKIKPHNSHEGKTFYV
EPI_ISL_425523   743 SLREVRTIKVFTTVDNINLHTQVVDMSMTYGQQFGPTYLDGADVTKIKPHNSHEGKTFYV
EPI_ISL_425535   743 SLREVRTIKVFTTVDNINLHTQVVDMSMTYGQQFGPTYLDGADVTKIKPHNSHEGKTFYV
EPI_ISL_425537   743 SLREVRTIKVFTTVDNINLHTQVVDMSMTYGQQFGPTYLDGADVTKIKPHNSHEGKTFYV
EPI_ISL_425549   743 SLREVRTIKVFTTVDNINLHTQVVDMSMTYGQQFGPTYLDGADVTKIKPHNSHEGKTFYV
EPI_ISL_425551   743 SLREVRTIKVFTTVDNINLHTQVVDMSMTYGQQFGPTYLDGADVTKIKPHNSHEGKTFYV
EPI_ISL_425562   743 SLREVRTIKVFTTVDNINLHTQVVDMSMTYGQQFGPTYLDGADVTKIKPHNSHEGKTFYV
EPI_ISL_425576   743 SLREVRTIKVFTTVDNINLHTQVVDMSMTYGQQFGPTYLDGADVTKIKPHNSHEGKTFYV
EPI_ISL_425586   743 SLREVRTIKVFTTVDNINLHTQVVDMSMTYGQQFGPTYLDGADVTKIKPHNSHEGKTFYV
EPI_ISL_425587   743 SLREVRTIKVFTTVDNINLHTQVVDMSMTYGQQFGPTYLDGADVTKIKPHNSHEGKTFYV
EPI_ISL_426817   743 SLREVRTIKVFTTVDNINLHTQVVDMSMTYGQQFGPTYLDGADVTKIKPHNSHEGKTFYV
EPI_ISL_426832   743 SLREVRTIKVFTTVDNINLHTQVVDMSMTYGQQFGPTYLDGADVTKIKPHNSHEGKTFYV
```

```
ORF1ab          1621 LPNDDTLRVEAFEYYHTTDPSFLGRYMSALNHTKKWKYPQVNGLTSIKWADNNCYLATAL
EPI_ISL_424224   803 LPNDDTLRVEAFEYYHTTDPSFLGRYMSALNHTKKWKYPQVNGLTSIKWADNNCYLATAL
EPI_ISL_424255   803 LPNDDTLRVEAFEYYHTTDPSFLGRYMSALNHTKKWKYPQVNGLTSIKWADNNCYLATAL
EPI_ISL_425501   803 LPNDDTLRVEAFEYYHTTDPSFLGRYMSALNHTKKWKYPQVNGLTSIKWADNNCYLATAL
EPI_ISL_425502   803 LPNDDTLRVEAFEYYHTTDPSFLGRYMSALNHTKKWKYPQVNGLTSIKWADNNCYLATAL
EPI_ISL_425504   803 LPNDDTLRVEAFEYYHTTDPSFLGRYMSALNHTKKWKYPQVNGLTSIKWADNNCYLATAL
EPI_ISL_425523   803 LPNDDTLRVEAFEYYHTTDPSFLGRYMSALNHTKKWKYPQVNGLTSIKWADNNCYLATAL
EPI_ISL_425535   803 LPNDDTLRVEAFEYYHTTDPSFLGRYMSALNHTKKWKYPQVNGLTSIKWADNNCYLATAL
EPI_ISL_425537   803 LPNDDTLRVEAFEYYHTTDPSFLGRYMSALNHTKKWKYPQVNGLTSIKWADNNCYLATAL
EPI_ISL_425549   803 LPNDDTLRVEAFEYYHTTDPSFLGRYMSALNHTKKWKYPQVNGLTSIKWADNNCYLATAL
EPI_ISL_425551   803 LPNDDTLRVEAFEYYHTTDPSFLGRYMSALNHTKKWKYPQVNGLTSIKWADNNCYLATAL
EPI_ISL_425562   803 LPNDDTLRVEAFEYYHTTDPSFLGRYMSALNHTKKWKYPQVNGLTSIKWADNNCYLATAL
EPI_ISL_425576   803 LPNDDTLRVEAFEYYHTTDPSFLGRYMSALNHTKKWKYPQVNGLTSIKWADNNCYLATAL
EPI_ISL_425586   803 LPNDDTLRVEAFEYYHTTDPSFLGRYMSALNHTKKWKYPQVNGLTSIKWADNNCYLATAL
EPI_ISL_425587   803 LPNDDTLRVEAFEYYHTTDPSFLGRYMSALNHTKKWKYPQVNGLTSIKWADNNCYLATAL
EPI_ISL_426817   803 LPNDDTLRVEAFEYYHTTDPSFLGRYMSALNHTKKWKYPQVNGLTSIKWADNNCYLATAL
EPI_ISL_426832   803 LPNDDTLRVEAFEYYHTTDPSFLGRYMSALNHTKKWKYPQVNGLTSIKWADNNCYLATAL
```

```
ORF1ab         1681 LTLQQIELKFNPPALQDAYYRARAGEAANFCALILAYCNKTVGELGDVRETMSYLFQHAN
EPI_ISL_424224  863 LTLQQIELKFNPPALQDAYYRARAGEAANFCALILAYCNKTVGELGDVRETMSYLFQHAN
EPI_ISL_424255  863 LTLQQIELKFNPPALQDAYYRARAGEAANFCALILAYCNKTVGELGDVRETMSYLFQHAN
EPI_ISL_425501  863 LTLQQIELKFNPPALQDAYYRARAGEAANFCALILAYCNKTVGELGDVRETMSYLFQHAN
EPI_ISL_425502  863 LTLQQIELKFNPPALQDAYYRARAGEAANFCALILAYCNKTVGELGDVRETMSYLFQHAN
EPI_ISL_425504  863 LTLQQIELKFNPPALQDAYYRARAGEAANFCALILAYCNKTVGELGDVRETMSYLFQHAN
EPI_ISL_425523  863 LTLQQIELKFNPPALQDAYYRARAGEAANFCALILAYCNKTVGELGDVRETMSYLFQHAN
EPI_ISL_425535  863 LTLQQIELKFNPPALQDAYYRARAGEAANFCALILAYCNKTVGELGDVRETMSYLFQHAN
EPI_ISL_425537  863 LTLQQIELKFNPPALQDAYYRARAGEAANFCALILAYCNKTVGELGDVRETMSYLFQHAN
EPI_ISL_425549  863 LTLQQIELKFNPPALQDAYYRARAGEAANFCALILAYCNKTVGELGDVRETMSYLFQHAN
EPI_ISL_425551  863 LTLQQIELKFNPPALQDAYYRARAGEAANFCALILAYCNKTVGELGDVRETMSYLFQHAN
EPI_ISL_425562  863 LTLQQIELKFNPPALQDAYYRARAGEAANFCALILAYCNKTVGELGDVRETMSYLFQHAN
EPI_ISL_425576  863 LTLQQIELKFNPPALQDAYYRARAGEAANFCALILAYCNKTVGELGDVRETMSYLFQHAN
EPI_ISL_425586  863 LTLQQIELKFNPPALQDAYYRARAGEAANFCALILAYCNKTVGELGDVRETMSYLFQHAN
EPI_ISL_425587  863 LTLQQIELKFNPPALQDAYYRARAGEAANFCALILAYCNKTVGELGDVRETMSYLFQHAN
EPI_ISL_426817  863 LTLQQIELKFNPPALQDAYYRARAGEAANFCALILAYCNKTVGELGDVRETMSYLFQHAN
EPI_ISL_426832  863 LTLQQIELKFNPPALQDAYYRARAGEAANFCALILAYCNKTVGELGDVRETMSYLFQHAN

ORF1ab         1741 LDSCKRVLNVVCKTCGQQQTTLKGVEAVMYMGTLSYEQFKKGVQIPCTCGKQATKYLVQQ
EPI_ISL_424224  923 LDSCKRVLNVVCKTCGQQQTTLKGVEAVMYMGTLSYEQFKKGVQIPCTCGKQATKYLVQQ
EPI_ISL_424255  923 LDSCKRVLNVVCKTCGQQQTTLKGVEAVMYMGTLSYEQFKKGVQIPCTCGKQATKYLVQQ
EPI_ISL_425501  923 LDSCKRVLNVVCKTCGQQQTTLKGVEAVMYMGTLSYEQFKKGVQIPCTCGKQATKYLVQQ
EPI_ISL_425502  923 LDSCKRVLNVVCKTCGQQQTTLKGVEAVMYMGTLSYEQFKKGVQIPCTCGKQATKYLVQQ
EPI_ISL_425504  923 LDSCKRVLNVVCKTCGQQQTTLKGVEAVMYMGTLSYEQFKKGVQIPCTCGKQATKYLVQQ
EPI_ISL_425523  923 LDSCKRVLNVVCKTCGQQQTTLKGVEAVMYMGTLSYEQFKKGVQIPCTCGKQATKYLVQQ
EPI_ISL_425535  923 LDSCKRVLNVVCKTCGQQQTTLKGVEAVMYMGTLSYEQFKKGVQIPCTCGKQATKYLVQQ
EPI_ISL_425537  923 LDSCKRVLNVVCKTCGQQQTTLKGVEAVMYMGTLSYEQFKKGVQIPCTCGKQATKYLVQQ
EPI_ISL_425549  923 LDSCKRVLNVVCKTCGQQQTTLKGVEAVMYMGTLSYEQFKKGVQIPCTCGKQATKYLVQQ
EPI_ISL_425551  923 LDSCKRVLNVVCKTCGQQQTTLKGVEAVMYMGTLSYEQFKKGVQIPCTCGKQATKYLVQQ
EPI_ISL_425562  923 LDSCKRVLNVVCKTCGQQQTTLKGVEAVMYMGTLSYEQFKKGVQIPCTCGKQATKYLVQQ
EPI_ISL_425576  923 LDSCKRVLNVVCKTCGQQQTTLKGVEAVMYMGTLSYEQFKKGVQIPCTCGKQATKYLVQQ
EPI_ISL_425586  923 LDSCKRVLNVVCKTCGQQQTTLKGVEAVMYMGTLSYEQFKKGVQIPCTCGKQATKYLVQQ
EPI_ISL_425587  923 LDSCKRVLNVVCKTCGQQQTTLKGVEAVMYMGTLSYEQFKKGVQIPCTCGKQATKYLVQQ
EPI_ISL_426817  923 LDSCKRVLNVVCKTCGQQQTTLKGVEAVMYMGTLSYEQFKKGVQIPCTCGKQATKYLVQQ
EPI_ISL_426832  923 LDSCKRVLNVVCKTCGQQQTTLKGVEAVMYMGTLSYEQFKKGVQIPCTCGKQATKYLVQQ

ORF1ab         1801 ESPFVMMSAPPAQYELKHGTFTCASEYTGNYQCGHYKHITSKETLYCIDGALLTKSSEYK
EPI_ISL_424224  983 ESPFVMMSAPPAQYELKHGTFTCASEYTGNYQCGHYKHITSKETLYCIDGALLTKSSEYK
EPI_ISL_424255  983 ESPFVMMSAPPAQYELKHGTFTCASEYTGNYQCGHYKHITSKETLYCIDGALLTKSSEYK
EPI_ISL_425501  983 ESPFVMMSAPPAQYELKHGTFTCASEYTGNYQCGHYKHITSKETLYCIDGALLTKSSEYK
EPI_ISL_425502  983 ESPFVMMSAPPAQYELKHGTFTCASEYTGNYQCGHYKHITSKETLYCIDGALLTKSSEYK
EPI_ISL_425504  983 ESPFVMMSAPPAQYELKHGTFTCASEYTGNYQCGHYKHITSKETLYCIDGALLTKSSEYK
EPI_ISL_425523  983 ESPFVMMSAPPAQYELKHGTFTCASEYTGNYQCGHYKHITSKETLYCIDGALLTKSSEYK
EPI_ISL_425535  983 ESPFVMMSAPPAQYELKHGTFTCASEYTGNYQCGHYKHITSKETLYCIDGALLTKSSEYK
EPI_ISL_425537  983 ESPFVMMSAPPAQYELKHGTFTCASEYTGNYQCGHYKHITSKETLYCIDGALLTKSSEYK
EPI_ISL_425549  983 ESPFVMMSAPPAQYELKHGTFTCASEYTGNYQCGHYKHITSKETLYCIDGALLTKSSEYK
EPI_ISL_425551  983 ESPFVMMSAPPAQYELKHGTFTCASEYTGNYQCGHYKHITSKETLYCIDGALLTKSSEYK
EPI_ISL_425562  983 ESPFVMMSAPPAQYELKHGTFTCASEYTGNYQCGHYKHITSKETLYCIDGALLTKSSEYK
EPI_ISL_425576  983 ESPFVMMSAPPAQYELKHGTFTCASEYTGNYQCGHYKHITSKETLYCIDGALLTKSSEYK
EPI_ISL_425586  983 ESPFVMMSAPPAQYELKHGTFTCASEYTGNYQCGHYKHITSKETLYCIDGALLTKSSEYK
EPI_ISL_425587  983 ESPFVMMSAPPAQYELKHGTFTCASEYTGNYQCGHYKHITSKETLYCIDGALLTKSSEYK
EPI_ISL_426817  983 ESPFVMMSAPPAQYELKHGTFTCASEYTGNYQCGHYKHITSKETLYCIDGALLTKSSEYK
EPI_ISL_426832  983 ESPFVMMSAPPAQYELKHGTFTCASEYTGNYQCGHYKHITSKETLYCIDGALLTKSSEYK

ORF1ab         1861 GPITDVFYKENSYTTTIKPVTYKLDGVVCTEIDPKLDNYYKKDNSYFTEQPIDLVPNQPY
EPI_ISL_424224 1043 GPITDVFYKENSYTTTIKPVTYKLDGVVCTEIDPKLDNYYKKDNSYFTEQPIDLVPNQPY
EPI_ISL_424255 1043 GPITDVFYKENSYTTTIKPVTYKLDGVVCTEIDPKLDNYYKKDNSYFTEQPIDLVPNQPY
EPI_ISL_425501 1043 GPITDVFYKENSYTTTIKPVTYKLDGVVCTEIDPKLDNYYKKDNSYFTEQPIDLVPNQPY
EPI_ISL_425502 1043 GPITDVFYKENSYTTTIKPVTYKLDGVVCTEIDPKLDNYYKKDNSYFTEQPIDLVPNQPY
EPI_ISL_425504 1043 GPITDVFYKENSYTTTIKPVTYKLDGVVCTEIDPKLDNYYKKDNSYFTEQPIDLVPNQPY
EPI_ISL_425523 1043 GPITDVFYKENSYTTTIKPVTYKLDGVVCTEIDPKLDNYYKKDNSYFTEQPIDLVPNQPY
EPI_ISL_425535 1043 GPITDVFYKENSYTTTIKPVTYKLDGVVCTEIDPKLDNYYKKDNSYFTEQPIDLVPNQPY
EPI_ISL_425537 1043 GPITDVFYKENSYTTTIKPVTYKLDGVVCTEIDPKLDNYYKKDNSYFTEQPIDLVPNQPY
```

EPI_ISL_425549	1043	GPITDVFYKENSYTTTIKPVTYKLDGVVCTEIDPKLDNYYKKDNSYFTEQPIDLVPNQPY
EPI_ISL_425551	1043	GPITDVFYKENSYTTTIKPVTYKLDGVVCTEIDPKLDNYYKKDNSYFTEQPIDLVPNQPY
EPI_ISL_425562	1043	GPITDVFYKENSYTTTIKPVTYKLDGVVCTEIDPKLDNYYKKDNSYFTEQPIDLVPNQPY
EPI_ISL_425576	1043	GPITDVFYKENSYTTTIKPVTYKLDGVVCTEIDPKLDNYYKKDNSYFTEQPIDLVPNQPY
EPI_ISL_425586	1043	GPITDVFYKENSYTTTIKPVTYKLDGVVCTEIDPKLDNYYKKDNSYFTEQPIDLVPNQPY
EPI_ISL_425587	1043	GPITDVFYKENSYTTTIKPVTYKLDGVVCTEIDPKLDNYYKKDNSYFTEQPIDLVPNQPY
EPI_ISL_426817	1043	GPITDVFYKENSYTTTIKPVTYKLDGVVCTEIDPKLDNYYKKDNSYFTEQPIDLVPNQPY
EPI_ISL_426832	1043	GPITDVFYKENSYTTTIKPVTYKLDGVVCTEIDPKLDNYYKKDNSYFTEQPIDLVPNQPY

```
ORF1ab         1921 PNASFDNFKFVCDNIKFADDLNQLTGYKKPASRELKVTFFPDLNGDVVAIDYKHYTPSFK
EPI_ISL_424224 1103 PNASFDNFKFVCDNIKFADDLNQLTGYKKPASRELKVTFFPDLNGDVVAIDYKHYTPSFK
EPI_ISL_424255 1103 PNASFDNFKFVCDNIKFADDLNQLTGYKKPASRELKVTFFPDLNGDVVAIDYKHYTPSFK
EPI_ISL_425501 1103 PNASFDNFKFVCDNIKFADDLNQLTGYKKPASRELKVTFFPDLNGDVVAIDYKHYTPSFK
EPI_ISL_425502 1103 PNASFDNFKFVCDNIKFADDLNQLTGYKKPASRELKVTFFPDLNGDVVAIDYKHYTPSFK
EPI_ISL_425504 1103 LNASFDNFKFVCDNIKFADDLNQLTGYKKPASRELKVTFFPDLNGDVVAIDYKHYTPSFK
EPI_ISL_425523 1103 PNASFDNFKFVCDNIKFADDLNQLTGYKKPASRELKVTFFPDLNGDVVAIDYKHYTPSFK
EPI_ISL_425535 1103 PNASFDNFKFVCDNIKFADDLNQLTGYKKPASRELKVTFFPDLNGDVVAIDYKHYTPSFK
EPI_ISL_425537 1103 PNASFDNFKFVCDNIKFADDLNQLTGYKKPASRELKVTFFPDLNGDVVAIDYKHYTPSFK
EPI_ISL_425549 1103 PNASFDNFKFVCDNIKFADDLNQLTGYKKPASRELKVTFFPDLNGDVVAIDYKHYTPSFK
EPI_ISL_425551 1103 PNASFDNFKFVCDNIKFADDLNQLTGYKKPASRELKVTFFPDLNGDVVAIDYKHYTPSFK
EPI_ISL_425562 1103 PNASFDNFKFVCDNIKFADDLNQLTGYKKPASRELKVTFFPDLNGDVVAIDYKHYTPSFK
EPI_ISL_425576 1103 PNASFDNFKFVCDNIKFADDLNQLTGYKKPASRELKVTFFPDLNGDVVAIDYKHYTPSFK
EPI_ISL_425586 1103 PNASFDNFKFVCDNIKFADDLNQLTGYKKPASRELKVTFFPDLNGDVVAIDYKHYTPSFK
EPI_ISL_425587 1103 PNASFDNFKFVCDNIKFADDLNQLTGYKKPASRELKVTFFPDLNGDVVAIDYKHYTPSFK
EPI_ISL_426817 1103 PNASFDNFKFVCDNIKFADDLNQLTGYKKPASRELKVTFFPDLNGDVVAIDYKHYTPSFK
EPI_ISL_426832 1103 PNASFDNFKFVCDNIKFADDLNQLTGYKKPASRELKVTFFPDLNGDVVAIDYKHYTPSFK

ORF1ab         1981 KGAKLLHKPIVWHVNNATNKATYKPNTWCIRCLWSTKPVETSNSFDVLKSEDAQGMDNLA
EPI_ISL_424224 1163 KGAKLLHKPIVWHVNNATNKATYKPNTWCIRCLWSTKPVETSNSFDVLKSEDAQGMDNLA
EPI_ISL_424255 1163 KGAKLLHKPIVWHVNNATNKATYKPNTWCIRCLWSTKPVETSNSFDVLKSEDAQGMDNLA
EPI_ISL_425501 1163 KGAKLLHKPIVWHVNNATNKATYKPNTWCIRCLWSTKPVETSNSFDVLKSEDAQGMDNLA
EPI_ISL_425502 1163 KGAKLLHKPIVWHVNNATNKATYKPNTWCIRCLWSTKPVETSNSFDVLKSEDAQGMDNLA
EPI_ISL_425504 1163 KGAKLLHKPIVWHVNNATNKATYKPNTWCIRCLWSTKPVETSNSFDVLKSEDAQGMDNLA
EPI_ISL_425523 1163 KGAKLLHKPIVWHVNNATNKATYKPNTWCIRCLWSTKPVETSNSFDVLKSEDAQGMDNLA
EPI_ISL_425535 1163 KGAKLLHKPIVWHVNNATNKATYKPNTWCIRCLWSTKPVETSNSFDVLKSEDAQGMDNLA
EPI_ISL_425537 1163 KGAKLLHKPIVWHVNNATNKATYKPNTWCIRCLWSTKPVETSNSFDVLKSEDAQGMDNLA
EPI_ISL_425549 1163 KGAKLLHKPIVWHVNNATNKATYKPNTWCIRCLWSTKPVETSNSFDVLKSEDAQGMDNLA
EPI_ISL_425551 1163 KGAKLLHKPIVWHVNNATNKATYKPNTWCIRCLWSTKPVETSNSFDVLKSEDAQGMDNLA
EPI_ISL_425562 1163 KGAKLLHKPIVWHVNNATNKATYKPNTWCIRCLWSTKPVETSNSFDVLKSEDAQGMDNLA
EPI_ISL_425576 1163 KGAKLLHKPIVWHVNNATNKATYKPNTWCIRCLWSTKPVETSNSFDVLKSEDAQGMDNLA
EPI_ISL_425586 1163 KGAKLLHKPIVWHVNNATNKATYKPNTWCIRCLWSTKPVETSNSFDVLKSEDAQGMDNLA
EPI_ISL_425587 1163 KGAKLLHKPIVWHVNNATNKATYKPNTWCIRCLWSTKPVETSNSFDVLKSEDAQGMDNLA
EPI_ISL_426817 1163 KGAKLLHKPIVWHVNNATNKATYKPNTWCIRCLWSTKPVETSNSFDVLKSEDAQGMDNLA
EPI_ISL_426832 1163 KGAKLLHKPIVWHVNNATNKATYKPNTWCIRCLWSTKPVETSNSFDVLKSEDAQGMDNLA

ORF1ab         2041 CEDLKPVSEEVVENPTIQKDVLECNVKTTEVVGDIILKPANNSLKITEEVGHTDLMAAYV
EPI_ISL_424224 1223 CEDLKPVSEEVVENPTIQKDVLECNVKTTEVVGDIILKPANNSLKITEEVGHTDLMAAYV
EPI_ISL_424255 1223 CEDLKPVSEEVVENPTIQKDVLECNVKTTEVVGDIILKPANNSLKITEEVGHTDLMAAYV
EPI_ISL_425501 1223 CEDLKPVSEEVVENPTIQKDVLECNVKTTEVVGDIILKPANNSLKITEEVGHTDLMAAYV
EPI_ISL_425502 1223 CEDLKPVSEEVVENPTIQKDVLECNVKTTEVVGDIILKPANNSLKITEEVGHTDLMAAYV
EPI_ISL_425504 1223 CEDLKPVSEEVVENPTIQKDVLECNVKTTEVVGDIILKPANNSLKITEEVGHTDLMAAYV
EPI_ISL_425523 1223 CEDLKPVSEEVVENPTIQKDVLECNVKTTEVVGDIILKPANNSLKITEEVGHTDLMAAYV
EPI_ISL_425535 1223 CEDLKPVSEEVVENPTIQKDVLECNVKTTEVVGDIILKPANNSLKITEEVGHTDLMAAYV
EPI_ISL_425537 1223 CEDLKPVSEEVVENPTIQKDVLECNVKTTEVVGDIILKPANNSLKITEEVGHTDLMAAYV
EPI_ISL_425549 1223 CEDLKPVSEEVVENPTIQKDVLECNVKTTEVVGDIILKPANNSLKITEEVGHTDLMAAYV
EPI_ISL_425551 1223 CEDLKPVSEEVVENPTIQKDVLECNVKTTEVVGDIILKPANNSLKITEEVGHTDLMAAYV
EPI_ISL_425562 1223 CEDLKPVSEEVVENPTIQKDVLECNVKTTEVVGDIILKPANNSLKITEEVGHTDLMAAYV
EPI_ISL_425576 1223 CEDLKPVSEEVVENPTIQKDVLECNVKTTEVVGDIILKPANNSLKITEEVGHTDLMAAYV
EPI_ISL_425586 1223 CEDLKPVSEEVVENPTIQKDVLECNVKTTEVVGDIILKPANNSLKITEEVGHTDLMAAYV
EPI_ISL_425587 1223 CEDLKPVSEEVVENPTIQKDVLECNVKTTEVVGDIILKPANNSLKITEEVGHTDLMAAYV
EPI_ISL_426817 1223 CEDLKPVSEEVVENPTIQKDVLECNVKTTEVVGDIILKPANNSLKITEEVGHTDLMAAYV
EPI_ISL_426832 1223 CEDLKPVSEEVVENPTIQKDVLECNVKTTEVVGDIILKPANNSLKITEEVGHTDLMAAYV
```

```
ORF1ab         2101 DNSSLTIKKPNELSRVLGLKTLATHGLAAVNSVPWDTIANYAKPFLNKVVSTTTNIVTRC
EPI_ISL_424224 1283 DNSSLTIKKPNELSRVLGLKTLATHGLAAVNSVPWDTIANYAKPFLNKVVSTTTNIVTRC
EPI_ISL_424255 1283 DNSSLTIKKPNELSRVLGLKTLATHGLAAVNSVPWDTIANYAKPFLNKVVSTTTNIVTRC
EPI_ISL_425501 1283 DNSSLTIKKPNELSRVLGLKTLATHGLAAVNSVPWDTIANYAKPFLNKVVSTTTNIVTRC
EPI_ISL_425502 1283 DNSSLTIKKPNELSRVLGLKTLATHGLAAVNSVPWDTIANYAKPFLNKVVSTTTNIVTRC
EPI_ISL_425504 1283 DNSSLTIKKPNELSRVLGLKTLATHGLAAVNSVPWDTIANYAKPFLNKVVSTTTNIVTRC
EPI_ISL_425523 1283 DNSSLTIKKPNELSRVLGLKTLATHGLAAVNSVPWDTIANYAKPFLNKVVSTTTNIVTRC
EPI_ISL_425535 1283 DNSSLTIKKPNELSRVLGLKTLATHGLAAVNSVPWDTIANYAKPFLNKVVSTTTNIVTRC
EPI_ISL_425537 1283 DNSSLTIKKPNELSRVLGLKTLATHGLAAVNSVPWDTIANYAKPFLNKVVSTTTNIVTRC
EPI_ISL_425549 1283 DNSSLTIKKPNELSRVLGLKTLATHGLAAVNSVPWDTIANYAKPFLNKVVSTTTNIVTRC
EPI_ISL_425551 1283 DNSSLTIKKPNELSRVLGLKTLATHGLAAVNSVPWDTIANYAKPFLNKVVSTTTNIVTRC
EPI_ISL_425562 1283 DNSSLTIKKPNELSRVLGLKTLATHGLAAVNSVPWDTIANYAKPFLNKVVSTTTNIVTRC
EPI_ISL_425576 1283 DNSSLTIKKPNELSRVLGLKTLATHGLAAVNSVPWDTIANYAKPFLNKVVSTTTNIVTRC
EPI_ISL_425586 1283 DNSSLTIKKPNELSRVLGLKTLATHGLAAVNSVPWDTIANYAKPFLNKVVSTTTNIVTRC
EPI_ISL_425587 1283 DNSSLTIKKPNELSRVLGLKTLATHGLAAVNSVPWDTIANYAKPFLNKVVSTTTNIVTRC
EPI_ISL_426817 1283 DNSSLTIKKPNELSRVLGLKTLATHGLAAVNSVPWDTIANYAKPFLNKVVSTTTNIVTRC
EPI_ISL_426832 1283 DNSSLTIKKPNELSRVLGLKTLATHGLAAVNSVPWDTIANYAKPFLNKVVSTTTNIVTRC

ORF1ab         2161 LNRVCTNYMPYFFTLLLQLCTFTRSTNSRIKASMPTTIAKNTVKSVGKFCLEASFNYLKS
EPI_ISL_424224 1343 LNRVCTNYMPYFFTLLLQLCTFTRSTNSRIKASMPTTIAKNTVKSVGKFCLEASFNYLKS
EPI_ISL_424255 1343 LNRVCTNYMPYFFTLLLQLCTFTRSTNSRIKASMPTTIAKNTVKSVGKFCLEASFNYLKS
EPI_ISL_425501 1343 LNRVCTNYMPYFFTLLLQLCTFTRSTNSRIKASMPTTIAKNTVKSVGKFCLEASFNYLKS
EPI_ISL_425502 1343 LNRVCTNYMPYFFTLLLQLCTFTRSTNSRIKASMPTTIAKNTVKSVGKFCLEASFNYLKS
EPI_ISL_425504 1343 LNRVCTNYMPYFFTLLLQLCTFTRSTNSRIKASMPTTIAKNTVKSVGKFCLEASFNYLKS
EPI_ISL_425523 1343 LNRVCTNYMPYFFTLLLQLCTFTRSTNSRIKASMPTTIAKNTVKSVGKFCLEASFNYLKS
EPI_ISL_425535 1343 LNRVCTNYMPYFFTLLLQLCTFTRSTNSRIKASMPTTIAKNTVKSVGKFCLEASFNYLKS
EPI_ISL_425537 1343 LNRVCTNYMPYFFTLLLQLCTFTRSTNSRIKASMPTTIAKNTVKSVGKFCLEASFNYLKS
EPI_ISL_425549 1343 LNRVCTNYMPYFFTLLLQLCTFTRSTNSRIKASMPTTIAKNTVKSVGKFCLEASFNYLKS
EPI_ISL_425551 1343 LNRVCTNYMPYFFTLLLQLCTFTRSTNSRIKASMPTTIAKNTVKSVGKFCLEASFNYLKS
EPI_ISL_425562 1343 LNRVCTNYMPYFFTLLLQLCTFTRSTNSRIKASMPTTIAKNTVKSVGKFCLEASFNYLKS
EPI_ISL_425576 1343 LNRVCTNYMPYFFTLLLQLCTFTRSTNSRIKASMPTTIAKNTVKSVGKFCLEASFNYLKS
EPI_ISL_425586 1343 LNRVCTNYMPYFFTLLLQLCTFTRSTNSRIKASMPTTIAKNTVKSVGKFCLEASFNYLKS
EPI_ISL_425587 1343 LNRVCTNYMPYFFTLLLQLCTFTRSTNSRIKASMPTTIAKNTVKSVGKFCLEASFNYLKS
EPI_ISL_426817 1343 LNRVCTNYMPYFFTLLLQLCTFTRSTNSRIKASMPTTIAKNTVKSVGKFCLEASFNYLKS
EPI_ISL_426832 1343 LNRVCTNYMPYFFTLLLQLCTFTRSTNSRIKASMPTTIAKNTVKSVGKFCLEASFNYLKS

ORF1ab         2221 PNFSKLINIIIWFLLLSVCLGSLIYSTAALGVLMSNLGMPSYCTGYREGYLNSTNVTIAT
EPI_ISL_424224 1403 PNFSKLINIIIWFLLLSVCLGSLIYSTAALGVLMSNLGMPSYCTGYREGYLNSTNVTIAT
EPI_ISL_424255 1403 PNFSKLINIIIWFLLLSVCLGSLIYSTAALGVLMSNLGMPSYCTGYREGYLNSTNVTIAT
EPI_ISL_425501 1403 PNFSKLINIIIWFLLLSVCLGSLIYSTAALGVLMSNLGMPSYCTGYREGYLNSTNVTIAT
EPI_ISL_425502 1403 PNFSKLINIIIWFLLLSVCLGSLIYSTAALGVLMSNLGMPSYCTGYREGYLNSTNVTIAT
EPI_ISL_425504 1403 PNFSKLINIIIWFLLLSVCLGSLIYSTAALGVLMSNLGMPSYCTGYREGYLNSTNVTIAT
EPI_ISL_425523 1403 PNFSKLINIIIWFLLLSVCLGSLIYSTAALGVLMSNLGMPSYCTGYREGYLNSTNVTIAT
EPI_ISL_425535 1403 PNFSKLINIIIWFLLLSVCLGSLIYSTAALGVLMSNLGMPSYCTGYREGYLNSTNVTIAT
EPI_ISL_425537 1403 PNFSKLINIIIWFLLLSVCLGSLIYSTAALGVLMSNLGMPSYCTGYREGYLNSTNVTIAT
EPI_ISL_425549 1403 PNFSKLINIIIWFLLLSVCLGSLIYSTAALGVLMSNLGMPSYCTGYREGYLNSTNVTIAT
EPI_ISL_425551 1403 PNFSKLINIIIWFLLLSVCLGSLIYSTAALGVLMSNLGMPSYCTGYREGYLNSTNVTIAT
EPI_ISL_425562 1403 PNFSKLINIIIWFLLLSVCLGSLIYSTAALGVLMSNLGMPSYCTGYREGYLNSTNVTIAT
EPI_ISL_425576 1403 PNFSKLINIIIWFLLLSVCLGSLIYSTAALGVLMSNLGMPSYCTGYREGYLNSTNVTIAT
EPI_ISL_425586 1403 PNFSKLINIIIWFLLLSVCLGSLIYSTAALGVLMSNLGMPSYCTGYREGYLNSTNVTIAT
EPI_ISL_425587 1403 PNFSKLINIIIWFLLLSVCLGSLIYSTAALGVLMSNLGMPSYCTGYREGYLNSTNVTIAT
EPI_ISL_426817 1403 PNFSKLINIIIWFLLLSVCLGSLIYSTAALGVLMSNLGMPSYCTGYREGYLNSTNVTIAT
EPI_ISL_426832 1403 PNFSKLINIIIWFLLLSVCLGSLIYSTAALGVLMSNLGMPSYCTGYREGYLNSTNVTIAT

ORF1ab         2281 YCTGSIPCSVCLSGLDSLDTYPSLETIQITISSFKWDLTAFGLVAEWFLAYILFTRFFYV
EPI_ISL_424224 1463 YCTGSIPCSVCLSGLDSLDTYPSLETIQITISSFKWDLTAFGLVAEWFLAYILFTRFFYV
EPI_ISL_424255 1463 YCTGSIPCSVCLSGLDSLDTYPSLETIQITISSFKWDLTAFGLVAEWFLAYILFTRFFYV
EPI_ISL_425501 1463 YCTGSIPCSVCLSGLDSLDTYPSLETIQITISSFKWDLTAFGLVAEWFLAYILFTRFFYV
EPI_ISL_425502 1463 YCTGSIPCSVCLSGLDSLDTYPSLETIQITISSFKWDLTAFGLVAEWFLAYILFTRFFYV
EPI_ISL_425504 1463 YCTGSIPCSVCLSGLDSLDTYPSLETIQITISSFKWDLTAFGLVAEWFLAYILFTRFFYV
EPI_ISL_425523 1463 YCTGSIPCSVCLSGLDSLDTYPSLETIQITISSFKWDLTAFGLVAEWFLAYILFTRFFYV
EPI_ISL_425535 1463 YCTGSIPCSVCLSGLDSLDTYPSLETIQITISSFKWDLTAFGLVAEWFLAYILFTRFFYV
EPI_ISL_425537 1463 YCTGSIPCSVCLSGLDSLDTYPSLETIQITISSFKWDLTAFGLVAEWFLAYILFTRFFYV
```

```
EPI_ISL_425549 1463 YCTGSIPCSVCLSGLDSLDTYPSLETIQITISSFKWDLTAFGLVAEWFLAYILFTRFFYV
EPI_ISL_425551 1463 YCTGSIPCSVCLSGLDSLDTYPSLETIQITISSFKWDLTAFGLVAEWFLAYILFTRFFYV
EPI_ISL_425562 1463 YCTGSIPCSVCLSGLDSLDTYPSLETIQITISSFKWDLTAFGLVAEWFLAYILFTRFFYV
EPI_ISL_425576 1463 YCTGSIPCSVCLSGLDSLDTYPSLETIQITISSFKWDLTAFGLVAEWFLAYILFTRFFYV
EPI_ISL_425586 1463 YCTGSIPCSVCLSGLDSLDTYPSLETIQITISSFKWDLTAFGLVAEWFLAYILFTRFFYV
EPI_ISL_425587 1463 YCTGSIPCSVCLSGLDSLDTYPSLETIQITISSFKWDLTAFGLVAEWFLAYILFTRFFYV
EPI_ISL_426817 1463 YCTGSIPCSVCLSGLDSLDTYPSLETIQITISSFKWDLTAFGLVAEWFLAYILFTRFFYV
EPI_ISL_426832 1463 YCTGSIPCSVCLSGLDSLDTYPSLETIQITISSFKWDLTAFGLVAEWFLAYILFTRFFYV
```

```
ORF1ab         2341 LGLAAIMQLFFSYFAVHFISNSWLMWLIINLVQMAPISAMVRMYIFFASFYYVWKSYVHV
EPI_ISL_424224 1523 LGLAAIMQLFFSYFAVHFISNSWLMWLIINLVQMAPISAMVRMYIFFASFYYVWKSYVHV
EPI_ISL_424255 1523 LGLAAIMQLFFSYFAVHFISNSWLMWLIINLVQMAPISAMVRMYIFFASFYYVWKSYVHV
EPI_ISL_425501 1523 LGLAAIMQLFFSYFAVHFISNSWLMWLIINLVQMAPISAMVRMYIFFASFYYVWKSYVHV
EPI_ISL_425502 1523 LGLAAIMQLFFSYFAVHFISNSWLMWLIINLVQMAPISAMVRMYIFFASFYYVWKSYVHV
EPI_ISL_425504 1523 LGLAAIMQLFFSYFAVHFISNSWLMWLIINLVQMAPISAMVRMYIFFASFYYVWKSYVHV
EPI_ISL_425523 1523 LGLAAIMQLFFSYFAVHFISNSWLMWLIINLVQMAPISAMVRMYIFFASFYYVWKSYVHV
EPI_ISL_425535 1523 LGLAAIMQLFFSYFAVHFISNSWLMWLIINLVQMAPISAMVRMYIFFASFYYVWKSYVHV
EPI_ISL_425537 1523 LGLAAIMQLFFSYFAVHFISNSWLMWLIINLVQMAPISAMVRMYIFFASFYYVWKSYVHV
EPI_ISL_425549 1523 LGLAAIMQLFFSYFAVHFISNSWLMWLIINLVQMAPISAMVRMYIFFASFYYVWKSYVHV
EPI_ISL_425551 1523 LGLAAIMQLFFSYFAVHFISNSWLMWLIINLVQMAPISAMVRMYIFFASFYYVWKSYVHV
EPI_ISL_425562 1523 LGLAAIMQLFFSYFAVHFISNSWLMWLIINLVQMAPISAMVRMYIFFASFYYVWKSYVHV
EPI_ISL_425576 1523 LGLAAIMQLFFSYFAVHFISNSWLMWLIINLVQMAPISAMVRMYIFFASFYYVWKSYVHV
EPI_ISL_425586 1523 LGLAAIMQLFFSYFAVHFISNSWLMWLIINLVQMAPISAMVRMYIFFASFYYVWKSYVHV
EPI_ISL_425587 1523 LGLAAIMQLFFSYFAVHFISNSWLMWLIINLVQMAPISAMVRMYIFFASFYYVWKSYVHV
EPI_ISL_426817 1523 LGLAAIMQLFFSYFAVHFISNSWLMWLIINLVQMAPISAMVRMYIFFASFYYVWKSYVHV
EPI_ISL_426832 1523 LGLAAIMQLFFSYFAVHFISNSWLMWLIINLVQMAPISAMVRMYIFFASFYYVWKSYVHV

ORF1ab         2401 VDGCNSSTCMMCYKRNRATRVECTTIVNGVRRSFYVYANGGKGFCKLHNWNCVNCDTFCA
EPI_ISL_424224 1583 VDGCNSSTCMMCYKRNRATRVECTTIVNGVRRSFYVYANGGKGFCKLHNWNCVNCDTFCA
EPI_ISL_424255 1583 VDGCNSSTCMMCYKRNRATRVECTTIVNGVRRSFYVYANGGKGFCKLHNWNCVNCDTFCA
EPI_ISL_425501 1583 VDGCNSSTCMMCYKRNRATRVECTTIVNGVRRSFYVYANGGKGFCKLHNWNCVNCDTFCA
EPI_ISL_425502 1583 VDGCSSSTCMMCYKRNRATRVECTTIVNGVRRSFYVYANGGKGFCKLHNWNCVNCDTFCA
EPI_ISL_425504 1583 VDGCNSSTCMMCYKRNRATRVECTTIVNGVRRSFYVYANGGKGFCKLHNWNCVNCDTFCA
EPI_ISL_425523 1583 VDGCNSSTCMMCYKRNRATRVECTTIVNGVRRSFYVYANGGKGFCKLHNWNCVNCDTFCA
EPI_ISL_425535 1583 VDGCNSSTCMMCYKRNRATRVECTTIVNGVRRSFYVYANGGKGFCKLHNWNCVNCDTFCA
EPI_ISL_425537 1583 VDGCNSSTCMMCYKRNRATRVECTTIVNGVRRSFYVYANGGKGFCKLHNWNCVNCDTFCA
EPI_ISL_425549 1583 VDGCNSSTCMMCYKRNRATRVECTTIVNGVRRSFYVYANGGKGFCKLHNWNCVNCDTFCA
EPI_ISL_425551 1583 VDGCNSSTCMMCYKRNRATRVECTTIVNGVRRSFYVYANGGKGFCKLHNWNCVNCDTFCA
EPI_ISL_425562 1583 VDGCNSSTCMMCYKRNRATRVECTTIVNGVRRSFYVYANGGKGFCKLHNWNCVNCDTFCA
EPI_ISL_425576 1583 VDGCNSSTCMMCYKRNRATRVECTTIVNGVRRSFYVYANGGKGFCKLHNWNCVNCDTFCA
EPI_ISL_425586 1583 VDGCNSSTCMMCYKRNRATRVECTTIVNGVRRSFYVYANGGKGFCKLHNWNCVNCDTFCA
EPI_ISL_425587 1583 VDGCNSSTCMMCYKRNRATRVECTTIVNGVRRSFYVYANGGKGFCKLHNWNCVNCDTFCA
EPI_ISL_426817 1583 VDGCNSSTCMMCYKRNRATRVECTTIVNGVRRSFYVYANGGKGFCKLHNWNCVNCDTFCA
EPI_ISL_426832 1583 VDGCNSSTCMMCYKRNRATRVECTTIVNGVRRSFYVYANGGKGFCKLHNWNCVNCDTFCA

ORF1ab         2461 GSTFISDEVARDLSLQFKRPINPTDQSSYIVDSVTVKNGSIHLYFDKAGQKTYERHSLSH
EPI_ISL_424224 1643 GSTFISDEVARDLSLQFKRPINPTDQSSYIVDSVTVKNGSIHLYFDKAGQKTYERHSLSH
EPI_ISL_424255 1643 GSTFISDEVARDLSLQFKRPINPTDQSSYIVDSVTVKNGSIHLYFDKAGQKTYERHSLSH
EPI_ISL_425501 1643 GSTFISDEVARDLSLQFKRPINPTDQSSYIVDSVTVKNGSIHLYFDKAGQKTYERHSLSH
EPI_ISL_425502 1643 GSTFISDEVARDLSLQFKRPINPTDQSSYIVDSVTVKNGSIHLYFDKAGQKTYERHSLSH
EPI_ISL_425504 1643 GSTFISDEVARDLSLQFKRPINPTDQSSYIVDSVTVKNGSIHLYFDKAGQKTYERHSLSH
EPI_ISL_425523 1643 GSTFISDEVARDLSLQFKRPINPTDQSSYIVDSVTVKNGSIHLYFDKAGQKTYERHSLSH
EPI_ISL_425535 1643 GSTFISDEVARDLSLQFKRPINPTDQSSYIVDSVTVKNGSIHLYFDKAGQKTYERHSLSH
EPI_ISL_425537 1643 GSTFISDEVARDLSLQFKRPINPTDQSSYIVDSVTVKNGSIHLYFDKAGQKTYERHSLSH
EPI_ISL_425549 1643 GSTFISDEVARDLSLQFKRPINPTDQSSYIVDSVTVKNGSIHLYFDKAGQKTYERHSLSH
EPI_ISL_425551 1643 GSTFISDEVARDLSLQFKRPINPTDQSSYIVDSVTVKNGSIHLYFDKAGQKTYERHSLSH
EPI_ISL_425562 1643 GSTFISDEVARDLSLQFKRPINPTDQSSYIVDSVTVKNGSIHLYFDKAGQKTYERHSLSH
EPI_ISL_425576 1643 GSTFISDEVARDLSLQFKRPINPTDQSSYIVDSVTVKNGSIHLYFDKAGQKTYERHSLSH
EPI_ISL_425586 1643 GSTFISDEVARDLSLQFKRPINPTDQSSYIVDSVTVKNGSIHLYFDKAGQKTYERHSLSH
EPI_ISL_425587 1643 GSTFISDEVARDLSLQFKRPINPTDQSSYIVDSVTVKNGSIHLYFDKAGQKTYERHSLSH
EPI_ISL_426817 1643 GSTFISDEVARDLSLQFKRPINPTDQSSYIVDSVTVKNGSIHLYFDKAGQKTYERHSLSH
EPI_ISL_426832 1643 GSTFISDEVARDLSLQFKRPINPTDQSSYIVDSVTVKNGSIHLYFDKAGQKTYERHSLSH
```

```
ORF1ab         2521 FVNLDNLRANNTKGSLPINVIVFDGKSKCEESSAKSASVYYSQLMCQPILLLDQALVSDV
EPI_ISL_424224 1703 FVNLDNLRANNTKGSLPINVIVFDGKSKCEESSAKSASVYYSQLMCQPILLLDQALVSDV
EPI_ISL_424255 1703 FVNLDNLRANNTKGSLPINVIVFDGKSKCEESSAKSASVYYSQLMCQPILLLDQALVSDV
EPI_ISL_425501 1703 FVNLDNLRANNTKGSLPINVIVFDGKSKCEESSAKSASVYYSQLMCQPILLLDQALVSDV
EPI_ISL_425502 1703 FVNLDNLRANNTKGSLPINVIVFDGKSKCEESSAKSASVYYSQLMCQPILLLDQALVSDV
EPI_ISL_425504 1703 FVNLDNLRANNTKGSLPINVIVFDGKSKCEESSAKSASVYYSQLMCQPILLLDQALVSDV
EPI_ISL_425523 1703 FVNLDNLRANNTKGSLPINVIVFDGKSKCEESSAKSASVYYSQLMCQPILLLDQALVSDV
EPI_ISL_425535 1703 FVNLDNLRANNTKGSLPINVIVFDGKSKCEESSAKSASVYYSQLMCQPILLLDQALVSDV
EPI_ISL_425537 1703 FVNLDNLRANNTKGSLPINVIVFDGKSKCEESSAKSASVYYSQLMCQPILLLDQALVSDV
EPI_ISL_425549 1703 FVNLDNLRANNTKGSLPINVIVFDGKSKCEESSAKSASVYYSQLMCQPILLLDQALVSDV
EPI_ISL_425551 1703 FVNLDNLRANNTKGSLPINVIVFDGKSKCEESSAKSASVYYSQLMCQPILLLDQALVSDV
EPI_ISL_425562 1703 FVNLDNLRANNTKGSLPINVIVFDGKSKCEESSAKSASVYYSQLMCQPILLLDQALVSDV
EPI_ISL_425576 1703 FVNLDNLRANNTKGSLPINVIVFDGKSKCEESSAKSASVYYSQLMCQPILLLDQALVSDV
EPI_ISL_425586 1703 FVNLDNLRANNTKGSLPINVIVFDGKSKCEESSAKSASVYYSQLMCQPILLLDQALVSDV
EPI_ISL_425587 1703 FVNLDNLRANNTKGSLPINVIVFDGKSKCEESSAKSASVYYSQLMCQPILLLDQALVSDV
EPI_ISL_426817 1703 FVNLDNLRANNTKGSLPINVIVFDGKSKCEESSAKSASVYYSQLMCQPILLLDQALVSDV
EPI_ISL_426832 1703 FVNLDNLRANNTKGSLPINVIVFDGKSKCEESSAKSASVYYSQLMCQPILLLDQALVSDV

ORF1ab         2581 GDSAEVAVKMFDAYVNTFSSTFNVPMEKLKTLVATAEAELAKNVSLDNVLSTFISAARQG
EPI_ISL_424224 1763 GDSAEVAVKMFDAYVNTFSSTFNVPMEKLKTLVATAEAELAKNVSLDNVLSTFISAARQG
EPI_ISL_424255 1763 GDSAEVAVKMFDAYVNTFSSTFNVSMEKLKTLVATAEAELAKNVSLDNVLSTFISAARQG
EPI_ISL_425501 1763 GDSAEVAVRMFDAYVNTFSSTFNVPMEKLKTLVATAEAELAKNVSLDNVLSTFISAARQG
EPI_ISL_425502 1763 GDSAEVAVKMFDAYVNTFSSTFNVPMEKLKTLVATAEAELAKNVSLDNVLSTFISAARQG
EPI_ISL_425504 1763 GDSAEVAVKMFDAYVNTFSSTFNVPMEKLKTLVATAEAELAKNVSLDNVLSTFISAARQG
EPI_ISL_425523 1763 GDSAEVAVKMFDAYVNTFSSTFNVPMEKLKTLVATAEAELAKNVSLDNVLSTFISAARQG
EPI_ISL_425535 1763 GDSAEVAVKMFDAYVNTFSSTFNVPMEKLKTLVATAEAELAKNVSLDNVLSTFISAARQG
EPI_ISL_425537 1763 GDSAEVAVKMFDAYVNTFSSTFNVPMEKLKTLVATAEAELAKNVSLDNVLSTFISAARQG
EPI_ISL_425549 1763 GDSAEVAVKMFDAYVNTFSSTFNVPMEKLKTLVATAEAELAKNVSLDNVLSTFISAARQG
EPI_ISL_425551 1763 GDSAEVAVKMFDAYVNTFSSTFNVPMEKLKTLVATAEAELAKNVSLDNVLSTFISAARQG
EPI_ISL_425562 1763 GDSAEVAVKMFDAYVNTFSSTFNVPMEKLKTLVATAEAELAKNVSLDNVLSTFISAARQG
EPI_ISL_425576 1763 GDSAEVAVKMFDAYVNTFSSTFNVPMEKLKTLVATAEAELAKNVSLDNVLSTFISAARQG
EPI_ISL_425586 1763 GDSAEVAVKMFDAYVNTFSSTFNVPMEKLKTLVATAEAELAKNVSLDNVLSTFISAARQG
EPI_ISL_425587 1763 GDSAEVAVKMFDAYVNTFSSTFNVPMEKLKTLVATAEAELAKNVSLDNVLSTFISAARQG
EPI_ISL_426817 1763 GDSAEVAVKMFDAYVNTFSSTFNVPMEKLKTLVATAEAELAKNVSLDNVLSTFISAARQG
EPI_ISL_426832 1763 GDSAEVAVKMFDAYVNTFSSTFNVPMEKLKTLVATAEAELAKNVSLDNVLSTFISAARQG

ORF1ab         2641 FVDSDVETKDVVECLKLSHQSDIEVTGDSCNNYMLTYNKVENMTPRDLGACIDCSARHIN
EPI_ISL_424224 1823 FVDSDVETKDVVECLKLSHQSDIEVTGDSCNNYMLTYNKVENMTPRDLGACIDCSARHIN
EPI_ISL_424255 1823 FVDSDVETKDVVECLKLSHQSDIEVTGDSCNNYMLTYNKVENMTPRDLGACIDCSARHIN
EPI_ISL_425501 1823 FVDSDVETKDVVECLKLSHQSDIEVTGDSCNNYMLTYNKVENMTPRDLGACIDCSARHIN
EPI_ISL_425502 1823 FVDSDVETKDVVECLKLSHQSDIEVTGDSCNNYMLTYNKVENMTPRDLGACIDCSARHIN
EPI_ISL_425504 1823 FVDSDVETKDVVECLKLSHQSDIEVTGDSCNNYMLTYNKVENMTPRDLGACIDCSARHIN
EPI_ISL_425523 1823 FVDSDVETKDVVECLKLSHQSDIEVTGDSCNNYMLTYNKVENMTPRDLGACIDCSARHIN
EPI_ISL_425535 1823 FVDSDVETKDVVECLKLSHQSDIEVTGDSCNNYMLTYNKVENMTPRDLGACIDCSARHIN
EPI_ISL_425537 1823 FVDSDVETKDVVECLKLSHQSDIEVTGDSCNNYMLTYNKVENMTPRDLGACIDCSARHIN
EPI_ISL_425549 1823 FVDSDVETKDVVECLKLSHQSDIEVTGDSCNNYMLTYNKVENMTPRDLGACIDCSARHIN
EPI_ISL_425551 1823 FVDSDVETKDVVECLKLSHQSDIEVTGDSCNNYMLTYNKVENMTPRDLGACIDCSARHIN
EPI_ISL_425562 1823 FVDSDVETKDVVECLKLSHQSDIEVTGDSCNNYMLTYNKVENMTPRDLGACIDCSARHIN
EPI_ISL_425576 1823 FVDSDVETKDVVECLKLSHQSDIEVTGDSCNNYMLTYNKVENMTPRDLGACIDCSARHIN
EPI_ISL_425586 1823 FVDSDVETKDVVECLKLSHQSDIEVTGDSCNNYMLTYNKVENMTPRDLGACIDCSARHIN
EPI_ISL_425587 1823 FVDSDVETKDVVECLKLSHQSDIEVTGDSCNNYMLTYNKVENMTPRDLGACIDCSARHIN
EPI_ISL_426817 1823 FVDSDVETKDVVECLKLSHQSDIEVTGDSCNNYMLTYNKVENMTPRDLGACIDCSARHIN
EPI_ISL_426832 1823 FVDSDVETKDVVECLKLSHQSDIEVTGDSCNNYMLTYNKVENMTPRDLGACIDCSARHIN

ORF1ab         2701 AQVAKSHNIALIWNVKDFMSLSEQLRKQIRSAAKKNNLPFKLTCATTRQVVNVVTTKIAL
EPI_ISL_424224 1883 AQVAKSHNIALIWNVKDFMSLSEQLRKQIRSAAKKNNLPFKLTCATTRQVVNVVTTKIAL
EPI_ISL_424255 1883 AQVAKSHNIALIWNVKDFMSLSEQLRKQIRSAAKKNNLPFKLTCATTRQVVNVVTTKIAL
EPI_ISL_425501 1883 AQVAKSHNIALIWNVKDFMSLSEQLRKQIRSAAKKNNLPFKLTCATTRQVVNVVTTKIAL
EPI_ISL_425502 1883 AQVAKSHNIALIWNVKDFMSLSEQLRKQIRSAAKKNNLPFKLTCATTRQVVNVVTTKIAL
EPI_ISL_425504 1883 AQVAKSHNIALIWNVKDFMSLSEQLRKQIRSAAKKNNLPFKLTCATTRQVVNVVTTKIAL
EPI_ISL_425523 1883 AQVAKSHNIALIWNVKDFMSLSEQLRKQIRSAAKKNNLPFKLTCATTRQVVNVVTTKIAL
EPI_ISL_425535 1883 AQVAKSHNIALIWNVKDFMSLSEQLRKQIRSAAKKNNLPFKLTCATTRQVVNVVTTKIAL
EPI_ISL_425537 1883 AQVAKSHNIALIWNVKDFMSLSEQLRKQIRSAAKKNNLPFKLTCATTRQVVNVVTTKIAL
```

```
EPI_ISL_425549 1883 AQVAKSHNIALIWNVKDFMSLSEQLRKQIRSAAKKNNLPFKLTCATTRQVVNVVTTKIAL
EPI_ISL_425551 1883 AQVAKSHNIALIWNVKDFMSLSEQLRKQIRSAAKKNNLPFKLTCATTRQVVNVVTTKIAL
EPI_ISL_425562 1883 AQVAKSHNIALIWNVKDFMSLSEQLRKQIRSAAKKNNLPFKLTCATTRQVVNVVTTKIAL
EPI_ISL_425576 1883 AQVAKSHNIALIWNVKDFMSLSEQLRKQIRSAAKKNNLPFKLTCATTRQVVNVVTTKIAL
EPI_ISL_425586 1883 AQVAKSHNIALIWNVKDFMSLSEQLRKQIRSAAKKNNLPFKLTCATTRQVVNVVTTKIAL
EPI_ISL_425587 1883 AQVAKSHNIALIWNVKDFMSLSEQLRKQIRSAAKKNNLPFKLTCATTRQVVNVVTTKIAL
EPI_ISL_426817 1883 AQVAKSHNIALIWNVKDFMSLSEQLRKQIRSAAKKNNLPFKLTCATTRQVVNVVTTKIAL
EPI_ISL_426832 1883 AQVAKSHNIALIWNVKDFMSLSEQLRKQIRSAAKKNNLPFKLTCATTRQVVNVVTTKIAL
```

```
ORF1ab         2761 KGGKIVNNWLKQLIKVTLVFLFVAAIFYLITPVHVMSKHTDFSSEIIGYKAIDGGVTRDI
EPI_ISL_424224 1943 KGG---------------------------------------------------------
EPI_ISL_424255 1943 KGG---------------------------------------------------------
EPI_ISL_425501 1943 KGG---------------------------------------------------------
EPI_ISL_425502 1943 KGG---------------------------------------------------------
EPI_ISL_425504 1943 KGG---------------------------------------------------------
EPI_ISL_425523 1943 KGG---------------------------------------------------------
EPI_ISL_425535 1943 KGG---------------------------------------------------------
EPI_ISL_425537 1943 KGG---------------------------------------------------------
EPI_ISL_425549 1943 KGG---------------------------------------------------------
EPI_ISL_425551 1943 KGG---------------------------------------------------------
EPI_ISL_425562 1943 KGG---------------------------------------------------------
EPI_ISL_425576 1943 KGG---------------------------------------------------------
EPI_ISL_425586 1943 KGG---------------------------------------------------------
EPI_ISL_425587 1943 KGG---------------------------------------------------------
EPI_ISL_426817 1943 KGG---------------------------------------------------------
EPI_ISL_426832 1943 KGG---------------------------------------------------------

ORF1ab         2821 ASTDTCFANKHADFDTWFSQRGGSYTNDKACPLIAAVITREVGFVVPGLPGTILRTTNGD
EPI_ISL_424224      ------------------------------------------------------------
EPI_ISL_424255      ------------------------------------------------------------
EPI_ISL_425501      ------------------------------------------------------------
EPI_ISL_425502      ------------------------------------------------------------
EPI_ISL_425504      ------------------------------------------------------------
EPI_ISL_425523      ------------------------------------------------------------
EPI_ISL_425535      ------------------------------------------------------------
EPI_ISL_425537      ------------------------------------------------------------
EPI_ISL_425549      ------------------------------------------------------------
EPI_ISL_425551      ------------------------------------------------------------
EPI_ISL_425562      ------------------------------------------------------------
EPI_ISL_425576      ------------------------------------------------------------
EPI_ISL_425586      ------------------------------------------------------------
EPI_ISL_425587      ------------------------------------------------------------
EPI_ISL_426817      ------------------------------------------------------------
EPI_ISL_426832      ------------------------------------------------------------

ORF1ab         2881 FLHFLPRVFSAVGNICYTPSKLIEYTDFATSACVLAAECTIFKDASGKPVPYCYDTNVLE
EPI_ISL_424224      ------------------------------------------------------------
EPI_ISL_424255      ------------------------------------------------------------
EPI_ISL_425501      ------------------------------------------------------------
EPI_ISL_425502      ------------------------------------------------------------
EPI_ISL_425504      ------------------------------------------------------------
EPI_ISL_425523      ------------------------------------------------------------
EPI_ISL_425535      ------------------------------------------------------------
EPI_ISL_425537      ------------------------------------------------------------
EPI_ISL_425549      ------------------------------------------------------------
EPI_ISL_425551      ------------------------------------------------------------
EPI_ISL_425562      ------------------------------------------------------------
EPI_ISL_425576      ------------------------------------------------------------
EPI_ISL_425586      ------------------------------------------------------------
EPI_ISL_425587      ------------------------------------------------------------
EPI_ISL_426817      ------------------------------------------------------------
EPI_ISL_426832      ------------------------------------------------------------
```

```
ORF1ab          2941 GSVAYESLRPDTRYVLMDGSIIQFPNTYLEGSVRVVTTFDSEYCRHGTCERSEAGVCVST
EPI_ISL_424224       ------------------------------------------------------------
EPI_ISL_424255       ------------------------------------------------------------
EPI_ISL_425501       ------------------------------------------------------------
EPI_ISL_425502       ------------------------------------------------------------
EPI_ISL_425504       ------------------------------------------------------------
EPI_ISL_425523       ------------------------------------------------------------
EPI_ISL_425535       ------------------------------------------------------------
EPI_ISL_425537       ------------------------------------------------------------
EPI_ISL_425549       ------------------------------------------------------------
EPI_ISL_425551       ------------------------------------------------------------
EPI_ISL_425562       ------------------------------------------------------------
EPI_ISL_425576       ------------------------------------------------------------
EPI_ISL_425586       ------------------------------------------------------------
EPI_ISL_425587       ------------------------------------------------------------
EPI_ISL_426817       ------------------------------------------------------------
EPI_ISL_426832       ------------------------------------------------------------

ORF1ab          3001 SGRWVLNNDYYRSLPGVFCGVDAVNLLTNMFTPLIQPIGALDISASIVAGGIVAIVVTCL
EPI_ISL_424224       ------------------------------------------------------------
EPI_ISL_424255       ------------------------------------------------------------
EPI_ISL_425501       ------------------------------------------------------------
EPI_ISL_425502       ------------------------------------------------------------
EPI_ISL_425504       ------------------------------------------------------------
EPI_ISL_425523       ------------------------------------------------------------
EPI_ISL_425535       ------------------------------------------------------------
EPI_ISL_425537       ------------------------------------------------------------
EPI_ISL_425549       ------------------------------------------------------------
EPI_ISL_425551       ------------------------------------------------------------
EPI_ISL_425562       ------------------------------------------------------------
EPI_ISL_425576       ------------------------------------------------------------
EPI_ISL_425586       ------------------------------------------------------------
EPI_ISL_425587       ------------------------------------------------------------
EPI_ISL_426817       ------------------------------------------------------------
EPI_ISL_426832       ------------------------------------------------------------

ORF1ab          3061 AYYFMRFRRAFGEYSHVVAFNTLLFLMSFTVLCLTPVYSFLPGVYSVIYLYLTFYLTNDV
EPI_ISL_424224       ------------------------------------------------------------
EPI_ISL_424255       ------------------------------------------------------------
EPI_ISL_425501       ------------------------------------------------------------
EPI_ISL_425502       ------------------------------------------------------------
EPI_ISL_425504       ------------------------------------------------------------
EPI_ISL_425523       ------------------------------------------------------------
EPI_ISL_425535       ------------------------------------------------------------
EPI_ISL_425537       ------------------------------------------------------------
EPI_ISL_425549       ------------------------------------------------------------
EPI_ISL_425551       ------------------------------------------------------------
EPI_ISL_425562       ------------------------------------------------------------
EPI_ISL_425576       ------------------------------------------------------------
EPI_ISL_425586       ------------------------------------------------------------
EPI_ISL_425587       ------------------------------------------------------------
EPI_ISL_426817       ------------------------------------------------------------
EPI_ISL_426832       ------------------------------------------------------------

ORF1ab          3121 SFLAHIQWMVMFTPLVPFWITIAYIICISTKHFYWFFSNYLKRRVVFNGVSFSTFEEAAL
EPI_ISL_424224       ------------------------------------------------------------
EPI_ISL_424255       ------------------------------------------------------------
EPI_ISL_425501       ------------------------------------------------------------
EPI_ISL_425502       ------------------------------------------------------------
EPI_ISL_425504       ------------------------------------------------------------
EPI_ISL_425523       ------------------------------------------------------------
EPI_ISL_425535       ------------------------------------------------------------
EPI_ISL_425537       ------------------------------------------------------------
```

```
EPI_ISL_425549      ------------------------------------------------------------
EPI_ISL_425551      ------------------------------------------------------------
EPI_ISL_425562      ------------------------------------------------------------
EPI_ISL_425576      ------------------------------------------------------------
EPI_ISL_425586      ------------------------------------------------------------
EPI_ISL_425587      ------------------------------------------------------------
EPI_ISL_426817      ------------------------------------------------------------
EPI_ISL_426832      ------------------------------------------------------------
```

```
ORF1ab          3181 CTFLLNKEMYLKLRSDVLLPLTQYNRYLALYNKYKYFSGAMDTTSYREAACCHLAKALND
EPI_ISL_424224       ------------------------------------------------------------
EPI_ISL_424255       ------------------------------------------------------------
EPI_ISL_425501       ------------------------------------------------------------
EPI_ISL_425502       ------------------------------------------------------------
EPI_ISL_425504       ------------------------------------------------------------
EPI_ISL_425523       ------------------------------------------------------------
EPI_ISL_425535       ------------------------------------------------------------
EPI_ISL_425537       ------------------------------------------------------------
EPI_ISL_425549       ------------------------------------------------------------
EPI_ISL_425551       ------------------------------------------------------------
EPI_ISL_425562       ------------------------------------------------------------
EPI_ISL_425576       ------------------------------------------------------------
EPI_ISL_425586       ------------------------------------------------------------
EPI_ISL_425587       ------------------------------------------------------------
EPI_ISL_426817       ------------------------------------------------------------
EPI_ISL_426832       ------------------------------------------------------------

ORF1ab          3241 FSNSGSDVLYQPPQTSITSAVLQSGFRKMAFPSGKVEGCMVQVTCGTTTLNGLWLDDVVY
EPI_ISL_424224       ------------------------------------------------------------
EPI_ISL_424255       ------------------------------------------------------------
EPI_ISL_425501       ------------------------------------------------------------
EPI_ISL_425502       ------------------------------------------------------------
EPI_ISL_425504       ------------------------------------------------------------
EPI_ISL_425523       ------------------------------------------------------------
EPI_ISL_425535       ------------------------------------------------------------
EPI_ISL_425537       ------------------------------------------------------------
EPI_ISL_425549       ------------------------------------------------------------
EPI_ISL_425551       ------------------------------------------------------------
EPI_ISL_425562       ------------------------------------------------------------
EPI_ISL_425576       ------------------------------------------------------------
EPI_ISL_425586       ------------------------------------------------------------
EPI_ISL_425587       ------------------------------------------------------------
EPI_ISL_426817       ------------------------------------------------------------
EPI_ISL_426832       ------------------------------------------------------------

ORF1ab          3301 CPRHVICTSEDMLNPNYEDLLIRKSNHNFLVQAGNVQLRVIGHSMQNCVLKLKVDTANPK
EPI_ISL_424224       ------------------------------------------------------------
EPI_ISL_424255       ------------------------------------------------------------
EPI_ISL_425501       ------------------------------------------------------------
EPI_ISL_425502       ------------------------------------------------------------
EPI_ISL_425504       ------------------------------------------------------------
EPI_ISL_425523       ------------------------------------------------------------
EPI_ISL_425535       ------------------------------------------------------------
EPI_ISL_425537       ------------------------------------------------------------
EPI_ISL_425549       ------------------------------------------------------------
EPI_ISL_425551       ------------------------------------------------------------
EPI_ISL_425562       ------------------------------------------------------------
EPI_ISL_425576       ------------------------------------------------------------
EPI_ISL_425586       ------------------------------------------------------------
EPI_ISL_425587       ------------------------------------------------------------
EPI_ISL_426817       ------------------------------------------------------------
EPI_ISL_426832       ------------------------------------------------------------
```

```
ORF1ab          3361 TPKYKFVRIQPGQTFSVLACYNGSPSGVYQCAMRPNFTIKGSFLNGSCGSVGFNIDYDCV
EPI_ISL_424224       ------------------------------------------------------------
EPI_ISL_424255       ------------------------------------------------------------
EPI_ISL_425501       ------------------------------------------------------------
EPI_ISL_425502       ------------------------------------------------------------
EPI_ISL_425504       ------------------------------------------------------------
EPI_ISL_425523       ------------------------------------------------------------
EPI_ISL_425535       ------------------------------------------------------------
EPI_ISL_425537       ------------------------------------------------------------
EPI_ISL_425549       ------------------------------------------------------------
EPI_ISL_425551       ------------------------------------------------------------
EPI_ISL_425562       ------------------------------------------------------------
EPI_ISL_425576       ------------------------------------------------------------
EPI_ISL_425586       ------------------------------------------------------------
EPI_ISL_425587       ------------------------------------------------------------
EPI_ISL_426817       ------------------------------------------------------------
EPI_ISL_426832       ------------------------------------------------------------

ORF1ab          3421 SFCYMHHMELPTGVHAGTDLEGNFYGPFVDRQTAQAAGTDTTITVNVLAWLYAAVINGDR
EPI_ISL_424224       ------------------------------------------------------------
EPI_ISL_424255       ------------------------------------------------------------
EPI_ISL_425501       ------------------------------------------------------------
EPI_ISL_425502       ------------------------------------------------------------
EPI_ISL_425504       ------------------------------------------------------------
EPI_ISL_425523       ------------------------------------------------------------
EPI_ISL_425535       ------------------------------------------------------------
EPI_ISL_425537       ------------------------------------------------------------
EPI_ISL_425549       ------------------------------------------------------------
EPI_ISL_425551       ------------------------------------------------------------
EPI_ISL_425562       ------------------------------------------------------------
EPI_ISL_425576       ------------------------------------------------------------
EPI_ISL_425586       ------------------------------------------------------------
EPI_ISL_425587       ------------------------------------------------------------
EPI_ISL_426817       ------------------------------------------------------------
EPI_ISL_426832       ------------------------------------------------------------

ORF1ab          3481 WFLNRFTTTLNDFNLVAMKYNYEPLTQDHVDILGPLSAQTGIAVLDMCASLKELLQNGMN
EPI_ISL_424224       ------------------------------------------------------------
EPI_ISL_424255       ------------------------------------------------------------
EPI_ISL_425501       ------------------------------------------------------------
EPI_ISL_425502       ------------------------------------------------------------
EPI_ISL_425504       ------------------------------------------------------------
EPI_ISL_425523       ------------------------------------------------------------
EPI_ISL_425535       ------------------------------------------------------------
EPI_ISL_425537       ------------------------------------------------------------
EPI_ISL_425549       ------------------------------------------------------------
EPI_ISL_425551       ------------------------------------------------------------
EPI_ISL_425562       ------------------------------------------------------------
EPI_ISL_425576       ------------------------------------------------------------
EPI_ISL_425586       ------------------------------------------------------------
EPI_ISL_425587       ------------------------------------------------------------
EPI_ISL_426817       ------------------------------------------------------------
EPI_ISL_426832       ------------------------------------------------------------

ORF1ab          3541 GRTILGSALLEDEFTPFDVVRQCSGVTFQSAVKRTIKGTHHWLLLTILTSLLVLVQSTQW
EPI_ISL_424224       ------------------------------------------------------------
EPI_ISL_424255       ------------------------------------------------------------
EPI_ISL_425501       ------------------------------------------------------------
EPI_ISL_425502       ------------------------------------------------------------
EPI_ISL_425504       ------------------------------------------------------------
EPI_ISL_425523       ------------------------------------------------------------
EPI_ISL_425535       ------------------------------------------------------------
EPI_ISL_425537       ------------------------------------------------------------
```

```
EPI_ISL_425549      ------------------------------------------------------------
EPI_ISL_425551      ------------------------------------------------------------
EPI_ISL_425562      ------------------------------------------------------------
EPI_ISL_425576      ------------------------------------------------------------
EPI_ISL_425586      ------------------------------------------------------------
EPI_ISL_425587      ------------------------------------------------------------
EPI_ISL_426817      ------------------------------------------------------------
EPI_ISL_426832      ------------------------------------------------------------
```

```
ORF1ab          3601 SLFFFLYENAFLPFAMGIIAMSAFAMMFVKHKHAFLCLFLLPSLATVAYFNMVYMPASWV
EPI_ISL_424224       ------------------------------------------------------------
EPI_ISL_424255       ------------------------------------------------------------
EPI_ISL_425501       ------------------------------------------------------------
EPI_ISL_425502       ------------------------------------------------------------
EPI_ISL_425504       ------------------------------------------------------------
EPI_ISL_425523       ------------------------------------------------------------
EPI_ISL_425535       ------------------------------------------------------------
EPI_ISL_425537       ------------------------------------------------------------
EPI_ISL_425549       ------------------------------------------------------------
EPI_ISL_425551       ------------------------------------------------------------
EPI_ISL_425562       ------------------------------------------------------------
EPI_ISL_425576       ------------------------------------------------------------
EPI_ISL_425586       ------------------------------------------------------------
EPI_ISL_425587       ------------------------------------------------------------
EPI_ISL_426817       ------------------------------------------------------------
EPI_ISL_426832       ------------------------------------------------------------

ORF1ab          3661 MRIMTWLDMVDTSLSGFKLKDCVMYASAVVLLILMTARTVYDDGARRVWTLMNVLTLVYK
EPI_ISL_424224       ------------------------------------------------------------
EPI_ISL_424255       ------------------------------------------------------------
EPI_ISL_425501       ------------------------------------------------------------
EPI_ISL_425502       ------------------------------------------------------------
EPI_ISL_425504       ------------------------------------------------------------
EPI_ISL_425523       ------------------------------------------------------------
EPI_ISL_425535       ------------------------------------------------------------
EPI_ISL_425537       ------------------------------------------------------------
EPI_ISL_425549       ------------------------------------------------------------
EPI_ISL_425551       ------------------------------------------------------------
EPI_ISL_425562       ------------------------------------------------------------
EPI_ISL_425576       ------------------------------------------------------------
EPI_ISL_425586       ------------------------------------------------------------
EPI_ISL_425587       ------------------------------------------------------------
EPI_ISL_426817       ------------------------------------------------------------
EPI_ISL_426832       ------------------------------------------------------------

ORF1ab          3721 VYYGNALDQAISMWALIISVTSNYSGVVTTVMFLARGIVFMCVEYCPIFFITGNTLQCIM
EPI_ISL_424224       ------------------------------------------------------------
EPI_ISL_424255       ------------------------------------------------------------
EPI_ISL_425501       ------------------------------------------------------------
EPI_ISL_425502       ------------------------------------------------------------
EPI_ISL_425504       ------------------------------------------------------------
EPI_ISL_425523       ------------------------------------------------------------
EPI_ISL_425535       ------------------------------------------------------------
EPI_ISL_425537       ------------------------------------------------------------
EPI_ISL_425549       ------------------------------------------------------------
EPI_ISL_425551       ------------------------------------------------------------
EPI_ISL_425562       ------------------------------------------------------------
EPI_ISL_425576       ------------------------------------------------------------
EPI_ISL_425586       ------------------------------------------------------------
EPI_ISL_425587       ------------------------------------------------------------
EPI_ISL_426817       ------------------------------------------------------------
EPI_ISL_426832       ------------------------------------------------------------
```

```
ORF1ab         3781 LVYCFLGYFCTCYFGLFCLLNRYFRLTLGVYDYLVSTQEFRYMNSQGLLPPKNSIDAFKL
EPI_ISL_424224      ------------------------------------------------------------
EPI_ISL_424255      ------------------------------------------------------------
EPI_ISL_425501      ------------------------------------------------------------
EPI_ISL_425502      ------------------------------------------------------------
EPI_ISL_425504      ------------------------------------------------------------
EPI_ISL_425523      ------------------------------------------------------------
EPI_ISL_425535      ------------------------------------------------------------
EPI_ISL_425537      ------------------------------------------------------------
EPI_ISL_425549      ------------------------------------------------------------
EPI_ISL_425551      ------------------------------------------------------------
EPI_ISL_425562      ------------------------------------------------------------
EPI_ISL_425576      ------------------------------------------------------------
EPI_ISL_425586      ------------------------------------------------------------
EPI_ISL_425587      ------------------------------------------------------------
EPI_ISL_426817      ------------------------------------------------------------
EPI_ISL_426832      ------------------------------------------------------------

ORF1ab         3841 NIKLLGVGGKPCIKVATVQSKMSDVKCTSVVLLSVLQQLRVESSSKLWAQCVQLHNDILL
EPI_ISL_424224      ------------------------------------------------------------
EPI_ISL_424255      ------------------------------------------------------------
EPI_ISL_425501      ------------------------------------------------------------
EPI_ISL_425502      ------------------------------------------------------------
EPI_ISL_425504      ------------------------------------------------------------
EPI_ISL_425523      ------------------------------------------------------------
EPI_ISL_425535      ------------------------------------------------------------
EPI_ISL_425537      ------------------------------------------------------------
EPI_ISL_425549      ------------------------------------------------------------
EPI_ISL_425551      ------------------------------------------------------------
EPI_ISL_425562      ------------------------------------------------------------
EPI_ISL_425576      ------------------------------------------------------------
EPI_ISL_425586      ------------------------------------------------------------
EPI_ISL_425587      ------------------------------------------------------------
EPI_ISL_426817      ------------------------------------------------------------
EPI_ISL_426832      ------------------------------------------------------------

ORF1ab         3901 AKDTTEAFEKMVSLLSVLLSMQGAVDINKLCEEMLDNRATLQAIASEFSSLPSYAAFATA
EPI_ISL_424224      ------------------------------------------------------------
EPI_ISL_424255      ------------------------------------------------------------
EPI_ISL_425501      ------------------------------------------------------------
EPI_ISL_425502      ------------------------------------------------------------
EPI_ISL_425504      ------------------------------------------------------------
EPI_ISL_425523      ------------------------------------------------------------
EPI_ISL_425535      ------------------------------------------------------------
EPI_ISL_425537      ------------------------------------------------------------
EPI_ISL_425549      ------------------------------------------------------------
EPI_ISL_425551      ------------------------------------------------------------
EPI_ISL_425562      ------------------------------------------------------------
EPI_ISL_425576      ------------------------------------------------------------
EPI_ISL_425586      ------------------------------------------------------------
EPI_ISL_425587      ------------------------------------------------------------
EPI_ISL_426817      ------------------------------------------------------------
EPI_ISL_426832      ------------------------------------------------------------

ORF1ab         3961 QEAYEQAVANGDSEVVLKKLKKSLNVAKSEFDRDAAMQRKLEKMADQAMTQMYKQARSED
EPI_ISL_424224      ------------------------------------------------------------
EPI_ISL_424255      ------------------------------------------------------------
EPI_ISL_425501      ------------------------------------------------------------
EPI_ISL_425502      ------------------------------------------------------------
EPI_ISL_425504      ------------------------------------------------------------
EPI_ISL_425523      ------------------------------------------------------------
EPI_ISL_425535      ------------------------------------------------------------
EPI_ISL_425537      ------------------------------------------------------------
```

```
EPI_ISL_425549      ------------------------------------------------------------
EPI_ISL_425551      ------------------------------------------------------------
EPI_ISL_425562      ------------------------------------------------------------
EPI_ISL_425576      ------------------------------------------------------------
EPI_ISL_425586      ------------------------------------------------------------
EPI_ISL_425587      ------------------------------------------------------------
EPI_ISL_426817      ------------------------------------------------------------
EPI_ISL_426832      ------------------------------------------------------------
```

```
ORF1ab         4021 KRAKVTSAMQTMLFTMLRKLDNDALNNIINNARDGCVPLNIIPLTTAAKLMVVIPDYNTY
EPI_ISL_424224      ------------------------------------------------------------
EPI_ISL_424255      ------------------------------------------------------------
EPI_ISL_425501      ------------------------------------------------------------
EPI_ISL_425502      ------------------------------------------------------------
EPI_ISL_425504      ------------------------------------------------------------
EPI_ISL_425523      ------------------------------------------------------------
EPI_ISL_425535      ------------------------------------------------------------
EPI_ISL_425537      ------------------------------------------------------------
EPI_ISL_425549      ------------------------------------------------------------
EPI_ISL_425551      ------------------------------------------------------------
EPI_ISL_425562      ------------------------------------------------------------
EPI_ISL_425576      ------------------------------------------------------------
EPI_ISL_425586      ------------------------------------------------------------
EPI_ISL_425587      ------------------------------------------------------------
EPI_ISL_426817      ------------------------------------------------------------
EPI_ISL_426832      ------------------------------------------------------------

ORF1ab         4081 KNTCDGTTFTYASALWEIQQVVDADSKIVQLSEISMDNSPNLAWPLIVTALRANSAVKLQ
EPI_ISL_424224      ------------------------------------------------------------
EPI_ISL_424255      ------------------------------------------------------------
EPI_ISL_425501      ------------------------------------------------------------
EPI_ISL_425502      ------------------------------------------------------------
EPI_ISL_425504      ------------------------------------------------------------
EPI_ISL_425523      ------------------------------------------------------------
EPI_ISL_425535      ------------------------------------------------------------
EPI_ISL_425537      ------------------------------------------------------------
EPI_ISL_425549      ------------------------------------------------------------
EPI_ISL_425551      ------------------------------------------------------------
EPI_ISL_425562      ------------------------------------------------------------
EPI_ISL_425576      ------------------------------------------------------------
EPI_ISL_425586      ------------------------------------------------------------
EPI_ISL_425587      ------------------------------------------------------------
EPI_ISL_426817      ------------------------------------------------------------
EPI_ISL_426832      ------------------------------------------------------------

ORF1ab         4141 NNELSPVALRQMSCAAGTTQTACTDDNALAYYNTTKGGRFVLALLSDLQDLKWARFPKSD
EPI_ISL_424224      ------------------------------------------------------------
EPI_ISL_424255      ------------------------------------------------------------
EPI_ISL_425501      ------------------------------------------------------------
EPI_ISL_425502      ------------------------------------------------------------
EPI_ISL_425504      ------------------------------------------------------------
EPI_ISL_425523      ------------------------------------------------------------
EPI_ISL_425535      ------------------------------------------------------------
EPI_ISL_425537      ------------------------------------------------------------
EPI_ISL_425549      ------------------------------------------------------------
EPI_ISL_425551      ------------------------------------------------------------
EPI_ISL_425562      ------------------------------------------------------------
EPI_ISL_425576      ------------------------------------------------------------
EPI_ISL_425586      ------------------------------------------------------------
EPI_ISL_425587      ------------------------------------------------------------
EPI_ISL_426817      ------------------------------------------------------------
EPI_ISL_426832      ------------------------------------------------------------
```

```
ORF1ab         4201 GTGTIYTELEPPCRFVTDTPKGPKVKYLYFIKGLNNLNRGMVLGSLAATVRLQAGNATEV
EPI_ISL_424224      ------------------------------------------------------------
EPI_ISL_424255      ------------------------------------------------------------
EPI_ISL_425501      ------------------------------------------------------------
EPI_ISL_425502      ------------------------------------------------------------
EPI_ISL_425504      ------------------------------------------------------------
EPI_ISL_425523      ------------------------------------------------------------
EPI_ISL_425535      ------------------------------------------------------------
EPI_ISL_425537      ------------------------------------------------------------
EPI_ISL_425549      ------------------------------------------------------------
EPI_ISL_425551      ------------------------------------------------------------
EPI_ISL_425562      ------------------------------------------------------------
EPI_ISL_425576      ------------------------------------------------------------
EPI_ISL_425586      ------------------------------------------------------------
EPI_ISL_425587      ------------------------------------------------------------
EPI_ISL_426817      ------------------------------------------------------------
EPI_ISL_426832      ------------------------------------------------------------

ORF1ab         4261 PANSTVLSFCAFAVDAAKAYKDYLASGGQPITNCVKMLCTHTGTGQAITVTPEANMDQES
EPI_ISL_424224      ------------------------------------------------------------
EPI_ISL_424255      ------------------------------------------------------------
EPI_ISL_425501      ------------------------------------------------------------
EPI_ISL_425502      ------------------------------------------------------------
EPI_ISL_425504      ------------------------------------------------------------
EPI_ISL_425523      ------------------------------------------------------------
EPI_ISL_425535      ------------------------------------------------------------
EPI_ISL_425537      ------------------------------------------------------------
EPI_ISL_425549      ------------------------------------------------------------
EPI_ISL_425551      ------------------------------------------------------------
EPI_ISL_425562      ------------------------------------------------------------
EPI_ISL_425576      ------------------------------------------------------------
EPI_ISL_425586      ------------------------------------------------------------
EPI_ISL_425587      ------------------------------------------------------------
EPI_ISL_426817      ------------------------------------------------------------
EPI_ISL_426832      ------------------------------------------------------------

ORF1ab         4321 FGGASCCLYCRCHIDHPNPKGFCDLKGKYVQIPTTCANDPVGFTLKNTVCTVCGMWKGYG
EPI_ISL_424224      ------------------------------------------------------------
EPI_ISL_424255      ------------------------------------------------------------
EPI_ISL_425501      ------------------------------------------------------------
EPI_ISL_425502      ------------------------------------------------------------
EPI_ISL_425504      ------------------------------------------------------------
EPI_ISL_425523      ------------------------------------------------------------
EPI_ISL_425535      ------------------------------------------------------------
EPI_ISL_425537      ------------------------------------------------------------
EPI_ISL_425549      ------------------------------------------------------------
EPI_ISL_425551      ------------------------------------------------------------
EPI_ISL_425562      ------------------------------------------------------------
EPI_ISL_425576      ------------------------------------------------------------
EPI_ISL_425586      ------------------------------------------------------------
EPI_ISL_425587      ------------------------------------------------------------
EPI_ISL_426817      ------------------------------------------------------------
EPI_ISL_426832      ------------------------------------------------------------

ORF1ab         4381 CSCDQLREPMLQSADAQSFLNRVCGVSAARLTPCGTGTSTDVVYRAFDIYNDKVAGFAKF
EPI_ISL_424224      ------------------------------------------------------------
EPI_ISL_424255      ------------------------------------------------------------
EPI_ISL_425501      ------------------------------------------------------------
EPI_ISL_425502      ------------------------------------------------------------
EPI_ISL_425504      ------------------------------------------------------------
EPI_ISL_425523      ------------------------------------------------------------
EPI_ISL_425535      ------------------------------------------------------------
EPI_ISL_425537      ------------------------------------------------------------
```

```
EPI_ISL_425549      ------------------------------------------------------------
EPI_ISL_425551      ------------------------------------------------------------
EPI_ISL_425562      ------------------------------------------------------------
EPI_ISL_425576      ------------------------------------------------------------
EPI_ISL_425586      ------------------------------------------------------------
EPI_ISL_425587      ------------------------------------------------------------
EPI_ISL_426817      ------------------------------------------------------------
EPI_ISL_426832      ------------------------------------------------------------
```

```
ORF1ab          4441 LKTNCCRFQEKDEDDNLIDSYFVVKRHTFSNYQHEETIYNLLKDCPAVAKHDFFKFRIDG
EPI_ISL_424224       ------------------------------------------------------------
EPI_ISL_424255       ------------------------------------------------------------
EPI_ISL_425501       ------------------------------------------------------------
EPI_ISL_425502       ------------------------------------------------------------
EPI_ISL_425504       ------------------------------------------------------------
EPI_ISL_425523       ------------------------------------------------------------
EPI_ISL_425535       ------------------------------------------------------------
EPI_ISL_425537       ------------------------------------------------------------
EPI_ISL_425549       ------------------------------------------------------------
EPI_ISL_425551       ------------------------------------------------------------
EPI_ISL_425562       ------------------------------------------------------------
EPI_ISL_425576       ------------------------------------------------------------
EPI_ISL_425586       ------------------------------------------------------------
EPI_ISL_425587       ------------------------------------------------------------
EPI_ISL_426817       ------------------------------------------------------------
EPI_ISL_426832       ------------------------------------------------------------

ORF1ab          4501 DMVPHISRQRLTKYTMADLVYALRHFDEGNCDTLKEILVTYNCCDDDYFNKKDWYDFVEN
EPI_ISL_424224       ------------------------------------------------------------
EPI_ISL_424255       ------------------------------------------------------------
EPI_ISL_425501       ------------------------------------------------------------
EPI_ISL_425502       ------------------------------------------------------------
EPI_ISL_425504       ------------------------------------------------------------
EPI_ISL_425523       ------------------------------------------------------------
EPI_ISL_425535       ------------------------------------------------------------
EPI_ISL_425537       ------------------------------------------------------------
EPI_ISL_425549       ------------------------------------------------------------
EPI_ISL_425551       ------------------------------------------------------------
EPI_ISL_425562       ------------------------------------------------------------
EPI_ISL_425576       ------------------------------------------------------------
EPI_ISL_425586       ------------------------------------------------------------
EPI_ISL_425587       ------------------------------------------------------------
EPI_ISL_426817       ------------------------------------------------------------
EPI_ISL_426832       ------------------------------------------------------------

ORF1ab          4561 PDILRVYANLGERVRQALLKTVQFCDAMRNAGIVGVLTLDNQDLNGNWYDFGDFIQTTPG
EPI_ISL_424224       ------------------------------------------------------------
EPI_ISL_424255       ------------------------------------------------------------
EPI_ISL_425501       ------------------------------------------------------------
EPI_ISL_425502       ------------------------------------------------------------
EPI_ISL_425504       ------------------------------------------------------------
EPI_ISL_425523       ------------------------------------------------------------
EPI_ISL_425535       ------------------------------------------------------------
EPI_ISL_425537       ------------------------------------------------------------
EPI_ISL_425549       ------------------------------------------------------------
EPI_ISL_425551       ------------------------------------------------------------
EPI_ISL_425562       ------------------------------------------------------------
EPI_ISL_425576       ------------------------------------------------------------
EPI_ISL_425586       ------------------------------------------------------------
EPI_ISL_425587       ------------------------------------------------------------
EPI_ISL_426817       ------------------------------------------------------------
EPI_ISL_426832       ------------------------------------------------------------
```

```
ORF1ab         4621 SGVPVVDSYYSLLMPILTLTRALTAESHVDTDLTKPYIKWDLLKYDFTEERLKLFDRYFK
EPI_ISL_424224      ------------------------------------------------------------
EPI_ISL_424255      ------------------------------------------------------------
EPI_ISL_425501      ------------------------------------------------------------
EPI_ISL_425502      ------------------------------------------------------------
EPI_ISL_425504      ------------------------------------------------------------
EPI_ISL_425523      ------------------------------------------------------------
EPI_ISL_425535      ------------------------------------------------------------
EPI_ISL_425537      ------------------------------------------------------------
EPI_ISL_425549      ------------------------------------------------------------
EPI_ISL_425551      ------------------------------------------------------------
EPI_ISL_425562      ------------------------------------------------------------
EPI_ISL_425576      ------------------------------------------------------------
EPI_ISL_425586      ------------------------------------------------------------
EPI_ISL_425587      ------------------------------------------------------------
EPI_ISL_426817      ------------------------------------------------------------
EPI_ISL_426832      ------------------------------------------------------------

ORF1ab         4681 YWDQTYHPNCVNCLDDRCILHCANFNVLFSTVFPPTSFGPLVRKIFVDGVPFVVSTGYHF
EPI_ISL_424224      ------------------------------------------------------------
EPI_ISL_424255      ------------------------------------------------------------
EPI_ISL_425501      ------------------------------------------------------------
EPI_ISL_425502      ------------------------------------------------------------
EPI_ISL_425504      ------------------------------------------------------------
EPI_ISL_425523      ------------------------------------------------------------
EPI_ISL_425535      ------------------------------------------------------------
EPI_ISL_425537      ------------------------------------------------------------
EPI_ISL_425549      ------------------------------------------------------------
EPI_ISL_425551      ------------------------------------------------------------
EPI_ISL_425562      ------------------------------------------------------------
EPI_ISL_425576      ------------------------------------------------------------
EPI_ISL_425586      ------------------------------------------------------------
EPI_ISL_425587      ------------------------------------------------------------
EPI_ISL_426817      ------------------------------------------------------------
EPI_ISL_426832      ------------------------------------------------------------

ORF1ab         4741 RELGVVHNQDVNLHSSRLSFKELLVYAADPAMHAASGNLLLDKRTTCFSVAALTNNVAFQ
EPI_ISL_424224      ------------------------------------------------------------
EPI_ISL_424255      ------------------------------------------------------------
EPI_ISL_425501      ------------------------------------------------------------
EPI_ISL_425502      ------------------------------------------------------------
EPI_ISL_425504      ------------------------------------------------------------
EPI_ISL_425523      ------------------------------------------------------------
EPI_ISL_425535      ------------------------------------------------------------
EPI_ISL_425537      ------------------------------------------------------------
EPI_ISL_425549      ------------------------------------------------------------
EPI_ISL_425551      ------------------------------------------------------------
EPI_ISL_425562      ------------------------------------------------------------
EPI_ISL_425576      ------------------------------------------------------------
EPI_ISL_425586      ------------------------------------------------------------
EPI_ISL_425587      ------------------------------------------------------------
EPI_ISL_426817      ------------------------------------------------------------
EPI_ISL_426832      ------------------------------------------------------------

ORF1ab         4801 TVKPGNFNKDFYDFAVSKGFFKEGSSVELKHFFFAQDGNAAISDYDYYRYNLPTMCDIRQ
EPI_ISL_424224      ------------------------------------------------------------
EPI_ISL_424255      ------------------------------------------------------------
EPI_ISL_425501      ------------------------------------------------------------
EPI_ISL_425502      ------------------------------------------------------------
EPI_ISL_425504      ------------------------------------------------------------
EPI_ISL_425523      ------------------------------------------------------------
EPI_ISL_425535      ------------------------------------------------------------
EPI_ISL_425537      ------------------------------------------------------------
```

```
EPI_ISL_425549      ------------------------------------------------------------
EPI_ISL_425551      ------------------------------------------------------------
EPI_ISL_425562      ------------------------------------------------------------
EPI_ISL_425576      ------------------------------------------------------------
EPI_ISL_425586      ------------------------------------------------------------
EPI_ISL_425587      ------------------------------------------------------------
EPI_ISL_426817      ------------------------------------------------------------
EPI_ISL_426832      ------------------------------------------------------------
```

```
ORF1ab          4861 LLFVVEVVDKYFDCYDGGCINANQVIVNNLDKSAGFPFNKWGKARLYYDSMSYEDQDALF
EPI_ISL_424224       ------------------------------------------------------------
EPI_ISL_424255       ------------------------------------------------------------
EPI_ISL_425501       ------------------------------------------------------------
EPI_ISL_425502       ------------------------------------------------------------
EPI_ISL_425504       ------------------------------------------------------------
EPI_ISL_425523       ------------------------------------------------------------
EPI_ISL_425535       ------------------------------------------------------------
EPI_ISL_425537       ------------------------------------------------------------
EPI_ISL_425549       ------------------------------------------------------------
EPI_ISL_425551       ------------------------------------------------------------
EPI_ISL_425562       ------------------------------------------------------------
EPI_ISL_425576       ------------------------------------------------------------
EPI_ISL_425586       ------------------------------------------------------------
EPI_ISL_425587       ------------------------------------------------------------
EPI_ISL_426817       ------------------------------------------------------------
EPI_ISL_426832       ------------------------------------------------------------

ORF1ab          4921 AYTKRNVIPTITQMNLKYAISAKNRARTVAGVSICSTMTNRQFHQKLLKSIAATRGATVV
EPI_ISL_424224       ------------------------------------------------------------
EPI_ISL_424255       ------------------------------------------------------------
EPI_ISL_425501       ------------------------------------------------------------
EPI_ISL_425502       ------------------------------------------------------------
EPI_ISL_425504       ------------------------------------------------------------
EPI_ISL_425523       ------------------------------------------------------------
EPI_ISL_425535       ------------------------------------------------------------
EPI_ISL_425537       ------------------------------------------------------------
EPI_ISL_425549       ------------------------------------------------------------
EPI_ISL_425551       ------------------------------------------------------------
EPI_ISL_425562       ------------------------------------------------------------
EPI_ISL_425576       ------------------------------------------------------------
EPI_ISL_425586       ------------------------------------------------------------
EPI_ISL_425587       ------------------------------------------------------------
EPI_ISL_426817       ------------------------------------------------------------
EPI_ISL_426832       ------------------------------------------------------------

ORF1ab          4981 IGTSKFYGGWHNMLKTVYSDVENPHLMGWDYPKCDRAMPNMLRIMASLVLARKHTTCCSL
EPI_ISL_424224       ------------------------------------------------------------
EPI_ISL_424255       ------------------------------------------------------------
EPI_ISL_425501       ------------------------------------------------------------
EPI_ISL_425502       ------------------------------------------------------------
EPI_ISL_425504       ------------------------------------------------------------
EPI_ISL_425523       ------------------------------------------------------------
EPI_ISL_425535       ------------------------------------------------------------
EPI_ISL_425537       ------------------------------------------------------------
EPI_ISL_425549       ------------------------------------------------------------
EPI_ISL_425551       ------------------------------------------------------------
EPI_ISL_425562       ------------------------------------------------------------
EPI_ISL_425576       ------------------------------------------------------------
EPI_ISL_425586       ------------------------------------------------------------
EPI_ISL_425587       ------------------------------------------------------------
EPI_ISL_426817       ------------------------------------------------------------
EPI_ISL_426832       ------------------------------------------------------------
```

```
ORF1ab         5041 SHRFYRLANECAQVLSEMVMCGGSLYVKPGGTSSGDATTAYANSVFNICQAVTANVNALL
EPI_ISL_424224      ------------------------------------------------------------
EPI_ISL_424255      ------------------------------------------------------------
EPI_ISL_425501      ------------------------------------------------------------
EPI_ISL_425502      ------------------------------------------------------------
EPI_ISL_425504      ------------------------------------------------------------
EPI_ISL_425523      ------------------------------------------------------------
EPI_ISL_425535      ------------------------------------------------------------
EPI_ISL_425537      ------------------------------------------------------------
EPI_ISL_425549      ------------------------------------------------------------
EPI_ISL_425551      ------------------------------------------------------------
EPI_ISL_425562      ------------------------------------------------------------
EPI_ISL_425576      ------------------------------------------------------------
EPI_ISL_425586      ------------------------------------------------------------
EPI_ISL_425587      ------------------------------------------------------------
EPI_ISL_426817      ------------------------------------------------------------
EPI_ISL_426832      ------------------------------------------------------------

ORF1ab         5101 STDGNKIADKYVRNLQHRLYECLYRNRDVDTDFVNEFYAYLRKHFSMMILSDDAVVCFNS
EPI_ISL_424224      ------------------------------------------------------------
EPI_ISL_424255      ------------------------------------------------------------
EPI_ISL_425501      ------------------------------------------------------------
EPI_ISL_425502      ------------------------------------------------------------
EPI_ISL_425504      ------------------------------------------------------------
EPI_ISL_425523      ------------------------------------------------------------
EPI_ISL_425535      ------------------------------------------------------------
EPI_ISL_425537      ------------------------------------------------------------
EPI_ISL_425549      ------------------------------------------------------------
EPI_ISL_425551      ------------------------------------------------------------
EPI_ISL_425562      ------------------------------------------------------------
EPI_ISL_425576      ------------------------------------------------------------
EPI_ISL_425586      ------------------------------------------------------------
EPI_ISL_425587      ------------------------------------------------------------
EPI_ISL_426817      ------------------------------------------------------------
EPI_ISL_426832      ------------------------------------------------------------

ORF1ab         5161 TYASQGLVASIKNFKSVLYYQNNVFMSEAKCWTETDLTKGPHEFCSQHTMLVKQGDDYVY
EPI_ISL_424224      ------------------------------------------------------------
EPI_ISL_424255      ------------------------------------------------------------
EPI_ISL_425501      ------------------------------------------------------------
EPI_ISL_425502      ------------------------------------------------------------
EPI_ISL_425504      ------------------------------------------------------------
EPI_ISL_425523      ------------------------------------------------------------
EPI_ISL_425535      ------------------------------------------------------------
EPI_ISL_425537      ------------------------------------------------------------
EPI_ISL_425549      ------------------------------------------------------------
EPI_ISL_425551      ------------------------------------------------------------
EPI_ISL_425562      ------------------------------------------------------------
EPI_ISL_425576      ------------------------------------------------------------
EPI_ISL_425586      ------------------------------------------------------------
EPI_ISL_425587      ------------------------------------------------------------
EPI_ISL_426817      ------------------------------------------------------------
EPI_ISL_426832      ------------------------------------------------------------

ORF1ab         5221 LPYPDPSRILGAGCFVDDIVKTDGTLMIERFVSLAIDAYPLTKHPNQEYADVFHLYLQYI
EPI_ISL_424224      ------------------------------------------------------------
EPI_ISL_424255      ------------------------------------------------------------
EPI_ISL_425501      ------------------------------------------------------------
EPI_ISL_425502      ------------------------------------------------------------
EPI_ISL_425504      ------------------------------------------------------------
EPI_ISL_425523      ------------------------------------------------------------
EPI_ISL_425535      ------------------------------------------------------------
EPI_ISL_425537      ------------------------------------------------------------
```

```
EPI_ISL_425549      ------------------------------------------------------------
EPI_ISL_425551      ------------------------------------------------------------
EPI_ISL_425562      ------------------------------------------------------------
EPI_ISL_425576      ------------------------------------------------------------
EPI_ISL_425586      ------------------------------------------------------------
EPI_ISL_425587      ------------------------------------------------------------
EPI_ISL_426817      ------------------------------------------------------------
EPI_ISL_426832      ------------------------------------------------------------
```

```
ORF1ab          5281 RKLHDELTGHMLDMYSVMLTNDNTSRYWEPEFYEAMYTPHTVLQAVGACVLCNSQTSLRC
EPI_ISL_424224       ------------------------------------------------------------
EPI_ISL_424255       ------------------------------------------------------------
EPI_ISL_425501       ------------------------------------------------------------
EPI_ISL_425502       ------------------------------------------------------------
EPI_ISL_425504       ------------------------------------------------------------
EPI_ISL_425523       ------------------------------------------------------------
EPI_ISL_425535       ------------------------------------------------------------
EPI_ISL_425537       ------------------------------------------------------------
EPI_ISL_425549       ------------------------------------------------------------
EPI_ISL_425551       ------------------------------------------------------------
EPI_ISL_425562       ------------------------------------------------------------
EPI_ISL_425576       ------------------------------------------------------------
EPI_ISL_425586       ------------------------------------------------------------
EPI_ISL_425587       ------------------------------------------------------------
EPI_ISL_426817       ------------------------------------------------------------
EPI_ISL_426832       ------------------------------------------------------------

ORF1ab          5341 GACIRRPFLCCKCCYDHVISTSHKLVLSVNPYVCNAPGCDVTDVTQLYLGGMSYYCKSHK
EPI_ISL_424224       ------------------------------------------------------------
EPI_ISL_424255       ------------------------------------------------------------
EPI_ISL_425501       ------------------------------------------------------------
EPI_ISL_425502       ------------------------------------------------------------
EPI_ISL_425504       ------------------------------------------------------------
EPI_ISL_425523       ------------------------------------------------------------
EPI_ISL_425535       ------------------------------------------------------------
EPI_ISL_425537       ------------------------------------------------------------
EPI_ISL_425549       ------------------------------------------------------------
EPI_ISL_425551       ------------------------------------------------------------
EPI_ISL_425562       ------------------------------------------------------------
EPI_ISL_425576       ------------------------------------------------------------
EPI_ISL_425586       ------------------------------------------------------------
EPI_ISL_425587       ------------------------------------------------------------
EPI_ISL_426817       ------------------------------------------------------------
EPI_ISL_426832       ------------------------------------------------------------

ORF1ab          5401 PPISFPLCANGQVFGLYKNTCVGSDNVTDFNAIATCDWTNAGDYILANTCTERLKLFAAE
EPI_ISL_424224       ------------------------------------------------------------
EPI_ISL_424255       ------------------------------------------------------------
EPI_ISL_425501       ------------------------------------------------------------
EPI_ISL_425502       ------------------------------------------------------------
EPI_ISL_425504       ------------------------------------------------------------
EPI_ISL_425523       ------------------------------------------------------------
EPI_ISL_425535       ------------------------------------------------------------
EPI_ISL_425537       ------------------------------------------------------------
EPI_ISL_425549       ------------------------------------------------------------
EPI_ISL_425551       ------------------------------------------------------------
EPI_ISL_425562       ------------------------------------------------------------
EPI_ISL_425576       ------------------------------------------------------------
EPI_ISL_425586       ------------------------------------------------------------
EPI_ISL_425587       ------------------------------------------------------------
EPI_ISL_426817       ------------------------------------------------------------
EPI_ISL_426832       ------------------------------------------------------------
```

```
ORF1ab          5461 TLKATEETFKLSYGIATVREVLSDRELHLSWEVGKPRPPLNRNYVFTGYRVTKNSKVQIG
EPI_ISL_424224       ------------------------------------------------------------
EPI_ISL_424255       ------------------------------------------------------------
EPI_ISL_425501       ------------------------------------------------------------
EPI_ISL_425502       ------------------------------------------------------------
EPI_ISL_425504       ------------------------------------------------------------
EPI_ISL_425523       ------------------------------------------------------------
EPI_ISL_425535       ------------------------------------------------------------
EPI_ISL_425537       ------------------------------------------------------------
EPI_ISL_425549       ------------------------------------------------------------
EPI_ISL_425551       ------------------------------------------------------------
EPI_ISL_425562       ------------------------------------------------------------
EPI_ISL_425576       ------------------------------------------------------------
EPI_ISL_425586       ------------------------------------------------------------
EPI_ISL_425587       ------------------------------------------------------------
EPI_ISL_426817       ------------------------------------------------------------
EPI_ISL_426832       ------------------------------------------------------------

ORF1ab          5521 EYTFEKGDYGDAVVYRGTTTYKLNVGDYFVLTSHTVMPLSAPTLVPQEHYVRITGLYPTL
EPI_ISL_424224       ------------------------------------------------------------
EPI_ISL_424255       ------------------------------------------------------------
EPI_ISL_425501       ------------------------------------------------------------
EPI_ISL_425502       ------------------------------------------------------------
EPI_ISL_425504       ------------------------------------------------------------
EPI_ISL_425523       ------------------------------------------------------------
EPI_ISL_425535       ------------------------------------------------------------
EPI_ISL_425537       ------------------------------------------------------------
EPI_ISL_425549       ------------------------------------------------------------
EPI_ISL_425551       ------------------------------------------------------------
EPI_ISL_425562       ------------------------------------------------------------
EPI_ISL_425576       ------------------------------------------------------------
EPI_ISL_425586       ------------------------------------------------------------
EPI_ISL_425587       ------------------------------------------------------------
EPI_ISL_426817       ------------------------------------------------------------
EPI_ISL_426832       ------------------------------------------------------------

ORF1ab          5581 NISDEFSSNVANYQKVGMQKYSTLQGPPGTGKSHFAIGLALYYPSARIVYTACSHAAVDA
EPI_ISL_424224       ------------------------------------------------------------
EPI_ISL_424255       ------------------------------------------------------------
EPI_ISL_425501       ------------------------------------------------------------
EPI_ISL_425502       ------------------------------------------------------------
EPI_ISL_425504       ------------------------------------------------------------
EPI_ISL_425523       ------------------------------------------------------------
EPI_ISL_425535       ------------------------------------------------------------
EPI_ISL_425537       ------------------------------------------------------------
EPI_ISL_425549       ------------------------------------------------------------
EPI_ISL_425551       ------------------------------------------------------------
EPI_ISL_425562       ------------------------------------------------------------
EPI_ISL_425576       ------------------------------------------------------------
EPI_ISL_425586       ------------------------------------------------------------
EPI_ISL_425587       ------------------------------------------------------------
EPI_ISL_426817       ------------------------------------------------------------
EPI_ISL_426832       ------------------------------------------------------------

ORF1ab          5641 LCEKALKYLPIDKCSRIIPARARVECFDKFKVNSTLEQYVFCTVNALPETTADIVVFDEI
EPI_ISL_424224       ------------------------------------------------------------
EPI_ISL_424255       ------------------------------------------------------------
EPI_ISL_425501       ------------------------------------------------------------
EPI_ISL_425502       ------------------------------------------------------------
EPI_ISL_425504       ------------------------------------------------------------
EPI_ISL_425523       ------------------------------------------------------------
EPI_ISL_425535       ------------------------------------------------------------
EPI_ISL_425537       ------------------------------------------------------------
```

```
EPI_ISL_425549      ------------------------------------------------------------
EPI_ISL_425551      ------------------------------------------------------------
EPI_ISL_425562      ------------------------------------------------------------
EPI_ISL_425576      ------------------------------------------------------------
EPI_ISL_425586      ------------------------------------------------------------
EPI_ISL_425587      ------------------------------------------------------------
EPI_ISL_426817      ------------------------------------------------------------
EPI_ISL_426832      ------------------------------------------------------------
```

```
ORF1ab          5701 SMATNYDLSVVNARLRAKHYVYIGDPAQLPAPRTLLTKGTLEPEYFNSVCRLMKTIGPDM
EPI_ISL_424224       ------------------------------------------------------------
EPI_ISL_424255       ------------------------------------------------------------
EPI_ISL_425501       ------------------------------------------------------------
EPI_ISL_425502       ------------------------------------------------------------
EPI_ISL_425504       ------------------------------------------------------------
EPI_ISL_425523       ------------------------------------------------------------
EPI_ISL_425535       ------------------------------------------------------------
EPI_ISL_425537       ------------------------------------------------------------
EPI_ISL_425549       ------------------------------------------------------------
EPI_ISL_425551       ------------------------------------------------------------
EPI_ISL_425562       ------------------------------------------------------------
EPI_ISL_425576       ------------------------------------------------------------
EPI_ISL_425586       ------------------------------------------------------------
EPI_ISL_425587       ------------------------------------------------------------
EPI_ISL_426817       ------------------------------------------------------------
EPI_ISL_426832       ------------------------------------------------------------

ORF1ab          5761 FLGTCRRCPAEIVDTVSALVYDNKLKAHKDKSAQCFKMFYKGVITHDVSSAINRPQIGVV
EPI_ISL_424224       ------------------------------------------------------------
EPI_ISL_424255       ------------------------------------------------------------
EPI_ISL_425501       ------------------------------------------------------------
EPI_ISL_425502       ------------------------------------------------------------
EPI_ISL_425504       ------------------------------------------------------------
EPI_ISL_425523       ------------------------------------------------------------
EPI_ISL_425535       ------------------------------------------------------------
EPI_ISL_425537       ------------------------------------------------------------
EPI_ISL_425549       ------------------------------------------------------------
EPI_ISL_425551       ------------------------------------------------------------
EPI_ISL_425562       ------------------------------------------------------------
EPI_ISL_425576       ------------------------------------------------------------
EPI_ISL_425586       ------------------------------------------------------------
EPI_ISL_425587       ------------------------------------------------------------
EPI_ISL_426817       ------------------------------------------------------------
EPI_ISL_426832       ------------------------------------------------------------

ORF1ab          5821 REFLTRNPAWRKAVFISPYNSQNAVASKILGLPTQTVDSSQGSEYDYVIFTQTTETAHSC
EPI_ISL_424224       ------------------------------------------------------------
EPI_ISL_424255       ------------------------------------------------------------
EPI_ISL_425501       ------------------------------------------------------------
EPI_ISL_425502       ------------------------------------------------------------
EPI_ISL_425504       ------------------------------------------------------------
EPI_ISL_425523       ------------------------------------------------------------
EPI_ISL_425535       ------------------------------------------------------------
EPI_ISL_425537       ------------------------------------------------------------
EPI_ISL_425549       ------------------------------------------------------------
EPI_ISL_425551       ------------------------------------------------------------
EPI_ISL_425562       ------------------------------------------------------------
EPI_ISL_425576       ------------------------------------------------------------
EPI_ISL_425586       ------------------------------------------------------------
EPI_ISL_425587       ------------------------------------------------------------
EPI_ISL_426817       ------------------------------------------------------------
EPI_ISL_426832       ------------------------------------------------------------
```

```
ORF1ab         5881 NVNRFNVAITRAKVGILCIMSDRDLYDKLQFTSLEIPRRNVATLQAENVTGLFKDCSKVI
EPI_ISL_424224      ------------------------------------------------------------
EPI_ISL_424255      ------------------------------------------------------------
EPI_ISL_425501      ------------------------------------------------------------
EPI_ISL_425502      ------------------------------------------------------------
EPI_ISL_425504      ------------------------------------------------------------
EPI_ISL_425523      ------------------------------------------------------------
EPI_ISL_425535      ------------------------------------------------------------
EPI_ISL_425537      ------------------------------------------------------------
EPI_ISL_425549      ------------------------------------------------------------
EPI_ISL_425551      ------------------------------------------------------------
EPI_ISL_425562      ------------------------------------------------------------
EPI_ISL_425576      ------------------------------------------------------------
EPI_ISL_425586      ------------------------------------------------------------
EPI_ISL_425587      ------------------------------------------------------------
EPI_ISL_426817      ------------------------------------------------------------
EPI_ISL_426832      ------------------------------------------------------------

ORF1ab         5941 TGLHPTQAPTHLSVDTKFKTEGLCVDIPGIPKDMTYRRLISMMGFKMNYQVNGYPNMFIT
EPI_ISL_424224      ------------------------------------------------------------
EPI_ISL_424255      ------------------------------------------------------------
EPI_ISL_425501      ------------------------------------------------------------
EPI_ISL_425502      ------------------------------------------------------------
EPI_ISL_425504      ------------------------------------------------------------
EPI_ISL_425523      ------------------------------------------------------------
EPI_ISL_425535      ------------------------------------------------------------
EPI_ISL_425537      ------------------------------------------------------------
EPI_ISL_425549      ------------------------------------------------------------
EPI_ISL_425551      ------------------------------------------------------------
EPI_ISL_425562      ------------------------------------------------------------
EPI_ISL_425576      ------------------------------------------------------------
EPI_ISL_425586      ------------------------------------------------------------
EPI_ISL_425587      ------------------------------------------------------------
EPI_ISL_426817      ------------------------------------------------------------
EPI_ISL_426832      ------------------------------------------------------------

ORF1ab         6001 REEAIRHVRAWIGFDVEGCHATREAVGTNLPLQLGFSTGVNLVAVPTGYVDTPNNTDFSR
EPI_ISL_424224      ------------------------------------------------------------
EPI_ISL_424255      ------------------------------------------------------------
EPI_ISL_425501      ------------------------------------------------------------
EPI_ISL_425502      ------------------------------------------------------------
EPI_ISL_425504      ------------------------------------------------------------
EPI_ISL_425523      ------------------------------------------------------------
EPI_ISL_425535      ------------------------------------------------------------
EPI_ISL_425537      ------------------------------------------------------------
EPI_ISL_425549      ------------------------------------------------------------
EPI_ISL_425551      ------------------------------------------------------------
EPI_ISL_425562      ------------------------------------------------------------
EPI_ISL_425576      ------------------------------------------------------------
EPI_ISL_425586      ------------------------------------------------------------
EPI_ISL_425587      ------------------------------------------------------------
EPI_ISL_426817      ------------------------------------------------------------
EPI_ISL_426832      ------------------------------------------------------------

ORF1ab         6061 VSAKPPPGDQFKHLIPLMYKGLPWNVVRIKIVQMLSDTLKNLSDRVVFVLWAHGFELTSM
EPI_ISL_424224      ------------------------------------------------------------
EPI_ISL_424255      ------------------------------------------------------------
EPI_ISL_425501      ------------------------------------------------------------
EPI_ISL_425502      ------------------------------------------------------------
EPI_ISL_425504      ------------------------------------------------------------
EPI_ISL_425523      ------------------------------------------------------------
EPI_ISL_425535      ------------------------------------------------------------
EPI_ISL_425537      ------------------------------------------------------------
```

```
EPI_ISL_425549      ------------------------------------------------------------
EPI_ISL_425551      ------------------------------------------------------------
EPI_ISL_425562      ------------------------------------------------------------
EPI_ISL_425576      ------------------------------------------------------------
EPI_ISL_425586      ------------------------------------------------------------
EPI_ISL_425587      ------------------------------------------------------------
EPI_ISL_426817      ------------------------------------------------------------
EPI_ISL_426832      ------------------------------------------------------------
```

```
ORF1ab          6121 KYFVKIGPERTCCLCDRRATCFSTASDTYACWHHSIGFDYVYNPFMIDVQQWGFTGNLQS
EPI_ISL_424224       ------------------------------------------------------------
EPI_ISL_424255       ------------------------------------------------------------
EPI_ISL_425501       ------------------------------------------------------------
EPI_ISL_425502       ------------------------------------------------------------
EPI_ISL_425504       ------------------------------------------------------------
EPI_ISL_425523       ------------------------------------------------------------
EPI_ISL_425535       ------------------------------------------------------------
EPI_ISL_425537       ------------------------------------------------------------
EPI_ISL_425549       ------------------------------------------------------------
EPI_ISL_425551       ------------------------------------------------------------
EPI_ISL_425562       ------------------------------------------------------------
EPI_ISL_425576       ------------------------------------------------------------
EPI_ISL_425586       ------------------------------------------------------------
EPI_ISL_425587       ------------------------------------------------------------
EPI_ISL_426817       ------------------------------------------------------------
EPI_ISL_426832       ------------------------------------------------------------

ORF1ab          6181 NHDLYCQVHGNAHVASCDAIMTRCLAVHECFVKRVDWTIEYPIIGDELKINAACRKVQHM
EPI_ISL_424224       ------------------------------------------------------------
EPI_ISL_424255       ------------------------------------------------------------
EPI_ISL_425501       ------------------------------------------------------------
EPI_ISL_425502       ------------------------------------------------------------
EPI_ISL_425504       ------------------------------------------------------------
EPI_ISL_425523       ------------------------------------------------------------
EPI_ISL_425535       ------------------------------------------------------------
EPI_ISL_425537       ------------------------------------------------------------
EPI_ISL_425549       ------------------------------------------------------------
EPI_ISL_425551       ------------------------------------------------------------
EPI_ISL_425562       ------------------------------------------------------------
EPI_ISL_425576       ------------------------------------------------------------
EPI_ISL_425586       ------------------------------------------------------------
EPI_ISL_425587       ------------------------------------------------------------
EPI_ISL_426817       ------------------------------------------------------------
EPI_ISL_426832       ------------------------------------------------------------

ORF1ab          6241 VVKAALLADKFPVLHDIGNPKAIKCVPQADVEWKFYDAQPCSDKAYKIEELFYSYATHSD
EPI_ISL_424224       ------------------------------------------------------------
EPI_ISL_424255       ------------------------------------------------------------
EPI_ISL_425501       ------------------------------------------------------------
EPI_ISL_425502       ------------------------------------------------------------
EPI_ISL_425504       ------------------------------------------------------------
EPI_ISL_425523       ------------------------------------------------------------
EPI_ISL_425535       ------------------------------------------------------------
EPI_ISL_425537       ------------------------------------------------------------
EPI_ISL_425549       ------------------------------------------------------------
EPI_ISL_425551       ------------------------------------------------------------
EPI_ISL_425562       ------------------------------------------------------------
EPI_ISL_425576       ------------------------------------------------------------
EPI_ISL_425586       ------------------------------------------------------------
EPI_ISL_425587       ------------------------------------------------------------
EPI_ISL_426817       ------------------------------------------------------------
EPI_ISL_426832       ------------------------------------------------------------
```

```
ORF1ab         6301 KFTDGVCLFWNCNVDRYPANSIVCRFDTRVLSNLNLPGCDGGSLYVNKHAFHTPAFDKSA
EPI_ISL_424224      ------------------------------------------------------------
EPI_ISL_424255      ------------------------------------------------------------
EPI_ISL_425501      ------------------------------------------------------------
EPI_ISL_425502      ------------------------------------------------------------
EPI_ISL_425504      ------------------------------------------------------------
EPI_ISL_425523      ------------------------------------------------------------
EPI_ISL_425535      ------------------------------------------------------------
EPI_ISL_425537      ------------------------------------------------------------
EPI_ISL_425549      ------------------------------------------------------------
EPI_ISL_425551      ------------------------------------------------------------
EPI_ISL_425562      ------------------------------------------------------------
EPI_ISL_425576      ------------------------------------------------------------
EPI_ISL_425586      ------------------------------------------------------------
EPI_ISL_425587      ------------------------------------------------------------
EPI_ISL_426817      ------------------------------------------------------------
EPI_ISL_426832      ------------------------------------------------------------

ORF1ab         6361 FVNLKQLPFFYYSDSPCESHGKQVVSDIDYVPLKSATCITRCNLGGAVCRHHANEYRLYL
EPI_ISL_424224      ------------------------------------------------------------
EPI_ISL_424255      ------------------------------------------------------------
EPI_ISL_425501      ------------------------------------------------------------
EPI_ISL_425502      ------------------------------------------------------------
EPI_ISL_425504      ------------------------------------------------------------
EPI_ISL_425523      ------------------------------------------------------------
EPI_ISL_425535      ------------------------------------------------------------
EPI_ISL_425537      ------------------------------------------------------------
EPI_ISL_425549      ------------------------------------------------------------
EPI_ISL_425551      ------------------------------------------------------------
EPI_ISL_425562      ------------------------------------------------------------
EPI_ISL_425576      ------------------------------------------------------------
EPI_ISL_425586      ------------------------------------------------------------
EPI_ISL_425587      ------------------------------------------------------------
EPI_ISL_426817      ------------------------------------------------------------
EPI_ISL_426832      ------------------------------------------------------------

ORF1ab         6421 DAYNMMISAGFSLWVYKQFDTYNLWNTFTRLQSLENVAFNVVNKGHFDGQQGEVPVSIIN
EPI_ISL_424224      ------------------------------------------------------------
EPI_ISL_424255      ------------------------------------------------------------
EPI_ISL_425501      ------------------------------------------------------------
EPI_ISL_425502      ------------------------------------------------------------
EPI_ISL_425504      ------------------------------------------------------------
EPI_ISL_425523      ------------------------------------------------------------
EPI_ISL_425535      ------------------------------------------------------------
EPI_ISL_425537      ------------------------------------------------------------
EPI_ISL_425549      ------------------------------------------------------------
EPI_ISL_425551      ------------------------------------------------------------
EPI_ISL_425562      ------------------------------------------------------------
EPI_ISL_425576      ------------------------------------------------------------
EPI_ISL_425586      ------------------------------------------------------------
EPI_ISL_425587      ------------------------------------------------------------
EPI_ISL_426817      ------------------------------------------------------------
EPI_ISL_426832      ------------------------------------------------------------

ORF1ab         6481 NTVYTKVDGVDVELFENKTTLPVNVAFELWAKRNIKPVPEVKILNNLGVDIAANTVIWDY
EPI_ISL_424224      ------------------------------------------------------------
EPI_ISL_424255      ------------------------------------------------------------
EPI_ISL_425501      ------------------------------------------------------------
EPI_ISL_425502      ------------------------------------------------------------
EPI_ISL_425504      ------------------------------------------------------------
EPI_ISL_425523      ------------------------------------------------------------
EPI_ISL_425535      ------------------------------------------------------------
EPI_ISL_425537      ------------------------------------------------------------
```

```
EPI_ISL_425549      ------------------------------------------------------------
EPI_ISL_425551      ------------------------------------------------------------
EPI_ISL_425562      ------------------------------------------------------------
EPI_ISL_425576      ------------------------------------------------------------
EPI_ISL_425586      ------------------------------------------------------------
EPI_ISL_425587      ------------------------------------------------------------
EPI_ISL_426817      ------------------------------------------------------------
EPI_ISL_426832      ------------------------------------------------------------
```

```
ORF1ab         6541 KRDAPAHISTIGVCSMTDIAKKPTETICAPLTVFFDGRVDGQVDLFRNARNGVLITEGSV
EPI_ISL_424224      ------------------------------------------------------------
EPI_ISL_424255      ------------------------------------------------------------
EPI_ISL_425501      ------------------------------------------------------------
EPI_ISL_425502      ------------------------------------------------------------
EPI_ISL_425504      ------------------------------------------------------------
EPI_ISL_425523      ------------------------------------------------------------
EPI_ISL_425535      ------------------------------------------------------------
EPI_ISL_425537      ------------------------------------------------------------
EPI_ISL_425549      ------------------------------------------------------------
EPI_ISL_425551      ------------------------------------------------------------
EPI_ISL_425562      ------------------------------------------------------------
EPI_ISL_425576      ------------------------------------------------------------
EPI_ISL_425586      ------------------------------------------------------------
EPI_ISL_425587      ------------------------------------------------------------
EPI_ISL_426817      ------------------------------------------------------------
EPI_ISL_426832      ------------------------------------------------------------

ORF1ab         6601 KGLQPSVGPKQASLNGVTLIGEAVKTQFNYYKKVDGVVQQLPETYFTQSRNLQEFKPRSQ
EPI_ISL_424224      ------------------------------------------------------------
EPI_ISL_424255      ------------------------------------------------------------
EPI_ISL_425501      ------------------------------------------------------------
EPI_ISL_425502      ------------------------------------------------------------
EPI_ISL_425504      ------------------------------------------------------------
EPI_ISL_425523      ------------------------------------------------------------
EPI_ISL_425535      ------------------------------------------------------------
EPI_ISL_425537      ------------------------------------------------------------
EPI_ISL_425549      ------------------------------------------------------------
EPI_ISL_425551      ------------------------------------------------------------
EPI_ISL_425562      ------------------------------------------------------------
EPI_ISL_425576      ------------------------------------------------------------
EPI_ISL_425586      ------------------------------------------------------------
EPI_ISL_425587      ------------------------------------------------------------
EPI_ISL_426817      ------------------------------------------------------------
EPI_ISL_426832      ------------------------------------------------------------

ORF1ab         6661 MEIDFLELAMDEFIERYKLEGYAFEHIVYGDFSHSQLGGLHLLIGLAKRFKESPFELEDF
EPI_ISL_424224      ------------------------------------------------------------
EPI_ISL_424255      ------------------------------------------------------------
EPI_ISL_425501      ------------------------------------------------------------
EPI_ISL_425502      ------------------------------------------------------------
EPI_ISL_425504      ------------------------------------------------------------
EPI_ISL_425523      ------------------------------------------------------------
EPI_ISL_425535      ------------------------------------------------------------
EPI_ISL_425537      ------------------------------------------------------------
EPI_ISL_425549      ------------------------------------------------------------
EPI_ISL_425551      ------------------------------------------------------------
EPI_ISL_425562      ------------------------------------------------------------
EPI_ISL_425576      ------------------------------------------------------------
EPI_ISL_425586      ------------------------------------------------------------
EPI_ISL_425587      ------------------------------------------------------------
EPI_ISL_426817      ------------------------------------------------------------
EPI_ISL_426832      ------------------------------------------------------------
```

```
ORF1ab          6721 IPMDSTVKNYFITDAQTGSSKCVCSVIDLLLDDFVEIIKSQDLSVVSKVVKVTIDYTEIS
EPI_ISL_424224       ------------------------------------------------------------
EPI_ISL_424255       ------------------------------------------------------------
EPI_ISL_425501       ------------------------------------------------------------
EPI_ISL_425502       ------------------------------------------------------------
EPI_ISL_425504       ------------------------------------------------------------
EPI_ISL_425523       ------------------------------------------------------------
EPI_ISL_425535       ------------------------------------------------------------
EPI_ISL_425537       ------------------------------------------------------------
EPI_ISL_425549       ------------------------------------------------------------
EPI_ISL_425551       ------------------------------------------------------------
EPI_ISL_425562       ------------------------------------------------------------
EPI_ISL_425576       ------------------------------------------------------------
EPI_ISL_425586       ------------------------------------------------------------
EPI_ISL_425587       ------------------------------------------------------------
EPI_ISL_426817       ------------------------------------------------------------
EPI_ISL_426832       ------------------------------------------------------------

ORF1ab          6781 FMLWCKDGHVETFYPKLQSSQAWQPGVAMPNLYKMQRMLLEKCDLQNYGDSATLPKGIMM
EPI_ISL_424224       ------------------------------------------------------------
EPI_ISL_424255       ------------------------------------------------------------
EPI_ISL_425501       ------------------------------------------------------------
EPI_ISL_425502       ------------------------------------------------------------
EPI_ISL_425504       ------------------------------------------------------------
EPI_ISL_425523       ------------------------------------------------------------
EPI_ISL_425535       ------------------------------------------------------------
EPI_ISL_425537       ------------------------------------------------------------
EPI_ISL_425549       ------------------------------------------------------------
EPI_ISL_425551       ------------------------------------------------------------
EPI_ISL_425562       ------------------------------------------------------------
EPI_ISL_425576       ------------------------------------------------------------
EPI_ISL_425586       ------------------------------------------------------------
EPI_ISL_425587       ------------------------------------------------------------
EPI_ISL_426817       ------------------------------------------------------------
EPI_ISL_426832       ------------------------------------------------------------

ORF1ab          6841 NVAKYTQLCQYLNTLTLAVPYNMRVIHFGAGSDKGVAPGTAVLRQWLPTGTLLVDSDLND
EPI_ISL_424224       ------------------------------------------------------------
EPI_ISL_424255       ------------------------------------------------------------
EPI_ISL_425501       ------------------------------------------------------------
EPI_ISL_425502       ------------------------------------------------------------
EPI_ISL_425504       ------------------------------------------------------------
EPI_ISL_425523       ------------------------------------------------------------
EPI_ISL_425535       ------------------------------------------------------------
EPI_ISL_425537       ------------------------------------------------------------
EPI_ISL_425549       ------------------------------------------------------------
EPI_ISL_425551       ------------------------------------------------------------
EPI_ISL_425562       ------------------------------------------------------------
EPI_ISL_425576       ------------------------------------------------------------
EPI_ISL_425586       ------------------------------------------------------------
EPI_ISL_425587       ------------------------------------------------------------
EPI_ISL_426817       ------------------------------------------------------------
EPI_ISL_426832       ------------------------------------------------------------

ORF1ab          6901 FVSDADSTLIGDCATVHTANKWDLIISDMYDPKTKNVTKENDSKEGFFTYICGFIQQKLA
EPI_ISL_424224       ------------------------------------------------------------
EPI_ISL_424255       ------------------------------------------------------------
EPI_ISL_425501       ------------------------------------------------------------
EPI_ISL_425502       ------------------------------------------------------------
EPI_ISL_425504       ------------------------------------------------------------
EPI_ISL_425523       ------------------------------------------------------------
EPI_ISL_425535       ------------------------------------------------------------
EPI_ISL_425537       ------------------------------------------------------------
```

```
EPI_ISL_425549      ------------------------------------------------------------
EPI_ISL_425551      ------------------------------------------------------------
EPI_ISL_425562      ------------------------------------------------------------
EPI_ISL_425576      ------------------------------------------------------------
EPI_ISL_425586      ------------------------------------------------------------
EPI_ISL_425587      ------------------------------------------------------------
EPI_ISL_426817      ------------------------------------------------------------
EPI_ISL_426832      ------------------------------------------------------------
```

```
ORF1ab          6961 LGGSVAIKITEHSWNADLYKLMGHFAWWTAFVTNVNASSSEAFLIGCNYLGKPREQIDGY
EPI_ISL_424224       ------------------------------------------------------------
EPI_ISL_424255       ------------------------------------------------------------
EPI_ISL_425501       ------------------------------------------------------------
EPI_ISL_425502       ------------------------------------------------------------
EPI_ISL_425504       ------------------------------------------------------------
EPI_ISL_425523       ------------------------------------------------------------
EPI_ISL_425535       ------------------------------------------------------------
EPI_ISL_425537       ------------------------------------------------------------
EPI_ISL_425549       ------------------------------------------------------------
EPI_ISL_425551       ------------------------------------------------------------
EPI_ISL_425562       ------------------------------------------------------------
EPI_ISL_425576       ------------------------------------------------------------
EPI_ISL_425586       ------------------------------------------------------------
EPI_ISL_425587       ------------------------------------------------------------
EPI_ISL_426817       ------------------------------------------------------------
EPI_ISL_426832       ------------------------------------------------------------

ORF1ab          7021 VMHANYIFWRNTNPIQLSSYSLFDMSKFPLKLRGTAVMSLKEGQINDMILSLLSKGRLII
EPI_ISL_424224       ------------------------------------------------------------
EPI_ISL_424255       ------------------------------------------------------------
EPI_ISL_425501       ------------------------------------------------------------
EPI_ISL_425502       ------------------------------------------------------------
EPI_ISL_425504       ------------------------------------------------------------
EPI_ISL_425523       ------------------------------------------------------------
EPI_ISL_425535       ------------------------------------------------------------
EPI_ISL_425537       ------------------------------------------------------------
EPI_ISL_425549       ------------------------------------------------------------
EPI_ISL_425551       ------------------------------------------------------------
EPI_ISL_425562       ------------------------------------------------------------
EPI_ISL_425576       ------------------------------------------------------------
EPI_ISL_425586       ------------------------------------------------------------
EPI_ISL_425587       ------------------------------------------------------------
EPI_ISL_426817       ------------------------------------------------------------
EPI_ISL_426832       ------------------------------------------------------------

ORF1ab          7081 RENNRVVISSDVLVNN
EPI_ISL_424224       ----------------
EPI_ISL_424255       ----------------
EPI_ISL_425501       ----------------
EPI_ISL_425502       ----------------
EPI_ISL_425504       ----------------
EPI_ISL_425523       ----------------
EPI_ISL_425535       ----------------
EPI_ISL_425537       ----------------
EPI_ISL_425549       ----------------
EPI_ISL_425551       ----------------
EPI_ISL_425562       ----------------
EPI_ISL_425576       ----------------
EPI_ISL_425586       ----------------
EPI_ISL_425587       ----------------
EPI_ISL_426817       ----------------
EPI_ISL_426832       ----------------
```
